# The contribution of non-essential *Schizosaccharomyces pombe* genes to fitness in response to altered nutrient supply and target of rapamycin activity

**DOI:** 10.1098/rsob.180015

**Published:** 2018-05-02

**Authors:** Shervi Lie, Peter Banks, Conor Lawless, David Lydall, Janni Petersen

**Affiliations:** 1 Flinders Centre for Innovation in Cancer, College of Medicine & Public Health, Flinders University, Bedford Park, Adelaide, South Australia 5042, Australia; 2 High Throughput Screening Facility, Newcastle Biomedicine, Newcastle University, Newcastle upon Tyne NE2 4HH, UK; 3 Institute for Cell & Molecular Biosciences, Newcastle University Medical School, Newcastle upon Tyne NE2 4HH, UK; 4 South Australia Health and Medical Research Institute, North Terrace, PO Box 11060, Adelaide, South Australia 5000 Australia

**Keywords:** *Schizosaccharomyces pombe*, Torin1, TOR substrates, nutrient stress, TORC1, TORC2

## Abstract

Nutrient fluctuations in the cellular environment promote changes in cell metabolism and growth to adapt cell proliferation accordingly. The target of rapamycin (TOR) signalling network plays a key role in the coordination of growth and cell proliferation with the nutrient environment and, importantly, nutrient limitation reduces TOR complex 1 (TORC1) signalling. We have performed global quantitative fitness profiling of the collection of *Schizosaccharomyces pombe* strains from which non-essential genes have been deleted. We identified genes that regulate fitness when cells are grown in a nutrient-rich environment compared with minimal environments, with varying nitrogen sources including ammonium, glutamate and proline. In addition, we have performed the first global screen for genes that regulate fitness when both TORC1 and TORC2 signalling is reduced by Torin1. Analysis of genes whose deletions altered fitness when nutrients were limited, or when TOR signalling was compromised, identified a large number of genes that regulate transmembrane transport, transcription and chromatin organization/regulation and vesicle-mediated transport. The ability to tolerate reduced TOR signalling placed demands upon a large number of biological processes including autophagy, mRNA metabolic processing and nucleocytoplasmic transport. Importantly, novel biological processes and all processes known to be regulated by TOR were identified in our screens. In addition, deletion of 62 genes conserved in humans gave rise to strong sensitivity or resistance to Torin1, and 29 of these 62 genes have novel links to TOR signalling. The identification of chromatin and transcriptional regulation, nutritional uptake and transport pathways in this powerful genetic model now paves the way for a molecular understanding of how cells adapt to the chronic and acute fluctuations in nutrient supply that all eukaryotes experience at some stage, and which is a key feature of cancer cells within solid tumours.

## 1. Introduction

Cell proliferation is exquisitely sensitive to nutrient resources and requires metabolic adaption to meet the demands of dynamic changes in environmental conditions. In the presence of an abundant supply of rich nutrients, cells maintain high levels of protein synthesis to increase biomass and promote division. Target of rapamycin (TOR), a protein kinase, is one of the major energy and nutrient sensors in eukaryotic cells. TOR coordinates the growth and cell cycle progression of a cell with its specific environmental context and nutrient environment by controlling a range of biological processes, including metabolism, cell migration and cell division. In general, the nutrient environments heavily impact upon cell proliferation in eukaryotes. The TOR protein kinase forms two functionally distinct multi-protein complexes: TOR complex 1 (TORC1) and TOR complex 2 (TORC2), which are defined by unique components highly conserved across species; in mammals Raptor defines mTORC1, while Rictor defines mTORC2 [1]. In fission yeast *Schizosaccharomyces pombe* Mip1 defines TORC1 and Ste20 defines (TORC2) [2–4]. In all eukaryotes, it is rapamycin-sensitive TORC1 that is the major nutrient sensor that integrates environmental cues with cell growth and proliferation. Fission yeast TORC2 is not essential for cell proliferation [5], and TORC2 exerts distinct functions by phosphorylating distinct substrates that are required for sexual differentiation, actin organization and dynamics, to name just a few [1,4,5].

Acute addition of rapamycin specifically reduces TORC1 activity and has emerged as a promising therapeutic agent in the treatment of a variety of diseases, including cancer, autoimmune diseases, cardiovascular disease and metabolic disorders, due to its anti-tumour and immunosuppressant properties [1]. By contrast, Torin1 is an ATP analogue that competitively binds and inhibits the kinase activity of both TORC1 and TORC2 [6,7]. Torin1 is a more potent inhibitor of TORC1 than rapamycin. Interestingly, the heightened impact of Torin1 on cell growth and proliferation in mammalian cells was not mediated through an additive impact on both TORC1 and TORC2 inhibition; rather, it arises from the inhibition of the rapamycin insensitive elements of TORC1 signalling [7].

Many laboratories have described how reduction in nutrients, including carbon fuel supply (which reduces cellular energy levels) and changes in amino acid concentrations, is actively sensed by cells to modulate TORC1 activity. We recently defined an additional mode of nitrogen sensing, by uncovering nitrogen-dependent control of TORC1 activity that acts independently of amino acid sensing to respond to fluctuations in AMPK [8].

Genome-wide studies using yeast gene deletion libraries have been conducted in both budding and fission yeasts to identify genes and signalling pathways that support viability upon reductions in TORC1 activity [9–13]. These studies have identified a large number of TORC1-dependent processes including tRNA modification, mitochondria biogenesis, metabolism, cell cycle and ageing. We set out to complement these screens with a system-level screen for genes required for viability when both TORC1 and TORC2 were compromised along with a screen for genes required upon nutrient stress to which TORC1 signalling naturally responds.

We used global quantitative fitness profiling [14,15] to compare the fitness of the collection of yeast strains from which non-essential genes have been deleted and grown in a nutrient-rich environment to the fitness of the same strains grown on minimal media in which the quality of the nitrogen supply was altered by provision of either the high-quality ammonium, intermediate glutamate or poor nitrogen source, proline. The impact of Torin1 upon growth served to place the outputs from these varied nutrient supply into the context of TOR signalling. Gene ontology analysis showed that genes for which their deletions altered fitness on minimal media (significance *p*-value = 0.05 or below) were regulating various biological processes, including amino acid metabolism, trans-membrane transport, transcription and chromatin organization among others. Of the 3307 deletion strains in the collection, loss of 241 genes showed decreased cell fitness while loss of 100 genes increased cell fitness when TOR signalling was inhibited by the inclusion of Torin1 to block both TORC1 and TORC2. Gene ontology analysis of these genes identified genes in a broad range of biological processes with transcription, trans-membrane transport, vesicle-mediated transport, carbohydrate derivate metabolic processes and chromatin organization topping the list, alongside genes involved in autophagy, mRNA metabolic processes and nucleo-cytoplasmic transport. Interestingly, reduction of TOR signalling through either a reduction in the quality of the nitrogen source or by Torin1 placed demands on transcription and chromatin organization and nutrient uptake by transmembrane transport.

## 2. Results

### 2.1. Quantitative fitness analysis of the *S. pombe* deletion collection in diverse nutrient environments

Synthetic genetic arrays that use yeast deletion libraries are standard tools to identify synthetic lethal genetic interactions on a genome-wide scale in both *S. cerevisiae* and *S. pombe* [16,17]. Here, we performed global quantitative fitness analysis (QFA), an established high-throughput experimental and computational method [14,15], with the *S. pombe* gene deletion library. We sought to identify non-essential genes whose deletion altered cell fitness, when nutritional supply was changed by comparison of growth on minimal and rich media, or when TOR signalling was reduced. *Schizosaccharomyces pombe* has the ability to proliferate on a diverse selection of nutrient environments, including complex ‘rich’ yeast extract with supplements (YES) and defined synthetic minimal medium based on Edinburgh minimal media (EMM) [18,19]. The source of nitrogen in EMM can be varied to provide varying qualities of nitrogen from the ready nitrogen supply provided by ammonium chloride in EMM2 [20] and the glutamate in EMMG to the poor nitrogen source, proline, in EMMP [21,22].

**Figure 1.**
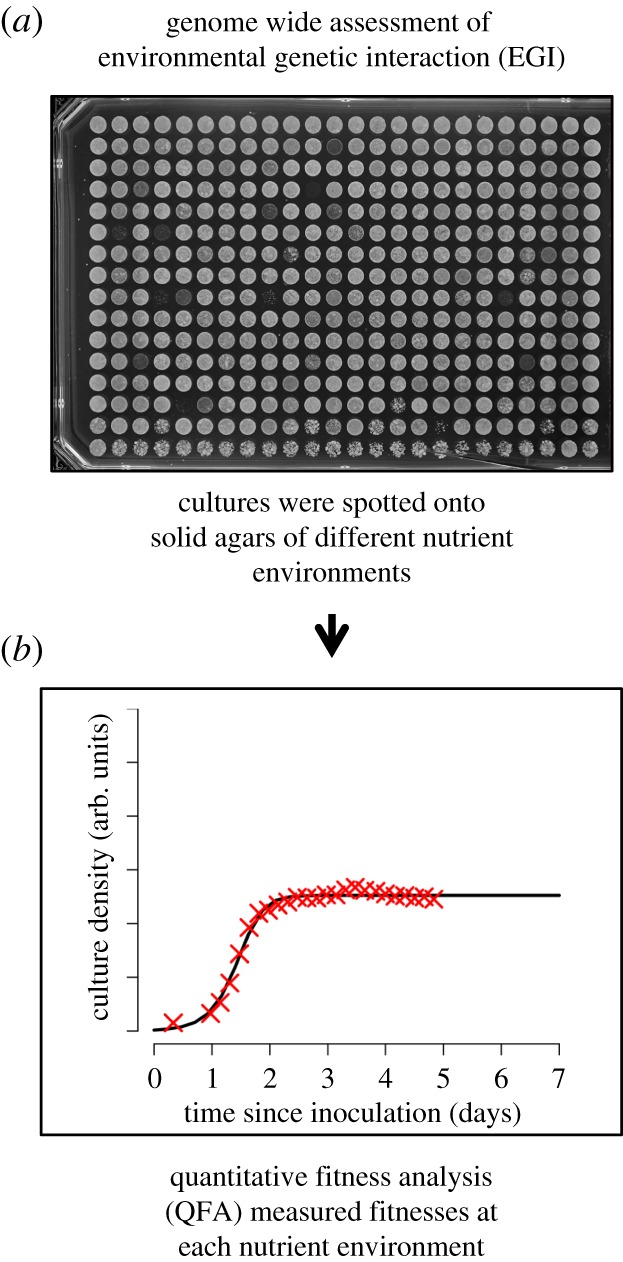
Overview of the robotic screen. (*a*) Deletion strains were cultured in liquid YES (rich media) and were spotted onto solid agar of the desired nutrient environment. (*b*) An individual robot-captured QFA growth curve is based on time course photography of forming colonies this is used to establish cell fitness.

**Figure 2.**
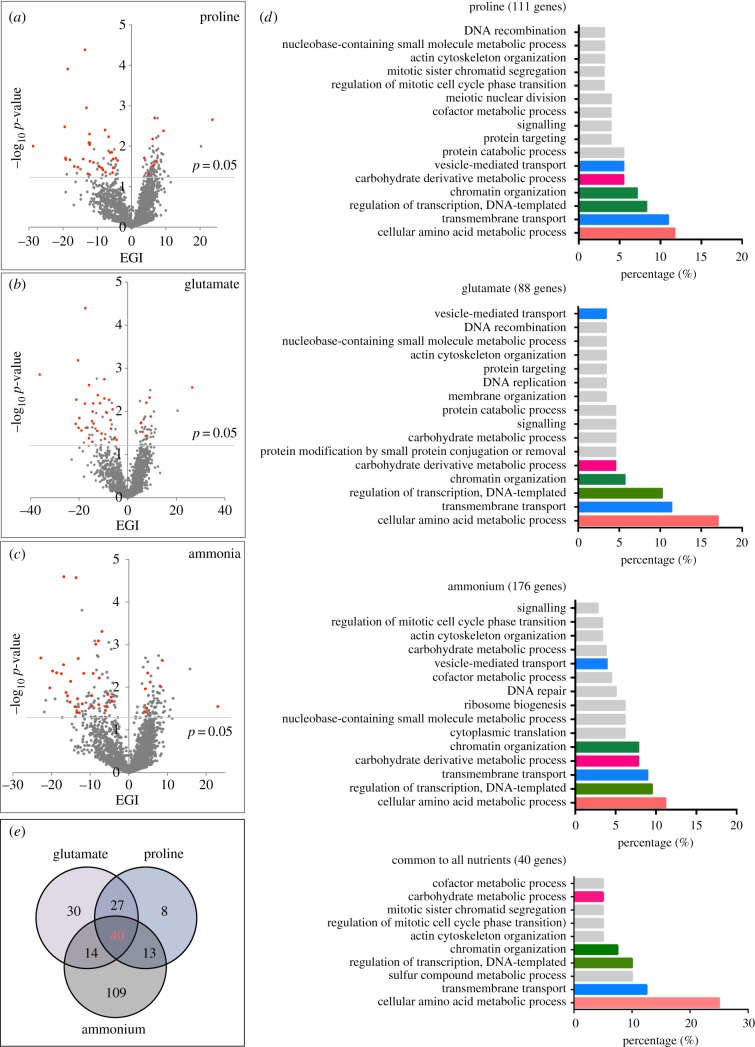
Altered cell fitness in minimal media. Cell fitness for all strains grown in minimal media was established and plotted against their fitness in rich media (see electronic supplementary material, figure S1). The impact of the environment on cell fitness, here entitled the EGI (environmental and genetic interaction), was calculated for each strain as the deviation from the solid line of equal fitness [14,15]. (*a*–*c*) The EGIs for all strains plotted against their significance as volcano plots. Significance *p*-value = 0.05 is indicated by line. For all three plots (*a*–*c*), the EGI is based on fitness in YES-rich media compared with the indicated minimal media. Red dots indicated 40 genes that showed altered fitness in all three minimal media compared with YES; these are listed in electronic supplementary material, figure S2 and also shown in (*d*) and (*e*). (*d*) Gene ontology analysis of gene deletions that altered cell fitness (≤ −3.00 EGI *p* = 0.05 or ≥ +3.00 EGI *p* = 0.05) in the indicated minimal nutrient environment, the top 90% of biological functions mapped are shown. All genes and the associated EGIs are listed in electronic supplementary material, tables S4–S6. Minimal media containing proline represent the poorest nutrient environment tested, the biological functions to which most gene deletions mapped are colour-coded, and this colour-code is used in the other screens to aid identifications. (*e*) Venn diagram illustrating the number of gene deletions that shows altered cell fitness in all nutrient environments tested; the 40 common genes are shown in red.

Each deletion strain was cultured in liquid YES (rich media) at 30°C, before the cultures were spotted onto solid agars of different nutrient environments (figure 1*a*). Growth curves which were based on time-course photography of the colony-forming deletion strains provided the QFA of each strain in each of the four individual nutrient environments (figure 1*b*): YES (rich media), and the three minimal media—EMM2, EMMG and EMMP. To determine the fitness of individual spotted yeast strains, estimated colony density for each strain was calculated by image processing using Colonyzer software. The colony size estimate was used to fit growth measurements to a logistic model and calculate growth parameters. The final fitness measure, as described by Addinal *et al*. [23], is the product of MDR (maximum doubling rate, population doublings per day) and MDP (maximum doubling potential, population doublings). The fitness of strains in each environment was established based on four independent replicates of each strain on each condition. The fitness established for each strain in rich media (YES) was plotted as a scatter plot against their fitness in ammonium-, glutamate- and proline (as nitrogen sources)-based media (electronic supplementary material, figure S1). Based on these scatter plots, the impact of the nutrient environment on cells fitness (deviation from the solid line overlaid as the line of equal fitness) of each genetic background was calculated [14,15], here entitled the environmental and genetic interaction (EGI) [14,15] (electronic supplementary material, tables S1–S3 show the EGIs for all strains grown in EMM2, EMMG and EMMP compared to YES). The EGIs were plotted against the significance as volcano plots (figure 2*a–c*) and gene deletions that show significant different fitness in altered nutrient environments are listed in (electronic supplementary material, tables S4–S6).

Loss of 111 genes had a differential impact on cell growth in comparisons between cell fitness grown in a complex nutrient-rich environment (YES) with the fitness of the same strains in EMMP (figure 2*a*,*d* and electronic supplementary material, table S4), whereas 88 and 176 gene deletions differentially impacted upon fitness when grown on EMMG and EMM2 compared to YES (figure 2*b*–*d*; electronic supplementary material, tables S5 and S6). Forty per cent of genes (94 of 241 unique genes) had an impact on cell fitness in at least two different minimal nutrient environments (figure 2*e*). Of these 94 genes, 40 genes impacted on cell fitness in all three minimal media (figure 2*a*–*c*, red dots; figure 2*e*,*d*; electronic supplementary material, figure S2). We used GO-term analysis to identify the biological roles of the genes (http://go.princeton.edu/cgi-bin/GOTermMapper). Minimal media containing proline represent the poorest nutrient environment tested. The biological functions to which most gene deletions mapped are colour-coded and this colour-code is used in the following screens to aid identifications. GO-term analysis established that regulation of cellular amino acid metabolic processes was observed in all three minimal nutrient environments, alongside transcription, chromatin organization and transmembrane transport (figure 2*d*). All these processes are known to be regulated by nutrients, as discussed later. Deletion of *hmt2* (a sulfide-quinone oxidoreductase), *mni1* (an exon junction regulating factor), *pha2* (a phrenate dehydratase) *SPCC320.03* (a transcription factor) and *SPCC794.03* (an amino acid permease) each impacted upon cell fitness in all three minimal nutrient environments tested (electronic supplementary material, figure S2). Importantly, mutations of these genes have previously been associated with altered viability when starved for nitrogen [24–26].

### 2.2. The effect of Torin1 on cell fitness of the *S. pombe* deletion collection

**Figure 3.**
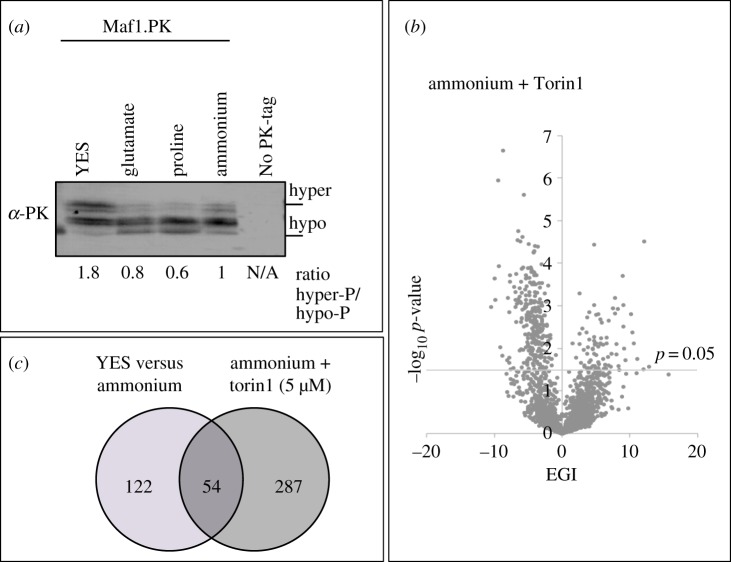
Altered cell fitness upon the addition of Torin1 to reduce TOR signalling. (*a*) TORC1 activity appears to be increased in rich media as Maf1 was hyper-phosphorylated. Phosphorylation of the direct TORC1 substrate Maf1 was analysed by western blotting of a Maf1.PK tagged strain. (*b*) Cell fitness for all strains grown in minimal media with ammonium as nitrogen source with either DMSO or Torin1 added was established and the EGIs were plotted against the significance as volcano plots. The cell fitness was based on four independent replicates. Significance *p*-value = 0.05 is indicated by line. Gene deletions that show significant different fitness (≤ −3.00 EGI *p* = 0.05 or ≥ +3.00 EGI *p* = 0.05) are listed in electronic supplementary material, table S5. (*c*) Venn diagram illustrating the number of gene deletions that show altered cell fitness when ammonium-based medium was compared with rich medium and when TOR signalling was further reduced with Torin1.

Three previous studies screened the same *S. pombe* deletion collection for strains that displayed sensitivity to inhibition of the subset of TORC1 activity that is sensitive to rapamycin on rich media or to the combined impact of the addition of rapamycin and caffeine on rich YES media [9–11]. However, TORC2 acts alongside TORC1 in coupling cell growth and metabolism to the demands of the nutrient environment [1,27,28]. We previously showed that fission yeast TORC1 activity was reduced in minimal media [8,29,30] and that Ppk32 (an *S. pombe* Scyl1 homolog) inhibits both TORC1 and TORC2 in a nutrient-dependent manner [31]. In line with these previous observations, Maf1 (a TORC1-specific substrate) [32] is hyper-phosphorylated in rich YES media (figure 3*a*).

To identify genes that regulate cell fitness in minimal media when both TORC1 and TORC2 signalling are reduced, 5 µM of Torin1 was added to the ammonium chloride containing EMM2 minimal media and compared with vehicle (DMSO) alone controls. The average fitness of four independent replicates was plotted as a scatter plot showing DMSO against fitness in the presence of Torin1 (electronic supplementary material, figure S4). The impact of Torin1 on the fitness of cells (EGIs—deviation from the solid line overlaid as the line of equal fitness) was plotted against the significance as volcano plots (figure 3*b*; electronic supplementary material, table S7). Gene deletions that conferred a significant difference in fitness when Torin1 was added are listed in electronic supplementary material, table S8. In this screen, 341 deletion strains significantly altered cell fitness: 241 gene deletions conferred sensitivity, while 100 imparted some level of resistance to 5 µM Torin1 (*p*-value ≤ 0.05). Importantly, several of the genes that altered fitness in response to Torin1 have previously been associated with regulation of the TOR pathway and thus serve as validation for this screen. Among these are *tsc2*, *gaf1*, *etr1*, *elp1*, *atg2*, *atg5*, *atg14*, *atg16*, *atg12*, *par2*, *toc1*, *crf1* and *ksp1* [10,11,33–40].

As shown above, the basal level of TORC1 signalling in EMM2 is below that seen in YES-rich media (figure 3*a*). Of the 176 genes, 54 genes that when deleted impacted upon cell fitness on EMM2 also showed altered fitness when TOR signalling was repressed further through the addition of Torin1 (figure 3*c*; electronic supplementary material, figure S4). By contrast, 287 genes only affected cell fitness when Torin1 was added to the EMM2 media (containing ammonium) (figure 3*c*).

**Figure 4.**
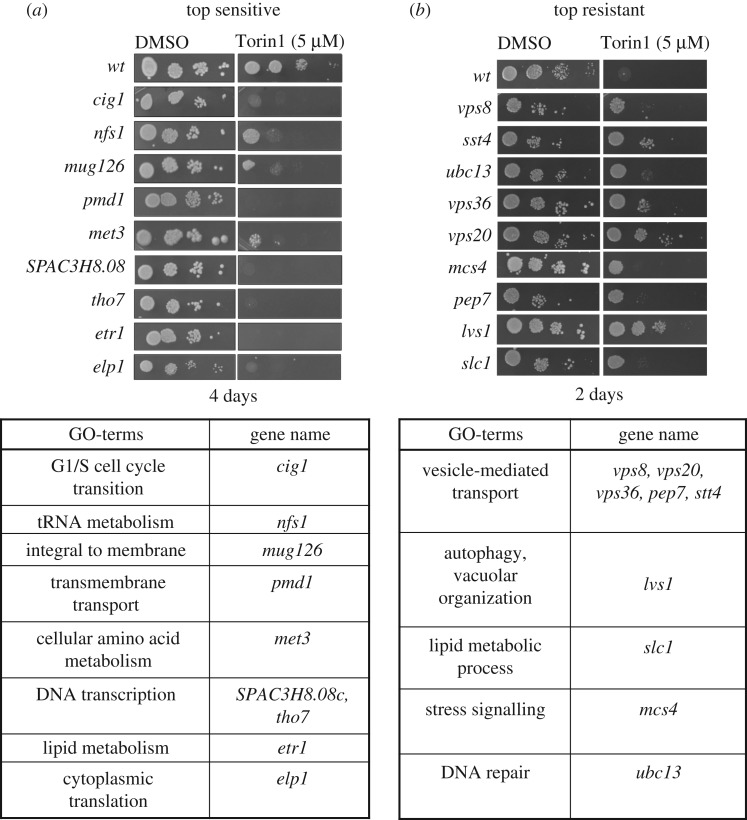
Growth of resistant and sensitive strains on Torin1. The relative (*a*) sensitivity and (*b*) resistance of genes most affected by Torin1 are shown with their GO-terms listed. The top sensitive deletion strains regulate diverse biological functions, while 50% of the top resistant strains regulate vesicle-mediated transport.

**Figure 5.**
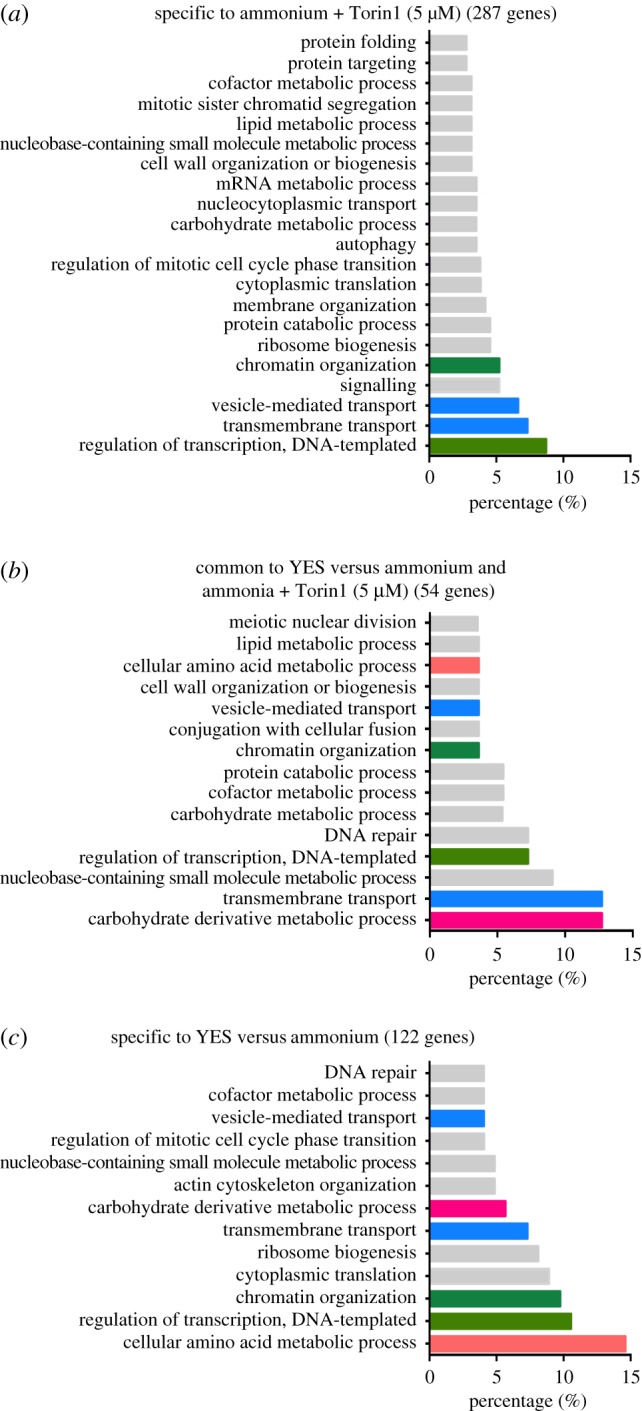
(*a*–*c*) Gene ontology analysis of all gene deletions that modify cell fitness on Torin1. Gene ontology analysis of the genes with significant EGIs from the three groups illustrated in the Venn diagram in figure 3*c*. The top 90% of biological functions mapped are shown.

The relative sensitivity and resistance of genes most affected by Torin1 are shown in figure 4. Interestingly, gene ontology analysis showed that the majority of strains, including *vps8*, *sst4*, *vps36*, *vps20*, *pep7* and *lvs1*, that displayed the highest levels of resistance to Torin1 regulate transport to vacuoles (lysosome in yeast) or vacuolar organization (figure 4*b*). These impacts may derive from the activation of TORC1 on vacuoles, such that changes in these TORC1 harbours could well increase local TORC1 activity to confer some resistance to the repressive impact of Torin1. By contrast, gene ontology analysis showed that the strains most sensitive to Torin1 included genes that regulate a diverse set of biological processes (figure 4*a*). Furthermore, as expected, gene ontology analysis of all 287 genes that impacted fitness when Torin1 was added identified a broad range of biological processes (figure 5*a*; electronic supplementary material, figure S4), with transcriptional regulation, transmembrane transport and chromatin organization displaying the broadest support. All processes known to be regulated by TOR signalling were identified in this screen. For example, two of the well-established biological processes regulated by TOR, autophagy and mRNA metabolism [41,42] were represented in the Torin1 treatment group (figure 5*a*).

Finally, of the 54 genes that when deleted impacted upon cell fitness on both EMM2 (ammonium) and when Torin1 was added to the media (figure 3*c*), genes regulating carbohydrate metabolic processes and transmembrane transport were identified (figure 5*b*). By contrast, genes regulating amino acid metabolic processes only impacted upon the comparisons between growth on EMM2 and rich medium (figure 5*c*).

### 2.3. Phloxine B enhances the sensitivity of the fitness screens

**Figure 6.**
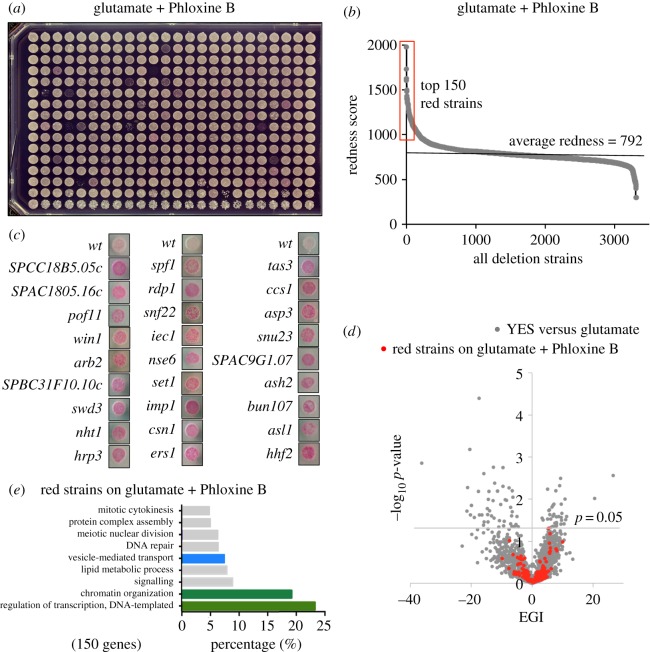
Phloxine B can enhance the sensitivity of a fitness screen. (*a*) To assess whether the vital red dye, Phloxine B, may be useful to enhance the sensitivity of fitness screens, Phloxine B was added to glutamate-based minimal media and the redness relating to each strain was established. (*b*) Average redness scores, based on four independent replicate experiments, are shown for all strains. (*c*) The redness of the indicated deletion strains compared with wild-type controls when grown on glutamate-based minimal media are shown. In electronic supplementary material, table S4, the average redness and significance for the top red strains are shown. (*d*) The identity of the top red strains is indicated on the volcano plot of EGIs of deletion strains grown on glutamate-based media (also shown in figure 2*b*). (*e*) Gene ontology analysis of the top 150 gene deletions strain that are red when grown on glutamate. The top 90% of biological functions of these genes are illustrated.

The screens described above all used standard rich or minimal media agar plates in accordance with well-established screening protocols. However, we wondered whether increased sensitivity might be achieved when performing global fitness screens. Dead and sick cells are unable to exclude the red Phloxine B dye. Therefore, colonies containing dying/sick cells will be a darker pink compared with healthy colonies [43]. Thus, the inclusion of Phloxine B may be a useful approach through which to identify additional strains that have an impact on cell fitness without reducing growth rate sufficiently to allow identification in the standard screens described above. We therefore added the vital dye Phloxine B to the minimal media EMMG (figure 6*a*) and monitored the intensity of red pigmentation of colonies in 4 independent replicates. The average red intensity score for all strains is shown in figure 6*b*, while average intensity and standard deviations for red strains are listed in electronic supplementary material, table S9. To confirm cell redness, 27 deletion strains with varying degree of redness (see electronic supplementary material, table S9) were compared with the white wild-type cells in figure 6*c*. This assay of Phloxine B uptake identified additional strains that did not lead to a significant reduction in cell fitness when tested on standard agar plates (shown by red dots in figure 6*d*). While it is unclear whether the addition of Phloxine B itself affects the sensitivity, we once more identified transcriptional regulation and chromatin organization (figure 6*e*) as major contributors to redness in this cohort, suggesting that the inclusion of Phloxine B might be useful to increase the sensitivity of future genome-wide screens of cell fitness.

## 3. Discussion

### 3.1. Chromatin organization and transcription

We have used global quantitative fitness analysis (QFA) to determine how the nutrient environment and reduced TOR signalling impact upon the fitness of *S. pombe* strains from which non-essential genes have been deleted, to determine the environmental genetic interaction (EGI) for each fission yeast gene deletion strain. Strains deleted for genes regulating transcription and chromatin organization were highly represented in all screens described above. In total, 53 genes regulating chromatin organization and 33 genes regulating transcription had altered fitness in one or more of the environments tested (tables 1 and 2).

**Table 1.** TOR and environmental impact on chromatin organization and regulation. List of all gene deletions identified in the five screens described in this study (figures 2, 3 and 6) with a role in chromatin organization and regulation. The EGIs for all genes identified on Torin1 (T), ammonium (A), glutamate (G) and proline (P) screens were ≤ −3.00 EGI *p* = 0.05 or ≥ +3.00 EGI *p* = 0.05. Only strains from the top 150 red strains on glutamate Phloxine B are included. Torin1 (T), ammonium (A), glutamate (G), proline (P); glutamate Phloxine B (red).

chromatin organization and regulation			
sensitive or relative resistance (nutrient or Torin1)	systematic ID	gene name	description
assembly			
sensitive (red)	SPBC8D2.03c	*hhf2*	histone H4 h4.2
resistant (A)	SPAC1834.03c	*hhf1*	histone H4 h4.1
sensitive (T)	SPAC1834.04	*hht1*	histone H3 h3.1
sensitive (red)	SPBC36B7.08c	*ccp1*	CENP-A nucleosome disassembly protein Ccp1
sensitive (T)	SPBC1703.14c	*Top1*	DNA topoisomerase I
remodelling/histone modification/silencing			
resistant (T)	SPBP22H7.05c	*abo2*	ATPase with bromodomain protein (predicted)
sensitive (T)	SPAC23H4.12	*alp13*	MRG family Clr6 histone deacetylase complex subunit Alp13
sensitive (red)	SPAC13G7.07	*arb2*	argonaute binding protein 2
sensitive (red)	SPAC23D3.09	*arp42*	SWI/SNF and RSC complex subunit Arp42
sensitive (red)	SPAC664.02c	*arp8*	Ino80 complex actin-like protein Arp8
resistant (T)	SPAC1071.06	*arp9*	SWI/SNF and RSC complex subunit Arp9
sensitive (red)	SPBC13G1.08c	*ash2*	Ash2-trithorax family protein
sensitive (T)	SPAC9E9.10c	*cbh1*	kinetochore protein, CENP-B homolog Cbh1
sensitive (red)	SPAC18G6.02c	*chp1*	chromodomain protein Chp1
sensitive (red)	SPCC663.12	*cid12*	poly(A) polymerase Cid12
sensitive (T)	SPBC800.03	*clr3*	histone deacetylase (class II) Clr3
sensitive (red)	SPBC428.08c	*clr4*	histone H3 lysine methyltransferase Clr4
sensitive (red)	SPBC215.03c	*csn1*	COP9/signalosome complex subunit Csn1
sensitive (T)	SPCC548.05c	*dbl5*	ubiquitin-protein ligase E3 Dbl5
sensitive (G, P, A)	SPCC188.13c	*dcr1*	dicer
sensitive (red)	SPAC17H9.10c	*ddb1*	damaged DNA-binding protein Ddb1
sensitive (red)	SPCC1393.05	*ers1*	RNA-silencing factor Ers1
resistant (T)	SPAC25A8.01c	*fft3*	SMARCAD1 family ATP-dependent DNA helicase Fft3
sensitive (G, P, A)	SPAC1952.05	*gcn5*	SAGA complex histone acetyltransferase catalytic subunit Gcn5
resistant (A)	SPBC31F10.13c	*hip1*	hira protein, histone chaperone Hip1
sensitive (T)	SPBC21D10.12	*hop1*	BAR adaptor protein Hob1
sensitive (red)	SPAC3G6.01	*hrp3*	ATP-dependent DNA helicase Hrp3
sensitive (red)	SPAC144.02	*iec1*	Ino80 complex subunit Iec1
sensitive (T)	SPAC25H1.02	*jmj1*	histone demethylase Jmj1 (predicted)
sensitive (red)	SPAC17G8.13c	*mst2*	histone acetyltransferase Mst2
sensitive (G, A)	SPBC28F2.10c	*ngg1*	SAGA complex subunit Ngg1/Ada3
sensitive (red)	SPAC10F6.08c	*nht1*	Ino80 complex HMG box subunit Nht1
sensitive (A)	SPAC664.03	*paf1*	RNA polymerase II-associated Paf1 complex (predicted)
sensitive (red)	SPCC613.12c	*raf1*	CLRC ubiquitin E3 ligase complex specificiy factor Raf1/Dos1
sensitive (red)	SPAC6F12.09	*rdp1*	RNA-directed RNA polymerase Rdp1
sensitive (red)	SPCC11E10.08	*rik1*	silencing protein Rik1
sensitive (red)	SPCC1259.07	*rxt3*	transcriptional regulatory protein Rxt3
resistant (T)	SPCC663.11	*saf1*	splicing-associated factor Saf1
sensitive (T)	SPAC31G5.18c	*sde2*	silencing defective protein Sde2
resistant (T)	SPCC306.04c	*set1*	histone lysine methyltransferase Set1
resistant (A)	SPBC16D10.07c	*sir2*	Sirtuin family histone deacetylase Sir2
sensitive (T)	SPCC1620.14c	*snf22*	ATP-dependent DNA helicase Snf22
sensitive (T)	SPBC26H8.09c	*snf59*	SWI/SNF complex subunit Snf59
resistant (G, P)	SPAC3H1.12c	*snt2*	Lid2 complex PHD finger subunit Snt2
resistant (P)	SPBC30B4.04c	*sol1*	SWI/SNF complex subunit Sol1
sensitive (T)	SPAC25G10.01	*SPAC25G10.01*	RNA-binding protein involved in histone acetylation
resistant (A)	SPCC594.05c	*spf1*	Set1C PHD Finger protein Spf1
sensitive (P, A)	SPCC1393.02c	*spt2*	non-specific DNA binding protein Spt2 (predicted)
resistant (A)	SPAC23H3.05c	*swd1*	Set1C complex subunit Swd1
resistant (A)	SPBC354.03	*swd3*	WD repeat protein Swd3
sensitive (red)	SPBC83.03c	*tas3*	RITS complex subunit 3
sensitive (G, P, A)	SPBP16F5.03c	*tra1*	SAGA complex phosphatidylinositol pseudokinase Tra1
resistant (P)	SPBC29A3.05	*vps71*	Swr1 complex subunit Vps71

**Table 2.** TOR and environmental impact on transcriptional regulation. List of all gene deletions identified in the five screens described in this study (figures 2, 3 and 6) with a role in transcriptional regulation. The EGIs for all genes identified in Torin1 (T), ammonium (A), glutamate (G) and proline (P) screens were ≤ −3.00 EGI *p* = 0.05 and ≥ +3.00 EGI *p* = 0.05. Only strains from the top 150 red strains on glutamate Phloxine B are included. Torin1 (T), ammonium (A), glutamate (G), proline (P), glutamate Phloxine B (red).

regulators of transcription			
sensitive or relative resistance (nutrient or Torin1)	systematic ID	gene name	description
sensitive (T)	SPCC1494.10	*adn3*	transcription factor Adn3
sensitive (red)	SPCC736.08	*cbf11*	CBF1/Su(H)/LAG-1 family transcription factor Cbf11
resistant (A)	SPCC1223.13	*cbf12*	CBF1/Su(H)/LAG-1 family transcription factor Cbf12
sensitive (red)	SPAC1556.08c	*cbs2*	AMP-activated protein kinase gamma subunit cbs2
sensitive (T)	SPBC1683.13c	*cha4*	transcription factor Cha4 (predicted)
sensitive (red)	SPAC1851.03	*ckb1*	CK2 family regulatory subunit Ckb1
sensitive (T)	SPBP23A10.14c	*ell1*	RNA polymerase II transcription elongation factor SpELL
sensitive (T)	SPBC36.07	*elp1*	elongator subunit Elp1 (predicted)
resistant (T)	SPCC1902.01	*gaf1*	transcription factor Gaf1
sensitive (T)	SPBPB8B6.04c	*grt1*	transcription factor Grt1 (predicted)
sensitive (T)	SPAC23C4.12	*hhp2*	serine/threonine protein kinase Hhp2
sensitive (red)	SPAC6B12.05c	*ies2*	Ino80 complex subunit Ies2
resistant (T)	SPBC317.01	*mbx2*	MADS-box transcription factor Pvg4
sensitive (red)	SPAC5D6.05	*med18*	mediator complex subunit Med18
sensitive (red)	SPAC821.07c	*moc3*	transcription factor Moc3
sensitive (T)	SPCC4G3.15c	*not2*	CCR4-Not complex NOT box subunit Not2
sensitive (T, red)	SPAC2F7.11	*nrd1*	RNA-binding protein Nrd1
sensitive (G, P)	SPBC725.11c	*php2*	CCAAT-binding factor complex subunit Php2
resistant (T)	SPBC3B8.02	*php5*	CCAAT-binding factor complex subunit Php5
sensitive (G, T)	SPAC32A11.03c	*phx1*	stationary phase-specific homeobox transcription factor Phx1
sensitive (T, red)	SPBC17G9.05	*rct1*	RRM-containing cyclophilin regulating transcription Rct1
sensitive (red)	SPAC6G9.10c	*sen1*	Nrd1 complex ATP-dependent 5′ to 3′ DNA/RNA helicase Sen1
resistant (G, A)	SPAC16.05c	*sfp1*	transcription factor Sfp1 (predicted)
sensitive (T)	SPAC105.03c	*SPAC105.03c*	transcription factor (predicted)
resistant (T)	SPAC22H10.11c	*SPAC22H10.11c*	TOR signalling pathway transcriptional corepressor Crf1
sensitive (T)	SPAC25B8.11	*SPAC25B8.11*	transcription factor (predicted)
sensitive (T)	SPAC3H8.08c	*SPAC3H8.08c*	transcription factor (predicted)
resistant (T)	SPBC1773.16c	*SPBC1773.16c*	transcription factor, zf-fungal binuclear cluster type (predicted)
sensitive (red)	SPBC530.08	*SPBC530.08*	membrane-tethered transcription factor (predicted)
sensitive (T)	SPCC320.03	*SPCC320.03*	transcription factor (predicted)
sensitive (A)	SPAC20H4.03c	*tfs1*	transcription elongation factor TFIIS
sensitive (red)	SPBC19C7.02	*ubr1*	N-end-recognizing protein, UBR ubiquitin-protein ligase E3 Ubr1
sensitive (red)	SPAC25G10.03	*zip1*	transcription factor Zip1

Modulation of chromatin organization to change transcriptional activation or repression is widely used as a major control across eukaryotes [44]. Chromatin structure in yeast and mammals is dynamically altered by covalent modification on histones by ATP-dependent chromatin remodelling activity [45]. For example, the Swi/Snf ATP-dependent chromatin remodelling activity operates in concert with the SAGA complex to set the accessibility for DNA transcription, replication and repair [46,47]. Deletion of *snf59*, *arp9*, *arp42* and *sol1*, each of which encodes Swi/Snf components (table 1), conferred sensitivity or resistance to Torin1 or the reduction in nitrogen quality arising from growth on proline. They also compromised fitness when fitness on glutamate medium was assessed with the vital stain Phloxine B. Consistently, deletion of *gcn5* and *tra1*, key components of the SAGA complex, also reduces fitness below levels seen on rich medium when cells are grown on any of the minimal media (table 1). These observations are consistent with previous reports of the role for the SAGA complex controlling the transcriptional programme upon nutrient starvation [48,49], and thus serve as further validation for our screen for mutants that alter fitness on minimal media.

Chromatin organization and regulation are intrinsically linked to ribosomal DNA (rDNA) transcription and so are key to the ribosomal biogenesis that drives cell growth and proliferation [50]. Nutrient availability modulates ribosomal biogenesis to couple growth and proliferation to environmental cues [51]. Importantly, ribosome biogenesis is regulated through TOR control of [52,53] RNA polymerase I activity [54]. Indeed, the reduction in ribosomal biogenesis [55,56], nucleolar size, chromatin remodelling and histone modification seen upon rapamycin treatment in yeast is also seen in mammalians [57,58]. Clr3 is one of the most prominent histone deacetylases that controls transcriptional silencing to regulate mating in yeast [59]. Clr3 also influences chromatin re-organization in the early response to nitrogen starvation in yeast [60]. Consistently, we found loss of Clr3 conferred sensitivity to Torin1. Furthermore, Dicer (Dcr1 in *S. pombe*) is required for RNA polymerase II release at transcription termination site to maintain genomic stability and rDNA copy number [61]. Dicer acts alongside argonaute and Rdp1 to process long double-stranded RNA (dsRNA) in the generation of the siRNA that mediates DNA silencing [62,63]. Consistent with earlier reports, we found that deletion of *dcr1* reduced fitness on minimal media with glutamate, proline or ammonium [64], while removal of *rdp1* compromised fitness on minimal glutamate media. The RNA-induced transcriptional silencing (RITS) complex and the Argonaute siRNA chaperone (ARC) are required for heterochromatin gene silencing at the centromeres [65]. A component of the ARC, Arb2 and a component of the RITS, Tas3, were identified as red when cultured in minimal media with glutamate and Phloxine B, suggesting that cell fitness on this minimal media is reduced when components of these complexes are deleted.

The histone deacetylase, Sir2, silences rDNA transcription compromising chromatin accessibility [66]. The strain lacking Sir2 was resistant to culture in minimal media with ammonium. This indicates that Sir2 may support an enhancement of ribosomal biogenesis to drive growth and proliferation. The Set1 complex, comprising Set1, Swd1 and Swd3 [67], also repressed rDNA transcription by methylating histone H3 at lysine 4 [68]. We found that set1, swd1 and swd3 mutants were resistant to Torin1 or ammonium (minimal media), which indicate that inactivation of these genes may allow rDNA transcription by counteracting the effect of Torin1 or ammonium on TOR complex inhibition. However, methylation at H3 lys 4 is also known to induce euchromatin structure to promote transcription [69]. In this scenario, Set1 complex mutations should inhibit transcription, to render these mutants sensitive. One possibility is that H3 lys 4 methylation may induce the expression of gene(s) that repress rDNA transcription, hence ribosomal biogenesis.

Interestingly, several of the transcription regulators that we find to play key roles in supporting cell fitness (table 2) have previously been associated with TOR signalling or nutrient sensing, including the TORC1-regulated sexual differentiation modulator Gaf1 [33,70], the TOR signalling pathway transcriptional corepressor CRF1 [36], the regulator of nitrogen use Cha4 [71], and Php2 and php5, which are both regulators of the cellular response to nitrogen starvation [72]. Finally, Mbx2 regulates invasive growth and flocculation, which are also physiological responses associated with nutrient starvation [73].

### 3.2. Transmembrane transport

Cells respond to alterations in their nutrient environment by regulating nutrient transporters and receptors [74]. Studies, in both yeast and mammalian cells, have determined that TOR regulates nutrient uptake [28,75,76]. For example, in *S. cerevisiae*, amino acid permeases such as Can1 are regulated in response to nutrient-availability. This process is conserved in both *S. pombe* and mammalian cells [77,78]. Consistently, we identified trans-membrane transporters as being required for normal fitness on minimal media and when TOR signalling is reduced (figures 2 and 5; table 3). A total of 26 identified transporters of vitamins, amino acids, minerals, sugars, proton and ions along with 16 known regulator of transmembrane transport were required for normal cell fitness.

**Table 3.** Regulators of transmembrane transport. List of all gene deletions identified in the five screens described in this study (figures 2, 3 and 6) with a role in transmembrane transport. The EGIs for all genes identified in Torin1 (T), ammonium (A), glutamate (G) and proline (P) screens were ≤ −3.00 EGI *p* = 0.05 or ≥ +3.00 EGI *p* = 0.05. Only strains from the top 150 red strains on glutamate Phloxine B are included. Torin1 (T), ammonium (A), glutamate (G), proline (P), glutamate Phloxine B (red).

regulators of transmembrane transport			
sensitive or relative resistance (nutrient or Torin1)	systematic ID	gene name	description
sensitive (T)	SPBC1604.11	*atp17*	F0-ATPase subunit F (predicted)
sensitive (T)	SPAC23C4.11	*atp18*	F0-ATPase subunit J (predicted)
sensitive (G, P, A)	SPBC18H10.16	*can1*	arginine transmembrane transporter Can1
resistant (P)	SPAC1399.03	*fur4*	uracil permease
sensitive (G, P, A)	SPAC1952.05	*gcn5*	SAGA complex histone acetyltransferase catalytic subunit Gcn5
sensitive (T)	SPAC1F8.01	*ght3*	hexose transmembrane transporter Ght3
sensitive (T)	SPCC1235.13	*ght6*	hexose transmembrane transporter Ght6
sensitive (A, T)	SPAC12G12.12	*gms2*	UDP-galactose transmembrane transporter Gms2 (predicted)
sensitive (T)	SPAC30D11.06c	*hfl1*	Lazarus1 family transmembrane transporter
resistant (G, P)	SPBC2G2.01c	*liz1*	pantothenate transmembrane transporter Liz1
sensitive (T)	SPAPB8E5.03	*mae1*	malic acid transport protein Mae1
sensitive (A, T)	SPBC25B2.02c	*mam1*	M-factor transmembrane transporter Mam1
sensitive (T)	SPBC9B6.09c	*mdl1*	mitochondrial peptide-transporting ATPase
sensitive (A, P)	SPBC25H2.08c	*mrs2*	mitochondrial magnesium ion transmembrane transporter Mrs2
resistant (P, T)	SPAC5D6.09c	*mug86*	acetate transmembrane transporter (predicted)
sensitive (T)	SPAC9G1.04	*oxa101*	mitochondrial inner membrane translocase Oxa101
resistant (G, P, A)	SPAC27F1.08	*pdt1*	Nramp family manganese ion transmembrane transporter
resistant (G, A)	SPAC22F8.04	*pet1*	phosphoenolpyruvate transmembrane transporter Pet1
sensitive (T)	SPAC22E12.01	*pet3*	phosphoenolpyruvate transmembrane transporter Pet3
sensitive (G, P, T)	SPCC553.03	*pex1*	AAA family ATPase Pex1 (predicted)
resistant (A, T)	SPBC8E4.01c	*pho84*	inorganic phosphate transmembrane transporter (predicted)
sensitive (T)	SPCC663.03	*pmd1*	leptomycin transmembrane transporter Pmd1
resistant (A, P, T)	SPAC11G7.02	*pub1*	HECT-type ubiquitin-protein ligase E3 Pub1
sensitive (A, T)	SPBC13E7.11	*rbd1*	mitochondrial rhomboid protease (predicted)
resistant (P)	SPAC11D3.08c	*SPAC11D3.08c*	amino acid permease, unknown 1 (predicted)
sensitive (T)	SPAC1399.02	*SPAC1399.02*	transmembrane transporter (predicted)
resistant (T)	SPAC14C4.07	*SPAC14C4.07*	transmembrane transporter (predicted)
resistant (P, T)	SPAC16A10.01	*SPAC16A10.01*	ThrE amino acid transmembrane transporter family protein
sensitive (A)	SPAC17H9.08	*SPAC17H9.08*	mitochondrial coenzyme A transmembrane transporter (predicted)
resistant (T)	SPAC6C3.06c	*SPAC6C3.06c*	P-type ATPase, calcium transporting (predicted)
sensitive (T)	SPBC1271.10c	*SPBC1271.10c*	transmembrane transporter (predicted)
resistant (G, P)	SPBC1652.02	*SPBC1652.02*	APC amino acid transmembrane transporter (predicted)
sensitive (A, T)	SPBC1703.13c	*SPBC1703.13c*	mitochondrial inorganic phosphate transmembrane transporter
resistant (A)	SPBC887.02	*SPBC887.02*	ClC chloride channel (predicted)
sensitive (T)	SPBC947.06c	*SPBC947.06c*	spermidine family transmembrane transporter (predicted)
resistant (T)	SPCC553.12c	*SPCC553.12c*	transmembrane transporter (predicted)
sensitive (G, P, A, T)	SPCC794.03	*SPCC794.03*	amino acid permease (predicted)
sensitive (G, P, A)	SPCPB1C11.03	*SPCPB1C11.03*	cysteine transmembrane transporter (predicted)
sensitive (A)	SPAC22F3.13	*tsc1*	hamartin
sensitive (T)	SPAC630.13c	*tsc2*	tuberin, GTPase activator Tsc2
resistant (G, A)	SPAC1B3.16c	*vht1*	vitamin H transmembrane transporter Vht1
resistant (T)	SPAP8A3.03	*zip3*	ZIP zinc transmembrane transporter Zip3 (predicted)

### 3.3. Autophagy

Five genes (*atg2*, *atg5*, *atg12*, *atg14* and *atg16*) that confer sensitivity to Torin1 treatment are classified as essential regulators of autophagy (table 4). It is well established that nutrient starvation and TOR signalling regulate autophagy. Two modes of autophagy—microautophagy and macroautophagy—are triggered in response to nutrient starvation throughout eukaryotes [37,79]. Autophagy degrades and recycles cytoplasmic components including organelles to generate amino acids and other essential molecules to extend survival when nutrient is limited [37,80,81]. While carbon and essential amino acid starvation both induce autophagy, nitrogen starvation triggers the most rapid induction of autophagy, in a TOR-dependent manner [41]. When nutrient supply is bountiful, TORC1 activation inhibits autophagy by phosphorylating Atg13, to prevent binding to Atg1, to block the induction of autophagy [38,81]. Nutrient starvation, TORC1 inhibition by rapamycin or mutation of Atg13 blocks Atg13 dephosphorylation, which induces the association with Atg1 and the induction of autophagy [38]. Furthermore, nitrogen starvation or rapamycin-induced inhibition of TORC1 stimulates the transcription of one of the essential *atg* genes, *atg*14, in a manner that is reliant upon the transcription factor *Gln*3 [82]. However, amino acid starvation can also regulate autophagy independently of the TORC1 signalling pathway [83,84]. Torin1 affects both TORC1 and TORC2. Interestingly, TORC2 has been reported to induce autophagy in response to amino acid starvation, but not nitrogen [85]. Along with these 5 ‘*atg*’ genes, three other regulators of autophagy—irs4, ctl1 and SPBC1711.11—also conferred sensitivity to Torin1 [86–88]. Thus, our findings are in agreement with numerous reports that autophagy is required for cell growth in response to reduced TOR signalling [79,89–91]. It was previously established that lipid synthesis is crucial for autophagosome biogenesis and is increased during starvation [92]. We also find that lipid metabolism is one of the biological pathways that is affected by growth in minimal media with poor nitrogen source (ammonium) and Torin1 treatment (figure 5).

**Table 4.** Regulators of autophagy. List of genes deletion identified in the Torin1-based screen with a role in autophagy. The EGIs for all genes listed were ≤ −3.00 EGI *p* = 0.05 or ≥ +3.00 EGI *p* = 0.05.

regulators of autophagy			
sensitive or relative resistance to Torin1	systematic ID	gene name	description
sensitive	SPAC1783.06c	*atg12*	autophagy-associated ubiquitin-like protein modifier Atg12
sensitive	SPAC25A8.02	*atg14*	autophagy-associated protein Atg14
sensitive	SPBC405.05	*atg16*	autophagy-associated protein Atg16
sensitive	SPAC458.06	*atg1803*	autophagy-associated WD repeat protein Atg18c
sensitive	SPBC31E1.01c	*atg2*	autophagy-associated protein Atg2
sensitive	SPBC4B4.10c	*atg5*	autophagy-associated protein Atg5
sensitive	SPCC1682.11c	*ctl1*	protein implicated in autophagy Ctl1
sensitive	SPAC1687.09	*irs4*	autophagy/CVT pathway ENTH/VHS domain protein Irs4
resistant	SPBC28E12.06c	*lvs1*	autophagy-associated protein, beige protein homologue, Lvs1
sensitive	PBC1711.11	*SPBC1711.11*	autophagy-associated protein (predicted)
resistant	SSPCC1322.14c	*vtc4*	vacuolar transporter chaperone (VTC) complex subunit

### 3.4. Nucleocytoplasmic transport

Genes involved in nucleocytoplasmic transport were also required for fitness when TOR signalling was inhibited (figure 5*a*). The nuclear import receptor Msn5 and the nucleoporins Nup61, Nup82 and Nup184 were among genes known to control nucleocytoplasmic transport. TOR signalling has previously been linked to the nuclear localization of transcription factors in yeast [93] and mammalian cells [94], and of ribosomal proteins [95]. Whether this is also regulated at the level of nuclear pores remains unclear.

### 3.5. Genes with human orthologues displaying a robust altered response to torin1

Deletion of 62 genes conserved in humans resulted in strong sensitivity or resistance to torin1 (EGI ≤ −6 and an EGI ≥6). Of these, 28 gene deletions conferred sensitivity and 34 gene deletions conferred resistance to Torin1 (table 5). The majority of the biological processes that these genes regulate have previously directly or indirectly been linked to TOR signalling. However, the cellular detoxification pathway and microtubule cytoskeletal nucleation process have not previously been linked to TOR. In addition, 29 of these 62 genes are novel links to TOR signalling [9–11,33,53,96–99] (table 5). Glo2 regulates cellular detoxification, which is associated with cellular ageing [10,100,101]. Deletion of *glo2* conferred resistance to Torin1, as evidenced by an EGI = 6.74 (table 5). PNKD, the human orthologue of Glo2, is abundant in the brain and mutation of this gene is found in paroxysmal nonkinesigenic dyskinesia disorder (PNKD). This rare disorder is characterized by involuntary movement stimulated by stress, alcohol and caffeine [102]. This is interesting considering that caffeine has been shown to decrease TOR signalling and subsequently increase lifespan in *S. pombe* [9]. An *alp16* deletion also conferred resistance to torin1 in this study (table 5). Alp16 regulates microtubule cytoskeletal nucleation [103], which has not previously been linked to TOR signalling. Interestingly, TUBGCP6, the human orthologue of Alp16, is commonly mutated or amplified in anaplastic large cell lymphoma (ALCL) [104].

**Table 5.** Genes with human orthologues, whose deletion confers robust Torin1 resistance or sensitivity. List of gene deletions identified in the Torin1-based screen. The EGIs for all genes listed were ≤ −6.00 EGI *p* = 0.05 or ≥ +6.00 EGI *p* = 0.05.

top Torin1-sensitive and -resistant genes with human orthologues						
					previously linked to TOR signalling	
EGI	P	pombe gene	function	human orthologue	function	gene
−10.45	0.001074	*cig1*	regulation of G1/S transition of mitotic cell cycle	CCNB1-3	✓	✓
−9.911	0.0002309	*nfs1*	mitochondrial [2Fe-2S] assembly and tRNA modification	NFS1	✓	NO
−9.405	1.12 × 10^−6^	*pmd1*	leptomycin transmembrane transporter	ABCB1	✓	✓
−8.785	0.01503	*tho7*	mRNA export from nucleus and transcription elongation	THOC7	✓	NO
−8.737	2.28 × 10^−7^	*etr1*	fatty acid biosynthetic process	MECR	✓	✓
−8.148	0.01914	*elp1*	tRNA metabolic process and cytoplasmic translation	ELP1	✓	✓
−8.247	0.04037	*shm2*	amino acid metabolic process	SHMT1	✓	✓
−8.041	0.0006646	*dal2*	nitrogen cycle metabolic process	ALLC	✓	✓
−7.846	0.001419	*lsm8*	mRNA cis splicing and rRNA processing	LSM8	✓	NO
−7.805	0.01662	*SPAC3H5.08c*	unknown human WDR44 downstream effector for RAB11	WDR44	✓	✓
−7.764	0.02212	*SPCC16C4.10*	carbohydrate metabolic process	PGLS	✓	✓
−7.565	0.03079	*msn5*	nucleocytoplasmic transport	XPO5	✓	NO
−7.185	0.02894	*SPCC31H12.03c*	nucleocytoplasmic transport	HNRNPUL1	✓	✓
−7.077	0.0003582	*hht1*	chromatin organization	HIST3H3	✓	NO
−6.83	0.001063	*mug161*	mRNA cis splicing	CWF19L1	✓	NO
−6.757	0.04475	*atg12*	autophagy	ATG12	✓	NO
−6.735	0.0001817	*hfl1*	transmembrane transport	TMEM184B	✓	✓
−6.641	0.000892	*apl1*	vesicle-mediated transport	AP1B1	✓	NO
−6.546	2.89 × 10^−5^	*gim3*	protein folding	PFDN4	✓	NO
−6.468	0.0006939	*atg5*	autophagy	ATG5	✓	✓
−6.453	0.0008298	*rpl1603*	cytoplasmic translation	RPL13A	✓	✓
−6.426	0.03551	*bun107*	ubiquitin-binding protein, regulator of deubiquitination	WDR48	✓	✓
−6.278	0.01108	*pac10*	protein folding	VBP1	✓	NO
−6.254	0.0006155	*ncs1*	calcium-mediated signalling	NCS1	✓	NO
−6.236	0.0003854	*hhp2*	casein kinase	CSNK1D	✓	✓
−6.161	3.04 × 10^−5^	*hrd3*	ubiquitin-dependent ERAD pathway	SEL1 L	✓	NO
−6.137	0.0008063	*SPBC1347.08c*	DNA replication	RNASEH2B	✓	NO
−6.021	0.0002738	*SPAP8A3.13c*	vacuolar import/degradation protein	GID4	✓	NO
6.023	0.04231	*pub3*	ubiquitin-dependent protein catabolic process	NEDD4	✓	✓
6.048	0.03804	*saf1*	mRNA cis splicing and chromatin silencing at centromere	WBP11	✓	✓
6.068	0.008319	*set1*	chromatin organization	SETD1A	✓	✓
6.283	0.04117	*SPBC1703.08c*	folic acid-containing compound biosynthetic process	MTHFS	✓	NO
6.294	0.0441	*bch1*	vesicle-mediated transport	TTC17	✓	NO
6.362	0.02402	*pnk1*	DNA repair	PNKP	✓	✓
6.434	0.03031	*snr1*	amino acid metabolic process	HIBCH	✓	NO
6.55	0.005573	*dis2*	protein serine/threonine phosphatase	PPP1CA	✓	✓
6.581	0.007766	*dis32*	mRNA metabolic process	DIS3L2	✓	✓
6.669	0.04109	*rtf1*	DNA replication	TTF1	✓	✓
6.735	0.01626	*glo2*	cellular detoxification	PNKD	NO	NO
6.863	0.002253	*trm1*	tRNA metabolic process	TRMT1	✓	✓
6.92	0.02525	*zrt2*	ER transmembrane transport	SLC39A13	✓	✓
6.985	0.03703	*rpl3702*	cytoplasmic translation	RPL37	✓	✓
7.003	0.04962	*alp16*	Microtubule organization centre, microtubule nucleation	TUBGCP6	NO	NO
7.254	0.01333	*trm112*	tRNA metabolic process	TRMT112	✓	✓
7.448	0.00116	*gaf1*	DNA transcription	GATA6	✓	✓
7.778	0.001511	*naa30*	protein maturation	NAA30	✓	NO
7.812	0.0006453	*fft3*	chromatin organization	SMARCAD1	✓	✓
7.976	0.01377	*fsv1*	vesicle-mediated transport	STX8	✓	NO
8.004	0.007904	*rpl1702*	cytoplasmic translation	RPL17	✓	✓
8.169	0.001191	*SPCC1827.03c*	acetyl-CoA metabolic process	ACSF3	✓	NO
8.998	0.000964	*bro1*	vesicle-mediated transport	PTPN23	✓	✓
8.999	0.003054	*SPAC19B12.11c*	ribosome biogenesis	ZNF593	✓	✓
9.368	0.01898	*SPBC15D4.13c*	signalling	ASCC1	✓	NO
9.391	0.0399	*wis4*	signalling	MAP3K4	✓	✓
10.17	0.001589	*slc1*	lipid metabolic process	AGPAT1	✓	NO
10.37	0.004282	*lvs1*	autophagy	WDFY3	✓	NO
10.55	0.007674	*pep7*	vesicle-mediated transport	RBSN	✓	✓
11.1	0.01796	*vps20*	vesicle-mediated transport	CHMP6	✓	NO
11.96	0.03154	*vps36*	vesicle-mediated transport	VPS36	✓	NO
12.07	3.08E-05	*ubc13*	DNA repair	UBE2N	✓	NO
12.83	0.02699	*sst4*	vesicle-mediated transport	STAM	✓	NO
15.7	0.04138	*vps8*	vesicle-mediated transport	VPS8	✓	✓

The impact of gene deletion on cell fitness presented here was based on four independent experiments and a *p*-value of 0.05 or below. Limited overlap was observed between the genes identified in screens that previously assessed the impact of rapamycin (TORC1 specific inhibitor) on rich media or the simultaneous addition of rapamycin and caffeine to rich media [9–11] compared with our Torin1 alone on minimal media (electronic supplementary material, figure S6). These distinctions probably reflect the difference between the media used in the previous studies (YES media) and the minimal media (EMM2) used in this study, as TOR signalling is very sensitive to the nutrient environment [1,5,8,105]. In fact, wild-type *S. pombe* cells are not sensitive to rapamycin on rich YES media, whereas wild-type cells stop proliferation when Torin1 is added to both YES and minimal media [6,9,106]. In addition, the impact of the supplementary addition of caffeine is unclear. Finally, Torin1 also inhibits TORC2 and, importantly, TORC1 and TORC2 regulate the activity of each other [1,5,27,107,108].

## 4. Conclusion

In response to nutrient limitation or a reduction in TOR signalling, we find that transcription, chromatin organization/regulation, and transmembrane and vesicle-mediated transport play key roles in supporting fitness. It is likely that alteration in chromatin regulation, presumably to facilitate changes in the transcriptional regulation, along with changes in transport of nutrients, ions and vitamins, aids cell adaptation to limited nutrient environments, a key feature of cancer cells within solid tumours. The majority of the biological processes identified in this study have previously been linked to TOR signalling; however, to our knowledge cellular detoxification and microtubule nucleation are novel TOR-related processes. In addition, half of the conserved genes, whose deletion strains showed robust altered response to Torin1 (table 5), represent novel links to TOR. Thus, these genes provide further insight into TOR-regulated biology. The definition of the gene sets described here will help guide targeted interrogation of a range of TOR-regulated biology to expand our understanding of this vital signalling network that impinges upon so many biological processes. Finally, we show that the inclusion of Phloxine B might be useful to increase the sensitivity of future genome-wide screens of cell fitness. This might become particularly useful if redundancy is likely to be an issue.

## 5. Material and methods

### 5.1. Yeast cell cultures and reagents

The fission yeast deletion library version 3 was used (Bioneer). Cell growth and maintenance protocol was according to the culture methods described previously [19]. Media used in this study include ‘rich’ media (YES) and Edinburgh minimal media (EMM) supplemented with different sources of nitrogen: 20 mM L-glutamate (EMMG), 20 mM proline (EMMP) or 93.5 mM ammonium (EMM2). Phloxine B (2′,4′,5′,7′-tetrabromo-4,5,6,7-tetrachlorofluorescein disodium salt or cyanosine) was added at 1 g l^−1^ and Torin1 was added at a concentration of 5 µM. For cell growth assays, cells were grown exponentially for 48 h to 2.5 × 10^6^ cells ml^−1^. A 10-fold dilution series was spotted on indicated plates.

### 5.2. Western blotting

TCA precipitation protocol was followed for total protein extracts from [109]. Antibodies were used at 1/500 anti-PK (V5). Alkaline phosphatase-coupled secondary antibodies were used for all blots followed by direct detection with NBT/BCIP (VWR) substrates on PVDF membranes.

### 5.3. Deletion library screen

Quantitative fitness analysis workflow was used to compare cell growth spotted onto solid agar plates made of different media [14]. Briefly, up to 96 yeast strains were cultured to saturation in the 200 µl liquid YES media in a 96-well culture dish in a temperature-controlled incubator (30°C). A sterile pin tool (V&P Scientific) in combination with a Beckman Coulter FX robot was used to spot the saturated liquid cultures on to solid agar plates made of YES, EMMG, EMMP, EMM2, EMM2 with 5 µM torin1, EMMG with 1 g l^−1^ Phloxine B in 384 format. After spotting, plates were transferred to an S&P Robotic automatic imager housed in a temperature-controlled room at 30°C. A Canon EOS Rebel Ti 35 mm DSLR camera captured an image of each agar plate at 5184 × 3456 px resolution immediately following the plates being placed in the imager after spotting to obtain a zero time point. Thereafter, images were captured every two hours for the duration of the experiment and growth curves were generated for the individual strain.

## Supplementary Material

Supplementary Tables 1-9

## Data accessibility

All relevant data are included within the article or in the electronic supplementary material.
