# Supplementary material for: The contribution of non-essential Schizosaccharomyces pombe genes to fitness in response to altered nutrient supply and target of rapamycin activity
Source: Open Biol. 2018 May 2;8(5):180015. doi: 10.1098/rsob.180015 (PMC5990653; doi:10.1098/rsob.180015)
Supplement: Supplementary Tables 1-9 [file rsob180015supp1.pdf]

Supplementary Figure 1: Scatterplots of cells fitness on rich vs minimal media.

Cell fitness for all strains grown in minimal media was established and plotted against their fitness in rich media. All cell fitness calculations were based on 4 independent screens. The environmental and genetic interaction (EGI) were calculated for each strain based on the deviation from the solid line, overlaid as the line of equal fitness [14,15]. The established EGIs were used in figure 2A, B, C.

Supplementary Figure 2:

List of genes that regulate cell fitness on all three minimal media tested with their biological roles included.

Supplementary Figure 3: Scatterplots of cells fitness on ammonium with DMSO vs Torin1.

Cell fitness for all strains grown on ammonium with 5uM Torin1 was established and plotted against their fitness on ammonium with vehicle control (DMSO). All cell fitness calculations were based on 4 independent screens. The environmental and genetic interaction (EGI) were calculated for each strain based on the deviation from the solid line, overlaid as the line of equal fitness [14,15]. The established EGIs were used in figure 3B.

Supplementary Figure 4: List of all Go-terms associated with altered fitness on Torin1.

Supplementary Figure 5:

List of 54 genes that regulate cell fitness on the minimal ammonium and when TOR signaling is further reduced by 5 uM Torin1 (also shown on figure 3 & 5).

Supplementary Figure 6: Overlap with previous screen of gene deletions that are sensitive to rapamycin.

The impact of gene deletion on cell fitness presented here where based on 4 independent repeat experiments and a p-value of 0.05 or below. We identified limited overlap between the genes identified in screens using rapamycin or combined rapamycin/caffeine on rich media. Importantly, in our study, the sensitivity to Torin1 were tested on minimal media, whilst previous screens used rich media. Furthermore limited overlap between the two screens published using rapamycin were seen.

Supplementary Table 1-3: Lists of the EGIs for all strains grown in EMM2, EMMG and EMMP compared to YES.

Supplementary Table 4-6: Lists of gene deletions that showed significantly different fitness (EGIs) in the minimal nutrient environments (EMM2, EMMG and EMMP).

Supplementary Table 7: Lists of the EGIs for all strains grown in EMM2 with 5 uM Torin1 compared to vehicle control (DMSO).

Supplementary Table 8: Lists of gene deletions that showed significantly different fitness (EGIs) when 5 uM Torin1 was added to EMM2

Supplementary Table 9: Lists of gene deletions that were red on glutamate + phloxin B added to EMM2

Lie et al. Supplementary Figure 1

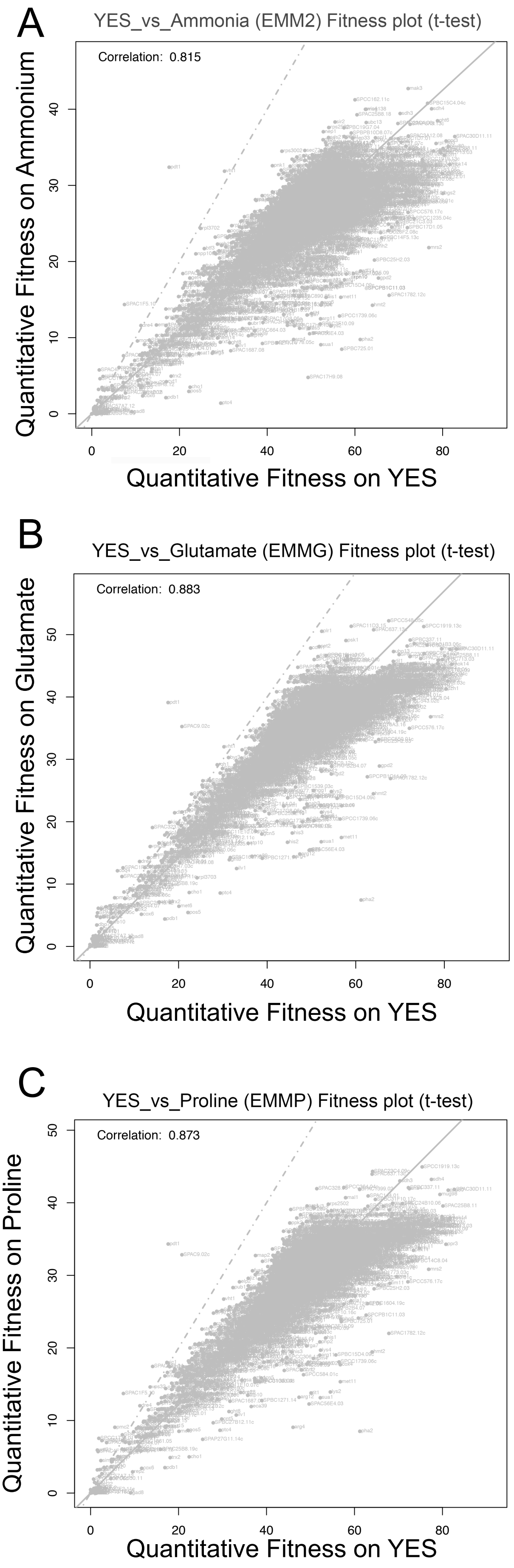

Lie et al. Supplementary Figure 2

Genes regulating cell fitness on all minimal media tested

Systematic_ID	Name	Description
SPAC31A2.09c	*apm4*	AP-2 adaptor complex mu subunit Apm4 (predicted)
SPAC4G9.09c	*arg11*	N-acetyl-gamma-glutamyl-phosphate reductase/acetylglutamate kinase
SPBC428.05c	*arg12*	argininosuccinate synthase Arg12
SPBC725.14	*arg6*	acetylglutamate synthase Arg6
SPAC4F10.14c	*btf3*	nascent polypeptide-associated complex beta subunit
SPBC18H10.16	*can1*	arginine transmembrane transporter Can1
SPBC16A3.16	*coa5*	mitochondrial inner membrane protein involved in cytochrome c oxidase assembly Coa5
SPCC188.13c	*dcr1*	dicer
SPAC1952.05	*gcn5*	SAGA complex histone acetyltransferase catalytic subunit Gcn5
SPAC23D3.04c	*gpd2*	glycerol-3-phosphate dehydrogenase Gpd2
SPBC11B10.02c	*his3*	histidinol-phosphate aminotransferase imidazole acetol phosphate transaminase His3
SPBC21H7.07c	*his5*	imidazoleglycerol-phosphate dehydratase His5
SPBC2G5.06c	*hmt2*	sulfide-quinone oxidoreductase
SPBC1685.15c	*klp6*	kinesin-8 family plus-end microtubule motor Klp6
SPAC343.16	*lys2*	homoaconitate hydratase Lys2
SPCC584.01c	*met10*	sulfite reductase NADPH flavoprotein subunit (predicted)
SPBC15D4.09c	*met3*	cystathionine gamma-synthase Met3
SPBC19C7.01	*mni1*	exon-exon junction complex disassembly factor Mni1 (predicted)
SPBC4F6.08c	*mrpl39*	mitochondrial ribosomal protein subunit L39 (predicted)
SPBC19C2.06c	*mug124*	Schizosaccharomyces pombe specific protein
SPBC19C7.12c	*omh1*	alpha-1,2-mannosyltransferase Omh1
SPAC27F1.08	*pdt1*	Nramp family manganese ion transmembrane transporter
SPBC30D10.16	*pha2*	phrenate dehydratase
SPBC23G7.08c	*rga7*	RhoGAP, GTPase activating protein Rga7
SPAC637.10c	*rpn10*	19S proteasome regulatory subunit Rpn10
SPAC144.17c	*SPAC144.17c*	6-phosphofructo-2-kinase (predicted)
SPAC1782.12c	*SPAC1782.12c*	DUF423 protein
SPAC27E2.03c	*SPAC27E2.03c*	Obg-Like ATPase (predicted)
SPAC27F1.05c	*SPAC27F1.05c*	aminotransferase class-III, unknown specificty
SPAC56E4.03	*SPAC56E4.03*	aromatic aminotransferase (predicted)
SPAC683.02c	*SPAC683.02c*	zf-CCHC type zinc finger protein (predicted)
SPAC6B12.14c	*SPAC6B12.14c*	conserved fungal protein
SPBC3B8.05	*SPBC3B8.05*	diphthamide biosynthesis protein Dph1 (predicted)
SPCC320.03	*SPCC320.03*	transcription factor (predicted)
SPCC794.03	*SPCC794.03*	amino acid permease (predicted)
SPCPB1C11.03	*SPCPB1C11.03*	cysteine transmembrane transporter (predicted)
SPBC27.08c	*sua1*	sulfate adenylyltransferase
SPAC343.15	*tit1*	tRNA isopentenyltransferase Tit1
SPBP16F5.03c	*tra1*	SAGA complex phosphatidylinositol pseudokinase Tra1
SPAC1250.03	*ubc14*	ubiquitin conjugating enzyme E2 Ubc14 (predicted)

Lie et al. Supplementary Figure 3

A

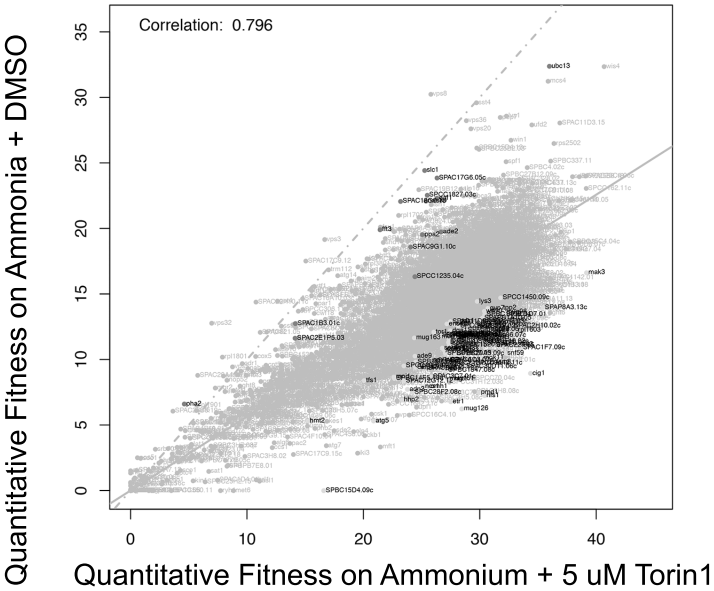

Lie et al. Supplementary Figure 4

Genes regulating cell fitness on Ammonium and on Ammonia + 5uM Torin1

Systematic_ID	Name	Description
SPCPB16A4.03c	*ade10*	bifunctional IMP cyclohydrolase/ phosphoribosylaminoimidazolecarboxamideformyltransferase
SPAC23H4.12	*alp13*	MRG family Clr6 histone deacetylase complex subunit Alp13
SPAC5H10.01	*dgc1*	mitochondrial D-glutamate cyclase Dgc1 (predicted)
SPAC458.04c	*dli1*	meiotic dynein intermediate light chain Dli1/Dil1
SPCC4B3.11c	*fra3*	mitochondrial Fe-S cluster transfer protein (predicted)
SPAC5H10.11	*gmh1*	alpha-1,2-galactosyltransferase Gmh1 (predicted)
SPAC12G12.12	*gms2*	Golgi UDP-galactose transmembrane transporter Gms2 (predicted)
SPAC23D3.04c	*gpd2*	glycerol-3-phosphate dehydrogenase Gpd2
SPAC23C4.12	*hhp2*	serine/threonine protein kinase Hhp2
SPAC1834.04	*hht1*	histone H3 h3.1
SPBC2G5.06c	*hmt2*	sulfide-quinone oxidoreductase
SPBC28F2.08c	*hrd3*	Hrd1 ubiquitin ligase complex subunit (predicted)
SPBC25B2.02c	*mam1*	M-factor transmembrane transporter Mam1
SPBC15D4.09c	*met3*	cystathionine gamma-synthase Met3
SPBC19C7.01	*mni1*	exon-exon junction complex disassembly factor, human partner of Y14 and mago ortholog
SPBC4F6.08c	*mrpl39*	mitochondrial ribosomal protein subunit L39 (predicted)
SPBC25B2.04c	*mtg1*	mitochondrial GTPase involved in translation Mtg1 (predicted)
SPBC19C2.06c	*mug124*	Schizosaccharomyces pombe specific protein
SPBC359.06	*mug14*	adducin, involved in actin cytoskeleton organization
SPBC23G7.06c	*nvj2*	nucleus-vacuole junction protein Nvj2
SPBC19C7.12c	*omh1*	alpha-1,2-mannosyltransferase Omh1
SPAC17D4.01	*pex7*	peroxin-7 (predicted)
SPBC30D10.16	*pha2*	phrenate dehydratase
SPBC14F5.13c	*pho8*	vacuolar membrane alkaline phosphatase (predicted)
SPBC8E4.01c	*pho84*	inorganic phosphate transmembrane transporter (predicted)
SPAC32A11.03c	*phx1*	stationary phase-specific homeobox transcription factor Phx1
SPAC23C11.04c	*pnk1*	DNA kinase/phosphatase Pnk1
SPBC16H5.07c	*ppa2*	serine/threonine protein phosphatase Ppa2
SPBC337.04	*ppk27*	calcium/calmodulin-dependent protein kinase Ppk27 (predicted)
SPAC11G7.02	*pub1*	HECT-type ubiquitin-protein ligase E3 Pub1
SPBC13E7.11	*rbd1*	mitochondrial rhomboid protease (predicted)
SPAC343.18	*rfp2*	SUMO-targeted ubiquitin-protein ligase subunit Rfp2
SPBC337.03	*rhn1*	RNA polymerase II transcription termination factor homolog
SPAC22F8.07c	*rtf1*	replication termination factor Rtf1
SPAC3C7.01c	*sac12*	inositol polyphosphate phosphatase (predicted)
SPCC70.06	*sac32*	nuclear export factor Sac32 (predicted)
SPBPB2B2.02	*say1*	ER sterol deacetylase Say1 (predicted)
SPAC4D7.01c	*sec71*	Sec7 domain protein, ARF GEF (predicted)
SPAC11E3.04c	*ubc13*	ubiquitin conjugating enzyme E2 Ubc13
SPCC1620.02	*wtf23*	wtf element Wtf23
SPAC144.17c		6-phosphofructo-2-kinase (predicted)
SPAC17A2.11		Schizosaccharomyces pombe specific protein
SPAC186.07c		hydroxyacid dehydrogenase (predicted)
SPAC1B3.01c		uracil phosphoribosyltransferase (predicted)
SPAC27E2.03c		Obg-like ATPase, human OLA1 ortholog (predicted)
SPBC13E7.07		Schizosaccharomyces specific protein
SPBC1703.13c		mitochondrial inorganic phosphate transmembrane transporter (predicted)
SPBC17D1.05		Schizosaccharomyces specific protein
SPBC19C7.05		vesicle-mediated transport protein (predicted)
SPBC21C3.03		mitochondrial membrane ABC1 kinase family protein, unknown role, human ADCK2 ortholog
SPBPB7E8.02		PSP1 family protein
SPCC320.03		transcription factor (predicted)
SPCC4B3.06c		NADPH-dependent FMN reductase (predicted)
SPCC794.03		amino acid permease (predicted)

Lie et al. Supplementary Figure 5

<u>All Go-terms associated with altered fitness on Torin1</u>

Actin Cytoskeleton Organization (GO:0030036)
Ascospore Formation (GO:0030437)
Autophagy (GO:0006914)
Carbohydrate Derivative Metabolic Process (GO:1901135)
Carbohydrate Metabolic Process (GO:0005975)
Cell Adhesion (GO:0007155)
Cell Wall Organization Or Biogenesis (GO:0071554)
Cellular Amino Acid Metabolic Process (GO:0006520)
Chromatin Organization (GO:0006325)
Cofactor Metabolic Process (GO:0051186)
Conjugation With Cellular Fusion (GO:0000747)
Cytoplasmic Translation (GO:0002181)
Detoxification (GO:0098754)
DNA Recombination (GO:0006310)
DNA Repair (GO:0006281)
DNA Replication (GO:0006260)
Establishment Or Maintenance Of Cell Polarity (GO:0007163)
Generation Of Precursor Metabolites And Energy (GO:0006091)
Lipid Metabolic Process (GO:0006629)
Meiotic Nuclear Division (GO:0140013)
Membrane Organization (GO:0061024)
Microtubule Cytoskeleton Organization (GO:0000226)
Mitochondrial Translation (GO:0032543)
Mitochondrion Organization (GO:0007005)
Mitotic Cytokinesis (GO:0000281)
Mitotic Sister Chromatid Segregation (GO:0000070)
mRNA Metabolic Process (GO:0016071)
Nitrogen Cycle Metabolic Process (GO:0071941)
Nucleobase-Containing Small Molecule Metabolic Process (GO:0055086)
Nucleocytoplasmic Transport (GO:0006913)
Peroxisome Organization (GO:0007031)
Protein Catabolic Process (GO:0030163)
Protein Complex Assembly (GO:0006461)
Protein Folding (GO:0006457)
Protein Glycosylation (GO:0006486)
Protein Maturation (GO:0051604)
Protein Modification By Small Protein Conjugation Or Removal (GO:0070647)
Protein Targeting (GO:0006605)
Regulation Of Mitotic Cell Cycle Phase Transition (GO:1901990)
Regulation Of Transcription, DNA-Templated (GO:0006355)
Ribosome Biogenesis (GO:0042254)
Signaling (GO:0023052)
SnoRNA Metabolic Process (GO:0016074)
Telomere organization (GO:0032200)
Transcription, DNA-templated (GO:0006351)
Transmembrane transport (GO:0055085)
tRNA metabolic process (GO:0006399)
Vesicle-mediated transport (GO:0016192)
Vitamin metabolic process (GO:0006766)

Lie et al. Supplementary Figure 6

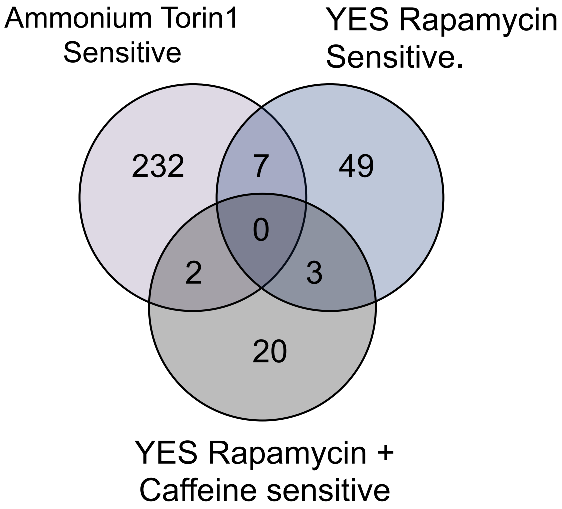

Supplementary Table 1

R package version: 0.0-10
Summary type: mean
Test type: t-test   Based on 4 independent repeat experimens

Control medium: YE5S
Control screen ID: QFA0002
Control libraries: PDLV4_384
Query medium: EMMP_Proline
Query screen ID: QFA0002

##					Proline Fitness	Yes_Fitness		
ORF	Gene	P	EGI	(p) -log10	mean	mean	Proline SE	YES SE
SPBC30D10.16	pha2	0.009921	-28.74	2.00344455	8.528	61.22	5.233	5.982
SPBC2G5.06C	hmt2	0.003323	-19.5	2.478469659	19.46	64	4.869	1.366
SPAC1782.12C	SPAC1782.12c	0.01958	-19.29	1.708187313	22	67.83	7.167	0.81
SPAC343.10	met11	0.1361	-19.23	0.866141875	15.34	56.78	15.62	0.7237
SPAC343.16	lys2	0.0212	-19.22	1.673664139	13.96	54.5	4.361	4.932
SPBC215.08C	arg4	0.04913	-18.93	1.308653236	9.083	46.01	10.32	3.308
SPBC27.08C	sua1	0.0001228	-18.64	3.910801633	13.16	52.23	2.404	0.8829
SPAC56E4.03	SPAC56E4.03	0.02158	-17.96	1.66594856	12.27	49.67	6.007	4.445
SPAC1296.02	cox4	0.2821	-16.87	0.549596914	17.71	56.81	21.06	3.062
SPAC343.15	tit1	0.03145	-16.71	1.50237935	13.79	50.1	3.014	4.729
SPCC1739.06C	SPCC1739.06c	0.07662	-16.31	1.115657852	18.09	56.5	10.36	1.92
SPBC25H2.08C	mrs2	0.04679	-15.96	1.329846955	30.84	76.87	8.255	1.163
SPBC428.05C	arg12	0.03305	-15.63	1.480828536	13.22	47.39	5.236	4.482
SPBC15D4.09C	SPBC15D4.09c	0.03669	-14.9	1.435452288	19.06	55.79	6.983	0.8561
SPBC19G7.07C	ppr3	0.2041	-14.86	0.690156995	34.31	80.77	15.09	1.738
SPCC576.17C	SPCC576.17c	0.1607	-14.8	0.793984123	29.12	72.13	13.15	1.676
SPBC725.14	arg6	0.02053	-14.12	1.687611051	17.83	52.48	5.701	1.303
SPCPB1C11.03	SPCPB1C11.03	4.17E-05	-13.59	4.3801765	24.53	62.62	1.552	0.8357
SPCC584.01C	SPCC584.01c	0.001121	-13.16	2.950394387	16.32	48.42	2.388	0.6009
SPAC57A7.08	pzh1	0.07174	-13.09	1.144238628	35.84	80.38	7.987	0.9504
SPCC1620.14C	snf22	0.134	-12.92	0.872895202	24.13	60.86	10.42	2.884
SPAC4G9.09C	arg11	0.04802	-12.74	1.318577844	19	52.14	6.823	2.614
SPBC14C8.04	SPBC14C8.04	0.04959	-12.54	1.304605892	31.97	73.12	6.74	1.296
SPCC553.03	pex1	0.008138	-12.49	2.089482314	29.86	69.57	3.885	2.164
SPBC106.05C	tim11	0.05858	-12.48	1.232250633	28.93	68.02	7.179	1.541
SPAC31A2.09C	apm4	0.004956	-12.26	2.304868702	18.55	50.61	3.403	1.923
SPCC188.13C	dcr1	0.009146	-12.23	2.038768803	16.97	47.96	3.696	2.319
SPBC1604.19C	SPBC1604.19c	0.2714	-12.22	0.566390157	26.12	62.97	14.82	2.356
SPAC683.02C	SPAC683.02c	0.02356	-12.14	1.627824714	17.82	49.21	4.905	0.7231
SPBC1105.02C	lys4	0.05107	-12.13	1.291834142	19.69	52.27	6.526	1.038
SPBC21H7.07C	his5	0.008406	-12.09	2.075410614	18.04	49.5	3.883	1.111
SPCC4B3.12	set9	0.2345	-12	0.629857153	31.93	72.15	13.24	1.782
SPCC63.04	mok14	0.2366	-11.81	0.62598526	37.9	81.65	13.1	1.89
SPAC25H1.02	jmj1	0.07412	-11.75	1.130064589	33.75	74.74	7.332	1.185
SPBC713.03	SPBC713.03	0.1953	-11.68	0.709297757	36.77	79.57	11.55	1.457
SPAC890.05	SPAC890.05	0.1503	-11.27	0.823041019	17.57	47.39	6.073	5.573
SPAC589.08C	dam1	0.2737	-11.26	0.562725203	31.73	70.61	13.76	1.957
SPBC582.09	pex11	0.08463	-11.19	1.072475659	33.48	73.37	7.441	1.787
SPAC23D3.04C	gpd2	0.02525	-11.12	1.597738618	28.66	65.35	4.694	2.377
SPAC25G10.05C	his1	0.1023	-11.11	0.990124366	21.4	53.4	6.397	4.245
SPBC1703.09	SPBC1703.09	0.2174	-11.1	0.66274046	36.36	77.95	11.7	1.06
SPBC25H2.03	SPBC25H2.03	0.4028	-11.08	0.394910538	28.16	64.46	18.6	2.106
SPBC725.01	SPBC725.01	0.2274	-11.08	0.64320954	23.63	57.02	11.97	1.982
SPAC24C9.07C	bgs2	0.1962	-10.95	0.707300997	37.76	80.01	10.86	1.436
SPBC1271.14	SPBC1271.14	0.3163	-10.93	0.499900808	12.76	38.92	14.05	4.631
SPAC1A6.03C	SPAC1A6.03c	0.1079	-10.92	0.966978555	35.94	76.98	7.969	0.9249
SPBC725.11C	php2	0.01193	-10.9	1.923359556	20.83	52.11	2.627	2.402
SPCC1235.13	ght6	0.08143	-10.8	1.089215565	37.19	78.83	7.073	1.689
SPBC3B8.02	php5	0.242	-10.7	0.616184634	23.95	56.91	12.05	1.692
SPCC16A11.08	atg20	0.07258	-10.54	1.139183036	31.67	69.33	6.583	1.443
SPAC15A10.13	ppk3	0.2514	-10.52	0.599634727	38.12	79.9	12.15	1.637
SPBC2G2.05	rpl1603	0.1544	-10.52	0.811352704	37.28	78.51	9.14	0.7601
SPAC227.14	SPAC227.14	0.1598	-10.42	0.796423225	32.33	70.22	9.239	1.545
SPBC428.02C	eca39	0.09244	-10.3	1.034140063	11.95	36.54	0.4296	4.224
SPBC8E4.02C	SPBC8E4.02c	0.1754	-10.08	0.755970411	35.84	75.43	9.362	1.927

SPAC1002.19	urg1	0.2377	-10.07	0.623970818	37.77	78.58	11.24	0.7896
SPCC550.01C	SPCC550.01c	0.03745	-10.04	1.426548178	29.65	65.19	4.861	0.8981
SPAPYUG7.04C	rpb9	0.1533	-9.966	0.814457845	31.22	67.65	8.619	0.5636
SPAC977.12	SPAC977.12	0.02662	-9.896	1.574791949	16.92	44.05	4.352	1.976
SPAC3C7.01C	SPAC3C7.01c	0.09678	-9.889	1.014214382	35.57	74.67	6.943	1.507
SPAC1687.09	SPAC1687.09	0.2636	-9.814	0.579054594	36.81	76.58	11.71	1.449
SPBC23G7.08C	rga7	0.03094	-9.602	1.509479691	20.29	49.09	3.654	2.574
SPAC57A10.10C	sla1	0.4203	-9.56	0.37644061	26.51	59.24	16.54	2.923
SPAC29A4.13	SPAC29A4.13	0.1441	-9.529	0.841336019	34.56	72.41	8.014	1.56
SPAC1B3.06C	SPAC1B3.06c	0.17	-9.486	0.769551079	36.84	76.09	8.683	1.508
SPBC15C4.04C	SPBC15C4.04c	0.24	-9.402	0.619788758	37.22	76.59	10.53	1.518
SPBP35G2.07	ilv1	0.09201	-9.397	1.036164969	10.77	33.13	3.065	3.925
SPBC1734.08	hse1	0.4337	-9.374	0.362810578	27.21	60.09	17.04	1.049
SPAC144.17C	SPAC144.17c	0.03227	-9.317	1.491201035	30.96	66.15	4.296	2.004
SPAC4A8.03C	ptc4	0.3092	-9.242	0.509760515	8.667	29.42	10.79	5.024
SPBC1711.13	his2	0.3407	-9.198	0.467627866	17.94	44.57	10.72	6.033
SPAC25B8.11	SPAC25B8.11	0.2648	-9.186	0.577082019	39.57	80.09	10.93	1.91
SPAC9E9.10C	cbh1	0.343	-9.146	0.46470588	33.52	70.08	13.24	1.982
SPAC57A10.06	mug15	0.1832	-9.107	0.737074531	36.73	75.3	8.685	1.394
SPAC22E12.01	SPAC22E12.01	0.2428	-9.088	0.614751318	37.64	76.75	10.27	1.171
SPAC20G4.02C	fus1	0.3226	-9.084	0.491335637	22.06	51.16	12.19	3.19
SPBC16A3.02C	SPBC16A3.02c	0.1568	-9.078	0.804653942	36.78	75.33	7.957	1.658
SPAC9G1.04	oxa101	0.1312	-9.036	0.882066165	34.49	71.5	7.262	1.277
SPBC1683.02	SPBC1683.02	0.1949	-9.006	0.710188161	32.43	68.06	8.869	1.715
SPAC12G12.09	SPAC12G12.09	0.3446	-9.003	0.462684727	26.04	57.56	12.73	3.291
SPBC887.10	mcs4	0.461	-8.979	0.336299075	32.71	68.47	17.36	1.874
SPBC28F2.08C	SPBC28F2.08c	0.09604	-8.946	1.017547849	31.15	65.86	6.261	1.378
SPBC1604.01	mug158	0.1699	-8.936	0.769806621	36.94	75.36	8.137	1.902
SPAPB8E5.03	mae1	0.2124	-8.861	0.672845488	37.18	75.63	9.199	1.386
SPCC70.09C	mug9	0.1827	-8.85	0.738261453	35.9	73.51	8.432	1.062
SPCC663.08C	SPCC663.08c	0.1971	-8.802	0.705313376	35.82	73.3	8.749	1.287
SPAC11G7.06C	mug132	0.2024	-8.79	0.693789492	35.38	72.55	8.861	1.513
SPAC3F10.09	SPAC3F10.09	0.06092	-8.787	1.215240105	22.93	52.1	5.033	0.685
SPCC1235.04C	SPCC1235.04c	0.1325	-8.736	0.877784122	36.01	73.5	7.025	0.7109
SPBC32H8.11	mei4	0.4239	-8.707	0.372736583	29.35	62.51	15.43	1.288
SPCC18B5.07C	nup61	0.09901	-8.678	1.004320939	31.23	65.56	6.054	0.434
SPBC1711.11	SPBC1711.11	0.1211	-8.671	0.916855857	33.97	70.05	6.695	1.752
SPCC757.03C	SPCC757.03c	0.1361	-8.667	0.866141875	34.83	71.45	7.077	0.9212
SPBC1289.16C	cao2	0.1796	-8.662	0.745693668	34.95	71.64	8.006	2.373
SPBC337.16	cho1	0.1761	-8.651	0.754240644	5.003	22.43	7.993	2.044
SPBC543.02C	SPBC543.02c	0.219	-8.647	0.659555885	34.62	71.06	9.16	1.034
SPCC777.07	omh3	0.1194	-8.635	0.922995673	35.92	73.19	6.644	1.51
SPBC4F6.08C	mrpl39	0.03472	-8.634	1.459420283	30.18	63.76	2.991	2.501
SPCC569.07	SPCC569.07	0.1411	-8.602	0.850472986	35.59	72.59	7.153	1.572
SPAC890.07C	rmt1	0.2071	-8.587	0.683819901	37.59	75.85	8.789	1.193
SPBC1773.03C	SPBC1773.03c	0.4281	-8.561	0.368454772	30.34	63.91	15.3	1.525
SPAC30D11.11	SPAC30D11.11	0.2864	-8.535	0.543026986	41.84	82.74	10.66	2.19
SPCC794.01C	SPCC794.01c	0.1255	-8.457	0.901356274	35.07	71.5	6.628	0.7709
SPCC306.11	SPCC306.11	0.3669	-8.451	0.435452288	18.72	44.63	12.76	2.77
SPAC2F7.11	nrd1	0.05064	-8.447	1.295506303	30.41	63.82	4.639	1.607
SPCC16A11.03C	SPCC16A11.03c	0.1779	-8.434	0.749824052	34.49	70.5	7.918	0.9817
SPAC23A1.15C	sec20	0.08516	-8.422	1.069764347	31.75	65.98	5.614	1.325
SPCC663.02	wtf14	0.4439	-8.362	0.352714855	28.92	61.24	15.59	0.9093
SPBC11B10.02C	his3	0.03831	-8.357	1.416687848	19.46	45.69	4.19	1.163
SPBP22H7.06	SPBP22H7.06	0.1193	-8.308	0.923359556	31.96	66.14	6.392	1.257
SPCC1840.09	SPCC1840.09	0.412	-8.296	0.385102784	22.66	50.85	13.87	3.163
SPBC1685.08	cti6	0.1069	-8.287	0.971022295	34.48	70.26	6.067	1.18
SPAC4G8.11C	atp10	0.2342	-8.265	0.630413109	13.49	35.74	6.951	4.594
SPBPB2B2.02	mug180	0.08281	-8.242	1.081917215	35.58	71.98	5.427	1.193
SPBC19C2.09	sre1	0.5338	-8.227	0.272621431	20.47	47.14	18.41	4.506
SPAC16.04	dus3	0.1769	-8.182	0.752272167	29.47	61.84	7.654	0.7482
SPBC18E5.01	SPBC18E5.01	0.1579	-8.179	0.80161787	35	70.93	7.163	1.762
SPAC23H4.10C	thi4	0.02012	-8.175	1.696372024	18.37	43.6	2.1	2.07
SPAC22G7.04	ubp13	0.517	-8.157	0.286509457	33.57	68.54	18.08	2.359
SPAP27G11.14C	SPAP27G11.14c	0.1267	-8.111	0.897223385	7.403	25.48	3.694	3.793
SPBC1778.05C	SPBC1778.05c	0.6064	-8.07	0.217240807	17.66	42.26	21.45	6.418
SPCC1393.08	SPCC1393.08	0.2062	-8.046	0.685711339	15.36	38.45	7.875	2.672
SPBC17D1.05	SPBC17D1.05	0.2226	-8.013	0.65247484	35.78	71.94	8.501	1.779
SPBC21B10.08C	SPBC21B10.08c	0.2274	-7.992	0.64320954	36.71	73.42	8.665	1.014

SPAC227.18	lys3	0.1937	-7.983	0.712870379	32.29	66.15	7.854	1.242
SPBC685.02	exo5	0.1692	-7.964	0.771599641	34.71	70.09	7.133	2.147
SPBC17G9.08C	cnt5	0.1098	-7.953	0.95939766	10.2	29.82	3.153	3.539
SPAC3A12.12	atp11	0.3213	-7.907	0.493089274	20.96	47.42	10.57	2.782
SPCC548.06C	ght8	0.385	-7.903	0.41453927	11.24	31.45	12.04	3.685
SPCC4G3.05C	mus81	0.2131	-7.9	0.67141655	37.96	75.33	8.218	1.229
SPAC105.03C	SPAC105.03c	0.3594	-7.892	0.444421927	37.47	74.51	11.8	2.184
SPAC4H3.06	SPAC4H3.06	0.2531	-7.836	0.596707855	36.88	73.45	9.043	1.648
SPBC3B9.08C	mnh1	0.1926	-7.808	0.715343717	38.19	75.56	7.665	0.942
SPBC776.15C	kgd2	0.383	-7.788	0.416801226	25.39	54.5	11.9	3.428
SPAC4F8.15	itr1	0.6176	-7.775	0.209292713	25.75	55.07	20.59	7.477
SPAC1952.05	gcn5	0.003954	-7.759	2.402963335	15.86	38.8	2.039	1.174
SPBC543.03C	pku80	0.3118	-7.757	0.506123889	41.73	81.29	10.21	2.467
SPAC2H10.02C	SPAC2H10.02c	0.115	-7.747	0.93930216	35.86	71.62	5.842	1.585
SPCC306.08C	SPCC306.08c	0.4778	-7.744	0.320753855	29.42	61.05	15.55	2.04
SPAPB2B4.07	SPAPB2B4.07	0.3776	-7.735	0.422968014	25.45	54.51	11.92	2.777
SPBC6B1.05C	atg7	0.5057	-7.705	0.296107046	27.38	57.63	16.56	2.337
SPAC31G5.04	SPAC31G5.04	0.4565	-7.697	0.340559218	15.4	37.94	13.01	5.401
SPBC14F5.13C	SPBC14F5.13c	0.1472	-7.697	0.83209219	32.53	66.08	6.51	1.635
SPBC18H10.16	can1	0.04356	-7.613	1.360912129	31.84	64.81	3.952	1.527
SPBC19C2.02	pmt1	0.2285	-7.562	0.641113796	34.07	68.38	8.118	1.869
SPBC30D10.10C	tor1	0.1974	-7.494	0.704652852	33.41	67.19	7.459	1.011
SPBC354.09C	SPBC354.09c	0.5028	-7.493	0.298604731	28.69	59.44	16.18	0.7849
SPBC13E7.11	SPBC13E7.11	0.05463	-7.484	1.262568799	31.52	64.07	4.223	1.075
SPBC577.11	SPBC577.11	0.2443	-7.477	0.612076533	34.61	69.14	8.242	2.337
SPAC27F1.03C	uch1	0.5476	-7.476	0.261536561	33.68	67.6	17.96	2.296
SPBC18H10.11C	ppr2	0.2744	-7.464	0.561615893	15.29	37.37	7.221	4.38
SPAC521.03	SPAC521.03	0.4156	-7.421	0.381324461	29.31	60.34	12.48	2.956
SPAC13G6.07C	rps601	0.2136	-7.408	0.670398752	37.4	73.59	7.72	1.121
SPAC11G7.01	SPAC11G7.01	0.4305	-7.336	0.366026844	29.7	60.84	13.19	1.251
SPBP8B7.23	SPBP8B7.23	0.2662	-7.325	0.574791949	36.26	71.59	8.812	0.84
SPBP8B7.04	mug45	0.4367	-7.302	0.359816808	27.93	57.86	12.99	2.836
SPBC27B12.11C	SPBC27B12.11c	0.221	-7.285	0.655607726	9.699	27.9	7.579	2.102
SPBC106.17C	cys2	0.08897	-7.268	1.050756409	15.52	37.44	4.913	0.925
SPAC821.07C	moc3	0.5288	-7.258	0.276708554	25.25	53.39	15.8	4.408
SPBC23G7.14	SPBC23G7.14	0.2161	-7.246	0.665345233	35.05	69.47	7.561	1.537
SPCP1E11.02	ppk38	0.162	-7.186	0.790484985	35.65	70.37	6.409	0.6466
SPAC144.02	iec1	0.4349	-7.18	0.361610592	30.4	61.74	13.02	1.457
SPBC15D4.12C	mug98	0.3071	-7.175	0.512720184	41.18	79.42	9.375	2.138
SPBC21C3.03	SPBC21C3.03	0.181	-7.168	0.742321425	35.21	69.6	6.75	1.543
SPAC2F3.12C	plp1	0.08351	-7.16	1.078261516	25.12	53.02	3.246	2.748
SPACUNK4.10	SPACUNK4.10	0.5572	-7.144	0.253988892	32.3	64.79	17.4	3.056
SPBC1198.11C	reb1	0.3991	-7.134	0.398918272	22.61	48.86	11.58	2.632
SPAC664.02C	arp8	0.407	-7.13	0.390405591	24.23	51.51	11.54	3.275
SPBC359.06	mug14	0.1154	-7.105	0.937794191	34.06	67.61	5.382	1.023
SPAP7G5.04C	lys1	0.45	-7.095	0.346787486	19.54	43.76	11.24	5.457
SPBC16G5.13	ptf2	0.35	-7.094	0.455931956	28.14	57.87	10.3	2.215
SPAP8A3.13C	SPAP8A3.13c	0.1296	-7.086	0.887394998	37.11	72.59	5.625	1.567
SPAC24C9.15C	spn5	0.2003	-7.086	0.698319051	35.96	70.71	7.101	1.204
SPAC3A12.08	SPAC3A12.08	0.1524	-7.035	0.817015033	36.9	72.17	6.058	1.468
SPCC1235.15	dga1	0.2224	-7.003	0.652865217	37.03	72.33	7.461	1.304
SPAC24C9.08	SPAC24C9.08	0.1414	-6.958	0.849550591	33.87	67.06	5.352	2.418
SPBC28F2.10C	ngg1	0.09815	-6.925	1.008109696	23.8	50.47	4.385	2.303
SPBC337.09	erg28	0.4519	-6.922	0.344957659	13.87	34.15	11.66	4.703
SPCC1259.03	rpa12	0.2583	-6.901	0.587875594	35.27	69.27	8.049	1.598
SPBC1718.07C	zfs1	0.5617	-6.877	0.250495576	15.85	37.33	15.83	5.343
SPBC1D7.01	SPBC1D7.01	0.2009	-6.863	0.697020063	35.71	69.93	6.888	1.176
SPAC3H8.09C	nab3	0.2938	-6.849	0.531948209	22.98	48.99	8.557	2.317
SPAC227.01C	SPAC227.01c	0.4618	-6.846	0.335546071	29.98	60.5	13.25	1.542
SPAC823.02	SPAC823.02	0.429	-6.845	0.367542708	27.51	56.44	12.23	1.399
SPAC4F10.16C	SPAC4F10.16c	0.4452	-6.843	0.351444844	24.83	52.03	12.52	2.391
SPAC4D7.03	pop2	0.02734	-6.833	1.56320149	30.69	61.63	0.6974	1.77
SPBP8B7.07C	set6	0.1404	-6.808	0.852632892	35.96	70.26	5.625	1.45
SPBC30D10.13C	pdb1	0.01323	-6.762	1.878440156	3.521	16.89	2.228	1.395
SPBC13G1.12	did2	0.5051	-6.76	0.296622631	25.16	52.43	14.63	1.277
SPCC1620.03	mug163	0.2474	-6.746	0.606600305	35.18	68.86	7.691	1.247
SPAC1834.04	hht1	0.1363	-6.745	0.865504144	37.02	71.89	5.521	0.9606
SPBC1778.10C	ppk21	0.1528	-6.708	0.815876646	35.92	70.02	5.808	1.106
SPBC16A3.16	SPBC16A3.16	0.005841	-6.684	2.233512794	31.85	63.29	1.888	1.113

SPBC21C3.01C	vps13a	0.6058	-6.675	0.217670731	25.81	53.36	18.04	4.716
SPAC1687.08	SPAC1687.08	0.3816	-6.625	0.418391634	12.66	31.67	9.662	3.618
SPBC23G7.04C	nif1	0.5616	-6.6	0.250572901	33.21	65.38	16.55	1.536
SPCC74.06	mak3	0.1775	-6.51	0.750801643	37.4	72.13	6.089	1.213
SPBC25B2.10	SPBC25B2.10	0.4094	-6.471	0.387852162	18.16	40.46	9.492	4.393
SPCC1322.01	rpm1	0.158	-6.447	0.801342913	20.2	43.76	5.653	1.362
SPAC222.13C	SPAC222.13c	0.3791	-6.421	0.421246216	27.12	55.09	9.47	3.26
SPAC31G5.07	dni1	0.4743	-6.402	0.323946875	28.82	57.85	12.54	2.517
SPAC3F10.05C	mug113	0.5578	-6.398	0.25352149	30.24	60.18	15.78	2.088
SPAC17A5.01	pex6	0.1218	-6.398	0.914352712	32.13	63.29	4.962	0.8379
SPBC16E9.13	ksp1	0.1791	-6.362	0.746904414	34.52	67.14	5.832	1.853
SPAC458.06	SPAC458.06	0.3931	-6.351	0.405496956	22.77	47.84	10.08	2.533
SPBC11B10.05C	rsp1	0.2252	-6.331	0.647431614	36.77	70.8	6.823	0.7273
SPBC16H5.04	SPBC16H5.04	0.2172	-6.327	0.663140179	35.65	68.96	6.612	1.408
SPBC2G2.06C	apl1	0.04899	-6.307	1.309892561	33.07	64.69	1.776	2.092
SPBC19C7.01	mni1	0.0143	-6.275	1.844663963	28.11	56.47	1.772	1.427
SPBC1105.01	rrp12	0.3977	-6.252	0.400444409	37.74	72.26	10.32	1.481
SPBC21D10.11C	nfs1	0.2778	-6.229	0.556267759	38.77	73.91	7.659	1.349
SPCC162.02C	SPCC162.02c	0.5758	-6.221	0.239728339	32.83	64.14	16.22	1.522
SPAC630.05	gyp7	0.124	-6.215	0.906578315	34.41	66.72	4.854	1.232
SPCC70.06	SPCC70.06	0.1037	-6.212	0.984221244	32.53	63.63	4.431	1.482
SPAC1F3.09	mug161	0.1528	-6.21	0.815876646	36.18	69.62	5.368	1.168
SPCC320.07C	mde7	0.2524	-6.205	0.597910649	36.74	70.54	7.07	1.694
SPCC1919.12C	SPCC1919.12c	0.2523	-6.157	0.598082749	36	69.25	6.831	2.184
SPBC12D12.07C	trx2	0.3206	-6.126	0.494036482	4.89	18.1	7.967	2.605
SPBC1539.03C	SPBC1539.03c	0.09178	-6.125	1.037251947	22.15	46.44	4.204	0.8849
SPBC17G9.05	rct1	0.4982	-6.075	0.302596277	29.94	59.16	12.11	3.678
SPAC11D3.14C	SPAC11D3.14c	0.02885	-6.041	1.539854183	31.98	62.45	2.753	1.15
SPAC13G7.06	met16	0.1702	-6.026	0.769040444	17.36	38.42	5.191	2.085
SPBC3B8.08	SPBC3B8.08	0.1643	-5.983	0.784362437	36.85	70.37	5.374	1.072
SPCC1223.09	SPCC1223.09	0.2208	-5.978	0.656000931	35.08	67.43	6.36	0.83
SPAC17G8.06C	SPAC17G8.06c	0.02307	-5.954	1.636952405	18.03	39.4	2.523	1.138
SPBC83.05	SPBC83.05	0.2184	-5.939	0.660747366	34.73	66.81	6.112	1.776
SPBC365.13C	hba1	0.5314	-5.935	0.27457845	31.62	61.69	13.65	1.798
SPAC1556.02C	sdh1	0.463	-5.935	0.334419009	28.04	55.81	11.21	2.555
SPCC548.05C	SPCC548.05c	0.09498	-5.909	1.022367835	35.15	67.45	3.812	1.828
SPAC17H9.08	SPAC17H9.08	0.4567	-5.899	0.340368988	24.12	49.31	10.13	3.956
SPBP8B7.18C	SPBP8B7.18c	0.3114	-5.873	0.506681392	24.28	49.53	5.897	3.914
SPBC1347.11	sro1	0.1805	-5.839	0.743522794	37.1	70.53	5.474	1.353
SPBC1861.01C	cnp3	0.551	-5.823	0.258848401	30.91	60.34	14.18	1.452
SPCC794.03	SPCC794.03	0.01407	-5.756	1.851705903	30.01	58.75	2.133	1.004
SPAC4F10.06	SPAC4F10.06	0.1709	-5.75	0.767257937	34.38	65.92	4.93	2.041
SPAC13A11.05	SPAC13A11.05	0.1212	-5.7	0.91649738	35.37	67.47	4.295	1.555
SPAC1527.01	mok11	0.08962	-5.673	1.04759506	31.26	60.66	3.813	1.307
SPBC2G2.15C	mrm2	0.2755	-5.67	0.559878397	37.93	71.62	6.964	0.9647
SPCC663.03	pmd1	0.2244	-5.651	0.648977147	35.55	67.68	6.068	0.8494
SPAC22F3.11C	snu23	0.5712	-5.617	0.243211801	25.97	51.88	14.51	0.9218
SPAC5H10.13C	gmh2	0.6059	-5.604	0.217599048	33.44	64.13	15.94	1.336
SPBPB2B2.12C	gal10	0.6901	-5.604	0.161087973	22.32	45.86	19.88	4.884
SPAC105.02C	SPAC105.02c	0.1311	-5.601	0.882397308	36.02	68.37	4.5	0.9871
SPBC56F2.06	mug147	0.1547	-5.597	0.810509686	32.47	62.52	4.454	2.056
SPAC23C4.06C	SPAC23C4.06c	0.6036	-5.596	0.219250769	36.88	69.78	15.55	2.592
SPBC26H8.01	thi2	0.2076	-5.579	0.682772651	22.89	46.77	3.965	3.092
SPBC19C7.12C	omh1	0.0448	-5.555	1.348721986	34.59	65.94	2.545	1.558
SPAC57A10.09C	nhp6	0.3913	-5.547	0.407490152	31.5	60.85	8.978	1.496
SPAP27G11.10C	nup184	0.08222	-5.541	1.085022528	32.05	61.75	2.66	2.07
SPCC24B10.07	gad8	0.1935	-5.514	0.713319031	0.02883	9.104	5.423	0.02883
SPAC1002.20	SPAC1002.20	0.2646	-5.504	0.57741016	36.23	68.56	6.427	1.677
SPAC1751.01C	gti1	0.515	-5.498	0.288192771	27.8	54.7	12.18	1.291
SPAC2G11.03C	vps45	0.2592	-5.488	0.586365003	25.84	51.45	2.05	3.909
SPAC4A8.10	SPAC4A8.10	0.1184	-5.461	0.926648298	27.01	53.34	3.247	2.244
SPAC23G3.03	sib2	0.2523	-5.459	0.598082749	36.37	68.71	6.31	0.9635
SPAC26A3.02	myh1	0.6508	-5.456	0.186552456	24.93	49.91	16.76	4.648
SPAC8E11.10	SPAC8E11.10	0.1792	-5.445	0.746661995	27.99	54.92	5.054	1.391
SPBC947.06C	SPBC947.06c	0.2403	-5.435	0.619246229	35.47	67.19	5.954	1.614
SPBC215.01	SPBC215.01	0.03432	-5.43	1.464452721	21.18	43.71	2.623	1.014
SPBP16F5.03C	tra1	0.02053	-5.409	1.687611051	29.4	57.18	1.425	1.376
SPAC607.07C	SPAC607.07c	0.5949	-5.392	0.225556031	32	61.41	14.59	2.512
SPAC3F10.04	gsa1	0.5706	-5.376	0.243668233	15.55	34.37	11.74	5.343

SPAC227.10	SPAC227.10	0.09158	-5.373	1.038199361	35.18	66.61	3.611	1.333
SPBC1347.03	meu14	0.5636	-5.339	0.249029016	31.17	59.96	13.34	1.915
SPBC23G7.06C	SPBC23G7.06c	0.09184	-5.335	1.036968125	34.87	66.03	3.624	1.225
SPBC725.07	pex5	0.1898	-5.312	0.721703792	23.49	47.31	4.522	2.286
SPBC19F5.01C	puc1	0.3075	-5.291	0.51215488	33.13	63.12	6.609	2.297
SPBC25B2.04C	mtg1	0.1411	-5.282	0.850472986	22.76	46.07	3.011	2.471
SPAC6G10.08	idp1	0.6177	-5.258	0.209222399	26.19	51.66	10.11	7.837
SPBC24C6.05	sec28	0.215	-5.243	0.66756154	23.63	47.43	4.916	2.258
SPCC737.05	SPCC737.05	0.6188	-5.225	0.208449695	31.08	59.64	15.24	2.316
SPBC1703.06	pof10	0.2742	-5.179	0.56193255	34.94	65.9	6.107	1.835
SPAC17A5.10	SPAC17A5.10	0.1289	-5.144	0.889747083	34.92	65.8	3.891	1.601
SPAC1071.02	mms19	0.07744	-5.136	1.111034656	19.31	40.16	3.201	1.297
SPAC922.06	SPAC922.06	0.2607	-5.109	0.583858969	36.29	68	5.777	1.869
SPCC663.15C	SPCC663.15c	0.3267	-5.093	0.485850866	36.1	67.67	7.009	1.456
SPAC14C4.16	dad3	0.01209	-5.059	1.917573699	32.95	62.44	1.736	0.9469
SPAC3C7.02C	SPAC3C7.02c	0.5982	-5.059	0.223153591	34.42	64.84	14.07	1.181
SPCC970.02	SPCC970.02	0.639	-5.05	0.194499142	30.17	57.86	14.99	4.078
SPBC646.13	sds23	0.0836	-5.047	1.077793723	18.75	39.09	1.864	2.011
SPBC1778.04	spo6	0.6341	-5.045	0.197842247	34.13	64.34	15.18	3.055
SPBC16G5.03	SPBC16G5.03	0.295	-5.026	0.530177984	36.23	67.77	6.394	1.392
SPBC577.13	syj2	0.5459	-5.025	0.262886906	26.63	51.99	11.63	2.793
SPBC19C2.06C	mug124	0.03413	-5.018	1.466863712	32.09	60.96	1.871	1.412
SPBC1271.07C	SPBC1271.07c	0.5066	-4.975	0.295334815	31.38	59.72	10.34	2.651
SPBC17D11.03C	SPBC17D11.03c	0.58	-4.973	0.236572006	32.6	61.72	12.98	1.948
SPAC13G7.11	SPAC13G7.11	0.2213	-4.972	0.655018586	30.21	57.79	5.291	0.7795
SPBC16G5.09	SPBC16G5.09	0.6477	-4.944	0.188626103	31.37	59.65	15.88	1.829
SPAC22A12.06C	SPAC22A12.06c	0.7006	-4.93	0.154529867	22.2	44.56	17.98	4.799
SPAPB17E12.12C	SPAPB17E12.12	0.6333	-4.929	0.198390512	32.65	61.73	14.81	2.927
SPCC737.07C	SPCC737.07c	0.1662	-4.921	0.779368981	36.28	67.67	4.315	1.456
SPBC29A3.09C	SPBC29A3.09c	0.218	-4.92	0.661543506	38.19	70.82	5.059	1.466
SPBC30B4.02C	SPBC30B4.02c	0.6392	-4.92	0.194363234	34.75	65.16	15.44	1.466
SPBC27B12.05	SPBC27B12.05	0.5782	-4.913	0.237921913	32.93	62.16	12.66	2.258
SPBC577.06C	stt4	0.4657	-4.904	0.331893762	26.89	52.23	9.519	1.454
SPAC30D11.06C	SPAC30D11.06c	0.2608	-4.893	0.583692413	36.62	68.18	5.666	1.461
SPAC24C9.12C	SPAC24C9.12c	0.5023	-4.881	0.299036822	26.18	51.02	9.975	2.71
SPAC4C5.03	SPAC4C5.03	0.2382	-4.88	0.623058243	27.19	52.67	5.354	1.314
SPAC17H9.11	gmf1	0.6366	-4.876	0.196133366	31.09	59.07	15.04	2.166
SPCC1450.09C	SPCC1450.09c	0.2333	-4.855	0.632085261	37	68.76	5.258	1.306
SPBC26H8.09C	snf59	0.2882	-4.83	0.540306024	37.74	69.93	6.03	1.376
SPBP8B7.30C	thi5	0.393	-4.814	0.40560745	22.55	44.94	7.498	2.234
SPBC9B6.09C	mdl1	0.1722	-4.807	0.763966853	35.43	66.09	4.307	1.406
SPAC323.01C	pos5	0.2346	-4.798	0.629671992	8.671	22.12	2.021	3.183
SPAC4F8.08	mug114	0.6303	-4.795	0.200452693	34.9	65.21	14.66	1.23
SPAC1039.03	SPAC1039.03	0.323	-4.79	0.490797478	32.74	61.64	6.521	1.41
SPBC17A3.03C	SPBC17A3.03c	0.2212	-4.767	0.655214877	35.5	66.15	4.778	1.776
SPAC26A3.16	dph1	0.3337	-4.755	0.476643793	34.92	65.17	6.312	2.188
SPCC736.13	SPCC736.13	0.3541	-4.75	0.450874073	35.62	66.31	6.777	1.988
SPBC3E7.16C	leu3	0.06643	-4.75	1.177635748	36.72	68.12	1.709	1.733
SPCC1884.02	nic1	0.1217	-4.741	0.914709422	26	50.49	3.026	1.879
SPAC3H5.04	aar2	0.387	-4.728	0.412289035	31.86	60.1	7.534	1.441
SPCC1393.02C	spt2	0.03098	-4.685	1.508918587	33.15	62.15	2.174	0.9217
SPBC16H5.03C	fub2	0.07642	-4.679	1.116792967	35.03	65.23	2.988	0.8675
SPAC664.14	amt2	0.4075	-4.679	0.389872387	24.26	47.54	7.778	1.663
SPBC1347.08C	SPBC1347.08c	0.1691	-4.667	0.771856392	36.04	66.86	4.205	1.162
SPBC119.03	SPBC119.03	0.2543	-4.66	0.59465364	38.79	71.37	5.392	0.9985
SPAC14C4.13	rad17	0.08205	-4.642	1.085921415	34.36	64.06	2.844	1.359
SPBC16A3.13	meu7	0.2058	-4.638	0.68655463	33.28	62.29	3.773	2.326
SPAC2C4.08	SPAC2C4.08	0.1312	-4.636	0.882066165	34.01	63.48	2.913	1.968
SPBC1A4.04	SPBC1A4.04	0.6801	-4.622	0.167427225	19.12	39	13.69	6.624
SPBC2G2.07C	mug178	0.0507	-4.613	1.294992041	17.72	36.69	2.398	1.172
SPAC1250.03	ubc14	0.01993	-4.612	1.700492701	34.05	63.5	1.596	1.089
SPCC24B10.20	SPCC24B10.20	0.3195	-4.608	0.495529138	36.98	68.32	6.322	0.7802
SPBC16G5.02C	SPBC16G5.02c	0.231	-4.605	0.63638802	36.31	67.21	4.534	2.019
SPAC23A1.14C	SPAC23A1.14c	0.5365	-4.601	0.270430274	27.17	52.19	10.8	0.9168
SPAC25B8.05	SPAC25B8.05	0.02859	-4.599	1.543785845	20.14	40.64	2.026	0.373
SPCC613.07	SPCC613.07	0.2001	-4.59	0.698752911	33.14	61.98	4.101	1.945
SPAC26F1.04C	etr1	0.2347	-4.582	0.62948691	35.3	65.51	5.067	0.5559
SPAC323.03C	SPAC323.03c	0.7163	-4.581	0.144905049	37.73	69.5	17.85	4.425
SPCC14G10.03C	ump1	0.5969	-4.567	0.224098421	12.97	28.81	10.62	4.963

SPAC2F7.10	akr1	0.5687	-4.557	0.245116772	29.17	55.4	11.41	2.152
SPBC365.20C	pnc1	0.5623	-4.54	0.250031916	24.65	47.94	11.29	1.688
SPBC1604.16C	SPBC1604.16c	0.6291	-4.53	0.201280315	30.15	56.96	13.63	2.05
SPBC8D2.10C	rmt3	0.683	-4.526	0.165579296	29.6	56.05	15.89	3.353
SPCC24B10.12	cgi121	0.6108	-4.523	0.214100972	27.78	53.06	12.91	1.863
SPBCPT2R1.02	SPBCPT2R1.02	0.572	-4.51	0.242603971	30.97	58.28	11.34	2.304
SPCC1494.10	adn3	0.1506	-4.507	0.822175028	34.9	64.72	3.874	0.7609
SPBC30D10.09C	SPBC30D10.09	0.665	-4.472	0.177178355	34.66	64.28	14.74	3.247
SPBC19C7.05	SPBC19C7.05	0.04861	-4.462	1.313274379	34.09	63.33	2.404	0.9042
SPAC6C3.07	mug68	0.6778	-4.457	0.168898435	35.97	66.41	15.69	2.3
SPAC343.07	mug28	0.5339	-4.435	0.272540079	29.78	56.2	9.99	2.281
SPBC23G7.12C	rpt6	0.3041	-4.415	0.51698358	37.09	68.18	5.633	1.581
SPCC794.09C	ef1a-a	0.6226	-4.411	0.205790884	27.75	52.82	11.8	4.38
SPBC1734.12C	alg12	0.5313	-4.387	0.274660184	33.53	62.29	10.04	1.542
SPCC63.03	SPCC63.03	0.6601	-4.378	0.180390267	32.46	60.5	14.66	1.543
SPCP31B10.07	eft202	0.5571	-4.353	0.254066842	30.28	56.89	10.74	1.316
SPCC18.15	SPCC18.15	0.3176	-4.331	0.498119506	37.07	68	5.797	1.343
SPAC29A4.12C	mug108	0.5888	-4.298	0.230032199	30	56.34	11.5	1.729
SPAC3A11.04	SPAC3A11.04	0.7161	-4.258	0.145026326	20.55	40.74	14.25	6.989
SPAC5D6.10C	mug116	0.1644	-4.253	0.784098187	32.95	61.1	2.594	2.133
SPCC1620.07C	SPCC1620.07c	0.3875	-4.246	0.411728293	34.85	64.22	6.757	1.364
SPAC29B12.13	SPAC29B12.13	0.646	-4.243	0.189767482	33.9	62.66	13.43	2.088
SPBC14C8.11C	SPBC14C8.11c	0.7087	-4.234	0.149537567	34.9	64.28	16.63	2.379
SPAC5D6.12	SPAC5D6.12	0.6345	-4.229	0.197568374	33.02	61.18	13.05	1.406
SPAC6F6.01	cch1	0.2797	-4.213	0.553307534	24.3	46.84	4.197	2.458
SPAC32A11.03C	phx1	0.1273	-4.205	0.895171596	31.69	58.96	2.082	1.939
SPBC17D1.02	dph2	0.6662	-4.204	0.176395372	33.37	61.72	14.3	1.797
SPCC1442.11C	SPCC1442.11c	0.3149	-4.194	0.501827339	37.78	68.95	5.45	1.628
SPBC530.04	mod5	0.6762	-4.189	0.169924834	27.64	52.28	13.75	4.24
SPAC7D4.03C	SPAC7D4.03c	0.6747	-4.187	0.17088929	25.9	49.41	13.22	4.842
SPCC1827.08C	pof7	0.5767	-4.178	0.239050049	31.95	59.35	10.48	2.556
SPCC1020.07	SPCC1020.07	0.1444	-4.162	0.840432807	26.64	50.6	2.981	1.688
SPBC13G1.08C	ash2	0.5418	-4.15	0.266160999	28.29	53.29	9.806	1.305
SPAC227.11C	SPAC227.11c	0.7095	-4.143	0.1490476	29.67	55.53	16.18	2.808
SPAC9.05	fml1	0.61	-4.141	0.214670165	27.67	52.25	11.68	2.084
SPAC144.14	klp8	0.617	-4.13	0.209714836	30.4	56.72	12.12	1.157
SPBC336.10C	tif512	0.06347	-4.107	1.197431502	33.17	61.23	1.531	1.465
SPAC27D7.02C	SPAC27D7.02c	0.04826	-4.102	1.316412682	35.49	65.03	1.583	1.306
SPAC12G12.12	SPAC12G12.12	0.1353	-4.092	0.868702203	29.78	55.63	3.043	1.448
SPCC16C4.04	SPCC16C4.04	0.4287	-4.084	0.367846516	28.87	54.13	7.128	1.556
SPAC18B11.09C	SPAC18B11.09c	0.03967	-4.07	1.4015378	32.89	60.71	1.651	1.173
SPAC637.10C	rpn10	0.02251	-4.066	1.647624505	32.11	59.43	1.654	0.8567
SPAC1250.02	mug95	0.2932	-4.049	0.532836034	25.31	48.23	4.627	2.05
SPBC6B1.02	ppk30	0.1624	-4.034	0.789413975	37.49	68.21	3.378	1.396
SPAC821.03C	SPAC821.03c	0.6066	-4.029	0.217097594	31.89	59	11.4	1.455
SPCC191.09C	gst1	0.3136	-4.008	0.503623946	36.01	65.73	5.325	1.19
SPBC543.10	get1	0.6116	-3.994	0.213532523	31.1	57.64	11.17	2.433
SPBC6B1.09C	nbs1	0.6969	-3.963	0.156829535	35.57	64.94	14.86	2.341
SPBP23A10.16	sdh4	0.5456	-3.944	0.263125638	43.23	77.49	8.728	2.898
SPAC9.07C	SPAC9.07c	0.6416	-3.939	0.192735645	35.6	64.94	12.03	2.699
SPCP1E11.11	puf6	0.2508	-3.928	0.600672468	28.72	53.63	3.885	1.974
SPAC1610.02C	SPAC1610.02c	0.3139	-3.92	0.503208684	20.95	40.86	2.822	3.028
SPAC6F12.12	par2	0.1626	-3.918	0.788879459	32.13	59.21	2.827	1.76
SPBC19G7.02	SPBC19G7.02	0.3198	-3.915	0.495121541	23.03	44.26	5.231	1.334
SPBC405.03C	SPBC405.03c	0.3472	-3.906	0.459420283	34.26	62.68	5.362	1.833
SPBC1683.04	SPBC1683.04	0.3325	-3.896	0.47820835	30.27	56.12	4.106	2.72
SPAC631.01C	acp2	0.3826	-3.893	0.417255034	28.23	52.76	5.395	2.468
SPAC13A11.04C	ubp8	0.6559	-3.878	0.183162369	24.37	46.4	12.12	3.33
SPAC22F8.03C	SPAC22F8.03c	0.2015	-3.878	0.69572495	36.44	66.23	3.873	0.8828
SPBC56F2.04	utp20	0.1149	-3.861	0.939679971	32.95	60.47	1.727	1.727
SPBC146.13C	myo1	0.628	-3.82	0.202040356	13.01	27.65	9.867	4.407
SPBC800.07C	tsf1	0.2216	-3.783	0.654430244	14.43	29.92	3.832	1.348
SPAC22H12.01C	mug35	0.7093	-3.78	0.14917004	32.39	59.42	15.03	1.436
SPCC1235.11	SPCC1235.11	0.6353	-3.773	0.197021145	13.79	28.86	9.699	4.68
SPAC1F7.06	SPAC1F7.06	0.3188	-3.757	0.496481687	36.82	66.66	4.928	1.468
SPBC685.04C	aps2	0.6884	-3.745	0.162159138	35.45	64.38	13.8	1.429
SPBC1683.11C	SPBC1683.11c	0.628	-3.727	0.202040356	32.1	58.86	11.06	2.056
SPBC56F2.11	met6	0.4251	-3.721	0.371508895	8.51	20.09	6.426	1.44
SPAP8A3.12C	tpp2	0.1469	-3.715	0.832978204	37.45	67.61	2.904	1.316

SPBC2D10.07C	SPBC2D10.07c	0.6378	-3.713	0.195315485	29.11	53.92	11.09	2.77
SPBC4F6.05C	SPBC4F6.05c	0.7271	-3.704	0.138405855	37.63	67.9	15.04	3.734
SPBP4H10.16C	SPBP4H10.16c	0.5586	-3.684	0.252899069	27.15	50.65	8.093	3.24
SPCC1223.04C	set11	0.2288	-3.681	0.64054398	32.23	58.98	2.928	2.08
SPAC25B8.19C	SPAC25B8.19c	0.5311	-3.675	0.274823699	6.022	15.93	6.73	3.711
SPCC777.10C	ubc12	0.2449	-3.654	0.611011215	28.16	52.27	3.696	1.685
SPBC660.07	ntp1	0.7068	-3.654	0.150703459	28.33	52.54	12.91	4.704
SPAC1B2.04	cox6	0.2827	-3.651	0.548674192	3.42	11.62	4.45	1.179
SPBC21C3.14C	SPBC21C3.14c	0.2706	-3.599	0.567672208	36.2	65.38	4.21	1.258
SPBC4C3.09	SPBC4C3.09	0.6685	-3.591	0.174898588	32.71	59.64	11.96	2.67
SPBC405.06	SPBC405.06	0.6777	-3.585	0.168962514	26.59	49.57	12.01	3.337
SPCC1183.10	wtf10	0.1903	-3.578	0.720561212	36.94	66.55	3.423	0.9612
SPBC1683.03C	SPBC1683.03c	0.7351	-3.557	0.133653577	31.54	57.65	15.42	2.325
SPCC1281.07C	SPCC1281.07c	0.715	-3.552	0.145693958	33.54	60.93	14.46	1.126
SPAC11E3.04C	ubc13	0.151	-3.536	0.821023053	34.55	62.56	1.417	1.831
SPAC8E11.01C	SPAC8E11.01c	0.7024	-3.531	0.153415497	36.4	65.6	13.63	1.622
SPAC23C4.12	hhp2	0.3026	-3.498	0.519131076	30.32	55.55	3.66	2.158
SPCC126.08C	SPCC126.08c	0.393	-3.494	0.40560745	38.11	68.33	5.013	2.214
SPBC13E7.07	SPBC13E7.07	0.2838	-3.49	0.546987609	37.11	66.69	4.101	1.472
SPAC513.06C	SPAC513.06c	0.6383	-3.474	0.194975156	30.7	56.14	10.67	1.898
SPBPB10D8.01	SPBPB10D8.01	0.6162	-3.467	0.210278306	26.94	49.95	9.371	3.013
SPAC4G9.19	SPAC4G9.19	0.536	-3.466	0.27083521	25.82	48.11	7.742	2.036
SPBC11B10.06	sws1	0.1393	-3.464	0.856048884	35.31	63.68	2.708	1.115
SPAC24B11.07C	SPAC24B11.07c	0.4482	-3.452	0.348528148	31.63	57.62	5.579	2.523
SPAC27D7.13C	ssm4	0.2585	-3.425	0.587539453	32.25	58.6	2.17	2.321
SPAPB1E7.12	rps602	0.2024	-3.422	0.693789492	35.22	63.48	2.956	1.566
SPCC24B10.02C	SPCC24B10.02c	0.3411	-3.421	0.467118281	38.34	68.6	4.662	1.53
SPAP32A8.02	SPAP32A8.02	0.2296	-3.405	0.639028116	36.21	65.08	3.314	1.516
SPAC869.06C	SPAC869.06c	0.7142	-3.401	0.146180154	23.92	44.88	12.01	4.889
SPAPB1A10.12C	alo1	0.6927	-3.384	0.159454812	28.64	52.61	10.92	4.647
SPBC947.04	SPBC947.04	0.2973	-3.375	0.526805091	29.27	53.62	4.313	0.9619
SPAPB18E9.04C	SPAPB18E9.04c	0.7146	-3.372	0.145936988	32.67	59.21	13.33	2.6
SPAC22H10.09	SPAC22H10.09	0.174	-3.37	0.759450752	20.78	39.67	2.379	1.631
SPCC645.11C	mug117	0.7354	-3.369	0.133476374	32.99	59.73	14.66	2.083
SPBC15D4.01C	klp9	0.2649	-3.364	0.576918042	35.23	63.39	3.838	1.252
SPAC1F8.01	ght3	0.3142	-3.36	0.502793819	31.56	57.36	1.926	2.688
SPAC11H11.04	mam2	0.583	-3.351	0.234331445	26.55	49.12	8.845	1.211
SPAC607.06C	SPAC607.06c	0.6827	-3.351	0.165770097	30.81	56.11	11.91	2.028
SPAC17A5.05C	SPAC17A5.05c	0.3757	-3.337	0.425158805	38.38	68.53	4.425	2.199
SPAC26H5.10C	tif51	0.2007	-3.33	0.697452628	35.38	63.59	2.641	1.668
SPAC664.07C	rad9	0.1178	-3.33	0.92885471	32.59	59	1.702	1.462
SPCC1259.11C	gyp2	0.3433	-3.31	0.464326197	27.95	51.34	4.459	1.608
SPAC10F6.16	mug134	0.7306	-3.31	0.136320332	31.42	57.05	13.81	2.958
SPBC16E9.07	mug100	0.1963	-3.295	0.7070797	23.15	43.45	2.2	1.801
SPAC8C9.08	rps5	0.7251	-3.29	0.139602095	30.91	56.18	13.37	3.031
SPBC25H2.05	egd2	0.1784	-3.284	0.74860515	34.61	62.24	2.572	1.48
SPAC6B12.07C	SPAC6B12.07c	0.462	-3.282	0.335358024	28.24	51.78	6.137	1.522
SPCC24B10.06	SPCC24B10.06	0.5833	-3.278	0.234108024	39.89	70.91	8.216	2.358
SPBC2D10.12	rhp23	0.2031	-3.254	0.692290077	17.82	34.62	3.243	0.8429
SPAC1782.08C	rex3	0.5686	-3.247	0.245193145	23.58	44.06	7.748	2.364
SPCC338.06C	SPCC338.06c	0.7132	-3.244	0.146788665	30.65	55.68	12.73	2.566
SPCC794.15	SPCC794.15	0.163	-3.244	0.787812396	33.95	61.1	2.254	1.506
SPAC110.01	ppk1	0.3465	-3.23	0.460296761	37.27	66.53	4.373	1.596
SPBC1685.13	fhn1	0.4019	-3.221	0.395881994	32.37	58.46	5.271	1.172
SPAC30.02C	SPAC30.02c	0.7443	-3.218	0.128251981	22.15	41.67	9.798	7.272
SPBC1711.09C	SPBC1711.09c	0.7208	-3.197	0.142185222	29.57	53.82	12.24	3.882
SPCC1223.12C	meu10	0.2152	-3.174	0.667157733	35.52	63.56	2.204	1.826
SPCC16C4.14C	sfc4	0.5228	-3.165	0.281664421	28.61	52.2	6.052	2.831
SPBC4B4.10C	atg5	0.651	-3.144	0.186419011	34.73	62.21	10.2	1.173
SPAC4D7.02C	SPAC4D7.02c	0.7591	-3.139	0.119701009	32.2	58.04	14.32	3.993
SPAC458.04C	dil1	0.2815	-3.119	0.550521601	38.58	68.49	3.784	1.022
SPAC3A11.10C	SPAC3A11.10c	0.7097	-3.112	0.148925195	36.16	64.51	12.38	1.206
SPBC8D2.18C	SPBC8D2.18c	0.6895	-3.111	0.161465729	30.19	54.7	10.92	2.896
SPBC1685.05	SPBC1685.05	0.1043	-3.108	0.981715692	33.35	59.89	1.457	1.314
SPBC365.04C	SPBC365.04c	0.5505	-3.108	0.259242677	25.91	47.66	7.329	1.631
SPBC16D10.03	pgp2	0.7018	-3.106	0.153786636	33.82	60.65	11.36	3.077
SPAC3H8.02	SPAC3H8.02	0.5626	-3.106	0.249800272	20.64	39	6.717	2.945
SPBC29B5.01	atf1	0.3267	-3.105	0.485850866	19.43	37.02	4.207	1.1
SPAC13F5.01C	msh1	0.6577	-3.102	0.181972158	24.79	45.82	9.54	3.024

SPCPB1C11.02	SPCPB1C11.02	0.6809	-3.096	0.166916666	31.72	57.19	10.95	1.811
SPAC343.18	rfp2	0.3416	-3.082	0.466482138	36.51	65.03	4.387	0.9406
SPAC12B10.16C	mug157	0.6819	-3.08	0.16627931	30.09	54.48	11.12	0.9979
SPCC16C4.07	scw1	0.3062	-3.059	0.513994814	32.16	57.85	2.776	2.134
SPAPB1A10.08	SPAPB1A10.08	0.4393	-3.04	0.357238797	28.14	51.22	5.407	1.283
SPAC9.12C	atp12	0.3785	-3.033	0.421934116	21.92	40.99	4.615	1.265
SPBC776.16	SPBC776.16	0.1504	-3.033	0.822752164	34.02	60.87	1.493	1.53
SPAC31G5.14	gcv1	0.7832	-3.026	0.106127321	34.5	61.65	16.03	2.973
SPAC922.05C	SPAC922.05c	0.7864	-3.025	0.104356495	31.58	56.84	16.39	2.655
SPCC569.03	SPCC569.03	0.7945	-3.025	0.099906098	23.81	44.08	12.05	8.327
SPBC1539.02	SPBC1539.02	0.2524	-3.021	0.597910649	34.46	61.57	2.074	1.973
SPAPB1A11.01	mfc1	0.3893	-3.018	0.409715596	26.89	49.13	3.969	2.173
SPAC18G6.05C	SPAC18G6.05c	0.2667	-3.015	0.573976984	27.29	49.77	3.078	1.587
SPBC336.05C	SPBC336.05c	0.3964	-3.004	0.401866354	26.92	49.16	4.22	2.033
SPAC19A8.14	SPAC19A8.14	0.7037	-3.002	0.152612449	31.68	56.98	11.11	2.879
SPCC1259.09C	pdx1	0.4966	-3.001	0.303993285	37.39	66.35	6.173	1.351
SPCC1393.10	ctr4	0.7717	-2.997	0.1125515	17.52	33.7	12.71	6.089
SPCC297.06C	SPCC297.06c	0.7583	-2.985	0.120158944	35.66	63.49	14.35	1.791
SPBC215.13	SPBC215.13	0.7162	-2.984	0.144965683	32.46	58.21	11.36	3.358
SPAC222.08C	SPAC222.08c	0.4139	-2.979	0.383104574	17.11	32.99	3.752	2.508
SPCC338.18	SPCC338.18	0.4394	-2.977	0.357139947	28.68	52	5.385	0.9772
SPAC31G5.11	pac2	0.7509	-2.971	0.124417896	16.96	32.74	11.23	5.736
SPBC56F2.09C	arg5	0.6685	-2.967	0.174898588	13.75	27.45	9.088	3.469
SPBC1703.14C	top1	0.4643	-2.963	0.333201316	35.07	62.47	5.632	1.228
SPAC2F3.16	SPAC2F3.16	0.7009	-2.949	0.15434394	32.87	58.84	11	2.395
SPAPB17E12.03	SPAPB17E12.03	0.5757	-2.929	0.239803771	29.99	54.07	7.192	2.061
SPCC4B3.11C	SPCC4B3.11c	0.1115	-2.898	0.952725133	36.21	64.24	1.91	1.025
SPAC630.14C	tup12	0.6514	-2.892	0.186152246	14.29	28.23	6.093	4.8
SPBC1685.14C	SPBC1685.14c	0.7537	-2.868	0.122801485	33.28	59.37	13.28	2.496
SPBC2F12.11C	rep2	0.2593	-2.863	0.586197483	2.943	9.536	3.201	1.091
SPCC16C4.09	sts5	0.6519	-2.855	0.185819019	31.38	56.23	8.889	2.265
SPBC354.01	gtp1	0.7839	-2.848	0.105739336	36.84	65.2	15.32	2.192
SPBC16E9.09C	SPBC16E9.09c	0.8399	-2.847	0.075772419	25.14	45.98	17.31	8.393
SPAC12G12.11C	SPAC12G12.11c	0.7373	-2.833	0.132355766	29.37	52.89	12.2	2.471
SPAC22A12.01C	pso2	0.7293	-2.826	0.137093786	30.88	55.37	10.99	3.808
SPAC144.03	ade2	0.2632	-2.801	0.579714115	35.05	62.18	2.794	1.496
SPCC126.12	SPCC126.12	0.2811	-2.801	0.551139154	33.82	60.16	3.003	1.485
SPCC569.06	SPCC569.06	0.7049	-2.797	0.151872489	28.64	51.65	8.789	4.562
SPAC6G9.09C	rpl2401	0.7378	-2.782	0.132061349	32.5	57.96	11.82	2.848
SPCC11E10.07C	SPCC11E10.07c	0.4009	-2.781	0.396963944	14.84	28.94	4.385	1.364
SPAC2E1P3.04	cao1	0.6741	-2.774	0.171275673	34.16	60.67	9.464	1.964
SPAC5H10.11	gmh1	0.2046	-2.772	0.689094371	34.46	61.16	1.652	1.615
SPBC119.16C	SPBC119.16c	0.7104	-2.756	0.148497047	34.18	60.67	10.54	2.541
SPBC1711.03	aim27	0.7207	-2.747	0.142245478	32.23	57.46	11.24	1.773
SPAC22G7.07C	SPAC22G7.07c	0.8039	-2.739	0.094797971	19.71	36.88	13.48	6.609
SPAC1635.01	SPAC1635.01	0.605	-2.733	0.218244625	28.03	50.54	6.712	2.838
SPCC11E10.06C	elp4	0.7613	-2.724	0.11844417	21.76	40.22	5.418	7.706
SPCP25A2.02C	rhp26	0.7103	-2.723	0.148558185	31.6	56.37	10.22	2.871
SPAC13C5.04	SPAC13C5.04	0.4579	-2.711	0.339229356	21.38	39.57	2.99	2.845
SPBC21B10.04C	nrf1	0.2169	-2.689	0.663740448	35.1	62.08	1.63	1.623
SPAC27F1.06C	SPAC27F1.06c	0.5082	-2.678	0.293965339	30.85	55.07	4.103	2.869
SPBC800.11	SPBC800.11	0.7059	-2.67	0.151256818	33.37	59.19	9.789	2.884
SPAC13G6.01C	rad8	0.7563	-2.665	0.1213059	32.32	57.47	12.43	2.495
SPCC188.12	spn6	0.6342	-2.651	0.197773762	30.62	54.64	7.364	2.697
SPBC1289.15	SPBC1289.15	0.7479	-2.651	0.126156467	32.68	58.04	11.63	3.055
SPAC2E1P5.03	SPAC2E1P5.03	0.111	-2.651	0.954677021	18.76	35.17	0.9799	1.192
SPAPB1A10.07C	SPAPB1A10.07c	0.7068	-2.619	0.150703459	27.35	49.23	9.91	2.323
SPBC1703.13C	SPBC1703.13c	0.2922	-2.614	0.534319788	37.29	65.54	2.844	1.449
SPAC14C4.01C	SPAC14C4.01c	0.7536	-2.606	0.12285911	33.33	59.03	11.98	2.493
SPBC13A2.02	nup82	0.3668	-2.606	0.435570673	33.01	58.51	2.14	2.268
SPCC338.02	mug112	0.7524	-2.601	0.123551213	31.7	56.35	11.5	3.279
SPBC609.04	caf5	0.7607	-2.589	0.118786584	37.31	65.54	12.65	1.243
SPAC1805.12C	uep1	0.7392	-2.588	0.131238042	31.72	56.35	10.71	3.287
SPAC24H6.09	gef1	0.7628	-2.578	0.117589316	31.73	56.36	12.54	1.961
SPCC1322.12C	bub1	0.7396	-2.57	0.131003098	26.1	47.09	10.41	3.608
SPBC13E7.06	msd1	0.831	-2.568	0.080398976	35.41	62.38	17.85	2.37
SPBC1711.06	rpl401	0.7485	-2.568	0.125808195	33.61	59.42	11.95	0.8431
SPBC31A8.01C	rtn1	0.5573	-2.559	0.253910957	32.07	56.88	6.202	1.205
SPAC694.04C	SPAC694.04c	0.7706	-2.551	0.113170995	31.54	55.99	12.12	3.632

SPBC18A7.01	SPBC18A7.01	0.5398	-2.536	0.26776712	28.27	50.6	5.428	1.976
SPAC3F10.17	SPAC3F10.17	0.7478	-2.525	0.126214539	34.98	61.61	11.47	1.974
SPBC16E9.12C	pab2	0.679	-2.524	0.168130226	32.44	57.43	9.008	0.8805
SPBC29A3.07C	sab14	0.2696	-2.506	0.569280112	21.99	40.25	2.988	0.6848
SPAC24B11.05	SPAC24B11.05	0.7829	-2.506	0.106293707	27.61	49.46	13.04	2.987
SPCC569.02C	SPCC569.02c	0.8375	-2.492	0.077015184	22.14	40.46	14.43	7.615
SPAC1834.09	mug51	0.3981	-2.488	0.400007822	34.96	61.51	2.727	2.156
SPAC1F5.07C	hem14	0.748	-2.484	0.126098402	14.97	28.66	9.195	4.802
SPAC5H10.06C	adh4	0.4675	-2.459	0.330218385	29.68	52.8	4.468	1.511
SPBC2G2.08	ade9	0.121	-2.452	0.91721463	27.63	49.41	1.694	0.8746
SPAC19G12.08	scs7	0.6784	-2.449	0.168514161	30.12	53.5	8.262	2.221
SPCC126.13C	SPCC126.13c	0.6136	-2.443	0.212114649	30.45	54.02	6.84	1.582
SPAC9E9.05	SPAC9E9.05	0.7332	-2.439	0.134777544	33.67	59.31	10.33	2.091
SPCC622.18	rpl6	0.6144	-2.43	0.211548793	31.05	54.99	6.983	1.105
SPAC20G4.04C	hus1	0.6726	-2.413	0.172243137	22.27	40.55	6.464	3.746
SPCC364.06	nap1	0.7973	-2.412	0.098378236	32.76	57.78	13.9	1.868
SPAC17G6.02C	tco1	0.6552	-2.411	0.183626111	32.61	57.52	7.82	1.354
SPAC25G10.03	zip1	0.2742	-2.401	0.56193255	13.23	25.68	2.467	1.308
SPBP35G2.05C	cki2	0.7104	-2.391	0.148497047	33.33	58.68	9.343	1.716
SPAC6B12.16	meu26	0.7481	-2.384	0.126040345	30.21	53.53	10.72	2.185
SPBC18A7.02C	SPBC18A7.02c	0.7144	-2.381	0.146058554	22.4	40.71	7.257	4.357
SPCC24B10.18	SPCC24B10.18	0.6949	-2.38	0.158077688	28.02	49.94	7.656	3.378
SPAC23C11.07	SPAC23C11.07	0.7193	-2.375	0.14308994	31.51	55.65	9.177	2.658
SPCC1259.08	SPCC1259.08	0.714	-2.373	0.146301788	30.12	53.37	9.331	1.903
SPBC21C3.06	SPBC21C3.06	0.7692	-2.371	0.113960724	32.59	57.43	11.64	2.491
SPAC9E9.09C	SPAC9E9.09c	0.762	-2.368	0.118045029	13.48	26.03	9.735	4.506
SPAPB17E12.05	rpl3703	0.7591	-2.367	0.119701009	12.36	24.2	8.643	5.166
SPBC2D10.15C	pth1	0.7931	-2.366	0.10067205	37.16	64.92	13.28	2.043
SPBP35G2.10	mit1	0.7329	-2.348	0.134955278	31.47	55.55	9.878	2.144
SPBC16G5.16	SPBC16G5.16	0.8318	-2.343	0.079981084	39.03	67.96	16.15	2.904
SPBC216.04C	SPBC216.04c	0.784	-2.327	0.105683937	32.82	57.74	12.22	2.68
SPCC1020.06C	tal1	0.8118	-2.323	0.090550953	21.14	38.54	10.01	7.063
SPAC3H1.04C	mdm31	0.3212	-2.322	0.493224463	22.9	41.43	2.894	1.179
SPCC4B3.06C	SPCC4B3.06c	0.305	-2.318	0.515700161	36.86	64.35	2.882	0.9956
SPAC6B12.05C	ies2	0.6382	-2.306	0.1950432	31.93	56.23	6.3	2.583
SPCC24B10.14C	xlf1	0.7537	-2.296	0.122801485	33.27	58.42	10.39	2.57
SPAC694.03	SPAC694.03	0.7458	-2.293	0.127377621	32.63	57.37	10.52	0.9643
SPAC110.02	pds5	0.7591	-2.282	0.119701009	13.07	25.22	9.07	4.454
SPAC1B3.04C	SPAC1B3.04c	0.7632	-2.277	0.117361638	25.05	44.89	10.77	2.577
SPBC1734.05C	spf31	0.5328	-2.268	0.273435784	33.88	59.37	5.043	1.283
SPBC18E5.13	SPBC18E5.13	0.774	-2.266	0.111259039	33.07	58.04	11.23	2.744
SPBC16H5.12C	SPBC16H5.12c	0.1374	-2.26	0.862013267	31.33	55.17	1.511	0.9442
SPAC186.08C	SPAC186.08c	0.5781	-2.259	0.237997031	32.06	56.37	5.034	2.288
SPAC22E12.05C	rer1	0.7997	-2.248	0.097072904	34.62	60.56	12.56	3.212
SPAC9G1.07	SPAC9G1.07	0.6652	-2.244	0.177047759	31.08	54.74	7.213	2.01
SPCC825.01	SPCC825.01	0.7955	-2.236	0.099359816	34.22	59.88	12.45	2.654
SPAC222.05C	mss1	0.4256	-2.226	0.370998381	28.36	50.23	3.838	0.896
SPAC10F6.14C	SPAC10F6.14c	0.7254	-2.222	0.139422449	32.29	56.69	9.149	1.795
SPAC17A5.16	ftp105	0.6753	-2.217	0.17050325	32.08	56.33	7.364	2.059
SPCC663.13C	naa50	0.7678	-2.217	0.114751892	34.28	59.95	11.11	1.394
SPCC18.06C	caf1	0.7845	-2.202	0.105407052	29.66	52.34	11.52	2.688
SPBC365.16	SPBC365.16	0.6635	-2.197	0.178159073	24.66	44.11	4.882	3.759
SPAC11D3.17	SPAC11D3.17	0.7615	-2.189	0.118330092	31.92	56.03	9.98	2.994
SPAC20H4.03C	tfs1	0.1811	-2.188	0.74208155	33.16	58.07	1.947	0.7743
SPBC13G1.10C	mug81	0.5357	-2.178	0.271078354	22.65	40.78	4.328	1.99
SPAC3G6.13C	rpl4101	0.8031	-2.171	0.095230374	33.09	57.92	12.7	2.364
SPAPB8E5.04C	SPAPB8E5.04c	0.8651	-2.165	0.062933688	22.39	40.33	16.55	6.765
SPAC4A8.06C	SPAC4A8.06c	0.6728	-2.16	0.172114017	27.11	48.09	7.022	2.142
SPAC3G9.04	ssu72	0.4173	-2.151	0.379551615	26.33	46.78	3.445	1.219
SPCC364.03	rpl1702	0.4154	-2.149	0.381533508	21.01	38.04	2.339	1.98
SPBC1683.13C	cha4	0.695	-2.148	0.158015195	41.97	72.46	5.363	4.056
SPBPB7E8.02	SPBPB7E8.02	0.3416	-2.134	0.466482138	30.74	53.99	1.655	1.753
SPBC3E7.10	fma1	0.8398	-2.13	0.07582413	20.85	37.75	11.97	6.983
SPAC23G3.04	ies4	0.6726	-2.129	0.172243137	33.95	59.26	6.651	2.473
SPBC12C2.05C	bzz1	0.7862	-2.128	0.104466961	33.09	57.85	10.98	3.095
SPAC18B11.04	ncs1	0.2141	-2.125	0.669383333	30.83	54.14	1.547	1.193
SPAC144.11	rps1102	0.8002	-2.125	0.096801453	18.55	33.96	7.067	6.71
SPBC106.13	SPBC106.13	0.7837	-2.122	0.105850153	23.76	42.51	9.416	4.642
SPCC18B5.05C	SPCC18B5.05c	0.6243	-2.122	0.204606665	29.29	51.6	5.679	2.146

SPBC106.10	pka1	0.4048	-2.115	0.392759496	23.47	42.03	3.255	1.217
SPCC297.05	SPCC297.05	0.7101	-2.113	0.148680487	28.7	50.61	8.291	1.374
SPAC19G12.13C	poz1	0.7567	-2.107	0.121076266	31.83	55.74	9.563	2.568
SPAC15F9.02	seh1	0.4479	-2.093	0.348818938	28.7	50.58	2.976	1.835
SPAC22E12.04	ccs1	0.3601	-2.081	0.443576879	16.61	30.71	2.466	1.468
SPCC594.07C	bqt3	0.7726	-2.07	0.112045296	29.77	52.3	9.991	2.865
SPBC18E5.10	SPBC18E5.10	0.8107	-2.068	0.091139827	31.7	55.47	11.75	3.926
SPAPB17E12.04C	csn2	0.7372	-2.066	0.132414673	27.21	48.09	8.347	2.796
SPBC20F10.02C	SPBC20F10.02c	0.3496	-2.064	0.456428576	36.18	62.81	1.681	1.714
SPBC25H2.09	SPBC25H2.09	0.7911	-2.062	0.101768615	32.38	56.58	10.7	3.371
SPAPYUG7.03C	mid2	0.416	-2.044	0.380906669	35.19	61.16	2.49	1.777
SPAC17D4.01	pex7	0.2767	-2.041	0.557990841	36.32	63.01	1.491	1.411
SPBC24C6.04	SPBC24C6.04	0.5692	-2.038	0.244735109	18.08	33.04	4.523	1.931
SPBC713.09	SPBC713.09	0.7837	-2.037	0.105850153	31.97	55.86	10.53	2.666
SPCC737.09C	hmt1	0.6126	-2.034	0.212823008	30.39	53.25	5.387	1.826
SPAC1783.06C	atg12	0.7465	-2.023	0.126970188	32.34	56.44	8.842	2.274
SPAC26F1.07	SPAC26F1.07	0.8118	-2.013	0.090550953	23.64	42.14	9.057	5.917
SPAPB24D3.04C	mag1	0.7666	-2.012	0.115431185	31.49	55.03	9.575	2.486
SPAC20G8.09C	SPAC20G8.09c	0.7513	-2.011	0.124186611	31.32	54.75	9.037	2.167
SPBPB8B6.04C	grt1	0.3298	-2.007	0.481749349	33.5	58.33	2.534	1.06
SPAPB24D3.08C	SPAPB24D3.08	0.8231	-2.006	0.084547398	35.82	62.14	13.25	1.954
SPCC1827.04	SPCC1827.04	0.8125	-2.006	0.09017663	32.34	56.41	11.85	3.333
SPAC57A7.05	SPAC57A7.05	0.8303	-2.003	0.080764962	31.04	54.28	12.69	4.289
SPCC306.05C	ins1	0.6935	-1.995	0.158953535	31.67	55.3	6.751	2.416
SPBC405.05	SPBC405.05	0.8048	-1.993	0.094312032	33.29	57.95	11.71	2.324
SPBC365.07C	SPBC365.07c	0.7832	-1.987	0.106127321	34.22	59.47	10.51	1.968
SPAPB24D3.02C	SPAPB24D3.02	0.8271	-1.982	0.082441979	34.81	60.44	13.35	2.14
SPAC3H1.10	SPAC3H1.10	0.6704	-1.98	0.173665994	31.42	54.86	6.173	2.242
SPBC12C2.07C	SPBC12C2.07c	0.6675	-1.968	0.17554873	30.35	53.08	6.369	1.782
SPBC23E6.03C	nta1	0.6228	-1.947	0.205651396	25.48	45.05	5.549	1.408
SPBC36B7.04	SPBC36B7.04	0.8219	-1.943	0.08518102	34.13	59.25	12.63	2.24
SPAC6F12.09	rdp1	0.468	-1.93	0.329754147	27.02	47.56	3.575	1.084
SPBP8B7.21	ubp3	0.8043	-1.929	0.094581931	30.55	53.34	11.52	1.553
SPAC343.12	rds1	0.7638	-1.918	0.117020346	32.97	57.3	9.011	2.352
SPAC6B12.08	mug185	0.8279	-1.916	0.082022117	24.17	42.85	10.75	5.302
SPBC354.10	def1	0.8558	-1.908	0.067627718	18.54	33.59	12.58	6.507
SPBP26C9.02C	car1	0.8076	-1.905	0.09280369	32.95	57.25	11.41	2.129
SPBC337.11	SPBC337.11	0.889	-1.902	0.051098239	42.09	72.27	16.95	7.939
SPCC622.11	SPCC622.11	0.8256	-1.902	0.083230316	33.6	58.32	12.37	2.898
SPBC17A3.10	pas4	0.7682	-1.898	0.114525697	16.29	29.87	6.91	4.49
SPBC16H5.09C	omh2	0.4694	-1.897	0.328456915	22.33	39.8	3.511	1.096
SPAC6G9.04	spo7	0.8128	-1.893	0.090016305	31.04	54.09	11.43	2.72
SPAC1782.01	ecm29	0.83	-1.888	0.080921908	35.69	61.72	12.98	1.968
SPCC1235.12C	mug146	0.8761	-1.879	0.05744632	39.94	68.69	17.83	2.604
SPBC577.03C	SPBC577.03c	0.6515	-1.879	0.18608558	29.32	51.25	5.249	2.294
SPAC4D7.01C	sec71	0.3173	-1.872	0.498529928	33.7	58.43	1.771	1.328
SPBC359.01	SPBC359.01	0.8303	-1.862	0.080764962	28.49	49.85	11.61	4.225
SPBC839.06	cta3	0.5156	-1.853	0.287687091	30.58	53.27	3.257	1.805
SPAC24B11.12C	SPAC24B11.12c	0.4378	-1.85	0.358724243	28.85	50.43	2.214	1.759
SPAC31G5.21	SPAC31G5.21	0.8124	-1.83	0.090230085	30.59	53.25	10.26	3.797
SPAC57A10.03	cyp1	0.7521	-1.823	0.123724411	30.56	53.19	7.772	2.696
SPAC1486.01	SPAC1486.01	0.6543	-1.811	0.18422308	24.63	43.42	4.645	2.604
SPCC2H8.05C	SPCC2H8.05c	0.8111	-1.81	0.090925599	32.58	56.48	11.02	2.12
SPCC4E9.02	cig1	0.5399	-1.807	0.267686673	38.36	65.97	2.613	2.258
SPAC3A12.09C	SPAC3A12.09c	0.7029	-1.806	0.153106457	32.03	55.59	6.028	2.588
SPAC890.06	nup155	0.7893	-1.805	0.102757897	31.87	55.32	9.492	2.598
SPAC13A11.06	SPAC13A11.06	0.8289	-1.805	0.08149786	33.3	57.67	12.08	2.573
SPCC622.08C	hta1	0.4626	-1.798	0.334794372	33.76	58.41	1.567	2.016
SPAC25B8.13C	isp7	0.6899	-1.796	0.161213855	31.32	54.39	5.871	2.302
SPBC4C3.04C	SPBC4C3.04c	0.8103	-1.793	0.091354161	32.9	56.99	11.04	1.595
SPBC354.15	fap1	0.7966	-1.79	0.098759698	34.52	59.64	10.16	1.798
SPAC11E3.14	SPAC11E3.14	0.6932	-1.787	0.159141446	26.83	47	5.941	2.284
SPAC56F8.12	SPAC56F8.12	0.6584	-1.786	0.181510178	26.61	46.64	5.644	1.515
SPAC139.05	SPAC139.05	0.8187	-1.786	0.08687521	33.97	58.74	11.26	2.387
SPBC23G7.16	ctr6	0.3979	-1.782	0.400226061	19.75	35.37	1.536	1.675
SPBC2G2.09C	crs1	0.815	-1.779	0.088842391	31.33	54.38	11.01	2.268
SPCC1322.09	SPCC1322.09	0.8692	-1.771	0.060880282	21	37.41	12	7.273
SPAPB1E7.04C	SPAPB1E7.04c	0.8741	-1.768	0.05843888	28.41	49.57	15.02	5.321
SPCC794.10	SPCC794.10	0.7999	-1.767	0.096964303	30.65	53.25	10.17	1.882

SPAC23H4.12	alp13	0.1852	-1.758	0.732359018	36.61	63.03	1.231	0.8981
SPBC19C7.09C	uve1	0.7552	-1.756	0.121938019	34.08	58.86	8.059	1.853
SPAC21E11.03C	pcr1	0.8189	-1.747	0.086769129	32.11	55.62	11.4	1.112
SPAC144.06	apl5	0.725	-1.746	0.139661993	25.54	44.82	6.173	2.857
SPAC23D3.10C	eng2	0.8326	-1.744	0.079563593	30.05	52.22	11.73	2.971
SPBC1348.02	SPBC1348.02	0.8743	-1.739	0.058339522	25.42	44.6	13.5	6.559
SPAC29B12.04	snz1	0.4872	-1.734	0.31229272	19.59	35.02	3.31	1.108
SPBC2G5.04C	SPBC2G5.04c	0.7616	-1.731	0.118273065	32.72	56.6	8.064	2.082
SPAC890.03	ppk16	0.7548	-1.725	0.122168109	27.58	48.13	8.05	1.446
SPBC14F5.10C	SPBC14F5.10c	0.7991	-1.714	0.097398869	14.42	26.5	8.182	4.066
SPBC1198.01	SPBC1198.01	0.7479	-1.713	0.126156467	31.14	53.96	7.943	0.6389
SPBC31F10.07	lsb5	0.5142	-1.71	0.288867928	24.27	42.68	2.742	1.815
SPAC2E1P3.05C	SPAC2E1P3.05c	0.5177	-1.703	0.285921835	28.84	50.17	3.645	0.9279
SPCC13B11.01	adh1	0.7194	-1.696	0.143029567	23.54	41.45	6.33	2.223
SPAC1296.03C	sxa2	0.8178	-1.695	0.087352894	31.71	54.87	10.33	2.865
SPCC320.06	SPCC320.06	0.7289	-1.693	0.13733205	30.72	53.24	5.264	3.383
SPAC17A2.09C	csx1	0.7647	-1.68	0.11650891	27.98	48.73	7.955	2.017
SPAC27D7.12C	but1	0.7926	-1.68	0.100945932	23.69	41.68	8.294	3.37
SPAC3G9.08	png1	0.7858	-1.674	0.104687976	12.1	22.62	7.725	3.509
SPAP27G11.12	SPAP27G11.12	0.6818	-1.657	0.166343003	29.17	50.64	5.047	2.29
SPAC23A1.03	apt1	0.8602	-1.65	0.065400562	36.21	62.18	13.8	2.222
SPAC105.01C	kha1	0.4901	-1.649	0.309715297	34.63	59.59	1.47	1.989
SPAC2F3.02	SPAC2F3.02	0.7948	-1.627	0.099742142	26.35	45.96	8.555	2.772
SPAC1705.02	SPAC1705.02	0.8458	-1.626	0.072732319	33.42	57.57	11.84	3.091
SPBPB21E7.01C	eno102	0.7795	-1.625	0.10818388	32.11	55.41	7.81	2.734
SPAC589.02C	med13	0.8634	-1.623	0.063787956	21.2	37.48	11.42	5.751
SPCC1672.09	SPCC1672.09	0.7793	-1.622	0.108295324	28.11	48.84	7.887	2.606
SPAC14C4.12C	laf1	0.7546	-1.614	0.122283199	25.33	44.26	6.829	2.571
SPBC12C2.04	SPBC12C2.04	0.8675	-1.613	0.061730517	21.3	37.63	11.73	5.875
SPAC10F6.13C	SPAC10F6.13c	0.7865	-1.608	0.104301273	13.45	24.74	5.561	4.53
SPAC1002.03C	gls2	0.8165	-1.604	0.088043811	35.29	60.6	10.23	1.414
SPAC10F6.15	SPAC10F6.15	0.7565	-1.603	0.121191068	31.85	54.95	7.505	1.432
SPBC4.01	dni2	0.8715	-1.598	0.059732609	34.03	58.52	13.95	3.773
SPBC1773.12	SPBC1773.12	0.5878	-1.598	0.230770418	14.32	26.14	3.42	1.855
SPCC622.16C	epe1	0.6709	-1.597	0.173342208	31.54	54.42	4.836	1.989
SPCC1840.11	csl4	0.8522	-1.594	0.06945847	24.39	42.67	8.613	6.285
SPBC18E5.07	SPBC18E5.07	0.8018	-1.587	0.095933948	31.81	54.85	8.917	2.39
SPCC584.12	mug42	0.8527	-1.577	0.069203737	33.44	57.51	12.1	3.026
SPCC1442.07C	SPCC1442.07c	0.3553	-1.576	0.449404793	32.89	56.61	1.65	1.207
SPBC2A9.02	SPBC2A9.02	0.6134	-1.555	0.212256228	27.46	47.66	3.389	2.064
SPCC61.03	SPCC61.03	0.844	-1.548	0.073657553	33.97	58.34	11.06	3.066
SPBC106.19	SPBC106.19	0.8685	-1.541	0.061230177	28.45	49.26	12.98	3.813
SPCC645.08C	snd1	0.828	-1.536	0.081969663	34.62	59.39	10.39	1.72
SPAC23A1.07	SPAC23A1.07	0.7513	-1.532	0.124186611	15.29	27.63	5.556	3.141
SPBP35G2.12	SPBP35G2.12	0.8015	-1.531	0.096096473	32.36	55.67	8.55	2.367
SPBC25B2.02C	mam1	0.4065	-1.531	0.39093945	36.4	62.31	1.501	1.426
SPBC1734.15	rsc4	0.7127	-1.529	0.147093241	28.98	50.12	4.884	2.612
SPAC23C4.08	rho3	0.8104	-1.528	0.091300568	30.39	52.42	9.299	1.699
SPAC9E9.14	vps24	0.7719	-1.524	0.112438959	23.17	40.56	7.15	2.378
SPBC2G2.13C	SPBC2G2.13c	0.7205	-1.522	0.142366015	29.74	51.35	6.085	1.374
SPBC25H2.10C	SPBC25H2.10c	0.8532	-1.518	0.068949153	29.59	51.1	11.86	2.574
SPCC1223.06	tea1	0.8621	-1.502	0.064442355	29.36	50.7	12.39	2.945
SPAC3F10.06C	SPAC3F10.06c	0.6406	-1.499	0.193413066	28.9	49.94	3.693	2.06
SPBC16D10.05	mok13	0.863	-1.494	0.063989204	34.08	58.44	12.39	2.997
SPAC869.09	SPAC869.09	0.7538	-1.485	0.122743867	29.7	51.23	6.171	2.459
SPBC1861.06C	mug131	0.7256	-1.484	0.139302726	29.76	51.33	5.513	2.18
SPBC1105.09	ubc15	0.7904	-1.482	0.102153068	28.43	49.14	8.038	1.728
SPBC21.03C	SPBC21.03c	0.8409	-1.472	0.075255648	22.64	39.61	8.835	4.501
SPAC22E12.11C	set3	0.39	-1.469	0.408935393	22.85	39.95	2.151	0.8603
SPCC1450.08C	wtf16	0.8666	-1.465	0.062181315	28.32	48.92	10.64	5.257
SPAC18G6.12C	SPAC18G6.12c	0.8015	-1.463	0.096096473	26.95	46.67	7.97	2.568
SPAC8C9.06C	ppr4	0.8402	-1.454	0.075617323	33.53	57.46	10.76	1.131
SPAC1B3.07C	vps28	0.6511	-1.441	0.186352305	15.05	27.09	3.855	1.906
SPBC21C3.20C	git1	0.6946	-1.44	0.158265221	19.01	33.59	3.801	2.615
SPAPYUG7.02C	sin1	0.7665	-1.429	0.115487841	15.39	27.62	5.509	3.322
SPAPB1E7.02C	mcl1	0.8619	-1.428	0.064543119	13.34	24.25	10.01	4.951
SPBC776.01	rpl29	0.884	-1.426	0.053547735	32.79	56.21	14.49	2.02
SPBC13G1.13	tfb2	0.5885	-1.423	0.230253533	30	51.61	3.419	1.321
SPAC25B8.18	SPAC25B8.18	0.7491	-1.421	0.125460203	35.37	60.44	5.572	2.521

SPAC227.04	SPAC227.04	0.903	-1.417	0.04431225	24.31	42.26	12.44	8.167
SPAC959.07	rps403	0.8755	-1.417	0.05774385	18.86	33.31	9.031	6.68
SPCP20C8.02C	SPCP20C8.02c	0.7148	-1.416	0.145815456	28.85	49.71	5.243	1.759
SPBC1604.11	atp17	0.394	-1.413	0.404503778	32.83	56.26	2.162	0.7342
SPAC25G10.06	rps2801	0.382	-1.41	0.417936637	35.92	61.32	1.368	1.222
SPBC428.17C	wpl1	0.8036	-1.407	0.094960072	33.75	57.74	8.34	1.256
SPBC1773.09C	mug184	0.7418	-1.405	0.129713171	28.8	49.62	5.534	2.233
SPBPJ4664.02	SPBPJ4664.02	0.8567	-1.401	0.067171233	34.55	59.05	11.47	1.721
SPBC839.15C	ef1a-c	0.9004	-1.401	0.045564514	19.53	34.38	12.91	7.305
SPBC215.10	SPBC215.10	0.8103	-1.382	0.091354161	32.94	56.38	8.137	2.139
SPBC2F12.12C	SPBC2F12.12c	0.8249	-1.381	0.083598696	11.91	21.84	7.32	3.974
SPBC660.06	SPBC660.06	0.8361	-1.379	0.077741777	26.31	45.48	8.645	3.546
SPAC222.04C	ies6	0.8338	-1.378	0.078938109	12.09	22.13	7.792	4.12
SPBC3H7.12	rav2	0.4315	-1.377	0.3650192	0.6593	3.345	2.366	0.6593
SPBC29A10.01	ccr1	0.8003	-1.375	0.096747183	32.39	55.47	6.913	3.015
SPCC285.10C	SPCC285.10c	0.8656	-1.368	0.062682752	33.82	57.8	11.73	2.425
SPBC530.08	SPBC530.08	0.8689	-1.368	0.061030203	33.65	57.51	11.91	2.748
SPAC3F10.02C	trk1	0.8385	-1.367	0.076496933	23.22	40.39	8.476	3.781
SPBC1921.01C	rpl35b	0.7541	-1.366	0.122571059	24.86	43.08	5.68	2.271
SPAC56F8.09	rrp8	0.87	-1.359	0.060480747	29.77	51.13	10.83	4.355
SPBC530.05	SPBC530.05	0.9066	-1.352	0.042584285	26.9	46.41	14.25	6.805
SPCC23B6.04C	SPCC23B6.04c	0.8748	-1.352	0.058091226	35.12	59.91	12.49	2.494
SPAC8F11.02C	dph3	0.7496	-1.35	0.125170422	24.83	43.01	4.261	3.089
SPAC4F8.11	SPAC4F8.11	0.6837	-1.348	0.16513442	26.44	45.64	4.668	1.17
SPAC19A8.11C	SPAC19A8.11c	0.8665	-1.345	0.062231433	33.18	56.71	11.59	2.448
SPAC9G1.05	SPAC9G1.05	0.7798	-1.344	0.108016769	28.16	48.47	6.255	2.516
SPCC777.17C	SPCC777.17c	0.8114	-1.343	0.090764997	32.04	54.84	8.023	1.968
SPBC19G7.17	SPBC19G7.17	0.8125	-1.343	0.09017663	23.68	41.11	7.023	3.299
SPAC23C4.11	atp18	0.7536	-1.341	0.12285911	34.92	59.57	6.243	1.063
SPCC306.02C	SPCC306.02c	0.8138	-1.34	0.089482314	23.26	40.41	7.303	3.095
SPAC6C3.03C	SPAC6C3.03c	0.534	-1.333	0.272458743	37.04	63.02	2.738	1.109
SPAC25G10.01	SPAC25G10.01	0.6062	-1.329	0.217384068	33.82	57.73	2.165	2.033
SPBC19C2.13C	ctu2	0.7388	-1.325	0.131473113	25.1	43.41	5.463	1.698
SPBC16H5.11C	skb1	0.7717	-1.32	0.1125515	30.99	53.07	6.499	1.535
SPAC227.05	SPAC227.05	0.3352	-1.32	0.47469599	33.64	57.43	1.099	1.045
SPAC13G6.12C	chs1	0.783	-1.312	0.106238238	29.05	49.88	6.104	2.589
SPBC29A3.18	cyt1	0.7899	-1.311	0.102427886	9.035	17	6.419	2.822
SPAC30D11.05	aps3	0.7543	-1.306	0.122455892	30.38	52.05	5.467	2.133
SPAC11D3.04C	SPAC11D3.04c	0.8795	-1.301	0.055764156	28.71	49.3	12.37	2.784
SPBC530.13	lsc1	0.8255	-1.3	0.083282922	30.93	52.94	8.648	1.492
SPAP8A3.03	SPAP8A3.03	0.7744	-1.298	0.111034656	26.64	45.89	6.106	2.117
SPAC1751.04	SPAC1751.04	0.8478	-1.297	0.071706588	32.1	54.85	9.288	2.956
SPBC26H8.05C	SPBC26H8.05c	0.8643	-1.296	0.063335487	33.15	56.58	10.99	2.312
SPCC1322.05C	SPCC1322.05c	0.825	-1.292	0.083546051	31.99	54.67	8.212	2.266
SPAC16E8.05C	mde1	0.9011	-1.289	0.04522701	18.48	32.47	12.07	6.697
SPBC1711.14	rec15	0.9253	-1.288	0.033717438	26.98	46.44	15.69	9.069
SPBC215.07C	SPBC215.07c	0.8792	-1.283	0.055912321	31.05	53.11	12.26	2.539
SPBC32F12.12C	SPBC32F12.12c	0.8468	-1.282	0.072219151	35.24	60	9.784	1.557
SPBC29A10.09C	SPBC29A10.09c	0.8675	-1.282	0.061730517	33.4	56.97	11.14	2.352
SPAC1805.08	dlc1	0.7214	-1.281	0.141823862	29.78	51.03	4.603	1.941
SPAPB1A10.05	SPAPB1A10.05	0.3936	-1.276	0.40494491	35.1	59.75	1.412	1.084
SPCPJ732.03	meu15	0.7943	-1.274	0.100015437	9.247	17.28	5.512	3.254
SPBC6B1.04	mde4	0.662	-1.274	0.179142011	14.58	26.04	3.385	1.85
SPCPB16A4.03C	ade10	0.6436	-1.273	0.191383965	25.88	44.61	1.569	2.361
SPAC23H3.03C	npr2	0.8144	-1.269	0.089162235	27.91	47.93	7.166	2.696
SPCC188.08C	ubp5	0.4717	-1.264	0.326334124	36.22	61.57	1.161	1.442
SPAC922.04	SPAC922.04	0.8287	-1.263	0.081602661	29.53	50.57	6.875	3.696
SPBC409.20C	psh3	0.8944	-1.251	0.048468209	19.28	33.73	8.645	7.294
SPAC23H3.09C	gly1	0.8383	-1.242	0.076600534	11.23	20.49	7.17	3.856
SPBC13E7.08C	SPBC13E7.08c	0.8538	-1.241	0.06864385	30.54	52.21	9.753	2.046
SPAC227.17C	SPAC227.17c	0.7767	-1.234	0.109746695	33.92	57.74	5.798	2.121
SPAC2G11.05C	SPAC2G11.05c	0.8655	-1.23	0.062732928	31.88	54.39	10.32	2.631
SPAPB2B4.02	grx5	0.9049	-1.227	0.043399412	21.79	37.81	11.88	6.668
SPBC1815.01	eno101	0.5236	-1.224	0.281000362	33.85	57.62	1.767	1.441
SPBC20F10.06	mad2	0.858	-1.223	0.066512712	34.23	58.24	9.794	2.317
SPBC8D2.19	mde3	0.7935	-1.21	0.100453069	24.33	41.96	5.526	2.86
SPCP31B10.04	SPCP31B10.04	0.8635	-1.204	0.063737658	32.54	55.42	9.632	3.062
SPBC725.06C	ppk31	0.879	-1.204	0.056011125	32.56	55.45	11	3.294
SPBC1289.13C	SPBC1289.13c	0.8991	-1.2	0.046192002	22.6	39.1	10.09	6.673

SPAC630.04C	SPAC630.04c	0.8451	-1.199	0.073091898	32.91	56.02	7.933	3.286
SPAC1834.07	klp3	0.8694	-1.198	0.060780364	33.78	57.45	10.28	2.776
SPAC29E6.05C	SPAC29E6.05c	0.872	-1.194	0.059483515	34.58	58.76	10.66	2.447
SPCC1682.11C	SPCC1682.11c	0.8235	-1.194	0.084336396	29.2	49.92	7.682	1.774
SPBC649.03	rhp14	0.8085	-1.194	0.092319976	5.784	11.46	5.466	3.34
SPAC22A12.17C	SPAC22A12.17c	0.8636	-1.193	0.063687366	24.87	42.82	8.605	4.075
SPCC1919.05	SPCC1919.05	0.9	-1.192	0.045757491	16.83	29.6	11.02	6.125
SPBC1685.06	cid11	0.8569	-1.191	0.067069857	32.72	55.7	9.686	1.707
SPAC13G6.15C	SPAC13G6.15c	0.7648	-1.19	0.116452121	29.23	49.97	5.531	1.608
SPBC11C11.01	SPBC11C11.01	0.8663	-1.189	0.062331686	33.9	57.64	10.23	2.163
SPBC1734.11	mas5	0.8498	-1.188	0.070683273	9.142	16.97	6.251	4.636
SPAC694.06C	mrc1	0.8872	-1.181	0.051978467	30.6	52.2	11.63	3.387
SPAC16C9.07	pom2	0.866	-1.18	0.062482108	31.27	53.3	9.725	2.906
SPBC23E6.09	ssn6	0.6545	-1.176	0.184090349	30.57	52.15	3.812	0.6374
SPCC4G3.17	SPCC4G3.17	0.8715	-1.173	0.059732609	34.56	58.69	10.38	2.485
SPBC25B2.03	SPBC25B2.03	0.8702	-1.17	0.060380921	32.49	55.29	9.615	3.449
SPBC29A10.12	SPBC29A10.12	0.8764	-1.17	0.057297631	32.12	54.68	10.64	2.824
SPBC12C2.08	dnm1	0.8561	-1.167	0.067475503	27.39	46.9	8.939	2.688
SPAC19B12.07C	SPAC19B12.07c	0.8132	-1.164	0.08980263	29.79	50.85	6.133	2.854
SPCC613.02	SPCC613.02	0.8457	-1.161	0.072783669	32.79	55.77	8.579	1.956
SPBC17A3.05C	SPBC17A3.05c	0.757	-1.159	0.12090412	19.51	33.95	4.558	2.248
SPBC21B10.02	SPBC21B10.02	0.7295	-1.152	0.136974704	30.87	52.6	3.939	2.085
SPAC17A2.12	SPAC17A2.12	0.8686	-1.149	0.061180175	29.05	49.6	6.577	5.288
SPCC1442.03	SPCC1442.03	0.8542	-1.148	0.068440433	28.59	48.85	7.511	3.854
SPAC19G12.09	SPAC19G12.09	0.7798	-1.144	0.108016769	27.06	46.33	4.895	2.525
SPAC630.07C	SPAC630.07c	0.8649	-1.143	0.063034103	28.45	48.61	8.834	3.443
SPCC1620.02	wtf23	0.59	-1.139	0.229147988	38.38	64.91	2.623	1.194
SPBC25B2.06C	btb2	0.8611	-1.126	0.064946411	32.32	54.93	9.588	1.13
SPCC132.04C	gdh2	0.8876	-1.123	0.051782706	32.14	54.63	10.8	3.669
SPAP8A3.07C	SPAP8A3.07c	0.9111	-1.122	0.040433953	19.5	33.87	11.49	6.624
SPAC1002.18	urg3	0.7652	-1.122	0.116225039	24.5	42.09	2.756	3.117
SPBC31F10.14C	hip3	0.6615	-1.121	0.179470151	21.95	37.89	2.418	1.929
SPAC8E11.05C	SPAC8E11.05c	0.9163	-1.121	0.037962313	20.69	35.83	12.1	7.09
SPBC21C3.18	spo4	0.8537	-1.117	0.068694719	26.28	45	8.044	3.021
SPBC1271.10C	SPBC1271.10c	0.659	-1.11	0.181114585	34.98	59.29	1.358	2.175
SPAC17A5.14	exo2	0.8846	-1.108	0.053253065	34.76	58.92	10.83	2.823
SPAC20H4.05C	SPAC20H4.05c	0.8846	-1.107	0.053253065	35.67	60.41	10.84	2.782
SPAC17C9.08	pnu1	0.8261	-1.102	0.082967378	31.15	52.97	6.608	2.553
SPCC320.12	atp23	0.7651	-1.102	0.116281798	8.422	15.64	3.778	2.666
SPBC1604.03C	SPBC1604.03c	0.8252	-1.095	0.083440781	28.54	48.67	6.233	2.819
SPAC18B11.08C	SPAC18B11.08c	0.878	-1.094	0.056505484	33.41	56.68	10.19	2.474
SPCC1450.07C	SPCC1450.07c	0.8878	-1.077	0.051684859	33.9	57.44	11.17	2.056
SPAC664.01C	swi6	0.8972	-1.068	0.047110735	30.38	51.66	11.62	3.236
SPAC1687.14C	SPAC1687.14c	0.8921	-1.067	0.049586461	35.95	60.81	11.51	2.147
SPAC30C2.07	SPAC30C2.07	0.7528	-1.065	0.12332039	22.74	39.1	3.532	2.408
SPBC32H8.02C	nep2	0.7954	-1.061	0.099414413	26.63	45.49	5.699	1.664
SPBC1711.01C	mat1-m	0.8992	-1.06	0.046143702	33.93	57.47	11.98	2.882
SPCC417.06C	mug27	0.8717	-1.056	0.059632954	33.33	56.49	9.509	1.926
SPCC4G3.09C	gyp3	0.8909	-1.054	0.050171041	30.27	51.46	10.84	2.965
SPAC607.08C	SPAC607.08c	0.86	-1.054	0.065501549	28.77	49	8.235	2.592
SPAC6F12.06	SPAC6F12.06	0.8066	-1.054	0.093341782	27.51	46.91	6.17	1.428
SPCC18.01C	adg3	0.6883	-1.051	0.16222223	34.13	57.78	3.392	1.368
SPAC26A3.07C	rpl1101	0.7937	-1.044	0.10034362	24.38	41.76	5.469	1.762
SPBC29A3.02C	his7	0.9273	-1.041	0.03277974	21.59	37.18	13.33	7.335
SPAC30D11.01C	SPAC30D11.01c	0.8526	-1.039	0.069254672	31.52	53.48	7.059	3.164
SPCC965.07C	gst2	0.7342	-1.038	0.13418562	18.33	31.82	3.709	1.841
SPAC20G4.08	SPAC20G4.08	0.8921	-1.034	0.049586461	32.2	54.6	11.31	1.578
SPBC8D2.04	hht2	0.9347	-1.013	0.029327757	24.22	41.45	14.1	8.186
SPCC18B5.11C	cds1	0.8681	-1.012	0.061430244	32.44	54.94	8.557	2.397
SPAC9G1.06C	cyk3	0.6515	-1.011	0.18608558	33.83	57.23	2.784	1.274
SPAC57A10.02	cdr2	0.8832	-1.01	0.05394094	13.43	23.73	7.888	4.512
SPCC338.08	ctp1	0.9031	-1.01	0.044264158	9.838	17.82	9.089	5.717
SPBC342.06C	rtt109	0.8597	-1.01	0.065653073	34.23	57.88	8.505	1.04
SPAC6G10.03C	SPAC6G10.03c	0.8921	-1.002	0.049586461	33.43	56.56	10.93	1.638
SPBC2D10.03C	SPBC2D10.03c	0.8454	-0.9836	0.072937757	32.81	55.51	7.282	1.597
SPAC1039.02	SPAC1039.02	0.7822	-0.9833	0.106682188	29.5	50.08	5.107	1.15
SPAC630.06C	SPAC630.06c	0.7397	-0.9824	0.130944381	27.83	47.32	3.701	1.682
SPAC144.04C	spe1	0.8345	-0.9756	0.078573659	32.57	55.1	6.697	1.558
SPAP11E10.01	SPAP11E10.01	0.8878	-0.9736	0.051684859	32.31	54.67	9.894	2.353

SPAC26H5.02C	SPAC26H5.02c	0.861	-0.9721	0.064996849	30.8	52.19	7.906	1.982
SPBC14C8.03	fma2	0.885	-0.9693	0.053056729	28.34	48.15	9.804	1.831
SPAC186.07C	SPAC186.07c	0.6047	-0.9689	0.218460031	39.56	66.57	1.83	1.376
SPBC27.04	uds1	0.8712	-0.9659	0.059882133	32.4	54.8	8.639	1.802
SPBC418.02	SPBC418.02	0.8732	-0.9591	0.058886273	31.95	54.06	8.14	2.811
SPBC106.11C	plg7	0.8596	-0.9548	0.065703593	30.56	51.77	7.807	1.685
SPCC737.06C	SPCC737.06c	0.8395	-0.9459	0.0759793	31.03	52.52	6.32	2.179
SPBP22H7.04	SPBP22H7.04	0.7483	-0.9457	0.125924255	31.68	53.59	3.515	1.822
SPAC1A6.05C	SPAC1A6.05c	0.8685	-0.9449	0.061230177	33.06	55.86	8.137	2.034
SPAC8F11.10C	pvg1	0.8702	-0.9436	0.060380921	11.12	19.82	6.252	4.017
SPAC824.08	gda1	0.8343	-0.9421	0.078677756	29.1	49.35	6.507	1.397
SPAC2F7.08C	snf5	0.9002	-0.936	0.045660991	32.27	54.54	10.38	3.107
SPAC2F7.06C	pol4	0.8916	-0.934	0.04982994	31.66	53.54	9.145	3.377
SPBC146.09C	lsd1	0.3257	-0.9332	0.487182241	22.69	38.81	1.214	0.4248
SPCC794.11C	SPCC794.11c	0.9215	-0.9267	0.03550466	31.19	52.75	13.08	3.937
SPAC1782.06C	phb1	0.6154	-0.9196	0.210842508	24.31	41.44	2.571	0.6347
SPCC1919.13C	SPCC1919.13c	0.892	-0.9187	0.049635146	44.96	75.36	8.384	3.978
SPBC1604.09C	SPBC1604.09c	0.839	-0.9182	0.076238039	31.09	52.58	6.475	1.528
SPCC1322.10	SPCC1322.10	0.8874	-0.9027	0.051880576	11.91	21.05	7.5	4.057
SPAC24H6.11C	SPAC24H6.11c	0.9346	-0.898	0.029374223	21.67	37.07	12.1	7.475
SPCC126.01C	SPCC126.01c	0.8517	-0.8967	0.069713353	20.19	34.63	3.814	3.912
SPCC320.04C	SPCC320.04c	0.8776	-0.8945	0.056703385	33.24	56.08	8.087	2.396
SPAC3H1.06C	SPAC3H1.06c	0.8829	-0.8844	0.054088483	30.5	51.56	7.887	3.102
SPAC31G5.12C	maf1	0.9366	-0.8778	0.028445847	21.62	36.95	12.23	7.518
SPCC330.11	btb1	0.9393	-0.8741	0.027195678	21.91	37.42	13.34	7.439
SPBC776.09	ste13	0.9053	-0.8716	0.04320748	13.68	23.91	8.281	4.892
SPBC13G1.03C	pex14	0.8681	-0.8558	0.061430244	21.52	36.76	5.994	3.325
SPBC725.10	SPBC725.10	0.7264	-0.8528	0.138824164	18.76	32.22	1.603	2.063
SPBC1604.07	atp4	0.8292	-0.8499	0.081340707	28.49	48.19	5.758	1.051
SPAC17D4.03C	cis4	0.7952	-0.8416	0.099523629	29.99	50.64	4.379	1.512
SPAC3H8.04	SPAC3H8.04	0.8325	-0.8407	0.079615758	28.86	48.78	4.653	2.54
SPAC1D4.03C	aut12	0.7769	-0.8403	0.109634879	28.44	48.1	4.086	1.265
SPBC365.08C	SPBC365.08c	0.8983	-0.8356	0.0465786	29.51	49.84	9.381	2.219
SPBC1703.08C	SPBC1703.08c	0.9023	-0.8334	0.044649043	30.31	51.15	9.595	2.583
SPAC18B11.03C	SPAC18B11.03c	0.9466	-0.831	0.0238335	24.63	41.82	14.1	8.235
SPBC2D10.20	ubc1	0.7443	-0.8234	0.128251981	30.55	51.53	3.518	1.004
SPBC16A3.18	cip1	0.819	-0.8222	0.086716098	34.24	57.59	4.362	2.176
SPAC630.13C	tsc2	0.7328	-0.8193	0.135014539	36.21	60.82	2.85	1.492
SPAC23G3.10C	ssr3	0.8557	-0.8188	0.067678468	29.02	49.01	3.436	3.716
SPAC1786.01C	SPAC1786.01c	0.7615	-0.8122	0.118330092	31	52.26	3.009	1.783
SPBC21C3.11	ubx4	0.8681	-0.8121	0.061430244	31.99	53.87	6.334	2.614
SPAC4D7.06C	SPAC4D7.06c	0.7479	-0.8117	0.126156467	21.81	37.15	1.739	2.123
SPACUNK4.12C	mug138	0.8274	-0.8059	0.082284483	37.26	62.52	4.15	2.604
SPCC550.11	SPCC550.11	0.6905	-0.8039	0.160836317	1.993	4.594	2.367	1.272
SPBC29A3.08	pof4	0.9089	-0.8029	0.041483897	30.39	51.23	8.005	4.634
SPBC21C3.08C	car2	0.391	-0.7885	0.407823243	0	1.295	1.295	0
SPBC1215.01	shy1	0.8959	-0.7873	0.047740463	25.08	42.48	8.013	2.985
SPAC30D11.07	nth1	0.8612	-0.7857	0.064895979	28.79	48.58	5.811	2.41
SPBC1706.01	tea4	0.9391	-0.7823	0.027288159	19.94	34.03	11.59	6.829
SPAC1002.05C	jmj2	0.853	-0.781	0.069050969	33.31	56	4.825	2.767
SPBC405.04C	ypt7	0.9076	-0.7763	0.042105513	32.97	55.43	9.57	2.335
SPAC1093.01	ppr5	0.85	-0.7753	0.070581074	26.65	45.05	4.375	2.884
SPBC18H10.02	lcf1	0.8775	-0.7583	0.056752875	16.27	27.97	5.535	3.461
SPBC577.12	mug71	0.7074	-0.7569	0.150334945	28.41	47.91	2.72	0.9263
SPAC17H9.06C	SPAC17H9.06c	0.921	-0.7561	0.03574037	33.96	57.03	10.27	3.648
SPBP35G2.08C	air1	0.3077	-0.7518	0.511872504	0	1.235	1.007	0
SPAC11E3.13C	gas5	0.9235	-0.7516	0.0345631	20.21	34.43	9.42	4.834
SPCC63.02C	aah3	0.391	-0.7493	0.407823243	0	1.231	1.231	0
SPBC2A9.03	SPBC2A9.03	0.8486	-0.7481	0.071296973	30.82	51.86	4.664	2.448
SPAC9.11	SPAC9.11	0.8322	-0.7406	0.079772289	23.04	39.07	4.563	1.824
SPCC126.02C	pku70	0.8594	-0.732	0.065804651	23.78	40.27	5.907	1.43
SPBC409.08	SPBC409.08	0.9494	-0.7216	0.022550773	21.08	35.8	13.3	7.307
SPCC11E10.01	SPCC11E10.01	0.924	-0.7209	0.034328029	23.3	39.45	8.94	4.783
SPAC1782.05	SPAC1782.05	0.8795	-0.7187	0.055764156	28.98	48.79	6.697	1.807
SPAC869.02C	SPAC869.02c	0.7167	-0.7179	0.144662596	33.99	57.02	1.854	1.503
SPBC36B7.08C	SPBC36B7.08c	0.9366	-0.7122	0.028445847	36.31	60.81	12.54	3.626
SPCC1919.07	SPCC1919.07	0.8889	-0.7117	0.051147094	26.97	45.47	5.712	3.429
SPAC19G12.02C	pms1	0.6478	-0.707	0.188559056	25.59	43.19	2.245	0.3723
SPBC18E5.14C	SPBC18E5.14c	0.9452	-0.705	0.024476287	15.57	26.74	9.941	7.729

SPAC1399.01C	SPAC1399.01c	0.9017	-0.7023	0.04493793	32.98	55.33	8.206	1.835
SPBC660.14	mik1	0.9409	-0.6856	0.026456531	27.53	46.34	10.92	5.864
SPBC409.06	uch2	0.8881	-0.6812	0.05153813	27.92	46.98	6.577	2.242
SPBC83.09C	SPBC83.09c	0.889	-0.6755	0.051098239	31.3	52.53	6.5	2.33
SPAC3C7.07C	SPAC3C7.07c	0.864	-0.6746	0.063486258	28.69	48.23	5.075	2.13
SPBC36B7.06C	mug20	0.9565	-0.6743	0.019315026	23.83	40.24	14.3	8.044
SPBC21B10.07	SPBC21B10.07	0.9395	-0.6665	0.027103216	24.39	41.16	10.51	5.473
SPAC17C9.14	SPAC17C9.14	0.9187	-0.6652	0.036826284	26.22	44.16	8.31	3.617
SPAC1039.09	isp5	0.8923	-0.6609	0.049489107	26.02	43.82	6.047	2.868
SPAC869.11	cat1	0.8823	-0.6604	0.054383721	20.56	34.85	4.977	3.014
SPBC16H5.13	SPBC16H5.13	0.9166	-0.6511	0.037820147	11.56	20.06	7.176	4.052
SPAC1250.04C	atl1	0.8395	-0.6509	0.0759793	25.08	42.26	4.463	1.339
SPBC776.03	SPBC776.03	0.8914	-0.6501	0.04992737	32.54	54.51	7.086	0.9146
SPBC17D11.04C	nto1	0.8286	-0.6406	0.081655071	31.9	53.45	3.362	1.959
SPCC24B10.16C	SPCC24B10.16	0.9551	-0.6393	0.019951155	21.62	36.56	13.15	7.373
SPCC1827.07C	SPCC1827.07c	0.9363	-0.6372	0.028584977	35.19	58.84	11.69	2.162
SPBC1685.09	rps29	0.6914	-0.6371	0.160270625	18.89	32.08	1.878	1.014
SPBC26H8.12	SPBC26H8.12	0.9027	-0.6362	0.044456558	5.925	10.78	6.032	3.377
SPCC1795.10C	SPCC1795.10c	0.865	-0.6353	0.062983893	28.49	47.84	3.695	2.776
SPCC622.15C	SPCC622.15c	0.8261	-0.6342	0.082967378	32.4	54.26	4.051	1.132
SPBC3E7.05C	SPBC3E7.05c	0.8728	-0.624	0.059085262	24.7	41.6	5.426	1.614
SPBC1685.07C	avt5	0.3041	-0.6235	0.51698358	0	1.024	0.8279	0
SPAC4G9.02	rnh201	0.917	-0.6226	0.037630664	32.95	55.14	8.078	2.816
SPBC713.07C	SPBC713.07c	0.9178	-0.6188	0.037251947	27.77	46.63	8.347	2.492
SPBC106.01	mph1	0.8862	-0.6172	0.052468254	22.88	38.59	3.4	3.531
SPAC13G6.04	tim8	0.8745	-0.6122	0.058240186	31.47	52.69	5.005	2.088
SPAC4G9.06C	chz1	0.9103	-0.6028	0.040815457	27.05	45.42	6.945	2.853
SPCC1753.02C	git3	0.8665	-0.5969	0.062231433	28.33	47.51	4.468	2.025
SPAPB8E5.08	SPAPB8E5.08	0.94	-0.5958	0.026872146	35.12	58.67	11.43	2.551
SPAC8E11.02C	rad24	0.9404	-0.5946	0.02668738	16.57	28.19	10.06	4.506
SPBC1198.03C	SPBC1198.03c	0.8902	-0.5868	0.05051241	29.53	49.47	5.685	2.079
SPAC821.10C	sod1	0.901	-0.5856	0.045275209	31.61	52.88	5.341	3.127
SPBC32F12.03C	gpx1	0.834	-0.5817	0.078833949	33.07	55.28	2.896	1.986
SPBC839.07	ibp1	0.72	-0.578	0.142667504	34.69	57.93	1.194	1.331
SPAC18G6.15	mal3	0.9477	-0.5755	0.023329119	26.1	43.81	11.04	5.02
SPBC29A10.11C	vps902	0.9049	-0.5688	0.043399412	29.99	50.19	7.006	1.198
SPAC14C4.14	atp1	0.9273	-0.5686	0.03277974	13.86	23.7	7.125	4.115
SPBC3H7.14	mug176	0.8705	-0.5681	0.060231225	28.49	47.72	3.847	2.381
SPAC1039.04	SPAC1039.04	0.929	-0.5679	0.031984286	37.09	61.85	9.589	0.7137
SPAC25H1.04	mug105	0.9481	-0.5644	0.023145853	24.58	41.3	10.95	4.924
SPBC17G9.09	tif213	0.9607	-0.5626	0.017412209	39.13	65.2	16.9	2.572
SPBC1683.12	SPBC1683.12	0.9417	-0.5617	0.02608743	27.15	45.52	10.27	3.786
SPAC13F5.03C	gld1	0.9619	-0.5616	0.016870075	22.38	37.69	13.72	7.55
SPAC4F10.11	spn1	0.9225	-0.5592	0.035033625	30.64	51.24	8.221	2.007
SPAC9.08C	SPAC9.08c	0.9092	-0.559	0.041340573	27.61	46.27	6.785	2.102
SPBP8B7.06	rpp201	0.9335	-0.5582	0.029885678	32.4	54.13	9.735	1.996
SPAC9G1.12	cpd1	0.8906	-0.5573	0.050317309	18.76	31.74	3.841	3.085
SPAC30D11.02C	SPAC30D11.02c	0.9471	-0.5551	0.023604163	34.31	57.27	12.32	2.115
SPCC1223.01	SPCC1223.01	0.9366	-0.5547	0.028445847	34.98	58.38	10.31	1.606
SPAC1639.02C	trk2	0.9211	-0.5544	0.035693218	5.841	10.5	6.283	3.952
SPBC3B9.11C	ctf1	0.9578	-0.5533	0.018725167	28.06	47	12.41	6.597
SPBC530.06C	SPBC530.06c	0.9419	-0.5515	0.025995203	14.73	25.1	8.386	5.151
SPAC3C7.12	tip1	0.9366	-0.5485	0.028445847	24.59	41.29	9.158	3.456
SPAC6C3.05	SPAC6C3.05	0.9111	-0.5473	0.040433953	29.64	49.58	6.149	2.815
SPBPB10D8.07C	SPBPB10D8.07	0.8732	-0.5464	0.058886273	35.47	59.15	4.54	1.715
SPAPB17E12.02	yip12	0.9148	-0.541	0.038673844	32.7	54.6	7.146	1.922
SPBC16E9.08	mcp4	0.9143	-0.5339	0.03891128	27.56	46.14	6.629	2.437
SPCC584.11C	SPCC584.11c	0.7826	-0.5332	0.106460156	19.54	32.98	2.43	1.095
SPAC9G1.11C	spn4	0.9096	-0.5318	0.041149548	31.86	53.21	6.617	1.79
SPCC794.12C	mae2	0.9459	-0.5307	0.024154775	25.08	42.07	7.778	5.797
SPBC216.06C	swi1	0.8996	-0.5296	0.045950553	30.56	51.07	4.49	2.954
SPCC1620.12C	SPCC1620.12c	0.8633	-0.5266	0.063838259	30.22	50.5	3.944	1.65
SPBC25D12.06	SPBC25D12.06	0.4562	-0.5249	0.340844719	0.2212	1.226	1.08	0.1191
SPAC19D5.02C	SPAC19D5.02c	0.9089	-0.5231	0.041483897	28.56	47.77	5.799	2.565
SPBP4H10.11C	lcf2	0.9109	-0.5207	0.040529298	28.39	47.48	6.059	2.458
SPAC4G9.11C	cmb1	0.8878	-0.515	0.051684859	27.2	45.53	4.519	2.145
SPAC27E2.11C	SPAC27E2.11c	0.5055	-0.5147	0.29627884	0.4921	1.654	0.8782	0.4921
SPBC2D10.18	abc1	0.525	-0.5143	0.279840697	0.7127	2.015	0.7957	0.6212
SPAC212.03	SPAC212.03	0.9207	-0.5118	0.035881857	25.93	43.44	6.042	3.279

SPAC27E2.02	SPAC27E2.02	0.6474	-0.5109	0.188827305	19.05	32.13	0.8556	0.9093
SPAC1782.04	cox24	0.9554	-0.5078	0.019814763	19.12	32.23	10.74	5.753
SPAC24H6.07	rps901	0.905	-0.507	0.043351421	8.068	14.09	4.731	2.881
SPAC18G6.04C	shm2	0.8916	-0.5067	0.04982994	27.7	46.33	2.448	3.17
SPBC17A3.02	SPBC17A3.02	0.9186	-0.5067	0.036873559	33.74	56.25	6.884	2.085
SPAC17A2.13C	rad25	0.9164	-0.4874	0.037914919	28.95	48.35	5.395	3.007
SPCC188.07	ccq1	0.929	-0.4859	0.031984286	18.58	31.32	5.242	4.123
SPCC663.11	saf1	0.8616	-0.4823	0.06469431	24.05	40.3	3.446	1.608
SPAPB24D3.07C	SPAPB24D3.07c	0.9663	-0.4749	0.01488802	21.63	36.3	12.98	7.351
SPAC22E12.03C	SPAC22E12.03c	0.9013	-0.4738	0.045130629	20.98	35.23	4.058	2.702
SPAC2C4.15C	ubx2	0.963	-0.4733	0.016373713	23.34	39.11	11.45	6.867
SPAC9G1.08C	SPAC9G1.08c	0.9674	-0.4718	0.014393917	25.12	42.04	11.8	8.414
SPBC21D10.09C	SPBC21D10.09c	0.8677	-0.4716	0.061630403	32.22	53.69	3.104	1.946
SPCC1259.10	pgp1	0.955	-0.4675	0.019996628	38.34	63.75	11.82	2.971
SPAPJ695.01C	SPAPJ695.01c	0.9653	-0.462	0.015337694	20.87	35.05	12.08	7.051
SPBC1711.04	SPBC1711.04	0.9543	-0.4492	0.020315076	33.14	55.17	11.17	2.836
SPBC29A3.10C	atp14	0.5794	-0.4479	0.237021509	0.2485	1.144	1.144	0.2485
SPBC119.06	sco1	0.9311	-0.4364	0.031003673	6.368	11.18	5.077	3.718
SPAC1782.07	qcr8	0.9436	-0.4342	0.025212068	24.97	41.73	8.119	3.123
SPCC4G3.15C	not2	0.9456	-0.4335	0.024292536	36.52	60.7	9.525	0.9497
SPCC794.07	lat1	0.9443	-0.4288	0.02489001	7.861	13.62	6.138	4.539
SPBC21D10.12	hob1	0.9316	-0.4287	0.03077052	29.75	49.57	6.58	2.558
SPBC2G2.02	syj1	0.9447	-0.4272	0.024706085	33.03	54.96	8.679	2.401
SPAC26H5.04	SPAC26H5.04	0.9201	-0.4248	0.036164969	32.18	53.55	5.506	2.247
SPCP1E11.07C	cwf18	0.9235	-0.4236	0.0345631	19.42	32.59	3.444	3.624
SPAPYUG7.06	mug67	0.9728	-0.4228	0.011976438	23.78	39.76	14.18	8.161
SPAC26F1.09	gyp51	0.8606	-0.4153	0.065198658	28.03	46.73	3.07	1.256
SPBC3E7.15C	lac1	0.797	-0.4136	0.098541679	0.8538	2.082	2.082	0.8538
SPBP23A10.05	ssr4	0.9631	-0.4112	0.016328617	37.12	61.64	13.03	2.415
SPCP31B10.05	tdp1	0.9691	-0.4059	0.013631406	22.27	37.24	12.13	6.815
SPAC10F6.11C	atg17	0.9128	-0.4057	0.039624369	28.54	47.55	5.433	0.9769
SPAC13C5.06C	mug121	0.9639	-0.4	0.01596802	29.67	49.39	9.908	5.962
SPBC1198.08	SPBC1198.08	0.9502	-0.3982	0.022184974	31.71	52.75	9.443	1.459
SPBC32H8.09	SPBC32H8.09	0.9513	-0.3978	0.021682503	12.74	21.58	7.402	4.337
SPBC557.02C	SPBC557.02c	0.9671	-0.3955	0.014528617	18.46	30.97	10.65	6.541
SPCC757.05C	SPCC757.05c	0.906	-0.3916	0.042871802	26.87	44.77	4.129	1.932
SPAC22A12.14C	SPAC22A12.14c	0.9596	-0.3893	0.017909761	34.51	57.32	11.17	2.329
SPAC19A8.02	SPAC19A8.02	0.8871	-0.3872	0.052027421	31.52	52.4	3.684	1.288
SPCC4F11.03C	SPCC4F11.03c	0.9457	-0.3865	0.024246611	30.37	50.52	8.214	1.801
SPAC31A2.13C	sft1	0.9243	-0.3854	0.034187047	21.24	35.52	2.653	3.467
SPAC30C2.08	SPAC30C2.08	0.906	-0.383	0.042871802	28.72	47.81	4.787	0.7741
SPAC823.13C	SPAC823.13c	0.9545	-0.38	0.020224067	33.27	55.27	9.661	2.069
SPAC23H3.06	apl6	0.9542	-0.3767	0.020360588	26.28	43.79	8.064	3.918
SPAC30D11.12	rpl3802	0.9275	-0.3657	0.032686082	30.29	50.36	5.335	2.019
SPBC30D10.03C	SPBC30D10.03c	0.9014	-0.3605	0.045082446	30.22	50.23	3.945	1.355
SPBC1703.11	SPBC1703.11	0.9495	-0.3555	0.022505031	31.22	51.86	7.639	2.593
SPAC22A12.07C	ogm1	0.9563	-0.3464	0.019405844	30.39	50.49	7.44	4.025
SPAC19B12.08	atg4	0.9645	-0.3435	0.015697768	33.81	56.1	10.93	2.911
SPAC11H11.02C	mug162	0.9485	-0.3428	0.022962665	28.94	48.1	6.756	2.959
SPBC19F8.01C	spn7	0.9689	-0.3426	0.013721044	21.83	36.43	9.819	5.929
SPAC23D3.13C	SPAC23D3.13c	0.9476	-0.3381	0.023374948	29.62	49.21	7.146	2.158
SPAC19G12.16C	adg2	0.977	-0.3372	0.010105436	20.7	34.56	13.45	7.65
SPBC8E4.05C	SPBC8E4.05c	0.9477	-0.3371	0.023329119	28.66	47.63	7.356	1.81
SPCC777.02	SPCC777.02	0.9138	-0.3353	0.039148846	30.74	51.04	4.301	1.304
SPBC23E6.02	SPBC23E6.02	0.9466	-0.3292	0.0238335	28.13	46.75	5.404	3.38
SPCC4G3.19	alp16	0.9153	-0.3265	0.038436538	28.08	46.67	4.448	0.9486
SPAC644.08	SPAC644.08	0.9587	-0.3261	0.018317273	31.9	52.93	9.097	2.039
SPAC3G9.11C	SPAC3G9.11c	0.9785	-0.3244	0.00943917	21.3	35.52	13.48	8.098
SPAC664.03	SPAC664.03	0.9171	-0.3244	0.037583307	22.3	37.17	2.269	2.608
SPBC16E9.02C	SPBC16E9.02c	0.9316	-0.3239	0.03077052	31.06	51.54	4.771	2.136
SPBC23G7.11	mag2	0.9192	-0.3177	0.036589984	30.82	51.14	3.895	1.829
SPBC115.03	SPBC115.03	0.9783	-0.3136	0.009527946	23.85	39.68	12.49	8.043
SPBC354.13	rga6	0.9235	-0.3135	0.0345631	32.1	53.24	4.37	1.597
SPBC947.15C	SPBC947.15c	0.9474	-0.3131	0.02346662	26.95	44.78	5.894	2.785
SPAC29A4.11	rga3	0.9513	-0.3059	0.021682503	30.9	51.26	6.471	2.702
SPAC8F11.08C	SPAC8F11.08c	0.9479	-0.303	0.023237477	29.15	48.37	5.248	3.091
SPBC646.08C	SPBC646.08c	0.9547	-0.2996	0.020133077	29.95	49.69	7.23	2.351
SPBC31F10.15C	atp15	0.9464	-0.299	0.023925268	16.61	27.78	4.947	3.167
SPCC330.02	rhp7	0.9248	-0.2922	0.033952179	28.28	46.93	4.496	0.9445

SPBC1348.01	SPBC1348.01	0.9642	-0.2912	0.015832873	31.26	51.83	7.751	4.038
SPCC736.04C	gma12	0.9674	-0.2878	0.014393917	35.2	58.3	10.48	1.407
SPBC3H7.10	elp6	0.9475	-0.2806	0.023420781	16.2	27.08	4.428	3.072
SPBC428.12C	SPBC428.12c	0.9555	-0.2748	0.019769309	32.27	53.45	6.33	2.699
SPBC17G9.10	rpl1102	0.9411	-0.2746	0.026364227	30.67	50.83	4.899	1.902
SPAC1B2.03C	SPAC1B2.03c	0.9352	-0.2738	0.029095502	23.34	38.78	4.442	1.727
SPCC1494.01	SPCC1494.01	0.934	-0.269	0.029653124	29.87	49.51	3.794	2.087
SPBC1289.11	spf38	0.9355	-0.2667	0.028956208	32.12	53.19	4.662	1.252
SPBC16A3.08C	SPBC16A3.08c	0.9697	-0.2632	0.013362604	12.58	21.1	7.876	4.602
SPBC146.02	SPBC146.02	0.9427	-0.2604	0.025626493	33.31	55.15	5.088	1.44
SPAC12B10.13	SPAC12B10.13	0.93	-0.2588	0.031517051	28.75	47.66	3.926	1.459
SPAC19A8.10	rfp1	0.9599	-0.2585	0.017774008	33.2	54.97	7.552	1.344
SPBC1773.05C	tms1	0.9649	-0.2573	0.015517694	33.16	54.9	7.742	2.956
SPBC31F10.05	mug37	0.9588	-0.2571	0.018271975	33.77	55.9	6.895	2.119
SPAC13G7.02C	ssa1	0.9681	-0.2562	0.01407978	29.67	49.16	7.201	4.32
SPACUNK4.13C	SPACUNK4.13c	0.9437	-0.2552	0.025166045	29.79	49.36	5.073	1.443
SPAC24C9.14	otu1	0.9565	-0.2549	0.019315026	32.43	53.69	6.274	2.267
SPBC17F3.01C	rga5	0.9628	-0.2524	0.016463918	30.43	50.4	7.245	2.651
SPAC25B8.09	SPAC25B8.09	0.9391	-0.2382	0.027288159	29.52	48.88	4.654	0.5673
SPAC12B10.01C	SPAC12B10.01c	0.9471	-0.2382	0.023604163	32.8	54.28	5.195	1.128
SPBC20F10.10	psl1	0.9324	-0.2373	0.030397735	25.82	42.8	3.866	1.206
SPAC6G10.12C	ace2	0.9719	-0.2347	0.012378418	12.85	21.5	7.759	4.291
SPBC1539.08	arf6	0.9792	-0.2341	0.009128595	12.63	21.13	10.47	5.814
SPBC4C3.12	Sep-01	0.9816	-0.2303	0.00806545	18.09	30.09	11.7	6.396
SPBC83.11	SPBC83.11	0.9675	-0.227	0.014349026	32.13	53.15	6.555	3.541
SPAC1565.03	SPAC1565.03	0.9763	-0.2257	0.010416711	34.76	57.47	10.58	3.172
SPAC31G5.10	eta2	0.9643	-0.2221	0.015787833	30.98	51.26	6.202	2.878
SPAC56F8.04C	ppt1	0.9753	-0.221	0.010861776	14.06	23.46	8.078	4.754
SPBPB7E8.01	SPBPB7E8.01	0.9622	-0.2198	0.016734647	10.93	18.31	4.078	3.668
SPAC8F11.03	msh3	0.9377	-0.2183	0.027936084	30.66	50.72	3.416	1.677
SPBC21B10.12	rec6	0.9817	-0.2179	0.008021209	36.26	59.92	13.99	2.412
SPBC428.08C	clr4	0.9618	-0.2172	0.016915227	25.08	41.56	5.803	2.511
SPAC4G8.05	ppk14	0.9607	-0.2162	0.017412209	22.54	37.38	5.881	2.133
SPCC965.11C	SPCC965.11c	0.9275	-0.2157	0.032686082	26.33	43.61	2.767	1.523
SPAC6G10.06	SPAC6G10.06	0.9458	-0.213	0.02420069	31.03	51.31	2.606	2.526
SPAP11E10.02C	mam3	0.978	-0.2125	0.009661145	29.62	49	10.72	3.238
SPCC1672.04C	SPCC1672.04c	0.8925	-0.2107	0.049391775	17.53	29.14	1.113	1.309
SPAC3G6.09C	tps2	0.9553	-0.2095	0.019860222	27.88	46.14	4.639	2.194
SPBC25B2.07C	mug164	0.9361	-0.2083	0.028677755	28.67	47.44	1.951	2.159
SPAC688.04C	gst3	0.9312	-0.2079	0.030957033	29.93	49.5	2.609	1.678
SPAC17G8.05	med20	0.9865	-0.2068	0.00590291	23.69	39.25	13.81	8.129
SPBC25H2.11C	spt7	0.3648	-0.2045	0.43794517	0.05911	0.4331	0.3389	0.05147
SPCC622.12C	gdh1	0.9853	-0.2014	0.006431517	20.61	34.18	12.56	7.204
SPAC3F10.16C	SPAC3F10.16c	0.5102	-0.1999	0.292259546	0.07929	0.4586	0.4586	0.07929
SPAC24B11.06C	sty1	0.6399	-0.1981	0.19388789	0.2523	0.7398	0.5389	0.2523
SPCC31H12.03C	SPCC31H12.03c	0.9754	-0.1946	0.010817249	27.36	45.26	7.776	3.769
SPBP23A10.02	pkr1	0.955	-0.193	0.019996628	34.46	56.93	3.69	2.388
SPBP23A10.10	ppk32	0.9701	-0.1907	0.013183495	26.23	43.39	4.144	4.138
SPCC1840.08C	SPCC1840.08c	0.9855	-0.1891	0.006343371	20.02	33.19	11.97	6.856
SPAC1A6.04C	plb1	0.9832	-0.1887	0.00735813	14.99	24.93	10.01	6.08
SPAC19D5.01	pyp2	0.9728	-0.1793	0.011976438	30.61	50.57	6.984	2.652
SPCC970.10C	brl2	0.9828	-0.1782	0.007534852	16.18	26.87	9.325	5.521
SPAC1093.03	SPAC1093.03	0.9675	-0.1767	0.014349026	26.25	43.41	5.392	2.527
SPAC9.06C	SPAC9.06c	0.9628	-0.1762	0.016463918	32.89	54.31	5.262	1.582
SPCC63.08C	atg1	0.9615	-0.1722	0.017050711	25.56	42.26	3.666	2.594
SPBPB10D8.05C	SPBPB10D8.05c	0.9632	-0.1698	0.016283526	31.45	51.94	4.483	2.227
SPAC26F1.10C	pyp1	0.9503	-0.1618	0.022139271	31.93	52.71	3.726	0.8942
SPAC5H10.01	SPAC5H10.01	0.9248	-0.1564	0.033952179	35.32	58.28	1.317	1.354
SPAC11E3.12	SPAC11E3.12	0.9812	-0.156	0.00824246	30.45	50.28	9.439	2.368
SPBC30B4.01C	wsc1	0.9902	-0.153	0.004277078	24.71	40.83	14.21	8.304
SPAC23G3.07C	snf30	0.9747	-0.1457	0.011129034	31.53	52.03	6.051	2.369
SPCC18B5.06	SPCC18B5.06	0.9572	-0.1421	0.01899731	26.39	43.58	3.359	1.491
SPCC576.14	dph5	0.9813	-0.1398	0.008198201	33.12	54.64	8.928	0.8604
SPBC25B2.08	SPBC25B2.08	0.972	-0.1333	0.012333735	30.02	49.52	5.07	1.882
SPCC1020.10	oca2	0.9754	-0.133	0.010817249	33.26	54.86	5.867	2.012
SPBC21B10.03C	SPBC21B10.03c	0.9693	-0.1322	0.013541787	19.26	31.86	4.851	1.326
SPCC24B10.08C	ada2	0.9792	-0.1317	0.009128595	25.24	41.67	5.981	3.177
SPAC2F7.02C	SPAC2F7.02c	0.9563	-0.131	0.019405844	32.53	53.65	2.565	1.678
SPAC688.13	scn1	0.9797	-0.131	0.008906892	27.54	45.46	7.073	2.308

SPAC23G3.02C	sib1	0.9844	-0.1285	0.006828395	33.84	55.79	9.549	2.038
SPAC1F7.09C	SPAC1F7.09c	0.9783	-0.127	0.009527946	32.41	53.45	6.133	2.414
SPAC17C9.12	SPAC17C9.12	0.9801	-0.1264	0.008729611	20.82	34.41	3.647	4.254
SPCC1840.05C	SPCC1840.05c	0.9877	-0.1236	0.005374946	34.74	57.27	11.86	1.974
SPBP16F5.08C	SPBP16F5.08c	0.9809	-0.1201	0.008375265	30.38	50.09	7.338	1.368
SPCC330.14C	rpl2402	0.9908	-0.12	0.004014002	22.35	36.92	11.89	6.854
SPAC30C2.02	mmd1	0.9789	-0.1104	0.009261671	28.52	47.03	4.175	3.094
SPAC17A5.09C	SPAC17A5.09c	0.9807	-0.109	0.008463825	30	49.46	5.296	2.875
SPAC3G6.02	dss1	0.993	-0.1018	0.003050752	22.2	36.63	13.18	7.668
SPAC16A10.07C	taz1	0.9773	-0.09911	0.009972101	32.44	53.45	4.731	1.616
SPAC823.10C	SPAC823.10c	0.9871	-0.09534	0.005638848	29.55	48.69	8.756	1.358
SPCC70.03C	SPCC70.03c	0.9827	-0.09166	0.007579044	29.75	49.01	3.673	3.362
SPAC869.05C	SPAC869.05c	0.9865	-0.09054	0.00590291	30.68	50.54	6.581	3.213
SPBPB2B2.07C	SPBPB2B2.07c	0.9931	-0.08279	0.003007018	25.29	41.68	11.59	5.885
SPAC1F7.07C	fip1	0.9852	-0.08203	0.006475597	30.52	50.26	6.249	1.68
SPBC1773.02C	SPBC1773.02c	0.9839	-0.07205	0.007049039	32.99	54.31	5.067	1.304
SPAC14C4.09	agn1	0.9899	-0.06985	0.004408676	22.08	36.38	5.053	4.302
SPCPJ732.02C	SPCPJ732.02c	0.9884	-0.06935	0.005067263	30.48	50.18	5.409	3.178
SPAC22E12.19	snt1	0.9883	-0.06835	0.005111205	30.02	49.42	6.416	2.085
SPAC17G8.08C	SPAC17G8.08c	0.9861	-0.06835	0.006079041	33.54	55.21	5.667	1.238
SPAC16E8.12C	SPAC16E8.12c	0.9869	-0.0673	0.005726851	31.61	52.03	5.324	2.194
SPBC2F12.09C	atf21	0.9954	-0.06617	0.002002364	20.34	33.52	13.29	7.53
SPAC732.02C	SPAC732.02c	0.9916	-0.06074	0.003663482	36.14	59.46	8.516	1.46
SPBC12C2.12C	glo1	0.9864	-0.05747	0.005946936	27.91	45.94	2.997	2.641
SPAC13G7.04C	mac1	0.9914	-0.05729	0.003751085	32.1	52.81	6.679	3.046
SPAC521.02	SPAC521.02	0.9917	-0.05619	0.003619687	23.86	39.29	7.205	2.732
SPCC1235.08C	pdh1	0.9933	-0.05204	0.002919565	26.84	44.17	8.072	3.319
SPCC1682.08C	SPCC1682.08c	0.9963	-0.05129	0.00160987	21.73	35.78	12.51	7.426
SPAC13G6.06C	gcv2	0.9862	-0.04678	0.006035002	32.08	52.77	3.457	1.489
SPAC4F10.17	SPAC4F10.17	0.9894	-0.04592	0.004628094	31.67	52.09	4.845	1.384
SPAC25B8.10	SPAC25B8.10	0.9942	-0.042	0.002526241	31.1	51.15	7.494	3.029
SPBC13G1.02	SPBC13G1.02	0.9958	-0.0411	0.001827878	30.91	50.84	10.7	3.309
SPBC2A9.04C	SPBC2A9.04c	0.9946	-0.03892	0.002351545	24.17	39.76	7.317	3.232
SPAPB8E5.02C	rpn502	0.9914	-0.03578	0.003751085	29.76	48.95	3.51	2.348
SPCC1020.13C	SPCC1020.13c	0.9947	-0.02353	0.002307882	25.46	41.86	4.411	2.049
SPACUNK4.19	mug153	0.995	-0.02344	0.002176919	30.54	50.21	4.999	1.8
SPCC132.03	SPCC132.03	0.9974	-0.02103	0.001130636	33.24	54.64	9.53	1.984
SPBC660.10	SPBC660.10	0.996	-0.02097	0.001740662	25.19	41.42	2.846	3.545
SPAC3C7.13C	SPAC3C7.13c	0.9986	-0.01786	0.000608438	23.68	38.92	12.14	6.617
SPAC821.13C	SPAC821.13c	0.9991	-0.01475	0.000391041	23.48	38.59	14.31	8.08
SPAC926.09C	fas1	0.9983	-0.01403	0.000738929	34.63	56.9	9.83	1.662
SPAC3C7.14C	obr1	0.9984	-0.01264	0.000695428	34.75	57.1	9.5	1.562
SPAC2C4.05	SPAC2C4.05	0.9982	-0.01097	0.000782434	33.64	55.28	6.951	1.269
SPAC1002.12C	SPAC1002.12c	0.9994	-0.008096	0.000260655	38.36	63.02	17.05	3.214
SPAC9E9.03	leu2	0.9993	-0.004701	0.000304113	27.75	45.59	6.459	3.475
SPCC1442.04C	SPCC1442.04c	0.9997	-0.003215	0.000130308	20.46	33.61	12.32	5.941
SPAC694.02	SPAC694.02	0.9992	-0.002479	0.000347575	16.76	27.53	2.463	1.721
SPCC757.09C	rnc1	1	-0.000244	0	24.38	40.05	8.086	2.961
SPBC25D12.02C	dnt1	1	0	0	0	0	0	0
SPAC12G12.13C	cid14	1	0	0	0	0	0	0
SPBC21C3.16C	spt4	1	0	0	0	0	0	0
SPBC685.07C	rpl2701	1	0	0	0	0	0	0
SPCC663.01C	ekc1	1	0	0	0	0	0	0
SPBC887.18C	hfi1	1	0	0	0	0	0	0
SPAC22G7.03	SPAC22G7.03	1	0.000134	0	30.3	49.78	6.334	2.387
SPBC1709.16C	SPBC1709.16c	0.9987	0.01377	0.00056495	27.15	44.57	10.51	4.735
SPAC328.06	ubp2	0.9958	0.01485	0.001827878	30.98	50.86	1.738	2.451
SPCC285.09C	cgs2	0.9964	0.01555	0.001566281	26.42	43.37	4.753	1.429
SPBC16A3.14	SPBC16A3.14	0.9979	0.01831	0.000912977	33.37	54.79	10.2	2.186
SPAC3G6.05	SPAC3G6.05	0.9975	0.02152	0.001087096	25.53	41.9	8.556	3.842
SPAC1952.10C	SPAC1952.10c	0.9968	0.02452	0.001391971	30.44	49.96	8.706	2.437
SPAC18G6.01C	SPAC18G6.01c	0.9967	0.02535	0.001435542	25.45	41.76	7.772	3.348
SPCC1183.06	ung1	0.9949	0.02641	0.002220569	32.19	52.84	6.152	0.8722
SPBC30D10.05C	SPBC30D10.05(	0.9973	0.02772	0.001174181	34.75	57.04	11.66	3.013
SPAC3C7.06C	pit1	0.9909	0.02816	0.003970172	28.52	46.8	3.677	0.4864
SPBC660.11	tcg1	0.9753	0.02977	0.010861776	0.7268	1.145	1.145	0.6049
SPCC825.05C	SPCC825.05c	0.9958	0.03218	0.001827878	14.87	24.38	7.112	3.903
SPBC119.04	mei3	0.9951	0.03339	0.002133274	28.08	46.07	7.261	2.618
SPBC1711.02	matmc_1	0.9924	0.03428	0.003313244	31.28	51.33	4.814	1.801

SPAC32A11.01	mug8	0.9974	0.03869	0.001130636	24.5	40.19	13.23	8.263
SPBC530.15C	SPBC530.15c	0.993	0.04001	0.003050752	30.73	50.41	6.124	2.254
SPAC4G9.16C	rpl901	0.8938	0.04088	0.04875965	0.2711	0.3782	0.2041	0.2711
SPBC21D10.07	cmc1	0.9929	0.04626	0.003094489	33.93	55.65	7.45	1.866
SPAC20H4.02	dsc3	0.9939	0.05064	0.002657309	7.753	12.65	7.399	4.517
SPCC1906.02C	SPCC1906.02c	0.9907	0.05355	0.004057837	30.69	50.33	5.846	2.555
SPAC664.10	klp2	0.9939	0.0553	0.002657309	26.3	43.11	8.521	4.554
SPBPJ4664.01	dps1	0.9942	0.06178	0.002526241	15	24.54	8.312	6.352
SPBC3D6.10	apn2	0.9864	0.06414	0.005946936	23.29	38.16	4.755	2.143
SPAC4D7.10C	spt20	0.3506	0.06441	0.455188088	0.06441	0	0	0.06441
SPCC550.03C	SPCC550.03c	0.9917	0.06529	0.003619687	23.17	37.94	7.843	3.586
SPAC1556.03	azr1	0.987	0.07047	0.005682847	32.59	53.42	5.706	2.195
SPAC139.02C	oac1	0.9812	0.07531	0.00824246	30.35	49.74	3.882	1.935
SPAC3H8.05C	mms1	0.9928	0.0785	0.003138232	16.64	27.2	9.649	5.942
SPAC328.03	tps1	0.9812	0.07851	0.00824246	5.571	9.023	3.515	2.368
SPBC30B4.03C	adn1	0.9759	0.08076	0.010594682	30.67	50.25	3.316	1.573
SPBC2G5.03	ctu1	0.9842	0.08269	0.006916639	27.87	45.64	1.779	3.741
SPCC1223.10C	eaf1	0.9907	0.08932	0.004057837	31.49	51.57	10.95	2.707
SPCC18B5.01C	bfr1	0.9924	0.08944	0.003313244	28.75	47.08	10.46	6.365
SPBPB2B2.05	SPBPB2B2.05	0.9815	0.09136	0.008109696	31.73	51.97	5.174	2.044
SPAC9E9.15	SPAC9E9.15	0.9913	0.09237	0.003794894	35.47	58.11	12.5	1.987
SPAC6F6.17	rif1	0.9848	0.09255	0.00665196	27.79	45.49	6.41	2.499
SPCC1223.11	ptc2	0.9865	0.09473	0.00590291	22.7	37.13	4.349	4.597
SPBC27B12.03C	erg32	0.9744	0.09646	0.011262725	34.51	56.52	4.098	1.376
SPCC622.01C	SPCC622.01c	0.9789	0.1012	0.009261671	30.65	50.19	4.633	2.342
SPBP4H10.09	rsv1	0.9929	0.1035	0.003094489	22.92	37.48	13.3	7.743
SPAC3H8.08C	SPAC3H8.08c	0.9762	0.1042	0.010461197	30.4	49.76	2.989	2.78
SPBC2D10.04	SPBC2D10.04	0.9804	0.1062	0.008596697	32.55	53.29	5.278	2.62
SPBC4F6.04	rpl2502	0.9918	0.107	0.003575896	22.47	36.73	12.83	6.226
SPAP27G11.16	SPAP27G11.16	0.9889	0.1138	0.004847623	35.41	57.97	11.82	2.522
SPAC824.07	SPAC824.07	0.9867	0.1202	0.005814872	33.59	54.98	10.42	2.402
SPBC28F2.02	mep33	0.9711	0.1247	0.012736046	36.31	59.43	3.991	2.231
SPBC19G7.09	ulp1	0.9775	0.1249	0.009883234	32.03	52.4	6.488	1.249
SPAC11D3.01C	SPAC11D3.01c	0.9834	0.1277	0.007269796	33.63	55.02	8.895	1.929
SPAC23A1.02C	SPAC23A1.02c	0.9898	0.1289	0.004452551	20.65	33.71	11.08	6.996
SPAC23C4.05C	SPAC23C4.05c	0.979	0.1345	0.009217308	27.41	44.8	7.012	2.274
SPBC18H10.19	atg14	0.9632	0.1354	0.016283526	25.96	42.42	2.254	2.425
SPAC29B12.11C	SPAC29B12.11c	0.9831	0.1369	0.007402304	32.98	53.95	8.584	3.242
SPAC3H5.08C	SPAC3H5.08c	0.9572	0.1405	0.01899731	30.37	49.65	3.905	0.4843
SPBC1718.03	ker1	0.9717	0.142	0.012467797	7.621	12.28	4.341	2.791
SPAC1142.01	SPAC1142.01	0.9668	0.1465	0.014663358	34.89	57.06	4.534	1.912
SPBC1709.10C	atx1	0.9794	0.149	0.0090399	30.12	49.22	7.314	3.265
SPCC18.03	SPCC18.03	0.9748	0.1492	0.011084479	29.91	48.89	6.149	2.492
SPAC1783.05	hrp1	0.9694	0.1505	0.013496985	28.67	46.84	5.498	1.563
SPCPB16A4.05C	SPCPB16A4.05	0.9753	0.1514	0.010861776	27.76	45.35	5.913	3.002
SPAC6B12.03C	SPAC6B12.03c	0.9831	0.1547	0.007402304	24.22	39.52	8.484	4.758
SPBC11C11.11C	SPBC11C11.11c	0.981	0.1579	0.008330993	28.63	46.76	8.536	3.627
SPBC713.11C	pmp3	0.9752	0.1599	0.010906307	30.06	49.11	5.997	3.31
SPAC29E6.09	SPAC29E6.09	0.9623	0.1623	0.016689514	31.43	51.35	4.573	1.713
SPCC1450.11C	cek1	0.977	0.1626	0.010105436	27.34	44.64	6.818	3.466
SPAC1002.17C	urg2	0.9561	0.1641	0.019496682	24.89	40.62	2.187	2.487
SPAC4A8.07C	SPAC4A8.07c	0.9531	0.1718	0.02086153	31.99	52.26	4.23	0.9151
SPAC27D7.10C	EMPTY	0.971	0.1773	0.01278077	31.64	51.68	5.864	3.022
SPBC18E5.11C	edc3	0.9778	0.1781	0.009749967	34.12	55.76	8.747	2.901
SPAPJ760.02C	abp1	0.933	0.1803	0.030118356	34.87	56.99	1.871	1.698
SPAC30D11.10	rad22	0.8745	0.1831	0.058240186	1.208	1.683	1.415	0.6982
SPAC637.07	moe1	0.9778	0.1877	0.009749967	12.63	20.43	7.834	4.366
SPAC19E9.01C	nup40	0.9626	0.1886	0.016554143	29.54	48.22	5.446	1.88
SPAC4F8.03	sdo1	0.6988	0.1937	0.155647104	0.6397	0.7327	0.3574	0.4352
SPAC1610.03C	crp79	0.9655	0.1937	0.015247722	32.47	53.02	5.561	2.638
SPCC1919.09	tif6	0.9535	0.1947	0.020679303	30.66	50.04	4.119	1.977
SPBC725.02	mpr1	0.9834	0.1959	0.007269796	18.17	29.53	10.57	6.323
SPAC7D4.08	SPAC7D4.08	0.9667	0.2018	0.014708281	27.08	44.15	6.718	2.029
SPCC330.12C	sdh3	0.9718	0.2059	0.012423105	43.02	70.32	5.179	4.571
SPCC1235.05C	fft2	0.94	0.2096	0.026872146	30.52	49.79	3.277	1.776
SPBC31F10.02	SPBC31F10.02	0.9421	0.2115	0.025902996	30.02	48.96	2.322	2.38
SPCC1322.16	phb2	0.9649	0.2143	0.015517694	29.17	47.57	7.122	1.337
SPAC17G6.17	pof8	0.9821	0.2144	0.007844289	19.21	31.19	9.955	6.883
SPAC9G1.03C	rpl3001	0.9652	0.2173	0.015382687	12.13	19.57	5.452	3.439

SPAC2C4.17C	SPAC2C4.17c	0.9763	0.2232	0.010416711	35.03	57.18	11.22	1.405
SPBC1539.10	nop16	0.9837	0.2268	0.007137329	18.08	29.32	12.68	7.304
SPCC622.19	jmj4	0.9541	0.2352	0.020406104	28.6	46.59	3.178	3.365
SPCC550.07	SPCC550.07	0.9625	0.2391	0.016599262	22.64	36.8	6.846	2.453
SPAC12G12.16C	SPAC12G12.16c	0.9697	0.2403	0.013362604	33.44	54.53	9.241	1.78
SPAC1952.12C	csn71	0.9653	0.2406	0.015337694	32.18	52.47	6.714	3.369
SPBC1289.10C	adn2	0.9757	0.2439	0.010683695	25.43	41.36	10.12	4.544
SPAC27E2.09	mak2	0.9736	0.2451	0.011619435	28.63	46.62	7.071	5.611
SPAC8E11.06	SPAC8E11.06	0.9711	0.2464	0.012736046	33.31	54.31	9.806	2.196
SPBP4H10.03	oxa102	0.9727	0.2512	0.012021084	14.6	23.58	8.156	5.011
SPBC405.02C	SPBC405.02c	0.9438	0.2513	0.025120027	32.74	53.36	5.174	1.092
SPAC1F3.10C	Oct-01	0.9636	0.2527	0.016103209	32.5	52.96	7.484	2.607
SPBC1685.04	SPBC1685.04	0.9598	0.2533	0.017819254	33.75	55.01	6.527	2.671
SPAC3G6.04	rnp24	0.9514	0.2544	0.021636853	32.32	52.67	5.69	1.922
SPCP1E11.04C	pal1	0.9704	0.2546	0.013049212	16.54	26.75	7.278	4.869
SPBC4B4.07C	usp102	0.9288	0.2565	0.032077793	16.73	27.05	3.754	1.506
SPAC5D6.04	SPAC5D6.04	0.9585	0.2626	0.018407883	31.3	50.99	6.582	2.651
SPCC645.02	gep4	0.97	0.2646	0.013228266	35.99	58.68	10.15	2.32
SPAC2F3.11	SPAC2F3.11	0.9631	0.2655	0.016328617	33.97	55.36	8.182	2.082
SPCC1795.01C	mad3	0.9682	0.2704	0.014034922	33.19	54.08	10.03	1.574
SPAC6G9.10C	sen1	0.9655	0.2709	0.015247722	28.31	46.06	8.65	2.713
SPCC777.03C	SPCC777.03c	0.9443	0.2756	0.02489001	33.35	54.33	5.269	1.949
SPAP7G5.06	per1	0.9168	0.2759	0.037725395	30.52	49.68	2.894	1.818
SPBC24C6.11	cwf14	0.9706	0.2774	0.012959713	14.89	24	8.423	5.082
SPAC26F1.01	sec74	0.9364	0.2791	0.028538595	31.58	51.41	4.313	2.077
SPAC1527.02	sft2	0.9613	0.2843	0.017141058	31.19	50.76	7.869	2.839
SPAC2G11.04	SPAC2G11.04	0.9304	0.2855	0.031330298	28.73	46.72	3.284	2.411
SPAC6G9.12	cfr1	0.9343	0.2909	0.029513651	29.67	48.26	4.463	1.997
SPCC594.06C	SPCC594.06c	0.9423	0.2963	0.025810809	28.92	47.01	6.106	0.7374
SPBC6B1.06C	ubp14	0.9463	0.2965	0.02397116	26.61	43.23	5.923	2.118
SPCC16C4.17	mug123	0.9749	0.2982	0.01103993	21.64	35.05	10.58	6.418
SPBC1861.03	mak10	0.9425	0.2982	0.025718641	27.47	44.64	5.499	2.068
SPAC19G12.04	SPAC19G12.04	0.9287	0.2987	0.032124554	24.97	40.53	2.444	2.788
SPAC4A8.04	isp6	0.9234	0.2988	0.03461013	26.42	42.91	2.683	2.468
SPAC25H1.05	meu29	0.942	0.3034	0.025949097	8.047	12.72	4.364	2.989
SPAC8C9.11	SPAC8C9.11	0.9378	0.3088	0.027889772	31.2	50.74	5.725	1.254
SPAC3F10.13	ucp6	0.9353	0.31	0.029049066	32.17	52.33	4.825	2.169
SPAC2F3.08	sut1	0.9567	0.3142	0.019224226	33.55	54.59	7.98	2.535
SPAC22A12.16	SPAC22A12.16	0.947	0.3157	0.023650021	31.59	51.37	5.47	3.109
SPAC1071.03C	SPAC1071.03c	0.9676	0.3186	0.01430414	35.21	57.31	11.46	2.259
SPCC1494.05C	ubp12	0.8678	0.3259	0.061580354	18.41	29.71	2.335	1.222
SPCC188.09C	SPCC188.09c	0.9595	0.3265	0.017955021	32.18	52.33	9.445	1.719
SPAC5H10.05C	SPAC5H10.05c	0.9252	0.3295	0.033764376	27.89	45.27	4.492	1.938
SPCC1442.02	SPCC1442.02	0.9505	0.3301	0.022047879	28.92	46.96	6.75	2.989
SPAC57A7.04C	pabp	0.9269	0.3332	0.032967118	31.65	51.44	4.982	1.626
SPCC23B6.01C	SPCC23B6.01c	0.7933	0.3358	0.100562546	16.73	26.92	1.439	0.8572
SPBC28E12.02	SPBC28E12.02	0.944	0.3377	0.025028006	28.27	45.89	6.672	2.041
SPBC32F12.11	tdh1	0.9258	0.3414	0.033482824	20.5	33.11	3.145	2.923
SPBC21B10.09	SPBC21B10.09	0.9762	0.3452	0.010461197	23.17	37.48	13.07	7.759
SPAC17A2.02C	SPAC17A2.02c	0.9627	0.3472	0.016509028	25.78	41.78	8.751	4.727
SPBC30B4.06C	SPBC30B4.06c	0.8939	0.3517	0.048711063	27.12	43.98	3.488	1.335
SPCC584.16C	SPCC584.16c	0.9738	0.3609	0.01153023	24.17	39.11	10.97	8.118
SPCC1322.03	SPCC1322.03	0.9649	0.3611	0.015517694	22.28	36.01	10.24	4.772
SPAC5H10.09C	SPAC5H10.09c	0.9183	0.3616	0.037015416	29.19	47.36	4.386	2.06
SPAC11E3.03	pcs1	0.95	0.362	0.022276395	20.57	33.19	6.328	3.974
SPBC11C11.09C	rpl502	0.3229	0.3623	0.490931955	0.3976	0.05804	0.05804	0.3394
SPBC609.05	pob3	0.9337	0.3646	0.029792641	26.5	42.93	5.21	2.754
SPBP8B7.31	SPBP8B7.31	0.9456	0.3652	0.024292536	26.23	42.49	7.061	2.742
SPAC18B11.07C	rhp6	0.9023	0.3683	0.044649043	30.08	48.81	4.073	1.393
SPAC3H1.08C	SPAC3H1.08c	0.9	0.3727	0.045757491	30.58	49.62	3.621	1.788
SPAC20G8.08C	fft1	0.9632	0.3736	0.016283526	16.51	26.51	8.885	5.573
SPAC17H9.12C	SPAC17H9.12c	0.9357	0.376	0.028863371	29.46	47.78	6.407	2.063
SPAC17G6.06	rps2401	0.9614	0.3773	0.017095882	25.07	40.56	8.809	5.219
SPBC83.01	ucp8	0.9548	0.3807	0.02008759	34.1	55.38	9.723	2.133
SPBPB10D8.06C	SPBPB10D8.06	0.9192	0.3871	0.036589984	33.61	54.57	3.919	2.771
SPBC902.02C	ctf18	0.9143	0.3898	0.03891128	28.77	46.62	4.751	1.879
SPBC4F6.10	vps901	0.9579	0.3898	0.018679827	14.99	23.99	8.086	5.094
SPCC417.05C	chr2	0.9739	0.3917	0.011485634	20.88	33.66	13.92	7.726
SPAC15F9.01C	SPAC15F9.01c	0.9372	0.3975	0.02816772	30.31	49.13	6.478	2.76

SPAC139.06	hat1	0.907	0.3996	0.042392713	32.45	52.64	4.707	1.505
SPAC19B12.11C	SPAC19B12.11c	0.8948	0.4055	0.048274025	24.75	39.99	3.169	2.214
SPCC61.05	SPCC61.05	0.877	0.4074	0.057000407	19.53	31.41	1.609	2.271
SPBC1D7.04	mlo3	0.9522	0.4095	0.021271823	13.76	21.93	7.563	4.669
SPAC4G8.04	SPAC4G8.04	0.9698	0.4119	0.01331782	21.63	34.85	12.15	7.381
SPAC644.11C	pkp1	0.8281	0.4164	0.081917215	25.58	41.34	1.62	1.533
SPAC11D3.15	SPAC11D3.15	0.9446	0.4173	0.024752059	36.32	58.97	6.647	4.1
SPBC651.11C	apm3	0.8529	0.4175	0.069101886	29.08	47.08	3.237	0.7406
SPCC830.07C	psi1	0.9395	0.4213	0.027103216	30.66	49.67	7.657	2.416
SPAC1B9.02C	sck1	0.9165	0.422	0.037867531	27.62	44.67	5.336	2.024
SPBC29B5.02C	isp4	0.8678	0.4285	0.061580354	30.07	48.69	3.785	0.637
SPAC4F10.07C	atg13	0.9337	0.4307	0.029792641	26.6	42.99	6.858	2.615
SPAC1783.02C	vps66	0.8539	0.4319	0.068592986	26.74	43.22	2.7	1.532
SPCC11E10.09C	SPCC11E10.09c	0.913	0.4333	0.039529222	29.27	47.36	4.542	2.607
SPCC1672.03C	SPCC1672.03c	0.9312	0.4376	0.030957033	30.59	49.53	7.206	1.867
SPCC553.04	cyp9	0.9529	0.4401	0.020952673	17.62	28.22	8.662	4.818
SPACUNK4.11C	SPACUNK4.11c	0.9009	0.4421	0.045323413	31.33	50.73	4.947	1.465
SPCC1795.12C	SPCC1795.12c	0.8943	0.4423	0.048516769	32.92	53.35	4.581	1.457
SPAC17G8.10C	dma1	0.8713	0.443	0.059832286	31.72	51.38	3.66	1.334
SPBC337.04	ppk27	0.8557	0.4438	0.067678468	29.1	47.07	1.906	2.001
SPBC17G9.07	rps2402	0.7811	0.4486	0.107293362	30.02	48.57	1.741	1.121
SPBC16C6.08C	qcr6	0.9036	0.4544	0.044023778	31.51	51.02	5.405	1.227
SPAC12B10.15C	SPAC12B10.15c	0.9212	0.4555	0.035646071	31.17	50.45	5.833	2.596
SPAC11D3.06	SPAC11D3.06	0.8865	0.4562	0.05232126	31.74	51.39	3.466	2.219
SPAC17G6.05C	SPAC17G6.05c	0.8145	0.4584	0.089108911	27.56	44.52	2.319	1.224
SPBC24C6.08C	bhd1	0.9241	0.4587	0.03428103	33.58	54.4	6.512	2.264
SPAC20H4.10	ufd2	0.8969	0.4593	0.047255976	35.32	57.26	4.883	1.547
SPCC18.09C	SPCC18.09c	0.8771	0.461	0.056950889	30.01	48.54	3.798	1.655
SPAC10F6.07C	mug94	0.9237	0.462	0.034469056	30.28	48.97	6.01	2.814
SPBC1773.08C	omh4	0.8912	0.4637	0.050024822	30.77	49.78	4.192	2.001
SPAC2C4.14C	ppk11	0.9284	0.4644	0.032264868	33.92	54.96	6.955	2.484
SPBC2F12.04	rpl1701	0.9337	0.4669	0.029792641	32.06	51.9	6.399	3.715
SPAC212.01C	SPAC212.01c	0.9134	0.4707	0.039338993	31.87	51.58	5.35	2.558
SPAC17A2.01	bsu1	0.9302	0.4719	0.031423665	30.03	48.56	7.159	2.695
SPAC1296.01C	SPAC1296.01c	0.7508	0.4766	0.124475736	34.32	55.59	0.9707	1.276
SPAC26A3.11	SPAC26A3.11	0.8858	0.4814	0.052664324	25.75	41.5	2.165	2.868
SPAPB17E12.13	rpl1802	0.9486	0.4819	0.02291688	27.43	44.27	7.676	5.434
SPBC216.05	rad3	0.8742	0.4875	0.058389198	27.17	43.84	3.214	2.207
SPBP8B7.11	nxt3	0.8519	0.4897	0.069611382	28.18	45.48	2.368	2.043
SPAPB1A10.14	SPAPB1A10.14	0.9588	0.4903	0.018271975	15.96	25.41	11.29	5.951
SPBC1105.10	rav1	0.391	0.4904	0.407823243	0.4904	1.96E-17	1.96E-17	0.4904
SPAC1851.02	slc1	0.9616	0.495	0.017005545	35.55	57.58	15.23	2.325
SPAC9.13C	cwf16	0.9669	0.4962	0.01461844	23.45	37.7	13.61	7.938
SPBP16F5.07	apm1	0.9142	0.4991	0.038958783	24.2	38.93	3.038	3.958
SPCC16C4.10	SPCC16C4.10	0.8291	0.4992	0.081393085	28.57	46.12	2.829	1.384
SPCC31H12.06	mug111	0.8684	0.5005	0.061280185	29.23	47.18	4.063	1.445
SPAC977.16C	dak2	0.9173	0.5012	0.037488606	31.09	50.25	6.29	2.543
SPAC1F5.08C	yam8	0.9366	0.5019	0.028445847	24.44	39.32	7.935	3.617
SPCC1281.08	wtf11	0.9478	0.5034	0.023283296	28.83	46.52	9.921	4.167
SPAC22F3.07C	atp20	0.9279	0.5046	0.032498825	28.57	46.1	6.926	3.265
SPCC16C4.06C	SPCC16C4.06c	0.8922	0.5059	0.049537781	29.66	47.89	4.913	1.92
SPBC1921.06C	pvg3	0.9313	0.5068	0.030910397	21.68	34.78	6.703	3.888
SPCC61.02	spt3	0.9343	0.5075	0.029513651	35.39	57.3	8.256	2.983
SPBC14C8.05C	meu17	0.8799	0.5124	0.055566682	27.64	44.56	3.705	2.339
SPBC83.02C	rpl4302	0.9658	0.5138	0.015112799	15.26	24.22	13	8.335
SPAC20G8.07C	erg2	0.9406	0.5139	0.026595026	35.83	58.01	10.15	1.764
SPAPB1E7.08C	SPAPB1E7.08c	0.9025	0.5155	0.044552789	30.86	49.85	5.328	2.376
SPBC1347.12	arp1	0.9284	0.5164	0.032264868	34.13	55.22	8.491	1.348
SPACUNK4.09	SPACUNK4.09	0.942	0.5189	0.025949097	35.25	57.05	10.06	2.766
SPAC19D5.11C	ctf8	0.9017	0.5196	0.04493793	27.99	45.13	5.677	1.995
SPBP4H10.04	ppb1	0.8541	0.5262	0.068491278	30.77	49.68	4.198	0.732
SPBC9B6.07	nop52	0.943	0.5287	0.025488307	9.991	15.54	7.731	5.299
SPBC1539.04	tts1	0.8969	0.5299	0.047255976	28.94	46.67	5.464	2.007
SPBC3B9.15C	scp1	0.9284	0.5312	0.032264868	33.26	53.76	8.021	2.758
SPAC1039.08	SPAC1039.08	0.9369	0.5365	0.028306761	12.88	20.27	7.305	4.745
SPBC11G11.03	mrt4	0.8995	0.5372	0.045998832	29.56	47.67	5.651	2.128
SPAC2F7.07C	cph2	0.9339	0.5379	0.029699625	12.56	19.75	7.412	4.28
SPAC6F6.06C	rax2	0.9231	0.5394	0.034751249	26.96	43.4	4.662	4.496
SPAC5H10.04	SPAC5H10.04	0.9475	0.54	0.023420781	27.85	44.86	8.784	5.773

SPAC3H8.10	spo20	0.901	0.5433	0.045275209	33.78	54.59	6.092	1.794
SPACUNK4.15	SPACUNK4.15	0.8462	0.5474	0.072526979	33.01	53.33	2.924	2.032
SPAPB21F2.03	SPAPB21F2.03	0.9615	0.5498	0.017050711	22.99	36.87	12.49	7.862
SPBC14F5.11C	mug186	0.9407	0.5524	0.026548856	37.31	60.37	10.77	2.283
SPAC29B12.03	spd1	0.8937	0.5542	0.048808242	30.81	49.69	5.965	1.382
SPAC6G9.14	SPAC6G9.14	0.9209	0.5596	0.035787527	22.71	36.39	5.494	4.233
SPCC613.06	rpl902	0.9353	0.5609	0.029049066	10.95	17.06	7.474	4.818
SPBC1773.14	arg7	0.9112	0.5657	0.040386289	28.43	45.76	7.173	1.926
SPCC4G3.04C	coq5	0.8998	0.566	0.045854011	7.872	12	4.63	3.254
SPBC2D10.19C	SPBC2D10.19c	0.9438	0.5675	0.025120027	27.73	44.61	9.51	5.105
SPBPB2B2.13	gal1	0.9503	0.5721	0.022139271	19.8	31.59	11.47	5.316
SPAC11D3.05	mfs2	0.8959	0.5724	0.047740463	29.01	46.71	5.734	2.275
SPBC3D6.05	ptp4	0.9368	0.5731	0.028353118	23.1	37	7.955	4.967
SPAC1805.11C	rps2602	0.8939	0.5734	0.048711063	32.66	52.7	4.208	3.216
SPAC22H10.02	SPAC22H10.02	0.7674	0.5753	0.114978205	29.09	46.83	2.144	1.323
SPBC106.12C	SPBC106.12c	0.9425	0.5754	0.025718641	27.54	44.29	10.79	3.759
SPCC70.02C	SPCC70.02c	0.9102	0.5778	0.040863169	30.27	48.78	6.299	3.054
SPBC23G7.07C	SPBC23G7.07c	0.9042	0.5791	0.043735497	25.38	40.73	6.399	2.395
SPAC4G9.10	arg3	0.9281	0.5793	0.032405227	32.57	52.54	8.584	3.156
SPAC13G6.10C	asl1	0.9196	0.5802	0.036401037	11.83	18.48	6.239	3.991
SPAC19A8.01C	sec73	0.8311	0.582	0.080346718	25.2	40.44	3.518	1.47
SPAPB8E5.06C	rpl302	0.9143	0.5874	0.03891128	27.47	44.16	6.735	3.235
SPAC4F8.10C	stg1	0.9333	0.5878	0.029978734	25.05	40.18	8.049	4.624
SPBC19G7.04	SPBC19G7.04	0.858	0.59	0.066512712	35.59	57.48	3.819	2.253
SPAC11E3.05	SPAC11E3.05	0.8416	0.5903	0.074894273	19.87	31.67	2.364	2.404
SPAC688.03C	SPAC688.03c	0.8574	0.5915	0.066816521	29.54	47.55	4.58	1.359
SPAC25H1.06	pcf3	0.877	0.5918	0.057000407	34.9	56.35	4.58	2.37
SPAC4F10.08	mug126	0.7023	0.5959	0.153477332	31.61	50.94	2.128	0.6794
SPBC609.02	ptn1	0.8821	0.5959	0.054482178	32.56	52.5	5.749	1.388
SPCC965.05C	thp1	0.9119	0.5963	0.040052784	28.83	46.38	7.373	2.427
SPBC28F2.05C	SPBC28F2.05c	0.8764	0.597	0.057297631	28.73	46.21	5.577	1.143
SPAC1565.01	SPAC1565.01	0.8866	0.5981	0.052272273	33.4	53.88	5.477	2.202
SPAC17A5.04C	mde10	0.9188	0.5988	0.036779013	21.46	34.26	6.032	4.263
SPAC167.01	ire1	0.8461	0.6028	0.072578305	32.21	51.91	4.421	1.105
SPBC2D10.06	rep1	0.883	0.6061	0.054039296	26.56	42.62	5.029	2.481
SPAC10F6.06	vip1	0.8912	0.6076	0.050024822	31.5	50.74	6.073	2.004
SPAC11D3.13	SPAC11D3.13	0.9392	0.6116	0.027241916	22.5	35.96	8.457	5.706
SPAC22G7.08	ppk8	0.9206	0.6143	0.035929029	28.7	46.14	8.465	2.735
SPAC6G9.01C	SPAC6G9.01c	0.8871	0.6179	0.052027421	25.23	40.43	2.328	3.819
SPAC29B12.12	SPAC29B12.12	0.8724	0.6217	0.059284343	33.81	54.51	5.59	1.231
SPCC132.02	hst2	0.8445	0.6237	0.073400346	32.44	52.26	4.17	1.641
SPAC12B10.10	SPAC12B10.10	0.8737	0.6278	0.058637664	30.28	48.71	4.977	2.242
SPAC3H8.03	img2	0.8764	0.628	0.057297631	27.93	44.84	3.729	3.116
SPBC21B10.06C	inp2	0.8762	0.6281	0.057396751	21.67	34.57	5.67	1.572
SPCC1020.05	SPCC1020.05	0.9076	0.6307	0.042105513	31.26	50.31	6.072	3.668
SPBC1861.05	SPBC1861.05	0.8813	0.6308	0.05487623	7.109	10.64	4.322	3.073
SPAC5D6.02C	mug165	0.7563	0.6314	0.1213059	23.42	37.43	1.779	1.599
SPCC320.08	SPCC320.08	0.8953	0.6326	0.048031416	28.73	46.16	6.669	2.031
SPBC16G5.11C	bag101	0.8971	0.6348	0.047159143	29.78	47.88	6.841	2.028
SPBC3H7.09	erf2	0.948	0.6408	0.023191663	20.07	31.92	11	6.64
SPAC29E6.07	SPAC29E6.07	0.8427	0.6426	0.074327006	30.27	48.66	4.734	0.8651
SPCC364.01	cif1	0.9074	0.6434	0.042201225	30.73	49.43	7.121	3.007
SPBC15C4.05	SPBC15C4.05	0.918	0.6436	0.037157319	33	53.14	8.936	2.205
SPBC428.15	SPBC428.15	0.8782	0.6455	0.056406567	31.28	50.32	5.203	2.489
SPBC651.03C	gyp10	0.9512	0.6462	0.021728158	27.94	44.84	12.02	6.998
SPBC1271.03C	SPBC1271.03c	0.8917	0.647	0.049781233	32.46	52.26	6.735	1.772
SPAC12G12.01C	SPAC12G12.01c	0.8811	0.6479	0.054974799	31.2	50.18	5.101	2.751
SPAC17H9.01	cid16	0.8628	0.6519	0.064089864	31.02	49.88	4.793	2.108
SPAC1420.03	rpn501	0.9115	0.6539	0.040243327	33.99	54.76	8.361	2.162
SPBC24C6.09C	SPBC24C6.09c	0.9066	0.6594	0.042584285	31.51	50.68	7.99	2.062
SPBC1709.14	SPBC1709.14	0.8954	0.6633	0.04798291	31.26	50.26	6.005	3.158
SPAC16E8.17C	SPAC16E8.17c	0.9358	0.6649	0.028816959	26.76	42.86	9.897	5.116
SPBC19F8.08	rps401	0.9458	0.6682	0.02420069	19.71	31.27	10.85	6.719
SPAC23H4.02	ppk9	0.8929	0.6687	0.049197177	30.41	48.85	7.038	1.851
SPAC17G6.04C	cpp1	0.7959	0.6699	0.099141495	27.52	44.11	3.766	0.7246
SPCC645.07	rgf1	0.8825	0.6738	0.054285286	22.86	36.45	6.134	2.181
SPCC1620.13	SPCC1620.13	0.9503	0.6755	0.022139271	23.97	38.27	13.62	6.213
SPAC19G12.15C	tpp1	0.9118	0.6759	0.040100412	9.011	13.69	6.82	4.126
SPCC1919.01	ppk34	0.8557	0.6791	0.067678468	31.99	51.44	4.947	1.874

SPBC12D12.06	srb11	0.9026	0.6828	0.044504671	7.663	11.47	4.573	4.517
SPBC1703.04	mlh1	0.9171	0.683	0.037583307	32.25	51.84	9.637	1.691
SPBC11B10.07C	ivn1	0.792	0.6862	0.101274818	27.46	43.97	2.857	1.78
SPAC1D4.11C	lkh1	0.8349	0.6884	0.078365539	31.6	50.78	3.867	2.109
SPAPB1A10.15	arv1	0.8756	0.6959	0.057694247	29.89	47.96	5.461	2.648
SPBC16E9.17C	rem1	0.8758	0.6999	0.057595059	32.02	51.45	6.109	2.032
SPBC32F12.06	pch1	0.8704	0.7005	0.060281118	31.62	50.79	5.267	2.563
SPCC4G3.08	psk1	0.9126	0.701	0.039719536	35.89	57.8	9.432	1.499
SPACUNK4.08	SPACUNK4.08	0.9031	0.7012	0.044264158	33.32	53.58	7.413	3.138
SPBPB2B2.09C	SPBPB2B2.09c	0.8655	0.7039	0.062732928	31.38	50.39	5.313	2.292
SPCC338.14	SPCC338.14	0.8077	0.705	0.092749917	31.07	49.88	3.402	1.839
SPAC1071.09C	SPAC1071.09c	0.8458	0.7053	0.072732319	32.14	51.63	5.335	0.8831
SPAC1805.09C	fmt1	0.9104	0.7064	0.040767751	29.77	47.75	7.153	4.149
SPBC2D10.11C	nap2	0.8185	0.7065	0.086981316	34.66	55.77	4.096	1.521
SPAC4G9.12	SPAC4G9.12	0.8969	0.7099	0.047255976	33.48	53.83	7.854	1.879
SPAC24C9.16C	cox8	0.9122	0.7108	0.039909932	27.27	43.62	7.516	4.148
SPAC4G9.05	mpf1	0.8922	0.7127	0.049537781	31.65	50.82	6.916	2.706
SPAC167.04	pam17	0.9106	0.7139	0.040672354	30.01	48.13	8.459	3.162
SPCC594.02C	SPCC594.02c	0.9177	0.7186	0.037299268	13.63	21.2	7.677	4.757
SPCC306.04C	set1	0.8585	0.7209	0.066259701	30.81	49.42	5.835	1.286
SPAC19D5.03	cid1	0.8658	0.7304	0.062582419	28.98	46.41	5.957	1.877
SPBC1685.10	rps27	0.9172	0.7321	0.037535954	33.88	54.45	9.889	2.798
SPAC23C11.14	zhf1	0.9155	0.7333	0.038341651	17.18	27.01	4.079	6.002
SPAC1687.10	mcp1	0.7677	0.7348	0.114808459	31.68	50.82	3.037	1.486
SPAPB2C8.01	SPAPB2C8.01	0.8205	0.7365	0.085921415	30.98	49.68	4.58	1.229
SPCP20C8.01C	SPCP20C8.01c	0.9232	0.7408	0.034704204	35.27	56.72	9.657	4.404
SPAC12G12.03	cip2	0.7909	0.7412	0.101878424	32.91	52.83	3.954	1.029
SPAC20G8.04C	SPAC20G8.04c	0.8223	0.7413	0.08496971	29.8	47.73	4.471	1.529
SPCC576.01C	SPCC576.01c	0.8965	0.7422	0.047449706	28.15	45.03	7.865	2.487
SPAC2G11.09	SPAC2G11.09	0.8917	0.7485	0.049781233	27.8	44.44	6.496	3.479
SPBC409.18	SPBC409.18	0.8895	0.7509	0.050854048	32.74	52.55	7.764	1.812
SPCC74.04	SPCC74.04	0.9416	0.7524	0.02613355	20.49	32.41	11.46	6.955
SPAC25H1.09	mde5	0.7917	0.7582	0.101439355	29.65	47.45	3.877	1.339
SPCC16C4.12	naa20	0.9521	0.7593	0.021317435	23.08	36.67	14.17	8.514
SPBC56F2.05C	SPBC56F2.05c	0.9325	0.7611	0.03035116	13.48	20.9	9.284	6.493
SPAC513.02	SPAC513.02	0.8094	0.7623	0.0918368	29.69	47.52	4.049	1.725
SPBC409.19C	SPBC409.19c	0.9227	0.7645	0.034939479	16.13	25.24	8.65	5.412
SPBC16G5.07C	SPBC16G5.07c	0.8549	0.7701	0.068084683	30.93	49.54	4.917	2.703
SPAC1952.11C	ure2	0.8522	0.7708	0.06945847	30.84	49.38	4.554	2.832
SPBC19G7.01C	msh2	0.8459	0.7714	0.072680975	21.79	34.53	5.591	1.527
SPBC115.02C	SPBC115.02c	0.8518	0.7743	0.069662364	31.93	51.18	5.121	2.443
SPBC32C12.03C	ppk25	0.8866	0.7745	0.052272273	28.61	45.72	6.803	3.125
SPBC16A3.03C	ppr7	0.8869	0.7749	0.052125345	28.49	45.53	7.026	2.949
SPAC1039.06	SPAC1039.06	0.8762	0.775	0.057396751	31.38	50.28	5.816	3.187
SPBC83.03C	tas3	0.8459	0.7758	0.072680975	23.14	36.73	5.829	1.094
SPBC3B9.09	vps36	0.8902	0.7785	0.05051241	32.02	51.31	6.733	3.519
SPCC613.03	SPCC613.03	0.9068	0.7803	0.042488489	30.17	48.27	8.566	3.639
SPCC24B10.19C	SPCC24B10.19	0.857	0.7807	0.067019178	30.67	49.1	5.217	2.663
SPCC553.01C	SPCC553.01c	0.794	0.7887	0.100179498	32.17	51.54	3.79	1.72
SPBC1604.20C	tea2	0.8166	0.7892	0.087990624	26.11	41.6	4.414	1.802
SPAC4A8.05C	myp2	0.8872	0.7961	0.051978467	27.9	44.53	7.41	2.853
SPAC26A3.17C	SPAC26A3.17c	0.7778	0.8008	0.109132061	32.32	51.78	2.852	2.079
SPBC609.03	iqw1	0.8097	0.8084	0.091675861	30.53	48.81	3.214	2.537
SPCC757.02C	SPCC757.02c	0.8936	0.8102	0.04885684	31.23	49.97	8.674	2.093
SPAC1952.17C	SPAC1952.17c	0.9423	0.8105	0.025810809	22.01	34.83	12.51	7.568
SPAC57A10.14	sgf11	0.881	0.8106	0.055024092	19.06	29.98	4.762	4.271
SPBC21C3.09C	SPBC21C3.09c	0.8406	0.8126	0.075410614	31.5	50.41	5.589	1.72
SPCC1235.02	bio2	0.8982	0.8137	0.046626949	31.28	50.05	6.829	4.455
SPCC285.15C	rps2802	0.9099	0.8189	0.041006335	23.37	37.05	7.58	5.176
SPAC1399.05C	SPAC1399.05c	0.872	0.8216	0.059483515	28.43	45.34	6.478	2.851
SPBC365.14C	uge1	0.9158	0.8268	0.038199361	17.56	27.48	6.93	6.15
SPAC23H4.17C	srb10	0.7227	0.8271	0.141041945	3.672	4.672	2.893	1.346
SPBC1703.07	SPBC1703.07	0.8656	0.8289	0.062682752	32.4	51.85	6.397	2.564
SPBP35G2.14	SPBP35G2.14	0.7361	0.8303	0.133063182	31.72	50.75	3.239	1.244
SPAC12G12.10	wdr21	0.9071	0.8332	0.042344833	26.44	42.06	8.96	4.102
SPAC2G11.13	atg22	0.8988	0.8373	0.046336936	33.01	52.84	9.308	2.494
SPBC4F6.12	pxl1	0.8028	0.8386	0.095392636	28.96	46.2	4.733	1.269
SPBC21C3.17C	SPBC21C3.17c	0.7968	0.8415	0.098650675	32.52	52.04	3.965	1.977
SPAP27G11.06C	vas2	0.9112	0.8458	0.040386289	15.34	23.81	8.328	5.209

SPAC57A7.07C	SPAC57A7.07c	0.8213	0.848	0.085498177	29.8	47.55	4.739	2.119
SPBC21.07C	ppk24	0.8361	0.8492	0.077741777	29.93	47.77	5.306	2.2
SPCC4B3.02C	SPCC4B3.02c	0.8315	0.8492	0.080137746	33.06	52.91	5.669	1.443
SPCC1672.06C	asp1	0.8748	0.85	0.058091226	33.2	53.13	6.228	3.512
SPAC1556.04C	cdd1	0.7812	0.8507	0.107237765	30.47	48.65	3.694	1.869
SPAC4H3.01	SPAC4H3.01	0.9049	0.8548	0.043399412	34.43	55.15	10.05	2.828
SPAC1783.08C	rpl1502	0.9384	0.8563	0.027612001	13.26	20.37	12.02	7.702
SPAC19G12.10C	cpy1	0.8092	0.8658	0.091944126	32.31	51.66	4.009	2.413
SPBC3E7.09	SPBC3E7.09	0.8813	0.8663	0.05487623	33.61	53.78	8.493	1.568
SPAC1687.19C	SPAC1687.19c	0.8546	0.8671	0.068237112	33.8	54.1	6.616	1.904
SPBC1709.09	rrf1	0.8993	0.8695	0.046095407	16.89	26.32	7.597	4.687
SPCC790.03	SPCC790.03	0.876	0.8702	0.057495894	11.31	17.15	6.195	3.786
SPAC644.06C	cdr1	0.8355	0.8749	0.078053546	8.896	13.17	4.407	3.009
SPAC1B1.02C	SPAC1B1.02c	0.8548	0.8765	0.068135487	29.18	46.49	3.775	3.918
SPCC1259.01C	rps1802	0.8955	0.8806	0.04793441	10.67	16.08	7.58	4.471
SPAC12B10.04	SPAC12B10.04	0.9291	0.8807	0.03193754	26.62	42.28	11.05	6.695
SPBC30B4.08	eri1	0.7758	0.8836	0.110250225	27.38	43.52	4.437	1.038
SPBC56F2.10C	alg5	0.738	0.8849	0.131943638	25.45	40.35	0.8163	2.378
SPAC20H4.08	SPAC20H4.08	0.8507	0.8854	0.070223567	30.66	48.9	6.066	2.538
SPAC13G6.08	SPAC13G6.08	0.7641	0.889	0.1168498	29.95	47.73	3.699	1.699
SPAC25A8.01C	fft3	0.78	0.8892	0.107905397	24.48	38.75	3.39	2.236
SPBC29B5.04C	SPBC29B5.04c	0.8428	0.89	0.074275473	31.77	50.73	6	2.188
SPCC23B6.03C	tel1	0.9291	0.8914	0.03193754	36.78	58.95	13.26	5.057
SPAC7D4.02C	sfp47	0.8843	0.8916	0.053400375	34.44	55.1	8.943	1.723
SPAC16A10.03C	SPAC16A10.03c	0.9221	0.8966	0.035221978	17.71	27.62	10	6.336
SPAC1F3.05	SPAC1F3.05	0.8728	0.8973	0.059085262	33.72	53.92	8.251	1.368
SPBC691.04	SPBC691.04	0.8188	0.8993	0.086822166	32.51	51.93	5.88	0.4483
SPAC328.01C	SPAC328.01c	0.791	0.9015	0.101823517	32.21	51.43	4.721	1.404
SPAC521.05	rps802	0.9269	0.9042	0.032967118	18.99	29.7	10.94	6.71
SPBC1709.13C	set10	0.9405	0.9054	0.0266412	25.2	39.91	13.07	8.49
SPBC16C6.06	vps10	0.816	0.9098	0.088309841	25.75	40.8	5.512	1.484
SPBC106.20	exo70	0.8772	0.9173	0.056901377	36.18	57.92	8.795	1.278
SPAC4C5.01	SPAC4C5.01	0.8848	0.918	0.053154886	26.38	41.82	8.048	3.55
SPBC14C8.16C	bot1	0.9295	0.9201	0.031750606	20.92	32.85	11.51	7.096
SPCP1E11.05C	are2	0.8989	0.9208	0.04628862	35.12	56.18	10.75	1.533
SPAC1B3.05	not3	0.909	0.9211	0.041436117	12.47	18.96	8.543	5.715
SPCC736.11	ago1	0.5963	0.9216	0.224535191	1.493	0.9393	0.9393	1.493
SPBC1348.14C	ght7	0.8274	0.9222	0.082284483	31.04	49.48	5.753	1.93
SPAC17G6.13	slt1	0.8895	0.9246	0.050854048	36.6	58.59	9.574	2.199
SPBC1861.02	abp2	0.8966	0.9265	0.047401265	37.61	60.25	9.126	3.931
SPAC22F3.03C	rdh54	0.8362	0.927	0.077689837	32.74	52.26	4.337	3.373
SPAC4F10.13C	mpd2	0.9016	0.9281	0.044986097	18.66	29.13	8.917	4.721
SPBC2D10.14C	myo51	0.7811	0.9307	0.107293362	35.14	56.2	4.806	1.085
SPAC140.01	sdh2	0.9385	0.9311	0.027565723	17.83	27.76	12.89	8.497
SPAC26F1.14C	aif1	0.7924	0.9329	0.101055533	31.38	50.01	5.019	1.291
SPBC1778.03C	SPBC1778.03c	0.8703	0.9332	0.060331016	27.63	43.85	6.87	3.527
SPCC70.10	SPCC70.10	0.9237	0.9343	0.034469056	28.45	45.19	11.14	6.443
SPAC212.08C	SPAC212.08c	0.7585	0.9344	0.120044415	29.21	46.44	2.704	2.372
SPAC140.03	arb1	0.8798	0.9345	0.055616042	31.73	50.58	7.705	3.584
SPCC74.02C	SPCC74.02c	0.9272	0.9369	0.032826577	21.11	33.14	11.15	7.113
SPBC215.05	gpd1	0.8509	0.9376	0.070121476	34.24	54.7	7.015	1.936
SPBC29A3.13	pdp1	0.8478	0.94	0.071706588	29.24	46.49	6.38	2.578
SPCC16A11.04	snx12	0.8396	0.9445	0.07592757	29.42	46.78	5.895	2.631
SPBC3B9.04	SPBC3B9.04	0.8505	0.9453	0.070325682	28.59	45.41	6.199	2.954
SPAC1142.05	ctr5	0.8144	0.9468	0.089162235	4.966	6.602	3.909	3.027
SPBC17A3.08	SPBC17A3.08	0.869	0.9519	0.060980224	30.65	48.79	7.491	3.066
SPBC543.09	yta12	0.7672	0.9522	0.115091406	30.83	49.08	4.128	1.739
SPAC25A8.02	SPAC25A8.02	0.7494	0.9557	0.125286311	31.99	50.97	3.823	1.631
SPBC1289.09	tim21	0.8699	0.9586	0.060530669	31.18	49.65	8.062	2.545
SPCC1393.05	ers1	0.7755	0.9596	0.110418198	27.74	43.99	5.026	0.398
SPAC12B10.07	acp1	0.7726	0.9614	0.112045296	33.46	53.38	4.424	1.633
SPAC4H3.04C	SPAC4H3.04c	0.8773	0.9647	0.056851871	27.31	43.27	7.896	3.536
SPCC24B10.11C	mft1	0.6831	0.9671	0.165515715	25.85	40.88	2.442	1.694
SPCC1223.02	nmt1	0.8285	0.9674	0.081707487	26.28	41.58	1.669	4.01
SPBC947.01	SPBC947.01	0.8016	0.9682	0.096042291	30.63	48.72	4.749	2.271
SPBC2D10.05	exg3	0.8349	0.977	0.078365539	31.13	49.53	6.394	2.118
SPAC4G9.15	SPAC4G9.15	0.8259	0.9792	0.083072534	9.925	14.69	4.692	3.159
SPAC5H10.12C	SPAC5H10.12c	0.9051	0.9795	0.043303435	33.28	53.05	11.27	3.666
SPBC8D2.02C	SPBC8D2.02c	0.7538	0.9799	0.122743867	30.41	48.35	4.172	1.507

SPAC56E4.06C	ggt2	0.8912	0.985	0.050024822	24.21	38.15	8.027	4.879
SPAC14C4.05C	man1	0.7465	0.9868	0.126970188	25.8	40.76	2.293	2.516
SPBC887.08	SPBC887.08	0.7507	0.9871	0.124533584	26.63	42.12	2.897	2.372
SPBC365.10	arp5	0.7898	0.9874	0.102482871	7.781	11.16	4.489	2.246
SPAC9E9.08	rad26	0.8135	0.9907	0.089642443	32.4	51.59	5.872	1.678
SPAC458.02C	SPAC458.02c	0.7851	0.9916	0.105075023	33.28	53.04	5.102	1.411
SPAC139.01C	SPAC139.01c	0.8709	0.9945	0.060031709	30.89	49.11	7.377	3.756
SPBP4H10.13	rps2302	0.8391	0.9948	0.076186279	23.29	36.62	5.696	3.157
SPAC3A11.07	SPAC3A11.07	0.9125	0.9984	0.039767127	18.15	28.17	10.06	6.2
SPAPB1A10.10C	ypt71	0.7716	0.9993	0.112607781	29.95	47.55	4.239	2.028
SPBC20F10.03	SPBC20F10.03	0.8008	0.9994	0.096475936	30.68	48.75	5.152	2.082
SPAC9.10	thi9	0.8553	1.003	0.067881528	33.67	53.66	7.498	2.498
SPAC17C9.07	alg8	0.6535	1.004	0.184754408	25.94	40.97	2.206	1.644
SPBC18E5.09C	EMPTY	0.7845	1.005	0.105407052	31.1	49.43	4.377	2.284
SPAC16.01	rho2	0.9315	1.007	0.030817141	26.4	41.7	13.35	7.759
SPAC3A11.02	cps3	0.938	1.013	0.027797162	20.75	32.42	14.83	8.641
SPBC29A10.16C	SPBC29A10.16c	0.9309	1.017	0.03109697	25.37	40	13.87	7.438
SPBC83.04	apc15	0.6101	1.019	0.214598975	32.27	51.33	2.555	1.058
SPAC1006.09	win1	0.8869	1.02	0.052125345	33.05	52.61	7.785	4.985
SPAC16C9.01C	SPAC16C9.01c	0.8031	1.023	0.095230374	30.88	49.05	5.315	2.182
SPBCPT2R1.01C	SPBCPT2R1.01	0.784	1.024	0.105683937	27.82	44.02	4.804	2.013
SPAC186.03	SPAC186.03	0.7625	1.031	0.117760152	31.51	50.06	3.998	2.165
SPAC7D4.04	taf1	0.7564	1.031	0.12124848	29.8	47.25	4.392	1.659
SPBP8B7.09C	los1	0.9272	1.032	0.032826577	20.12	31.35	12.39	7.764
SPCC1682.14	rpl1902	0.8632	1.033	0.063888568	20.46	31.91	7.099	3.772
SPBC660.05	SPBC660.05	0.8794	1.034	0.055813539	29.89	47.4	7.279	4.799
SPAC22E12.06C	gmh3	0.7986	1.034	0.097670694	31.43	49.93	5.74	1.484
SPAC1F8.08	SPAC1F8.08	0.8549	1.035	0.068084683	34.29	54.63	8.12	1.889
SPBC1306.02	SPBC1306.02	0.8918	1.039	0.049732532	15.43	23.64	8.48	5.183
SPBC29A3.03C	SPBC29A3.03c	0.791	1.039	0.101823517	32.62	51.87	5.035	2.126
SPBC17D1.07C	SPBC17D1.07c	0.7419	1.043	0.129654629	30.88	49.02	3.444	2.177
SPAC6B12.06C	rrg9	0.8713	1.043	0.059832286	34.08	54.27	9.155	2.329
SPBP19A11.02C	SPBP19A11.02c	0.8456	1.048	0.072835026	32.69	51.97	7.606	2.023
SPCC364.07	SPCC364.07	0.8699	1.049	0.060530669	28.85	45.67	8.209	3.509
SPAC25G10.04C	rec10	0.8445	1.05	0.073400346	35.94	57.31	7.683	1.794
SPBC2A9.05C	tvp23	0.8121	1.053	0.09039049	27.28	43.08	6.191	1.771
SPBC31F10.17C	SPBC31F10.17c	0.9046	1.056	0.043543417	40.47	64.74	12.61	3.059
SPCC330.01C	rhp16	0.7212	1.06	0.141944282	32.93	52.34	3.984	1.397
SPAC1F7.01C	spt6	0.6794	1.067	0.167874457	29.4	46.54	3.716	0.739
SPAC31A2.14	bun107	0.8701	1.069	0.060430831	27.68	43.71	7.343	4.388
SPAC3H1.14	SPAC3H1.14	0.8193	1.071	0.086557045	29.72	47.06	5.856	2.7
SPAC22F3.06C	lon1	0.7947	1.071	0.099796787	27.67	43.69	3.44	3.298
SPAC1399.04C	SPAC1399.04c	0.8908	1.072	0.050219792	32.13	51.01	10.2	4.085
SPBC14F5.07	doa10	0.6788	1.073	0.168258166	31.13	49.37	3.442	1.247
SPBP4H10.14C	SPBP4H10.14c	0.7307	1.073	0.136260893	30.96	49.1	4.005	1.671
SPBC29A3.21	SPBC29A3.21	0.9147	1.078	0.038721321	31.69	50.29	11.69	6.52
SPAC22H12.03	SPAC22H12.03	0.7976	1.083	0.098214855	30.89	48.96	5.84	1.78
SPBC18H10.06C	swd2	0.8847	1.083	0.053203973	27.45	43.3	8.92	4.934
SPBC409.16C	SPBC409.16c	0.819	1.086	0.086716098	27.73	43.77	6.301	2.36
SPBC3B9.05	SPBC3B9.05	0.7406	1.09	0.130416292	25.54	40.17	2.866	2.59
SPCC737.04	SPCC737.04	0.8511	1.091	0.070019409	29.9	47.32	7.177	3.428
SPBC691.05C	SPBC691.05c	0.806	1.092	0.093664958	32.89	52.23	5.949	2.148
SPAC57A7.12	SPAC57A7.12	0.4517	1.092	0.345149909	2.301	1.986	0.817	1.297
SPBC1778.09	SPBC1778.09	0.7884	1.096	0.103253384	32.16	51.03	5.213	2.238
SPAC4F8.01	did4	0.7796	1.096	0.10812817	33.92	53.91	5.034	2.117
SPBC15D4.13C	SPBC15D4.13c	0.8249	1.098	0.083598696	32.72	51.95	5.945	3.066
SPBC2D10.09	SPBC2D10.09	0.8419	1.098	0.07473949	31.32	49.64	7.928	1.78
SPAC19D5.07	uga1	0.8095	1.102	0.091783147	31.49	49.91	5.507	2.8
SPCC830.06	SPCC830.06	0.9005	1.102	0.045516283	19.78	30.68	8.274	6.745
SPCC613.11C	meu23	0.7125	1.103	0.147215131	29.48	46.61	4.313	0.8801
SPBC691.01	pfa5	0.6491	1.104	0.187688391	31.2	49.44	3.245	1.13
SPAC4G8.08	SPAC4G8.08	0.8105	1.11	0.091246981	31.57	50.04	5.664	2.766
SPAC1F12.05	SPAC1F12.05	0.9122	1.111	0.039909932	26.58	41.84	10.88	7.028
SPCC965.14C	SPCC965.14c	0.8155	1.111	0.088576035	32.93	52.26	6.396	2.278
SPAC4H3.07C	SPAC4H3.07c	0.9098	1.115	0.041054068	26.33	41.42	10.79	6.782
SPBC4.02C	SPBC4.02c	0.8555	1.12	0.067779986	34.31	54.52	8.674	2.349
SPBC19F8.04C	SPBC19F8.04c	0.6963	1.121	0.157203605	25.33	39.77	2.146	2.363
SPBC3H7.07C	ser2	0.7908	1.122	0.101933339	33.1	52.53	5.122	2.567
SPAC1783.07C	pap1	0.6238	1.124	0.20495463	17.71	27.25	3.156	0.9191

SPBC947.14C	cbp6	0.8802	1.126	0.055418636	10.12	14.77	6.305	5.984
SPAC25G10.09C	pan1	0.7612	1.127	0.11850122	25.86	40.63	4.892	1.859
SPBC8D2.03C	hhf2	0.9182	1.127	0.037062712	24	37.57	12.3	7.388
SPAC4G9.14	SPAC4G9.14	0.8926	1.127	0.049343117	37.1	59.08	11.89	3.013
SPBC1921.05	ape2	0.6334	1.128	0.198321941	31.54	49.96	3.097	1.179
SPBC3E7.07C	SPBC3E7.07c	0.7083	1.131	0.149782758	29.88	47.22	3.782	1.715
SPAC30D11.13	hus5	0.7324	1.132	0.135251664	2.7	2.576	2.576	2.7
SPAC4F10.18	nup37	0.9312	1.132	0.030957033	26.5	41.67	14.52	8.934
SPBC776.11	rpl2801	0.8872	1.133	0.051978467	17.39	26.7	9.471	5.035
SPBC1347.09	SPBC1347.09	0.8561	1.134	0.067475503	32.35	51.28	9.009	2.019
SPBP35G2.06C	nup131	0.8393	1.137	0.076082777	31.84	50.43	7.189	3.063
SPAC328.10C	rps502	0.8466	1.137	0.072321736	21.84	34	6.441	4.044
SPAC27D7.05C	apc14	0.7908	1.142	0.101933339	34.7	55.11	6.058	1.642
SPBC12D12.09	rev7	0.7681	1.144	0.114582235	28.86	45.53	5.258	1.78
SPAC23C4.03	hrk1	0.9122	1.147	0.039909932	23.52	36.74	12.78	6.197
SPBPB2B2.08	SPBPB2B2.08	0.7991	1.148	0.097398869	28.27	44.55	5.38	2.802
SPAC343.20	SPAC343.20	0.7102	1.15	0.148619332	32.45	51.41	4.174	1.427
SPAC3C7.08C	elf1	0.9142	1.157	0.038958783	22.12	34.44	11.69	7.439
SPAPB24D3.01	SPAPB24D3.01	0.7483	1.158	0.125924255	26.9	42.28	4.426	2.136
SPAC11E3.15	rpl22	0.791	1.161	0.101823517	33.67	53.4	5.58	2.417
SPAC926.07C	dlc2	0.8395	1.161	0.0759793	33.66	53.38	8.053	2.242
SPAPJ691.03	SPAPJ691.03	0.7242	1.164	0.14014148	39.56	63.07	1.05	2.959
SPCC1020.08	SPCC1020.08	0.8059	1.167	0.093718844	33.41	52.95	6.754	1.689
SPBP35G2.02	SPBP35G2.02	0.7993	1.168	0.097290187	29.46	46.48	4.629	3.363
SPAC1420.01C	SPAC1420.01c	0.7724	1.17	0.112157735	30.91	48.85	5.291	2.089
SPCC663.06C	SPCC663.06c	0.7994	1.173	0.097235856	26.11	40.97	5.915	2.515
SPCC1840.06	atp5	0.778	1.174	0.109020403	33.55	53.18	5.747	1.769
SPAPB1E7.11C	SPAPB1E7.11c	0.8796	1.175	0.055714779	10.67	15.6	7.989	5.862
SPAC9G1.02	wis4	0.8936	1.181	0.04885684	39.16	62.38	12.38	3.503
SPBC713.08	mim1	0.8593	1.181	0.065855188	13.49	20.23	7.095	4.696
SPCC4B3.07	nro1	0.7381	1.182	0.131884795	30.91	48.83	4.953	1.355
SPAC8C9.12C	SPAC8C9.12c	0.9239	1.184	0.034375033	25.27	39.57	13.26	8.71
SPAC1556.01C	rad50	0.8715	1.185	0.059732609	8.887	12.65	7.531	5.308
SPAC1687.16C	erg31	0.7655	1.186	0.116054805	33.24	52.65	5.102	2.15
SPBC36.04	cys11	0.7231	1.189	0.140801638	31.69	50.11	3.989	2.081
SPAC23C11.08	php3	0.8295	1.19	0.08118361	33.01	52.26	7.986	1.715
SPAC4F10.04	SPAC4F10.04	0.826	1.195	0.083019953	19.14	29.48	7.146	2.788
SPAPB1A11.04C	SPAPB1A11.04c	0.6804	1.196	0.167235695	34.4	54.54	4.106	1.008
SPBC13E7.09	vrp1	0.7661	1.197	0.115714538	32.89	52.06	5.749	1.346
SPAC1F7.08	fio1	0.8983	1.197	0.0465786	21.18	32.82	12.41	4.708
SPCC1682.13	laf2	0.7437	1.198	0.128602219	30.74	48.52	4.625	2.052
SPAC1F7.10	SPAC1F7.10	0.8117	1.199	0.090604454	30.57	48.24	7.009	2.047
SPAC4A8.02C	SPAC4A8.02c	0.7718	1.199	0.112495226	33.77	53.5	5.537	1.979
SPAC926.06C	SPAC926.06c	0.7711	1.203	0.112889297	31.71	50.1	5.29	2.259
SPBC16A3.12C	SPBC16A3.12c	0.7738	1.208	0.111371275	33.67	53.32	5.265	2.396
SPCC777.04	SPCC777.04	0.6918	1.213	0.160019442	31.26	49.36	4.11	1.422
SPAC26H5.11	mug56	0.7978	1.215	0.098105968	32.78	51.85	5.958	2.692
SPBC1861.09	ppk22	0.8514	1.216	0.069866354	28.2	44.33	6.508	4.783
SPBC428.03C	pho4	0.7235	1.219	0.140561465	30.5	48.1	4.969	1.013
SPBC1685.02C	rps1202	0.891	1.223	0.050122296	18.46	28.32	9.557	6.273
SPCC18B5.09C	SPCC18B5.09c	0.6662	1.224	0.176395372	31.71	50.08	3.987	1.027
SPBC1683.10C	pcl1	0.8484	1.226	0.07139934	24.03	37.46	8.792	2.842
SPAC14C4.04	B22918-2	0.6484	1.226	0.188156994	28.59	44.95	2.908	1.844
SPAC22F3.02	atf31	0.7129	1.227	0.146971385	33.13	52.4	4.105	1.951
SPAC2F3.07C	SPAC2F3.07c	0.7482	1.227	0.125982296	32.68	51.66	5.186	1.734
SPCC777.06C	SPCC777.06c	0.6813	1.227	0.166661611	26.47	41.46	2.154	2.476
SPAC22F8.12C	shf1	0.8061	1.228	0.093611079	22.87	35.55	5.786	3.238
SPAC513.03	mfm2	0.5274	1.23	0.277859875	24.29	37.88	2.515	0.9742
SPCC1753.05	rsm1	0.9104	1.23	0.040767751	22.55	35.02	12.04	7.494
SPBC1105.05	exg1	0.7574	1.231	0.120674699	32.31	51.05	5.192	2.074
SPBC17G9.12C	SPBC17G9.12c	0.8531	1.233	0.069000058	29.44	46.33	8.133	4.004
SPAC1B3.11C	ypt4	0.525	1.234	0.279840697	28.55	44.88	2.307	1.165
SPBC29A10.10C	SPBC29A10.10c	0.8456	1.236	0.072835026	36.32	57.63	9.206	1.903
SPAC16E8.14C	tae1	0.7635	1.237	0.117190959	30.18	47.53	5.485	1.993
SPBC32H8.03	bem46	0.858	1.238	0.066512712	28.73	45.16	8.442	4.171
SPBC6B1.03C	SPBC6B1.03c	0.7195	1.238	0.142969202	31.55	49.78	4.279	1.989
SPAC1006.01	psp3	0.7044	1.239	0.152180653	30.75	48.47	4.118	1.823
SPBC1921.04C	SPBC1921.04c	0.7277	1.242	0.138047625	31.41	49.56	3.386	2.697
SPAC11E3.09	pyp3	0.8302	1.249	0.080817271	31.76	50.12	7.862	2.738

SPCC13B11.04C	SPCC13B11.04c	0.7309	1.252	0.136142038	28.26	44.37	5.206	1.177
SPBC646.09C	int6	0.6232	1.255	0.205372555	31.54	49.74	3.472	1.096
SPAC5H10.10	SPAC5H10.10	0.7102	1.255	0.148619332	32.4	51.15	4.908	0.8714
SPAC4H3.03C	SPAC4H3.03c	0.7822	1.255	0.106682188	30.92	48.73	6.186	2.038
SPAC17A5.11	rec12	0.7156	1.257	0.145329668	32.16	50.77	4.064	2.164
SPCC1840.02C	bgs4	0.766	1.262	0.11577123	32.11	50.68	5.923	1.677
SPBPB10D8.04C	SPBPB10D8.04c	0.7632	1.264	0.117361638	32.22	50.84	4.838	2.716
SPCP31B10.06	mug190	0.8449	1.265	0.07319469	34.94	55.31	9.266	2.183
SPAC140.02	gar2	0.8545	1.266	0.068287933	27.48	43.06	8.865	3.767
SPAC23A1.06C	cmk2	0.8227	1.268	0.084758503	33.83	53.49	7.572	2.739
SPBC418.01C	his4	0.5428	1.268	0.265360161	29.62	46.57	2.345	1.351
SPBC887.06C	snx3	0.8297	1.27	0.08107891	34.32	54.29	8.157	2.518
SPBC1921.03C	mex67	0.8717	1.274	0.059632954	20.33	31.3	9.935	4.495
SPAC22E12.18	SPAC22E12.18	0.6945	1.275	0.15832775	5.957	7.69	3.434	2.277
SPAC23A1.09	SPAC23A1.09	0.7406	1.276	0.130416292	31.22	49.18	5.413	1.46
SPBC2D10.13	est1	0.8986	1.278	0.046433586	20.75	31.99	10.85	6.978
SPBC839.17C	fkh1	0.74	1.28	0.13076828	33.34	52.66	5.424	1.449
SPBP8B7.02	SPBP8B7.02	0.7228	1.281	0.140981856	34.3	54.23	3.999	2.44
SPAC13G7.09C	SPAC13G7.09c	0.5885	1.282	0.230253533	30.36	47.76	3.262	0.9344
SPAC1687.12C	coq4	0.7311	1.286	0.136023216	5.051	6.184	4.011	2.603
SPBC800.12C	SPBC800.12c	0.7357	1.287	0.133299244	31.28	49.27	4.573	2.335
SPBC839.02	SPBC839.02	0.8243	1.291	0.0839147	26.79	41.88	7.387	3.24
SPCC1450.02	bdf1	0.9089	1.291	0.041483897	22.92	35.52	12.08	7.932
SPCC1620.04C	mug55	0.7496	1.296	0.125170422	30.58	48.11	5.48	1.879
SPBC651.07	EMPTY	0.656	1.296	0.183096161	29.99	47.14	3.202	1.963
SPBC543.08	SPBC543.08	0.7243	1.296	0.140081515	31.67	49.9	4.874	1.802
SPAC3A11.11C	SPAC3A11.11c	0.9036	1.296	0.044023778	23.29	36.13	11.39	7.56
SPCC970.07C	raf2	0.9096	1.297	0.041149548	21.31	32.87	12.39	7.939
SPAC186.09	SPAC186.09	0.7666	1.301	0.115431185	31.9	50.26	6.233	1.524
SPAC22A12.10	SPAC22A12.10	0.8187	1.302	0.08687521	30.08	47.27	7.354	3.025
SPAC1006.06	rgf2	0.6957	1.307	0.157577997	32.54	51.31	4.802	0.9944
SPBC11G11.01	fis1	0.889	1.308	0.051098239	21.66	33.43	10.44	6.34
SPAC2G11.06	vps4	0.5129	1.309	0.289967301	25.55	39.81	2.238	1.3
SPBC16D10.11C	rps1801	0.8805	1.31	0.05527064	18.1	27.59	9.355	6.109
SPBC530.10C	anc1	0.6061	1.31	0.217455716	27.1	42.36	1.577	2.147
SPBC25B2.01	SPBC25B2.01	0.7257	1.31	0.139242877	31.3	49.26	4.26	2.442
SPAC1093.06C	dhc1	0.7445	1.311	0.128135298	28.3	44.33	4.362	2.775
SPBC19C7.10	bqt4	0.6843	1.312	0.16475346	32.12	50.6	4.428	1.365
SPCC74.09	mug24	0.739	1.316	0.131355562	27.95	43.75	5.45	1.658
SPBC27B12.10C	tom7	0.8572	1.317	0.066917838	14.05	20.91	7.541	5.296
SPAC23H3.11C	SPAC23H3.11c	0.7075	1.318	0.150273556	31.96	50.33	4.408	1.978
SPCC24B10.22	pog1	0.68	1.325	0.167491087	32.88	51.84	3.325	2.291
SPBC56F2.08C	SPBC56F2.08c	0.6678	1.33	0.175353585	25.06	38.97	4.015	1.609
SPAP27G11.08C	meu32	0.7608	1.332	0.118729496	34.87	55.08	6.052	1.822
SPBC1271.01C	pof13	0.7009	1.332	0.15434394	31.61	49.73	4.046	2.201
SPAC3A12.03C	meu34	0.712	1.335	0.147520006	28.03	43.86	5.084	1.338
SPBC1A4.09	SPBC1A4.09	0.8355	1.335	0.078053546	7.984	10.92	6.254	5.02
SPBC215.04	git11	0.7673	1.337	0.115034802	26.56	41.43	4.061	3.512
SPBC16G5.15C	fkh2	0.3889	1.339	0.410162057	20.86	32.07	1.249	1.201
SPBC146.06C	SPBC146.06c	0.6957	1.34	0.157577997	31.37	49.33	4.166	2.043
SPBC1E8.03C	SPBC1E8.03c	0.7133	1.341	0.146727776	34.04	53.72	4.582	2.058
SPAC589.07C	atg18	0.9144	1.343	0.038863783	25.22	39.23	13.72	8.598
SPCC4G3.02	aph1	0.4519	1.344	0.344957659	13.51	19.98	2.036	1.12
SPBC1347.13C	SPBC1347.13c	0.9057	1.347	0.043015632	22.95	35.49	12.24	7.951
SPCC736.08	cbf11	0.6163	1.349	0.210207832	26.19	40.8	1.771	2.257
SPBC1347.02	fkbp39	0.8076	1.354	0.09280369	25.04	38.91	6.882	3.252
SPAC29B12.08	SPAC29B12.08	0.8829	1.355	0.054088483	25.99	40.47	11.29	5.491
SPBC649.02	rps1902	0.9021	1.355	0.044745317	22.12	34.12	12.07	7.587
SPBP22H7.08	rps1002	0.6722	1.355	0.172501492	34.04	53.69	4.509	1.144
SPBC336.13C	SPBC336.13c	0.7786	1.355	0.108685601	10.3	14.69	4.963	3.475
SPBC15D4.06	naa30	0.6792	1.357	0.168002323	28.9	45.25	3.823	2.081
SPBC4B4.06	vps25	0.8604	1.358	0.065299598	28.29	44.24	10.58	3.451
SPBC660.12C	SPBC660.12c	0.828	1.359	0.081969663	34.93	55.15	9.073	1.854
SPBC4B4.12C	SPBC4B4.12c	0.7224	1.359	0.141222263	33.23	52.35	5.207	1.703
SPAC22A12.02C	mug103	0.6998	1.361	0.155026062	35.17	55.53	4.516	1.902
SPAC30C2.06C	dml1	0.674	1.363	0.171340103	19.41	29.65	3.516	2.222
SPAPB8E5.05	mfm1	0.7756	1.367	0.1103622	30.49	47.84	6.28	2.464
SPBC8E4.04	SPBC8E4.04	0.8198	1.368	0.086292086	33.62	52.98	7.871	3.098
SPAC821.06	spn2	0.7822	1.369	0.106682188	32.11	50.49	5.608	3.277

SPAC8C9.09C	mug129	0.9148	1.378	0.038673844	17.12	25.85	13.65	9.121
SPBC902.05C	idh2	0.6645	1.38	0.177505015	29.68	46.49	4.469	1.148
SPCC584.02	cuf2	0.7603	1.381	0.11901501	28.37	44.33	5.261	2.904
SPAPB1A11.02	SPAPB1A11.02	0.8017	1.382	0.095988116	30.39	47.65	7.347	2.673
SPAC607.10	spo3	0.7883	1.386	0.103308473	32.95	51.85	5.931	3.362
SPAC17A2.06C	vps8	0.6181	1.393	0.208941256	26.98	42.02	3.056	1.888
SPCC663.04	rpl39	0.696	1.394	0.15739076	4.313	4.794	2.97	2.846
SPBC725.15	ura5	0.68	1.398	0.167491087	32.87	51.7	4.183	1.968
SPAC17C9.16C	SPAC17C9.16c	0.7227	1.399	0.141041945	31.61	49.62	5.556	1.446
SPBC2A9.06C	SPBC2A9.06c	0.6987	1.4	0.155709257	28.26	44.12	4.685	1.891
SPBC4B4.03	rsc1	0.6902	1.401	0.161025045	33.01	51.92	4.53	1.856
SPBC18H10.04C	sce3	0.8365	1.401	0.077534055	29.25	45.75	8.357	4.019
SPAC23H4.08	iwr1	0.6724	1.403	0.172372295	27.41	42.72	2.433	2.735
SPAC823.15	ppa1	0.7785	1.403	0.108741383	32.05	50.34	6.583	2.507
SPCC126.11C	SPCC126.11c	0.8516	1.404	0.069764347	34.33	54.09	10.58	2.867
SPBC342.01C	alg6	0.8787	1.404	0.056159374	16.58	24.93	9.647	6.57
SPBC17D1.06	dbp3	0.7291	1.404	0.137212902	27.74	43.26	4.99	2.379
SPAC1071.11	SPAC1071.11	0.7978	1.404	0.098105968	16.89	25.43	7.698	2.116
SPAC22E12.14C	sck2	0.6048	1.417	0.218388218	31.19	48.91	3.953	0.6558
SPBC27B12.09C	SPBC27B12.09(	0.6877	1.421	0.162600976	33.22	52.24	4.077	2.274
SPBC1921.07C	sgf29	0.8683	1.422	0.061330199	18.15	27.49	11.05	4.638
SPAC26H5.07C	SPAC26H5.07c	0.8733	1.424	0.05883654	18.28	27.69	9.708	6.188
SPAC6F6.02C	pof5	0.771	1.428	0.112945622	30.08	47.07	6.544	2.386
SPAC10F6.17C	SPAC10F6.17c	0.7301	1.429	0.136617652	33.5	52.69	5.22	2.319
SPCC1259.14C	meu27	0.7168	1.43	0.144602003	30.7	48.08	5.418	1.673
SPAC31A2.12	SPAC31A2.12	0.8228	1.431	0.084705717	22.34	34.35	7.961	3.704
SPAC26F1.08C	SPAC26F1.08c	0.5648	1.431	0.248105312	30.53	47.79	3.525	0.7285
SPCC4G3.10C	rhp42	0.7011	1.433	0.154220033	34.25	53.91	5.304	1.26
SPAC2F3.05C	SPAC2F3.05c	0.7528	1.434	0.12332039	31.04	48.63	4.614	3.311
SPBC1604.02C	ppr1	0.7697	1.435	0.113678513	7.758	10.39	4.872	3.779
SPBC13G1.04C	SPBC13G1.04c	0.7969	1.438	0.098596173	31.31	49.07	6.959	3.23
SPAC6F6.04C	SPAC6F6.04c	0.7723	1.442	0.112213965	35.48	55.92	6.782	2.243
SPAC7D4.13C	SPAC7D4.13c	0.8406	1.449	0.075410614	26.71	41.5	8.549	4.518
SPAC1002.07C	ats1	0.631	1.449	0.199970641	29.78	46.54	3.568	1.863
SPCC1739.12	ppe1	0.6997	1.45	0.155088126	29.47	46.03	4.691	2.141
SPBP8B7.27	mug30	0.6818	1.453	0.166343003	33.28	52.29	4.442	1.992
SPCC285.16C	msh6	0.5816	1.453	0.235375602	33.57	52.76	3.175	1.571
SPAC25B8.03	psd2	0.5517	1.453	0.258297016	25.1	38.84	3.372	0.9129
SPAC13F5.04C	SPAC13F5.04c	0.7914	1.457	0.101603954	33.8	53.12	7.005	3.05
SPCC1442.13C	SPCC1442.13c	0.831	1.457	0.080398976	15.38	22.87	7.654	4.579
SPAC17A2.07C	SPAC17A2.07c	0.7086	1.457	0.149598852	32.16	50.43	5.546	1.309
SPCC736.02	SPCC736.02	0.4343	1.457	0.362210171	27.33	42.5	2.09	1.184
SPAC959.06C	SPAC959.06c	0.7028	1.457	0.153168247	31	48.53	5.224	1.645
SPCC1183.11	SPCC1183.11	0.8134	1.457	0.089695832	23.76	36.63	5.954	4.645
SPBC36.10	SPBC36.10	0.8544	1.46	0.06833876	26.73	41.51	9.814	4.695
SPAC1A6.06C	meu31	0.9054	1.46	0.04315951	24.46	37.78	13.45	8.473
SPAC1486.04C	alm1	0.8657	1.462	0.062632582	25.99	40.3	9.241	6.075
SPAC11D3.03C	SPAC11D3.03c	0.6997	1.463	0.155088126	33.29	52.27	4.869	2.03
SPBC19C7.08C	SPBC19C7.08c	0.7211	1.464	0.142004504	34.38	54.07	5.065	2.388
SPBC18H10.13	rps1402	0.6023	1.465	0.220187137	28.05	43.67	3.968	0.9199
SPAC5H10.07	SPAC5H10.07	0.6518	1.469	0.185885644	32.83	51.51	3.719	2.109
SPBC947.05C	frp2	0.7572	1.471	0.120789395	29.86	46.63	6.386	2.255
SPCC1393.03	rps1501	0.7132	1.473	0.146788665	34.08	53.56	5.736	1.271
SPAC17G8.13C	mst2	0.7101	1.477	0.148680487	34.85	54.82	5.264	2.19
SPAC1834.10C	SPAC1834.10c	0.7404	1.479	0.13053359	28.4	44.22	5.185	2.862
SPAC644.07	SPAC644.07	0.7876	1.48	0.103694293	6.846	8.815	5.788	4.071
SPAC1783.01	SPAC1783.01	0.7475	1.482	0.126388803	34.03	53.45	5.586	2.774
SPCC962.04	rps1201	0.8737	1.483	0.058637664	19.17	29.05	10	6.541
SPCC1494.09C	SPCC1494.09c	0.7954	1.483	0.099414413	27.93	43.44	7.112	3.318
SPAC23H3.15C	SPAC23H3.15c	0.8937	1.484	0.048808242	24.01	37	13.3	6.889
SPBC839.14C	SPBC839.14c	0.717	1.484	0.144480844	28.25	43.96	4.941	2.48
SPCC1183.04C	pet127	0.5925	1.485	0.227311645	23.11	35.53	3.366	1.632
SPCC1259.07	rxt3	0.8901	1.488	0.050561199	19.31	29.27	11.64	7.502
SPAC23H3.13C	gpa2	0.8255	1.489	0.083282922	16.25	24.25	8.202	4.089
SPCC1183.09C	pmp31	0.6655	1.489	0.17685194	32.3	50.61	4.525	1.715
SPBC19C7.02	ubr1	0.8094	1.491	0.0918368	23.59	36.3	7.636	3.632
SPCC4B3.03C	SPCC4B3.03c	0.9028	1.491	0.04440845	23.84	36.71	13.48	8.348
SPAC1805.07C	dad2	0.8118	1.492	0.090550953	19.5	29.58	6.237	4.629
SPBC32F12.07C	SPBC32F12.07(	0.6758	1.499	0.170181813	29.61	46.17	4.626	1.881

SPCC1739.04C	SPCC1739.04c	0.8241	1.499	0.084020086	25.24	39	6.212	5.201
SPBP4G3.03	SPBP4G3.03	0.6699	1.505	0.173990022	33.64	52.78	5.027	1.143
SPCC584.15C	SPCC584.15c	0.8319	1.511	0.079928876	34.68	54.49	9.879	2.969
SPAC13C5.05C	SPAC13C5.05c	0.7292	1.512	0.13715334	31.74	49.66	5.682	2.268
SPAC637.03	SPAC637.03	0.8511	1.513	0.070019409	37.39	58.94	11.8	2.11
SPBC18E5.05C	iki1	0.6581	1.513	0.181708109	4.661	5.17	3.007	2.788
SPAC3H1.05	SPAC3H1.05	0.8829	1.514	0.054088483	22.37	34.25	10.45	7.505
SPAP32A8.03C	SPAP32A8.03c	0.6221	1.519	0.206139799	29.15	45.39	4.063	1.496
SPBC3B8.03	SPBC3B8.03	0.5419	1.52	0.266080849	32.81	51.4	2.565	1.756
SPCC1235.03	SPCC1235.03	0.8276	1.522	0.082179518	29.45	45.88	8.06	4.544
SPCC162.10	ppk33	0.516	1.523	0.287350298	30	46.78	3.336	0.5718
SPAC2E1P3.01	SPAC2E1P3.01	0.7442	1.523	0.128310334	28.4	44.15	5.925	2.583
SPBC1711.15C	SPBC1711.15c	0.8059	1.524	0.093718844	32.87	51.5	8.477	2.762
SPAC4G8.03C	SPAC4G8.03c	0.6357	1.527	0.196747789	35.71	56.14	4.539	1.113
SPAC3G9.01	nsk1	0.834	1.527	0.078833949	28.13	43.69	7.668	5.18
SPAC1296.04	mug65	0.8144	1.528	0.089162235	29.68	46.23	8.376	3.522
SPCC330.06C	SPCC330.06c	0.4272	1.529	0.369368756	1.947	0.6862	0.6862	1.646
SPAC222.07C	hri2	0.702	1.535	0.153662888	27.87	43.25	5.309	1.966
SPBC1289.08	uap1	0.7054	1.536	0.151564545	26	40.19	3.313	3.264
SPBC32H8.05	SPBC32H8.05	0.7351	1.539	0.133653577	30.36	47.34	5.968	2.306
SPAC4A8.14	SPAC4A8.14	0.8794	1.539	0.055813539	25.01	38.55	10.66	7.233
SPBC336.06C	rnh1	0.6738	1.54	0.171468993	31.82	49.74	4.895	1.717
SPCC285.05	SPCC285.05	0.7826	1.54	0.106460156	32.03	50.08	7.689	2.393
SPCC1739.05	set5	0.8265	1.544	0.082757142	14.88	21.9	7.411	5.009
SPCC569.04	SPCC569.04	0.6269	1.545	0.20280173	32.42	50.72	4.65	0.5483
SPCC548.07C	ght1	0.6798	1.546	0.16761884	24.24	37.28	3.873	2.672
SPAC328.02	SPAC328.02	0.5924	1.549	0.22738495	35.08	55.07	3.928	1.233
SPBC3F6.05	rga1	0.6848	1.553	0.164436248	35.48	55.72	4.504	2.394
SPAC2F3.18C	SPAC2F3.18c	0.4853	1.553	0.313989709	32.45	50.75	2.519	1.418
SPAPB1A10.03	nxt1	0.7451	1.557	0.127785437	30.1	46.88	6.338	2.375
SPBC16A3.17C	SPBC16A3.17c	0.6557	1.562	0.183294816	23.72	36.4	4.443	1.915
SPAC644.09	SPAC644.09	0.7191	1.563	0.143210711	33.05	51.72	5.7	2.199
SPAC3F10.11C	abc2	0.7196	1.566	0.142908845	32.21	50.33	5.959	1.91
SPAC186.06	SPAC186.06	0.7083	1.568	0.149782758	33.89	53.09	4.984	2.593
SPAC589.03C	SPAC589.03c	0.7164	1.573	0.144844423	34.04	53.34	5.769	2.088
SPCC320.14	SPCC320.14	0.7494	1.573	0.125286311	30.26	47.12	6.191	2.783
SPBC337.10C	SPBC337.10c	0.5858	1.575	0.232250633	26.48	40.9	2.392	2.285
SPAC11E3.08C	nse6	0.7883	1.577	0.103308473	22.29	34.03	5.273	4.574
SPAC1834.08	mak1	0.4994	1.579	0.301551462	30.69	47.81	2.965	1.219
SPAC977.17	SPAC977.17	0.8625	1.58	0.064240896	30.61	47.69	9.905	6.321
SPCC11E10.05C	ynd1	0.6749	1.581	0.170760572	32.42	50.65	4.832	2.012
SPBC646.17C	dic1	0.7631	1.584	0.117418546	31.97	49.91	6.753	2.828
SPAC22F8.09	rrp16	0.6535	1.585	0.184754408	28.38	44.01	4.529	1.876
SPBC800.08	gcd10	0.7059	1.586	0.151256818	32.45	50.7	5.933	1.507
SPBC1773.17C	SPBC1773.17c	0.6966	1.587	0.15701653	34.32	53.76	5.507	1.843
SPAC23D3.09	arp42	0.8412	1.597	0.075100736	19.56	29.51	9.809	4.734
SPAC19D5.06C	din1	0.7975	1.599	0.098269308	31.65	49.35	7.132	4.086
SPCC417.11C	SPCC417.11c	0.814	1.602	0.089375595	29.44	45.72	8.184	4.191
SPBC1D7.03	mug80	0.7934	1.603	0.100507804	19.79	29.87	6.475	4.325
SPBP4H10.08	qcr10	0.7188	1.604	0.143391932	32.67	51.04	5.526	2.572
SPBC646.02	cwf11	0.6606	1.613	0.180061431	29.44	45.7	3.591	2.716
SPAC227.06	SPAC227.06	0.7518	1.615	0.123897679	30.12	46.81	6.024	3.212
SPCC1620.08	SPCC1620.08	0.82	1.616	0.086186148	35.8	56.15	9.933	2.832
SPBC146.04	SPBC146.04	0.6241	1.622	0.204745817	35.66	55.91	4.352	1.625
SPCC825.04C	naa40	0.6879	1.623	0.162474691	27.28	42.14	4.754	2.529
SPAC1F5.03C	SPAC1F5.03c	0.6484	1.624	0.188156994	34.67	54.29	4.97	1.333
SPCC338.10C	cox5	0.774	1.624	0.111259039	11.61	16.4	5.786	4.097
SPBC21B10.05C	pop3	0.6688	1.625	0.174703736	32.78	51.18	5.109	1.745
SPCC162.11C	SPCC162.11c	0.6651	1.628	0.177113052	38.15	59.99	4.167	2.521
SPAC26A3.14C	SPAC26A3.14c	0.6579	1.633	0.181840114	30.41	47.27	4.267	2.356
SPBC32H8.06	mug93	0.773	1.636	0.111820506	29.81	46.28	7.512	2.816
SPAC25H1.07	emc1	0.863	1.637	0.063989204	18.99	28.5	10.36	6.551
SPBC1105.14	rsv2	0.6292	1.645	0.201211286	32.69	51	4.389	1.776
SPAC23H4.01C	SPAC23H4.01c	0.5823	1.646	0.23485321	30.8	47.89	3.292	2.002
SPAC17A5.18C	rec25	0.6492	1.647	0.187621489	31.13	48.43	3.75	2.573
SPAC630.10	SPAC630.10	0.4703	1.651	0.327625021	20.32	30.67	2.22	1.656
SPCC965.10	SPCC965.10	0.6623	1.662	0.178945245	34.19	53.43	4.724	2.175
SPAC23C4.16C	atg15	0.5237	1.667	0.280917426	32.78	51.1	3.405	1.274
SPAC589.10C	SPAC589.10c	0.8698	1.669	0.060580597	21.86	33.16	11.62	6.716

SPCC584.03C	SPCC584.03c	0.7577	1.675	0.120502713	26.46	40.71	4.053	4.486
SPCC5E4.07	rpl2802	0.5335	1.677	0.272865576	3.538	3.058	2.15	2.139
SPAC32A11.02C	SPAC32A11.02c	0.683	1.678	0.165579296	27.81	42.93	4.953	2.484
SPBP35G2.11C	SPBP35G2.11c	0.6074	1.679	0.216525212	21.56	32.66	3.573	2.206
SPAC3H1.13	ppk13	0.7671	1.683	0.115148017	31.84	49.53	7.689	2.616
SPAC14C4.06C	SPAC14C4.06c	0.794	1.684	0.100179498	18.72	27.98	7.268	4.299
SPBC16H5.05C	cyp7	0.6868	1.686	0.163169714	30.02	46.54	4.273	3.009
SPAC57A10.12C	ura3	0.8547	1.689	0.068186296	17.5	25.98	9.917	6.75
SPAPB17E12.08	SPAPB17E12.08	0.7005	1.695	0.15459186	22.41	34.02	3.039	3.691
SPAC22G7.11C	SPAC22G7.11c	0.8796	1.706	0.055714779	22.57	34.28	12	7.941
SPBC365.03C	rpl2101	0.6338	1.707	0.198047765	6.083	7.187	3.22	2.894
SPAC31F12.01	zds1	0.8356	1.71	0.078001569	9.525	12.84	8.459	6.231
SPAC8F11.05C	mug130	0.7688	1.717	0.114186625	32.3	50.24	7.515	3.144
SPCC1919.15	brl1	0.8129	1.719	0.089962876	14.52	21.02	7.935	4.997
SPAC6G9.15C	SPAC6G9.15c	0.8235	1.722	0.084336396	33.75	52.6	11.16	2.392
SPBP8B7.24C	atg8	0.7164	1.724	0.144844423	31.81	49.43	5.424	3.094
SPAC11E3.01C	swr1	0.5127	1.725	0.290136683	34.63	54.05	2.785	1.809
SPAC806.08C	mod21	0.7623	1.726	0.11787408	30.99	48.07	7.78	2.547
SPCC63.14	SPCC63.14	0.7119	1.727	0.147581007	33.29	51.84	6.017	2.493
SPBC1198.09	ubc16	0.6821	1.73	0.16615195	31.86	49.49	5.095	2.549
SPBC1539.06	SPBC1539.06	0.7286	1.734	0.137510833	23.37	35.54	5.144	3.591
SPBC15D4.05	SPBC15D4.05	0.5475	1.735	0.261615876	32.41	50.38	3.906	1.207
SPAC5D6.01	rps2202	0.6728	1.738	0.172114017	28.57	44.07	5.013	2.44
SPAC27D7.11C	SPAC27D7.11c	0.831	1.738	0.080398976	25.51	39.05	9.033	5.528
SPCC1259.04	iec3	0.7485	1.739	0.125808195	33.19	51.67	7.076	2.802
SPCC1393.13	SPCC1393.13	0.8891	1.741	0.05104939	20.43	30.69	13.89	8.474
SPAC7D4.05	SPAC7D4.05	0.5732	1.742	0.241693818	33.6	52.33	4.12	1.419
SPAC26H5.08C	bgl2	0.8837	1.742	0.053695145	24.1	36.72	13.09	8.18
SPAC1F7.13C	rpl801	0.4491	1.743	0.347656945	4.658	4.788	2.007	1.863
SPAC20H4.04	fml2	0.6323	1.747	0.199076818	34.26	53.4	4.821	1.771
SPBC947.08C	hip4	0.5635	1.751	0.24910608	29.52	45.62	3.918	1.537
SPBC31F10.16	SPBC31F10.16	0.5753	1.756	0.240105626	28.27	43.55	2.666	2.45
SPAC227.07C	pab1	0.5931	1.757	0.226872076	11.64	16.24	3.31	2.497
SPAC17G8.14C	pck1	0.6246	1.758	0.20439802	30.91	47.89	4.792	1.676
SPAC1556.06.1	meu1-1	0.7239	1.759	0.140321423	29.33	45.29	6.109	2.937
SPAC23H3.14	SPAC23H3.14	0.5798	1.76	0.236721789	31.52	48.88	4.545	0.8514
SPBC3B8.07C	dsd1	0.578	1.762	0.238072162	28.95	44.66	3.862	1.842
SPAC6G9.08	ubp6	0.5252	1.762	0.279675283	32.41	50.35	3.17	1.759
SPAC17D4.04	SPAC17D4.04	0.6599	1.765	0.180521872	28.83	44.46	5.346	1.901
SPAC22H10.08	SPAC22H10.08	0.646	1.774	0.189767482	30.15	46.61	4.883	2.118
SPAC11D3.09	SPAC11D3.09	0.7188	1.778	0.143391932	34.32	53.45	4.826	3.666
SPAC12B10.09	SPAC12B10.09	0.689	1.782	0.161780778	25.61	39.14	5.294	2.749
SPAC1D4.06C	csk1	0.861	1.782	0.064996849	25.77	39.4	9.719	7.714
SPAC2E12.03C	SPAC2E12.03c	0.6814	1.782	0.166597871	34.06	53.02	5.224	2.629
SPCC191.10	SPCC191.10	0.6711	1.786	0.173212761	35.59	55.52	6.063	1.169
SPAC17A2.05	osm1	0.639	1.787	0.194499142	26.88	41.22	2.917	3.099
SPAC688.14	set13	0.4646	1.787	0.332920795	27.54	42.3	2.857	1.483
SPAPYUK71.03C	syn1	0.8366	1.788	0.07748214	27.45	42.16	8.417	6.507
SPAC167.07C	SPAC167.07c	0.5009	1.789	0.300248968	17.08	25.12	2.384	2.016
SPCC417.09C	SPCC417.09c	0.6208	1.79	0.207048292	34.96	54.48	4.366	2.16
SPBC16H5.08C	SPBC16H5.08c	0.7703	1.791	0.113340102	27.41	42.09	8.369	2.729
SPBC3B8.04C	SPBC3B8.04c	0.5889	1.792	0.229958446	34.19	53.22	4.497	1.413
SPAC1F3.03	SPAC1F3.03	0.6909	1.792	0.160584807	25.13	38.33	2.628	3.871
SPAC24C9.05C	mug70	0.8568	1.792	0.067120542	20.54	30.79	10.63	6.97
SPAC16C9.04C	mot2	0.7908	1.799	0.101933339	23.23	35.2	8.055	4.236
SPBC409.17C	SPBC409.17c	0.7725	1.804	0.112101512	34.55	53.79	8.891	2.147
SPAC18G6.10	lem2	0.5478	1.804	0.261377972	30.68	47.43	3.118	2.101
SPCC1259.02C	SPCC1259.02c	0.6453	1.809	0.190238335	33.63	52.26	4.665	2.418
SPBC1703.03C	SPBC1703.03c	0.7721	1.813	0.112326448	22.67	34.26	7.344	3.969
SPAC222.15	meu13	0.6855	1.814	0.163992541	30.61	47.3	5.642	2.498
SPAC3H1.07	aru1	0.6882	1.815	0.162285332	32.07	49.7	6.259	1.828
SPCC297.03	ssp1	0.4914	1.82	0.308564848	32.47	50.35	3.741	0.6679
SPCC18.17C	SPCC18.17c	0.6648	1.822	0.177308989	35.15	54.74	4.873	2.68
SPAC13A11.01C	rga8	0.8087	1.822	0.092212557	32.32	50.1	7.501	5.556
SPBC1105.13C	SPBC1105.13c	0.5874	1.827	0.231066058	30.75	47.51	4.078	1.985
SPCC70.04C	SPCC70.04c	0.4884	1.829	0.311224345	34.06	52.95	1.567	2.206
SPBC646.06C	agn2	0.7226	1.829	0.141102043	36.3	56.62	7.228	1.944
SPCC576.12C	mhf2	0.2401	1.834	0.61960784	26.96	41.28	1.629	0.9981
SPAC23D3.11	ayr1	0.5128	1.836	0.290051983	33.29	51.67	3.922	0.889

SPBC577.14C	spa1	0.7149	1.837	0.145754703	29.65	45.68	6.862	2.202
SPCC645.12C	SPCC645.12c	0.6502	1.84	0.186953035	32.57	50.47	4.554	2.68
SPAC1D4.05C	SPAC1D4.05c	0.6537	1.843	0.184621515	30.91	47.75	5.492	1.918
SPCC777.12C	SPCC777.12c	0.6546	1.846	0.184023999	29.24	45	5.467	1.99
SPAC8E11.07C	alp31	0.8449	1.846	0.07319469	21.68	32.59	8.099	7.505
SPAC31A2.06	atp25	0.4781	1.851	0.320481256	33.69	52.3	3.396	1.243
SPBC337.15C	coq7	0.7515	1.851	0.124071015	8.891	11.56	7.622	3.033
SPBC1685.01	pmp1	0.6973	1.852	0.156580335	33.8	52.48	6.524	2.034
SPBC2A9.13	SPBC2A9.13	0.7914	1.856	0.101603954	36.45	56.83	9.316	3.47
SPAC212.02	SPAC212.02	0.6405	1.856	0.193480866	32.27	49.96	5.412	1.71
SPBC839.04	rpl803	0.6165	1.857	0.210066919	29.65	45.65	4.337	2.319
SPBC215.11C	SPBC215.11c	0.6143	1.858	0.211619485	33.97	52.75	5.146	1.345
SPCC23B6.02C	SPCC23B6.02c	0.5857	1.859	0.232324776	33.84	52.54	4.91	0.8121
SPAC23A1.16C	rtr1	0.6269	1.863	0.20280173	33.84	52.52	5.178	1.698
SPBC20F10.05	nrl1	0.565	1.867	0.247951552	33.94	52.69	3.874	1.949
SPCC594.04C	SPCC594.04c	0.8761	1.868	0.05744632	24.93	37.88	12.9	8.379
SPBC16C6.03C	SPBC16C6.03c	0.7196	1.874	0.142908845	24.16	36.6	6.527	2.97
SPAC222.16C	csn3	0.6864	1.878	0.163422725	33.7	52.27	6.503	1.762
SPBC428.14	SPBC428.14	0.6387	1.881	0.194703084	35.14	54.63	4.744	2.47
SPAC1B3.03C	wis2	0.5782	1.882	0.237921913	31.58	48.79	4.513	1.553
SPAC13A11.03	mcp7	0.6868	1.885	0.163169714	32.66	50.55	6.145	2.335
SPBC12C2.01C	SPBC12C2.01c	0.6547	1.885	0.183957659	15.36	22.14	6.021	1.292
SPBP4H10.10	SPBP4H10.10	0.6564	1.887	0.182831428	34.95	54.31	5.495	2.194
SPAP27G11.02	SPAP27G11.02	0.5499	1.888	0.25971628	30.75	47.42	3.724	1.93
SPAC9E9.13	wos2	0.6079	1.89	0.216167857	31.94	49.36	5.251	1.085
SPBC530.09C	SPBC530.09c	0.6698	1.892	0.174054857	31.95	49.37	4.904	2.985
SPCC1450.12	SPCC1450.12	0.8849	1.893	0.053105805	26.7	40.74	14.24	9.046
SPAC1A6.08C	mug125	0.8539	1.895	0.068592986	24.26	36.74	12.2	6.461
SPAC1071.08	rpp203	0.8511	1.895	0.070019409	23.77	35.93	11.84	6.436
SPAC17C9.10	stm1	0.5773	1.899	0.238598443	32.67	50.54	4.687	1.333
SPAC6B12.15	cpc2	0.8393	1.899	0.076082777	15.81	22.85	10.12	6.518
SPAC3G9.03	rpl2301	0.7749	1.899	0.110754339	10.39	13.94	5.448	5.347
SPBC4C3.06	syp1	0.4637	1.9	0.333762904	34.38	53.35	3.28	1.339
SPAP8A3.02C	ofd2	0.8089	1.9	0.092105165	27.52	42.08	9.393	4.861
SPAC1B3.10C	SPAC1B3.10c	0.4709	1.9	0.32707131	33.77	52.35	2.932	1.707
SPAC1A6.01C	SPAC1A6.01c	0.6931	1.904	0.159204101	30.34	46.71	6.156	2.613
SPBC1683.09C	frp1	0.4002	1.904	0.397722916	17.8	26.11	2.029	1.684
SPAC23C11.15	pst2	0.6286	1.904	0.201625623	32.87	50.86	3.297	3.114
SPCPB16A4.06C	SPCPB16A4.06	0.6608	1.915	0.179929966	31.37	48.39	5.096	2.753
SPBC16D10.07C	sir2	0.6481	1.917	0.188357979	35.67	55.44	4.527	3.048
SPAC20G4.03C	hri1	0.6718	1.922	0.17276	33.59	52.01	6.202	1.943
SPCC338.04	cid2	0.6567	1.925	0.182632984	33.01	51.05	5.1	2.698
SPBC1734.06	rhp18	0.8756	1.925	0.057694247	16.67	24.22	13.3	8.562
SPAC16C9.06C	upf1	0.7275	1.926	0.138167002	29.78	45.76	4.369	4.488
SPBC21C3.07C	SPBC21C3.07c	0.6247	1.933	0.204328494	31.56	48.67	4.54	2.532
SPCC18B5.10C	SPCC18B5.10c	0.7955	1.936	0.099359816	23.39	35.24	7.093	5.662
SPAC688.10	rev3	0.6507	1.936	0.186619193	31.1	47.9	5.152	2.572
SPBP4H10.19C	SPBP4H10.19c	0.6466	1.939	0.1893643	32.22	49.74	4.544	2.914
SPAC589.11	mug82	0.4362	1.946	0.360314339	24.49	37.03	3.225	1.195
SPAC16A10.05C	dad1	0.6654	1.956	0.176917203	10.1	13.38	4.281	3.569
SPAPB8E5.10	SPAPB8E5.10	0.4636	1.957	0.333856573	29.74	45.64	3.586	1.09
SPBP4H10.05C	spe2	0.6289	1.959	0.201418405	34.59	53.59	4.353	2.788
SPBC800.02	whi5	0.4548	1.964	0.342179544	31.37	48.3	3.296	1.383
SPAC4H3.14C	SPAC4H3.14c	0.8746	1.964	0.058190527	26.86	40.9	12.66	9.081
SPAC25B8.04C	mss51	0.6014	1.965	0.220836576	31.76	48.95	3.429	2.868
SPCC757.12	SPCC757.12	0.5088	1.967	0.293452897	33.45	51.71	3.45	1.85
SPAC1805.14	SPAC1805.14	0.6701	1.969	0.173860382	29.69	45.53	6.076	2.299
SPAC30.01C	sec72	0.6647	1.976	0.177374321	31.56	48.6	5.989	2.27
SPAC22H12.05C	SPAC22H12.05	0.4968	1.984	0.303818413	29.9	45.86	3.5	1.717
SPAC3A11.03	SPAC3A11.03	0.5543	1.985	0.256255121	31.24	48.06	3.63	2.273
SPBC3D6.02	but2	0.5859	1.985	0.232176502	34.32	53.11	4.64	1.937
SPAC3H5.10	rpl3202	0.8163	1.986	0.088150204	13.11	18.28	8.698	6.224
SPAC31G5.03	rps1101	0.233	1.987	0.632644079	3.229	2.041	1.195	1.262
SPCC1322.06	kap113	0.4633	1.987	0.3341377	25.91	39.29	3.446	1.378
SPAC6B12.09	trm10	0.5615	1.988	0.250650239	33.39	51.58	3.04	2.63
SPCC70.08C	SPCC70.08c	0.5535	1.989	0.256882375	29.85	45.76	3.601	2.29
SPAC10F6.04	SPAC10F6.04	0.7217	1.991	0.141643295	31.42	48.35	7.663	2.406
SPAC16E8.08	SPAC16E8.08	0.4806	1.992	0.318216234	26.72	40.61	2.041	2.282
SPAC1071.07C	rps1502	0.7675	1.993	0.114921616	14.19	20.03	6.851	4.897

SPBC1709.11C	png2	0.4139	1.993	0.383104574	30.56	46.93	2.298	1.777
SPBC28F2.07	sfr1	0.4852	1.993	0.314079208	33.22	51.29	3.165	1.864
SPBC947.02	apl2	0.4912	1.998	0.308741642	33.19	51.24	3.771	1.399
SPAC6C3.08	SPAC6C3.08	0.5855	2	0.232473101	31.52	48.49	4.467	2.141
SPAC1093.02	SPAC1093.02	0.695	2.002	0.158015195	33.27	51.36	7.092	2.029
SPBC887.11	pus2	0.6563	2.003	0.182897596	34.06	52.66	6.216	1.811
SPBC557.05	SPBC557.05	0.5799	2.003	0.236646891	32.79	50.58	4.016	2.397
SPAC4D7.11	dsc4	0.7138	2.005	0.146423456	11.49	15.57	5.369	4.046
SPBC2G2.17C	SPBC2G2.17c	0.716	2.005	0.145086978	33.79	52.21	7.243	2.773
SPBP35G2.04C	SPBP35G2.04c	0.8753	2.006	0.057843072	26.01	39.43	13.9	8.859
SPBC32H8.08C	omh5	0.8532	2.012	0.068949153	21.88	32.64	11.6	7.647
SPCC1450.16C	SPCC1450.16c	0.4487	2.012	0.34804393	26.14	39.63	2.563	1.923
SPAC1805.06C	hem2	0.4025	2.013	0.395234115	27.94	42.59	1.209	2.007
SPAPB24D3.03	SPAPB24D3.03	0.6084	2.013	0.215810795	35.05	54.27	4.011	2.807
SPBC23G7.13C	SPBC23G7.13c	0.6975	2.013	0.156455788	29.95	45.88	6.628	2.789
SPAC1006.03C	red1	0.7956	2.014	0.099305226	16.07	23.08	7.919	5.65
SPAC8C9.10C	SPAC8C9.10c	0.8645	2.014	0.063235002	24.51	36.96	12.49	8.363
SPAC56F8.06C	alg10	0.4269	2.015	0.369673845	26.99	41.02	3.33	1.124
SPAC2G11.10C	SPAC2G11.10c	0.5798	2.016	0.236721789	31.98	49.22	4.268	2.256
SPBC16A3.10	SPBC16A3.10	0.5298	2.017	0.275888046	30.14	46.2	3.041	2.38
SPAC31G5.19	abo1	0.7831	2.017	0.106182776	22.51	33.66	9.566	3.805
SPCC1739.10	mug33	0.7841	2.023	0.105628546	24.83	37.46	9.19	4.27
SPAC926.03	rlc1	0.6547	2.026	0.183957659	27.82	42.37	5.618	2.592
SPAC1F8.02C	SPAC1F8.02c	0.6472	2.027	0.188961491	17.93	26.12	6.293	1.429
SPBC19C2.10	SPBC19C2.10	0.5141	2.029	0.288952396	31.65	48.66	4.017	1.547
SPAPB1E7.06C	eme1	0.707	2.03	0.150580586	10.99	14.73	5.185	4.048
SPBC3E7.11C	SPBC3E7.11c	0.5641	2.032	0.2486439	33.2	51.19	4.1	2.201
SPBC1A4.03C	top2	0.6872	2.034	0.162916849	31.99	49.21	6.458	2.721
SPBP16F5.05C	SPBP16F5.05c	0.7449	2.039	0.127902026	18.14	26.45	6.812	4.311
SPCC1322.07C	mug150	0.571	2.041	0.243363892	31.45	48.31	3.938	2.42
SPAP8A3.04C	hsp9	0.5237	2.042	0.280917426	24.01	36.09	3.199	2.297
SPAC29A4.20	elp3	0.6324	2.043	0.199008139	27.57	41.93	5.39	2.346
SPAC17G6.15C	SPAC17G6.15c	0.8187	2.046	0.08687521	31.35	48.13	10.37	5.751
SPBC17D11.02C	hrd1	0.6219	2.051	0.206279443	34.54	53.36	5.044	2.464
SPAC806.07	ndk1	0.6174	2.053	0.209433375	34.82	53.82	4.895	2.503
SPBC6B1.10	prp17	0.7788	2.053	0.108574057	33.47	51.61	9.444	3.896
SPAC1F12.04C	SPAC1F12.04c	0.5083	2.055	0.29387989	30.64	46.95	3.931	1.637
SPCC1840.10	lsm8	0.4209	2.064	0.375821074	31.31	48.03	2.991	1.541
SPAC3F10.18C	rpl4102	0.8241	2.065	0.084020086	26.86	40.73	10.88	5.927
SPAC3G6.03C	SPAC3G6.03c	0.587	2.067	0.231361899	31.42	48.22	4.894	1.971
SPCC1322.08	srk1	0.3662	2.068	0.43628166	34.27	52.9	2.857	1.161
SPBC3B8.10C	nem1	0.5501	2.069	0.259558355	31.28	47.97	4.968	0.7873
SPCC830.10	SPCC830.10	0.6136	2.071	0.212114649	30.77	47.14	5.816	1.297
SPAC2H10.01	SPAC2H10.01	0.5906	2.076	0.228706557	32.07	49.27	4.538	2.384
SPBC31E1.01C	atg2	0.4821	2.079	0.316862869	35.4	54.73	3.724	1.561
SPBC16H5.06	rip1	0.5925	2.081	0.227311645	18.25	26.56	4.842	2.178
SPAC17H9.03C	rdl1	0.8358	2.09	0.077897633	20.21	29.77	10.77	7.081
SPCC330.07C	SPCC330.07c	0.6231	2.091	0.205442249	34	52.42	4.767	2.807
SPAC6G10.11C	ubi3	0.5923	2.094	0.227458267	34.2	52.73	4.694	2.345
SPCC1795.03	gms1	0.6834	2.097	0.165325026	32.43	49.82	6.728	2.601
SPAC4G8.07C	SPAC4G8.07c	0.4856	2.099	0.313721322	27.94	42.44	2.749	2.257
SPBC17G9.02C	SPBC17G9.02c	0.6005	2.1	0.221486988	34.59	53.36	5.394	1.792
SPBC17A3.09C	aim22	0.6228	2.101	0.205651396	19.33	28.3	4.762	2.833
SPBC1709.06	dus2	0.5962	2.107	0.224608028	33	50.75	4.797	2.367
SPBC21B10.13C	yox1	0.5444	2.109	0.264081883	16.07	22.93	3.666	2.408
SPAC30C2.05	erv14	0.6366	2.11	0.196133366	31.7	48.6	6.146	1.817
SPBC3F6.01C	SPBC3F6.01c	0.4353	2.114	0.361211333	32.26	49.52	2.024	2.156
SPBC800.03	clr3	0.6963	2.114	0.157203605	32.01	49.11	7.781	1.626
SPBC1604.12	SPBC1604.12	0.6019	2.117	0.220475657	33.78	52.01	5.169	2.164
SPCC16C4.13C	rpl1201	0.7449	2.123	0.127902026	14.18	19.8	6.617	4.742
SPBC26H8.03	cho2	0.8587	2.127	0.066158537	26.77	40.48	14.06	7.59
SPAC823.14	ptf1	0.5312	2.13	0.274741934	35.19	54.3	3.997	2.08
SPBC1198.14C	fbp1	0.4451	2.135	0.351542406	31.17	47.69	3.431	1.544
SPAC22A12.03C	csn4	0.5808	2.136	0.235973392	33.51	51.53	4.797	2.179
SPBP8B7.22	erd2	0.5337	2.136	0.272702797	29.6	45.12	4.029	2.104
SPBC354.12	gpd3	0.848	2.139	0.071604148	21.74	32.2	12.08	7.754
SPBC23E6.05	arx1	0.6763	2.141	0.169860613	32.66	50.13	6.723	2.574
SPBC3H7.08C	SPBC3H7.08c	0.4471	2.145	0.34959533	30.73	46.95	2.825	1.995
SPAC6G9.05	pcd1	0.5742	2.146	0.240936812	32.3	49.52	4.635	2.239

SPBC1271.05C	SPBC1271.05c	0.6464	2.148	0.189498652	23.06	34.35	4.867	3.315
SPBC119.08	pmk1	0.6305	2.148	0.200314909	26.85	40.57	5.743	2.342
SPAC22F8.02C	pvg5	0.3819	2.149	0.418050342	26.31	39.69	2.119	1.852
SPAC18G6.02C	chp1	0.1915	2.154	0.717831222	21.12	31.15	1.518	1.126
SPBC29A10.03C	pcf1	0.5515	2.155	0.258454483	31.96	48.96	4.988	1.365
SPBC947.10	dsc1	0.7469	2.157	0.126737541	14.45	20.2	6.814	4.838
SPBC32F12.08C	duo1	0.7609	2.159	0.118672416	15.23	21.47	7.205	5.159
SPAC1556.05C	cgr1	0.7024	2.16	0.153415497	25.9	38.99	4.827	4.468
SPAC4F10.19C	SPAC4F10.19c	0.3215	2.163	0.492819023	23.35	34.81	2.595	1.209
SPCC965.08C	alr1	0.4985	2.163	0.302334837	28.73	43.64	3.336	2.211
SPBC56F2.03	SPBC56F2.03	0.6233	2.172	0.205302873	34.13	52.5	5.791	2.199
SPBC56F2.02	rpl1901	0.6202	2.177	0.207468238	6.999	7.921	3.577	3.504
SPAC26A3.06	SPAC26A3.06	0.4004	2.184	0.397505931	31.14	47.56	3.563	0.7866
SPAC8C9.19	SPAC8C9.19	0.6809	2.185	0.166916666	22.27	32.99	4.918	4.051
SPAC23C4.07	tht2	0.6571	2.186	0.182368533	31.14	47.56	6.516	2.392
SPAC8C9.04	SPAC8C9.04	0.8401	2.187	0.075669015	23.2	34.52	11.08	7.878
SPAC683.03	SPAC683.03	0.485	2.188	0.314258261	36.75	56.77	3.93	1.672
SPAC14C4.10C	SPAC14C4.10c	0.5683	2.19	0.245422344	33.59	51.57	5.195	1.637
SPBC3B9.13C	rpp102	0.8044	2.192	0.094527938	17.92	25.83	9.339	6.27
SPCC63.06	SPCC63.06	0.8239	2.192	0.084125497	19.92	29.12	10.38	6.997
SPAC23D3.01	SPAC23D3.01	0.5718	2.194	0.242755849	32.22	49.32	4.623	2.344
SPBC18H10.18C	SPBC18H10.18	0.7623	2.2	0.11787408	35.32	54.41	10.25	2.715
SPBPB2B2.06C	SPBPB2B2.06c	0.4591	2.203	0.338092707	31.73	48.5	3.699	1.606
SPAC25B8.07C	SPAC25B8.07c	0.3429	2.205	0.464832515	29.45	44.75	3.211	0.2975
SPBC409.07C	wis1	0.7648	2.208	0.116452121	26.18	39.38	7.734	5.243
SPAC29A4.17C	SPAC29A4.17c	0.6955	2.21	0.157702866	26.28	39.53	7.474	2.766
SPBC32H8.13C	mok12	0.8125	2.211	0.09017663	26.2	39.4	9.881	6.585
SPBC839.13C	rpl1601	0.6363	2.213	0.196338077	23.04	34.21	5.122	3.168
SPBC3H7.03C	SPBC3H7.03c	0.7982	2.213	0.097888277	11.51	15.28	7.921	6.686
SPBC409.10	ade7	0.443	2.216	0.353596274	27.46	41.47	3.958	0.987
SPCC5E4.10C	SPCC5E4.10c	0.6583	2.216	0.181576145	29.65	45.07	5.183	3.567
SPAC17G8.07	yaf9	0.5383	2.217	0.26897562	30.17	45.91	4.527	1.948
SPAC26F1.12C	SPAC26F1.12c	0.6245	2.221	0.204467557	34.82	53.55	5.814	2.399
SPBC29A3.12	rps902	0.812	2.221	0.090443971	23.93	35.66	9.459	6.818
SPAC3G6.11	chl1	0.8446	2.222	0.073348923	26.47	39.83	13.98	6.705
SPBC1A4.02C	leu1	0.7447	2.224	0.128018646	36.39	56.12	9.238	3.134
SPAC31G5.15	psd3	0.8505	2.225	0.070325682	24.19	36.08	12.6	8.3
SPBC405.07	rpl3602	0.8102	2.227	0.091407761	18.63	26.94	9.919	6.504
SPCC23B6.05C	ssb3	0.4975	2.239	0.303206915	29.41	44.63	4.694	0.7536
SPAC17C9.11C	SPAC17C9.11c	0.5569	2.246	0.254222782	35.88	55.24	5.1	1.729
SPBC28F2.03	ppi1	0.3458	2.254	0.461175011	30.8	46.9	2.223	1.726
SPCC4B3.04C	nte1	0.4271	2.254	0.369470429	34.23	52.52	3.19	1.797
SPBC317.01	mbx2	0.3269	2.256	0.485585079	24.07	35.83	1.25	1.87
SPAC144.05	SPAC144.05	0.3927	2.258	0.405939099	28.1	42.45	1.801	2.124
SPAC8E11.04C	SPAC8E11.04c	0.6217	2.26	0.206419133	30.31	46.07	6.107	2.139
SPBC15D4.07C	atg9	0.5266	2.274	0.278519145	32.71	49.99	4.873	1.467
SPAC23C4.17	SPAC23C4.17	0.3913	2.275	0.407490152	34.66	53.2	3.347	1.328
SPCC965.12	SPCC965.12	0.6723	2.276	0.172436889	29.94	45.44	6.543	3.201
SPCC126.06	twf1	0.5325	2.284	0.273680388	25.96	38.88	3.049	2.867
SPBCPT2R1.08C	tlh2	0.8499	2.285	0.070632171	20.17	29.38	12.95	8.451
SPAC821.09	eng1	0.7194	2.286	0.143029567	37.41	57.69	9.261	1.632
SPAC2F7.17	SPAC2F7.17	0.4622	2.286	0.335170059	34.33	52.63	3.738	1.799
SPBC582.04C	SPBC582.04c	0.4974	2.286	0.303294219	27.31	41.11	4.266	1.762
SPBC21H7.04	dbp7	0.2681	2.287	0.571703186	3.223	1.537	1.051	1.824
SPBC83.16C	SPBC83.16c	0.6386	2.288	0.194771086	29.76	45.12	6.404	2.406
SPBC18H10.15	ppk23	0.53	2.29	0.27572413	32.1	48.97	4.574	1.975
SPAC29B12.05C	SPAC29B12.05	0.5867	2.291	0.231583912	13.55	18.5	4.029	3.297
SPAC31A2.15C	dcc1	0.4693	2.292	0.328549446	27.18	40.87	2.174	2.582
SPAC1952.03	otu2	0.8073	2.293	0.092965048	18.97	27.39	9.943	6.649
SPAC227.15	SPAC227.15	0.4877	2.296	0.311847244	35	53.72	4.349	1.525
SPBC3D6.04C	mad1	0.8076	2.311	0.09280369	22.71	33.51	11.43	5.813
SPAC23C11.02C	rps23	0.7965	2.311	0.09881422	21.14	30.94	8.805	6.667
SPBC106.02C	srx1	0.4795	2.312	0.319211388	28.89	43.65	4.612	0.8273
SPBC31E1.02C	pmr1	0.5185	2.312	0.285251239	34.54	52.94	4.498	1.928
SPAC15E1.06	vps29	0.7667	2.313	0.115374537	34.74	53.27	11.31	2.2
SPBC18H10.07	SPBC18H10.07	0.799	2.317	0.097453221	19.02	27.44	9.348	6.574
SPBP35G2.03C	sgo1	0.645	2.321	0.190440285	33.03	50.44	5.781	3.243
SPBC887.04C	lub1	0.5222	2.326	0.282163133	36.29	55.79	5.102	1.121
SPBC557.04	ppk29	0.499	2.328	0.301899454	34.32	52.54	3.96	2.156

SPAC23D3.12	SPAC23D3.12	0.4276	2.328	0.368962303	22.32	32.83	2.43	2.265
SPAC20H4.07	rhp57	0.573	2.329	0.241845378	24.68	36.71	5.211	2.239
SPCC576.13	swc5	0.8232	2.331	0.084494638	24.14	35.82	10.58	7.619
SPCC1827.02C	SPCC1827.02c	0.6909	2.336	0.160584807	12.68	16.98	5.999	4.233
SPBC2F12.05C	SPBC2F12.05c	0.4861	2.339	0.313274379	30.66	46.53	4.695	1.006
SPAC12B10.12C	rhp41	0.5364	2.341	0.270511231	29.59	44.75	3.319	2.913
SPAC4G8.06C	trm12	0.4166	2.343	0.380280734	32.32	49.24	3.562	1.55
SPBC36.06C	spo9	0.5052	2.344	0.296536658	30.44	46.15	4.905	1.131
SPAC24H6.03	cul3	0.6283	2.344	0.20183294	31.84	48.45	5.977	2.781
SPBC13A2.04C	SPBC13A2.04c	0.8149	2.344	0.088895682	26.2	39.18	11.12	6.786
SPAC1687.05	pli1	0.4655	2.346	0.332080315	32.46	49.47	4.045	1.687
SPBC354.05C	sre2	0.6208	2.347	0.207048292	9.03	10.98	4.52	3.724
SPBC30D10.14	SPBC30D10.14	0.4125	2.348	0.384576047	29.27	44.22	2.162	2.265
SPAC26A3.09C	rga2	0.8504	2.349	0.070376748	25.57	38.14	13.35	8.723
SPCC1682.12C	ubp16	0.5426	2.35	0.265520211	32.2	49.03	4.889	2.053
SPBPB8B6.05C	SPBPB8B6.05c	0.5049	2.353	0.296794629	31.77	48.31	4.413	1.911
SPBP4H10.17C	SPBP4H10.17c	0.3864	2.357	0.412962882	31.73	48.25	3.221	1.573
SPAC30C2.04	SPAC30C2.04	0.1553	2.357	0.808828544	22.89	33.73	2.059	0.3509
SPCC895.09C	ucp12	0.6962	2.357	0.157265981	30.36	46	7.03	3.839
SPAC4D7.07C	SPAC4D7.07c	0.5889	2.364	0.229958446	30.14	45.62	5.385	2.504
SPAC6F6.11C	SPAC6F6.11c	0.4679	2.364	0.329846955	32.01	48.69	3.178	2.35
SPAC16E8.01	shd1	0.6568	2.365	0.182566856	20.97	30.56	6.109	3.433
SPCC16A11.15C	SPCC16A11.15c	0.6584	2.369	0.181510178	31.06	47.12	6.485	3.202
SPAC25H1.03	mug66	0.5478	2.374	0.261377972	33.1	50.46	5.303	1.725
SPBC23E6.08	sat1	0.2258	2.374	0.646276062	17.22	24.39	2.165	1.16
SPBC337.07C	SPBC337.07c	0.4985	2.381	0.302334837	28.52	42.93	2.519	2.857
SPAC23H4.09	cdb4	0.5498	2.394	0.259795264	34.34	52.48	5.571	1.401
SPCC1494.07	SPCC1494.07	0.6522	2.394	0.185619206	33.95	51.83	7.062	2.534
SPCC31H12.02C	mug73	0.3536	2.4	0.451487744	30.89	46.79	2.029	1.997
SPBC19F8.03C	SPBC19F8.03c	0.4054	2.403	0.392116256	33.61	51.26	3.919	1.002
SPCC1906.04	wtf20	0.4347	2.405	0.36181036	30.95	46.88	3.292	2.059
SPBC106.07C	SPBC106.07c	0.6385	2.406	0.194839098	9.918	12.34	4.507	3.98
SPCC285.13C	nup60	0.4267	2.41	0.369877357	25.69	38.24	2.448	2.359
SPAC57A10.08C	SPAC57A10.08c	0.4905	2.413	0.309360988	33.77	51.5	4.769	1.342
SPAC11H11.03C	SPAC11H11.03c	0.7165	2.413	0.144783805	24.44	36.18	6.344	5
SPAC29E6.10C	SPAC29E6.10c	0.602	2.413	0.220403509	21.41	31.2	2.99	3.885
SPBC337.13C	gtr1	0.3901	2.415	0.40882405	29.98	45.28	1.852	2.271
SPBC11B10.08	SPBC11B10.08	0.5986	2.416	0.222863287	34.86	53.29	5.447	2.805
SPBC29B5.03C	rpl26	0.5592	2.416	0.252432837	34.39	52.51	4.968	2.461
SPAC6F6.13C	SPAC6F6.13c	0.6212	2.417	0.206768553	35.91	55.01	6.427	2.404
SPAC823.16C	mug179	0.5866	2.418	0.231657941	33.39	50.87	5.775	2.238
SPCC1739.08C	SPCC1739.08c	0.5983	2.418	0.223080997	33.57	51.16	2.751	3.893
SPCC330.03C	SPCC330.03c	0.4672	2.419	0.330497166	33.69	51.36	4.635	0.9912
SPBC32F12.02	rec14	0.5636	2.419	0.249029016	26.62	39.76	5.065	2.466
SPCC1442.17C	ist1	0.4653	2.421	0.332266947	33.38	50.86	4.184	1.725
SPCC594.01	SPCC594.01	0.5681	2.431	0.245575211	29.39	44.28	5.516	2.148
SPAC2E1P3.02C	amt3	0.6578	2.435	0.181906131	32.42	49.26	6.759	3.2
SPCC1682.01	qcr9	0.7657	2.438	0.115941353	10.57	13.35	7.708	6.219
SPBC685.03	SPBC685.03	0.4289	2.438	0.367643954	29.01	43.65	2.624	2.357
SPAC3H5.12C	rpl501	0.7483	2.439	0.125924255	16.29	22.75	7.662	5.549
SPAC1F12.02C	SPAC1F12.02c	0.6111	2.442	0.213887716	27.14	40.57	6.211	2.468
SPBC32F12.05C	cwf12	0.7654	2.442	0.116111542	17.04	23.99	8.616	5.793
SPAC13G7.13C	msa1	0.8339	2.442	0.078886026	25.65	38.12	13.24	7.706
SPAC11G7.03	idh1	0.4585	2.447	0.33866066	29.96	45.2	2.475	2.635
SPAC8C9.17C	spc34	0.8331	2.449	0.079302866	25.22	37.4	11.8	8.478
SPBC725.05C	SPBC725.05c	0.4411	2.452	0.355462942	31.77	48.15	3.775	1.874
SPBC1861.07	SPBC1861.07	0.5402	2.454	0.26744542	30.8	46.57	4.924	2.275
SPBC1289.14	SPBC1289.14	0.6287	2.456	0.20155654	27.98	41.92	4.1	4.064
SPCC1739.03	hrr1	0.5616	2.456	0.250572901	27.53	41.18	3.24	3.413
SPAPJ698.02C	rps002	0.5598	2.456	0.251967106	9.752	11.98	4.094	3.089
SPAC26H5.05	SPAC26H5.05	0.783	2.457	0.106238238	18.74	26.75	8.321	6.819
SPAC977.15	SPAC977.15	0.6467	2.459	0.189297139	28.19	42.26	6.385	3.288
SPAC10F6.08C	nht1	0.4418	2.46	0.354774288	30.93	46.76	3.769	1.903
SPBC11C11.10	SPBC11C11.10	0.6323	2.461	0.199076818	20.79	30.11	4.999	3.803
SPAC24H6.10C	SPAC24H6.10c	0.5354	2.463	0.271321633	31.53	47.74	4.815	2.315
SPBC16G5.05C	SPBC16G5.05c	0.6558	2.464	0.183228588	25.99	38.65	4.767	4.336
SPCC550.14	vgl1	0.5722	2.464	0.242452147	24.78	36.65	4.755	2.94
SPBC14C8.15	SPBC14C8.15	0.4066	2.469	0.390832626	29.8	44.89	3.342	1.873
SPAP14E8.02	SPAP14E8.02	0.518	2.469	0.28567024	34.4	52.44	5.012	1.806

SPCC794.02	wtf5	0.8507	2.47	0.070223567	27.12	40.49	14.1	9.175
SPBC660.08	SPBC660.08	0.4924	2.47	0.307681956	36.66	56.15	4.909	1.373
SPBC800.05C	atb2	0.5522	2.471	0.257903598	27.34	40.85	3.776	3.155
SPBC1734.04	anp1	0.5565	2.471	0.254534831	18.03	25.55	3.986	3.296
SPAC631.02	SPAC631.02	0.7945	2.473	0.099906098	20.23	29.16	9.869	6.804
SPAC750.06C	SPAC750.06c	0.499	2.477	0.301899454	30.92	46.71	5.2	0.8815
SPAC6B12.12	tom70	0.5974	2.477	0.223734782	26.56	39.56	5.047	3.208
SPAC3A12.06C	SPAC3A12.06c	0.615	2.479	0.211124884	33.73	51.33	6.254	2.664
SPBC14C8.09C	SPBC14C8.09c	0.4956	2.483	0.304868702	34.87	53.2	5.097	1.105
SPBC1709.01	chs2	0.4789	2.489	0.319755163	26.69	39.76	2.762	2.782
SPCC1235.09	hif2	0.7115	2.49	0.147825096	30.89	46.64	6.57	5.002
SPCC777.15	SPCC777.15	0.6178	2.492	0.209152096	29.79	44.85	6.041	2.968
SPCC613.12C	raf1	0.7823	2.494	0.10662667	27.14	40.49	10.27	5.944
SPAC19E9.02	fin1	0.6276	2.495	0.202317065	28.61	42.89	5.611	3.488
SPBC530.14C	dsk1	0.5424	2.496	0.265680319	28.06	41.99	4.186	2.904
SPBC25H2.15	SPBC25H2.15	0.5572	2.497	0.253988892	9.107	10.86	3.717	3.285
SPBC8D2.12C	SPBC8D2.12c	0.2769	2.497	0.557677044	33.25	50.52	2.762	1.201
SPBC3D6.06C	prs5	0.6604	2.499	0.180192935	29.29	44.01	6.407	3.747
SPAC25B8.06C	SPAC25B8.06c	0.7084	2.499	0.149721447	8.447	9.77	6.412	5.242
SPBC887.15C	sur2	0.7976	2.501	0.098214855	20.65	29.82	10.15	6.974
SPAC12B10.05	icp55	0.5886	2.501	0.230179742	35.39	54.03	6.261	1.99
SPAC20G8.10C	atg6	0.4018	2.503	0.395990068	30.7	46.32	3.444	1.812
SPCC364.05	vps3	0.7872	2.504	0.103914915	21.24	30.77	10.48	6.161
SPAC1F7.12	yak3	0.5504	2.505	0.259321575	32.45	49.19	3.945	3.13
SPBC3E7.12C	chr1	0.6016	2.516	0.220692172	30.58	46.1	6.592	1.984
SPAC11D3.10	SPAC11D3.10	0.4264	2.516	0.370182804	33.91	51.57	3.662	1.925
SPAC17G6.03	SPAC17G6.03	0.4291	2.518	0.367441485	28.97	43.44	2.279	2.555
SPCC550.08	SPCC550.08	0.4046	2.527	0.392974122	31.78	48.05	3.897	1.439
SPAC144.01	SPAC144.01	0.7847	2.528	0.105296347	40.95	63.11	12.5	4.305
SPBC4B4.11	SPBC4B4.11	0.488	2.528	0.311580178	32.12	48.6	4.925	1.474
SPBC16G5.06	SPBC16G5.06	0.534	2.529	0.272458743	30.91	46.61	5.749	1.167
SPBC216.02	mcp5	0.4319	2.529	0.364616796	32.12	48.61	4.023	1.691
SPAC13G6.14	aps1	0.7334	2.529	0.134659094	15.87	21.91	7.393	5.458
SPBC24C6.10C	dip1	0.4213	2.531	0.375408541	35.39	53.97	4.303	1.051
SPBC27.06C	mgr2	0.4195	2.54	0.377268035	8.886	10.42	3.722	1.846
SPAC29A4.16	hal4	0.7587	2.543	0.119929916	33.91	51.52	11.1	3.944
SPCC162.05	coq3	0.7257	2.544	0.139242877	9.905	12.09	6.1	5.769
SPAC823.09C	SPAC823.09c	0.6939	2.547	0.158703113	30.93	46.62	7.68	4.008
SPBC1718.06	msp1	0.6407	2.549	0.193345276	27	40.16	3.875	4.523
SPAC1F8.06	fta5	0.3634	2.549	0.439615077	35.28	53.77	3.494	1.428
SPBC776.04	sec2302	0.5363	2.552	0.270592203	29.04	43.51	3.249	3.292
SPBC582.08	SPBC582.08	0.5946	2.553	0.225775095	36.36	55.53	6.565	1.961
SPAC630.15	mug177	0.4566	2.555	0.340464093	28.45	42.54	2.787	2.68
SPCC1450.05C	rox3	0.5718	2.564	0.242755849	30.59	46.04	5.897	2.261
SPAC1002.02	pom34	0.5794	2.564	0.237021509	30.44	45.78	5.964	2.372
SPAC25B8.17	SPAC25B8.17	0.5105	2.565	0.292004254	34.78	52.91	5.439	1.255
SPAC11D3.02C	SPAC11D3.02c	0.6006	2.572	0.221414672	33.56	50.91	7.048	1.301
SPAC30.03C	tsn1	0.5132	2.576	0.289713352	27.24	40.51	3.846	2.863
SPBC1709.04C	cyp3	0.5648	2.58	0.248105312	30.44	45.77	5.693	2.38
SPAC869.04	SPAC869.04	0.5055	2.581	0.29627884	32.26	48.75	2.505	3.212
SPBC902.04	SPBC902.04	0.3885	2.587	0.410608977	28.87	43.18	1.893	2.438
SPAC19G12.06C	hta2	0.534	2.587	0.272458743	34.42	52.29	5.565	1.832
SPAC13G7.05	are1	0.738	2.59	0.131943638	28.57	42.68	8.625	5.203
SPAC22G7.05	SPAC22G7.05	0.6202	2.597	0.207468238	16.93	23.54	5.313	3.97
SPAC227.03C	SPAC227.03c	0.4641	2.598	0.333388432	35.66	54.31	4.852	1.283
SPAC30D11.09	cwf19	0.4995	2.598	0.301464507	24.03	35.2	4.003	2.67
SPBC36B7.02	SPBC36B7.02	0.3112	2.6	0.506960412	29.39	44	2.957	1.502
SPCC613.01	SPCC613.01	0.4762	2.601	0.322210609	30.83	46.36	4.585	1.935
SPBPB10D8.02C	SPBPB10D8.02	0.5137	2.604	0.289290434	29.78	44.65	5.061	2.086
SPBC3D6.13C	pdi2	0.4929	2.606	0.307241182	32.9	49.75	5.298	1.198
SPAC521.04C	SPAC521.04c	0.5427	2.608	0.265440178	32.7	49.44	5.781	1.824
SPAC630.11	vps55	0.5175	2.608	0.286089646	34.44	52.29	4.987	2.241
SPAC23C4.02	crn1	0.5523	2.616	0.257824957	31.88	48.07	5.452	2.467
SPBC713.05	SPBC713.05	0.4813	2.619	0.317584138	31.14	46.85	4.985	1.561
SPAC8C9.16C	mug63	0.8388	2.619	0.076341578	26.24	38.8	13.37	9.246
SPBC146.11C	mug97	0.2935	2.622	0.532391894	31.51	47.45	1.259	2.017
SPBC336.03	efc25	0.4166	2.623	0.380280734	32.39	48.9	3.988	1.734
SPBPB2B2.01	SPBPB2B2.01	0.8336	2.624	0.079042294	20.66	29.63	13.75	8.545
SPAC22A12.11	dak1	0.6061	2.625	0.217455716	32	48.25	6.77	2.388

SPAC17C9.05C	pmc3	0.5756	2.626	0.239879215	33.42	50.59	5.647	2.788
SPAC227.13C	isu1	0.5394	2.628	0.268089058	31.54	47.5	4.919	2.707
SPBC18H10.10C	saf4	0.4307	2.63	0.365825128	32.46	49	3.816	2.069
SPAC139.03	SPAC139.03	0.4467	2.631	0.349984048	34.92	53.03	4.359	1.789
SPCC790.02	pep3	0.8499	2.633	0.070632171	18.86	26.65	13.02	10.65
SPBC1271.06C	mug96	0.4373	2.634	0.359220523	28.37	42.27	1.878	2.829
SPCC16C4.11	pef1	0.5565	2.638	0.254534831	26.24	38.77	4.02	3.429
SPAC1565.07C	SPAC1565.07c	0.4333	2.642	0.363211311	31.78	47.86	3.702	2.195
SPCC126.04C	sgf73	0.4158	2.647	0.381115515	30.12	45.12	4.321	1.342
SPBC25D12.05	trm1	0.3305	2.647	0.480828536	28.89	43.1	2.495	1.968
SPAC18B11.02C	SPAC18B11.02c	0.4863	2.651	0.31309573	28.7	42.79	2.58	3.12
SPAC12B10.11	exg2	0.4935	2.653	0.306712843	29.74	44.5	5.037	1.869
SPCC1739.07	cti1	0.5295	2.653	0.276134036	15.17	20.55	3.501	3.309
SPBC1198.06C	SPBC1198.06c	0.737	2.654	0.132532512	18.36	25.81	8.376	5.828
SPCP1E11.06	apl4	0.7809	2.658	0.107404577	20.53	29.35	11.33	5.975
SPAC1142.06	get3	0.1905	2.66	0.72010502	32.58	49.14	2.027	1.312
SPBC354.08C	SPBC354.08c	0.5713	2.66	0.243135776	33.56	50.75	5.984	2.486
SPAC1F8.03C	str3	0.6923	2.662	0.159705668	28.26	42.05	8.163	4.023
SPAC1002.14	itt1	0.4604	2.662	0.336864685	27.75	41.21	3.059	2.777
SPAC6F6.09	eaf6	0.3467	2.666	0.460046158	23.8	34.71	3.302	1.655
SPAC1805.10	SPAC1805.10	0.4586	2.67	0.33856595	32.44	48.91	4.64	1.758
SPBP8B7.25	cyp4	0.5755	2.672	0.239954672	32.49	48.98	5.596	2.951
SPAC8C9.14	prr1	0.8131	2.673	0.089856039	24.03	35.08	11.85	8.056
SPBC947.03C	naa38	0.157	2.674	0.804100348	33.17	50.09	2.256	0.8264
SPCC569.05C	SPCC569.05c	0.5119	2.684	0.29081487	31.69	47.65	5.122	2.216
SPAC19G12.12	dlp1	0.6141	2.685	0.211760903	16.95	23.43	5.41	4.027
SPAC22G7.06C	ura1	0.5784	2.688	0.237771716	31.05	46.59	5.19	3.309
SPAC3C7.09	set8	0.4451	2.69	0.351542406	29.24	43.62	2.932	2.723
SPACUNK4.14	mdb1	0.5107	2.691	0.291834142	31.59	47.47	5.483	1.76
SPBC336.01	fbh1	0.7596	2.691	0.119415044	18.26	25.57	9.157	6.28
SPCC4B3.15	mid1	0.4396	2.691	0.356942316	27.45	40.67	1.233	2.985
SPAC15E1.04	SPAC15E1.04	0.8004	2.692	0.09669292	19.23	27.17	10.81	7.758
SPAC15A10.05C	mug182	0.8235	2.694	0.084336396	25.3	37.13	11.35	9.222
SPAC1A6.10	SPAC1A6.10	0.546	2.696	0.262807357	28.31	42.07	5.638	2.393
SPBC36B7.03	sec63	0.4754	2.696	0.322940823	36.08	54.84	5.041	1.614
SPAC823.03	ppk15	0.1747	2.697	0.757707095	31.67	47.58	2.23	1.091
SPCC1919.03C	amk2	0.4161	2.699	0.380802284	23.18	33.64	1.629	2.784
SPAC16C9.05	cph1	0.8156	2.7	0.088522783	24.48	35.77	11.94	8.346
SPAC1D4.02C	SPAC1D4.02c	0.5539	2.701	0.256568635	34.67	52.51	5.977	2.207
SPBP8B7.08C	SPBP8B7.08c	0.2753	2.706	0.560193789	28.78	42.83	2.743	1.511
SPAC13C5.07	rad32	0.7523	2.706	0.123608938	19.11	26.94	7.589	6.704
SPAC1296.06	tah18	0.7297	2.713	0.136855654	12.53	16.13	8.27	5.815
SPAC3F10.15C	spo12	0.1895	2.713	0.722390786	24.87	36.39	0.6111	1.606
SPAC19B12.10	sst2	0.4031	2.724	0.394587202	26.79	39.53	3.131	2.341
SPBC13E7.04	atp16	0.7048	2.727	0.151934105	23.22	33.66	7.557	5.339
SPAC1D4.09C	rtf2	0.5315	2.729	0.274496731	25.73	37.79	3.968	3.299
SPBC20F10.07	SPBC20F10.07	0.5858	2.73	0.232250633	24.88	36.39	4.403	3.873
SPBC27B12.04C	SPBC27B12.04c	0.6073	2.735	0.216596719	34.13	51.56	7.346	2.083
SPCC1672.12C	get4	0.7923	2.735	0.101110344	16.34	22.34	10.68	7.496
SPAC1952.06C	SPAC1952.06c	0.5409	2.735	0.266883019	29.07	43.26	5.919	2.075
SPAC17H9.04C	SPAC17H9.04c	0.3949	2.738	0.403512866	34.31	51.86	2.72	2.45
SPBC2G5.02C	SPBC2G5.02c	0.7663	2.74	0.115601174	25.06	36.66	11.72	5.099
SPAC630.09C	mug58	0.5927	2.742	0.227165073	31.35	46.99	6.357	2.897
SPAC27F1.10	SPAC27F1.10	0.6197	2.743	0.207818504	33.54	50.59	6.252	3.605
SPAC1687.07	SPAC1687.07	0.4113	2.745	0.38584129	32.8	49.36	4.63	0.8985
SPCC1919.10C	myo52	0.3418	2.758	0.466227942	25.19	36.85	3.245	1.797
SPAC10F6.12C	mam4	0.1183	2.763	0.927015255	30.63	45.77	1.686	1.114
SPBC21C3.12C	SPBC21C3.12c	0.7475	2.767	0.126388803	20.82	29.65	9.701	6.014
SPBC409.11	meu18	0.2851	2.768	0.545002783	26.33	38.7	2.248	1.894
SPBC947.09	SPBC947.09	0.4752	2.77	0.323123568	36.1	54.74	5.145	1.708
SPBC8D2.01	gsk31	0.3878	2.772	0.411392195	31.38	46.99	4.067	1.578
SPBC1709.18	tif452	0.3547	2.774	0.450138812	35.86	54.34	3.897	1.285
SPBC11C11.08	srp1	0.588	2.777	0.230622674	38.14	58.08	4.746	3.874
SPAC8F11.09C	nnt1	0.371	2.779	0.43062609	33.16	49.91	4.157	1.133
SPBC1E8.02	SPBC1E8.02	0.4924	2.779	0.307681956	33.4	50.29	5.79	0.7465
SPBC21B10.10	rps402	0.5948	2.785	0.22562904	25.28	36.95	6.329	3.102
SPCC191.11	inv1	0.5632	2.792	0.249337354	27.67	40.86	4.722	3.531
SPBC1348.07	SPBC1348.07	0.4397	2.794	0.356843534	34.96	52.83	4.333	2.089
SPAC1142.08	fhl1	0.7541	2.794	0.122571059	22.12	31.74	9.821	6.072

SPBC428.10	SPBC428.10	0.437	2.795	0.359518563	36.04	54.6	4.565	1.816
SPAC3A11.09	sod22	0.4129	2.796	0.384155117	29.46	43.8	2.852	2.62
SPCC18.02	SPCC18.02	0.2188	2.797	0.659952682	29	43.04	1.85	1.659
SPAC24H6.13	SPAC24H6.13	0.4657	2.801	0.331893762	32.25	48.38	4.709	2.14
SPAC806.04C	SPAC806.04c	0.4625	2.802	0.334888263	35.03	52.94	4.518	2.264
SPAC637.09	SPAC637.09	0.3166	2.806	0.499489089	33.02	49.62	3.597	1.215
SPAPB2B4.03	cig2	0.3327	2.808	0.477947199	32.09	48.09	3.872	0.9714
SPAC22F3.09C	res2	0.5815	2.812	0.235450281	16.23	22.04	5.133	3.876
SPCC757.07C	ctt1	0.5374	2.813	0.269702338	27.45	40.46	3.583	3.641
SPAC821.05	SPAC821.05	0.2353	2.813	0.628378073	23.34	33.72	3.048	0.8062
SPAC823.05C	tlg2	0.7202	2.815	0.142546883	7.243	7.274	7.274	6.01
SPBC15D4.10C	amo1	0.7235	2.817	0.140561465	15.51	20.85	7.635	5.978
SPAC16A10.02	sub1	0.5305	2.818	0.275314612	31.31	46.81	5.641	2.429
SPCC16A11.10C	oca8	0.6713	2.819	0.173083353	31.81	47.61	8.319	3.739
SPBC27B12.08	sip1	0.6731	2.819	0.171920409	28.82	42.7	7.536	4.404
SPAC17G6.08	pep7	0.5229	2.82	0.281581358	34.93	52.75	5.865	1.991
SPAC17G8.09	shg1	0.323	2.827	0.490797478	23.93	34.66	2.627	2.064
SPBC725.09C	hob3	0.7629	2.83	0.117532385	18.2	25.25	9.654	6.76
SPAC24H6.08	SPAC24H6.08	0.4699	2.832	0.327994555	28.57	42.27	2.322	3.266
SPBC29A10.05	exo1	0.3902	2.833	0.408712735	29.43	43.68	2.475	2.597
SPBC543.05C	SPBC543.05c	0.5236	2.837	0.281000362	31.39	46.89	5.822	2.127
SPAC27D7.14C	tpr1	0.5594	2.843	0.252277538	15.8	21.28	4.832	3.729
SPBC725.04	SPBC725.04	0.4512	2.848	0.345630909	29.66	44.04	2.867	3.005
SPAC29E6.01	pof11	0.5071	2.853	0.294906389	26.51	38.86	4.984	2.671
SPAC56F8.05C	mug64	0.5461	2.854	0.262727823	29.65	44.02	6.157	2.324
SPAC19A8.04	erg5	0.6505	2.856	0.186752699	36.33	54.98	8.2	3.217
SPBC8E4.03	SPBC8E4.03	0.5129	2.856	0.289967301	28.59	42.26	5.53	2.294
SPAC1142.02C	SPAC1142.02c	0.2589	2.86	0.58686795	28.75	42.52	2.187	1.839
SPBC29A10.07	pom152	0.5036	2.865	0.297914279	35.32	53.31	5.891	1.558
SPCC1494.08C	SPCC1494.08c	0.4308	2.865	0.365724305	23.9	34.55	4.177	2.24
SPCC191.05C	SPCC191.05c	0.8049	2.873	0.094258073	26.73	39.19	13.66	7.384
SPCC297.04C	set7	0.6117	2.874	0.21346152	34.79	52.43	7.489	2.712
SPBC23G7.10C	SPBC23G7.10c	0.5036	2.875	0.297914279	35.11	52.96	5.549	2.133
SPBC651.05C	dot2	0.591	2.887	0.228412519	31.07	46.3	5.25	3.943
SPCC1020.11C	SPCC1020.11c	0.7465	2.893	0.126970188	21.74	30.95	9.225	6.434
SPBC17D11.01	nep1	0.5302	2.893	0.275560277	35.03	52.78	5.148	3.008
SPAC22H12.02	tfg3	0.673	2.896	0.171984936	24.62	35.68	8.453	3.989
SPAC3C7.04	SPAC3C7.04	0.2587	2.896	0.587203571	22.29	31.86	2.094	1.899
SPBC691.03C	apl3	0.2305	2.901	0.63732907	32.54	48.69	2.25	1.676
SPAC11D3.07C	SPAC11D3.07c	0.4489	2.909	0.347850395	33.91	50.92	4.318	2.446
SPBC902.06	mto2	0.4433	2.91	0.353302269	35.13	52.92	5.088	1.536
SPBC19F8.02	SPBC19F8.02	0.3877	2.911	0.411504199	30.23	44.88	4.381	1.504
SPCC1223.03C	gut2	0.4527	2.912	0.344189506	32.42	48.47	4.572	2.312
SPCC1742.01	SPCC1742.01	0.6005	2.912	0.221486988	30.45	45.23	6.875	3.169
SPAC16C9.02C	SPAC16C9.02c	0.4664	2.917	0.331241458	32.41	48.45	5.425	1.557
SPCC16C4.20C	SPCC16C4.20c	0.4521	2.923	0.344765493	31.46	46.87	3.668	2.851
SPBC1652.01	SPBC1652.01	0.2691	2.924	0.570086302	31.11	46.3	2.542	1.828
SPAC17H9.19C	cdt2	0.771	2.925	0.112945622	20.62	29.06	10.58	7.122
SPAC750.08C	SPAC750.08c	0.4666	2.925	0.331055266	25.54	37.14	3.115	3.186
SPAC13D6.03C	trm9	0.3613	2.927	0.442132038	33.11	49.58	4.157	1.405
SPAC2C4.06C	SPAC2C4.06c	0.4111	2.929	0.386052523	31.19	46.43	4.883	1.133
SPBC14F5.09C	ade8	0.3296	2.929	0.482012797	27.38	40.17	1.742	2.429
SPBPJ4664.05	SPBPJ4664.05	0.5396	2.93	0.267928059	35.84	54.06	6.568	1.812
SPAC11E3.10	SPAC11E3.10	0.5783	2.933	0.237846808	33.98	51	6.153	3.295
SPAC664.12C	SPAC664.12c	0.2325	2.934	0.633577043	34.21	51.37	3.174	0.774
SPAC1F5.09C	shk2	0.5515	2.935	0.258454483	35.52	53.52	6.295	2.572
SPBC29A10.02	spo5	0.1071	2.935	0.970210529	30.23	44.84	2.141	0.6276
SPAC23C11.04C	pnk1	0.4712	2.937	0.326794718	28	41.17	5.385	1.82
SPBC342.04	rpn1301	0.3305	2.941	0.480828536	34.32	51.54	3.063	2.055
SPAC29A4.14C	SPAC29A4.14c	0.2296	2.944	0.639028116	28.3	41.65	1.809	1.842
SPBC646.15C	SPBC646.15c	0.4634	2.947	0.334043971	32.34	48.28	4.72	2.418
SPCC1183.02	SPCC1183.02	0.3241	2.951	0.489320969	29.29	43.26	2.42	2.272
SPBC16E9.11C	pub3	0.5493	2.951	0.260190401	31.21	46.41	4.326	3.795
SPAP27G11.15	slx1	0.3915	2.952	0.407268234	34.34	51.56	3.787	2.215
SPAC23C11.01	SPAC23C11.01	0.3461	2.962	0.460798401	28.43	41.84	2.697	2.352
SPCC1322.02	SPCC1322.02	0.3153	2.966	0.501276029	34.23	51.35	3.755	1.339
SPCC576.11	rpl15	0.7067	2.967	0.150764909	12.33	15.38	7.358	5.999
SPAC30.04C	abc4	0.3796	2.967	0.420673796	24.33	35.09	3.719	2.159
SPAC23C11.06C	SPAC23C11.06c	0.8164	2.968	0.088097004	26.91	39.33	13.48	9.07

SPBC2G5.01	SPBC2G5.01	0.3903	2.969	0.408601449	30.27	44.84	2.894	2.636
SPCC162.04C	wtf13	0.3281	2.979	0.48399377	33.17	49.59	3.96	1.255
SPBC6B1.08C	ofd1	0.3308	2.988	0.480434499	30.07	44.48	2.025	2.444
SPAC14C4.15C	SPAC14C4.15c	0.3247	2.991	0.488517711	27.5	40.27	2.537	2.28
SPAC6G9.03C	mug183	0.5993	2.995	0.222355722	31.86	47.42	6.815	3.442
SPAC3A12.10	rpl2001	0.6095	2.996	0.21502629	16.57	22.29	7.542	3.06
SPAC6F12.03C	fsv1	0.4776	2.996	0.320935682	26.18	38.08	4.997	2.516
SPBC428.07	meu6	0.342	3.007	0.465973894	32.63	48.65	4.314	0.8
SPAC1782.09C	clp1	0.2847	3.007	0.545612533	33.35	49.84	3.44	1.411
SPBC1734.09	SPBC1734.09	0.3211	3.014	0.493359694	28.95	42.6	2.086	2.393
SPAC1687.21	SPAC1687.21	0.3435	3.015	0.464073259	32.25	48.02	4.125	1.367
SPBC651.10	nse5	0.6694	3.02	0.174314292	15.58	20.63	6.286	5.488
SPAC4F10.20	grx1	0.1432	3.022	0.844056982	30.24	44.71	0.4368	1.539
SPBC8E4.01C	SPBC8E4.01c	0.3441	3.024	0.463315327	27.88	40.83	2.696	2.406
SPBC776.14	plh1	0.3982	3.026	0.399898744	31.5	46.77	4.856	1.234
SPCC126.03	pus1	0.3653	3.028	0.437350328	23.6	33.8	3.132	2.418
SPAC8C9.03	cgs1	0.5618	3.03	0.250418265	16.29	21.78	5.262	3.961
SPBC1289.06C	ppr8	0.6379	3.033	0.195247398	35.56	53.44	4.57	5.336
SPBC83.19C	SPBC83.19c	0.4051	3.041	0.392437757	29.24	43.03	2.634	2.912
SPAC2C4.10C	SPAC2C4.10c	0.6013	3.043	0.220908796	28.22	41.35	5.319	4.435
SPCC31H12.08C	ccr4	0.7668	3.044	0.115317896	27.21	39.69	11.02	7.158
SPAC513.05	ams1	0.488	3.05	0.311580178	31.63	46.94	5.944	1.775
SPBP4H10.18C	SPBP4H10.18c	0.4621	3.054	0.335264031	34.18	51.12	5.341	2.04
SPCC965.09	SPCC965.09	0.4439	3.054	0.352714855	34.6	51.82	5.059	2.026
SPBC530.07C	SPBC530.07c	0.5465	3.058	0.262409834	13.22	16.69	5.787	3.236
SPBC1198.07C	SPBC1198.07c	0.2985	3.058	0.525055665	33.55	50.09	3.278	1.796
SPBC577.15C	sim3	0.4316	3.06	0.364918564	4.478	2.329	2.329	3.215
SPAC4G9.13C	vps26	0.5159	3.064	0.287434472	15.58	20.56	4.621	3.626
SPCC191.03C	SPCC191.03c	0.5812	3.068	0.235674394	34.21	51.15	6.443	3.506
SPAC1782.11	met14	0.7151	3.07	0.145633222	26.18	37.96	8.862	6.225
SPAC22H10.11C	SPAC22H10.11c	0.185	3.07	0.732828272	13.35	16.88	1.692	1.707
SPBC1773.16C	SPBC1773.16c	0.3701	3.073	0.431680915	30.43	44.94	2.662	2.665
SPAC3C7.10	pex13	0.3538	3.076	0.451242171	33.69	50.28	3.417	2.244
SPAC1B1.04C	SPAC1B1.04c	0.2541	3.078	0.594995335	34.7	51.94	3.478	0.982
SPAC17A2.10C	SPAC17A2.10c	0.464	3.082	0.333482019	34.47	51.56	4.819	2.627
SPBC4B4.04	SPBC4B4.04	0.3338	3.082	0.476513668	30.94	45.76	3.116	2.229
SPAC25A8.03C	SPAC25A8.03c	0.3625	3.083	0.440691989	29.87	44	2.468	2.666
SPBC11C11.07	rpl1801	0.4663	3.083	0.331334585	14.3	18.42	3.092	3.405
SPAC23G3.12C	SPAC23G3.12c	0.4339	3.089	0.36261035	32.04	47.55	5.176	1.768
SPCC162.12	tco89	0.6583	3.092	0.181576145	17.78	24.13	8.737	3.941
SPBC18H10.05	SPBC18H10.05	0.4316	3.095	0.364918564	35.55	53.3	3.702	2.88
SPBC4F6.09	str1	0.4282	3.096	0.368353337	32.22	47.84	5.254	1.521
SPCC1682.15	mug122	0.6706	3.099	0.173536451	33.96	50.69	7.586	5.168
SPCC162.01C	SPCC162.01c	0.4382	3.106	0.358327627	35.7	53.54	4.342	2.648
SPAC57A10.07	SPAC57A10.07	0.268	3.111	0.571865206	32.88	48.9	3.737	0.6172
SPAC27D7.08C	SPAC27D7.08c	0.3088	3.112	0.510322708	27.99	40.87	2.702	2.237
SPAC1639.01C	SPAC1639.01c	0.6866	3.114	0.163296201	25.79	37.25	7.816	5.865
SPAPB18E9.01	trm5	0.3936	3.118	0.40494491	36.16	54.28	5.145	0.2992
SPAC4A8.09C	cwf21	0.3292	3.119	0.482540173	32.07	47.56	4.066	1.471
SPAC30D11.14C	SPAC30D11.14c	0.2588	3.12	0.587035728	31.19	46.11	2.721	1.87
SPCC970.05	rpl3601	0.3046	3.122	0.516270101	33.29	49.55	4.11	0.6654
SPCC11E10.08	rik1	0.7578	3.123	0.120445399	21.87	30.8	10.35	7.33
SPBC3D6.15	rps2501	0.7097	3.126	0.148925195	27.43	39.92	8.867	6.203
SPBC8D2.16C	SPBC8D2.16c	0.3508	3.128	0.454940415	29.02	42.52	1.777	2.75
SPCC338.07C	naa15	0.2574	3.13	0.589391457	31.1	45.94	3.484	1.187
SPBC16D10.08C	SPBC16D10.08c	0.4485	3.133	0.348237553	33.5	49.87	4.621	2.649
SPCC1442.16C	zta1	0.1853	3.133	0.732124581	30.41	44.81	2.104	1.637
SPAC5H10.02C	SPAC5H10.02c	0.283	3.134	0.548213564	32.31	47.93	3.251	1.769
SPBC19G7.18C	SPBC19G7.18c	0.6079	3.138	0.216167857	28.22	41.19	7.554	3.498
SPAC6C3.06C	SPAC6C3.06c	0.167	3.139	0.777283529	29.9	43.95	1.613	1.665
SPAC31A2.16	gef2	0.3647	3.143	0.438064237	28.74	42.04	2.487	2.742
SPCC1827.03C	SPCC1827.03c	0.2877	3.147	0.541060138	29.25	42.88	3.177	1.881
SPBC1271.08C	SPBC1271.08c	0.4105	3.148	0.386686839	34.41	51.34	4.649	2.126
SPAC29B12.10C	pgt1	0.7851	3.15	0.105075023	24.22	34.62	12.05	8.251
SPAC343.11C	msc1	0.4277	3.152	0.36886075	31.92	47.25	5.293	1.639
SPAC17A5.08	SPAC17A5.08	0.7846	3.153	0.105351696	20.73	28.88	11.78	8.368
SPAC56F8.02	SPAC56F8.02	0.2768	3.156	0.557833914	28.05	40.88	3.62	1.351
SPCC24B10.13	skb5	0.5292	3.157	0.276380164	29.81	43.77	4.187	3.927
SPAC26H5.03	pcf2	0.3876	3.158	0.411616232	30.28	44.55	4.635	1.796

SPAC222.14C	SPAC222.14c	0.315	3.163	0.501689446	30.58	45.04	3.84	1.639
SPBP4G3.02	pho1	0.3913	3.164	0.407490152	33.47	49.78	4.507	2.011
SPAC688.12C	SPAC688.12c	0.3249	3.166	0.488250289	35.05	52.38	4.076	1.493
SPAC13G6.03	gpi7	0.3076	3.173	0.512013669	35.59	53.25	4.101	1.098
SPCC31H12.05C	sds21	0.3867	3.175	0.412625828	36.04	53.99	3.623	2.581
SPCC63.13	SPCC63.13	0.3872	3.184	0.412064651	32.4	47.98	4.368	2.123
SPBC16C6.02C	vps1302	0.5918	3.185	0.227825039	18.73	25.53	6.142	4.433
SPBC660.09	mug168	0.3561	3.186	0.448428026	32.29	47.81	4.135	1.925
SPAC1327.01C	SPAC1327.01c	0.5148	3.19	0.288361462	25.62	36.85	3.477	3.995
SPBC23G7.15C	rpp202	0.2892	3.194	0.538801711	32.22	47.68	2.736	2.164
SPAC22F8.05	SPAC22F8.05	0.5253	3.196	0.279592599	27.21	39.44	4.197	3.937
SPAC6G9.16C	SPAC6G9.16c	0.5015	3.2	0.299729063	28.9	42.22	3.425	3.865
SPAC24B11.13	hem3	0.366	3.202	0.436518915	32.41	47.98	4.71	1.345
SPAC664.04C	rps1602	0.4312	3.203	0.365321248	28.29	41.21	5.407	1.705
SPBCPT2R1.03	SPBCPT2R1.03	0.4244	3.206	0.372224625	29.08	42.51	2.744	3.247
SPBC21.05C	ral2	0.6138	3.208	0.211973116	27.59	40.05	6.896	4.581
SPAC1782.02C	SPAC1782.02c	0.5931	3.211	0.226872076	36.1	54.02	7.505	3.349
SPAC977.14C	SPAC977.14c	0.3181	3.213	0.497436331	24.24	34.54	2.693	2.411
SPAC26A3.10	cnt6	0.4165	3.213	0.380384994	31.59	46.6	4.581	2.396
SPAC1002.06C	bqt2	0.4468	3.214	0.349886836	34.19	50.88	5.522	1.938
SPBC1347.07	rex2	0.3371	3.22	0.472241247	27.91	40.55	2.929	2.487
SPBC1711.05	SPBC1711.05	0.4554	3.222	0.341606974	26.11	37.6	4.622	2.896
SPAC29B12.06C	rcd1	0.4992	3.222	0.301725423	23.91	33.97	5.022	3.276
SPCC1919.11	mug137	0.5166	3.228	0.286845598	35.52	53.05	6.373	2.546
SPBC31F10.03	SPBC31F10.03	0.2791	3.229	0.554240164	30.66	45.05	1.329	2.413
SPAC6G9.13C	bqt1	0.5776	3.229	0.238372815	37.17	55.76	8.096	2.041
SPBC2D10.16	mhf1	0.145	3.233	0.838631998	30.59	44.93	2.599	1.018
SPAC22H10.03C	kap114	0.3058	3.233	0.514562519	31.69	46.74	3.904	1.566
SPCC338.11C	rrg1	0.2992	3.238	0.524038411	28.28	41.13	2.322	2.402
SPAC23G3.05C	SPAC23G3.05c	0.5114	3.243	0.291239276	28.9	42.15	6.322	2.523
SPBC3E7.02C	hsp16	0.2745	3.247	0.561457651	32.7	48.38	3.222	1.854
SPCC613.08	SPCC613.08	0.5813	3.249	0.235599677	27.25	39.42	4.274	4.825
SPBC18E5.08	SPBC18E5.08	0.3586	3.257	0.445389715	29.69	43.43	2.795	2.738
SPAC27D7.06	SPAC27D7.06	0.4097	3.263	0.387534036	27.83	40.35	2.606	3.212
SPAC20H4.11C	rho5	0.4924	3.269	0.307681956	35.84	53.5	4.579	3.481
SPAC222.12C	atp2	0.3382	3.27	0.470826397	28.16	40.89	2.711	2.616
SPAC3G9.07C	hos2	0.1984	3.275	0.702458332	31.79	46.84	3.262	0.5832
SPAC5D6.08C	mes1	0.3936	3.281	0.40494491	34.09	50.6	5.36	0.794
SPBC30D10.18C	rpl102	0.2988	3.288	0.524619407	30.56	44.8	2.185	2.474
SPAC589.12	SPAC589.12	0.7155	3.292	0.145390362	15.2	19.56	9.483	6.702
SPCC11E10.03	mug1	0.2708	3.293	0.56735134	28.82	41.93	2.339	2.253
SPBC902.03	SPBC902.03	0.3618	3.295	0.441531437	35.48	52.87	4.78	1.412
SPAC13G6.02C	rps101	0.23	3.298	0.638272164	26.01	37.3	2.51	1.921
SPBC887.02	SPBC887.02	0.08598	3.304	1.065602559	29.01	42.22	1.908	1.113
SPBC2F12.03C	SPBC2F12.03c	0.2529	3.315	0.597051171	32.04	47.19	3.285	1.681
SPCC757.11C	SPCC757.11c	0.2979	3.315	0.525929497	33.04	48.82	4.226	1.004
SPAPB1E7.07	glt1	0.2448	3.322	0.611188587	31.04	45.53	2.934	1.857
SPBC28F2.11	SPBC28F2.11	0.3251	3.326	0.487983031	30.72	45	3.803	2.062
SPAC11D3.08C	SPAC11D3.08c	0.03302	3.333	1.481222931	27.81	40.21	1.157	0.9478
SPBC16C6.11	rpl3201	0.7698	3.339	0.113622093	14.74	18.73	10.83	8.647
SPAC977.11	SPAC977.11	0.3476	3.34	0.458920232	29.55	43.05	2.13	2.879
SPAC186.01	SPAC186.01	0.2006	3.341	0.697669071	33.67	49.81	3.324	0.7793
SPCC126.09	SPCC126.09	0.4249	3.344	0.371713269	33.4	49.38	5.438	1.945
SPBC839.05C	rps1701	0.7396	3.347	0.131003098	16.29	21.25	9.615	7.59
SPAC1952.09C	SPAC1952.09c	0.356	3.347	0.448550002	36.93	55.17	4.474	1.888
SPBC800.10C	SPBC800.10c	0.445	3.35	0.351639989	30.96	45.36	5.643	2.13
SPAC17H9.14C	SPAC17H9.14c	0.433	3.352	0.363512104	29.66	43.21	2.546	3.536
SPCC13B11.03C	SPCC13B11.03c	0.3058	3.354	0.514562519	28.96	42.07	2.354	2.547
SPAC12G12.15	sif3	0.3095	3.354	0.509339347	27.8	40.16	3.31	2.247
SPCC338.05C	mms2	0.7915	3.362	0.101549081	23.48	33.05	12.81	9.31
SPBC365.11	SPBC365.11	0.2248	3.362	0.648203693	33.91	50.17	3.004	1.68
SPCC550.15C	SPCC550.15c	0.2818	3.366	0.550059011	31.2	45.72	3.822	1.571
SPCC18.10	SPCC18.10	0.3342	3.366	0.475993554	29.39	42.74	2.411	2.754
SPCC1322.15	rpl3402	0.4282	3.366	0.368353337	33.65	49.75	5.029	2.498
SPBC12D12.02C	cdm1	0.382	3.369	0.417936637	33.68	49.8	4.128	2.538
SPCC777.13	vps35	0.5696	3.369	0.244430019	13.16	16.08	5.375	4.511
SPAC513.04	SPAC513.04	0.3351	3.373	0.474825572	35.16	52.21	4.266	1.852
SPCC663.10	SPCC663.10	0.2696	3.379	0.569280112	27.67	39.9	3.62	1.656
SPAC23H3.08C	bub3	0.528	3.382	0.277366077	33.39	49.28	6.218	3.337

SPCC1840.04	pca1	0.5202	3.387	0.283829652	34.71	51.45	6.222	3.189
SPCC162.06C	SPCC162.06c	0.3939	3.39	0.404614019	33.52	49.48	3.677	2.912
SPAC3A11.06	mvp1	0.4023	3.395	0.395449967	31.54	46.23	5.391	1.634
SPBPB2B2.19C	SPBPB2B2.19c	0.7763	3.4	0.109970414	17.34	22.89	11.67	8.935
SPAC24B11.10C	chr3	0.4185	3.406	0.378304538	35.24	52.29	5.588	1.749
SPAC140.04	SPAC140.04	0.5463	3.408	0.262568799	28.41	41.07	6.93	3.224
SPAC6C3.04	cit1	0.3736	3.415	0.427593132	34.87	51.67	4.993	1.681
SPAC56F8.14C	mug115	0.3226	3.421	0.491335637	26.79	38.39	2.621	2.671
SPBPB2B2.18	SPBPB2B2.18	0.2473	3.425	0.606775884	35.49	52.66	3.745	1.216
SPAPB2B4.04C	SPAPB2B4.04c	0.266	3.431	0.575118363	32.2	47.26	2.991	2.115
SPCC11E10.04	ppr6	0.4301	3.432	0.366430557	31.17	45.56	3.438	3.407
SPBC543.07	pek1	0.1283	3.433	0.891773344	30.96	45.22	2.362	1.308
SPCC1450.06C	grx3	0.2204	3.437	0.65678841	33	48.56	3.471	1.221
SPBC12C2.09C	SPBC12C2.09c	0.4126	3.443	0.384470776	31.91	46.76	5.108	2.336
SPCC4F11.04C	imt2	0.7573	3.447	0.120732043	17.18	22.55	11.06	8.23
SPAC17A2.14	SPAC17A2.14	0.268	3.449	0.571865206	31.93	46.78	3.91	1.388
SPAPB24D3.09C	pdr1	0.3798	3.45	0.42044504	29.63	43.01	2.929	3.091
SPAC3C7.05C	mug191	0.3274	3.451	0.484921325	28.87	41.75	4.076	2.064
SPBC19F8.06C	meu22	0.2728	3.454	0.564155634	34.19	50.49	3.552	1.864
SPAC3C7.03C	rhp55	0.137	3.457	0.863279433	26.7	38.17	2.694	1.087
SPBC32F12.01C	css1	0.7133	3.457	0.146727776	22.47	31.22	10.57	6.253
SPCC645.13	SPCC645.13	0.4048	3.459	0.392759496	35.51	52.65	5.12	2.226
SPAC9E9.11	plr1	0.2653	3.461	0.57626275	35.31	52.31	3.322	1.961
SPBC16E9.15	SPBC16E9.15	0.4397	3.461	0.356843534	34.45	50.9	3.064	3.633
SPAC3H1.11	hsr1	0.1137	3.462	0.944239535	30.57	44.53	0.6961	1.583
SPCP1E11.10	SPCP1E11.10	0.665	3.465	0.177178355	11.48	13.17	6.684	6.356
SPCPB16A4.02C	SPCPB16A4.02	0.5213	3.467	0.282912275	26.69	38.14	6.092	3.486
SPCC24B10.03	SPCC24B10.03	0.3275	3.467	0.484788696	36.13	53.65	4.701	1.246
SPBC18H10.09	SPBC18H10.09	0.6136	3.469	0.212114649	26.9	38.49	7.213	4.812
SPAC24B11.08C	SPAC24B11.08c	0.4317	3.475	0.364817951	34.99	51.77	5.993	1.622
SPBC1773.06C	SPBC1773.06c	0.4366	3.475	0.359916269	32.68	47.97	3.633	3.473
SPBC4B4.08	ght2	0.3252	3.477	0.487849463	31.17	45.49	2.3	2.813
SPBC1D7.05	byr2	0.6665	3.484	0.176199846	24.91	35.19	8.641	5.908
SPAC2F7.09C	SPAC2F7.09c	0.406	3.485	0.391473966	29.79	43.21	3.465	3.225
SPAC3A11.13	SPAC3A11.13	0.2886	3.49	0.539703673	34.9	51.59	4.184	1.427
SPBC16E9.06C	uvi31	0.3521	3.496	0.453333975	30.86	44.95	4.622	1.964
SPCC14G10.04	SPCC14G10.04	0.3465	3.497	0.460296761	19.02	25.5	4.608	1.891
SPAC29A4.19C	cta5	0.2425	3.498	0.615288257	31.88	46.62	3.104	1.926
SPBC29A3.14C	trt1	0.7005	3.498	0.15459186	20.94	28.65	8.396	6.954
SPAC926.02	SPAC926.02	0.1989	3.501	0.701365217	30.31	44.04	2.424	1.901
SPBC30D10.04	swi3	0.1272	3.507	0.895512889	25.41	35.98	1.897	1.575
SPBC31F10.08	mde2	0.2101	3.508	0.677573948	29.75	43.11	2.446	1.98
SPCC830.08C	yop1	0.4761	3.51	0.322301819	34.52	50.94	6.41	2.344
SPCC417.12	SPCC417.12	0.3582	3.517	0.445874418	29.78	43.13	2.506	3.071
SPAC25B8.01	dap1	0.6684	3.519	0.174963559	34.96	51.64	10.77	4.119
SPBC342.03	gas4	0.3345	3.523	0.475603878	30.38	44.12	3.007	2.762
SPBPB2B2.11	SPBPB2B2.11	0.4071	3.525	0.390298898	29.94	43.39	5.36	2.153
SPAC26H5.09C	SPAC26H5.09c	0.2438	3.525	0.612966299	29.24	42.24	2.246	2.284
SPAC22G7.01C	SPAC22G7.01c	0.3142	3.526	0.502793819	31.26	45.56	4.525	1.464
SPAC1071.12C	stp1	0.3238	3.529	0.489723156	32.92	48.27	3.465	2.505
SPBC16G5.17	SPBC16G5.17	0.3613	3.53	0.442132038	25.28	35.73	4.514	2.266
SPAC31A2.02	trm112	0.2956	3.53	0.52929557	19.13	25.62	3.26	2.347
SPBC365.06	pmt3	0.2758	3.534	0.559405738	29.43	42.53	2.53	2.449
SPBC21C3.02C	dep1	0.2971	3.538	0.527097348	33.7	49.54	4.14	1.738
SPBC215.14C	vps20	0.5149	3.538	0.288277108	34.04	50.11	5.555	3.829
SPBC83.18C	fic1	0.5212	3.539	0.282995593	29.51	42.66	6.497	3.356
SPBC428.04	apq12	0.3902	3.54	0.408712735	34.11	50.21	5.663	1.166
SPAC1071.05	SPAC1071.05	0.2708	3.551	0.56735134	24.13	33.81	3.961	1.576
SPBC3D6.09	dpb4	0.499	3.552	0.301899454	34.36	50.6	3.578	4.311
SPCC5E4.05C	SPCC5E4.05c	0.3374	3.553	0.471854922	29.39	42.43	3.175	2.765
SPBC16C6.09	ogm4	0.4464	3.555	0.350275814	28.02	40.18	4.927	3.17
SPAC23A1.17	SPAC23A1.17	0.1006	3.56	0.997402019	30.17	43.72	2.112	1.31
SPAC10F6.05C	ubc6	0.245	3.565	0.610833916	34.57	50.92	3.275	1.917
SPCC31H12.04C	rpl1202	0.5764	3.566	0.239276028	23.03	31.97	7.568	3.889
SPBC106.16	pre6	0.3457	3.568	0.46130062	29.35	42.35	2.063	3.091
SPCC1442.14C	SPCC1442.14c	0.4906	3.572	0.309272456	35.71	52.79	6.954	2.183
SPAPB1A10.13	SPAPB1A10.13	0.1909	3.577	0.719194072	29.06	41.86	1.082	2.121
SPAC14C4.07	SPAC14C4.07	0.07236	3.58	1.140501442	30.84	44.78	2.241	0.6114
SPAC17A5.07C	ulp2	0.3013	3.581	0.521000868	30.4	44.04	3.802	2.16

SPBC36.07	iki3	0.05742	3.59	1.240936812	23.71	33.06	1.762	1.092
SPAC15A10.10	mde6	0.785	3.591	0.105130343	26.38	37.44	13.71	9.411
SPBC577.02	rpl3801	0.7197	3.591	0.142848497	21.27	29.04	10.03	7.319
SPBC18H10.08C	ubp4	0.4463	3.592	0.350373113	36.46	53.99	6.351	1.858
SPBC29A10.14	rec8	0.25	3.598	0.602059991	30.27	43.8	1.228	2.508
SPAC23A1.04C	mnl1	0.2878	3.598	0.54090921	30.22	43.73	2.879	2.498
SPAC869.10C	put4	0.2941	3.605	0.531504975	34.09	50.08	4.538	1.134
SPAC1142.03C	swi2	0.1041	3.606	0.98254927	30.65	44.41	1.695	1.522
SPBC15C4.02	SPBC15C4.02	0.5704	3.609	0.243820483	36.15	53.44	8.839	2.301
SPAC1039.05C	klf1	0.3486	3.61	0.457672617	29.89	43.16	2.482	3.09
SPAC1F8.04C	SPAC1F8.04c	0.2444	3.612	0.611898798	30.51	44.19	2.881	2.165
SPAPJ696.01C	vps17	0.6765	3.613	0.169732199	18.15	23.88	8.158	6.54
SPAC1F7.11C	SPAC1F7.11c	0.2564	3.617	0.591081979	34.26	50.33	3.545	1.905
SPAC11H11.05C	fta6	0.2372	3.621	0.624885315	33.28	48.72	3.518	1.714
SPAC17G8.11C	imt3	0.1143	3.627	0.94195377	32.19	46.91	1.64	1.614
SPAC29A4.09	SPAC29A4.09	0.5222	3.63	0.282163133	8.029	7.226	4.917	4.374
SPBC16D10.01C	SPBC16D10.01	0.4827	3.632	0.316322701	34.49	50.69	7.336	1.185
SPAC1805.15C	pub2	0.2216	3.632	0.654430244	29.43	42.38	1.01	2.354
SPBC577.05C	rec27	0.7815	3.635	0.107071018	16.93	21.84	12.65	9.839
SPAC13D6.04C	btb3	0.3648	3.641	0.43794517	28.42	40.7	3.791	2.89
SPBC19C2.04C	ubp11	0.3305	3.641	0.480828536	28.02	40.05	2.565	2.957
SPBC776.17	SPBC776.17	0.6521	3.653	0.1856858	17.68	23.04	7.782	6.047
SPACUNK4.16C	SPACUNK4.16c	0.3321	3.654	0.478731124	35.26	51.92	4.883	1.59
SPCC24B10.10C	SPCC24B10.10	0.3409	3.656	0.467372999	30.33	43.81	2.293	3.103
SPBPB2B2.10C	gal7	0.3178	3.658	0.497846107	26.77	37.97	2.364	2.911
SPAC343.04C	SPAC343.04c	0.3197	3.659	0.495257364	29.51	42.45	1.724	3.012
SPAC16A10.04	rho4	0.3544	3.662	0.450506287	37.36	55.35	5.042	1.849
SPAC12G12.07C	SPAC12G12.07	0.4841	3.678	0.315064917	29.61	42.59	6.125	3.219
SPCC4G3.11	mug154	0.1774	3.68	0.751046385	27.93	39.83	2.299	1.924
SPAC17A2.11	SPAC17A2.11	0.128	3.681	0.89279003	32.19	46.82	2.003	1.656
SPBC21D10.10	bdc1	0.2023	3.682	0.694004117	25.78	36.3	2.779	1.931
SPAC1834.05	alg9	0.1841	3.686	0.734946211	29.18	41.87	3.285	1.349
SPBC216.01C	SPBC216.01c	0.49	3.686	0.30980392	35.06	51.53	6.065	3.39
SPBC23E6.10C	SPBC23E6.10c	0.2115	3.691	0.674689628	30.73	44.41	0.8462	2.329
SPAC17C9.09C	tim13	0.2777	3.692	0.55642412	28.86	41.34	2.245	2.659
SPAC1786.02	SPAC1786.02	0.7234	3.697	0.140621496	22.66	31.15	10.52	7.619
SPCPB16A4.04C	trm8	0.2254	3.7	0.647046088	28.55	40.81	3.667	1.512
SPAC6C3.02C	SPAC6C3.02c	0.3301	3.702	0.481354476	35.82	52.76	5.207	0.6842
SPBC365.01	SPBC365.01	0.3962	3.705	0.402085529	34.61	50.77	4.835	2.789
SPAC14C4.08	mug5	0.3845	3.71	0.415103656	29.9	43.01	3.155	3.367
SPAC4F10.02	aap1	0.1672	3.711	0.776763727	30.79	44.48	1.455	2.029
SPBC8D2.11	SPBC8D2.11	0.4103	3.712	0.386898483	33.13	48.32	5.962	1.896
SPAC15A10.16	bud6	0.6608	3.721	0.179929966	27.24	38.64	8.852	6.307
SPAC589.06C	SPAC589.06c	0.2621	3.722	0.581532979	26.64	37.65	2.148	2.587
SPAC26F1.02	pnn1	0.2995	3.722	0.523603173	36.58	53.97	4.506	1.675
SPCC417.02	dad5	0.7285	3.724	0.137570444	23.24	32.05	10.82	7.815
SPAC29A4.02C	SPAC29A4.02c	0.2482	3.731	0.605198223	31.01	44.82	3.057	2.234
SPCC1753.03C	rec7	0.4023	3.732	0.395449967	34.28	50.17	5.183	2.669
SPBC21D10.08C	SPBC21D10.08	0.5082	3.734	0.293965339	34.48	50.5	7.347	2.731
SPBC36.11	SPBC36.11	0.2532	3.735	0.596536299	31.19	45.1	1.23	2.627
SPBC354.03	swd3	0.1907	3.74	0.719649307	33.9	49.54	3.624	0.8225
SPBC15C4.06C	SPBC15C4.06c	0.4307	3.742	0.365825128	28.32	40.36	3.875	3.685
SPAC1952.16	rga9	0.5235	3.745	0.281083314	35.16	51.6	7.587	2.93
SPAC1610.01	SPAC1610.01	0.6945	3.747	0.15832775	20.12	26.89	9.191	7.132
SPBC1706.03	fzo1	0.3669	3.75	0.435452288	31.4	45.42	2.856	3.315
SPAC4F10.14C	btf3	0.01952	3.75	1.709520187	19.38	25.68	0.9225	0.9423
SPAC29B12.02C	set2	0.3111	3.753	0.507099989	35.25	51.74	4.126	2.278
SPAC977.05C	SPAC977.05c	0.4139	3.754	0.383104574	30.01	43.13	3.463	3.633
SPAC22H12.04C	rps102	0.2366	3.754	0.62598526	37.89	56.06	3.978	1.337
SPAC1B3.15C	SPAC1B3.15c	0.4048	3.766	0.392759496	32.13	46.58	5.902	2.013
SPBC11C11.06C	SPBC11C11.06	0.3598	3.77	0.443938841	35.96	52.87	5.372	1.745
SPBC17D11.08	SPBC17D11.08	0.05375	3.77	1.269621531	30.43	43.79	0.6498	1.27
SPAC1687.23C	SPAC1687.23c	0.378	3.778	0.4225082	35.08	51.42	5.221	2.332
SPAC1565.04C	ste4	0.6362	3.78	0.196406335	26.96	38.07	8.251	5.982
SPAC23D3.03C	SPAC23D3.03c	0.1956	3.78	0.70863115	32.63	47.38	3.716	0.8268
SPBC24C6.06	gpa1	0.6746	3.783	0.170953663	32.02	46.38	9.962	6.408
SPAC1610.04	mug99	0.7862	3.785	0.104466961	28.23	40.15	14.22	10.14
SPCC613.10	qcr2	0.5491	3.789	0.260348556	17.47	22.47	6.761	4.325
SPAC15E1.09	grx2	0.3198	3.801	0.495121541	27.07	38.23	4.428	2.222

SPAC1B3.17	clr2	0.4103	3.801	0.386898483	36.63	53.92	3.335	3.683
SPBC2D10.17	clr1	0.3518	3.802	0.453704165	33.39	48.6	5.059	2.094
SPBC15D4.03	slm9	0.5838	3.807	0.233735909	32.27	46.75	8.037	4.391
SPAC922.07C	SPAC922.07c	0.3684	3.815	0.433680378	29.07	41.49	2.75	3.417
SPAC22H10.13	zym1	0.2314	3.815	0.635636645	29.51	42.21	2.403	2.387
SPAC13G7.12C	SPAC13G7.12c	0.6532	3.817	0.184953824	20.3	27.07	8.1	6.366
SPAC3H5.09C	SPAC3H5.09c	0.3183	3.823	0.497163361	29.33	41.9	2.502	3.041
SPBC215.02	bob1	0.2376	3.823	0.624153564	33.28	48.39	4.085	1.316
SPBC4F6.16C	ero11	0.1566	3.832	0.805208242	30.17	43.26	2.191	1.908
SPAC869.08	pcm2	0.3701	3.841	0.431680915	35.17	51.47	5.58	1.861
SPAC11H11.01	sst6	0.198	3.847	0.70333481	31.03	44.65	0.8192	2.336
SPCC576.02	SPCC576.02	0.3378	3.848	0.471340355	34.89	50.99	4.031	2.756
SPAC1B3.02C	SPAC1B3.02c	0.5738	3.849	0.241239456	38.81	57.43	8.31	4.008
SPAC637.11	rpm2	0.3342	3.852	0.475993554	29.72	42.48	5.262	1.507
SPACUNK12.02C	cmk1	0.3262	3.852	0.486516043	35.36	51.75	4.573	2.269
SPCP1E11.03	mug170	0.2587	3.857	0.587203571	30.9	44.43	2.617	2.575
SPBP35G2.13C	swc2	0.1828	3.873	0.738023809	33.49	48.65	3.374	1.494
SPACUNK4.17	SPACUNK4.17	0.3227	3.875	0.491201035	33.12	48.04	5.18	1.414
SPBC3B9.06C	atg3	0.2844	3.888	0.546070408	29.77	42.52	1.715	2.936
SPCC16A11.07	coq10	0.3004	3.889	0.522300072	35.02	51.13	3.933	2.459
SPAC21E11.04	ppr1	0.3795	3.9	0.42078822	35.01	51.1	4.229	3.189
SPAP7G5.03	prm1	0.6143	3.914	0.211619485	29.13	41.42	7.26	5.856
SPCC338.16	pof3	0.625	3.914	0.204119983	15.15	18.45	7.634	5.982
SPCC285.14	trs130	0.5965	3.915	0.224389552	16.05	19.93	6.71	5.645
SPBC30B4.04C	sol1	0.02255	3.919	1.646853454	30.78	44.12	1.515	0.895
SPAC1556.08C	cbs2	0.459	3.92	0.338187314	25.04	34.69	5.502	3.905
SPAC1687.06C	rpl44	0.2073	3.92	0.683400698	29.18	41.48	1.716	2.403
SPCC1840.07C	SPCC1840.07c	0.202	3.924	0.694648631	33.26	48.18	3.741	1.394
SPAC3A11.14C	pkl1	0.4803	3.924	0.318487413	35.97	52.64	7.216	2.677
SPAC26A3.04	rpl2002	0.3486	3.93	0.457672617	28.57	40.47	4.478	2.74
SPAPB1A11.03	SPAPB1A11.03	0.3041	3.931	0.51698358	35.84	52.42	4.624	2.021
SPAC23H3.12C	SPAC23H3.12c	0.2691	3.934	0.570086302	34.01	49.4	4.417	1.673
SPBC1709.05	sks2	0.2521	3.935	0.598427154	28.78	40.81	2.313	2.652
SPAC664.15	caf4	0.3113	3.946	0.506820879	29.04	41.22	1.711	3.192
SPAC3F10.12C	SPAC3F10.12c	0.5096	3.952	0.292770581	33.58	48.66	6.072	4.248
SPBC4F6.11C	SPBC4F6.11c	0.2668	3.959	0.573814175	29.87	42.56	2.822	2.673
SPBC12C2.02C	ste20	0.2768	3.96	0.557833914	21.45	28.74	3.591	2.724
SPAC1A6.09C	lag1	0.5626	3.962	0.249800272	32.5	46.87	8.011	4.237
SPAC13F5.05	SPAC13F5.05	0.4457	3.964	0.350957366	33.66	48.78	5.463	3.541
SPAC3F10.10C	map3	0.2104	3.974	0.676954265	34.41	50	3.541	1.825
SPCC417.07C	mto1	0.1872	3.989	0.727694156	28.34	39.99	3.793	1.031
SPAC328.04	SPAC328.04	0.363	3.996	0.440093375	34.15	49.54	5.469	2.239
SPAC1071.04C	spc2	0.7473	3.997	0.126505018	17.34	21.92	12.75	8.933
SPBC211.06	gfh1	0.235	4.004	0.628932138	32.91	47.49	4.377	0.9868
SPAC3H5.07	rpl702	0.1856	4.007	0.731422028	22	29.55	3.213	1.831
SPBC2G2.03C	sbh1	0.758	4.009	0.120330794	16.84	21.08	12.28	9.879
SPAC1687.22C	puf3	0.2201	4.01	0.657379957	28.71	40.58	1.806	2.544
SPAC1250.05	rpl3002	0.421	4.018	0.375717904	35.8	52.21	6.096	2.76
SPAC1D4.01	SPAC1D4.01	0.5267	4.02	0.278436682	16.29	20.16	6.826	4.306
SPBC651.06	mug166	0.7154	4.023	0.145451064	26.77	37.36	14.03	6.052
SPAC13G6.09	SPAC13G6.09	0.4044	4.024	0.393188853	14.43	17.09	4.402	3.817
SPAC3G6.06C	rad2	0.3583	4.024	0.445753192	30.1	42.84	5.729	1.831
SPAC20H4.06C	SPAC20H4.06c	0.3458	4.025	0.461175011	24.95	34.38	3.053	3.361
SPAC22F8.07C	rtf1	0.1435	4.026	0.843148099	16.66	20.75	1.682	2.025
SPCC1450.03	SPCC1450.03	0.7738	4.033	0.111371275	21.89	29.33	13.76	10.44
SPBC839.03C	SPBC839.03c	0.2525	4.037	0.597738618	36.21	52.85	4.279	1.757
SPAC22A12.04C	rps2201	0.5174	4.042	0.286173576	23.87	32.57	6.027	4.571
SPAC343.06C	SPAC343.06c	0.2617	4.042	0.582196277	34.03	49.26	4.772	0.851
SPAC1071.06	arp9	0.04705	4.046	1.327440372	31.55	45.18	2.017	1.047
SPAC25B8.15C	SPAC25B8.15c	0.1987	4.053	0.701802133	34.69	50.33	4.014	0.9209
SPAC343.19	lsb6	0.3497	4.058	0.456304368	35.22	51.18	5.761	1.615
SPBC577.04	SPBC577.04	0.1964	4.066	0.706858517	32.08	46.01	4.018	0.8061
SPAC644.14C	rhp51	0.2155	4.069	0.666552726	34.21	49.52	4.156	1.21
SPBC15C4.01C	oca3	0.1365	4.075	0.864867349	33.13	47.72	2.133	1.923
SPAC3H1.03	mug151	0.1433	4.075	0.84375381	29.88	42.39	1.31	2.076
SPBC14C8.17C	spt8	0.342	4.079	0.465973894	24.06	32.82	4.232	2.99
SPAC869.03C	SPAC869.03c	0.2201	4.08	0.657379957	31.92	45.74	2.672	2.437
SPAC16E8.13	SPAC16E8.13	0.1722	4.084	0.763966853	33.24	47.89	3.602	1.314
SPBC651.12C	SPBC651.12c	0.2409	4.089	0.6181632	31.64	45.26	4.407	1.416

SPAC3G6.01	hrp3	0.2853	4.091	0.544698228	33.54	48.37	4.67	1.942
SPAC31G5.18C	sde2	0.1746	4.092	0.757955761	30.38	43.18	1.438	2.302
SPCC74.03C	ssp2	0.2085	4.094	0.680893941	38.29	56.18	4.184	0.8518
SPBC12C2.03C	SPBC12C2.03c	0.1538	4.094	0.813043665	30.85	43.95	2.752	1.862
SPAC31G5.17C	rps1001	0.1777	4.098	0.750312572	28.85	40.66	1.747	2.303
SPBC11C11.02	imp2	0.3066	4.098	0.513427849	32.03	45.88	3.323	3.002
SPCC24B10.21	tpi1	0.1479	4.101	0.830031826	31.83	45.55	2.18	2.016
SPCC622.17	apn1	0.2636	4.101	0.579054594	31.12	44.38	2.326	2.87
SPBC31F10.12	SPBC31F10.12	0.1385	4.107	0.858550227	32.85	47.21	3.246	1.249
SPAC29B12.14C	SPAC29B12.14c	0.6386	4.108	0.194771086	31.92	45.69	7.44	6.886
SPAC1D4.13	byr1	0.5363	4.109	0.270592203	27.91	39.09	6.793	4.983
SPCC1281.03C	emc4	0.2078	4.113	0.682354457	34.38	49.72	3.738	1.789
SPAC13F5.07C	SPAC13F5.07c	0.2585	4.114	0.587539453	30.01	42.53	1.775	2.906
SPAC23E2.03C	ste7	0.6158	4.114	0.210560315	29.02	40.91	6.95	6.766
SPAC23C11.13C	hpt1	0.5291	4.121	0.276462238	22.77	30.63	7.203	4.341
SPBC1271.12	kes1	0.4425	4.121	0.354086725	32.13	46.01	5.064	3.929
SPAC824.09C	SPAC824.09c	0.6653	4.123	0.176982477	23.32	31.54	10.64	6.346
SPBC2G2.14	SPBC2G2.14	0.172	4.128	0.764471553	33.83	48.79	3.32	1.719
SPAC3H8.07C	pac10	0.1413	4.137	0.849857838	35.08	50.83	3.406	0.9965
SPBC119.12	SPBC119.12	0.3541	4.147	0.450874073	30.09	42.61	5.624	2.203
SPAC9E9.12C	ybt1	0.2621	4.151	0.581532979	30.77	43.72	2.497	2.868
SPCC1840.03	sal3	0.6168	4.152	0.209855635	18.9	24.23	8.142	6.092
SPCC1442.01	ste6	0.7414	4.154	0.129947418	26.9	37.36	12.53	9.261
SPBC4.05	mlo2	0.2387	4.156	0.622147581	32.44	46.46	4.41	1.519
SPBC1198.12	mfr1	0.3066	4.157	0.513427849	30.55	43.36	2.635	3.219
SPBC342.05	crb2	0.1545	4.158	0.811071516	31.26	44.52	2.582	1.987
SPAC959.05C	SPAC959.05c	0.4459	4.162	0.350762528	31.36	44.68	6.49	3.205
SPCPJ732.01	vps5	0.701	4.169	0.154281982	20.88	27.45	9.718	8.422
SPAC1952.02	SPAC1952.02	0.6856	4.17	0.163929191	23.33	31.47	10.66	7.349
SPBC31F10.13C	hip1	0.153	4.171	0.815308569	34.82	50.34	3.275	1.554
SPBC1734.13	atp3	0.2176	4.172	0.662341109	29.32	41.31	2.01	2.613
SPBC1683.08	ght4	0.2927	4.175	0.533577278	34.95	50.54	4.655	2.22
SPCC306.09C	cap1	0.2462	4.183	0.608711951	20.97	27.57	1.713	2.868
SPBC1271.09	SPBC1271.09	0.2697	4.185	0.569119054	33.55	48.24	4.061	2.395
SPBC32H8.01C	SPBC32H8.01c	0.2267	4.187	0.64454848	35.84	52	4.229	1.622
SPCC757.04	SPCC757.04	0.2699	4.188	0.568797115	32.74	46.9	3.894	2.499
SPCC1020.09	gnr1	0.1353	4.19	0.868702203	32.55	46.59	2.043	2.001
SPAC1805.16C	SPAC1805.16c	0.1673	4.203	0.776504059	30.59	43.33	2.708	2.083
SPBC28E12.04	SPBC28E12.04	0.2537	4.208	0.595679533	31.59	44.98	3.856	2.366
SPBC16A3.01	spn3	0.1639	4.213	0.785421046	36.39	52.85	3.733	1.049
SPAPJ691.02	SPAPJ691.02	0.6636	4.215	0.178093623	30.21	42.7	10.25	6.782
SPAC664.13	SPAC664.13	0.1225	4.215	0.911863911	29	40.7	1.938	1.935
SPBC36B7.05C	SPBC36B7.05c	0.2085	4.218	0.680893941	32.93	47.16	4.331	0.6604
SPAC27D7.03C	mei2	0.4337	4.221	0.362810578	35.77	51.82	5.396	3.802
SPBC106.04	ada1	0.6593	4.234	0.180916924	20.94	27.45	9.248	7.159
SPAC24C9.02C	SPAC24C9.02c	0.2304	4.239	0.637517525	35.02	50.56	4.266	1.738
SPBP8B7.13	vac7	0.2357	4.24	0.627640417	36.11	52.35	4.298	1.792
SPAC8E11.03C	dmc1	0.09217	4.244	1.035410413	31.96	45.53	2.346	1.562
SPCC622.14	SPCC622.14	0.277	4.248	0.557520231	34.93	50.4	5.113	1.336
SPCC736.09C	SPCC736.09c	0.07579	4.25	1.120388093	36.58	53.11	2.37	1.358
SPCC306.07C	SPCC306.07c	0.3596	4.251	0.444180317	30.61	43.3	3.425	3.642
SPAC19B12.04	rps3001	0.1713	4.252	0.766242637	31.61	44.93	3.91	0.5633
SPAC1F3.07C	rsc58	0.5788	4.257	0.237471478	23.34	31.34	7.806	5.782
SPAP14E8.04	oma1	0.1096	4.261	0.960189446	29.97	42.22	1.048	1.921
SPAC22F8.04	SPAC22F8.04	0.09829	4.263	1.007490665	19.59	25.17	2.208	1.691
SPBC3H7.05C	SPBC3H7.05c	0.3349	4.267	0.475084852	30.33	42.8	5.593	2.099
SPAC15E1.02C	SPAC15E1.02c	0.7383	4.269	0.131767132	26.56	36.61	12.96	9.282
SPAC1486.02C	dsc2	0.6162	4.271	0.210278306	18.38	23.17	7.564	6.58
SPBC21C3.19	SPBC21C3.19	0.5122	4.282	0.290560426	27.76	38.56	5.712	5.013
SPAC17H9.09C	ras1	0.5746	4.282	0.240634378	26.36	36.26	7.768	5.75
SPBC428.11	SPBC428.11	0.7452	4.29	0.127727154	28.07	39.06	13.45	9.563
SPAC1F5.05C	SPAC1F5.05c	0.1848	4.293	0.733298033	33.82	48.5	3.55	1.871
SPBC359.04C	SPBC359.04c	0.2035	4.293	0.691435586	30.15	42.47	2.569	2.49
SPAC589.05C	SPAC589.05c	0.1948	4.303	0.710411047	35.52	51.28	3.799	1.793
SPAPB17E12.14C	SPAPB17E12.14	0.112	4.306	0.950781977	32.79	46.79	1.548	1.947
SPAC21E11.05C	cyp8	0.1951	4.306	0.709742731	35.22	50.78	3.808	1.791
SPAC26F1.05	mug106	0.09439	4.307	1.025074014	33.68	48.25	2.131	1.704
SPAC11G7.04	ubi1	0.4285	4.31	0.368049174	19.82	25.47	6.573	3.086
SPBC16E9.18	psd1	0.7371	4.311	0.132473589	27.9	38.74	12.52	9.565

SPBC1271.15C	SPBC1271.15c	0.1451	4.323	0.838332588	33.23	47.48	2.361	2.086
SPCC737.03C	ima1	0.0286	4.324	1.543633967	33.27	47.54	1.986	0.615
SPAC1834.03C	hhf1	0.1453	4.324	0.837734386	30.67	43.28	1.046	2.224
SPBC29A10.06C	ely5	0.2684	4.335	0.571217489	31.28	44.27	2.632	3.044
SPBC365.12C	ish1	0.1921	4.344	0.716472635	30.53	43.01	2.853	2.353
SPBC9B6.03	SPBC9B6.03	0.2215	4.345	0.654626269	34.94	50.25	2.819	2.614
SPAC23A1.11	rpl1602	0.1928	4.36	0.71489297	31.38	44.38	1.605	2.586
SPAC1805.03C	trm13	0.2168	4.368	0.663940722	34.3	49.16	3.773	2.176
SPAC1805.04	nup132	0.3141	4.372	0.502932064	30.72	43.28	5.312	2.245
SPAC652.01	SPAC652.01	0.5813	4.374	0.235599677	30.08	42.22	7.543	6.221
SPBC31F10.09C	nut2	0.611	4.375	0.21395879	18.02	22.42	7.867	6.552
SPAC922.03	SPAC922.03	0.2197	4.376	0.658169943	30.94	43.64	2.725	2.647
SPAC17C9.15C	SPAC17C9.15c	0.2387	4.376	0.622147581	29.5	41.28	4.756	1.348
SPBC1778.02	rap1	0.1706	4.381	0.768020973	32.68	46.48	3.935	1.197
SPBC1105.11C	hht3	0.2991	4.387	0.524183587	29.78	41.71	2.945	3.298
SPBC16C6.01C	SPBC16C6.01c	0.3774	4.391	0.423198104	16.58	20.02	4.633	3.887
SPAC2C4.16C	rps801	0.5523	4.392	0.257824957	16.18	19.37	6.623	5.643
SPCPB1C11.01	amt1	0.1797	4.4	0.745451923	29.22	40.77	2.874	2.278
SPAC56F8.16	esc1	0.2245	4.406	0.648783655	35.53	51.12	4.232	1.941
SPAC15A10.11	ubr11	0.3275	4.422	0.484788696	26.3	35.94	4.828	2.927
SPBPJ4664.03	mfm3	0.153	4.43	0.815308569	33.19	47.24	3.567	1.549
SPBC56F2.01	pof12	0.7601	4.438	0.119129267	18.87	23.71	13.75	11.02
SPBC365.02C	cox10	0.05771	4.442	1.238748926	31.16	43.89	0.7627	1.536
SPBC428.06C	rxt2	0.7265	4.443	0.138764381	22.43	29.55	12.4	9.449
SPAC15A10.03C	rhp54	0.5907	4.451	0.228633029	18.41	22.93	7.718	6.234
SPAC5D6.07C	pxa1	0.1317	4.454	0.880414225	30.42	42.64	2.034	2.122
SPCC126.15C	sec65	0.7277	4.454	0.138047625	19.15	24.14	11.59	9.885
SPBC15D4.15	pho2	0.1841	4.457	0.734946211	34.58	49.49	4.197	1.151
SPAC1786.04	SPAC1786.04	0.2152	4.469	0.667157733	36.25	52.21	4.249	1.861
SPAC14C4.03	mek1	0.3063	4.476	0.513853003	28.95	40.21	2.767	3.475
SPBC31F10.10C	SPBC31F10.10c	0.2896	4.484	0.538201442	33.84	48.23	3.05	3.274
SPBC3H7.11	SPBC3H7.11	0.3467	4.487	0.460046158	32.86	46.6	5.986	2.34
SPBP4H10.12	SPBP4H10.12	0.2444	4.499	0.611898798	34.2	48.79	4.492	2.125
SPAC19A8.05C	sst4	0.2748	4.502	0.560983272	35.56	51.01	3.562	3.01
SPAC31A2.11C	cuf1	0.1309	4.509	0.883060353	35.47	50.86	2.375	2.076
SPCC4B3.13	SPCC4B3.13	0.2536	4.518	0.595850751	30.62	42.88	2.038	3.143
SPBC16A3.07C	nrm1	0.6185	4.519	0.208660296	18.58	23.09	8.799	6.712
SPBC3E7.08C	rad13	0.07151	4.521	1.145633222	31.41	44.17	1.672	1.692
SPBC660.17C	SPBC660.17c	0.2533	4.524	0.59636481	34.91	49.92	4.804	1.973
SPCC736.14	dis1	0.7345	4.528	0.1340082	28.54	39.44	13.26	9.831
SPAPB1E7.05	gde1	0.07793	4.532	1.108295324	30.64	42.89	0.9365	1.768
SPBC1778.01C	zuo1	0.3362	4.535	0.473402291	16.41	19.51	4.396	3.666
SPBC215.06C	SPBC215.06c	0.3701	4.538	0.431680915	34.37	49	6.97	1.237
SPAC22H10.07	scd2	0.63	4.549	0.200659451	19.89	25.2	9.022	7.053
SPCC24B10.15	SPCC24B10.15	0.2469	4.56	0.60747891	36.03	51.7	4.385	2.336
SPAC16E8.06C	nop12	0.1556	4.568	0.807990407	34.18	48.65	3.9	1.264
SPBC16A3.06	SPBC16A3.06	0.5233	4.569	0.281249265	30.87	43.2	8.417	4.368
SPAC212.04C	SPAC212.04c	0.2527	4.575	0.597394758	34.5	49.16	4.814	2.039
SPBC685.06	rps001	0.5899	4.577	0.229221604	17.41	21.08	7.848	6.423
SPAC139.04C	fap2	0.1681	4.579	0.774432287	30.69	42.88	2.064	2.483
SPAC2F7.03C	pom1	0.08316	4.581	1.080085519	23.59	31.22	2.454	1.619
SPBC776.06C	SPBC776.06c	0.7248	4.596	0.139781815	25.58	34.46	12.65	9.748
SPAC2G11.07C	ptc3	0.08861	4.6	1.052517263	30.96	43.3	0.9097	1.888
SPAC13C5.03	tht1	0.5215	4.611	0.282745687	29.49	40.87	7.315	5.407
SPCC663.12	cid12	0.5863	4.621	0.231880106	19.29	24.09	7.519	6.545
SPAC13G7.03	upf3	0.2186	4.632	0.660349842	29.76	41.28	3.868	2.413
SPAC20G4.01	caf16	0.03969	4.636	1.401318901	31.87	44.73	1.264	1.436
SPAC1399.02	SPAC1399.02	0.4803	4.639	0.318487413	41.86	61.14	3.909	5.487
SPAC323.07C	SPAC323.07c	0.571	4.65	0.243363892	26.21	35.42	8.556	6.084
SPAC22F8.11	plc1	0.2702	4.661	0.568314655	24.57	32.71	5.034	2.251
SPBC19G7.16	iws1	0.4964	4.681	0.304168227	17.11	20.41	5.719	5.686
SPAC8C9.05	SPAC8C9.05	0.2344	4.684	0.630042393	36.25	51.85	4.738	1.967
SPAC4C5.02C	ryh1	0.06345	4.688	1.197568374	18.22	22.23	2.716	1.131
SPAC4F10.05C	SPAC4F10.05c	0.1084	4.693	0.964970718	5.739	1.718	1.386	2.105
SPCC18B5.03	wee1	0.0407	4.715	1.390405591	23.58	30.99	2.409	0.8736
SPBC146.10	mug57	0.1954	4.715	0.709075441	31.7	44.32	2.261	2.767
SPCC553.12C	SPCC553.12c	0.204	4.716	0.690369833	28.33	38.79	2.14	2.855
SPBC17A3.06	SPBC17A3.06	0.07352	4.723	1.133594502	21.86	28.14	2.633	1.474
SPAPB21F2.02	SPAPB21F2.02	0.232	4.748	0.634512015	38.15	54.86	5.163	1.089

SPAC5D6.13	SPAC5D6.13	0.1373	4.754	0.862329463	29.97	41.42	3.904	0.6458
SPAC13G7.07	arb2	0.1505	4.758	0.8224635	28.88	39.62	2.508	2.367
SPAC15A10.06	SPAC15A10.06	0.2457	4.767	0.609594844	6.971	3.621	2.452	3.211
SPCC18.13	SPCC18.13	0.04381	4.777	1.358426747	27.85	37.9	2.078	1.382
SPAC22F3.08C	rok1	0.4489	4.779	0.347850395	20.79	26.3	6.331	4.456
SPBC1711.12	SPBC1711.12	0.34	4.78	0.468521083	33.06	46.45	3.158	4.022
SPBC14F5.03C	kap123	0.106	4.794	0.974694135	27.46	37.23	0.6897	2.113
SPCC962.05	ast1	0.07278	4.8	1.137987949	31.2	43.37	1.034	1.825
SPCC777.08C	bit61	0.05235	4.801	1.281083314	31.92	44.54	0.9868	1.616
SPBC2F12.15C	pfa3	0.2376	4.801	0.624153564	26.65	35.9	2.118	3.203
SPAC23H3.05C	swd1	0.2299	4.808	0.638461029	32.61	45.66	5.185	1.176
SPAC3A12.17C	cys12	0.07067	4.832	1.150764909	32.3	45.11	1.086	1.819
SPCC74.05	rpl2702	0.5531	4.841	0.257196342	22.53	29.05	8.943	5.456
SPCC285.17	spp27	0.1714	4.863	0.765989182	29.93	41.18	2.033	2.683
SPBC8D2.17	SPBC8D2.17	0.6855	4.867	0.163992541	26.2	35.03	11.84	8.863
SPAC167.06C	mug143	0.2216	4.87	0.654430244	31.7	44.07	5.155	1.066
SPAC15E1.07C	moa1	0.7103	4.894	0.148558185	22.8	29.41	13.1	9.674
SPBC1773.01	SPBC1773.01	0.2749	4.895	0.56082526	32.88	45.97	4.159	3.167
SPBC4C3.08	mug136	0.05343	4.909	1.272214826	31.66	43.94	2.623	1.23
SPCC1902.01	gaf1	0.0786	4.91	1.104577454	32.48	45.29	1.974	1.886
SPAC16.05C	sfp1	0.09008	4.91	1.045371622	28.29	38.4	0.5013	2.003
SPAC22H10.04	SPAC22H10.04	0.1073	4.911	0.969400278	35.21	49.76	3.153	1.74
SPCC4B3.05C	hem12	0.2425	4.916	0.615288257	32.19	44.79	5.175	1.965
SPAC27F1.05C	SPAC27F1.05c	0.04904	4.923	1.309449538	32.65	45.54	1.775	1.602
SPBC3B8.06	SPBC3B8.06	0.5644	4.926	0.248412995	37.48	53.47	10.18	5.157
SPBC2A9.07C	SPBC2A9.07c	0.09037	4.942	1.043975718	30.74	42.37	2.216	1.974
SPCC24B10.09	rps1702	0.3739	4.966	0.427244535	22.86	29.4	6.507	3.301
SPCC24B10.17	emp24	0.1395	4.967	0.855425792	28.51	38.67	1.999	2.471
SPAC637.13C	SPAC637.13c	0.6826	4.967	0.165833716	43.95	64.03	14.89	7.136
SPAC5D6.06C	alg14	0.521	4.973	0.283162277	26.71	35.7	8.119	5.705
SPAP7G5.05	rpl1002	0.5543	4.974	0.256255121	30.51	41.94	8.141	6.181
SPCC188.02	par1	0.5983	4.984	0.223080997	21.05	26.39	8.922	7.088
SPAC11E3.11C	SPAC11E3.11c	0.3608	4.991	0.442733471	24.24	31.62	5.768	3.623
SPCC1739.15	wtf21	0.1276	4.991	0.894149326	5.936	1.552	1.552	2.407
SPAC17H9.10C	ddb1	0.4509	4.995	0.345919765	23.78	30.86	7.464	4.207
SPAC15A10.07	SPAC15A10.07	0.5182	5.001	0.285502591	27.15	36.39	7.828	5.502
SPCC550.12	arp6	0.264	5.017	0.578396073	30.89	42.49	5.809	1.638
SPBC19G7.06	mbx1	0.2846	5.029	0.545765104	28.24	38.13	3.996	3.464
SPBC354.04	SPBC354.04	0.1676	5.036	0.775725986	36.04	50.92	4.063	2.016
SPAC29A4.18	prw1	0.5005	5.069	0.300595918	30.03	41	6.779	6.026
SPBC26H8.11C	SPBC26H8.11c	0.3306	5.072	0.480697151	32.18	44.53	5.025	3.671
SPAC23H3.04	SPAC23H3.04	0.2041	5.082	0.690156995	34.88	48.95	4.573	2.192
SPAC1006.04C	mcp3	0.05852	5.088	1.232695683	31.49	43.36	2.681	1.436
SPBC651.04	SPBC651.04	0.2268	5.088	0.64435695	38.34	54.62	5.124	1.998
SPBC26H8.13C	SPBC26H8.13c	0.07579	5.11	1.120388093	34	47.45	3.216	1.147
SPAC27D7.04	omt2	0.1632	5.111	0.787279846	31.66	43.61	4.496	1.354
SPBC2A9.11C	SPBC2A9.11c	0.143	5.127	0.844663963	32.83	45.5	2.589	2.504
SPBC776.05	SPBC776.05	0.6607	5.132	0.179995693	24.98	32.6	11.14	8.769
SPAC11D3.18C	SPAC11D3.18c	0.5321	5.142	0.274006741	28.59	38.52	8.661	6.047
SPAC1296.05C	SPAC1296.05c	0.1421	5.151	0.847405922	32.52	44.95	1.273	2.62
SPCC364.02C	bis1	0.3236	5.154	0.489991487	34.28	47.84	6.115	2.987
SPCC1393.09C	SPCC1393.09c	0.1833	5.157	0.736837535	36.15	50.9	4.201	2.276
SPAC1486.08	SPAC1486.08	0.1454	5.158	0.837435593	35.28	49.49	3.084	2.412
SPBC3E7.06C	fnx2	0.1636	5.16	0.786216701	36.45	51.39	4.052	2.1
SPAC6B12.02C	mus7	0.209	5.165	0.679853714	37.33	52.83	4.243	2.608
SPAC1F8.05	isp3	0.2743	5.168	0.561774192	29.92	40.66	3.965	3.489
SPBC3H7.13	SPBC3H7.13	0.1544	5.177	0.811352704	31.85	43.81	1.791	2.731
SPAC23C11.10	SPAC23C11.10	0.1298	5.18	0.886725308	31.43	43.12	2.149	2.482
SPBC16D10.02	trm11	0.04622	5.185	1.335170059	32.75	45.28	0.8096	1.648
SPBC530.03C	bag102	0.1102	5.187	0.957818405	35.44	49.7	3.844	0.6695
SPAC1952.07	rad1	0.1263	5.19	0.898596649	30.26	41.18	2.549	2.388
SPAC959.08	rpl2102	0.4718	5.203	0.326242063	23.96	30.81	8.813	4.088
SPBC27.02C	ask1	0.1298	5.206	0.886725308	31.61	43.37	3.939	1.639
SPBC21C3.13	rps1901	0.6039	5.219	0.21903497	21.93	27.45	8.627	7.866
SPAC6F12.04	SPAC6F12.04	0.06688	5.222	1.174703736	31.17	42.63	1.021	1.917
SPBC1604.08C	imp1	0.01149	5.233	1.939679971	29.2	39.36	1.867	0.8143
SPBC4.06	SPBC4.06	0.1553	5.236	0.808828544	35.12	49.09	2.777	2.648
SPAC13C5.02	dre4	0.3285	5.242	0.483464626	12.06	11.2	4.328	4.08
SPAC2F3.15	lsk1	0.3001	5.243	0.522734005	27.48	36.53	5.835	2.936

SPCC895.07	alp14	0.2418	5.253	0.616543703	35.13	49.08	4.183	3.121
SPBC725.03	SPBC725.03	0.6622	5.261	0.179010824	25.92	33.94	11.51	9.016
SPAC57A7.13	SPAC57A7.13	0.1252	5.261	0.902395671	32.67	45.02	2.078	2.488
SPCC736.06	SPCC736.06	0.4896	5.269	0.310158591	19.33	23.1	7.745	5.38
SPAC3G9.05	SPAC3G9.05	0.1313	5.284	0.881735274	31.82	43.59	2.468	2.505
SPCC895.05	for3	0.4684	5.288	0.329383114	16.78	18.88	5.447	5.839
SPAC2C4.07C	SPAC2C4.07c	0.1407	5.299	0.851705903	33.52	46.35	1.875	2.667
SPCC1840.12	SPCC1840.12	0.07077	5.301	1.150150804	30.27	41.01	1.84	1.988
SPAC23C4.09C	SPAC23C4.09c	0.4077	5.303	0.389659289	44.33	64.1	3.206	5.356
SPAC8C9.07	SPAC8C9.07	0.211	5.311	0.675717545	25.36	32.94	4.942	2.25
SPBC23E6.01C	SPBC23E6.01c	0.4815	5.317	0.317403709	26.24	34.37	5.896	5.993
SPBC18H10.20C	SPBC18H10.20	0.1065	5.321	0.972650392	35.06	48.85	2.393	2.292
SPAC1687.13C	csn5	0.1814	5.333	0.741362717	31.91	43.66	2.73	2.97
SPBC1604.18C	SPBC1604.18c	0.1579	5.333	0.80161787	31.94	43.71	2.923	2.701
SPAC27E2.01	SPAC27E2.01	0.101	5.337	0.995678626	34.57	48.02	3.645	1.526
SPBC530.01	gyp1	0.2464	5.337	0.608359297	29.92	40.38	4.704	3.01
SPBC1734.07C	SPBC1734.07c	0.2613	5.341	0.58286059	34.79	48.37	4.856	3.131
SPAC644.13C	SPAC644.13c	0.1481	5.342	0.829444941	34.71	48.24	4.522	1.195
SPBC2G2.10C	mug110	0.1678	5.348	0.775208044	36.67	51.45	4.088	2.704
SPBC776.02C	dis2	0.1844	5.363	0.734239083	30.78	41.75	4.105	2.558
SPAC23E2.01	fep1	0.512	5.365	0.290730039	36.24	50.71	5.708	6.695
SPCC1223.05C	rpl3702	0.2464	5.369	0.608359297	20.48	24.82	5.01	2.856
SPBP8B7.10C	SPBP8B7.10c	0.13	5.374	0.886056648	32.91	45.22	4.051	1.72
SPAC11D3.16C	SPAC11D3.16c	0.1214	5.384	0.915781313	31.66	43.17	1.635	2.54
SPCP31B10.02	SPCP31B10.02	0.07264	5.389	1.138824164	36.2	50.6	3.378	0.8882
SPBC1683.07	mal1	0.05148	5.416	1.288361462	40.71	57.98	2.979	1.09
SPBC16C6.04	SPBC16C6.04	0.6495	5.423	0.187420845	25.61	33.16	11.19	9.016
SPAC23H4.16C	SPAC23H4.16c	0.03603	5.427	1.443335738	30.71	41.53	2.456	1.345
SPAC17A5.02C	dbr1	0.4286	5.43	0.367947833	17.08	19.13	5.789	5.261
SPAC1687.15	gsk3	0.2118	5.436	0.674074044	30.81	41.68	5.345	1.933
SPBC13E7.03C	SPBC13E7.03c	0.06194	5.463	1.208028799	32.15	43.84	1.393	1.965
SPBC1685.11	rlp1	0.08168	5.468	1.087884271	36.39	50.79	3.511	1.33
SPAC25G10.02	cce1	0.2018	5.477	0.695078838	31.07	42.03	4.975	2.27
SPCC1223.13	cbf12	0.05416	5.477	1.266321344	33.07	45.32	2.388	1.75
SPCC1281.04	SPCC1281.04	0.1411	5.482	0.850472986	26.9	35.18	4.449	1.511
SPCC965.13	SPCC965.13	0.2258	5.486	0.646276062	37.34	52.33	5.754	1.681
SPBC21H7.06C	SPBC21H7.06c	0.1775	5.491	0.750801643	37.23	52.13	3.535	2.836
SPAPB1A10.09	ase1	0.08358	5.492	1.077897633	34.32	47.35	3.629	1.046
SPAC22F3.12C	rgs1	0.6814	5.505	0.166597871	28.82	38.3	13.18	9.899
SPAC13D6.02C	byr3	0.07806	5.514	1.107571453	24.57	31.3	2.162	2.121
SPAC17H9.13C	SPAC17H9.13c	0.06869	5.517	1.163106484	33.64	46.2	3.386	0.885
SPBC887.17	SPBC887.17	0.1204	5.519	0.919373513	35.06	48.52	3.096	2.366
SPAC2C4.09	SPAC2C4.09	0.1691	5.52	0.771856392	33.37	45.74	3.018	2.913
SPAC27E2.07	pvg2	0.0404	5.522	1.393618635	30.81	41.53	2.227	1.609
SPAC19A8.03	SPAC19A8.03	0.05808	5.547	1.235973392	28.31	37.38	3.114	1.316
SPAC1B3.08	SPAC1B3.08	0.1798	5.551	0.745210313	34	46.73	5.099	1.62
SPCC126.10	iah1	0.1235	5.559	0.908333042	36.43	50.7	4.299	1.306
SPBC215.03C	csn1	0.1413	5.571	0.849857838	23.83	29.99	4.036	2.176
SPCC736.07C	SPCC736.07c	0.4583	5.576	0.338850143	20.63	24.72	8.407	4.827
SPAC22F3.13	tsc1	0.2609	5.579	0.583525921	38.93	54.78	4.793	3.405
SPBC216.03	SPBC216.03	0.6507	5.582	0.186619193	20.44	24.41	11.92	9.159
SPAC4G9.20C	SPAC4G9.20c	0.1331	5.605	0.875821945	30.82	41.41	2.592	2.68
SPAC11E3.06	map1	0.225	5.606	0.647817482	38.24	53.6	5.797	1.88
SPCC663.09C	SPCC663.09c	0.03275	5.611	1.484788696	32.4	44	1.721	1.625
SPBC1105.08	SPBC1105.08	0.1166	5.63	0.93330145	31.19	41.99	3.654	2.117
SPAC637.06	SPAC637.06	0.6075	5.638	0.216453718	22.15	27.13	10.56	8.145
SPBC337.03	SPBC337.03	0.07729	5.653	1.111876693	31.94	43.19	1.597	2.209
SPAC14C4.11	SPAC14C4.11	0.1697	5.67	0.770318158	37.33	52.01	2.621	3.082
SPBC800.04C	rpl4301	0.1407	5.672	0.851705903	7.239	2.573	2.573	2.798
SPBC16E9.14C	zrg17	0.163	5.678	0.787812396	35.29	48.65	4.159	2.52
SPCC1795.09	yps1	0.08584	5.679	1.066310291	33.78	46.16	2.491	2.227
SPCC1442.05C	SPCC1442.05c	0.2795	5.687	0.553618188	25.91	33.21	5.048	3.65
SPAC2F7.04	pmc2	0.0506	5.693	1.295849483	9.069	5.546	2.157	2.247
SPAC29A4.05	cam2	0.2043	5.729	0.689731633	31.38	42.13	5.655	1.72
SPBC530.11C	SPBC530.11c	0.1011	5.774	0.995248844	34.31	46.87	3.51	2.081
SPAC5D6.09C	mug86	0.02668	5.78	1.573814175	32.31	43.58	1.384	1.585
SPAC869.07C	mel1	0.08293	5.783	1.081288335	33.21	45.04	2.031	2.307
SPAC9G1.10C	SPAC9G1.10c	0.06339	5.784	1.197979248	30.94	41.33	1.286	2.091
SPAC513.07	SPAC513.07	0.06747	5.792	1.17088929	33.48	45.48	1.877	2.143

SPCC594.05C	spf1	0.1287	5.795	0.890421453	33.73	45.88	4.601	1.183
SPAC1805.02C	SPAC1805.02c	0.04757	5.799	1.322666849	36.63	50.64	2.379	1.798
SPAC688.06C	slx4	0.01815	5.827	1.741123371	32.91	44.48	1.49	1.434
SPAC328.09	SPAC328.09	0.08354	5.834	1.078105529	35.4	48.57	3.117	2.072
SPAC4G8.13C	prz1	0.67	5.84	0.173925197	29.5	38.86	12.94	10.34
SPBC1683.06C	SPBC1683.06c	0.09978	5.851	1.0009565	35.74	49.09	1.956	2.526
SPAC824.04	SPAC824.04	0.06402	5.852	1.19368433	36.46	50.28	3.24	1.637
SPAC688.11	end4	0.3086	5.856	0.510604078	16.14	16.89	4.844	4.286
SPAC7D4.12C	SPAC7D4.12c	0.2352	5.861	0.628562683	36.61	50.51	4.952	3.26
SPBC1711.08	SPBC1711.08	0.03626	5.874	1.4405722	34.61	47.2	2.668	1.453
SPBC1703.12	ubp9	0.1512	5.877	0.820448209	31.61	42.26	2.906	2.97
SPBC11B10.10C	pht1	0.2648	5.877	0.577082019	21.94	26.38	5.011	3.658
SPAC19B12.12C	yip11	0.2416	5.895	0.61690307	35.45	48.55	5.166	3.27
SPBC16A3.19	eaf7	0.09821	5.907	1.007844289	36.68	50.55	3.711	1.99
SPCC4B3.08	lsg1	0.1184	5.913	0.926648298	36.44	50.14	4.525	1.144
SPCC584.13	SPCC584.13	0.08644	5.919	1.063285242	30.94	41.1	2.072	2.401
SPBC3H7.06C	pof9	0.1001	5.921	0.999565923	31.72	42.38	3.522	2.166
SPAC13D6.01	pof14	0.1233	5.929	0.909036923	31.65	42.25	2.398	2.777
SPAC1F3.06C	spo15	0.2239	5.937	0.649945906	34.03	46.14	1.541	3.874
SPAC4G8.10	gos1	0.3055	5.941	0.514988785	29.73	39.08	5.321	4.163
SPAC4H3.02C	swc3	0.5722	5.951	0.242452147	32.02	42.82	10.49	7.629
SPBC21.02	SPBC21.02	0.4562	5.972	0.340844719	16.62	17.49	5.846	6.438
SPAC2E1P5.02C	mug109	0.1366	5.973	0.864549301	33.41	45.07	4.809	1.534
SPAC27D7.09C	SPAC27D7.09c	0.2013	5.986	0.696156225	38.83	53.95	5.686	2.142
SPAC6G10.02C	tea3	0.0902	5.987	1.044793462	30.62	40.46	2.302	2.452
SPAC6B12.04C	SPAC6B12.04c	0.07378	5.988	1.132061349	30.82	40.79	1.629	2.299
SPBC106.03	SPBC106.03	0.1965	5.997	0.706637445	32.54	43.6	5.357	2.469
SPBC359.05	abc3	0.1034	6.005	0.985479461	33.09	44.49	3.546	2.263
SPAC15A10.09C	SPAC15A10.09c	0.4645	6.011	0.333014282	30.54	40.29	7.6	6.102
SPBP18G5.03	toc1	0.06101	6.048	1.214598975	33.03	44.31	2.064	2.148
SPAP8A3.14C	SPAP8A3.14c	0.1303	6.05	0.885055584	31.47	41.76	4.844	1.096
SPAC4H3.05	srs2	0.6304	6.054	0.200383796	28.24	36.44	12.56	9.153
SPBC1539.07C	SPBC1539.07c	0.2811	6.059	0.551139154	33.27	44.7	5.085	4.032
SPCC1739.14	npp106	0.5035	6.069	0.298000525	20.52	23.73	7.989	6.932
SPAC926.05C	dph4	0.03113	6.072	1.506820879	36.81	50.49	2.725	1.357
SPBC29A3.05	vps71	0.04064	6.077	1.391046301	33.17	44.5	2.868	1.539
SPBC146.12	coq6	0.3046	6.085	0.516270101	36.83	50.5	4.579	4.53
SPCC16A11.01	SPCC16A11.01	0.06029	6.098	1.219754716	31.23	41.28	3.302	1.683
SPBC18E5.04	rpl1001	0.4189	6.101	0.37788964	23.32	28.28	6.473	5.744
SPAC1805.01C	ppk6	0.07391	6.107	1.131296798	34.2	46.15	3.448	1.88
SPAC57A7.09	SPAC57A7.09	0.1282	6.12	0.892111975	37.29	51.19	3.598	2.664
SPBC119.05C	SPBC119.05c	0.07918	6.128	1.101384503	33.53	45.02	1.935	2.413
SPAC5D6.05	Sep-11	0.4096	6.131	0.387640052	25.27	31.43	7.13	5.74
SPCC320.03	SPCC320.03	0.006592	6.137	2.180982801	34.22	46.13	1.834	1.002
SPBC16H5.07C	ppa2	0.01006	6.142	1.997402019	29.51	38.38	1.388	1.298
SPBC359.03C	aat1	0.1838	6.164	0.735654493	32.95	44	1.787	3.58
SPBC25H2.14	mug16	0.5895	6.201	0.229516191	30.38	39.72	13.99	6.722
SPBC19G7.10C	SPBC19G7.10c	0.01323	6.202	1.878440156	22.9	27.43	0.6833	1.299
SPBP22H7.05C	abo2	0.1678	6.206	0.775208044	34.19	45.97	1.234	3.436
SPBC13G1.14C	SPBC13G1.14c	0.6327	6.215	0.198802166	28.25	36.19	12.44	9.707
SPAC31G5.09C	spk1	0.2658	6.254	0.575445023	33.18	44.22	5.796	4.076
SPAC323.05C	SPAC323.05c	0.1952	6.265	0.709520187	20.01	22.57	4.674	3.21
SPAC767.01C	vps1	0.07715	6.275	1.11266407	32.01	42.27	1.426	2.444
SPBC1105.04C	cbp1	0.08509	6.318	1.070121476	37.28	50.86	3.075	2.387
SPBC582.06C	mcp6	0.628	6.321	0.202040356	27.41	34.63	13.41	9.3
SPCC663.14C	SPCC663.14c	0.1132	6.324	0.946153573	31.95	42.1	3.378	2.674
SPBC1685.15C	klp6	0.03085	6.337	1.510744832	31.99	42.13	1.628	1.816
SPAC15E1.05C	SPAC15E1.05c	0.5759	6.339	0.239652921	28.05	35.67	12.35	7.642
SPBC9B6.11C	SPBC9B6.11c	0.1392	6.354	0.856360765	34.56	46.33	2.469	3.166
SPAC1952.15C	rec24	0.01434	6.366	1.843450849	31.76	41.72	0.9525	1.417
SPAC1142.07C	vps32	0.3335	6.369	0.476904162	14.63	13.57	5.61	4.923
SPAC23G3.08C	ubp7	0.02454	6.378	1.610125442	33.27	44.17	1.426	1.703
SPCC1739.01	SPCC1739.01	0.2166	6.408	0.664341548	7.553	1.88	1.88	4.092
SPAC824.05	vps16	0.3878	6.453	0.411392195	25.66	31.56	7.127	5.772
SPBC16C6.05	SPBC16C6.05	0.04135	6.464	1.383524486	32.83	43.31	3.331	0.9958
SPBC19G7.03C	rps3002	0.04992	6.466	1.301725423	32.86	43.35	3.466	1.446
SPBC4F6.06	kin1	0.03629	6.467	1.440213032	17.41	17.97	1.834	1.945
SPAC186.05C	SPAC186.05c	0.04432	6.535	1.353400248	33.42	44.16	3.237	1.622
SPBC1773.04	SPBC1773.04	0.09708	6.568	1.012870232	36.39	48.99	3.512	2.546

SPBC56F2.14	mrpl44	0.2104	6.569	0.676954265	34.63	46.09	2.561	4.089
SPCC550.09	SPCC550.09	0.1566	6.58	0.805208242	32.55	42.67	3.239	3.393
SPBC25B2.11	pof2	0.08523	6.599	1.069407512	34.95	46.57	3.738	2.258
SPAC57A10.04	mug10	0.07396	6.644	1.131003098	35.59	47.54	2.569	2.505
SPCC16A11.16C	rpn1302	0.3421	6.661	0.465846926	34.28	45.36	6.803	4.933
SPCC1223.15C	spc19	0.02835	6.673	1.547446937	33.56	44.16	3.028	1.259
SPAC19G12.03	cda1	0.2116	6.684	0.674484337	37.91	51.29	4.299	3.904
SPCC1020.12C	xap5	0.1979	6.705	0.703554206	31.23	40.29	4.542	3.662
SPAC27E2.03C	SPAC27E2.03c	0.001995	6.729	2.7000571	32.23	41.88	1.568	0.5451
SPAC15A10.15	sgo2	0.5005	6.759	0.300595918	28.56	35.82	10.5	6.929
SPBP8B7.28C	stc1	0.1414	6.796	0.849550591	32.41	42.07	3.416	3.302
SPCC16C4.01	sif2	0.1634	6.859	0.786747948	32.46	42.05	2.211	3.733
SPAC22F3.04	mug62	0.02311	6.907	1.636200055	33.45	43.59	2.515	1.681
SPBC354.07C	SPBC354.07c	0.0546	6.916	1.262807357	34.15	44.73	3.785	1.647
SPAC694.05C	rps2502	0.1763	6.923	0.753747688	39.82	54.04	3.378	3.818
SPBC582.10C	SPBC582.10c	0.06514	6.944	1.186152246	35.6	47.06	2.787	2.469
SPCC553.08C	SPCC553.08c	0.5302	6.999	0.275560277	32.07	41.19	11.69	7.729
SPAC824.02	SPAC824.02	0.4627	7	0.334700501	25.2	29.89	10.16	6.43
SPAC589.09	SPAC589.09	0.06909	7.06	1.160584807	35.3	46.39	3.646	2.295
SPCC364.04C	SPCC364.04c	0.04013	7.098	1.39653084	42.14	57.56	3.623	1.211
SPAC1851.03	ckb1	0.1363	7.134	0.865504144	33.72	43.67	4.433	3.133
SPAC1952.08C	SPAC1952.08c	0.06139	7.138	1.211902367	35.91	47.26	2.098	2.56
SPAC1B3.01C	SPAC1B3.01c	0.006346	7.144	2.197499932	32.78	42.1	2	1.245
SPAC20G4.07C	sts1	0.1768	7.186	0.752517739	22.59	25.3	6.159	2.728
SPAC20G8.02	SPAC20G8.02	0.04316	7.194	1.364918564	32.6	41.73	2.436	2.258
SPCC162.03	SPCC162.03	0.0235	7.222	1.628932138	35.26	46.06	3.069	1.4
SPAC22G7.02	kap111	0.01272	7.245	1.895512889	37.9	50.35	1.68	1.625
SPCC1322.14C	vtc4	0.05811	7.25	1.235749125	33.16	42.56	1.365	2.523
SPBC1709.12	rid1	0.2305	7.284	0.63732907	38.1	50.61	3.527	4.73
SPCC285.04	SPCC285.04	0.09441	7.31	1.024982002	34.28	44.3	4.558	2.4
SPBC336.14C	ppk26	0.02519	7.315	1.598771833	34.48	44.61	0.9714	1.885
SPAC3H1.12C	snt2	0.04351	7.321	1.361410917	30.11	37.44	3.285	2.059
SPAC15E1.10	SPAC15E1.10	0.5201	7.34	0.283913146	30.4	37.88	12.89	7.34
SPAC6F6.03C	SPAC6F6.03c	0.06661	7.347	1.176460566	36.04	47.13	2.729	2.672
SPAC6B12.14C	SPAC6B12.14c	0.02509	7.375	1.600499339	36.36	47.61	2.415	1.936
SPAC1A6.07	SPAC1A6.07	0.1076	7.392	0.968187729	34.85	45.11	3.243	3.214
SPBC651.02	SPBC651.02	0.1508	7.404	0.821598658	34.47	44.46	5.348	3.1
SPAC1851.04C	ric1	0.1753	7.43	0.756218084	18	17.36	5.961	3.73
SPAC1002.01	SPAC1002.01	0.01988	7.469	1.70158362	35.66	46.3	2.787	1.659
SPBC119.14	rti1	0.07943	7.515	1.100015437	37.4	49.09	4.104	2.54
SPAC15A10.08	ain1	0.09245	7.528	1.034093085	37.46	49.16	3.679	2.961
SPBP23A10.14C	ell1	0.002044	7.531	2.689519109	32.8	41.5	1.506	1.093
SPCC126.07C	SPCC126.07c	0.2242	7.539	0.649364392	37.45	49.13	4.013	4.758
SPAC16E8.18	SPAC16E8.18	0.01749	7.568	1.757210191	34.34	43.97	2.914	1.465
SPAC2G11.12	rqh1	0.08517	7.58	1.069713353	25.05	28.69	3.239	2.98
SPAC3A11.05C	kms1	0.1268	7.623	0.896880746	34.89	44.78	5.631	2.49
SPBC1718.02	hop1	0.0118	7.659	1.928117993	36.7	47.71	2.117	1.653
SPAC1527.03	SPAC1527.03	0.08596	7.714	1.065703593	36.67	47.56	2.516	3.134
SPBC2G2.01C	liz1	0.005725	7.777	2.242224509	29.61	35.86	1.458	1.432
SPCC191.06	SPCC191.06	0.06294	7.786	1.201073261	37.13	48.19	3.795	2.511
SPAC3A12.13C	SPAC3A12.13c	0.03427	7.798	1.465085896	24.8	27.93	3.479	1.906
SPBPJ4664.06	gpt1	0.2401	7.815	0.61960784	38.31	50.09	5.013	4.999
SPBC1652.02	SPBC1652.02	0.03616	7.847	1.441771578	35.38	45.23	3.153	2.182
SPCC13B11.02C	SPCC13B11.02c	0.02992	7.866	1.524038411	34.71	44.09	1.858	2.23
SPAC13G6.13	SPAC13G6.13	0.126	7.906	0.899629455	34.67	43.96	6.1	2.082
SPBC83.17	SPBC83.17	0.1037	7.926	0.984221244	36.03	46.16	2.571	3.482
SPBC28E12.06C	lvs1	0.07861	8.101	1.104522204	36.35	46.4	1.666	3.17
SPAC18G6.13	SPAC18G6.13	0.09421	8.142	1.025902996	31.94	39.09	2.214	3.443
SPAC1399.03	fur4	0.03606	8.226	1.442974278	30.65	36.83	2.051	2.471
SPAC1B3.16C	vht1	0.05009	8.318	1.300248968	26.75	30.28	4.474	1.841
SPBC106.08C	mug2	0.2845	8.349	0.545917729	39.26	50.78	4.346	6.235
SPAC56E4.07	SPAC56E4.07	0.09535	8.423	1.020679303	35.03	43.7	2.428	3.579
SPCC132.01C	SPCC132.01c	0.02185	8.472	1.660548559	33.64	41.34	2.662	2.147
SPCC895.06	elp2	0.5077	8.511	0.294392837	29.13	33.87	11.38	9.801
SPAC16A10.01	SPAC16A10.01	0.02448	8.585	1.611188587	36.02	45.06	1.841	2.286
SPAC11G7.02	pub1	0.01517	8.637	1.819014419	28.33	32.36	1.874	2.023
SPAC1805.05	cki3	0.1191	8.642	0.924088239	39.45	50.61	3.371	3.996
SPAC323.04	SPAC323.04	0.06484	8.889	1.188156994	17.47	14.09	4.836	3.311
SPAC1F5.10	SPAC1F5.10	0.01607	9.195	1.793984123	13.75	7.478	3.53	0.9867

SPBC3B8.05	SPBC3B8.05	0.004165	9.303	2.380384994	38.37	47.75	2.2	1.553
SPAPB2B4.06	SPAPB2B4.06	0.04135	9.366	1.383524486	37.92	46.91	3.981	2.676
SPCC965.06	SPCC965.06	0.1123	9.407	0.949620244	36.11	43.86	4.418	4.12
SPCC1494.03	arz1	0.1372	9.63	0.862645889	39.58	49.19	2.967	4.807
SPAC12B10.14C	ppk2	0.04303	9.637	1.366228654	37.22	45.31	3.874	2.888
SPCC1795.06	map2	0.3884	9.915	0.410720779	32.73	37.47	13.13	7.04
SPAC328.05	SPAC328.05	0.05551	10.64	1.255628773	41.96	51.45	2.415	3.672
SPBP8B7.26	SPBP8B7.26	0.07814	11.37	1.107126593	39.09	45.53	2.651	4.453
SPAC9.02C	SPAC9.02c	0.01024	20.2	1.989700043	32.85	20.78	5.163	4.252
SPAC27F1.08	pdt1	0.002218	23.58	2.654038458	34.32	17.64	5.731	2.178

Supplementary Table 2

R package version: 0.0-10
Summary type: mean Based on 4 independent repeat experimens
Test type: t-test

Control medium: YE5S
Control screen ID: QFA0002
Control libraries: PDLV4_384
Query medium: EMMG_Glutamate
Query screen ID: QFA0002
##

ORF	Gene	P	EGI	(p) -log10	Glutamate Fitness mean	Yes_Fitness mean	Glutamate SE	YES SE
SPBC30D10.16	pha2	0.001392	-36.17	2.856360765	7.46	61.22	5.233	1.057
SPAC343.10	met11	0.13	-22.97	0.886056648	17.49	56.78	15.62	1.581
SPAC1782.12C	SPAC1782.12c	0.01939	-21.39	1.712422191	26.95	67.83	7.167	1.754
SPBC2G5.06C	hmt2	0.005318	-21.12	2.274251667	24.49	64	4.869	1.311
SPBC27.08C	sua1	0.0006528	-20.42	3.185219854	16.81	52.23	2.404	0.5107
SPAC343.15	tit1	0.0239	-20.18	1.621602099	15.53	50.1	3.014	5.252
SPAC56E4.03	SPAC56E4.03	0.01415	-19.89	1.84924356	15.5	49.67	6.007	3.913
SPBC21H7.07C	his5	0.01419	-19.86	1.848017605	15.42	49.5	3.883	4.582
SPCC1739.06C	SPCC1739.06c	0.06931	-19.84	1.159204101	20.43	56.5	10.36	2.39
SPBC428.05C	arg12	0.0275	-18.95	1.560667306	14.82	47.39	5.236	5.146
SPBC215.08C	arg4	0.08385	-18.38	1.076496933	14.41	46.01	10.32	1.993
SPBC25H2.08C	mrs2	0.05309	-17.94	1.274987275	36.84	76.87	8.255	1.08
SPAC1296.02	cox4	0.3236	-17.91	0.489991487	22.58	56.81	21.06	4.256
SPAC23D3.04C	gpd2	0.006565	-17.61	2.18276527	28.96	65.35	4.694	2.028
SPCPB1C11.03	SPCPB1C11.03	4.01E-05	-17.41	4.396855627	27.22	62.62	1.552	1.188
SPAC25G10.05C	his1	0.0238	-17.3	1.623423043	20.75	53.4	6.397	2.168
SPBC725.14	arg6	0.02366	-16.5	1.62598526	20.9	52.48	5.701	0.9787
SPCC576.17C	SPCC576.17c	0.1779	-16.4	0.749824052	35	72.13	13.15	1.578
SPAC57A7.08	pzh1	0.06287	-15.99	1.20155654	41.3	80.38	7.987	1.578
SPBC15D4.09C	SPBC15D4.09c	0.04178	-15.97	1.379031564	23.79	55.79	6.983	1.935
SPAC31A2.09C	apm4	0.002469	-15.88	2.60747891	20.19	50.61	3.403	1.874
SPBC1105.02C	lys4	0.0351	-15.72	1.454692884	21.53	52.27	6.526	2.977
SPBC1711.13	his2	0.1529	-15.04	0.815592515	16.73	44.57	10.72	4.552
SPBC19G7.07C	ppr3	0.2579	-15.01	0.588548658	42.56	80.77	15.09	1.831
SPAC683.02C	SPAC683.02c	0.01676	-14.68	1.775725986	20.39	49.21	4.905	1.702
SPAC3F10.09	SPAC3F10.09	0.02323	-14.56	1.63395079	22.57	52.1	5.033	0.9386
SPAC890.05	SPAC890.05	0.03511	-14.52	1.454569171	19.25	47.39	6.073	2.38
SPAC4G9.09C	arg11	0.05074	-14.52	1.294649537	22.64	52.14	6.823	2.107
SPBC11B10.02C	his3	0.01006	-14.37	1.997402019	18.2	45.69	4.19	2.327
SPCC188.13C	dcr1	0.00651	-14.18	2.186419011	20.01	47.96	3.696	2.182
SPAC343.16	lys2	0.01906	-14	1.719877104	24.85	54.5	4.361	3.115
SPAC17H9.13C	SPAC17H9.13c	0.1082	-13.73	0.965772739	19.2	46.2	3.386	6.155
SPAC24C9.07C	bgs2	0.1743	-13.7	0.758702613	43.32	80.01	10.86	1.976
SPBC1271.14	SPBC1271.14	0.2865	-13.57	0.542875374	14.16	38.92	14.05	5.017
SPCC550.01C	SPCC550.01c	0.02517	-13.28	1.599116784	33.18	65.19	4.861	1.234
SPCC16A11.08	atg20	0.06088	-13.16	1.215525356	36.26	69.33	6.583	1.849
SPBC25H2.03	SPBC25H2.03	0.3996	-13.1	0.39837452	32.84	64.46	18.6	2.767
SPCC63.04	mok14	0.261	-12.94	0.583359493	45.26	81.65	13.1	1.759
SPCC4B3.12	set9	0.2663	-12.91	0.574628834	38.51	72.15	13.24	2
SPCC553.03	pex1	0.0109	-12.85	1.962573502	36.73	69.57	3.885	1.847
SPAC25H1.02	jmj1	0.08863	-12.68	1.052419251	40.59	74.74	7.332	1.906
SPAC2G11.03C	vps45	0.001745	-12.66	2.758204569	24.01	51.45	2.05	0.4846
SPAC1A6.03C	SPAC1A6.03c	0.1089	-12.65	0.96297212	42.21	76.98	7.969	1.669
SPBC23G7.08C	rga7	0.02833	-12.5	1.547753425	22.49	49.09	3.654	3.38
SPAC4A8.03C	ptc4	0.2323	-12.39	0.63395079	8.578	29.42	10.79	4.958
SPBC14C8.04	SPBC14C8.04	0.07719	-12.38	1.112438959	39.73	73.12	6.74	1.498
SPAC15A10.13	ppk3	0.2511	-12.3	0.600153287	44.64	79.9	12.15	1.319
SPCC584.01C	SPCC584.01c	0.004191	-12.28	2.377682339	22.22	48.42	2.388	0.3963
SPAC3H8.09C	nab3	0.1418	-12.06	0.848323769	22.86	48.99	8.557	2.531
SPAC589.08C	dam1	0.3129	-11.92	0.504594437	38.4	70.61	13.76	1.786
SPBC428.02C	eca39	0.09731	-11.56	1.011842527	14.49	36.54	0.4296	4.855
SPAC57A10.06	mug15	0.158	-11.54	0.801342913	42.13	75.3	8.685	1.702
SPAP7G5.04C	lys1	0.3129	-11.48	0.504594437	19.71	43.76	11.24	6.613
SPBC887.10	mcs4	0.428	-11.4	0.368556231	37.4	68.47	17.36	2.297
SPBC1703.09	SPBC1703.09	0.2678	-11.37	0.572189427	44.18	77.95	11.7	1.809

SPBC4F6.08C	mrpl39	0.006463	-11.36	2.189565844	34.08	63.76	2.991	1.673
SPBC25B2.04C	mtg1	0.006447	-11.34	2.19064233	21.49	46.07	3.011	1.567
SPBC543.02C	SPBC543.02c	0.1806	-11.33	0.743282254	39.31	71.06	9.16	1.601
SPCC1235.13	ght6	0.1069	-11.3	0.971022295	44.88	78.83	7.073	1.985
SPAC30D11.11	SPAC30D11.11	0.2353	-11.28	0.628378073	47.69	82.74	10.66	1.588
SPBC776.15C	kgd2	0.2938	-11.18	0.531948209	27.66	54.5	11.9	3.878
SPAC17H9.08	SPAC17H9.08	0.2293	-11.16	0.639595945	23.98	49.31	10.13	3.301
SPBP8B7.18C	SPBP8B7.18c	0.07154	-11.14	1.145451064	24.16	49.53	5.897	2.311
SPBP35G2.07	ilv1	0.06685	-11.1	1.174898588	12.51	33.13	3.065	4.093
SPAC1687.09	SPAC1687.09	0.2785	-11.09	0.5551748	43.49	76.58	11.71	2.051
SPAPYUG7.04C	rpb9	0.1683	-11.08	0.773915884	37.14	67.65	8.619	1.257
SPBC1685.08	cti6	0.07817	-11.03	1.106959888	39.04	70.26	6.067	2.072
SPBC28F2.10C	ngg1	0.04608	-10.97	1.33648753	25.01	50.47	4.385	3.06
SPBC15C4.04C	SPBC15C4.04c	0.2417	-10.93	0.61672335	43.66	76.59	10.53	1.154
SPBPB2B2.02	mug180	0.06045	-10.84	1.218603695	40.46	71.98	5.427	1.771
SPAC24C9.15C	spn5	0.1201	-10.79	0.920456993	39.6	70.71	7.101	1.289
SPCC794.01C	SPCC794.01c	0.1041	-10.72	0.98254927	40.24	71.5	6.628	1.626
SPBC713.03	SPBC713.03	0.287	-10.7	0.542118103	46.01	79.57	11.55	1.854
SPAC144.17C	SPAC144.17c	0.03264	-10.68	1.48624985	36.47	66.15	4.296	2.134
SPBC16A3.02C	SPBC16A3.02c	0.1587	-10.66	0.799423073	43.03	75.33	7.957	2.53
SPCC1235.04C	SPCC1235.04c	0.1207	-10.65	0.91829273	41.73	73.5	7.025	1.402
SPBC1604.19C	SPBC1604.19c	0.3936	-10.59	0.40494491	34.28	62.97	14.82	2.273
SPBC2G2.05	rpl1603	0.2032	-10.58	0.692076296	45.38	78.51	9.14	1.608
SPAC25B8.11	SPAC25B8.11	0.2749	-10.45	0.56082526	46.63	80.09	10.93	1.967
SPAC11G7.06C	mug132	0.2011	-10.34	0.696587929	41.36	72.55	8.861	1.89
SPAC22E12.01	SPAC22E12.01	0.2542	-10.34	0.594824454	44.37	76.75	10.27	1.626
SPAC323.01C	pos5	0.01331	-10.31	1.875821945	5.453	22.12	2.021	2.341
SPBC543.03C	pku80	0.2543	-10.26	0.59465364	47.67	81.29	10.21	1.437
SPCC663.08C	SPCC663.08c	0.2032	-10.22	0.692076296	42.02	73.3	8.749	2.37
SPBC1683.02	SPBC1683.02	0.2063	-10.22	0.685500772	38.29	68.06	8.869	2.084
SPAC3A12.12	atp11	0.2788	-10.22	0.554707231	23.57	47.42	10.57	3.186
SPBP23A10.16	sdh4	0.2015	-10.2	0.69572495	45.03	77.49	8.728	2.084
SPBC17D1.05	SPBC17D1.05	0.1928	-10.19	0.71489297	41.08	71.94	8.501	2.113
SPCC70.09C	mug9	0.1902	-10.17	0.720789487	42.22	73.51	8.432	1.966
SPCC1620.14C	snf22	0.2646	-10.17	0.57741016	33.2	60.86	10.42	0.9908
SPCC569.07	SPCC569.07	0.1395	-10.12	0.855425792	41.61	72.59	7.153	1.818
SPAC1002.19	urg1	0.2985	-10.11	0.525055665	45.89	78.58	11.24	1.834
SPBC28F2.08C	SPBC28F2.08c	0.105	-10.08	0.978810701	36.85	65.86	6.261	1.789
SPBC1778.05C	SPBC1778.05c	0.5721	-10.04	0.242528052	20.08	42.26	21.45	5.48
SPAPB2B4.07	SPAPB2B4.07	0.3322	-9.956	0.478600372	28.9	54.51	11.92	2.595
SPAC9G1.04	oxa101	0.1501	-9.906	0.823619308	41.05	71.5	7.262	1.618
SPCC757.03C	SPCC757.03c	0.1453	-9.809	0.837734386	41.11	71.45	7.077	1.538
SPAC9E9.10C	cbh1	0.3789	-9.8	0.421475395	40.14	70.08	13.24	2.036
SPAC890.07C	rmt1	0.2186	-9.798	0.660349842	44.26	75.85	8.789	2.131
SPBC21B10.08C	SPBC21B10.08c	0.2181	-9.632	0.661344334	42.7	73.42	8.665	1.731
SPAC1952.05	gcn5	0.005004	-9.594	2.300682699	18.06	38.8	2.039	1.658
SPBC16A3.16	SPBC16A3.16	0.001772	-9.59	2.751536282	35.52	63.29	1.888	1.152
SPCC24B10.06	SPCC24B10.06	0.2008	-9.579	0.697236292	40.96	70.91	8.216	1.551
SPBC1604.01	mug158	0.2102	-9.405	0.677367288	44.3	75.36	8.137	2.586
SPAC105.03C	SPAC105.03c	0.3515	-9.394	0.454074671	43.71	74.51	11.8	2.424
SPBC3B9.08C	mnh1	0.1864	-9.371	0.729554092	44.48	75.56	7.665	1.999
SPBC18H10.16	can1	0.04293	-9.367	1.367239112	36.82	64.81	3.952	2.27
SPAC227.18	lys3	0.1951	-9.304	0.709742731	37.84	66.15	7.854	1.37
SPBC14F5.13C	SPBC14F5.13c	0.1368	-9.295	0.863913903	37.8	66.08	6.51	1.534
SPCC1259.03	rpa12	0.205	-9.29	0.688246139	40.08	69.27	8.049	1.729
SPBC3B8.02	php5	0.3628	-9.269	0.440332722	31.29	56.91	12.05	1.86
SPBC359.06	mug14	0.09107	-9.243	1.040624663	38.94	67.61	5.382	1.108
SPCC777.10C	ubc12	0.03363	-9.163	1.473273133	28.09	52.27	3.696	1.899
SPCC4G3.05C	mus81	0.2173	-9.16	0.662940274	44.52	75.33	8.218	1.701
SPAC29A4.13	SPAC29A4.13	0.2136	-9.145	0.670398752	42.46	72.41	8.014	2.42
SPBC685.02	exo5	0.1715	-9.083	0.765735876	40.87	70.09	7.133	1.54
SPAC13G6.07C	rps601	0.1982	-9.083	0.70289635	43.37	73.59	7.72	1.668
SPAP8A3.13C	SPAP8A3.13c	0.1054	-9.066	0.977159389	42.67	72.59	5.625	1.801
SPBC106.05C	tim11	0.1743	-9.055	0.758702613	39.42	68.02	7.179	1.235
SPAC4H3.06	SPAC4H3.06	0.2683	-8.97	0.571379327	43.38	73.45	9.043	2.706
SPBC1734.08	hse1	0.5182	-8.918	0.285502591	33.91	60.09	17.04	1.853
SPAC4G8.11C	atp10	0.301	-8.904	0.521433504	16.57	35.74	6.951	6.075
SPBC725.11C	php2	0.01027	-8.854	1.988429556	28.28	52.11	2.627	1.34
SPBC15D4.12C	mug98	0.2793	-8.851	0.553929064	47.76	79.42	9.375	1.506

SPBP8B7.23	SPBP8B7.23	0.2577	-8.844	0.588885581	42.18	71.59	8.812	1.951
SPBC18E5.01	SPBC18E5.01	0.1876	-8.83	0.726767166	41.72	70.93	7.163	2.351
SPCC16A11.03C	SPCC16A11.03c	0.2196	-8.777	0.658367664	41.47	70.5	7.918	1.712
SPAPB8E5.03	mae1	0.2791	-8.707	0.554240164	45.19	75.63	9.199	1.684
SPACUNK4.10	SPACUNK4.10	0.539	-8.686	0.268411235	37.49	64.79	17.4	2.687
SPCC1322.01	rpm1	0.1178	-8.651	0.92885471	22.54	43.76	5.653	1.391
SPBP8B7.07C	set6	0.1176	-8.647	0.929592678	41.43	70.26	5.625	1.78
SPAC323.03C	SPAC323.03c	0.5553	-8.645	0.255472327	40.89	69.5	17.85	3.878
SPAC3A12.08	SPAC3A12.08	0.1395	-8.615	0.855425792	42.82	72.17	6.058	1.86
SPBC3B8.08	SPBC3B8.08	0.1085	-8.533	0.964570262	41.62	70.37	5.374	1.313
SPCC548.06C	ght8	0.4086	-8.49	0.388701638	13.92	31.45	12.04	3.195
SPBC19C7.12C	omh1	0.01063	-8.474	1.973466735	38.52	65.94	2.545	1.23
SPAC1F3.09	mug161	0.112	-8.408	0.950781977	41.21	69.62	5.368	1.523
SPAC227.01C	SPAC227.01c	0.4432	-8.406	0.353400248	34.71	60.5	13.25	2.025
SPCP1E11.02	ppk38	0.1635	-8.4	0.786482243	41.75	70.37	6.409	1.738
SPAC17A5.01	pex6	0.09772	-8.355	1.010016542	36.75	63.29	4.962	1.98
SPAC2F3.12C	plp1	0.08044	-8.301	1.094527938	29.48	53.02	3.246	3.122
SPAC1687.08	SPAC1687.08	0.32	-8.301	0.494850022	14.27	31.67	9.662	2.021
SPBC21D10.11C	nfs1	0.2344	-8.286	0.630042393	44.39	73.91	7.659	2.345
SPAC630.05	gyp7	0.09297	-8.233	1.031657169	39.32	66.72	4.854	1.53
SPBC106.17C	cys2	0.09672	-8.211	1.014483712	18.47	37.44	4.913	1.626
SPCC1235.15	dga1	0.2265	-8.21	0.644931794	43.34	72.33	7.461	2.162
SPBP22H7.06	SPBP22H7.06	0.1721	-8.191	0.76421913	38.95	66.14	6.392	1.901
SPBC30D10.10C	tor1	0.2267	-8.129	0.64454848	39.76	67.19	7.459	1.758
SPCC1620.03	mug163	0.2382	-8.08	0.623058243	41	68.86	7.691	1.318
SPBC13E7.11	SPBC13E7.11	0.06854	-8.078	1.1640559	37.58	64.07	4.223	1.147
SPCC1393.08	SPCC1393.08	0.2616	-8.065	0.58236226	19.34	38.45	7.875	2.779
SPAC105.02C	SPAC105.02c	0.08158	-8.037	1.088416299	40.69	68.37	4.5	1.401
SPAC1834.04	hht1	0.1352	-7.995	0.869023308	43.24	71.89	5.521	1.83
SPCC306.11	SPCC306.11	0.4631	-7.936	0.334325219	23.87	44.63	12.76	3.559
SPAC144.02	iec1	0.4594	-7.918	0.337809009	36.08	61.74	13.02	1.676
SPBC56F2.11	met6	0.2313	-7.852	0.635824367	6.466	20.09	6.426	3.663
SPBC1D7.01	SPBC1D7.01	0.214	-7.802	0.669586227	42.04	69.93	6.888	1.841
SPAC227.14	SPAC227.14	0.3246	-7.776	0.488651485	42.27	70.22	9.239	1.203
SPBC11B10.05C	rsp1	0.2131	-7.775	0.67141655	42.68	70.8	6.823	1.963
SPBC577.11	SPBC577.11	0.289	-7.763	0.539102157	41.51	69.14	8.242	2.422
SPBC1289.16C	cao2	0.2816	-7.704	0.55036735	43.35	71.64	8.006	2.464
SPCC1919.12C	SPCC1919.12c	0.22	-7.69	0.657577319	41.66	69.25	6.831	2.163
SPAC2F7.11	nrd1	0.1002	-7.686	0.999132278	37.8	63.82	4.639	1.714
SPBC19C2.02	pmt1	0.2861	-7.671	0.543482142	41.07	68.38	8.118	2.257
SPCC70.06	SPCC70.06	0.09074	-7.66	1.042201225	37.69	63.63	4.431	1.721
SPBC19C7.01	mni1	0.005376	-7.638	2.26954074	32.61	56.47	1.772	1.278
SPBC30D10.13C	pdb1	0.05222	-7.625	1.282163133	4.413	16.89	2.228	2.558
SPBC25B2.10	SPBC25B2.10	0.396	-7.562	0.402304814	21.27	40.46	9.492	4.616
SPBC1539.03C	SPBC1539.03c	0.08271	-7.51	1.082441979	25.59	46.44	4.204	0.9042
SPBC6B1.05C	atg7	0.5805	-7.48	0.236197776	33.59	57.63	16.56	3.535
SPBC1773.03C	SPBC1773.03c	0.5493	-7.425	0.260190401	38.12	63.91	15.3	2.309
SPBC30D10.09C	SPBC30D10.09c	0.5377	-7.416	0.269459964	38.4	64.28	14.74	2.678
SPBC1778.10C	ppk21	0.1818	-7.341	0.740406121	42.56	70.02	5.808	2.105
SPBC337.16	cho1	0.3105	-7.319	0.507938395	8.664	22.43	7.993	2.927
SPAC31G5.04	SPAC31G5.04	0.5485	-7.291	0.260823368	19.75	37.94	13.01	6.638
SPBC17G9.05	rct1	0.4887	-7.264	0.310957661	34.9	59.16	12.11	4.246
SPBC21C3.01C	vps13a	0.6318	-7.223	0.199420378	30.8	53.36	18.04	5.439
SPAC24C9.08	SPAC24C9.08	0.1632	-7.215	0.787279846	40.58	67.06	5.352	2.13
SPCP31B10.07	eft202	0.4197	-7.214	0.377061031	33.33	56.89	10.74	1.708
SPBC32H8.11	mei4	0.564	-7.207	0.248720896	37.35	62.51	15.43	2.429
SPCC1450.09C	SPCC1450.09c	0.1506	-7.188	0.822175028	41.82	68.76	5.258	1.485
SPBC16H5.03C	fub2	0.03885	-7.11	1.410608977	39.38	65.23	2.988	1.542
SPCC162.02C	SPCC162.02c	0.5874	-7.097	0.231066058	38.62	64.14	16.22	2.482
SPBC23G7.14	SPBC23G7.14	0.292	-7.092	0.534617149	42.42	69.47	7.561	2.312
SPAC4F8.15	itr1	0.6969	-7.069	0.156829535	32.18	55.07	20.59	8.797
SPBC1271.07C	SPBC1271.07c	0.4193	-7.068	0.377475138	35.5	59.72	10.34	2.486
SPAC24C9.12C	SPAC24C9.12c	0.4147	-7.032	0.382265965	29.33	51.02	9.975	3.102
SPAC1250.03	ubc14	0.01572	-7.031	1.803547458	38.22	63.5	1.596	1.658
SPAC3C7.01C	SPAC3C7.01c	0.2537	-7.026	0.595679533	46.19	74.67	6.943	1.428
SPCC663.02	wtf14	0.5801	-7.024	0.236497135	36.62	61.24	15.59	3.096
SPBC1734.12C	alg12	0.4091	-6.971	0.388170521	37.42	62.29	10.04	2.052
SPCC74.06	mak3	0.2206	-6.913	0.656394492	44.49	72.13	6.089	2.155
SPBC24C6.05	sec28	0.1431	-6.899	0.844360366	26.9	47.43	4.916	1.466

SPBC582.09	pex11	0.2896	-6.898	0.538201442	45.39	73.37	7.441	1.669
SPBC83.05	SPBC83.05	0.2377	-6.885	0.623970818	40.73	66.81	6.112	2.729
SPAC27F1.03C	uch1	0.6337	-6.847	0.198116293	41.33	67.6	17.96	2.59
SPBC21C3.03	SPBC21C3.03	0.2563	-6.823	0.591251394	42.78	69.6	6.75	1.658
SPCC794.03	SPCC794.03	0.02498	-6.792	1.602407566	35.08	58.75	2.133	1.698
SPBC8E4.02C	SPBC8E4.02c	0.394	-6.786	0.404503778	46.97	75.43	9.362	2.225
SPBC2G2.15C	mrm2	0.2781	-6.782	0.555799011	44.26	71.62	6.964	2.228
SPAC57A10.09C	nhp6	0.3817	-6.778	0.41827784	36.59	60.85	8.978	2.547
SPBC16G5.16	SPBC16G5.16	0.6025	-6.764	0.220042949	41.67	67.96	16.15	2.442
SPBC577.06C	stt4	0.4004	-6.708	0.397505931	30.52	52.23	9.519	1.615
SPBC725.07	pex5	0.1715	-6.662	0.765735876	27.05	47.31	4.522	2.817
SPAC23G3.03	sib2	0.243	-6.645	0.614393726	42.33	68.71	6.31	1.796
SPBCPT2R1.02	SPBCPT2R1.02	0.4844	-6.632	0.314795866	34.91	58.28	11.34	2.775
SPAC11D3.14C	SPAC11D3.14c	0.04426	-6.618	1.35398859	37.89	62.45	2.753	1.698
SPCC18B5.05C	SPCC18B5.05c	0.214	-6.593	0.669586227	30.18	51.6	5.679	2.1
SPBC16G5.09	SPBC16G5.09	0.6071	-6.575	0.216739767	35.94	59.65	15.88	2.56
SPAC32A11.03C	phx1	0.01997	-6.552	1.699621935	35.47	58.96	2.082	1.463
SPAC977.12	SPAC977.12	0.1244	-6.517	0.90517962	24.88	44.05	4.352	1.33
SPAC4D7.03	pop2	0.006342	-6.512	2.197773762	37.41	61.63	0.6974	1.157
SPBC17A3.03C	SPBC17A3.03c	0.1643	-6.508	0.784362437	40.64	66.15	4.778	2.083
SPAC11G7.01	SPAC11G7.01	0.5536	-6.494	0.256803919	36.87	60.84	13.19	3.422
SPAC9.07C	SPAC9.07c	0.5126	-6.455	0.290221398	39.83	64.94	12.03	2.16
SPAC57A10.10C	sla1	0.6277	-6.455	0.202247871	35.77	59.24	16.54	2.792
SPCC320.07C	mde7	0.3082	-6.454	0.511167366	43.82	70.54	7.07	2.421
SPBPB2B2.12C	gal10	0.7074	-6.412	0.150334945	26.27	45.86	19.88	7.518
SPAC17G8.06C	SPAC17G8.06c	0.1327	-6.407	0.877129077	21.68	39.4	2.523	3.06
SPCC24B10.20	SPCC24B10.20	0.2592	-6.371	0.586365003	42.32	68.32	6.322	1.706
SPAC823.02	SPAC823.02	0.5271	-6.355	0.278106984	33.87	56.44	12.23	2.459
SPAC29A4.12C	mug108	0.499	-6.349	0.301899454	33.8	56.34	11.5	1.666
SPBC1683.13C	cha4	0.2119	-6.346	0.673869043	45.3	72.46	5.363	2.19
SPBC119.03	SPBC119.03	0.2034	-6.343	0.691649051	44.52	71.37	5.392	1.694
SPAC922.06	SPAC922.06	0.2419	-6.338	0.616364132	42.13	68	5.777	2.365
SPAC1002.20	SPAC1002.20	0.2772	-6.325	0.557206774	42.54	68.56	6.427	2.231
SPBC18H10.11C	ppr2	0.3232	-6.276	0.490528648	20.36	37.37	7.221	2.147
SPAC821.07C	moc3	0.6278	-6.264	0.202178689	31.79	53.39	15.8	3.687
SPAC17H9.11	gmf1	0.6025	-6.263	0.220042949	35.84	59.07	15.04	1.732
SPBC947.06C	SPBC947.06c	0.2579	-6.258	0.588548658	41.63	67.19	5.954	2.381
SPBC1861.01C	cnp3	0.5811	-6.248	0.235749125	36.75	60.34	14.18	1.024
SPBP16F5.03C	tra1	0.008902	-6.218	2.05051241	34.53	57.18	1.425	1.244
SPAPB17E12.05	rpl3703	0.4763	-6.206	0.322119418	11.04	24.2	8.643	5.351
SPBC19C2.09	sre1	0.6902	-6.205	0.161025045	27.39	47.14	18.41	6.282
SPAC1751.01C	gti1	0.5353	-6.2	0.271402757	32.78	54.7	12.18	2.524
SPCC663.03	pmd1	0.2534	-6.188	0.596193389	42.05	67.68	6.068	1.54
SPBC36B7.08C	SPBC36B7.08c	0.5493	-6.187	0.260190401	37.15	60.81	12.54	2.851
SPAC6B12.07C	SPAC6B12.07c	0.2551	-6.171	0.593289541	30.73	51.78	6.137	1.093
SPBC19F5.01C	puc1	0.305	-6.149	0.515700161	38.83	63.12	6.609	2.474
SPAC8E11.10	SPAC8E11.10	0.1977	-6.146	0.703993331	33	54.92	5.054	1.919
SPCC663.15C	SPCC663.15c	0.3309	-6.094	0.480303233	42.13	67.67	7.009	2.485
SPBC1778.04	spo6	0.6207	-6.043	0.207118255	39.81	64.34	15.18	2.461
SPAC16.04	dus3	0.3544	-5.987	0.450506287	38.09	61.84	7.654	0.9041
SPCC1620.07C	SPCC1620.07c	0.3109	-5.981	0.507379278	39.79	64.22	6.757	1.711
SPCC1223.09	SPCC1223.09	0.2896	-5.959	0.538201442	42.1	67.43	6.36	1.773
SPBC29A3.09C	SPBC29A3.09c	0.2106	-5.948	0.676541633	44.52	70.82	5.059	1.926
SPBC26H8.09C	snf59	0.2716	-5.915	0.566070234	43.92	69.93	6.03	1.731
SPAC1635.01	SPAC1635.01	0.3371	-5.883	0.472241247	30.13	50.54	6.712	2.749
SPAC1B3.06C	SPAC1B3.06c	0.4247	-5.866	0.371917739	48.36	76.09	8.683	2.142
SPAC227.10	SPAC227.10	0.1164	-5.86	0.93404702	41.62	66.61	3.611	1.782
SPBC23G7.12C	rpt6	0.2526	-5.84	0.597566654	42.75	68.18	5.633	1.804
SPAC29B12.13	SPAC29B12.13	0.5893	-5.837	0.229663559	38.82	62.66	13.43	1.957
SPBC3E7.16C	leu3	0.04452	-5.817	1.351444844	42.73	68.12	1.709	1.842
SPBC1685.14C	SPBC1685.14c	0.5916	-5.812	0.227971835	36.5	59.37	13.28	2.791
SPAC13A11.05	SPAC13A11.05	0.1707	-5.795	0.767766479	42.29	67.47	4.295	1.965
SPCC794.09C	ef1a-a	0.574	-5.795	0.241088108	31.85	52.82	11.8	4.625
SPBC12D12.07C	trx2	0.4129	-5.789	0.384155117	7.108	18.1	7.967	3.018
SPBC1347.11	sro1	0.2577	-5.777	0.588885581	44.49	70.53	5.474	2.24
SPCC736.13	SPCC736.13	0.3353	-5.762	0.474566447	41.49	66.31	6.777	2.163
SPBC30B4.02C	SPBC30B4.02c	0.6435	-5.729	0.191451449	40.71	65.16	15.44	2.483
SPBC16G5.03	SPBC16G5.03	0.314	-5.728	0.503070352	42.57	67.77	6.394	2.086
SPAC13G7.11	SPAC13G7.11	0.2319	-5.723	0.634699251	35.47	57.79	5.291	1.449

SPBP35G2.05C	cki2	0.4699	-5.722	0.327994555	36.1	58.68	9.343	2.637
SPAC5H10.13C	gmh2	0.658	-5.683	0.181774106	40.02	64.13	15.94	3.034
SPCC1442.11C	SPCC1442.11c	0.2679	-5.674	0.572027286	43.47	68.95	5.45	2.365
SPBC16H5.04	SPBC16H5.04	0.3324	-5.671	0.478338985	43.47	68.96	6.612	2.15
SPCC306.08C	SPCC306.08c	0.6521	-5.671	0.1856858	37.84	61.05	15.55	3.182
SPAC664.02C	arp8	0.5739	-5.619	0.241163775	31.09	51.51	11.54	4.292
SPAC26A3.16	dph1	0.3034	-5.604	0.517984424	40.84	65.17	6.312	0.9299
SPCC16C4.04	SPCC16C4.04	0.3603	-5.564	0.443335738	33.01	54.13	7.128	1.502
SPBC56F2.06	mug147	0.1993	-5.533	0.700492701	39.03	62.52	4.454	1.991
SPCC24B10.02C	SPCC24B10.02c	0.2071	-5.513	0.683819901	43.38	68.6	4.662	1.727
SPAC22F3.11C	snu23	0.6401	-5.487	0.193752173	31.49	51.88	14.51	2.871
SPAP27G11.10C	nup184	0.09543	-5.478	1.020315076	38.53	61.75	2.66	2.018
SPAC26F1.04C	etr1	0.2299	-5.453	0.638461029	41.24	65.51	5.067	0.9943
SPAPB1A10.08	SPAPB1A10.08	0.2785	-5.433	0.5551748	31.07	51.22	5.407	2.219
SPAC14C4.16	dad3	0.04158	-5.43	1.381115515	39.07	62.44	1.736	1.646
SPCC1494.10	adn3	0.1456	-5.425	0.836838625	40.7	64.72	3.874	1.302
SPAC521.03	SPAC521.03	0.5973	-5.417	0.223807485	37.59	60.34	12.48	2.985
SPBC337.09	erg28	0.6126	-5.406	0.212823008	18.93	34.15	11.66	5.652
SPAC4D7.02C	SPAC4D7.02c	0.6378	-5.394	0.195315485	35.97	58.04	14.32	2.081
SPAC2E1P3.04	cao1	0.5059	-5.378	0.295935321	37.86	60.67	9.464	3.033
SPBC19C2.06C	mug124	0.04035	-5.333	1.394156461	38.11	60.96	1.871	1.538
SPAC1039.03	SPAC1039.03	0.3507	-5.281	0.455064234	38.65	61.64	6.521	1.793
SPAC9.05	fml1	0.5777	-5.273	0.238297632	31.97	52.25	11.68	2.032
SPBC23G7.04C	nif1	0.6887	-5.265	0.161969917	41.33	65.38	16.55	2.18
SPAC3F10.05C	mug113	0.6787	-5.246	0.168322151	37.65	60.18	15.78	2.892
SPBC660.07	ntp1	0.6284	-5.245	0.201763824	32.2	52.54	12.91	4.019
SPCC18.15	SPCC18.15	0.3046	-5.205	0.516270101	43.26	68	5.797	1.444
SPAC13A11.04C	ubp8	0.6138	-5.192	0.211973116	27.88	46.4	12.12	4.065
SPAC3F10.04	gsa1	0.6459	-5.181	0.189834715	19.31	34.37	11.74	6.652
SPAC6C3.07	mug68	0.6807	-5.162	0.167044249	42.17	66.41	15.69	2.611
SPAC14C4.13	rad17	0.09385	-5.145	1.027565723	40.51	64.06	2.844	1.555
SPAC1F7.06	SPAC1F7.06	0.2681	-5.131	0.571703186	42.37	66.66	4.928	2.146
SPAC13G7.06	met16	0.2762	-5.123	0.558776326	22.26	38.42	5.191	1.808
SPBC16E9.13	ksp1	0.3361	-5.116	0.473531488	42.74	67.14	5.832	2.372
SPAC12G12.12	SPAC12G12.12	0.09801	-5.112	1.008729611	34.54	55.63	3.043	1.203
SPACUNK4.12C	mug138	0.2499	-5.111	0.602233744	39.45	62.52	4.15	3.063
SPCC613.07	SPCC613.07	0.2011	-5.102	0.696587929	39.07	61.98	4.101	1.888
SPBC609.04	caf5	0.6193	-5.093	0.20809892	41.61	65.54	12.65	2.499
SPAC3C7.02C	SPAC3C7.02c	0.6501	-5.083	0.187019834	41.13	64.84	14.07	1.791
SPBC8D2.10C	rmt3	0.6962	-5.074	0.157265981	34.87	56.05	15.89	4.05
SPBC1711.11	SPBC1711.11	0.3755	-5.058	0.425390059	44.87	70.05	6.695	1.529
SPBC26H8.01	thi2	0.2123	-5.056	0.673050006	28.28	46.77	3.965	2.229
SPAC6B12.05C	ies2	0.3685	-5.052	0.433562508	35.02	56.23	6.3	2.33
SPBC354.09C	SPBC354.09c	0.6947	-5.05	0.158202701	37.31	59.44	16.18	2.359
SPAC18B11.09C	SPAC18B11.09c	0.02582	-5.022	1.588043762	38.25	60.71	1.651	1.234
SPAC17A5.10	SPAC17A5.10	0.2037	-5.012	0.691008971	41.89	65.8	3.891	2.11
SPBC1105.01	rrp12	0.5497	-5.011	0.259874263	46.49	72.26	10.32	1.653
SPBC1A4.04	SPBC1A4.04	0.6917	-5.005	0.160082224	22.79	39	13.69	6.92
SPCC191.09C	gst1	0.3071	-4.999	0.512720184	41.84	65.73	5.325	2.175
SPBC2G2.06C	apl1	0.06507	-4.967	1.186619193	41.14	64.69	1.776	1.747
SPCC24B10.07	gad8	0.2924	-4.945	0.534022632	1.544	9.104	5.423	0.7522
SPAPB18E9.04C	SPAPB18E9.04c	0.645	-4.942	0.190440285	37.26	59.21	13.33	2.381
SPBC405.03C	SPBC405.03c	0.306	-4.927	0.514278574	39.75	62.68	5.362	1.856
SPCC1281.07C	SPCC1281.07c	0.6736	-4.925	0.171597922	38.5	60.93	14.46	3.057
SPAC922.05C	SPAC922.05c	0.7084	-4.915	0.149721447	35.6	56.84	16.39	3.107
SPAC23A1.15C	sec20	0.3109	-4.908	0.507379278	42.12	65.98	5.614	0.9988
SPBP35G2.10	mit1	0.544	-4.907	0.2644011	34.68	55.55	9.878	1.957
SPAC13G6.01C	rad8	0.632	-4.907	0.199282922	36.05	57.47	12.43	3.269
SPCC330.12C	sdh3	0.3346	-4.904	0.475474063	45.21	70.32	5.179	2.825
SPCC1259.10	pgp1	0.6106	-4.901	0.2142432	40.53	63.75	11.82	2.52
SPAC8E11.01C	SPAC8E11.01c	0.6521	-4.896	0.1856858	41.85	65.6	13.63	1.783
SPBC9B6.09C	mdl1	0.2711	-4.884	0.566870482	42.22	66.09	4.307	2.594
SPAC1610.02C	SPAC1610.02c	0.1296	-4.877	0.887394998	24.24	40.86	2.822	1.913
SPBC23G7.06C	SPBC23G7.06c	0.2011	-4.87	0.696587929	42.19	66.03	3.624	2.181
SPBC14C8.11C	SPBC14C8.11c	0.7151	-4.87	0.145633222	40.94	64.28	16.63	3.243
SPAC8C9.08	rps5	0.6527	-4.867	0.185286387	35.18	56.18	13.37	2.708
SPBC577.13	syj2	0.6042	-4.858	0.218819279	32.2	51.99	11.63	1.908
SPBC405.06	SPBC405.06	0.6311	-4.845	0.19990182	30.48	49.57	12.01	3.774
SPCC126.08C	SPCC126.08c	0.3141	-4.817	0.502932064	43.88	68.33	5.013	2.453

SPAC3A11.10C	SPAC3A11.10c	0.6268	-4.817	0.202871012	41.16	64.51	12.38	1.684
SPBC6B1.09C	nbs1	0.6862	-4.803	0.163549286	41.48	64.94	14.86	2.561
SPCC1393.02C	spt2	0.1264	-4.795	0.898252926	39.5	62.15	2.174	2.161
SPAC694.03	SPAC694.03	0.5746	-4.785	0.240634378	36.1	57.37	10.52	1.79
SPBC1718.07C	zfs1	0.73	-4.777	0.13667714	21.83	37.33	15.83	6.577
SPAC607.06C	SPAC607.06c	0.6195	-4.763	0.207958689	35.23	56.11	11.91	2.002
SPAC631.01C	acp2	0.3595	-4.738	0.444301105	32.87	52.76	5.395	2.766
SPAC458.06	SPAC458.06	0.565	-4.726	0.247951552	29.37	47.84	10.08	1.905
SPBC609.05	pob3	0.3348	-4.72	0.475214551	25.88	42.93	5.21	2.434
SPBC2D10.07C	SPBC2D10.07c	0.6003	-4.718	0.221631657	33.71	53.92	11.09	2.158
SPAC6F12.12	par2	0.1349	-4.701	0.86998805	37.5	59.21	2.827	1.824
SPBC13G1.08C	ash2	0.5587	-4.689	0.252821329	33.29	53.29	9.806	1.985
SPCC338.06C	SPCC338.06c	0.6465	-4.688	0.189431471	34.99	55.68	12.73	2.174
SPAC14C4.01C	SPAC14C4.01c	0.6309	-4.65	0.200039473	37.42	59.03	11.98	2.274
SPBC119.16C	SPBC119.16c	0.5898	-4.636	0.229295232	38.6	60.67	10.54	2.223
SPBC12C2.05C	bzz1	0.6099	-4.634	0.214741367	36.59	57.85	10.98	2.93
SPBC16G5.13	ptf2	0.5785	-4.626	0.237696637	36.62	57.87	10.3	1.677
SPAC4F10.06	SPAC4F10.06	0.2984	-4.6	0.525201181	42.38	65.92	4.93	1.672
SPAC637.10C	rpn10	0.04534	-4.563	1.343518484	37.79	59.43	1.654	1.363
SPAC26A3.02	myh1	0.7435	-4.562	0.128719027	31.01	49.91	16.76	5.214
SPAC10F6.14C	SPAC10F6.14c	0.5434	-4.558	0.264880366	35.85	56.69	9.149	1.867
SPCC24B10.12	cgi121	0.6636	-4.528	0.178093623	33.29	53.06	12.91	2.557
SPBC1289.15	SPBC1289.15	0.6401	-4.517	0.193752173	36.85	58.04	11.63	3.346
SPAC3F10.17	SPAC3F10.17	0.6255	-4.516	0.203772686	39.39	61.61	11.47	2.08
SPBPB10D8.07C	SPBPB10D8.07c	0.264	-4.504	0.578396073	37.66	59.15	4.54	1.135
SPCC1827.08C	pof7	0.5952	-4.5	0.225337077	37.8	59.35	10.48	1.775
SPBC19C7.05	SPBC19C7.05	0.135	-4.497	0.869666232	40.64	63.33	2.404	1.953
SPCC126.12	SPCC126.12	0.1447	-4.494	0.839531469	38.38	60.16	3.003	1.549
SPBC336.10C	tif512	0.0731	-4.481	1.136082623	39.16	61.23	1.531	1.672
SPAC17A5.05C	SPAC17A5.05c	0.2993	-4.477	0.523893283	44.36	68.53	4.425	2.308
SPAP8A3.12C	tpp2	0.162	-4.468	0.790484985	43.72	67.61	2.904	1.885
SPBC685.04C	aps2	0.6842	-4.468	0.16481693	41.41	64.38	13.8	2.026
SPCC126.13C	SPCC126.13c	0.4338	-4.466	0.362710452	34.04	54.02	6.84	1.301
SPAC12G12.09	SPAC12G12.09	0.6693	-4.453	0.174379175	36.57	57.56	12.73	3.211
SPAC3H5.04	aar2	0.483	-4.444	0.316052869	38.39	60.1	7.534	1.989
SPAC4F10.16C	SPAC4F10.16c	0.6581	-4.417	0.181708109	32.66	52.03	12.52	1.704
SPBC16A3.13	meu7	0.2729	-4.402	0.563996464	39.99	62.29	3.773	2.46
SPBC1347.08C	SPBC1347.08c	0.2839	-4.393	0.546834607	43.26	66.86	4.205	2.166
SPBC1683.03C	SPBC1683.03c	0.7237	-4.374	0.140441427	36.71	57.65	15.42	3.039
SPAC31G5.14	gcv1	0.7322	-4.363	0.135370275	39.57	61.65	16.03	2.622
SPAC343.18	rfp2	0.2812	-4.335	0.550984684	42.01	65.03	4.387	1.69
SPCC1020.07	SPCC1020.07	0.1862	-4.297	0.730020323	31.77	50.6	2.981	1.939
SPAC222.08C	SPAC222.08c	0.2387	-4.297	0.622147581	19.22	32.99	3.752	1.822
SPBC1683.04	SPBC1683.04	0.2862	-4.289	0.543330371	35.7	56.12	4.106	2.154
SPBP8B7.04	mug45	0.6833	-4.288	0.165388579	36.95	57.86	12.99	2.831
SPBC543.10	get1	0.637	-4.28	0.195860568	36.8	57.64	11.17	2.367
SPCC1259.09C	pdx1	0.4245	-4.274	0.372122305	43.01	66.35	6.173	2.005
SPAC2F3.16	SPAC2F3.16	0.633	-4.272	0.19859629	37.66	58.84	11	2.403
SPAC20G4.02C	fus1	0.6623	-4.266	0.178945245	32.2	51.16	12.19	2.059
SPBC4F6.05C	SPBC4F6.05c	0.7328	-4.249	0.135014539	44.14	67.9	15.04	4.44
SPAC110.01	ppk1	0.2957	-4.246	0.529148675	43.17	66.53	4.373	1.856
SPBC1685.13	fhn1	0.3534	-4.24	0.451733455	37.42	58.46	5.271	1.443
SPBC1711.03	aim27	0.6389	-4.238	0.194567112	36.71	57.46	11.24	1.893
SPCC737.06C	SPCC737.06c	0.4341	-4.237	0.362410214	33.19	52.52	6.32	1.878
SPAC227.11C	SPAC227.11c	0.7451	-4.237	0.127785437	35.34	55.53	16.18	3.525
SPCC1840.09	SPCC1840.09	0.724	-4.225	0.140261434	32.01	50.85	13.87	5.386
SPCPB1C11.02	SPCPB1C11.02	0.6391	-4.206	0.194431182	36.55	57.19	10.95	2.661
SPBC13E7.06	msd1	0.7689	-4.206	0.114130139	40.25	62.38	17.85	3.699
SPCC1235.11	SPCC1235.11	0.6522	-4.188	0.185619206	16.38	28.86	9.699	5.464
SPBC13E7.07	SPBC13E7.07	0.3137	-4.182	0.503485481	43.35	66.69	4.101	2.416
SPAP32A8.02	SPAP32A8.02	0.2312	-4.172	0.63601217	42.21	65.08	3.314	2.047
SPBC21C3.06	SPBC21C3.06	0.6571	-4.168	0.182368533	36.76	57.43	11.64	2.268
SPCC645.11C	mug117	0.7262	-4.161	0.138943755	38.41	59.73	14.66	3.421
SPAC9.12C	atp12	0.3389	-4.157	0.469928431	25.06	40.99	4.615	2.188
SPBC16E9.12C	pab2	0.5767	-4.152	0.239050049	36.78	57.43	9.008	2.227
SPBC31F10.17C	SPBC31F10.17c	0.6855	-4.15	0.163992541	41.99	64.74	12.61	2.924
SPCC569.02C	SPCC569.02c	0.7644	-4.147	0.116679322	24.69	40.46	14.43	8.264
SPCC622.18	rpl6	0.486	-4.134	0.313363731	35.06	54.99	6.983	2.08
SPBC16D10.03	pgp2	0.6603	-4.134	0.180258703	39.09	60.65	11.36	3.223

SPCC825.01	SPCC825.01	0.6829	-4.129	0.165642887	38.54	59.88	12.45	2.841
SPCC364.06	nap1	0.708	-4.128	0.149966742	37.05	57.78	13.9	1.88
SPBC365.20C	pnc1	0.6488	-4.103	0.187889159	30.07	47.94	11.29	1.551
SPAPB24D3.04C	mag1	0.6027	-4.083	0.219898809	35.13	55.03	9.575	2.224
SPBC146.02	SPBC146.02	0.353	-4.08	0.452225295	35.23	55.15	5.088	1.31
SPCC18B5.07C	nup61	0.4204	-4.072	0.376337293	42.65	65.56	6.054	1.073
SPBC6B1.02	ppk30	0.2913	-4.053	0.535659515	44.56	68.21	3.378	2.544
SPAPB17E12.03	SPAPB17E12.03	0.5335	-4.028	0.272865576	34.51	54.07	7.192	3.17
SPCC320.12	atp23	0.3808	-4.02	0.41930306	7.129	15.64	3.778	3.273
SPBC1773.09C	mug184	0.3962	-4.02	0.402085529	31.35	49.62	5.534	1.48
SPAC22H12.01C	mug35	0.7345	-4.012	0.1340082	38.33	59.42	15.03	1.525
SPBC21C3.14C	SPBC21C3.14c	0.2828	-4.011	0.548520595	42.59	65.38	4.21	1.175
SPBC1703.14C	top1	0.4151	-4.01	0.381847267	40.51	62.47	5.632	1.919
SPAPB1E7.12	rps602	0.1912	-4.005	0.718512112	41.24	63.48	2.956	1.694
SPBC336.05C	SPBC336.05c	0.3212	-3.998	0.493224463	31.04	49.16	4.22	2.08
SPAC1002.03C	gls2	0.637	-3.979	0.195860568	39.21	60.6	10.23	2.691
SPAC31G5.07	dni1	0.7064	-3.962	0.150949309	37.27	57.85	12.54	4.087
SPCC4G3.15C	not2	0.603	-3.961	0.219682688	39.3	60.7	9.525	1.064
SPBC106.10	pka1	0.2105	-3.947	0.6767479	26	42.03	3.255	1.492
SPBC11B10.06	sws1	0.1907	-3.936	0.719649307	41.45	63.68	2.708	1.842
SPAC30D11.06C	SPAC30D11.06c	0.4279	-3.933	0.368657714	44.66	68.18	5.666	2
SPBC776.01	rpl29	0.7346	-3.924	0.133949076	36.13	56.21	14.49	2.643
SPAC2E1P3.05C	SPAC2E1P3.05c	0.2366	-3.923	0.62598526	31.83	50.17	3.645	1.125
SPAC144.01	SPAC144.01	0.6966	-3.907	0.15701653	41.07	63.11	12.5	2.297
SPCC1223.04C	set11	0.2293	-3.877	0.639595945	38.16	58.98	2.928	2.009
SPCC63.03	SPCC63.03	0.7399	-3.852	0.130826973	39.27	60.5	14.66	2.069
SPAC22E12.05C	rer1	0.7088	-3.834	0.149476291	39.33	60.56	12.56	3.23
SPAC10F6.16	mug134	0.7377	-3.82	0.132120217	36.84	57.05	13.81	4.022
SPBC725.01	SPBC725.01	0.6915	-3.817	0.160207816	36.82	57.02	11.97	2.282
SPAC25G10.03	zip1	0.2777	-3.808	0.55642412	14.49	25.68	2.467	2.609
SPCC1223.12C	meu10	0.1654	-3.795	0.781464495	41.51	63.56	2.204	1.81
SPBC17D1.02	dph2	0.7389	-3.784	0.131414333	40.2	61.72	14.3	2.211
SPAC5H10.11	gmh1	0.1039	-3.772	0.983384452	39.82	61.16	1.652	1.548
SPCC297.05	SPCC297.05	0.5743	-3.772	0.240861184	32.3	50.61	8.291	1.387
SPBC428.17C	wpl1	0.6017	-3.765	0.220619989	37.39	57.74	8.34	3.133
SPBC1198.11C	reb1	0.7188	-3.761	0.143391932	31.06	48.86	11.58	5.453
SPAC4F8.08	mug114	0.7483	-3.756	0.125924255	42.72	65.21	14.66	2.699
SPCC1183.10	wtf10	0.2832	-3.752	0.547906751	43.68	66.55	3.423	2.03
SPCC1235.12C	mug146	0.7905	-3.736	0.102098126	45.22	68.69	17.83	2.462
SPBC56F2.04	utp20	0.1241	-3.734	0.906228219	39.36	60.47	1.727	1.654
SPAPYUG7.03C	mid2	0.2129	-3.724	0.671824339	39.87	61.16	2.49	1.993
SPCPB16A4.03C	ade10	0.2881	-3.722	0.540456742	28.07	44.61	1.569	2.604
SPAC19G12.08	scs7	0.5928	-3.721	0.227091805	34.41	53.5	8.262	2.573
SPAC144.03	ade2	0.2121	-3.704	0.673459331	40.61	62.18	2.794	1.744
SPBC1683.11C	SPBC1683.11c	0.6844	-3.69	0.164689999	38.26	58.86	11.06	2.909
SPAC1071.02	mms19	0.2578	-3.682	0.588717087	24.94	40.16	3.201	1.84
SPAC3G6.13C	rpl4101	0.7167	-3.672	0.144662596	37.61	57.92	12.7	2.084
SPAC25B8.13C	isp7	0.4771	-3.665	0.321390583	35.1	54.39	5.871	2.217
SPBC1703.06	pof10	0.4957	-3.664	0.304781081	43.3	65.9	6.107	2.381
SPCC297.06C	SPCC297.06c	0.7528	-3.664	0.12332039	41.58	63.49	14.35	3.456
SPAC664.01C	swi6	0.7019	-3.655	0.153724758	33.16	51.66	11.62	3.147
SPAPB1A11.01	mfc1	0.3498	-3.652	0.456180195	31.37	49.13	3.969	2.2
SPCC338.18	SPCC338.18	0.4297	-3.65	0.366834646	33.41	52	5.385	1.609
SPAC12B10.16C	mug157	0.6778	-3.646	0.168898435	35.18	54.48	11.12	0.8675
SPBC776.16	SPBC776.16	0.1217	-3.644	0.914709422	39.74	60.87	1.493	1.654
SPAC23C4.09C	SPAC23C4.09c	0.2887	-3.641	0.539553216	42.05	64.1	3.206	2.135
SPBC25H2.05	egd2	0.1907	-3.639	0.719649307	40.72	62.24	2.572	1.647
SPBC800.11	SPBC800.11	0.6538	-3.623	0.184555084	38.56	59.19	9.789	2.665
SPAC26H5.10C	tif51	0.2373	-3.621	0.624702262	41.7	63.59	2.641	2.016
SPBC36B7.04	SPBC36B7.04	0.7193	-3.618	0.14308994	38.61	59.25	12.63	2.086
SPBC18E5.07	SPBC18E5.07	0.6313	-3.609	0.199764211	35.48	54.85	8.917	2.895
SPCC1259.08	SPCC1259.08	0.6398	-3.602	0.193955764	34.44	53.37	9.331	2.491
SPBC646.13	sds23	0.07409	-3.58	1.130240405	24.28	39.09	1.864	0.9189
SPAC6F6.01	cch1	0.3338	-3.58	0.476513668	29.8	46.84	4.197	1.335
SPCC777.07	omh3	0.5361	-3.578	0.270754193	48.58	73.19	6.644	2.489
SPBC354.01	gtp1	0.7724	-3.577	0.112157735	42.89	65.2	15.32	3.523
SPBC16D10.05	mok13	0.723	-3.56	0.140861703	38.09	58.44	12.39	2.903
SPBC365.13C	hba1	0.7452	-3.555	0.127727154	40.41	61.69	13.65	2.718
SPAC31G5.11	pac2	0.7462	-3.535	0.127144755	19.8	32.74	11.23	6.66

SPAC1782.01	ecm29	0.7354	-3.525	0.133476374	40.46	61.72	12.98	2.68
SPBC609.02	ptn1	0.4607	-3.521	0.336581788	33.9	52.5	5.749	1.122
SPAC139.01C	SPAC139.01c	0.5925	-3.521	0.227311645	31.48	49.11	7.377	3.208
SPAC24B11.05	SPAC24B11.05	0.7374	-3.502	0.132296867	31.75	49.46	13.04	2.548
SPCC1884.02	nic1	0.2684	-3.498	0.571217489	32.49	50.49	3.026	1.883
SPAC5D6.12	SPAC5D6.12	0.7399	-3.498	0.130826973	40.11	61.18	13.05	2.932
SPAC7D4.03C	SPAC7D4.03c	0.7584	-3.489	0.120101676	31.73	49.41	13.22	5.039
SPCP25A2.02C	rhp26	0.6842	-3.481	0.16481693	36.7	56.37	10.22	3.258
SPBC1198.01	SPBC1198.01	0.5914	-3.463	0.22811868	35	53.96	7.943	1.52
SPBC2D10.15C	pth1	0.7482	-3.448	0.125982296	42.82	64.92	13.28	3.074
SPAC6G9.09C	rpl2401	0.7241	-3.44	0.140201453	37.87	57.96	11.82	3.36
SPCC569.06	SPCC569.06	0.6803	-3.439	0.167299529	33.37	51.65	8.789	4.848
SPCC1223.06	tea1	0.735	-3.435	0.133712661	32.7	50.7	12.39	3.312
SPAC664.07C	rad9	0.1253	-3.421	0.902048929	38.63	59	1.702	1.475
SPAC4A8.10	SPAC4A8.10	0.3346	-3.421	0.475474063	34.59	53.34	3.247	2.299
SPAC1A6.05C	SPAC1A6.05c	0.6251	-3.418	0.204050501	36.4	55.86	8.137	2.982
SPAC2C4.08	SPAC2C4.08	0.3072	-3.41	0.512578789	41.83	63.48	2.913	2.24
SPAC23A1.03	apt1	0.7593	-3.405	0.1195866	40.91	62.18	13.8	3.019
SPAC23H4.12	alp13	0.1201	-3.401	0.920456993	41.52	63.03	1.231	1.566
SPCC1322.05C	SPCC1322.05c	0.6202	-3.389	0.207468238	35.58	54.67	8.212	2.368
SPAC22A12.06C	SPAC22A12.06c	0.8193	-3.382	0.086557045	28.38	44.56	17.98	5.307
SPAC18G6.05C	SPAC18G6.05c	0.2785	-3.378	0.5551748	32.09	49.77	3.078	1.777
SPAC9G1.02	wis4	0.7332	-3.37	0.134777544	41.09	62.38	12.38	2.219
SPAPB1A10.12C	alo1	0.7201	-3.363	0.142607189	34.13	52.61	10.92	4.156
SPBC21D10.08C	SPBC21D10.08c	0.5889	-3.361	0.229958446	32.63	50.5	7.347	2.376
SPAC139.05	SPAC139.05	0.7147	-3.332	0.145876218	38.53	58.74	11.26	2.554
SPBC530.08	SPBC530.08	0.73	-3.328	0.13667714	37.66	57.51	11.91	2.747
SPAC30D11.02C	SPAC30D11.02c	0.7444	-3.327	0.128193636	37.49	57.27	12.32	3.696
SPAC23C4.08	rho3	0.6606	-3.31	0.180061431	34.05	52.42	9.299	2.005
SPCC338.02	mug112	0.7242	-3.301	0.14014148	36.86	56.35	11.5	2.835
SPAC22A12.01C	pso2	0.7136	-3.299	0.146545159	36.16	55.37	10.99	2.865
SPBC16H5.09C	omh2	0.3034	-3.296	0.517984424	25.07	39.8	3.511	1.362
SPBP8B7.30C	thi5	0.5982	-3.284	0.223153591	28.75	44.94	7.498	2.07
SPAC144.04C	spe1	0.575	-3.281	0.240332155	35.99	55.1	6.697	2.624
SPBC16G5.02C	SPBC16G5.02c	0.4632	-3.268	0.334231449	44.63	67.21	4.534	2.62
SPAC1556.02C	sdh1	0.7157	-3.262	0.145268983	36.52	55.81	11.21	1.976
SPCC1450.07C	SPCC1450.07c	0.7162	-3.247	0.144965683	37.69	57.44	11.17	2.034
SPBC6B1.04	mde4	0.2858	-3.244	0.543937776	15.32	26.04	3.385	1.114
SPAC17A5.16	ftp105	0.5999	-3.24	0.221921138	36.91	56.33	7.364	2.228
SPAC1705.02	SPAC1705.02	0.7364	-3.238	0.13288622	37.79	57.57	11.84	2.886
SPBC354.15	fap1	0.6941	-3.235	0.158577956	39.27	59.64	10.16	2.276
SPAC8C9.06C	ppr4	0.7028	-3.232	0.153168247	37.72	57.46	10.76	0.8477
SPBC800.07C	tsf1	0.3369	-3.211	0.472498989	18.11	29.92	3.832	1.076
SPAC5H10.06C	adh4	0.4265	-3.207	0.370080964	34.42	52.8	4.468	1.878
SPAC694.04C	SPAC694.04c	0.7498	-3.2	0.125054564	36.71	55.99	12.12	3.637
SPCC61.03	SPCC61.03	0.7272	-3.195	0.13834613	38.38	58.34	11.06	3.291
SPAC23C4.12	hhp2	0.3774	-3.193	0.423198104	36.4	55.55	3.66	2.083
SPBC4.01	dni2	0.7797	-3.186	0.108072466	38.52	58.52	13.95	3.739
SPBC13G1.12	did2	0.7893	-3.174	0.102757897	34.19	52.43	14.63	3.673
SPAC24B11.07C	SPAC24B11.07c	0.5272	-3.169	0.278024598	37.9	57.62	5.579	2.44
SPCC737.07C	SPCC737.07c	0.4163	-3.161	0.380593589	45.07	67.67	4.315	1.787
SPCC24B10.14C	xlf1	0.7124	-3.144	0.147276089	38.49	58.42	10.39	2.791
SPBC31F10.14C	hip3	0.1907	-3.136	0.719649307	23.87	37.89	2.418	1.188
SPAC23C11.07	SPAC23C11.07	0.6706	-3.136	0.173536451	36.53	55.65	9.177	1.673
SPAC17D4.01	pex7	0.154	-3.133	0.812479279	41.77	63.01	1.491	1.557
SPCC737.05	SPCC737.05	0.7973	-3.121	0.098378236	39.38	59.64	15.24	2.942
SPAC17G6.02C	tco1	0.6279	-3.12	0.202109517	37.87	57.52	7.82	2.01
SPAC1B2.04	cox6	0.4352	-3.119	0.361311113	5.16	11.62	4.45	1.837
SPAC25B8.18	SPAC25B8.18	0.5984	-3.107	0.223008415	39.97	60.44	5.572	3.934
SPAC227.17C	SPAC227.17c	0.5512	-3.106	0.258690791	38.04	57.74	5.798	2.567
SPBC21B10.05C	pop3	0.4874	-3.089	0.312114475	33.39	51.18	5.109	1.847
SPBC25B2.02C	mam1	0.1776	-3.086	0.750557039	41.32	62.31	1.501	1.661
SPBPB21E7.01C	eno102	0.6531	-3.083	0.185020316	36.4	55.41	7.81	3.243
SPAC458.04C	dil1	0.38	-3.075	0.420216403	45.73	68.49	3.784	1.726
SPCP1E11.11	puf6	0.4105	-3.071	0.386686839	35.15	53.63	3.885	2.061
SPAC24H6.09	gef1	0.7586	-3.06	0.119987162	37.11	56.36	12.54	2.053
SPAC23A1.14C	SPAC23A1.14c	0.7261	-3.049	0.139003563	34.15	52.19	10.8	2.305
SPAC186.08C	SPAC186.08c	0.4963	-3.021	0.304255725	37.16	56.37	5.034	1.977
SPCC622.16C	epe1	0.4916	-3.019	0.308388126	35.77	54.42	4.836	2.17

SPBC4C3.09	SPBC4C3.09	0.7568	-3.019	0.121018877	39.48	59.64	11.96	3.09
SPBC1539.02	SPBC1539.02	0.2729	-3.017	0.563996464	40.86	61.57	2.074	1.99
SPAC3A12.09C	SPAC3A12.09c	0.5727	-2.998	0.242072817	36.62	55.59	6.028	2.471
SPBC18E5.10	SPBC18E5.10	0.7561	-2.993	0.121420762	36.54	55.47	11.75	3.235
SPAC664.14	amt2	0.6393	-2.99	0.194295296	30.89	47.54	7.778	1.938
SPBC16E9.09C	SPBC16E9.09c	0.8575	-2.98	0.066765871	29.79	45.98	17.31	9.98
SPAC637.03	SPAC637.03	0.7628	-2.974	0.117589316	39.03	58.94	11.8	3.805
SPBC1711.01C	mat1-m	0.7624	-2.972	0.117817112	37.99	57.47	11.98	3.323
SPBC19G7.04	SPBC19G7.04	0.3867	-2.971	0.412625828	38	57.48	3.819	1.887
SPAC22F8.03C	SPAC22F8.03c	0.3931	-2.954	0.405496956	44.24	66.23	3.873	1.494
SPAC4C5.03	SPAC4C5.03	0.5165	-2.95	0.286929674	34.59	52.67	5.354	1.654
SPAC1834.09	mug51	0.3332	-2.938	0.477295007	40.9	61.51	2.727	2.005
SPBC19G7.17	SPBC19G7.17	0.7014	-2.934	0.154034238	26.36	41.11	7.023	5.302
SPCC18.06C	caf1	0.7516	-2.93	0.124013229	34.37	52.34	11.52	2.406
SPAC23C4.06C	SPAC23C4.06c	0.8384	-2.925	0.07654873	46.81	69.78	15.55	8.027
SPBC215.10	SPBC215.10	0.681	-2.915	0.166852888	37.26	56.38	8.137	3.286
SPBC15D4.01C	klp9	0.4191	-2.905	0.377682339	42.27	63.39	3.838	1.877
SPAC23H3.03C	npr2	0.6293	-2.896	0.201142268	31.26	47.93	7.166	2.188
SPAC57A7.05	SPAC57A7.05	0.7833	-2.886	0.106071873	35.8	54.28	12.69	3.812
SPCC4B3.11C	SPCC4B3.11c	0.2913	-2.884	0.535659515	42.9	64.24	1.91	2.046
SPAC3H8.02	SPAC3H8.02	0.622	-2.884	0.206209615	24.91	39	6.717	2.643
SPCC794.15	SPCC794.15	0.3299	-2.88	0.481617684	40.67	61.1	2.254	2.166
SPAC17A5.14	exo2	0.7446	-2.877	0.128076968	39.12	58.92	10.83	2.779
SPBC713.09	SPBC713.09	0.7403	-2.868	0.130592251	36.94	55.86	10.53	2.941
SPAC27D7.13C	ssm4	0.3122	-2.859	0.505567101	38.91	58.6	2.17	2.056
SPAC2F7.10	akr1	0.7518	-2.857	0.123897679	36.62	55.4	11.41	1.682
SPCC16C4.07	scw1	0.3619	-2.854	0.441411417	38.38	57.85	2.776	2.109
SPAC20G4.08	SPAC20G4.08	0.7502	-2.853	0.12482294	36.06	54.6	11.31	1.69
SPAPB24D3.08C	SPAPB24D3.08c	0.7888	-2.85	0.103033098	41.44	62.14	13.25	2.847
SPAC2H10.02C	SPAC2H10.02c	0.5899	-2.847	0.229221604	48.2	71.62	5.842	2.688
SPBC11C11.01	SPBC11C11.01	0.7309	-2.842	0.136142038	38.24	57.64	10.23	2.284
SPBPB10D8.01	SPBPB10D8.01	0.7133	-2.839	0.146727776	32.76	49.95	9.371	2.654
SPCC1322.12C	bub1	0.7411	-2.821	0.130123187	30.74	47.09	10.41	2.84
SPCC16C4.10	SPCC16C4.10	0.4879	-2.818	0.311669182	30.05	46.12	2.829	3.186
SPAC20H4.05C	SPAC20H4.05c	0.7536	-2.809	0.12285911	40.25	60.41	10.84	3.168
SPAC19G12.13C	poz1	0.7129	-2.805	0.146971385	36.92	55.74	9.563	1.573
SPAC144.14	klp8	0.7751	-2.804	0.110642263	37.62	56.72	12.12	2.95
SPBC21B10.12	rec6	0.8016	-2.802	0.096042291	39.9	59.92	13.99	2.672
SPAC20H4.03C	tfs1	0.1774	-2.798	0.751046385	38.59	58.07	1.947	1.186
SPAC3A11.04	SPAC3A11.04	0.8342	-2.789	0.078729814	26.25	40.74	14.25	7.648
SPBC21B10.04C	nrf1	0.2293	-2.773	0.639595945	41.47	62.08	1.63	1.68
SPBC32F12.12C	SPBC32F12.12c	0.7271	-2.762	0.138405855	40	60	9.784	2.233
SPAC607.07C	SPAC607.07c	0.8161	-2.751	0.088256622	41.02	61.41	14.59	3.681
SPAC23G3.04	ies4	0.668	-2.749	0.175223538	39.48	59.26	6.651	3.815
SPAC9G1.07	SPAC9G1.07	0.6614	-2.724	0.179535809	36.29	54.74	7.213	2.74
SPCC417.06C	mug27	0.7279	-2.722	0.137928281	37.54	56.49	9.509	2.657
SPBC216.04C	SPBC216.04c	0.7895	-2.714	0.102647866	38.44	57.74	12.22	3.885
SPBP4H10.16C	SPBP4H10.16c	0.7132	-2.712	0.146788665	33.39	50.65	8.093	3.956
SPBC13A2.02	nup82	0.3572	-2.708	0.44708855	38.99	58.51	2.14	2.213
SPCC737.09C	hmt1	0.5698	-2.706	0.244277555	35.25	53.25	5.387	2.23
SPAC11E3.03	pcs1	0.644	-2.705	0.191114133	20.95	33.19	6.328	3.205
SPCC1259.11C	gyp2	0.5026	-2.703	0.298777516	33.89	51.34	4.459	1.982
SPAC22E12.04	ccs1	0.2626	-2.702	0.580705278	19.18	30.71	2.466	1.259
SPAC186.07C	SPAC186.07c	0.3171	-2.695	0.498803758	44.75	66.57	1.83	2.051
SPAC13A11.06	SPAC13A11.06	0.7815	-2.695	0.107071018	38.4	57.67	12.08	2.666
SPAC22A12.14C	SPAC22A12.14c	0.7661	-2.691	0.115714538	38.16	57.32	11.17	2.692
SPAC6B12.08	mug185	0.7935	-2.661	0.100453069	27.88	42.85	10.75	5.945
SPBC25H2.10C	SPBC25H2.10c	0.7809	-2.66	0.107404577	33.76	51.1	11.86	2.733
SPCC777.17C	SPCC777.17c	0.6963	-2.643	0.157203605	36.44	54.84	8.023	2.71
SPAC3H1.06C	SPAC3H1.06c	0.6903	-2.642	0.160962127	34.1	51.56	7.887	2.588
SPAC1296.03C	sxa2	0.7535	-2.642	0.122916743	36.47	54.87	10.33	2.622
SPCC14G10.03C	ump1	0.8017	-2.609	0.095988116	17.92	28.81	10.62	6.42
SPAC29B12.04	snz1	0.3646	-2.581	0.438183336	22.38	35.02	3.31	0.8419
SPBC17G9.09	tif213	0.8466	-2.576	0.072321736	43.89	65.2	16.9	2.57
SPAC27D7.02C	SPAC27D7.02c	0.2848	-2.567	0.545460015	43.78	65.03	1.583	1.823
SPBC405.05	SPBC405.05	0.7854	-2.566	0.104909103	38.74	57.95	11.71	2.608
SPBC405.04C	ypt7	0.7557	-2.553	0.121650578	36.95	55.43	9.57	3.632
SPAC1751.04	SPAC1751.04	0.7445	-2.54	0.128135298	36.55	54.85	9.288	3.081
SPBC17D11.04C	nto1	0.4189	-2.536	0.37788964	35.56	53.45	3.362	1.622

SPAC343.12	rds1	0.7341	-2.536	0.134244776	38.3	57.3	9.011	2.703
SPAC2F7.06C	pol4	0.7445	-2.531	0.128135298	35.63	53.54	9.145	3.271
SPAC6G10.08	idp1	0.7923	-2.521	0.101110344	34.29	51.66	10.11	5.613
SPAC30D11.05	aps3	0.6122	-2.518	0.213106675	34.58	52.05	5.467	2.579
SPBC1703.13C	SPBC1703.13c	0.4456	-2.513	0.351054818	44.2	65.54	2.844	2.313
SPBC1734.05C	spf31	0.5527	-2.507	0.257510535	39.81	59.37	5.043	1.423
SPBC947.04	SPBC947.04	0.5332	-2.499	0.273109859	35.71	53.62	4.313	2.156
SPAC1002.12C	SPAC1002.12c	0.8541	-2.498	0.068491278	42.41	63.02	17.05	3.38
SPBC27B12.11C	SPBC27B12.11c	0.6995	-2.496	0.155212281	17.39	27.9	7.579	2.751
SPBC1685.05	SPBC1685.05	0.3361	-2.494	0.473531488	40.19	59.89	1.457	2.067
SPBC1347.03	meu14	0.8154	-2.493	0.088629293	40.24	59.96	13.34	2.793
SPAC6C3.03C	SPAC6C3.03c	0.3503	-2.486	0.455559863	42.43	63.02	2.738	1.462
SPBC26H8.05C	SPBC26H8.05c	0.7825	-2.485	0.106515654	37.84	56.58	10.99	3.033
SPBC1685.06	cid11	0.7601	-2.479	0.119129267	37.22	55.7	9.686	3.204
SPAC222.13C	SPAC222.13c	0.747	-2.477	0.126679398	36.79	55.09	9.47	2.268
SPAC13F5.01C	msh1	0.7512	-2.47	0.124244421	30.19	45.82	9.54	2.508
SPCC622.11	SPCC622.11	0.8046	-2.47	0.094419972	39.09	58.32	12.37	2.918
SPBC530.06C	SPBC530.06c	0.7729	-2.455	0.111876693	15.44	25.1	8.386	5.514
SPCC4B3.06C	SPCC4B3.06c	0.4215	-2.452	0.375202421	43.41	64.35	2.882	1.964
SPAC16C9.07	pom2	0.77	-2.452	0.113509275	35.54	53.3	9.725	3.814
SPAC20G8.09C	SPAC20G8.09c	0.7349	-2.45	0.133771753	36.57	54.75	9.037	1.745
SPAPB8E5.04C	SPAPB8E5.04c	0.8703	-2.444	0.060331016	26.3	40.33	16.55	8.021
SPBC12C2.07C	SPBC12C2.07c	0.6413	-2.441	0.19293876	35.39	53.08	6.369	1.642
SPCC320.04C	SPCC320.04c	0.7134	-2.44	0.146666895	37.53	56.08	8.087	2.195
SPAPB17E12.12C	SPAPB17E12.12c	0.8382	-2.433	0.076652344	41.56	61.73	14.81	3.413
SPBC25H2.09	SPBC25H2.09	0.7825	-2.43	0.106515654	37.9	56.58	10.7	3.069
SPAC589.02C	med13	0.8415	-2.428	0.07494588	24.29	37.48	11.42	8.303
SPBC2G2.09C	crs1	0.7859	-2.421	0.104632711	36.33	54.38	11.01	2.656
SPBC365.07C	SPBC365.07c	0.7731	-2.412	0.111764327	39.98	59.47	10.51	1.864
SPAC1399.01C	SPAC1399.01c	0.7304	-2.404	0.136439235	37.03	55.33	8.206	2.941
SPBP35G2.12	SPBP35G2.12	0.7356	-2.399	0.133358279	37.28	55.67	8.55	2.617
SPAPB24D3.02C	SPAPB24D3.02c	0.8215	-2.399	0.085392432	40.68	60.44	13.35	2.547
SPBC2D10.12	rhp23	0.39	-2.392	0.408935393	22.28	34.62	3.243	0.8759
SPBC14F5.11C	mug186	0.7821	-2.382	0.106737714	40.65	60.37	10.77	2.141
SPCC1919.13C	SPCC1919.13c	0.7292	-2.38	0.13715334	51.33	75.36	8.384	2.274
SPAC1805.09C	fmt1	0.7019	-2.371	0.153724758	31.66	47.75	7.153	2.816
SPCC1223.01	SPCC1223.01	0.7743	-2.37	0.111090741	39.24	58.38	10.31	2.136
SPCC1840.05C	SPCC1840.05c	0.8033	-2.367	0.095122233	38.45	57.27	11.86	2.519
SPBC337.11	SPBC337.11	0.8786	-2.363	0.056208801	49.14	72.27	16.95	8.465
SPAC21E11.03C	pcr1	0.7936	-2.355	0.100398341	37.28	55.62	11.4	1.692
SPAC4G9.14	SPAC4G9.14	0.8119	-2.343	0.090497459	39.76	59.08	11.89	3.663
SPAC890.06	nup155	0.7649	-2.331	0.116395339	37.1	55.32	9.492	2.664
SPAC1250.02	mug95	0.6145	-2.329	0.211478113	32.05	48.23	4.627	2.885
SPCC11E10.07C	SPCC11E10.07c	0.5568	-2.326	0.254300773	18.3	28.94	4.385	1.98
SPAC26F1.07	SPAC26F1.07	0.806	-2.301	0.093664958	27.73	42.14	9.057	6.221
SPAC926.09C	fas1	0.7718	-2.28	0.112495226	38.27	56.9	9.83	1.944
SPAC732.02C	SPAC732.02c	0.7489	-2.279	0.125576169	40.1	59.46	8.516	2.752
SPAC105.01C	kha1	0.334	-2.268	0.476253533	40.2	59.59	1.47	1.833
SPBC13E7.08C	SPBC13E7.08c	0.7719	-2.265	0.112438959	34.94	52.21	9.753	1.993
SPBC839.06	cta3	0.5074	-2.258	0.294649537	35.71	53.27	3.257	2.208
SPCC4E9.02	cig1	0.5441	-2.258	0.264321274	44.76	65.97	2.613	2.935
SPBC1348.02	SPBC1348.02	0.8552	-2.258	0.067932308	29.53	44.6	13.5	6.815
SPBC8D2.18C	SPBC8D2.18c	0.797	-2.254	0.098541679	36.73	54.7	10.92	2.339
SPBC342.06C	rtt109	0.7364	-2.253	0.13288622	39	57.88	8.505	0.8659
SPCC1620.02	wtf23	0.4549	-2.249	0.342084063	44.01	64.91	2.623	2.103
SPBC29A3.07C	sab14	0.3915	-2.248	0.407268234	26.43	40.25	2.988	1.022
SPCC1827.04	SPCC1827.04	0.8176	-2.248	0.087459117	37.96	56.41	11.85	3.458
SPBPB8B6.04C	grt1	0.3876	-2.242	0.411616232	39.33	58.33	2.534	1.587
SPAC4F10.11	spn1	0.7333	-2.239	0.134718315	34.28	51.24	8.221	1.52
SPCC23B6.04C	SPCC23B6.04c	0.8315	-2.233	0.080137746	40.47	59.91	12.49	4.292
SPBC1711.09C	SPBC1711.09c	0.8275	-2.23	0.082231998	36.13	53.82	12.24	4.066
SPBC215.01	SPBC215.01	0.3621	-2.223	0.441171475	28.93	43.71	2.623	1.201
SPBC29A10.12	SPBC29A10.12	0.7973	-2.222	0.098378236	36.75	54.68	10.64	2.724
SPCC162.11C	SPCC162.11c	0.5139	-2.217	0.289121382	40.54	59.99	4.167	0.6203
SPAC869.09	SPAC869.09	0.669	-2.208	0.174573882	34.31	51.23	6.171	1.926
SPCC23B6.03C	tel1	0.8395	-2.207	0.0759793	39.8	58.95	13.26	3.845
SPAC1F8.01	ght3	0.5107	-2.201	0.291834142	38.68	57.36	1.926	2.755
SPBC1734.11	mas5	0.7518	-2.19	0.123897679	9.903	16.97	6.251	4.883
SPBP26C9.02C	car1	0.8088	-2.189	0.092158857	38.62	57.25	11.41	1.952

SPAC1805.12C	uep1	0.8124	-2.17	0.090230085	37.99	56.35	10.71	4.025
SPBC215.13	SPBC215.13	0.8178	-2.168	0.087352894	39.32	58.21	11.36	3.524
SPAC9E9.15	SPAC9E9.15	0.8296	-2.149	0.081131257	39.27	58.11	12.5	2.531
SPAP11E10.01	SPAP11E10.01	0.79	-2.146	0.102372909	36.82	54.67	9.894	2.584
SPBC3B9.09	vps36	0.7129	-2.137	0.146971385	34.43	51.31	6.733	2.604
SPAC4D7.01C	sec71	0.3623	-2.114	0.440931666	39.53	58.43	1.771	1.715
SPAC2F7.08C	snf5	0.8071	-2.109	0.093072653	36.76	54.54	10.38	3.315
SPBC18H10.06C	swd2	0.7956	-2.106	0.099305226	28.76	43.3	8.92	4.794
SPBC839.07	ibp1	0.323	-2.104	0.490797478	39.19	57.93	1.194	1.69
SPCC576.14	dph5	0.7706	-2.099	0.113170995	36.84	54.64	8.928	2.02
SPAC17G8.13C	mst2	0.6187	-2.084	0.208519884	36.99	54.82	5.264	1.517
SPAC630.04C	SPAC630.04c	0.766	-2.073	0.11577123	37.85	56.02	7.933	3.386
SPBC83.09C	SPBC83.09c	0.7155	-2.068	0.145390362	35.37	52.53	6.5	2.67
SPAC343.07	mug28	0.8133	-2.063	0.089749228	37.99	56.2	9.99	4.224
SPAC1687.14C	SPAC1687.14c	0.8207	-2.06	0.085815567	41.28	60.81	11.51	1.786
SPBC1773.12	SPBC1773.12	0.5315	-2.051	0.274496731	16.58	26.14	3.42	1.881
SPBC2G2.07C	mug178	0.6336	-2.047	0.198184831	24.1	36.69	2.398	3.47
SPAPB1E7.04C	SPAPB1E7.04c	0.8789	-2.047	0.056060536	33.28	49.57	15.02	7.004
SPBC27B12.05	SPBC27B12.05	0.8378	-2.045	0.076859644	42.25	62.16	12.66	1.956
SPAC11D3.01C	SPAC11D3.01c	0.7878	-2.041	0.103584024	37.18	55.02	8.895	3.293
SPAC2G11.05C	SPAC2G11.05c	0.8076	-2.012	0.09280369	36.75	54.39	10.32	2.144
SPBC29A3.21	SPBC29A3.21	0.8462	-2.008	0.072526979	33.83	50.29	11.69	5.235
SPAPYUG7.02C	sin1	0.6912	-1.995	0.160396271	17.69	27.62	5.509	2.949
SPAC4G9.19	SPAC4G9.19	0.758	-1.99	0.120330794	32.3	48.11	7.742	2.471
SPAC19A8.10	rfp1	0.7495	-1.987	0.125228363	37.19	54.97	7.552	2.176
SPAC17H9.12C	SPAC17H9.12c	0.7243	-1.986	0.140081515	32.07	47.78	6.407	2.716
SPCC1322.16	phb2	0.7297	-1.984	0.136855654	31.92	47.57	7.122	1.548
SPBC1711.14	rec15	0.8955	-1.983	0.04793441	31.11	46.44	15.69	9.152
SPAC19A8.11C	SPAC19A8.11c	0.8322	-1.976	0.079772289	38.44	56.71	11.59	2.684
SPAC18B11.08C	SPAC18B11.08c	0.8158	-1.973	0.088416299	38.43	56.68	10.19	3.216
SPAC1783.06C	atg12	0.7833	-1.97	0.106071873	38.25	56.44	8.842	2.167
SPCC132.04C	gdh2	0.8299	-1.968	0.080974235	36.97	54.63	10.8	3.923
SPAC513.06C	SPAC513.06c	0.8231	-1.963	0.084547398	38.05	56.14	10.67	3.127
SPBC2D10.03C	SPBC2D10.03c	0.7422	-1.96	0.12947905	37.61	55.51	7.282	1.946
SPCC1442.07C	SPCC1442.07c	0.3516	-1.953	0.453951134	38.4	56.61	1.65	1.523
SPAC6G9.04	spo7	0.8324	-1.949	0.079667928	36.6	54.09	11.43	2.678
SPAC1F7.08	fio1	0.8513	-1.942	0.069917367	21.45	32.82	12.41	4.1
SPAC144.06	apl5	0.7308	-1.923	0.136201461	30.02	44.82	6.173	2.952
SPCC4G3.17	SPCC4G3.17	0.8154	-1.923	0.088629293	39.91	58.69	10.38	1.832
SPAC3C7.07C	SPAC3C7.07c	0.6939	-1.917	0.158703113	32.45	48.23	5.075	2.889
SPAC9E9.05	SPAC9E9.05	0.8209	-1.907	0.085709744	40.37	59.31	10.33	2.767
SPAC694.06C	mrc1	0.8401	-1.906	0.075669015	35.3	52.2	11.63	3.022
SPCC970.02	SPCC970.02	0.8806	-1.903	0.055221319	39.33	57.86	14.99	5.375
SPBC839.15C	ef1a-c	0.8817	-1.903	0.054679159	22.6	34.38	12.91	8.089
SPAC1006.09	win1	0.7982	-1.899	0.097888277	35.6	52.61	7.785	4.412
SPCC613.02	SPCC613.02	0.784	-1.898	0.105683937	37.85	55.77	8.579	1.987
SPBC20F10.02C	SPBC20F10.02c	0.4991	-1.897	0.30181243	42.87	62.81	1.681	2.29
SPBC17G9.08C	cnt5	0.4951	-1.888	0.305307074	19.36	29.82	3.153	1.204
SPAC644.08	SPAC644.08	0.7952	-1.886	0.099523629	35.84	52.93	9.097	1.786
SPAC22E12.11C	set3	0.3458	-1.867	0.461175011	26.6	39.95	2.151	0.9297
SPAC25G10.01	SPAC25G10.01	0.4789	-1.867	0.319755163	39.28	57.73	2.165	1.924
SPBC660.06	SPBC660.06	0.7989	-1.866	0.097507579	30.55	45.48	8.645	3.103
SPAC19B12.08	atg4	0.8311	-1.863	0.080346718	38.12	56.1	10.93	2.253
SPBC18E5.13	SPBC18E5.13	0.8394	-1.851	0.076031035	39.52	58.04	11.23	2.962
SPAC869.06C	SPAC869.06c	0.8595	-1.847	0.065754119	30.14	44.88	12.01	4.958
SPAC1527.01	mok11	0.5778	-1.842	0.238222462	41.39	60.66	3.813	1.44
SPAC30D11.01C	SPAC30D11.01c	0.7707	-1.839	0.113114641	36.28	53.48	7.059	3.238
SPCC285.10C	SPCC285.10c	0.8449	-1.827	0.07319469	39.37	57.8	11.73	2.291
SPAC23D3.10C	eng2	0.8501	-1.823	0.070529984	35.4	52.22	11.73	3.458
SPAC29E6.05C	SPAC29E6.05c	0.838	-1.812	0.076755981	40.06	58.76	10.66	3.402
SPBC17D11.03C	SPBC17D11.03c	0.8608	-1.811	0.065097742	42.17	61.72	12.98	2.533
SPBC21D10.07	cmc1	0.7722	-1.807	0.112270203	37.86	55.65	7.45	2.47
SPBC3E7.05C	SPBC3E7.05c	0.7022	-1.806	0.153539175	27.84	41.6	5.426	2.196
SPCP31B10.04	SPCP31B10.04	0.8272	-1.804	0.082389474	37.69	55.42	9.632	3.72
SPBC1734.15	rsc4	0.7523	-1.782	0.123608938	33.94	50.12	4.884	4.109
SPCC16C4.14C	sfc4	0.7352	-1.778	0.133594502	35.42	52.2	6.052	2.443
SPBC1711.06	rpl401	0.8528	-1.777	0.069152808	40.57	59.42	11.95	2.617
SPAC19A8.14	SPAC19A8.14	0.845	-1.773	0.073143291	38.83	56.98	11.11	3.052
SPBC1289.06C	ppr8	0.6518	-1.756	0.185885644	36.33	53.44	4.57	1.602

SPBC18H10.02	lcf1	0.7271	-1.756	0.138405855	18.18	27.97	5.535	2.945
SPAC890.03	ppk16	0.7857	-1.753	0.104743247	32.55	48.13	8.05	1.657
SPBPJ4664.02	SPBPJ4664.02	0.8501	-1.748	0.070529984	40.33	59.05	11.47	2.76
SPCC645.08C	snd1	0.8342	-1.747	0.078729814	40.58	59.39	10.39	2.312
SPAC9.06C	SPAC9.06c	0.7134	-1.743	0.146666895	36.96	54.31	5.262	2.472
SPBC2G2.02	syj1	0.8149	-1.723	0.088895682	37.45	54.96	8.679	3.14
SPBC4C3.04C	SPBC4C3.04c	0.8425	-1.721	0.07443009	38.9	56.99	11.04	1.425
SPCC1672.09	SPCC1672.09	0.7865	-1.717	0.104301273	33.09	48.84	7.887	1.723
SPAC11E3.04C	ubc13	0.4965	-1.715	0.304080747	42.87	62.56	1.417	2.081
SPBC776.09	ste13	0.8353	-1.696	0.078157519	15.34	23.91	8.281	5.104
SPBC31F10.10C	SPBC31F10.10c	0.5501	-1.69	0.259558355	32.68	48.23	3.05	1.518
SPAC57A10.03	cyp1	0.8036	-1.688	0.094960072	36.22	53.19	7.772	3.263
SPBC530.05	SPBC530.05	0.9008	-1.688	0.045371622	31.39	46.41	14.25	8.03
SPAC56F8.12	SPAC56F8.12	0.7147	-1.686	0.145876218	31.55	46.64	5.644	1.45
SPBC31F10.15C	atp15	0.7107	-1.685	0.148313685	18.11	27.78	4.947	2.705
SPCC306.04C	set1	0.7174	-1.668	0.144238628	33.55	49.42	5.835	0.6811
SPAPB1A10.07C	SPAPB1A10.07c	0.8329	-1.667	0.079407138	33.42	49.23	9.91	1.954
SPAC1F3.10C	Oct-01	0.7889	-1.657	0.102978044	36.09	52.96	7.484	2.262
SPAC1834.07	klp3	0.8494	-1.652	0.070887743	39.29	57.45	10.28	3.707
SPAPB17E12.04C	csn2	0.811	-1.644	0.090979146	32.63	48.09	8.347	2.463
SPAC1782.08C	rex3	0.8084	-1.64	0.092373695	29.76	44.06	7.748	3.24
SPAC5D6.10C	mug116	0.5968	-1.638	0.224171186	41.91	61.1	2.594	2.268
SPBC354.10	def1	0.8931	-1.629	0.049099911	22.31	33.59	12.58	7.366
SPAC110.02	pds5	0.8554	-1.627	0.067830754	16.35	25.22	9.07	5.588
SPCC188.09C	SPCC188.09c	0.8299	-1.626	0.080974235	35.67	52.33	9.445	2.033
SPAC13G7.04C	mac1	0.7882	-1.62	0.103363569	36.02	52.81	6.679	3.191
SPAPJ691.03	SPAPJ691.03	0.4999	-1.618	0.301116863	43.33	63.07	1.05	2.039
SPAC31G5.21	SPAC31G5.21	0.8485	-1.615	0.071348153	36.34	53.25	10.26	3.062
SPBC16D10.07C	sir2	0.718	-1.601	0.143875556	37.91	55.44	4.527	2.907
SPAC1639.02C	trk2	0.792	-1.594	0.101274818	5.892	10.5	6.283	3.885
SPAC12G12.11C	SPAC12G12.11c	0.8758	-1.591	0.057595059	36.11	52.89	12.2	4.051
SPAPB8E5.08	SPAPB8E5.08	0.8635	-1.586	0.063737658	40.23	58.67	11.43	2.795
SPAC1D4.03C	aut12	0.6754	-1.584	0.170438944	32.69	48.1	4.086	2.086
SPAC6G9.10C	sen1	0.8323	-1.572	0.079720105	31.26	46.06	8.65	3.344
SPBC18A7.01	SPBC18A7.01	0.7431	-1.565	0.128952739	34.5	50.6	5.428	2.325
SPBC428.12C	SPBC428.12c	0.7867	-1.563	0.10419085	36.53	53.45	6.33	3.134
SPAC22H10.09	SPAC22H10.09	0.6433	-1.56	0.191586449	26.72	39.67	2.379	2.59
SPCC4F11.03C	SPCC4F11.03c	0.8192	-1.558	0.086610056	34.45	50.52	8.214	2.581
SPAC25B8.05	SPAC25B8.05	0.5249	-1.555	0.279923427	27.41	40.64	2.026	1.728
SPAC10F6.13C	SPAC10F6.13c	0.8231	-1.551	0.084547398	16.08	24.74	5.561	5.3
SPAC22G7.04	ubp13	0.9227	-1.551	0.034939479	47.3	68.54	18.08	8.096
SPCC18B5.11C	cds1	0.8243	-1.55	0.0839147	37.61	54.94	8.557	2.315
SPAC1782.06C	phb1	0.4757	-1.544	0.322666849	27.99	41.44	2.571	0.6771
SPBC17A3.02	SPBC17A3.02	0.8018	-1.533	0.095933948	38.55	56.25	6.884	3.09
SPBC1271.03C	SPBC1271.03c	0.7948	-1.532	0.099742142	35.72	52.26	6.735	2.838
SPBC13G1.03C	pex14	0.8002	-1.53	0.096801453	24.67	36.76	5.994	3.894
SPBC28F2.02	mep33	0.7431	-1.528	0.128952739	40.83	59.43	3.991	3.414
SPCC1020.10	oca2	0.7695	-1.523	0.113791376	37.57	54.86	5.867	2.598
SPAC6B12.16	meu26	0.8571	-1.522	0.066968505	36.63	53.53	10.72	1.643
SPBC29A10.16C	SPBC29A10.16c	0.9148	-1.51	0.038673844	27	40	13.87	9.243
SPAC19B12.07C	SPAC19B12.07c	0.7873	-1.509	0.103859749	34.73	50.85	6.133	3.031
SPAC821.03C	SPAC821.03c	0.8678	-1.509	0.061580354	40.54	59	11.4	2.226
SPBC1105.09	ubc15	0.8221	-1.506	0.085075352	33.51	49.14	8.038	2.602
SPAC12B10.01C	SPAC12B10.01c	0.7383	-1.49	0.131767132	37.19	54.28	5.195	1.973
SPBC530.04	mod5	0.8985	-1.485	0.046481919	35.77	52.28	13.75	5.008
SPCC645.02	gep4	0.8626	-1.482	0.064190546	40.34	58.68	10.15	3.594
SPAC1071.03C	SPAC1071.03c	0.8716	-1.475	0.059682778	39.37	57.31	11.46	2.311
SPBC4B4.10C	atg5	0.8557	-1.473	0.067678468	42.86	62.21	10.2	1.871
SPAC6C3.05	SPAC6C3.05	0.7963	-1.467	0.098923284	33.87	49.58	6.149	3.176
SPAC1782.07	qcr8	0.8348	-1.464	0.07841756	28.28	41.73	8.119	3.267
SPBC14C8.03	fma2	0.8507	-1.464	0.070223567	32.85	48.15	9.804	1.756
SPAC23C4.03	hrk1	0.9107	-1.462	0.040624663	24.73	36.74	12.78	8.563
SPAC10F6.15	SPAC10F6.15	0.8159	-1.451	0.088363067	37.71	54.95	7.505	2.365
SPCC2H8.05C	SPCC2H8.05c	0.8687	-1.443	0.061130178	38.81	56.48	11.02	1.968
SPBC17F3.01C	rga5	0.8272	-1.437	0.082389474	34.49	50.4	7.245	3.552
SPBP8B7.21	ubp3	0.8755	-1.435	0.05774385	36.58	53.34	11.52	2.21
SPCC622.15C	SPCC622.15c	0.6701	-1.434	0.173860382	37.23	54.26	4.051	1.198
SPBC23G7.16	ctr6	0.474	-1.431	0.324221658	23.78	35.37	1.536	1.506
SPCC622.08C	hta1	0.5596	-1.428	0.252122294	40.2	58.41	1.567	1.984

SPBC2D10.20	ubc1	0.6483	-1.428	0.188223978	35.3	51.53	3.518	1.546
SPAC3C7.08C	elf1	0.8967	-1.422	0.04735283	23.12	34.44	11.69	6.332
SPBC29A3.18	cyt1	0.7999	-1.414	0.096964303	10.7	17	6.419	2.974
SPAC27F1.06C	SPAC27F1.06c	0.7358	-1.413	0.133240217	37.83	55.07	4.103	2.722
SPBC1711.04	SPBC1711.04	0.8764	-1.406	0.057297631	37.92	55.17	11.17	2.834
SPBC577.03C	SPBC577.03c	0.7669	-1.404	0.115261262	35.12	51.25	5.249	2.492
SPCC663.13C	naa50	0.8732	-1.404	0.058886273	41.33	59.95	11.11	1.96
SPBC530.13	lsc1	0.8447	-1.401	0.073297506	36.33	52.94	8.648	2.661
SPAP27G11.14C	SPAP27G11.14c	0.6612	-1.4	0.179667155	16.76	25.48	3.694	1.422
SPBC1271.10C	SPBC1271.10c	0.4646	-1.389	0.332920795	40.87	59.29	1.358	1.47
SPAC4H3.01	SPAC4H3.01	0.8727	-1.389	0.059135024	37.92	55.15	10.05	4.019
SPCC1682.11C	SPCC1682.11c	0.8307	-1.387	0.08055579	34.19	49.92	7.682	2.718
SPAC227.05	SPAC227.05	0.4394	-1.38	0.357139947	39.55	57.43	1.099	1.432
SPBC29A10.09C	SPBC29A10.09c	0.8787	-1.376	0.056159374	39.22	56.97	11.14	2.841
SPCC594.07C	bqt3	0.8687	-1.372	0.061130178	35.9	52.3	9.991	3.2
SPBC418.02	SPBC418.02	0.8535	-1.355	0.068796475	37.17	54.06	8.14	3.892
SPCC4G3.09C	gyp3	0.879	-1.355	0.056011125	35.32	51.46	10.84	3.166
SPAC664.03	SPAC664.03	0.6624	-1.35	0.178879676	25.14	37.17	2.269	2.431
SPAC2F3.08	sut1	0.8474	-1.347	0.07191154	37.56	54.59	7.98	3.424
SPAC18G6.12C	SPAC18G6.12c	0.8355	-1.342	0.078053546	31.92	46.67	7.97	2.022
SPAC630.13C	tsc2	0.6816	-1.336	0.166470418	42.01	60.82	2.85	2.34
SPBC30D10.05C	SPBC30D10.05c	0.8861	-1.336	0.052517263	39.32	57.04	11.66	2.527
SPAC2F3.02	SPAC2F3.02	0.8519	-1.33	0.069611382	31.43	45.96	8.555	2.778
SPAC18B11.07C	rhp6	0.7234	-1.326	0.140621496	33.46	48.81	4.073	2.055
SPAC57A7.04C	pabp	0.7689	-1.319	0.114130139	35.34	51.44	4.982	2.353
SPCC188.08C	ubp5	0.6221	-1.308	0.206139799	42.57	61.57	1.161	2.297
SPBP23A10.05	ssr4	0.9024	-1.305	0.044600913	42.63	61.64	13.03	3.633
SPAC869.11	cat1	0.7731	-1.293	0.111764327	23.55	34.85	4.977	2.355
SPAC25B8.19C	SPAC25B8.19c	0.8485	-1.293	0.071348153	10.06	15.93	6.73	4.362
SPAC9G1.11C	spn4	0.8188	-1.292	0.086822166	36.63	53.21	6.617	2.47
SPCC1906.02C	SPCC1906.02c	0.7977	-1.291	0.098160408	34.58	50.33	5.846	2.295
SPCC584.12	mug42	0.8958	-1.291	0.047788942	39.7	57.51	12.1	3.314
SPAC17A2.13C	rad25	0.7714	-1.272	0.112720365	33.19	48.35	5.395	1.339
SPAC2F3.11	SPAC2F3.11	0.8545	-1.272	0.068287933	38.18	55.36	8.182	2.986
SPBC106.20	exo70	0.8557	-1.271	0.067678468	40	57.92	8.795	1.674
SPCC297.03	ssp1	0.8123	-1.264	0.090283547	34.62	50.35	3.741	4.289
SPAC24C9.14	otu1	0.8176	-1.263	0.087459117	37	53.69	6.274	2.637
SPAC824.07	SPAC824.07	0.8834	-1.251	0.053842605	37.93	54.98	10.42	2.99
SPBC725.06C	ppk31	0.8866	-1.249	0.052272273	38.27	55.45	11	2.233
SPBC3B9.11C	ctf1	0.9151	-1.243	0.038531445	32.25	47	12.41	6.79
SPCC132.03	SPCC132.03	0.8713	-1.225	0.059832286	37.71	54.64	9.53	1.763
SPBC1773.05C	tms1	0.8594	-1.205	0.065804651	37.92	54.9	7.742	3.357
SPAC1851.02	slc1	0.9189	-1.204	0.036731748	39.84	57.58	15.23	0.8198
SPBC119.04	mei3	0.8453	-1.199	0.072989131	31.63	46.07	7.261	2.611
SPAC3F10.02C	trk1	0.893	-1.191	0.049148541	27.6	40.39	8.476	5.961
SPBC29B5.01	atf1	0.8146	-1.19	0.089055594	25.2	37.02	4.207	3.807
SPBC13G1.02	SPBC13G1.02	0.8932	-1.182	0.049051286	35.05	50.84	10.7	3.204
SPCC1450.08C	wtf16	0.9048	-1.179	0.043447408	33.69	48.92	10.64	5.581
SPAC1F5.03C	SPAC1F5.03c	0.7766	-1.176	0.109802614	37.51	54.29	4.97	1.593
SPAC57A10.12C	ura3	0.9046	-1.176	0.043543417	17.34	25.98	9.917	6.556
SPBC1198.08	SPBC1198.08	0.8785	-1.171	0.056258234	36.42	52.75	9.443	2.463
SPBC660.12C	SPBC660.12c	0.8803	-1.155	0.055369298	38.15	55.15	9.073	3.268
SPCC1223.10C	eaf1	0.8959	-1.155	0.047740463	35.6	51.57	10.95	2.667
SPCC1393.10	ctr4	0.9213	-1.154	0.035598929	22.87	33.7	12.71	6.488
SPAC1B3.04C	SPAC1B3.04c	0.8948	-1.153	0.048274025	30.84	44.89	10.77	2.725
SPCPJ732.03	meu15	0.8217	-1.149	0.085286713	11.17	17.28	5.512	2.866
SPBC36B7.06C	mug20	0.9368	-1.149	0.028353118	27.53	40.24	14.3	9.451
SPAC821.05	SPAC821.05	0.7227	-1.148	0.141041945	22.89	33.72	3.048	2.193
SPAC1039.04	SPAC1039.04	0.8781	-1.148	0.056456023	42.93	61.85	9.589	0.9612
SPAC222.04C	ies6	0.8821	-1.147	0.054482178	14.63	22.13	7.792	4.903
SPBC24C6.08C	bhd1	0.8454	-1.146	0.072937757	37.63	54.4	6.512	3.125
SPAC3C7.14C	obr1	0.8825	-1.146	0.054285286	39.55	57.1	9.5	2.644
SPBC1921.05	ape2	0.6998	-1.135	0.155026062	34.47	49.96	3.097	1.717
SPAC8E11.06	SPAC8E11.06	0.8847	-1.134	0.053203973	37.57	54.31	9.806	2.02
SPAC56F8.09	rrp8	0.9061	-1.126	0.04282387	35.32	51.13	10.83	4.778
SPAC1556.03	azr1	0.8349	-1.122	0.078365539	36.95	53.42	5.706	3.144
SPAC24B11.12C	SPAC24B11.12c	0.6276	-1.11	0.202317065	34.83	50.43	2.214	1.491
SPAC227.04	SPAC227.04	0.9368	-1.107	0.028353118	29.01	42.26	12.44	10
SPAC30D11.12	rpl3802	0.8188	-1.087	0.086822166	34.8	50.36	5.335	2.418

SPBC1703.04	mlh1	0.8882	-1.084	0.051489231	35.86	51.84	9.637	2.1
SPAC22H12.04C	rps102	0.8436	-1.083	0.073863429	38.87	56.06	3.978	4.38
SPAC2C4.05	SPAC2C4.05	0.8578	-1.079	0.066613958	38.32	55.28	6.951	2.824
SPAC1565.01	SPAC1565.01	0.8242	-1.078	0.08396739	37.32	53.88	5.477	2.449
SPAC13C5.06C	mug121	0.9124	-1.065	0.039814723	34.13	49.39	9.908	6.013
SPAC18B11.03C	SPAC18B11.03c	0.9422	-1.058	0.0258569	28.75	41.82	14.1	9.731
SPBC2D10.14C	myo51	0.8064	-1.051	0.093449481	39	56.2	4.806	2.201
SPAC20G8.07C	erg2	0.8995	-1.051	0.045998832	40.29	58.01	10.15	2.952
SPAC16.01	rho2	0.9408	-1.051	0.026502691	28.67	41.7	13.35	9.694
SPBC56F2.09C	arg5	0.8978	-1.049	0.046820399	18.52	27.45	9.088	4.308
SPBC1703.11	SPBC1703.11	0.8749	-1.045	0.058041583	35.92	51.86	7.639	3.16
SPAC1039.08	SPAC1039.08	0.8861	-1.035	0.052517263	13.41	20.27	7.305	4.555
SPAC6F6.04C	SPAC6F6.04c	0.8593	-1.022	0.065855188	38.83	55.92	6.782	2.514
SPBC32F12.03C	gpx1	0.7131	-1.018	0.146849564	38.38	55.28	2.896	1.634
SPAC20H4.10	ufd2	0.8075	-1.015	0.092857469	39.8	57.26	4.883	1.829
SPBC16H5.12C	SPBC16H5.12c	0.5754	-1.004	0.240030142	38.32	55.17	1.511	1.306
SPAC11H11.04	mam2	0.8866	-0.9979	0.052272273	34.01	49.12	8.845	1.544
SPBC2D10.04	SPBC2D10.04	0.8297	-0.9836	0.08107891	37	53.29	5.278	2.142
SPBC27.04	uds1	0.8891	-0.9813	0.05104939	38.08	54.8	8.639	2.351
SPBC1861.02	abp2	0.9018	-0.9809	0.044889769	41.96	60.25	9.126	3.83
SPCC622.19	jmj4	0.7764	-0.9787	0.109914473	32.23	46.59	3.178	2.392
SPCC830.06	SPCC830.06	0.9185	-0.975	0.036920839	20.89	30.68	8.274	6.965
SPBC21C3.11	ubx4	0.8679	-0.9664	0.061530312	37.43	53.87	6.334	3.218
SPAC23C4.11	atp18	0.8473	-0.9645	0.071962794	41.49	59.57	6.243	1.389
SPAC19D5.02C	SPAC19D5.02c	0.8437	-0.9563	0.073811951	33.09	47.77	5.799	1.935
SPAC9E9.09C	SPAC9E9.09c	0.9201	-0.9555	0.036164969	17.6	26.03	9.735	5.93
SPBC106.19	SPBC106.19	0.9279	-0.9542	0.032498825	34.15	49.26	12.98	3.498
SPAC6G10.03C	SPAC6G10.03c	0.912	-0.9521	0.040005162	39.35	56.56	10.93	1.775
SPAC1399.02	SPAC1399.02	0.8004	-0.9516	0.09669292	42.62	61.14	3.909	2.271
SPBC1815.01	eno101	0.5768	-0.9506	0.238974748	40.11	57.62	1.767	0.9974
SPBC106.12C	SPBC106.12c	0.9231	-0.947	0.034751249	30.62	44.29	10.79	5.332
SPBC16E9.07	mug100	0.6604	-0.9468	0.180192935	30.02	43.45	2.2	1.315
SPAC12G12.16C	SPAC12G12.16c	0.9048	-0.9445	0.043447408	37.92	54.53	9.241	3.539
SPAC823.13C	SPAC823.13c	0.9057	-0.9354	0.043015632	38.45	55.27	9.661	2.731
SPBP23A10.02	pkr1	0.8185	-0.9322	0.086981316	39.64	56.93	3.69	2.864
SPBC21C3.08C	car2	0.391	-0.9231	0.407823243	0	1.295	1.295	0
SPAC4A8.06C	SPAC4A8.06c	0.8688	-0.9218	0.061080188	33.35	48.09	7.022	1.332
SPAC6F12.06	SPAC6F12.06	0.8613	-0.9207	0.064845553	32.51	46.91	6.17	2.343
SPAC9G1.06C	cyk3	0.7512	-0.9167	0.124244421	39.87	57.23	2.784	1.92
SPCC1795.12C	SPCC1795.12c	0.8313	-0.9127	0.080242219	37.11	53.35	4.581	2.461
SPAPB8E5.02C	rpn502	0.8078	-0.9104	0.092696151	33.97	48.95	3.51	2.563
SPCC663.11	saf1	0.8134	-0.909	0.089695832	27.81	40.3	3.446	2.746
SPBC19G7.02	SPBC19G7.02	0.8464	-0.908	0.072424345	30.63	44.26	5.231	2.448
SPBC29A10.10C	SPBC29A10.10c	0.9085	-0.9065	0.041675068	40.16	57.63	9.206	3.565
SPBC18E5.11C	edc3	0.9013	-0.9053	0.045130629	38.84	55.76	8.747	2.906
SPAC1565.03	SPAC1565.03	0.92	-0.9017	0.036212173	40.06	57.47	10.58	3.9
SPAC23A1.06C	cmk2	0.8959	-0.9005	0.047740463	37.22	53.49	7.572	3.739
SPCC1259.01C	rps1802	0.9021	-0.8886	0.044745317	10.57	16.08	7.58	4.311
SPAC222.05C	mss1	0.7855	-0.8885	0.104853811	34.91	50.23	3.838	1.407
SPCC1795.01C	mad3	0.9113	-0.8885	0.04033863	37.65	54.08	10.03	2.004
SPBC23E6.03C	nta1	0.8563	-0.8601	0.067374056	31.25	45.05	5.549	2.133
SPBC215.07C	SPBC215.07c	0.9312	-0.8381	0.030957033	37.01	53.11	12.26	2.248
SPAC26H5.02C	SPAC26H5.02c	0.8985	-0.8175	0.046481919	36.38	52.19	7.906	2.06
SPAC2C4.17C	SPAC2C4.17c	0.9275	-0.8154	0.032686082	39.93	57.18	11.22	2.405
SPCC736.04C	gma12	0.9203	-0.8145	0.036070578	40.74	58.3	10.48	0.6815
SPAC29E6.09	SPAC29E6.09	0.8464	-0.8024	0.072424345	35.8	51.35	4.573	2.221
SPCC794.11C	SPCC794.11c	0.9402	-0.8021	0.026779753	36.8	52.75	13.08	3.73
SPAC1952.10C	SPAC1952.10c	0.9099	-0.8019	0.041006335	34.81	49.96	8.706	2.37
SPAC3H8.10	spo20	0.8764	-0.8014	0.057297631	38.1	54.59	6.092	2.195
SPAC8F11.08C	SPAC8F11.08c	0.8776	-0.8004	0.056703385	33.67	48.37	5.248	3.282
SPAC824.08	gda1	0.8799	-0.7959	0.055566682	34.38	49.35	6.507	1.644
SPBC30B4.03C	adn1	0.7964	-0.794	0.098868749	35.02	50.25	3.316	1.736
SPAP11E10.02C	mam3	0.9283	-0.7901	0.03231165	34.13	49	10.72	3.098
SPAC13C5.04	SPAC13C5.04	0.803	-0.7887	0.095284455	27.41	39.57	2.99	2.147
SPBC16E9.02C	SPBC16E9.02c	0.8404	-0.7777	0.075513956	35.96	51.54	4.771	1.187
SPAC4G9.10	arg3	0.9116	-0.7776	0.040195683	36.67	52.54	8.584	2.394
SPCC63.08C	atg1	0.8179	-0.7764	0.087299792	29.34	42.26	3.666	1.87
SPBC20F10.06	mad2	0.9225	-0.7748	0.035033625	40.73	58.24	9.794	2.649
SPAC25H1.05	meu29	0.8639	-0.7729	0.063536526	8.292	12.72	4.364	3

SPBC26H8.12	SPBC26H8.12	0.9087	-0.7698	0.041579472	6.911	10.78	6.032	4.782
SPCC1322.09	SPCC1322.09	0.953	-0.7697	0.020907099	25.89	37.41	12	9.152
SPAC1F7.09C	SPAC1F7.09c	0.895	-0.766	0.048176965	37.33	53.45	6.133	3.422
SPBC15D4.13C	SPBC15D4.13c	0.8879	-0.7648	0.051635944	36.26	51.95	5.945	2.973
SPAC644.09	SPAC644.09	0.8812	-0.7619	0.054925512	36.1	51.72	5.7	2.655
SPAC630.06C	SPAC630.06c	0.8223	-0.7555	0.08496971	32.97	47.32	3.701	1.814
SPAC1F12.04C	SPAC1F12.04c	0.8265	-0.7533	0.082757142	32.71	46.95	3.931	1.664
SPAC2C4.14C	ppk11	0.8967	-0.7527	0.04735283	38.41	54.96	6.955	2.296
SPBC365.08C	SPBC365.08c	0.9227	-0.7455	0.034939479	34.78	49.84	9.381	2.707
SPBC146.13C	myo1	0.94	-0.7441	0.026872146	18.96	27.65	9.867	6.351
SPAC11E3.12	SPAC11E3.12	0.9254	-0.7432	0.033670505	35.09	50.28	9.439	3.312
SPAC4G9.12	SPAC4G9.12	0.9107	-0.7406	0.040624663	37.62	53.83	7.854	2.75
SPAC607.08C	SPAC607.08c	0.9153	-0.7406	0.038436538	34.18	49	8.235	2.973
SPBC359.01	SPBC359.01	0.9403	-0.7374	0.026733564	34.79	49.85	11.61	4.254
SPAC11E3.14	SPAC11E3.14	0.8833	-0.7295	0.05389177	32.77	47	5.941	2.007
SPAC4G8.03C	SPAC4G8.03c	0.8528	-0.7283	0.069152808	39.28	56.14	4.539	1.829
SPBC1685.09	rps29	0.7174	-0.7153	0.144238628	22.14	32.08	1.878	1.328
SPAC806.08C	mod21	0.9134	-0.7076	0.039338993	33.55	48.07	7.78	2.639
SPBC947.15C	SPBC947.15c	0.8877	-0.7014	0.05173378	31.21	44.78	5.894	2.087
SPBC30B4.01C	wsc1	0.962	-0.6987	0.016824928	28.4	40.83	14.21	9.781
SPAC1610.03C	crp79	0.8906	-0.6954	0.050317309	37.09	53.02	5.561	2.744
SPBC1604.09C	SPBC1604.09c	0.9062	-0.6947	0.042775942	36.78	52.58	6.475	3.215
SPAC22H12.03	SPAC22H12.03	0.8911	-0.6846	0.050073556	34.21	48.96	5.84	2.258
SPBC428.08C	clr4	0.8922	-0.6827	0.049537781	28.94	41.56	5.803	2.39
SPAPJ760.02C	abp1	0.7776	-0.6804	0.109243748	39.93	56.99	1.871	1.864
SPBC3D6.05	ptp4	0.9341	-0.6773	0.029606628	25.69	37	7.955	5.429
SPBC6B1.06C	ubp14	0.8901	-0.6756	0.050561199	30.13	43.23	5.923	1.809
SPCC330.11	btb1	0.9604	-0.6741	0.017547849	25.99	37.42	13.34	8.882
SPAC4G9.02	rnh201	0.9188	-0.6709	0.036779013	38.63	55.14	8.078	2.205
SPAC17G8.08C	SPAC17G8.08c	0.8951	-0.6664	0.048128443	38.68	55.21	5.667	2.618
SPCC757.02C	SPCC757.02c	0.9261	-0.6447	0.033342116	34.97	49.97	8.674	1.959
SPBC29A10.01	ccr1	0.9176	-0.6433	0.037346595	38.89	55.47	6.913	3.3
SPACUNK4.19	mug153	0.8821	-0.6408	0.054482178	35.14	50.21	4.999	2.021
SPAC4A8.07C	SPAC4A8.07c	0.8493	-0.6364	0.070938876	36.61	52.26	4.23	0.7299
SPAC6F12.09	rdp1	0.8413	-0.6335	0.075049111	33.26	47.56	3.575	1.594
SPCC1281.08	wtf11	0.947	-0.6283	0.023650021	32.53	46.52	9.921	5.643
SPAC23G3.02C	sib1	0.9346	-0.6256	0.029374223	39.14	55.79	9.549	2.049
SPAC22A12.16	SPAC22A12.16	0.9067	-0.6255	0.042536384	35.99	51.37	5.47	3.307
SPAC11D3.17	SPAC11D3.17	0.9407	-0.6118	0.026548856	39.32	56.03	9.98	3.052
SPAC29B12.08	SPAC29B12.08	0.9556	-0.6031	0.019723859	28.24	40.47	11.29	6.533
SPAC1486.01	SPAC1486.01	0.8855	-0.5984	0.052811434	30.35	43.42	4.645	2.164
SPAC1687.19C	SPAC1687.19c	0.9146	-0.5948	0.038768803	37.96	54.1	6.616	2.265
SPBC1604.16C	SPBC1604.16c	0.9562	-0.594	0.019451261	40	56.96	13.63	2.59
SPBC651.03C	gyp10	0.9626	-0.5867	0.016554143	31.37	44.84	12.02	8.387
SPAC4F8.11	SPAC4F8.11	0.881	-0.5863	0.055024092	31.94	45.64	4.668	1.6
SPAC17C9.08	pnu1	0.919	-0.5843	0.036684489	37.17	52.97	6.608	2.757
SPAC13G6.12C	chs1	0.9076	-0.5756	0.042105513	34.97	49.88	6.104	1.627
SPCC306.02C	SPCC306.02c	0.9318	-0.5754	0.030677294	28.23	40.41	7.303	3.766
SPAC17H9.06C	SPAC17H9.06c	0.948	-0.5715	0.023191663	40.07	57.03	10.27	3.889
SPAC139.06	hat1	0.8996	-0.5613	0.045950553	36.96	52.64	4.707	2.618
SPBC23E6.09	ssn6	0.851	-0.5609	0.07007044	36.61	52.15	3.812	0.4441
SPCC594.06C	SPCC594.06c	0.912	-0.5596	0.040005162	32.94	47.01	6.106	1.951
SPAC27E2.11C	SPAC27E2.11c	0.5487	-0.5593	0.26066504	0.6193	1.654	0.8782	0.6193
SPBC1773.02C	SPBC1773.02c	0.9074	-0.5565	0.042201225	38.15	54.31	5.067	2.807
SPAC926.07C	dlc2	0.9367	-0.5469	0.02839948	37.5	53.38	8.053	3.093
SPACUNK4.09	SPACUNK4.09	0.9472	-0.5449	0.023558311	40.12	57.05	10.06	2.863
SPAC167.01	ire1	0.8827	-0.539	0.054186873	36.46	51.91	4.421	1.36
SPAC9G1.12	cpd1	0.9013	-0.5368	0.045130629	22.08	31.74	3.841	3.118
SPAC14C4.12C	laf1	0.9309	-0.536	0.03109697	31.01	44.26	6.829	3.331
SPAC26F1.09	gyp51	0.8428	-0.5307	0.074275473	32.77	46.73	3.07	1.283
SPAC31G5.10	eta2	0.9261	-0.5284	0.033342116	36	51.26	6.202	3.171
SPCC1442.04C	SPCC1442.04c	0.9673	-0.5279	0.014438812	23.42	33.61	12.32	8.693
SPAC29B12.12	SPAC29B12.12	0.9161	-0.5238	0.038057117	38.33	54.51	5.59	2.55
SPBC713.07C	SPBC713.07c	0.9391	-0.5225	0.027288159	32.71	46.63	8.347	2.41
SPBPB7E8.02	SPBPB7E8.02	0.788	-0.5191	0.103473783	37.96	53.99	1.655	1.417
SPAC23C11.15	pst2	0.923	-0.5148	0.034798299	35.74	50.86	3.297	4.459
SPAC25G10.06	rps2801	0.8185	-0.5119	0.086981316	43.19	61.32	1.368	1.866
SPAC23D3.13C	SPAC23D3.13c	0.9316	-0.5075	0.03077052	34.56	49.21	7.146	2.235
SPAC9.08C	SPAC9.08c	0.9338	-0.5018	0.02974613	32.48	46.27	6.785	3.115

SPBC2D10.11C	nap2	0.898	-0.5008	0.046723663	39.25	55.77	4.096	2.337
SPAC1556.01C	rad50	0.9476	-0.4991	0.023374948	8.517	12.65	7.531	4.917
SPAC630.07C	SPAC630.07c	0.9448	-0.4967	0.024660115	34.14	48.61	8.834	2.334
SPBC713.11C	pmp3	0.9308	-0.491	0.031143625	34.51	49.11	5.997	3.316
SPAC8C9.09C	mug129	0.9706	-0.4801	0.012959713	17.95	25.85	13.65	7.784
SPAC12B10.07	acp1	0.9067	-0.4753	0.042536384	37.57	53.38	4.424	2.242
SPAC1399.05C	SPAC1399.05c	0.9332	-0.4736	0.03002527	31.84	45.34	6.478	2.742
SPAC16C9.06C	upf1	0.9397	-0.4706	0.027010773	32.14	45.76	4.369	5.032
SPBC17A3.10	pas4	0.9444	-0.4697	0.024844022	20.82	29.87	6.91	4.176
SPBC21C3.20C	git1	0.8855	-0.4686	0.052811434	23.47	33.59	3.801	1.469
SPBC27B12.09C	SPBC27B12.09c	0.907	-0.4621	0.042392713	36.77	52.24	4.077	2.429
SPBC8D2.19	mde3	0.9261	-0.4562	0.033342116	29.45	41.96	5.526	2.528
SPAC16E8.05C	mde1	0.9695	-0.4537	0.013452187	22.69	32.47	12.07	7.42
SPBC83.01	ucp8	0.9545	-0.4533	0.020224067	39.02	55.38	9.723	2.776
SPBC2F12.12C	SPBC2F12.12c	0.9508	-0.4532	0.021910827	15.11	21.84	7.32	4.733
SPCC1620.12C	SPCC1620.12c	0.8928	-0.451	0.049245818	35.54	50.5	3.944	1.454
SPBC1685.07C	avt5	0.5414	-0.436	0.266481749	0.2939	1.024	0.8279	0.2939
SPAC1F5.07C	hem14	0.9574	-0.4328	0.018906577	19.99	28.66	9.195	4.047
SPCC1620.13	SPCC1620.13	0.9752	-0.4318	0.010906307	26.84	38.27	13.62	9.131
SPCC1827.07C	SPCC1827.07c	0.9638	-0.4306	0.016013078	41.51	58.84	11.69	3.067
SPBC8D2.04	hht2	0.9766	-0.4243	0.01028328	29.12	41.45	14.1	9.585
SPAC1420.03	rpn501	0.9513	-0.4235	0.021682503	38.61	54.76	8.361	2.669
SPAC17A5.18C	rec25	0.9156	-0.4214	0.038294216	34.09	48.43	3.75	2.717
SPCC569.03	SPCC569.03	0.9714	-0.4136	0.012601901	31	44.08	12.05	6.946
SPBC1778.03C	SPBC1778.03c	0.9416	-0.4111	0.02613355	30.84	43.85	6.87	1.939
SPBC1683.12	SPBC1683.12	0.9629	-0.4093	0.016418813	32.03	45.52	10.27	4.014
SPAP8A3.03	SPAP8A3.03	0.9343	-0.4092	0.029513651	32.29	45.89	6.106	1.655
SPBC2D10.18	abc1	0.6474	-0.3932	0.188827305	1.043	2.015	0.7957	0.6212
SPAC9G1.05	SPAC9G1.05	0.9416	-0.3864	0.02613355	34.16	48.47	6.255	2.223
SPAC5H10.01	SPAC5H10.01	0.8259	-0.3839	0.083072534	41.15	58.28	1.317	1.371
SPAPB1A10.05	SPAPB1A10.05	0.855	-0.3827	0.068033885	42.2	59.75	1.412	1.712
SPAC5D6.08C	mes1	0.9396	-0.3763	0.027056992	35.69	50.6	5.36	2.809
SPAC17G6.13	slt1	0.9623	-0.3761	0.016689514	41.38	58.59	9.574	3.131
SPBPB2B2.01	SPBPB2B2.01	0.9771	-0.3722	0.010060987	20.74	29.63	13.75	7.613
SPCC1795.10C	SPCC1795.10c	0.9082	-0.3675	0.041818502	33.73	47.84	3.695	1.481
SPCC757.09C	rnc1	0.9635	-0.3569	0.016148281	28.19	40.05	8.086	4.751
SPAC869.05C	SPAC869.05c	0.9497	-0.356	0.022413562	35.66	50.54	6.581	2.565
SPCC18.01C	adg3	0.9054	-0.353	0.04315951	40.83	57.78	3.392	1.457
SPBP35G2.14	SPBP35G2.14	0.8896	-0.3526	0.050805226	35.81	50.75	3.239	0.4408
SPAC10F6.06	vip1	0.9456	-0.3519	0.024292536	35.81	50.74	6.073	2.252
SPBC17G9.02C	SPBC17G9.02c	0.9405	-0.3429	0.0266412	37.69	53.36	5.394	2.031
SPAC3C7.06C	pit1	0.9128	-0.3388	0.039624369	33.02	46.8	3.677	1.289
SPAC12B10.15C	SPAC12B10.15c	0.9476	-0.3387	0.023374948	35.62	50.45	5.833	2.608
SPAC6F6.06C	rax2	0.9338	-0.3324	0.02974613	30.6	43.4	4.662	1.824
SPAC1142.01	SPAC1142.01	0.9385	-0.3311	0.027565723	40.34	57.06	4.534	2.529
SPBC3E7.10	fma1	0.9778	-0.3276	0.009749967	26.58	37.75	11.97	7.374
SPAC1527.02	sft2	0.9632	-0.3231	0.016283526	35.86	50.76	7.869	3.602
SPCC576.01C	SPCC576.01c	0.9602	-0.3218	0.017638298	31.77	45.03	7.865	2.287
SPAC4G9.05	mpf1	0.9574	-0.3183	0.018906577	35.9	50.82	6.916	2.766
SPAC27D7.05C	apc14	0.9515	-0.3179	0.021591207	38.96	55.11	6.058	2.433
SPBC543.09	yta12	0.9295	-0.3145	0.031750606	34.67	49.08	4.128	1.646
SPACUNK4.11C	SPACUNK4.11c	0.9408	-0.3132	0.026502691	35.84	50.73	4.947	1.872
SPBC691.04	SPBC691.04	0.9496	-0.3105	0.022459294	36.7	51.93	5.88	1.982
SPBC83.03C	tas3	0.9498	-0.3096	0.022367835	25.87	36.73	5.829	2.073
SPBC3E7.15C	lac1	0.8805	-0.2985	0.05527064	1.185	2.082	2.082	1.185
SPACUNK4.08	SPACUNK4.08	0.9649	-0.2974	0.015517694	37.89	53.58	7.413	3.708
SPBPB2B2.05	SPBPB2B2.05	0.9543	-0.2771	0.020315076	36.76	51.97	5.174	2.799
SPAC12B10.13	SPAC12B10.13	0.9314	-0.2763	0.030863766	33.69	47.66	3.926	1.117
SPCC24B10.08C	ada2	0.9664	-0.2758	0.014843079	29.42	41.67	5.981	4.602
SPBC25B2.03	SPBC25B2.03	0.9729	-0.2758	0.011931797	39.13	55.29	9.615	3.491
SPBC29A3.08	pof4	0.9718	-0.2747	0.012423105	36.24	51.23	8.005	4.776
SPAC27D7.10C	EMPTY	0.9589	-0.2746	0.018226681	36.56	51.68	5.864	2.907
SPBC691.05C	SPBC691.05c	0.9584	-0.2739	0.018453195	36.95	52.23	5.949	2.652
SPCC1442.03	SPCC1442.03	0.9666	-0.2711	0.014753209	34.55	48.85	7.511	2.995
SPAC18G6.15	mal3	0.9796	-0.2626	0.008951224	30.96	43.81	11.04	5.871
SPBP16F5.08C	SPBP16F5.08c	0.9679	-0.2592	0.01416951	35.44	50.09	7.338	3.21
SPBC1604.03C	SPBC1604.03c	0.9632	-0.2553	0.016283526	34.43	48.67	6.233	2.844
SPBC19C7.02	ubr1	0.9717	-0.251	0.012467797	25.62	36.3	7.636	4.015
SPCC63.02C	aah3	0.8324	-0.2406	0.079667928	0.6366	1.231	1.231	0.6366

SPCC132.02	hst2	0.9538	-0.2346	0.020542682	37.01	52.26	4.17	2.491
SPCC1919.05	SPCC1919.05	0.9839	-0.2312	0.007049039	20.86	29.6	11.02	7.649
SPAC23G3.10C	ssr3	0.9673	-0.2286	0.014438812	34.7	49.01	3.436	4.67
SPBC21C3.12C	SPBC21C3.12c	0.9807	-0.2231	0.008463825	20.91	29.65	9.701	5.875
SPAC24B11.06C	sty1	0.6602	-0.2209	0.18032448	0.3064	0.7398	0.5389	0.3064
SPBC21D10.09C	SPBC21D10.09c	0.9446	-0.2197	0.024752059	38.05	53.69	3.104	2.073
SPBC8D2.02C	SPBC8D2.02c	0.9558	-0.2161	0.019632974	34.24	48.35	4.172	2.246
SPAC23C11.02C	rps23	0.983	-0.2028	0.007446482	21.84	30.94	8.805	6.655
SPAC6G9.15C	SPAC6G9.15c	0.9818	-0.201	0.007976972	37.29	52.6	11.16	2.041
SPAC1039.06	SPAC1039.06	0.9706	-0.1902	0.012959713	35.64	50.28	5.816	2.626
SPAC3A11.03	SPAC3A11.03	0.9503	-0.1863	0.022139271	34.07	48.06	3.63	1.097
SPBC21C3.18	spo4	0.9783	-0.1856	0.009527946	31.88	45	8.044	2.948
SPAC19G12.09	SPAC19G12.09	0.9627	-0.1827	0.016509028	32.84	46.33	4.895	1.036
SPAC1F5.08C	yam8	0.9796	-0.1779	0.008951224	27.85	39.32	7.935	3.413
SPCC4G3.10C	rhp42	0.9695	-0.1777	0.013452187	38.24	53.91	5.304	2.282
SPBC405.02C	SPBC405.02c	0.9672	-0.1767	0.014483712	37.85	53.36	5.174	1.674
SPBPB10D8.06C	SPBPB10D8.06c	0.9659	-0.1733	0.015067834	38.72	54.57	3.919	2.711
SPBC12C2.08	dnm1	0.9819	-0.1687	0.00793274	33.26	46.9	8.939	2.904
SPBC1105.14	rsv2	0.9656	-0.1625	0.015202743	36.19	51	4.389	1.731
SPAC1782.04	cox24	0.9881	-0.1621	0.005199101	22.81	32.23	10.74	7.141
SPAC30C2.02	mmd1	0.9681	-0.1566	0.01407978	33.36	47.03	4.175	2.266
SPAC4H3.02C	swc3	0.9906	-0.1554	0.004101676	30.36	42.82	10.49	10.15
SPAC869.02C	SPAC869.02c	0.9434	-0.1523	0.025304128	40.48	57.02	1.854	1.573
SPAC17A2.01	bsu1	0.9815	-0.1467	0.008109696	34.46	48.56	7.159	3.193
SPBC1E8.03C	SPBC1E8.03c	0.9713	-0.1452	0.012646611	38.14	53.72	4.582	2.02
SPAC4G8.04	SPAC4G8.04	0.9908	-0.1452	0.004014002	24.69	34.85	12.15	8.51
SPAC9E9.08	rad26	0.9772	-0.1435	0.010016542	36.62	51.59	5.872	2.237
SPAC4H3.03C	SPAC4H3.03c	0.9793	-0.1407	0.009084245	34.59	48.73	6.186	2.671
SPBC32F12.11	tdh1	0.9641	-0.1345	0.015877917	23.46	33.11	3.145	1.773
SPAC20G4.04C	hus1	0.9806	-0.1339	0.008508111	28.76	40.55	6.464	2.419
SPAC30D11.07	nth1	0.9812	-0.1333	0.00824246	34.49	48.58	5.811	3.505
SPCC1183.06	ung1	0.9796	-0.126	0.008951224	37.53	52.84	6.152	1.416
SPAC167.04	pam17	0.9876	-0.126	0.005418919	34.17	48.13	8.459	4.899
SPBC1348.01	SPBC1348.01	0.9861	-0.1235	0.006079041	36.81	51.83	7.751	3.959
SPAC31F12.01	zds1	0.9887	-0.1216	0.004935466	9.027	12.84	8.459	5.91
SPAC4F8.01	did4	0.9786	-0.1195	0.009394789	38.3	53.91	5.034	2.277
SPAC821.09	eng1	0.9884	-0.1101	0.005067263	41.01	57.69	9.261	2.555
SPBC27B12.03C	erg32	0.9757	-0.1066	0.010683695	40.18	56.52	4.098	1.565
SPBC2G2.08	ade9	0.9547	-0.1062	0.020133077	35.11	49.41	1.694	1.328
SPBC1604.11	atp17	0.9627	-0.1049	0.016509028	39.99	56.26	2.162	1.498
SPAC16A10.07C	taz1	0.9798	-0.1027	0.008862565	37.99	53.45	4.731	1.833
SPBC29B5.04C	SPBC29B5.04c	0.9853	-0.09984	0.006431517	36.05	50.73	6	2.912
SPBC36.04	cys11	0.9782	-0.09732	0.009572342	35.61	50.11	3.989	1.838
SPAC1A6.04C	plb1	0.9923	-0.09547	0.003357009	17.67	24.93	10.01	6.205
SPBC216.06C	swi1	0.9847	-0.09282	0.006696062	36.31	51.07	4.49	3.368
SPAC4G9.16C	rpl901	0.7071	-0.08901	0.150519163	0.1805	0.3782	0.2041	0.1805
SPAC8C9.16C	mug63	0.995	-0.08684	0.002176919	27.57	38.8	13.37	9.204
SPBC1711.02	matmc_1	0.9854	-0.07982	0.006387442	36.5	51.33	4.814	2.379
SPCP31B10.06	mug190	0.9925	-0.07422	0.003269485	39.34	55.31	9.266	3.407
SPAC5D6.04	SPAC5D6.04	0.9906	-0.06734	0.004101676	36.27	50.99	6.582	2.734
SPBC16A3.14	SPBC16A3.14	0.9935	-0.06626	0.002832129	38.98	54.79	10.2	2.325
SPCC16A11.04	snx12	0.9897	-0.06377	0.00449643	33.27	46.78	5.895	1.994
SPAC23A1.09	SPAC23A1.09	0.9894	-0.06274	0.004628094	34.99	49.18	5.413	2.307
SPBC25D12.06	SPBC25D12.06	0.9527	-0.06082	0.021043835	0.8127	1.226	1.08	0.6477
SPCC24B10.11C	mft1	0.9817	-0.05834	0.008021209	29.08	40.88	2.442	1.672
SPAPB2C8.01	SPAPB2C8.01	0.9879	-0.05771	0.005287015	35.35	49.68	4.58	1.47
SPCC70.02C	SPCC70.02c	0.9923	-0.05568	0.003357009	34.71	48.78	6.299	3.191
SPBC1703.07	SPBC1703.07	0.9927	-0.05364	0.003181978	36.9	51.85	6.397	3.235
SPBC21B10.02	SPBC21B10.02	0.9879	-0.0493	0.005287015	37.44	52.6	3.939	1.227
SPBC2A9.03	SPBC2A9.03	0.991	-0.04893	0.003926346	36.91	51.86	4.664	2.45
SPAC1F7.07C	fip1	0.9931	-0.04446	0.003007018	35.78	50.26	6.249	1.948
SPAC1F3.05	SPAC1F3.05	0.995	-0.04212	0.002176919	38.38	53.92	8.251	2.131
SPBC3H7.09	erf2	0.9972	-0.03636	0.00121773	22.71	31.92	11	6.349
SPAC4F10.17	SPAC4F10.17	0.9933	-0.0345	0.002919565	37.09	52.09	4.845	1.795
SPCC191.10	SPCC191.10	0.9949	-0.03311	0.002220569	39.54	55.52	6.063	2.215
SPBC1778.09	SPBC1778.09	0.9944	-0.0318	0.002438884	36.34	51.03	5.213	2.13
SPCC18B5.01C	bfr1	0.9977	-0.03034	0.001000028	33.53	47.08	10.46	6.688
SPCC1620.08	SPCC1620.08	0.9974	-0.02642	0.001130636	39.99	56.15	9.933	3.048
SPAC17A2.09C	csx1	0.9978	-0.01711	0.0009565	34.71	48.73	7.955	1.214

SPAC26F1.01	sec74	0.9965	-0.01598	0.001522697	36.62	51.41	4.313	1.552
SPBC354.05C	sre2	0.9983	-0.01105	0.000738929	7.813	10.98	4.52	3.902
SPAPB1E7.11C	SPAPB1E7.11c	0.9996	-0.004536	0.000173753	11.11	15.6	7.989	5.68
SPBC1105.10	rav1	0.391	-1.40E-17	0.407823243	0	1.96E-17	1.96E-17	0
SPAC12G12.13C	cid14	1	0	0	0	0	0	0
SPBC685.07C	rpl2701	1	0	0	0	0	0	0
SPBC887.18C	hfi1	1	0	0	0	0	0	0
SPCC663.01C	ekc1	0.391	1.29E-17	0.407823243	1.29E-17	0	0	1.29E-17
SPBC25D12.02C	dnt1	0.391	3.03E-17	0.407823243	3.03E-17	0	0	3.03E-17
SPBC2D10.09	SPBC2D10.09	0.9996	0.0037	0.000173753	35.38	49.64	7.928	2.701
SPBC660.10	SPBC660.10	0.9993	0.00413	0.000304113	29.52	41.42	2.846	3.67
SPAC5H10.12C	SPAC5H10.12c	0.9995	0.006132	0.000217202	37.82	53.05	11.27	3.715
SPAC4D7.10C	spt20	0.3506	0.01383	0.455188088	0.01383	0	0	0.01383
SPBC24C6.10C	dip1	0.9959	0.01868	0.001784268	38.48	53.97	4.303	1.643
SPBC1773.17C	SPBC1773.17c	0.9963	0.02242	0.00160987	38.34	53.76	5.507	2.527
SPCC1919.15	brl1	0.9969	0.03062	0.001348404	15.01	21.02	7.935	5.021
SPBC11C11.09C	rpl502	0.6631	0.04156	0.178420972	0.08293	0.05804	0.05804	0.08293
SPBC3H7.12	rav2	0.9856	0.04275	0.006299305	2.427	3.345	2.366	1.524
SPAC7D4.02C	sfp47	0.9948	0.04836	0.002264223	39.32	55.1	8.943	2.812
SPBC4B4.12C	SPBC4B4.12c	0.9914	0.04841	0.003751085	37.36	52.35	5.207	2.123
SPAC25G10.04C	rec10	0.9933	0.05415	0.002919565	40.9	57.31	7.683	2.614
SPBC776.03	SPBC776.03	0.9925	0.05708	0.003269485	38.91	54.51	7.086	2.677
SPCC1919.03C	amk2	0.9837	0.05998	0.007137329	24.04	33.64	1.629	2.506
SPBC3D6.15	rps2501	0.9942	0.06577	0.002526241	28.52	39.92	8.867	6.289
SPBPB2B2.10C	gal7	0.9742	0.06662	0.011351875	27.13	37.97	2.364	0.9935
SPAC25H1.06	pcf3	0.9882	0.0674	0.00515515	40.23	56.35	4.58	2.905
SPAC22E12.19	snt1	0.9878	0.08554	0.005330978	35.31	49.42	6.416	2.71
SPCC1739.05	set5	0.991	0.08559	0.003926346	15.69	21.9	7.411	5.023
SPBC1706.01	tea4	0.9939	0.09345	0.002657309	24.35	34.03	11.59	8.247
SPAC8E11.02C	rad24	0.9922	0.0939	0.003400777	20.18	28.19	10.06	5.832
SPBC16A3.12C	SPBC16A3.12c	0.9845	0.09525	0.00678428	38.1	53.32	5.265	2.804
SPAC5H10.09C	SPAC5H10.09c	0.9807	0.09819	0.008463825	33.85	47.36	4.386	2.295
SPBC4B4.06	vps25	0.9907	0.1058	0.004057837	31.63	44.24	10.58	4.072
SPAC959.06C	SPAC959.06c	0.9805	0.108	0.008552402	34.69	48.53	5.224	1.912
SPBP4H10.08	qcr10	0.9828	0.1085	0.007534852	36.48	51.04	5.526	2.773
SPBC31A8.01C	rtn1	0.9836	0.1086	0.00718148	40.65	56.88	6.202	2.358
SPBC651.11C	apm3	0.9646	0.1169	0.015652742	33.67	47.08	3.237	0.8794
SPAC17G6.08	pep7	0.9815	0.118	0.008109696	37.71	52.75	5.865	2.418
SPAC11D3.04C	SPAC11D3.04c	0.9904	0.1184	0.004189368	35.25	49.3	12.37	2.68
SPAC1782.05	SPAC1782.05	0.983	0.1186	0.007446482	34.89	48.79	6.697	2.138
SPCC777.03C	SPCC777.03c	0.9785	0.119	0.00943917	38.84	54.33	5.269	1.833
SPAC139.02C	oac1	0.9746	0.1191	0.011173593	35.57	49.74	3.882	2.267
SPCC285.16C	msh6	0.9617	0.1241	0.016960384	37.73	52.76	3.175	0.8757
SPBC13G1.13	tfb2	0.967	0.1251	0.014573526	36.91	51.61	3.419	1.526
SPAC1039.02	SPAC1039.02	0.9762	0.1289	0.010461197	35.82	50.08	5.107	1.841
SPCPJ732.02C	SPCPJ732.02c	0.9806	0.1295	0.008508111	35.9	50.18	5.409	3.328
SPCC1840.06	atp5	0.9781	0.134	0.009616741	38.03	53.18	5.747	2.169
SPCC188.12	spn6	0.9827	0.1411	0.007579044	39.09	54.64	7.364	3.288
SPBC15C4.05	SPBC15C4.05	0.9845	0.142	0.00678428	38.02	53.14	8.936	2.571
SPAC821.13C	SPAC821.13c	0.9918	0.1491	0.003575896	27.65	38.59	14.31	9.547
SPAC644.07	SPAC644.07	0.9796	0.1494	0.008951224	6.432	8.815	5.788	4.002
SPAC15F9.02	seh1	0.9548	0.1496	0.02008759	36.2	50.58	2.976	1.347
SPBC1703.08C	SPBC1703.08c	0.9846	0.1498	0.006740169	36.6	51.15	9.595	2.48
SPCP1E11.04C	pal1	0.9832	0.1499	0.00735813	19.21	26.75	7.278	4.461
SPAC17A2.02C	SPAC17A2.02c	0.9848	0.1528	0.00665196	29.93	41.78	8.751	4.476
SPAC22F3.13	tsc1	0.97	0.1539	0.013228266	39.2	54.78	4.793	1.828
SPCC1919.07	SPCC1919.07	0.9753	0.1539	0.010861776	32.56	45.47	5.712	2.38
SPBC24C6.09C	SPBC24C6.09c	0.9812	0.1571	0.00824246	36.28	50.68	7.99	2.666
SPAC1783.01	SPAC1783.01	0.976	0.1592	0.010550182	38.26	53.45	5.586	3.144
SPAC9.10	thi9	0.9798	0.161	0.008862565	38.41	53.66	7.498	2.778
SPBC17G9.10	rpl1102	0.9702	0.1635	0.01313873	36.39	50.83	4.899	2.275
SPBC839.17C	fkh1	0.9698	0.1664	0.01331782	37.7	52.66	5.424	1.4
SPBC14F5.07	doa10	0.9555	0.1667	0.019769309	35.35	49.37	3.442	1.417
SPAC26A3.07C	rpl1101	0.9717	0.1675	0.012467797	29.93	41.76	5.469	2.219
SPBC1685.10	rps27	0.983	0.1717	0.007446482	38.98	54.45	9.889	2.749
SPBC646.08C	SPBC646.08c	0.9757	0.1761	0.010683695	35.59	49.69	7.23	1.64
SPBC17D1.07C	SPBC17D1.07c	0.961	0.1767	0.017276612	35.11	49.02	3.444	2.453
SPCC790.02	pep3	0.99	0.1777	0.004364805	19.17	26.65	13.02	9.959
SPAC9E9.14	vps24	0.9762	0.1807	0.010461197	29.09	40.56	7.15	2.647

SPBC8D2.03C	hhf2	0.9877	0.183	0.005374946	26.96	37.57	12.3	7.288
SPCC965.14C	SPCC965.14c	0.9744	0.1881	0.011262725	37.44	52.26	6.396	3.241
SPCC61.02	spt3	0.9785	0.1965	0.00943917	41.03	57.3	8.256	3.666
SPAC589.07C	atg18	0.9891	0.1979	0.004759798	28.15	39.23	13.72	9.822
SPBC13E7.04	atp16	0.9793	0.2013	0.009084245	24.19	33.66	7.557	5.384
SPAC57A7.12	SPAC57A7.12	0.8609	0.2025	0.065047292	1.618	1.986	0.817	0.9684
SPBC14F5.10C	SPBC14F5.10c	0.9791	0.2046	0.009172949	19.09	26.5	8.182	4.664
SPAC30D11.10	rad22	0.8799	0.2048	0.055566682	1.404	1.683	1.415	0.8152
SPAC24H6.07	rps901	0.9663	0.2134	0.01488802	10.25	14.09	4.731	3.491
SPBC2F12.04	rpl1701	0.9729	0.2141	0.011931797	37.2	51.9	6.399	3.969
SPAC23H4.02	ppk9	0.9708	0.2151	0.012870232	35.03	48.85	7.038	2.375
SPAC25A8.01C	fft3	0.9426	0.222	0.025672565	27.84	38.75	3.39	1.678
SPBC30D10.03C	SPBC30D10.03c	0.948	0.2225	0.023191663	36.02	50.23	3.945	1.61
SPBC1709.14	SPBC1709.14	0.9693	0.2241	0.013541787	36.04	50.26	6.005	3.581
SPAC20G8.08C	fft1	0.9811	0.2243	0.008286724	19.12	26.51	8.885	6.477
SPBC3F6.05	rga1	0.9615	0.2253	0.017050711	39.94	55.72	4.504	3.114
SPAC589.03C	SPAC589.03c	0.964	0.2257	0.015922966	38.24	53.34	5.769	2.372
SPAC140.03	arb1	0.975	0.2321	0.010995384	36.28	50.58	7.705	4.508
SPBC25B2.07C	mug164	0.9127	0.2352	0.039671949	34.05	47.44	1.951	1.514
SPAC821.10C	sod1	0.9654	0.2399	0.015292706	37.92	52.88	5.341	3.703
SPAC8C9.11	SPAC8C9.11	0.9599	0.2458	0.017774008	36.41	50.74	5.725	2.199
SPBC1685.04	SPBC1685.04	0.9677	0.2512	0.014259259	39.46	55.01	6.527	3.683
SPAC15A10.10	mde6	0.9853	0.2555	0.006431517	26.94	37.44	13.71	9.061
SPAC13G6.04	tim8	0.9531	0.2605	0.02086153	37.82	52.69	5.005	2.242
SPAC11E3.15	rpl22	0.9581	0.2671	0.01858916	38.33	53.4	5.58	2.776
SPAC15A10.05C	mug182	0.9811	0.2743	0.008286724	26.74	37.13	11.35	7.63
SPAC1783.07C	pap1	0.9239	0.2765	0.034375033	19.7	27.25	3.156	1.603
SPAC1687.10	mcp1	0.9207	0.2779	0.035881857	36.5	50.82	3.037	1.558
SPBC409.08	SPBC409.08	0.9837	0.2814	0.007137329	25.8	35.8	13.3	9.249
SPBC3E7.09	SPBC3E7.09	0.9663	0.2866	0.01488802	38.61	53.78	8.493	1.798
SPBP16F5.07	apm1	0.9423	0.2956	0.025810809	28.04	38.93	3.038	3.239
SPBC4.02C	SPBC4.02c	0.9683	0.2959	0.013990068	39.15	54.52	8.674	3.424
SPCC794.10	SPCC794.10	0.9713	0.2959	0.012646611	38.25	53.25	10.17	2.64
SPAC17G8.05	med20	0.9833	0.2964	0.007313961	28.27	39.25	13.81	9.313
SPAC1687.05	pli1	0.9331	0.2981	0.030071811	35.56	49.47	4.045	1.757
SPCC320.06	SPCC320.06	0.9622	0.3044	0.016734647	38.25	53.24	5.264	4.876
SPAC31A2.13C	sft1	0.9292	0.3129	0.031890799	25.62	35.52	2.653	2.776
SPBC4F6.12	pxl1	0.9398	0.3147	0.026964559	33.24	46.2	4.733	2.086
SPBC21C3.16C	spt4	0.3506	0.3173	0.455188088	0.3173	0	0	0.3173
SPAC17D4.03C	cis4	0.9349	0.3173	0.02923484	36.41	50.64	4.379	1.979
SPCC1753.02C	git3	0.9374	0.3173	0.028075051	34.18	47.51	4.468	2.171
SPAP27G11.08C	meu32	0.9504	0.3175	0.022093572	39.58	55.08	6.052	2.157
SPBC428.15	SPBC428.15	0.946	0.3213	0.024108864	36.18	50.32	5.203	2.6
SPBC16G5.15C	fkh2	0.8757	0.3223	0.05764465	23.18	32.07	1.249	1.73
SPAC14C4.14	atp1	0.9612	0.3235	0.017186238	17.22	23.7	7.125	3.836
SPCC330.06C	SPCC330.06c	0.7469	0.324	0.126737541	0.813	0.6862	0.6862	0.813
SPAC1952.12C	csn71	0.9595	0.3269	0.017955021	37.72	52.47	6.714	3.897
SPBC28E12.02	SPBC28E12.02	0.9532	0.3285	0.020815966	33.03	45.89	6.672	2.296
SPAC8C9.12C	SPAC8C9.12c	0.9814	0.3311	0.008153946	28.53	39.57	13.26	9.813
SPAC29B12.11C	SPAC29B12.11c	0.9612	0.3427	0.017186238	38.8	53.95	8.584	2.522
SPBC23E6.02	SPBC23E6.02	0.9474	0.3444	0.02346662	33.66	46.75	5.404	3.18
SPBC902.06	mto2	0.9362	0.352	0.028631363	38.07	52.92	5.088	2.064
SPAC19A8.05C	sst4	0.9176	0.3552	0.037346595	36.71	51.01	3.562	2.089
SPAC9G1.03C	rpl3001	0.9462	0.3565	0.024017056	14.3	19.57	5.452	3.236
SPAC13G6.06C	gcv2	0.9136	0.3576	0.039243909	37.97	52.77	3.457	1.969
SPAC15F9.01C	SPAC15F9.01c	0.9467	0.3586	0.023787623	35.37	49.13	6.478	2.041
SPBC1347.09	SPBC1347.09	0.9619	0.3601	0.016870075	36.91	51.28	9.009	3.056
SPBC19C7.10	bqt4	0.9258	0.3645	0.033482824	36.43	50.6	4.428	1.981
SPBC646.06C	agn2	0.9524	0.3676	0.021180613	40.72	56.62	7.228	2.728
SPBC1539.08	arf6	0.9721	0.3694	0.012289057	15.43	21.13	10.47	6.858
SPAC630.14C	tup12	0.9576	0.3696	0.018815863	20.49	28.23	6.093	5.055
SPBC106.01	mph1	0.9065	0.3717	0.042632192	27.88	38.59	3.4	1.81
SPAC3G6.04	rnp24	0.9417	0.375	0.02608743	37.91	52.67	5.69	2.728
SPAC2G11.13	atg22	0.9598	0.3758	0.017819254	38.03	52.84	9.308	2.074
SPBC20F10.05	nrl1	0.9146	0.3818	0.038768803	37.93	52.69	3.874	1.985
SPCC4G3.19	alp16	0.918	0.383	0.037157319	33.64	46.67	4.448	1.506
SPAC1639.01C	SPAC1639.01c	0.963	0.3848	0.016373713	26.94	37.25	7.816	5.961
SPBC19C7.09C	uve1	0.957	0.3864	0.019088062	42.34	58.86	8.059	3.688
SPCC1840.10	lsm8	0.8847	0.3903	0.053203973	34.62	48.03	2.991	1.427

SPBC32H8.02C	nep2	0.9356	0.3913	0.028909787	32.81	45.49	5.699	2.12
SPAC25A8.02	SPAC25A8.02	0.9058	0.3927	0.042967684	36.72	50.97	3.823	1.582
SPBC13E7.09	vrp1	0.9317	0.3936	0.030723905	37.5	52.06	5.749	1.253
SPBC31F10.05	mug37	0.9458	0.3958	0.02420069	40.23	55.9	6.895	2.486
SPBC17D11.02C	hrd1	0.9293	0.3992	0.031844063	38.43	53.36	5.044	2.33
SPAPB17E12.02	yip12	0.9476	0.3999	0.023374948	39.31	54.6	7.146	2.697
SPBPB10D8.05C	SPBPB10D8.05c	0.9224	0.4016	0.035080706	37.42	51.94	4.483	2.299
SPBC24C6.06	gpa1	0.9671	0.4067	0.014528617	33.46	46.38	9.962	6.595
SPAC1F12.05	SPAC1F12.05	0.969	0.4138	0.013676223	30.23	41.84	10.88	6.619
SPBC16C6.02C	vps1302	0.946	0.4161	0.024108864	18.61	25.53	6.142	4.15
SPAP7G5.06	per1	0.8869	0.4186	0.052125345	35.83	49.68	2.894	1.924
SPBC25B2.08	SPBC25B2.08	0.9254	0.4221	0.033670505	35.72	49.52	5.07	2.322
SPCC613.12C	raf1	0.9651	0.4236	0.015427684	29.28	40.49	10.27	5.673
SPCC1020.06C	tal1	0.9662	0.4268	0.014932967	27.9	38.54	10.01	6.514
SPAC1786.01C	SPAC1786.01c	0.893	0.4306	0.049148541	37.67	52.26	3.009	2.194
SPAC12G12.01C	SPAC12G12.01c	0.925	0.4375	0.033858267	36.2	50.18	5.101	2.542
SPAC4G9.06C	chz1	0.939	0.4412	0.027334408	32.81	45.42	6.945	2.245
SPAC10F6.07C	mug94	0.9375	0.4418	0.028028724	35.35	48.97	6.01	3.273
SPBC14F5.03C	kap123	0.7593	0.4419	0.1195866	26.98	37.23	0.6897	1.251
SPBC115.02C	SPBC115.02c	0.9267	0.4424	0.033060837	36.92	51.18	5.121	2.793
SPAC2F7.07C	cph2	0.9539	0.4425	0.020497151	14.52	19.75	7.412	5.092
SPAC26H5.11	mug56	0.9344	0.4426	0.02946717	37.39	51.85	5.958	2.873
SPAC56F8.04C	ppt1	0.958	0.4442	0.018634491	17.16	23.46	8.078	5.685
SPAC18G6.04C	shm2	0.9262	0.4452	0.033295223	33.46	46.33	2.448	4.032
SPBC29A3.03C	SPBC29A3.03c	0.9226	0.4521	0.03498655	37.42	51.87	5.035	2.619
SPAC7D4.08	SPAC7D4.08	0.9364	0.4533	0.028538595	31.92	44.15	6.718	2.441
SPAC22H10.02	SPAC22H10.02	0.8376	0.4546	0.076963331	33.83	46.83	2.144	1.476
SPAC2C4.15C	ubx2	0.9685	0.4567	0.013900375	28.33	39.11	11.45	7.525
SPAC3G9.11C	SPAC3G9.11c	0.9732	0.4708	0.0117979	25.79	35.52	13.48	9.413
SPBC29A3.10C	atp14	0.6841	0.4725	0.16488041	1.288	1.144	1.144	0.7466
SPAC22G7.08	ppk8	0.9518	0.4741	0.0214543	33.36	46.14	8.465	4.463
SPBC146.06C	SPBC146.06c	0.9011	0.484	0.04522701	35.64	49.33	4.166	2.244
SPBC1604.12	SPBC1604.12	0.9154	0.4927	0.038389092	37.56	52.01	5.169	2.444
SPCC1840.11	csl4	0.9542	0.4935	0.020360588	30.91	42.67	8.613	5.505
SPAC17A5.09C	SPAC17A5.09c	0.9129	0.4944	0.039576793	35.74	49.46	5.296	2.022
SPAC19G12.10C	cpy1	0.9044	0.4967	0.043639446	37.31	51.66	4.009	2.749
SPCC1393.03	rps1501	0.9168	0.4973	0.037725395	38.67	53.56	5.736	1.832
SPAP8A3.07C	SPAP8A3.07c	0.9649	0.4983	0.015517694	24.64	33.87	11.49	7.133
SPBC1289.10C	adn2	0.9561	0.5001	0.019496682	29.98	41.36	10.12	4.794
SPAPYUG7.06	mug67	0.9729	0.5002	0.011931797	28.84	39.76	14.18	9.87
SPAC8F11.02C	dph3	0.8957	0.5013	0.047837426	31.15	43.01	4.261	2.015
SPBC18H10.19	atg14	0.8028	0.5029	0.095392636	30.74	42.42	2.254	1.038
SPAC8E11.05C	SPAC8E11.05c	0.969	0.5037	0.013676223	26.04	35.83	12.1	8.957
SPBC1348.14C	ght7	0.9127	0.5039	0.039671949	35.77	49.48	5.753	1.236
SPBC11G11.01	fis1	0.9608	0.5091	0.017367006	24.33	33.43	10.44	6.588
SPAC20G4.03C	hri1	0.9251	0.5118	0.033811319	37.58	52.01	6.202	2.695
SPBC4C3.12	Sep-01	0.9666	0.5119	0.014753209	21.95	30.09	11.7	8.223
SPCC1840.08C	SPCC1840.08c	0.9669	0.5204	0.01461844	24.18	33.19	11.97	8.505
SPCC1672.03C	SPCC1672.03c	0.9311	0.521	0.031003673	35.82	49.53	7.206	2.438
SPAC13F5.04C	SPAC13F5.04c	0.9332	0.5214	0.03002527	38.38	53.12	7.005	3.177
SPCC737.04	SPCC737.04	0.9355	0.5217	0.028956208	34.25	47.32	7.177	3.406
SPBPB7E8.01	SPBPB7E8.01	0.9268	0.5242	0.033013975	13.57	18.31	4.078	4.588
SPBC646.09C	int6	0.8498	0.5252	0.070683273	35.98	49.74	3.472	0.7199
SPBC1921.01C	rpl35b	0.9181	0.5262	0.037110013	31.23	43.08	5.68	2.727
SPCC23B6.01C	SPCC23B6.01c	0.7969	0.5287	0.098596173	19.72	26.92	1.439	1.656
SPBC1347.12	arp1	0.9397	0.5304	0.027010773	39.88	55.22	8.491	2.627
SPBC1709.10C	atx1	0.9348	0.5436	0.029281296	35.63	49.22	7.314	3.611
SPAC4F10.08	mug126	0.7532	0.5451	0.123089689	36.85	50.94	2.128	0.5446
SPAC458.02C	SPAC458.02c	0.9054	0.5502	0.04315951	38.35	53.04	5.102	2.504
SPAC23H4.17C	srb10	0.8698	0.5514	0.060580597	3.881	4.672	2.893	2.474
SPAC1B1.02C	SPAC1B1.02c	0.8854	0.5536	0.052860482	33.69	46.49	3.775	2.512
SPAC637.07	moe1	0.9439	0.5598	0.025074014	15.12	20.43	7.834	5.203
SPBC1711.15C	SPBC1711.15c	0.9414	0.5607	0.026225806	37.26	51.5	8.477	4.059
SPBC725.10	SPBC725.10	0.6897	0.5682	0.161339774	23.53	32.22	1.603	0.7038
SPAC3G6.02	dss1	0.9664	0.576	0.014843079	26.69	36.63	13.18	9.139
SPAC25H1.04	mug105	0.9551	0.578	0.019951155	30.01	41.3	10.95	5.949
SPAC11E3.09	pyp3	0.9305	0.5822	0.031283623	36.3	50.12	7.862	2.905
SPCC1259.07	rxt3	0.9604	0.5926	0.017547849	21.45	29.27	11.64	7.885
SPAC13C5.07	rad32	0.9475	0.5945	0.023420781	19.79	26.94	7.589	6.741

SPBC17A3.05C	SPBC17A3.05c	0.9071	0.5965	0.042344833	24.79	33.95	4.558	3.664
SPAC26F1.10C	pyp1	0.8707	0.6005	0.060131456	38.17	52.71	3.726	2.33
SPBC23G7.11	mag2	0.8805	0.6124	0.05527064	37.06	51.14	3.895	2.743
SPBC16A3.08C	SPBC16A3.08c	0.9413	0.6168	0.026271941	15.66	21.1	7.876	5.755
SPBC21D10.12	hob1	0.9116	0.617	0.040195683	35.94	49.57	6.58	2.361
SPCC1672.06C	asp1	0.9167	0.6203	0.037772769	38.49	53.13	6.228	3.542
SPAC23H3.11C	SPAC23H3.11c	0.8798	0.6295	0.055616042	36.5	50.33	4.408	2.444
SPAC694.02	SPAC694.02	0.806	0.6308	0.093664958	20.25	27.53	2.463	1.721
SPAC30.02C	SPAC30.02c	0.9436	0.6315	0.025212068	30.33	41.67	9.798	4.868
SPBC21.03C	SPBC21.03c	0.9382	0.6316	0.027704571	28.86	39.61	8.835	4.58
SPAP8A3.02C	ofd2	0.9418	0.6321	0.026041314	30.62	42.08	9.393	4.856
SPAC30C2.07	SPAC30C2.07	0.8857	0.6328	0.052713355	28.5	39.1	3.532	3.369
SPBC660.11	tcg1	0.7198	0.6335	0.142788158	1.449	1.145	1.145	1.449
SPAC1782.11	met14	0.9436	0.6342	0.025212068	27.69	37.96	8.862	6.141
SPAC1834.10C	SPAC1834.10c	0.8918	0.6379	0.049732532	32.15	44.22	5.185	2.515
SPBC1718.03	ker1	0.8902	0.6384	0.05051241	9.393	12.28	4.341	3.174
SPAC607.10	spo3	0.909	0.6385	0.041436117	37.59	51.85	5.931	3.27
SPAC3F10.13	ucp6	0.8775	0.6402	0.056752875	37.93	52.33	4.825	1.913
SPCC4B3.02C	SPCC4B3.02c	0.9053	0.6417	0.04320748	38.35	52.91	5.669	3.213
SPCC584.11C	SPCC584.11c	0.7592	0.6482	0.119643801	24.15	32.98	2.43	0.9954
SPCP1E11.07C	cwf18	0.863	0.65	0.063989204	23.88	32.59	3.444	2.645
SPBC19G7.01C	msh2	0.8974	0.6526	0.047013935	25.26	34.53	5.591	2.725
SPAC22A12.07C	ogm1	0.9272	0.6542	0.032826577	36.64	50.49	7.44	4.338
SPBC409.18	SPBC409.18	0.9176	0.6564	0.037346595	38.11	52.55	7.764	2.187
SPAC29B12.03	spd1	0.8896	0.6595	0.050805226	36.07	49.69	5.965	1.199
SPBC1215.01	shy1	0.9265	0.6595	0.033154576	30.94	42.48	8.013	3.7
SPAC521.05	rps802	0.9537	0.6611	0.020588217	21.83	29.7	10.94	7.658
SPAC31G5.12C	maf1	0.9569	0.6629	0.019133445	26.99	36.95	12.23	7.918
SPAC12G12.03	cip2	0.8693	0.6651	0.06083032	38.32	52.83	3.954	2.655
SPBC1198.06C	SPBC1198.06c	0.9369	0.6675	0.028306761	19.06	25.81	8.376	5.744
SPCC569.04	SPCC569.04	0.8578	0.6706	0.066613958	36.82	50.72	4.65	1.093
SPAPB18E9.01	trm5	0.889	0.672	0.051098239	39.36	54.28	5.145	2.777
SPAC1296.06	tah18	0.9375	0.6731	0.028028724	12.17	16.13	8.27	6.033
SPCC970.07C	raf2	0.9583	0.6751	0.018498512	24.1	32.87	12.39	8.707
SPAC186.06	SPAC186.06	0.8842	0.6776	0.053449489	38.51	53.09	4.984	2.673
SPBC11C11.11C	SPBC11C11.11c	0.9295	0.6787	0.031750606	34	46.76	8.536	4.053
SPBC16G5.07C	SPBC16G5.07c	0.8777	0.6807	0.056653902	35.99	49.54	4.917	2.34
SPCC31H12.05C	sds21	0.8087	0.6872	0.092212557	39.16	53.99	3.623	0.3758
SPCC1919.01	ppk34	0.8696	0.689	0.060680469	37.35	51.44	4.947	1.816
SPBC1198.03C	SPBC1198.03c	0.8913	0.6893	0.049976093	35.95	49.47	5.685	2.566
SPAC222.16C	csn3	0.9054	0.6932	0.04315951	37.94	52.27	6.503	3.064
SPAC688.10	rev3	0.876	0.6952	0.057495894	34.83	47.9	5.152	2.083
SPAC23H3.06	apl6	0.9211	0.6999	0.035693218	31.91	43.79	8.064	3.477
SPCC1620.04C	mug55	0.88	0.7003	0.055517328	34.99	48.11	5.48	1.989
SPBC365.04C	SPBC365.04c	0.9082	0.7061	0.041818502	34.68	47.66	7.329	2.465
SPBPB2B2.07C	SPBPB2B2.07c	0.9487	0.7169	0.022871099	30.42	41.68	11.59	6.747
SPBC1604.02C	ppr1	0.8906	0.7202	0.050317309	8.122	10.39	4.872	3.79
SPCC330.02	rhp7	0.8448	0.7217	0.073246095	34.17	46.93	4.496	1.284
SPBC29A3.02C	his7	0.9564	0.7251	0.019360433	27.22	37.18	13.33	8.468
SPAC1399.04C	SPAC1399.04c	0.9353	0.73	0.029049066	37.09	51.01	10.2	4.502
SPCC553.01C	SPCC553.01c	0.824	0.734	0.084072788	37.47	51.54	3.79	1.573
SPAC4F8.10C	stg1	0.9227	0.7371	0.034939479	29.38	40.18	8.049	4.456
SPAC26A3.14C	SPAC26A3.14c	0.8506	0.7373	0.070274622	34.43	47.27	4.267	2.162
SPBC1921.07C	sgf29	0.9465	0.7379	0.023879382	20.33	27.49	11.05	6.996
SPAC2E12.03C	SPAC2E12.03c	0.8746	0.7435	0.058190527	38.53	53.02	5.224	2.506
SPBC18A7.02C	SPBC18A7.02c	0.9039	0.7453	0.043879614	29.76	40.71	7.257	2.704
SPCC965.07C	gst2	0.8534	0.7461	0.068847361	23.42	31.82	3.709	2.822
SPBC146.04	SPBC146.04	0.8499	0.749	0.070632171	40.6	55.91	4.352	2.143
SPAC1039.09	isp5	0.8917	0.7523	0.049781233	31.98	43.82	6.047	3.031
SPBC337.04	ppk27	0.7405	0.7593	0.130474937	34.31	47.07	1.906	1.665
SPBC27B12.08	sip1	0.9258	0.7624	0.033482824	31.2	42.7	7.536	5.731
SPBC12C2.04	SPBC12C2.04	0.9457	0.7657	0.024246611	27.59	37.63	11.73	6.773
SPAC8C9.19	SPAC8C9.19	0.8646	0.7667	0.063184769	24.28	32.99	4.918	2.469
SPAC18G6.01C	SPAC18G6.01c	0.909	0.7674	0.041436117	30.53	41.76	7.772	3.139
SPCC1235.02	bio2	0.9087	0.7731	0.041579472	36.44	50.05	6.829	4.252
SPCC830.07C	psi1	0.9112	0.775	0.040386289	36.17	49.67	7.657	3.762
SPCC584.03C	SPCC584.03c	0.8689	0.7761	0.061030203	29.79	40.71	4.053	3.368
SPAC806.07	ndk1	0.8622	0.7763	0.064391981	39.13	53.82	4.895	2.451
SPAC521.02	SPAC521.02	0.9013	0.7781	0.045130629	28.78	39.29	7.205	3.015

SPBC3B9.15C	scp1	0.9109	0.7823	0.040529298	39.1	53.76	8.021	3.383
SPBP23A10.10	ppk32	0.8735	0.7825	0.058737091	31.71	43.39	4.144	3.659
SPCC16C4.09	sts5	0.9194	0.7847	0.036495501	40.86	56.23	8.889	3.758
SPBC16E9.17C	rem1	0.8757	0.7876	0.05764465	37.46	51.45	6.109	1.847
SPCC736.11	ago1	0.6476	0.7884	0.18869316	1.458	0.9393	0.9393	1.458
SPCC16C4.06C	SPCC16C4.06c	0.8588	0.7934	0.066107964	34.93	47.89	4.913	2.411
SPAC1142.05	ctr5	0.8581	0.7941	0.066462098	5.5	6.602	3.909	3.213
SPCC1393.13	SPCC1393.13	0.956	0.7958	0.019542108	22.67	30.69	13.89	9.682
SPBC18H10.18C	SPBC18H10.18c	0.9249	0.8023	0.033905221	39.58	54.41	10.25	3.309
SPBC1685.01	pmp1	0.8805	0.803	0.05527064	38.21	52.48	6.524	1.865
SPBPB2B2.06C	SPBPB2B2.06c	0.8119	0.8065	0.090497459	35.37	48.5	3.699	1.862
SPAC23E2.01	fep1	0.8883	0.8071	0.051440338	36.95	50.71	5.708	3.707
SPAC15E1.06	vps29	0.9296	0.8075	0.031703885	38.77	53.27	11.31	2.849
SPBC16H5.11C	skb1	0.8888	0.8119	0.051195954	38.64	53.07	6.499	3.017
SPCC285.05	SPCC285.05	0.9115	0.8182	0.040243327	36.51	50.08	7.689	4.423
SPAC521.04C	SPAC521.04c	0.8809	0.8201	0.05507339	36.05	49.44	5.781	3.228
SPBC32C12.03C	ppk25	0.8992	0.8215	0.046143702	33.41	45.72	6.803	3.873
SPAC328.02	SPAC328.02	0.8116	0.8248	0.090657962	40.07	55.07	3.928	1.71
SPBC16H5.13	SPBC16H5.13	0.9145	0.8269	0.03881629	15.12	20.06	7.176	5.322
SPAC644.11C	pkp1	0.6793	0.8291	0.167938385	30.29	41.34	1.62	1.469
SPCC31H12.06	mug111	0.7985	0.8309	0.09772508	34.46	47.18	4.063	0.8526
SPCC1322.08	srk1	0.7863	0.8311	0.104411724	38.53	52.9	2.857	2.109
SPCC613.11C	meu23	0.815	0.8317	0.088842391	34.05	46.61	4.313	1.28
SPAC57A7.07C	SPAC57A7.07c	0.8444	0.8331	0.073451775	34.72	47.55	4.739	2.215
SPBP35G2.03C	sgo1	0.879	0.8344	0.056011125	36.78	50.44	5.781	3.235
SPAC6B12.06C	rrg9	0.9115	0.8437	0.040243327	39.52	54.27	9.155	2.919
SPAC30C2.06C	dml1	0.8017	0.8446	0.095988116	21.97	29.65	3.516	2.007
SPBC336.13C	SPBC336.13c	0.8721	0.8462	0.059433714	11.32	14.69	4.963	3.586
SPAC1F8.08	SPAC1F8.08	0.8974	0.8474	0.047013935	39.78	54.63	8.12	2.077
SPBC16G5.06	SPBC16G5.06	0.8578	0.8504	0.066613958	34.07	46.61	5.749	1.748
SPCC16C4.17	mug123	0.9347	0.8506	0.029327757	25.83	35.05	10.58	6.478
SPCC1235.09	hif2	0.8832	0.8544	0.05394094	34.1	46.64	6.57	2.943
SPAC11D3.03C	SPAC11D3.03c	0.8304	0.8546	0.080712659	38.11	52.27	4.869	1.377
SPAC23A1.02C	SPAC23A1.02c	0.939	0.8561	0.027334408	24.88	33.71	11.08	7.269
SPCC553.04	cyp9	0.92	0.8657	0.036212173	20.98	28.22	8.662	5.49
SPBC1A4.02C	leu1	0.9109	0.8691	0.040529298	40.86	56.12	9.238	3.22
SPBC1921.04C	SPBC1921.04c	0.7903	0.8724	0.102208018	36.19	49.56	3.386	1.998
SPAC17H9.09C	ras1	0.9115	0.8742	0.040243327	26.72	36.26	7.768	5.382
SPBP35G2.08C	air1	0.6672	0.8823	0.175743962	1.762	1.235	1.007	1.762
SPCC1322.10	SPCC1322.10	0.9111	0.8832	0.040433953	15.88	21.05	7.5	5.387
SPCC18.17C	SPCC18.17c	0.8463	0.8836	0.072475659	39.9	54.74	4.873	2.624
SPCC1259.02C	SPCC1259.02c	0.8347	0.8853	0.078469586	38.13	52.26	4.665	2.298
SPAC2H10.01	SPAC2H10.01	0.8302	0.8864	0.080817271	36	49.27	4.538	2.244
SPCC1494.01	SPCC1494.01	0.8159	0.8878	0.088363067	36.17	49.51	3.794	2.449
SPBC25H2.11C	spt7	0.2361	0.8895	0.626904013	1.198	0.4331	0.3389	0.6569
SPBPB2B2.09C	SPBPB2B2.09c	0.8496	0.8965	0.070785496	36.81	50.39	5.313	2.419
SPCC584.15C	SPCC584.15c	0.9137	0.8994	0.039196375	39.73	54.49	9.879	3.441
SPAC823.10C	SPAC823.10c	0.897	0.9058	0.047207557	35.6	48.69	8.756	1.885
SPAPB21F2.03	SPAPB21F2.03	0.9461	0.9064	0.024062958	27.18	36.87	12.49	9.274
SPAC19D5.01	pyp2	0.8736	0.9069	0.058687375	36.95	50.57	6.984	1.939
SPAC212.03	SPAC212.03	0.8835	0.9086	0.053793446	31.87	43.44	6.042	4.098
SPBC1604.07	atp4	0.8601	0.9118	0.065451052	35.26	48.19	5.758	2.722
SPAC31G5.03	rps1101	0.6531	0.9126	0.185020316	2.367	2.041	1.195	1.694
SPAC1250.04C	atl1	0.8005	0.9166	0.096638664	31.03	42.26	4.463	1.146
SPBP8B7.13	vac7	0.8175	0.9167	0.087512239	38.23	52.35	4.298	2.224
SPAC977.16C	dak2	0.8635	0.9176	0.063737658	36.73	50.25	6.29	2.31
SPAC3H5.10	rpl3202	0.9199	0.9193	0.036259381	13.95	18.28	8.698	6.2
SPAC3F10.16C	SPAC3F10.16c	0.3969	0.9206	0.401318901	1.247	0.4586	0.4586	0.979
SPBC19F8.02	SPBC19F8.02	0.8183	0.9208	0.087087449	32.9	44.88	4.381	2.197
SPBP8B7.06	rpp201	0.9105	0.9232	0.04072005	39.5	54.13	9.735	3.468
SPAC57A10.08C	SPAC57A10.08c	0.8299	0.9233	0.080974235	37.63	51.5	4.769	2.275
SPAC13G6.08	SPAC13G6.08	0.7872	0.9287	0.103914915	34.95	47.73	3.699	1.946
SPBC3H7.14	mug176	0.8518	0.9298	0.069662364	34.94	47.72	3.847	3.871
SPAC6F6.17	rif1	0.8737	0.9322	0.058637664	33.35	45.49	6.41	3.224
SPAC6C3.08	SPAC6C3.08	0.827	0.9335	0.08249449	35.49	48.49	4.467	2.551
SPBC17G9.12C	SPBC17G9.12c	0.8992	0.9384	0.046143702	33.95	46.33	8.133	4.035
SPAC2G11.04	SPAC2G11.04	0.7965	0.9395	0.09881422	34.23	46.72	3.284	2.581
SPBC887.06C	snx3	0.8918	0.9452	0.049732532	39.64	54.29	8.157	3.068
SPCC338.08	ctp1	0.9286	0.9477	0.032171321	13.65	17.82	9.089	7.781

SPBC691.01	pfa5	0.7407	0.948	0.130357655	36.18	49.44	3.245	1.409
SPBC428.11	SPBC428.11	0.9466	0.9488	0.0238335	28.79	39.06	13.45	9.613
SPAC821.06	spn2	0.8578	0.9491	0.066613958	36.93	50.49	5.608	3.105
SPAC17A2.14	SPAC17A2.14	0.7886	0.9509	0.103143227	34.29	46.78	3.91	1.896
SPAC10F6.17C	SPAC10F6.17c	0.8283	0.9525	0.081812339	38.5	52.69	5.22	1.806
SPAC1B3.11C	ypt4	0.7248	0.9539	0.139781815	32.94	44.88	2.307	1.989
SPAC16C9.01C	SPAC16C9.01c	0.8531	0.9556	0.069000058	35.91	49.05	5.315	3.165
SPBC24C6.11	cwf14	0.9131	0.9559	0.039481657	18.06	24	8.423	5.874
SPBC23G7.07C	SPBC23G7.07c	0.8577	0.9568	0.06666459	29.99	40.73	6.399	2.104
SPBC19G7.09	ulp1	0.8626	0.9577	0.064190546	38.3	52.4	6.488	2.445
SPBC21C3.09C	SPBC21C3.09c	0.844	0.9593	0.073657553	36.88	50.41	5.589	2.341
SPAC11D3.13	SPAC11D3.13	0.9082	0.9659	0.041818502	26.59	35.96	8.457	5.296
SPBC16G5.11C	bag101	0.8674	0.9682	0.061780582	35.09	47.88	6.841	2.49
SPAC26F1.12C	SPAC26F1.12c	0.8481	0.969	0.071552937	39.13	53.55	5.814	2.412
SPAC688.13	scn1	0.8774	0.9725	0.05680237	33.37	45.46	7.073	3.251
SPCC4B3.07	nro1	0.8249	0.9754	0.083598696	35.77	48.83	4.953	2.251
SPBC16D10.01C	SPBC16D10.01c	0.8669	0.9764	0.062030997	37.1	50.69	7.336	1.378
SPAC17G6.05C	SPAC17G6.05c	0.6565	0.9776	0.18276527	32.7	44.52	2.319	1.244
SPBC18E5.05C	iki1	0.7874	0.9807	0.10380459	4.666	5.17	3.007	2.846
SPCC1682.13	laf2	0.818	0.981	0.087246696	35.56	48.52	4.625	2.376
SPBP4H10.11C	lcf2	0.8646	0.9811	0.063184769	34.82	47.48	6.059	3.403
SPBC31F10.07	lsb5	0.6961	0.9812	0.157328366	31.4	42.68	2.742	1.358
SPAC23H4.09	cdb4	0.8503	0.9816	0.070427821	38.38	52.48	5.571	2.983
SPAC3H8.04	SPAC3H8.04	0.8495	0.9821	0.070836617	35.75	48.78	4.653	3.68
SPAC212.08C	SPAC212.08c	0.742	0.9828	0.129596095	34.08	46.44	2.704	2.098
SPAC15E1.02C	SPAC15E1.02c	0.9419	0.9836	0.025995203	27.07	36.61	12.96	9.056
SPAC6G9.12	cfr1	0.8233	0.9876	0.084441885	35.38	48.26	4.463	2.788
SPCC306.05C	ins1	0.8845	0.9884	0.053302163	40.4	55.3	6.751	4.395
SPCC1235.05C	fft2	0.7744	0.9911	0.111034656	36.48	49.79	3.277	2.339
SPBC3H7.07C	ser2	0.8206	0.9918	0.085868487	38.43	52.53	5.122	1.928
SPAC3H1.10	SPAC3H1.10	0.8499	0.9924	0.070632171	40.09	54.86	6.173	2.268
SPBC428.10	SPBC428.10	0.7928	0.9933	0.100836359	39.91	54.6	4.565	1.399
SPAC1B1.04C	SPAC1B1.04c	0.7433	0.9952	0.128835867	38.01	51.94	3.478	1.445
SPBP4H10.05C	spe2	0.8207	0.9982	0.085815567	39.19	53.59	4.353	2.854
SPBC660.08	SPBC660.08	0.8077	0.9993	0.092749917	41.02	56.15	4.909	1.625
SPBC16C6.08C	qcr6	0.8412	1	0.075100736	37.36	51.02	5.405	2.8
SPBC16C6.11	rpl3201	0.9325	1.001	0.03035116	14.35	18.73	10.83	8.3
SPAC1B3.07C	vps28	0.7711	1.002	0.112889297	20.31	27.09	3.855	1.763
SPBPB10D8.04C	SPBPB10D8.04c	0.8111	1.002	0.090925599	37.24	50.84	4.838	1.961
SPBC354.12	gpd3	0.9371	1.002	0.028214062	23.95	32.2	12.08	8.614
SPBC1734.04	anp1	0.831	1.004	0.080398976	19.22	25.55	3.986	3.633
SPCC330.03C	SPCC330.03c	0.8035	1.011	0.095014119	37.62	51.36	4.635	1.973
SPCP1E11.05C	are2	0.9093	1.012	0.041292809	41.05	56.18	10.75	3.342
SPCC338.04	cid2	0.8211	1.013	0.085603948	37.4	51.05	5.1	2.201
SPBC1709.18	tif452	0.7647	1.015	0.11650891	39.75	54.34	3.897	1.597
SPAC23H3.13C	gpa2	0.9014	1.016	0.045082446	18.3	24.25	8.202	5.264
SPAC30D11.13	hus5	0.7757	1.02	0.110306209	2.856	2.576	2.576	2.856
SPBC106.11C	plg7	0.8833	1.021	0.05389177	37.92	51.77	7.807	3.579
SPBC30B4.08	eri1	0.7869	1.023	0.104080455	32.04	43.52	4.437	1.646
SPBC409.06	uch2	0.8561	1.029	0.067475503	34.51	46.98	6.577	2.626
SPAC15A10.16	bud6	0.9097	1.031	0.041101805	28.57	38.64	8.852	6.312
SPCC24B10.18	SPCC24B10.18	0.8746	1.032	0.058190527	36.63	49.94	7.656	2.916
SPAC25B8.10	SPAC25B8.10	0.8726	1.035	0.059184791	37.49	51.15	7.494	2.975
SPBP8B7.27	mug30	0.7805	1.041	0.107627093	38.31	52.29	4.442	1.513
SPCC126.02C	pku70	0.8382	1.042	0.076652344	29.74	40.27	5.907	2.371
SPCC162.05	coq3	0.8883	1.042	0.051440338	9.661	12.09	6.1	5.61
SPAC29A4.11	rga3	0.8555	1.044	0.067779986	37.58	51.26	6.471	2.904
SPAPB17E12.13	rpl1802	0.9015	1.047	0.045034269	32.6	44.27	7.676	5.985
SPAC23C4.07	tht2	0.8495	1.049	0.070836617	34.94	47.56	6.516	2.386
SPBC14C8.05C	meu17	0.7955	1.05	0.099359816	32.8	44.56	3.705	2.774
SPBC947.01	SPBC947.01	0.8131	1.051	0.089856039	35.77	48.72	4.749	2.553
SPBC2D10.16	mhf1	0.6649	1.053	0.177243667	33.07	44.93	2.599	1.367
SPAC513.03	mfm2	0.7194	1.053	0.143029567	28.05	37.88	2.515	2.141
SPAC823.16C	mug179	0.8323	1.054	0.079720105	37.31	50.87	5.775	2.293
SPCC645.12C	SPCC645.12c	0.7957	1.056	0.099250642	37.03	50.47	4.554	2.113
SPAC1783.02C	vps66	0.7218	1.06	0.141583122	31.86	43.22	2.7	2.038
SPCC1223.11	ptc2	0.8352	1.065	0.078209514	27.53	37.13	4.349	3.784
SPBC1709.11C	png2	0.6575	1.066	0.182104243	34.51	46.93	2.298	1.594
SPCC24B10.19C	SPCC24B10.19c	0.8299	1.069	0.080974235	36.06	49.1	5.217	2.959

SPCC4B3.15	mid1	0.626	1.07	0.203425667	30.06	40.67	1.233	1.842
SPBC21.07C	ppk24	0.8256	1.072	0.083230316	35.12	47.77	5.306	2.676
SPAC19G12.15C	tpp1	0.8621	1.072	0.064442355	10.83	13.69	6.82	3.3
SPBC29A10.07	pom152	0.8352	1.077	0.078209514	39.07	53.31	5.891	2.552
SPBP4H10.10	SPBP4H10.10	0.8465	1.077	0.072373038	39.78	54.31	5.495	3.615
SPBC1773.08C	omh4	0.7663	1.08	0.115601174	36.56	49.78	4.192	1.689
SPAC25B8.09	SPAC25B8.09	0.7958	1.083	0.099196065	35.92	48.88	4.654	2.191
SPBC27.06C	mgr2	0.8416	1.084	0.074894273	8.513	10.42	3.722	4.409
SPBC1198.14C	fbp1	0.7948	1.085	0.099742142	35.08	47.69	3.431	3.14
SPBC16H5.08C	SPBC16H5.08c	0.8769	1.087	0.05704993	31.08	42.09	8.369	2.85
SPBC660.05	SPBC660.05	0.8875	1.088	0.051831638	34.87	47.4	7.279	5.236
SPCC777.04	SPCC777.04	0.7575	1.09	0.120617363	36.27	49.36	4.11	1.575
SPBC25B2.06C	btb2	0.8863	1.091	0.052419251	40.24	54.93	9.588	1.922
SPBC336.01	fbh1	0.9091	1.092	0.041388342	19.32	25.57	9.157	6.445
SPCC162.10	ppk33	0.7323	1.093	0.135310966	34.43	46.78	3.336	1.899
SPAC823.05C	tlg2	0.8976	1.095	0.046917156	6.279	7.274	7.274	6.279
SPAC32A11.01	mug8	0.9353	1.096	0.029049066	29.74	40.19	13.23	8.874
SPCC970.05	rpl3601	0.7437	1.098	0.128602219	36.41	49.55	4.11	1.077
SPAC1805.11C	rps2602	0.7995	1.101	0.097181532	38.66	52.7	4.208	2.864
SPAC18B11.04	ncs1	0.6199	1.103	0.207678364	39.69	54.14	1.547	1.773
SPCC1442.02	SPCC1442.02	0.8603	1.103	0.065350077	34.57	46.96	6.75	3.555
SPCC1020.08	SPCC1020.08	0.8505	1.107	0.070325682	38.85	52.95	6.754	2.801
SPBP35G2.06C	nup131	0.8583	1.111	0.066360887	37.05	50.43	7.189	2.916
SPBC947.08C	hip4	0.7948	1.115	0.099742142	33.63	45.62	3.918	3.003
SPCC965.11C	SPCC965.11c	0.6422	1.116	0.192329699	32.2	43.61	2.767	1.078
SPAC13A11.01C	rga8	0.8762	1.116	0.057396751	36.82	50.1	7.501	4.286
SPBC530.15C	SPBC530.15c	0.8304	1.117	0.080712659	37.05	50.41	6.124	2.278
SPBC32H8.03	bem46	0.8831	1.118	0.053990115	33.3	45.16	8.442	4.037
SPBC2A9.04C	SPBC2A9.04c	0.8736	1.119	0.058687375	29.45	39.76	7.317	4.248
SPAPB1E7.02C	mcl1	0.9146	1.121	0.038768803	18.4	24.25	10.01	7.036
SPCC330.01C	rhp16	0.7495	1.122	0.125228363	38.43	52.34	3.984	1.726
SPAP14E8.02	SPAP14E8.02	0.8091	1.123	0.091997799	38.5	52.44	5.012	2.624
SPAC3F10.11C	abc2	0.8201	1.123	0.086133188	36.99	50.33	5.959	1.851
SPAC17H9.04C	SPAC17H9.04c	0.7482	1.125	0.125982296	38.08	51.86	2.72	2.709
SPBP4H10.04	ppb1	0.744	1.126	0.128427064	36.53	49.68	4.198	1.164
SPBC530.09C	SPBC530.09c	0.8103	1.126	0.091354161	36.31	49.37	4.904	2.8
SPAC19G12.16C	adg2	0.9339	1.142	0.029699625	25.77	34.56	13.45	9.075
SPAC2F7.02C	SPAC2F7.02c	0.8057	1.145	0.093826636	39.38	53.65	2.565	3.969
SPAC23A1.16C	rtr1	0.8046	1.146	0.094419972	38.58	52.52	5.178	2.393
SPAC17C9.11C	SPAC17C9.11c	0.7933	1.15	0.100562546	40.52	55.24	5.1	1.991
SPAC13G6.14	aps1	0.8864	1.155	0.052370253	16.77	21.91	7.393	5.679
SPAC24H6.11C	SPAC24H6.11c	0.9305	1.155	0.031283623	27.57	37.07	12.1	9.331
SPCC16A11.15C	SPCC16A11.15c	0.8442	1.157	0.073554652	34.74	47.12	6.485	3.174
SPBC106.13	SPBC106.13	0.9006	1.159	0.045468057	31.46	42.51	9.416	5.818
SPAC4F8.03	sdo1	0.1649	1.162	0.782779344	1.684	0.7327	0.3574	0.7228
SPCC1682.01	qcr9	0.8931	1.163	0.049099911	10.68	13.35	7.708	6.21
SPBC1709.06	dus2	0.7832	1.167	0.106127321	37.34	50.75	4.797	2.114
SPAC1002.07C	ats1	0.7285	1.17	0.137570444	34.34	46.54	3.568	1.952
SPBC1289.09	tim21	0.8656	1.17	0.062682752	36.55	49.65	8.062	3.127
SPBC32F12.06	pch1	0.8231	1.172	0.084547398	37.37	50.79	5.267	3.325
SPCC1442.01	ste6	0.9306	1.175	0.031236952	27.8	37.36	12.53	9.363
SPAC1093.06C	dhc1	0.7742	1.177	0.111146833	32.77	44.33	4.362	2.37
SPAC1565.04C	ste4	0.8892	1.177	0.051000546	28.31	38.07	8.251	5.846
SPAC3C7.13C	SPAC3C7.13c	0.9169	1.177	0.037678027	28.92	38.92	12.14	6.414
SPAC328.06	ubp2	0.7497	1.179	0.125112489	37.43	50.86	1.738	3.212
SPCC1919.09	tif6	0.7482	1.181	0.125982296	36.85	50.04	4.119	1.88
SPBC19C7.08C	SPBC19C7.08c	0.7885	1.183	0.103198302	39.72	54.07	5.065	2.094
SPAC1B9.02C	sck1	0.8038	1.185	0.094851998	33.02	44.67	5.336	2.46
SPAC328.01C	SPAC328.01c	0.782	1.186	0.106793247	37.84	51.43	4.721	2.293
SPCC18B5.09C	SPCC18B5.09c	0.7471	1.187	0.126621264	36.88	50.08	3.987	2.04
SPCC1322.03	SPCC1322.03	0.9008	1.188	0.045371622	26.85	36.01	10.24	5.454
SPBC557.02C	SPBC557.02c	0.9146	1.188	0.038768803	23.26	30.97	10.65	7.435
SPAC212.01C	SPAC212.01c	0.8066	1.192	0.093341782	37.95	51.58	5.35	2.635
SPBC29A10.11C	vps902	0.8413	1.193	0.075049111	36.96	50.19	7.006	2.586
SPAC19A8.04	erg5	0.866	1.193	0.062482108	40.38	54.98	8.2	3.267
SPBC36.10	SPBC36.10	0.8947	1.194	0.048322563	30.78	41.51	9.814	5.014
SPBC14C8.17C	spt8	0.7798	1.197	0.108016769	24.59	32.82	4.232	2.765
SPCP20C8.02C	SPCP20C8.02c	0.8251	1.197	0.083493413	36.63	49.71	5.243	3.595
SPCC330.07C	SPCC330.07c	0.7994	1.202	0.097235856	38.56	52.42	4.767	2.983

SPAC25B8.06C	SPAC25B8.06c	0.8634	1.204	0.063787956	8.167	9.77	6.412	5.123
SPAC31A2.14	bun107	0.8595	1.205	0.065754119	32.35	43.71	7.343	3.852
SPAC13C5.05C	SPAC13C5.05c	0.8247	1.206	0.083704005	36.6	49.66	5.682	3.268
SPAC23H3.15C	SPAC23H3.15c	0.9224	1.21	0.035080706	27.58	37	13.3	7.16
SPBC21B10.09	SPBC21B10.09	0.9302	1.211	0.031423665	27.93	37.48	13.07	9.443
SPAC6C3.06C	SPAC6C3.06c	0.5634	1.212	0.249183157	32.53	43.95	1.613	1.601
SPAC19G12.12	dlp1	0.8314	1.213	0.08018998	17.91	23.43	5.41	4.051
SPBC4F6.04	rpl2502	0.9178	1.214	0.037251947	27.39	36.73	12.83	6.518
SPBC18E5.09C	EMPTY	0.772	1.216	0.1123827	36.44	49.43	4.377	2.509
SPBC1271.01C	pof13	0.8089	1.216	0.092105165	36.66	49.73	4.046	3.832
SPBC725.15	ura5	0.7587	1.217	0.119929916	38.06	51.7	4.183	2.312
SPCC1795.03	gms1	0.8386	1.217	0.076445142	36.73	49.82	6.728	3.038
SPBC29A3.14C	trt1	0.8951	1.217	0.048128443	21.64	28.65	8.396	6.521
SPBC2G5.02C	SPBC2G5.02c	0.9103	1.217	0.040815457	27.35	36.66	11.72	6.065
SPBC4B4.03	rsc1	0.7687	1.221	0.114243119	38.23	51.92	4.53	2.273
SPAC227.03C	SPAC227.03c	0.7859	1.221	0.104632711	39.93	54.31	4.852	2.519
SPCC1259.04	iec3	0.839	1.223	0.076238039	38.05	51.67	7.076	2.617
SPBPJ4664.01	dps1	0.9002	1.224	0.045660991	18.71	24.54	8.312	7.231
SPAC13F5.03C	gld1	0.9323	1.224	0.030444316	28.08	37.69	13.72	9.755
SPBC800.10C	SPBC800.10c	0.8086	1.236	0.092266263	33.56	45.36	5.643	2.719
SPBC8E4.05C	SPBC8E4.05c	0.8386	1.239	0.076445142	35.18	47.63	7.356	2.251
SPAC25H1.09	mde5	0.7044	1.24	0.152180653	35.06	47.45	3.877	1.309
SPCC962.04	rps1201	0.9083	1.241	0.041770686	21.94	29.05	10	7.471
SPBC2G2.17C	SPBC2G2.17c	0.8408	1.242	0.075307297	38.45	52.21	7.243	2.736
SPCC31H12.03C	SPCC31H12.03c	0.8662	1.242	0.062381821	33.5	45.26	7.776	4.351
SPCC24B10.03	SPCC24B10.03	0.765	1.245	0.116338565	39.48	53.65	4.701	2.083
SPAC16C9.05	cph1	0.9239	1.245	0.034375033	26.74	35.77	11.94	9.138
SPBC1289.11	spf38	0.7653	1.249	0.116168287	39.16	53.19	4.662	2.169
SPAC1952.11C	ure2	0.7746	1.25	0.110922507	36.45	49.38	4.554	2.61
SPAC4F10.18	nup37	0.9352	1.252	0.029095502	30.95	41.67	14.52	10.55
SPBC29B5.03C	rpl26	0.7813	1.254	0.107182176	38.68	52.51	4.968	2.429
SPAC20H4.02	dsc3	0.8787	1.255	0.056159374	10.27	12.65	7.399	5.855
SPBC21.05C	ral2	0.8442	1.259	0.073554652	29.8	40.05	6.896	3.919
SPBP8B7.09C	los1	0.9232	1.266	0.034704204	23.61	31.35	12.39	8.978
SPBC3D6.04C	mad1	0.9175	1.268	0.037393927	25.15	33.51	11.43	8.466
SPCC1020.13C	SPCC1020.13c	0.7434	1.27	0.128777443	31.1	41.86	4.411	1.886
SPAC10F6.04	SPAC10F6.04	0.8436	1.27	0.073863429	35.73	48.35	7.663	2.644
SPAC664.10	klp2	0.8752	1.275	0.057892691	32	43.11	8.521	4.844
SPAC630.11	vps55	0.7716	1.281	0.112607781	38.55	52.29	4.987	2.197
SPCC553.08C	SPCC553.08c	0.927	1.283	0.032920266	30.64	41.19	11.69	10.5
SPBC3B8.04C	SPBC3B8.04c	0.7417	1.286	0.129771721	39.22	53.22	4.497	1.803
SPAC17C9.07	alg8	0.5832	1.287	0.234182485	30.48	40.97	2.206	1.526
SPBC17G9.07	rps2402	0.6587	1.293	0.181312337	35.91	48.57	1.741	2.438
SPCC1259.14C	meu27	0.772	1.298	0.1123827	35.57	48.08	5.418	1.62
SPCC11E10.01	SPCC11E10.01	0.8766	1.306	0.057198534	29.42	39.45	8.94	4.898
SPAC688.03C	SPAC688.03c	0.759	1.307	0.119758224	35.19	47.55	4.58	2.4
SPBC12C2.12C	glo1	0.7631	1.314	0.117418546	34.05	45.94	2.997	3.423
SPAC27D7.12C	but1	0.854	1.314	0.068542129	31.02	41.68	8.294	3.272
SPBC4C3.06	syp1	0.6384	1.32	0.194907122	39.35	53.35	3.28	1.184
SPAP27G11.16	SPAP27G11.16	0.8902	1.322	0.05051241	42.64	57.97	11.82	3.04
SPBC14C8.16C	bot1	0.9143	1.323	0.03891128	24.73	32.85	11.51	8.474
SPAC17C9.12	SPAC17C9.12	0.7494	1.325	0.125286311	25.85	34.41	3.647	2.985
SPBC1773.14	arg7	0.8192	1.327	0.086610056	33.94	45.76	7.173	1.78
SPCC18B5.03	wee1	0.5648	1.33	0.248105312	23.42	30.99	2.409	1.337
SPAC823.15	ppa1	0.8111	1.345	0.090925599	37.23	50.34	6.583	2.493
SPAC19A8.01C	sec73	0.6814	1.347	0.166597871	30.17	40.44	3.518	1.841
SPAC11E3.13C	gas5	0.8759	1.348	0.057545474	25.89	34.43	9.42	4.771
SPAC3A11.02	cps3	0.9299	1.349	0.031563752	24.46	32.42	14.83	10.22
SPBC3B8.03	SPBC3B8.03	0.6242	1.35	0.204676236	37.98	51.4	2.565	1.869
SPBC19C2.13C	ctu2	0.7682	1.353	0.114525697	32.29	43.41	5.463	1.844
SPBC354.03	swd3	0.6637	1.354	0.178028182	36.66	49.54	3.624	1.349
SPBC18E5.14C	SPBC18E5.14c	0.8994	1.354	0.046047117	20.41	26.74	9.941	7.427
SPBC1709.09	rrf1	0.8662	1.36	0.062381821	20.12	26.32	7.597	5.518
SPBC16C6.06	vps10	0.7763	1.365	0.109970414	30.44	40.8	5.512	2.28
SPCC11E10.08	rik1	0.9034	1.367	0.044119914	23.32	30.8	10.35	7.888
SPBC2D10.13	est1	0.9075	1.368	0.042153366	24.17	31.99	10.85	8.217
SPBC3F6.01C	SPBC3F6.01c	0.6201	1.369	0.207538269	36.66	49.52	2.024	2.165
SPCC320.08	SPCC320.08	0.8141	1.369	0.089322245	34.27	46.16	6.669	2.801
SPAC1610.04	mug99	0.9274	1.38	0.032732908	29.99	40.15	14.22	10.41

SPAC3G6.05	SPAC3G6.05	0.8611	1.386	0.064946411	31.25	41.9	8.556	4.475
SPAC18G6.02C	chp1	0.5878	1.394	0.230770418	23.6	31.15	1.518	2.13
SPBC4B4.07C	usp102	0.7552	1.394	0.121938019	20.68	27.05	3.754	3.318
SPAC11H11.02C	mug162	0.8073	1.394	0.092965048	35.68	48.1	6.756	2.408
SPAC17H9.01	cid16	0.7412	1.397	0.130064589	36.94	49.88	4.793	2.083
SPBC776.11	rpl2801	0.8843	1.397	0.053400375	20.43	26.7	9.471	6.253
SPCC16C4.20C	SPCC16C4.20c	0.7048	1.398	0.151934105	34.8	46.87	3.668	2.351
SPBC839.04	rpl803	0.7109	1.399	0.148191486	33.93	45.65	4.337	1.756
SPBC19C2.10	SPBC19C2.10	0.688	1.403	0.162411562	36.08	48.66	4.017	1.61
SPAC1296.04	mug65	0.8418	1.403	0.074791079	34.35	46.23	8.376	2.86
SPAC9.13C	cwf16	0.9222	1.405	0.035174882	28.27	37.7	13.61	9.811
SPAC3G9.08	png1	0.8568	1.407	0.067120542	17.53	22.62	7.725	5.042
SPCC550.03C	SPCC550.03c	0.8603	1.409	0.065350077	28.45	37.94	7.843	5.248
SPBC1289.13C	SPBC1289.13c	0.8896	1.409	0.050805226	29.28	39.1	10.09	6.561
SPAC2F3.07C	SPAC2F3.07c	0.7483	1.413	0.125924255	38.23	51.66	5.186	1.855
SPBC12D12.09	rev7	0.762	1.416	0.118045029	33.86	45.53	5.258	2.363
SPCC74.02C	SPCC74.02c	0.9068	1.416	0.042488489	25.04	33.14	11.15	8.448
SPBC3D6.10	apn2	0.7511	1.419	0.124302238	28.61	38.16	4.755	2.577
SPBC83.11	SPBC83.11	0.8104	1.419	0.091300568	39.3	53.15	6.555	3.132
SPBC28E12.04	SPBC28E12.04	0.7247	1.42	0.139841739	33.47	44.98	3.856	2.689
SPAC22F3.03C	rdh54	0.7919	1.42	0.101329657	38.67	52.26	4.337	4.093
SPBC365.03C	rpl2101	0.7018	1.426	0.153786636	6.548	7.187	3.22	2.831
SPAC3H8.05C	mms1	0.8903	1.427	0.050463627	20.81	27.2	9.649	7.146
SPCC584.16C	SPCC584.16c	0.9088	1.429	0.041531682	29.3	39.11	10.97	9.041
SPAC22G7.05	SPAC22G7.05	0.8015	1.435	0.096096473	18.21	23.54	5.313	4.126
SPAC12B10.05	icp55	0.7895	1.437	0.102647866	39.94	54.03	6.261	2.426
SPBC6B1.03C	SPBC6B1.03c	0.7143	1.44	0.14611935	36.92	49.78	4.279	2.152
SPCC18.03	SPCC18.03	0.7852	1.443	0.105019709	36.29	48.89	6.149	2.399
SPAC1F3.03	SPAC1F3.03	0.7155	1.444	0.145390362	28.76	38.33	2.628	3.231
SPBC713.05	SPBC713.05	0.7624	1.449	0.117817112	34.84	46.85	4.985	2.875
SPAC1093.01	ppr5	0.7729	1.449	0.111876693	33.56	45.05	4.375	3.64
SPBC20F10.10	psl1	0.6911	1.453	0.160459107	31.95	42.8	3.866	2.112
SPCC23B6.02C	SPCC23B6.02c	0.7311	1.453	0.136023216	38.89	52.54	4.91	1.892
SPBP4H10.17C	SPBP4H10.17c	0.6415	1.457	0.192803339	35.85	48.25	3.221	1.879
SPAC31A2.06	atp25	0.675	1.458	0.170696227	38.73	52.3	3.396	2.255
SPBC577.05C	rec27	0.9165	1.458	0.037867531	17.02	21.84	12.65	9.83
SPAC16E8.17C	SPAC16E8.17c	0.8731	1.46	0.058936012	32	42.86	9.897	5.133
SPBC1539.10	nop16	0.9136	1.461	0.039243909	22.36	29.32	12.68	9.228
SPBC1861.07	SPBC1861.07	0.7486	1.465	0.125750177	34.65	46.57	4.924	2.563
SPAC140.04	SPAC140.04	0.7927	1.467	0.100891142	30.74	41.07	6.93	1.581
SPAC4C5.01	SPAC4C5.01	0.8366	1.467	0.07748214	31.27	41.82	8.048	3.562
SPAC15E1.07C	moa1	0.9143	1.471	0.03891128	22.43	29.41	13.1	9.198
SPBC2A9.02	SPBC2A9.02	0.6703	1.477	0.173730781	35.45	47.66	3.389	2.25
SPBC146.09C	lsd1	0.2976	1.481	0.526367073	29.14	38.81	1.214	0.9665
SPBC31E1.01C	atg2	0.6643	1.481	0.177635748	40.49	54.73	3.724	1.833
SPBP4H10.14C	SPBP4H10.14c	0.6663	1.481	0.176330187	36.47	49.1	4.005	1.474
SPAC27E2.02	SPAC27E2.02	0.4433	1.489	0.353302269	24.39	32.13	0.8556	1.634
SPAC3G9.03	rpl2301	0.8292	1.489	0.081340707	11.43	13.94	5.448	5.314
SPAC22E12.03C	SPAC22E12.03c	0.728	1.498	0.137868621	26.61	35.23	4.058	2.918
SPAC23G3.07C	snf30	0.7745	1.5	0.110978578	38.58	52.03	6.051	2.419
SPAC22F3.02	atf31	0.6734	1.501	0.171726888	38.85	52.4	4.105	1.615
SPAPB24D3.03	SPAPB24D3.03	0.7628	1.502	0.117589316	40.18	54.27	4.011	3.782
SPBC1861.05	SPBC1861.05	0.7719	1.509	0.112438959	9.093	10.64	4.322	3.891
SPAC1420.01C	SPAC1420.01c	0.7369	1.51	0.132591443	36.32	48.85	5.291	1.892
SPAC25B8.17	SPAC25B8.17	0.7396	1.512	0.131003098	39.22	52.91	5.439	1.758
SPBC409.17C	SPBC409.17c	0.8359	1.515	0.077845675	39.85	53.79	8.891	2.625
SPAC11E3.01C	swr1	0.6165	1.517	0.210066919	40.04	54.05	2.785	2.076
SPAC17A5.11	rec12	0.7119	1.517	0.147581007	37.7	50.77	4.064	2.636
SPBC19F8.08	rps401	0.9008	1.517	0.045371622	23.81	31.27	10.85	8.726
SPBC691.03C	apl3	0.637	1.519	0.195860568	36.22	48.69	2.25	2.567
SPAPB8E5.06C	rpl302	0.8051	1.525	0.094150173	33	44.16	6.735	3.408
SPAC56E4.06C	ggt2	0.8637	1.527	0.06363708	28.72	38.15	8.027	6.311
SPCC13B11.01	adh1	0.8021	1.529	0.095771484	31.07	41.45	6.33	3.685
SPBC32H8.09	SPBC32H8.09	0.8516	1.53	0.069764347	16.91	21.58	7.402	5.787
SPAC22G7.07C	SPAC22G7.07c	0.8961	1.53	0.047643523	27.81	36.88	13.48	5.602
SPAC3H1.08C	SPAC3H1.08c	0.6192	1.531	0.208169052	36.89	49.62	3.621	1.238
SPCC622.01C	SPCC622.01c	0.7412	1.532	0.130064589	37.3	50.19	4.633	2.946
SPBC12D12.06	srb11	0.7701	1.535	0.113452877	9.706	11.47	4.573	3.808
SPBC106.02C	srx1	0.696	1.536	0.15739076	32.65	43.65	4.612	1.658

SPAC637.11	rpm2	0.7507	1.536	0.124533584	31.81	42.48	5.262	2.653
SPBC14C8.15	SPBC14C8.15	0.6896	1.542	0.161402747	33.53	44.89	3.342	2.797
SPAC13G6.15C	SPAC13G6.15c	0.7837	1.547	0.105850153	37.16	49.97	5.531	3.672
SPCC1235.08C	pdh1	0.8244	1.549	0.083862017	33.03	44.17	8.072	3.249
SPAC1006.03C	red1	0.8531	1.549	0.069000058	18	23.08	7.919	5.685
SPBC902.02C	ctf18	0.7161	1.55	0.145026326	34.77	46.62	4.751	2.188
SPCC18B5.06	SPCC18B5.06	0.5917	1.552	0.227898431	32.61	43.58	3.359	1.222
SPBC21B10.07	SPBC21B10.07	0.8782	1.553	0.056406567	30.89	41.16	10.51	6.151
SPBC1706.03	fzo1	0.731	1.556	0.136082623	33.93	45.42	2.856	3.62
SPCC63.14	SPCC63.14	0.7803	1.557	0.107738393	38.5	51.84	6.017	3.14
SPBC800.12C	SPBC800.12c	0.7188	1.558	0.143391932	36.67	49.27	4.573	2.513
SPAC4G9.15	SPAC4G9.15	0.7244	1.558	0.140021558	12.03	14.69	4.692	2.545
SPAC1093.03	SPAC1093.03	0.7586	1.561	0.119987162	32.5	43.41	5.392	2.938
SPBP4H10.19C	SPBP4H10.19c	0.7329	1.565	0.134955278	37.01	49.74	4.544	2.941
SPAC19D5.07	uga1	0.764	1.566	0.116906641	37.14	49.91	5.507	3.051
SPAC19G12.02C	pms1	0.5268	1.569	0.278354234	32.35	43.19	2.245	1.701
SPAPJ695.01C	SPAPJ695.01c	0.9045	1.569	0.043591429	26.55	35.05	12.08	9.114
SPAC1F7.11C	SPAC1F7.11c	0.6544	1.57	0.184156709	37.44	50.33	3.545	2.17
SPAC23C11.04C	pnk1	0.7463	1.573	0.127086558	30.92	41.17	5.385	2.555
SPAC6F6.02C	pof5	0.7811	1.576	0.107293362	35.12	47.07	6.544	2.636
SPCC757.05C	SPCC757.05c	0.6798	1.578	0.16761884	33.49	44.77	4.129	2.111
SPAC806.04C	SPAC806.04c	0.7481	1.578	0.126040345	39.31	52.94	4.518	3.412
SPAC6B12.03C	SPAC6B12.03c	0.8364	1.588	0.077585976	29.76	39.52	8.484	4.133
SPAC12B10.10	SPAC12B10.10	0.7182	1.589	0.143754599	36.31	48.71	4.977	2.167
SPBC56F2.03	SPBC56F2.03	0.7551	1.59	0.12199553	39.01	52.5	5.791	2.491
SPAC4A8.14	SPAC4A8.14	0.8866	1.591	0.052272273	29.06	38.55	10.66	7.521
SPAC17G6.04C	cpp1	0.621	1.592	0.2069084	33.03	44.11	3.766	1.334
SPCC338.14	SPCC338.14	0.6578	1.592	0.181906131	37.14	49.88	3.402	2.408
SPCC965.10	SPCC965.10	0.7094	1.596	0.149108816	39.67	53.43	4.724	2.262
SPCC24B10.16C	SPCC24B10.16c	0.897	1.596	0.047207557	27.65	36.56	13.15	7.144
SPAC19E9.01C	nup40	0.7353	1.598	0.133535434	35.96	48.22	5.446	2.186
SPBC17A3.08	SPBC17A3.08	0.8094	1.6	0.0918368	36.37	48.79	7.491	3.334
SPBC1685.02C	rps1202	0.8784	1.601	0.056307673	21.78	28.32	9.557	7.362
SPAC688.04C	gst3	0.5933	1.61	0.226725652	36.89	49.5	2.609	2.163
SPBC3D6.02	but2	0.7097	1.613	0.148925195	39.47	53.11	4.64	2.448
SPAC19G12.06C	hta2	0.7469	1.613	0.126737541	38.88	52.29	5.565	2.593
SPAC23C11.08	php3	0.8115	1.613	0.090711476	38.86	52.26	7.986	2.861
SPAC19D5.03	cid1	0.7486	1.614	0.125750177	34.69	46.41	5.957	2.074
SPAC12G12.10	wdr21	0.8456	1.616	0.072835026	31.59	42.06	8.96	4.678
SPAC16A10.03C	SPAC16A10.03c	0.88	1.62	0.055517328	21.3	27.62	10	7.419
SPBC409.20C	psh3	0.8545	1.622	0.068287933	25.66	33.73	8.645	5.807
SPBC1921.03C	mex67	0.8635	1.626	0.063737658	23.93	31.3	9.935	5.62
SPAC6G10.12C	ace2	0.8461	1.628	0.072578305	16.95	21.5	7.759	5.828
SPAC23C4.17	SPAC23C4.17	0.5852	1.629	0.232695683	39.54	53.2	3.347	1.452
SPBC18H10.07	SPBC18H10.07	0.8753	1.629	0.057843072	21.18	27.44	9.348	7.384
SPCC895.09C	ucp12	0.8133	1.631	0.089749228	34.41	46	7.03	4.299
SPCC1682.08C	SPCC1682.08c	0.8878	1.631	0.051684859	27.13	35.78	12.51	6.511
SPCC1322.07C	mug150	0.6665	1.632	0.176199846	36.06	48.31	3.938	2.246
SPAC7D4.13C	SPAC7D4.13c	0.8366	1.633	0.07748214	31.21	41.5	8.549	4.462
SPAC26A3.11	SPAC26A3.11	0.6822	1.635	0.166088285	31.22	41.5	2.165	3.262
SPAC23C4.05C	SPAC23C4.05c	0.7919	1.637	0.101329657	33.56	44.8	7.012	3.103
SPAC23C4.16C	atg15	0.5681	1.64	0.245575211	38.06	51.1	3.405	1.051
SPCC965.05C	thp1	0.7921	1.64	0.101219987	34.7	46.38	7.373	2.581
SPCC338.05C	mms2	0.9055	1.642	0.043111545	25.2	33.05	12.81	9.611
SPAC9E9.13	wos2	0.741	1.644	0.130181792	36.82	49.36	5.251	2.902
SPBC582.08	SPBC582.08	0.7833	1.645	0.106071873	41.22	55.53	6.565	3.234
SPAC22G7.03	SPAC22G7.03	0.7604	1.646	0.118957892	37.12	49.78	6.334	2.324
SPAC922.04	SPAC922.04	0.8253	1.648	0.083388155	37.69	50.57	6.875	5.205
SPBC3B9.04	SPBC3B9.04	0.7689	1.65	0.114130139	34.02	45.41	6.199	2.988
SPAC9E9.03	leu2	0.7578	1.652	0.120445399	34.14	45.59	6.459	1.969
SPAC1A6.09C	lag1	0.8261	1.653	0.082967378	35.06	46.87	8.011	4.358
SPBC216.02	mcp5	0.6723	1.656	0.172436889	36.3	48.61	4.023	2.369
SPBC8D2.12C	SPBC8D2.12c	0.5836	1.657	0.233884717	37.66	50.52	2.762	2.076
SPAC14C4.04	B22918-2	0.5565	1.659	0.254534831	33.7	44.95	2.908	1.665
SPAC14C4.09	agn1	0.824	1.659	0.084072788	27.58	36.38	5.053	5.907
SPAC25H1.07	emc1	0.879	1.659	0.056011125	21.97	28.5	10.36	7.397
SPAC3H1.07	aru1	0.7641	1.662	0.1168498	37.08	49.7	6.259	2.756
SPCC1450.11C	cek1	0.7857	1.664	0.104743247	33.48	44.64	6.818	3.186
SPAC57A10.02	cdr2	0.8497	1.667	0.070734382	18.58	23.73	7.888	6.267

SPAC12B10.11	exg2	0.7184	1.668	0.143633676	33.38	44.5	5.037	2.527
SPAC2E1P5.03	SPAC2E1P5.03	0.1925	1.669	0.715569266	26.74	35.17	0.9799	0.8873
SPAC26F1.14C	aif1	0.7356	1.669	0.133358279	37.31	50.01	5.019	3.067
SPAC1071.07C	rps1502	0.8024	1.669	0.09560908	15.94	20.03	6.851	4.091
SPBC9B6.07	nop52	0.8493	1.672	0.070938876	12.75	15.54	7.731	6.362
SPCC757.04	SPCC757.04	0.6527	1.683	0.185286387	35.11	46.9	3.894	2.208
SPBC119.06	sco1	0.7736	1.683	0.111483539	9.649	11.18	5.077	4.255
SPAC1687.16C	erg31	0.7098	1.686	0.148864005	39.21	52.65	5.102	2.257
SPCC1672.04C	SPCC1672.04c	0.3118	1.689	0.506123889	22.46	29.14	1.113	1.276
SPCC285.09C	cgs2	0.688	1.691	0.162411562	32.6	43.37	4.753	2.074
SPCC126.09	SPCC126.09	0.7251	1.695	0.139602095	36.89	49.38	5.438	2.392
SPBC56F2.01	pof12	0.9111	1.695	0.040433953	18.59	23.71	13.75	10.75
SPCC4F11.04C	imt2	0.8882	1.699	0.051489231	17.77	22.55	11.06	8.489
SPBC20F10.03	SPBC20F10.03	0.7243	1.7	0.140081515	36.44	48.75	5.152	2.738
SPBP19A11.02C	SPBP19A11.02c	0.793	1.703	0.100726813	38.74	51.97	7.606	2.835
SPAPB2B4.02	grx5	0.8786	1.703	0.056208801	28.65	37.81	11.88	6.462
SPBC25H2.15	SPBC25H2.15	0.6974	1.707	0.156518057	9.446	10.86	3.717	3.228
SPAC8C9.03	cgs1	0.7566	1.713	0.121133663	17.24	21.78	5.262	3.909
SPBC19F8.01C	spn7	0.8675	1.713	0.061730517	27.67	36.43	9.819	6.921
SPAC16A10.05C	dad1	0.7076	1.714	0.150212176	11.25	13.38	4.281	3.275
SPBC2D10.06	rep1	0.7175	1.714	0.144178095	32.09	42.62	5.029	2.724
SPBC1A4.09	SPBC1A4.09	0.7966	1.715	0.098759698	9.498	10.92	6.254	4.768
SPBC21B10.03C	SPBC21B10.03c	0.6651	1.717	0.177113052	24.42	31.86	4.851	1.186
SPAC15E1.05C	SPAC15E1.05c	0.8961	1.723	0.047643523	27.14	35.67	12.35	9.084
SPBC16D10.11C	rps1801	0.8664	1.726	0.062281556	21.39	27.59	9.355	7.219
SPAC4A8.05C	myp2	0.7925	1.727	0.101000729	33.46	44.53	7.41	3.295
SPAC186.03	SPAC186.03	0.6637	1.728	0.178028182	37.41	50.06	3.998	2.481
SPAC17A5.04C	mde10	0.8083	1.728	0.092427421	26.15	34.26	6.032	5.273
SPBC29B5.02C	isp4	0.5963	1.736	0.224535191	36.43	48.69	3.785	1.426
SPCC736.07C	SPCC736.07c	0.8516	1.737	0.069764347	19.35	24.72	8.407	6.568
SPBC3B9.13C	rpp102	0.8628	1.739	0.064089864	20.15	25.83	9.339	6.972
SPBC428.06C	rxt2	0.8928	1.739	0.049245818	22.8	29.55	12.4	8.658
SPAC1071.04C	spc2	0.8938	1.741	0.04875965	17.36	21.92	12.75	8.577
SPAC4H3.04C	SPAC4H3.04c	0.8008	1.742	0.096475936	32.58	43.27	7.896	3.325
SPAPB2B4.03	cig2	0.6249	1.747	0.204189475	36.02	48.09	3.872	1.938
SPAC22F3.09C	res2	0.7478	1.754	0.126214539	17.46	22.04	5.133	3.898
SPAC1D4.11C	lkh1	0.6205	1.755	0.207258214	37.95	50.78	3.867	1.888
SPAC683.03	SPAC683.03	0.631	1.755	0.199970641	42.22	56.77	3.93	2.018
SPBC1347.13C	SPBC1347.13c	0.8955	1.756	0.04793441	27.05	35.49	12.24	9.376
SPCC777.02	SPCC777.02	0.6739	1.758	0.171404544	38.14	51.04	4.301	2.522
SPAC26A3.09C	rga2	0.9025	1.761	0.044552789	28.94	38.14	13.35	9.977
SPBC1604.20C	tea2	0.6446	1.763	0.190709699	31.41	41.6	4.414	1.703
SPAC186.09	SPAC186.09	0.7574	1.768	0.120674699	37.59	50.26	6.233	3.139
SPBC2D10.19C	SPBC2D10.19c	0.8505	1.77	0.070325682	33.56	44.61	9.51	5.9
SPBC342.04	rpn1301	0.6521	1.773	0.1856858	38.5	51.54	3.063	3.012
SPCPB16A4.06C	SPCPB16A4.06c	0.7196	1.775	0.142908845	36.26	48.39	5.096	2.997
SPBC16A3.07C	nrm1	0.8539	1.778	0.068592986	18.23	23.09	8.799	6.797
SPAC26H5.04	SPAC26H5.04	0.7437	1.779	0.128602219	39.94	53.55	5.506	3.395
SPBC29A3.13	pdp1	0.7484	1.78	0.125866221	34.91	46.49	6.38	2.599
SPAC1783.08C	rpl1502	0.8938	1.78	0.04875965	16.3	20.37	12.02	9.476
SPAC4F10.04	SPAC4F10.04	0.778	1.784	0.109020403	22.8	29.48	7.146	3.169
SPAC22G7.11C	SPAC22G7.11c	0.8917	1.784	0.049781233	26.21	34.28	12	9.201
SPBPB2B2.13	gal1	0.8675	1.786	0.061730517	24.3	31.59	11.47	6.143
SPCC126.15C	sec65	0.8887	1.788	0.05124482	18.99	24.14	11.59	9.043
SPAC1805.10	SPAC1805.10	0.6704	1.79	0.173665994	36.65	48.91	4.64	2.203
SPAC750.06C	SPAC750.06c	0.7003	1.79	0.154715874	35.08	46.71	5.2	2.363
SPAC1D4.13	byr1	0.7961	1.79	0.099032376	29.65	39.09	6.793	4.772
SPAC17C9.10	stm1	0.6718	1.792	0.17276	37.81	50.54	4.687	2.188
SPBC32H8.08C	omh5	0.8858	1.792	0.052664324	25.06	32.64	11.6	8.643
SPBC557.04	ppk29	0.6092	1.793	0.215240105	39.24	52.54	3.96	1.681
SPBC577.12	mug71	0.6157	1.796	0.210630846	35.94	47.91	2.72	2.76
SPAC29A4.16	hal4	0.8454	1.797	0.072937757	38.52	51.52	11.1	3.532
SPCC126.11C	SPCC126.11c	0.8375	1.8	0.077015184	40.35	54.09	10.58	3.329
SPAC29B12.05C	SPAC29B12.05c	0.6658	1.803	0.176656209	14.99	18.5	4.029	2.908
SPAC3H8.08C	SPAC3H8.08c	0.7025	1.806	0.153353671	37.27	49.76	2.989	3.9
SPCC1739.08C	SPCC1739.08c	0.5325	1.809	0.273680388	38.27	51.16	2.751	1.903
SPAC4G9.11C	cmb1	0.6274	1.81	0.202455486	34.26	45.53	4.519	1.192
SPCC74.09	mug24	0.7035	1.81	0.152735898	32.99	43.75	5.45	2.227
SPAC1F7.12	yak3	0.6805	1.812	0.16717187	36.87	49.19	3.945	3.103

SPBC317.01	mbx2	0.6022	1.813	0.220259249	27.35	35.83	1.25	2.901
SPAC29A4.20	elp3	0.723	1.816	0.140861703	31.7	41.93	5.39	2.999
SPAC10F6.08C	nht1	0.6074	1.82	0.216525212	35.15	46.76	3.769	1.99
SPBC15D4.05	SPBC15D4.05	0.6023	1.823	0.220187137	37.73	50.38	3.906	1.737
SPBC776.17	SPBC776.17	0.8332	1.823	0.079250739	18.25	23.04	7.782	6.156
SPBC23G7.10C	SPBC23G7.10c	0.7191	1.825	0.143210711	39.57	52.96	5.549	2.74
SPAC3A12.06C	SPAC3A12.06c	0.7405	1.825	0.130474937	38.4	51.33	6.254	2.698
SPBC336.06C	rnh1	0.6693	1.828	0.174379175	37.28	49.74	4.895	1.999
SPBC725.02	mpr1	0.872	1.83	0.059483515	22.87	29.53	10.57	7.858
SPBC649.02	rps1902	0.8894	1.83	0.050902875	26.14	34.12	12.07	9.226
SPBC29A10.03C	pcf1	0.6726	1.831	0.172243137	36.72	48.96	4.988	1.973
SPBC1861.03	mak10	0.7087	1.831	0.149537567	33.65	44.64	5.499	2.469
SPBC2F12.03C	SPBC2F12.03c	0.5547	1.834	0.255941834	35.46	47.19	3.285	1.741
SPCC1919.11	mug137	0.7455	1.834	0.127552352	39.64	53.05	6.373	2.82
SPCC1393.05	ers1	0.6776	1.843	0.169026603	33.19	43.99	5.026	2.145
SPAC6C3.02C	SPAC6C3.02c	0.6905	1.843	0.160836317	39.45	52.76	5.207	2.303
SPAC26A3.17C	SPAC26A3.17c	0.6051	1.845	0.218172847	38.75	51.78	2.852	2.687
SPAC1F7.10	SPAC1F7.10	0.747	1.847	0.126679398	36.23	48.24	7.009	1.844
SPCC1183.09C	pmp31	0.6236	1.849	0.205093893	37.92	50.61	4.525	1.306
SPBC216.03	SPBC216.03	0.8881	1.851	0.05153813	19.25	24.41	11.92	9.313
SPBC947.09	SPBC947.09	0.6997	1.855	0.155088126	40.87	54.74	5.145	2.719
SPCC1442.13C	SPCC1442.13c	0.8032	1.855	0.0951763	18.15	22.87	7.654	4.559
SPCC364.01	cif1	0.7727	1.856	0.111989088	37.08	49.43	7.121	3.387
SPCC1753.05	rsm1	0.8793	1.856	0.055862927	26.82	35.02	12.04	7.968
SPAP27G11.06C	vas2	0.8403	1.863	0.075565636	18.83	23.81	8.328	6.563
SPCC576.11	rpl15	0.8281	1.865	0.081917215	12.83	15.38	7.358	6.315
SPAC1002.02	pom34	0.7347	1.867	0.13388996	34.5	45.78	5.964	3.055
SPAC20G8.10C	atg6	0.5451	1.87	0.263523818	34.88	46.32	3.444	1.511
SPBC21C3.07C	SPBC21C3.07c	0.7465	1.871	0.126970188	36.56	48.67	4.54	4.45
SPCC613.10	qcr2	0.8	1.872	0.096910013	17.89	22.47	6.761	5.169
SPBP8B7.11	nxt3	0.6279	1.875	0.202109517	34.29	45.48	2.368	3.073
SPBC1861.06C	mug131	0.7388	1.881	0.131473113	38.46	51.33	5.513	3.682
SPAC227.07C	pab1	0.5837	1.884	0.233810307	13.46	16.24	3.31	2.39
SPAC20H4.04	fml2	0.6504	1.885	0.186819467	39.94	53.4	4.821	1.826
SPAC12B10.09	SPAC12B10.09	0.7541	1.887	0.122571059	29.78	39.14	5.294	4.34
SPAC6G9.08	ubp6	0.623	1.89	0.205511953	37.77	50.35	3.17	2.853
SPCC965.08C	alr1	0.5827	1.896	0.234554982	33	43.64	3.336	2.238
SPBC2G2.03C	sbh1	0.8899	1.896	0.050658793	16.92	21.08	12.28	9.775
SPAC19D5.11C	ctf8	0.6887	1.898	0.161969917	34.06	45.13	5.677	1.758
SPAC1F7.01C	spt6	0.5869	1.909	0.23143589	35.08	46.54	3.716	1.991
SPCC330.14C	rpl2402	0.8665	1.909	0.062231433	28.22	36.92	11.89	6.795
SPCC1753.03C	rec7	0.6964	1.911	0.157141238	37.67	50.17	5.183	2.829
SPBC16D10.08C	SPBC16D10.08c	0.644	1.912	0.191114133	37.46	49.87	4.621	2.079
SPBC660.14	mik1	0.8591	1.914	0.065956281	34.94	46.34	10.92	6.777
SPCC417.11C	SPCC417.11c	0.801	1.916	0.096367484	34.5	45.72	8.184	4.298
SPBC1347.02	fkbp39	0.7684	1.924	0.114412644	29.66	38.91	6.882	3.837
SPBC3H7.03C	SPBC3H7.03c	0.8343	1.926	0.078677756	12.81	15.28	7.921	6.753
SPAC823.14	ptf1	0.6419	1.927	0.192532624	40.63	54.3	3.997	2.716
SPAC1952.17C	SPAC1952.17c	0.8855	1.934	0.052811434	26.76	34.83	12.51	9.277
SPAC1805.04	nup132	0.7161	1.937	0.145026326	32.79	43.28	5.312	3.381
SPAP32A8.03C	SPAP32A8.03c	0.6006	1.942	0.221414672	34.29	45.39	4.063	1.945
SPAC27E2.09	mak2	0.7636	1.945	0.11713408	35.17	46.62	7.071	3.521
SPBC1861.09	ppk22	0.7947	1.945	0.099796787	33.54	44.33	6.508	5.431
SPCC16C4.11	pef1	0.6103	1.947	0.21445663	29.58	38.77	4.02	2.195
SPCC663.04	rpl39	0.6126	1.949	0.212823008	5.365	4.794	2.97	2.949
SPAC6G10.11C	ubi3	0.6542	1.951	0.18428946	39.53	52.73	4.694	2.404
SPAC11E3.10	SPAC11E3.10	0.7395	1.952	0.131061822	38.3	51	6.153	3.466
SPAC22A12.03C	csn4	0.6657	1.953	0.176721443	38.68	51.53	4.797	2.582
SPBC336.03	efc25	0.6417	1.955	0.192667961	36.81	48.9	3.988	2.803
SPAPB1E7.08C	SPAPB1E7.08c	0.6875	1.956	0.162727297	37.48	49.85	5.328	2.599
SPAPB1A11.04C	SPAPB1A11.04c	0.6234	1.957	0.205233202	40.83	54.54	4.106	2.383
SPAC19B12.11C	SPAC19B12.11c	0.5413	1.962	0.266561973	30.47	39.99	3.169	2.017
SPAC17G8.07	yaf9	0.6367	1.965	0.19606515	34.69	45.91	4.527	2.238
SPBP8B7.25	cyp4	0.7176	1.967	0.14411757	36.88	48.98	5.596	3.301
SPBC3H7.10	elp6	0.7014	1.974	0.154034238	21.27	27.08	4.428	3.748
SPAC22E12.06C	gmh3	0.68	1.976	0.167491087	37.56	49.93	5.74	1.769
SPBC32F12.05C	cwf12	0.8354	1.978	0.078105529	19.07	23.99	8.616	6.735
SPAC1093.02	SPAC1093.02	0.7503	1.979	0.124765054	38.59	51.36	7.092	2.999
SPBC13A2.04C	SPBC13A2.04c	0.8435	1.989	0.073914913	29.91	39.18	11.12	5.413

SPCPB16A4.05C	SPCPB16A4.05c	0.6963	1.992	0.157203605	34.32	45.35	5.913	2.283
SPBPJ4664.05	SPBPJ4664.05	0.734	1.992	0.13430394	40.52	54.06	6.568	2.98
SPCC550.07	SPCC550.07	0.7399	1.993	0.130826973	28.22	36.8	6.846	2.891
SPAC17G6.17	pof8	0.843	1.993	0.074172425	24.23	31.19	9.955	6.515
SPAC8E11.07C	alp31	0.8461	1.995	0.072578305	25.22	32.59	8.099	7.925
SPBC887.08	SPBC887.08	0.5569	2.001	0.254222782	32.02	42.12	2.897	2.461
SPAC1002.05C	jmj2	0.6287	2.006	0.20155654	41.92	56	4.825	1.775
SPBP4H10.09	rsv1	0.8877	2.006	0.05173378	28.71	37.48	13.3	9.781
SPAC22G7.06C	ura1	0.6962	2.007	0.157265981	35.21	46.59	5.19	3.204
SPBC6B1.10	prp17	0.8005	2.008	0.096638664	38.79	51.61	9.444	3.242
SPAC1296.01C	SPAC1296.01c	0.3521	2.009	0.453333975	41.63	55.59	0.9707	1.772
SPBC1539.04	tts1	0.6841	2.009	0.16488041	35.27	46.67	5.464	2.573
SPAC13A11.03	mcp7	0.7176	2.011	0.14411757	38.04	50.55	6.145	2.924
SPAC22F3.07C	atp20	0.76	2.012	0.119186408	34.87	46.1	6.926	3.88
SPAC5H10.07	SPAC5H10.07	0.6611	2.028	0.179732843	38.74	51.51	3.719	3.492
SPBC428.14	SPBC428.14	0.6824	2.028	0.165960982	40.96	54.63	4.744	3.29
SPAC29B12.14C	SPAC29B12.14c	0.8025	2.028	0.095554959	34.59	45.69	7.44	5.66
SPCC1840.02C	bgs4	0.6995	2.029	0.155212281	38.15	50.68	5.923	2.609
SPAC25G10.09C	pan1	0.6116	2.031	0.213532523	30.99	40.63	4.892	1.117
SPBC2D10.05	exg3	0.7131	2.032	0.146849564	37.33	49.53	6.394	2.502
SPAC18G6.10	lem2	0.5617	2.038	0.250495576	35.84	47.43	3.118	2.463
SPBP4H10.18C	SPBP4H10.18c	0.6787	2.038	0.168322151	38.47	51.12	5.341	2.684
SPCP31B10.05	tdp1	0.8623	2.039	0.064341614	28.58	37.24	12.13	7.193
SPBC56F2.02	rpl1901	0.6087	2.041	0.215596698	7.686	7.921	3.577	2.789
SPAC11D3.05	mfs2	0.6735	2.043	0.1716624	35.33	46.71	5.734	1.941
SPCC622.12C	gdh1	0.873	2.046	0.058985756	26.4	34.18	12.56	8.375
SPAC14C4.05C	man1	0.5611	2.047	0.250959731	31.1	40.76	2.293	2.733
SPAC15E1.10	SPAC15E1.10	0.8834	2.047	0.053842605	29.04	37.88	12.89	9.722
SPAC17G6.06	rps2401	0.8044	2.048	0.094527938	30.95	40.56	8.809	4.777
SPCC417.05C	chr2	0.8806	2.049	0.055221319	26.04	33.66	13.92	8.499
SPAC13G6.10C	asl1	0.7464	2.051	0.127028369	15.22	18.48	6.239	4.107
SPAC20H4.07	rhp57	0.6785	2.052	0.168450148	28.21	36.71	5.211	2.88
SPCC663.10	SPCC663.10	0.5893	2.053	0.229663559	30.49	39.9	3.62	2.446
SPAC1486.02C	dsc2	0.8072	2.055	0.093018847	18.57	23.17	7.564	5.978
SPAC140.01	sdh2	0.8818	2.055	0.054629906	21.84	27.76	12.89	9.548
SPAC9.11	SPAC9.11	0.5911	2.063	0.228339041	29.91	39.07	4.563	1.41
SPCC970.10C	brl2	0.84	2.065	0.075720714	21.21	26.87	9.325	7.194
SPBC1105.05	exg1	0.6451	2.072	0.190372958	38.45	51.05	5.192	2.011
SPBC15C4.02	SPBC15C4.02	0.7886	2.073	0.103143227	40.16	53.44	8.839	3.722
SPAPB1A10.03	nxt1	0.7147	2.075	0.145876218	35.48	46.88	6.338	2.905
SPBC646.17C	dic1	0.7304	2.075	0.136439235	37.65	49.91	6.753	3.048
SPAC16E8.14C	tae1	0.6779	2.081	0.168834366	35.96	47.53	5.485	2.678
SPAC13D6.03C	trm9	0.5894	2.082	0.229589868	37.42	49.58	4.157	2.098
SPCC13B11.04C	SPCC13B11.04c	0.6282	2.096	0.201902068	33.72	44.37	5.206	1.488
SPAC23H4.10C	thi4	0.7956	2.096	0.099305226	33.17	43.6	2.1	7.016
SPAC323.07C	SPAC323.07c	0.8074	2.097	0.092911255	27.34	35.42	8.556	5.837
SPBC2F12.09C	atf21	0.8725	2.097	0.059234564	25.98	33.52	13.29	8.163
SPCC320.14	SPCC320.14	0.6974	2.099	0.156518057	35.68	47.12	6.191	2.517
SPBC29A3.12	rps902	0.852	2.103	0.069560405	27.52	35.66	9.459	8.408
SPAC4G8.06C	trm12	0.5004	2.105	0.300682699	37.2	49.24	3.562	1.36
SPAC3H8.07C	pac10	0.5208	2.105	0.283329024	38.33	50.83	3.406	1.889
SPBC16H5.05C	cyp7	0.6204	2.105	0.207328211	35.27	46.54	4.273	2.637
SPAC17A2.07C	SPAC17A2.07c	0.6767	2.106	0.169603824	38.05	50.43	5.546	2.68
SPAC17G8.11C	imt3	0.3738	2.108	0.427360703	35.54	46.91	1.64	1.731
SPBC16E9.18	psd1	0.8721	2.109	0.059433714	29.72	38.74	12.52	8.829
SPAC212.02	SPAC212.02	0.6395	2.11	0.194159451	37.72	49.96	5.412	1.579
SPAP27G11.12	SPAP27G11.12	0.6764	2.112	0.169796401	38.2	50.64	5.047	3.198
SPBC215.14C	vps20	0.6881	2.112	0.162348442	37.82	50.11	5.555	3.045
SPBC32H8.06	mug93	0.7661	2.115	0.115714538	35.1	46.28	7.512	4.151
SPCPJ732.01	vps5	0.8549	2.115	0.068084683	21.68	27.45	9.718	8.617
SPAC23E2.03C	ste7	0.7812	2.134	0.107237765	31.29	40.91	6.95	5.678
SPAC12B10.04	SPAC12B10.04	0.8392	2.137	0.076134525	32.27	42.28	11.05	6.262
SPAC15E1.04	SPAC15E1.04	0.8552	2.138	0.067932308	21.5	27.17	10.81	8.168
SPBC32F12.02	rec14	0.6452	2.139	0.190305641	30.47	39.76	5.065	2.492
SPAC6G9.13C	bqt1	0.7656	2.14	0.115998075	41.88	55.76	8.096	3.598
SPAC1A6.06C	meu31	0.883	2.14	0.054039296	29.06	37.78	13.45	10.12
SPBC21C3.17C	SPBC21C3.17c	0.638	2.141	0.195179321	39.23	52.04	3.965	3.265
SPAC4D7.06C	SPAC4D7.06c	0.5294	2.142	0.276216063	28.62	37.15	1.739	2.864
SPBC947.14C	cbp6	0.7667	2.147	0.115374537	12.67	14.77	6.305	5.247

SPAC2F7.17	SPAC2F7.17	0.5029	2.15	0.298518364	39.66	52.63	3.738	1.243
SPBC8E4.04	SPBC8E4.04	0.75	2.15	0.124938737	39.91	52.98	7.871	2.98
SPAC1556.06.1	meu1-1	0.7111	2.151	0.148069321	34.43	45.29	6.109	3.398
SPCC1682.12C	ubp16	0.6109	2.152	0.214029875	37.09	49.03	4.889	1.846
SPAC6C3.04	cit1	0.613	2.152	0.212539525	38.97	51.67	4.993	1.723
SPCC338.10C	cox5	0.7205	2.156	0.142366015	13.85	16.4	5.786	4.003
SPBC725.05C	SPBC725.05c	0.5945	2.157	0.225848141	36.47	48.15	3.775	2.737
SPCC11E10.09C	SPCC11E10.09c	0.6683	2.16	0.175028539	35.91	47.36	4.542	3.536
SPAC32A11.02C	SPAC32A11.02c	0.6253	2.161	0.203911571	32.76	42.93	4.953	2.197
SPBC17D1.06	dbp3	0.6482	2.163	0.188290973	32.99	43.26	4.99	2.745
SPBC557.05	SPBC557.05	0.5736	2.165	0.241390857	38.21	50.58	4.016	2.226
SPAC15A10.11	ubr11	0.5929	2.165	0.22701855	27.78	35.94	4.828	1.447
SPAC1805.08	dlc1	0.5953	2.173	0.225264117	38.54	51.03	4.603	1.978
SPBC26H8.03	cho2	0.887	2.173	0.05207638	31.02	40.48	14.06	10.7
SPBC83.16C	SPBC83.16c	0.6987	2.175	0.155709257	34.33	45.12	6.404	2.683
SPBC2F12.05C	SPBC2F12.05c	0.5945	2.178	0.225848141	35.34	46.53	4.695	1.834
SPAC26H5.08C	bgl2	0.8767	2.179	0.057148993	28.35	36.72	13.09	9.707
SPAC1782.02C	SPAC1782.02c	0.7425	2.183	0.129303542	40.69	54.02	7.505	3.304
SPBC31F10.02	SPBC31F10.02	0.3846	2.188	0.41499072	37.08	48.96	2.322	1.645
SPAC1834.08	mak1	0.4073	2.188	0.39008559	36.26	47.81	2.965	1.14
SPCC790.03	SPCC790.03	0.7542	2.191	0.122513472	14.42	17.15	6.195	5.012
SPAC3C7.12	tip1	0.7757	2.195	0.110306209	31.62	41.29	9.158	3.121
SPAC3H8.03	img2	0.6618	2.199	0.179273237	34.16	44.84	3.729	3.812
SPBC215.02	bob1	0.5288	2.2	0.276708554	36.69	48.39	4.085	1.35
SPBC902.05C	idh2	0.5768	2.201	0.238974748	35.33	46.49	4.469	1.842
SPACUNK4.13C	SPACUNK4.13c	0.6344	2.202	0.197636826	37.38	49.36	5.073	2.449
SPBC1105.13C	SPBC1105.13c	0.5642	2.203	0.248566918	36.06	47.51	4.078	2.11
SPBC902.03	SPBC902.03	0.6054	2.207	0.217957583	39.89	52.87	4.78	2.108
SPBC409.16C	SPBC409.16c	0.6995	2.212	0.155212281	33.41	43.77	6.301	3.046
SPAC6G9.01C	SPAC6G9.01c	0.4885	2.219	0.311135432	31.03	40.43	2.328	2.475
SPBC30B4.04C	sol1	0.2297	2.223	0.638839005	33.66	44.12	1.515	1.188
SPAC30C2.08	SPAC30C2.08	0.6037	2.225	0.219178824	36.3	47.81	4.787	2.125
SPAC14C4.10C	SPAC14C4.10c	0.62	2.229	0.207608311	38.99	51.57	5.195	1.974
SPCC736.08	cbf11	0.3098	2.23	0.508918587	31.31	40.8	1.771	1.554
SPBC530.07C	SPBC530.07c	0.7103	2.235	0.148558185	14.13	16.69	5.787	3.989
SPCC1450.12	SPCC1450.12	0.8846	2.237	0.053253065	31.27	40.74	14.24	10.73
SPAC6F6.13C	SPAC6F6.13c	0.7219	2.24	0.141522958	41.45	55.01	6.427	3.87
SPAC8C9.10C	SPAC8C9.10c	0.8712	2.247	0.059882133	28.58	36.96	12.49	9.844
SPBC1271.08C	SPBC1271.08c	0.6063	2.248	0.217312432	38.84	51.34	4.649	2.441
SPBC28F2.07	sfr1	0.5411	2.249	0.266722466	38.81	51.29	3.165	2.633
SPAC4G9.13C	vps26	0.6587	2.252	0.181312337	16.91	20.56	4.621	3.747
SPBC1D7.04	mlo3	0.7934	2.252	0.100507804	17.88	21.93	7.563	6.203
SPBC28F2.05C	SPBC28F2.05c	0.6166	2.263	0.20999648	35.2	46.21	5.577	1.092
SPBC428.03C	pho4	0.6083	2.266	0.215882184	36.55	48.1	4.969	2.155
SPBC2F12.11C	rep2	0.5838	2.268	0.233735909	9.065	9.536	3.201	3.16
SPAC22F8.09	rrp16	0.6063	2.268	0.217312432	33.64	44.01	4.529	2.631
SPCC613.03	SPCC613.03	0.7763	2.268	0.109970414	36.67	48.27	8.566	4.527
SPCC736.14	dis1	0.8757	2.272	0.05764465	30.38	39.44	13.26	10.21
SPAC4G8.05	ppk14	0.6681	2.274	0.175158528	28.92	37.38	5.881	2.736
SPAC1B3.02C	SPAC1B3.02c	0.7563	2.281	0.1213059	43.21	57.43	8.31	3.647
SPAC1952.02	SPAC1952.02	0.8423	2.282	0.074533199	24.71	31.47	10.66	7.926
SPAC23H3.09C	gly1	0.7754	2.286	0.110474203	16.89	20.49	7.17	5.699
SPBC582.04C	SPBC582.04c	0.5428	2.289	0.265360161	31.59	41.11	4.266	1.718
SPCC777.13	vps35	0.7176	2.291	0.14411757	13.75	16.08	5.375	4.659
SPAC11D3.02C	SPAC11D3.02c	0.6934	2.293	0.159016163	38.57	50.91	7.048	1.981
SPAC22A12.17C	SPAC22A12.17c	0.773	2.293	0.111820506	32.81	42.82	8.605	4.427
SPBC1A4.03C	top2	0.6934	2.299	0.159016163	37.37	49.21	6.458	3.038
SPAC15A10.09C	SPAC15A10.09c	0.7921	2.301	0.101219987	31.02	40.29	7.6	6.342
SPCC297.04C	set7	0.7362	2.304	0.133004187	39.67	52.43	7.489	3.691
SPAC589.12	SPAC589.12	0.8153	2.304	0.088682558	16.24	19.56	9.483	6.93
SPBC83.02C	rpl4302	0.8626	2.307	0.064190546	19.57	24.22	13	8.796
SPAC3G6.09C	tps2	0.6797	2.31	0.16768273	35.2	46.14	4.639	4.161
SPBC409.07C	wis1	0.771	2.31	0.112945622	30.38	39.38	7.734	5.208
SPBP4G3.02	pho1	0.5704	2.314	0.243820483	37.79	49.78	4.507	2.064
SPBC13G1.04C	SPBC13G1.04c	0.7373	2.316	0.132355766	37.29	49.07	6.959	4.332
SPBC11B10.07C	ivn1	0.3829	2.319	0.416914634	33.66	43.97	2.857	1.295
SPCC645.07	rgf1	0.6735	2.319	0.1716624	28.29	36.45	6.134	2.802
SPCC594.04C	SPCC594.04c	0.8639	2.32	0.063536526	29.31	37.88	12.9	9.155
SPCC4G3.04C	coq5	0.6796	2.322	0.16774663	10.88	12	4.63	4.196

SPBC31E1.02C	pmr1	0.5731	2.329	0.241769592	40.06	52.94	4.498	2.186
SPCC1827.02C	SPCC1827.02c	0.7332	2.332	0.134777544	14.44	16.98	5.999	4.929
SPCC1450.02	bdf1	0.8599	2.333	0.065552051	27.65	35.52	12.08	9.276
SPAC4A8.02C	SPAC4A8.02c	0.6317	2.334	0.199489123	40.46	53.5	5.537	2.296
SPBC216.01C	SPBC216.01c	0.6769	2.335	0.169475486	39.06	51.53	6.065	3.076
SPCC825.04C	naa40	0.6174	2.349	0.209433375	32.38	42.14	4.754	2.893
SPBC342.01C	alg6	0.8273	2.35	0.082336976	20.12	24.93	9.647	7.678
SPAC3A11.11C	SPAC3A11.11c	0.8419	2.35	0.07473949	28.1	36.13	11.39	7.84
SPAC24B11.08C	SPAC24B11.08c	0.6512	2.351	0.186285608	39.25	51.77	5.993	2.338
SPAC31G5.19	abo1	0.783	2.352	0.106238238	26.34	33.66	9.566	4.376
SPCC576.12C	mhf2	0.3216	2.355	0.49268396	31.77	41.28	1.629	1.806
SPAC57A10.07	SPAC57A10.07	0.4694	2.355	0.328456915	37.2	48.9	3.737	1.33
SPBC4B4.11	SPBC4B4.11	0.5924	2.355	0.22738495	36.99	48.6	4.925	2.157
SPAC5D6.01	rps2202	0.6322	2.356	0.199145508	33.77	44.07	5.013	3.003
SPAC8C9.14	prr1	0.8569	2.36	0.067069857	27.36	35.08	11.85	9.257
SPBC21H7.04	dbp7	0.2761	2.364	0.558933593	3.459	1.537	1.051	1.898
SPBC9B6.03	SPBC9B6.03	0.4512	2.369	0.345630909	38.18	50.25	2.819	2.146
SPBC18H10.15	ppk23	0.5938	2.369	0.226359807	37.27	48.97	4.574	2.642
SPBC23E6.05	arx1	0.689	2.369	0.161780778	38.1	50.13	6.723	2.85
SPCC364.07	SPCC364.07	0.7383	2.374	0.131767132	34.92	45.67	8.209	3.258
SPAC17C9.05C	pmc3	0.6894	2.378	0.161528721	38.43	50.59	5.647	3.987
SPAC10F6.11C	atg17	0.634	2.381	0.197910742	36.27	47.55	5.433	2.699
SPAC29A4.17C	SPAC29A4.17c	0.7075	2.381	0.150273556	30.55	39.53	7.474	2.64
SPCC1020.05	SPCC1020.05	0.721	2.383	0.142064735	38.24	50.31	6.072	4.666
SPBC713.08	mim1	0.7677	2.392	0.114808459	16.81	20.23	7.095	5.848
SPAC1D4.06C	csk1	0.8034	2.393	0.095068173	30.48	39.4	9.719	6.037
SPAPJ698.02C	rps002	0.5687	2.395	0.245116772	10.94	11.98	4.094	2.695
SPCC16C4.12	naa20	0.8704	2.396	0.060281118	28.53	36.67	14.17	9.791
SPAC16C9.02C	SPAC16C9.02c	0.6168	2.411	0.209855635	36.94	48.45	5.425	2.338
SPAC631.02	SPAC631.02	0.827	2.411	0.08249449	23.2	29.16	9.869	7.866
SPBCPT2R1.01C	SPBCPT2R1.01c	0.5823	2.414	0.23485321	33.79	44.02	4.804	2.292
SPBC609.03	iqw1	0.6081	2.414	0.216024997	37.2	48.81	3.214	3.772
SPBC1711.05	SPBC1711.05	0.5839	2.416	0.233661525	29.21	37.6	4.622	2.541
SPBC36B7.03	sec63	0.6068	2.417	0.216954428	41.5	54.84	5.041	2.591
SPAC26F1.08C	SPAC26F1.08c	0.435	2.418	0.361510743	36.48	47.79	3.525	1.284
SPAC5H10.10	SPAC5H10.10	0.591	2.418	0.228412519	38.87	51.15	4.908	2.378
SPAC3F10.06C	SPAC3F10.06c	0.6334	2.419	0.198321941	38.01	49.94	3.693	3.987
SPAPB1A11.02	SPAPB1A11.02	0.731	2.42	0.136082623	36.38	47.65	7.347	4.179
SPBC685.03	SPBC685.03	0.4584	2.421	0.338755391	33.53	43.65	2.624	2.31
SPBC646.15C	SPBC646.15c	0.604	2.421	0.218963061	36.83	48.28	4.72	2.865
SPCC188.02	par1	0.8079	2.422	0.092642392	21.23	26.39	8.922	7.092
SPBC11B10.08	SPBC11B10.08	0.683	2.424	0.165579296	40.4	53.29	5.447	4.107
SPAC22A12.10	SPAC22A12.10	0.7265	2.424	0.138764381	36.11	47.27	7.354	3.995
SPAC23A1.17	SPAC23A1.17	0.3085	2.425	0.510744832	33.58	43.72	2.112	1.506
SPBC11C11.07	rpl1801	0.5846	2.428	0.233141189	15.55	18.42	3.092	3.527
SPAC1565.07C	SPAC1565.07c	0.5323	2.429	0.273843534	36.54	47.86	3.702	2.546
SPCC777.15	SPCC777.15	0.6664	2.429	0.176265012	34.39	44.85	6.041	3.154
SPBC12C2.01C	SPBC12C2.01c	0.6249	2.43	0.204189475	18.21	22.14	6.021	1.59
SPAC1002.18	urg3	0.6345	2.43	0.197568374	32.43	42.09	2.756	4.151
SPAC9G1.08C	SPAC9G1.08c	0.8644	2.431	0.063285242	32.39	42.04	11.8	10.7
SPAC25B8.01	dap1	0.7943	2.437	0.100015437	39.24	51.64	10.77	4.371
SPAC2E1P3.01	SPAC2E1P3.01	0.6497	2.441	0.187287133	33.91	44.15	5.925	2.807
SPAC1805.06C	hem2	0.299	2.442	0.524328812	32.8	42.59	1.209	1.745
SPBC3H7.08C	SPBC3H7.08c	0.4297	2.442	0.366834646	35.9	46.95	2.825	2.065
SPAC589.10C	SPAC589.10c	0.8317	2.444	0.080033299	26.08	33.16	11.62	7.243
SPACUNK12.02C	cmk1	0.5847	2.445	0.233066906	39.33	51.75	4.573	2.693
SPAC959.07	rps403	0.7728	2.445	0.111932887	26.18	33.31	9.031	4.859
SPAC29A4.18	prw1	0.7479	2.446	0.126156467	31.67	41	6.779	5.679
SPBC29A10.02	spo5	0.2759	2.448	0.5592483	34.41	44.84	2.141	1.295
SPBCPT2R1.08C	tlh2	0.8618	2.449	0.06459351	23.39	29.38	12.95	9.811
SPAC3G6.11	chl1	0.8498	2.454	0.070683273	30.84	39.83	13.98	7.327
SPCC4B3.03C	SPCC4B3.03c	0.8677	2.454	0.061630403	28.62	36.71	13.48	10.34
SPAC1142.06	get3	0.3071	2.46	0.512720184	37.48	49.14	2.027	1.66
SPCC11E10.05C	ynd1	0.5556	2.461	0.255237763	38.56	50.65	4.832	1.756
SPAC5H10.04	SPAC5H10.04	0.7665	2.464	0.115487841	34.44	44.86	8.784	4.828
SPCC70.04C	SPCC70.04c	0.4891	2.465	0.310602337	40.2	52.95	1.567	3.023
SPAC22H10.08	SPAC22H10.08	0.5942	2.47	0.226067353	35.69	46.61	4.883	2.652
SPCC777.06C	SPCC777.06c	0.574	2.473	0.241088108	32.02	41.46	2.154	3.575
SPAC23H3.08C	bub3	0.6632	2.477	0.178355482	37.6	49.28	6.218	3.043

SPAC17H9.19C	cdt2	0.8279	2.483	0.082022117	23.19	29.06	10.58	7.917
SPAC4F10.07C	atg13	0.6909	2.484	0.160584807	33.13	42.99	6.858	3.328
SPBC530.14C	dsk1	0.5749	2.489	0.240407691	32.42	41.99	4.186	2.952
SPAC3G6.01	hrp3	0.5687	2.495	0.245116772	36.97	48.37	4.67	2.423
SPBC115.03	SPBC115.03	0.8335	2.496	0.079094396	30.78	39.68	12.49	7.031
SPCC23B6.05C	ssb3	0.5567	2.498	0.254378779	34.31	44.63	4.694	2.144
SPBP35G2.04C	SPBP35G2.04c	0.8631	2.499	0.063938883	30.6	39.43	13.9	9.73
SPBC25D12.05	trm1	0.4235	2.505	0.373146585	33.22	43.1	2.495	2.204
SPBC365.16	SPBC365.16	0.5649	2.507	0.248028425	33.94	44.11	4.882	2.104
SPAC14C4.06C	SPAC14C4.06c	0.7563	2.509	0.1213059	22.45	27.98	7.268	5.722
SPBC30B4.06C	SPBC30B4.06c	0.513	2.51	0.289882635	33.85	43.98	3.488	2.618
SPCC965.09	SPCC965.09	0.6164	2.513	0.21013737	39.44	51.82	5.059	3.097
SPBC577.14C	spa1	0.6788	2.517	0.168258166	35.08	45.68	6.862	2.983
SPAC17H9.03C	rdl1	0.831	2.519	0.080398976	23.73	29.77	10.77	8.284
SPBC21.02	SPBC21.02	0.7164	2.522	0.144844423	14.99	17.49	5.846	5.131
SPCC569.05C	SPCC569.05c	0.603	2.529	0.219682688	36.49	47.65	5.122	2.783
SPBC2G2.10C	mug110	0.5466	2.53	0.262330373	39.2	51.45	4.088	2.877
SPCC11E10.06C	elp4	0.646	2.531	0.189767482	31.2	40.22	5.418	3.535
SPAC7D4.04	taf1	0.5009	2.532	0.300248968	36.2	47.25	4.392	1.427
SPAC6G9.05	pcd1	0.5734	2.535	0.241542311	37.83	49.52	4.635	2.671
SPAC1F7.13C	rpl801	0.2607	2.536	0.583858969	5.949	4.788	2.007	1.622
SPAC1D4.02C	SPAC1D4.02c	0.6198	2.537	0.207748428	39.96	52.51	5.977	2.126
SPCP1E11.10	SPCP1E11.10	0.7468	2.544	0.126795691	11.93	13.17	6.684	5.809
SPBC839.02	SPBC839.02	0.7135	2.55	0.146606023	32.4	41.88	7.387	3.978
SPBC1198.09	ubc16	0.6241	2.553	0.204745817	37.82	49.49	5.095	3.353
SPAC31G5.15	psd3	0.8489	2.556	0.071143466	28.27	36.08	12.6	9.188
SPAC688.06C	slx4	0.1961	2.557	0.707522406	34.26	44.48	1.49	1.292
SPBC1921.06C	pvg3	0.7127	2.557	0.147093241	27.35	34.78	6.703	4.584
SPAPB8E5.05	mfm1	0.651	2.559	0.186419011	36.66	47.84	6.28	2.896
SPAC22H12.02	tfg3	0.7436	2.562	0.128660619	27.99	35.68	8.453	4.374
SPAC11E3.08C	nse6	0.5896	2.563	0.229442525	26.81	34.03	5.273	2.39
SPBC21B10.10	rps402	0.6948	2.563	0.15814019	28.9	36.95	6.329	4.29
SPBC2A9.13	SPBC2A9.13	0.7446	2.563	0.128076968	43.07	56.83	9.316	3.226
SPAC1F8.06	fta5	0.4223	2.567	0.374378919	40.89	53.77	3.494	1.566
SPCC63.06	SPCC63.06	0.8227	2.568	0.084758503	23.32	29.12	10.38	8.093
SPCC794.02	wtf5	0.8677	2.572	0.061630403	31.43	40.49	14.1	10.85
SPBC530.10C	anc1	0.3583	2.578	0.445753192	32.77	42.36	1.577	2.118
SPCC70.10	SPCC70.10	0.8123	2.582	0.090283547	34.79	45.19	11.14	6.698
SPBC660.09	mug168	0.531	2.583	0.274905479	36.65	47.81	4.135	2.525
SPAC222.15	meu13	0.6281	2.583	0.201971207	36.29	47.3	5.642	3.041
SPAC17C9.14	SPAC17C9.14	0.739	2.59	0.131355562	34.06	44.16	8.31	4.427
SPAC2E1P3.02C	amt3	0.6922	2.592	0.159768405	37.7	49.26	6.759	3.943
SPBC776.06C	SPBC776.06c	0.8401	2.592	0.075669015	27.15	34.46	12.65	8.372
SPBP22H7.08	rps1002	0.5459	2.599	0.262886906	40.86	53.69	4.509	2.456
SPCC1322.15	rpl3402	0.5339	2.602	0.272540079	38.06	49.75	5.029	1.276
SPCC74.05	rpl2702	0.8081	2.602	0.092534893	23.31	29.05	8.943	8.001
SPCC5E4.07	rpl2802	0.4522	2.603	0.344669442	4.782	3.058	2.15	2.782
SPAC1B3.10C	SPAC1B3.10c	0.4866	2.607	0.312827895	39.92	52.35	2.932	2.806
SPBC365.10	arp5	0.4973	2.608	0.303381541	10.56	11.16	4.489	1.449
SPBC3E7.12C	chr1	0.6499	2.608	0.187153463	35.47	46.1	6.592	2.636
SPAC10F6.05C	ubc6	0.4569	2.613	0.340178842	38.91	50.92	3.275	2.314
SPBP4H10.13	rps2302	0.6506	2.615	0.186685941	28.71	36.62	5.696	3.691
SPBC800.08	gcd10	0.6187	2.616	0.208519884	38.75	50.7	5.933	2.529
SPAC3C7.10	pex13	0.4649	2.618	0.332640454	38.45	50.28	3.417	2.306
SPBC3E7.11C	SPBC3E7.11c	0.5156	2.62	0.287687091	39.1	51.19	4.1	2.406
SPAC17G8.14C	pck1	0.5545	2.62	0.25609845	36.75	47.89	4.792	2.372
SPAC977.17	SPAC977.17	0.8035	2.621	0.095014119	36.61	47.69	9.905	7.195
SPCC1322.02	SPCC1322.02	0.4528	2.622	0.344093582	39.22	51.35	3.755	1.82
SPAC637.09	SPAC637.09	0.4631	2.629	0.334325219	38	49.62	3.597	2.156
SPBC2G2.14	SPBC2G2.14	0.5097	2.629	0.292685366	37.4	48.79	3.32	2.9
SPAC19E9.02	fin1	0.6288	2.63	0.201487467	33.2	42.89	5.611	3.25
SPAC29E6.01	pof11	0.5937	2.631	0.226432951	30.32	38.86	4.984	3.025
SPAC1805.15C	pub2	0.3584	2.632	0.445631999	32.83	42.38	1.01	2.22
SPAC8F11.05C	mug130	0.6962	2.637	0.157265981	38.44	50.24	7.515	3.472
SPAP7G5.05	rpl1002	0.7392	2.637	0.131238042	32.53	41.94	8.141	4.834
SPAC1610.01	SPAC1610.01	0.7972	2.639	0.09843271	21.81	26.89	9.191	7.314
SPAC27D7.14C	tpr1	0.592	2.642	0.227678293	17.81	21.28	4.832	3.367
SPAC4D7.07C	SPAC4D7.07c	0.6058	2.643	0.217670731	35.15	45.62	5.385	2.943
SPAC4H3.07C	SPAC4H3.07c	0.8006	2.646	0.096584414	32.17	41.42	10.79	6.409

SPAC1142.02C	SPAC1142.02c	0.3695	2.65	0.432385557	32.96	42.52	2.187	2.108
SPBC30D10.14	SPBC30D10.14	0.4386	2.651	0.357931373	34.17	44.22	2.162	2.602
SPAC1A6.01C	SPAC1A6.01c	0.646	2.655	0.189767482	35.95	46.71	6.156	3.265
SPBC1709.16C	SPBC1709.16c	0.7986	2.656	0.097670694	34.42	44.57	10.51	6.545
SPBC337.10C	SPBC337.10c	0.462	2.657	0.335358024	31.81	40.9	2.392	2.735
SPAC16E8.12C	SPAC16E8.12c	0.5977	2.657	0.223516744	39.74	52.03	5.324	2.859
SPBC651.05C	dot2	0.6497	2.661	0.187287133	35.66	46.3	5.25	4.122
SPBC19F8.04C	SPBC19F8.04c	0.2949	2.666	0.530325227	31.01	39.77	2.146	1.66
SPBC36.11	SPBC36.11	0.5025	2.668	0.298863934	34.81	45.1	1.23	3.256
SPAC17C9.16C	SPAC17C9.16c	0.5864	2.668	0.231806038	38.03	49.62	5.556	2.301
SPCC5E4.10C	SPCC5E4.10c	0.5945	2.67	0.225848141	34.79	45.07	5.183	2.971
SPAC343.11C	msc1	0.5563	2.673	0.25469094	36.35	47.25	5.293	1.847
SPAC23H4.08	iwr1	0.5047	2.674	0.296966695	33.12	42.72	2.433	3.263
SPCC1442.17C	ist1	0.5214	2.674	0.282828973	38.92	50.86	4.184	2.548
SPBC16A3.03C	ppr7	0.7078	2.674	0.150089442	35.12	45.53	7.026	4.598
SPAPB24D3.07C	SPAPB24D3.07c	0.8308	2.675	0.080503512	28.55	36.3	12.98	7.594
SPCC191.03C	SPCC191.03c	0.6489	2.677	0.187822226	39.13	51.15	6.443	3.119
SPAC24H6.03	cul3	0.6363	2.681	0.196338077	37.21	48.45	5.977	3.263
SPAC25B8.07C	SPAC25B8.07c	0.3369	2.685	0.472498989	34.58	44.75	3.211	0.877
SPAC3C7.03C	rhp55	0.3319	2.686	0.478992748	29.89	38.17	2.694	1.664
SPCC1235.03	SPCC1235.03	0.7234	2.687	0.140621496	35.38	45.88	8.06	4.37
SPAC1556.08C	cbs2	0.6425	2.692	0.192126868	27.41	34.69	5.502	4.1
SPBPB2B2.18	SPBPB2B2.18	0.4501	2.693	0.346690987	40.23	52.66	3.745	1.965
SPAC6F12.03C	fsv1	0.5605	2.694	0.251424383	29.83	38.08	4.997	2.488
SPAC1486.04C	alm1	0.7624	2.695	0.117817112	31.41	40.3	9.241	5.375
SPBC32F12.07C	SPBC32F12.07c	0.5779	2.7	0.238147306	35.61	46.17	4.626	3.194
SPCC4G3.02	aph1	0.1911	2.702	0.718739313	16.94	19.98	2.036	1.037
SPBC409.19C	SPBC409.19c	0.7656	2.704	0.115998075	20.69	25.24	8.65	6.089
SPAC222.07C	hri2	0.5552	2.705	0.255550543	33.53	43.25	5.309	1.92
SPAC3H1.13	ppk13	0.6977	2.706	0.156331277	38.01	49.53	7.689	3.664
SPAC140.02	gar2	0.7452	2.709	0.127727154	33.39	43.06	8.865	4.799
SPBC887.11	pus2	0.6905	2.71	0.160836317	40.24	52.66	6.216	4.734
SPBC26H8.11C	SPBC26H8.11c	0.531	2.715	0.274905479	34.45	44.53	5.025	1.771
SPAC6F6.09	eaf6	0.3992	2.716	0.398809467	27.46	34.71	3.302	1.827
SPAC22H10.07	scd2	0.7848	2.716	0.105241006	20.67	25.2	9.022	7.002
SPBC119.08	pmk1	0.6327	2.721	0.198802166	31.64	40.57	5.743	3.524
SPAC823.03	ppk15	0.2533	2.722	0.59636481	36.63	47.58	2.23	1.452
SPCC1494.05C	ubp12	0.3752	2.723	0.42573717	23.89	29.71	2.335	2.28
SPAC926.03	rlc1	0.6081	2.726	0.216024997	32.92	42.37	5.618	3.03
SPBC800.03	clr3	0.6789	2.729	0.168194191	37.73	49.11	7.781	2.672
SPCC1322.06	kap113	0.3537	2.732	0.45136494	30.73	39.29	3.446	0.7205
SPAC343.20	SPAC343.20	0.531	2.733	0.274905479	39.37	51.41	4.174	2.837
SPBC21C3.02C	dep1	0.5464	2.735	0.262489309	38.04	49.54	4.14	3.1
SPAC24B11.10C	chr3	0.5856	2.736	0.232398932	40	52.29	5.588	2.499
SPAC20H4.08	SPAC20H4.08	0.6152	2.737	0.210983673	37.59	48.9	6.066	2.734
SPAC1D4.05C	SPAC1D4.05c	0.6015	2.747	0.220764368	36.78	47.75	5.492	3.06
SPAC3A11.09	sod22	0.4652	2.748	0.332360294	33.96	43.8	2.852	2.736
SPAC17A2.11	SPAC17A2.11	0.331	2.749	0.480172006	36.12	46.82	2.003	2.021
SPAC3H1.14	SPAC3H1.14	0.6452	2.756	0.190305641	36.3	47.06	5.856	3.861
SPAC8C9.17C	spc34	0.8322	2.756	0.079772289	29.41	37.4	11.8	9.181
SPBC16C6.01C	SPBC16C6.01c	0.6024	2.758	0.220115037	17.02	20.02	4.633	3.979
SPAC13G6.09	SPAC13G6.09	0.603	2.758	0.219682688	14.94	17.09	4.402	4.117
SPAP7G5.03	prm1	0.693	2.758	0.159266765	32.28	41.42	7.26	4.162
SPBC83.18C	fic1	0.6446	2.761	0.190709699	33.16	42.66	6.497	3.246
SPBC947.02	apl2	0.526	2.762	0.279014256	39.28	51.24	3.771	3.094
SPBC1D7.05	byr2	0.728	2.762	0.137868621	27.84	35.19	8.641	4.743
SPAC2C4.06C	SPAC2C4.06c	0.5405	2.765	0.267204302	35.85	46.43	4.883	2.404
SPBC1D7.03	mug80	0.6466	2.767	0.1893643	24.06	29.87	6.475	3.359
SPCC24B10.22	pog1	0.4701	2.772	0.327809749	39.72	51.84	3.325	2.701
SPCC777.12C	SPCC777.12c	0.5454	2.775	0.263284866	34.85	45	5.467	1.571
SPAC1783.05	hrp1	0.5474	2.776	0.261695207	36.16	46.84	5.498	1.579
SPCC830.08C	yop1	0.6222	2.778	0.206069993	39.09	50.94	6.41	2.652
SPBC1306.02	SPBC1306.02	0.7667	2.779	0.115374537	19.63	23.64	8.48	6.6
SPCC550.08	SPCC550.08	0.4035	2.788	0.394156461	37.03	48.05	3.897	1.067
SPAC13C5.03	tht1	0.7106	2.79	0.148374797	31.92	40.87	7.315	5.206
SPBC839.03C	SPBC839.03c	0.4584	2.794	0.338755391	40.46	52.85	4.279	1.618
SPBC887.04C	lub1	0.5618	2.794	0.250418265	42.56	55.79	5.102	2.702
SPBC2A9.05C	tvp23	0.6032	2.805	0.219538667	33.51	43.08	6.191	2.428
SPBC543.07	pek1	0.251	2.817	0.600326279	35.04	45.22	2.362	1.435

SPBC651.12C	SPBC651.12c	0.492	2.817	0.308034897	35.07	45.26	4.407	2.175
SPCC1450.03	SPCC1450.03	0.8472	2.819	0.072014053	23.72	29.33	13.76	10
SPAC1A6.10	SPAC1A6.10	0.5744	2.82	0.240785569	32.8	42.07	5.638	2.424
SPAC5D6.02C	mug165	0.4508	2.822	0.346016093	29.5	37.43	1.779	2.96
SPAC8F11.03	msh3	0.496	2.822	0.304518324	38.97	50.72	3.416	3.026
SPAC29A4.14C	SPAC29A4.14c	0.1216	2.823	0.915066425	32.5	41.65	1.809	0.7414
SPAP27G11.02	SPAP27G11.02	0.4431	2.823	0.35349825	36.62	47.42	3.724	2.173
SPAC1F8.04C	SPAC1F8.04c	0.3306	2.829	0.480697151	34.32	44.19	2.881	1.698
SPAC6G10.06	SPAC6G10.06	0.3179	2.836	0.497709472	39.41	51.31	2.606	1.825
SPBC2G2.13C	SPBC2G2.13c	0.5918	2.837	0.227825039	39.44	51.35	6.085	2.344
SPAC6B12.12	tom70	0.6064	2.837	0.217240807	31.03	39.56	5.047	3.782
SPCC1682.14	rpl1902	0.6594	2.842	0.180851057	25.58	31.91	7.099	3.384
SPAC328.03	tps1	0.5142	2.845	0.288867928	9.275	9.023	3.515	3.232
SPAC4H3.14C	SPAC4H3.14c	0.8289	2.846	0.08149786	31.99	40.9	12.66	8.806
SPAC3G9.04	ssu72	0.4901	2.852	0.309715297	36.19	46.78	3.445	2.994
SPAC29E6.07	SPAC29E6.07	0.4682	2.855	0.329568591	37.53	48.66	4.734	0.987
SPAC17D4.04	SPAC17D4.04	0.5235	2.855	0.281083314	34.54	44.46	5.346	1.441
SPBC6B1.08C	ofd1	0.4466	2.859	0.350081281	34.56	44.48	2.025	2.931
SPBC1348.07	SPBC1348.07	0.5673	2.859	0.246187216	40.51	52.83	4.333	3.57
SPAC1952.03	otu2	0.7967	2.859	0.098705183	22.38	27.39	9.943	7.901
SPAC1006.06	rgf2	0.5282	2.862	0.277201603	39.43	51.31	4.802	2.527
SPAC1556.04C	cdd1	0.5302	2.863	0.275560277	37.53	48.65	3.694	3.378
SPBC25B2.01	SPBC25B2.01	0.5255	2.874	0.27942728	37.98	49.26	4.26	2.996
SPCP1E11.06	apl4	0.8089	2.874	0.092105165	23.79	29.35	11.33	7.997
SPCC736.06	SPCC736.06	0.7478	2.878	0.126214539	19.34	23.1	7.745	6.512
SPAC6B12.15	cpc2	0.7966	2.879	0.098759698	19.17	22.85	10.12	7.878
SPBC2A9.06C	SPBC2A9.06c	0.5005	2.881	0.300595918	34.33	44.12	4.685	2.162
SPAC20G8.04C	SPAC20G8.04c	0.4949	2.889	0.305482546	36.91	47.73	4.471	2.339
SPBC16C6.03C	SPBC16C6.03c	0.6322	2.897	0.199145508	28.98	36.6	6.527	3.319
SPAC23A1.07	SPAC23A1.07	0.5574	2.898	0.253833036	22.59	27.63	5.556	2.354
SPAC3G6.06C	rad2	0.5843	2.898	0.233364114	33.43	42.84	5.729	2.854
SPAC513.02	SPAC513.02	0.4223	2.903	0.374378919	36.77	47.52	4.049	1.61
SPAC5H10.02C	SPAC5H10.02c	0.3154	2.907	0.501138311	37.07	47.93	3.251	1.074
SPAPB8E5.10	SPAPB8E5.10	0.4401	2.916	0.356448631	35.44	45.64	3.586	2.431
SPCC594.02C	SPCC594.02c	0.7413	2.919	0.130006	18.03	21.2	7.677	6.412
SPBC13G1.10C	mug81	0.4309	2.92	0.365623506	31.99	40.78	4.328	1.266
SPCC737.03C	ima1	0.2031	2.923	0.692290077	36.81	47.54	1.986	1.393
SPCC1442.14C	SPCC1442.14c	0.6259	2.925	0.203495048	40.55	52.79	6.954	2.609
SPAC13G7.13C	msa1	0.8396	2.927	0.07592757	30.1	38.12	13.24	10.14
SPBC354.08C	SPBC354.08c	0.6405	2.928	0.193480866	39.1	50.75	5.984	4.156
SPAC1952.16	rga9	0.6637	2.928	0.178028182	39.7	51.6	7.587	3.311
SPAC1486.08	SPAC1486.08	0.3424	2.93	0.465466244	38.2	49.49	3.084	1.787
SPAC13G7.12C	SPAC13G7.12c	0.7434	2.933	0.128777443	22.23	27.07	8.1	6.308
SPAC26A3.06	SPAC26A3.06	0.3944	2.934	0.404063094	36.83	47.56	3.563	1.914
SPAC227.06	SPAC227.06	0.5952	2.934	0.225337077	36.3	46.81	6.024	2.919
SPCC126.01C	SPCC126.01c	0.5154	2.935	0.287855586	27.62	34.63	3.814	3.254
SPCC613.06	rpl902	0.7468	2.937	0.126795691	15.1	17.06	7.474	6.834
SPAC1687.23C	SPAC1687.23c	0.5269	2.94	0.278271801	39.59	51.42	5.221	2.185
SPBC337.13C	gtr1	0.2728	2.944	0.564155634	35.22	45.28	1.852	1.885
SPAC17G6.03	SPAC17G6.03	0.4089	2.947	0.388382889	33.91	43.44	2.279	2.683
SPAC24C9.05C	mug70	0.789	2.951	0.102922997	24.89	30.79	10.63	7.338
SPBC1685.11	rlp1	0.4343	2.952	0.362210171	39.15	50.79	3.511	2.481
SPBC3D6.09	dpb4	0.5538	2.955	0.256647049	39.02	50.6	3.578	3.911
SPAC8C9.04	SPAC8C9.04	0.8174	2.955	0.087565367	27.56	34.52	11.08	9.343
SPAPB24D3.01	SPAPB24D3.01	0.4478	2.964	0.348915911	33.09	42.28	4.426	1.676
SPBC1198.07C	SPBC1198.07c	0.3241	2.965	0.489320969	38.66	50.09	3.278	1.356
SPBC2G5.04C	SPBC2G5.04c	0.6711	2.965	0.173212761	43.3	56.6	8.064	3.141
SPAP27G11.15	slx1	0.5061	2.966	0.295763663	39.71	51.56	3.787	3.202
SPAC977.15	SPAC977.15	0.6138	2.967	0.211973116	33.09	42.26	6.385	3.16
SPAC823.09C	SPAC823.09c	0.6834	2.968	0.165325026	36.2	46.62	7.68	4.217
SPBC15D4.03	slm9	0.7115	2.968	0.147825096	36.28	46.75	8.037	5.065
SPCC1020.11C	SPCC1020.11c	0.7569	2.972	0.120961495	25.03	30.95	9.225	6.397
SPAC2G11.09	SPAC2G11.09	0.6382	2.973	0.1950432	34.65	44.44	6.496	3.809
SPBC18H10.08C	ubp4	0.602	2.976	0.220403509	41.45	53.99	6.351	2.86
SPBC31F10.16	SPBC31F10.16	0.254	2.978	0.595166283	34.02	43.55	2.666	1.302
SPAC652.01	SPAC652.01	0.6938	2.98	0.158765704	33.07	42.22	7.543	5.103
SPBC31F10.09C	nut2	0.738	2.981	0.131943638	18.96	22.42	7.867	6.388
SPAC2G11.10C	SPAC2G11.10c	0.5058	2.982	0.296021175	38.06	49.22	4.268	2.917
SPAC11D3.08C	SPAC11D3.08c	0.2901	2.987	0.537452271	31.64	40.21	1.157	2.106

SPBC337.07C	SPBC337.07c	0.4503	2.987	0.346498053	33.58	42.93	2.519	3.012
SPAC16A10.02	sub1	0.5372	2.989	0.269863996	36.35	46.81	5.641	1.939
SPBC1734.06	rhp18	0.8354	2.993	0.078105529	20.25	24.22	13.3	10.02
SPBC577.15C	sim3	0.4036	2.996	0.394048842	4.656	2.329	2.329	2.825
SPBC776.05	SPBC776.05	0.8082	3	0.092481154	26.24	32.6	11.14	8.752
SPAC2F3.05C	SPAC2F3.05c	0.6602	3.001	0.18032448	37.66	48.63	4.614	5.513
SPBC1271.12	kes1	0.6043	3.005	0.218747406	35.8	46.01	5.064	4.138
SPBC577.04	SPBC577.04	0.4458	3.006	0.350859936	35.8	46.01	4.018	2.3
SPCC364.05	vps3	0.7698	3.01	0.113622093	24.94	30.77	10.48	6.374
SPBC646.02	cwf11	0.4439	3.014	0.352714855	35.59	45.7	3.591	2.642
SPAC5D6.06C	alg14	0.7018	3.015	0.153786636	28.46	35.7	8.119	5.097
SPAC11D3.18C	SPAC11D3.18c	0.7293	3.02	0.137093786	30.47	38.52	8.661	5.919
SPAC22F8.02C	pvg5	0.3694	3.025	0.432503109	31.31	39.69	2.119	2.653
SPAC824.09C	SPAC824.09c	0.7983	3.025	0.097833871	25.5	31.54	10.64	8.404
SPCC1739.12	ppe1	0.4917	3.026	0.308299792	35.83	46.03	4.691	2.382
SPAC8E11.04C	SPAC8E11.04c	0.6213	3.026	0.206698646	35.86	46.07	6.107	3.846
SPBC36.06C	spo9	0.4961	3.028	0.304430773	35.92	46.15	4.905	2.199
SPCC965.12	SPCC965.12	0.6031	3.028	0.219610672	35.42	45.44	6.543	2.832
SPCC736.02	SPCC736.02	0.2671	3.031	0.573326112	33.32	42.5	2.09	1.849
SPBP4H10.03	oxa102	0.7536	3.031	0.12285911	19.83	23.58	8.156	7.14
SPAC13G7.09C	SPAC13G7.09c	0.3767	3.035	0.42400438	37.08	47.76	3.262	2.169
SPBC21B10.13C	yox1	0.43	3.038	0.366531544	19.38	22.93	3.666	2.463
SPCC162.04C	wtf13	0.4396	3.04	0.356942316	38.38	49.59	3.96	2.337
SPAC688.12C	SPAC688.12c	0.425	3.041	0.37161107	40.37	52.38	4.076	1.988
SPAC3A12.03C	meu34	0.5183	3.043	0.285418791	34.3	43.86	5.084	2.498
SPBC36B7.02	SPBC36B7.02	0.2622	3.046	0.581367313	34.41	44	2.957	1.106
SPAC2G11.06	vps4	0.1674	3.048	0.776244546	31.42	39.81	2.238	1.023
SPBC21B10.06C	inp2	0.5617	3.057	0.250495576	27.69	34.57	5.67	2.852
SPAC926.06C	SPAC926.06c	0.5358	3.064	0.270997291	38.77	50.1	5.29	2.701
SPBC1709.04C	cyp3	0.5702	3.064	0.243972787	35.68	45.77	5.693	3.061
SPAC19B12.04	rps3001	0.4011	3.065	0.396747338	35.09	44.93	3.91	1.869
SPAC26H5.07C	SPAC26H5.07c	0.758	3.067	0.120330794	22.8	27.69	9.708	6.52
SPAC16C9.04C	mot2	0.6969	3.068	0.156829535	28.16	35.2	8.055	4.819
SPAC11H11.05C	fta6	0.4014	3.072	0.396422632	37.79	48.72	3.518	2.297
SPAC1805.07C	dad2	0.6669	3.074	0.175939283	24.16	29.58	6.237	5.131
SPAC23G3.12C	SPAC23G3.12c	0.5277	3.075	0.277612906	36.96	47.55	5.176	2.686
SPBC215.04	git11	0.4511	3.076	0.345727173	32.61	41.43	4.061	2.479
SPBC11G11.03	mrt4	0.561	3.079	0.251037139	37.05	47.67	5.651	2.925
SPAC11G7.03	idh1	0.4366	3.08	0.359916269	35.29	45.2	2.475	3.014
SPBP35G2.11C	SPBP35G2.11c	0.3775	3.087	0.423083044	26.36	32.66	3.573	1.979
SPAC3G9.01	nsk1	0.6712	3.095	0.173148052	34.23	43.69	7.668	4.241
SPBC3B9.05	SPBC3B9.05	0.5594	3.104	0.252277538	31.73	40.17	2.866	4.263
SPAPJ691.02	SPAPJ691.02	0.7675	3.105	0.114921616	33.54	42.7	10.25	6.885
SPCC18.10	SPCC18.10	0.4528	3.107	0.344093582	33.57	42.74	2.411	3.202
SPAC19D5.06C	din1	0.6604	3.107	0.180192935	38.28	49.35	7.132	4.394
SPBC3D6.06C	prs5	0.5706	3.109	0.243668233	34.47	44.01	6.407	2.203
SPACUNK4.16C	SPACUNK4.16c	0.5168	3.115	0.286677495	40.12	51.92	4.883	2.874
SPCC794.07	lat1	0.6672	3.115	0.175743962	12.82	13.62	6.138	5.311
SPCC576.13	swc5	0.7735	3.118	0.111539682	28.64	35.82	10.58	7.098
SPAC4F10.20	grx1	0.3448	3.122	0.462432743	34.98	44.71	0.4368	2.547
SPAC17A2.12	SPAC17A2.12	0.6094	3.126	0.21509755	38.48	49.6	6.577	3.364
SPBC14F5.09C	ade8	0.3398	3.127	0.468776625	31.75	40.17	1.742	2.468
SPCC594.01	SPCC594.01	0.5533	3.127	0.25703933	34.69	44.28	5.516	3.029
SPAC1006.01	psp3	0.4376	3.133	0.358922687	37.68	48.47	4.118	2.342
SPBC4B4.04	SPBC4B4.04	0.3042	3.135	0.51684079	35.75	45.76	3.116	1.655
SPCC1906.04	wtf20	0.3703	3.136	0.431446288	36.55	46.88	3.292	2.232
SPBC409.10	ade7	0.4272	3.136	0.369368756	32.69	41.47	3.958	2.356
SPBC1347.07	rex2	0.5348	3.137	0.271808601	32.04	40.55	2.929	3.99
SPAC17C9.15C	SPAC17C9.15c	0.4448	3.143	0.351835221	32.56	41.28	4.756	1.558
SPBC800.02	whi5	0.3481	3.145	0.458295977	37.57	48.3	3.296	1.997
SPBC582.06C	mcp6	0.8279	3.146	0.082022117	27.83	34.63	13.41	10.02
SPAC23D3.09	arp42	0.738	3.155	0.131943638	24.19	29.51	9.809	5.646
SPAC31A2.12	SPAC31A2.12	0.6724	3.158	0.172372295	27.64	34.35	7.961	4.234
SPBC1703.03C	SPBC1703.03c	0.6641	3.165	0.17776652	27.58	34.26	7.344	4.536
SPBC649.03	rhp14	0.5966	3.168	0.224316751	11.34	11.46	5.466	4.119
SPAC3H1.04C	mdm31	0.2423	3.17	0.615646586	32.7	41.43	2.894	1.181
SPAC343.19	lsb6	0.513	3.171	0.289882635	39.65	51.18	5.761	1.621
SPAP8A3.04C	hsp9	0.5438	3.173	0.264560797	28.89	36.09	3.199	4.095
SPAC1A6.08C	mug125	0.7925	3.175	0.101000729	29.36	36.74	12.2	7.579

SPAC5H10.05C	SPAC5H10.05c	0.4182	3.176	0.378615972	35.44	45.27	4.492	1.535
SPAC15A10.03C	rhp54	0.7264	3.176	0.138824164	19.52	22.93	7.718	6.669
SPAPB1A10.10C	ypt71	0.5122	3.177	0.290560426	37.07	47.55	4.239	3.412
SPBP8B7.08C	SPBP8B7.08c	0.2562	3.178	0.591420875	33.7	42.83	2.743	1.593
SPCC1223.02	nmt1	0.6014	3.181	0.220836576	32.82	41.58	1.669	5.124
SPAC1B3.15C	SPAC1B3.15c	0.5481	3.185	0.261140198	36.39	46.58	5.902	2.605
SPAC23D3.01	SPAC23D3.01	0.472	3.195	0.326058001	38.34	49.32	4.623	2.514
SPBC337.15C	coq7	0.6554	3.195	0.183493563	11.44	11.56	7.622	4.058
SPAC19G12.03	cda1	0.461	3.202	0.336299075	39.75	51.29	4.299	2.667
SPBC8D2.11	SPBC8D2.11	0.5873	3.208	0.231139999	37.65	48.32	5.962	3.629
SPAC1250.05	rpl3002	0.582	3.21	0.235077015	40.42	52.21	6.096	3.374
SPAC6G9.16C	SPAC6G9.16c	0.5334	3.212	0.272946989	33.3	42.22	3.425	3.962
SPAC11D3.10	SPAC11D3.10	0.4459	3.214	0.350762528	39.96	51.57	3.662	2.947
SPAC1687.07	SPAC1687.07	0.421	3.22	0.375717904	38.4	49.36	4.63	1.454
SPBC16G5.17	SPBC16G5.17	0.4656	3.237	0.331987028	28.7	35.73	4.514	2.612
SPAC13G7.05	are1	0.6958	3.237	0.157515576	33.65	42.68	8.625	4.915
SPCC191.11	inv1	0.6375	3.238	0.195519811	32.36	40.86	4.722	5.322
SPAC1F3.07C	rsc58	0.6839	3.24	0.165007396	25.58	31.34	7.806	5.46
SPAC20G4.01	caf16	0.02809	3.242	1.551448261	35.12	44.73	1.264	0.5589
SPCC1827.03C	SPCC1827.03c	0.316	3.242	0.500312917	33.8	42.88	3.177	1.9
SPAC3F10.18C	rpl4102	0.7508	3.247	0.124475736	32.27	40.73	10.88	5.877
SPAC3G9.07C	hos2	0.3093	3.249	0.50962008	36.63	46.84	3.262	1.741
SPBC1718.06	msp1	0.4516	3.25	0.345246067	31.87	40.16	3.875	2.945
SPBC56F2.08C	SPBC56F2.08c	0.4218	3.251	0.374893425	31.03	38.97	4.015	2.447
SPAC17A2.05	osm1	0.4254	3.252	0.371202514	32.63	41.22	2.917	2.997
SPCC645.13	SPCC645.13	0.5163	3.252	0.287097875	40.77	52.65	5.12	2.97
SPBC651.10	nse5	0.6242	3.252	0.204676236	17.96	20.63	6.286	4.429
SPCC285.15C	rps2802	0.6569	3.254	0.182500738	29.66	37.05	7.58	4.375
SPCC550.11	SPCC550.11	0.3777	3.258	0.422853015	6.532	4.594	2.367	2.9
SPBC3B8.07C	dsd1	0.3731	3.26	0.428174751	35.09	44.66	3.862	1.92
SPCC61.05	SPCC61.05	0.2298	3.262	0.638649976	25.65	31.41	1.609	2.067
SPAC328.10C	rps502	0.5903	3.265	0.228927217	27.5	34	6.441	3.406
SPAC22E12.18	SPAC22E12.18	0.4368	3.269	0.35971737	8.749	7.69	3.434	3.051
SPAPB24D3.09C	pdr1	0.4814	3.271	0.317493914	33.92	43.01	2.929	3.572
SPCC594.05C	spf1	0.4231	3.272	0.373556975	35.97	45.88	4.601	1.766
SPCC338.07C	naa15	0.3517	3.273	0.453827632	36.01	45.94	3.484	2.072
SPAC1071.08	rpp203	0.7819	3.273	0.106848787	28.88	35.93	11.84	7.508
SPCC16A11.10C	oca8	0.6865	3.274	0.163359458	37.21	47.61	8.319	4.933
SPAC23D3.12	SPAC23D3.12	0.3224	3.275	0.491604967	26.67	32.83	2.43	2.331
SPCC550.12	arp6	0.5015	3.275	0.299729063	33.56	42.49	5.809	1.541
SPAC1B2.03C	SPAC1B2.03c	0.3961	3.282	0.402195158	30.92	38.78	4.442	1.428
SPBPB10D8.02C	SPBPB10D8.02c	0.4474	3.286	0.34930402	35.11	44.65	5.061	1.497
SPAC17A2.10C	SPAC17A2.10c	0.5024	3.298	0.298950369	40.04	51.56	4.819	3.09
SPBC27B12.04C	SPBC27B12.04c	0.606	3.302	0.217527376	40.05	51.56	7.346	2.884
SPBC1271.09	SPBC1271.09	0.454	3.305	0.342944147	37.69	48.24	4.061	2.946
SPBC56F2.10C	alg5	0.1802	3.307	0.744245213	32.07	40.35	0.8163	1.689
SPAC167.06C	mug143	0.4868	3.311	0.31264943	34.72	44.07	5.155	2.473
SPCC191.05C	SPCC191.05c	0.8187	3.311	0.08687521	31.24	39.19	13.66	9.813
SPAC56F8.16	esc1	0.395	3.321	0.403402904	39.75	51.12	4.232	1.92
SPCC830.10	SPCC830.10	0.5022	3.323	0.299123292	36.92	47.14	5.816	1.807
SPBC1271.15C	SPBC1271.15c	0.2894	3.333	0.538501473	37.17	47.48	2.361	2.293
SPAC630.15	mug177	0.3858	3.343	0.413637777	33.66	42.54	2.787	2.79
SPCC74.04	SPCC74.04	0.76	3.344	0.119186408	26.45	32.41	11.46	6.494
SPBP35G2.02	SPBP35G2.02	0.5474	3.349	0.261695207	36.47	46.48	4.629	4.071
SPAC2G11.07C	ptc3	0.2419	3.35	0.616364132	34.21	43.3	0.9097	2.069
SPAC3F10.12C	SPAC3F10.12c	0.6167	3.35	0.209926052	38.03	48.66	6.072	4.645
SPBC543.08	SPBC543.08	0.4554	3.351	0.341606974	38.91	49.9	4.874	2.28
SPCC1450.05C	rox3	0.5349	3.354	0.271727402	36.16	46.04	5.897	2.803
SPBC3E7.07C	SPBC3E7.07c	0.3908	3.357	0.408045445	37.01	47.22	3.782	2.427
SPAC513.05	ams1	0.5535	3.358	0.256882375	36.81	46.94	5.944	3.236
SPAC959.05C	SPAC959.05c	0.5962	3.359	0.224608028	35.2	44.68	6.49	3.811
SPAC26A3.10	cnt6	0.4151	3.361	0.381847267	36.58	46.6	4.581	1.887
SPCC757.12	SPCC757.12	0.3996	3.362	0.39837452	40.22	51.71	3.45	2.772
SPBC56F2.05C	SPBC56F2.05c	0.727	3.364	0.138465589	18.26	20.9	9.284	6.38
SPCC777.08C	bit61	0.2613	3.368	0.58286059	35.11	44.54	0.9868	2.199
SPAC23H3.14	SPAC23H3.14	0.4108	3.371	0.386369565	38.21	48.88	4.545	1.877
SPBC12C2.09C	SPBC12C2.09c	0.4822	3.374	0.316772794	36.7	46.76	5.108	2.601
SPAC1B3.05	not3	0.7069	3.375	0.150642018	16.89	18.96	8.543	6.011
SPAC3A11.07	SPAC3A11.07	0.7668	3.376	0.115317896	23.45	28.17	10.06	8.168

SPBC15D4.07C	atg9	0.4762	3.378	0.322210609	39.01	49.99	4.873	2.752
SPAC29B12.06C	rcd1	0.4769	3.379	0.321572678	27.59	33.97	5.022	2.607
SPCC576.02	SPCC576.02	0.4395	3.38	0.357041121	39.72	50.99	4.031	2.902
SPCC1494.09C	SPCC1494.09c	0.6093	3.382	0.215168822	34.34	43.44	7.112	3.639
SPAC24C9.16C	cox8	0.6229	3.388	0.205581669	34.48	43.62	7.516	3.671
SPCC70.03C	SPCC70.03c	0.4688	3.389	0.329012397	38.32	49.01	3.673	3.487
SPBC16H5.06	rip1	0.4777	3.396	0.320844759	22.33	26.56	4.842	2.773
SPAC1851.03	ckb1	0.4247	3.401	0.371917739	34.53	43.67	4.433	2.37
SPBC3B8.06	SPBC3B8.06	0.6989	3.406	0.15558496	41.51	53.47	10.18	3.972
SPAC22H12.05C	SPAC22H12.05c	0.3158	3.407	0.500587874	36.09	45.86	3.5	1.819
SPBC1652.01	SPBC1652.01	0.3205	3.407	0.494171966	36.4	46.3	2.542	2.537
SPAC30C2.05	erv14	0.5384	3.407	0.268894949	38.05	48.6	6.146	2.733
SPBC83.04	apc15	0.438	3.411	0.358525889	40	51.33	2.555	3.57
SPAC1952.06C	SPAC1952.06c	0.5214	3.412	0.282828973	34.25	43.26	5.919	2.59
SPCC550.14	vgl1	0.4716	3.414	0.326426204	29.54	36.65	4.755	2.865
SPAC1142.03C	swi2	0.1517	3.418	0.819014419	35.07	44.41	1.695	1.533
SPBC902.04	SPBC902.04	0.313	3.423	0.504455662	34.2	43.18	1.893	2.516
SPBC342.03	gas4	0.3675	3.425	0.434742657	34.87	44.12	3.007	2.64
SPAC26F1.02	pnn1	0.4541	3.425	0.342848498	41.89	53.97	4.506	2.824
SPCC663.06C	SPCC663.06c	0.4976	3.426	0.303119628	32.62	40.97	5.915	1.876
SPBC16A3.18	cip1	0.4819	3.433	0.317043074	44.48	57.59	4.362	3.362
SPCC31H12.08C	ccr4	0.7578	3.434	0.120445399	31.72	39.69	11.02	7.171
SPAC27D7.11C	SPAC27D7.11c	0.7174	3.435	0.144238628	31.26	39.05	9.033	6.365
SPAC22E12.14C	sck2	0.3746	3.436	0.426432227	38.29	48.91	3.953	2.186
SPBC215.06C	SPBC215.06c	0.5547	3.444	0.255941834	38.36	49	6.97	1.952
SPBC215.05	gpd1	0.5933	3.444	0.226725652	42.43	54.7	7.015	3.434
SPAC30.04C	abc4	0.4041	3.448	0.39351115	28.46	35.09	3.719	2.782
SPAC227.15	SPAC227.15	0.4815	3.452	0.317403709	41.74	53.72	4.349	3.398
SPAC977.11	SPAC977.11	0.4218	3.455	0.374893425	34.14	43.05	2.13	3.361
SPBC1773.16C	SPBC1773.16c	0.4225	3.46	0.374173287	35.49	44.94	2.662	3.279
SPAC3F10.10C	map3	0.3874	3.461	0.411840384	39.09	50	3.541	2.724
SPBC23G7.13C	SPBC23G7.13c	0.5561	3.462	0.254847105	36.16	45.88	6.628	2.779
SPBC365.11	SPBC365.11	0.3284	3.469	0.483596852	39.23	50.17	3.004	2.453
SPAC25A8.03C	SPAC25A8.03c	0.3291	3.472	0.482672118	34.83	44	2.468	2.555
SPAC8F11.09C	nnt1	0.3952	3.48	0.403183064	39.05	49.91	4.157	2.356
SPBP8B7.31	SPBP8B7.31	0.6131	3.486	0.212468684	33.77	42.49	7.061	4.157
SPAC16E8.08	SPAC16E8.08	0.3619	3.488	0.441411417	32.43	40.61	2.041	2.907
SPBC947.05C	frp2	0.5787	3.488	0.237546518	36.72	46.63	6.386	3.806
SPAC17G6.15C	SPAC17G6.15c	0.7314	3.491	0.135845044	37.79	48.13	10.37	6.276
SPAC4A8.04	isp6	0.4686	3.496	0.329197716	34.08	42.91	2.683	3.772
SPAC1F8.03C	str3	0.6607	3.497	0.179995693	33.47	42.05	8.163	4.833
SPAC1805.01C	ppk6	0.2969	3.498	0.527389802	36.39	46.15	3.448	1.78
SPAC24C9.02C	SPAC24C9.02c	0.3987	3.5	0.399353764	39.53	50.56	4.266	2.335
SPBC2D10.17	clr1	0.4652	3.504	0.332360294	38.14	48.6	5.059	2.637
SPCC162.06C	SPCC162.06c	0.4454	3.507	0.351249787	38.77	49.48	3.677	3.38
SPBC1683.09C	frp1	0.2293	3.508	0.639595945	22.11	26.11	2.029	1.998
SPBC18H10.13	rps1402	0.3335	3.509	0.476904162	34.63	43.67	3.968	1.631
SPCC63.13	SPCC63.13	0.4049	3.51	0.392652223	37.71	47.98	4.368	2.344
SPAC144.11	rps1102	0.6029	3.511	0.219754716	27.72	33.96	7.067	3.91
SPBC19F8.03C	SPBC19F8.03c	0.3854	3.513	0.41408829	40.04	51.26	3.919	2.501
SPCC4B3.04C	nte1	0.4459	3.519	0.350762528	40.95	52.52	3.19	3.603
SPCC794.12C	mae2	0.6603	3.519	0.180258703	33.5	42.07	7.778	5.22
SPAC23H3.05C	swd1	0.4329	3.52	0.363612414	36.06	45.66	5.185	1.687
SPBC365.14C	uge1	0.6615	3.526	0.179470151	23.11	27.48	6.93	5.844
SPBC11C11.06C	SPBC11C11.06c	0.4724	3.535	0.325690111	41.22	52.87	5.372	2.479
SPAC23C4.02	crn1	0.5313	3.535	0.274660184	37.79	48.07	5.452	3.636
SPBC12C2.03C	SPBC12C2.03c	0.3913	3.537	0.407490152	34.86	43.95	2.752	3.057
SPCC584.02	cuf2	0.4668	3.538	0.330869153	35.13	44.33	5.261	2.507
SPAC5D6.05	Sep-11	0.6519	3.539	0.185819019	25.94	31.43	7.13	5.752
SPAC27D7.08C	SPAC27D7.08c	0.2891	3.541	0.538951908	32.67	40.87	2.702	2.227
SPBC8D2.01	gsk31	0.3615	3.548	0.441891698	37.04	46.99	4.067	2.07
SPCC16A11.07	coq10	0.4047	3.549	0.392866796	39.99	51.13	3.933	2.798
SPBC839.14C	SPBC839.14c	0.4074	3.55	0.389978975	34.88	43.96	4.941	1.563
SPAC18B11.02C	SPAC18B11.02c	0.3733	3.557	0.42794201	34.05	42.79	2.58	2.964
SPBC16A3.17C	SPBC16A3.17c	0.4284	3.557	0.368150538	29.5	36.4	4.443	2.732
SPACUNK4.14	mdb1	0.4803	3.559	0.318487413	37.39	47.47	5.483	2.58
SPBC15D4.10C	amo1	0.7067	3.562	0.150764909	18.42	20.85	7.635	7.157
SPAC1B3.03C	wis2	0.4031	3.565	0.394587202	38.34	48.79	4.513	2.259
SPBC19G7.18C	SPBC19G7.18c	0.6212	3.574	0.206768553	32.93	41.19	7.554	4.225

SPBC1709.01	chs2	0.3531	3.575	0.452102282	31.91	39.76	2.762	2.763
SPBC1289.08	uap1	0.4621	3.575	0.335264031	32.22	40.19	3.313	3.653
SPBC3B8.10C	nem1	0.4459	3.576	0.350762528	37.77	47.97	4.968	2.532
SPAC31A2.15C	dcc1	0.3645	3.577	0.438302467	32.71	40.87	2.174	2.991
SPAC17A2.06C	vps8	0.3863	3.578	0.413075292	33.53	42.02	3.056	3.109
SPBC725.03	SPBC725.03	0.7823	3.578	0.10662667	27.76	33.94	11.51	9.259
SPCC1739.03	hrr1	0.4113	3.585	0.38584129	32.93	41.18	3.24	3.153
SPAC22H10.03C	kap114	0.3436	3.586	0.463946845	36.89	46.74	3.904	2.061
SPBC28F2.03	ppi1	0.2797	3.588	0.553307534	37.01	46.9	2.223	2.506
SPAC1071.09C	SPAC1071.09c	0.4672	3.592	0.330497166	40.39	51.63	5.335	2.563
SPCC24B10.21	tpi1	0.2347	3.593	0.62948691	36.06	45.55	2.18	2.055
SPBC31F10.13C	hip1	0.2994	3.605	0.523748204	39.48	50.34	3.275	2.149
SPBC4F6.10	vps901	0.7034	3.608	0.152797636	20.7	23.99	8.086	6.936
SPAC24H6.13	SPAC24H6.13	0.4315	3.61	0.3650192	38.09	48.38	4.709	2.631
SPAC2C4.16C	rps801	0.6486	3.62	0.188023056	17.42	19.37	6.623	5.871
SPBC12C2.02C	ste20	0.2603	3.624	0.584525832	24.1	28.74	3.591	1.694
SPBC16E9.08	mcp4	0.5608	3.624	0.251191995	36.51	46.14	6.629	3.461
SPCC1183.04C	pet127	0.3329	3.625	0.477686205	28.95	35.53	3.366	2.47
SPBC2G2.01C	liz1	0.03147	3.633	1.502103257	29.19	35.86	1.458	0.465
SPCC895.06	elp2	0.7788	3.634	0.108574057	27.77	33.87	11.38	9.321
SPAC29B12.10C	pgt1	0.7702	3.636	0.113396486	28.31	34.62	12.05	8.228
SPAC4G8.08	SPAC4G8.08	0.4841	3.642	0.315064917	39.3	50.04	5.664	2.663
SPAC21E11.04	ppr1	0.3943	3.645	0.404173223	40.07	51.1	4.229	2.577
SPBC16E9.06C	uvi31	0.4044	3.651	0.393188853	35.69	44.95	4.622	2.335
SPCC1742.01	SPCC1742.01	0.5813	3.651	0.235599677	35.88	45.23	6.875	3.875
SPAC8F11.10C	pvg1	0.5828	3.651	0.234480457	17.78	19.82	6.252	4.441
SPBC4C3.08	mug136	0.1742	3.655	0.758951849	34.97	43.94	2.623	1.346
SPAC23G3.05C	SPAC23G3.05c	0.5289	3.655	0.276626433	33.7	42.15	6.322	3.016
SPBC947.10	dsc1	0.6547	3.658	0.183957659	18.05	20.2	6.814	6.048
SPBC27B12.10C	tom7	0.6805	3.659	0.16717187	18.56	20.91	7.541	6.516
SPAC12G12.07C	SPAC12G12.07c	0.5339	3.663	0.272540079	34.02	42.59	6.125	3.399
SPBC1778.01C	zuo1	0.4742	3.665	0.32403845	17.57	19.51	4.396	3.867
SPBC215.11C	SPBC215.11c	0.4224	3.672	0.37427609	41.27	52.75	5.146	1.997
SPAC23D3.11	ayr1	0.3119	3.673	0.505984625	40.5	51.67	3.922	1.677
SPBC19F8.06C	meu22	0.3517	3.677	0.453827632	39.66	50.49	3.552	2.619
SPCC757.11C	SPCC757.11c	0.3384	3.683	0.470569646	38.48	48.82	4.226	1.706
SPCC622.17	apn1	0.3241	3.69	0.489320969	35.32	44.38	2.326	2.747
SPAC31G5.09C	spk1	0.5177	3.691	0.285921835	35.21	44.22	5.796	3.722
SPAC11D3.07C	SPAC11D3.07c	0.4287	3.693	0.367846516	39.98	50.92	4.318	3.079
SPAC21E11.05C	cyp8	0.3453	3.694	0.461803422	39.89	50.78	3.808	2.367
SPAC6F6.11C	SPAC6F6.11c	0.4112	3.698	0.385946894	38.4	48.69	3.178	3.464
SPAC1F8.02C	SPAC1F8.02c	0.4826	3.704	0.316412682	22.32	26.12	6.293	1.586
SPAC24H6.10C	SPAC24H6.10c	0.4675	3.705	0.330218385	37.73	47.74	4.815	3.323
SPAC1805.14	SPAC1805.14	0.4834	3.706	0.315693354	36.15	45.53	6.076	2.184
SPAPB17E12.08	SPAPB17E12.08	0.3624	3.708	0.440811811	27.95	34.02	3.039	3.036
SPAC6G9.03C	mug183	0.5615	3.714	0.250650239	37.51	47.42	6.815	3.546
SPAC4F10.02	aap1	0.1439	3.716	0.841939206	35.41	44.48	1.455	1.682
SPAC3H1.11	hsr1	0.1975	3.716	0.7044329	35.45	44.53	0.6961	1.99
SPAC869.08	pcm2	0.4276	3.717	0.368962303	40.4	51.47	5.58	1.203
SPBC3H7.11	SPBC3H7.11	0.5081	3.724	0.294050805	36.94	46.6	5.986	3.078
SPAC1805.05	cki3	0.2998	3.725	0.523168371	39.79	50.61	3.371	2.236
SPAC11E3.05	SPAC11E3.05	0.3209	3.725	0.493630283	26.29	31.67	2.364	2.915
SPCC417.02	dad5	0.7645	3.73	0.11662251	26.57	32.05	10.82	9.044
SPAC1002.14	itt1	0.3606	3.733	0.442974278	33.1	41.21	3.059	2.895
SPBC651.07	EMPTY	0.366	3.733	0.436518915	37.33	47.14	3.202	3.031
SPAC977.14C	SPAC977.14c	0.2488	3.734	0.604149624	28.35	34.54	2.693	2.082
SPAPB1E7.05	gde1	0.1237	3.736	0.9076303	34.3	42.89	0.9365	1.561
SPCC1739.15	wtf21	0.1405	3.736	0.852323676	4.842	1.552	1.552	1.821
SPBC543.05C	SPBC543.05c	0.4581	3.739	0.339039708	37.16	46.89	5.822	1.958
SPAC3H5.08C	SPAC3H5.08c	0.3317	3.742	0.479254528	39.13	49.65	3.905	2.165
SPBC15D4.15	pho2	0.3306	3.745	0.480697151	39.01	49.49	4.197	1.746
SPAC664.04C	rps1602	0.4576	3.747	0.339513984	33.12	41.21	5.407	2.658
SPAC7D4.05	SPAC7D4.05	0.3278	3.753	0.484391051	41.05	52.33	4.12	1.841
SPBC32H8.13C	mok12	0.7141	3.755	0.146240967	31.83	39.4	9.881	6.778
SPAC3C7.09	set8	0.3666	3.76	0.435807539	34.85	43.62	2.932	3.019
SPAC11G7.02	pub1	0.2088	3.762	0.680269506	26.82	32.36	1.874	2.232
SPAC17G8.09	shg1	0.3758	3.766	0.425043224	28.47	34.66	2.627	3.185
SPAC24B11.13	hem3	0.3665	3.77	0.435926021	37.96	47.98	4.71	1.663
SPCC1393.09C	SPCC1393.09c	0.365	3.772	0.437707136	40.05	50.9	4.201	2.395

SPBC887.15C	sur2	0.7496	3.782	0.125170422	25.03	29.82	10.15	8.677
SPAC589.11	mug82	0.1992	3.788	0.700710666	30.18	37.03	3.225	0.736
SPAC15A10.06	SPAC15A10.06	0.3832	3.791	0.4165745	6.371	3.621	2.452	3.505
SPAC12G12.15	sif3	0.2755	3.792	0.559878397	32.41	40.16	3.31	2.007
SPBC337.03	SPBC337.03	0.1798	3.795	0.745210313	34.57	43.19	1.597	1.935
SPBC32H8.05	SPBC32H8.05	0.473	3.799	0.325138859	37.54	47.34	5.968	2.38
SPBC4F6.09	str1	0.4234	3.804	0.373249146	37.9	47.84	5.254	2.226
SPAC14C4.08	mug5	0.4036	3.806	0.394048842	34.46	43.01	3.155	3.358
SPCC70.08C	SPCC70.08c	0.3265	3.807	0.486116814	36.42	45.76	3.601	2.472
SPBC776.04	sec2302	0.4014	3.808	0.396422632	34.82	43.51	3.249	3.309
SPAC6G9.14	SPAC6G9.14	0.5723	3.812	0.242376254	29.75	36.39	5.494	4.854
SPBC725.09C	hob3	0.7302	3.815	0.136558171	21.81	25.25	9.654	7.993
SPAC14C4.11	SPAC14C4.11	0.4203	3.816	0.37644061	40.88	52.01	2.621	3.861
SPAC14C4.15C	SPAC14C4.15c	0.2602	3.825	0.584692708	32.52	40.27	2.537	2.314
SPAC1805.16C	SPAC1805.16c	0.4439	3.827	0.352714855	34.71	43.33	2.708	3.897
SPBC887.02	SPBC887.02	0.1222	3.83	0.912928794	33.92	42.22	1.908	1.498
SPCC550.15C	SPCC550.15c	0.333	3.83	0.477555766	36.41	45.72	3.822	2.405
SPAPB1A10.14	SPAPB1A10.14	0.7313	3.833	0.135904427	21.94	25.41	11.29	6.965
SPAC25B8.04C	mss51	0.2309	3.838	0.636576067	38.72	48.95	3.429	1.336
SPAC23C11.13C	hpt1	0.6543	3.841	0.18422308	25.67	30.63	7.203	6.316
SPBC1271.06C	mug96	0.2907	3.843	0.536554968	33.97	42.27	1.878	2.687
SPBC16E9.15	SPBC16E9.15	0.4515	3.843	0.345342245	40.12	50.9	3.064	4.128
SPBC21C3.13	rps1901	0.7182	3.843	0.143754599	23.4	27.45	8.627	8.045
SPBC18E5.08	SPBC18E5.08	0.3053	3.847	0.515273196	34.8	43.43	2.795	2.612
SPBP8B7.10C	SPBP8B7.10c	0.3501	3.849	0.455807889	36.08	45.22	4.051	2.457
SPAC27F1.10	SPAC27F1.10	0.5215	3.851	0.282745687	39.91	50.59	6.252	3.452
SPBC106.07C	SPBC106.07c	0.3944	3.855	0.404063094	12.65	12.34	4.507	2.697
SPAC56F8.06C	alg10	0.3105	3.857	0.507938395	33.09	41.02	3.33	2.546
SPBC36.07	iki3	0.08115	3.863	1.090711476	27.42	33.06	1.762	1.349
SPAPYUK71.03C	syn1	0.6574	3.864	0.1821703	33.91	42.16	8.417	5.712
SPAC2F7.04	pmc2	0.2315	3.867	0.635449005	7.82	5.546	2.157	2.643
SPAC3G6.03C	SPAC3G6.03c	0.4344	3.868	0.362110183	38.24	48.22	4.894	3.018
SPBC685.06	rps001	0.6823	3.88	0.166024629	18.9	21.08	7.848	7.053
SPAC11H11.01	sst6	0.2361	3.887	0.626904013	35.71	44.65	0.8192	2.351
SPBC26H8.13C	SPBC26H8.13c	0.2883	3.887	0.540155358	37.71	47.45	3.216	2.424
SPCC622.14	SPCC622.14	0.4112	3.889	0.385946894	39.81	50.4	5.113	2.377
SPAC343.04C	SPAC343.04c	0.2929	3.901	0.533280628	34.16	42.45	1.724	2.76
SPAC4A8.09C	cwf21	0.3142	3.901	0.502793819	37.79	47.56	4.066	1.98
SPBC947.03C	naa38	0.3061	3.903	0.51413667	39.6	50.09	2.256	2.987
SPAC3H5.12C	rpl501	0.6697	3.903	0.174119701	20.11	22.75	7.662	6.76
SPAC222.14C	SPAC222.14c	0.2634	3.914	0.579384229	36.01	45.04	3.84	1.35
SPBC32H8.01C	SPBC32H8.01c	0.3899	3.916	0.409046765	40.98	52	4.229	2.962
SPAC22F3.12C	rgs1	0.7758	3.916	0.110250225	31.22	38.3	13.18	9.196
SPBC1683.10C	pcl1	0.6005	3.917	0.221486988	30.61	37.46	8.792	2.972
SPBC14C8.09C	SPBC14C8.09c	0.5611	3.923	0.250959731	41.84	53.2	5.097	5.191
SPBC1773.06C	SPBC1773.06c	0.2933	3.925	0.532687937	38.11	47.97	3.633	2.204
SPBC31F10.08	mde2	0.2783	3.931	0.555486794	34.65	43.11	2.446	2.567
SPAC30C2.04	SPAC30C2.04	0.3129	3.936	0.504594437	27.98	33.73	2.059	3.114
SPCC417.09C	SPCC417.09c	0.3633	3.939	0.439734602	42.77	54.48	4.366	2.496
SPAC1142.08	fhl1	0.7038	3.939	0.152550738	26.56	31.74	9.821	6.967
SPAC20H4.11C	rho5	0.5761	3.942	0.239502125	42.07	53.5	4.579	5.706
SPBC146.12	coq6	0.4632	3.944	0.334231449	39.93	50.5	4.579	3.823
SPAC1071.06	arp9	0.3094	3.949	0.509479691	36.15	45.18	2.017	2.895
SPCC74.03C	ssp2	0.3071	3.954	0.512720184	43.99	56.18	4.184	1.773
SPAC4H3.05	srs2	0.7589	3.96	0.119815447	29.93	36.44	12.56	8.474
SPAPB1A11.03	SPAPB1A11.03	0.3755	3.962	0.425390059	41.32	52.42	4.624	2.461
SPBC106.08C	mug2	0.3814	3.963	0.418619311	40.15	50.78	4.346	2.827
SPAC22H10.04	SPAC22H10.04	0.2564	3.967	0.591081979	39.43	49.76	3.153	2.226
SPAC13G6.03	gpi7	0.5274	3.97	0.277859875	41.92	53.25	4.101	5.046
SPAC19A8.02	SPAC19A8.02	0.303	3.972	0.518557371	41.32	52.4	3.684	2.349
SPBC3E7.02C	hsp16	0.2877	3.976	0.541060138	38.46	48.38	3.222	2.516
SPAC139.04C	fap2	0.3069	3.978	0.513003112	34.54	42.88	2.064	2.893
SPBCPT2R1.03	SPBCPT2R1.03	0.3976	3.98	0.400553624	34.27	42.51	2.744	3.581
SPAC6B12.09	trm10	0.4124	3.981	0.384681343	40.74	51.58	3.04	3.864
SPAC23A1.04C	mnl1	0.3106	3.982	0.507798549	35.15	43.73	2.879	2.755
SPCP1E11.03	mug170	0.3571	3.984	0.44721015	35.65	44.43	2.617	3.229
SPAC15A10.07	SPAC15A10.07	0.5741	3.989	0.241012453	29.92	36.39	7.828	3.622
SPAC4C5.02C	ryh1	0.1609	3.99	0.793443956	19.83	22.23	2.716	1.548
SPCC364.03	rpl1702	0.1846	3.992	0.733768303	31.1	38.04	2.339	2.059

SPAC14C4.07	SPAC14C4.07	0.2155	3.992	0.666552726	35.9	44.78	2.241	2.189
SPAC25B8.15C	SPAC25B8.15c	0.3742	4	0.426896217	39.87	50.33	4.014	3.028
SPAC513.04	SPAC513.04	0.3724	4.005	0.428990328	41.22	52.21	4.266	2.83
SPCC417.12	SPCC417.12	0.3299	4.015	0.481617684	34.75	43.13	2.506	3.043
SPBPB8B6.05C	SPBPB8B6.05c	0.3932	4.016	0.405386491	38.45	48.31	4.413	3.029
SPAC31A2.16	gef2	0.2611	4.022	0.583193128	33.98	42.04	2.487	2.494
SPAPB1A10.15	arv1	0.4216	4.023	0.375099398	38.2	47.96	5.461	2.454
SPCC965.13	SPCC965.13	0.5086	4.027	0.293623644	41.32	52.33	5.754	4.004
SPCC895.05	for3	0.5898	4.031	0.229295232	17.48	18.88	5.447	5.853
SPAC23C11.01	SPAC23C11.01	0.252	4.032	0.598599459	33.85	41.84	2.697	2.363
SPAC1687.21	SPAC1687.21	0.3709	4.033	0.430743167	38.26	48.02	4.125	2.959
SPAC30.03C	tsn1	0.3495	4.035	0.45655282	32.91	40.51	3.846	2.88
SPAP14E8.04	oma1	0.1878	4.038	0.726304412	34.13	42.22	1.048	2.117
SPAC1002.06C	bqt2	0.4805	4.043	0.318306608	40.3	50.88	5.522	3.659
SPAC167.07C	SPAC167.07c	0.2385	4.044	0.622511617	21.95	25.12	2.384	2.362
SPAC922.03	SPAC922.03	0.3035	4.06	0.517841305	35.16	43.64	2.725	2.808
SPCC1494.07	SPCC1494.07	0.5334	4.063	0.272946989	41	51.83	7.062	3.454
SPCC613.01	SPCC613.01	0.3368	4.068	0.472627917	37.11	46.36	4.585	1.992
SPCC11E10.03	mug1	0.3126	4.075	0.505011026	33.96	41.93	2.339	2.978
SPBP8B7.24C	atg8	0.4564	4.08	0.340654364	39.3	49.43	5.424	3.355
SPBC23E6.10C	SPBC23E6.10c	0.2048	4.084	0.688670048	35.73	44.41	0.8462	2.245
SPCC285.14	trs130	0.6193	4.084	0.20809892	18.29	19.93	6.71	6.129
SPBC13G1.14C	SPBC13G1.14c	0.7697	4.088	0.113678513	29.88	36.19	12.44	9.967
SPBC30D10.18C	rpl102	0.4302	4.092	0.366329594	36.02	44.8	2.185	4.107
SPBC16A3.10	SPBC16A3.10	0.2249	4.094	0.648010545	37.02	46.2	3.041	2.003
SPBC19G7.16	iws1	0.5446	4.096	0.263922363	18.64	20.41	5.719	5.174
SPBC3E7.06C	fnx2	0.33	4.1	0.48148606	40.73	51.39	4.052	2.567
SPCC1450.06C	grx3	0.3389	4.1	0.469928431	38.71	48.56	3.471	3.056
SPAC3A11.06	mvp1	0.4039	4.101	0.393726147	37.05	46.23	5.391	2.335
SPBC29A10.05	exo1	0.1745	4.107	0.758204569	35.24	43.68	2.475	1.865
SPAC6B12.02C	mus7	0.3785	4.107	0.421934116	41.76	52.83	4.243	3.084
SPAC227.13C	isu1	0.4685	4.107	0.329290405	37.96	47.5	4.919	3.979
SPAC29A4.02C	SPAC29A4.02c	0.3908	4.109	0.408045445	36.05	44.82	3.057	3.581
SPAC3F10.15C	spo12	0.2509	4.11	0.600499339	30.05	36.39	0.6111	2.586
SPAC222.12C	atp2	0.2776	4.128	0.556580538	33.27	40.89	2.711	2.651
SPBC17D11.08	SPBC17D11.08	0.282	4.129	0.549750892	35.34	43.79	0.6498	2.842
SPBC216.05	rad3	0.1806	4.131	0.743282254	35.37	43.84	3.214	1.299
SPAC12B10.12C	rhp41	0.4351	4.144	0.361410917	36.04	44.75	3.319	4.04
SPAPB17E12.14C	SPAPB17E12.14c	0.1992	4.149	0.700710666	37.5	46.79	1.548	2.262
SPAC1786.02	SPAC1786.02	0.7354	4.15	0.133476374	26.35	31.15	10.52	8.99
SPAC4F10.19C	SPAC4F10.19c	0.1449	4.151	0.838931615	28.96	34.81	2.595	1.641
SPBC725.04	SPBC725.04	0.3911	4.151	0.407712184	35.53	44.04	2.867	3.669
SPAC15A10.15	sgo2	0.7042	4.16	0.152303979	29.69	35.82	10.5	7.285
SPCC548.05C	SPCC548.05c	0.5476	4.162	0.261536561	52.23	67.45	3.812	5.771
SPAC1327.01C	SPAC1327.01c	0.3526	4.167	0.452717692	30.43	36.85	3.477	3.279
SPAC4F10.05C	SPAC4F10.05c	0.1014	4.168	0.993962045	5.392	1.718	1.386	1.794
SPCC162.12	tco89	0.6464	4.168	0.189498652	21.36	24.13	8.737	5.98
SPCP20C8.01C	SPCP20C8.01c	0.6488	4.17	0.187889159	44.6	56.72	9.657	5.279
SPAPB1E7.06C	eme1	0.5421	4.172	0.265920593	14.67	14.73	5.185	5.241
SPBC8E4.03	SPBC8E4.03	0.4249	4.175	0.371713269	34.3	42.26	5.53	2.809
SPAC56F8.05C	mug64	0.4448	4.175	0.351835221	35.55	44.02	6.157	2.405
SPBC839.13C	rpl1601	0.4274	4.178	0.369165482	28.56	34.21	5.122	3.276
SPCC18.09C	SPCC18.09c	0.3674	4.18	0.434860848	38.77	48.54	3.798	3.309
SPCC1183.02	SPCC1183.02	0.367	4.181	0.435333936	35.01	43.26	2.42	3.536
SPBC211.06	gfh1	0.3243	4.182	0.489053051	38.03	47.49	4.377	2.28
SPAC630.09C	mug58	0.475	4.188	0.32330639	37.68	46.99	6.357	3.017
SPBC36B7.05C	SPBC36B7.05c	0.2966	4.192	0.527828853	37.8	47.16	4.331	1.829
SPCC1682.15	mug122	0.6303	4.201	0.200452693	40.33	50.69	7.586	6.268
SPAC3C7.04	SPAC3C7.04	0.1352	4.202	0.869023308	26.91	31.86	2.094	1.898
SPCC31H12.02C	mug73	0.2331	4.204	0.632457726	37.55	46.79	2.029	2.699
SPCC1739.10	mug33	0.6172	4.206	0.209574083	30.9	37.46	9.19	4.467
SPAC22A12.11	dak1	0.4912	4.209	0.308741642	38.6	48.25	6.77	2.975
SPBC31F10.12	SPBC31F10.12	0.1917	4.211	0.717377887	37.86	47.21	3.246	1.61
SPBC1105.04C	cbp1	0.2728	4.211	0.564155634	40.46	50.86	3.075	2.696
SPAC343.06C	SPAC343.06c	0.4049	4.213	0.392652223	39.32	49.26	4.772	3.249
SPBC409.11	meu18	0.1696	4.216	0.770574152	31.8	38.7	2.248	1.986
SPBC800.04C	rpl4301	0.3406	4.218	0.467755356	6.052	2.573	2.573	3.513
SPAC22F8.12C	shf1	0.4527	4.218	0.344189506	29.56	35.55	5.786	3.224
SPCC1442.16C	zta1	0.2491	4.219	0.603626272	36.15	44.81	2.104	2.62

SPAC144.05	SPAC144.05	0.212	4.22	0.673664139	34.48	42.45	1.801	2.379
SPAC1786.04	SPAC1786.04	0.3013	4.221	0.521000868	41.43	52.21	4.249	2.109
SPAC328.09	SPAC328.09	0.2892	4.229	0.538801711	38.84	48.57	3.117	2.855
SPAC19B12.10	sst2	0.212	4.23	0.673664139	32.4	39.53	3.131	2.043
SPAC1834.03C	hhf1	0.2668	4.231	0.573814175	35.08	43.28	1.046	2.801
SPAC1D4.01	SPAC1D4.01	0.607	4.231	0.216811309	18.6	20.16	6.826	6.068
SPAC29A4.19C	cta5	0.2817	4.232	0.550213153	37.46	46.62	3.104	2.79
SPAC1805.03C	trm13	0.326	4.24	0.4867824	39.28	49.16	3.773	2.911
SPBC1683.08	ght4	0.3755	4.241	0.425390059	40.26	50.54	4.655	2.93
SPBC1778.02	rap1	0.2587	4.242	0.587203571	37.37	46.48	3.935	1.818
SPCC31H12.04C	rpl1202	0.5734	4.246	0.241542311	27.03	31.97	7.568	4.655
SPAC31G5.18C	sde2	0.1423	4.247	0.8467951	35.02	43.18	1.438	2.136
SPBC3D6.13C	pdi2	0.3771	4.25	0.423543468	39.71	49.75	5.298	2.204
SPCPB1C11.01	amt1	0.3186	4.251	0.496754229	33.31	40.77	2.874	3.071
SPBC1E8.02	SPBC1E8.02	0.4161	4.256	0.380802284	40.1	50.29	5.79	2.434
SPCC338.16	pof3	0.6484	4.265	0.188156994	17.42	18.45	7.634	6.996
SPBC19G7.03C	rps3002	0.2236	4.271	0.650528201	35.17	43.35	3.466	1.914
SPBC405.07	rpl3602	0.6939	4.272	0.158703113	23.47	26.94	9.919	7.546
SPAC186.01	SPAC186.01	0.2397	4.286	0.620331966	39.79	49.81	3.324	2.273
SPAC23H4.01C	SPAC23H4.01c	0.4221	4.287	0.374584648	38.42	47.89	3.292	4.271
SPCC18.02	SPCC18.02	0.2462	4.291	0.608711951	34.97	43.04	1.85	2.673
SPAC26H5.05	SPAC26H5.05	0.6355	4.291	0.196884445	23.36	26.75	8.321	6.226
SPAC22H10.11C	SPAC22H10.11c	0.112	4.305	0.950781977	16.34	16.88	1.692	1.892
SPBC17A3.09C	aim22	0.3747	4.306	0.426316307	24.47	28.3	4.762	2.93
SPCC895.07	alp14	0.3856	4.316	0.413862975	39.29	49.08	4.183	3.508
SPAC27E2.01	SPAC27E2.01	0.2435	4.324	0.613501034	38.54	48.02	3.645	2.077
SPBPB2B2.11	SPBPB2B2.11	0.3975	4.331	0.400662867	35.25	43.39	5.36	2.773
SPAC31G5.17C	rps1001	0.1864	4.333	0.729554092	33.31	40.66	1.747	2.263
SPBC15D4.06	naa30	0.3162	4.336	0.500038134	36.58	45.25	3.823	2.755
SPBC428.04	apq12	0.4026	4.338	0.395126229	40.12	50.21	5.663	2.495
SPAC26H5.03	pcf2	0.3858	4.343	0.413637777	36.09	44.55	4.635	3.264
SPBC365.01	SPBC365.01	0.496	4.344	0.304518324	40.53	50.77	4.835	4.856
SPCPB16A4.02C	SPCPB16A4.02c	0.471	4.354	0.326979093	31.54	38.14	6.092	3.613
SPAC1782.09C	clp1	0.2479	4.358	0.605723473	39.88	49.84	3.44	2.362
SPAC13G6.02C	rps101	0.1909	4.362	0.719194072	30.95	37.3	2.51	2.326
SPAC3A11.14C	pkl1	0.5236	4.37	0.281000362	41.89	52.64	7.216	3.848
SPAC15A10.08	ain1	0.2002	4.371	0.698535927	39.41	49.16	3.679	1.173
SPCC4G3.11	mug154	0.2343	4.373	0.630227711	32.76	39.83	2.299	2.581
SPCC5E4.05C	SPCC5E4.05c	0.3164	4.375	0.499763525	34.62	42.43	3.175	3.078
SPAC688.14	set13	0.3046	4.376	0.516270101	34.52	42.3	2.857	3.057
SPCC1840.03	sal3	0.6021	4.389	0.220331373	21.66	24.23	8.142	5.473
SPAC17H9.14C	SPAC17H9.14c	0.325	4.404	0.488116639	35.2	43.21	2.546	3.327
SPAC1296.05C	SPAC1296.05c	0.2369	4.406	0.625434939	36.44	44.95	1.273	2.684
SPBC29A3.05	vps71	0.1364	4.41	0.86518563	36.12	44.5	2.868	1.488
SPBC31F10.03	SPBC31F10.03	0.1965	4.418	0.706637445	36.53	45.05	1.329	2.388
SPAC17A5.08	SPAC17A5.08	0.7267	4.418	0.13864484	25	28.88	11.78	8.657
SPAC26F1.05	mug106	0.2872	4.437	0.541815564	38.82	48.25	2.131	3.077
SPBP16F5.05C	SPBP16F5.05c	0.5312	4.443	0.274741934	23.29	26.45	6.812	4.599
SPCC11E10.04	ppr6	0.4597	4.452	0.337525496	36.92	45.56	3.438	4.924
SPBC106.16	pre6	0.2586	4.458	0.587371479	34.64	42.35	2.063	2.863
SPBC1271.05C	SPBC1271.05c	0.4318	4.462	0.364717362	28.94	34.35	4.867	3.993
SPCC306.09C	cap1	0.1226	4.464	0.91150953	24.11	27.57	1.713	2.058
SPCC126.04C	sgf73	0.2795	4.465	0.553618188	36.62	45.12	4.321	2.055
SPAC11D3.06	SPAC11D3.06	0.2537	4.469	0.595679533	41.09	51.39	3.466	2.535
SPBC1773.01	SPBC1773.01	0.2951	4.478	0.530030791	37.24	45.97	4.159	2.528
SPBC365.02C	cox10	0.1036	4.484	0.984640245	35.76	43.89	0.7627	1.967
SPBC23G7.15C	rpp202	0.2954	4.484	0.529589509	38.47	47.68	2.736	3.292
SPAC57A7.09	SPAC57A7.09	0.3061	4.487	0.51413667	40.97	51.19	3.598	3.07
SPAC17A5.07C	ulp2	0.3064	4.487	0.513711239	35.88	44.04	3.802	2.957
SPCC24B10.15	SPCC24B10.15	0.4149	4.488	0.382056565	41.33	51.7	4.385	4.034
SPAC17H9.10C	ddb1	0.5592	4.488	0.252432837	26.48	30.86	7.464	4.937
SPAC1B3.17	clr2	0.5366	4.492	0.270349332	42.92	53.92	3.335	6.191
SPBC428.07	meu6	0.3331	4.493	0.477425367	39.17	48.65	4.314	2.961
SPAC16A10.04	rho4	0.394	4.501	0.404503778	43.95	55.35	5.042	3.333
SPBC1734.09	SPBC1734.09	0.3278	4.51	0.484391051	34.87	42.6	2.086	3.492
SPCC1795.09	yps1	0.2593	4.513	0.586197483	37.41	46.16	2.491	3.053
SPAC1F12.02C	SPAC1F12.02c	0.4621	4.524	0.335264031	33.44	40.57	6.211	3.664
SPAC664.13	SPAC664.13	0.1218	4.528	0.914352712	33.54	40.7	1.938	1.856
SPBC18H10.09	SPBC18H10.09	0.523	4.533	0.281498311	31.96	38.49	7.213	4.25

SPAC1687.22C	puf3	0.2395	4.544	0.620694482	33.46	40.58	1.806	2.785
SPBC21H7.06C	SPBC21H7.06c	0.2246	4.545	0.648590248	41.7	52.13	3.535	2.21
SPAC29E6.10C	SPAC29E6.10c	0.2942	4.55	0.531357332	26.79	31.2	2.99	3.265
SPAC139.03	SPAC139.03	0.2596	4.552	0.585695312	42.35	53.03	4.359	1.717
SPCPB16A4.04C	trm8	0.2271	4.573	0.643782866	33.66	40.81	3.667	2.151
SPBC8D2.16C	SPBC8D2.16c	0.2247	4.574	0.648396928	34.88	42.52	1.777	2.689
SPAC1071.05	SPAC1071.05	0.2311	4.574	0.636200055	28.67	33.81	3.961	1.843
SPAC1834.05	alg9	0.1823	4.576	0.739213331	34.42	41.87	3.285	1.905
SPBC651.06	mug166	0.7279	4.577	0.137928281	31.2	37.36	14.03	7.516
SPAC644.14C	rhp51	0.3075	4.584	0.51215488	39.87	49.52	4.156	2.852
SPAC977.05C	SPAC977.05c	0.2927	4.587	0.533577278	35.33	43.13	3.463	3.095
SPCC736.09C	SPCC736.09c	0.1479	4.594	0.830031826	42.45	53.11	2.37	2.161
SPBC16C6.04	SPBC16C6.04	0.7264	4.594	0.138824164	28.23	33.16	11.19	9.631
SPBC2G5.01	SPBC2G5.01	0.2418	4.598	0.616543703	36.56	44.84	2.894	2.662
SPBPB2B2.08	SPBPB2B2.08	0.3413	4.6	0.466863712	36.35	44.55	5.38	2.01
SPBC342.05	crb2	0.3476	4.6	0.458920232	36.33	44.52	2.582	3.705
SPAC31A2.11C	cuf1	0.1741	4.604	0.759201229	40.85	50.86	2.375	2.405
SPCC1840.07C	SPCC1840.07c	0.2771	4.606	0.557363474	38.94	48.18	3.741	2.781
SPAC1F3.06C	spo15	0.4065	4.608	0.39093945	37.49	46.14	1.541	4.404
SPAC1006.04C	mcp3	0.2686	4.612	0.570893992	35.52	43.36	2.681	2.967
SPBC11C11.08	srp1	0.5068	4.613	0.295163394	46.01	58.08	4.746	5.496
SPAC4G9.20C	SPAC4G9.20c	0.3421	4.614	0.465846926	34.13	41.41	2.592	3.663
SPBC365.06	pmt3	0.3188	4.615	0.496481687	34.93	42.53	2.53	3.448
SPBC1709.05	sks2	0.2543	4.62	0.59465364	33.7	40.81	2.313	2.912
SPAC589.06C	SPAC589.06c	0.2567	4.623	0.590574131	31.45	37.65	2.148	2.952
SPBC1604.18C	SPBC1604.18c	0.1881	4.631	0.725611204	35.78	43.71	2.923	2.315
SPCC1494.08C	SPCC1494.08c	0.3302	4.634	0.481222931	29.26	34.55	4.177	3.203
SPAC15E1.09	grx2	0.2884	4.642	0.540004744	31.89	38.23	4.428	2.389
SPBC16C6.09	ogm4	0.3841	4.644	0.415555693	33.28	40.18	4.927	3.485
SPBC1539.06	SPBC1539.06	0.5459	4.644	0.262886906	29.97	35.54	5.144	5.912
SPBC4B4.08	ght2	0.3464	4.65	0.460422117	37.07	45.49	2.3	3.768
SPBC660.17C	SPBC660.17c	0.3284	4.652	0.483596852	40.23	49.92	4.804	2.686
SPAC57A10.14	sgf11	0.4494	4.665	0.347366932	26.03	29.98	4.762	4.433
SPAC922.07C	SPAC922.07c	0.3124	4.673	0.505288975	34.24	41.49	2.75	3.403
SPCC13B11.03C	SPCC13B11.03c	0.3307	4.678	0.480565805	34.66	42.07	2.354	3.634
SPAC664.12C	SPAC664.12c	0.1386	4.679	0.85823677	41.29	51.37	3.174	1.392
SPBP22H7.04	SPBP22H7.04	0.1888	4.681	0.72399801	42.88	53.59	3.515	1.869
SPBC365.12C	ish1	0.2315	4.684	0.635449005	35.33	43.01	2.853	2.648
SPBC577.02	rpl3801	0.6808	4.702	0.166980453	25.4	29.04	10.03	8.194
SPBC8D2.17	SPBC8D2.17	0.7336	4.708	0.134540677	29.68	35.03	11.84	10.13
SPAC18G6.13	SPAC18G6.13	0.14	4.709	0.853871964	32.57	39.09	2.214	2.043
SPAC1556.05C	cgr1	0.3712	4.71	0.430392032	32.5	38.99	4.827	3.453
SPCC825.05C	SPCC825.05c	0.5244	4.714	0.280337317	22.09	24.38	7.112	4.791
SPAC1F5.05C	SPAC1F5.05c	0.3231	4.715	0.490663042	39.28	48.5	3.55	3.527
SPAC30D11.14C	SPAC30D11.14c	0.2632	4.717	0.579714115	37.58	46.11	2.721	3.195
SPBP4G3.03	SPBP4G3.03	0.4298	4.721	0.366733589	42.34	52.78	5.027	4.26
SPAC3A12.17C	cys12	0.1211	4.724	0.916855857	36.88	45.11	1.086	1.943
SPAC22F3.06C	lon1	0.3703	4.732	0.431446288	35.87	43.69	3.44	3.908
SPAC13D6.04C	btb3	0.3268	4.735	0.485717952	33.74	40.7	3.791	3.318
SPAC27D7.06	SPAC27D7.06	0.2428	4.758	0.614751318	33.52	40.35	2.606	2.862
SPBC29A10.14	rec8	0.2284	4.761	0.6413039	35.98	43.8	1.228	2.825
SPCC1739.07	cti1	0.2667	4.762	0.573976984	19.41	20.55	3.501	2.968
SPCC4B3.05C	hem12	0.3425	4.778	0.465339424	36.7	44.79	5.175	2.757
SPAC11D3.09	SPAC11D3.09	0.5472	4.781	0.261853911	42.87	53.45	4.826	6.506
SPCC1739.01	SPCC1739.01	0.1293	4.785	0.888401475	6.125	1.88	1.88	2.257
SPCC962.05	ast1	0.1526	4.797	0.816445466	35.71	43.37	1.034	2.216
SPCC1739.04C	SPCC1739.04c	0.4113	4.801	0.38584129	32.59	39	6.212	2.99
SPAC3H5.07	rpl702	0.1953	4.804	0.709297757	25.87	29.55	3.213	2.371
SPAC19A8.03	SPAC19A8.03	0.2478	4.806	0.605898698	31.45	37.38	3.114	2.987
SPAC24H6.08	SPAC24H6.08	0.1676	4.812	0.775725986	34.94	42.27	2.322	2.311
SPBC3B9.06C	atg3	0.2192	4.813	0.65915945	35.12	42.52	1.715	2.788
SPAC16E8.01	shd1	0.4451	4.818	0.351542406	26.59	30.56	6.109	3.97
SPAC22F3.08C	rok1	0.5036	4.821	0.297914279	23.57	26.3	6.331	5.051
SPBC23E6.08	sat1	0.07164	4.83	1.144844423	22.21	24.39	2.165	1.583
SPAC926.02	SPAC926.02	0.25	4.843	0.602059991	36.23	44.04	2.424	3.014
SPAC644.06C	cdr1	0.437	4.852	0.359518563	14.24	13.17	4.407	4.829
SPAC13F5.07C	SPAC13F5.07c	0.3453	4.854	0.461803422	35.17	42.53	1.775	3.959
SPBC18H10.20C	SPBC18H10.20c	0.1663	4.856	0.779107751	39.67	48.85	2.393	2.501
SPBC1709.12	rid1	0.297	4.866	0.527243551	40.94	50.61	3.527	3.399

SPBC20F10.07	SPBC20F10.07	0.458	4.866	0.339134522	30.8	36.39	4.403	4.941
SPCC757.07C	ctt1	0.3202	4.867	0.494578672	33.71	40.46	3.583	3.449
SPAC9G1.10C	SPAC9G1.10c	0.2049	4.869	0.688458042	34.32	41.33	1.286	2.693
SPBC418.01C	his4	0.1666	4.883	0.778325003	38.07	46.57	2.345	2.535
SPBC800.05C	atb2	0.436	4.89	0.360513511	34	40.85	3.776	4.81
SPAC1F5.09C	shk2	0.429	4.893	0.367542708	43.03	53.52	6.295	3.598
SPACUNK4.15	SPACUNK4.15	0.4254	4.905	0.371202514	42.91	53.33	2.924	5.121
SPCC364.04C	SPCC364.04c	0.2142	4.907	0.669180534	45.93	57.56	3.623	2.408
SPAC4G8.13C	prz1	0.7179	4.921	0.143936047	32.62	38.86	12.94	9.153
SPBC354.04	SPBC354.04	0.2292	4.922	0.639785387	41.22	50.92	4.063	2.222
SPAC212.04C	SPAC212.04c	0.2851	4.922	0.545002783	39.96	49.16	4.814	2.313
SPAC4G8.07C	SPAC4G8.07c	0.3362	4.922	0.473402291	35.17	42.44	2.749	3.848
SPBC32F12.01C	css1	0.6687	4.926	0.174768677	27.18	31.22	10.57	7.949
SPBC21D10.10	bdc1	0.1904	4.928	0.720333056	30.8	36.3	2.779	2.646
SPAC630.10	SPAC630.10	0.1015	4.935	0.993533958	26.79	30.67	2.22	1.977
SPCC285.13C	nup60	0.2402	4.946	0.619426997	32.2	38.24	2.448	2.995
SPBC16A3.06	SPBC16A3.06	0.5747	4.948	0.240558803	35.74	43.2	8.417	5.797
SPAC1039.05C	klf1	0.3177	4.951	0.497982785	35.71	43.16	2.482	3.716
SPAC31A2.02	trm112	0.2185	4.968	0.660548559	23.23	25.62	3.26	2.755
SPBC106.04	ada1	0.6555	4.99	0.183427304	24.55	27.45	9.248	8.311
SPBC4.05	mlo2	0.2315	5.001	0.635449005	38.12	46.46	4.41	1.883
SPAC3A12.10	rpl2001	0.4879	5.002	0.311669182	20.89	22.29	7.542	4.063
SPBC19G7.10C	SPBC19G7.10c	0.02977	5.007	1.526221165	24.56	27.43	0.6833	1.38
SPBC530.11C	SPBC530.11c	0.2229	5.008	0.651889932	38.41	46.87	3.51	2.701
SPAC16E8.13	SPAC16E8.13	0.2034	5.01	0.691649051	39.14	47.89	3.602	2.39
SPAC56F8.14C	mug115	0.2581	5.014	0.588211995	32.38	38.39	2.621	3.179
SPBC32F12.08C	duo1	0.5835	5.019	0.23395914	20.32	21.47	7.205	6.927
SPBC4F6.11C	SPBC4F6.11c	0.2483	5.027	0.60502328	35.36	42.56	2.822	3.061
SPBC1289.14	SPBC1289.14	0.3418	5.029	0.466227942	34.91	41.92	4.1	3.677
SPAC1002.17C	urg2	0.246	5.031	0.609064893	33.98	40.62	2.187	3.13
SPBC28F2.11	SPBC28F2.11	0.3047	5.034	0.516127546	37.1	45	3.803	3.54
SPAC16.05C	sfp1	0.005799	5.038	2.236646891	32.41	38.4	0.5013	0.8657
SPAC26A3.04	rpl2002	0.3161	5.045	0.500175504	33.89	40.47	4.478	3.329
SPBC651.04	SPBC651.04	0.3643	5.047	0.438540829	43.98	54.62	5.124	3.621
SPBC3E7.08C	rad13	0.1205	5.053	0.919012953	36.53	44.17	1.672	2.104
SPCC1902.01	gaf1	0.3044	5.054	0.516555352	37.33	45.29	1.974	3.7
SPCC162.01C	SPCC162.01c	0.4357	5.055	0.36081244	43.22	53.54	4.342	5.098
SPAC1D4.09C	rtf2	0.2115	5.056	0.674689628	31.99	37.79	3.968	2.212
SPAC23C11.06C	SPAC23C11.06c	0.7127	5.061	0.147093241	33.09	39.33	13.48	8.909
SPBC776.14	plh1	0.2765	5.065	0.558304864	38.39	46.77	4.856	2.331
SPBC146.11C	mug97	0.08661	5.068	1.062431961	38.88	47.45	1.259	2.071
SPAPB1A10.09	ase1	0.1435	5.078	0.843148099	38.82	47.35	3.629	1.022
SPCC1020.09	gnr1	0.08037	5.089	1.094906032	38.29	46.59	2.043	1.896
SPBC83.19C	SPBC83.19c	0.2667	5.09	0.573976984	35.76	43.03	2.634	3.312
SPAC824.05	vps16	0.5304	5.09	0.275396485	27.58	31.56	7.127	6.058
SPACUNK4.17	SPACUNK4.17	0.3009	5.095	0.521577812	39.33	48.04	5.18	2.466
SPBC336.14C	ppk26	0.09854	5.103	1.006387442	36.9	44.61	0.9714	1.876
SPAC869.03C	SPAC869.03c	0.2811	5.113	0.551139154	37.71	45.74	2.672	3.461
SPAC23H3.04	SPAC23H3.04	0.3167	5.121	0.499351937	40.01	48.95	4.573	3.37
SPBC25H2.14	mug16	0.7268	5.126	0.138585081	33.44	39.72	13.99	9.821
SPAC3H5.09C	SPAC3H5.09c	0.2626	5.129	0.580705278	34.99	41.9	2.502	3.318
SPAC20H4.06C	SPAC20H4.06c	0.3244	5.131	0.488919154	29.63	34.38	3.053	3.856
SPAC1687.06C	rpl44	0.2101	5.135	0.677573948	34.7	41.48	1.716	2.895
SPBP22H7.05C	abo2	0.3198	5.137	0.495121541	37.9	45.97	1.234	3.927
SPAPB1A10.13	SPAPB1A10.13	0.2086	5.139	0.680685696	34.98	41.86	1.082	2.86
SPBC1734.07C	SPBC1734.07c	0.3079	5.141	0.511590311	39.61	48.37	4.856	3.042
SPAC637.13C	SPAC637.13c	0.7138	5.144	0.146423456	50.78	64.03	14.89	8.074
SPBP8B7.22	erd2	0.2148	5.147	0.667965723	37.3	45.12	4.029	2.321
SPAC513.07	SPAC513.07	0.2695	5.157	0.56944123	37.57	45.48	1.877	3.442
SPAC4F10.13C	mpd2	0.5525	5.161	0.257667718	25.92	29.13	8.917	5.159
SPAPB2B4.04C	SPAPB2B4.04c	0.205	5.162	0.688246139	38.84	47.26	2.991	2.702
SPBC1604.08C	imp1	0.05014	5.166	1.29981567	33.22	39.36	1.867	1.617
SPBC16A3.01	spn3	0.2426	5.175	0.615109203	42.84	52.85	3.733	2.971
SPAC26H5.09C	SPAC26H5.09c	0.2148	5.189	0.667965723	35.29	42.24	2.246	2.947
SPBP35G2.13C	swc2	0.1199	5.191	0.921180817	39.86	48.65	3.374	1.253
SPAC22F8.05	SPAC22F8.05	0.2811	5.192	0.551139154	33.3	39.44	4.197	3.203
SPBC2A9.11C	SPBC2A9.11c	0.2068	5.193	0.684449466	37.62	45.5	2.589	2.835
SPCC548.07C	ght1	0.2184	5.197	0.660747366	31.77	37.28	3.873	2.449
SPAC27E2.07	pvg2	0.08618	5.201	1.06459351	34.8	41.53	2.227	1.952

SPCC306.07C	SPCC306.07c	0.2755	5.208	0.559878397	36.07	43.3	3.425	3.321
SPCC1223.13	cbf12	0.05936	5.213	1.226506108	37.51	45.32	2.388	1.301
SPAC11D3.16C	SPAC11D3.16c	0.2172	5.22	0.663140179	35.98	43.17	1.635	3.005
SPAC25H1.03	mug66	0.4029	5.224	0.394802733	41.19	50.46	5.303	4.394
SPAC664.15	caf4	0.2276	5.226	0.642827742	34.6	41.22	1.711	3.103
SPCC188.07	ccq1	0.2718	5.226	0.565750548	27.55	31.32	5.242	1.818
SPCC338.11C	rrg1	0.2817	5.231	0.550213153	34.55	41.13	2.322	3.589
SPBC18E5.04	rpl1001	0.4763	5.247	0.322119418	25.4	28.28	6.473	5.135
SPAC17C9.09C	tim13	0.2687	5.25	0.570732334	34.71	41.34	2.245	3.483
SPBC1683.07	mal1	0.1864	5.267	0.729554092	46.59	57.98	2.979	2.782
SPAC2F7.03C	pom1	0.07739	5.272	1.111315153	27.53	31.22	2.454	1.754
SPAC27D7.04	omt2	0.2089	5.272	0.68006156	36.35	43.61	4.496	1.612
SPBC354.13	rga6	0.2665	5.312	0.574302787	43.26	53.24	4.37	3.018
SPCC13B11.02C	SPCC13B11.02c	0.1583	5.319	0.800519085	36.75	44.09	1.858	2.544
SPAC2C4.09	SPAC2C4.09	0.3664	5.319	0.436044535	37.92	45.74	3.018	4.5
SPCC1919.10C	myo52	0.1153	5.324	0.938170693	31.58	36.85	3.245	1.651
SPAC869.10C	put4	0.2072	5.327	0.683610249	41.02	50.08	4.538	1.575
SPAPB21F2.02	SPAPB21F2.02	0.29	5.342	0.537602002	44.44	54.86	5.163	2.7
SPAC1071.11	SPAC1071.11	0.4371	5.344	0.359419194	23.47	25.43	7.698	3.098
SPAC6F12.04	SPAC6F12.04	0.1645	5.354	0.783834098	35.73	42.63	1.021	2.567
SPAC19G12.04	SPAC19G12.04	0.2718	5.367	0.565750548	34.25	40.53	2.444	3.58
SPAC13F5.05	SPAC13F5.05	0.4324	5.37	0.364114315	40.13	48.78	5.463	5.023
SPCC126.06	twf1	0.2549	5.383	0.593630165	33.09	38.88	3.049	3.339
SPAC8C9.07	SPAC8C9.07	0.2952	5.393	0.529883647	28.87	32.94	4.942	3.107
SPAC1071.12C	stp1	0.275	5.395	0.560667306	39.8	48.27	3.465	3.676
SPAC22A12.04C	rps2201	0.3828	5.395	0.417028071	28.61	32.57	6.027	3.782
SPBC27.02C	ask1	0.191	5.413	0.718966633	36.32	43.37	3.939	2.345
SPCC320.03	SPCC320.03	0.01876	5.423	1.726767166	38.3	46.13	1.834	0.8287
SPAC1805.02C	SPAC1805.02c	0.08348	5.424	1.07841756	41.52	50.64	2.379	1.976
SPAC30.01C	sec72	0.4106	5.426	0.386581055	40.06	48.6	5.989	4.408
SPAC13G7.03	upf3	0.1716	5.428	0.765482716	34.85	41.28	3.868	2.096
SPBC16E9.11C	pub3	0.3294	5.428	0.482276405	38.51	46.41	4.326	3.845
SPCC1183.11	SPCC1183.11	0.4439	5.428	0.352714855	31.54	36.63	5.954	4.881
SPAC2F7.09C	SPAC2F7.09c	0.3199	5.445	0.49498576	36.24	43.21	3.465	4.246
SPAPJ696.01C	vps17	0.5962	5.467	0.224608028	22.49	23.88	8.158	7.803
SPBC839.05C	rps1701	0.5906	5.477	0.228706557	20.62	21.25	9.615	6.782
SPAC8E11.03C	dmc1	0.1178	5.491	0.92885471	37.94	45.53	2.346	2.212
SPBC215.03C	csn1	0.2005	5.491	0.697885623	26.86	29.99	4.036	2.499
SPAC4D7.11	dsc4	0.3335	5.493	0.476904162	16.59	15.57	5.369	3.552
SPCC126.03	pus1	0.245	5.496	0.610833916	29.58	33.8	3.132	3.303
SPBC21C3.19	SPBC21C3.19	0.5068	5.502	0.295163394	32.98	38.56	5.712	6.274
SPAC3G9.05	SPAC3G9.05	0.09706	5.507	1.012959713	36.57	43.59	2.468	1.969
SPBC1734.13	atp3	0.2277	5.507	0.642636969	34.95	41.31	2.01	3.269
SPAC27D7.09C	SPAC27D7.09c	0.4425	5.53	0.354086725	43.98	53.95	5.686	5.328
SPAC8C9.05	SPAC8C9.05	0.2579	5.539	0.588548658	42.49	51.85	4.738	2.85
SPBC13E7.03C	SPBC13E7.03c	0.1717	5.554	0.765229705	36.79	43.84	1.393	2.759
SPAC1B3.01C	SPAC1B3.01c	0.06825	5.556	1.165897344	35.56	42.1	2	1.782
SPAC23H3.12C	SPAC23H3.12c	0.2652	5.569	0.57642648	40.78	49.4	4.417	3.26
SPBC8E4.01C	SPBC8E4.01c	0.1328	5.58	0.876801925	34.68	40.83	2.696	2.326
SPCC1450.16C	SPCC1450.16c	0.2638	5.581	0.578725209	33.82	39.63	2.563	3.639
SPBC18H10.04C	sce3	0.507	5.593	0.294992041	38.2	45.75	8.357	5.223
SPCC1840.12	SPCC1840.12	0.269	5.6	0.57024772	34.83	41.01	1.84	3.735
SPBC1683.06C	SPBC1683.06c	0.1385	5.62	0.858550227	40.6	49.09	1.956	2.783
SPAC27F1.05C	SPAC27F1.05c	0.0179	5.623	1.747146969	38.08	45.54	1.775	1.015
SPCC16C4.13C	rpl1201	0.5173	5.625	0.286257522	19.73	19.8	6.617	6.616
SPCC24B10.13	skb5	0.2931	5.641	0.532984182	36.84	43.77	4.187	3.646
SPAC23C11.14	zhf1	0.1761	5.646	0.754240644	24.9	27.01	4.079	2.2
SPAC13C5.02	dre4	0.3503	5.654	0.455559863	13.63	11.2	4.328	4.575
SPAC1952.15C	rec24	0.06923	5.657	1.159705668	35.39	41.72	0.9525	1.761
SPBC4.06	SPBC4.06	0.1762	5.657	0.753994096	40.64	49.09	2.777	3.022
SPAC3H1.03	mug151	0.2144	5.657	0.668775219	35.86	42.39	1.31	3.212
SPBC2A9.07C	SPBC2A9.07c	0.223	5.658	0.651695137	35.86	42.37	2.216	3.309
SPCC16A11.16C	rpn1302	0.3795	5.663	0.42078822	37.99	45.36	6.803	3.389
SPBC9B6.11C	SPBC9B6.11c	0.1836	5.668	0.736127323	38.69	46.33	2.469	2.918
SPBC15C4.01C	oca3	0.186	5.677	0.730487056	39.69	47.72	2.133	2.97
SPAC13G7.07	arb2	0.1485	5.679	0.828273546	33.91	39.62	2.508	2.817
SPAC2C4.10C	SPAC2C4.10c	0.2725	5.692	0.564633493	35.16	41.35	5.319	2.612
SPCC126.10	iah1	0.2142	5.695	0.669180534	41.83	50.7	4.299	2.713
SPBC651.02	SPBC651.02	0.2354	5.716	0.628193541	37.4	44.46	5.348	1.384

SPAC19B12.12C	yip11	0.2928	5.729	0.533428928	40.33	48.55	5.166	3.329
SPAC11E3.11C	SPAC11E3.11c	0.3741	5.738	0.427012292	28.27	31.62	5.768	4.336
SPCC1281.03C	emc4	0.3491	5.75	0.457050151	41.19	49.72	3.738	4.843
SPAC13G7.02C	ssa1	0.5757	5.756	0.239803771	40.79	49.16	7.201	8.147
SPAC22F8.11	plc1	0.2936	5.762	0.532243949	29.07	32.71	5.034	3.492
SPBC887.17	SPBC887.17	0.2059	5.765	0.686343653	40.35	48.52	3.096	3.325
SPAC22G7.02	kap111	0.2221	5.769	0.653451441	41.65	50.35	1.68	3.366
SPCC1442.05C	SPCC1442.05c	0.3129	5.769	0.504594437	29.44	33.21	5.048	3.804
SPCC4B3.13	SPCC4B3.13	0.1688	5.788	0.772627558	36.35	42.88	2.038	2.868
SPAC23A1.11	rpl1602	0.1846	5.804	0.733768303	37.43	44.38	1.605	3.017
SPCC24B10.10C	SPCC24B10.10c	0.2236	5.807	0.650528201	37.03	43.81	2.293	3.4
SPAC3H1.05	SPAC3H1.05	0.6559	5.808	0.183162369	30.22	34.25	10.45	9.849
SPBC17D11.01	nep1	0.4202	5.824	0.376543952	43.44	52.78	5.148	5.555
SPBP8B7.02	SPBP8B7.02	0.4416	5.826	0.354970935	44.48	54.23	3.999	6.243
SPAC22A12.02C	mug103	0.4629	5.826	0.334512819	45.4	55.53	4.516	6.495
SPAC23G3.08C	ubp7	0.02329	5.831	1.632830511	37.31	44.17	1.426	1.53
SPBC354.07C	SPBC354.07c	0.1669	5.831	0.777543663	37.71	44.73	3.785	2.542
SPBC4F6.16C	ero11	0.2812	5.831	0.550984684	36.66	43.26	2.191	4.017
SPAC9E9.12C	ybt1	0.2059	5.836	0.686343653	37	43.72	2.497	3.228
SPCC16C4.01	sif2	0.2722	5.839	0.565111879	35.81	42.05	2.211	3.924
SPBC17A3.06	SPBC17A3.06	0.05906	5.842	1.228706557	25.9	28.14	2.633	1.659
SPAC57A7.13	SPAC57A7.13	0.1914	5.852	0.718058067	37.94	45.02	2.078	3.117
SPAC688.11	end4	0.2867	5.853	0.542572307	17.89	16.89	4.844	3.624
SPAC328.04	SPAC328.04	0.276	5.866	0.559090918	41.17	49.54	5.469	2.895
SPBC16A3.19	eaf7	0.1593	5.868	0.797784224	41.9	50.55	3.711	2.517
SPAC1851.04C	ric1	0.3617	5.87	0.441651491	18.24	17.36	5.961	4.549
SPBC1709.13C	set10	0.713	5.877	0.14691047	34.32	39.91	13.07	12
SPAC926.05C	dph4	0.08394	5.884	1.076031035	41.87	50.49	2.725	2.073
SPBC359.05	abc3	0.179	5.889	0.747146969	37.6	44.49	3.546	2.918
SPAC30D11.09	cwf19	0.1817	5.891	0.740645073	30.98	35.2	4.003	2.657
SPAC3A11.13	SPAC3A11.13	0.1633	5.901	0.787013815	42.67	51.59	4.184	2.123
SPAC5D6.07C	pxa1	0.1879	5.916	0.72607322	36.31	42.64	2.034	3.117
SPBC11C11.10	SPBC11C11.10	0.3235	5.957	0.490125715	27.42	30.11	4.999	4.029
SPCC1672.12C	get4	0.594	5.978	0.226213555	21.9	22.34	10.68	7.405
SPCC663.12	cid12	0.4638	5.98	0.333669256	23.15	24.09	7.519	5.45
SPCC1223.05C	rpl3702	0.3189	5.987	0.496345481	23.68	24.82	5.01	4.181
SPAC1687.13C	csn5	0.1959	6	0.707965564	37.11	43.66	2.73	3.2
SPBC146.10	mug57	0.1724	6.018	0.763462739	37.61	44.32	2.261	3.013
SPBC19C2.04C	ubp11	0.08648	6.021	1.063084319	34.56	40.05	2.565	2.063
SPCC18B5.10C	SPCC18B5.10c	0.5141	6.025	0.288952396	31.14	35.24	7.093	6.748
SPBPJ4664.06	gpt1	0.2637	6.036	0.57888987	41.74	50.09	5.013	3.343
SPBC11C11.02	imp2	0.2484	6.061	0.604848408	38.76	45.88	3.323	3.708
SPAC14C4.03	mek1	0.251	6.063	0.600326279	34.72	40.21	2.767	3.815
SPAC29A4.09	SPAC29A4.09	0.3781	6.076	0.422393323	11.23	7.226	4.917	5.249
SPAC25B8.03	psd2	0.1052	6.096	0.97798426	33.77	38.84	3.372	2.093
SPAC22H10.13	zym1	0.1617	6.099	0.79128998	36.18	42.21	2.403	2.94
SPAC5D6.13	SPAC5D6.13	0.1127	6.12	0.948076084	35.64	41.42	3.904	1.272
SPAC4F10.14C	btf3	0.02966	6.123	1.527828853	24.42	25.68	0.9225	1.699
SPAC824.04	SPAC824.04	0.07438	6.125	1.128543826	41.96	50.28	3.24	1.462
SPBC30D10.04	swi3	0.07783	6.139	1.10885297	31.78	35.98	1.897	2.381
SPAC23H4.16C	SPAC23H4.16c	0.04677	6.146	1.33003263	35.75	41.53	2.456	1.731
SPAC6B12.04C	SPAC6B12.04c	0.2269	6.184	0.644165504	35.25	40.79	1.629	3.656
SPAPB1E7.07	glt1	0.102	6.185	0.991399828	38.63	45.53	2.934	2.415
SPCC553.12C	SPCC553.12c	0.1395	6.222	0.855425792	33.87	38.79	2.14	2.785
SPBC2F12.15C	pfa3	0.1355	6.224	0.868060705	31.81	35.9	2.118	2.746
SPAC3A12.13C	SPAC3A12.13c	0.1131	6.23	0.946537395	26.14	27.93	3.479	2.26
SPAC57A10.04	mug10	0.1954	6.248	0.709075441	40.13	47.54	2.569	3.665
SPBC359.03C	aat1	0.2573	6.254	0.589560214	37.61	44	1.787	4.037
SPBC24C6.04	SPBC24C6.04	0.2214	6.295	0.654822383	29.84	33.04	4.523	3.124
SPAC1952.07	rad1	0.1106	6.317	0.956244873	35.67	41.18	2.549	2.489
SPBC16C6.05	SPBC16C6.05	0.07545	6.321	1.122340756	37.19	43.31	3.331	1.592
SPCC1223.15C	spc19	0.07797	6.325	1.108072466	37.8	44.16	3.028	2.052
SPBC3H7.13	SPBC3H7.13	0.1035	6.339	0.98505965	37.56	43.81	1.791	2.454
SPBC530.01	gyp1	0.2134	6.339	0.670805585	35.12	40.38	4.704	3.078
SPAC959.08	rpl2102	0.4439	6.353	0.352714855	28.31	30.81	8.813	4.446
SPAC694.05C	rps2502	0.1333	6.366	0.875169851	44.88	54.04	3.378	2.753
SPAC1142.07C	vps32	0.3729	6.383	0.428407617	16.05	13.57	5.61	5.243
SPBC16G5.05C	SPBC16G5.05c	0.2116	6.44	0.674484337	33.98	38.65	4.767	2.944
SPAC1952.09C	SPAC1952.09c	0.2821	6.474	0.549596914	45.79	55.17	4.474	4.393

SPBC1198.12	mfr1	0.1733	6.48	0.761201437	37.38	43.36	2.635	3.551
SPBC359.04C	SPBC359.04c	0.1752	6.481	0.756465898	36.75	42.47	2.569	3.268
SPBC2G5.03	ctu1	0.2343	6.483	0.630227711	39.01	45.64	1.779	4.34
SPAC13D6.01	pof14	0.1532	6.492	0.814741235	36.61	42.25	2.398	3.048
SPBC3H7.06C	pof9	0.08934	6.507	1.048954052	36.71	42.38	3.522	1.946
SPBC18H10.10C	saf4	0.3036	6.527	0.517698233	41.45	49	3.816	4.951
SPCC18.13	SPCC18.13	0.01549	6.529	1.809948582	33.54	37.9	2.078	1.235
SPCC1840.04	pca1	0.2629	6.534	0.580209414	43.2	51.45	6.222	2.657
SPAC23C11.10	SPAC23C11.10	0.1677	6.542	0.775466937	37.28	43.12	2.149	3.23
SPBC1105.11C	hht3	0.1464	6.545	0.834458923	36.27	41.71	2.945	2.941
SPAC323.05C	SPAC323.05c	0.201	6.558	0.696803943	22.64	22.57	4.674	3.12
SPAC186.05C	SPAC186.05c	0.0617	6.563	1.209714836	38.04	44.16	3.237	1.523
SPAC750.08C	SPAC750.08c	0.1393	6.631	0.856048884	33.1	37.14	3.115	2.868
SPBP4H10.12	SPBP4H10.12	0.1487	6.635	0.827689031	41.41	48.79	4.492	2.309
SPBC3H7.05C	SPBC3H7.05c	0.2229	6.655	0.651889932	37.16	42.8	5.593	2.71
SPCC24B10.17	emp24	0.1037	6.662	0.984221244	34.22	38.67	1.999	2.586
SPBC11B10.10C	pht1	0.1964	6.684	0.706858517	25.49	26.38	5.011	2.855
SPCC126.07C	SPCC126.07c	0.1907	6.691	0.719649307	41.7	49.13	4.013	3.493
SPAC2F3.18C	SPAC2F3.18c	0.2644	6.713	0.577738549	42.88	50.75	2.519	4.813
SPBC19G7.06	mbx1	0.2829	6.714	0.548367053	33.89	38.13	3.996	4.485
SPCC613.08	SPCC613.08	0.3052	6.731	0.515415471	34.83	39.42	4.274	4.74
SPCC663.09C	SPCC663.09c	0.1952	6.748	0.709520187	38.11	44	1.721	3.619
SPBC16D10.02	trm11	0.04441	6.766	1.352519227	39.04	45.28	0.8096	1.683
SPAC16E8.06C	nop12	0.08811	6.779	1.054974799	41.45	48.65	3.9	1.376
SPAC22F8.07C	rtf1	0.02531	6.787	1.596707855	21.58	20.75	1.682	1.824
SPAC6G10.02C	tea3	0.103	6.802	0.987162775	35.64	40.46	2.302	2.941
SPAC22F8.04	SPAC22F8.04	0.01419	6.824	1.848017605	24.77	25.17	2.208	1.018
SPCC364.02C	bis1	0.2823	6.824	0.549289122	40.92	47.84	6.115	3.777
SPBC83.17	SPBC83.17	0.2486	6.827	0.604498876	39.73	46.16	2.571	4.299
SPCC24B10.09	rps1702	0.3248	6.866	0.488383979	27.82	29.4	6.507	4.413
SPBC28E12.06C	lvs1	0.09381	6.872	1.027750864	39.94	46.4	1.666	2.524
SPBC582.10C	SPBC582.10c	0.1483	6.896	0.828858849	40.44	47.06	2.787	3.475
SPBC1105.08	SPBC1105.08	0.09619	6.936	1.016870075	36.86	41.99	3.654	2.356
SPAC7D4.12C	SPAC7D4.12c	0.2266	6.937	0.644740094	42.94	50.51	4.952	3.747
SPAC1952.08C	SPAC1952.08c	0.1058	6.959	0.975514332	40.64	47.26	2.098	3.072
SPAC56F8.02	SPAC56F8.02	0.0783	6.966	1.106238238	36.1	40.88	3.62	1.956
SPCC1739.14	npp106	0.5078	7.004	0.294307303	23.92	23.73	7.989	8.072
SPAC23D3.03C	SPAC23D3.03c	0.08923	7.013	1.049489107	40.78	47.38	3.716	2.195
SPBPJ4664.03	mfm3	0.09508	7.061	1.021910827	40.73	47.24	3.567	2.502
SPAC27D7.03C	mei2	0.2925	7.062	0.53387413	43.99	51.82	5.396	4.73
SPBC23E6.01C	SPBC23E6.01c	0.4555	7.074	0.341511619	31.57	34.37	5.896	7.258
SPCC1322.14C	vtc4	0.01318	7.101	1.88008459	37.43	42.56	1.365	1.339
SPBC1711.08	SPBC1711.08	0.1122	7.105	0.950007143	40.75	47.2	2.668	3.151
SPBC4F6.06	kin1	0.02278	7.115	1.64244628	19.93	17.97	1.834	1.842
SPAC3C7.05C	mug191	0.1086	7.117	0.964170175	36.87	41.75	4.076	2.375
SPAC16A10.01	SPAC16A10.01	0.1424	7.125	0.846490011	39.24	45.06	1.841	3.2
SPAC22G7.01C	SPAC22G7.01c	0.1448	7.162	0.839231438	39.63	45.56	4.525	2.783
SPBC29A10.06C	ely5	0.1342	7.172	0.872247484	38.72	44.27	2.632	3.142
SPAC1399.03	fur4	0.1696	7.191	0.770574152	33.44	36.83	2.051	3.563
SPAC637.06	SPAC637.06	0.5851	7.231	0.232769902	26.57	27.13	10.56	9.964
SPAC824.02	SPAC824.02	0.4782	7.236	0.320390428	28.54	29.89	10.16	6.234
SPBC776.02C	dis2	0.2498	7.266	0.602407566	37.02	41.75	4.105	4.44
SPAC2E1P5.02C	mug109	0.1334	7.281	0.87484417	39.4	45.07	4.809	2.233
SPAC5D6.09C	mug86	0.08794	7.286	1.055813539	38.35	43.58	1.384	2.545
SPAC11E3.06	map1	0.3175	7.293	0.49825627	45.49	53.6	5.797	5.222
SPAC1F5.10	SPAC1F5.10	0.05698	7.296	1.244277555	12.63	7.478	3.53	0.8005
SPAC1527.03	SPAC1527.03	0.1061	7.355	0.974284616	41.25	47.56	2.516	3.218
SPAC2C4.07C	SPAC2C4.07c	0.1492	7.358	0.826231177	40.39	46.35	1.875	3.385
SPBC56F2.14	mrpl44	0.1784	7.368	0.74860515	40.21	46.09	2.561	3.767
SPCC550.09	SPCC550.09	0.07318	7.372	1.135607595	37.78	42.67	3.239	2.489
SPCC14G10.04	SPCC14G10.04	0.1058	7.396	0.975514332	25.57	25.5	4.608	1.272
SPCC663.14C	SPCC663.14c	0.1131	7.402	0.946537395	37.4	42.1	3.378	3.129
SPBC1685.15C	klp6	0.03723	7.413	1.429106964	37.44	42.13	1.628	1.914
SPBC15C4.06C	SPBC15C4.06c	0.1475	7.459	0.83120798	36.23	40.36	3.875	3.233
SPBC1711.12	SPBC1711.12	0.1354	7.473	0.868381336	40.58	46.45	3.158	3.249
SPCC285.17	spp27	0.1213	7.476	0.916139199	36.83	41.18	2.033	3.108
SPAC589.05C	SPAC589.05c	0.1313	7.479	0.881735274	44.02	51.28	3.799	3.286
SPBC530.03C	bag102	0.08181	7.482	1.087193607	42.9	49.7	3.844	2.272
SPCC584.13	SPCC584.13	0.1355	7.532	0.868060705	36.82	41.1	2.072	3.308

SPBC1703.12	ubp9	0.1579	7.554	0.80161787	37.67	42.26	2.906	3.962
SPBC16H5.07C	ppa2	0.08788	7.591	1.056109952	34.94	38.38	1.388	2.64
SPAC869.04	SPAC869.04	0.4142	7.595	0.382789905	42.34	48.75	2.505	7.948
SPAC2F3.15	lsk1	0.1772	7.602	0.751536282	33.64	36.53	5.835	2.427
SPAC869.07C	mel1	0.1659	7.611	0.780153614	39.71	45.04	2.031	3.718
SPAC3H1.12C	snt2	0.04455	7.628	1.351152292	34.31	37.44	3.285	1.821
SPAC4G8.10	gos1	0.2929	7.647	0.533280628	35.5	39.08	5.321	5.359
SPCC4B3.08	lsg1	0.1011	7.695	0.995248844	43.43	50.14	4.525	2.135
SPAC27E2.03C	SPAC27E2.03c	0.006294	7.733	2.201073261	37.58	41.88	1.568	1.197
SPAC1687.12C	coq4	0.1972	7.735	0.705093089	12.14	6.184	4.011	4.377
SPAC25G10.02	cce1	0.1459	7.792	0.835944708	37.75	42.03	4.975	3.004
SPAC17A5.02C	dbr1	0.2983	7.824	0.525346747	21.46	19.13	5.789	5.438
SPAC20G8.02	SPAC20G8.02	0.07704	7.841	1.113283726	37.58	41.73	2.436	3.028
SPBP8B7.26	SPBP8B7.26	0.2241	7.849	0.649558143	40.3	45.53	2.651	4.612
SPCC4G3.08	psk1	0.4351	7.88	0.361410917	49.07	57.8	9.432	6.603
SPAC20G4.07C	sts1	0.1755	7.884	0.755722879	25.92	25.3	6.159	2.102
SPAC6F6.03C	SPAC6F6.03c	0.07296	7.898	1.136915175	41.49	47.13	2.729	2.624
SPCC1795.06	map2	0.5165	7.904	0.286929674	34.61	37.47	13.13	6.48
SPAC767.01C	vps1	0.04294	7.941	1.36713796	38.06	42.27	1.426	2.088
SPAC1A6.07	SPAC1A6.07	0.09009	7.945	1.045323413	40.09	45.11	3.243	3.11
SPBP23A10.14C	ell1	0.03864	7.99	1.412962882	37.57	41.5	1.506	2.441
SPAC13D6.02C	byr3	0.02637	7.998	1.57888987	30.31	31.3	2.162	2.151
SPCC132.01C	SPCC132.01c	0.03619	8.021	1.441411417	37.48	41.34	2.662	2.271
SPAC22F3.04	mug62	0.163	8.06	0.787812396	39.13	43.59	2.515	3.915
SPAC10F6.12C	mam4	0.1249	8.077	0.903437562	40.7	45.77	1.686	3.863
SPAC589.09	SPAC589.09	0.06528	8.082	1.185219854	41.14	46.39	3.646	2.474
SPAC644.13C	SPAC644.13c	0.1217	8.127	0.914709422	42.51	48.24	4.522	3.156
SPBPB2B2.19C	SPBPB2B2.19c	0.6139	8.133	0.211902367	24.45	22.89	11.67	12.25
SPBP8B7.28C	stc1	0.2134	8.183	0.670805585	38.17	42.07	3.416	4.638
SPBC16E9.14C	zrg17	0.2514	8.261	0.599634727	42.93	48.65	4.159	5.555
SPBC1773.04	SPBC1773.04	0.08229	8.262	1.084652938	43.18	48.99	3.512	3.038
SPAC1F8.05	isp3	0.1392	8.274	0.856360765	37.26	40.66	3.965	3.557
SPBC25B2.11	pof2	0.08478	8.314	1.071706588	41.5	46.57	3.738	3.01
SPBC119.05C	SPBC119.05c	0.1585	8.334	0.799970733	40.42	45.02	1.935	3.946
SPAC17G8.10C	dma1	0.1902	8.397	0.720789487	45.02	51.38	3.66	4.81
SPCC1020.12C	xap5	0.1533	8.4	0.814457845	37.12	40.29	4.542	3.955
SPAC11G7.04	ubi1	0.2242	8.442	0.649364392	26.6	25.47	6.573	4.087
SPBC119.12	SPBC119.12	0.1401	8.451	0.853561865	38.82	42.61	5.624	2.785
SPCC1281.04	SPCC1281.04	0.09242	8.556	1.034234036	33.63	35.18	4.449	2.858
SPCC1223.03C	gut2	0.1344	8.564	0.871600731	43.11	48.47	4.572	3.71
SPCC285.04	SPCC285.04	0.09811	8.579	1.008286724	40.15	44.3	4.558	2.931
SPBP18G5.03	toc1	0.09214	8.635	1.035551792	40.22	44.31	2.064	3.143
SPBC18H10.05	SPBC18H10.05	0.356	8.691	0.448550002	46.68	53.3	3.702	7.808
SPAC56E4.07	SPAC56E4.07	0.2488	8.692	0.604149624	39.84	43.7	2.428	5.476
SPAC2G11.12	rqh1	0.02596	8.707	1.585695312	29.16	28.69	3.239	1.748
SPAC11H11.03C	SPAC11H11.03c	0.2168	8.858	0.663940722	34.64	36.18	6.344	4.311
SPAC1687.15	gsk3	0.108	8.866	0.966576245	38.57	41.68	5.345	2.537
SPBC106.03	SPBC106.03	0.1141	8.966	0.942714356	40.04	43.6	5.357	2.899
SPBC3B8.05	SPBC3B8.05	0.004754	9.029	2.322940823	43.06	47.75	2.2	1.293
SPAC323.04	SPAC323.04	0.08297	9.044	1.08107891	19.09	14.09	4.836	3.403
SPAC1B3.08	SPAC1B3.08	0.08816	9.093	1.054728418	42.4	46.73	5.099	2.351
SPCC1494.03	arz1	0.1249	9.176	0.903437562	44.24	49.19	2.967	4.347
SPCC191.06	SPCC191.06	0.06119	9.178	1.213319547	43.52	48.19	3.795	2.928
SPAC13G6.13	SPAC13G6.13	0.1237	9.187	0.9076303	40.51	43.96	6.1	2.012
SPBC119.14	rti1	0.05697	9.268	1.244353781	44.25	49.09	4.104	2.627
SPBC1539.07C	SPBC1539.07c	0.1577	9.268	0.802168307	41.13	44.7	5.085	4.415
SPBC1718.02	hop1	0.003219	9.294	2.492279023	43.3	47.71	2.117	1.072
SPAC11D3.15	SPAC11D3.15	0.4998	9.335	0.301203748	51.36	58.97	6.647	11.66
SPCC16A11.01	SPCC16A11.01	0.03946	9.448	1.403842919	38.86	41.28	3.302	2.395
SPAC6B12.14C	SPAC6B12.14c	0.01308	9.5	1.883392256	43.44	47.61	2.415	2.071
SPCC162.03	SPCC162.03	0.02713	9.563	1.566550206	42.39	46.06	3.069	2.443
SPAP8A3.14C	SPAP8A3.14c	0.06776	9.568	1.169026603	39.33	41.76	4.844	2.354
SPCC965.06	SPCC965.06	0.1014	9.599	0.993962045	40.86	43.86	4.418	3.799
SPAC1002.01	SPAC1002.01	0.03221	9.658	1.492009275	42.65	46.3	2.787	2.74
SPCC417.07C	mto1	0.1065	9.712	0.972650392	38.21	39.99	3.793	4.17
SPAC16E8.18	SPAC16E8.18	0.02175	9.776	1.662540739	41.12	43.97	2.914	2.379
SPAC328.05	SPAC328.05	0.1574	9.848	0.802995272	46.51	51.45	2.415	5.268
SPCP31B10.02	SPCP31B10.02	0.1345	9.958	0.871277716	46.02	50.6	3.378	4.87
SPAC1B3.16C	vht1	0.03867	10.46	1.412625828	32.04	30.28	4.474	2.021

SPAC3A11.05C	kms1	0.09412	10.49	1.026318081	42.41	44.78	5.631	3.389
SPBC1652.02	SPBC1652.02	0.01001	10.76	1.999565923	42.99	45.23	3.153	1.212
SPAC29A4.05	cam2	0.06779	10.83	1.168834366	40.86	42.13	5.655	1.857
SPAC12B10.14C	ppk2	0.03083	11.01	1.511026475	43.3	45.31	3.874	2.782
SPAC29B12.02C	set2	0.2002	11.24	0.698535927	48.12	51.74	4.126	6.76
SPAPB2B4.06	SPAPB2B4.06	0.02578	11.35	1.588717087	44.78	46.91	3.981	2.594
SPBC12D12.02C	cdm1	0.1891	12.39	0.723308471	47.88	49.8	4.128	7.248
SPAC9E9.11	plr1	0.2655	13.28	0.575935475	50.56	52.31	3.322	9.67
SPAC9.02C	SPAC9.02c	0.009571	20.47	2.019042684	35.28	20.78	5.163	4.018
SPAC27F1.08	pdt1	0.002758	26.55	2.559405738	39.12	17.64	5.731	2.394

Supplementary Table 3

R package version: 0.0-10
Summary type: mean
Test type: t-test   Based on 4 independent repeat experimens

Control medium: YE5S
Control screen ID: QFA0002
Control libraries: PDLV4_384
Query medium: EMM_Ammonia
Query screen ID: QFA0002
##

ORF	Gene	P	EGI	(p) -log10	Ammonia Fitness mean	Yes_Fitness mean	Ammonia SE	YES SE
SPBC30D10.16	pha2	0.002063	-22.77	2.686	9.76	61.22	5.233	0.9494
SPBC725.01	SPBC725.01	0.0375	-21.8	1.426	8.496	57.02	11.97	1.657
SPAC17H9.08	SPAC17H9.08	0.02003	-21.4	1.698	4.804	49.31	10.13	2.743
SPAC1782.12C	SPAC1782.12c	0.01038	-20.45	1.984	15.59	67.83	7.167	0.9078
SPBC2G5.06C	hmt2	0.004161	-19.73	2.381	14.27	64	4.869	0.38
SPBC25H2.08C	mrs2	0.01852	-19.01	1.732	21.83	76.87	8.255	1.278
SPBC27.08C	sua1	0.00458	-18.68	2.339	9.069	52.23	2.404	3.025
SPAC343.16	lys2	0.004813	-17.43	2.318	11.53	54.5	4.361	3.079
SPCC1739.06C	SPCC1739.06c	0.05097	-17.18	1.293	12.84	56.5	10.36	0.7812
SPAC23D3.04C	gpd2	0.002958	-16.88	2.529	17.83	65.35	4.694	1.155
SPCPB1C11.03	SPCPB1C11.03	2.56E-05	-16.79	4.592	16.48	62.62	1.552	1.067
SPAC31A2.09C	apm4	0.01326	-16.25	1.877	10.64	50.61	3.403	3.63
SPAC3F10.09	SPAC3F10.09	0.02098	-15.88	1.678	11.8	52.1	5.033	4.052
SPAC56E4.03	SPAC56E4.03	0.01587	-15.86	1.799	10.52	49.67	6.007	3.52
SPAC4G9.09C	arg11	0.02229	-15.21	1.652	12.49	52.14	6.823	0.7415
SPBC21H7.07C	his5	0.00723	-15.07	2.141	11.23	49.5	3.883	2.937
SPAC343.10	met11	0.1712	-14.85	0.766	15.32	56.78	15.62	0.8508
SPBC215.08C	arg4	0.07201	-14.68	1.143	9.76	46.01	10.32	3.429
SPAC4A8.03C	ptc4	0.08669	-14.21	1.062	1.419	29.42	10.79	1.205
SPBC25H2.03	SPBC25H2.03	0.2939	-14.05	0.532	20.2	64.46	18.6	6.954
SPBC428.05C	arg12	0.02867	-13.93	1.543	11.25	47.39	5.236	3.824
SPCC663.02	wtf14	0.2401	-13.78	0.620	18.76	61.24	15.59	6.451
SPBC17D1.05	SPBC17D1.05	0.04821	-13.74	1.317	24.48	71.94	8.501	1.885
SPAC637.10C	rpn10	2.68E-05	-13.64	4.572	17.93	59.43	1.654	0.7646
SPAC24C9.07C	bgs2	0.0984	-13.55	1.007	28.96	80.01	10.86	1.104
SPBC1105.02C	lys4	0.02845	-13.48	1.546	14.29	52.27	6.526	0.5712
SPAC343.15	tit1	0.03902	-13.43	1.409	13.19	50.1	3.014	4.089
SPCC1235.04C	SPCC1235.04c	0.03493	-13.4	1.457	25.64	73.5	7.025	0.6572
SPBC725.14	arg6	0.01851	-13.28	1.733	14.6	52.48	5.701	0.7929
SPBC16G5.09	SPBC16G5.09	0.258	-13.25	0.588	18.44	59.65	15.88	6.277
SPBC1778.05C	SPBC1778.05c	0.3389	-13.12	0.470	9.331	42.26	21.45	3.292
SPBC23G7.08C	rga7	0.002122	-13.09	2.673	12.99	49.09	3.654	1.448
SPAC25G10.05C	his1	0.0249	-13.05	1.604	15.32	53.4	6.397	1.229
SPBC6B1.05C	atg7	0.236	-13.01	0.627	17.61	57.63	16.56	1.122
SPAC1296.02	cox4	0.332	-13.01	0.479	17.18	56.81	21.06	2.428
SPAC17H9.13C	SPAC17H9.13c	0.02874	-12.96	1.542	11.59	46.2	3.386	3.625
SPBC15D4.09C	SPBC15D4.09c	0.03969	-12.81	1.401	16.82	55.79	6.983	0.4666
SPAC4D7.02C	SPAC4D7.02c	0.254	-12.48	0.595	18.36	58.04	14.32	6.26
SPAC5H10.13C	gmh2	0.3072	-12.08	0.513	21.99	64.13	15.94	6.659
SPBC11B10.02C	his3	0.02431	-12.06	1.614	12.21	45.69	4.19	3.185
SPCPB16A4.03C	ade10	0.000158	-12.05	3.803	11.65	44.61	1.569	1.072
SPBC14F5.13C	SPBC14F5.13c	0.03617	-12	1.442	23.11	66.08	6.51	0.9123
SPBC776.15C	kgd2	0.1642	-11.88	0.785	17.07	54.5	11.9	3.427
SPAPYUG7.04C	rpb9	0.07744	-11.87	1.111	24.08	67.65	8.619	1.124
SPCC576.17C	SPCC576.17c	0.1881	-11.85	0.726	26.47	72.13	13.15	1.117
SPBC21C3.03	SPBC21C3.03	0.04268	-11.82	1.370	25.17	69.6	6.75	0.7971
SPBC19C2.02	pmt1	0.06727	-11.82	1.172	24.51	68.38	8.118	1.122
SPAC3C7.01C	SPAC3C7.01c	0.04801	-11.75	1.319	27.92	74.67	6.943	0.598
SPBC1711.13	his2	0.1394	-11.67	0.856	12.01	44.57	10.72	3.36

SPCC188.13C	dcr1	0.00472	-11.65	2.326	13.84	47.96	3.696	0.9515
SPBC21C3.01C	vps13a	0.3564	-11.63	0.448	16.72	53.36	18.04	6.35
SPBC13G1.12	did2	0.2832	-11.51	0.548	16.34	52.43	14.63	5.784
SPBC1271.14	SPBC1271.14	0.23	-11.45	0.638	9.232	38.92	14.05	3.131
SPAC25H1.02	jmj1	0.05517	-11.4	1.258	28.3	74.74	7.332	1.309
SPBC28F2.08C	SPBC28F2.08c	0.03557	-11.17	1.449	23.82	65.86	6.261	1.511
SPCC663.08C	SPCC663.08c	0.0916	-11.16	1.038	27.78	73.3	8.749	1.405
SPCC16A11.08	atg20	0.04291	-11.1	1.367	25.74	69.33	6.583	1.407
SPBC359.06	mug14	0.02302	-11.01	1.638	24.91	67.61	5.382	1.292
SPAC15A10.13	ppk3	0.1907	-10.87	0.720	31.59	79.9	12.15	1.314
SPAC22F3.13	tsc1	0.01622	-10.84	1.790	18.27	54.78	4.793	1.914
SPCC4G3.05C	mus81	0.08618	-10.81	1.065	29.21	75.33	8.218	1.089
SPCC1620.03	mug163	0.07624	-10.75	1.118	25.84	68.86	7.691	0.7187
SPCC24B10.12	cgi121	0.2899	-10.67	0.538	17.52	53.06	12.91	6.111
SPCC63.04	mok14	0.2255	-10.62	0.647	32.76	81.65	13.1	1.733
SPBC1539.03C	SPBC1539.03c	0.0157	-10.4	1.804	14.27	46.44	4.204	0.5572
SPBC28F2.10C	ngg1	0.01337	-10.35	1.874	16.47	50.47	4.385	1.69
SPBPB2B2.02	mug180	0.03093	-10.25	1.510	28	71.98	5.427	1.116
SPAC57A10.10C	sla1	0.3294	-10.21	0.482	21.26	59.24	16.54	0.4895
SPAP7G5.04C	lys1	0.2298	-10.19	0.639	13.05	43.76	11.24	4.663
SPAC4H3.06	SPAC4H3.06	0.1241	-10.08	0.906	28.95	73.45	9.043	1.463
SPBC1685.13	fhn1	0.02898	-10.01	1.538	21.05	58.46	5.271	1.801
SPBC30D10.10C	tor1	0.08243	-9.983	1.084	25.72	67.19	7.459	1.049
SPAC11G7.06C	mug132	0.1226	-9.981	0.912	28.56	72.55	8.861	0.879
SPBC543.02C	SPBC543.02c	0.1312	-9.976	0.882	27.78	71.06	9.16	0.9516
SPAC57A10.06	mug15	0.1185	-9.905	0.926	30.1	75.3	8.685	1.179
SPAC17A5.01	pex6	0.02496	-9.801	1.603	23.82	63.29	4.962	1.421
SPAC890.05	SPAC890.05	0.04863	-9.765	1.313	15.41	47.39	6.073	1.772
SPBC11B10.05C	rsp1	0.07058	-9.756	1.151	27.86	70.8	6.823	0.9055
SPAC22E12.01	SPAC22E12.01	0.1739	-9.671	0.760	31.11	76.75	10.27	1.31
SPAC144.17C	SPAC144.17c	0.01565	-9.646	1.805	25.5	66.15	4.296	1.45
SPCC757.03C	SPCC757.03c	0.07849	-9.621	1.105	28.34	71.45	7.077	1.173
SPBC1685.08	cti6	0.05291	-9.583	1.276	27.74	70.26	6.067	1.09
SPBC685.02	exo5	0.0819	-9.561	1.087	27.68	70.09	7.133	1.049
SPAC3H8.09C	nab3	0.124	-9.545	0.907	16.48	48.99	8.557	1.267
SPAC11G7.01	SPAC11G7.01	0.295	-9.532	0.530	22.79	60.84	13.19	4.098
SPAC683.02C	SPAC683.02c	0.02761	-9.518	1.559	16.63	49.21	4.905	1.8
SPCC777.10C	ubc12	0.01568	-9.492	1.805	18.28	52.27	3.696	2.056
SPBC577.11	SPBC577.11	0.119	-9.37	0.924	27.36	69.14	8.242	1.251
SPAC1687.09	SPAC1687.09	0.2324	-9.305	0.634	31.38	76.58	11.71	1.37
SPCC1235.15	dga1	0.09837	-9.255	1.007	29.17	72.33	7.461	1.046
SPAC1A6.03C	SPAC1A6.03c	0.1145	-9.233	0.941	31.67	76.98	7.969	1.123
SPCC584.01C	SPCC584.01c	0.004758	-9.216	2.323	16.51	48.42	2.388	0.1962
SPBC2G2.15C	mrm2	0.08676	-9.177	1.062	28.87	71.62	6.964	0.7487
SPAC1002.19	urg1	0.2252	-9.12	0.647	32.63	78.58	11.24	1.511
SPBC365.16	SPBC365.16	0.03097	-9.058	1.509	14.38	44.11	4.882	1.552
SPCC70.06	SPCC70.06	0.02252	-9.056	1.647	24.75	63.63	4.431	1.171
SPBC1289.06C	ppr8	0.03017	-8.988	1.520	19.4	53.44	4.57	0.6405
SPBC428.02C	eca39	0.08283	-8.95	1.082	10.47	36.54	0.4296	3.495
SPBC4B4.10C	atg5	0.1988	-8.923	0.702	24.13	62.21	10.2	1.476
SPBC25B2.04C	mtg1	0.009943	-8.914	2.002	15.56	46.07	3.011	0.3063
SPAC1834.04	hht1	0.04905	-8.913	1.309	29.29	71.89	5.521	1.109
SPCC70.09C	mug9	0.1404	-8.862	0.853	30.19	73.51	8.432	1.443
SPAC23H3.03C	npr2	0.1027	-8.834	0.988	16.63	47.93	7.166	0.2739
SPAC664.03	SPAC664.03	0.02072	-8.816	1.684	10.93	37.17	2.269	2.247
SPAC323.01C	pos5	0.000831	-8.796	3.080	2.958	22.12	2.021	0.7644
SPCC24B10.20	SPCC24B10.20	0.07606	-8.78	1.119	27.52	68.32	6.322	0.7905
SPAC24C9.15C	spn5	0.09828	-8.776	1.008	28.79	70.71	7.101	1.25
SPAC25B8.13C	isp7	0.1078	-8.759	0.967	20.14	54.39	5.871	3.422
SPAC4G8.11C	atp10	0.14	-8.734	0.854	10.25	35.74	6.951	3.57
SPAC57A7.08	pzh1	0.1296	-8.724	0.887	33.98	80.38	7.987	1.218
SPBC336.05C	SPBC336.05c	0.2445	-8.724	0.612	17.39	49.16	4.22	5.945
SPAC3H8.02	SPAC3H8.02	0.09059	-8.714	1.043	12.01	39	6.717	2.073

SPAC227.14	SPAC227.14	0.1739	-8.696	0.760	28.61	70.22	9.239	1.005
SPCC18.15	SPCC18.15	0.06429	-8.693	1.192	27.43	68	5.797	0.5657
SPBP35G2.07	ilv1	0.04837	-8.648	1.315	8.953	33.13	3.065	2.848
SPBC31A8.01C	rtn1	0.3335	-8.648	0.477	21.57	56.88	6.202	7.202
SPCC1322.01	rpm1	0.05662	-8.627	1.247	14.62	43.76	5.653	1.286
SPCC1840.09	SPCC1840.09	0.3324	-8.615	0.478	18.4	50.85	13.87	2.088
SPBC14C8.11C	SPBC14C8.11c	0.4024	-8.612	0.395	25.54	64.28	16.63	0.9372
SPAC23E2.01	fep1	0.05997	-8.6	1.222	18.34	50.71	5.708	1.031
SPAC22F3.11C	snu23	0.427	-8.587	0.370	18.98	51.88	14.51	6.459
SPAC1250.03	ubc14	0.000975	-8.528	3.011	25.21	63.5	1.596	1.077
SPAC1687.08	SPAC1687.08	0.1989	-8.525	0.701	8.302	31.67	9.662	2.033
SPAC1705.02	SPAC1705.02	0.2698	-8.52	0.569	22.07	57.57	11.84	1.117
SPAC17G8.06C	SPAC17G8.06c	0.05939	-8.491	1.226	12.44	39.4	2.523	3.01
SPAC1B3.06C	SPAC1B3.06c	0.1624	-8.483	0.789	31.94	76.09	8.683	0.9054
SPCC569.07	SPCC569.07	0.1093	-8.453	0.961	30.12	72.59	7.153	1.103
SPBC1683.02	SPBC1683.02	0.1718	-8.432	0.765	27.73	68.06	8.869	1.648
SPBC337.16	cho1	0.1405	-8.404	0.852	3.512	22.43	7.993	1.515
SPAC29A4.13	SPAC29A4.13	0.1421	-8.375	0.847	30.1	72.41	8.014	1.438
SPAC1F3.09	mug161	0.0536	-8.338	1.271	28.65	69.62	5.368	1.348
SPAC22F3.07C	atp20	0.2727	-8.333	0.564	16.16	46.1	6.926	5.701
SPBC3B9.08C	mnh1	0.1313	-8.329	0.882	31.82	75.56	7.665	1.036
SPBC21B10.07	SPBC21B10.07	0.2854	-8.323	0.545	13.55	41.16	10.51	4.306
SPAC343.07	mug28	0.3049	-8.313	0.516	21.55	56.2	9.99	5.171
SPCC1393.02C	spt2	0.00392	-8.298	2.407	24.72	62.15	2.174	1.377
SPBC1683.03C	SPBC1683.03c	0.3898	-8.254	0.409	22.38	57.65	15.42	1.251
SPBC21B10.08C	SPBC21B10.08c	0.1704	-8.246	0.769	30.76	73.42	8.665	1.084
SPBC21D10.11C	nfs1	0.1369	-8.191	0.864	31.08	73.91	7.659	1.763
SPAC2G11.03C	vps45	0.001809	-8.158	2.743	19.18	51.45	2.05	0.5437
SPAC890.07C	rmt1	0.1812	-8.088	0.742	32.21	75.85	8.789	1.05
SPBC543.03C	pku80	0.2339	-8.083	0.631	35.11	81.29	10.21	1.176
SPCC794.01C	SPCC794.01c	0.1027	-8.078	0.988	29.91	71.5	6.628	0.8505
SPAC922.05C	SPAC922.05c	0.4357	-8.018	0.361	22.18	56.84	16.39	2.858
SPBP8B7.18C	SPBP8B7.18c	0.07985	-8.013	1.098	18.3	49.53	5.897	1.806
SPCC1322.16	phb2	0.1221	-8.011	0.913	17.26	47.57	7.122	0.9567
SPBC713.03	SPBC713.03	0.2857	-7.999	0.544	34.28	79.57	11.55	1.366
SPAC23G3.03	sib2	0.09368	-7.998	1.028	28.51	68.71	6.31	0.8723
SPCC4B3.12	set9	0.3445	-7.913	0.463	30.42	72.15	13.24	1.185
SPAC1B3.01C	SPAC1B3.01c	0.001926	-7.883	2.715	14.49	42.1	2	0.5211
SPBC19C7.01	mni1	0.000817	-7.882	3.088	22.12	56.47	1.772	0.839
SPBC428.17C	wpl1	0.2591	-7.862	0.587	22.82	57.74	8.34	4.49
SPBC23G7.06C	SPBC23G7.06c	0.01835	-7.859	1.736	27.22	66.03	3.624	0.9621
SPBC106.05C	tim11	0.1305	-7.84	0.884	28.3	68.02	7.179	0.7624
SPBP8B7.07C	set6	0.075	-7.819	1.125	29.51	70.26	5.625	0.8298
SPAC6F6.04C	SPAC6F6.04c	0.3912	-7.805	0.408	21.9	55.92	6.782	7.371
SPCC550.01C	SPCC550.01c	0.05357	-7.802	1.271	26.83	65.19	4.861	0.5619
SPBC1734.12C	alg12	0.2465	-7.792	0.608	25.3	62.29	10.04	2.034
SPBC660.07	ntp1	0.347	-7.774	0.460	20.14	52.54	12.91	2.159
SPBC18H10.16	can1	0.02538	-7.762	1.596	26.67	64.81	3.952	1.22
SPBC14C8.04	SPBC14C8.04	0.1183	-7.756	0.927	31.09	73.12	6.74	1.807
SPAC25B8.11	SPAC25B8.11	0.2794	-7.712	0.554	34.84	80.09	10.93	1.523
SPBC13E7.07	SPBC13E7.07	0.03137	-7.639	1.503	27.8	66.69	4.101	0.9437
SPBC15D4.12C	mug98	0.2238	-7.635	0.650	34.56	79.42	9.375	1.206
SPBC19C7.12C	omh1	0.006078	-7.634	2.216	27.4	65.94	2.545	1.245
SPAC10F6.07C	mug94	0.3411	-7.625	0.467	18.4	48.97	6.01	6.396
SPCC1682.11C	SPCC1682.11c	0.1859	-7.615	0.731	18.91	49.92	7.682	2.946
SPAPB8E5.03	mae1	0.2194	-7.589	0.659	32.59	75.63	9.199	1.292
SPAC9G1.04	oxa101	0.1429	-7.566	0.845	30.42	71.5	7.262	1.109
SPBC1198.11C	reb1	0.3122	-7.562	0.506	18.4	48.86	11.58	1.855
SPAC30D11.11	SPAC30D11.11	0.2765	-7.534	0.558	36.43	82.74	10.66	0.9275
SPBC29A3.09C	SPBC29A3.09c	0.06389	-7.513	1.195	30.11	70.82	5.059	0.7173
SPBC13E7.11	SPBC13E7.11	0.03597	-7.512	1.444	26.53	64.07	4.223	1
SPBC16A3.02C	SPBC16A3.02c	0.1757	-7.496	0.755	32.52	75.33	7.957	1.629
SPBC17G9.09	tif213	0.4679	-7.469	0.330	27.17	65.2	16.9	1.116

SPBC1604.01	mug158	0.1846	-7.454	0.734	32.58	75.36	8.137	1.573
SPBC16G5.03	SPBC16G5.03	0.113	-7.453	0.947	28.56	67.77	6.394	0.8818
SPCC24B10.02C	SPCC24B10.02c	0.05016	-7.43	1.300	29.02	68.6	4.662	1.045
SPAC323.03C	SPAC323.03c	0.4973	-7.424	0.303	29.5	69.5	17.85	2.395
SPCC777.07	omh3	0.1241	-7.405	0.906	31.48	73.19	6.644	1.074
SPBP8B7.23	SPBP8B7.23	0.2139	-7.364	0.670	30.67	71.59	8.812	0.8696
SPCC548.06C	ght8	0.3419	-7.351	0.466	9.358	31.45	12.04	2.104
SPBC18H10.11C	ppr2	0.1972	-7.321	0.705	12.53	37.37	7.221	3.257
SPBC947.06C	SPBC947.06c	0.09955	-7.312	1.002	28.39	67.19	5.954	1.145
SPAC15A10.11	ubr11	0.1909	-7.239	0.719	11.86	35.94	4.828	4.054
SPAC1002.12C	SPAC1002.12c	0.4871	-7.219	0.312	26.26	63.02	17.05	1.654
SPBC18E5.01	SPBC18E5.01	0.1545	-7.182	0.811	30.5	70.93	7.163	1.252
SPBP8B7.31	SPBP8B7.31	0.3239	-7.163	0.490	15.41	42.49	7.061	5.422
SPBC1105.01	rrp12	0.2841	-7.155	0.547	31.24	72.26	10.32	0.9228
SPCC132.02	hst2	0.3926	-7.142	0.406	20.62	52.26	4.17	7.019
SPAC9.06C	SPAC9.06c	0.4178	-7.128	0.379	21.73	54.31	5.262	7.349
SPCC1259.09C	pdx1	0.1157	-7.109	0.937	28.14	66.35	6.173	0.9427
SPAC19D5.07	uga1	0.3349	-7.091	0.475	19.43	49.91	5.507	5.864
SPAC343.18	rfp2	0.04944	-7.071	1.306	27.48	65.03	4.387	0.8586
SPBC19G7.07C	ppr3	0.4457	-7.069	0.351	35.85	80.77	15.09	1.424
SPBC577.06C	stt4	0.2628	-7.048	0.580	20.7	52.23	9.519	1.694
SPAC3F10.04	gsa1	0.3781	-7.045	0.422	11.22	34.37	11.74	3.749
SPBC685.04C	aps2	0.4133	-7.035	0.384	27.17	64.38	13.8	1.809
SPAC19B12.10	sst2	0.08086	-7.005	1.092	14	39.53	3.131	2.736
SPBC8E4.02C	SPBC8E4.02c	0.2557	-6.997	0.592	33.08	75.43	9.362	1.121
SPCC1393.08	SPCC1393.08	0.2718	-6.996	0.566	13.43	38.45	7.875	3.99
SPBC405.03C	SPBC405.03c	0.08607	-6.993	1.065	26.31	62.68	5.362	1.064
SPBC1778.10C	ppk21	0.1044	-6.992	0.981	30.21	70.02	5.808	1.034
SPBP16F5.03C	tra1	0.00049	-6.948	3.310	23.43	57.18	1.425	0.6607
SPCC1223.09	SPCC1223.09	0.1325	-6.885	0.878	28.94	67.43	6.36	0.7822
SPBC1703.09	SPBC1703.09	0.3517	-6.879	0.454	34.54	77.95	11.7	1.169
SPBC30D10.13C	pdb1	0.004911	-6.839	2.309	2.136	16.89	2.228	1.016
SPAC13G7.06	met16	0.08413	-6.828	1.075	13.58	38.42	5.191	1.167
SPBC713.05	SPBC713.05	0.3638	-6.807	0.439	18.08	46.85	4.985	6.117
SPAC17C9.15C	SPAC17C9.15c	0.06908	-6.796	1.161	15.13	41.28	4.756	1.424
SPBC119.03	SPBC119.03	0.09431	-6.772	1.025	31.15	71.37	5.392	1.161
SPAC30D11.06C	SPAC30D11.06c	0.1073	-6.743	0.969	29.48	68.18	5.666	0.9616
SPAC19B12.08	atg4	0.3333	-6.733	0.477	23.07	56.1	10.93	1.3
SPAC30D11.07	nth1	0.3366	-6.731	0.473	19.08	48.58	5.811	5.488
SPAC29A4.12C	mug108	0.3672	-6.703	0.435	23.23	56.34	11.5	2.475
SPAC110.01	ppk1	0.0574	-6.656	1.241	28.69	66.53	4.373	0.9221
SPAC1D4.03C	aut12	0.1007	-6.651	0.997	18.9	48.1	4.086	2.63
SPAC3A12.12	atp11	0.3456	-6.635	0.461	18.56	47.42	10.57	2.834
SPBC1A4.04	SPBC1A4.04	0.4775	-6.635	0.321	14.08	39	13.69	4.716
SPCC132.04C	gdh2	0.3456	-6.622	0.461	22.41	54.63	10.8	2.314
SPCC1919.13C	SPCC1919.13c	0.2347	-6.619	0.629	33.42	75.36	8.384	0.9129
SPBC56F2.09C	arg5	0.3197	-6.604	0.495	7.983	27.45	9.088	3.639
SPAC26A3.16	dph1	0.1423	-6.592	0.847	28.03	65.17	6.312	0.8835
SPAC16C9.01C	SPAC16C9.01c	0.4156	-6.577	0.381	19.48	49.05	5.315	6.686
SPBC17D11.03C	SPBC17D11.03c	0.4144	-6.548	0.383	26.24	61.72	12.98	1.079
SPAPYUG7.02C	sin1	0.1364	-6.546	0.865	8.129	27.62	5.509	2.932
SPBC13E7.06	msd1	0.5415	-6.54	0.266	26.6	62.38	17.85	1.18
SPAC1039.03	SPAC1039.03	0.1559	-6.519	0.807	26.23	61.64	6.521	1.259
SPBC9B6.09C	mdl1	0.05808	-6.513	1.236	28.6	66.09	4.307	1.199
SPAC31G5.04	SPAC31G5.04	0.4747	-6.503	0.324	13.65	37.94	13.01	4.891
SPACUNK4.10	SPACUNK4.10	0.5347	-6.5	0.272	27.93	64.79	17.4	1.292
SPAC17A2.07C	SPAC17A2.07c	0.4373	-6.451	0.359	20.34	50.43	5.546	6.893
SPAC13G6.07C	rps601	0.2151	-6.444	0.667	32.66	73.59	7.72	1.056
SPBC12C2.05C	bzz1	0.354	-6.423	0.451	24.31	57.85	10.98	1.19
SPBC3B8.08	SPBC3B8.08	0.1073	-6.393	0.969	30.99	70.37	5.374	1.157
SPAC3F10.05C	mug113	0.5072	-6.391	0.295	25.58	60.18	15.78	2.024
SPCC1281.07C	SPCC1281.07c	0.4671	-6.382	0.331	25.99	60.93	14.46	0.1601
SPBC1703.13C	SPBC1703.13c	0.01592	-6.381	1.798	28.44	65.54	2.844	1.025

SPBC1709.14	SPBC1709.14	0.3894	-6.378	0.410	20.32	50.26	6.005	5.911
SPAC24C9.08	SPAC24C9.08	0.1124	-6.362	0.949	29.27	67.06	5.352	1.623
SPAC2E1P3.04	cao1	0.2968	-6.348	0.528	25.89	60.67	9.464	0.716
SPAC167.01	ire1	0.136	-6.335	0.866	21.25	51.91	4.421	2.81
SPCC61.03	SPCC61.03	0.3769	-6.308	0.424	24.69	58.34	11.06	2.408
SPAC23C4.12	hhp2	0.03986	-6.27	1.399	23.25	55.55	3.66	1.031
SPBC16H5.04	SPBC16H5.04	0.1812	-6.223	0.742	30.41	68.96	6.612	1.735
SPBC609.04	caf5	0.4529	-6.195	0.344	28.62	65.54	12.65	3.413
SPBC337.13C	gtr1	0.02774	-6.194	1.557	17.86	45.28	1.852	1.719
SPBC1347.08C	SPBC1347.08c	0.06309	-6.191	1.200	29.33	66.86	4.205	0.8421
SPBC2G2.05	rpl1603	0.2939	-6.188	0.532	35.53	78.51	9.14	0.911
SPAC17H9.04C	SPAC17H9.04c	0.1391	-6.171	0.857	21.38	51.86	2.72	3.076
SPBC25H2.05	egd2	0.01298	-6.158	1.887	26.91	62.24	2.572	0.6758
SPCC1259.07	rxt3	0.427	-6.135	0.370	9.416	29.27	11.64	3.394
SPBC6B1.06C	ubp14	0.1457	-6.112	0.837	16.85	43.23	5.923	0.9584
SPCC1442.11C	SPCC1442.11c	0.1228	-6.107	0.911	30.53	68.95	5.45	1.125
SPCC1494.10	adn3	0.05383	-6.057	1.269	28.33	64.72	3.874	0.7505
SPAC1565.01	SPAC1565.01	0.1299	-6.042	0.886	22.59	53.88	5.477	1.438
SPAC1952.05	gcn5	0.03487	-6.041	1.458	14.58	38.8	2.039	1.791
SPAC458.04C	dil1	0.04963	-6.041	1.304	30.35	68.49	3.784	0.903
SPBC1861.06C	mug131	0.4845	-6.038	0.315	21.23	51.33	5.513	7.268
SPAC139.01C	SPAC139.01c	0.2275	-6.037	0.643	20.06	49.11	7.377	1.602
SPBC1604.16C	SPBC1604.16c	0.4758	-5.99	0.323	24.27	56.96	13.63	1.91
SPCP20C8.02C	SPCP20C8.02c	0.4683	-5.985	0.329	20.43	49.71	5.243	6.928
SPBPB8B6.05C	SPBPB8B6.05c	0.4531	-5.975	0.344	19.69	48.31	4.413	6.745
SPAC144.02	iec1	0.4541	-5.969	0.343	26.83	61.74	13.02	1.088
SPBC365.13C	hba1	0.4759	-5.969	0.322	26.81	61.69	13.65	1.651
SPBC405.05	SPBC405.05	0.4192	-5.949	0.378	24.84	57.95	11.71	1.999
SPBC1198.01	SPBC1198.01	0.2584	-5.909	0.588	22.76	53.96	7.943	1.117
SPAC20H4.03C	tfs1	0.01247	-5.902	1.904	24.95	58.07	1.947	1.281
SPBC609.05	pob3	0.1467	-5.9	0.834	16.91	42.93	5.21	2.159
SPBC16E9.13	ksp1	0.1565	-5.9	0.805	29.77	67.14	5.832	1.518
SPAPB17E12.03	SPAPB17E12.03	0.2903	-5.9	0.537	22.83	54.07	7.192	3.347
SPAC977.12	SPAC977.12	0.08251	-5.852	1.083	17.55	44.05	4.352	1.355
SPCC16C4.20C	SPCC16C4.20c	0.1677	-5.84	0.775	19.06	46.87	3.668	3.064
SPCC285.10C	SPCC285.10c	0.4328	-5.808	0.364	24.9	57.8	11.73	2.229
SPBC25B2.08	SPBC25B2.08	0.4828	-5.801	0.316	20.51	49.52	5.07	6.981
SPBPB2B2.12C	gal10	0.6388	-5.795	0.195	18.57	45.86	19.88	4.364
SPBC11B10.07C	ivn1	0.05067	-5.772	1.295	17.59	43.97	2.857	1.797
SPBC4F6.08C	mrpl39	0.02802	-5.76	1.553	28.11	63.76	2.991	0.7127
SPAC1610.02C	SPAC1610.02c	0.02335	-5.756	1.632	15.95	40.86	2.822	1.067
SPBC1604.19C	SPBC1604.19c	0.5216	-5.73	0.283	27.73	62.97	14.82	1.172
SPBC1734.08	hse1	0.5816	-5.72	0.235	26.21	60.09	17.04	2.686
SPAC1635.01	SPAC1635.01	0.2074	-5.713	0.683	21.14	50.54	6.712	0.2929
SPBC337.11	SPBC337.11	0.5994	-5.709	0.222	32.69	72.27	16.95	4.589
SPAPJ760.02C	abp1	0.004458	-5.707	2.351	24.57	56.99	1.871	0.724
SPBC106.17C	cys2	0.1137	-5.703	0.944	14.19	37.44	4.913	1.08
SPCC737.06C	SPCC737.06c	0.1931	-5.69	0.714	22.21	52.52	6.32	1.432
SPCC162.02C	SPCC162.02c	0.5609	-5.677	0.251	28.4	64.14	16.22	1.655
SPBC3B8.02	php5	0.4418	-5.671	0.355	24.57	56.91	12.05	0.6763
SPCC1235.12C	mug146	0.5944	-5.655	0.226	30.84	68.69	17.83	1.246
SPAC1B1.04C	SPAC1B1.04c	0.1227	-5.654	0.911	21.94	51.94	3.478	2.496
SPCC645.11C	mug117	0.5244	-5.644	0.280	26.09	59.73	14.66	1.404
SPAPB1A11.01	mfc1	0.1723	-5.638	0.764	20.47	49.13	3.969	2.908
SPCC1919.10C	myo52	0.06761	-5.637	1.170	13.94	36.85	3.245	1.851
SPCP1E11.02	ppk38	0.1969	-5.637	0.706	31.75	70.37	6.409	0.9631
SPAC22H10.09	SPAC22H10.09	0.01346	-5.633	1.871	15.45	39.67	2.379	0.9482
SPAPB1A10.08	SPAPB1A10.08	0.395	-5.625	0.403	21.59	51.22	5.407	5.272
SPAC23H4.10C	thi4	0.1483	-5.623	0.829	17.54	43.6	2.1	2.913
SPAC24H6.10C	SPAC24H6.10c	0.4845	-5.617	0.315	19.75	47.74	4.815	6.802
SPAC22A12.10	SPAC22A12.10	0.3096	-5.56	0.509	19.55	47.27	7.354	3.099
SPAC144.04C	spe1	0.2261	-5.526	0.646	23.75	55.1	6.697	1.556
SPBC409.20C	psh3	0.4089	-5.518	0.388	12.4	33.73	8.645	4.183

SPBC530.04	mod5	0.5425	-5.506	0.266	22.27	52.28	13.75	4.145
SPAC12G12.09	SPAC12G12.09	0.4795	-5.487	0.319	25.1	57.56	12.73	1.163
SPCC737.05	SPCC737.05	0.5505	-5.487	0.259	26.2	59.64	15.24	1.604
SPCC794.03	SPCC794.03	0.01365	-5.484	1.865	25.73	58.75	2.133	1.115
SPCC1620.02	wtf23	0.02202	-5.483	1.657	29	64.91	2.623	1.046
SPCC4B3.06C	SPCC4B3.06c	0.02859	-5.467	1.544	28.72	64.35	2.882	0.8846
SPCC4B3.11C	SPCC4B3.11c	0.005956	-5.459	2.225	28.67	64.24	1.91	0.6331
SPCC1259.03	rpa12	0.2988	-5.449	0.525	31.35	69.27	8.049	1.408
SPAC10F6.16	mug134	0.5145	-5.435	0.289	24.88	57.05	13.81	0.9972
SPAC630.10	SPAC630.10	0.01468	-5.426	1.833	10.87	30.67	2.22	0.3895
SPCC1393.10	ctr4	0.5112	-5.404	0.291	12.5	33.7	12.71	3.426
SPBC30B4.02C	SPBC30B4.02c	0.5628	-5.387	0.250	29.23	65.16	15.44	1.74
SPBC21C3.06	SPBC21C3.06	0.4536	-5.379	0.343	25.13	57.43	11.64	1.443
SPBC215.13	SPBC215.13	0.4487	-5.346	0.348	25.58	58.21	11.36	1.708
SPAC105.03C	SPAC105.03c	0.4634	-5.345	0.334	34.24	74.51	11.8	1.66
SPCC1795.12C	SPCC1795.12c	0.4845	-5.339	0.315	23.01	53.35	4.581	6.465
SPCC24B10.06	SPCC24B10.06	0.3136	-5.337	0.504	32.34	70.91	8.216	1.227
SPBC947.15C	SPBC947.15c	0.2764	-5.332	0.558	18.46	44.78	5.894	3.167
SPCC24B10.08C	ada2	0.3782	-5.313	0.422	16.83	41.67	5.981	4.531
SPAPB1A11.02	SPAPB1A11.02	0.2942	-5.309	0.531	20.01	47.65	7.347	2.261
SPBC18E5.09C	EMPTY	0.4764	-5.308	0.322	20.95	49.43	4.377	6.313
SPAC922.06	SPAC922.06	0.1864	-5.298	0.730	30.83	68	5.777	1.304
SPAC8E11.10	SPAC8E11.10	0.1606	-5.284	0.794	23.9	54.92	5.054	1.805
SPAPB17E12.12C	SPAPB17E12.12(	0.5563	-5.268	0.255	27.53	61.73	14.81	1.757
SPAC1F5.07C	hem14	0.43	-5.266	0.367	9.963	28.66	9.195	3.815
SPBC1271.12	kes1	0.2071	-5.26	0.684	19.18	46.01	5.064	2.568
SPAC5H10.12C	SPAC5H10.12c	0.4582	-5.238	0.339	22.95	53.05	11.27	2.078
SPCC1919.12C	SPCC1919.12c	0.2545	-5.223	0.594	31.57	69.25	6.831	1.508
SPBC839.07	ibp1	0.0009	-5.193	3.046	25.59	57.93	1.194	0.5569
SPCC23B6.03C	tel1	0.5181	-5.185	0.286	26.13	58.95	13.26	1.212
SPBC1703.06	pof10	0.2179	-5.165	0.662	29.85	65.9	6.107	1.479
SPCC622.11	SPCC622.11	0.4956	-5.165	0.305	25.82	58.32	12.37	1.643
SPAC17A5.14	exo2	0.4391	-5.147	0.357	26.16	58.92	10.83	0.895
SPAC31G5.14	gcv1	0.5915	-5.146	0.228	27.61	61.65	16.03	1.521
SPBC1348.02	SPBC1348.02	0.5653	-5.114	0.248	18.59	44.6	13.5	4.148
SPBC21C3.14C	SPBC21C3.14c	0.103	-5.104	0.987	29.63	65.38	4.21	0.9956
SPCC825.01	SPCC825.01	0.5164	-5.093	0.287	26.72	59.88	12.45	2.755
SPAC1002.03C	gls2	0.4284	-5.092	0.368	27.11	60.6	10.23	1.788
SPBC19C7.05	SPBC19C7.05	0.01971	-5.085	1.705	28.56	63.33	2.404	0.8341
SPAC12G12.11C	SPAC12G12.11c	0.5165	-5.048	0.287	23.05	52.89	12.2	2.996
SPCC364.06	nap1	0.5666	-5.032	0.247	25.67	57.78	13.9	3.313
SPBC2G2.07C	mug178	0.02013	-5.03	1.696	14.47	36.69	2.398	0.7835
SPBC23E6.08	sat1	0.232	-5.003	0.635	7.954	24.39	2.165	3.312
SPAC12G12.12	SPAC12G12.12	0.04572	-4.991	1.340	24.57	55.63	3.043	0.7551
SPAC17A5.10	SPAC17A5.10	0.09071	-4.99	1.042	29.97	65.8	3.891	1.045
SPCC63.03	SPCC63.03	0.5679	-4.99	0.246	27.16	60.5	14.66	0.6853
SPAPB2B4.07	SPAPB2B4.07	0.5386	-4.987	0.269	23.98	54.51	11.92	4.166
SPBC26H8.05C	SPBC26H8.05c	0.4765	-4.976	0.322	25.09	56.58	10.99	2.504
SPBC800.03	clr3	0.3223	-4.953	0.492	21.14	49.11	7.781	1.191
SPCC663.15C	SPCC663.15c	0.2837	-4.925	0.547	31.03	67.67	7.009	1.233
SPCC320.07C	mde7	0.2864	-4.915	0.543	32.56	70.54	7.07	1.107
SPBC660.10	SPBC660.10	0.106	-4.899	0.975	17.11	41.42	2.846	2.04
SPBP8B7.04	mug45	0.5307	-4.892	0.275	25.85	57.86	12.99	0.7619
SPAC27F1.03C	uch1	0.6473	-4.877	0.189	31.04	67.6	17.96	1.637
SPAC821.03C	SPAC821.03c	0.4815	-4.876	0.317	26.47	59	11.4	0.8816
SPBC1289.15	SPBC1289.15	0.5153	-4.874	0.288	25.96	58.04	11.63	3.062
SPCC2H8.05C	SPCC2H8.05c	0.4739	-4.871	0.324	25.14	56.48	11.02	1.59
SPBC17D1.02	dph2	0.5697	-4.858	0.244	27.93	61.72	14.3	0.9835
SPBC83.05	SPBC83.05	0.2426	-4.843	0.615	30.65	66.81	6.112	1.457
SPAC13G6.08	SPAC13G6.08	0.5524	-4.843	0.258	20.52	47.73	3.699	7.106
SPBC11C11.08	srp1	0.2007	-4.833	0.697	26.02	58.08	4.746	2.214
SPBC2G2.01C	liz1	0.159	-4.823	0.799	14.23	35.86	1.458	2.595
SPAC32A11.03C	phx1	0.02264	-4.817	1.645	26.51	58.96	2.082	1.13

SPBC29A3.18	cyt1	0.2344	-4.795	0.630	4.234	17	6.419	1.659
SPBC1347.03	meu14	0.5516	-4.784	0.258	27.07	59.96	13.34	1.329
SPBC11C11.11C	SPBC11C11.11c	0.3894	-4.753	0.410	20.09	46.76	8.536	1.958
SPAC22F8.03C	SPAC22F8.03c	0.1022	-4.747	0.991	30.44	66.23	3.873	1.076
SPBC839.15C	ef1a-c	0.5885	-4.734	0.230	13.53	34.38	12.91	4.518
SPAC26F1.07	SPAC26F1.07	0.4659	-4.717	0.332	17.67	42.14	9.057	3.634
SPAC823.02	SPAC823.02	0.5307	-4.715	0.275	25.27	56.44	12.23	1.998
SPAC17D4.01	pex7	0.01204	-4.71	1.919	28.77	63.01	1.491	1.023
SPAC26F1.04C	etr1	0.1812	-4.706	0.742	30.1	65.51	5.067	1.087
SPBC337.09	erg28	0.5468	-4.697	0.262	13.45	34.15	11.66	3.797
SPBC12D12.07C	trx2	0.3713	-4.669	0.430	4.946	18.1	7.967	1.985
SPBC26H8.09C	snf59	0.2483	-4.664	0.605	32.49	69.93	6.03	1.248
SPAC821.05	SPAC821.05	0.2218	-4.623	0.654	13.29	33.72	3.048	2.862
SPAC144.14	klp8	0.5258	-4.618	0.279	25.52	56.72	12.12	0.58
SPAC22A12.17C	SPAC22A12.17c	0.4611	-4.611	0.336	18.14	42.82	8.605	3.63
SPAC227.10	SPAC227.10	0.09546	-4.606	1.020	30.79	66.61	3.611	1.146
SPBC16H5.11C	skb1	0.4986	-4.591	0.302	23.61	53.07	6.499	5.284
SPAC23G3.02C	sib1	0.4576	-4.581	0.340	25.06	55.79	9.549	2.409
SPAC22E12.05C	rer1	0.5463	-4.581	0.263	27.59	60.56	12.56	1.424
SPBC19C2.06C	mug124	0.01694	-4.555	1.771	27.83	60.96	1.871	0.9725
SPAC9.10	thi9	0.348	-4.547	0.458	23.97	53.66	7.498	1.501
SPAC27E2.02	SPAC27E2.02	0.001284	-4.544	2.891	12.53	32.13	0.8556	0.6075
SPCC24B10.07	gad8	0.2132	-4.54	0.671	0.2975	9.104	5.423	0.2975
SPAC1002.20	SPAC1002.20	0.2874	-4.518	0.542	31.91	68.56	6.427	1.352
SPCC16C4.09	sts5	0.4194	-4.514	0.377	25.36	56.23	8.889	1.518
SPAC17C9.10	stm1	0.2174	-4.497	0.663	22.36	50.54	4.687	2.091
SPBC8D2.10C	rmt3	0.6432	-4.496	0.192	25.28	56.05	15.89	2.917
SPAC22A12.14C	SPAC22A12.14c	0.5103	-4.475	0.292	25.98	57.32	11.17	1.302
SPCC16A11.03C	SPCC16A11.03c	0.3679	-4.471	0.434	32.99	70.5	7.918	0.7155
SPAC13G6.01C	rad8	0.5576	-4.468	0.254	26.06	57.47	12.43	2.094
SPAC2F3.12C	plp1	0.4305	-4.443	0.366	23.73	53.02	3.246	4.736
SPAC9E9.10C	cbh1	0.5756	-4.429	0.240	32.8	70.08	13.24	1.001
SPCP1E11.05C	are2	0.5595	-4.403	0.252	25.45	56.18	10.75	4.205
SPBC1711.11	SPBC1711.11	0.3149	-4.4	0.502	32.82	70.05	6.695	1.385
SPCC970.02	SPCC970.02	0.6431	-4.393	0.192	26.35	57.86	14.99	3.812
SPAC589.08C	dam1	0.597	-4.39	0.224	33.12	70.61	13.76	1.849
SPCC1442.07C	SPCC1442.07c	0.02089	-4.38	1.680	25.7	56.61	1.65	1.078
SPAC630.13C	tsc2	0.06472	-4.376	1.189	27.94	60.82	2.85	1.164
SPCC584.15C	SPCC584.15c	0.4934	-4.367	0.307	24.58	54.49	9.879	2.579
SPAC186.07C	SPAC186.07c	0.01416	-4.347	1.849	31.02	66.57	1.83	0.7873
SPAC227.18	lys3	0.3796	-4.337	0.421	30.81	66.15	7.854	0.9986
SPBC1711.01C	mat1-m	0.5526	-4.33	0.258	26.2	57.47	11.98	1.689
SPBC16A3.16	SPBC16A3.16	0.0215	-4.32	1.668	29.31	63.29	1.888	0.9758
SPCC663.03	pmd1	0.2771	-4.319	0.557	31.64	67.68	6.068	0.9613
SPAC227.11C	SPAC227.11c	0.6585	-4.319	0.181	25.19	55.53	16.18	2.555
SPCC126.12	SPCC126.12	0.07177	-4.317	1.144	27.65	60.16	3.003	1.061
SPCC1223.06	tea1	0.5825	-4.314	0.235	22.62	50.7	12.39	3.049
SPAC105.02C	SPAC105.02c	0.1725	-4.3	0.763	32.02	68.37	4.5	1.026
SPBC582.09	pex11	0.3616	-4.3	0.442	34.68	73.37	7.441	1.049
SPCPJ732.01	vps5	0.5562	-4.3	0.255	10.28	27.45	9.718	4.571
SPCC4B3.02C	SPCC4B3.02c	0.5905	-4.299	0.229	23.81	52.91	5.669	6.739
SPCC548.05C	SPCC548.05c	0.1243	-4.292	0.906	31.54	67.45	3.812	1.016
SPBC2D10.04	SPBC2D10.04	0.6163	-4.269	0.210	24.05	53.29	5.278	7.329
SPAC6B12.05C	ies2	0.3374	-4.244	0.472	25.63	56.23	6.3	2.225
SPBC56F2.10C	alg5	0.2373	-4.235	0.625	17.21	40.35	0.8163	2.874
SPAC7D4.02C	sfp47	0.4465	-4.199	0.350	25.08	55.1	8.943	1.049
SPCC18B5.01C	bfr1	0.5585	-4.193	0.253	20.82	47.08	10.46	3.77
SPBC4C3.09	SPBC4C3.09	0.5587	-4.187	0.253	27.5	59.64	11.96	0.8529
SPBC19C2.09	sre1	0.7261	-4.186	0.139	20.86	47.14	18.41	5.581
SPBC1778.04	spo6	0.6463	-4.158	0.190	30.03	64.34	15.18	1.743
SPCC1795.01C	mad3	0.5048	-4.131	0.297	24.6	54.08	10.03	1.653
SPCC306.11	SPCC306.11	0.6314	-4.126	0.200	19.58	44.63	12.76	4.434
SPCC553.03	pex1	0.1912	-4.115	0.719	32.85	69.57	3.885	1.879

SPAC5H10.01	SPAC5H10.01	0.007884	-4.114	2.103	26.85	58.28	1.317	0.7783
SPAC139.06	hat1	0.5326	-4.109	0.274	23.86	52.64	4.707	5.506
SPAC29B12.04	snz1	0.1155	-4.094	0.937	14.51	35.02	3.31	1.316
SPBC18E5.10	SPBC18E5.10	0.5717	-4.088	0.243	25.39	55.47	11.75	2.138
SPBP22H7.06	SPBP22H7.06	0.3376	-4.082	0.472	31.06	66.14	6.392	1.698
SPBC216.04C	SPBC216.04c	0.5765	-4.079	0.239	26.6	57.74	12.22	0.9783
SPAC8E11.01C	SPAC8E11.01c	0.6175	-4.078	0.209	30.77	65.6	13.63	1.594
SPAP27G11.10C	nup184	0.07491	-4.071	1.125	28.74	61.75	2.66	1.249
SPBC23G7.04C	nif1	0.6776	-4.07	0.169	30.67	65.38	16.55	1.478
SPAC6C3.03C	SPAC6C3.03c	0.06368	-4.068	1.196	29.42	63.02	2.738	0.9146
SPAC29B12.13	SPAC29B12.13	0.6136	-4.047	0.212	29.24	62.66	13.43	1.293
SPAC4C5.02C	ryh1	0.3273	-4.032	0.485	7.779	22.23	2.716	3.327
SPAC1565.03	SPAC1565.03	0.529	-4.005	0.277	26.53	57.47	10.58	0.7176
SPAC14C4.13	rad17	0.07629	-4.004	1.118	30.03	64.06	2.844	1.008
SPBC365.07C	SPBC365.07c	0.5338	-3.986	0.273	27.61	59.47	10.51	1.433
SPAC2H10.02C	SPAC2H10.02c	0.2977	-3.984	0.526	34.07	71.62	5.842	1.13
SPCC1840.11	csl4	0.5744	-3.984	0.241	18.69	42.67	8.613	4.906
SPCC1884.02	nic1	0.3564	-3.982	0.448	22.85	50.49	3.026	3.496
SPCP31B10.07	eft202	0.5424	-3.982	0.266	26.24	56.89	10.74	1.495
SPCC4G3.09C	gyp3	0.5858	-3.973	0.232	23.37	51.46	10.84	3.684
SPCC1020.07	SPCC1020.07	0.08373	-3.937	1.077	22.95	50.6	2.981	0.5459
SPBC25B2.02C	mam1	0.01613	-3.924	1.792	29.18	62.31	1.501	0.871
SPBC31F10.17C	SPBC31F10.17c	0.6154	-3.923	0.211	30.47	64.74	12.61	2.625
SPAC14C4.01C	SPAC14C4.01c	0.5857	-3.911	0.232	27.45	59.03	11.98	1.158
SPCC1620.14C	snf22	0.5349	-3.894	0.272	28.44	60.86	10.42	0.8922
SPBC1685.05	SPBC1685.05	0.01515	-3.882	1.820	27.94	59.89	1.457	0.8504
SPBC36B7.04	SPBC36B7.04	0.6162	-3.881	0.210	27.6	59.25	12.63	2.344
SPAC11D3.04C	SPAC11D3.04c	0.6063	-3.879	0.217	22.31	49.3	12.37	2.02
SPAC22H12.01C	mug35	0.6929	-3.878	0.159	27.69	59.42	15.03	4.657
SPAC2F7.11	nrd1	0.2996	-3.875	0.523	30.04	63.82	4.639	2.361
SPAC11D3.14C	SPAC11D3.14c	0.07341	-3.873	1.134	29.3	62.45	2.753	0.8811
SPAPB17E12.05	rpl3703	0.5977	-3.873	0.224	8.983	24.2	8.643	5.214
SPBC1683.11C	SPBC1683.11c	0.5601	-3.871	0.252	27.4	58.86	11.06	0.9998
SPCC23B6.04C	SPCC23B6.04c	0.6117	-3.867	0.213	27.97	59.91	12.49	2.16
SPBPJ4664.02	SPBPJ4664.02	0.5766	-3.859	0.239	27.51	59.05	11.47	1.374
SPBC16G5.02C	SPBC16G5.02c	0.2309	-3.848	0.637	31.86	67.21	4.534	1.463
SPAC12G12.16C	SPAC12G12.16c	0.547	-3.844	0.262	25.13	54.53	9.241	3.419
SPBC25B2.10	SPBC25B2.10	0.6186	-3.844	0.209	17.65	40.46	9.492	5.312
SPAC1B2.04	cox6	0.222	-3.825	0.654	2.346	11.62	4.45	1.356
SPAP27G11.16	SPAP27G11.16	0.5957	-3.824	0.225	26.98	57.97	11.82	1.971
SPAC1F7.06	SPAC1F7.06	0.2504	-3.818	0.601	31.6	66.66	4.928	1.13
SPBC6B1.09C	nbs1	0.6738	-3.815	0.171	30.69	64.94	14.86	2.797
SPAC3F10.17	SPAC3F10.17	0.5823	-3.814	0.235	28.92	61.61	11.47	1.55
SPAC23C4.08	rho3	0.5018	-3.809	0.299	24.04	52.42	9.299	1.093
SPAC17A5.05C	SPAC17A5.05c	0.2149	-3.799	0.668	32.61	68.53	4.425	1.16
SPAC30D11.09	cwf19	0.2672	-3.793	0.573	14.91	35.2	4.003	2.256
SPAC144.03	ade2	0.07859	-3.781	1.105	29.26	62.18	2.794	0.7224
SPAC1952.10C	SPAC1952.10c	0.4868	-3.781	0.313	22.76	49.96	8.706	1.643
SPAC17A5.11	rec12	0.5822	-3.779	0.235	23.19	50.77	4.064	5.91
SPAC694.02	SPAC694.02	0.09774	-3.763	1.010	10.87	27.53	2.463	1.403
SPAC1687.14C	SPAC1687.14c	0.5892	-3.761	0.230	28.55	60.81	11.51	1.608
SPAC31G5.10	eta2	0.4924	-3.759	0.308	23.47	51.26	6.202	3.937
SPAC8E11.06	SPAC8E11.06	0.5285	-3.744	0.277	25.11	54.31	9.806	1.062
SPBC336.10C	tif512	0.02562	-3.73	1.591	28.8	61.23	1.531	0.9553
SPBC1683.13C	cha4	0.2883	-3.722	0.540	34.78	72.46	5.363	0.9212
SPAC607.07C	SPAC607.07c	0.6689	-3.721	0.175	28.91	61.41	14.59	1.766
SPAC1420.01C	SPAC1420.01c	0.3455	-3.713	0.462	22.24	48.85	5.291	2.27
SPCP20C8.01C	SPCP20C8.01c	0.6022	-3.712	0.220	26.43	56.72	9.657	4.369
SPAC5H10.11	gmh1	0.02152	-3.665	1.667	28.83	61.16	1.652	0.7952
SPCC1620.12C	SPCC1620.12c	0.3473	-3.661	0.459	23.17	50.5	3.944	2.881
SPCC1223.04C	set11	0.1111	-3.64	0.954	27.7	58.98	2.928	1.118
SPBC3B8.06	SPBC3B8.06	0.5851	-3.639	0.233	24.77	53.47	10.18	3.062
SPAPYUG7.03C	mid2	0.1031	-3.635	0.987	28.86	61.16	2.49	1.353

SPAC1805.09C	fmt1	0.4348	-3.635	0.362	21.73	47.75	7.153	1.853
SPBC30D10.09C	SPBC30D10.09c	0.6928	-3.634	0.159	30.52	64.28	14.74	3.505
SPAC664.02C	arp8	0.6488	-3.629	0.188	23.74	51.51	11.54	4.373
SPAC824.07	SPAC824.07	0.5658	-3.626	0.247	25.59	54.98	10.42	1.412
SPBC1773.03C	SPBC1773.03c	0.6877	-3.613	0.163	30.34	63.91	15.3	0.8201
SPAPJ698.02C	rps002	0.2738	-3.6	0.563	2.768	11.98	4.094	2.048
SPCC126.08C	SPCC126.08c	0.2969	-3.599	0.527	32.71	68.33	5.013	1.542
SPBC11C11.01	SPBC11C11.01	0.5597	-3.598	0.252	27.03	57.64	10.23	1.116
SPAC227.01C	SPAC227.01c	0.6511	-3.573	0.186	28.57	60.5	13.25	1.511
SPAC24C9.12C	SPAC24C9.12c	0.5759	-3.571	0.240	23.54	51.02	9.975	2.629
SPAC6B12.07C	SPAC6B12.07c	0.4481	-3.556	0.349	23.95	51.78	6.137	2.923
SPAC17G6.04C	cpp1	0.2458	-3.553	0.609	19.88	44.11	3.766	1.904
SPAC20G4.07C	sts1	0.4107	-3.544	0.386	9.9	25.3	6.159	2.248
SPCC4G3.17	SPCC4G3.17	0.5796	-3.541	0.237	27.64	58.69	10.38	1.937
SPCC126.11C	SPCC126.11c	0.5873	-3.538	0.231	25.2	54.09	10.58	2.006
SPBC4F6.05C	SPBC4F6.05c	0.7058	-3.53	0.151	32.55	67.9	15.04	3.486
SPBC20F10.02C	SPBC20F10.02c	0.05992	-3.523	1.222	29.85	62.81	1.681	1.198
SPCC613.07	SPCC613.07	0.2221	-3.509	0.653	29.42	61.98	4.101	1.215
SPBPB7E8.02	SPBPB7E8.02	0.02947	-3.5	1.531	25.19	53.99	1.655	0.8614
SPAC22A12.06C	SPAC22A12.06c	0.7534	-3.484	0.123	20.19	44.56	17.98	3.996
SPAC22H10.07	scd2	0.5791	-3.47	0.237	9.917	25.2	9.022	3.41
SPBC29A3.07C	sab14	0.1438	-3.461	0.842	17.92	40.25	2.988	1.291
SPCC4G3.15C	not2	0.5549	-3.438	0.256	28.81	60.7	9.525	1.506
SPBC19F5.01C	puc1	0.4306	-3.432	0.366	30.1	63.12	6.609	1.852
SPAC27D7.02C	SPAC27D7.02c	0.08313	-3.431	1.080	31.12	65.03	1.583	1.349
SPBC23G7.12C	rpt6	0.3618	-3.431	0.442	32.79	68.18	5.633	1.588
SPBC839.17C	fkh1	0.3416	-3.421	0.466	24.56	52.66	5.424	1.404
SPBC119.16C	SPBC119.16c	0.596	-3.414	0.225	28.82	60.67	10.54	1.811
SPAC11D3.17	SPAC11D3.17	0.6198	-3.402	0.208	26.37	56.03	9.98	3.702
SPBC106.04	ada1	0.6088	-3.388	0.216	11.19	27.45	9.248	3.878
SPCC1235.13	ght6	0.4466	-3.386	0.350	38.5	78.83	7.073	1.33
SPBC30D10.05C	SPBC30D10.05c	0.6351	-3.38	0.197	26.92	57.04	11.66	2.13
SPAC17H9.06C	SPAC17H9.06c	0.597	-3.374	0.224	26.93	57.03	10.27	2.171
SPAC1782.08C	rex3	0.5779	-3.342	0.238	20.07	44.06	7.748	3.915
SPAC139.05	SPAC139.05	0.6215	-3.342	0.207	27.87	58.74	11.26	1.477
SPBC12C2.07C	SPBC12C2.07c	0.3984	-3.338	0.400	24.87	53.08	6.369	0.5048
SPAC144.06	apl5	0.4666	-3.336	0.331	20.48	44.82	6.173	2.758
SPAC23H4.12	alp13	0.01581	-3.334	1.801	30.15	63.03	1.231	0.7494
SPCC613.01	SPCC613.01	0.2798	-3.331	0.553	21.3	46.36	4.585	1.149
SPBC215.10	SPBC215.10	0.5478	-3.326	0.261	26.63	56.38	8.137	2.845
SPAC9E9.05	SPAC9E9.05	0.594	-3.319	0.226	28.19	59.31	10.33	1.319
SPCC1322.05C	SPCC1322.05c	0.5198	-3.316	0.284	25.73	54.67	8.212	1.738
SPBC800.07C	tsf1	0.2051	-3.307	0.688	12.59	29.92	3.832	0.7016
SPAC17H9.12C	SPAC17H9.12c	0.4193	-3.303	0.377	22.08	47.78	6.407	1.359
SPBC405.06	SPBC405.06	0.6954	-3.27	0.158	23.07	49.57	12.01	4.694
SPBC36B7.08C	SPBC36B7.08c	0.665	-3.264	0.177	29.05	60.81	12.54	1.841
SPCC1223.12C	meu10	0.07487	-3.249	1.126	30.52	63.56	2.204	0.9259
SPBC16E9.02C	SPBC16E9.02c	0.3808	-3.248	0.419	24.14	51.54	4.771	2.315
SPAC521.03	SPAC521.03	0.6588	-3.248	0.181	28.81	60.34	12.48	0.6595
SPCC338.02	mug112	0.6403	-3.241	0.194	26.7	56.35	11.5	1.719
SPAC1851.02	slc1	0.7194	-3.241	0.143	27.35	57.58	15.23	1.725
SPBC800.11	SPBC800.11	0.5911	-3.24	0.228	28.21	59.19	9.789	1.869
SPBC16E9.12C	pab2	0.5669	-3.227	0.246	27.29	57.43	9.008	1.971
SPAP27G11.14C	SPAP27G11.14c	0.3974	-3.222	0.401	10.32	25.48	3.694	2.898
SPAC14C4.12C	laf1	0.4434	-3.221	0.353	20.29	44.26	6.829	0.6964
SPAC10F6.08C	nht1	0.2797	-3.211	0.553	21.63	46.76	3.769	1.811
SPAC9G1.12	cpd1	0.2246	-3.202	0.649	13.66	31.74	3.841	0.9663
SPBC646.06C	agn2	0.5557	-3.2	0.255	26.88	56.62	7.228	3.392
SPBC56F2.06	mug147	0.2787	-3.197	0.555	30.02	62.52	4.454	0.9357
SPBC2G5.04C	SPBC2G5.04c	0.5381	-3.193	0.269	26.88	56.6	8.064	2.135
SPCC965.07C	gst2	0.206	-3.19	0.686	13.72	31.82	3.709	0.6667
SPAC31G5.21	SPAC31G5.21	0.6224	-3.186	0.206	25.11	53.25	10.26	2.517
SPBC18E5.13	SPBC18E5.13	0.6384	-3.184	0.195	27.66	58.04	11.23	1.668

SPAC926.09C	fas1	0.5975	-3.182	0.224	27.05	56.9	9.83	1.78
SPCC550.03C	SPCC550.03c	0.5958	-3.175	0.225	16.99	37.94	7.843	3.843
SPBC1604.11	atp17	0.06828	-3.16	1.166	26.73	56.26	2.162	0.7616
SPBC1604.20C	tea2	0.5281	-3.159	0.277	18.94	41.6	4.414	4.017
SPCC1183.10	wtf10	0.1935	-3.152	0.713	32.21	66.55	3.423	1.001
SPAC18G6.05C	SPAC18G6.05c	0.4151	-3.151	0.382	23.29	49.77	3.078	3.113
SPCC1620.07C	SPCC1620.07c	0.4613	-3.146	0.336	30.97	64.22	6.757	1.402
SPBC29B5.01	atf1	0.2673	-3.141	0.573	16.53	37.02	4.207	1.022
SPAC4F10.06	SPAC4F10.06	0.3439	-3.14	0.464	31.88	65.92	4.93	1.42
SPAC17H9.11	gmf1	0.7236	-3.137	0.141	28.25	59.07	15.04	1.463
SPBC530.13	lsc1	0.5564	-3.127	0.255	25	52.94	8.648	1.503
SPAC2F3.16	SPAC2F3.16	0.6348	-3.126	0.197	28.13	58.84	11	1.33
SPBC530.01	gyp1	0.3219	-3.125	0.492	18.33	40.38	4.704	1.278
SPAC13A11.06	SPAC13A11.06	0.6701	-3.124	0.174	27.52	57.67	12.08	2.126
SPCP25A2.02C	rhp26	0.6204	-3.116	0.207	26.84	56.37	10.22	2.02
SPCC188.09C	SPCC188.09c	0.5866	-3.102	0.232	24.7	52.33	9.445	1.255
SPBC776.01	rpl29	0.7387	-3.097	0.132	26.77	56.21	14.49	4.137
SPAPJ691.03	SPAPJ691.03	0.01626	-3.094	1.789	30.41	63.07	1.05	0.7242
SPBC16D10.05	mok13	0.6822	-3.092	0.166	27.96	58.44	12.39	2.316
SPAC3H8.03	img2	0.2688	-3.09	0.571	20.73	44.84	3.729	1.561
SPBC15C4.02	SPBC15C4.02	0.5851	-3.075	0.233	25.32	53.44	8.839	2.275
SPBC543.10	get1	0.662	-3.072	0.179	27.55	57.64	11.17	2.762
SPBC56F2.11	met6	0.4452	-3.068	0.351	7.606	20.09	6.426	1.094
SPAC630.05	gyp7	0.3342	-3.065	0.476	32.39	66.72	4.854	1.078
SPBC4F6.06	kin1	0.4189	-3.061	0.378	6.489	17.97	1.834	3.208
SPBC1105.09	ubc15	0.5437	-3.044	0.265	23.06	49.14	8.038	1.653
SPBC725.11C	php2	0.1146	-3.035	0.941	24.65	52.11	2.627	0.4473
SPBC18H10.08C	ubp4	0.5208	-3.034	0.283	25.65	53.99	6.351	2.892
SPCC594.07C	bqt3	0.6459	-3.034	0.190	24.75	52.3	9.991	3.216
SPBC725.07	pex5	0.5068	-3.032	0.295	22.1	47.31	4.522	3.519
SPAC1250.02	mug95	0.3259	-3.03	0.487	22.6	48.23	4.627	1.21
SPBC16D10.03	pgp2	0.656	-3.026	0.183	29.2	60.65	11.36	1.455
SPAC19A8.11C	SPAC19A8.11c	0.6619	-3.023	0.179	27.11	56.71	11.59	1.395
SPAC4F8.15	itr1	0.8323	-3.015	0.080	26.24	55.07	20.59	8.037
SPAC3C7.02C	SPAC3C7.02c	0.72	-3.011	0.143	31.44	64.84	14.07	1.99
SPCC584.11C	SPCC584.11c	0.1268	-2.998	0.897	14.52	32.98	2.43	1.081
SPCC4G3.08	psk1	0.6125	-2.989	0.213	27.72	57.8	9.432	2.157
SPAPB1E7.12	rps602	0.1694	-2.986	0.771	30.74	63.48	2.956	1.012
SPBC29A10.09C	SPBC29A10.09c	0.6519	-2.98	0.186	27.29	56.97	11.14	0.9999
SPBC18E5.11C	edc3	0.5777	-2.974	0.238	26.65	55.76	8.747	1.433
SPBC16G5.13	ptf2	0.6282	-2.971	0.202	27.77	57.87	10.3	1.01
SPAC57A7.05	SPAC57A7.05	0.7323	-2.97	0.135	25.87	54.28	12.69	4.741
SPBC29B5.04C	SPBC29B5.04c	0.4275	-2.968	0.369	23.99	50.73	6	0.8688
SPCC569.02C	SPCC569.02c	0.7772	-2.964	0.109	18.53	40.46	14.43	6.42
SPAC12B10.16C	mug157	0.6653	-2.962	0.177	25.98	54.48	11.12	2.302
SPAC2C4.17C	SPAC2C4.17c	0.6685	-2.959	0.175	27.42	57.18	11.22	2.334
SPBC887.10	mcs4	0.7751	-2.955	0.111	33.42	68.47	17.36	2.518
SPBC16H5.08C	SPBC16H5.08c	0.5651	-2.952	0.248	19.41	42.09	8.369	1.43
SPBC146.02	SPBC146.02	0.3784	-2.949	0.422	26.35	55.15	5.088	1.325
SPBC21.03C	SPBC21.03c	0.5944	-2.939	0.226	18.11	39.61	8.835	1.961
SPBC354.01	gtp1	0.7456	-2.939	0.127	31.7	65.2	15.32	1.751
SPBC17G9.10	rpl1102	0.5504	-2.938	0.259	24.07	50.83	4.899	3.804
SPBC1289.16C	cao2	0.559	-2.917	0.253	35.14	71.64	8.006	1.68
SPBC409.19C	SPBC409.19c	0.6369	-2.906	0.196	10.5	25.24	8.65	3.585
SPAC9.08C	SPAC9.08c	0.5496	-2.902	0.260	21.68	46.27	6.785	2.798
SPAPB24D3.04C	mag1	0.6206	-2.902	0.207	26.33	55.03	9.575	1.766
SPCC18B5.05C	SPCC18B5.05c	0.4619	-2.896	0.335	24.52	51.6	5.679	2.057
SPCC1259.10	pgp1	0.678	-2.895	0.169	30.97	63.75	11.82	0.8993
SPBC2A9.13	SPBC2A9.13	0.6272	-2.89	0.203	27.3	56.83	9.316	2.503
SPBC1539.02	SPBC1539.02	0.1339	-2.884	0.873	29.83	61.57	2.074	1.243
SPCC18B5.07C	nup61	0.452	-2.878	0.345	31.95	65.56	6.054	1.23
SPAC1F3.10C	Oct-01	0.5475	-2.875	0.262	25.27	52.96	7.484	1.907
SPAC1751.01C	gti1	0.7004	-2.874	0.155	26.19	54.7	12.18	2.489

SPBC1711.04	SPBC1711.04	0.7005	-2.872	0.155	26.44	55.17	11.17	3.821
SPAC869.02C	SPAC869.02c	0.0716	-2.87	1.145	27.42	57.02	1.854	0.862
SPBC1773.09C	mug184	0.4221	-2.87	0.375	23.49	49.62	5.534	1.331
SPBP8B7.25	cyp4	0.4129	-2.868	0.384	23.16	48.98	5.596	0.8328
SPAC9G1.07	SPAC9G1.07	0.5554	-2.864	0.255	26.22	54.74	7.213	2.436
SPBC12C2.02C	ste20	0.3387	-2.863	0.470	12.4	28.74	3.591	2.17
SPBC1815.01	eno101	0.05052	-2.851	1.297	27.76	57.62	1.767	0.6204
SPAC23A1.06C	cmk2	0.5658	-2.848	0.247	25.57	53.49	7.572	2.264
SPCC1183.06	ung1	0.485	-2.821	0.314	25.25	52.84	6.152	1.775
SPAC23A1.14C	SPAC23A1.14c	0.6744	-2.819	0.171	24.91	52.19	10.8	2.435
SPCC1223.02	nmt1	0.3235	-2.818	0.490	19.28	41.58	1.669	2.329
SPAC11E3.10	SPAC11E3.10	0.4678	-2.814	0.330	24.28	51	6.153	1.247
SPAC823.16C	mug179	0.669	-2.814	0.175	24.22	50.87	5.775	5.369
SPAC458.06	SPAC458.06	0.6672	-2.803	0.176	22.61	47.84	10.08	2.946
SPAC22A12.01C	pso2	0.6792	-2.803	0.168	26.61	55.37	10.99	2.339
SPAC19A8.04	erg5	0.5987	-2.8	0.223	26.41	54.98	8.2	2.373
SPCC830.07C	psi1	0.6067	-2.8	0.217	23.59	49.67	7.657	3.139
SPBC13E7.09	vrp1	0.4408	-2.793	0.356	24.87	52.06	5.749	1.094
SPCC1020.13C	SPCC1020.13c	0.5165	-2.792	0.287	19.45	41.86	4.411	3.273
SPAC16C9.07	pom2	0.6475	-2.787	0.189	25.53	53.3	9.725	2.319
SPBC359.01	SPBC359.01	0.7021	-2.786	0.154	23.7	49.85	11.61	2.87
SPBC15D4.07C	atg9	0.3849	-2.784	0.415	23.78	49.99	4.873	1.274
SPAC16.04	dus3	0.5529	-2.782	0.257	30.08	61.84	7.654	1.25
SPAC3C7.06C	pit1	0.3939	-2.767	0.405	22.1	46.8	3.677	2.287
SPAC1834.08	mak1	0.3931	-2.758	0.405	22.64	47.81	2.965	2.5
SPAC14C4.16	dad3	0.126	-2.722	0.900	30.45	62.44	1.736	1.203
SPCC191.09C	gst1	0.4318	-2.708	0.365	32.21	65.73	5.325	1.315
SPAC1783.01	SPAC1783.01	0.508	-2.702	0.294	25.7	53.45	5.586	2.422
SPAC2G11.10C	SPAC2G11.10c	0.4167	-2.695	0.380	23.46	49.22	4.268	2.097
SPCC188.07	ccq1	0.5871	-2.682	0.231	13.96	31.32	5.242	3.731
SPAC25H1.05	meu29	0.4154	-2.681	0.382	4.076	12.72	4.364	1.998
SPBC776.16	SPBC776.16	0.09298	-2.678	1.032	29.66	60.87	1.493	1.058
SPCC16A11.07	coq10	0.3959	-2.678	0.402	24.49	51.13	3.933	2.054
SPCC1450.09C	SPCC1450.09c	0.4276	-2.663	0.369	33.87	68.76	5.258	1.141
SPAC19G12.08	scs7	0.628	-2.66	0.202	25.77	53.5	8.262	2.706
SPAC144.01	SPAC144.01	0.7258	-2.651	0.139	30.88	63.11	12.5	2.21
SPBC1685.01	pmp1	0.5075	-2.648	0.295	25.24	52.48	6.524	0.9027
SPAC1527.02	sft2	0.5727	-2.648	0.242	24.32	50.76	7.869	0.4645
SPAC2F7.02C	SPAC2F7.02c	0.5718	-2.645	0.243	25.86	53.65	2.565	4.045
SPBC365.04C	SPBC365.04c	0.5731	-2.644	0.242	22.68	47.66	7.329	1.931
SPAC222.13C	SPAC222.13c	0.6399	-2.644	0.194	26.63	55.09	9.47	1.055
SPAC20G4.08	SPAC20G4.08	0.6927	-2.64	0.159	26.37	54.6	11.31	1.026
SPAPB21F2.03	SPAPB21F2.03	0.7767	-2.632	0.110	16.96	36.87	12.49	5.878
SPAC10F6.14C	SPAC10F6.14c	0.6438	-2.625	0.191	27.5	56.69	9.149	1.997
SPCC11E10.06C	elp4	0.5017	-2.624	0.300	18.75	40.22	5.418	2.259
SPAC20G4.02C	fus1	0.7159	-2.621	0.145	24.56	51.16	12.19	1.257
SPBC27B12.08	sip1	0.5989	-2.619	0.223	20.07	42.7	7.536	2.384
SPBP23A10.05	ssr4	0.7344	-2.618	0.134	30.13	61.64	13.03	1.512
SPBPB8B6.04C	grt1	0.1466	-2.611	0.834	28.38	58.33	2.534	0.491
SPBC1718.07C	zfs1	0.8215	-2.596	0.085	17.24	37.33	15.83	7.088
SPBC342.06C	rtt109	0.6266	-2.593	0.203	28.16	57.88	8.505	1.994
SPAC186.09	SPAC186.09	0.5103	-2.591	0.292	24.11	50.26	6.233	1.392
SPAC9.12C	atp12	0.4731	-2.58	0.325	19.2	40.99	4.615	2.312
SPAC6C3.07	mug68	0.78	-2.565	0.108	32.72	66.41	15.69	1.278
SPAC22E12.04	ccs1	0.2529	-2.557	0.597	13.76	30.71	2.466	1.533
SPAC29A4.16	hal4	0.7085	-2.557	0.150	24.82	51.52	11.1	2.391
SPBC15D4.01C	klp9	0.3343	-2.556	0.476	31.12	63.39	3.838	1.251
SPBC409.17C	SPBC409.17c	0.6321	-2.555	0.199	26.03	53.79	8.891	1.156
SPAC5D6.04	SPAC5D6.04	0.5477	-2.553	0.261	24.54	50.99	6.582	1.793
SPAC22G7.04	ubp13	0.8155	-2.53	0.089	33.89	68.54	18.08	3.046
SPAC1071.03C	SPAC1071.03c	0.716	-2.528	0.145	27.92	57.31	11.46	2.081
SPBC1289.13C	SPBC1289.13c	0.7501	-2.527	0.125	18.25	39.1	10.09	5.353
SPBC13E7.08C	SPBC13E7.08c	0.6799	-2.52	0.168	25.22	52.21	9.753	2.358

SPBC11B10.06	sws1	0.1964	-2.517	0.707	31.32	63.68	2.708	0.8825
SPAC18B11.04	ncs1	0.1463	-2.51	0.835	26.25	54.14	1.547	1.223
SPBC365.10	arp5	0.3967	-2.481	0.402	3.448	11.16	4.489	1.12
SPAC869.11	cat1	0.4667	-2.466	0.331	16.05	34.85	4.977	1.687
SPAC664.07C	rad9	0.08204	-2.453	1.086	28.89	59	1.702	0.7371
SPBC8E4.04	SPBC8E4.04	0.6157	-2.441	0.211	25.71	52.98	7.871	1.607
SPAC6B12.12	tom70	0.5594	-2.437	0.252	18.58	39.56	5.047	2.892
SPBC23G7.14	SPBC23G7.14	0.6027	-2.432	0.220	34.48	69.47	7.561	1.522
SPBC26H8.01	thi2	0.4158	-2.419	0.381	22.43	46.77	3.965	1.787
SPBC23E6.05	arx1	0.572	-2.412	0.243	24.22	50.13	6.723	1.672
SPCC297.06C	SPCC297.06c	0.7884	-2.41	0.103	31.32	63.49	14.35	3.599
SPBC30D10.03C	SPBC30D10.03c	0.3646	-2.409	0.438	24.28	50.23	3.945	1.173
SPBC1703.12	ubp9	0.5886	-2.409	0.230	20.05	42.26	2.906	3.797
SPCC1450.07C	SPCC1450.07c	0.7209	-2.399	0.142	28.12	57.44	11.17	1.791
SPAC7D4.03C	SPAC7D4.03c	0.7914	-2.385	0.102	23.87	49.41	13.22	4.927
SPAC6F12.12	par2	0.2453	-2.377	0.610	29.08	59.21	2.827	1.028
SPAPB1A10.12C	alo1	0.7526	-2.362	0.123	25.59	52.61	10.92	4.135
SPBC13G1.08C	ash2	0.7085	-2.343	0.150	25.97	53.29	9.806	2.735
SPBP23A10.02	pkr1	0.3704	-2.342	0.431	27.9	56.93	3.69	1.38
SPAC25B8.07C	SPAC25B8.07c	0.5018	-2.34	0.299	21.44	44.75	3.211	2.748
SPAC1039.06	SPAC1039.06	0.5401	-2.332	0.268	24.38	50.28	5.816	1.707
SPAP27G11.08C	meu32	0.5385	-2.329	0.269	26.94	55.08	6.052	1.281
SPAC27F1.10	SPAC27F1.10	0.5654	-2.295	0.248	24.58	50.59	6.252	1.61
SPCC297.05	SPCC297.05	0.66	-2.289	0.180	24.6	50.61	8.291	2.011
SPBC215.05	gpd1	0.604	-2.274	0.219	26.79	54.7	7.015	1.583
SPBC119.04	mei3	0.6237	-2.268	0.205	22.21	46.07	7.261	1.907
SPAC4F8.08	mug114	0.7955	-2.263	0.099	32.38	65.21	14.66	2.166
SPAC1851.03	ckb1	0.6821	-2.261	0.166	20.94	43.67	4.433	4.601
SPBC119.06	sco1	0.5398	-2.26	0.268	3.679	11.18	5.077	2.179
SPCC188.08C	ubp5	0.1409	-2.259	0.851	30.45	61.57	1.161	1.117
SPBC29A10.11C	vps902	0.6079	-2.254	0.216	24.41	50.19	7.006	1.63
SPAC1B3.04C	SPAC1B3.04c	0.7369	-2.229	0.133	21.62	44.89	10.77	2.347
SPAC23A1.15C	sec20	0.5339	-2.214	0.273	32.84	65.98	5.614	1.337
SPAC24H6.09	gef1	0.7721	-2.201	0.112	27.74	56.36	12.54	2.366
SPAC26H5.10C	tif51	0.2713	-2.197	0.567	31.59	63.59	2.641	1.136
SPAC869.05C	SPAC869.05c	0.6258	-2.197	0.204	24.65	50.54	6.581	2.415
SPBC56F2.04	utp20	0.1503	-2.179	0.823	29.95	60.47	1.727	0.9508
SPCC16C4.07	scw1	0.275	-2.174	0.561	28.56	57.85	2.776	1.008
SPAC1D4.01	SPAC1D4.01	0.6707	-2.167	0.173	8.542	20.16	6.826	3.216
SPAC31G5.11	pac2	0.7956	-2.144	0.099	15.25	32.74	11.23	5.195
SPBP8B7.21	ubp3	0.7559	-2.141	0.122	26.2	53.34	11.52	1.752
SPBC13A2.04C	SPBC13A2.04c	0.8185	-2.14	0.087	18.68	39.18	11.12	6.68
SPAC1834.09	mug51	0.3124	-2.132	0.505	30.55	61.51	2.727	1.276
SPAC23C4.11	atp18	0.5804	-2.126	0.236	29.52	59.57	6.243	1.169
SPBC21B10.06C	inp2	0.5964	-2.123	0.224	16.24	34.57	5.67	2.287
SPBC83.09C	SPBC83.09c	0.6207	-2.117	0.207	25.79	52.53	6.5	2.035
SPCC576.14	dph5	0.6991	-2.116	0.155	26.91	54.64	8.928	1.812
SPBC1706.01	tea4	0.8058	-2.098	0.094	15.98	34.03	11.59	5.351
SPAPB8E5.04C	SPAPB8E5.04c	0.8572	-2.083	0.067	19.35	40.33	16.55	6.697
SPBC106.20	exo70	0.6986	-2.08	0.156	28.69	57.92	8.795	1.712
SPBC354.09C	SPBC354.09c	0.8286	-2.078	0.082	29.5	59.44	16.18	2.287
SPAC14C4.14	atp1	0.7026	-2.073	0.153	10.52	23.7	7.125	3.526
SPAC29B12.14C	SPAC29B12.14c	0.7136	-2.071	0.147	22.2	45.69	7.44	3.647
SPAC31G5.18C	sde2	0.3422	-2.056	0.466	20.89	43.18	1.438	1.756
SPBC1711.06	rpl401	0.7913	-2.056	0.102	29.51	59.42	11.95	3.71
SPAC25G10.01	SPAC25G10.01	0.2412	-2.051	0.618	28.62	57.73	2.165	1.077
SPAC13A11.05	SPAC13A11.05	0.4607	-2.046	0.337	33.8	67.47	4.295	1.088
SPBC14F5.11C	mug186	0.7449	-2.046	0.128	30.03	60.37	10.77	0.4559
SPBC1198.09	ubc16	0.5278	-2.044	0.278	24.25	49.49	5.095	1.227
SPAC6G10.02C	tea3	0.2243	-2.042	0.649	19.45	40.46	2.302	0.8439
SPAC4F10.16C	SPAC4F10.16c	0.7839	-2.041	0.106	25.6	52.03	12.52	1.738
SPAC23H3.13C	gpa2	0.7331	-2.031	0.135	10.85	24.25	8.202	3.636
SPBC1734.11	mas5	0.7143	-2.025	0.146	6.991	16.97	6.251	4.084

SPBC25B2.07C	mug164	0.29	-2.017	0.538	23.19	47.44	1.951	1.379
SPBC1347.12	arp1	0.6934	-2.001	0.159	27.34	55.22	8.491	1.169
SPAC23C4.16C	atg15	0.5677	-1.991	0.246	25.16	51.1	3.405	2.719
SPCC1827.08C	pof7	0.7548	-1.987	0.122	29.54	59.35	10.48	1.992
SPBC3H7.10	elp6	0.5912	-1.954	0.228	12.43	27.08	4.428	2.517
SPCC338.06C	SPCC338.06c	0.8032	-1.952	0.095	27.63	55.68	12.73	2.842
SPBC26H8.12	SPBC26H8.12	0.6943	-1.945	0.158	3.781	10.78	6.032	3.456
SPBC18H10.18C	SPBC18H10.18c	0.7656	-1.945	0.116	26.96	54.41	10.25	2.842
SPAC17H9.19C	cdt2	0.7969	-1.945	0.099	13.49	29.06	10.58	4.53
SPAC11D3.08C	SPAC11D3.08c	0.1481	-1.943	0.829	19.42	40.21	1.157	0.9607
SPAC22H10.02	SPAC22H10.02	0.2023	-1.936	0.694	22.95	46.83	2.144	0.645
SPCC1919.11	mug137	0.6162	-1.932	0.210	26.25	53.05	6.373	0.9511
SPBC1683.04	SPBC1683.04	0.5057	-1.927	0.296	27.89	56.12	4.106	1.606
SPCC306.05C	ins1	0.6589	-1.919	0.181	27.46	55.3	6.751	1.926
SPAC9E9.03	leu2	0.6585	-1.916	0.181	22.31	45.59	6.459	2.224
SPAC4G9.10	arg3	0.7122	-1.909	0.147	26.01	52.54	8.584	1.423
SPAPB18E9.04C	SPAPB18E9.04c	0.8187	-1.899	0.087	29.56	59.21	13.33	3.221
SPAP8A3.04C	hsp9	0.4114	-1.889	0.386	17.28	36.09	3.199	1.28
SPAC2C4.05	SPAC2C4.05	0.6664	-1.882	0.176	27.49	55.28	6.951	1.699
SPAC890.06	nup155	0.7521	-1.865	0.124	27.53	55.32	9.492	2.263
SPAC3A12.08	SPAC3A12.08	0.6289	-1.862	0.201	36.48	72.17	6.058	1.584
SPAC22G7.06C	ura1	0.6291	-1.857	0.201	22.9	46.59	5.19	2.386
SPCP1E11.06	apl4	0.8176	-1.853	0.087	13.74	29.35	11.33	4.754
SPAC6G9.15C	SPAC6G9.15c	0.7853	-1.848	0.105	26.1	52.6	11.16	2.205
SPBC32H8.11	mei4	0.8381	-1.844	0.077	31.37	62.51	15.43	1.442
SPAC1782.07	qcr8	0.7558	-1.843	0.122	20.33	41.73	8.119	3.654
SPBC215.07C	SPBC215.07c	0.8109	-1.827	0.091	26.39	53.11	12.26	3.01
SPAC637.13C	SPAC637.13c	0.8379	-1.827	0.077	32.19	64.03	14.89	2.582
SPBC577.13	syj2	0.7983	-1.812	0.098	25.81	51.99	11.63	2.386
SPBC1347.11	sro1	0.5911	-1.793	0.228	35.68	70.53	5.474	0.905
SPBC4.01	dni2	0.8352	-1.792	0.078	29.3	58.52	13.95	3.222
SPBC18H10.19	atg14	0.2736	-1.789	0.563	20.75	42.42	2.254	0.8499
SPAC1782.04	cox24	0.8249	-1.789	0.084	15.34	32.23	10.74	5.225
SPBC646.08C	SPBC646.08c	0.7074	-1.788	0.150	24.62	49.69	7.23	2.336
SPAC12B10.01C	SPAC12B10.01c	0.6044	-1.786	0.219	27.05	54.28	5.195	1.681
SPAC694.06C	mrc1	0.8058	-1.78	0.094	25.96	52.2	11.63	2.842
SPAC17A5.18C	rec25	0.4833	-1.776	0.316	23.95	48.43	3.75	1.243
SPAC8C9.08	rps5	0.8301	-1.776	0.081	28.08	56.18	13.37	3.138
SPBC32F12.03C	gpx1	0.3816	-1.775	0.418	27.59	55.28	2.896	1.045
SPAC19D5.01	pyp2	0.698	-1.762	0.156	25.11	50.57	6.984	2.117
SPCC306.08C	SPCC306.08c	0.8504	-1.757	0.070	30.68	61.05	15.55	2.654
SPAC15E1.06	vps29	0.7963	-1.755	0.099	26.55	53.27	11.31	1.942
SPCC553.01C	SPCC553.01c	0.5242	-1.727	0.281	25.66	51.54	3.79	1.557
SPBC3D6.04C	mad1	0.8038	-1.722	0.095	16.08	33.51	11.43	2.213
SPAC1782.01	ecm29	0.8266	-1.721	0.083	31.07	61.72	12.98	2.504
SPCPB16A4.04C	trm8	0.4656	-1.708	0.332	19.97	40.81	3.667	0.849
SPAC6G9.10C	sen1	0.7712	-1.707	0.113	22.77	46.06	8.65	3.165
SPCC613.03	SPCC613.03	0.7796	-1.697	0.108	23.95	48.27	8.566	3.569
SPAC2C4.14C	ppk11	0.6851	-1.696	0.164	27.5	54.96	6.955	1.067
SPCC794.07	lat1	0.7406	-1.696	0.130	5.539	13.62	6.138	3.643
SPAC3H5.04	aar2	0.7223	-1.693	0.141	30.24	60.1	7.534	1.979
SPAC22G7.07C	SPAC22G7.07c	0.8647	-1.684	0.063	17.91	36.88	13.48	6.185
SPCC1235.11	SPCC1235.11	0.8177	-1.676	0.087	13.65	28.86	9.699	4.672
SPAC6G10.03C	SPAC6G10.03c	0.7989	-1.675	0.098	28.37	56.56	10.93	1.912
SPAC630.14C	tup12	0.7722	-1.673	0.112	13.32	28.23	6.093	4.447
SPCC550.07	SPCC550.07	0.7459	-1.668	0.127	17.88	36.8	6.846	3.304
SPAC4H3.07C	SPAC4H3.07c	0.8198	-1.664	0.086	20.34	41.42	10.79	3.937
SPCC1442.02	SPCC1442.02	0.7289	-1.663	0.137	23.29	46.96	6.75	2.827
SPAC13F5.03C	gld1	0.878	-1.652	0.057	18.37	37.69	13.72	7.294
SPBPB2B2.13	gal1	0.8662	-1.649	0.062	15.13	31.59	11.47	7.113
SPAC18G6.15	mal3	0.8199	-1.639	0.086	21.64	43.81	11.04	3.479
SPBP8B7.09C	los1	0.8578	-1.638	0.067	15.02	31.35	12.39	5.769
SPBC4C3.04C	SPBC4C3.04c	0.8062	-1.637	0.094	28.64	56.99	11.04	2.061

SPAC22H12.04C	rps102	0.616	-1.635	0.210	28.15	56.06	3.978	2.256
SPAC8C9.07	SPAC8C9.07	0.6009	-1.619	0.221	15.88	32.94	4.942	1.131
SPAPB1E7.02C	mcl1	0.8301	-1.618	0.081	11.27	24.25	10.01	4.874
SPAC5D6.12	SPAC5D6.12	0.8381	-1.612	0.077	30.89	61.18	13.05	2.499
SPCC1450.08C	wtf16	0.8215	-1.611	0.085	24.38	48.92	10.64	3.764
SPAC1F3.03	SPAC1F3.03	0.7188	-1.609	0.143	18.76	38.33	2.628	3.901
SPBC31F10.09C	nut2	0.7807	-1.593	0.108	10.32	22.42	7.867	3.518
SPCC794.15	SPCC794.15	0.3262	-1.59	0.487	30.87	61.1	2.254	0.8594
SPBC1347.09	SPBC1347.09	0.7673	-1.59	0.115	25.66	51.28	9.009	1.325
SPAC30D11.02C	SPAC30D11.02c	0.8465	-1.588	0.072	28.84	57.27	12.32	4.246
SPCC1259.01C	rps1802	0.7668	-1.587	0.115	6.958	16.08	7.58	3.129
SPBC354.08C	SPBC354.08c	0.7711	-1.58	0.113	25.38	50.75	5.984	4.085
SPAC1071.02	mms19	0.4719	-1.575	0.326	19.76	40.16	3.201	1.108
SPBC6B1.04	mde4	0.5108	-1.572	0.292	12.27	26.04	3.385	1.332
SPAC1527.01	mok11	0.5356	-1.563	0.271	30.67	60.66	3.813	1.18
SPAC1851.04C	ric1	0.7055	-1.561	0.152	7.662	17.36	5.961	2.51
SPAC19B12.07C	SPAC19B12.07c	0.6799	-1.557	0.168	25.46	50.85	6.133	1.277
SPCC285.09C	cgs2	0.6671	-1.551	0.176	21.49	43.37	4.753	2.319
SPAC56F8.04C	ppt1	0.7947	-1.537	0.100	10.93	23.46	8.078	3.665
SPBC83.01	ucp8	0.7889	-1.536	0.103	27.89	55.38	9.723	1.121
SPAC57A7.04C	pabp	0.6694	-1.529	0.174	25.8	51.44	4.982	2.132
SPBC530.15C	SPBC530.15c	0.7654	-1.525	0.116	25.26	50.41	6.124	3.64
SPAC9E9.09C	SPAC9E9.09c	0.8317	-1.522	0.080	12.31	26.03	9.735	4.489
SPBC27.04	uds1	0.7672	-1.52	0.115	27.6	54.8	8.639	1.194
SPAC3H1.13	ppk13	0.7547	-1.517	0.122	24.8	49.53	7.689	2.027
SPCC11E10.07C	SPCC11E10.07c	0.568	-1.515	0.246	13.86	28.94	4.385	0.5654
SPCC736.13	SPCC736.13	0.7157	-1.515	0.145	33.71	66.31	6.777	1.411
SPAC2F7.10	akr1	0.8307	-1.508	0.081	27.92	55.4	11.41	2.654
SPBC16H5.13	SPBC16H5.13	0.7733	-1.499	0.112	9.16	20.06	7.176	3.184
SPAC6B12.16	meu26	0.8196	-1.489	0.086	26.95	53.53	10.72	2.182
SPBC6B1.02	ppk30	0.5108	-1.469	0.292	34.77	68.21	3.378	1.03
SPAC1A6.04C	plb1	0.8746	-1.445	0.058	11.8	24.93	10.01	6.947
SPBC418.02	SPBC418.02	0.7783	-1.443	0.109	27.28	54.06	8.14	2.126
SPCC126.13C	SPCC126.13c	0.7384	-1.441	0.132	27.26	54.02	6.84	1.783
SPAC9.05	fml1	0.845	-1.437	0.073	26.33	52.25	11.68	3.082
SPBC21B10.04C	nrf1	0.4364	-1.428	0.360	31.55	62.08	1.63	1.447
SPAC4G9.14	SPAC4G9.14	0.8558	-1.427	0.068	29.96	59.08	11.89	3.965
SPAC17A2.09C	csx1	0.7694	-1.411	0.114	24.48	48.73	7.955	1.468
SPAC23D3.10C	eng2	0.8586	-1.408	0.066	26.34	52.22	11.73	4.22
SPAC1039.04	SPAC1039.04	0.8499	-1.407	0.071	31.46	61.85	9.589	4.978
SPBC342.01C	alg6	0.8445	-1.401	0.073	11.84	24.93	9.647	4.527
SPAC19G12.15C	tpp1	0.7912	-1.382	0.102	5.892	13.69	6.82	3.435
SPBC29A10.12	SPBC29A10.12	0.84	-1.378	0.076	27.68	54.68	10.64	3.112
SPAC1782.06C	phb1	0.4034	-1.375	0.394	20.64	41.44	2.571	0.5378
SPAC8F11.08C	SPAC8F11.08c	0.6978	-1.368	0.156	24.33	48.37	5.248	1.824
SPAC22F3.02	atf31	0.6499	-1.362	0.187	26.48	52.4	4.105	1.831
SPCC1322.06	kap113	0.6293	-1.346	0.201	19.53	39.29	3.446	1.911
SPAC4C5.03	SPAC4C5.03	0.7155	-1.346	0.145	26.64	52.67	5.354	2.047
SPAPB8E5.02C	rpn502	0.6157	-1.345	0.211	24.66	48.95	3.51	1.727
SPAC343.12	rds1	0.8045	-1.342	0.094	29.1	57.3	9.011	1.586
SPBC1711.14	rec15	0.9109	-1.342	0.041	23.33	46.44	15.69	7.924
SPAC2F7.07C	cph2	0.8009	-1.334	0.096	9.161	19.75	7.412	3.159
SPBC1271.10C	SPBC1271.10c	0.2734	-1.333	0.563	30.17	59.29	1.358	0.8349
SPAPJ691.02	SPAPJ691.02	0.8472	-1.332	0.072	21.36	42.7	10.25	3.698
SPBC36.10	SPBC36.10	0.8639	-1.329	0.064	20.73	41.51	9.814	5.288
SPAC3A11.04	SPAC3A11.04	0.901	-1.326	0.045	20.32	40.74	14.25	6.848
SPAC22E12.18	SPAC22E12.18	0.6087	-1.316	0.216	2.77	7.69	3.434	1.613
SPBC17A3.05C	SPBC17A3.05c	0.6621	-1.314	0.179	16.72	33.95	4.558	1.461
SPBC354.05C	sre2	0.6997	-1.292	0.155	4.541	10.98	4.52	2.234
SPBC1604.09C	SPBC1604.09c	0.7676	-1.278	0.115	26.66	52.58	6.475	2.234
SPAC1B3.02C	SPAC1B3.02c	0.8008	-1.273	0.096	29.24	57.43	8.31	1.625
SPBC16G5.16	SPBC16G5.16	0.8919	-1.272	0.050	34.83	67.96	16.15	0.8775
SPAC4F10.04	SPAC4F10.04	0.8059	-1.265	0.094	14.4	29.48	7.146	3.128

SPAC13G7.12C	SPAC13G7.12c	0.8458	-1.251	0.073	13.13	27.07	8.1	4.403
SPAC23C11.15	pst2	0.5509	-1.247	0.259	25.78	50.86	3.297	0.7975
SPBC3H7.12	rav2	0.4135	-1.244	0.384	0.5336	3.345	2.366	0.5336
SPBC530.06C	SPBC530.06c	0.8494	-1.243	0.071	12.09	25.1	8.386	4.411
SPCC306.02C	SPCC306.02c	0.8124	-1.235	0.090	20.24	40.41	7.303	3.101
SPAC8C9.06C	ppr4	0.8484	-1.235	0.071	29.29	57.46	10.76	1.884
SPCC1840.05C	SPCC1840.05c	0.8608	-1.235	0.065	29.19	57.27	11.86	1.777
SPCC18.13	SPCC18.13	0.4778	-1.232	0.321	18.9	37.9	2.078	1.194
SPBC530.05	SPBC530.05	0.8977	-1.229	0.047	23.43	46.41	14.25	5.063
SPCPJ732.03	meu15	0.7876	-1.228	0.104	7.955	17.28	5.512	3.223
SPAC1639.02C	trk2	0.785	-1.224	0.105	4.357	10.5	6.283	2.865
SPAC13G7.13C	msa1	0.9015	-1.222	0.045	19.03	38.12	13.24	6.326
SPAC6G9.13C	bqt1	0.8007	-1.214	0.097	28.41	55.76	8.096	1.14
SPBC1105.14	rsv2	0.7728	-1.212	0.112	25.89	51	4.389	3.241
SPAC19A8.10	rfp1	0.8057	-1.21	0.094	27.99	54.97	7.552	2.37
SPAPB8E5.08	SPAPB8E5.08	0.8596	-1.208	0.066	29.96	58.67	11.43	1.884
SPBC6B1.10	prp17	0.8457	-1.197	0.073	26.22	51.61	9.444	2.976
SPBC115.03	SPBC115.03	0.9083	-1.178	0.042	19.91	39.68	12.49	7.213
SPBC1215.01	shy1	0.8334	-1.164	0.079	21.41	42.48	8.013	3.118
SPBC800.05C	atb2	0.7793	-1.161	0.108	20.54	40.85	3.776	3.371
SPBC32F12.05C	cwf12	0.8545	-1.155	0.068	11.59	23.99	8.616	3.92
SPCC16A11.10C	oca8	0.8581	-1.146	0.066	24.15	47.61	8.319	4.263
SPAC23H4.08	iwr1	0.634	-1.145	0.198	21.55	42.72	2.433	1.866
SPCC757.02C	SPCC757.02c	0.8381	-1.141	0.077	25.41	49.97	8.674	2.584
SPCC16C4.14C	sfc4	0.7704	-1.139	0.113	26.6	52.2	6.052	1.798
SPAC8F11.10C	pvg1	0.8191	-1.138	0.087	9.395	19.82	6.252	3.412
SPBP35G2.10	mit1	0.8564	-1.126	0.067	28.39	55.55	9.878	2.631
SPBC21D10.08C	SPBC21D10.08c	0.8181	-1.124	0.087	25.71	50.5	7.347	2.514
SPAC1F7.01C	spt6	0.6183	-1.122	0.209	23.6	46.54	3.716	0.5792
SPCC736.04C	gma12	0.8673	-1.121	0.062	29.85	58.3	10.48	3.062
SPAC18B11.09C	SPAC18B11.09c	0.3921	-1.12	0.407	31.14	60.71	1.651	0.84
SPBC1711.03	aim27	0.8684	-1.118	0.061	29.41	57.46	11.24	1.995
SPCC553.08C	SPCC553.08c	0.912	-1.115	0.040	20.77	41.19	11.69	7.408
SPBC13A2.02	nup82	0.5513	-1.114	0.259	29.97	58.51	2.14	1.347
SPBP23A10.16	sdh4	0.828	-1.111	0.082	40.06	77.49	8.728	0.8454
SPCC1494.07	SPCC1494.07	0.7956	-1.108	0.099	26.43	51.83	7.062	1.34
SPAC1687.23C	SPAC1687.23c	0.7241	-1.103	0.140	26.22	51.42	5.221	0.78
SPAPB1E7.08C	SPAPB1E7.08c	0.7517	-1.102	0.124	25.38	49.85	5.328	1.677
SPCC550.08	SPCC550.08	0.742	-1.095	0.130	24.44	48.05	3.897	2.403
SPBP8B7.06	rpp201	0.8636	-1.077	0.064	27.68	54.13	9.735	2.909
SPAC1782.02C	SPAC1782.02c	0.8405	-1.071	0.075	27.63	54.02	7.505	3.152
SPCC550.14	vgl1	0.7599	-1.065	0.119	18.41	36.65	4.755	2.162
SPBC3D6.05	ptp4	0.8453	-1.064	0.073	18.59	37	7.955	3.024
SPBC1685.09	rps29	0.4005	-1.063	0.397	15.98	32.08	1.878	0.5783
SPAC25G10.06	rps2801	0.543	-1.063	0.265	31.51	61.32	1.368	1.443
SPAC25G10.03	zip1	0.665	-1.056	0.177	12.59	25.68	2.467	1.897
SPAC26F1.12C	SPAC26F1.12c	0.7673	-1.051	0.115	27.4	53.55	5.814	1.19
SPCC1020.08	SPCC1020.08	0.7946	-1.047	0.100	27.09	52.95	6.754	0.998
SPAC18B11.08C	SPAC18B11.08c	0.8668	-1.035	0.062	29.08	56.68	10.19	1.989
SPAC25B8.04C	mss51	0.6649	-1.034	0.177	24.97	48.95	3.429	1.337
SPCC1259.14C	meu27	0.7487	-1.032	0.126	24.51	48.08	5.418	0.7183
SPBC1D7.01	SPBC1D7.01	0.8042	-1.028	0.095	36.13	69.93	6.888	1.214
SPBC17A3.03C	SPBC17A3.03c	0.7388	-1.027	0.131	34.12	66.15	4.778	1.404
SPCC18B5.09C	SPCC18B5.09c	0.7336	-1.024	0.135	25.58	50.08	3.987	1.935
SPAC17A5.02C	dbr1	0.8215	-1.022	0.085	9.144	19.13	5.789	3.055
SPAC513.06C	SPAC513.06c	0.8714	-1.021	0.060	28.81	56.14	10.67	1.454
SPCC1442.14C	SPCC1442.14c	0.809	-1.014	0.092	27.03	52.79	6.954	1.275
SPBP26C9.02C	car1	0.8881	-0.9882	0.052	29.43	57.25	11.41	2.611
SPBC26H8.13C	SPBC26H8.13c	0.7088	-0.9838	0.149	24.23	47.45	3.216	1.84
SPAC24B11.07C	SPAC24B11.07c	0.8026	-0.9802	0.096	29.63	57.62	5.579	2.281
SPBC1773.06C	SPBC1773.06c	0.7375	-0.9796	0.132	24.51	47.97	3.633	2.014
SPCC320.04C	SPCC320.04c	0.8432	-0.976	0.074	28.82	56.08	8.087	1.699
SPCC1919.01	ppk34	0.8105	-0.9755	0.091	26.35	51.44	4.947	2.871

SPAP27G11.06C	vas2	0.8744	-0.9734	0.058	11.68	23.81	8.328	3.897
SPAP8A3.12C	tpp2	0.684	-0.9702	0.165	34.95	67.61	2.904	1.664
SPCC320.06	SPCC320.06	0.8424	-0.9651	0.074	27.32	53.24	5.264	3.696
SPAC3H1.06C	SPAC3H1.06c	0.8632	-0.9645	0.064	26.43	51.56	7.887	3.331
SPBC17F3.01C	rga5	0.8467	-0.9631	0.072	25.82	50.4	7.245	2.784
SPCC16C4.17	mug123	0.9094	-0.9611	0.041	17.66	35.05	10.58	5.829
SPAPJ695.01C	SPAPJ695.01c	0.9215	-0.9497	0.036	17.67	35.05	12.08	6.655
SPAC3F10.02C	trk1	0.8848	-0.9425	0.053	20.52	40.39	8.476	4.315
SPAC821.07C	moc3	0.9239	-0.9369	0.034	27.43	53.39	15.8	3.867
SPBC577.12	mug71	0.8198	-0.9271	0.086	24.53	47.91	2.72	3.525
SPBC354.10	def1	0.93	-0.9229	0.032	16.92	33.59	12.58	7.538
SPBC21C3.11	ubx4	0.8256	-0.9207	0.083	27.7	53.87	6.334	2.101
SPBC25H2.09	SPBC25H2.09	0.8906	-0.917	0.050	29.14	56.58	10.7	2.676
SPAC4F10.07C	atg13	0.8362	-0.9166	0.078	21.93	42.99	6.858	2.083
SPBC19G7.09	ulp1	0.8099	-0.9057	0.092	26.93	52.4	6.488	0.1909
SPBC28E12.04	SPBC28E12.04	0.7708	-0.9037	0.113	22.99	44.98	3.856	2.142
SPBC1921.05	ape2	0.7721	-0.8991	0.112	25.64	49.96	3.097	2.447
SPCC1442.13C	SPCC1442.13c	0.878	-0.8955	0.057	11.25	22.87	7.654	3.839
SPBC21C3.09C	SPBC21C3.09c	0.787	-0.8951	0.104	25.89	50.41	5.589	0.742
SPBC646.13	sds23	0.768	-0.8938	0.115	19.88	39.09	1.864	2.643
SPAC1B3.07C	vps28	0.7283	-0.891	0.138	13.5	27.09	3.855	1.301
SPBC13G1.03C	pex14	0.8769	-0.8909	0.057	18.64	36.76	5.994	4.469
SPBC14F5.03C	kap123	0.5392	-0.8897	0.268	18.89	37.23	0.6897	1.258
SPAC9E9.15	SPAC9E9.15	0.9051	-0.8891	0.043	29.98	58.11	12.5	2.039
SPCC613.02	SPCC613.02	0.8646	-0.8863	0.063	28.74	55.77	8.579	1.692
SPAC458.02C	SPAC458.02c	0.7801	-0.8854	0.108	27.29	53.04	5.102	1.22
SPBC1685.04	SPBC1685.04	0.8268	-0.8814	0.083	28.35	55.01	6.527	1.504
SPCC1322.10	SPCC1322.10	0.8741	-0.8731	0.058	10.31	21.05	7.5	3.457
SPAC2F7.04	pmc2	0.6245	-0.8712	0.204	2.075	5.546	2.157	1.309
SPCC1919.15	brl1	0.8815	-0.8669	0.055	10.3	21.02	7.935	3.638
SPBC2G2.08	ade9	0.6168	-0.8661	0.210	25.38	49.41	1.694	1.357
SPAC323.04	SPAC323.04	0.8265	-0.8416	0.083	6.645	14.09	4.836	2.756
SPAC11E3.05	SPAC11E3.05	0.7112	-0.841	0.148	15.98	31.67	2.364	1.751
SPAC2E1P3.05C	SPAC2E1P3.05c	0.7523	-0.8371	0.124	25.82	50.17	3.645	1.627
SPAC25B8.01	dap1	0.9035	-0.8325	0.044	26.6	51.64	10.77	3.09
SPBC1539.08	arf6	0.9204	-0.8303	0.036	10.4	21.13	10.47	5.696
SPBC19G7.01C	msh2	0.8237	-0.8265	0.084	17.52	34.53	5.591	1.894
SPCC16C4.06C	SPCC16C4.06c	0.7867	-0.8256	0.104	24.62	47.89	4.913	1.171
SPCC1682.08C	SPCC1682.08c	0.9331	-0.8114	0.030	18.2	35.78	12.51	6.454
SPAC16.01	rho2	0.9373	-0.8104	0.028	21.35	41.7	13.35	6.872
SPBC32C12.03C	ppk25	0.858	-0.8091	0.067	23.48	45.72	6.803	2.33
SPCC737.04	SPCC737.04	0.8747	-0.8042	0.058	24.34	47.32	7.177	3.041
SPBC17A3.02	SPBC17A3.02	0.8519	-0.8018	0.070	29.08	56.25	6.884	1.728
SPBC2D10.18	abc1	0.1426	-0.7932	0.846	0.2777	2.015	0.7957	0.2777
SPAC10F6.13C	SPAC10F6.13c	0.8264	-0.7912	0.083	12.35	24.74	5.561	1.715
SPCC1223.01	SPCC1223.01	0.8993	-0.7859	0.046	30.23	58.38	10.31	1.913
SPAC1002.02	pom34	0.8491	-0.7842	0.071	23.54	45.78	5.964	2.33
SPCC1442.04C	SPCC1442.04c	0.9247	-0.7842	0.034	17.07	33.61	12.32	4.447
SPBC1734.05C	spf31	0.8037	-0.7815	0.095	30.76	59.37	5.043	1.242
SPAC9G1.08C	SPAC9G1.08c	0.938	-0.7785	0.028	21.56	42.04	11.8	7.254
SPAC29B12.05C	SPAC29B12.05c	0.7999	-0.7747	0.097	9.053	18.5	4.029	2.099
SPAC11D3.02C	SPAC11D3.02c	0.8605	-0.7552	0.065	26.29	50.91	7.048	1.478
SPAC23A1.03	apt1	0.9307	-0.7495	0.031	32.29	62.18	13.8	3.53
SPAC16E8.17C	SPAC16E8.17c	0.9084	-0.7489	0.042	22.02	42.86	9.897	3.261
SPBC428.10	SPBC428.10	0.7952	-0.7481	0.100	28.26	54.6	4.565	1.217
SPBC2D10.03C	SPBC2D10.03c	0.8673	-0.7432	0.062	28.75	55.51	7.282	1.559
SPAC31G5.12C	maf1	0.9359	-0.7424	0.029	18.89	36.95	12.23	6.009
SPBC725.06C	ppk31	0.9201	-0.7383	0.036	28.72	55.45	11	3.873
SPAC31G5.07	dni1	0.9259	-0.7321	0.033	30	57.85	12.54	3.319
SPBCPT2R1.01C	SPBCPT2R1.01c	0.8359	-0.7311	0.078	22.66	44.02	4.804	2.211
SPCC1620.08	SPCC1620.08	0.9041	-0.7264	0.044	29.11	56.15	9.933	2.001
SPCC1393.03	rps1501	0.8366	-0.7231	0.077	27.73	53.56	5.736	1.222
SPCPJ732.02C	SPCPJ732.02c	0.858	-0.72	0.067	25.94	50.18	5.409	2.566

SPCC23B6.01C	SPCC23B6.01c	0.5504	-0.7147	0.259	13.59	26.92	1.439	0.8313
SPCC1223.15C	spc19	0.7103	-0.7124	0.149	22.75	44.16	3.028	0.8012
SPBC16H5.05C	cyp7	0.8197	-0.7119	0.086	24.02	46.54	4.273	1.94
SPAPB24D3.02C	SPAPB24D3.02c	0.9328	-0.7119	0.030	31.4	60.44	13.35	3.675
SPBC4C3.12	Sep-01	0.9422	-0.7116	0.026	15.27	30.09	11.7	7.055
SPBC354.15	fap1	0.907	-0.7093	0.042	30.98	59.64	10.16	1.709
SPAC8C9.04	SPAC8C9.04	0.9387	-0.7086	0.027	17.63	34.52	11.08	6.579
SPCC24B10.11C	mft1	0.7215	-0.7054	0.142	21.01	40.88	2.442	1.371
SPAC15F9.01C	SPAC15F9.01c	0.86	-0.703	0.066	25.4	49.13	6.478	1.469
SPAC19G12.13C	poz1	0.9084	-0.7012	0.042	28.92	55.74	9.563	2.729
SPAC11H11.04	mam2	0.8943	-0.7007	0.049	25.39	49.12	8.845	1.425
SPBC428.11	SPBC428.11	0.9465	-0.6956	0.024	20.06	39.06	13.45	6.904
SPAC1399.02	SPAC1399.02	0.7785	-0.6906	0.109	31.79	61.14	3.909	1.003
SPAC3H1.04C	mdm31	0.8085	-0.6906	0.092	21.32	41.43	2.894	2.233
SPBC21C3.08C	car2	0.391	-0.6882	0.408	0	1.295	1.295	0
SPAC1399.01C	SPAC1399.01c	0.8908	-0.6822	0.050	28.71	55.33	8.206	1.618
SPBC16A3.07C	nrm1	0.9178	-0.6822	0.037	11.59	23.09	8.799	4.284
SPAC27D7.13C	ssm4	0.6724	-0.6794	0.172	30.46	58.6	2.17	1.003
SPAC4G9.02	rnh201	0.8912	-0.6793	0.050	28.62	55.14	8.078	1.83
SPBC2F12.12C	SPBC2F12.12c	0.9066	-0.677	0.043	10.93	21.84	7.32	3.937
SPAC24B11.05	SPAC24B11.05	0.9366	-0.6752	0.028	25.6	49.46	13.04	4.122
SPAP27G11.12	SPAP27G11.12	0.867	-0.6748	0.062	26.23	50.64	5.047	2.779
SPAC27E2.11C	SPAC27E2.11c	0.2555	-0.6729	0.593	0.2058	1.654	0.8782	0.2058
SPBC725.02	mpr1	0.9344	-0.6634	0.029	15.02	29.53	10.57	5.312
SPAC1039.02	SPAC1039.02	0.8505	-0.6624	0.070	25.94	50.08	5.107	1.969
SPBC17G9.05	rct1	0.9317	-0.6582	0.031	30.77	59.16	12.11	3.381
SPBCPT2R1.02	SPBCPT2R1.02	0.9247	-0.6577	0.034	30.31	58.28	11.34	2.549
SPAC644.11C	pkp1	0.7786	-0.6555	0.109	21.31	41.34	1.62	2.006
SPCC895.09C	ucp12	0.9293	-0.6543	0.032	23.79	46	7.03	5.941
SPCC63.02C	aah3	0.391	-0.6539	0.408	0	1.231	1.231	0
SPAC21E11.03C	pcr1	0.9258	-0.6396	0.033	28.91	55.62	11.4	2.135
SPAC3A11.10C	SPAC3A11.10c	0.9304	-0.6386	0.031	33.64	64.51	12.38	1.71
SPAC664.01C	swi6	0.9339	-0.635	0.030	26.81	51.66	11.62	3.861
SPBC25D12.06	SPBC25D12.06	0.3052	-0.6348	0.515	0.01643	1.226	1.08	0.01643
SPBC1271.07C	SPBC1271.07c	0.9187	-0.6305	0.037	31.1	59.72	10.34	1.716
SPBC9B6.03	SPBC9B6.03	0.8547	-0.6287	0.068	26.07	50.25	2.819	2.876
SPAPB18E9.01	trm5	0.8767	-0.6269	0.057	28.21	54.28	5.145	2.742
SPBC1711.09C	SPBC1711.09c	0.9368	-0.6255	0.028	27.97	53.82	12.24	3.696
SPBC9B6.07	nop52	0.9236	-0.6254	0.035	7.633	15.54	7.731	4.707
SPBC1773.12	SPBC1773.12	0.7878	-0.6248	0.104	13.27	26.14	3.42	1.253
SPAC8C9.09C	mug129	0.9514	-0.6223	0.022	13.11	25.85	13.65	6.572
SPBC21C3.20C	git1	0.8053	-0.6152	0.094	17.23	33.59	3.801	1.231
SPAC212.01C	SPAC212.01c	0.8574	-0.6124	0.067	26.79	51.58	5.35	1.518
SPAC926.05C	dph4	0.7216	-0.6104	0.142	26.22	50.49	2.725	0.6902
SPCC24B10.14C	xlf1	0.9234	-0.6103	0.035	30.43	58.42	10.39	2.246
SPAC7D4.04	taf1	0.8338	-0.6046	0.079	24.5	47.25	4.392	1.425
SPACUNK4.09	SPACUNK4.09	0.9205	-0.6016	0.036	29.71	57.05	10.06	1.773
SPBC146.13C	myo1	0.9358	-0.6004	0.029	14.09	27.65	9.867	4.85
SPCC285.16C	msh6	0.7629	-0.599	0.118	27.43	52.76	3.175	0.788
SPBC27B12.05	SPBC27B12.05	0.937	-0.5902	0.028	32.43	62.16	12.66	1.692
SPAP11E10.01	SPAP11E10.01	0.9221	-0.5883	0.035	28.46	54.67	9.894	2.051
SPBC3E7.09	SPBC3E7.09	0.9111	-0.5875	0.040	27.99	53.78	8.493	2.046
SPAC26A3.07C	rpl1101	0.8693	-0.5865	0.061	21.6	41.76	5.469	1.733
SPAP32A8.02	SPAP32A8.02	0.8045	-0.5784	0.094	34	65.08	3.314	1.367
SPAC24B11.10C	chr3	0.8673	-0.5758	0.062	27.21	52.29	5.588	1.291
SPCC18B5.06	SPCC18B5.06	0.8156	-0.5594	0.089	22.6	43.58	3.359	1.436
SPBC24C6.11	cwf14	0.9297	-0.5568	0.032	12.19	24	8.423	4.073
SPACUNK4.11C	SPACUNK4.11c	0.8775	-0.5548	0.057	26.4	50.73	4.947	2.226
SPCC613.10	qcr2	0.9203	-0.5548	0.036	11.38	22.47	6.761	3.918
SPAC20G8.08C	fft1	0.9398	-0.5464	0.027	13.54	26.51	8.885	5.083
SPAC25B8.05	SPAC25B8.05	0.7503	-0.5455	0.125	21.05	40.64	2.026	1.231
SPCC338.08	ctp1	0.9414	-0.5418	0.026	8.926	17.82	9.089	5.16
SPBC32F12.11	tdh1	0.7751	-0.5377	0.111	17.05	33.11	3.145	0.4995

SPBC2D10.15C	pth1	0.949	-0.5312	0.023	33.96	64.92	13.28	3.411
SPAP8A3.13C	SPAP8A3.13c	0.8815	-0.5272	0.055	38.04	72.59	5.625	1.484
SPAC12G12.01C	SPAC12G12.01c	0.8797	-0.5237	0.056	26.14	50.18	5.101	1.882
SPCC1795.03	gms1	0.903	-0.5236	0.044	25.95	49.82	6.728	1.94
SPBC16H5.03C	fub2	0.8041	-0.5231	0.095	34.13	65.23	2.988	1.236
SPAC3C7.12	tip1	0.9345	-0.5225	0.029	21.41	41.29	9.158	3.645
SPBC1604.02C	ppr1	0.8901	-0.5223	0.051	4.996	10.39	4.872	2.658
SPBC336.14C	ppk26	0.6408	-0.521	0.193	23.18	44.61	0.9714	0.9103
SPCC970.10C	brl2	0.9425	-0.5209	0.026	13.75	26.87	9.325	4.841
SPBC660.06	SPBC660.06	0.9302	-0.5202	0.031	23.64	45.48	8.645	3.336
SPBC24C6.08C	bhd1	0.9011	-0.5193	0.045	28.38	54.4	6.512	1.925
SPBC21B10.03C	SPBC21B10.03c	0.9085	-0.5168	0.042	16.41	31.86	4.851	3.439
SPAC2C4.08	SPAC2C4.08	0.8126	-0.5133	0.090	33.21	63.48	2.913	1.375
SPCC11E10.05C	ynd1	0.8783	-0.5097	0.056	26.4	50.65	4.832	1.874
SPAC29E6.05C	SPAC29E6.05c	0.9391	-0.5053	0.027	30.71	58.76	10.66	2.601
SPAC23C11.07	SPAC23C11.07	0.9302	-0.5052	0.031	29.06	55.65	9.177	2.434
SPBC2G2.17C	SPBC2G2.17c	0.9152	-0.5031	0.038	27.24	52.21	7.243	2.31
SPAC110.02	pds5	0.9444	-0.4992	0.025	12.9	25.22	9.07	4.885
SPCC736.11	ago1	0.391	-0.4991	0.408	0	0.9393	0.9393	0
SPAC2E1P3.01	SPAC2E1P3.01	0.8902	-0.4978	0.051	22.96	44.15	5.925	1.223
SPCC737.07C	SPCC737.07c	0.856	-0.4943	0.068	35.46	67.67	4.315	1.162
SPBC18E5.05C	iki1	0.8317	-0.4832	0.080	2.264	5.17	3.007	1.559
SPBC20F10.06	mad2	0.9359	-0.4784	0.029	30.47	58.24	9.794	2.004
SPBC19F8.01C	spn7	0.9553	-0.4778	0.020	18.88	36.43	9.819	6.274
SPAC57A10.02	cdr2	0.941	-0.4767	0.026	12.13	23.73	7.888	4.529
SPAC27F1.06C	SPAC27F1.06c	0.8901	-0.4694	0.051	28.79	55.07	4.103	2.417
SPAC23H4.17C	srb10	0.8169	-0.4682	0.088	2.014	4.672	2.893	1.165
SPBC1773.05C	tms1	0.9409	-0.4653	0.026	28.7	54.9	7.742	4.393
SPCC622.08C	hta1	0.7487	-0.4614	0.126	30.57	58.41	1.567	1.089
SPBC215.14C	vps20	0.9036	-0.4569	0.044	26.16	50.11	5.555	2.057
SPBC25H2.10C	SPBC25H2.10c	0.951	-0.4521	0.022	26.7	51.1	11.86	2.892
SPAC1399.04C	SPAC1399.04c	0.9473	-0.4508	0.024	26.65	51.01	10.2	3.595
SPAC3G6.13C	rpl4101	0.9554	-0.4501	0.020	30.32	57.92	12.7	3.541
SPBP35G2.06C	nup131	0.919	-0.4451	0.037	26.35	50.43	7.189	1.5
SPBC2F12.05C	SPBC2F12.05c	0.9162	-0.4409	0.038	24.28	46.53	4.695	3.141
SPCC285.05	SPCC285.05	0.9235	-0.4362	0.035	26.17	50.08	7.689	1.065
SPAC20G4.01	caf16	0.7317	-0.4348	0.136	23.33	44.73	1.264	0.9974
SPCC663.14C	SPCC663.14c	0.867	-0.4339	0.062	21.93	42.1	3.378	1.714
SPCC777.17C	SPCC777.17c	0.9331	-0.4328	0.030	28.7	54.84	8.023	2.387
SPAC3H1.07	aru1	0.911	-0.4247	0.040	25.98	49.7	6.259	1.266
SPAC9E9.13	wos2	0.892	-0.4232	0.050	25.8	49.36	5.251	0.7822
SPCC24B10.18	SPCC24B10.18	0.931	-0.4177	0.031	26.12	49.94	7.656	2.067
SPBC365.11	SPBC365.11	0.8804	-0.4126	0.055	26.24	50.17	3.004	2.08
SPBC839.14C	SPBC839.14c	0.8944	-0.4031	0.048	22.95	43.96	4.941	1.119
SPAC6B12.03C	SPAC6B12.03c	0.9461	-0.4019	0.024	20.6	39.52	8.484	3.469
SPAC105.01C	kha1	0.776	-0.4012	0.110	31.26	59.59	1.47	1.091
SPAC57A7.07C	SPAC57A7.07c	0.8956	-0.4005	0.048	24.86	47.55	4.739	1.425
SPBC543.07	pek1	0.8776	-0.3928	0.057	23.63	45.22	2.362	2.071
SPAC1D4.02C	SPAC1D4.02c	0.9319	-0.3863	0.031	27.51	52.51	5.977	2.952
SPAC2G11.13	atg22	0.946	-0.3847	0.024	27.69	52.84	9.308	1.991
SPAC688.03C	SPAC688.03c	0.8875	-0.3828	0.052	24.88	47.55	4.58	0.6289
SPAC19D5.06C	din1	0.94	-0.3815	0.027	25.84	49.35	7.132	3.03
SPBC776.02C	dis2	0.9004	-0.3784	0.046	21.8	41.75	4.105	1.903
SPAC1039.08	SPAC1039.08	0.9454	-0.3758	0.024	10.4	20.27	7.305	3.546
SPCC18B5.10C	SPCC18B5.10c	0.9482	-0.3659	0.023	18.36	35.24	7.093	3.863
SPBC3H7.03C	SPBC3H7.03c	0.9554	-0.3598	0.020	7.758	15.28	7.921	4.508
SPCC1753.02C	git3	0.9116	-0.3565	0.040	24.89	47.51	4.468	1.952
SPAC644.09	SPAC644.09	0.927	-0.3556	0.033	27.12	51.72	5.7	2.137
SPCC1494.05C	ubp12	0.8182	-0.3494	0.087	15.43	29.71	2.335	0.7317
SPAC977.05C	SPAC977.05c	0.9105	-0.3468	0.041	22.57	43.13	3.463	2.307
SPAC343.19	lsb6	0.9194	-0.3442	0.036	26.85	51.18	5.761	0.7855
SPAC17A2.10C	SPAC17A2.10c	0.9162	-0.3373	0.038	27.06	51.56	4.819	1.661
SPCC191.10	SPCC191.10	0.9291	-0.3307	0.032	29.17	55.52	6.063	1.351

SPBC3H7.07C	ser2	0.9175	-0.3261	0.037	27.58	52.53	5.122	1.169
SPAC823.05C	tlg2	0.9529	-0.3226	0.021	3.542	7.274	7.274	3.542
SPAC1093.02	SPAC1093.02	0.9392	-0.3182	0.027	26.97	51.36	7.092	0.8989
SPBC29A3.13	pdp1	0.9429	-0.3156	0.026	24.39	46.49	6.38	2.494
SPBC16C6.02C	vps1302	0.9444	-0.3151	0.025	13.25	25.53	6.142	3.004
SPBC365.08C	SPBC365.08c	0.9558	-0.3151	0.020	26.17	49.84	9.381	1.899
SPCC622.18	rpl6	0.9442	-0.3131	0.025	28.9	54.99	6.983	2.048
SPBC16A3.13	meu7	0.8963	-0.3092	0.048	32.79	62.29	3.773	1.003
SPBC27B12.11C	SPBC27B12.11c	0.9493	-0.3053	0.023	14.52	27.9	7.579	2.1
SPAC1834.10C	SPAC1834.10c	0.9316	-0.3015	0.031	23.19	44.22	5.185	1.909
SPAC23C11.08	php3	0.9509	-0.2974	0.022	27.47	52.26	7.986	1.576
SPBC31F10.14C	hip3	0.9315	-0.296	0.031	19.84	37.89	2.418	2.976
SPBC29A3.10C	atp14	0.6963	-0.286	0.157	0.3218	1.144	1.144	0.3218
SPAC1783.02C	vps66	0.8943	-0.2787	0.049	22.68	43.22	2.7	1.41
SPBC106.19	SPBC106.19	0.9739	-0.2761	0.011	25.9	49.26	12.98	4.111
SPAC4H3.04C	SPAC4H3.04c	0.9623	-0.2754	0.017	22.71	43.27	7.896	3.692
SPAC5H10.09C	SPAC5H10.09c	0.9317	-0.2752	0.031	24.89	47.36	4.386	2.012
SPAC8E11.05C	SPAC8E11.05c	0.9742	-0.2734	0.011	18.77	35.83	12.1	4.891
SPBC1718.03	ker1	0.9365	-0.2705	0.028	6.256	12.28	4.341	2.297
SPAC14C4.07	SPAC14C4.07	0.9288	-0.2675	0.032	23.52	44.78	2.241	2.558
SPAC6B12.08	mug185	0.9724	-0.2592	0.012	22.51	42.85	10.75	4.334
SPBC11C11.07	rpl1801	0.9158	-0.253	0.038	9.532	18.42	3.092	1.603
SPAC3F10.18C	rpl4102	0.9741	-0.2517	0.011	21.39	40.73	10.88	4.632
SPAC6G10.12C	ace2	0.9684	-0.2434	0.014	11.18	21.5	7.759	4.214
SPBC25B2.06C	btb2	0.9674	-0.2389	0.014	28.95	54.93	9.588	2.05
SPAC630.06C	SPAC630.06c	0.9345	-0.2341	0.029	24.91	47.32	3.701	1.897
SPCC61.02	spt3	0.9665	-0.2318	0.015	30.21	57.3	8.256	2.913
SPAC22G7.05	SPAC22G7.05	0.9551	-0.2283	0.020	12.28	23.54	5.313	2.808
SPAC4H3.01	SPAC4H3.01	0.9736	-0.2255	0.012	29.08	55.15	10.05	3.727
SPBP4H10.14C	SPBP4H10.14c	0.9379	-0.2123	0.028	25.87	49.1	4.005	1.497
SPAC1556.02C	sdh1	0.9746	-0.2104	0.011	29.44	55.81	11.21	1.436
SPBP8B7.13	vac7	0.9526	-0.2067	0.021	27.61	52.35	4.298	2.431
SPAC19A8.14	SPAC19A8.14	0.9771	-0.1979	0.010	30.07	56.98	11.11	2.7
SPBC8D2.18C	SPBC8D2.18c	0.9774	-0.1907	0.010	28.87	54.7	10.92	2.54
SPBC713.09	SPBC713.09	0.977	-0.1893	0.010	29.49	55.86	10.53	2.643
SPBC1718.06	msp1	0.975	-0.1886	0.011	21.15	40.16	3.875	5.253
SPAC1250.04C	atl1	0.9447	-0.1859	0.025	22.27	42.26	4.463	0.8125
SPCC16C4.11	pef1	0.9473	-0.179	0.024	20.42	38.77	4.02	1.458
SPAC3C7.08C	elf1	0.9846	-0.1767	0.007	18.12	34.44	11.69	6.195
SPBC23G7.13C	SPBC23G7.13c	0.9683	-0.1759	0.014	24.2	45.88	6.628	2.309
SPAC637.11	rpm2	0.9614	-0.1736	0.017	22.4	42.48	5.262	1.985
SPBC2G2.02	syj1	0.9763	-0.1677	0.010	29.03	54.96	8.679	2.719
SPAC6G9.04	spo7	0.9817	-0.1663	0.008	28.57	54.09	11.43	3.24
SPBC215.01	SPBC215.01	0.9373	-0.1655	0.028	23.06	43.71	2.623	1.462
SPBC21C3.13	rps1901	0.9824	-0.1608	0.008	14.42	27.45	8.627	5.259
SPBC1685.07C	avt5	0.7953	-0.1587	0.099	0.3854	1.024	0.8279	0.3854
SPAC3H5.08C	SPAC3H5.08c	0.962	-0.1422	0.017	26.24	49.65	3.905	1.968
SPAC9G1.03C	rpl3001	0.9741	-0.1392	0.011	10.26	19.57	5.452	2.919
SPCC1739.05	set5	0.9815	-0.1333	0.008	11.5	21.9	7.411	3.878
SPBC1711.15C	SPBC1711.15c	0.9794	-0.1325	0.009	27.23	51.5	8.477	1.693
SPBC530.07C	SPBC530.07c	0.9741	-0.1313	0.011	8.738	16.69	5.787	2.345
SPBC6B1.03C	SPBC6B1.03c	0.9742	-0.1213	0.011	26.33	49.78	4.279	2.778
SPCC4E9.02	cig1	0.9613	-0.1206	0.017	34.93	65.97	2.613	1.927
SPCC1450.02	bdf1	0.9899	-0.1202	0.004	18.75	35.52	12.08	6.418
SPAC31A2.14	bun107	0.9791	-0.1189	0.009	23.1	43.71	7.343	1.74
SPAC1071.04C	spc2	0.9885	-0.1171	0.005	11.53	21.92	12.75	3.594
SPBC660.05	SPBC660.05	0.9821	-0.1169	0.008	25.07	47.4	7.279	3.14
SPCC11E10.08	rik1	0.9889	-0.1136	0.005	16.25	30.8	10.35	5.54
SPAC12G12.15	sif3	0.9714	-0.1127	0.013	21.22	40.16	3.31	2.432
SPAC30C2.06C	dml1	0.9703	-0.112	0.013	15.64	29.65	3.516	2.197
SPCC191.03C	SPCC191.03c	0.9789	-0.1117	0.009	27.07	51.15	6.443	2.094
SPBC23G7.11	mag2	0.9777	-0.1081	0.010	27.06	51.14	3.895	3.052
SPAC30D11.05	aps3	0.983	-0.1076	0.007	27.55	52.05	5.467	3.845

SPBC1685.14C	SPBC1685.14c	0.9896	-0.107	0.005	31.44	59.37	13.28	3.068
SPBC1271.03C	SPBC1271.03c	0.9826	-0.1012	0.008	27.67	52.26	6.735	2.64
SPAC1420.03	rpn501	0.9842	-0.1009	0.007	29	54.76	8.361	1.743
SPAC823.13C	SPAC823.13c	0.9866	-0.1006	0.006	29.26	55.27	9.661	2.297
SPCC1235.05C	fft2	0.9751	-0.09846	0.011	26.36	49.79	3.277	2.464
SPBP16F5.08C	SPBP16F5.08c	0.9823	-0.09602	0.008	26.52	50.09	7.338	1.009
SPBC14F5.10C	SPBC14F5.10c	0.9885	-0.08964	0.005	13.99	26.5	8.182	4.077
SPAC694.04C	SPAC694.04c	0.9908	-0.0887	0.004	29.66	55.99	12.12	3.503
SPAC20G8.09C	SPAC20G8.09c	0.9878	-0.08578	0.005	29	54.75	9.037	2.256
SPAC57A7.12	SPAC57A7.12	0.9204	-0.08393	0.036	0.971	1.986	0.817	0.6983
SPBC106.11C	plg7	0.9863	-0.08108	0.006	27.43	51.77	7.807	1.491
SPCC1235.02	bio2	0.9873	-0.07329	0.006	26.52	50.05	6.829	2.474
SPBC30B4.08	eri1	0.9783	-0.07052	0.010	23.05	43.52	4.437	0.4208
SPBC19C7.09C	uve1	0.9898	-0.06741	0.004	31.21	58.86	8.059	2.584
SPAC17G8.07	yaf9	0.9859	-0.05961	0.006	24.33	45.91	4.527	2.156
SPBC23G7.10C	SPBC23G7.10c	0.9873	-0.05897	0.006	28.08	52.96	5.549	1.958
SPAC20H4.07	rhp57	0.9869	-0.05434	0.006	19.45	36.71	5.211	1.491
SPAC1952.12C	csn71	0.9907	-0.04986	0.004	27.83	52.47	6.714	1.974
SPBC651.11C	apm3	0.9817	-0.04577	0.008	24.97	47.08	3.237	0.7669
SPBC3E7.16C	leu3	0.9739	-0.04201	0.011	36.15	68.12	1.709	0.8327
SPBC29A3.03C	SPBC29A3.03c	0.9901	-0.04075	0.004	27.52	51.87	5.035	1.592
SPBC16H5.06	rip1	0.9904	-0.03718	0.004	14.08	26.56	4.842	1.367
SPCC126.03	pus1	0.9879	-0.03564	0.005	17.92	33.8	3.132	1.522
SPAC17G6.15C	SPAC17G6.15c	0.9964	-0.0336	0.002	25.54	48.13	10.37	4.559
SPCC330.03C	SPCC330.03c	0.994	-0.03284	0.003	27.26	51.36	4.635	3.345
SPAC6B12.06C	rrg9	0.9953	-0.03199	0.002	28.8	54.27	9.155	1.115
SPBC31E1.01C	atg2	0.9909	-0.02604	0.004	29.05	54.73	3.724	0.7978
SPAC22F3.06C	lon1	0.9932	-0.02403	0.003	23.19	43.69	3.44	2.001
SPBC1105.08	SPBC1105.08	0.9925	-0.02299	0.003	22.28	41.99	3.654	1.275
SPCC16C4.10	SPCC16C4.10	0.9939	-0.02268	0.003	24.48	46.12	2.829	2.394
SPBC3F6.01C	SPBC3F6.01c	0.9903	-0.02161	0.004	26.29	49.52	2.024	1.321
SPBC3B9.04	SPBC3B9.04	0.9977	-0.01205	0.001	24.12	45.41	6.199	2.183
SPBC16E9.09C	SPBC16E9.09c	0.9994	-0.00964	0.000	24.42	45.98	17.31	8.239
SPAPB17E12.04C	csn2	0.9987	-0.00921	0.001	25.54	48.09	8.347	2.755
SPAC227.04	SPAC227.04	0.9994	-0.00731	0.000	22.45	42.26	12.44	7.642
SPAC1783.07C	pap1	0.9989	-0.00264	0.000	14.48	27.25	3.156	0.6283
SPBC11C11.09C	rpl502	0.9887	-0.00062	0.005	0.03022	0.05804	0.05804	0.03022
SPBC1105.10	rav1	0.391	-1.04E-17	0.408	0	1.96E-17	1.96E-17	0
SPBC25D12.02C	dnt1	1	0	0.000	0	0	0	0
SPAC12G12.13C	cid14	1	0	0.000	0	0	0	0
SPBC21C3.16C	spt4	1	0	0.000	0	0	0	0
SPAC4D7.10C	spt20	1	0	0.000	0	0	0	0
SPBC685.07C	rpl2701	1	0	0.000	0	0	0	0
SPCC663.01C	ekc1	1	0	0.000	0	0	0	0
SPBC887.18C	hfi1	1	0	0.000	0	0	0	0
SPBC660.14	mik1	1	0.000665	0.000	24.62	46.34	10.92	8.559
SPBP4H10.03	oxa102	0.9998	0.001318	0.000	12.53	23.58	8.156	4.194
SPBP35G2.12	SPBP35G2.12	0.9993	0.004565	0.000	29.58	55.67	8.55	2.726
SPBC28E12.02	SPBC28E12.02	0.999	0.005249	0.000	24.39	45.89	6.672	1.251
SPBC19G7.02	SPBC19G7.02	0.9984	0.007114	0.001	23.52	44.26	5.231	1.967
SPAC22F8.05	SPAC22F8.05	0.9983	0.01123	0.001	20.97	39.44	4.197	4.504
SPAC3F10.11C	abc2	0.9975	0.01166	0.001	26.75	50.33	5.959	1.476
SPAC24B11.06C	sty1	0.9777	0.01322	0.010	0.4063	0.7398	0.5389	0.3664
SPBC317.01	mbx2	0.9936	0.01365	0.003	19.05	35.83	1.25	1.458
SPAC9.07C	SPAC9.07c	0.9984	0.01409	0.001	34.52	64.94	12.03	2.222
SPAC27D7.03C	mei2	0.9969	0.01823	0.001	27.55	51.82	5.396	3.499
SPBC8D2.02C	SPBC8D2.02c	0.9949	0.02133	0.002	25.71	48.35	4.172	2.323
SPBC1652.02	SPBC1652.02	0.9936	0.02212	0.003	24.05	45.23	3.153	2.023
SPBC2D10.20	ubc1	0.9927	0.02427	0.003	27.4	51.53	3.518	1.713
SPAC19G12.02C	pms1	0.9839	0.03028	0.007	22.98	43.19	2.245	0.7947
SPAC4A8.10	SPAC4A8.10	0.9908	0.03291	0.004	28.37	53.34	3.247	2.102
SPAC750.06C	SPAC750.06c	0.9947	0.03427	0.002	24.85	46.71	5.2	4.086
SPBC902.03	SPBC902.03	0.9947	0.03508	0.002	28.13	52.87	4.78	4.279

SPAC23H3.08C	bub3	0.9912	0.04348	0.004	26.23	49.28	6.218	1.708
SPBC1683.08	ght4	0.9926	0.04377	0.003	26.9	50.54	4.655	3.74
SPAC29B12.11C	SPAC29B12.11c	0.9926	0.05399	0.003	28.72	53.95	8.584	3.145
SPBC1604.18C	SPBC1604.18c	0.9787	0.05781	0.009	23.28	43.71	2.923	1.381
SPAC22E12.19	snt1	0.9879	0.05891	0.005	26.32	49.42	6.416	1.298
SPCC1672.06C	asp1	0.9883	0.05893	0.005	28.29	53.13	6.228	1.93
SPAC1296.03C	sxa2	0.9922	0.06433	0.003	29.22	54.87	10.33	2.856
SPAC26H5.11	mug56	0.9862	0.06772	0.006	27.61	51.85	5.958	1.973
SPBC24C6.05	sec28	0.9849	0.07072	0.007	25.27	47.43	4.916	2.471
SPAC13G6.14	aps1	0.9898	0.07417	0.004	11.71	21.91	7.393	3.932
SPCC965.09	SPCC965.09	0.9787	0.08199	0.009	27.61	51.82	5.059	1.057
SPAC23H3.06	apl6	0.9874	0.08273	0.006	23.35	43.79	8.064	2.522
SPBC31F10.05	mug37	0.9842	0.08278	0.007	29.78	55.9	6.895	1.404
SPAC5D6.10C	mug116	0.9667	0.08504	0.015	32.55	61.1	2.594	1.382
SPAC1A6.06C	meu31	0.9934	0.08509	0.003	20.16	37.78	13.45	6.889
SPAPB1E7.04C	SPAPB1E7.04c	0.993	0.09085	0.003	26.43	49.57	15.02	5.79
SPBC15C4.04C	SPBC15C4.04c	0.9881	0.09153	0.005	40.78	76.59	10.53	1.1
SPAC17G6.02C	tco1	0.9842	0.09444	0.007	30.66	57.52	7.82	1.696
SPBC2D10.19C	SPBC2D10.19c	0.9879	0.09555	0.005	23.8	44.61	9.51	3.23
SPBC577.14C	spa1	0.9841	0.09798	0.007	24.37	45.68	6.862	2.996
SPAC24H6.11C	SPAC24H6.11c	0.9902	0.103	0.004	19.8	37.07	12.1	4.844
SPAPB24D3.08C	SPAPB24D3.08c	0.9899	0.1033	0.004	33.12	62.14	13.25	3.071
SPAC29E6.10C	SPAC29E6.10c	0.9742	0.1034	0.011	16.68	31.2	2.99	2.596
SPCC417.09C	SPCC417.09c	0.9836	0.1117	0.007	29.06	54.48	4.366	4.591
SPAC1805.07C	dad2	0.9864	0.1122	0.006	15.83	29.58	6.237	5.309
SPBC17G9.12C	SPBC17G9.12c	0.9822	0.1129	0.008	24.73	46.33	8.133	2.04
SPCC330.06C	SPCC330.06c	0.8504	0.1198	0.070	0.4844	0.6862	0.6862	0.4844
SPAC26H5.08C	bgl2	0.9905	0.1225	0.004	19.63	36.72	13.09	6.958
SPBC3B9.15C	scp1	0.9803	0.1324	0.009	28.69	53.76	8.021	2.816
SPAC19G12.16C	adg2	0.9895	0.1347	0.005	18.5	34.56	13.45	6.714
SPAC13A11.03	mcp7	0.9715	0.1387	0.013	27	50.55	6.145	1.691
SPBC365.14C	uge1	0.9826	0.1407	0.008	14.74	27.48	6.93	4.93
SPAC56F8.09	rrp8	0.9843	0.1429	0.007	27.31	51.13	10.83	3.871
SPAC4F10.13C	mpd2	0.9814	0.1446	0.008	15.62	29.13	8.917	3.557
SPBC902.02C	ctf18	0.9647	0.1457	0.016	24.91	46.62	4.751	1.882
SPAC1783.06C	atg12	0.9787	0.1459	0.009	30.13	56.44	8.842	2.1
SPAC17C9.07	alg8	0.9581	0.151	0.019	21.92	40.97	2.206	2.45
SPAC23C4.07	tht2	0.9716	0.1527	0.013	25.42	47.56	6.516	2.156
SPCC1020.09	gnr1	0.9331	0.1576	0.030	24.91	46.59	2.043	1.429
SPAPB17E12.13	rpl1802	0.9764	0.1618	0.010	23.68	44.27	7.676	3.298
SPBC29A3.14C	trt1	0.982	0.1661	0.008	15.39	28.65	8.396	5.439
SPAC806.07	ndk1	0.9538	0.1708	0.021	28.76	53.82	4.895	0.9245
SPBC19G7.16	iws1	0.9699	0.1714	0.013	11.02	20.41	5.719	3.261
SPBC16A3.14	SPBC16A3.14	0.9771	0.1744	0.010	29.28	54.79	10.2	1.703
SPBC29A10.01	ccr1	0.9676	0.1776	0.014	29.65	55.47	6.913	1.893
SPAC1687.19C	SPAC1687.19c	0.9653	0.179	0.015	28.92	54.1	6.616	1.65
SPBP8B7.30C	thi5	0.9697	0.1794	0.013	24.06	44.94	7.498	2.029
SPAC23D3.12	SPAC23D3.12	0.9252	0.18	0.034	17.62	32.83	2.43	1.31
SPBC651.05C	dot2	0.9681	0.1808	0.014	24.78	46.3	5.25	3.32
SPAC13C5.05C	SPAC13C5.05c	0.9633	0.1817	0.016	26.56	49.66	5.682	2.264
SPAC3H8.04	SPAC3H8.04	0.9614	0.1834	0.017	26.1	48.78	4.653	2.66
SPAC1250.05	rpl3002	0.9644	0.1861	0.016	27.92	52.21	6.096	2.323
SPAC14C4.09	agn1	0.9641	0.1868	0.016	19.51	36.38	5.053	2.942
SPAC16A10.05C	dad1	0.9555	0.1888	0.020	7.299	13.38	4.281	2.419
SPBC1703.11	SPBC1703.11	0.9706	0.1894	0.013	27.74	51.86	7.639	2.757
SPBC106.07C	SPBC106.07c	0.9688	0.1895	0.014	6.745	12.34	4.507	3.936
SPBC1683.10C	pcl1	0.9744	0.1903	0.011	20.09	37.46	8.792	3.2
SPAC1556.06.1	meu1-1	0.9605	0.1932	0.018	24.26	45.29	6.109	1.761
SPAC29B12.12	SPAC29B12.12	0.9567	0.1953	0.019	29.16	54.51	5.59	1.68
SPBC106.08C	mug2	0.9494	0.1968	0.023	27.18	50.78	4.346	1.871
SPBC660.11	tcg1	0.797	0.2081	0.099	0.8164	1.145	1.145	0.4751
SPAC17H9.01	cid16	0.94	0.2136	0.027	26.71	49.88	4.793	0.7036
SPCC1840.03	sal3	0.974	0.2136	0.011	13.09	24.23	8.142	4.574

SPBPB2B2.08	SPBPB2B2.08	0.9517	0.2145	0.021	23.88	44.55	5.38	1.792
SPAC18B11.07C	rhp6	0.9517	0.2179	0.021	26.15	48.81	4.073	2.68
SPBC2A9.04C	SPBC2A9.04c	0.9662	0.2204	0.015	21.34	39.76	7.317	3.108
SPBC887.15C	sur2	0.9782	0.2238	0.010	16.07	29.82	10.15	5.712
SPCC594.06C	SPCC594.06c	0.957	0.224	0.019	25.2	47.01	6.106	2.285
SPAC3F10.16C	SPAC3F10.16c	0.6769	0.2271	0.169	0.4708	0.4586	0.4586	0.4708
SPAC26A3.11	SPAC26A3.11	0.9319	0.2278	0.031	22.28	41.5	2.165	2.241
SPCC794.10	SPCC794.10	0.9697	0.2315	0.013	28.53	53.25	10.17	1.824
SPAC19G12.06C	hta2	0.9514	0.2353	0.022	28.02	52.29	5.565	2.204
SPAC30D11.01C	SPAC30D11.01c	0.9593	0.2392	0.018	28.66	53.48	7.059	2.423
SPBC4B4.12C	SPBC4B4.12c	0.9397	0.2409	0.027	28.05	52.35	5.207	1.142
SPAC3C7.10	pex13	0.9343	0.2469	0.030	26.96	50.28	3.417	2.222
SPAC10F6.17C	SPAC10F6.17c	0.9415	0.2491	0.026	28.24	52.69	5.22	1.651
SPBC36B7.05C	SPBC36B7.05c	0.9348	0.2556	0.029	25.31	47.16	4.331	1.912
SPAC1296.01C	SPAC1296.01c	0.8584	0.2592	0.066	29.8	55.59	0.9707	1.261
SPBPB7E8.01	SPBPB7E8.01	0.9509	0.2604	0.022	9.988	18.31	4.078	3.389
SPBC2G2.09C	crs1	0.9699	0.2619	0.013	29.15	54.38	11.01	2.982
SPBC2F12.09C	atf21	0.9794	0.2625	0.009	18.07	33.52	13.29	6.754
SPBC2D10.05	exg3	0.9491	0.2671	0.023	26.58	49.53	6.394	2.076
SPBC1683.12	SPBC1683.12	0.9683	0.271	0.014	24.46	45.52	10.27	3.527
SPAC17C9.08	pnu1	0.9466	0.2715	0.024	28.42	52.97	6.608	1.481
SPAC1B2.03C	SPAC1B2.03c	0.9314	0.2718	0.031	20.88	38.78	4.442	1.888
SPCC18.06C	caf1	0.9708	0.2731	0.013	28.08	52.34	11.52	3.545
SPBC2D10.07C	SPBC2D10.07c	0.9678	0.2822	0.014	28.93	53.92	11.09	3.007
SPCC757.09C	rnc1	0.9571	0.2826	0.019	21.56	40.05	8.086	2.554
SPBC409.11	meu18	0.9385	0.2843	0.028	20.85	38.7	2.248	3.236
SPAC26A3.14C	SPAC26A3.14c	0.9295	0.2844	0.032	25.4	47.27	4.267	2.091
SPBC1348.01	SPBC1348.01	0.9559	0.2887	0.020	27.82	51.83	7.751	2.803
SPAC637.07	moe1	0.9592	0.3037	0.018	11.16	20.43	7.834	3.878
SPBP8B7.28C	stc1	0.9545	0.307	0.020	22.66	42.07	3.416	4.712
SPAC1F7.12	yak3	0.9451	0.3091	0.025	26.45	49.19	3.945	3.71
SPCC18.03	SPCC18.03	0.9363	0.3119	0.029	26.29	48.89	6.149	1.729
SPAC13A11.04C	ubp8	0.9684	0.3119	0.014	24.96	46.4	12.12	3.821
SPBC365.03C	rpl2101	0.9213	0.3133	0.036	4.132	7.187	3.22	2.591
SPBC409.18	SPBC409.18	0.9458	0.3151	0.024	28.23	52.55	7.764	1.289
SPBC1D7.03	mug80	0.9396	0.3153	0.027	16.19	29.87	6.475	1.928
SPBPB2B2.01	SPBPB2B2.01	0.9743	0.3177	0.011	16.06	29.63	13.75	5.965
SPBC887.08	SPBC887.08	0.8932	0.3182	0.049	22.7	42.12	2.897	1.669
SPAC1786.01C	SPAC1786.01c	0.8854	0.324	0.053	28.09	52.26	3.009	1.443
SPAC694.03	SPAC694.03	0.9576	0.3266	0.019	30.81	57.37	10.52	1.1
SPBC13G1.02	SPBC13G1.02	0.9619	0.3273	0.017	27.34	50.84	10.7	3.156
SPBC405.04C	ypt7	0.9597	0.33	0.018	29.78	55.43	9.57	3.61
SPAC1952.15C	rec24	0.8347	0.3327	0.078	22.5	41.72	0.9525	1.4
SPAC5H10.06C	adh4	0.9235	0.3328	0.035	28.38	52.8	4.468	2.327
SPBC21C3.18	spo4	0.949	0.3359	0.023	24.24	45	8.044	2.574
SPAC1805.01C	ppk6	0.8889	0.3444	0.051	24.86	46.15	3.448	1.486
SPBC3E7.15C	lac1	0.8559	0.3473	0.068	1.453	2.082	2.082	1.453
SPBC1921.03C	mex67	0.9637	0.3489	0.016	16.98	31.3	9.935	5.117
SPAC24H6.13	SPAC24H6.13	0.9053	0.3505	0.043	26.05	48.38	4.709	1.212
SPAC26A3.02	myh1	0.9732	0.3509	0.012	26.87	49.91	16.76	4.24
SPBC713.08	mim1	0.9493	0.3511	0.023	11.1	20.23	7.095	3.713
SPAC25B8.19C	SPAC25B8.19c	0.9587	0.353	0.018	8.816	15.93	6.73	5.428
SPBC16E9.08	mcp4	0.9394	0.3579	0.027	24.87	46.14	6.629	2.807
SPAC1952.11C	ure2	0.9116	0.3584	0.040	26.6	49.38	4.554	1.921
SPAC4G9.06C	chz1	0.9347	0.3594	0.029	24.49	45.42	6.945	1.892
SPCC622.15C	SPCC622.15c	0.885	0.3596	0.053	29.19	54.26	4.051	0.9019
SPBC16A3.08C	SPBC16A3.08c	0.9549	0.3613	0.020	11.57	21.1	7.876	4.483
SPAC13G6.10C	asl1	0.9444	0.3641	0.025	10.18	18.48	6.239	3.745
SPAC2G11.06	vps4	0.8463	0.367	0.072	21.52	39.81	2.238	1.366
SPAC31F12.01	zds1	0.9556	0.3705	0.020	7.191	12.84	8.459	4.737
SPAC19A8.03	SPAC19A8.03	0.8662	0.3712	0.062	20.23	37.38	3.114	1.304
SPBC530.08	SPBC530.08	0.9617	0.3764	0.017	30.93	57.51	11.91	3.941
SPBC776.09	ste13	0.9533	0.3796	0.021	13.08	23.91	8.281	4.403

SPAC222.04C	ies6	0.949	0.3868	0.023	12.14	22.13	7.792	4.067
SPBCPT2R1.08C	tlh2	0.9692	0.3892	0.014	16	29.38	12.95	6.774
SPCC364.07	SPCC364.07	0.9415	0.3914	0.026	24.65	45.67	8.209	2.589
SPAC1F8.04C	SPAC1F8.04c	0.8885	0.3928	0.051	23.87	44.19	2.881	2.19
SPCC1795.10C	SPCC1795.10c	0.8819	0.3959	0.055	25.81	47.84	3.695	1.629
SPCC1393.09C	SPCC1393.09c	0.8847	0.3999	0.053	27.44	50.9	4.201	1.373
SPBC31F10.10C	SPBC31F10.10c	0.9126	0.4017	0.040	26.02	48.23	3.05	3.063
SPCC320.12	atp23	0.8915	0.406	0.050	8.718	15.64	3.778	2.029
SPAC10F6.04	SPAC10F6.04	0.9338	0.4075	0.030	26.09	48.35	7.663	2.251
SPBC16G5.05C	SPBC16G5.05c	0.9304	0.4115	0.031	20.94	38.65	4.767	3.713
SPAC2F3.11	SPAC2F3.11	0.9346	0.4159	0.029	29.83	55.36	8.182	1.979
SPBPB10D8.01	SPBPB10D8.01	0.9468	0.4162	0.024	26.95	49.95	9.371	3.249
SPAC9E9.08	rad26	0.915	0.422	0.039	27.83	51.59	5.872	2.12
SPAC23C4.03	hrk1	0.9627	0.4227	0.017	19.95	36.74	12.78	5.366
SPBC1921.06C	pvg3	0.9259	0.4269	0.033	18.91	34.78	6.703	2.56
SPAPB1A10.05	SPAPB1A10.05	0.7845	0.4277	0.105	32.18	59.75	1.412	1.275
SPAC1B3.05	not3	0.9577	0.4297	0.019	10.5	18.96	8.543	6.263
SPAC1805.16C	SPAC1805.16c	0.8765	0.4311	0.057	23.46	43.33	2.708	2.213
SPAC227.07C	pab1	0.8663	0.432	0.062	9.061	16.24	3.31	1.803
SPBC1861.03	mak10	0.8932	0.4337	0.049	24.15	44.64	5.499	0.6499
SPAC4G9.16C	rpl901	0.3141	0.4372	0.503	0.6381	0.3782	0.2041	0.3925
SPAC644.07	SPAC644.07	0.9203	0.4383	0.036	5.122	8.815	5.788	3.008
SPCC11E10.01	SPCC11E10.01	0.9476	0.4392	0.023	21.4	39.45	8.94	4.306
SPBC651.10	nse5	0.9341	0.4395	0.030	11.4	20.63	6.286	3.846
SPAC23H3.09C	gly1	0.9367	0.4462	0.028	11.33	20.49	7.17	3.809
SPAC13G7.04C	mac1	0.9309	0.4469	0.031	28.51	52.81	6.679	3.445
SPAC13G6.12C	chs1	0.9104	0.4504	0.041	26.95	49.88	6.104	1.992
SPAC821.13C	SPAC821.13c	0.967	0.4532	0.015	20.96	38.59	14.31	7.234
SPAC8E11.02C	rad24	0.9533	0.4558	0.021	15.43	28.19	10.06	5.214
SPAC3H5.10	rpl3202	0.9466	0.4593	0.024	10.17	18.28	8.698	4.678
SPCC1322.15	rpl3402	0.8824	0.4737	0.054	26.91	49.75	5.029	1.429
SPAC3A11.14C	pkl1	0.9135	0.4812	0.039	28.45	52.64	7.216	1.625
SPCC1322.02	SPCC1322.02	0.877	0.4824	0.057	27.77	51.35	3.755	2.221
SPAC19E9.01C	nup40	0.8768	0.4925	0.057	26.11	48.22	5.446	0.4758
SPAC30.04C	abc4	0.9138	0.4975	0.039	19.14	35.09	3.719	3.869
SPCC16C4.12	naa20	0.9668	0.4991	0.015	19.98	36.67	14.17	8.678
SPAC6B12.15	cpc2	0.9466	0.5076	0.024	12.65	22.85	10.12	4.88
SPCC584.03C	SPCC584.03c	0.8885	0.5087	0.051	22.14	40.71	4.053	2.721
SPAC22F3.04	mug62	0.7924	0.5113	0.101	23.67	43.59	2.515	1.29
SPAC15E1.04	SPAC15E1.04	0.9517	0.5115	0.021	14.95	27.17	10.81	5.723
SPCC663.04	rpl39	0.839	0.5126	0.076	3.06	4.794	2.97	1.826
SPBC725.10	SPBC725.10	0.637	0.5158	0.196	17.63	32.22	1.603	0.5812
SPBC530.09C	SPBC530.09c	0.8874	0.5179	0.052	26.75	49.37	4.904	2.341
SPCC663.11	saf1	0.8139	0.5191	0.089	21.93	40.3	3.446	0.9944
SPAC30D11.12	rpl3802	0.8807	0.5207	0.055	27.28	50.36	5.335	1.677
SPAC20H4.05C	SPAC20H4.05c	0.9371	0.5212	0.028	32.62	60.41	10.84	2.302
SPAC631.01C	acp2	0.881	0.5225	0.055	28.56	52.76	5.395	1.658
SPAC3G9.08	png1	0.914	0.5257	0.039	12.54	22.62	7.725	2.081
SPAC1039.09	isp5	0.9059	0.5396	0.043	23.82	43.82	6.047	2.969
SPCC417.05C	chr2	0.9604	0.542	0.018	18.43	33.66	13.92	7.412
SPCC5E4.07	rpl2802	0.7715	0.5526	0.113	2.177	3.058	2.15	1.411
SPAC227.17C	SPAC227.17c	0.8851	0.5531	0.053	31.23	57.74	5.798	1.942
SPCC594.04C	SPCC594.04c	0.9575	0.5538	0.019	20.68	37.88	12.9	7.235
SPBC215.06C	SPBC215.06c	0.8991	0.556	0.046	26.59	49	6.97	1.857
SPAC22H12.02	tfg3	0.924	0.5573	0.034	19.51	35.68	8.453	3.32
SPCC1322.09	SPCC1322.09	0.9511	0.5574	0.022	20.43	37.41	12	5.936
SPAC12G12.03	cip2	0.8101	0.5584	0.091	28.63	52.83	3.954	0.4235
SPCC777.13	vps35	0.8972	0.5626	0.047	9.105	16.08	5.375	3.043
SPBC21D10.09C	SPBC21D10.09c	0.7828	0.5627	0.106	29.09	53.69	3.104	1.01
SPBC2G5.03	ctu1	0.8076	0.5647	0.093	24.81	45.64	1.779	1.966
SPAC22H12.03	SPAC22H12.03	0.8798	0.5658	0.056	26.58	48.96	5.84	1.707
SPCC737.09C	hmt1	0.8691	0.5696	0.061	28.86	53.25	5.387	1.588
SPAC20G4.04C	hus1	0.8966	0.5703	0.047	22.11	40.55	6.464	2.392

SPAC977.16C	dak2	0.8945	0.5721	0.048	27.27	50.25	6.29	2.404
SPBC16G5.06	SPBC16G5.06	0.9061	0.5735	0.043	25.34	46.61	5.749	3.513
SPAC1952.17C	SPAC1952.17c	0.9524	0.579	0.021	19.08	34.83	12.51	6.521
SPAC24B11.12C	SPAC24B11.12c	0.7462	0.5837	0.127	27.38	50.43	2.214	1.257
SPAC17G6.17	pof8	0.9395	0.5876	0.027	17.16	31.19	9.955	5.207
SPAC22E12.11C	set3	0.6645	0.5923	0.178	21.82	39.95	2.151	0.5677
SPAC1805.10	SPAC1805.10	0.8311	0.5936	0.080	26.58	48.91	4.64	0.7944
SPAC167.06C	mug143	0.8781	0.594	0.056	24.01	44.07	5.155	2.502
SPAC4G9.15	SPAC4G9.15	0.8794	0.5949	0.056	8.402	14.69	4.692	2.808
SPBC14C8.15	SPBC14C8.15	0.8707	0.5956	0.060	24.44	44.89	3.342	2.984
SPAC17H9.09C	ras1	0.9193	0.5967	0.037	19.86	36.26	7.768	4.052
SPBC16A3.06	SPBC16A3.06	0.9201	0.6041	0.036	23.56	43.2	8.417	3.635
SPAC4D7.03	pop2	0.4517	0.605	0.345	33.35	61.63	0.6974	0.6396
SPBC25H2.11C	spt7	0.3659	0.6057	0.437	0.8358	0.4331	0.3389	0.6068
SPBC25B2.01	SPBC25B2.01	0.8874	0.6059	0.052	26.78	49.26	4.26	3.389
SPAC4F8.03	sdo1	0.4012	0.6106	0.397	0.9999	0.7327	0.3574	0.6629
SPCC1827.04	SPCC1827.04	0.9341	0.6206	0.030	30.59	56.41	11.85	3.282
SPAC12B10.07	acp1	0.8997	0.6219	0.046	28.99	53.38	4.424	4.048
SPACUNK4.08	SPACUNK4.08	0.8912	0.6256	0.050	29.09	53.58	7.413	1.732
SPCC663.13C	naa50	0.9245	0.6271	0.034	32.48	59.95	11.11	1.776
SPBC25H2.14	mug16	0.9484	0.6298	0.023	21.73	39.72	13.99	5.599
SPAC8C9.03	cgs1	0.8755	0.6302	0.058	12.2	21.78	5.262	2.789
SPAC25H1.03	mug66	0.8691	0.6335	0.061	27.45	50.46	5.303	2.366
SPBC3E7.05C	SPBC3E7.05c	0.8603	0.6354	0.065	22.74	41.6	5.426	1.864
SPBC24C6.09C	SPBC24C6.09c	0.8961	0.6368	0.048	27.56	50.68	7.99	1.699
SPBC29A10.16C	SPBC29A10.16c	0.9531	0.6379	0.021	21.89	40	13.87	7.344
SPCC31H12.06	mug111	0.789	0.642	0.103	25.71	47.18	4.063	0.4899
SPAC13C5.06C	mug121	0.9271	0.6425	0.033	26.88	49.39	9.908	4.179
SPBC16G5.07C	SPBC16G5.07c	0.8451	0.6453	0.073	26.97	49.54	4.917	1.747
SPCC1620.04C	mug55	0.8613	0.646	0.065	26.21	48.11	5.48	1.983
SPAC4H3.03C	SPAC4H3.03c	0.8759	0.6504	0.058	26.54	48.73	6.186	2.224
SPBC21D10.12	hob1	0.8691	0.6544	0.061	26.99	49.57	6.58	1.235
SPAC630.07C	SPAC630.07c	0.9046	0.6559	0.044	26.48	48.61	8.834	2.128
SPAC3G9.03	rpl2301	0.909	0.6597	0.041	8.069	13.94	5.448	4.662
SPCC1235.08C	pdh1	0.9117	0.66	0.040	24.13	44.17	8.072	3.761
SPAC1A6.09C	lag1	0.9182	0.6602	0.037	25.56	46.87	8.011	4.458
SPBP35G2.03C	sgo1	0.8545	0.6613	0.068	27.46	50.44	5.781	1.451
SPAC1F7.13C	rpl801	0.7114	0.663	0.148	3.207	4.788	2.007	1.392
SPCC830.08C	yop1	0.878	0.6674	0.057	27.73	50.94	6.41	2.366
SPBC15D4.06	naa30	0.8468	0.6688	0.072	24.71	45.25	3.823	2.609
SPBC12C2.04	SPBC12C2.04	0.9376	0.6714	0.028	20.66	37.63	11.73	5.356
SPAC16E8.05C	mde1	0.9454	0.6725	0.024	17.92	32.47	12.07	6.906
SPAC13C5.07	rad32	0.9214	0.6795	0.036	14.99	26.94	7.589	5.204
SPCC736.07C	SPCC736.07c	0.9217	0.6823	0.035	13.82	24.72	8.407	4.936
SPAC4G8.05	ppk14	0.8858	0.6854	0.053	20.55	37.38	5.881	3.339
SPCC1223.10C	eaf1	0.9198	0.6855	0.036	28.09	51.57	10.95	2.707
SPBC902.06	mto2	0.8323	0.6901	0.080	28.81	52.92	5.088	1.479
SPAC17A5.16	ftp105	0.8808	0.692	0.055	30.62	56.33	7.364	1.907
SPBC1921.07C	sgf29	0.9357	0.6952	0.029	15.3	27.49	11.05	5.813
SPBC31F10.02	SPBC31F10.02	0.8381	0.6977	0.077	26.71	48.96	2.322	2.955
SPAC3H1.05	SPAC3H1.05	0.9368	0.6981	0.028	18.9	34.25	10.45	6.359
SPBC16H5.12C	SPBC16H5.12c	0.5293	0.7001	0.276	30.01	55.17	1.511	0.6717
SPAC9.13C	cwf16	0.9473	0.7034	0.024	20.73	37.7	13.61	7.219
SPBC660.12C	SPBC660.12c	0.8997	0.7054	0.046	30.01	55.15	9.073	2.119
SPBC1711.02	matmc_1	0.8144	0.7081	0.089	27.98	51.33	4.814	1.231
SPBC1703.07	SPBC1703.07	0.8684	0.7105	0.061	28.26	51.85	6.397	2.265
SPCC584.12	mug42	0.9231	0.712	0.035	31.27	57.51	12.1	2.566
SPAC16E8.14C	tae1	0.8334	0.7121	0.079	25.97	47.53	5.485	1.266
SPBC21C3.07C	SPBC21C3.07c	0.8521	0.7192	0.070	26.58	48.67	4.54	2.795
SPAC630.04C	SPAC630.04c	0.8931	0.7201	0.049	30.48	56.02	7.933	2.883
SPBC8D2.11	SPBC8D2.11	0.9104	0.7216	0.041	26.39	48.32	5.962	5.206
SPBC25H2.15	SPBC25H2.15	0.8215	0.7246	0.085	6.493	10.86	3.717	2.35
SPAC9.11	SPAC9.11	0.7874	0.728	0.104	21.48	39.07	4.563	0.5706

SPBC18A7.02C	SPBC18A7.02c	0.89	0.7333	0.051	22.36	40.71	7.257	3.306
SPCC830.06	SPCC830.06	0.9222	0.734	0.035	17.04	30.68	8.274	5.692
SPBP4H10.08	qcr10	0.8315	0.7378	0.080	27.85	51.04	5.526	1.433
SPAC1834.07	klp3	0.9146	0.7386	0.039	31.26	57.45	10.28	3.639
SPAC664.10	klp2	0.8966	0.7406	0.047	23.65	43.11	8.521	2.995
SPAC11E3.12	SPAC11E3.12	0.9037	0.7458	0.044	27.46	50.28	9.439	3.025
SPBC577.03C	SPBC577.03c	0.8284	0.7469	0.082	27.98	51.25	5.249	1.704
SPAC3A11.02	cps3	0.9468	0.7493	0.024	17.98	32.42	14.83	7.345
SPBC21D10.07	cmc1	0.8724	0.7503	0.059	30.32	55.65	7.45	1.934
SPCC970.07C	raf2	0.9376	0.7517	0.028	18.21	32.87	12.39	6.441
SPAC18G6.01C	SPAC18G6.01c	0.8873	0.752	0.052	22.94	41.76	7.772	2.929
SPBC12C2.03C	SPBC12C2.03c	0.8038	0.7522	0.095	24.1	43.95	2.752	2.466
SPBC428.12C	SPBC428.12c	0.8538	0.7525	0.069	29.15	53.45	6.33	1.912
SPBC646.15C	SPBC646.15c	0.805	0.7542	0.094	26.4	48.28	4.72	1.438
SPCC417.06C	mug27	0.8971	0.7547	0.047	30.77	56.49	9.509	2.133
SPBC1861.05	SPBC1861.05	0.8649	0.756	0.063	6.41	10.64	4.322	3.548
SPCC970.05	rpl3601	0.7867	0.7565	0.104	27.08	49.55	4.11	1.515
SPAC16A10.01	SPAC16A10.01	0.606	0.7584	0.218	24.7	45.06	1.841	0.9936
SPCC70.02C	SPCC70.02c	0.8723	0.7587	0.059	26.68	48.78	6.299	3.039
SPAC19E9.02	fin1	0.846	0.7619	0.073	23.55	42.89	5.611	2.265
SPAC30D11.10	rad22	0.5612	0.7736	0.251	1.668	1.683	1.415	1.002
SPCC576.01C	SPCC576.01c	0.8835	0.777	0.054	24.7	45.03	7.865	2.844
SPBC1709.06	dus2	0.8054	0.7782	0.094	27.74	50.75	4.797	1.574
SPAC6F6.01	cch1	0.7772	0.7797	0.109	25.66	46.84	4.197	1.353
SPAC56F8.06C	alg10	0.8248	0.7817	0.084	22.58	41.02	3.33	2.847
SPCC1840.08C	SPCC1840.08c	0.9345	0.7819	0.029	18.42	33.19	11.97	6.536
SPAC1399.03	fur4	0.7602	0.7821	0.119	20.35	36.83	2.051	2.15
SPAC12B10.09	SPAC12B10.09	0.8611	0.7834	0.065	21.58	39.14	5.294	3.235
SPBC1306.02	SPBC1306.02	0.9059	0.7842	0.043	13.34	23.64	8.48	4.49
SPAC19D5.11C	ctf8	0.8451	0.7872	0.073	24.76	45.13	5.677	2.395
SPAC926.02	SPAC926.02	0.7887	0.7988	0.103	24.2	44.04	2.424	2.495
SPAC4G9.05	mpf1	0.8523	0.8038	0.069	27.8	50.82	6.916	1.739
SPCC188.12	spn6	0.8743	0.8106	0.058	29.84	54.64	7.364	2.942
SPBC2A9.02	SPBC2A9.02	0.8241	0.8107	0.084	26.13	47.66	3.389	2.955
SPAC607.06C	SPAC607.06c	0.912	0.8112	0.040	30.63	56.11	11.91	2.758
SPCC338.10C	cox5	0.8601	0.8166	0.065	9.532	16.4	5.786	3.203
SPAC186.08C	SPAC186.08c	0.8197	0.8175	0.086	30.77	56.37	5.034	2.141
SPBC16E9.07	mug100	0.6052	0.8213	0.218	23.91	43.45	2.2	0.9445
SPBC2F12.11C	rep2	0.7856	0.8243	0.105	5.891	9.536	3.201	2.331
SPAC22E12.14C	sck2	0.7988	0.8273	0.098	26.81	48.91	3.953	2.285
SPCC1020.10	oca2	0.8342	0.8279	0.079	29.97	54.86	5.867	2.113
SPBC12D12.09	rev7	0.8015	0.8312	0.096	25.02	45.53	5.258	1.367
SPAC12B10.05	icp55	0.8303	0.8324	0.081	29.54	54.03	6.261	1.497
SPBC1685.10	rps27	0.8957	0.8389	0.048	29.77	54.45	9.889	3.042
SPAC27D7.10C	EMPTY	0.8305	0.8416	0.081	28.3	51.68	5.864	2.07
SPAC15A10.10	mde6	0.9397	0.845	0.027	20.74	37.44	13.71	7.846
SPAC13F5.01C	msh1	0.8861	0.8455	0.053	25.19	45.82	9.54	2.267
SPAC732.02C	SPAC732.02c	0.8835	0.8462	0.054	32.44	59.46	8.516	3.139
SPAC821.09	eng1	0.8856	0.8468	0.053	31.5	57.69	9.261	2.602
SPAC6C3.04	cit1	0.8137	0.8478	0.090	28.3	51.67	4.993	2.187
SPBPB2B2.09C	SPBPB2B2.09c	0.8227	0.8527	0.085	27.63	50.39	5.313	2.293
SPBC14F5.07	doa10	0.7505	0.8538	0.125	27.09	49.37	3.442	1.798
SPBC56F2.14	mrpl44	0.6381	0.8551	0.195	25.34	46.09	2.561	1.054
SPCP31B10.05	tdp1	0.9268	0.8565	0.033	20.64	37.24	12.13	6.189
SPAPB17E12.02	yip12	0.8522	0.8566	0.069	29.86	54.6	7.146	2.133
SPCC364.02C	bis1	0.8341	0.8575	0.079	26.27	47.84	6.115	2.149
SPBC1289.14	SPBC1289.14	0.8469	0.8608	0.072	23.13	41.92	4.1	3.627
SPBC1D7.04	mlo3	0.8866	0.8633	0.052	12.51	21.93	7.563	4.183
SPAC4A8.04	isp6	0.735	0.87	0.134	23.67	42.91	2.683	1.982
SPCC1840.06	atp5	0.8216	0.8733	0.085	29.13	53.18	5.747	2.062
SPBC2G5.02C	SPBC2G5.02c	0.916	0.8769	0.038	20.36	36.66	11.72	4.952
SPBC1709.04C	cyp3	0.8002	0.8787	0.097	25.2	45.77	5.693	1.168
SPBC83.03C	tas3	0.8243	0.8796	0.084	20.39	36.73	5.829	2.152

SPAC29A4.17C	SPAC29A4.17c	0.8561	0.8868	0.067	21.89	39.53	7.474	2.403
SPCC1020.06C	tal1	0.8946	0.888	0.048	21.37	38.54	10.01	3.532
SPAC16A10.03C	SPAC16A10.03c	0.9131	0.889	0.039	15.56	27.62	10	5.721
SPAC25G10.04C	rec10	0.8545	0.8919	0.068	31.34	57.31	7.683	2.108
SPBC428.03C	pho4	0.7966	0.8976	0.099	26.45	48.1	4.969	2.015
SPBC1773.14	arg7	0.8395	0.8978	0.076	25.21	45.76	7.173	1.674
SPAC1A6.08C	mug125	0.9143	0.9232	0.039	20.44	36.74	12.2	5.026
SPBC4F6.10	vps901	0.8881	0.9299	0.052	13.68	23.99	8.086	4.655
SPBC691.04	SPBC691.04	0.7989	0.9311	0.098	28.52	51.93	5.88	1.417
SPCC584.16C	SPCC584.16c	0.9254	0.9311	0.034	21.71	39.11	10.97	7.52
SPBC21H7.04	dbp7	0.5017	0.9339	0.300	1.751	1.537	1.051	1.217
SPAPB1E7.07	glt1	0.6569	0.936	0.183	25.12	45.53	2.934	1.252
SPAC11E3.11C	SPAC11E3.11c	0.8652	0.9367	0.063	17.74	31.62	5.768	4.281
SPAC24H6.07	rps901	0.8124	0.9431	0.090	8.427	14.09	4.731	2.851
SPBC146.11C	mug97	0.5827	0.9463	0.235	26.16	47.45	1.259	1.445
SPBC13G1.13	tfb2	0.7672	0.9479	0.115	28.37	51.61	3.419	2.448
SPAC323.07C	SPAC323.07c	0.8837	0.9547	0.054	19.77	35.42	8.556	4.519
SPAC19G12.12	dlp1	0.8236	0.9557	0.084	13.4	23.43	5.41	3.071
SPAC1556.01C	rad50	0.8793	0.9605	0.056	7.682	12.65	7.531	4.547
SPBC16A3.12C	SPBC16A3.12c	0.7718	0.9711	0.112	29.3	53.32	5.265	1.454
SPBC691.05C	SPBC691.05c	0.7916	0.9805	0.101	28.73	52.23	5.949	1.467
SPAC31G5.15	psd3	0.9235	0.9818	0.035	20.15	36.08	12.6	7.162
SPAC823.15	ppa1	0.8228	0.9853	0.085	27.73	50.34	6.583	2.292
SPBC1773.08C	omh4	0.7786	0.9861	0.109	27.43	49.78	4.192	2.504
SPAC3C7.07C	SPAC3C7.07c	0.7829	0.9873	0.106	26.61	48.23	5.075	2.1
SPBC902.04	SPBC902.04	0.5366	0.9901	0.270	23.93	43.18	1.893	1.126
SPBC23E6.01C	SPBC23E6.01c	0.8445	0.9922	0.073	19.25	34.37	5.896	3.687
SPCC1682.15	mug122	0.8976	0.9975	0.047	27.93	50.69	7.586	6.18
SPCC794.09C	ef1a-a	0.9094	0.9984	0.041	29.06	52.82	11.8	5.608
SPBC1703.08C	SPBC1703.08c	0.8693	1.001	0.061	28.18	51.15	9.595	2.654
SPAC1A6.05C	SPAC1A6.05c	0.8581	1.012	0.066	30.69	55.86	8.137	3.244
SPAC869.06C	SPAC869.06c	0.909	1.013	0.041	24.86	44.88	12.01	5.615
SPAC1687.07	SPAC1687.07	0.7686	1.017	0.114	27.24	49.36	4.63	2.202
SPCC736.14	dis1	0.9269	1.023	0.033	21.98	39.44	13.26	8.036
SPBC359.04C	SPBC359.04c	0.6171	1.025	0.210	23.59	42.47	2.569	1.385
SPAC22H12.05C	SPAC22H12.05c	0.6312	1.028	0.200	25.39	45.86	3.5	0.6463
SPBC3B8.03	SPBC3B8.03	0.5956	1.029	0.225	28.34	51.4	2.565	1.229
SPAC17A2.01	bsu1	0.8139	1.031	0.089	26.83	48.56	7.159	1.528
SPBC16C6.08C	qcr6	0.7743	1.032	0.111	28.14	51.02	5.405	1.847
SPBC4B4.04	SPBC4B4.04	0.6655	1.036	0.177	25.35	45.76	3.116	1.568
SPCC622.19	jmj4	0.7271	1.037	0.138	25.79	46.59	3.178	2.263
SPAC2G11.09	SPAC2G11.09	0.8171	1.043	0.088	24.66	44.44	6.496	2.568
SPBC887.06C	snx3	0.8309	1.044	0.080	29.89	54.29	8.157	1.373
SPAC13F5.04C	SPAC13F5.04c	0.8086	1.045	0.092	29.27	53.12	7.005	1.568
SPAC328.09	SPAC328.09	0.6766	1.057	0.170	26.86	48.57	3.117	1.754
SPAC212.08C	SPAC212.08c	0.757	1.057	0.121	25.73	46.44	2.704	2.87
SPBC11C11.10	SPBC11C11.10	0.7635	1.058	0.117	17.06	30.11	4.999	2.038
SPAC23G3.10C	ssr3	0.8106	1.058	0.091	27.1	49.01	3.436	3.733
SPBC12D12.06	srb11	0.7984	1.061	0.098	7.153	11.47	4.573	3.13
SPBC215.04	git11	0.7804	1.064	0.108	23.08	41.43	4.061	2.926
SPBC21D10.10	bdc1	0.6186	1.065	0.209	20.35	36.3	2.779	1.392
SPCC584.02	cuf2	0.7381	1.065	0.132	24.62	44.33	5.261	0.9556
SPBC1861.01C	cnp3	0.9117	1.066	0.040	33.12	60.34	14.18	5.235
SPAC22H10.08	SPAC22H10.08	0.7266	1.067	0.139	25.83	46.61	4.883	1.186
SPAC6G9.09C	rpl2401	0.887	1.067	0.052	31.86	57.96	11.82	3.313
SPAC1F5.08C	yam8	0.8309	1.068	0.080	21.96	39.32	7.935	2.111
SPCC297.03	ssp1	0.8133	1.07	0.090	27.82	50.35	3.741	3.786
SPAC23C4.06C	SPAC23C4.06c	0.909	1.076	0.041	38.15	69.78	15.55	3.062
SPCC132.03	SPCC132.03	0.8495	1.084	0.071	30.11	54.64	9.53	1.633
SPAC824.09C	SPAC824.09c	0.9007	1.091	0.045	17.85	31.54	10.64	6.189
SPBPB2B2.19C	SPBPB2B2.19c	0.9156	1.091	0.038	13.25	22.89	11.67	7.655
SPAC30D11.14C	SPAC30D11.14c	0.718	1.093	0.144	25.59	46.11	2.721	2.462
SPAC9E9.12C	ybt1	0.6507	1.097	0.187	24.33	43.72	2.497	1.867

SPBC776.03	SPBC776.03	0.7985	1.097	0.098	30.06	54.51	7.086	1.363
SPAC12B10.10	SPAC12B10.10	0.7707	1.099	0.113	26.98	48.71	4.977	2.445
SPBC32F12.06	pch1	0.7866	1.1	0.104	28.09	50.79	5.267	2.696
SPAC32A11.01	mug8	0.9164	1.108	0.038	22.46	40.19	13.23	7.273
SPCC594.02C	SPCC594.02c	0.8597	1.109	0.066	12.37	21.2	7.677	4.408
SPAC56E4.06C	ggt2	0.8477	1.11	0.072	21.38	38.15	8.027	3.517
SPAC31G5.03	rps1101	0.5099	1.113	0.293	2.198	2.041	1.195	1.41
SPBC21B10.05C	pop3	0.8019	1.116	0.096	28.31	51.18	5.109	3.266
SPBC2D10.12	rhp23	0.6021	1.119	0.220	19.51	34.62	3.243	1.035
SPCPB1C11.02	SPCPB1C11.02	0.8709	1.121	0.060	31.5	57.19	10.95	2.921
SPAC1F7.10	SPAC1F7.10	0.7977	1.127	0.098	26.76	48.24	7.009	1.792
SPBP35G2.08C	air1	0.5812	1.13	0.236	1.786	1.235	1.007	1.786
SPAC17G8.05	med20	0.9201	1.138	0.036	21.99	39.25	13.81	8.035
SPBC30B4.06C	SPBC30B4.06c	0.6547	1.141	0.184	24.51	43.98	3.488	1.561
SPCC18.17C	SPCC18.17c	0.6961	1.143	0.157	30.23	54.74	4.873	0.7423
SPAC3C7.14C	obr1	0.8433	1.143	0.074	31.48	57.1	9.5	1.954
SPAC26H5.07C	SPAC26H5.07c	0.8937	1.147	0.049	15.86	27.69	9.708	6.39
SPAC30.02C	SPAC30.02c	0.8868	1.149	0.052	23.29	41.67	9.798	5.715
SPBC14C8.03	fma2	0.8613	1.15	0.065	26.73	48.15	9.804	3.478
SPBC146.06C	SPBC146.06c	0.7057	1.151	0.151	27.36	49.33	4.166	1.877
SPCC74.04	SPCC74.04	0.8986	1.155	0.046	18.38	32.41	11.46	6.2
SPCC1682.01	qcr9	0.8599	1.159	0.066	8.253	13.35	7.708	4.773
SPCC622.12C	gdh1	0.9085	1.166	0.042	19.32	34.18	12.56	7.078
SPAC17G8.09	shg1	0.5949	1.167	0.226	19.58	34.66	2.627	1.539
SPAC22A12.16	SPAC22A12.16	0.7517	1.167	0.124	28.46	51.37	5.47	1.953
SPBC56F2.05C	SPBC56F2.05c	0.882	1.167	0.055	12.27	20.9	9.284	5.689
SPCC1682.12C	ubp16	0.7188	1.171	0.143	27.22	49.03	4.889	1.647
SPAC1071.07C	rps1502	0.8338	1.177	0.079	11.82	20.03	6.851	3.944
SPAC20H4.02	dsc3	0.8368	1.179	0.077	7.9	12.65	7.399	3.816
SPCC553.04	cyp9	0.8629	1.184	0.064	16.18	28.22	8.662	4.689
SPBC3H7.09	erf2	0.8946	1.187	0.048	18.14	31.92	11	6.286
SPBC365.20C	pnc1	0.8692	1.19	0.061	26.66	47.94	11.29	3.29
SPBC36.11	SPBC36.11	0.6223	1.195	0.206	25.16	45.1	1.23	2.122
SPAC823.10C	SPAC823.10c	0.826	1.195	0.083	27.06	48.69	8.756	2.106
SPAC11H11.03C	SPAC11H11.03c	0.8349	1.196	0.078	20.42	36.18	6.344	4.32
SPAPB1A10.14	SPAPB1A10.14	0.9004	1.199	0.046	14.7	25.41	11.29	6.952
SPAC140.03	arb1	0.8335	1.205	0.079	28.08	50.58	7.705	3.647
SPBP19A11.02C	SPBP19A11.02c	0.7887	1.206	0.103	28.82	51.97	7.606	0.9772
SPAC750.08C	SPAC750.08c	0.7057	1.207	0.151	20.94	37.14	3.115	2.53
SPCC364.01	cif1	0.7991	1.21	0.097	27.47	49.43	7.121	2.465
SPAC11E3.09	pyp3	0.8162	1.21	0.088	27.84	50.12	7.862	2.645
SPAC4D7.11	dsc4	0.7893	1.211	0.103	9.485	15.57	5.369	3.257
SPAC57A10.03	cyp1	0.7971	1.218	0.098	29.48	53.19	7.772	1.586
SPAC16A10.07C	taz1	0.7361	1.222	0.133	29.62	53.45	4.731	2.378
SPAC1006.01	psp3	0.7979	1.225	0.098	26.98	48.47	4.118	3.953
SPAC186.06	SPAC186.06	0.71	1.227	0.149	29.43	53.09	4.984	1.645
SPAC1782.05	SPAC1782.05	0.7826	1.23	0.106	27.15	48.79	6.697	2.289
SPAC29E6.09	SPAC29E6.09	0.6744	1.232	0.171	28.52	51.35	4.573	1.281
SPBC16D10.01C	SPBC16D10.01c	0.7791	1.234	0.108	28.16	50.69	7.336	1.206
SPCC613.12C	raf1	0.8587	1.235	0.066	22.75	40.49	10.27	3.735
SPBC16G5.15C	fkh2	0.5376	1.236	0.270	18.27	32.07	1.249	1.707
SPAC1805.12C	uep1	0.8614	1.237	0.065	31.18	56.35	10.71	3.607
SPBC530.10C	anc1	0.47	1.24	0.328	23.74	42.36	1.577	1.348
SPBC56F2.02	rpl1901	0.7287	1.24	0.137	5.449	7.921	3.577	2.807
SPAC17H9.10C	ddb1	0.8184	1.24	0.087	17.63	30.86	7.464	3.302
SPBC29A10.07	pom152	0.7319	1.241	0.136	29.57	53.31	5.891	1.258
SPBC776.17	SPBC776.17	0.8475	1.241	0.072	13.48	23.04	7.782	4.589
SPBC725.05C	SPBC725.05c	0.7105	1.247	0.148	26.83	48.15	3.775	2.489
SPCC18.02	SPCC18.02	0.5815	1.248	0.235	24.12	43.04	1.85	1.861
SPAC25B8.10	SPAC25B8.10	0.7871	1.249	0.104	28.43	51.15	7.494	1.701
SPCC550.11	SPCC550.11	0.5292	1.253	0.276	3.694	4.594	2.367	1.392
SPAC1D4.09C	rtf2	0.7488	1.257	0.126	21.33	37.79	3.968	3.074
SPAC15A10.06	SPAC15A10.06	0.649	1.26	0.188	3.184	3.621	2.452	2.248

SPAC9G1.06C	cyk3	0.4809	1.262	0.318	31.67	57.23	2.784	0.6972
SPCC61.05	SPCC61.05	0.5599	1.263	0.252	17.95	31.41	1.609	1.809
SPCC1322.07C	mug150	0.6633	1.263	0.178	26.93	48.31	3.938	1.793
SPAC3H8.05C	mms1	0.8732	1.264	0.059	15.71	27.2	9.649	5.598
SPBC29A3.08	pof4	0.8096	1.27	0.092	28.49	51.23	8.005	2.632
SPBC32H8.09	SPBC32H8.09	0.8339	1.27	0.079	12.73	21.58	7.402	4.257
SPBC2G2.14	SPBC2G2.14	0.6993	1.272	0.155	27.2	48.79	3.32	2.573
SPAC22F3.12C	rgs1	0.9056	1.273	0.043	21.62	38.3	13.18	7.534
SPAC222.07C	hri2	0.6993	1.276	0.155	24.26	43.25	5.309	1.233
SPCC1442.01	ste6	0.9009	1.276	0.045	21.13	37.36	12.53	7.22
SPAC1142.05	ctr5	0.7308	1.279	0.136	4.787	6.602	3.909	2.856
SPAC7D4.08	SPAC7D4.08	0.7838	1.279	0.106	24.74	44.15	6.718	2.642
SPAC222.05C	mss1	0.6343	1.281	0.198	27.97	50.23	3.838	1.526
SPBC23E6.03C	nta1	0.72	1.285	0.143	25.22	45.05	5.549	1.646
SPAPB1E7.11C	SPAPB1E7.11c	0.8503	1.286	0.070	9.574	15.6	7.989	5.163
SPAC26H5.02C	SPAC26H5.02c	0.807	1.287	0.093	29.02	52.19	7.906	2.722
SPBC32F12.12C	SPBC32F12.12c	0.8264	1.288	0.083	33.16	60	9.784	1.692
SPAC26F1.09	gyp51	0.5111	1.29	0.291	26.12	46.73	3.07	0.7611
SPAC1952.06C	SPAC1952.06c	0.7181	1.295	0.144	24.28	43.26	5.919	1.07
SPAC4F10.18	nup37	0.9105	1.299	0.041	23.44	41.67	14.52	7.949
SPAC17A2.05	osm1	0.7005	1.301	0.155	23.2	41.22	2.917	2.778
SPBC543.09	yta12	0.6443	1.302	0.191	27.38	49.08	4.128	1.51
SPBC336.13C	SPBC336.13c	0.7562	1.309	0.121	9.116	14.69	4.963	3.039
SPAC1296.04	mug65	0.8249	1.31	0.084	25.87	46.23	8.376	3.486
SPCP1E11.07C	cwf18	0.6239	1.315	0.205	18.63	32.59	3.444	1.77
SPCC364.05	vps3	0.8824	1.315	0.054	17.66	30.77	10.48	6.442
SPACUNK4.19	mug153	0.6927	1.316	0.159	27.99	50.21	4.999	1.686
SPBPB10D8.04C	SPBPB10D8.04c	0.682	1.319	0.166	28.33	50.84	4.838	1.619
SPAC18G6.12C	SPAC18G6.12c	0.8045	1.322	0.094	26.12	46.67	7.97	2.8
SPBC1773.17C	SPBC1773.17c	0.7189	1.328	0.143	29.89	53.76	5.507	1.906
SPAC521.02	SPAC521.02	0.7803	1.328	0.108	22.2	39.29	7.205	2.389
SPBC1709.13C	set10	0.8883	1.328	0.051	22.53	39.91	13.07	5.816
SPBC409.06	uch2	0.7525	1.329	0.123	26.29	46.98	6.577	1.883
SPCC306.09C	cap1	0.6031	1.332	0.220	15.98	27.57	1.713	2.182
SPBC3D6.06C	prs5	0.7297	1.333	0.137	24.72	44.01	6.407	1.073
SPCC1827.02C	SPCC1827.02c	0.7865	1.35	0.104	10.37	16.98	5.999	3.538
SPCC576.02	SPCC576.02	0.6477	1.352	0.189	28.45	50.99	4.031	1.817
SPAC11D3.13	SPAC11D3.13	0.8487	1.353	0.071	20.46	35.96	8.457	5.084
SPBC1347.07	rex2	0.6912	1.356	0.160	22.9	40.55	2.929	2.806
SPAC4F10.17	SPAC4F10.17	0.6752	1.358	0.171	29.03	52.09	4.845	1.65
SPBC1703.14C	top1	0.7018	1.359	0.154	34.55	62.47	5.632	1.441
SPBC16D10.02	trm11	0.06715	1.361	1.173	25.42	45.28	0.8096	0.4324
SPAC25A8.02	SPAC25A8.02	0.6057	1.363	0.218	28.44	50.97	3.823	1.438
SPAC29B12.08	SPAC29B12.08	0.8623	1.366	0.064	22.87	40.47	11.29	4.539
SPBPB10D8.06C	SPBPB10D8.06c	0.7612	1.371	0.119	30.37	54.57	3.919	3.713
SPCC1827.07C	SPCC1827.07c	0.8538	1.371	0.069	32.63	58.84	11.69	3.295
SPAC8F11.05C	mug130	0.7755	1.376	0.110	28.07	50.24	7.515	2.188
SPAC644.08	SPAC644.08	0.8115	1.379	0.091	29.5	52.93	9.097	2.527
SPCC1259.04	iec3	0.7557	1.382	0.122	28.83	51.67	7.076	1.8
SPAC1071.09C	SPAC1071.09c	0.6823	1.387	0.166	28.82	51.63	5.335	1.42
SPAC8C9.14	prr1	0.8855	1.391	0.053	20.03	35.08	11.85	6.787
SPAC806.08C	mod21	0.7836	1.393	0.106	26.93	48.07	7.78	2.434
SPCC330.02	rhp7	0.6115	1.395	0.214	26.33	46.93	4.496	0.7896
SPCC965.14C	SPCC965.14c	0.7628	1.397	0.118	29.16	52.26	6.396	2.818
SPAC1A6.10	SPAC1A6.10	0.7432	1.401	0.129	23.75	42.07	5.638	2.772
SPBC1604.08C	imp1	0.2969	1.402	0.527	22.32	39.36	1.867	0.7017
SPAC17A2.13C	rad25	0.7007	1.405	0.154	27.09	48.35	5.395	1.94
SPBC1539.04	tts1	0.7046	1.405	0.152	26.2	46.67	5.464	1.971
SPAC4A8.06C	SPAC4A8.06c	0.7759	1.406	0.110	26.95	48.09	7.022	2.867
SPCC162.04C	wtf13	0.6792	1.408	0.168	27.75	49.59	3.96	2.461
SPAP27G11.02	SPAP27G11.02	0.6388	1.409	0.195	26.6	47.42	3.724	2.052
SPAC22E12.06C	gmh3	0.7003	1.412	0.155	27.94	49.93	5.74	1.594
SPCC1682.13	laf2	0.6455	1.413	0.190	27.19	48.52	4.625	1.521

SPCC576.11	rpl15	0.8425	1.415	0.074	9.588	15.38	7.358	5.545
SPAC637.03	SPAC637.03	0.8674	1.415	0.062	32.73	58.94	11.8	5.145
SPBC365.02C	cox10	0.3091	1.417	0.510	24.73	43.89	0.7627	1.136
SPBC18A7.01	SPBC18A7.01	0.6975	1.419	0.156	28.3	50.6	5.428	1.901
SPBC646.17C	dic1	0.766	1.421	0.116	27.94	49.91	6.753	2.795
SPAC23G3.04	ies4	0.7449	1.423	0.128	32.91	59.26	6.651	2.147
SPBC16C6.06	vps10	0.6716	1.424	0.173	23.1	40.8	5.512	1.022
SPBC1683.09C	frp1	0.4073	1.432	0.390	15.3	26.11	2.029	1.191
SPAC1751.04	SPAC1751.04	0.8133	1.435	0.090	30.58	54.85	9.288	2.968
SPCC1795.09	yps1	0.6793	1.438	0.168	25.96	46.16	2.491	2.957
SPCC1450.11C	cek1	0.7381	1.446	0.132	25.17	44.64	6.818	1.839
SPCC338.07C	naa15	0.6258	1.448	0.204	25.86	45.94	3.484	2.122
SPAC4G9.13C	vps26	0.7045	1.448	0.152	12.37	20.56	4.621	2.819
SPBC23E6.02	SPBC23E6.02	0.7299	1.449	0.137	26.29	46.75	5.404	2.793
SPAC8C9.17C	spc34	0.8714	1.45	0.060	21.32	37.4	11.8	5.864
SPAPB2B4.03	cig2	0.5772	1.452	0.239	27	48.09	3.872	1.311
SPBC1348.07	SPBC1348.07	0.6994	1.452	0.155	29.52	52.83	4.333	2.74
SPAC513.03	mfm2	0.7294	1.461	0.137	21.59	37.88	2.515	3.686
SPCC4B3.07	nro1	0.6366	1.464	0.196	27.41	48.83	4.953	1.157
SPBC119.12	SPBC119.12	0.7468	1.467	0.127	24.11	42.61	5.624	3.147
SPCC825.05C	SPCC825.05c	0.7708	1.469	0.113	14.42	24.38	7.112	2.971
SPCC1906.04	wtf20	0.728	1.47	0.138	26.38	46.88	3.292	3.555
SPAP7G5.03	prm1	0.7984	1.471	0.098	23.48	41.42	7.26	3.934
SPAC15A10.16	bud6	0.8355	1.471	0.078	22	38.64	8.852	5.116
SPAC630.09C	mug58	0.7065	1.474	0.151	26.44	46.99	6.357	1.354
SPAC6C3.05	SPAC6C3.05	0.7407	1.481	0.130	27.82	49.58	6.149	2.746
SPBC1773.04	SPBC1773.04	0.616	1.482	0.210	27.51	48.99	3.512	2.089
SPCC1223.03C	gut2	0.6272	1.487	0.203	27.24	48.47	4.572	1.548
SPCC126.07C	SPCC126.07c	0.683	1.487	0.166	27.59	49.13	4.013	2.722
SPAC20G8.07C	erg2	0.8106	1.488	0.091	32.31	58.01	10.15	2.161
SPBC21B10.02	SPBC21B10.02	0.5868	1.489	0.232	29.44	52.6	3.939	1.509
SPCC338.04	cid2	0.6697	1.489	0.174	28.61	51.05	5.1	1.888
SPAC19D5.03	cid1	0.7086	1.489	0.150	26.14	46.41	5.957	2.04
SPAC1687.22C	puf3	0.4483	1.494	0.348	23.05	40.58	1.806	1.543
SPBC17D1.07C	SPBC17D1.07c	0.7482	1.496	0.126	27.54	49.02	3.444	3.963
SPCC1919.05	SPCC1919.05	0.8696	1.496	0.061	17.22	29.6	11.02	6.472
SPAC1093.01	ppr5	0.7443	1.497	0.128	25.43	45.05	4.375	3.67
SPAC3A12.03C	meu34	0.6954	1.502	0.158	24.8	43.86	5.084	2.462
SPAC3G9.11C	SPAC3G9.11c	0.8866	1.502	0.052	20.38	35.52	13.48	7.12
SPAC1B3.11C	ypt4	0.3974	1.506	0.401	25.35	44.88	2.307	1.106
SPAC6F6.13C	SPAC6F6.13c	0.7117	1.509	0.148	30.74	55.01	6.427	1.738
SPAC1071.12C	stp1	0.6023	1.511	0.220	27.16	48.27	3.465	2.038
SPAC3H8.10	spo20	0.7083	1.512	0.150	30.51	54.59	6.092	2.024
SPCC297.04C	set7	0.7609	1.514	0.119	29.37	52.43	7.489	2.528
SPBC119.05C	SPBC119.05c	0.423	1.515	0.374	25.43	45.02	1.935	1.416
SPBPB21E7.01C	eno102	0.7629	1.515	0.118	30.95	55.41	7.81	2.283
SPAC19B12.12C	yip11	0.6385	1.519	0.195	27.31	48.55	5.166	1.209
SPBC649.03	rhp14	0.7336	1.519	0.135	7.608	11.46	5.466	3.114
SPAC4G9.12	SPAC4G9.12	0.7684	1.52	0.114	30.12	53.83	7.854	2.542
SPBC15D4.13C	SPBC15D4.13c	0.7399	1.521	0.131	29.12	51.95	5.945	3.023
SPAC56F8.16	esc1	0.6224	1.522	0.206	28.68	51.12	4.232	1.875
SPAP7G5.06	per1	0.6475	1.522	0.189	27.92	49.68	2.894	2.715
SPAC6G9.05	pcd1	0.708	1.525	0.150	27.84	49.52	4.635	2.992
SPBC582.08	SPBC582.08	0.7231	1.526	0.141	31.03	55.53	6.565	2.092
SPBC31F10.15C	atp15	0.702	1.527	0.154	16.28	27.78	4.947	2.894
SPAPB8E5.06C	rpl302	0.7496	1.532	0.125	25	44.16	6.735	2.85
SPBC11G11.01	fis1	0.8568	1.534	0.067	19.3	33.43	10.44	5.961
SPBC3B8.07C	dsd1	0.7483	1.535	0.126	25.26	44.66	3.862	4
SPAC2F3.08	sut1	0.7721	1.536	0.112	30.54	54.59	7.98	2.701
SPBC18E5.07	SPBC18E5.07	0.7898	1.538	0.102	30.68	54.85	8.917	2.697
SPAC17G8.08C	SPAC17G8.08c	0.672	1.54	0.173	30.87	55.21	5.667	1.598
SPBP16F5.07	apm1	0.7311	1.54	0.136	22.22	38.93	3.038	3.855
SPAC23H4.01C	SPAC23H4.01c	0.6817	1.542	0.166	26.99	47.89	3.292	3.072

SPCC4F11.03C	SPCC4F11.03c	0.7534	1.546	0.123	28.39	50.52	8.214	1.291
SPBC646.02	cwf11	0.6545	1.547	0.184	25.83	45.7	3.591	2.657
SPBC691.03C	apl3	0.7328	1.552	0.135	27.42	48.69	2.25	4.026
SPAC23H4.02	ppk9	0.7221	1.554	0.141	27.51	48.85	7.038	1.626
SPAC1687.16C	erg31	0.67	1.555	0.174	29.53	52.65	5.102	2.155
SPBC27B12.04C	SPBC27B12.04c	0.7258	1.555	0.139	28.95	51.56	7.346	1.255
SPAC3F10.06C	SPAC3F10.06c	0.7214	1.556	0.142	28.09	49.94	3.693	3.605
SPAC4G9.19	SPAC4G9.19	0.7608	1.557	0.119	27.12	48.11	7.742	2.552
SPAC57A10.09C	nhp6	0.7879	1.557	0.104	33.89	60.85	8.978	2.67
SPCC569.04	SPCC569.04	0.5887	1.56	0.230	28.51	50.72	4.65	0.9584
SPAC18G6.13	SPAC18G6.13	0.4609	1.561	0.336	22.33	39.09	2.214	1.581
SPAC4C5.01	SPAC4C5.01	0.7602	1.562	0.119	23.78	41.82	8.048	2.22
SPBC21.07C	ppk24	0.6804	1.565	0.167	26.95	47.77	5.306	2.254
SPBC947.10	dsc1	0.7847	1.565	0.105	12.3	20.2	6.814	4.104
SPAC227.05	SPAC227.05	0.1276	1.566	0.894	32.08	57.43	1.099	0.6637
SPAC10F6.11C	atg17	0.6541	1.569	0.184	26.83	47.55	5.433	1.556
SPAC4G8.07C	SPAC4G8.07c	0.5607	1.571	0.251	24.12	42.44	2.749	2.072
SPBPB2B2.06C	SPBPB2B2.06c	0.5611	1.575	0.251	27.34	48.5	3.699	1.635
SPBC902.05C	idh2	0.6537	1.577	0.185	26.28	46.49	4.469	2.352
SPCC162.05	coq3	0.79	1.58	0.102	8.005	12.09	6.1	4.622
SPAC688.10	rev3	0.6361	1.581	0.196	27.03	47.9	5.152	1.511
SPAC12B10.13	SPAC12B10.13	0.5166	1.582	0.287	26.9	47.66	3.926	0.7334
SPBP4H10.09	rsv1	0.8839	1.582	0.054	21.49	37.48	13.3	7.601
SPAC8E11.07C	alp31	0.8441	1.583	0.074	18.9	32.59	8.099	6.342
SPBC354.13	rga6	0.7454	1.589	0.128	29.88	53.24	4.37	3.995
SPBC36.07	iki3	0.6694	1.594	0.174	19.16	33.06	1.762	3.296
SPBC8D2.01	gsk31	0.5505	1.597	0.259	26.56	46.99	4.067	1.233
SPAPYUG7.06	mug67	0.8912	1.597	0.050	22.72	39.76	14.18	8.263
SPCC4G3.11	mug154	0.4774	1.6	0.321	22.76	39.83	2.299	1.706
SPAC17D4.04	SPAC17D4.04	0.6675	1.6	0.176	25.22	44.46	5.346	2.093
SPAC1002.07C	ats1	0.5219	1.601	0.282	26.33	46.54	3.568	1.374
SPAC4A8.14	SPAC4A8.14	0.8403	1.601	0.076	22.08	38.55	10.66	5.07
SPCC285.17	spp27	0.6	1.602	0.222	23.48	41.18	2.033	2.602
SPBC23G7.16	ctr6	0.3768	1.605	0.424	20.4	35.37	1.536	1.431
SPBP4H10.17C	SPBP4H10.17c	0.5517	1.605	0.258	27.24	48.25	3.221	1.883
SPBC2A9.03	SPBC2A9.03	0.6317	1.605	0.199	29.16	51.86	4.664	1.979
SPAC6F6.06C	rax2	0.6411	1.607	0.193	24.67	43.4	4.662	2.138
SPBC25B2.03	SPBC25B2.03	0.797	1.608	0.099	30.99	55.29	9.615	2.981
SPAC27D7.11C	SPAC27D7.11c	0.842	1.612	0.075	22.36	39.05	9.033	6.058
SPAC27D7.04	omt2	0.5978	1.613	0.223	24.78	43.61	4.496	1.597
SPAC23G3.07C	snf30	0.6788	1.613	0.168	29.26	52.03	6.051	1.736
SPAPJ696.01C	vps17	0.8111	1.614	0.091	14.3	23.88	8.158	4.79
SPAC16C9.02C	SPAC16C9.02c	0.6237	1.615	0.205	27.36	48.45	5.425	0.8739
SPBC21.05C	ral2	0.7561	1.615	0.121	22.89	40.05	6.896	3.544
SPAC11E3.01C	swr1	0.5394	1.619	0.268	30.34	54.05	2.785	1.986
SPAC31A2.16	gef2	0.5471	1.619	0.262	23.96	42.04	2.487	2.133
SPAPB2B4.02	grx5	0.8496	1.62	0.071	21.71	37.81	11.88	5.186
SPCC1494.08C	SPCC1494.08c	0.6313	1.621	0.200	19.98	34.55	4.177	2.316
SPBC16E9.17C	rem1	0.6552	1.623	0.184	28.96	51.45	6.109	0.6487
SPBC428.15	SPBC428.15	0.6436	1.626	0.191	28.36	50.32	5.203	1.824
SPAC22A12.07C	ogm1	0.8178	1.626	0.087	28.45	50.49	7.44	5.443
SPBC15C4.05	SPBC15C4.05	0.7738	1.629	0.111	29.86	53.14	8.936	2.435
SPAC2G11.05C	SPAC2G11.05c	0.8036	1.629	0.095	30.53	54.39	10.32	2.833
SPAC56F8.05C	mug64	0.6557	1.63	0.183	25.02	44.02	6.157	0.6063
SPAC1A6.07	SPAC1A6.07	0.5694	1.632	0.245	25.6	45.11	3.243	2.087
SPBC36.04	cys11	0.6256	1.632	0.204	28.25	50.11	3.989	2.361
SPBC8D2.04	hht2	0.8818	1.638	0.055	23.66	41.45	14.1	7.439
SPBC1604.12	SPBC1604.12	0.6451	1.639	0.190	29.27	52.01	5.169	1.94
SPAC4G8.08	SPAC4G8.08	0.6914	1.639	0.160	28.22	50.04	5.664	2.525
SPBC1289.09	tim21	0.7557	1.647	0.122	28.02	49.65	8.062	2.594
SPCC18.01C	adg3	0.5594	1.651	0.252	32.35	57.78	3.392	1.971
SPBC947.09	SPBC947.09	0.6686	1.653	0.175	30.74	54.74	5.145	2.453
SPAC3H5.12C	rpl501	0.8014	1.654	0.096	13.74	22.75	7.662	4.784

SPAC328.03	tps1	0.6182	1.656	0.209	6.45	9.023	3.515	2.523
SPAC25A8.01C	fft3	0.4903	1.662	0.310	22.25	38.75	3.39	1.351
SPBC1652.01	SPBC1652.01	0.3668	1.664	0.436	26.26	46.3	2.542	1.023
SPAC17A5.04C	mde10	0.7231	1.665	0.141	19.87	34.26	6.032	3.134
SPBC1105.13C	SPBC1105.13c	0.6116	1.666	0.214	26.91	47.51	4.078	2.233
SPAC23H3.11C	SPAC23H3.11c	0.5993	1.667	0.222	28.41	50.33	4.408	1.872
SPBC2F12.04	rpl1701	0.6932	1.674	0.159	29.25	51.9	6.399	2.115
SPAC19G12.04	SPAC19G12.04	0.6289	1.676	0.201	23.21	40.53	2.444	2.941
SPBC32H8.06	mug93	0.7319	1.678	0.136	26.27	46.28	7.512	2.329
SPAC869.04	SPAC869.04	0.7483	1.679	0.126	27.58	48.75	2.505	4.647
SPBC660.09	mug168	0.7051	1.68	0.152	27.08	47.81	4.135	3.568
SPAC4G8.03C	SPAC4G8.03c	0.5793	1.682	0.237	31.51	56.14	4.539	1.499
SPCC1322.03	SPCC1322.03	0.8098	1.691	0.092	20.82	36.01	10.24	3.896
SPAC14C4.04	B22918-2	0.4897	1.695	0.310	25.58	44.95	2.908	1.707
SPBC1A4.02C	leu1	0.7704	1.696	0.113	31.51	56.12	9.238	2.394
SPCC16C4.01	sif2	0.5715	1.697	0.243	24.04	42.05	2.211	2.51
SPAPB24D3.07C	SPAPB24D3.07c	0.8705	1.697	0.060	20.99	36.3	12.98	7.211
SPAC1F3.07C	rsc58	0.7852	1.708	0.105	18.36	31.34	7.806	4.537
SPBC6B1.08C	ofd1	0.5942	1.71	0.226	25.34	44.48	2.025	2.748
SPBC32F12.01C	css1	0.8462	1.711	0.073	18.3	31.22	10.57	6.306
SPBC1685.06	cid11	0.7872	1.712	0.104	31.31	55.7	9.686	3.096
SPCC4G3.10C	rhp42	0.6612	1.714	0.180	30.36	53.91	5.304	2.421
SPAC2E12.03C	SPAC2E12.03c	0.6125	1.717	0.213	29.89	53.02	5.224	1.525
SPAC13D6.03C	trm9	0.5353	1.72	0.271	28.06	49.58	4.157	1.339
SPCC74.09	mug24	0.6717	1.723	0.173	24.97	43.75	5.45	2.561
SPAC13G6.09	SPAC13G6.09	0.6625	1.725	0.179	10.81	17.09	4.402	3.073
SPBC3D6.15	rps2501	0.7969	1.727	0.099	22.94	39.92	8.867	4.6
SPCC4G3.02	aph1	0.4066	1.732	0.391	12.35	19.98	2.036	1.589
SPAC1556.05C	cgr1	0.682	1.735	0.166	22.45	38.99	4.827	3.101
SPAC1687.05	pli1	0.5576	1.74	0.254	28.03	49.47	4.045	1.792
SPBC3B9.09	vps36	0.7421	1.74	0.130	29	51.31	6.733	3.56
SPAC16C9.06C	upf1	0.7735	1.742	0.112	26.05	45.76	4.369	5.175
SPAC1296.06	tah18	0.8055	1.744	0.094	10.31	16.13	8.27	5.377
SPBC106.02C	srx1	0.5791	1.746	0.237	24.94	43.65	4.612	1.652
SPCC4F11.04C	imt2	0.8657	1.749	0.063	13.73	22.55	11.06	7.934
SPAC23C4.09C	SPAC23C4.09c	0.4393	1.753	0.357	35.81	64.1	3.206	1.232
SPAC22F3.09C	res2	0.6777	1.757	0.169	13.47	22.04	5.133	3.111
SPBC3B9.06C	atg3	0.3788	1.758	0.422	24.35	42.52	1.715	1.569
SPAC1D4.06C	csk1	0.8222	1.759	0.085	22.69	39.4	9.719	5.428
SPBC16A3.19	eaf7	0.5154	1.761	0.288	28.62	50.55	3.711	1.604
SPAC30D11.13	hus5	0.6322	1.766	0.199	3.134	2.576	2.576	3.134
SPCC70.04C	SPCC70.04c	0.4091	1.768	0.388	29.9	52.95	1.567	1.745
SPAC22A12.11	dak1	0.7011	1.768	0.154	27.41	48.25	6.77	2.47
SPBC582.10C	SPBC582.10c	0.5464	1.778	0.262	26.78	47.06	2.787	2.32
SPAC19A8.01C	sec73	0.448	1.779	0.349	23.27	40.44	3.518	1.068
SPAC1952.08C	SPAC1952.08c	0.4961	1.779	0.304	26.89	47.26	2.098	2.132
SPAC589.12	SPAC589.12	0.8125	1.78	0.090	12.17	19.56	9.483	5.369
SPAC25H1.09	mde5	0.4547	1.782	0.342	26.99	47.45	3.877	0.4337
SPAC19D5.02C	SPAC19D5.02c	0.6395	1.784	0.194	27.16	47.77	5.799	1.812
SPBC543.08	SPBC543.08	0.5596	1.785	0.252	28.3	49.9	4.874	1.09
SPBC409.10	ade7	0.5851	1.785	0.233	23.82	41.47	3.958	2.271
SPAC3A11.06	mvp1	0.5911	1.789	0.228	26.35	46.23	5.391	1.067
SPBC26H8.03	cho2	0.8575	1.79	0.067	23.3	40.48	14.06	5.912
SPCC23B6.05C	ssb3	0.5778	1.795	0.238	25.51	44.63	4.694	1.718
SPAC644.06C	cdr1	0.6522	1.798	0.186	8.798	13.17	4.407	2.971
SPAC1610.03C	crp79	0.6383	1.803	0.195	29.98	53.02	5.561	2.098
SPCP1E11.11	puf6	0.5824	1.805	0.235	30.3	53.63	3.885	2.319
SPCC1322.12C	bub1	0.7964	1.807	0.099	26.82	47.09	10.41	3.698
SPCC1672.04C	SPCC1672.04c	0.05289	1.809	1.277	17.29	29.14	1.113	0.4403
SPAC16.05C	sfp1	0.04853	1.812	1.314	22.22	38.4	0.5013	0.5981
SPBC24C6.04	SPBC24C6.04	0.6815	1.813	0.167	19.37	33.04	4.523	3.423
SPBC337.15C	coq7	0.7292	1.817	0.137	7.961	11.56	7.622	2.907
SPCC1235.09	hif2	0.6853	1.818	0.164	26.6	46.64	6.57	2.417

SPAC25B8.06C	SPAC25B8.06c	0.7497	1.828	0.125	7.018	9.77	6.412	4.456
SPAC521.04C	SPAC521.04c	0.7593	1.828	0.120	28.09	49.44	5.781	4.75
SPAC15A10.05C	mug182	0.8579	1.831	0.067	21.56	37.13	11.35	7.693
SPAC4F8.11	SPAC4F8.11	0.6047	1.833	0.218	26.08	45.64	4.668	2.26
SPBC3B9.05	SPBC3B9.05	0.6102	1.833	0.215	23.17	40.17	2.866	2.98
SPCC126.06	twf1	0.5836	1.839	0.234	22.5	38.88	3.049	2.686
SPBC3E7.06C	fnx2	0.6768	1.839	0.170	29.14	51.39	4.052	3.554
SPCC736.06	SPCC736.06	0.7802	1.847	0.108	14.12	23.1	7.745	4.798
SPAC6G10.06	SPAC6G10.06	0.329	1.85	0.483	29.11	51.31	2.606	1.036
SPBC8D2.19	mde3	0.6563	1.851	0.183	24.14	41.96	5.526	2.648
SPAC22G7.11C	SPAC22G7.11c	0.8518	1.853	0.070	20.06	34.28	12	7.038
SPAC57A7.09	SPAC57A7.09	0.539	1.854	0.268	29.05	51.19	3.598	2.107
SPAC139.03	SPAC139.03	0.6387	1.855	0.195	30.03	53.03	4.359	2.939
SPCC1259.08	SPCC1259.08	0.7829	1.856	0.106	30.21	53.37	9.331	4.096
SPBC15D4.10C	amo1	0.8371	1.856	0.077	12.94	20.85	7.635	7.505
SPAC57A10.12C	ura3	0.8192	1.857	0.087	15.66	25.98	9.917	5.98
SPAC1F7.11C	SPAC1F7.11c	0.6236	1.859	0.205	28.6	50.33	3.545	3.019
SPAC3A11.07	SPAC3A11.07	0.8207	1.859	0.086	16.83	28.17	10.06	5.752
SPAC3A11.11C	SPAC3A11.11c	0.8405	1.859	0.075	21.05	36.13	11.39	6.453
SPBC16G5.11C	bag101	0.6614	1.866	0.180	27.3	47.88	6.841	1.567
SPBC17G9.02C	SPBC17G9.02c	0.5831	1.879	0.234	30.23	53.36	5.394	1.35
SPBC21.02	SPBC21.02	0.7331	1.88	0.135	11.17	17.49	5.846	4.222
SPAC589.03C	SPAC589.03c	0.5953	1.881	0.225	30.22	53.34	5.769	1.063
SPAC1093.03	SPAC1093.03	0.6307	1.881	0.200	24.95	43.41	5.392	2.355
SPCC790.03	SPCC790.03	0.717	1.884	0.144	11	17.15	6.195	3.703
SPAC8F11.02C	dph3	0.5308	1.885	0.275	24.74	43.01	4.261	1.684
SPAPB1A10.07C	SPAPB1A10.07c	0.7682	1.892	0.115	28.05	49.23	9.91	3.006
SPBC651.12C	SPBC651.12c	0.5893	1.893	0.230	25.94	45.26	4.407	2.355
SPBC2G2.06C	apl1	0.242	1.895	0.616	36.26	64.69	1.776	1.109
SPAC17A5.09C	SPAC17A5.09c	0.6113	1.899	0.214	28.18	49.46	5.296	2.133
SPBC1921.04C	SPBC1921.04c	0.5727	1.901	0.242	28.23	49.56	3.386	2.603
SPAC13A11.01C	rga8	0.7535	1.903	0.123	28.52	50.1	7.501	4.199
SPAC27E2.01	SPAC27E2.01	0.4974	1.904	0.303	27.42	48.02	3.645	1.788
SPAC688.12C	SPAC688.12c	0.4688	1.909	0.329	29.74	52.38	4.076	1.048
SPBC18H10.02	lcf1	0.6643	1.912	0.178	16.77	27.97	5.535	3.155
SPBC21B10.13C	yox1	0.5635	1.915	0.249	14.1	22.93	3.666	2.443
SPCC613.11C	meu23	0.4751	1.916	0.323	26.68	46.61	4.313	0.7402
SPAC3G6.02	dss1	0.8565	1.918	0.067	21.38	36.63	13.18	7.358
SPBC1198.07C	SPBC1198.07c	0.6061	1.925	0.217	28.54	50.09	3.278	3.026
SPBC1105.05	exg1	0.6771	1.928	0.169	29.05	51.05	5.192	3.426
SPAC212.03	SPAC212.03	0.69	1.938	0.161	25.02	43.44	6.042	3.334
SPBPB10D8.05C	SPBPB10D8.05c	0.6229	1.943	0.206	29.54	51.94	4.483	2.888
SPCC11E10.09C	SPCC11E10.09c	0.6918	1.944	0.160	27.11	47.36	4.542	3.946
SPAC12B10.04	SPAC12B10.04	0.825	1.945	0.084	24.41	42.28	11.05	6.038
SPBC1A4.09	SPBC1A4.09	0.7446	1.946	0.128	7.748	10.92	6.254	4.806
SPAC56F8.02	SPAC56F8.02	0.4365	1.947	0.360	23.67	40.88	3.62	1.282
SPAC1071.11	SPAC1071.11	0.7278	1.958	0.138	15.47	25.43	7.698	3.468
SPAC11D3.05	mfs2	0.6338	1.961	0.198	26.78	46.71	5.734	2.434
SPCC5E4.10C	SPCC5E4.10c	0.6151	1.964	0.211	25.91	45.07	5.183	2.475
SPCC330.07C	SPCC330.07c	0.6474	1.964	0.189	29.82	52.42	4.767	3.188
SPAC1002.14	itt1	0.4843	1.968	0.315	23.86	41.21	3.059	2.069
SPBC1539.10	nop16	0.8519	1.969	0.070	17.55	29.32	12.68	7.519
SPBC12C2.08	dnm1	0.7552	1.971	0.122	26.89	46.9	8.939	3.705
SPAC3F10.13	ucp6	0.5313	1.982	0.275	29.78	52.33	4.825	1.429
SPCC645.13	SPCC645.13	0.6267	1.982	0.203	29.95	52.65	5.12	2.751
SPBC409.08	SPBC409.08	0.8495	1.982	0.071	21	35.8	13.3	7.082
SPAC1565.07C	SPAC1565.07c	0.6059	1.983	0.218	27.41	47.86	3.702	3.029
SPAC1093.06C	dhc1	0.6078	1.984	0.216	25.54	44.33	4.362	2.831
SPBC30B4.03C	adn1	0.422	1.989	0.375	28.68	50.25	3.316	1.483
SPBC1709.01	chs2	0.5836	1.989	0.234	23.11	39.76	2.762	3.024
SPAC13C5.03	tht1	0.7285	1.991	0.138	23.7	40.87	7.315	4.059
SPBC1685.02C	rps1202	0.8027	1.992	0.095	17.04	28.32	9.557	5.684
SPBC530.11C	SPBC530.11c	0.5263	1.993	0.279	26.9	46.87	3.51	2.294

SPAC212.02	SPAC212.02	0.5772	1.994	0.239	28.54	49.96	5.412	1.698
SPBC4F6.16C	ero11	0.3859	1.998	0.414	24.98	43.26	2.191	1.762
SPAC2C4.15C	ubx2	0.828	1.999	0.082	22.78	39.11	11.45	6.372
SPCC162.10	ppk33	0.4749	2	0.323	26.86	46.78	3.336	1.934
SPAC14C4.10C	SPAC14C4.10c	0.5579	2.002	0.253	29.4	51.57	5.195	1.58
SPAC29A4.20	elp3	0.5528	2.003	0.257	24.28	41.93	5.39	1.168
SPAC10F6.06	vip1	0.6004	2.003	0.222	28.96	50.74	6.073	1.451
SPAC1F7.07C	fip1	0.6073	2.008	0.217	28.71	50.26	6.249	1.417
SPBC3H7.05C	SPBC3H7.05c	0.6232	2.01	0.205	24.75	42.8	5.593	2.489
SPCC16C4.04	SPCC16C4.04	0.651	2.013	0.186	30.77	54.13	7.128	1.652
SPCC777.04	SPCC777.04	0.4703	2.014	0.328	28.24	49.36	4.11	1.378
SPBC36.06C	spo9	0.5408	2.014	0.267	26.53	46.15	4.905	1.624
SPAC23H4.09	cdb4	0.5882	2.016	0.230	29.9	52.48	5.571	1.846
SPAC1F7.08	fio1	0.8387	2.017	0.076	19.46	32.82	12.41	6.826
SPBC211.06	gfh1	0.4554	2.018	0.342	27.25	47.49	4.377	0.5811
SPCC188.02	par1	0.7873	2.023	0.104	16.04	26.39	8.922	5.371
SPAC3F10.15C	spo12	0.1765	2.036	0.753	21.37	36.39	0.6111	1.158
SPCC1753.03C	rec7	0.5754	2.038	0.240	28.69	50.17	5.183	2.036
SPBC8D2.03C	hhf2	0.8319	2.038	0.080	22	37.57	12.3	6.459
SPCC338.11C	rrg1	0.4596	2.042	0.338	23.9	41.13	2.322	2.216
SPAC29B12.03	spd1	0.5899	2.042	0.229	28.44	49.69	5.965	1.508
SPAC30C2.02	mmd1	0.5186	2.043	0.285	27.03	47.03	4.175	1.987
SPAC26F1.08C	SPAC26F1.08c	0.4456	2.048	0.351	27.44	47.79	3.525	1.667
SPBC1289.08	uap1	0.5559	2.05	0.255	23.4	40.19	3.313	2.737
SPBC115.02C	SPBC115.02c	0.5769	2.05	0.239	29.24	51.18	5.121	2.145
SPAC17G6.13	slt1	0.7499	2.05	0.125	33.18	58.59	9.574	3.367
SPAC16E8.13	SPAC16E8.13	0.3881	2.051	0.411	27.5	47.89	3.602	0.9704
SPCC63.14	SPCC63.14	0.6548	2.052	0.184	29.59	51.84	6.017	2.968
SPBC1604.03C	SPBC1604.03c	0.6425	2.053	0.192	27.91	48.67	6.233	2.564
SPAC13G7.05	are1	0.7148	2.055	0.146	24.73	42.68	8.625	2.663
SPAC3A12.06C	SPAC3A12.06c	0.6289	2.056	0.201	29.33	51.33	6.254	2.245
SPAC23C11.10	SPAC23C11.10	0.476	2.061	0.322	24.97	43.12	2.149	2.382
SPAC212.04C	SPAC212.04c	0.6052	2.065	0.218	28.18	49.16	4.814	2.789
SPBC839.03C	SPBC839.03c	0.4854	2.068	0.314	30.15	52.85	4.279	1.563
SPCC330.12C	sdh3	0.5636	2.07	0.249	39.43	70.32	5.179	1.938
SPCC1322.08	srk1	0.5362	2.079	0.271	30.18	52.9	2.857	2.724
SPCC1442.03	SPCC1442.03	0.6858	2.08	0.164	28.04	48.85	7.511	2.791
SPAC24C9.14	otu1	0.6266	2.081	0.203	30.61	53.69	6.274	2.267
SPBC27B12.10C	tom7	0.7494	2.086	0.125	13.2	20.91	7.541	4.769
SPBC29B5.02C	isp4	0.4047	2.088	0.393	27.96	48.69	3.785	1.065
SPBPB10D8.02C	SPBPB10D8.02c	0.5329	2.092	0.273	25.81	44.65	5.061	1.579
SPCC1753.05	rsm1	0.8287	2.094	0.082	20.7	35.02	12.04	6.704
SPAC10F6.05C	ubc6	0.5629	2.101	0.250	29.16	50.92	3.275	2.907
SPCC757.11C	SPCC757.11c	0.4411	2.102	0.355	28.04	48.82	4.226	1.044
SPBC1734.04	anp1	0.4983	2.107	0.303	15.68	25.55	3.986	2.169
SPAC22F8.09	rrp16	0.4813	2.108	0.318	25.49	44.01	4.529	1.352
SPAC1556.03	azr1	0.592	2.111	0.228	30.49	53.42	5.706	2.133
SPAC1805.15C	pub2	0.03925	2.112	1.406	24.63	42.38	1.01	0.5957
SPBC1E8.03C	SPBC1E8.03c	0.488	2.112	0.312	30.65	53.72	4.582	1.413
SPBP8B7.27	mug30	0.6168	2.112	0.210	29.89	52.29	4.442	3.212
SPAC11E3.13C	gas5	0.7688	2.113	0.114	20.4	34.43	9.42	4.702
SPBC3E7.10	fma1	0.8401	2.117	0.076	22.18	37.75	11.97	7.761
SPBC1347.13C	SPBC1347.13c	0.8344	2.118	0.079	20.98	35.49	12.24	7.19
SPAC27D7.12C	but1	0.7123	2.119	0.147	24.26	41.68	8.294	3.213
SPAC688.14	set13	0.5564	2.12	0.255	24.59	42.3	2.857	2.972
SPAC222.16C	csn3	0.6462	2.125	0.190	29.9	52.27	6.503	2.702
SPAC13G7.09C	SPAC13G7.09c	0.4027	2.127	0.395	27.51	47.76	3.262	1.605
SPAC343.06C	SPAC343.06c	0.5911	2.127	0.228	28.3	49.26	4.772	2.762
SPCC962.04	rps1201	0.8011	2.129	0.096	17.56	29.05	10	6.082
SPBC336.03	efc25	0.6362	2.131	0.196	28.11	48.9	3.988	3.655
SPAC56F8.12	SPAC56F8.12	0.5723	2.134	0.242	26.91	46.64	5.644	1.872
SPBC16D10.08C	SPBC16D10.08c	0.6456	2.135	0.190	28.63	49.87	4.621	3.629
SPAC17C9.14	SPAC17C9.14	0.7044	2.142	0.152	25.61	44.16	8.31	3.018

SPAC20G8.02	SPAC20G8.02	0.2654	2.144	0.576	24.32	41.73	2.436	1.169
SPCC645.12C	SPCC645.12c	0.5074	2.146	0.295	28.96	50.47	4.554	1.824
SPAC23H3.14	SPAC23H3.14	0.4411	2.15	0.355	28.12	48.88	4.545	0.3809
SPAC1805.11C	rps2602	0.457	2.15	0.340	30.15	52.7	4.208	1.47
SPCC777.02	SPCC777.02	0.5789	2.15	0.237	29.27	51.04	4.301	2.854
SPBC83.17	SPBC83.17	0.2608	2.155	0.584	26.68	46.16	2.571	1.053
SPAP8A3.07C	SPAP8A3.07c	0.8254	2.156	0.083	20.15	33.87	11.49	7.085
SPAC607.10	spo3	0.5872	2.16	0.231	29.71	51.85	5.931	1.992
SPBC839.02	SPBC839.02	0.7017	2.161	0.154	24.41	41.88	7.387	3.675
SPBC20F10.10	psl1	0.5788	2.164	0.237	24.9	42.8	3.866	3.029
SPAC1142.07C	vps32	0.6417	2.165	0.193	9.374	13.57	5.61	3.264
SPCC14G10.04	SPCC14G10.04	0.4511	2.17	0.346	15.72	25.5	4.608	0.7891
SPAC1039.05C	klf1	0.3459	2.177	0.461	25.11	43.16	2.482	1.66
SPBC21C3.02C	dep1	0.6121	2.182	0.213	28.5	49.54	4.14	3.394
SPAC1F8.01	ght3	0.3406	2.188	0.468	32.66	57.36	1.926	1.797
SPBC428.14	SPBC428.14	0.5807	2.189	0.236	31.21	54.63	4.744	2.775
SPAC5D6.06C	alg14	0.7371	2.189	0.132	21.16	35.7	8.119	4.716
SPCC790.02	pep3	0.8587	2.192	0.066	16.35	26.65	13.02	9.491
SPAC1687.21	SPAC1687.21	0.5988	2.193	0.223	27.71	48.02	4.125	3.25
SPAC1639.01C	SPAC1639.01c	0.7386	2.194	0.132	21.99	37.25	7.816	4.926
SPAC1952.03	otu2	0.7876	2.194	0.104	16.75	27.39	9.943	5.72
SPAC1002.17C	urg2	0.4756	2.198	0.323	23.78	40.62	2.187	2.555
SPAC31A2.13C	sft1	0.2173	2.2	0.663	21.07	35.52	2.653	0.3597
SPBC651.03C	gyp10	0.8066	2.201	0.093	26.02	44.84	12.02	5.755
SPAP8A3.03	SPAP8A3.03	0.6163	2.202	0.210	26.58	45.89	6.106	2.601
SPBC1734.06	rhp18	0.8513	2.202	0.070	15.07	24.22	13.3	8.729
SPCC965.13	SPCC965.13	0.6	2.203	0.222	30.01	52.33	5.754	2.537
SPAC10F6.15	SPAC10F6.15	0.6575	2.204	0.182	31.4	54.95	7.505	2.444
SPCC777.12C	SPCC777.12c	0.5255	2.206	0.279	26.11	45	5.467	1.313
SPBC4B4.07C	usp102	0.4	2.207	0.398	16.58	27.05	3.754	1.356
SPAC3H1.10	SPAC3H1.10	0.6163	2.211	0.210	31.36	54.86	6.173	2.581
SPAC13G6.06C	gcv2	0.5758	2.215	0.240	30.25	52.77	3.457	3.203
SPBC17G9.08C	cnt5	0.305	2.218	0.516	18.06	29.82	3.153	0.9624
SPAC227.15	SPAC227.15	0.6103	2.219	0.214	30.76	53.72	4.349	3.385
SPAC821.10C	sod1	0.5984	2.222	0.223	30.32	52.88	5.341	2.816
SPAC12B10.12C	rhp41	0.5679	2.223	0.246	26	44.75	3.319	3.164
SPBPJ4664.05	SPBPJ4664.05	0.6085	2.223	0.216	30.94	54.06	6.568	2.085
SPBC1539.06	SPBC1539.06	0.7048	2.224	0.152	21.11	35.54	5.144	4.803
SPBC14C8.16C	bot1	0.8143	2.225	0.089	19.68	32.85	11.51	6.692
SPBC577.15C	sim3	0.4225	2.228	0.374	3.466	2.329	2.329	2.216
SPBC11B10.10C	pht1	0.5326	2.233	0.274	16.25	26.38	5.011	2.051
SPCC548.07C	ght1	0.6141	2.234	0.212	22.04	37.28	3.873	3.598
SPCC1739.04C	SPCC1739.04c	0.714	2.234	0.146	22.95	39	6.212	4.746
SPAC1F12.05	SPAC1F12.05	0.8096	2.235	0.092	24.46	41.84	10.88	6.724
SPAC4G8.13C	prz1	0.8362	2.235	0.078	22.88	38.86	12.94	7.729
SPBP4H10.10	SPBP4H10.10	0.5536	2.238	0.257	31.09	54.31	5.495	2.002
SPBC1683.06C	SPBC1683.06c	0.3598	2.245	0.444	28.33	49.09	1.956	1.947
SPAC589.06C	SPAC589.06c	0.3162	2.247	0.500	22.25	37.65	2.148	1.679
SPAC24H6.03	cul3	0.591	2.247	0.228	27.99	48.45	5.977	2.334
SPAC9G1.10C	SPAC9G1.10c	0.1609	2.248	0.793	24.2	41.33	1.286	1.173
SPAC2C4.06C	SPAC2C4.06c	0.4913	2.25	0.309	26.92	46.43	4.883	1.56
SPAC9G1.05	SPAC9G1.05	0.6062	2.252	0.217	28	48.47	6.255	2.438
SPCC1450.16C	SPCC1450.16c	0.4767	2.26	0.322	23.32	39.63	2.563	2.58
SPBC1198.12	mfr1	0.6311	2.261	0.200	25.3	43.36	2.635	4.095
SPAC2E1P5.03	SPAC2E1P5.03	0.02859	2.269	1.544	20.96	35.17	0.9799	0.5915
SPCC663.10	SPCC663.10	0.4074	2.27	0.390	23.47	39.9	3.62	1.666
SPAC688.04C	gst3	0.5233	2.27	0.281	28.57	49.5	2.609	2.959
SPBP4H10.05C	spe2	0.5647	2.271	0.248	30.75	53.59	4.353	2.91
SPAC1F8.08	SPAC1F8.08	0.6862	2.275	0.164	31.3	54.63	8.12	3.143
SPBC19C2.13C	ctu2	0.5061	2.277	0.296	25.34	43.41	5.463	1.123
SPAPYUK71.03C	syn1	0.716	2.278	0.145	24.68	42.16	8.417	3.952
SPCC1450.12	SPCC1450.12	0.8435	2.282	0.074	23.93	40.74	14.24	8.078
SPBC3H7.14	mug176	0.4674	2.287	0.330	27.64	47.72	3.847	2.124

SPAC4F8.01	did4	0.4934	2.292	0.307	30.94	53.91	5.034	1.556
SPCC1840.12	SPCC1840.12	0.1662	2.293	0.779	24.08	41.01	1.84	1.076
SPAC23H3.15C	SPAC23H3.15c	0.8319	2.293	0.080	21.95	37	13.3	7.547
SPBC25D12.05	trm1	0.3599	2.295	0.444	25.2	43.1	2.495	1.873
SPAC17C9.05C	pmc3	0.6092	2.295	0.215	29.17	50.59	5.647	3.019
SPBC15C4.01C	oca3	0.4722	2.302	0.326	27.65	47.72	2.133	2.676
SPAC17C9.09C	tim13	0.3013	2.306	0.521	24.27	41.34	2.245	1.632
SPAC959.07	rps403	0.7279	2.306	0.138	20	33.31	9.031	4.109
SPAC15A10.03C	rhp54	0.7339	2.308	0.134	14.49	22.93	7.718	5.003
SPAC3A11.03	SPAC3A11.03	0.3283	2.31	0.484	27.84	48.06	3.63	0.7443
SPAC11H11.05C	fta6	0.5078	2.313	0.294	28.2	48.72	3.518	2.673
SPAC222.14C	SPAC222.14c	0.398	2.317	0.400	26.25	45.04	3.84	1.494
SPAC15E1.02C	SPAC15E1.02c	0.8306	2.317	0.081	21.77	36.61	12.96	7.746
SPBC1198.08	SPBC1198.08	0.7245	2.32	0.140	30.34	52.75	9.443	3.738
SPCC285.13C	nup60	0.4786	2.325	0.320	22.64	38.24	2.448	2.703
SPAC2F3.07C	SPAC2F3.07c	0.5431	2.326	0.265	29.77	51.66	5.186	2.321
SPAC4G8.06C	trm12	0.3981	2.33	0.400	28.49	49.24	3.562	1.724
SPCC11E10.03	mug1	0.4492	2.331	0.348	24.61	41.93	2.339	2.517
SPAC4G8.04	SPAC4G8.04	0.8173	2.339	0.088	20.86	34.85	12.15	7.22
SPAC8C9.10C	SPAC8C9.10c	0.8225	2.34	0.085	21.97	36.96	12.49	7.45
SPAC140.01	sdh2	0.8269	2.344	0.083	17.09	27.76	12.89	7.637
SPBC21B10.12	rec6	0.7893	2.345	0.103	34.18	59.92	13.99	3.582
SPBC19C7.10	bqt4	0.4221	2.351	0.375	29.24	50.6	4.428	1.261
SPAPB1A11.03	SPAPB1A11.03	0.5109	2.351	0.292	30.2	52.42	4.624	2.298
SPAC6B12.02C	mus7	0.4152	2.352	0.382	30.42	52.83	4.243	1.389
SPAP11E10.02C	mam3	0.7512	2.358	0.124	28.39	49	10.72	4.193
SPBC1271.09	SPBC1271.09	0.6003	2.36	0.222	27.99	48.24	4.061	3.622
SPCC14G10.03C	ump1	0.7943	2.36	0.100	17.67	28.81	10.62	6.551
SPAC3G6.05	SPAC3G6.05	0.6962	2.361	0.157	24.62	41.9	8.556	3.51
SPAC6G10.11C	ubi3	0.4591	2.362	0.338	30.38	52.73	4.694	1.574
SPAC23D3.13C	SPAC23D3.13c	0.6324	2.363	0.199	28.51	49.21	7.146	2.709
SPCC594.01	SPCC594.01	0.6371	2.364	0.196	25.89	44.28	5.516	3.733
SPBC18H10.06C	swd2	0.7328	2.364	0.135	25.37	43.3	8.92	4.86
SPBC28F2.05C	SPBC28F2.05c	0.516	2.365	0.287	26.92	46.21	5.577	1.591
SPAC16C9.05	cph1	0.8164	2.368	0.088	21.37	35.77	11.94	7.401
SPAC1486.01	SPAC1486.01	0.4315	2.369	0.365	25.44	43.42	4.645	1.168
SPAC57A10.08C	SPAC57A10.08c	0.4436	2.376	0.353	29.74	51.5	4.769	1.243
SPCC613.08	SPCC613.08	0.6477	2.376	0.189	23.32	39.42	4.274	4.299
SPBC18H10.04C	sce3	0.717	2.377	0.144	26.69	45.75	8.357	4.408
SPAC11E3.15	rpl22	0.5512	2.382	0.259	30.76	53.4	5.58	2.313
SPBC119.08	pmk1	0.5802	2.382	0.236	23.94	40.57	5.743	2.697
SPAC22A12.03C	csn4	0.4715	2.383	0.327	29.76	51.53	4.797	1.715
SPBC23E6.09	ssn6	0.3837	2.388	0.416	30.1	52.15	3.812	1.509
SPBC23G7.15C	rpp202	0.5728	2.388	0.242	27.72	47.68	2.736	3.607
SPCC1739.15	wtf21	0.3115	2.389	0.507	3.214	1.552	1.552	1.899
SPBC83.04	apc15	0.3537	2.389	0.451	29.66	51.33	2.555	1.925
SPCC338.14	SPCC338.14	0.3988	2.389	0.399	28.89	49.88	3.402	1.91
SPBC26H8.11C	SPBC26H8.11c	0.4868	2.391	0.313	26.05	44.53	5.025	1.757
SPCPB16A4.06C	SPCPB16A4.06c	0.5449	2.391	0.264	28.1	48.39	5.096	2.559
SPAC631.02	SPAC631.02	0.7752	2.392	0.111	17.89	29.16	9.869	6.042
SPBC8D2.17	SPBC8D2.17	0.8095	2.393	0.092	21.01	35.03	11.84	7.11
SPAPB17E12.08	SPAPB17E12.08	0.4766	2.395	0.322	20.47	34.02	3.039	2.66
SPCC757.12	SPCC757.12	0.5027	2.399	0.299	29.87	51.71	3.45	2.783
SPBC29A3.02C	his7	0.8258	2.399	0.083	22.15	37.18	13.33	7.656
SPBC20F10.03	SPBC20F10.03	0.5618	2.402	0.250	28.3	48.75	5.152	2.796
SPAC21E11.04	ppr1	0.5304	2.408	0.275	29.56	51.1	4.229	2.822
SPCC4B3.03C	SPCC4B3.03c	0.8336	2.413	0.079	21.92	36.71	13.48	8.329
SPCPB16A4.05C	SPCPB16A4.05c	0.5747	2.415	0.241	26.51	45.35	5.913	2.573
SPBC359.05	abc3	0.4833	2.416	0.316	26.06	44.49	3.546	2.604
SPAC821.06	spn2	0.495	2.424	0.305	29.25	50.49	5.608	1.263
SPAC926.03	rlc1	0.5112	2.426	0.291	24.94	42.37	5.618	1.657
SPAC31A2.12	SPAC31A2.12	0.6795	2.426	0.168	20.67	34.35	7.961	3.648
SPCC965.06	SPCC965.06	0.5076	2.427	0.294	25.73	43.86	4.418	2.521

SPAC630.11	vps55	0.4526	2.436	0.344	30.22	52.29	4.987	1.316
SPBC947.14C	cbp6	0.6792	2.436	0.168	10.28	14.77	6.305	4.471
SPAC4A8.02C	SPAC4A8.02c	0.6032	2.438	0.220	30.86	53.5	5.537	3.328
SPAC3G9.07C	hos2	0.2574	2.44	0.589	27.33	46.84	3.262	0.5209
SPAC589.07C	atg18	0.8283	2.444	0.082	23.29	39.23	13.72	7.957
SPAC17H9.03C	rdl1	0.7856	2.445	0.105	18.26	29.77	10.77	6.404
SPAC6C3.06C	SPAC6C3.06c	0.2911	2.446	0.536	25.8	43.95	1.613	1.84
SPCC794.02	wtf5	0.8311	2.455	0.080	23.97	40.49	14.1	8.083
SPAC4D7.06C	SPAC4D7.06c	0.1447	2.456	0.840	22.2	37.15	1.739	1.127
SPAC6G9.14	SPAC6G9.14	0.6823	2.458	0.166	21.79	36.39	5.494	4.847
SPAC8C9.16C	mug63	0.824	2.458	0.084	23.08	38.8	13.37	7.834
SPBC19G7.17	SPBC19G7.17	0.655	2.467	0.184	24.31	41.11	7.023	3.692
SPBC725.04	SPBC725.04	0.3746	2.468	0.426	25.86	44.04	2.867	2.053
SPCC1919.09	tif6	0.397	2.469	0.401	29.06	50.04	4.119	1.556
SPAC521.05	rps802	0.7833	2.474	0.106	18.26	29.7	10.94	6.342
SPAC13D6.02C	byr3	0.493	2.476	0.307	19.11	31.3	2.162	3.062
SPAC56E4.07	SPAC56E4.07	0.3251	2.483	0.488	25.7	43.7	2.428	1.892
SPBC1105.04C	cbp1	0.3883	2.485	0.411	29.51	50.86	3.075	2.099
SPBC19F8.04C	SPBC19F8.04c	0.3549	2.488	0.450	23.62	39.77	2.146	2.133
SPBC1734.09	SPBC1734.09	0.2832	2.492	0.548	25.12	42.6	2.086	1.756
SPAC29A4.19C	cta5	0.6136	2.495	0.212	27.27	46.62	3.104	4.243
SPAC2C4.16C	rps801	0.6709	2.498	0.173	12.79	19.37	6.623	4.334
SPBC651.02	SPBC651.02	0.5121	2.5	0.291	26.12	44.46	5.348	2.168
SPCC965.08C	alr1	0.3815	2.501	0.419	25.69	43.64	3.336	1.966
SPCC663.06C	SPCC663.06c	0.5088	2.501	0.293	24.27	40.97	5.915	1.473
SPBPB2B2.11	SPBPB2B2.11	0.4929	2.502	0.307	25.55	43.39	5.36	1.841
SPAC26F1.14C	aif1	0.4935	2.504	0.307	29.08	50.01	5.019	2.151
SPCP31B10.04	SPCP31B10.04	0.6898	2.511	0.161	31.96	55.42	9.632	2.981
SPAC5H10.02C	SPAC5H10.02c	0.3815	2.513	0.419	27.98	47.93	3.251	2.018
SPAC1F8.03C	str3	0.6827	2.513	0.166	24.86	42.05	8.163	3.926
SPBP4H10.19C	SPBP4H10.19c	0.4715	2.523	0.327	28.95	49.74	4.544	2.224
SPAC589.05C	SPAC589.05c	0.3088	2.524	0.510	29.77	51.28	3.799	0.7395
SPAC5D6.02C	mug165	0.1284	2.525	0.891	22.41	37.43	1.779	1.071
SPAC26F1.01	sec74	0.4008	2.531	0.397	29.85	51.41	4.313	1.557
SPBC1198.14C	fbp1	0.5895	2.533	0.230	27.87	47.69	3.431	3.944
SPAC26A3.10	cnt6	0.3989	2.534	0.399	27.3	46.6	4.581	1.197
SPAC1610.04	mug99	0.8301	2.534	0.081	23.87	40.15	14.22	8.409
SPAC6F12.09	rdp1	0.3022	2.538	0.520	27.81	47.56	3.575	1.107
SPBC17G9.07	rps2402	0.1896	2.544	0.722	28.35	48.57	1.741	1.409
SPCC777.06C	SPCC777.06c	0.3454	2.544	0.462	24.57	41.46	2.154	2.134
SPAC3A12.09C	SPAC3A12.09c	0.5343	2.546	0.272	32.08	55.59	6.028	2.092
SPCC191.05C	SPCC191.05c	0.8205	2.549	0.086	23.37	39.19	13.66	7.923
SPAC1F12.02C	SPAC1F12.02c	0.5808	2.55	0.236	24.1	40.57	6.211	2.858
SPCPB1C11.01	amt1	0.3099	2.552	0.509	24.21	40.77	2.874	1.718
SPAC5H10.05C	SPAC5H10.05c	0.3731	2.556	0.428	26.61	45.27	4.492	0.8354
SPCC569.06	SPCC569.06	0.7093	2.558	0.149	30	51.65	8.789	4.585
SPBC12C2.01C	SPBC12C2.01c	0.5632	2.559	0.249	14.32	22.14	6.021	2.686
SPAC1071.06	arp9	0.2832	2.56	0.548	26.56	45.18	2.017	1.831
SPAC27D7.14C	tpr1	0.4762	2.562	0.322	13.87	21.28	4.832	2.378
SPAC16E8.08	SPAC16E8.08	0.3629	2.563	0.440	24.14	40.61	2.041	2.275
SPBC83.19C	SPBC83.19c	0.3914	2.563	0.407	25.43	43.03	2.634	2.341
SPCP1E11.04C	pal1	0.6779	2.565	0.169	16.78	26.75	7.278	4.423
SPAC26H5.03	pcf2	0.542	2.566	0.266	26.24	44.55	4.635	3.099
SPCC162.01C	SPCC162.01c	0.5593	2.57	0.252	31.02	53.54	4.342	3.42
SPAC4G9.11C	cmb1	0.3685	2.571	0.434	26.76	45.53	4.519	0.6514
SPAC3H1.08C	SPAC3H1.08c	0.3354	2.573	0.474	28.94	49.62	3.621	1.51
SPAC30C2.08	SPAC30C2.08	0.4829	2.576	0.316	27.98	47.81	4.787	2.32
SPAC6F12.06	SPAC6F12.06	0.547	2.579	0.262	27.5	46.91	6.17	2.319
SPAC1142.03C	swi2	0.1464	2.58	0.834	26.18	44.41	1.695	1.232
SPCC736.02	SPCC736.02	0.3509	2.581	0.455	25.16	42.5	2.09	2.214
SPAC11D3.01C	SPAC11D3.01c	0.7227	2.584	0.141	31.82	55.02	8.895	5.09
SPAC1687.10	mcp1	0.2591	2.589	0.587	29.59	50.82	3.037	1.292
SPAC140.04	SPAC140.04	0.5668	2.592	0.247	24.41	41.07	6.93	2.043

SPCC965.10	SPCC965.10	0.3893	2.593	0.410	30.98	53.43	4.724	0.9059
SPCC338.18	SPCC338.18	0.4548	2.593	0.342	30.22	52	5.385	1.327
SPCC1259.02C	SPCC1259.02c	0.4152	2.594	0.382	30.36	52.26	4.665	1.549
SPAC869.03C	SPAC869.03c	0.3946	2.595	0.404	26.89	45.74	2.672	2.392
SPBC337.07C	SPBC337.07c	0.3084	2.598	0.511	25.41	42.93	2.519	1.885
SPAC824.08	gda1	0.5432	2.599	0.265	28.82	49.35	6.507	1.953
SPBC106.13	SPBC106.13	0.7382	2.599	0.132	25.19	42.51	9.416	5.48
SPBC29A3.12	rps902	0.7801	2.599	0.108	21.54	35.66	9.459	7.285
SPAC1142.08	fhl1	0.7727	2.6	0.112	19.46	31.74	9.821	6.809
SPBC691.01	pfa5	0.2761	2.606	0.559	28.87	49.44	3.245	1.299
SPAC13G6.15C	SPAC13G6.15c	0.5109	2.609	0.292	29.16	49.97	5.531	2.281
SPBPJ4664.01	dps1	0.7051	2.609	0.152	15.65	24.54	8.312	4.861
SPCC1442.17C	ist1	0.4447	2.613	0.352	29.64	50.86	4.184	2.295
SPAC9E9.11	plr1	0.2941	2.615	0.532	30.41	52.31	3.322	1.419
SPAC11D3.03C	SPAC11D3.03c	0.398	2.615	0.400	30.39	52.27	4.869	0.9127
SPCC1840.04	pca1	0.5434	2.615	0.265	29.95	51.45	6.222	2.309
SPAC13G7.02C	ssa1	0.6801	2.615	0.167	28.73	49.16	7.201	4.658
SPBC216.06C	swi1	0.5377	2.619	0.269	29.75	51.07	4.49	3.197
SPAC4A8.05C	myp2	0.6382	2.619	0.195	26.28	44.53	7.41	3.531
SPAC17H9.14C	SPAC17H9.14c	0.3047	2.622	0.516	25.58	43.21	2.546	1.878
SPBC13E7.04	atp16	0.6727	2.622	0.172	20.5	33.66	7.557	4.559
SPBC3H7.08C	SPBC3H7.08c	0.3556	2.629	0.449	27.57	46.95	2.825	2.126
SPBC1348.14C	ght7	0.5681	2.629	0.246	28.92	49.48	5.753	3.101
SPAPB2C8.01	SPAPB2C8.01	0.4173	2.632	0.380	29.03	49.68	4.58	1.755
SPBC1198.06C	SPBC1198.06c	0.7126	2.634	0.147	16.35	25.81	8.376	5.406
SPBC14C8.05C	meu17	0.465	2.636	0.333	26.31	44.56	3.705	2.72
SPAC1952.16	rga9	0.663	2.637	0.178	30.05	51.6	7.587	4.109
SPBC16C6.04	SPBC16C6.04	0.7931	2.642	0.101	20.26	33.16	11.19	7.55
SPCC18B5.03	wee1	0.2745	2.65	0.561	19.12	30.99	2.409	1.768
SPAC22H10.11C	SPAC22H10.11c	0.0924	2.651	1.034	11.62	16.88	1.692	0.972
SPAC22F8.02C	pvg5	0.3359	2.653	0.474	23.74	39.69	2.119	2.191
SPBC106.10	pka1	0.3648	2.653	0.438	24.98	42.03	3.255	2.074
SPBC530.14C	dsk1	0.4802	2.653	0.319	24.96	41.99	4.186	2.723
SPAC15E1.07C	moa1	0.8093	2.657	0.092	18.28	29.41	13.1	7.894
SPCC965.05C	thp1	0.6042	2.658	0.219	27.3	46.38	7.373	2.834
SPAC4D7.07C	SPAC4D7.07c	0.5141	2.66	0.289	26.9	45.62	5.385	2.553
SPCP1E11.10	SPCP1E11.10	0.7163	2.661	0.145	9.658	13.17	6.684	5.927
SPAC3H1.12C	snt2	0.2423	2.665	0.616	22.56	37.44	3.285	0.9568
SPBC29A3.05	vps71	0.1786	2.668	0.748	26.31	44.5	2.868	0.506
SPBC1D7.05	byr2	0.7043	2.669	0.152	21.37	35.19	8.641	5.134
SPAC977.17	SPAC977.17	0.7714	2.671	0.113	28.01	47.69	9.905	7.001
SPAC22F8.11	plc1	0.4813	2.675	0.318	20.05	32.71	5.034	2.349
SPBC2G5.01	SPBC2G5.01	0.2514	2.677	0.600	26.5	44.84	2.894	1.442
SPBC13G1.10C	mug81	0.4629	2.677	0.335	24.34	40.78	4.328	2.521
SPAC29E6.01	pof11	0.4654	2.677	0.332	23.32	38.86	4.984	2.174
SPBC3H7.06C	pof9	0.3419	2.679	0.466	25.2	42.38	3.522	1.799
SPAC18G6.02C	chp1	0.4124	2.679	0.385	19.23	31.15	1.518	2.769
SPAC3G6.03C	SPAC3G6.03c	0.3983	2.68	0.400	28.3	48.22	4.894	1.163
SPBC660.17C	SPBC660.17c	0.4514	2.682	0.345	29.2	49.92	4.804	2.128
SPCC895.06	elp2	0.7882	2.685	0.103	20.68	33.87	11.38	7.386
SPAC31A2.06	atp25	0.4128	2.692	0.384	30.48	52.3	3.396	2.447
SPAC23D3.01	SPAC23D3.01	0.4473	2.693	0.349	28.89	49.32	4.623	2.221
SPAC23C11.14	zhf1	0.4356	2.695	0.361	17.05	27.01	4.079	2.387
SPAC1F8.05	isp3	0.5306	2.697	0.275	24.3	40.66	3.965	3.408
SPCC24B10.13	skb5	0.5624	2.697	0.250	25.95	43.77	4.187	3.731
SPCC70.03C	SPCC70.03c	0.5123	2.699	0.290	28.74	49.01	3.673	3.286
SPAC6G9.03C	mug183	0.5409	2.703	0.267	27.9	47.42	6.815	1.907
SPAC17A2.02C	SPAC17A2.02c	0.6724	2.709	0.172	24.91	41.78	8.751	3.932
SPAC6F12.03C	fsv1	0.5204	2.71	0.284	22.94	38.08	4.997	2.95
SPBC106.12C	SPBC106.12c	0.7421	2.712	0.130	26.24	44.29	10.79	5.383
SPAC1687.15	gsk3	0.4754	2.714	0.323	24.86	41.68	5.345	2.126
SPACUNK4.16C	SPACUNK4.16c	0.501	2.714	0.300	30.3	51.92	4.883	2.763
SPAC2F3.15	lsk1	0.5652	2.715	0.248	22.12	36.53	5.835	3.208

SPBC18E5.14C	SPBC18E5.14c	0.7578	2.717	0.120	16.92	26.74	9.941	6.531
SPBC16A3.01	spn3	0.6147	2.721	0.211	30.8	52.85	3.733	4.591
SPAC3G6.06C	rad2	0.4908	2.722	0.309	25.48	42.84	5.729	2.063
SPAC4F10.08	mug126	0.2653	2.723	0.576	29.79	50.94	2.128	1.852
SPBC4F6.04	rpl2502	0.7957	2.723	0.099	22.24	36.73	12.83	7.395
SPBP4G3.02	pho1	0.3927	2.725	0.406	29.17	49.78	4.507	1.696
SPAC186.05C	SPAC186.05c	0.4134	2.726	0.384	26.19	44.16	3.237	2.544
SPBC365.12C	ish1	0.5381	2.729	0.269	25.58	43.01	2.853	3.76
SPAC24C9.16C	cox8	0.6425	2.73	0.192	25.91	43.62	7.516	3.91
SPBC11C11.06C	SPBC11C11.06c	0.4432	2.733	0.353	30.83	52.87	5.372	1.589
SPAC23A1.16C	rtr1	0.4989	2.733	0.302	30.64	52.52	5.178	2.619
SPCC4G3.04C	coq5	0.5414	2.735	0.266	9.112	12	4.63	3.406
SPCC191.11	inv1	0.5767	2.737	0.239	24.45	40.86	4.722	3.85
SPAC1F8.02C	SPAC1F8.02c	0.5289	2.738	0.277	16.61	26.12	6.293	2.318
SPBPB2B2.07C	SPBPB2B2.07c	0.7493	2.739	0.125	24.88	41.68	11.59	5.385
SPAC637.06	SPAC637.06	0.7549	2.74	0.122	17.15	27.13	10.56	6.224
SPCC338.16	pof3	0.6809	2.743	0.167	12.55	18.45	7.634	4.873
SPAC227.06	SPAC227.06	0.4783	2.744	0.320	27.62	46.81	6.024	1.493
SPAC2F7.17	SPAC2F7.17	0.3608	2.745	0.443	30.71	52.63	3.738	1.937
SPAC17D4.03C	cis4	0.3472	2.747	0.459	29.65	50.64	4.379	1.2
SPAC869.09	SPAC869.09	0.5186	2.748	0.285	29.97	51.23	6.171	2.25
SPBC4B4.11	SPBC4B4.11	0.4111	2.75	0.386	28.57	48.6	4.925	1.597
SPBC19C7.08C	SPBC19C7.08c	0.5071	2.753	0.295	31.48	54.07	5.065	2.826
SPBC146.04	SPBC146.04	0.3927	2.763	0.406	32.47	55.91	4.352	1.899
SPCC757.07C	ctt1	0.5062	2.766	0.296	24.27	40.46	3.583	3.346
SPBC609.02	ptn1	0.5154	2.766	0.288	30.66	52.5	5.749	2.577
SPCC663.09C	SPCC663.09c	0.3586	2.768	0.445	26.15	44	1.721	2.49
SPBC18H10.20C	SPBC18H10.20c	0.2883	2.776	0.540	28.73	48.85	2.393	1.969
SPBC18H10.07	SPBC18H10.07	0.7296	2.777	0.137	17.35	27.44	9.348	5.831
SPCC576.13	swc5	0.7539	2.777	0.123	21.81	35.82	10.58	6.319
SPCC16A11.15C	SPCC16A11.15c	0.5983	2.778	0.223	27.82	47.12	6.485	3.616
SPAC25H1.07	emc1	0.7625	2.78	0.118	17.92	28.5	10.36	6.835
SPBC4B4.03	rsc1	0.3874	2.786	0.412	30.37	51.92	4.53	1.734
SPAC2H10.01	SPAC2H10.01	0.3892	2.79	0.410	28.97	49.27	4.538	1.76
SPAC25G10.02	cce1	0.4194	2.792	0.377	25.12	42.03	4.975	1.783
SPCC18B5.11C	cds1	0.6179	2.796	0.209	31.99	54.94	8.557	2.62
SPAC1786.04	SPAC1786.04	0.3127	2.803	0.505	30.54	52.21	4.249	0.8579
SPBC557.02C	SPBC557.02c	0.7616	2.804	0.118	19.26	30.97	10.65	6.765
SPAC3G6.09C	tps2	0.5446	2.805	0.264	27.32	46.14	4.639	3.568
SPAP14E8.02	SPAP14E8.02	0.4167	2.806	0.380	30.67	52.44	5.012	1.739
SPBC1198.03C	SPBC1198.03c	0.5099	2.81	0.293	29.09	49.47	5.685	2.634
SPAC7D4.13C	SPAC7D4.13c	0.6699	2.811	0.174	24.86	41.5	8.549	4.329
SPCC162.12	tco89	0.6516	2.813	0.186	15.63	24.13	8.737	3.651
SPAC1687.12C	coq4	0.4075	2.815	0.390	6.101	6.184	4.011	2.333
SPBC776.14	plh1	0.4275	2.816	0.369	27.66	46.77	4.856	2.055
SPCC1442.16C	zta1	0.3021	2.818	0.520	26.62	44.81	2.104	2.146
SPCC1672.03C	SPCC1672.03c	0.5659	2.818	0.247	29.13	49.53	7.206	2.553
SPBPB2B2.05	SPBPB2B2.05	0.4394	2.819	0.357	30.43	51.97	5.174	1.967
SPBC32H8.08C	omh5	0.7732	2.819	0.112	20.16	32.64	11.6	7.022
SPAC11E3.14	SPAC11E3.14	0.5041	2.821	0.297	27.8	47	5.941	2.378
SPAC24H6.08	SPAC24H6.08	0.4379	2.823	0.359	25.28	42.27	2.322	3.036
SPBC947.01	SPBC947.01	0.4464	2.824	0.350	28.71	48.72	4.749	2.377
SPBC3D6.09	dpb4	0.2661	2.828	0.575	29.71	50.6	3.578	1.237
SPBC1709.11C	png2	0.323	2.83	0.491	27.76	46.93	2.298	2.249
SPAC5D6.09C	mug86	0.3235	2.84	0.490	26	43.58	1.384	2.372
SPAC1002.01	SPAC1002.01	0.162	2.845	0.790	27.44	46.3	2.787	0.8991
SPBC20F10.05	nrl1	0.2634	2.845	0.579	30.84	52.69	3.874	0.543
SPAC30.03C	tsn1	0.3746	2.846	0.426	24.37	40.51	3.846	2.151
SPBC1271.01C	pof13	0.5007	2.848	0.300	29.27	49.73	4.046	3.296
SPCC126.15C	sec65	0.8052	2.848	0.094	15.67	24.14	11.59	9.091
SPBC25B2.11	pof2	0.2753	2.851	0.560	27.59	46.57	3.738	1.21
SPBC32H8.05	SPBC32H8.05	0.4691	2.856	0.329	28.01	47.34	5.968	1.765
SPBC16D10.11C	rps1801	0.7239	2.86	0.140	17.52	27.59	9.355	5.899

SPAC1805.06C	hem2	0.05127	2.862	1.290	25.49	42.59	1.209	0.9427
SPCC24B10.03	SPCC24B10.03	0.3847	2.862	0.415	31.37	53.65	4.701	1.7
SPCC1672.09	SPCC1672.09	0.5658	2.862	0.247	28.81	48.84	7.887	1.882
SPBC557.05	SPBC557.05	0.4465	2.864	0.350	29.74	50.58	4.016	2.775
SPCC965.11C	SPCC965.11c	0.1545	2.866	0.811	26.04	43.61	2.767	0.8481
SPCC1494.09C	SPCC1494.09c	0.5593	2.869	0.252	25.95	43.44	7.112	2.644
SPAC869.07C	mel1	0.2511	2.87	0.600	26.8	45.04	2.031	1.912
SPBC21H7.06C	SPBC21H7.06c	0.3284	2.87	0.484	30.57	52.13	3.535	1.936
SPAC32A11.02C	SPAC32A11.02c	0.365	2.874	0.438	25.68	42.93	4.953	0.9275
SPAC139.04C	fap2	0.1799	2.875	0.745	25.66	42.88	2.064	1.515
SPAC24B11.08C	SPAC24B11.08c	0.4494	2.875	0.347	30.38	51.77	5.993	1.259
SPAC2C4.10C	SPAC2C4.10c	0.5994	2.876	0.222	24.85	41.35	5.319	4.298
SPAC869.10C	put4	0.4925	2.877	0.308	29.49	50.08	4.538	3.095
SPAC20H4.06C	SPAC20H4.06c	0.3575	2.882	0.447	21.15	34.38	3.053	2.358
SPBC32H8.02C	nep2	0.4489	2.882	0.348	27.05	45.49	5.699	1.751
SPBC4F6.12	pxl1	0.3774	2.883	0.423	27.43	46.2	4.733	1.605
SPAC959.06C	SPAC959.06c	0.4026	2.885	0.395	28.67	48.53	5.224	1.432
SPAC13F5.07C	SPAC13F5.07c	0.2569	2.893	0.590	25.49	42.53	1.775	2.001
SPAC25H1.04	mug105	0.7203	2.905	0.142	24.85	41.3	10.95	5.093
SPCC31H12.03C	SPCC31H12.03c	0.5911	2.906	0.228	26.95	45.26	7.776	2.981
SPAC6F6.09	eaf6	0.2384	2.908	0.623	21.35	34.71	3.302	1.334
SPCC645.02	gep4	0.6643	2.915	0.178	34.09	58.68	10.15	3.302
SPBC582.04C	SPBC582.04c	0.4171	2.917	0.380	24.76	41.11	4.266	2.462
SPAC15A10.15	sgo2	0.7653	2.917	0.116	21.95	35.82	10.5	7.445
SPCC777.15	SPCC777.15	0.4407	2.918	0.356	26.74	44.85	6.041	1.04
SPAC4F8.10C	stg1	0.6832	2.918	0.165	24.27	40.18	8.049	5.279
SPAC1002.05C	jmj2	0.3548	2.921	0.450	32.67	56	4.825	1.143
SPCC1739.10	mug33	0.6712	2.924	0.173	22.83	37.46	9.19	4.368
SPAC644.14C	rhp51	0.3322	2.928	0.479	29.24	49.52	4.156	1.651
SPAC31A2.15C	dcc1	0.2642	2.932	0.578	24.65	40.87	2.174	2.011
SPBC30D10.14	SPBC30D10.14	0.3045	2.933	0.516	26.43	44.22	2.162	2.253
SPCC320.08	SPCC320.08	0.5348	2.933	0.272	27.46	46.16	6.669	2.668
SPBC428.04	apq12	0.5302	2.934	0.276	29.61	50.21	5.663	3.216
SPBC1709.09	rrf1	0.6454	2.941	0.190	16.92	26.32	7.597	4.533
SPBC713.11C	pmp3	0.5406	2.946	0.267	29.04	49.11	5.997	3.237
SPCP1E11.03	mug170	0.3077	2.948	0.512	26.55	44.43	2.617	2.197
SPCC757.04	SPCC757.04	0.3767	2.949	0.424	27.87	46.9	3.894	2.291
SPBC1718.02	hop1	0.1004	2.954	0.998	28.3	47.71	2.117	1.023
SPAC2C4.09	SPAC2C4.09	0.335	2.954	0.475	27.26	45.74	3.018	2.284
SPAC27E2.09	mak2	0.5187	2.954	0.285	27.72	46.62	7.071	1.924
SPAC1805.05	cki3	0.1972	2.958	0.705	29.85	50.61	3.371	0.4084
SPBC609.03	iqw1	0.5031	2.958	0.298	28.89	48.81	3.214	3.669
SPAC2F7.06C	pol4	0.6154	2.96	0.211	31.41	53.54	9.145	2.564
SPAC11H11.02C	mug162	0.5216	2.964	0.283	28.52	48.1	6.756	2.401
SPBC215.03C	csn1	0.2629	2.965	0.580	18.9	29.99	4.036	0.5338
SPCC417.07C	mto1	0.5226	2.965	0.282	24.21	39.99	3.793	3.782
SPAC9E9.14	vps24	0.5298	2.965	0.276	24.52	40.56	7.15	2.183
SPCC1494.01	SPCC1494.01	0.3789	2.972	0.421	29.28	49.51	3.794	2.384
SPAC824.02	SPAC824.02	0.7006	2.978	0.155	18.86	29.89	10.16	5.029
SPAC186.03	SPAC186.03	0.2686	2.989	0.571	29.59	50.06	3.998	1.019
SPBC713.07C	SPBC713.07c	0.6282	2.999	0.202	27.77	46.63	8.347	3.852
SPBC17D11.02C	hrd1	0.3736	3	0.428	31.35	53.36	5.044	1.452
SPBC428.07	meu6	0.4019	3.003	0.396	28.85	48.65	4.314	2.415
SPAC1786.02	SPAC1786.02	0.7413	3.006	0.130	19.56	31.15	10.52	6.642
SPAC3C7.05C	mug191	0.3913	3.007	0.407	25.19	41.75	4.076	2.426
SPBC725.15	ura5	0.4325	3.008	0.364	30.48	51.7	4.183	2.786
SPBC15C4.06C	SPBC15C4.06c	0.5092	3.011	0.293	24.46	40.36	3.875	3.683
SPBC13G1.04C	SPBC13G1.04c	0.5248	3.013	0.280	29.08	49.07	6.959	2.423
SPBC15D4.03	slm9	0.5828	3.015	0.234	27.85	46.75	8.037	2.885
SPAC1F3.05	SPAC1F3.05	0.5974	3.015	0.224	31.66	53.92	8.251	3.112
SPAC17G8.14C	pck1	0.478	3.018	0.321	28.46	47.89	4.792	3.059
SPAC6G9.16C	SPAC6G9.16c	0.3523	3.024	0.453	25.45	42.22	3.425	2.363
SPCC74.02C	SPCC74.02c	0.7523	3.027	0.124	20.64	33.14	11.15	6.971

SPBC18H10.10C	saf4	0.457	3.029	0.340	29.06	49	3.816	3.173
SPAC11D3.10	SPAC11D3.10	0.5265	3.029	0.279	30.43	51.57	3.662	3.953
SPBC21B10.10	rps402	0.541	3.035	0.267	22.67	36.95	6.329	3.26
SPAC2G11.04	SPAC2G11.04	0.3645	3.036	0.438	27.86	46.72	3.284	2.519
SPAC688.13	scn1	0.5245	3.04	0.280	27.19	45.46	7.073	2.385
SPAC1783.08C	rpl1502	0.7783	3.042	0.109	13.87	20.37	12.02	8.086
SPCC584.13	SPCC584.13	0.284	3.046	0.547	24.88	41.1	2.072	2.239
SPBP35G2.14	SPBP35G2.14	0.2058	3.048	0.687	30.01	50.75	3.239	1.243
SPBC3B8.10C	nem1	0.3974	3.051	0.401	28.54	47.97	4.968	2.034
SPBC4B4.06	vps25	0.6914	3.053	0.160	26.56	44.24	10.58	4.682
SPBC216.02	mcp5	0.3267	3.057	0.486	28.89	48.61	4.023	1.899
SPAC343.11C	msc1	0.3867	3.064	0.413	28.17	47.25	5.293	1.551
SPBC405.02C	SPBC405.02c	0.3651	3.067	0.438	31.42	53.36	5.174	1.259
SPAC1486.08	SPAC1486.08	0.4105	3.068	0.387	29.36	49.49	3.084	2.975
SPBC2G2.10C	mug110	0.3781	3.076	0.422	30.41	51.45	4.088	2.585
SPBP35G2.05C	cki2	0.6826	3.078	0.166	34.26	58.68	9.343	5.168
SPCC777.03C	SPCC777.03c	0.4035	3.08	0.394	31.95	54.33	5.269	1.92
SPBC1271.15C	SPBC1271.15c	0.09143	3.083	1.039	28.31	47.48	2.361	0.7889
SPCC70.08C	SPCC70.08c	0.3804	3.087	0.420	27.4	45.76	3.601	2.612
SPBC1289.11	spf38	0.477	3.088	0.321	31.35	53.19	4.662	3.211
SPBC17A3.08	SPBC17A3.08	0.5617	3.089	0.250	29.01	48.79	7.491	3.041
SPAC23D3.11	ayr1	0.239	3.09	0.622	30.54	51.67	3.922	0.7187
SPACUNK12.02C	cmk1	0.3305	3.09	0.481	30.59	51.75	4.573	1.531
SPBC1921.01C	rpl35b	0.4381	3.09	0.358	25.98	43.08	5.68	2.126
SPAP32A8.03C	SPAP32A8.03c	0.2797	3.094	0.553	27.21	45.39	4.063	1.372
SPCC1450.06C	grx3	0.5104	3.096	0.292	28.9	48.56	3.471	3.903
SPAC186.01	SPAC186.01	0.4182	3.103	0.379	29.57	49.81	3.324	3.03
SPAC23A1.02C	SPAC23A1.02c	0.7425	3.103	0.129	21.01	33.71	11.08	6.82
SPCC1620.13	SPCC1620.13	0.7831	3.103	0.106	23.44	38.27	13.62	7.981
SPAC664.14	amt2	0.5939	3.109	0.226	28.37	47.54	7.778	3.658
SPAC3F10.12C	SPAC3F10.12c	0.521	3.111	0.283	28.97	48.66	6.072	3.23
SPCC74.05	rpl2702	0.733	3.112	0.135	18.55	29.05	8.943	7.219
SPBC4C3.06	syp1	0.3906	3.113	0.408	31.46	53.35	3.28	2.818
SPAC8F11.03	msh3	0.4168	3.113	0.380	30.06	50.72	3.416	3.009
SPAC25G10.09C	pan1	0.3487	3.116	0.458	24.7	40.63	4.892	1.501
SPBC29A10.10C	SPBC29A10.10c	0.6146	3.118	0.211	33.74	57.63	9.206	3.154
SPBC23G7.07C	SPBC23G7.07c	0.4841	3.123	0.315	24.77	40.73	6.399	2.393
SPCC285.14	trs130	0.624	3.123	0.205	13.71	19.93	6.71	4.847
SPAC1783.05	hrp1	0.4562	3.125	0.341	28.01	46.84	5.498	2.617
SPAC19G12.09	SPAC19G12.09	0.36	3.126	0.444	27.74	46.33	4.895	1.715
SPAC29A4.14C	SPAC29A4.14c	0.1149	3.13	0.940	25.26	41.65	1.809	1.362
SPAC3H1.14	SPAC3H1.14	0.435	3.137	0.362	28.14	47.06	5.856	2.013
SPAC11D3.15	SPAC11D3.15	0.6623	3.138	0.179	34.47	58.97	6.647	5.769
SPAC1486.04C	alm1	0.6239	3.142	0.205	24.55	40.3	9.241	3.533
SPBC12D12.02C	cdm1	0.2964	3.144	0.528	29.6	49.8	4.128	1.62
SPAC630.15	mug177	0.185	3.147	0.733	25.75	42.54	2.787	1.492
SPAC24C9.05C	mug70	0.6767	3.148	0.170	19.51	30.79	10.63	4.411
SPCC895.07	alp14	0.363	3.15	0.440	29.23	49.08	4.183	2.303
SPCC18.10	SPCC18.10	0.252	3.152	0.599	25.86	42.74	2.411	2.07
SPAC23E2.03C	ste7	0.599	3.155	0.223	24.89	40.91	6.95	4.548
SPBC19F8.08	rps401	0.7357	3.155	0.133	19.77	31.27	10.85	6.796
SPBC646.09C	int6	0.3556	3.157	0.449	29.58	49.74	3.472	2.529
SPAC16A10.04	rho4	0.5019	3.159	0.299	32.57	55.35	5.042	3.495
SPBP22H7.04	SPBP22H7.04	0.4189	3.16	0.378	31.63	53.59	3.515	3.061
SPCC4B3.04C	nte1	0.4167	3.161	0.380	31.06	52.52	3.19	3.115
SPCC1919.03C	amk2	0.467	3.165	0.331	21.04	33.64	1.629	3.761
SPAC11D3.07C	SPAC11D3.07c	0.4451	3.173	0.352	30.23	50.92	4.318	3.105
SPBC365.01	SPBC365.01	0.5558	3.173	0.255	30.15	50.77	4.835	4.315
SPCC1322.14C	vtc4	0.09539	3.178	1.020	25.79	42.56	1.365	1.336
SPAC1B9.02C	sck1	0.4407	3.179	0.356	26.91	44.67	5.336	2.603
SPBP8B7.24C	atg8	0.5265	3.179	0.279	29.44	49.43	5.424	3.728
SPAC637.09	SPAC637.09	0.2401	3.18	0.620	29.55	49.62	3.597	1.49
SPBC1861.02	abp2	0.5907	3.18	0.229	35.19	60.25	9.126	2.613

SPAC27D7.05C	apc14	0.4302	3.193	0.366	32.48	55.11	6.058	1.839
SPAC14C4.15C	SPAC14C4.15c	0.1831	3.196	0.737	24.59	40.27	2.537	1.629
SPBC29B5.03C	rpl26	0.3427	3.205	0.465	31.11	52.51	4.968	1.518
SPAC12G12.10	wdr21	0.6269	3.205	0.203	25.55	42.06	8.96	4.052
SPAC922.07C	SPAC922.07c	0.2701	3.213	0.568	25.26	41.49	2.75	2.161
SPBC3D6.13C	pdi2	0.4254	3.213	0.371	29.65	49.75	5.298	2.484
SPBP4H10.11C	lcf2	0.4612	3.217	0.336	28.44	47.48	6.059	2.489
SPAC1782.11	met14	0.6583	3.217	0.182	23.39	37.96	8.862	5.338
SPAC29B12.10C	pgt1	0.7516	3.219	0.124	21.61	34.62	12.05	7.295
SPAC20G4.03C	hri1	0.4881	3.226	0.311	30.86	52.01	6.202	2.861
SPBC418.01C	his4	0.4261	3.227	0.370	27.97	46.57	2.345	3.402
SPCC417.12	SPCC417.12	0.2591	3.234	0.587	26.15	43.13	2.506	2.163
SPBC3E7.11C	SPBC3E7.11c	0.4247	3.235	0.372	30.43	51.19	4.1	3.051
SPCC63.08C	atg1	0.3784	3.236	0.422	25.69	42.26	3.666	2.755
SPBP35G2.02	SPBP35G2.02	0.382	3.237	0.418	27.93	46.48	4.629	2.393
SPAC18G6.04C	shm2	0.2674	3.238	0.573	27.85	46.33	2.448	2.233
SPAC4H3.05	srs2	0.7612	3.238	0.119	22.6	36.44	12.56	7.677
SPAC25B8.17	SPAC25B8.17	0.45	3.239	0.347	31.35	52.91	5.439	2.779
SPCC1393.05	ers1	0.4043	3.245	0.393	26.62	43.99	5.026	2.437
SPAC1565.04C	ste4	0.6309	3.253	0.200	23.48	38.07	8.251	4.96
SPBC8E4.05C	SPBC8E4.05c	0.5283	3.255	0.277	28.56	47.63	7.356	2.85
SPAC1006.03C	red1	0.6228	3.255	0.206	15.52	23.08	7.919	4.658
SPAC767.01C	vps1	0.2625	3.256	0.581	25.71	42.27	1.426	2.337
SPCC18.09C	SPCC18.09c	0.3952	3.256	0.403	29.04	48.54	3.798	2.889
SPAC2F3.18C	SPAC2F3.18c	0.2582	3.258	0.588	30.22	50.75	2.519	2.175
SPAC57A10.07	SPAC57A10.07	0.2522	3.259	0.598	29.24	48.9	3.737	1.618
SPCC622.14	SPCC622.14	0.4038	3.262	0.394	30.04	50.4	5.113	2.406
SPCC569.03	SPCC569.03	0.7231	3.262	0.141	26.68	44.08	12.05	6.01
SPAC13G6.04	tim8	0.5021	3.267	0.299	31.26	52.69	5.005	3.689
SPAC4A8.07C	SPAC4A8.07c	0.2596	3.269	0.586	31.03	52.26	4.23	1.18
SPAC823.09C	SPAC823.09c	0.541	3.272	0.267	28.04	46.62	7.68	2.922
SPAC30C2.07	SPAC30C2.07	0.6142	3.272	0.212	24.05	39.1	3.532	5.647
SPAC11D3.06	SPAC11D3.06	0.3187	3.274	0.497	30.58	51.39	3.466	2.362
SPCC1902.01	gaf1	0.2827	3.282	0.549	27.35	45.29	1.974	2.438
SPBC106.01	mph1	0.22	3.287	0.658	23.79	38.59	3.4	1.573
SPAC31A2.02	trm112	0.1849	3.293	0.733	16.91	25.62	3.26	1.323
SPCC330.01C	rhp16	0.4112	3.299	0.386	31.11	52.34	3.984	3.038
SPAC1556.04C	cdd1	0.4014	3.3	0.396	29.15	48.65	3.694	3.029
SPAC1F5.09C	shk2	0.4393	3.301	0.357	31.73	53.52	6.295	2.064
SPAC23C4.17	SPAC23C4.17	0.4134	3.307	0.384	31.57	53.2	3.347	3.227
SPAC890.03	ppk16	0.5221	3.31	0.282	28.88	48.13	8.05	2.085
SPBC1773.16C	SPBC1773.16c	0.2611	3.317	0.583	27.19	44.94	2.662	2.212
SPAC57A10.14	sgf11	0.5113	3.317	0.291	19.25	29.98	4.762	3.961
SPCC1494.03	arz1	0.2982	3.318	0.525	29.45	49.19	2.967	2.399
SPBC16E9.15	SPBC16E9.15	0.2435	3.32	0.614	30.36	50.9	3.064	1.971
SPAC7D4.05	SPAC7D4.05	0.2601	3.321	0.585	31.12	52.33	4.12	1.458
SPCC1739.03	hrr1	0.3128	3.323	0.505	25.2	41.18	3.24	2.439
SPBC557.04	ppk29	0.4817	3.324	0.317	31.24	52.54	3.96	3.809
SPCC1393.13	SPCC1393.13	0.781	3.324	0.107	19.63	30.69	13.89	8.713
SPCC31H12.02C	mug73	0.3077	3.326	0.512	28.19	46.79	2.029	2.634
SPBC577.05C	rec27	0.7717	3.327	0.113	14.93	21.84	12.65	8.619
SPCC622.01C	SPCC622.01c	0.3363	3.337	0.473	30	50.19	4.633	2.019
SPBP4H10.12	SPBP4H10.12	0.2624	3.341	0.581	29.27	48.79	4.492	0.8679
SPBC685.03	SPBC685.03	0.1791	3.346	0.747	26.54	43.65	2.624	1.689
SPBC19C2.10	SPBC19C2.10	0.2828	3.346	0.549	29.2	48.66	4.017	1.86
SPBC18H10.09	SPBC18H10.09	0.5308	3.348	0.275	23.8	38.49	7.213	3.253
SPAC922.04	SPAC922.04	0.6685	3.348	0.175	30.22	50.57	6.875	6.372
SPBC1709.10C	atx1	0.5086	3.352	0.294	29.51	49.22	7.314	2.706
SPAC17G6.03	SPAC17G6.03	0.2234	3.359	0.651	26.44	43.44	2.279	2.078
SPBP23A10.14C	ell1	0.3789	3.359	0.421	25.41	41.5	1.506	3.224
SPAC17G6.05C	SPAC17G6.05c	0.1508	3.362	0.822	27.01	44.52	2.319	1.604
SPAC25B8.09	SPAC25B8.09	0.3442	3.364	0.463	29.34	48.88	4.654	2.141
SPBP8B7.22	erd2	0.2305	3.365	0.637	27.34	45.12	4.029	1.17

SPCC550.09	SPCC550.09	0.3467	3.365	0.460	26.03	42.67	3.239	2.749
SPAC15E1.10	SPAC15E1.10	0.7605	3.365	0.119	23.49	37.88	12.89	8.007
SPAC57A10.04	mug10	0.227	3.366	0.644	28.63	47.54	2.569	2.046
SPAPB1A10.03	nxt1	0.4118	3.366	0.385	28.27	46.88	6.338	1.521
SPAC607.08C	SPAC607.08c	0.5519	3.367	0.258	29.4	49	8.235	2.997
SPAC589.02C	med13	0.7494	3.375	0.125	23.29	37.48	11.42	8.017
SPAC26H5.04	SPAC26H5.04	0.4526	3.376	0.344	31.83	53.55	5.506	3.019
SPAC6F12.04	SPAC6F12.04	0.00814	3.383	2.089	26.03	42.63	1.021	0.6628
SPBC8E4.03	SPBC8E4.03	0.3628	3.385	0.440	25.84	42.26	5.53	1.628
SPBC649.02	rps1902	0.7391	3.388	0.131	21.51	34.12	12.07	7.282
SPBPB2B2.18	SPBPB2B2.18	0.3032	3.399	0.518	31.38	52.66	3.745	2.265
SPAC26F1.05	mug106	0.3391	3.402	0.470	29.04	48.25	2.131	2.912
SPBP18G5.03	toc1	0.07756	3.409	1.110	26.95	44.31	2.064	1.167
SPCC1020.05	SPCC1020.05	0.5523	3.412	0.258	30.14	50.31	6.072	4.325
SPAC23D3.09	arp42	0.6668	3.413	0.176	19.09	29.51	9.809	5.452
SPBC15D4.05	SPBC15D4.05	0.3555	3.415	0.449	30.18	50.38	3.906	2.685
SPAC5H10.04	SPAC5H10.04	0.605	3.417	0.218	27.25	44.86	8.784	4.168
SPAC977.14C	SPAC977.14c	0.1598	3.419	0.796	21.77	34.54	2.693	1.576
SPBC27B12.03C	erg32	0.4473	3.419	0.349	33.45	56.52	4.098	3.53
SPAC19A8.02	SPAC19A8.02	0.3497	3.421	0.456	31.26	52.4	3.684	2.713
SPCC1020.12C	xap5	0.4003	3.422	0.398	24.83	40.29	4.542	2.898
SPAC1071.08	rpp203	0.7493	3.428	0.125	22.52	35.93	11.84	8.048
SPAC22G7.01C	SPAC22G7.01c	0.2697	3.435	0.569	27.64	45.56	4.525	1.32
SPAC140.02	gar2	0.5719	3.443	0.243	26.32	43.06	8.865	3.244
SPCC16A11.04	snx12	0.46	3.448	0.337	28.3	46.78	5.895	3.044
SPAC56F8.14C	mug115	0.2596	3.451	0.586	23.85	38.39	2.621	2.323
SPAP8A3.02C	ofd2	0.6397	3.452	0.194	25.81	42.08	9.393	4.917
SPBC28F2.03	ppi1	0.3023	3.463	0.520	28.38	46.9	2.223	2.691
SPBC651.04	SPBC651.04	0.545	3.467	0.264	32.49	54.62	5.124	4.586
SPBC21B10.09	SPBC21B10.09	0.7559	3.47	0.122	23.39	37.48	13.07	8.08
SPCC13B11.04C	SPCC13B11.04c	0.31	3.471	0.509	27.04	44.37	5.206	1.116
SPAC3C7.03C	rhp55	0.1192	3.474	0.924	23.76	38.17	2.694	1.261
SPBC21C3.12C	SPBC21C3.12c	0.6622	3.476	0.179	19.23	29.65	9.701	5.835
SPBCPT2R1.03	SPBCPT2R1.03	0.2702	3.48	0.568	26.06	42.51	2.744	2.396
SPBC1778.01C	zuo1	0.322	3.483	0.492	13.85	19.51	4.396	2.461
SPAC8F11.09C	nnt1	0.2899	3.485	0.538	30	49.91	4.157	2.032
SPAC26H5.05	SPAC26H5.05	0.658	3.488	0.182	17.7	26.75	8.321	6.01
SPAC23C4.05C	SPAC23C4.05c	0.4829	3.489	0.316	27.29	44.8	7.012	2.767
SPAC977.11	SPAC977.11	0.3053	3.494	0.515	26.37	43.05	2.13	2.75
SPCC1827.03C	SPCC1827.03c	0.2245	3.496	0.649	26.28	42.88	3.177	1.946
SPBC4F6.09	str1	0.3406	3.497	0.468	28.92	47.84	5.254	1.819
SPAC328.06	ubp2	0.3557	3.498	0.449	30.52	50.86	1.738	3.161
SPCC1259.11C	gyp2	0.3028	3.502	0.519	30.78	51.34	4.459	1.998
SPAC23A1.09	SPAC23A1.09	0.3272	3.514	0.485	29.65	49.18	5.413	1.353
SPBC428.06C	rxt2	0.7457	3.514	0.127	19.22	29.55	12.4	7.96
SPBC16C6.01C	SPBC16C6.01c	0.4075	3.516	0.390	14.15	20.02	4.633	3.288
SPAC6F6.02C	pof5	0.4185	3.52	0.378	28.53	47.07	6.544	1.916
SPBC215.11C	SPBC215.11c	0.3059	3.525	0.514	31.55	52.75	5.146	1.329
SPBC16A3.03C	ppr7	0.5229	3.53	0.282	27.72	45.53	7.026	3.625
SPBC947.08C	hip4	0.3865	3.533	0.413	27.77	45.62	3.918	3.108
SPAC3C7.13C	SPAC3C7.13c	0.6918	3.535	0.160	24.22	38.92	12.14	5.516
SPAC19G12.10C	cpy1	0.36	3.541	0.444	30.99	51.66	4.009	2.841
SPAC2F3.02	SPAC2F3.02	0.5696	3.543	0.244	27.96	45.96	8.555	3.728
SPBC56F2.03	SPBC56F2.03	0.4486	3.547	0.348	31.44	52.5	5.791	3.112
SPBP8B7.26	SPBP8B7.26	0.08516	3.548	1.070	27.74	45.53	2.651	0.1764
SPBC31F10.16	SPBC31F10.16	0.3122	3.55	0.506	26.69	43.55	2.666	2.777
SPAC24C9.02C	SPAC24C9.02c	0.2207	3.552	0.656	30.42	50.56	4.266	0.9161
SPBC800.04C	rpl4301	0.1695	3.561	0.771	4.928	2.573	2.573	1.798
SPAC1610.01	SPAC1610.01	0.6623	3.564	0.179	17.85	26.89	9.191	6.013
SPBC16E9.11C	pub3	0.4004	3.565	0.398	28.22	46.41	4.326	3.165
SPBC1A4.03C	top2	0.4781	3.567	0.320	29.71	49.21	6.458	3.235
SPBC1271.08C	SPBC1271.08c	0.2759	3.568	0.559	30.85	51.34	4.649	1.562
SPAC1006.04C	mcp3	0.1511	3.569	0.821	26.61	43.36	2.681	1.629

SPACUNK4.17	SPACUNK4.17	0.3048	3.592	0.516	29.12	48.04	5.18	1.438
SPCC613.06	rpl902	0.6431	3.601	0.192	12.67	17.06	7.474	6.15
SPCC1235.03	SPCC1235.03	0.5289	3.602	0.277	27.98	45.88	8.06	3.236
SPBC16C6.11	rpl3201	0.7251	3.603	0.140	13.55	18.73	10.83	7.851
SPAC6F6.03C	SPAC6F6.03c	0.2465	3.604	0.608	28.65	47.13	2.729	2.334
SPBC17D11.04C	nto1	0.1374	3.607	0.862	32	53.45	3.362	0.8516
SPBC16E9.06C	uvi31	0.2492	3.608	0.603	27.49	44.95	4.622	1.121
SPAC4F10.11	spn1	0.5027	3.61	0.299	30.83	51.24	8.221	2.371
SPBC29A10.14	rec8	0.1585	3.612	0.800	26.89	43.8	1.228	1.939
SPCC1739.01	SPCC1739.01	0.1814	3.618	0.741	4.617	1.88	1.88	2.046
SPBC19G7.06	mbx1	0.4622	3.618	0.335	23.87	38.13	3.996	3.981
SPAC3H1.03	mug151	0.1241	3.619	0.906	26.14	42.39	1.31	1.723
SPAC1296.05C	SPAC1296.05c	0.1199	3.627	0.921	27.51	44.95	1.273	1.7
SPCC13B11.01	adh1	0.4733	3.627	0.325	25.65	41.45	6.33	3.342
SPBC543.05C	SPBC543.05c	0.3331	3.63	0.477	28.54	46.89	5.822	1.018
SPAP27G11.15	slx1	0.3975	3.643	0.401	31.04	51.56	3.787	3.377
SPBC36B7.06C	mug20	0.7616	3.644	0.118	25.03	40.24	14.3	8.59
SPAC22H10.13	zym1	0.3454	3.646	0.462	26.07	42.21	2.403	3.158
SPBC2F12.15C	pfa3	0.2704	3.647	0.568	22.72	35.9	2.118	2.63
SPAC18B11.02C	SPAC18B11.02c	0.1855	3.656	0.732	26.39	42.79	2.58	1.978
SPBC1709.12	rid1	0.4588	3.658	0.338	30.55	50.61	3.527	4.08
SPBC1706.03	fzo1	0.2467	3.66	0.608	27.79	45.42	2.856	2.353
SPAC29E6.07	SPAC29E6.07	0.2539	3.662	0.595	29.51	48.66	4.734	1.177
SPAC328.01C	SPAC328.01c	0.3066	3.663	0.513	30.99	51.43	4.721	2.097
SPAC3A12.10	rpl2001	0.4687	3.664	0.329	15.51	22.29	7.542	2.394
SPAC328.05	SPAC328.05	0.1811	3.668	0.742	31	51.45	2.415	1.993
SPAC6G9.08	ubp6	0.2233	3.678	0.651	30.43	50.35	3.17	2.102
SPBP4H10.04	ppb1	0.232	3.68	0.635	30.07	49.68	4.198	1.582
SPCC576.12C	mhf2	0.03944	3.681	1.404	25.61	41.28	1.629	1.082
SPCC895.05	for3	0.4642	3.686	0.333	13.71	18.88	5.447	3.702
SPCC162.06C	SPCC162.06c	0.3589	3.687	0.445	29.98	49.48	3.677	3.091
SPCC162.03	SPCC162.03	0.1159	3.691	0.936	28.16	46.06	3.069	1.09
SPAC6F6.11C	SPAC6F6.11c	0.4111	3.692	0.386	29.56	48.69	3.178	3.681
SPCC1840.07C	SPCC1840.07c	0.2288	3.696	0.641	29.3	48.18	3.741	1.912
SPAC23H4.16C	SPAC23H4.16c	0.1123	3.697	0.950	25.76	41.53	2.456	1.492
SPAC25A8.03C	SPAC25A8.03c	0.1139	3.702	0.943	27.08	44	2.468	1.504
SPBC29A3.21	SPBC29A3.21	0.6989	3.705	0.156	30.42	50.29	11.69	6.686
SPAC15A10.07	SPAC15A10.07	0.5192	3.706	0.285	23.04	36.39	7.828	3.448
SPBC29A10.03C	pcf1	0.2999	3.711	0.523	29.72	48.96	4.988	1.856
SPAC18B11.03C	SPAC18B11.03c	0.7599	3.711	0.119	25.93	41.82	14.1	8.842
SPAC12B10.15C	SPAC12B10.15c	0.4544	3.713	0.343	30.52	50.45	5.833	3.454
SPAC11D3.09	SPAC11D3.09	0.6208	3.715	0.207	32.11	53.45	4.826	6.438
SPBC1773.02C	SPBC1773.02c	0.2953	3.717	0.530	32.57	54.31	5.067	1.705
SPBC19G7.18C	SPBC19G7.18c	0.5125	3.717	0.290	25.6	41.19	7.554	3.514
SPCC830.10	SPCC830.10	0.3337	3.72	0.477	28.76	47.14	5.816	1.454
SPAC29A4.18	prw1	0.5114	3.723	0.291	25.51	41	6.779	4.186
SPAC22F8.04	SPAC22F8.04	0.1928	3.724	0.715	17.1	25.17	2.208	2.147
SPAC23A1.07	SPAC23A1.07	0.2993	3.724	0.524	18.41	27.63	5.556	0.717
SPBC30D10.04	swi3	0.2411	3.727	0.618	22.85	35.98	1.897	2.509
SPAC23C11.13C	hpt1	0.5078	3.727	0.294	20	30.63	7.203	3.656
SPCC962.05	ast1	0.003368	3.732	2.473	26.77	43.37	1.034	0.575
SPCC794.12C	mae2	0.5888	3.735	0.230	26.09	42.07	7.778	5.053
SPCC24B10.17	emp24	0.1941	3.739	0.712	24.28	38.67	1.999	2.191
SPAC3F10.10C	map3	0.396	3.74	0.402	30.3	50	3.541	3.525
SPAC22H10.03C	kap114	0.3299	3.745	0.482	28.58	46.74	3.904	2.825
SPCC31H12.08C	ccr4	0.7297	3.748	0.137	24.84	39.69	11.02	8.468
SPCC737.03C	ima1	0.0909	3.757	1.041	29.02	47.54	1.986	1.493
SPCC306.07C	SPCC306.07c	0.3169	3.761	0.499	26.77	43.3	3.425	2.86
SPCC126.10	iah1	0.2158	3.77	0.666	30.71	50.7	4.299	1.344
SPCC1183.11	SPCC1183.11	0.53	3.771	0.276	23.23	36.63	5.954	4.638
SPBC8D2.16C	SPBC8D2.16c	0.07891	3.773	1.103	26.37	42.52	1.777	1.446
SPAC1486.02C	dsc2	0.5994	3.776	0.222	16.09	23.17	7.564	5.461
SPBC2D10.14C	myo51	0.2558	3.78	0.592	33.64	56.2	4.806	1.419

SPBC2D10.16	mhf1	0.07112	3.782	1.148	27.65	44.93	2.599	0.9645
SPCC417.02	dad5	0.692	3.785	0.160	20.81	32.05	10.82	7.035
SPAC5H10.07	SPAC5H10.07	0.1869	3.787	0.728	31.15	51.51	3.719	1.574
SPAC23G3.12C	SPAC23G3.12c	0.299	3.791	0.524	29.05	47.55	5.176	1.797
SPAC4F10.02	aap1	0.1227	3.793	0.911	27.42	44.48	1.455	1.793
SPCC1450.05C	rox3	0.4893	3.793	0.310	28.25	46.04	5.897	4.062
SPBC11C11.02	imp2	0.2682	3.794	0.572	28.17	45.88	3.323	2.515
SPAC20G8.10C	atg6	0.2504	3.799	0.601	28.41	46.32	3.444	2.337
SPCC1281.04	SPCC1281.04	0.2794	3.799	0.554	22.49	35.18	4.449	2.145
SPBC800.02	whi5	0.1784	3.802	0.749	29.47	48.3	3.296	1.777
SPCC1281.08	wtf11	0.6279	3.806	0.202	28.52	46.52	9.921	5.272
SPAC222.12C	atp2	0.2697	3.809	0.569	25.53	40.89	2.711	2.674
SPBC776.04	sec2302	0.2173	3.813	0.663	26.93	43.51	3.249	2.143
SPAC1142.06	get3	0.22	3.814	0.658	29.92	49.14	2.027	2.414
SPBC365.06	pmt3	0.2594	3.814	0.586	26.41	42.53	2.53	2.63
SPCC23B6.02C	SPCC23B6.02c	0.3121	3.815	0.506	31.73	52.54	4.91	2.26
SPAC29A4.09	SPAC29A4.09	0.3623	3.815	0.441	7.654	7.226	4.917	2.852
SPAC1F5.03C	SPAC1F5.03c	0.29	3.818	0.538	32.66	54.29	4.97	1.912
SPCC1183.09C	pmp31	0.4479	3.826	0.349	30.72	50.61	4.525	3.973
SPAC11E3.03	pcs1	0.422	3.832	0.375	21.47	33.19	6.328	2.899
SPCC965.12	SPCC965.12	0.3811	3.835	0.419	27.98	45.44	6.543	1.908
SPBC405.07	rpl3602	0.663	3.835	0.178	18.15	26.94	9.919	6.483
SPBC354.04	SPBC354.04	0.4459	3.837	0.351	30.89	50.92	4.063	4.066
SPAC926.06C	SPAC926.06c	0.4564	3.842	0.341	30.46	50.1	5.29	3.885
SPAPB1A10.15	arv1	0.2994	3.844	0.524	29.32	47.96	5.461	1.54
SPAC8C9.19	SPAC8C9.19	0.2884	3.846	0.540	21.37	32.99	4.918	1.982
SPAPB1E7.06C	eme1	0.4611	3.847	0.336	11.67	14.73	5.185	3.988
SPAC57A7.13	SPAC57A7.13	0.1183	3.85	0.927	27.77	45.02	2.078	1.729
SPAC21E11.05C	cyp8	0.2511	3.854	0.600	30.84	50.78	3.808	2.256
SPBC216.05	rad3	0.2311	3.857	0.636	27.15	43.84	3.214	2.304
SPAC14C4.06C	SPAC14C4.06c	0.4852	3.863	0.314	18.73	27.98	7.268	3.469
SPBC354.07C	SPBC354.07c	0.254	3.867	0.595	27.63	44.73	3.785	2.306
SPAC23C11.01	SPAC23C11.01	0.1636	3.871	0.786	26.1	41.84	2.697	1.938
SPBC216.01C	SPBC216.01c	0.5013	3.872	0.300	31.25	51.53	6.065	4.316
SPAPB1A10.09	ase1	0.2341	3.88	0.631	29.04	47.35	3.629	2.203
SPBC8D2.12C	SPBC8D2.12c	0.2713	3.885	0.567	30.73	50.52	2.762	2.74
SPCC126.01C	SPCC126.01c	0.2601	3.886	0.585	22.29	34.63	3.814	2.37
SPCC645.08C	snd1	0.5826	3.894	0.235	35.45	59.39	10.39	3.713
SPAC589.09	SPAC589.09	0.1699	3.895	0.770	28.54	46.39	3.646	1.552
SPBC3D6.10	apn2	0.3516	3.907	0.454	24.18	38.16	4.755	2.922
SPAC23G3.05C	SPAC23G3.05c	0.3628	3.907	0.440	26.3	42.15	6.322	1.96
SPBC30B4.01C	wsc1	0.7316	3.907	0.136	25.6	40.83	14.21	7.817
SPAC823.14	ptf1	0.2718	3.909	0.566	32.76	54.3	3.997	2.427
SPBC21C3.19	SPBC21C3.19	0.4768	3.91	0.322	24.4	38.56	5.712	4.128
SPAC2C4.07C	SPAC2C4.07c	0.2545	3.917	0.594	28.54	46.35	1.875	2.743
SPAC652.01	SPAC652.01	0.5377	3.918	0.269	26.35	42.22	7.543	4.726
SPAC8C9.12C	SPAC8C9.12c	0.7372	3.921	0.132	24.94	39.57	13.26	8.622
SPAC17G8.13C	mst2	0.2404	3.928	0.619	33.05	54.82	5.264	1.467
SPBC21C3.17C	SPBC21C3.17c	0.3403	3.928	0.468	31.58	52.04	3.965	3.101
SPBC18E5.08	SPBC18E5.08	0.1299	3.929	0.886	27	43.43	2.795	1.67
SPBC1734.07C	SPBC1734.07c	0.3686	3.929	0.433	29.63	48.37	4.856	3.098
SPAC4D7.01C	sec71	0.02902	3.933	1.537	34.98	58.43	1.771	1.005
SPBC409.07C	wis1	0.4694	3.934	0.328	24.86	39.38	7.734	2.955
SPAC9G1.11C	spn4	0.3905	3.935	0.408	32.21	53.21	6.617	2.299
SPAC17C9.12	SPAC17C9.12	0.1893	3.938	0.723	22.22	34.41	3.647	1.821
SPAC18G6.10	lem2	0.1686	3.942	0.773	29.14	47.43	3.118	1.89
SPCC1739.07	cti1	0.3491	3.942	0.457	14.86	20.55	3.501	3.307
SPAC922.03	SPAC922.03	0.2168	3.945	0.664	27.13	43.64	2.725	2.383
SPBC3E7.02C	hsp16	0.2673	3.945	0.573	29.65	48.38	3.222	2.667
SPAC22F8.12C	shf1	0.4349	3.948	0.362	22.84	35.55	5.786	3.571
SPAP7G5.05	rpl1002	0.5405	3.951	0.267	26.23	41.94	8.141	4.286
SPCC364.04C	SPCC364.04c	0.1311	3.952	0.882	34.53	57.56	3.623	0.8305
SPBC83.11	SPBC83.11	0.4111	3.953	0.386	32.19	53.15	6.555	2.786

SPAC23H3.12C	SPAC23H3.12c	0.331	3.966	0.480	30.21	49.4	4.417	2.907
SPAC5H10.10	SPAC5H10.10	0.3264	3.969	0.486	31.15	51.15	4.908	2.644
SPBC582.06C	mcp6	0.7433	3.97	0.129	22.37	34.63	13.41	9.087
SPBC2D10.06	rep1	0.2758	3.971	0.559	26.62	42.62	5.029	1.896
SPBP35G2.04C	SPBP35G2.04c	0.7062	3.975	0.151	24.92	39.43	13.9	6.818
SPAC11E3.08C	nse6	0.2831	3.976	0.548	22.06	34.03	5.273	1.766
SPBC146.09C	lsd1	0.09196	3.978	1.036	24.6	38.81	1.214	1.666
SPCC13B11.03C	SPCC13B11.03c	0.2273	3.98	0.643	26.33	42.07	2.354	2.544
SPBC19F8.02	SPBC19F8.02	0.2201	3.995	0.657	27.84	44.88	4.381	1.711
SPAC5D6.01	rps2202	0.3384	4.001	0.471	27.42	44.07	5.013	2.774
SPAC22F3.03C	rdh54	0.4069	4.006	0.391	31.77	52.26	4.337	3.775
SPCC5E4.05C	SPCC5E4.05c	0.1756	4.01	0.755	26.56	42.43	3.175	1.982
SPBC342.04	rpn1301	0.3144	4.01	0.503	31.39	51.54	3.063	3.147
SPAC1F12.04C	SPAC1F12.04c	0.1744	4.02	0.758	28.96	46.95	3.931	1.507
SPBC83.02C	rpl4302	0.7261	4.02	0.139	16.89	24.22	13	8.47
SPAC222.15	meu13	0.333	4.025	0.478	29.16	47.3	5.642	2.342
SPBC32H8.13C	mok12	0.5964	4.025	0.224	24.96	39.4	9.881	4.926
SPAC29B12.02C	set2	0.176	4.029	0.754	31.52	51.74	4.126	1.295
SPAC1687.06C	rpl44	0.1129	4.031	0.947	26.07	41.48	1.716	1.826
SPBC2D10.09	SPBC2D10.09	0.4297	4.036	0.367	30.41	49.64	7.928	1.895
SPBC800.10C	SPBC800.10c	0.3321	4.043	0.479	28.14	45.36	5.643	2.357
SPBC36B7.02	SPBC36B7.02	0.08552	4.045	1.068	27.42	44	2.957	1.114
SPAC8E11.03C	dmc1	0.09395	4.046	1.027	28.24	45.53	2.346	1.582
SPBC27.06C	mgr2	0.1459	4.048	0.836	9.587	10.42	3.722	1.303
SPCC330.11	btb1	0.7265	4.048	0.139	23.93	37.42	13.34	8.444
SPAC644.13C	SPAC644.13c	0.2401	4.049	0.620	29.68	48.24	4.522	1.945
SPBPJ4664.03	mfm3	0.2595	4.05	0.586	29.15	47.24	3.567	2.604
SPAC1D4.11C	lkh1	0.2765	4.056	0.558	31.04	50.78	3.867	2.667
SPCP31B10.06	mug190	0.5693	4.056	0.245	33.44	55.31	9.266	4.6
SPAC227.03C	SPAC227.03c	0.2795	4.057	0.554	32.91	54.31	4.852	2.226
SPBC3B8.05	SPBC3B8.05	0.03741	4.059	1.427	29.43	47.75	2.2	0.9525
SPBC1711.08	SPBC1711.08	0.1657	4.06	0.781	29.14	47.2	2.668	2.09
SPAC16E8.12C	SPAC16E8.12c	0.3878	4.065	0.411	31.71	52.03	5.324	3.315
SPAC6B12.09	trm10	0.4025	4.065	0.395	31.47	51.58	3.04	4.025
SPCC569.05C	SPCC569.05c	0.265	4.071	0.577	29.39	47.65	5.122	1.793
SPBP8B7.11	nxt3	0.2182	4.075	0.661	28.24	45.48	2.368	2.543
SPAC139.02C	oac1	0.1939	4.078	0.712	30.5	49.74	3.882	1.871
SPBC17A3.10	pas4	0.4691	4.079	0.329	19.95	29.87	6.91	3.793
SPCC24B10.22	pog1	0.2662	4.08	0.575	31.62	51.84	3.325	2.751
SPAC2E1P5.02C	mug109	0.2175	4.081	0.663	28.03	45.07	4.809	1.196
SPAC19A8.05C	sst4	0.4938	4.081	0.306	31.18	51.01	3.562	5.059
SPBC2G2.13C	SPBC2G2.13c	0.351	4.084	0.455	31.37	51.35	6.085	2.371
SPAC1399.05C	SPAC1399.05c	0.3831	4.089	0.417	28.18	45.34	6.478	2.613
SPAC23A1.11	rpl1602	0.02599	4.091	1.585	27.67	44.38	1.605	1.073
SPCC132.01C	SPCC132.01c	0.1426	4.099	0.846	26.06	41.34	2.662	1.935
SPCC1739.12	ppe1	0.2996	4.105	0.523	28.56	46.03	4.691	2.619
SPAC806.04C	SPAC806.04c	0.4069	4.107	0.391	32.24	52.94	4.518	3.853
SPBC2A9.05C	tvp23	0.3352	4.113	0.475	27	43.08	6.191	2.013
SPBP4H10.18C	SPBP4H10.18c	0.2813	4.117	0.551	31.28	51.12	5.341	1.931
SPBC31F10.08	mde2	0.08676	4.124	1.062	27.03	43.11	2.446	1.528
SPBC32H8.03	bem46	0.4961	4.124	0.304	28.12	45.16	8.442	3.469
SPAC14C4.03	mek1	0.214	4.125	0.670	25.49	40.21	2.767	2.486
SPAC14C4.08	mug5	0.2221	4.125	0.653	26.98	43.01	3.155	2.465
SPBC651.07	EMPTY	0.2526	4.127	0.598	29.17	47.14	3.202	2.707
SPAC17C9.16C	SPAC17C9.16c	0.2894	4.133	0.539	30.5	49.62	5.556	1.871
SPAC17A2.14	SPAC17A2.14	0.3199	4.133	0.495	28.99	46.78	3.91	3.134
SPAC12B10.11	exg2	0.2912	4.136	0.536	27.78	44.5	5.037	2.361
SPCC736.08	cbf11	0.05854	4.137	1.233	25.81	40.8	1.771	1.43
SPAC25H1.06	pcf3	0.3645	4.142	0.438	34.08	56.35	4.58	3.406
SPAC3G9.01	nsk1	0.4648	4.143	0.333	27.36	43.69	7.668	3.384
SPBP35G2.13C	swc2	0.1	4.146	1.000	29.99	48.65	3.374	0.8281
SPAC27D7.08C	SPAC27D7.08c	0.1149	4.146	0.940	25.86	40.87	2.702	1.717
SPCC1450.03	SPCC1450.03	0.7502	4.149	0.125	19.73	29.33	13.76	10.01

SPCC622.16C	epe1	0.2265	4.151	0.645	33.07	54.42	4.836	1.561
SPCC70.10	SPCC70.10	0.6419	4.153	0.193	28.16	45.19	11.14	6.078
SPAC4A8.09C	cwf21	0.4091	4.157	0.388	29.42	47.56	4.066	4.037
SPAC13D6.01	pof14	0.2501	4.158	0.602	26.61	42.25	2.398	2.841
SPAC664.12C	SPAC664.12c	0.1172	4.163	0.931	31.46	51.37	3.174	1.524
SPBC776.06C	SPBC776.06c	0.7293	4.169	0.137	22.48	34.46	12.65	9.265
SPBC3E7.12C	chr1	0.3414	4.172	0.467	28.67	46.1	6.592	1.749
SPAC17A5.08	SPAC17A5.08	0.6899	4.175	0.161	19.52	28.88	11.78	7.727
SPBC1709.18	tif452	0.1592	4.176	0.798	33.05	54.34	3.897	1.515
SPAC8E11.04C	SPAC8E11.04c	0.3795	4.179	0.421	28.66	46.07	6.107	2.977
SPBC18E5.04	rpl1001	0.5095	4.181	0.293	19.21	28.28	6.473	4.824
SPCC285.04	SPCC285.04	0.3239	4.187	0.490	27.73	44.3	4.558	3.032
SPAC11E3.06	map1	0.4042	4.189	0.393	32.67	53.6	5.797	3.502
SPAC2F7.08C	snf5	0.5535	4.191	0.257	33.17	54.54	10.38	3.664
SPBC18H10.15	ppk23	0.3149	4.194	0.502	30.21	48.97	4.574	2.938
SPAC3G9.04	ssu72	0.1244	4.196	0.905	29.05	46.78	3.445	1.441
SPCC330.14C	rpl2402	0.7007	4.197	0.154	23.81	36.92	11.89	8.228
SPCC24B10.19C	SPCC24B10.19c	0.3276	4.2	0.485	30.28	49.1	5.217	2.801
SPBC4.05	mlo2	0.1703	4.205	0.769	28.89	46.46	4.41	0.759
SPAC3C7.09	set8	0.1983	4.207	0.703	27.38	43.62	2.932	2.388
SPBC342.05	crb2	0.1813	4.209	0.742	27.86	44.52	2.582	2.324
SPBC3E7.07C	SPBC3E7.07c	0.301	4.211	0.521	29.3	47.22	3.782	3.067
SPBC17D11.08	SPBC17D11.08	0.06191	4.214	1.208	27.48	43.79	0.6498	1.481
SPAC17G6.08	pep7	0.3953	4.214	0.403	32.24	52.75	5.865	3.385
SPAC2E1P3.02C	amt3	0.4191	4.215	0.378	30.39	49.26	6.759	3.27
SPAC16E8.18	SPAC16E8.18	0.1087	4.219	0.964	27.58	43.97	2.914	1.618
SPAC328.04	SPAC328.04	0.3946	4.22	0.404	30.54	49.54	5.469	3.552
SPAC1556.08C	cbs2	0.3944	4.222	0.404	22.65	34.69	5.502	3.801
SPCC1840.02C	bgs4	0.3009	4.224	0.522	31.15	50.68	5.923	1.847
SPAC19G12.03	cda1	0.2664	4.227	0.574	31.48	51.29	4.299	2.579
SPBC29A10.06C	ely5	0.1598	4.235	0.796	27.75	44.27	2.632	2.165
SPBPB2B2.10C	gal7	0.03685	4.25	1.434	24.42	37.97	2.364	0.8988
SPBC83.16C	SPBC83.16c	0.3696	4.255	0.432	28.23	45.12	6.404	2.745
SPCC1919.07	SPCC1919.07	0.3159	4.264	0.500	28.42	45.47	5.712	2.417
SPAC3G6.11	chl1	0.6679	4.265	0.175	25.42	39.83	13.98	5.813
SPAC23A1.04C	mnl1	0.1507	4.269	0.822	27.5	43.73	2.879	2.055
SPAC589.11	mug82	0.08215	4.282	1.085	23.96	37.03	3.225	0.6822
SPCC126.02C	pku70	0.3658	4.284	0.437	25.68	40.27	5.907	3.055
SPAC3A12.17C	cys12	0.002054	4.286	2.687	28.26	45.11	1.086	0.2679
SPAC3G9.05	SPAC3G9.05	0.2655	4.286	0.576	27.44	43.59	2.468	3.052
SPBC947.02	apl2	0.2944	4.286	0.531	31.51	51.24	3.771	3.078
SPAC4H3.02C	swc3	0.6529	4.287	0.185	27.04	42.82	10.49	7.112
SPBC409.16C	SPBC409.16c	0.3401	4.29	0.468	27.55	43.77	6.301	2.37
SPBC3B9.11C	ctf1	0.668	4.29	0.175	29.26	47	12.41	6.863
SPAC22E12.03C	SPAC22E12.03c	0.1782	4.292	0.749	23.01	35.23	4.058	1.791
SPBC428.08C	clr4	0.3282	4.292	0.484	26.37	41.56	5.803	2.583
SPBC1685.15C	klp6	0.01083	4.301	1.965	26.69	42.13	1.628	0.8009
SPAC23C11.02C	rps23	0.6168	4.302	0.210	20.74	30.94	8.805	6.623
SPBC660.08	SPBC660.08	0.2762	4.31	0.559	34.14	56.15	4.909	2.477
SPAC20H4.10	ufd2	0.2022	4.319	0.694	34.74	57.26	4.883	1.224
SPAC4G9.20C	SPAC4G9.20c	0.1635	4.323	0.786	26.33	41.41	2.592	2.258
SPAC824.05	vps16	0.4852	4.324	0.314	21.09	31.56	7.127	4.685
SPAC13G7.11	SPAC13G7.11	0.2246	4.33	0.649	35.04	57.79	5.291	0.9783
SPAC1952.02	SPAC1952.02	0.6507	4.33	0.187	21.05	31.47	10.66	7.08
SPBP4H10.16C	SPBP4H10.16c	0.4832	4.334	0.316	31.25	50.65	8.093	3.886
SPCC825.04C	naa40	0.2655	4.336	0.576	26.72	42.14	4.754	2.467
SPBC1773.01	SPBC1773.01	0.3444	4.338	0.463	28.76	45.97	4.159	3.525
SPCC24B10.09	rps1702	0.3569	4.338	0.447	19.96	29.4	6.507	2.59
SPBC577.04	SPBC577.04	0.384	4.341	0.416	28.79	46.01	4.018	3.978
SPBC337.10C	SPBC337.10c	0.1419	4.343	0.848	26.08	40.9	2.392	2.132
SPCC24B10.15	SPCC24B10.15	0.3514	4.343	0.454	31.81	51.7	4.385	3.546
SPBP23A10.10	ppk32	0.3158	4.344	0.501	27.4	43.39	4.144	3.245
SPBC1289.10C	adn2	0.5535	4.344	0.257	26.32	41.36	10.12	4.335

SPBC15D4.15	pho2	0.1679	4.35	0.775	30.64	49.49	4.197	1.579
SPCC31H12.04C	rpl1202	0.4491	4.352	0.348	21.34	31.97	7.568	3.56
SPBC1271.06C	mug96	0.2465	4.353	0.608	26.81	42.27	1.878	2.997
SPAC26A3.04	rpl2002	0.2065	4.358	0.685	25.86	40.47	4.478	1.926
SPAC1952.07	rad1	0.06792	4.363	1.168	26.24	41.18	2.549	1.419
SPAC6C3.02C	SPAC6C3.02c	0.2303	4.369	0.638	32.4	52.76	5.207	1.554
SPCC16A11.16C	rpn1302	0.3947	4.37	0.404	28.47	45.36	6.803	3.095
SPAC5D6.08C	mes1	0.2497	4.375	0.603	31.26	50.6	5.36	1.795
SPBC9B6.11C	SPBC9B6.11c	0.08151	4.38	1.089	29	46.33	2.469	1.611
SPBC3B8.04C	SPBC3B8.04c	0.3033	4.383	0.518	32.66	53.22	4.497	3.048
SPBC2A9.07C	SPBC2A9.07c	0.122	4.387	0.914	26.9	42.37	2.216	2.024
SPCC126.09	SPCC126.09	0.2503	4.387	0.602	30.62	49.38	5.438	1.725
SPBPJ4664.06	gpt1	0.4194	4.388	0.377	31	50.09	5.013	4.226
SPBC4F6.11C	SPBC4F6.11c	0.07929	4.39	1.101	27	42.56	2.822	1.441
SPAC1F3.06C	spo15	0.057	4.393	1.244	28.91	46.14	1.541	1.532
SPAC15E1.05C	SPAC15E1.05c	0.6134	4.394	0.212	23.35	35.67	12.35	4.946
SPBC887.02	SPBC887.02	0.03834	4.399	1.416	26.83	42.22	1.908	1.288
SPCC74.06	mak3	0.2742	4.4	0.562	42.72	72.13	6.089	1.188
SPAC15A10.09C	SPAC15A10.09c	0.5007	4.4	0.300	25.81	40.29	7.6	4.62
SPBC839.06	cta3	0.2676	4.401	0.573	32.7	53.27	3.257	3.049
SPBC800.12C	SPBC800.12c	0.3228	4.401	0.491	30.58	49.27	4.573	3.248
SPBC530.03C	bag102	0.155	4.402	0.810	30.81	49.7	3.844	1.764
SPBC2F12.03C	SPBC2F12.03c	0.2389	4.406	0.622	29.48	47.19	3.285	2.799
SPAC1805.08	dlc1	0.267	4.411	0.573	31.52	51.03	4.603	2.646
SPBP16F5.05C	SPBP16F5.05c	0.4647	4.413	0.333	18.47	26.45	6.812	4.327
SPCPB16A4.02C	SPCPB16A4.02c	0.3753	4.416	0.426	24.68	38.14	6.092	3.286
SPAC6F6.17	rif1	0.3264	4.417	0.486	28.59	45.49	6.41	2.241
SPAC13G7.07	arb2	0.06847	4.428	1.164	25.48	39.62	2.508	1.481
SPBC2G2.03C	sbh1	0.7063	4.434	0.151	15.63	21.08	12.28	9.062
SPCC1183.02	SPCC1183.02	0.1814	4.436	0.741	27.42	43.26	2.42	2.495
SPBC31F10.07	lsb5	0.04789	4.443	1.320	27.12	42.68	2.742	0.8478
SPBC119.14	rti1	0.1344	4.448	0.872	30.53	49.09	4.104	1.039
SPBC1734.15	rsc4	0.3471	4.455	0.460	31.08	50.12	4.884	3.476
SPBC1861.09	ppk22	0.4128	4.463	0.384	28.02	44.33	6.508	3.71
SPCC191.06	SPCC191.06	0.1514	4.464	0.820	30.07	48.19	3.795	1.813
SPCC1739.08C	SPCC1739.08c	0.1231	4.47	0.910	31.65	51.16	2.751	1.976
SPAC144.05	SPAC144.05	0.1691	4.47	0.772	27.03	42.45	1.801	2.471
SPAC16E8.06C	nop12	0.118	4.474	0.928	30.32	48.65	3.9	0.9768
SPAC13D6.04C	btb3	0.2158	4.481	0.666	26.1	40.7	3.791	2.513
SPAC513.04	SPAC513.04	0.3631	4.482	0.440	32.22	52.21	4.266	3.85
SPAC12B10.14C	ppk2	0.1249	4.491	0.903	28.56	45.31	3.874	1.331
SPAC14C4.05C	man1	0.159	4.492	0.799	26.15	40.76	2.293	2.367
SPAC27D7.09C	SPAC27D7.09c	0.3753	4.498	0.426	33.16	53.95	5.686	3.584
SPAC11D3.18C	SPAC11D3.18c	0.5072	4.498	0.295	24.96	38.52	8.661	4.744
SPCC24B10.21	tpi1	0.03085	4.499	1.511	28.7	45.55	2.18	1.106
SPAC1834.05	alg9	0.08507	4.501	1.070	26.75	41.87	3.285	1.236
SPBP22H7.08	rps1002	0.1827	4.507	0.738	33.03	53.69	4.509	1.728
SPAPB24D3.09C	pdr1	0.2482	4.51	0.605	27.36	43.01	2.929	3.023
SPBC32F12.07C	SPBC32F12.07c	0.1992	4.515	0.701	29.05	46.17	4.626	1.898
SPAC1F8.06	fta5	0.09074	4.528	1.042	33.1	53.77	3.494	1.076
SPAC26H5.09C	SPAC26H5.09c	0.1377	4.542	0.861	26.98	42.24	2.246	2.23
SPBC32H8.01C	SPBC32H8.01c	0.3161	4.543	0.500	32.17	52	4.229	3.424
SPAC977.15	SPAC977.15	0.3225	4.549	0.491	27	42.26	6.385	2.446
SPAC1142.02C	SPAC1142.02c	0.07441	4.552	1.128	27.14	42.52	2.187	1.694
SPBC16A3.10	SPBC16A3.10	0.09331	4.566	1.030	29.11	46.2	3.041	1.624
SPAC167.04	pam17	0.5372	4.566	0.270	30.14	48.13	8.459	5.324
SPAC23A1.17	SPAC23A1.17	0.03023	4.575	1.520	27.8	43.72	2.112	1.168
SPAC1952.09C	SPAC1952.09c	0.2877	4.575	0.541	33.89	55.17	4.474	3.088
SPBC19G7.10C	SPBC19G7.10c	0.1384	4.582	0.859	19.16	27.43	0.6833	2.292
SPCC24B10.10C	SPCC24B10.10c	0.1438	4.582	0.842	27.86	43.81	2.293	2.296
SPAC222.08C	SPAC222.08c	0.1435	4.583	0.843	22.11	32.99	3.752	1.854
SPCC16A11.01	SPCC16A11.01	0.1438	4.589	0.842	26.52	41.28	3.302	2.077
SPAC1805.04	nup132	0.3897	4.59	0.409	27.59	43.28	5.312	4.015

SPBP35G2.11C	SPBP35G2.11c	0.243	4.596	0.614	21.95	32.66	3.573	2.924
SPBP4H10.13	rps2302	0.297	4.6	0.527	24.06	36.62	5.696	2.648
SPACUNK4.14	mdb1	0.2428	4.602	0.615	29.82	47.47	5.483	1.929
SPAC1006.06	rgf2	0.2679	4.602	0.572	31.86	51.31	4.802	2.771
SPCC1742.01	SPCC1742.01	0.3643	4.603	0.439	28.63	45.23	6.875	2.911
SPBC947.05C	frp2	0.297	4.606	0.527	29.38	46.63	6.386	2.016
SPBC28F2.02	mep33	0.3262	4.61	0.487	36.19	59.43	3.991	3.649
SPBC29A10.02	spo5	0.07738	4.615	1.111	28.44	44.84	2.141	1.759
SPBC56F2.01	pof12	0.7228	4.622	0.141	17.22	23.71	13.75	9.989
SPBC32F12.08C	duo1	0.5174	4.626	0.286	16.03	21.47	7.205	5.474
SPBC32F12.02	rec14	0.2172	4.63	0.663	25.75	39.76	5.065	1.943
SPBC19F8.06C	meu22	0.1938	4.635	0.713	31.46	50.49	3.552	2.507
SPAC6B12.04C	SPAC6B12.04c	0.1159	4.649	0.936	26.32	40.79	1.629	2.147
SPBC1271.05C	SPBC1271.05c	0.3308	4.656	0.480	22.91	34.35	4.867	3.519
SPAC3H5.09C	SPAC3H5.09c	0.167	4.666	0.777	26.93	41.9	2.502	2.511
SPBC2A9.06C	SPBC2A9.06c	0.2345	4.671	0.630	28.11	44.12	4.685	2.51
SPAC1B3.03C	wis2	0.1888	4.674	0.724	30.59	48.79	4.513	2.032
SPCC645.07	rgf1	0.2882	4.675	0.540	24.04	36.45	6.134	2.257
SPBC1703.04	mlh1	0.4531	4.678	0.344	32.22	51.84	9.637	2.452
SPCC285.15C	rps2802	0.4583	4.679	0.339	24.36	37.05	7.58	4.314
SPBC3H7.11	SPBC3H7.11	0.2411	4.681	0.618	29.44	46.6	5.986	1.04
SPAC1A6.01C	SPAC1A6.01c	0.2673	4.698	0.573	29.52	46.71	6.156	1.792
SPAC29A4.05	cam2	0.24	4.707	0.620	27.09	42.13	5.655	1.855
SPBC13G1.14C	SPBC13G1.14c	0.6689	4.708	0.175	23.94	36.19	12.44	8.102
SPBC106.16	pre6	0.1367	4.712	0.864	27.21	42.35	2.063	2.33
SPAC11G7.04	ubi1	0.3361	4.713	0.474	18.25	25.47	6.573	2.825
SPCC553.12C	SPCC553.12c	0.1214	4.726	0.916	25.33	38.79	2.14	2.203
SPBC1709.16C	SPBC1709.16c	0.5694	4.729	0.245	28.41	44.57	10.51	5.526
SPBC2D10.17	clr1	0.1833	4.731	0.737	30.55	48.6	5.059	1.319
SPAC6G10.08	idp1	0.5294	4.731	0.276	32.18	51.66	10.11	4.612
SPAC4F10.20	grx1	0.03356	4.732	1.474	28.48	44.71	0.4368	1.299
SPAC1527.03	SPAC1527.03	0.2078	4.739	0.682	30.01	47.56	2.516	2.895
SPAC3H1.11	hsr1	0.08513	4.74	1.070	28.4	44.53	0.6961	1.894
SPAP14E8.04	oma1	0.06184	4.747	1.209	27.18	42.22	1.048	1.691
SPBC336.06C	rnh1	0.2057	4.749	0.687	31.18	49.74	4.895	2.075
SPBC24C6.10C	dip1	0.175	4.752	0.757	33.43	53.97	4.303	2.073
SPBC4B4.08	ght2	0.1992	4.753	0.701	28.92	45.49	2.3	2.852
SPBC24C6.06	gpa1	0.5661	4.757	0.247	29.4	46.38	9.962	6.119
SPAC26A3.17C	SPAC26A3.17c	0.1229	4.759	0.910	32.27	51.78	2.852	2.122
SPAC23H3.04	SPAC23H3.04	0.3675	4.764	0.435	30.77	48.95	4.573	4.135
SPACUNK4.13C	SPACUNK4.13c	0.2331	4.767	0.632	30.99	49.36	5.073	2.369
SPAC6B12.14C	SPAC6B12.14c	0.03355	4.77	1.474	30.07	47.61	2.415	1.166
SPBC1861.07	SPBC1861.07	0.2053	4.771	0.688	29.51	46.57	4.924	2.073
SPAC17G6.06	rps2401	0.4863	4.776	0.313	26.32	40.56	8.809	4.42
SPAC144.11	rps1102	0.4412	4.777	0.355	22.82	33.96	7.067	4.399
SPAC688.11	end4	0.2539	4.789	0.595	13.76	16.89	4.844	2.786
SPBC19C2.04C	ubp11	0.165	4.793	0.783	26.07	40.05	2.565	2.563
SPBC1734.13	atp3	0.2024	4.798	0.694	26.75	41.31	2.01	2.928
SPBC2D10.13	est1	0.626	4.801	0.203	21.8	31.99	10.85	7.328
SPAC17G8.10C	dma1	0.08586	4.803	1.066	32.1	51.38	3.66	0.5652
SPAC1F5.05C	SPAC1F5.05c	0.1975	4.818	0.704	30.59	48.5	3.55	2.682
SPAPB1A11.04C	SPAPB1A11.04c	0.3314	4.822	0.480	33.8	54.54	4.106	3.887
SPAC11H11.01	sst6	0.08073	4.823	1.093	28.55	44.65	0.8192	1.891
SPAC926.07C	dlc2	0.3883	4.825	0.411	33.18	53.38	8.053	2.825
SPBC13E7.03C	SPBC13E7.03c	0.07411	4.829	1.130	28.12	43.84	1.393	1.856
SPAC2F3.05C	SPAC2F3.05c	0.4103	4.834	0.387	30.67	48.63	4.614	4.732
SPAC823.03	ppk15	0.177	4.847	0.752	30.13	47.58	2.23	2.729
SPBC106.03	SPBC106.03	0.2924	4.851	0.534	28.02	43.6	5.357	3.09
SPAC4F10.05C	SPAC4F10.05c	0.1488	4.854	0.827	5.766	1.718	1.386	2.525
SPAC1002.18	urg3	0.1512	4.86	0.820	27.22	42.09	2.756	2.456
SPBC56F2.08C	SPBC56F2.08c	0.1123	4.876	0.950	25.58	38.97	4.015	1.402
SPBC342.03	gas4	0.134	4.879	0.873	28.32	44.12	3.007	2.261
SPAC15A10.08	ain1	0.1085	4.882	0.965	31	49.16	3.679	1.685

SPAC27E2.03C	SPAC27E2.03c	0.004605	4.887	2.337	27.14	41.88	1.568	0.4381
SPCC16C4.13C	rpl1201	0.474	4.891	0.324	15.41	19.8	6.617	5.279
SPAPB8E5.05	mfm1	0.2589	4.894	0.587	30.31	47.84	6.28	1.837
SPBC887.17	SPBC887.17	0.2596	4.895	0.586	30.68	48.52	3.096	3.399
SPAC10F6.12C	mam4	0.03951	4.91	1.403	29.23	45.77	1.686	1.514
SPAC1834.03C	hhf1	0.002527	4.917	2.597	27.91	43.28	1.046	0.7571
SPBC14C8.17C	spt8	0.1536	4.92	0.814	22.36	32.82	4.232	1.996
SPCC550.12	arp6	0.2285	4.932	0.641	27.51	42.49	5.809	1.816
SPAC31G5.09C	spk1	0.2576	4.945	0.589	28.44	44.22	5.796	2.839
SPAC17A2.06C	vps8	0.1065	4.95	0.973	27.28	42.02	3.056	2.015
SPAC20G8.04C	SPAC20G8.04c	0.1438	4.963	0.842	30.32	47.73	4.471	1.668
SPAC15F9.02	seh1	0.3723	4.966	0.429	31.84	50.58	2.976	4.634
SPBC36B7.03	sec63	0.2756	4.973	0.560	34.11	54.84	5.041	3.151
SPCC794.11C	SPCC794.11c	0.6153	4.976	0.211	33	52.75	13.08	6.315
SPCC63.06	SPCC63.06	0.5664	4.978	0.247	20.45	29.12	10.38	6.074
SPBC776.11	rpl2801	0.4978	4.994	0.303	19.18	26.7	9.471	4.753
SPAC683.03	SPAC683.03	0.2341	4.995	0.631	35.16	56.77	3.93	3.078
SPCC736.09C	SPCC736.09c	0.09946	4.999	1.002	33.22	53.11	2.37	2.117
SPAC167.07C	SPAC167.07c	0.1198	5.001	0.922	18.35	25.12	2.384	2.304
SPCC622.17	apn1	0.03502	5.005	1.456	28.58	44.38	2.326	1.364
SPBC3B9.13C	rpp102	0.5699	5.006	0.244	18.73	25.83	9.339	6.646
SPAC4G8.10	gos1	0.3742	5.01	0.427	25.77	39.08	5.321	4.31
SPAC11E3.04C	ubc13	0.01559	5.017	1.807	38.26	62.56	1.417	1.186
SPCC4B3.05C	hem12	0.2309	5.017	0.637	28.82	44.79	5.175	2.568
SPBC4.02C	SPBC4.02c	0.4343	5.022	0.362	33.99	54.52	8.674	3.81
SPBP8B7.08C	SPBP8B7.08c	0.1379	5.032	0.860	27.79	42.83	2.743	2.438
SPAC22G7.02	kap111	0.0244	5.033	1.613	31.78	50.35	1.68	1.337
SPBP8B7.02	SPBP8B7.02	0.2338	5.034	0.631	33.85	54.23	3.999	3.089
SPAC23D3.03C	SPAC23D3.03c	0.2916	5.045	0.535	30.22	47.38	3.716	3.743
SPAPB17E12.14C	SPAPB17E12.14	0.06325	5.049	1.199	29.91	46.79	1.548	1.831
SPBC8E4.01C	SPBC8E4.01c	0.0446	5.051	1.351	26.74	40.83	2.696	1.388
SPAC1D4.05C	SPAC1D4.05c	0.2101	5.051	0.678	30.42	47.75	5.492	2.013
SPAC29A4.02C	SPAC29A4.02c	0.1649	5.06	0.783	28.87	44.82	3.057	2.652
SPAC3A11.09	sod22	0.112	5.062	0.951	28.33	43.8	2.852	2.189
SPBC4.06	SPBC4.06	0.3122	5.065	0.506	31.15	49.09	2.777	4.087
SPBC800.08	gcd10	0.2316	5.081	0.635	32.02	50.7	5.933	2.019
SPAC23C11.06C	SPAC23C11.06c	0.6123	5.082	0.213	25.98	39.33	13.48	6.244
SPAC1006.09	win1	0.4945	5.084	0.306	33.04	52.61	7.785	5.589
SPAC8C9.11	SPAC8C9.11	0.3055	5.085	0.515	32.04	50.74	5.725	3.365
SPAC11G7.03	idh1	0.1033	5.089	0.986	29.1	45.2	2.475	2.183
SPBC20F10.07	SPBC20F10.07	0.3229	5.092	0.491	24.43	36.39	4.403	3.994
SPAC513.05	ams1	0.2364	5.097	0.626	30.03	46.94	5.944	2.144
SPBC83.18C	fic1	0.2991	5.098	0.524	27.76	42.66	6.497	2.84
SPBC947.03C	naa38	0.01525	5.102	1.817	31.72	50.09	2.256	0.7701
SPAC26A3.06	SPAC26A3.06	0.1281	5.102	0.892	30.37	47.56	3.563	2.174
SPAC323.05C	SPAC323.05c	0.2113	5.103	0.675	17.09	22.57	4.674	2.668
SPCC13B11.02C	SPCC13B11.02c	0.1724	5.106	0.763	28.53	44.09	1.858	2.86
SPAC959.05C	SPAC959.05c	0.2969	5.137	0.527	28.87	44.68	6.49	2.866
SPAPB2B4.06	SPAPB2B4.06	0.1485	5.138	0.828	30.06	46.91	3.981	2.263
SPAC22F3.08C	rok1	0.374	5.14	0.427	19.12	26.3	6.331	4.141
SPBP22H7.05C	abo2	0.08119	5.142	1.090	29.56	45.97	1.234	2.039
SPAC14C4.11	SPAC14C4.11	0.2213	5.145	0.655	32.78	52.01	2.621	3.279
SPAC29A4.11	rga3	0.4111	5.147	0.386	32.38	51.26	6.471	4.658
SPBC887.11	pus2	0.3295	5.149	0.482	33.13	52.66	6.216	3.553
SPAC3H8.08C	SPAC3H8.08c	0.2365	5.15	0.626	31.59	49.76	2.989	3.387
SPCC364.03	rpl1702	0.07944	5.159	1.100	25.37	38.04	2.339	1.996
SPBC28F2.07	sfr1	0.1316	5.173	0.881	32.43	51.29	3.165	2.379
SPAC1142.01	SPAC1142.01	0.2811	5.179	0.551	35.5	57.06	4.534	3.577
SPBC30B4.04C	sol1	0.06375	5.182	1.196	28.62	44.12	1.515	1.884
SPAC328.02	SPAC328.02	0.2324	5.182	0.634	34.44	55.07	3.928	3.211
SPBC27B12.09C	SPBC27B12.09c	0.1765	5.197	0.753	32.95	52.24	4.077	2.593
SPAC1B3.17	clr2	0.09148	5.204	1.039	33.85	53.92	3.335	1.89
SPAC30C2.04	SPAC30C2.04	0.03428	5.207	1.465	23.13	33.73	2.059	1.503

SPAC1805.14	SPAC1805.14	0.2574	5.229	0.589	29.42	45.53	6.076	2.625
SPBC1539.07C	SPBC1539.07c	0.2869	5.23	0.542	28.98	44.7	5.085	3.532
SPAC27D7.06	SPAC27D7.06	0.1465	5.232	0.834	26.67	40.35	2.606	2.648
SPCC63.13	SPCC63.13	0.158	5.232	0.801	30.73	47.98	4.368	2.267
SPBC18H10.13	rps1402	0.117	5.246	0.932	28.45	43.67	3.968	1.937
SPAC664.15	caf4	0.08536	5.248	1.069	27.15	41.22	1.711	2.132
SPAC589.10C	SPAC589.10c	0.5779	5.257	0.238	22.88	33.16	11.62	6.46
SPBC4C3.08	mug136	0.05367	5.272	1.270	28.62	43.94	2.623	1.681
SPAC513.02	SPAC513.02	0.102	5.278	0.991	30.52	47.52	4.049	1.632
SPBC17D1.06	dbp3	0.1929	5.278	0.715	28.26	43.26	4.99	2.429
SPBC1E8.02	SPBC1E8.02	0.2487	5.281	0.604	32	50.29	5.79	2.753
SPAC1D4.13	byr1	0.3086	5.295	0.511	26.07	39.09	6.793	3.476
SPAPB8E5.10	SPAPB8E5.10	0.08988	5.332	1.046	29.58	45.64	3.586	1.825
SPBC19F8.03C	SPBC19F8.03c	0.07728	5.336	1.112	32.57	51.26	3.919	1.008
SPAC13C5.04	SPAC13C5.04	0.2099	5.338	0.678	26.36	39.57	2.99	3.264
SPAC1B3.15C	SPAC1B3.15c	0.2193	5.343	0.659	30.09	46.58	5.902	2.23
SPBC1685.11	rlp1	0.05932	5.347	1.227	32.33	50.79	3.511	1.184
SPAC16A10.02	sub1	0.1728	5.348	0.762	30.22	46.81	5.641	1.058
SPAPB2B4.04C	SPAPB2B4.04c	0.1061	5.354	0.974	30.46	47.26	2.991	2.258
SPAC6G9.12	cfr1	0.2666	5.371	0.574	31.01	48.26	4.463	3.607
SPBC1778.09	SPBC1778.09	0.1795	5.372	0.746	32.49	51.03	5.213	2.152
SPAC20H4.04	fml2	0.1907	5.372	0.720	33.74	53.4	4.821	2.59
SPAC664.13	SPAC664.13	0.04633	5.378	1.334	27	40.7	1.938	1.743
SPAPB1A10.13	SPAPB1A10.13	0.07361	5.406	1.133	27.65	41.86	1.082	2.051
SPBC216.03	SPBC216.03	0.6297	5.409	0.201	18.38	24.41	11.92	8.505
SPBC887.04C	lub1	0.2773	5.419	0.557	35.06	55.79	5.102	3.594
SPAC328.10C	rps502	0.2468	5.42	0.608	23.49	34	6.441	2.382
SPAC3G6.04	rnp24	0.2358	5.424	0.627	33.41	52.67	5.69	2.791
SPAC22A12.02C	mug103	0.2674	5.43	0.573	34.93	55.53	4.516	3.654
SPAC1782.09C	clp1	0.1448	5.438	0.839	31.92	49.84	3.44	2.613
SPAC26A3.09C	rga2	0.6507	5.444	0.187	25.71	38.14	13.35	8.922
SPBPB10D8.07C	SPBPB10D8.07c	0.1173	5.445	0.931	36.87	59.15	4.54	1.627
SPAC3A11.05C	kms1	0.1745	5.447	0.758	29.24	44.78	5.631	1.6
SPAC869.08	pcm2	0.1751	5.447	0.757	32.79	51.47	5.58	1.718
SPBC354.12	gpd3	0.6064	5.463	0.217	22.57	32.2	12.08	7.714
SPBC3F6.05	rga1	0.2432	5.468	0.614	35.07	55.72	4.504	3.417
SPAC31G5.17C	rps1001	0.04334	5.469	1.363	27.07	40.66	1.747	1.742
SPAC23G3.08C	ubp7	0.1266	5.469	0.898	28.94	44.17	1.426	2.631
SPBC12C2.12C	glo1	0.1974	5.473	0.705	29.88	45.94	2.997	3.232
SPAC1327.01C	SPAC1327.01c	0.1187	5.482	0.926	25.06	36.85	3.477	2.346
SPAC17A2.12	SPAC17A2.12	0.2757	5.496	0.560	31.85	49.6	6.577	2.945
SPBC839.04	rpl803	0.2261	5.497	0.646	29.75	45.65	4.337	3.295
SPBC1683.07	mal1	0.0403	5.504	1.395	36.31	57.98	2.979	1.382
SPAC22F8.07C	rtf1	0.02185	5.505	1.661	16.53	20.75	1.682	1.425
SPBC11B10.08	SPBC11B10.08	0.2569	5.509	0.590	33.82	53.29	5.447	3.3
SPBC215.02	bob1	0.07964	5.511	1.099	31.22	48.39	4.085	1.113
SPAC22G7.03	SPAC22G7.03	0.2199	5.511	0.658	31.96	49.78	6.334	2.01
SPCC757.05C	SPCC757.05c	0.1251	5.517	0.903	29.31	44.77	4.129	2.186
SPAC22G7.08	ppk8	0.3609	5.522	0.443	30.03	46.14	8.465	3.231
SPAC3H8.07C	pac10	0.07352	5.529	1.134	32.53	50.83	3.406	1.801
SPCP31B10.02	SPCP31B10.02	0.05675	5.533	1.246	32.42	50.6	3.378	1.489
SPAC27F1.05C	SPAC27F1.05c	0.007518	5.534	2.124	29.73	45.54	1.775	0.2515
SPAC11D3.16C	SPAC11D3.16c	0.04547	5.541	1.342	28.48	43.17	1.635	1.793
SPAC3C7.04	SPAC3C7.04	0.2623	5.546	0.581	22.47	31.86	2.094	3.997
SPBC14C8.09C	SPBC14C8.09c	0.2524	5.573	0.598	33.84	53.2	5.097	3.438
SPAC1071.05	SPAC1071.05	0.09164	5.587	1.038	23.55	33.81	3.961	1.806
SPAC8C9.05	SPAC8C9.05	0.1558	5.593	0.807	33.14	51.85	4.738	2.351
SPAP8A3.14C	SPAP8A3.14c	0.1614	5.612	0.792	27.8	41.76	4.844	2.389
SPBC1347.02	fkbp39	0.2935	5.641	0.532	26.32	38.91	6.882	3.253
SPAC6G9.01C	SPAC6G9.01c	0.06683	5.647	1.175	27.13	40.43	2.328	2.065
SPAC13C5.02	dre4	0.2599	5.652	0.585	11.6	11.2	4.328	3.801
SPAC227.13C	isu1	0.3116	5.655	0.506	30.89	47.5	4.919	4.287
SPAC1002.06C	bqt2	0.1716	5.656	0.765	32.69	50.88	5.522	2.065

SPBC19C7.02	ubr1	0.3654	5.656	0.437	24.94	36.3	7.636	4.114
SPBC685.06	rps001	0.4847	5.657	0.315	16.86	21.08	7.848	6.265
SPBC16E9.18	psd1	0.6286	5.662	0.202	26.24	38.74	12.52	8.846
SPBC725.09C	hob3	0.5716	5.678	0.243	19.09	25.25	9.654	7.881
SPAC1805.02C	SPAC1805.02c	0.09629	5.69	1.016	32.6	50.64	2.379	2.407
SPAC26F1.02	pnn1	0.2276	5.694	0.643	34.37	53.97	4.506	3.428
SPAC16E8.01	shd1	0.2868	5.695	0.542	21.93	30.56	6.109	3.626
SPBC359.03C	aat1	0.05612	5.698	1.251	29.08	44	1.787	1.981
SPCC74.03C	ssp2	0.0835	5.722	1.078	35.57	56.18	4.184	1.509
SPAC17G8.11C	imt3	0.0054	5.725	2.268	30.65	46.91	1.64	1.01
SPAC6C3.08	SPAC6C3.08	0.2215	5.725	0.655	31.49	48.49	4.467	3.393
SPAC1805.03C	trm13	0.06246	5.729	1.204	31.85	49.16	3.773	1.393
SPAC15E1.09	grx2	0.1466	5.731	0.834	26.04	38.23	4.428	2.505
SPCC11E10.04	ppr6	0.2933	5.731	0.533	29.94	45.56	3.438	4.377
SPAC2G11.07C	ptc3	0.05775	5.735	1.238	28.74	43.3	0.9097	1.965
SPAC23C4.02	crn1	0.3054	5.737	0.515	31.28	48.07	5.452	4.154
SPBP4G3.03	SPBP4G3.03	0.2166	5.748	0.664	33.79	52.78	5.027	3.176
SPAC24B11.13	hem3	0.1013	5.758	0.994	31.25	47.98	4.71	1.154
SPBC1711.12	SPBC1711.12	0.1663	5.767	0.779	30.45	46.45	3.158	3.087
SPBC28F2.11	SPBC28F2.11	0.1151	5.777	0.939	29.68	45	3.803	2.381
SPBC947.04	SPBC947.04	0.2076	5.777	0.683	34.26	53.62	4.313	3.315
SPAC2F7.09C	SPAC2F7.09c	0.2803	5.778	0.552	28.74	43.21	3.465	4.265
SPBC1105.11C	hht3	0.0706	5.781	1.151	27.94	41.71	2.945	2.068
SPAC19B12.11C	SPAC19B12.11c	0.07494	5.783	1.125	27.03	39.99	3.169	2.067
SPAC1F7.09C	SPAC1F7.09c	0.1943	5.792	0.712	34.19	53.45	6.133	2.11
SPAPB21F2.02	SPAPB21F2.02	0.2078	5.801	0.682	34.95	54.86	5.163	3.054
SPBC1711.05	SPBC1711.05	0.171	5.802	0.767	25.78	37.6	4.622	2.8
SPBC336.01	fbh1	0.5152	5.812	0.288	19.4	25.57	9.157	6.789
SPAC4H3.14C	SPAC4H3.14c	0.5912	5.818	0.228	27.55	40.9	12.66	7.729
SPBC1604.07	atp4	0.3391	5.82	0.470	31.42	48.19	5.758	4.61
SPBC16A3.18	cip1	0.1593	5.833	0.798	36.43	57.59	4.362	2.775
SPCC4G3.19	alp16	0.1179	5.843	0.928	30.64	46.67	4.448	2.155
SPAC29B12.06C	rcd1	0.228	5.849	0.642	23.9	33.97	5.022	3.408
SPCC338.05C	mms2	0.634	5.853	0.198	23.41	33.05	12.81	9.41
SPAC16C9.04C	mot2	0.4091	5.856	0.388	24.56	35.2	8.055	5.009
SPAPB24D3.03	SPAPB24D3.03	0.3596	5.865	0.444	34.7	54.27	4.011	5.25
SPBC14F5.09C	ade8	0.0276	5.871	1.559	27.21	40.17	1.742	1.628
SPBC146.10	mug57	0.05755	5.876	1.240	29.42	44.32	2.261	2.045
SPBC16H5.09C	omh2	0.07751	5.879	1.111	27.02	39.8	3.511	2.034
SPCC1840.10	lsm8	0.07736	5.896	1.111	31.42	48.03	2.991	2.201
SPAC5D6.13	SPAC5D6.13	0.0591	5.9	1.228	27.91	41.42	3.904	1.201
SPBC16G5.17	SPBC16G5.17	0.109	5.901	0.963	24.88	35.73	4.514	1.994
SPBC2D10.11C	nap2	0.09179	5.904	1.037	35.54	55.77	4.096	1.976
SPAC13G6.13	SPAC13G6.13	0.1722	5.913	0.764	29.27	43.96	6.1	1.661
SPCC126.04C	sgf73	0.1683	5.919	0.774	29.89	45.12	4.321	2.961
SPAPB24D3.01	SPAPB24D3.01	0.08269	5.921	1.083	28.38	42.28	4.426	1.318
SPCC1442.05C	SPCC1442.05c	0.1638	5.921	0.786	23.57	33.21	5.048	2.596
SPBC1778.02	rap1	0.07952	5.938	1.100	30.63	46.48	3.935	1.876
SPCC663.12	cid12	0.4047	5.941	0.393	18.74	24.09	7.519	5.248
SPBC839.05C	rps1701	0.5222	5.945	0.282	17.24	21.25	9.615	7.04
SPAC13G6.03	gpi7	0.2178	5.956	0.662	34.25	53.25	4.101	3.611
SPBC27.02C	ask1	0.05785	5.96	1.238	29	43.37	3.939	1.054
SPAC30C2.05	erv14	0.1811	5.971	0.742	31.8	48.6	6.146	2.035
SPCC1672.12C	get4	0.4389	5.973	0.358	17.85	22.34	10.68	4.389
SPBC31E1.02C	pmr1	0.1402	5.982	0.853	34.11	52.94	4.498	2.58
SPAC343.20	SPAC343.20	0.1021	5.983	0.991	33.3	51.41	4.174	2.17
SPBC17A3.09C	aim22	0.2726	5.986	0.564	21.02	28.3	4.762	4.142
SPAC1B1.02C	SPAC1B1.02c	0.1179	5.995	0.928	30.69	46.49	3.775	2.564
SPBC23E6.10C	SPBC23E6.10c	0.02215	5.998	1.655	29.59	44.41	0.8462	1.476
SPBC577.02	rpl3801	0.5384	5.998	0.269	21.42	29.04	10.03	7.427
SPAC17A5.07C	ulp2	0.1042	6.046	0.982	29.45	44.04	3.802	2.407
SPBC3H7.13	SPBC3H7.13	0.02344	6.067	1.630	29.34	43.81	1.791	1.604
SPAC13F5.05	SPAC13F5.05	0.3173	6.074	0.499	31.99	48.78	5.463	4.641

SPCC320.14	SPCC320.14	0.2284	6.085	0.641	31.12	47.12	6.191	3.123
SPCC306.04C	set1	0.1557	6.089	0.808	32.35	49.42	5.835	1.921
SPCC1906.02C	SPCC1906.02c	0.2449	6.109	0.611	32.85	50.33	5.846	3.568
SPBC12C2.09C	SPBC12C2.09c	0.108	6.128	0.967	30.97	46.76	5.108	1.434
SPBC1709.05	sks2	0.06565	6.13	1.183	27.81	40.81	2.313	2.243
SPAC5D6.07C	pxa1	0.1036	6.153	0.985	28.81	42.64	2.034	2.707
SPAC17C9.11C	SPAC17C9.11c	0.1312	6.175	0.882	35.52	55.24	5.1	2.239
SPCC417.11C	SPCC417.11c	0.3419	6.18	0.466	30.47	45.72	8.184	4.116
SPCC1183.04C	pet127	0.03727	6.199	1.429	25.08	35.53	3.366	1.443
SPAC22A12.04C	rps2201	0.2607	6.203	0.584	23.51	32.57	6.027	3.814
SPAC27E2.07	pvg2	0.01265	6.216	1.898	28.28	41.53	2.227	1.308
SPBC1703.03C	SPBC1703.03c	0.3205	6.222	0.494	24.42	34.26	7.344	4.214
SPBC11G11.03	mrt4	0.1823	6.237	0.739	31.56	47.67	5.651	2.842
SPAC11G7.02	pub1	0.01229	6.245	1.910	23.44	32.36	1.874	1.378
SPCC1281.03C	emc4	0.07251	6.248	1.140	32.67	49.72	3.738	2.073
SPBC651.06	mug166	0.5514	6.259	0.259	26.11	37.36	14.03	6.535
SPBC776.05	SPBC776.05	0.5535	6.261	0.257	23.58	32.6	11.14	7.974
SPBC3D6.02	but2	0.1072	6.262	0.970	34.48	53.11	4.64	2.193
SPAPB1A10.10C	ypt71	0.1733	6.262	0.761	31.53	47.55	4.239	3.285
SPCC4B3.08	lsg1	0.08406	6.271	1.075	32.91	50.14	4.525	1.756
SPAC25B8.15C	SPAC25B8.15c	0.06857	6.28	1.164	33.02	50.33	4.014	1.847
SPAC17A2.11	SPAC17A2.11	0.009449	6.281	2.025	31.16	46.82	2.003	1.264
SPBC16H5.07C	ppa2	0.01544	6.295	1.811	26.69	38.38	1.388	1.469
SPAC343.04C	SPAC343.04c	0.06547	6.317	1.184	28.87	42.45	1.724	2.315
SPAC3A12.13C	SPAC3A12.13c	0.04059	6.327	1.392	21.17	27.93	3.479	1.55
SPCC4B3.13	SPCC4B3.13	0.07577	6.348	1.121	29.13	42.88	2.038	2.462
SPAC19B12.04	rps3001	0.05503	6.369	1.259	30.24	44.93	3.91	1.638
SPBC3E7.08C	rad13	0.02077	6.378	1.683	29.84	44.17	1.672	1.628
SPBC16A3.17C	SPBC16A3.17c	0.09301	6.397	1.031	25.73	36.4	4.443	2.162
SPAC3H5.07	rpl702	0.07477	6.437	1.126	22.14	29.55	3.213	2.378
SPAC22H10.04	SPAC22H10.04	0.1595	6.445	0.797	32.88	49.76	3.153	3.416
SPBC18H10.05	SPBC18H10.05	0.3578	6.455	0.446	34.77	53.3	3.702	5.824
SPBC1778.03C	SPBC1778.03c	0.1901	6.475	0.721	29.77	43.85	6.87	2.201
SPAC3A11.13	SPAC3A11.13	0.05927	6.481	1.227	33.89	51.59	4.184	1.568
SPBC16C6.03C	SPBC16C6.03c	0.2393	6.487	0.621	25.93	36.6	6.527	3.554
SPCC550.15C	SPCC550.15c	0.1316	6.522	0.881	30.81	45.72	3.822	3.036
SPAPB1E7.05	gde1	0.01814	6.572	1.741	29.36	42.89	0.9365	1.526
SPCC4B3.15	mid1	0.2254	6.596	0.647	28.2	40.67	1.233	4.332
SPAC513.07	SPAC513.07	0.08887	6.616	1.051	30.78	45.48	1.877	2.73
SPAC1687.13C	csn5	0.02896	6.627	1.538	29.82	43.66	2.73	1.776
SPAC31G5.19	abo1	0.3844	6.668	0.415	24.55	33.66	9.566	4.967
SPBC31F10.12	SPBC31F10.12	0.03025	6.676	1.519	31.76	47.21	3.246	1.614
SPAC5D6.05	Sep-11	0.3237	6.687	0.490	23.39	31.43	7.13	5.297
SPBC28E12.06C	lvs1	0.05163	6.703	1.287	31.35	46.4	1.666	2.251
SPAC20H4.08	SPAC20H4.08	0.1436	6.704	0.843	32.69	48.9	6.066	2.213
SPBC31F10.13C	hip1	0.04104	6.721	1.387	33.47	50.34	3.275	1.913
SPCC1020.11C	SPCC1020.11c	0.393	6.721	0.406	23.17	30.95	9.225	5.409
SPCC24B10.16C	SPCC24B10.16c	0.5168	6.729	0.287	26.15	36.56	13.15	6.829
SPAC1B3.10C	SPAC1B3.10c	0.1127	6.748	0.948	34.56	52.35	2.932	3.049
SPACUNK4.12C	mug138	0.113	6.77	0.947	39.99	62.52	4.15	3.295
SPAC959.08	rpl2102	0.2645	6.799	0.578	23.17	30.81	8.813	2.636
SPBC146.12	coq6	0.1809	6.82	0.743	33.65	50.5	4.579	3.685
SPBC16C6.09	ogm4	0.1051	6.832	0.978	28.18	40.18	4.927	2.44
SPCC1223.11	ptc2	0.2326	6.84	0.633	26.57	37.13	4.349	4.398
SPAC31A2.11C	cuf1	0.1781	6.861	0.749	33.88	50.86	2.375	3.914
SPAC9G1.02	wis4	0.4082	6.874	0.389	40.02	62.38	12.38	3.776
SPAC20H4.11C	rho5	0.2443	6.877	0.612	35.3	53.5	4.579	4.541
SPBC31F10.03	SPBC31F10.03	0.03885	6.904	1.411	30.84	45.05	1.329	2.079
SPAC7D4.12C	SPAC7D4.12c	0.3658	6.939	0.437	33.78	50.51	4.952	6.286
SPBC19G7.04	SPBC19G7.04	0.05336	7.029	1.273	37.57	57.48	3.819	2.619
SPAC2G11.12	rqh1	0.03921	7.038	1.407	22.28	28.69	3.239	2.028
SPCC777.08C	bit61	0.02862	7.04	1.543	30.71	44.54	0.9868	1.876
SPAC13G7.03	upf3	0.04045	7.149	1.393	29.08	41.28	3.868	1.802

SPBC839.13C	rpl1601	0.129	7.169	0.889	25.35	34.21	5.122	3.023
SPAC25B8.18	SPAC25B8.18	0.1737	7.184	0.760	39.3	60.44	5.572	3.565
SPAC664.04C	rps1602	0.09973	7.249	1.001	29.15	41.21	5.407	2.329
SPBC725.03	SPBC725.03	0.5215	7.252	0.283	25.28	33.94	11.51	8.639
SPBC354.03	swd3	0.02351	7.287	1.629	33.61	49.54	3.624	1.026
SPACUNK4.15	SPACUNK4.15	0.08546	7.323	1.068	35.66	53.33	2.924	2.958
SPAC824.04	SPAC824.04	0.01832	7.336	1.737	34.05	50.28	3.24	0.5956
SPAC9.02C	SPAC9.02c	0.06763	7.383	1.170	18.43	20.78	5.163	1.289
SPAC4F10.19C	SPAC4F10.19c	0.01284	7.403	1.891	25.9	34.81	2.595	1.584
SPAC2F7.03C	pom1	0.01341	7.423	1.873	24.01	31.22	2.454	1.647
SPBC16E9.14C	zrg17	0.2364	7.468	0.626	33.31	48.65	4.159	4.929
SPBC30D10.18C	rpl102	0.09337	7.495	1.030	31.3	44.8	2.185	3.157
SPBC337.04	ppk27	0.003555	7.547	2.449	32.56	47.07	1.906	1.233
SPBC2A9.11C	SPBC2A9.11c	0.08697	7.589	1.061	31.76	45.5	2.589	3.105
SPCC31H12.05C	sds21	0.02053	7.604	1.688	36.29	53.99	3.623	0.9674
SPAC688.06C	slx4	0.001817	7.635	2.741	31.27	44.48	1.49	1.097
SPCC594.05C	spf1	0.04841	7.644	1.315	32.02	45.88	4.601	1.775
SPCC1682.14	rpl1902	0.1983	7.781	0.703	24.73	31.91	7.099	3.837
SPCC1223.13	cbf12	0.0227	7.809	1.644	31.89	45.32	2.388	2.044
SPBC16C6.05	SPBC16C6.05	0.0138	7.93	1.860	30.94	43.31	3.331	1.409
SPAC26F1.10C	pyp1	0.06731	8.004	1.172	36.01	52.71	3.726	2.877
SPAC25B8.03	psd2	0.07516	8.032	1.124	28.67	38.84	3.372	3.069
SPAC4F10.14C	btf3	0.009489	8.075	2.023	21.72	25.68	0.9225	1.525
SPCC1795.06	map2	0.3952	8.14	0.403	28.05	37.47	13.13	5.444
SPAC3G6.01	hrp3	0.09178	8.194	1.037	33.89	48.37	4.67	3.189
SPBC17A3.06	SPBC17A3.06	0.009776	8.277	2.010	23.23	28.14	2.633	1.685
SPAC13G6.02C	rps101	0.02324	8.325	1.634	28.14	37.3	2.51	2.194
SPCC1739.14	npp106	0.3558	8.397	0.449	21	23.73	7.989	7.065
SPBC29A10.05	exo1	0.0505	8.402	1.297	31.61	43.68	2.475	2.815
SPBP8B7.10C	SPBP8B7.10c	0.1094	8.49	0.961	32.52	45.22	4.051	3.751
SPCC320.03	SPCC320.03	0.002344	8.673	2.630	33.18	46.13	1.834	0.194
SPAC30.01C	sec72	0.1904	8.773	0.720	34.59	48.6	5.989	4.876
SPAC12G12.07C	SPAC12G12.07c	0.09505	8.805	1.022	31.43	42.59	6.125	3.025
SPBC16D10.07C	sir2	0.03852	8.874	1.414	38.33	55.44	4.527	3.026
SPAC694.05C	rps2502	0.01183	8.907	1.927	37.62	54.04	3.378	1.734
SPAC1B3.08	SPAC1B3.08	0.06597	8.926	1.181	33.75	46.73	5.099	2.907
SPBC17D11.01	nep1	0.2424	9.002	0.615	37.05	52.78	5.148	6.027
SPBC337.03	SPBC337.03	0.02823	9.057	1.549	32	43.19	1.597	2.448
SPAC23H3.05C	swd1	0.03599	9.182	1.444	33.44	45.66	5.185	1.367
SPCC162.11C	SPCC162.11c	0.02119	9.368	1.674	41.24	59.99	4.167	2.053
SPAC1F5.10	SPAC1F5.10	0.04717	10.4	1.326	14.37	7.478	3.53	3.401
SPAC23C11.04C	pnk1	0.03332	10.72	1.477	32.6	41.17	5.385	2.64
SPCC1223.05C	rpl3702	0.05319	11.18	1.274	24.36	24.82	5.01	3.684
SPBC19G7.03C	rps3002	0.01799	11.47	1.745	34.5	43.35	3.466	2.811
SPAC1B3.16C	vht1	0.003751	15.79	2.426	31.88	30.28	4.474	0.9368
SPAC27F1.08	pdt1	0.02827	23.03	1.549	32.4	17.64	5.731	6.394

Supplementary Table 4: Altered growth: Yes vs proline P=0.05

R package version: 0.0-10
Summary type: mean
Test type: t-test
Control medium: YE5S
Control libraries: PDLV4_384
Query medium: Proline
Query libraries: PDLV4_384

Sensitive to growth on Proline compared to YES
Relative resistent to growth on Proline compared to YES

##						Proline	YES		
ORF	Gene		P	Q	EGI	Fitness_Sum	Fitness_Sum	Proline_SE	YES_SE
SPBC30D10.16	pha2	phrenate dehydratase	0.009921	1	-28.74	8.528	61.22	5.233	5.982
SPBC2G5.06C	hmt2	sulfide-quinone oxidoreductase	0.003323	1	-19.5	19.46	64	4.869	1.366
SPAC1782.12C	SPAC1782.12c	DUF423 protein	0.01958	1	-19.29	22	67.83	7.167	0.81
SPAC343.16	lys2	homoaconitate hydratase Lys2	0.0212	1	-19.22	13.96	54.5	4.361	4.932
SPBC215.08C	arg4	arginine specific carbamoyl-phosphate synthase Arg4	0.04913	1	-18.93	9.083	46.01	10.32	3.308
SPBC27.08C	sua1	sulfate adenylyltransferase	0.0001228	0.203	-18.64	13.16	52.23	2.404	0.8829
SPAC56E4.03	SPAC56E4.03	aromatic aminotransferase (predicted)	0.02158	1	-17.96	12.27	49.67	6.007	4.445
SPAC343.15	tit1	tRNA isopentenyltransferase Tit1	0.03145	1	-16.71	13.79	50.1	3.014	4.729
SPBC25H2.08C	mrs2	magnesium ion transmembrane transporter Mrs2 (predicted)	0.04679	1	-15.96	30.84	76.87	8.255	1.163
SPBC428.05C	arg12	argininosuccinate synthase Arg12	0.03305	1	-15.63	13.22	47.39	5.236	4.482
SPBC15D4.09C	SPBC15D4.09c	cystathionine gamma-synthase Met3	0.03669	1	-14.9	19.06	55.79	6.983	0.8561
SPBC725.14	arg6	acetylglutamate synthase Arg6	0.02053	1	-14.12	17.83	52.48	5.701	1.303
SPCPB1C11.03	SPCPB1C11.03	cysteine transmembrane transporter (predicted)	4.17E-05	0.138	-13.59	24.53	62.62	1.552	0.8357
SPCC584.01C	SPCC584.01c	sulfite reductase NADPH flavoprotein subunit (predicted)	0.001121	1	-13.16	16.32	48.42	2.388	0.6009
SPAC4G9.09C	arg11	N-acetyl-gamma-glutamyl-phosphate reductase/acetylglutamate kinase	0.04802	1	-12.74	19	52.14	6.823	2.614
SPBC14C8.04	SPBC14C8.04	acetolactate synthase regulatory unit Ilv6 (predicted)	0.04959	1	-12.54	31.97	73.12	6.74	1.296
SPCC553.03	pex1	AAA family ATPase Pex1 (predicted)	0.008138	1	-12.49	29.86	69.57	3.885	2.164
SPAC31A2.09C	apm4	AP-2 adaptor complex mu subunit Apm4 (predicted)	0.004956	1	-12.26	18.55	50.61	3.403	1.923
SPCC188.13C	dcr1	dicer	0.009146	1	-12.23	16.97	47.96	3.696	2.319
SPAC683.02C	SPAC683.02c	zf-CCHC type zinc finger protein (predicted)	0.02356	1	-12.14	17.82	49.21	4.905	0.7231
SPBC21H7.07C	his5	imidazoleglycerol-phosphate dehydratase His5	0.008406	1	-12.09	18.04	49.5	3.883	1.111
SPAC23D3.04C	gpd2	glycerol-3-phosphate dehydrogenase Gpd2	0.02525	1	-11.12	28.66	65.35	4.694	2.377
SPBC725.11C	php2	CCAAT-binding factor complex subunit Php2	0.01193	1	-10.9	20.83	52.11	2.627	2.402
SPCC550.01C	SPCC550.01c	mitochondrial respiratory chain complex assembly protein (predicted)	0.03745	1	-10.04	29.65	65.19	4.861	0.8981
SPAC977.12	SPAC977.12	L-asparaginase (predicted)	0.02662	1	-9.896	16.92	44.05	4.352	1.976
SPBC23G7.08C	rga7	RhoGAP, GTPase activating protein Rga7	0.03094	1	-9.602	20.29	49.09	3.654	2.574
SPAC144.17C	SPAC144.17c	6-phosphofructo-2-kinase (predicted)	0.03227	1	-9.317	30.96	66.15	4.296	2.004
SPBC4F6.08C	mrpl39	mitochondrial ribosomal protein subunit L39 (predicted)	0.03472	1	-8.634	30.18	63.76	2.991	2.501
SPBC11B10.02C	his3	histidinol-phosphate aminotransferase imidazole acetol phosphate transami	0.03831	1	-8.357	19.46	45.69	4.19	1.163
SPAC23H4.10C	thi4	bifunctional thiamine-phosphate dipyrophosphorylase/hydroxyethylthiazole k	0.02012	1	-8.175	18.37	43.6	2.1	2.07
SPAC1952.05	gcn5	SAGA complex histone acetyltransferase catalytic subunit Gcn5	0.003954	1	-7.759	15.86	38.8	2.039	1.174

SPBC18H10.16	can1	arginine transmembrane transporter Can1	0.04356	1	-7.613	31.84	64.81	3.952	1.527
SPAC4D7.03	pop2	F-box/WD repeat protein Pop2	0.02734	1	-6.833	30.69	61.63	0.6974	1.77
SPBC30D10.13C	pdb1	pyruvate dehydrogenase e1 component beta subunit Pdb1	0.01323	1	-6.762	3.521	16.89	2.228	1.395
SPBC16A3.16	SPBC16A3.16	mitochondrial inner membrane protein involved in cytochrome c oxidase ass	0.005841	1	-6.684	31.85	63.29	1.888	1.113
SPBC2G2.06C	apl1	AP-2 adaptor complex beta subunit Apl1 (predicted)	0.04899	1	-6.307	33.07	64.69	1.776	2.092
SPBC19C7.01	mni1	exon-exon junction complex disassembly factor Mni1 (predicted)	0.0143	1	-6.275	28.11	56.47	1.772	1.427
SPAC11D3.14C	SPAC11D3.14c	5-oxoprolinase (ATP-hydrolizing) (predicted)	0.02885	1	-6.041	31.98	62.45	2.753	1.15
SPAC17G8.06C	SPAC17G8.06c	dihydroxy-acid dehydratase (predicted)	0.02307	1	-5.954	18.03	39.4	2.523	1.138
SPCC794.03	SPCC794.03	amino acid permease (predicted)	0.01407	1	-5.756	30.01	58.75	2.133	1.004
SPBC19C7.12C	omh1	alpha-1,2-mannosyltransferase Omh1	0.0448	1	-5.555	34.59	65.94	2.545	1.558
SPBC215.01	SPBC215.01	GTPase activating protein (predicted)	0.03432	1	-5.43	21.18	43.71	2.623	1.014
SPBP16F5.03C	tra1	SAGA complex phosphatidylinositol pseudokinase Tra1	0.02053	1	-5.409	29.4	57.18	1.425	1.376
SPAC14C4.16	dad3	DASH complex subunit Dad3	0.01209	1	-5.059	32.95	62.44	1.736	0.9469
SPBC19C2.06C	mug124	Schizosaccharomyces pombe specific protein	0.03413	1	-5.018	32.09	60.96	1.871	1.412
SPCC1393.02C	spt2	non-specific DNA binding protein Spt2 (predicted)	0.03098	1	-4.685	33.15	62.15	2.174	0.9217
SPAC1250.03	ubc14	ubiquitin conjugating enzyme E2 Ubc14 (predicted)	0.01993	1	-4.612	34.05	63.5	1.596	1.089
SPAC25B8.05	SPAC25B8.05	tRNA-pseudouridine synthase Deg1 (predicted)	0.02859	1	-4.599	20.14	40.64	2.026	0.373
SPBC19C7.05	SPBC19C7.05	vesicle-mediated transport protein (predicted)	0.04861	1	-4.462	34.09	63.33	2.404	0.9042
SPAC27D7.02C	SPAC27D7.02c	GRIP domain protein Grp1 (predicted)	0.04826	1	-4.102	35.49	65.03	1.583	1.306
SPAC18B11.09C	SPAC18B11.09c	serine O-acetyltransferase activity (predicted)	0.03967	1	-4.07	32.89	60.71	1.651	1.173
SPAC637.10C	rpn10	19S proteasome regulatory subunit Rpn10	0.02251	1	-4.066	32.11	59.43	1.654	0.8567
SPAC11D3.08C	SPAC11D3.08c	amino acid permease, unknown 1 (predicted)	0.03302	1	3.333	27.81	40.21	1.157	0.9478
SPAC4F10.14C	btf3	nascent polypeptide-associated complex beta subunit	0.01952	1	3.75	19.38	25.68	0.9225	0.9423
SPBC30B4.04C	sol1	SWI/SNF complex subunit Sol1	0.02255	1	3.919	30.78	44.12	1.515	0.895
SPAC1071.06	arp9	SWI/SNF and RSC complex subunit Arp9	0.04705	1	4.046	31.55	45.18	2.017	1.047
SPCC737.03C	ima1	inner nuclear membrane protein Ima1	0.0286	1	4.324	33.27	47.54	1.986	0.615
SPAC20G4.01	caf16	CCR4-Not complex subunit Caf16 (predicted)	0.03969	1	4.636	31.87	44.73	1.264	1.436
SPCC18B5.03	wee1	M phase inhibitor protein kinase Wee1	0.0407	1	4.715	23.58	30.99	2.409	0.8736
SPCC18.13	SPCC18.13	tRNA (guanine-N7-)-methyltransferase subunit Trm82 (predicted)	0.04381	1	4.777	27.85	37.9	2.078	1.382
SPAC27F1.05C	SPAC27F1.05c	aminotransferase class-III, unknown specificty	0.04904	1	4.923	32.65	45.54	1.775	1.602
SPBC16D10.02	trm11	tRNA (guanine-N2-)-methyltransferase catalytic subunit Trm11 (predicted)	0.04622	1	5.185	32.75	45.28	0.8096	1.648
SPBC1604.08C	imp1	importin alpha	0.01149	1	5.233	29.2	39.36	1.867	0.8143
SPAC23H4.16C	SPAC23H4.16c	Schizosaccharomyces specific protein	0.03603	1	5.427	30.71	41.53	2.456	1.345
SPAC27E2.07	pvg2	galactose residue biosynthesis protein Pvg2	0.0404	1	5.522	30.81	41.53	2.227	1.609
SPCC663.09C	SPCC663.09c	short chain dehydrogenase (predicted)	0.03275	1	5.611	32.4	44	1.721	1.625
SPAC5D6.09C	mug86	acetate transmembrane transporter (predicted)	0.02668	1	5.78	32.31	43.58	1.384	1.585
SPAC1805.02C	SPAC1805.02c	electron transfer flavoprotein beta subunit EtfB (predicted)	0.04757	1	5.799	36.63	50.64	2.379	1.798
SPAC688.06C	slx4	structure-specific endonuclease subunit Slx4	0.01815	1	5.827	32.91	44.48	1.49	1.434
SPBC1711.08	SPBC1711.08	chaperone activator Aha1	0.03626	1	5.874	34.61	47.2	2.668	1.453
SPAC926.05C	dph4	diphthamide biosynthesis protein Dph4 (predicted)	0.03113	1	6.072	36.81	50.49	2.725	1.357
SPBC29A3.05	vps71	Swr1 complex subunit Vps71	0.04064	1	6.077	33.17	44.5	2.868	1.539
SPCC320.03	SPCC320.03	transcription factor (predicted)	0.006592	1	6.137	34.22	46.13	1.834	1.002
SPBC16H5.07C	ppa2	serine/threonine protein phosphatase Ppa2	0.01006	1	6.142	29.51	38.38	1.388	1.298
SPBC19G7.10C	SPBC19G7.10c	topoisomerase II-associated deadenylation-dependent mRNA-decapping fa	0.01323	1	6.202	22.9	27.43	0.6833	1.299

SPBC1685.15C	klp6	kinesin-8 family plus-end microtubule motor Klp6	0.03085	1	6.337	31.99	42.13	1.628	1.816
SPAC1952.15C	rec24	meiotic recombination protein Rec24	0.01434	1	6.366	31.76	41.72	0.9525	1.417
SPAC23G3.08C	ubp7	ubiquitin C-terminal hydrolase Ubp7	0.02454	1	6.378	33.27	44.17	1.426	1.703
SPBC16C6.05	SPBC16C6.05	translation machinery associated protein ortholog Tma22 (predicted)	0.04135	1	6.464	32.83	43.31	3.331	0.9958
SPBC19G7.03C	rps3002	40S ribosomal protein S30 (predicted)	0.04992	1	6.466	32.86	43.35	3.466	1.446
SPBC4F6.06	kin1	microtubule affinity-regulating kinase Kin1	0.03629	1	6.467	17.41	17.97	1.834	1.945
SPAC186.05C	SPAC186.05c	human TMEM165 homolog, implicated in calcium transport	0.04432	1	6.535	33.42	44.16	3.237	1.622
SPCC1223.15C	spc19	DASH complex subunit Spc19	0.02835	1	6.673	33.56	44.16	3.028	1.259
SPAC27E2.03C	SPAC27E2.03c	Obg-Like ATPase (predicted)	0.001995	1	6.729	32.23	41.88	1.568	0.5451
SPAC22F3.04	mug62	AMP binding enzyme (predicted)	0.02311	1	6.907	33.45	43.59	2.515	1.681
SPCC364.04C	SPCC364.04c	CASP family protein involved in Golgi vesicle transport Coy1 (predicted)	0.04013	1	7.098	42.14	57.56	3.623	1.211
SPAC1B3.01C	SPAC1B3.01c	uracil phosphoribosyltransferase (predicted)	0.006346	1	7.144	32.78	42.1	2	1.245
SPAC20G8.02	SPAC20G8.02	mitochondrial DDHD family phospholipase (predicted)	0.04316	1	7.194	32.6	41.73	2.436	2.258
SPCC162.03	SPCC162.03	short chain dehydrogenase (predicted)	0.0235	1	7.222	35.26	46.06	3.069	1.4
SPAC22G7.02	kap111	karyopherin Kap111 (predicted)	0.01272	1	7.245	37.9	50.35	1.68	1.625
SPBC336.14C	ppk26	PAN complex protein phosphotransferase subunit Ppk26 (predicted)	0.02519	1	7.315	34.48	44.61	0.9714	1.885
SPAC3H1.12C	snt2	Lid2 complex PHD finger subunit Snt2	0.04351	1	7.321	30.11	37.44	3.285	2.059
SPAC6B12.14C	SPAC6B12.14c	conserved fungal protein	0.02509	1	7.375	36.36	47.61	2.415	1.936
SPAC1002.01	SPAC1002.01	conserved fungal protein	0.01988	1	7.469	35.66	46.3	2.787	1.659
SPBP23A10.14C	ell1	RNA polymerase II transcription elongation factor SpELL	0.002044	1	7.531	32.8	41.5	1.506	1.093
SPAC16E8.18	SPAC16E8.18	Schizosaccharomyces pombe specific protein	0.01749	1	7.568	34.34	43.97	2.914	1.465
SPBC1718.02	hop1	linear element associated protein Hop1	0.0118	1	7.659	36.7	47.71	2.117	1.653
SPBC2G2.01C	liz1	pantothenate transmembrane transporter Liz1	0.005725	1	7.777	29.61	35.86	1.458	1.432
SPAC3A12.13C	SPAC3A12.13c	translation initiation factor eIF3j (p35)	0.03427	1	7.798	24.8	27.93	3.479	1.906
SPBC1652.02	SPBC1652.02	APC amino acid transmembrane transporter (predicted)	0.03616	1	7.847	35.38	45.23	3.153	2.182
SPCC13B11.02C	SPCC13B11.02c	Schizosaccharomyces pombe specific protein	0.02992	1	7.866	34.71	44.09	1.858	2.23
SPAC1399.03	fur4	uracil permease	0.03606	1	8.226	30.65	36.83	2.051	2.471
SPCC132.01C	SPCC132.01c	microtubule regulator Mtr1	0.02185	1	8.472	33.64	41.34	2.662	2.147
SPAC16A10.01	SPAC16A10.01	ThrE amino acid transmembrane transporter family protein	0.02448	1	8.585	36.02	45.06	1.841	2.286
SPAC11G7.02	pub1	HECT-type ubiquitin-protein ligase E3 Pub1	0.01517	1	8.637	28.33	32.36	1.874	2.023
SPAC1F5.10	SPAC1F5.10	exon junction complex subunit, ATP-dependent RNA helicase Fal1	0.01607	1	9.195	13.75	7.478	3.53	0.9867
SPBC3B8.05	SPBC3B8.05	diphthamide biosynthesis protein Dph1 (predicted)	0.004165	1	9.303	38.37	47.75	2.2	1.553
SPAPB2B4.06	SPAPB2B4.06	acyl-coenzyme A thioesterase	0.04135	1	9.366	37.92	46.91	3.981	2.676
SPAC12B10.14C	ppk2	pseudokinase Tea5	0.04303	1	9.637	37.22	45.31	3.874	2.888
SPAC9.02C	SPAC9.02c	polyamine N-acetyltransferase (predicted)	0.01024	1	20.2	32.85	20.78	5.163	4.252
SPAC27F1.08	pdt1	Nramp family manganese ion transmembrane transporter	0.002218	1	23.58	34.32	17.64	5.731	2.178

Supplementary Table 5: Altered growth: Yes vs ammonium P=0.05

R package version: 0.0-10
Summary type: mean
Test type: t-test
Control medium: YE5S **Sensitive to growth on ammonium compared to YES**
Control libraries: PDLV4_384 **Relative resistent to growth on ammonium compared to YES**
Query medium: Ammonium_AC
Query libraries: PDLV4_384

##

ORF	Gene	description	P	EGI	Ammonium Fitness_Sum	YES Fitness_Sum	Ammonium_SE	YES_SE
SPBC30D10.16	pha2	phrenate dehydratase	0.002063	-22.77	9.76	61.22	5.233	0.9494
SPBC725.01	maa1	mitochondrial aspartate aminotransferase Maa1 (predicted)	0.0375	-21.8	8.496	57.02	11.97	1.657
SPAC17H9.08	SPAC17H9.08	mitochondrial coenzyme A transmembrane transporter (predicted)	0.02003	-21.4	4.804	49.31	10.13	2.743
SPAC1782.12C	SPAC1782.12c	DUF423 protein	0.01038	-20.45	15.59	67.83	7.167	0.9078
SPBC2G5.06C	hmt2	sulfide-quinone oxidoreductase	0.004161	-19.73	14.27	64	4.869	0.38
SPBC25H2.08C	mrs2	magnesium ion transmembrane transporter Mrs2 (predicted)	0.01852	-19.01	21.83	76.87	8.255	1.278
SPBC27.08C	sua1	sulfate adenylyltransferase	0.00458	-18.68	9.069	52.23	2.404	3.025
SPAC343.16	lys2	homoaconitate hydratase Lys2	0.004813	-17.43	11.53	54.5	4.361	3.079
SPAC23D3.04C	gpd2	glycerol-3-phosphate dehydrogenase Gpd2	0.002958	-16.88	17.83	65.35	4.694	1.155
SPCPB1C11.03	SPCPB1C11.03	cysteine transmembrane transporter (predicted)	2.56E-05	-16.79	16.48	62.62	1.552	1.067
SPAC31A2.09C	apm4	AP-2 adaptor complex mu subunit Apm4 (predicted)	0.01326	-16.25	10.64	50.61	3.403	3.63
SPAC3F10.09	SPAC3F10.09	1-(5-phosphoribosyl)-5-[(5-phosphoribosylamino) methylideneamino]imidazole-4-carboxamide isomerase	0.02098	-15.88	11.8	52.1	5.033	4.052
SPAC56E4.03	SPAC56E4.03	aromatic aminotransferase (predicted)	0.01587	-15.86	10.52	49.67	6.007	3.52
SPAC4G9.09C	arg11	N-acetyl-gamma-glutamyl-phosphate reductase/acetylglutamate kinase	0.02229	-15.21	12.49	52.14	6.823	0.7415
SPBC21H7.07C	his5	imidazoleglycerol-phosphate dehydratase His5	0.00723	-15.07	11.23	49.5	3.883	2.937
SPBC428.05C	arg12	argininosuccinate synthase Arg12	0.02867	-13.93	11.25	47.39	5.236	3.824
SPBC17D1.05	SPBC17D1.05	Schizosaccharomyces specific protein	0.04821	-13.74	24.48	71.94	8.501	1.885
SPAC637.10C	rpn10	19S proteasome regulatory subunit Rpn10	2.68E-05	-13.64	17.93	59.43	1.654	0.7646
SPBC1105.02C	lys4	homocitrate synthase	0.02845	-13.48	14.29	52.27	6.526	0.5712
SPAC343.15	tit1	tRNA isopentenyltransferase Tit1	0.03902	-13.43	13.19	50.1	3.014	4.089
SPCC1235.04C	nad1	FAD synthetase Nad1 (predicted)	0.03493	-13.4	25.64	73.5	7.025	0.6572
SPBC725.14	arg6	acetylglutamate synthase Arg6	0.01851	-13.28	14.6	52.48	5.701	0.7929
SPBC23G7.08C	rga7	RhoGAP, GTPase activating protein Rga7	0.002122	-13.09	12.99	49.09	3.654	1.448
SPAC25G10.05C	his1	ATP phosphoribosyltransferase	0.0249	-13.05	15.32	53.4	6.397	1.229
SPAC17H9.13C	pro2	glutamate 5-kinase Pro2	0.02874	-12.96	11.59	46.2	3.386	3.625
SPBC15D4.09C	met3	cystathionine gamma-synthase Met3	0.03969	-12.81	16.82	55.79	6.983	0.4666
SPBC11B10.02C	his3	histidinol-phosphate aminotransferase imidazole acetol phosphate transaminase His3	0.02431	-12.06	12.21	45.69	4.19	3.185
SPCPB16A4.03C	ade10	bifunctional IMP cyclohydrolase/phosphoribosylaminoimidazolecarboxamideformyltransferase	0.0001575	-12.05	11.65	44.61	1.569	1.072
SPBC14F5.13C	pho8	vacuolar membrane alkaline phosphatase (predicted)	0.03617	-12	23.11	66.08	6.51	0.9123
SPBC21C3.03	SPBC21C3.03	ABC1 kinase family protein	0.04268	-11.82	25.17	69.6	6.75	0.7971
SPAC3C7.01C	sac12	inositol polyphosphate phosphatase (predicted)	0.04801	-11.75	27.92	74.67	6.943	0.598

SPCC188.13C	dcr1	dicer	0.00472	-11.65	13.84	47.96	3.696	0.9515
SPBC28F2.08C	hrd3	Hrd1 ubiquitin ligase complex subunit (predicted)	0.03557	-11.17	23.82	65.86	6.261	1.511
SPCC16A11.08	atg20	autophagy associated sorting nexin Atg20 (predicted)	0.04291	-11.1	25.74	69.33	6.583	1.407
SPBC359.06	mug14	adducin	0.02302	-11.01	24.91	67.61	5.382	1.292
SPAC22F3.13	tsc1	hamartin	0.01622	-10.84	18.27	54.78	4.793	1.914
SPBC1539.03C	arg41	argininosuccinate lyase (predicted)	0.0157	-10.4	14.27	46.44	4.204	0.5572
SPBC28F2.10C	ngg1	SAGA complex subunit Ngg1/Ada3	0.01337	-10.35	16.47	50.47	4.385	1.69
SPBPB2B2.02	mug180	esterase/lipase (predicted)	0.03093	-10.25	28	71.98	5.427	1.116
SPBC1685.13	fhn1	plasma membrane organization protein Fhn1	0.02898	-10.01	21.05	58.46	5.271	1.801
SPAC17A5.01	pex6	peroxin-6 (predicted)	0.02496	-9.801	23.82	63.29	4.962	1.421
SPAC890.05	SPAC890.05	ribosome biogenesis protein, G-patch domain, PINX1 family (predicted)	0.04863	-9.765	15.41	47.39	6.073	1.772
SPAC144.17C	SPAC144.17c	6-phosphofructo-2-kinase (predicted)	0.01565	-9.646	25.5	66.15	4.296	1.45
SPAC683.02C	SPAC683.02c	zf-CCHC type zinc finger protein (predicted)	0.02761	-9.518	16.63	49.21	4.905	1.8
SPCC777.10C	ubc12	NEDD8-conjugating enzyme Ubc12	0.01568	-9.492	18.28	52.27	3.696	2.056
SPCC584.01C	met10	sulfite reductase NADPH flavoprotein subunit (predicted)	0.004758	-9.216	16.51	48.42	2.388	0.1962
SPBC365.16	SPBC365.16	conserved protein	0.03097	-9.058	14.38	44.11	4.882	1.552
SPCC70.06	sac32	nuclear export factor Sac32 (predicted)	0.02252	-9.056	24.75	63.63	4.431	1.171
SPBC1289.06C	ppr8	mitochondrial PPR repeat protein Ppr8	0.03017	-8.988	19.4	53.44	4.57	0.6405
SPBC25B2.04C	mtg1	mitochondrial GTPase involved in translation Mtg1 (predicted)	0.009943	-8.914	15.56	46.07	3.011	0.3063
SPAC1834.04	hht1	histone H3 h3.1	0.04905	-8.913	29.29	71.89	5.521	1.109
SPAC664.03	paf1	RNA polymerase II associated Paf1 complex (predicted)	0.02072	-8.816	10.93	37.17	2.269	2.247
SPAC323.01C	pos5	mitochondrial NADH kinase Pos5 (predicted)	0.0008314	-8.796	2.958	22.12	2.021	0.7644
SPBP35G2.07	ilv1	acetolactate synthase catalytic subunit	0.04837	-8.648	8.953	33.13	3.065	2.848
SPAC1250.03	ubc14	ubiquitin conjugating enzyme E2 Ubc14 (predicted)	0.000975	-8.528	25.21	63.5	1.596	1.077
SPCC1393.02C	spt2	non-specific DNA binding protein Spt2 (predicted)	0.00392	-8.298	24.72	62.15	2.174	1.377
SPAC2G11.03C	vps45	vacuolar sorting protein Vps45	0.001809	-8.158	19.18	51.45	2.05	0.5437
SPAC1B3.01C	SPAC1B3.01c	uracil phosphoribosyltransferase (predicted)	0.001926	-7.883	14.49	42.1	2	0.5211
SPBC19C7.01	mni1	exon-exon junction complex disassembly factor Mni1 (predicted)	0.0008171	-7.882	22.12	56.47	1.772	0.839
SPBC23G7.06C	SPBC23G7.06c	ERMES complex complex subunit Mmm1-related protein	0.01835	-7.859	27.22	66.03	3.624	0.9621
SPBC18H10.16	can1	arginine transmembrane transporter Can1	0.02538	-7.762	26.67	64.81	3.952	1.22
SPBC13E7.07	SPBC13E7.07	Schizosaccharomyces specific protein	0.03137	-7.639	27.8	66.69	4.101	0.9437
SPBC19C7.12C	omh1	alpha-1,2-mannosyltransferase Omh1	0.006078	-7.634	27.4	65.94	2.545	1.245
SPBC13E7.11	rbd1	mitochondrial rhomboid protease (predicted)	0.03597	-7.512	26.53	64.07	4.223	1
SPCC24B10.02C	SPCC24B10.02c	NAD/NADH kinase (predicted)	0.05016	-7.43	29.02	68.6	4.662	1.045
SPAC343.18	rfp2	SUMO-targeted ubiquitin-protein ligase subunit Rfp2	0.04944	-7.071	27.48	65.03	4.387	0.8586
SPBP16F5.03C	tra1	SAGA complex phosphatidylinositol pseudokinase Tra1	0.0004901	-6.948	23.43	57.18	1.425	0.6607
SPBC30D10.13C	pdb1	pyruvate dehydrogenase e1 component beta subunit Pdb1	0.004911	-6.839	2.136	16.89	2.228	1.016
SPBC1703.13C	SPBC1703.13c	mitochondrial inorganic phosphate transmembrane transporter (predicted)	0.01592	-6.381	28.44	65.54	2.844	1.025
SPAC23C4.12	hhp2	serine/threonine protein kinase Hhp2	0.03986	-6.27	23.25	55.55	3.66	1.031
SPBC337.13C	gtr1	Gtr1/RagA G protein Gtr1 (predicted)	0.02774	-6.194	17.86	45.28	1.852	1.719
SPBC25H2.05	egd2	nascent polypeptide-associated complex alpha subunit Egd2	0.01298	-6.158	26.91	62.24	2.572	0.6758
SPAC1952.05	gcn5	SAGA complex histone acetyltransferase catalytic subunit Gcn5	0.03487	-6.041	14.58	38.8	2.039	1.791
SPAC458.04C	dli1	meiotic dynein intermediate light chain Dli1/Dil1	0.04963	-6.041	30.35	68.49	3.784	0.903
SPAC20H4.03C	tfs1	transcription elongation factor TFIIS	0.01247	-5.902	24.95	58.07	1.947	1.281

SPBC4F6.08C	mrpl39	mitochondrial ribosomal protein subunit L39 (predicted)	0.02802	-5.76	28.11	63.76	2.991	0.7127
SPAC1610.02C	mrpl1	mitochondrial ribosomal protein subunit L1 (predicted)	0.02335	-5.756	15.95	40.86	2.822	1.067
SPAPJ760.02C	abp1	cofilin/tropomyosin family, drebrin ortholog Abp1	0.004458	-5.707	24.57	56.99	1.871	0.724
SPAC22H10.09	SPAC22H10.09	Schizosaccharomyces specific protein	0.01346	-5.633	15.45	39.67	2.379	0.9482
SPCC794.03	SPCC794.03	amino acid permease (predicted)	0.01365	-5.484	25.73	58.75	2.133	1.115
SPCC1620.02	wtf23	wtf element Wtf23	0.02202	-5.483	29	64.91	2.623	1.046
SPCC4B3.06C	SPCC4B3.06c	NADPH-dependent FMN reductase (predicted)	0.02859	-5.467	28.72	64.35	2.882	0.8846
SPCC4B3.11C	fra3	mitochondrial transcriptional repressor protein BolA domain (predicted)	0.005956	-5.459	28.67	64.24	1.91	0.6331
SPAC630.10	bmt2	rRNA (adenine) methyltransferase activity Bmt2 (predicted)	0.01468	-5.426	10.87	30.67	2.22	0.3895
SPBC839.07	ibp1	Cdc25 family phosphatase Ibp1	0.0008999	-5.193	25.59	57.93	1.194	0.5569
SPBC19C7.05	SPBC19C7.05	vesicle-mediated transport protein (predicted)	0.01971	-5.085	28.56	63.33	2.404	0.8341
SPBC2G2.07C	mug178	mitochondrial ribosomal protein subunit L51-b (predicted)	0.02013	-5.03	14.47	36.69	2.398	0.7835
SPAC12G12.12	gms2	UDP-galactose transmembrane transporter Gms2 (predicted)	0.04572	-4.991	24.57	55.63	3.043	0.7551
SPAC32A11.03C	phx1	stationary phase-specific homeobox transcription factor Phx1	0.02264	-4.817	26.51	58.96	2.082	1.13
SPAC17D4.01	pex7	peroxin-7 (predicted)	0.01204	-4.71	28.77	63.01	1.491	1.023
SPBC19C2.06C	mug124	Schizosaccharomyces pombe specific protein	0.01694	-4.555	27.83	60.96	1.871	0.9725
SPAC27E2.02	yih1	IMPACT homolog, cytoplasmic translational regulator Yih1 (predicted)	0.001284	-4.544	12.53	32.13	0.8556	0.6075
SPCC1442.07C	wss2	ubiquitin/metalloprotease fusion protein Udp7	0.02089	-4.38	25.7	56.61	1.65	1.078
SPAC186.07C	SPAC186.07c	hydroxyacid dehydrogenase (predicted)	0.01416	-4.347	31.02	66.57	1.83	0.7873
SPBC16A3.16	coa5	mitochondrial inner membrane protein involved in cytochrome c oxidase assembly Coa5 (predicted)	0.0215	-4.32	29.31	63.29	1.888	0.9758
SPAC5H10.01	SPAC5H10.01	DUF1445 family mitochondrial protein (predicted)	0.007884	-4.114	26.85	58.28	1.317	0.7783
SPBC25B2.02C	mam1	M-factor transmembrane transporter Mam1	0.01613	-3.924	29.18	62.31	1.501	0.871
SPBC1685.05	htr11	serine protease (predicted)	0.01515	-3.882	27.94	59.89	1.457	0.8504
SPBC336.10C	tif512	translation elongation factor eIF5A (predicted)	0.02562	-3.73	28.8	61.23	1.531	0.9553
SPAC5H10.11	gmh1	alpha-1,2-galactosyltransferase Gmh1 (predicted)	0.02152	-3.665	28.83	61.16	1.652	0.7952
SPBPB7E8.02	SPBPB7E8.02	PSP1 family protein	0.02947	-3.5	25.19	53.99	1.655	0.8614
SPAC23H4.12	alp13	MRG family Clr6 histone deacetylase complex subunit Alp13	0.01581	-3.334	30.15	63.03	1.231	0.7494
SPAPJ691.03	mic10	MICOS complex subunit Mic10 (predicted)	0.01626	-3.094	30.41	63.07	1.05	0.7242
SPAC6F12.04	tvp15	COPI-coated vesicle associated protein (predicted)	0.00814	3.383	26.03	42.63	1.021	0.6628
SPCC576.12C	mhf2	kinetochore protein, CENP-X ortholog, FANCM-MHF complex subunit Mhf2	0.03944	3.681	25.61	41.28	1.629	1.082
SPCC962.05	ast1	asteroid homolog, XP-G family protein	0.003368	3.732	26.77	43.37	1.034	0.575
SPAC4D7.01C	sec71	Sec7 domain protein, ARF GEF (predicted)	0.02902	3.933	34.98	58.43	1.771	1.005
SPBC3B8.05	SPBC3B8.05	diphthamide biosynthesis protein Dph1 (predicted)	0.03741	4.059	29.43	47.75	2.2	0.9525
SPAC23A1.11	rpl1602	60S ribosomal protein L13/L16 (predicted)	0.02599	4.091	27.67	44.38	1.605	1.073
SPBPB2B2.10C	gal7	galactose-1-phosphate uridylyltransferase Gal7	0.03685	4.25	24.42	37.97	2.364	0.8988
SPAC3A12.17C	cys12	cysteine synthase-like protein Cys12	0.002054	4.286	28.26	45.11	1.086	0.2679
SPBC1685.15C	klp6	kinesin-8 family plus-end microtubule motor Klp6	0.01083	4.301	26.69	42.13	1.628	0.8009
SPBC887.02	SPBC887.02	ClC chloride channel (predicted)	0.03834	4.399	26.83	42.22	1.908	1.288
SPBC31F10.07	lsb5	cortical component Lsb5 (predicted)	0.04789	4.443	27.12	42.68	2.742	0.8478
SPCC24B10.21	tpi1	triosephosphate isomerase	0.03085	4.499	28.7	45.55	2.18	1.106
SPAC23A1.17	bbc1	WIP family cytoskeletal protein Bbc1 (predicted)	0.03023	4.575	27.8	43.72	2.112	1.168
SPAC4F10.20	grx1	glutaredoxin Grx1	0.03356	4.732	28.48	44.71	0.4368	1.299
SPAC6B12.14C	SPAC6B12.14c	conserved fungal protein	0.03355	4.77	30.07	47.61	2.415	1.166
SPAC27E2.03C	SPAC27E2.03c	Obg-Like ATPase (predicted)	0.004605	4.887	27.14	41.88	1.568	0.4381

SPAC10F6.12C	mam4	protein-S isoprenylcysteine O-methyltransferase Mam4	0.03951	4.91	29.23	45.77	1.686	1.514
SPAC1834.03C	hhf1	histone H4 h4.1	0.002527	4.917	27.91	43.28	1.046	0.7571
SPCC622.17	apn1	AP endonuclease, minor transcript isoform Apn1	0.03502	5.005	28.58	44.38	2.326	1.364
SPAC11E3.04C	ubc13	ubiquitin conjugating enzyme E2 Ubc13	0.01559	5.017	38.26	62.56	1.417	1.186
SPAC22G7.02	kap111	karyopherin Kap111 (predicted)	0.0244	5.033	31.78	50.35	1.68	1.337
SPBC8E4.01C	pho84	inorganic phosphate transmembrane transporter (predicted)	0.0446	5.051	26.74	40.83	2.696	1.388
SPBC947.03C	naa38	NatC N-acetyltransferase non-catalytic Sm-like domain subunit Naa38 (predicted)	0.01525	5.102	31.72	50.09	2.256	0.7701
SPAC30C2.04	asc1	cofactor for cytoplasmic methionyl-and glutamyl-tRNA synthetases Asc1 (predicted)	0.03428	5.207	23.13	33.73	2.059	1.503
SPAC664.13	SPAC664.13	Schizosaccharomyces pombe specific protein	0.04633	5.378	27	40.7	1.938	1.743
SPAC31G5.17C	rps1001	40S ribosomal protein S10 (predicted)	0.04334	5.469	27.07	40.66	1.747	1.742
SPBC1683.07	mal1	maltase alpha-glucosidase Mal1	0.0403	5.504	36.31	57.98	2.979	1.382
SPAC22F8.07C	rtf1	replication termination factor Rtf1	0.02185	5.505	16.53	20.75	1.682	1.425
SPAC27F1.05C	SPAC27F1.05c	aminotransferase class-III, unknown specificity	0.007518	5.534	29.73	45.54	1.775	0.2515
SPAC11D3.16C	SPAC11D3.16c	Schizosaccharomyces specific protein	0.04547	5.541	28.48	43.17	1.635	1.793
SPAC17G8.11C	imt3	mannosyltransferase Imt3	0.0054	5.725	30.65	46.91	1.64	1.01
SPBC14F5.09C	ade8	adenylosuccinate lyase Ade8	0.0276	5.871	27.21	40.17	1.742	1.628
SPBC23E6.10C	mri1	methylthioribose-1-phosphate isomerase Mri1 (predicted)	0.02215	5.998	29.59	44.41	0.8462	1.476
SPBC3H7.13	far10	SIP/FAR complex FHA domain subunit Far10/Csc1	0.02344	6.067	29.34	43.81	1.791	1.604
SPCC1183.04C	pet127	mitochondrial RNA metabolism pathway protein Pet127	0.03727	6.199	25.08	35.53	3.366	1.443
SPAC27E2.07	pvg2	galactose residue biosynthesis protein Pvg2	0.01265	6.216	28.28	41.53	2.227	1.308
SPAC11G7.02	pub1	HECT-type ubiquitin-protein ligase E3 Pub1	0.01229	6.245	23.44	32.36	1.874	1.378
SPAC17A2.11	SPAC17A2.11	Schizosaccharomyces pombe specific protein	0.009449	6.281	31.16	46.82	2.003	1.264
SPBC16H5.07C	ppa2	serine/threonine protein phosphatase Ppa2	0.01544	6.295	26.69	38.38	1.388	1.469
SPAC3A12.13C	hcr1	translation initiation factor eIF3j (p35)	0.04059	6.327	21.17	27.93	3.479	1.55
SPBC3E7.08C	rad13	DNA repair nuclease Rad13	0.02077	6.378	29.84	44.17	1.672	1.628
SPAPB1E7.05	gde1	glycerophosphoryl diester phosphodiesterase Gde1 (predicted)	0.01814	6.572	29.36	42.89	0.9365	1.526
SPAC1687.13C	csn5	COP9/signalosome complex protease subunit Csn5	0.02896	6.627	29.82	43.66	2.73	1.776
SPBC31F10.12	tma20	RNA-binding protein Tma20 (predicted)	0.03025	6.676	31.76	47.21	3.246	1.614
SPBC31F10.13C	hip1	hira protein, histone chaperone Hip1	0.04104	6.721	33.47	50.34	3.275	1.913
SPBC31F10.03	ggg1	glutathione-specific gamma-glutamylcyclotransferase Ggg1 (predicted)	0.03885	6.904	30.84	45.05	1.329	2.079
SPAC2G11.12	rqh1	RecQ type DNA helicase Rqh1	0.03921	7.038	22.28	28.69	3.239	2.028
SPCC777.08C	bit61	TORC2 subunit Bit61	0.02862	7.04	30.71	44.54	0.9868	1.876
SPAC13G7.03	upf3	up-frameshift suppressor 3 family protein (predicted)	0.04045	7.149	29.08	41.28	3.868	1.802
SPBC354.03	swd3	WD repeat protein Swd3	0.02351	7.287	33.61	49.54	3.624	1.026
SPAC824.04	swd22	mRNA cleavage and polyadenylation specificity factor complex subunit, WD repeat protein Swd22	0.01832	7.336	34.05	50.28	3.24	0.5956
SPAC4F10.19C	hit1	zf-HIT family C/D snoRNP assembly protein Hit1 (predicted)	0.01284	7.403	25.9	34.81	2.595	1.584
SPAC2F7.03C	pom1	DYRK family protein kinase Pom1	0.01341	7.423	24.01	31.22	2.454	1.647
SPBC337.04	ppk27	serine/threonine protein kinase Ppk27 (predicted)	0.003555	7.547	32.56	47.07	1.906	1.233
SPCC31H12.05C	sds21	serine/threonine protein phosphatase PP1 subfamily, Sds21	0.02053	7.604	36.29	53.99	3.623	0.9674
SPAC688.06C	slx4	structure-specific endonuclease subunit Slx4	0.001817	7.635	31.27	44.48	1.49	1.097
SPCC594.05C	spf1	Set1C PHD Finger protein Spf1	0.04841	7.644	32.02	45.88	4.601	1.775
SPCC1223.13	cbf12	CBF1/Su(H)/LAG-1 family transcription factor Cbf12	0.0227	7.809	31.89	45.32	2.388	2.044
SPBC16C6.05	tma22	translation machinery associated protein ortholog Tma22 (predicted)	0.0138	7.93	30.94	43.31	3.331	1.409
SPAC4F10.14C	btf3	nascent polypeptide-associated complex beta subunit	0.009489	8.075	21.72	25.68	0.9225	1.525

SPBC17A3.06	SPBC17A3.06	phosphoprotein phosphatase (predicted)	0.009776	8.277	23.23	28.14	2.633	1.685
SPAC13G6.02C	rps101	40S ribosomal protein S3a	0.02324	8.325	28.14	37.3	2.51	2.194
SPCC320.03	SPCC320.03	transcription factor (predicted)	0.002344	8.673	33.18	46.13	1.834	0.194
SPBC16D10.07C	sir2	Sirtuin family histone deacetylase Sir2	0.03852	8.874	38.33	55.44	4.527	3.026
SPAC694.05C	rps2502	40S ribosomal protein S25 (predicted)	0.01183	8.907	37.62	54.04	3.378	1.734
SPBC337.03	rhn1	RNA polymerase II transcription termination factor homolog	0.02823	9.057	32	43.19	1.597	2.448
SPAC23H3.05C	swd1	Set1C complex subunit Swd1	0.03599	9.182	33.44	45.66	5.185	1.367
SPCC162.11C	urk1	uridine kinase/uracil phosphoribosyltransferase (predicted)	0.02119	9.368	41.24	59.99	4.167	2.053
SPAC1F5.10	fal1	exon junction complex subunit, ATP-dependent RNA helicase Fal1	0.04717	10.4	14.37	7.478	3.53	3.401
SPAC23C11.04C	pnk1	DNA kinase/phosphatase Pnk1	0.03332	10.72	32.6	41.17	5.385	2.64
SPBC19G7.03C	rps3002	40S ribosomal protein S30 (predicted)	0.01799	11.47	34.5	43.35	3.466	2.811
SPAC1B3.16C	vht1	vitamin H transmembrane transporter Vht1	0.003751	15.79	31.88	30.28	4.474	0.9368
SPAC27F1.08	pdt1	Nramp family manganese ion transmembrane transporter	0.02827	23.03	32.4	17.64	5.731	6.394

Supplementary Table 6: Altered growth: Yes vs Glutamate P=0.05

R package version: 0.0-10
Summary type: mean
Test type: t-test
Control medium: YE5S
Control libraries: PDLV4_384
Query medium: Glutamate
Query libraries: PDLV4_384

Sensitive to growth on glutamate compared to YES
Relative resistent to growth on glutamate compared to YES

##

ORF	Gene	description	P	EGI	Glutamate Fitness_Sum	YES Fitness_Sum	Glutamate_SE	YES_SE
SPBC30D10.16	pha2	phrenate dehydratase	0.001392	-36.17	7.46	61.22	5.233	1.057
SPAC1782.12C	SPAC1782.12c	DUF423 protein	0.01939	-21.39	26.95	67.83	7.167	1.754
SPBC2G5.06C	hmt2	sulfide-quinone oxidoreductase	0.005318	-21.12	24.49	64	4.869	1.311
SPBC27.08C	sua1	sulfate adenylyltransferase	0.0006528	-20.42	16.81	52.23	2.404	0.5107
SPAC343.15	tit1	tRNA isopentenyltransferase Tit1	0.0239	-20.18	15.53	50.1	3.014	5.252
SPAC56E4.03	SPAC56E4.03	aromatic aminotransferase (predicted)	0.01415	-19.89	15.5	49.67	6.007	3.913
SPBC21H7.07C	his5	imidazoleglycerol-phosphate dehydratase His5	0.01419	-19.86	15.42	49.5	3.883	4.582
SPBC428.05C	arg12	argininosuccinate synthase Arg12	0.0275	-18.95	14.82	47.39	5.236	5.146
SPAC23D3.04C	gpd2	glycerol-3-phosphate dehydrogenase Gpd2	0.006565	-17.61	28.96	65.35	4.694	2.028
SPCPB1C11.03	SPCPB1C11.03	cysteine transmembrane transporter (predicted)	4.01E-05	-17.41	27.22	62.62	1.552	1.188
SPAC25G10.05C	his1	ATP phosphoribosyltransferase	0.0238	-17.3	20.75	53.4	6.397	2.168
SPBC725.14	arg6	acetylglutamate synthase Arg6	0.02366	-16.5	20.9	52.48	5.701	0.9787
SPBC15D4.09C	met3	cystathionine gamma-synthase Met3	0.04178	-15.97	23.79	55.79	6.983	1.935
SPAC31A2.09C	apm4	AP-2 adaptor complex mu subunit Apm4 (predicted)	0.002469	-15.88	20.19	50.61	3.403	1.874
SPBC1105.02C	lys4	homocitrate synthase	0.0351	-15.72	21.53	52.27	6.526	2.977
SPAC683.02C	SPAC683.02c	zf-CCHC type zinc finger protein (predicted)	0.01676	-14.68	20.39	49.21	4.905	1.702
SPAC3F10.09	SPAC3F10.09	1-(5-phosphoribosyl)-5-[(5-phosphoribosylamino) methylideneamino]imidazole-4-carboxamide isomerase	0.02323	-14.56	22.57	52.1	5.033	0.9386
SPAC4G9.09C	arg11	N-acetyl-gamma-glutamyl-phosphate reductase/acetylglutamate kinase	0.05074	-14.52	22.64	52.14	6.823	2.107
SPAC890.05	SPAC890.05	ribosome biogenesis protein, G-patch domain, PINX1 family (predicted)	0.03511	-14.52	19.25	47.39	6.073	2.38
SPBC11B10.02C	his3	histidinol-phosphate aminotransferase imidazole acetol phosphate transaminase His3	0.01006	-14.37	18.2	45.69	4.19	2.327
SPCC188.13C	dcr1	dicer	0.00651	-14.18	20.01	47.96	3.696	2.182
SPAC343.16	lys2	homoaconitate hydratase Lys2	0.01906	-14	24.85	54.5	4.361	3.115
SPCC550.01C	coa4	mitochondrial respiratory chain complex assembly protein (predicted)	0.02517	-13.28	33.18	65.19	4.861	1.234
SPCC553.03	pex1	AAA family ATPase Pex1 (predicted)	0.0109	-12.85	36.73	69.57	3.885	1.847
SPAC2G11.03C	vps45	vacuolar sorting protein Vps45	0.001745	-12.66	24.01	51.45	2.05	0.4846
SPBC23G7.08C	rga7	RhoGAP, GTPase activating protein Rga7	0.02833	-12.5	22.49	49.09	3.654	3.38
SPCC584.01C	met10	sulfite reductase NADPH flavoprotein subunit (predicted)	0.004191	-12.28	22.22	48.42	2.388	0.3963
SPBC4F6.08C	mrpl39	mitochondrial ribosomal protein subunit L39 (predicted)	0.006463	-11.36	34.08	63.76	2.991	1.673
SPBC25B2.04C	mtg1	mitochondrial GTPase involved in translation Mtg1 (predicted)	0.006447	-11.34	21.49	46.07	3.011	1.567
SPBC28F2.10C	ngg1	SAGA complex subunit Ngg1/Ada3	0.04608	-10.97	25.01	50.47	4.385	3.06

SPAC144.17C	SPAC144.17c	6-phosphofructo-2-kinase (predicted)	0.03264	-10.68	36.47	66.15	4.296	2.134
SPAC323.01C	pos5	mitochondrial NADH kinase Pos5 (predicted)	0.01331	-10.31	5.453	22.12	2.021	2.341
SPAC1952.05	gcn5	SAGA complex histone acetyltransferase catalytic subunit Gcn5	0.005004	-9.594	18.06	38.8	2.039	1.658
SPBC16A3.16	coa5	mitochondrial inner membrane protein involved in cytochrome c oxidase assembly Coa5 (predicted)	0.001772	-9.59	35.52	63.29	1.888	1.152
SPBC18H10.16	can1	arginine transmembrane transporter Can1	0.04293	-9.367	36.82	64.81	3.952	2.27
SPCC777.10C	ubc12	NEDD8-conjugating enzyme Ubc12	0.03363	-9.163	28.09	52.27	3.696	1.899
SPBC725.11C	php2	CCAAT-binding factor complex subunit Php2	0.01027	-8.854	28.28	52.11	2.627	1.34
SPBC19C7.12C	omh1	alpha-1,2-mannosyltransferase Omh1	0.01063	-8.474	38.52	65.94	2.545	1.23
SPBC19C7.01	mni1	exon-exon junction complex disassembly factor Mni1 (predicted)	0.005376	-7.638	32.61	56.47	1.772	1.278
SPBC16H5.03C	uba2	SUMO activating enzyme E1-type Uba2 (predicted)	0.03885	-7.11	39.38	65.23	2.988	1.542
SPAC1250.03	ubc14	ubiquitin conjugating enzyme E2 Ubc14 (predicted)	0.01572	-7.031	38.22	63.5	1.596	1.658
SPCC794.03	SPCC794.03	amino acid permease (predicted)	0.02498	-6.792	35.08	58.75	2.133	1.698
SPAC11D3.14C	SPAC11D3.14c	5-oxoprolinase (ATP-hydrolizing) (predicted)	0.04426	-6.618	37.89	62.45	2.753	1.698
SPAC32A11.03C	phx1	stationary phase-specific homeobox transcription factor Phx1	0.01997	-6.552	35.47	58.96	2.082	1.463
SPAC4D7.03	pop2	F-box/WD repeat protein Pop2	0.006342	-6.512	37.41	61.63	0.6974	1.157
SPBP16F5.03C	tra1	SAGA complex phosphatidylinositol pseudokinase Tra1	0.008902	-6.218	34.53	57.18	1.425	1.244
SPBC3E7.16C	leu3	2-isopropylmalate synthase Leu3	0.04452	-5.817	42.73	68.12	1.709	1.842
SPAC14C4.16	dad3	DASH complex subunit Dad3	0.04158	-5.43	39.07	62.44	1.736	1.646
SPBC19C2.06C	mug124	Schizosaccharomyces pombe specific protein	0.04035	-5.333	38.11	60.96	1.871	1.538
SPAC18B11.09C	SPAC18B11.09c	serine O-acetyltransferase activity (predicted)	0.02582	-5.022	38.25	60.71	1.651	1.234
SPAC637.10C	rpn10	19S proteasome regulatory subunit Rpn10	0.04534	-4.563	37.79	59.43	1.654	1.363
SPAC20G4.01	caf16	CCR4-Not complex subunit Caf16 (predicted)	0.02809	3.242	35.12	44.73	1.264	0.5589
SPBC2G2.01C	liz1	pantothenate transmembrane transporter Liz1	0.03147	3.633	29.19	35.86	1.458	0.465
SPBC19G7.10C	pdc2	topoisomerase II-associated deadenylation-dependent mRNA-decapping factor Pdc2 (predicted)	0.02977	5.007	24.56	27.43	0.6833	1.38
SPAC16.05C	sfp1	transcription factor Sfp1 (predicted)	0.005799	5.038	32.41	38.4	0.5013	0.8657
SPBC1604.08C	imp1	importin alpha	0.05014	5.166	33.22	39.36	1.867	1.617
SPCC320.03	SPCC320.03	transcription factor (predicted)	0.01876	5.423	38.3	46.13	1.834	0.8287
SPAC27F1.05C	SPAC27F1.05c	aminotransferase class-III, unknown specificty	0.0179	5.623	38.08	45.54	1.775	1.015
SPAC23G3.08C	ubp7	ubiquitin C-terminal hydrolase Ubp7	0.02329	5.831	37.31	44.17	1.426	1.53
SPAC4F10.14C	btf3	nascent polypeptide-associated complex beta subunit	0.02966	6.123	24.42	25.68	0.9225	1.699
SPAC23H4.16C	SPAC23H4.16c	Schizosaccharomyces specific protein	0.04677	6.146	35.75	41.53	2.456	1.731
SPCC18.13	trm82	tRNA (guanine-N7-)-methyltransferase subunit Trm82 (predicted)	0.01549	6.529	33.54	37.9	2.078	1.235
SPBC16D10.02	trm11	tRNA (guanine-N2-)-methyltransferase catalytic subunit Trm11 (predicted)	0.04441	6.766	39.04	45.28	0.8096	1.683
SPAC22F8.07C	rtf1	replication termination factor Rtf1	0.02531	6.787	21.58	20.75	1.682	1.824
SPAC22F8.04	pet1	phosphoenolpyruvate transmembrane transporter Pet1	0.01419	6.824	24.77	25.17	2.208	1.018
SPCC1322.14C	vtc4	vacuolar transporter chaperone (VTC) complex subunit (predicted)	0.01318	7.101	37.43	42.56	1.365	1.339
SPBC4F6.06	kin1	microtubule affinity-regulating kinase Kin1	0.02278	7.115	19.93	17.97	1.834	1.842
SPBC1685.15C	klp6	kinesin-8 family plus-end microtubule motor Klp6	0.03723	7.413	37.44	42.13	1.628	1.914
SPAC3H1.12C	snt2	Lid2 complex PHD finger subunit Snt2	0.04455	7.628	34.31	37.44	3.285	1.821
SPAC27E2.03C	SPAC27E2.03c	Obg-Like ATPase (predicted)	0.006294	7.733	37.58	41.88	1.568	1.197
SPAC767.01C	vps1	dynamin family protein Vps1	0.04294	7.941	38.06	42.27	1.426	2.088
SPBP23A10.14C	ell1	RNA polymerase II transcription elongation factor SpELL	0.03864	7.99	37.57	41.5	1.506	2.441
SPAC13D6.02C	byr3	translational activator, zf-CCHC type zinc finger protein (predicted)	0.02637	7.998	30.31	31.3	2.162	2.151
SPCC132.01C	mtr1	microtubule regulator Mtr1	0.03619	8.021	37.48	41.34	2.662	2.271

SPAC2G11.12	rqh1	RecQ type DNA helicase Rqh1	0.02596	8.707	29.16	28.69	3.239	1.748
SPBC3B8.05	SPBC3B8.05	diphthamide biosynthesis protein Dph1 (predicted)	0.004754	9.029	43.06	47.75	2.2	1.293
SPBC1718.02	hop1	linear element associated protein Hop1	0.003219	9.294	43.3	47.71	2.117	1.072
SPCC16A11.01	sfk1	plasma membrane protein involved in inositol lipid-mediated signaling Sfk1 (predicted)	0.03946	9.448	38.86	41.28	3.302	2.395
SPAC6B12.14C	SPAC6B12.14c	conserved fungal protein	0.01308	9.5	43.44	47.61	2.415	2.071
SPCC162.03	SPCC162.03	short chain dehydrogenase (predicted)	0.02713	9.563	42.39	46.06	3.069	2.443
SPAC1002.01	SPAC1002.01	conserved fungal protein	0.03221	9.658	42.65	46.3	2.787	2.74
SPAC16E8.18	SPAC16E8.18	Schizosaccharomyces pombe specific protein	0.02175	9.776	41.12	43.97	2.914	2.379
SPAC1B3.16C	vht1	vitamin H transmembrane transporter Vht1	0.03867	10.46	32.04	30.28	4.474	2.021
SPBC1652.02	SPBC1652.02	APC amino acid transmembrane transporter (predicted)	0.01001	10.76	42.99	45.23	3.153	1.212
SPAC12B10.14C	tea5	pseudokinase Tea5	0.03083	11.01	43.3	45.31	3.874	2.782
SPAPB2B4.06	SPAPB2B4.06	acyl-coenzyme A thioesterase	0.02578	11.35	44.78	46.91	3.981	2.594
SPAC9.02C	SPAC9.02c	polyamine N-acetyltransferase (predicted)	0.009571	20.47	35.28	20.78	5.163	4.018
SPAC27F1.08	pdt1	Nramp family manganese ion transmembrane transporter	0.002758	26.55	39.12	17.64	5.731	2.394

Supplementary Table 7

R package version: 0.0-10
Summary type: mean   Based on 4 independent repeat experimens
Test type: t-test

Control medium: EMM2_ammonia +DMSO
Control screen ID: QFA0002
Control libraries: PDLV4_384
Query medium: EMM2_ammonia + 5um Torin1
Query screen ID: QFA0002

###

ORF	Gene	P	EGI	(p) -log10	Torin1 Fitness mean	DMSO_Fitness mean	Torin1 SE	DMSO SE
SPCC4E9.02	cig1	0.001074	-10.45	2.968995719	8.943	34.36	1.874	0.3581
SPBC21D10.11C	nfs1	0.0002309	-9.911	3.636576067	7.246	30.4	0.9279	0.09295
SPAC4F10.08	mug126	0.0007203	-9.817	3.142486585	6.25	28.47	1.738	1.158
SPCC663.03	pmd1	1.12E-06	-9.405	5.950007143	7.49	29.94	0.5597	0.3487
SPBC15D4.09C	SPBC15D4.09c	0.0001159	-9.363	3.935916564	0	16.59	0.6226	0
SPAC3H8.08C	SPAC3H8.08c	0.009334	-9.107	2.029932203	7.563	29.54	3.295	1.452
SPCC24B10.11C	mft1	0.01503	-8.785	1.823041019	3.333	21.47	1.181	1.935
SPAC26F1.04C	etr1	2.28E-07	-8.737	6.642827742	6.784	27.5	0.1737	0.1734
SPAC18G6.04C	shm2	0.04037	-8.247	1.393941251	6.836	26.73	2.96	2.536
SPBC36.07	iki3	0.01914	-8.148	1.718058067	2.853	19.49	3.35	1.721
SPCC70.04C	SPCC70.04c	0.1064	-8.065	0.973058372	8.559	29.46	1.566	3.574
SPAC1F7.09C	SPAC1F7.09c	0.0006646	-8.041	3.177439663	10.89	33.55	1.64	0.6077
SPBC27B12.03C	erg32	0.1304	-7.945	0.884722409	10.2	32.16	2.998	3.858
SPCC1840.10	lsm8	0.001419	-7.846	2.848017605	9.809	31.29	1.719	1.018
SPAC3H5.08C	SPAC3H5.08c	0.01662	-7.805	1.779368981	7.082	26.38	3.326	1.106
SPCC16C4.10	SPCC16C4.10	0.02212	-7.764	1.655214877	5.758	23.96	2.556	1.988
SPBC26H8.09C	snf59	0.0002721	-7.695	3.565271458	10.48	32.2	0.9931	0.2107
SPBC3E7.16C	leu3	0.000192	-7.65	3.716698771	11.15	33.32	0.784	0.126
SPAC3C7.07C	SPAC3C7.07c	0.0526	-7.636	1.279014256	7.434	26.71	1.719	2.577
SPAC328.01C	SPAC328.01c	0.03079	-7.565	1.511590311	9.944	31.03	3.3	1.943
SPAC16C9.06C	upf1	0.06801	-7.514	1.167427225	6.35	24.57	3.822	2.576
SPAC2G11.06	vps4	0.1353	-7.189	0.868702203	5.75	22.93	1.498	3.566
SPCC31H12.03C	SPCC31H12.03c	0.02894	-7.185	1.538501473	8.278	27.4	3.574	1.217
SPAC1851.03	ckb1	0.05633	-7.153	1.249260249	4.205	20.13	4.384	1.438
SPAC2F3.12C	plp1	0.071	-7.129	1.148741651	7.119	25.25	4.042	2.321
SPAC1834.04	hht1	0.0003582	-7.077	3.445874418	8.526	27.65	1.299	0.5411
SPCC1442.11C	SPCC1442.11c	0.0004827	-6.905	3.316322701	9.728	29.48	1.346	0.6082
SPAC1527.01	mok11	0.0007203	-6.876	3.142486585	10.14	30.15	1.39	0.7458
SPAC1F3.09	mug161	0.001063	-6.83	2.973466735	8.541	27.24	1.182	0.205
SPCC1235.13	ght6	0.002416	-6.767	2.61690307	13.57	36.04	1.542	0.3035
SPAC1783.06C	atg12	0.04475	-6.757	1.34920696	10.68	30.9	1.634	2.166
SPAC30D11.06C	SPAC30D11.06c	0.0001817	-6.735	3.740645073	9.453	28.69	1.07	0.4184
SPBC337.04	ppk27	0.03499	-6.722	1.456056058	10.65	30.79	1.422	1.978
SPBC25H2.03	SPBC25H2.03	0.06061	-6.694	1.217455716	9.575	28.83	2.153	2.389
SPBC2G2.06C	apl1	0.000892	-6.641	3.049635146	11.29	31.77	1.307	0.3566
SPBC36B7.03	sec63	0.1108	-6.63	0.95546024	12.4	33.72	3.154	2.921
SPAC227.05	SPAC227.05	2.89E-05	-6.546	4.539252458	11.08	31.23	0.5855	0.4254
SPBC19C7.12C	omh1	1.75E-05	-6.509	4.757210191	7.937	25.6	0.5773	0.3897
SPBC4B4.10C	atg5	0.0006939	-6.468	3.158703113	5.318	20.89	1.336	0.5026
SPBC2G2.05	rpl1603	0.0008298	-6.453	3.08102657	12.23	33.11	1.045	0.1842
SPAC31A2.14	bun107	0.03551	-6.426	1.449649328	5.361	20.89	1.502	1.911
SPAC31G5.19	abo1	0.08423	-6.358	1.074533199	7.169	23.97	4.481	1.497
SPCC11E10.06C	elp4	0.104	-6.309	0.982966661	4.506	19.17	3.424	2.607
SPAC3H8.07C	pac10	0.01108	-6.278	1.95546024	11.86	32.14	1.357	1.353
SPCC569.06	SPCC569.06	0.118	-6.261	0.928117993	10.74	30.13	4.545	2.269
SPAC18B11.04	ncs1	0.0006155	-6.254	3.210771943	7.931	25.14	1.149	0.3292
SPAC23C4.12	hhp2	0.0003854	-6.236	3.41408829	6.914	23.3	1.165	0.5107
SPBC28F2.08C	SPBC28F2.08c	3.04E-05	-6.161	4.51684079	7.504	24.22	0.6134	0.4068
SPBC1347.08C	SPBC1347.08c	0.0008063	-6.137	3.09350334	9.174	27.13	0.9395	0.1429
SPBC19G7.07C	ppr3	0.002417	-6.066	2.61672335	12.61	33.09	1.43	0.3152
SPBC6B1.05C	atg7	0.1743	-6.062	0.758702613	3.42	16.8	0.4953	3.42
SPBC405.05	SPBC405.05	0.01039	-6.061	1.983384452	6.857	22.89	2.029	1.186

SPCC191.09C	gst1	0.0008629	-6.023	3.064039531	11.67	31.36	1.215	0.3609
SPAP8A3.13C	SPAP8A3.13c	0.0002738	-6.021	3.562566556	13.97	35.42	1.053	0.4861
SPAC23G3.03	sib2	0.0001365	-5.994	3.864867349	9.429	27.33	0.921	0.4172
SPBC582.09	pex11	0.00204	-5.984	2.690369833	11.83	31.57	1.38	0.3279
SPAC2H10.02C	SPAC2H10.02c	0.001959	-5.931	2.707965564	12.56	32.77	1.44	0.4137
SPAC1B3.06C	SPAC1B3.06c	0.0004959	-5.925	3.304605892	11.25	30.44	1.162	0.5167
SPCC1682.11C	SPCC1682.11c	0.02834	-5.916	1.547600154	6.591	22.16	1.658	1.653
SPAC343.18	rfp2	0.001248	-5.895	2.903785415	9.274	26.88	0.6902	0.734
SPAC3C7.01C	SPAC3C7.01c	2.43E-05	-5.865	4.614930224	8.7	25.81	0.628	0.2438
SPAC1D4.06C	csk1	0.1235	-5.856	0.908333042	5.831	20.71	4.924	0.6548
SPAC29A4.20	elp3	0.04448	-5.787	1.351835221	8.801	25.85	2.269	1.813
SPBC1289.16C	cao2	0.01091	-5.751	1.962175249	12.33	32.04	1.893	0.2125
SPAC22H12.02	tfg3	0.1161	-5.701	0.93516778	5.476	19.81	3.006	2.516
SPCC1494.10	adn3	0.0001275	-5.701	3.894489815	7.727	23.8	0.4263	0.4323
SPBC337.03	SPBC337.03	0.02176	-5.686	1.662341109	12.56	32.34	2.536	1.109
SPBC660.17C	SPBC660.17c	0.05364	-5.671	1.270511231	11.44	30.32	2.451	1.864
SPBC21D10.09C	SPBC21D10.09c	2.42E-06	-5.644	5.616005211	9.734	27.25	0.4189	0.2237
SPAC1002.19	urg1	0.003061	-5.632	2.51413667	11.61	30.55	1.588	0.5574
SPAC3G9.04	ssu72	0.03836	-5.557	1.416121402	11.02	29.38	1.708	1.701
SPAC4F8.15	itr1	0.3269	-5.553	0.485585079	9.933	27.44	7.421	3.01
SPBC3B9.08C	mnh1	0.001146	-5.544	2.940815382	11.44	30.1	1.145	0.3021
SPAC13G6.10C	asl1	0.03742	-5.534	1.426896217	0.7238	11.09	2.962	0.7238
SPAC22H12.04C	rps102	0.3311	-5.515	0.480040819	11.18	29.59	2.738	4.677
SPCC74.06	mak3	0.0004177	-5.469	3.379135525	16.63	39.17	0.9998	0.354
SPAC6C3.03C	SPAC6C3.03c	0.001047	-5.457	2.980053318	9.96	27.32	0.4418	0.5952
SPCC162.02C	SPCC162.02c	0.178	-5.455	0.749579998	11.18	29.47	1.789	3.11
SPCC777.07	omh3	0.004659	-5.415	2.33170729	11.77	30.45	1.445	0.2481
SPAC10F6.11C	atg17	0.3729	-5.404	0.428407617	9.18	25.84	2.436	5.101
SPBC20F10.02C	SPBC20F10.02c	0.0005643	-5.388	3.24848995	11.37	29.7	0.9368	0.2486
SPCC16C4.07	scw1	0.001089	-5.355	2.96297212	10.03	27.26	1.077	0.2741
SPBC1105.01	rrp12	0.0002309	-5.353	3.636576067	11.57	30	0.9015	0.4376
SPAC890.07C	rmt1	0.000192	-5.349	3.716698771	11.89	30.54	0.8079	0.2679
SPCC1620.02	wtf23	0.0007189	-5.342	3.143331516	10.67	28.38	0.9594	0.2394
SPAC31G5.18C	sde2	0.009465	-5.297	2.023879382	4.575	17.49	1.852	0.9376
SPBPB2B2.02	mug180	0.0008895	-5.294	3.050854048	9.606	26.4	1.048	0.2905
SPBC16H5.04	SPBC16H5.04	0.002175	-5.281	2.662540739	11.06	28.96	1.057	0.7866
SPCC1919.15	brl1	0.09978	-5.277	1.0009565	0.8116	10.79	4.049	0.8116
SPBC1683.13C	cha4	0.0003482	-5.273	3.458171233	13.01	32.4	0.9253	0.325
SPBC29A3.09C	SPBC29A3.09c	0.0002196	-5.266	3.658367664	10.46	27.88	0.8841	0.4004
SPBC543.03C	pku80	0.002852	-5.246	2.544850479	13.44	33.11	1.335	0.3274
SPAC6F12.12	par2	0.001857	-5.225	2.731188096	9.951	26.89	1.237	0.3423
SPAC9E9.10C	cbh1	0.0009244	-5.216	3.034140063	12.35	31.13	1.122	0.3867
SPAC186.07C	SPAC186.07c	0.001646	-5.216	2.783570169	11.85	30.24	1.294	0.4913
SPBC577.06C	stt4	0.1112	-5.171	0.953895213	7.004	21.58	1.851	2.34
SPAC458.06	SPAC458.06	0.03409	-5.151	1.467372999	4.263	16.68	1.731	1.509
SPAC17C9.15C	SPAC17C9.15c	0.04547	-5.136	1.342275046	2.764	14	1.128	1.648
SPBC1734.05C	spf31	0.001828	-5.134	2.738023809	10.84	28.32	1.302	0.4895
SPAC3C7.06C	pit1	0.01902	-5.127	1.720789487	7.337	22.09	1.891	1.197
SPCC18B5.07C	nup61	0.00711	-5.115	2.148130399	11.73	29.85	1.721	0.5017
SPBC17G9.05	rct1	0.02402	-5.108	1.619426997	11.68	29.75	2.342	0.5016
SPBC23G7.12C	rpt6	0.004845	-5.107	2.314706219	11.42	29.28	1.539	0.4246
SPAC23A1.15C	sec20	0.0009268	-5.103	3.033013975	12.29	30.83	1.124	0.4341
SPBC31E1.01C	atg2	0.02113	-5.095	1.675100503	10.89	28.32	1.727	1.277
SPAC3H1.05	SPAC3H1.05	0.2667	-5.061	0.573976984	7.161	21.66	5.791	2.481
SPAC458.04C	dil1	0.001399	-5.057	2.854182286	11.61	29.53	0.9152	0.1405
SPAC4F10.06	SPAC4F10.06	0.005676	-5.043	2.245957613	11.44	29.2	1.441	0.259
SPAC2E1P3.04	cao1	0.2364	-5.02	0.626352528	9.425	25.6	0.33	3.4
SPCC1919.12C	SPCC1919.12c	0.007915	-5.004	2.101549081	11.59	29.4	1.745	0.5356
SPCC4B3.08	lsg1	0.2821	-5.001	0.549596914	15.32	36	2.224	3.765
SPBC56F2.11	met6	0.05011	-4.988	1.300075597	0	8.84	2.78	0
SPAC24B11.12C	SPAC24B11.12c	0.000132	-4.985	3.879426069	9.327	25.36	0.7238	0.4071
SPBC1734.12C	alg12	0.3109	-4.982	0.507379278	9.405	25.5	1.649	4.068
SPAC1399.02	SPAC1399.02	0.008778	-4.981	2.056604423	11.94	30	1.723	0.425
SPAC2F7.09C	SPAC2F7.09c	0.2276	-4.955	0.642827742	13.27	32.3	3.698	2.981
SPAP32A8.02	SPAP32A8.02	0.01708	-4.937	1.767512134	13.83	33.26	1.055	1.185
SPBC8E4.02C	SPBC8E4.02c	0.004466	-4.926	2.350081281	12.46	30.82	1.508	0.5055
SPAC17A5.16	ftp105	0.03518	-4.925	1.453704165	12.27	30.47	1.558	1.465
SPAC25A8.02	SPAC25A8.02	0.0304	-4.916	1.517126416	9.501	25.55	1.729	1.378
SPAC5H10.01	SPAC5H10.01	3.56E-05	-4.889	4.448062305	9.904	26.22	0.5456	0.198

SPBC1703.14C	top1	0.001883	-4.883	2.72514968	12.86	31.45	0.6137	0.6664
SPBC13A2.02	nup82	0.01111	-4.883	1.954285941	11.06	28.25	1.574	0.116
SPAC12G12.12	SPAC12G12.12	0.0005595	-4.874	3.252199909	8.375	23.48	0.8003	0.1887
SPAC24C9.12C	SPAC24C9.12c	0.2504	-4.823	0.601365675	8.727	24.01	3.514	3.144
SPBC1711.08	SPBC1711.08	0.07063	-4.815	1.151010794	12.2	30.15	2.274	1.731
SPAPYUG7.03C	mid2	0.0004777	-4.801	3.320844759	10.93	27.88	0.9157	0.3412
SPCC1620.14C	snf22	0.001996	-4.796	2.699839463	11.37	28.64	1.169	0.335
SPBC9B6.09C	mdl1	0.001684	-4.795	2.773657913	10.81	27.65	1.079	0.2758
SPCC1620.07C	SPCC1620.07c	0.001458	-4.791	2.836242476	11.13	28.21	0.9949	0.2258
SPBC651.06	mug166	0.4016	-4.785	0.396206296	11	27.97	6.56	3.796
SPBC19C7.05	SPBC19C7.05	0.0002203	-4.779	3.656985503	10.48	27.03	0.752	0.2559
SPBC947.05C	frp2	0.08781	-4.767	1.056456023	12.01	29.72	3.008	1.609
SPBC17A3.03C	SPBC17A3.03c	0.01761	-4.763	1.754240644	12.44	30.48	1.829	0.1752
SPBC11B10.06	sws1	0.0002858	-4.761	3.543937776	11.67	29.12	0.836	0.3461
SPAC23C4.09C	SPAC23C4.09c	0.009505	-4.758	2.022047879	13.11	31.67	1.682	0.4133
SPBC1D7.01	SPBC1D7.01	0.001783	-4.691	2.748848657	13.42	32.1	1.158	0.3857
SPAC2F7.11	nrd1	0.002287	-4.655	2.640733835	10.76	27.32	1.195	0.6163
SPAC3A11.13	SPAC3A11.13	0.0814	-4.649	1.089375595	14.62	34.14	0.9517	1.841
SPAC13A11.05	SPAC13A11.05	0.002291	-4.643	2.639974911	11.07	27.85	1.2	0.6079
SPAC3A12.08	SPAC3A12.08	0.002345	-4.627	2.629857153	14.26	33.48	1.193	0.3648
SPAC57A7.07C	SPAC57A7.07c	0.05328	-4.621	1.273435784	9.409	24.86	1.344	1.578
SPAC27D7.02C	SPAC27D7.02c	0.01035	-4.619	1.98505965	12.05	29.54	1.657	0.3842
SPAC926.05C	dph4	0.08597	-4.615	1.065653073	10.62	27	1.014	1.872
SPAC3G6.09C	tps2	0.4305	-4.613	0.366026844	10.92	27.52	3.246	4.908
SPAC664.07C	rad9	0.0007428	-4.606	3.129128105	10.87	27.42	0.9602	0.3531
SPBC32F12.03C	gpx1	0.0008797	-4.602	3.055665408	9.96	25.81	0.9014	0.2454
SPCC1020.06C	tal1	0.2675	-4.6	0.572676214	7.781	21.94	4.683	2.68
SPBC1604.11	atp17	0.000127	-4.591	3.896196279	9.392	24.78	0.5638	0.1526
SPBC428.10	SPBC428.10	0.3402	-4.586	0.468265691	10.34	26.45	2.107	3.991
SPAC589.07C	atg18	0.4385	-4.575	0.358030402	8.235	22.7	7.74	3.321
SPBC1711.11	SPBC1711.11	0.01319	-4.575	1.879755204	12.38	30.05	1.666	0.2635
SPCC4G3.05C	mus81	0.0001381	-4.563	3.859806321	11.27	28.06	0.6867	0.2685
SPAC1F7.06	SPAC1F7.06	0.0001611	-4.559	3.79290446	12.3	29.87	0.6574	0.3978
SPBC1271.10C	SPBC1271.10c	0.004949	-4.55	2.305482546	9.992	25.77	0.4054	0.7067
SPAC1B3.08	SPAC1B3.08	0.1715	-4.543	0.765735876	15.66	35.8	4.021	1.818
SPCC594.01	SPCC594.01	0.2108	-4.522	0.676129393	10.44	26.51	3.929	2.344
SPBC15D4.12C	mug98	0.001468	-4.493	2.833273944	13.63	32.11	1.094	0.4472
SPAC23G3.08C	ubp7	0.05819	-4.492	1.235151643	11.39	28.14	1.863	1.541
SPAC3H8.04	SPAC3H8.04	0.3066	-4.47	0.513427849	11.29	27.92	2.608	3.524
SPAC1F8.01	ght3	0.03985	-4.449	1.399571674	13.06	31.03	2.441	0.6509
SPBC1683.04	SPBC1683.04	0.0118	-4.446	1.928117993	10.47	26.43	1.47	0.1178
SPAC26A3.16	dph1	0.0003114	-4.432	3.506681392	10.8	26.99	0.7821	0.3949
SPCC553.01C	SPCC553.01c	0.01413	-4.416	1.849857838	8.161	22.29	1.234	1.014
SPBC16A3.13	meu7	0.005835	-4.411	2.23395914	12.6	30.14	1.445	0.5993
SPBC21B10.04C	nrf1	0.009898	-4.403	2.004452551	12.07	29.2	1.489	0.2651
SPAC25H1.02	jmj1	0.001335	-4.403	2.874518734	10.77	26.89	1.05	0.4233
SPBC30D10.09C	SPBC30D10.09c	0.1521	-4.402	0.817870786	12.8	30.48	2.116	2.265
SPCC613.07	SPCC613.07	0.003393	-4.396	2.46941614	11.16	27.57	1.274	0.4643
SPCC794.03	SPCC794.03	0.000769	-4.379	3.11407366	9.419	24.45	0.6885	0.5277
SPAC25G10.01	SPAC25G10.01	0.007361	-4.367	2.133063182	10.49	26.33	1.198	0.04664
SPCC297.05	SPCC297.05	0.02278	-4.363	1.64244628	8.364	22.55	1.933	0.354
SPAC4C5.02C	ryh1	0.1046	-4.357	0.980468315	0	7.722	3.352	0
SPCC18.15	SPCC18.15	0.001139	-4.341	2.943476276	11.11	27.37	0.2071	0.4196
SPCC895.06	elp2	0.4758	-4.326	0.322575562	8.808	23.28	8.372	3.056
SPCPB1C11.02	SPCPB1C11.02	0.06086	-4.324	1.215668052	12.65	30.09	1.774	1.512
SPAC9G1.04	oxa101	0.0007914	-4.307	3.101603954	11.88	28.69	0.4859	0.4911
SPBC15D4.01C	klp9	0.004473	-4.285	2.349401102	12.21	29.23	1.085	0.1514
SPBC713.03	SPBC713.03	0.007055	-4.285	2.151502982	14.01	32.42	1.421	0.3846
SPBC16G5.02C	SPBC16G5.02c	0.01063	-4.28	1.973466735	12.43	29.61	1.624	0.5554
SPAC22E12.01	SPAC22E12.01	0.002456	-4.268	2.609771638	12.45	29.63	1.153	0.4307
SPCC622.08C	hta1	0.004994	-4.267	2.301551462	11.93	28.7	1.29	0.3478
SPAC17D4.01	pex7	0.00318	-4.26	2.49757288	11.11	27.25	0.8772	0.03797
SPBC776.16	SPBC776.16	0.003985	-4.254	2.399571674	11.65	28.18	1.263	0.4128
SPBC16A3.02C	SPBC16A3.02c	0.01505	-4.24	1.8224635	12.77	30.15	1.679	0.3606
SPBC1271.12	kes1	0.2292	-4.239	0.639785387	5.283	16.88	3.057	2.592
SPAC24C9.15C	spn5	0.001732	-4.232	2.761452112	10.88	26.77	0.9834	0.2709
SPBC14F5.13C	SPBC14F5.13c	0.0001633	-4.225	3.787013815	8.558	22.65	0.5727	0.377
SPBC25B2.02C	mam1	0.0008277	-4.225	3.082127045	11.52	27.9	0.9067	0.4344
SPCC1223.09	SPCC1223.09	5.58E-05	-4.224	4.253677235	11.79	28.37	0.4662	0.3108

SPAC11G7.01	SPAC11G7.01	0.06437	-4.215	1.191316491	11.76	28.31	2.182	1.391
SPBC359.06	mug14	0.00224	-4.214	2.649751982	9.049	23.5	1.068	0.319
SPBC15D4.07C	atg9	0.06557	-4.203	1.183294816	9.172	23.7	1.046	1.54
SPAC1782.01	ecm29	0.2391	-4.195	0.621420424	13.34	31.07	1.998	2.81
SPCC1322.16	phb2	0.04256	-4.193	1.370998381	4.744	15.84	1.715	1.278
SPAPB8E5.03	mae1	0.00217	-4.188	2.663540266	12.63	29.8	1.105	0.4696
SPBPB8B6.04C	grt1	0.0002866	-4.182	3.542723814	11.83	28.38	0.6032	0.4161
SPAC25G10.06	rps2801	0.0003019	-4.173	3.520136887	12.55	29.63	0.7356	0.2924
SPCC1223.12C	meu10	0.006082	-4.172	2.215953584	11.38	27.56	1.184	0.1831
SPAC105.03C	SPAC105.03c	0.02538	-4.167	1.595508382	14.05	32.28	1.835	0.1966
SPBC146.02	SPBC146.02	0.09584	-4.163	1.018453195	9.519	24.25	3.134	0.8287
SPCC645.07	rgf1	0.04504	-4.156	1.346401618	9.057	23.42	2.017	1.189
SPAC664.02C	arp8	0.2163	-4.148	0.664943481	8.909	23.14	4.206	1.795
SPAC17H9.04C	SPAC17H9.04c	0.222	-4.138	0.653647026	7.159	20.02	4.755	0.7237
SPAC23C4.11	atp18	0.006032	-4.131	2.219538667	11.76	28.16	0.6357	0.7424
SPBC119.03	SPBC119.03	0.0001689	-4.125	3.77237035	12.54	29.53	0.6025	0.1973
SPBC2G2.15C	mrm2	0.0003106	-4.124	3.507798549	11.82	28.25	0.7038	0.3897
SPAC31G5.11	pac2	0.2722	-4.123	0.565111879	3.595	13.68	4.595	2.201
SPAC3F10.02C	trk1	0.2746	-4.11	0.561299467	6.581	18.95	4.589	2.221
SPAC24C9.07C	bgs2	0.0001907	-4.108	3.719649307	11.3	27.3	0.6296	0.3621
SPAC1142.01	SPAC1142.01	0.4228	-4.106	0.373865021	16.23	36.04	2.792	4.296
SPCC4B3.06C	SPCC4B3.06c	5.28E-05	-4.098	4.277201603	11.65	27.91	0.487	0.2927
SPBC21D10.12	hob1	0.03112	-4.095	1.506960412	11.54	27.7	1.536	1.14
SPCC63.04	mok14	0.01426	-4.088	1.845880474	12.79	29.91	1.563	0.2937
SPAC2C4.08	SPAC2C4.08	0.01458	-4.086	1.836242476	12.87	30.04	1.683	0.5849
SPBC19C7.01	mni1	0.000826	-4.082	3.083019953	8.683	22.62	0.7168	0.156
SPAC25B8.11	SPAC25B8.11	0.01883	-4.059	1.72514968	14.35	32.62	1.602	0.1645
SPAC22F8.03C	SPAC22F8.03c	3.97E-05	-4.054	4.401100113	12.88	30.01	0.3977	0.2804
SPBC1861.02	abp2	0.07367	-4.052	1.13270933	15.74	35.07	2.551	1.173
SPAC5H10.11	gmh1	0.0004709	-4.05	3.32707131	11.79	28.07	0.7739	0.2944
SPAC9G1.11C	spn4	0.1356	-4.04	0.86774031	13.14	30.45	1.894	1.967
SPAC1687.09	SPAC1687.09	0.01717	-4.025	1.765229705	12.55	29.37	1.611	0.2739
SPAP8A3.12C	tpp2	0.001094	-4.023	2.960982678	13.94	31.84	0.9199	0.4141
SPAC13G6.07C	rps601	0.001955	-4.023	2.708853238	12.96	30.09	0.9554	0.2565
SPBC646.15C	SPBC646.15c	0.1224	-4.017	0.912218582	9.723	24.35	2.008	1.841
SPBC16A3.19	eaf7	0.1465	-4.015	0.834162375	11.44	27.38	1.346	2.069
SPAC30D11.11	SPAC30D11.11	0.004848	-4.008	2.314437389	15.64	34.82	1.119	0.6703
SPAC105.02C	SPAC105.02c	0.000585	-4.008	3.232844134	12.24	28.79	0.709	0.4494
SPBPB7E8.02	SPBPB7E8.02	0.000772	-4.006	3.1123827	9.815	24.49	0.5784	0.07452
SPCC548.05C	SPCC548.05c	0.01551	-3.993	1.809388202	13	30.12	1.642	0.437
SPBC56F2.04	utp20	0.002769	-3.958	2.557677044	11.94	28.17	1.082	0.3605
SPAC630.13C	tsc2	0.001329	-3.957	2.876475019	11.49	27.38	0.8111	0.1898
SPCC663.15C	SPCC663.15c	0.001384	-3.951	2.85886391	12.71	29.52	0.8732	0.5049
SPAC30.04C	abc4	0.1344	-3.95	0.871600731	6.572	18.65	3.352	1.134
SPCC24B10.08C	ada2	0.1345	-3.944	0.871277716	9.716	24.21	1.712	1.928
SPAC1002.20	SPAC1002.20	0.00803	-3.921	2.095284455	12.67	29.39	1.368	0.6086
SPAC13C5.04	SPAC13C5.04	0.2252	-3.909	0.647431614	9.756	24.22	3.911	1.854
SPCC31H12.08C	ccr4	0.5425	-3.906	0.265600257	9.894	24.46	8.304	3.806
SPBC56F2.06	mug147	0.002558	-3.906	2.59209946	11.59	27.46	1.066	0.4047
SPBC23G7.14	SPBC23G7.14	0.01446	-3.889	1.839831707	13.48	30.78	1.59	0.49
SPAPB1A10.05	SPAPB1A10.05	0.0003178	-3.887	3.497846107	13.17	30.22	0.6945	0.3369
SPAC13G7.13C	msa1	0.4035	-3.884	0.394156461	6.603	18.59	6.318	2.354
SPBC800.03	clr3	0.04932	-3.879	1.306976932	7.835	20.76	0.96	1.285
SPCC188.08C	ubp5	0.004115	-3.878	2.38563016	12.63	29.25	1.082	0.6088
SPCC1259.11C	gyp2	0.2232	-3.858	0.65130581	12.77	29.46	1.861	2.471
SPBC16G5.03	SPBC16G5.03	0.0001928	-3.849	3.71489297	11.97	28.04	0.541	0.3537
SPBC29A3.03C	SPBC29A3.03c	0.05177	-3.827	1.285921835	11.42	27.01	1.465	1.274
SPBC13E7.07	SPBC13E7.07	0.00282	-3.827	2.549750892	11.16	26.56	1.057	0.3676
SPAC11G7.06C	mug132	0.006589	-3.815	2.181180492	11.9	27.86	0.4225	0.6603
SPAC5H10.13C	gmh2	0.2521	-3.804	0.598427154	13.83	31.25	1.792	2.64
SPBC1347.11	sro1	0.002959	-3.802	2.528855035	14.39	32.24	1.073	0.4356
SPBC16G5.06	SPBC16G5.06	0.1853	-3.797	0.732124581	10.68	25.65	3.118	1.829
SPBC947.06C	SPBC947.06c	0.00232	-3.79	2.634512015	11.83	27.69	0.7899	0.5707
SPCC18.01C	adg3	0.007715	-3.789	2.11266407	12.42	28.72	1.313	0.5753
SPCC1020.10	oca2	0.1653	-3.788	0.781727146	12.63	29.09	1.981	2.018
SPAC1B3.11C	ypt4	0.2717	-3.776	0.565910362	10.21	24.79	0.5934	2.807
SPCC794.09C	ef1a-a	0.2766	-3.77	0.558147824	10.91	26.01	4.51	1.797
SPAC23H4.12	alp13	0.002508	-3.757	2.600672468	12.67	29.11	0.9359	0.239
SPAC24C9.08	SPAC24C9.08	0.003126	-3.755	2.505011026	11.98	27.88	0.7572	0.6056

SPAC3G6.01	hrp3	0.446	-3.746	0.350665141	15.78	34.61	2.644	4.147
SPBC14C8.04	SPBC14C8.04	0.001781	-3.74	2.749336081	13.43	30.42	0.8164	0.5211
SPAC12G12.15	sif3	0.317	-3.739	0.498940738	8.935	22.46	2.374	3.004
SPAC110.01	ppk1	0.0005005	-3.732	3.300595918	11.94	27.78	0.5986	0.4126
SPCC1020.07	SPCC1020.07	0.001302	-3.724	2.885389016	9.527	23.48	0.853	0.4434
SPCC126.08C	SPCC126.08c	0.0208	-3.72	1.681936665	14.02	31.44	1.616	0.7505
SPBP23A10.02	pkr1	0.005052	-3.718	2.296536658	11.14	26.34	1.136	0.3169
SPAC5H10.06C	adh4	0.07514	-3.715	1.124128809	12.18	28.17	2.271	1.154
SPAC23A1.03	apt1	0.2312	-3.705	0.63601217	14.66	32.55	1.865	2.419
SPCC4G3.15C	not2	0.03449	-3.704	1.462306806	11.81	27.5	1.84	0.2304
SPCC1450.02	bdf1	0.3553	-3.701	0.449404793	6.541	18.15	4.905	2.442
SPAC6C3.04	cit1	0.0386	-3.697	1.413412695	11.47	26.88	1.541	1.077
SPBC577.11	SPBC577.11	0.002855	-3.68	2.544393887	11.1	26.19	0.9542	0.2491
SPAC29B12.06C	rcd1	0.08023	-3.67	1.095663208	8.902	22.28	2.556	0.8004
SPAC22F8.02C	pvg5	0.4354	-3.666	0.361111575	9.395	23.15	1.762	4.027
SPAC589.08C	dam1	0.02172	-3.65	1.663140179	13.26	29.97	1.672	0.5133
SPCC14G10.03C	ump1	0.3934	-3.646	0.405165644	4.931	15.2	5.632	2.325
SPCC1442.02	SPCC1442.02	0.4878	-3.645	0.311758204	9.161	22.69	3.887	4.297
SPBC1539.02	SPBC1539.02	0.004411	-3.644	2.355462942	12.27	28.19	1.077	0.3038
SPBC19C2.02	pmt1	0.007384	-3.641	2.131708312	9.952	24.09	1.244	0.5585
SPBC119.08	pmk1	0.07216	-3.632	1.141703475	9.82	23.84	2.085	1.181
SPAC14C4.06C	SPAC14C4.06c	0.226	-3.624	0.645891561	7.382	19.5	4.226	0.1705
SPBC947.04	SPBC947.04	0.4089	-3.623	0.388382889	15.32	33.57	3.244	3.535
SPCC1223.04C	set11	0.003811	-3.617	2.418961051	11.04	25.98	1.073	0.4682
SPAC4G9.14	SPAC4G9.14	0.3262	-3.61	0.486516043	14.64	32.34	2.731	2.901
SPAC26H5.10C	tif51	0.003726	-3.61	2.428757149	13.41	30.16	1.067	0.4611
SPCC1322.08	srk1	0.3987	-3.61	0.399353764	12.94	29.34	2.867	3.492
SPBC17D11.04C	nto1	0.1343	-3.586	0.871923987	14.36	31.81	1.683	1.737
SPAC22E12.04	ccs1	0.01148	-3.582	1.940058112	3.284	12.17	1.387	0.5072
SPCC132.04C	gdh2	0.1661	-3.582	0.779630368	9.309	22.84	2.537	1.749
SPAC144.04C	spe1	0.175	-3.58	0.756961951	11.13	26.07	0.5255	2.026
SPCC645.13	SPCC645.13	0.1421	-3.577	0.847405922	13.17	29.68	2.375	1.625
SPAC57A10.10C	sla1	0.07246	-3.573	1.13990167	8.776	21.88	0.5113	1.336
SPCC24B10.20	SPCC24B10.20	4.06E-05	-3.572	4.391902054	11.93	27.47	0.3724	0.2482
SPCC417.06C	mug27	0.1302	-3.555	0.885389016	13.41	30.06	2.338	1.531
SPBC83.05	SPBC83.05	0.01531	-3.549	1.815024809	12.52	28.48	1.456	0.3923
SPCC569.07	SPCC569.07	0.001063	-3.537	2.973466735	11.88	27.33	0.5723	0.4541
SPBC17D1.02	dph2	0.05404	-3.528	1.26728466	12.32	28.08	1.602	1.144
SPCC736.13	SPCC736.13	0.02318	-3.527	1.634886568	13.51	30.19	1.63	0.4252
SPBC2G2.08	ade9	0.0008432	-3.524	3.074069402	10.26	24.43	0.7519	0.2719
SPAP27G11.10C	nup184	0.007875	-3.521	2.103749438	11.96	27.43	1.219	0.3586
SPAC227.10	SPAC227.10	0.003801	-3.514	2.42010213	12.57	28.5	1.008	0.2933
SPAC4F10.04	SPAC4F10.04	0.2553	-3.507	0.592949185	4.101	13.48	4.109	1.444
SPAC7D4.04	taf1	0.3065	-3.502	0.513569521	9.741	23.47	1.909	2.775
SPAC823.16C	mug179	0.5026	-3.494	0.298777516	9.199	22.49	5.235	3.883
SPAC458.02C	SPAC458.02c	0.1934	-3.488	0.71354353	12.49	28.32	1.746	2.042
SPAC144.02	iec1	0.1034	-3.477	0.985479461	10.4	24.6	2.548	1.055
SPAC14C4.13	rad17	0.002918	-3.461	2.534914712	12.12	27.62	0.9649	0.3378
SPAC15A10.13	ppk3	0.007018	-3.46	2.153786636	12.43	28.15	0.9072	0.6629
SPCC1183.10	wtf10	0.00123	-3.448	2.910094889	13.69	30.38	0.7256	0.4387
SPBC13E7.11	SPBC13E7.11	0.005413	-3.445	2.266561973	10.8	25.25	0.5893	0.6148
SPAC19A8.04	erg5	0.06268	-3.424	1.202871012	10.7	25.02	2.103	0.8679
SPAC630.05	gyp7	0.0013	-3.411	2.886056648	13.91	30.7	0.7231	0.4396
SPAC1D4.01	SPAC1D4.01	0.09786	-3.409	1.009394789	0.8002	7.46	2.562	0.8002
SPAC22F3.03C	rdh54	0.4902	-3.407	0.309626693	14.15	31.1	3.736	4.021
SPBC32H8.08C	omh5	0.538	-3.399	0.269217724	8.126	20.42	7.196	3.233
SPBC1685.04	SPBC1685.04	0.2213	-3.395	0.655018586	12.46	28.1	1.716	2.154
SPBC12C2.05C	bzz1	0.2804	-3.393	0.552221991	10.23	24.14	1.19	2.562
SPAC32A11.03C	phx1	0.01016	-3.392	1.993106292	11.27	25.98	1.207	0.2758
SPCC1919.13C	SPCC1919.13c	0.01036	-3.389	1.984640245	14.54	31.77	1.279	0.4687
SPCC320.07C	mde7	0.007204	-3.382	2.142426296	13.58	30.06	1.166	0.4686
SPBC12C2.12C	glo1	0.2646	-3.381	0.57741016	12.96	28.97	3.078	2.116
SPAC7D4.12C	SPAC7D4.12c	0.6589	-3.358	0.181180492	15.85	34.04	4.885	6.501
SPAC869.02C	SPAC869.02c	0.0002874	-3.356	3.541513236	11.81	26.89	0.52	0.3298
SPBC14C8.05C	meu17	0.3454	-3.338	0.461677667	12.13	27.41	2.262	2.866
SPBC31F10.02	SPBC31F10.02	0.2118	-3.337	0.674074044	12.16	27.47	2.494	1.899
SPCC4B3.11C	SPCC4B3.11c	0.0007004	-3.331	3.154653863	12.56	28.16	0.6676	0.3613
SPCC24B10.06	SPCC24B10.06	0.029	-3.329	1.537602002	13.72	30.21	1.537	0.1624
SPCC553.03	pex1	0.02391	-3.321	1.621420424	12.2	27.5	1.508	0.6796

SPAC17C9.07	alg8	0.2781	-3.32	0.555799011	9.115	22.04	2.531	2.324
SPBC342.01C	alg6	0.3658	-3.318	0.436756299	3.653	12.35	4.655	2.127
SPBC685.02	exo5	0.00275	-3.317	2.560667306	11.48	26.22	0.8977	0.287
SPAC1783.05	hrp1	0.2907	-3.315	0.536554968	12.32	27.71	3.229	2.195
SPAC821.06	spn2	0.1368	-3.306	0.863913903	13.14	29.15	0.7358	1.65
SPBC2D10.11C	nap2	0.1823	-3.305	0.739213331	16.19	34.55	1.775	1.854
SPAPB1A11.04C	SPAPB1A11.04c	0.4744	-3.305	0.32385532	15.19	32.78	3.702	3.708
SPAC328.09	SPAC328.09	0.3058	-3.304	0.514562519	12.28	27.62	2.153	2.57
SPCC1235.15	dga1	0.001413	-3.301	2.849857838	12.2	27.48	0.5921	0.4501
SPAC31G5.12C	maf1	0.4703	-3.293	0.327625021	7.546	19.21	6.249	2.341
SPCC830.07C	psi1	0.3676	-3.292	0.434624497	9.486	22.64	2.845	2.887
SPBC1703.04	mlh1	0.07849	-3.29	1.105185671	14.15	30.9	1.377	1.27
SPBC4F6.08C	mrpl39	0.01514	-3.281	1.819874125	11.06	25.42	1.364	0.4853
SPBC2G5.06C	hmt2	0.0003759	-3.277	3.424927674	5.314	15.22	0.5794	0.3206
SPAC23G3.04	ies4	0.08703	-3.276	1.060331016	15.02	32.42	1.961	1.159
SPBC26H8.05C	SPBC26H8.05c	0.1963	-3.273	0.7070797	8.389	20.67	3.314	1.149
SPAC1834.09	mug51	0.02355	-3.266	1.628009089	12.55	28.04	1.532	0.5166
SPAC24B11.10C	chr3	0.1761	-3.265	0.754240644	11.98	27.01	1.454	1.83
SPAC17G6.13	slt1	0.1648	-3.25	0.783042793	14.1	30.75	2.52	1.482
SPAC31A2.13C	sft1	0.05112	-3.248	1.291409155	9.018	21.74	0.5874	1.073
SPAC1250.02	mug95	0.2641	-3.246	0.578231599	10.44	24.25	1.63	2.317
SPAC1952.05	gcn5	0.0642	-3.242	1.192464972	5.339	15.21	1.358	1.154
SPBC1604.01	mug158	0.03143	-3.241	1.502655619	13.9	30.38	1.644	0.5983
SPBC19C2.06C	mug124	0.001795	-3.237	2.745935547	11.74	26.55	0.8123	0.2917
SPCC1450.09C	SPCC1450.09c	0.0008753	-3.225	3.057843072	14.72	31.79	0.5415	0.399
SPBC1683.02	SPBC1683.02	0.004124	-3.214	2.384681343	11.6	26.25	0.9732	0.4119
SPBC1703.13C	SPBC1703.13c	0.01428	-3.208	1.845271793	11.85	26.68	1.29	0.3467
SPBPB2B2.13	gal1	0.4679	-3.208	0.329846955	6.115	16.52	5.714	2.577
SPCC1795.09	yps1	0.2933	-3.208	0.532687937	11.49	26.05	2.851	2.241
SPBC19C7.09C	uve1	0.2142	-3.208	0.669180534	14.49	31.36	1.871	1.961
SPAC17A5.10	SPAC17A5.10	0.007859	-3.206	2.104632711	12.57	27.96	1.123	0.3634
SPAC13G7.11	SPAC13G7.11	0.3221	-3.206	0.492009275	15.89	33.83	1.748	2.644
SPBC13E7.08C	SPBC13E7.08c	0.2096	-3.205	0.678608722	10.25	23.84	2.086	1.892
SPBC19F8.03C	SPBC19F8.03c	0.2762	-3.195	0.558776326	15.13	32.48	1.104	2.388
SPBC31F10.17C	SPBC31F10.17c	0.2043	-3.191	0.689731633	14.39	31.15	1.666	1.922
SPAC30D11.05	aps3	0.3125	-3.188	0.505149978	12.62	28.01	3.05	2.298
SPBC1604.18C	SPBC1604.18c	0.1911	-3.184	0.718739313	9.984	23.34	1.854	1.813
SPCC1753.05	rsm1	0.4949	-3.183	0.305482546	8.612	20.9	6.339	2.47
SPAC1952.17C	SPAC1952.17c	0.5467	-3.181	0.262250926	8.263	20.28	7.015	2.988
SPAC29A4.13	SPAC29A4.13	0.01652	-3.16	1.781989957	13.09	28.8	1.29	0.6079
SPBC21C3.03	SPBC21C3.03	0.003387	-3.157	2.470184803	10.55	24.3	0.6719	0.5177
SPAPB2B4.02	grx5	0.305	-3.149	0.515700161	8.811	21.19	4.49	0.5799
SPBC839.02	SPBC839.02	0.3498	-3.146	0.456180195	10.87	24.84	3.796	2.244
SPAC821.09	eng1	0.2215	-3.144	0.654626269	15.12	32.36	2.023	1.931
SPAC23C4.08	rho3	0.109	-3.142	0.962573502	10.35	23.91	0.8535	1.413
SPAC630.09C	mug58	0.1184	-3.141	0.926648298	12.2	27.18	0.8338	1.465
SPCC1393.13	SPCC1393.13	0.5856	-3.13	0.232398932	7.889	19.53	8.143	2.777
SPBC23G7.06C	SPBC23G7.06c	0.001867	-3.129	2.728855682	11.88	26.6	0.7947	0.3636
SPCC16A11.10C	oca8	0.2844	-3.125	0.546070408	10.51	24.17	4.242	0.4809
SPCC736.09C	SPCC736.09c	0.2772	-3.125	0.557206774	14.94	32.01	1.372	2.325
SPBC16C6.05	SPBC16C6.05	0.2045	-3.121	0.689306688	14.56	31.33	1.433	1.902
SPBC1703.12	ubp9	0.3375	-3.116	0.471726223	7.148	18.19	3.819	2.072
SPBC1604.19C	SPBC1604.19c	0.1862	-3.111	0.730020323	12.55	27.76	1.379	1.799
SPBC30B4.03C	adn1	0.1676	-3.107	0.775725986	12.65	27.93	1.84	1.63
SPAC4H3.01	SPAC4H3.01	0.3695	-3.105	0.432385557	13.77	29.91	3.649	2.442
SPBC1703.06	pof10	0.01618	-3.097	1.791021483	13.12	28.74	1.242	0.2481
SPAC10F6.08C	nht1	0.06703	-3.096	1.173730781	8.4	20.37	2.021	0.6292
SPAC23D3.04C	gpd2	0.0282	-3.084	1.549750892	7.632	18.99	1.48	0.2972
SPAC144.06	apl5	0.0584	-3.076	1.233587153	9.406	22.12	1.284	1.055
SPCC70.06	SPCC70.06	0.01228	-3.069	1.910801633	10.52	24.09	1.15	0.2571
SPBC530.13	lsc1	0.1525	-3.061	0.816730156	10.96	24.85	0.8628	1.612
SPBC18E5.01	SPBC18E5.01	0.01417	-3.06	1.84863015	12.86	28.22	1.251	0.4252
SPCC576.14	dph5	0.2947	-3.06	0.530619864	13.24	28.89	1.875	2.327
SPBP35G2.05C	cki2	0.3459	-3.049	0.461049438	15.86	33.52	2.985	2.423
SPCP1E11.06	apl4	0.4246	-3.043	0.37202001	4.694	13.71	5.454	1.6
SPBC17D1.05	SPBC17D1.05	0.04585	-3.042	1.33866066	8.699	20.81	1.7	0.6879
SPCC320.03	SPCC320.03	0.01149	-3.034	1.939679971	15.13	32.18	0.3842	0.617
SPBC1703.09	SPBC1703.09	0.007732	-3.021	2.111708155	15.38	32.61	1.039	0.4758
SPAC144.17C	SPAC144.17c	0.003735	-3.005	2.427709394	10.7	24.29	0.8547	0.2447

SPBC1685.08	cti6	0.002328	-2.997	2.633017024	12.07	26.7	0.7812	0.2518
SPAC13A11.04C	ubp8	0.3047	-2.988	0.516127546	11.33	25.38	3.529	1.762
SPCC576.17C	SPCC576.17c	0.01111	-2.986	1.954285941	10.89	24.6	1.016	0.1464
SPAC23A1.09	SPAC23A1.09	0.03104	-2.985	1.508078287	13.21	28.7	1.363	0.7359
SPBP8B7.07C	set6	0.0002933	-2.977	3.532687937	12.71	27.79	0.4778	0.2898
SPAC9.13C	cwf16	0.6052	-2.971	0.218101081	9.317	21.78	7.423	3.469
SPBC31F10.12	SPBC31F10.12	0.2968	-2.962	0.527536103	16.09	33.76	1.647	2.286
SPCP1E11.02	ppk38	0.002112	-2.956	2.675306086	13.22	28.67	0.7052	0.4128
SPCC24B10.02C	SPCC24B10.02c	0.005735	-2.954	2.241466578	12.86	28.02	0.4808	0.5299
SPCC18.13	SPCC18.13	0.1834	-2.951	0.736600669	7.92	19.27	0.6554	1.715
SPAC343.07	mug28	0.3237	-2.951	0.489857301	13.71	29.52	2.363	2.329
SPAC3H8.02	SPAC3H8.02	0.3425	-2.949	0.465339424	2.601	9.836	3.833	1.866
SPAC9.07C	SPAC9.07c	0.3329	-2.947	0.477686205	15.96	33.51	1.853	2.469
SPBC25H2.15	SPBC25H2.15	0.1006	-2.945	0.997402019	0.673	6.411	2.268	0.4692
SPAC30.03C	tsn1	0.407	-2.945	0.390405591	11.85	26.22	2.189	2.935
SPAC16.01	rho2	0.5473	-2.941	0.261774552	8.774	20.76	7.607	0.9418
SPCC1259.09C	pdx1	0.0009939	-2.939	3.002657309	12.93	28.12	0.4446	0.3702
SPAC20H4.03C	tfs1	0.0001066	-2.93	3.972242795	8.393	20.07	0.2613	0.2298
SPBC2F12.09C	atf21	0.5491	-2.916	0.260348556	7.501	18.46	6.659	2.586
SPCC1259.03	rpa12	0.02661	-2.916	1.574955125	13.18	28.52	1.302	0.132
SPAC922.06	SPAC922.06	0.005438	-2.912	2.264560797	13.23	28.61	0.8299	0.1563
SPAC14C4.10C	SPAC14C4.10c	0.2817	-2.91	0.550213153	13.09	28.36	0.9853	2.206
SPBC1711.14	rec15	0.629	-2.908	0.201349355	10.14	23.12	7.71	3.692
SPAC9.12C	atp12	0.3408	-2.9	0.467500414	7.053	17.64	2.081	2.442
SPBC409.08	SPBC409.08	0.5883	-2.895	0.230401152	8.624	20.41	6.989	3.157
SPBC839.06	cta3	0.54	-2.891	0.26760624	15.15	31.96	2.485	4.054
SPBC405.03C	SPBC405.03c	0.002715	-2.886	2.566230166	11.56	25.6	0.5201	0.4495
SPAC9G1.08C	SPAC9G1.08c	0.6259	-2.876	0.203495048	10.04	22.88	7.257	3.817
SPAC227.04	SPAC227.04	0.5556	-2.872	0.255237763	9.379	21.71	7.341	1.669
SPAC227.17C	SPAC227.17c	0.2333	-2.867	0.632085261	14.4	30.6	2.05	1.781
SPBC15C4.02	SPBC15C4.02	0.1973	-2.866	0.704872915	11.71	25.83	2.112	1.557
SPAC630.04C	SPAC630.04c	0.2917	-2.856	0.535063571	14.65	31.02	3.053	1.772
SPBC106.05C	tim11	0.003364	-2.855	2.473144013	12.17	26.62	0.601	0.4676
SPBC609.04	caf5	0.4225	-2.846	0.374173287	14.19	30.19	2.635	2.865
SPBC16G5.13	ptf2	0.01246	-2.841	1.904481958	12.2	26.66	1.094	0.2843
SPBC336.14C	ppk26	0.2832	-2.841	0.547906751	9.422	21.73	1.471	2.128
SPAC57A7.05	SPAC57A7.05	0.438	-2.835	0.358525889	10.23	23.16	3.837	2.628
SPAPB1A10.12C	alo1	0.4177	-2.816	0.379135525	12.17	26.55	3.538	2.532
SPBPB7E8.01	SPBPB7E8.01	0.2573	-2.816	0.589560214	1.854	8.277	2.133	1.854
SPBC543.02C	SPBC543.02c	0.005026	-2.813	2.298777516	12.04	26.31	0.8074	0.1733
SPAPB1E7.07	glt1	0.1822	-2.812	0.739451627	11.89	26.06	0.9248	1.624
SPAC25B8.13C	isp7	0.2945	-2.808	0.530914701	8.948	20.83	3.763	1.014
SPAC4F10.20	grx1	0.3296	-2.807	0.482012797	13.55	28.99	1.486	2.364
SPBC16H5.12C	SPBC16H5.12c	0.007723	-2.806	2.112213965	11.94	26.13	0.984	0.3434
SPAC30D11.07	nth1	0.2157	-2.805	0.666149855	11.75	25.79	1.565	1.733
SPAC30D11.12	rpl3802	0.2743	-2.803	0.561774192	12.32	26.8	1.594	2.036
SPBC29B5.04C	SPBC29B5.04c	0.1654	-2.803	0.781464495	10.78	24.06	0.6085	1.54
SPAC20G4.07C	sts1	0.2397	-2.792	0.620331966	1.932	8.372	3.131	1.133
SPAC22E12.05C	rer1	0.2021	-2.79	0.694433686	12.66	27.39	1.323	1.684
SPAC27F1.03C	uch1	0.2506	-2.785	0.601018933	14.98	31.48	2.617	1.615
SPCC330.12C	sdh3	0.085	-2.778	1.070581074	18.95	38.5	1.786	0.8891
SPCC1840.08C	SPCC1840.08c	0.5655	-2.777	0.247567391	7.569	18.34	6.234	2.899
SPBC902.06	mto2	0.2884	-2.775	0.540004744	13.6	29.02	1.052	2.135
SPAC27D7.13C	ssm4	0.03831	-2.769	1.416687848	13.23	28.35	1.498	0.3873
SPAC22A12.16	SPAC22A12.16	0.314	-2.739	0.503070352	13.51	28.8	1.688	2.19
SPAC9.06C	SPAC9.06c	0.2379	-2.739	0.623605558	14.14	29.9	1.333	1.826
SPAC4G9.10	arg3	0.3142	-2.726	0.502793819	11.71	25.58	1.577	2.194
SPAC17G6.17	pof8	0.5429	-2.714	0.265280158	6.357	16.08	6.282	2.173
SPBC215.02	bob1	0.2478	-2.714	0.605898698	15.44	32.18	0.774	1.889
SPBC543.07	pek1	0.193	-2.702	0.714442691	11.65	25.44	0.9711	1.608
SPCC794.02	wtf5	0.6924	-2.698	0.159642941	11.18	24.6	8.334	4.482
SPAC23C11.06C	SPAC23C11.06c	0.633	-2.697	0.19859629	11.46	25.09	5.936	4.173
SPAC22G7.11C	SPAC22G7.11c	0.6016	-2.696	0.220692172	8.584	19.99	6.961	2.879
SPAC3A11.02	cps3	0.6409	-2.68	0.193209728	7.454	17.96	7.717	3.252
SPBC651.05C	dot2	0.4453	-2.67	0.351347305	11.19	24.55	3.164	2.698
SPAPB24D3.02C	SPAPB24D3.02c	0.2068	-2.67	0.684449466	14.75	30.87	1.969	1.501
SPBC1683.07	mal1	0.4554	-2.667	0.341606974	18.45	37.42	1.5	3.063
SPBC1709.06	dus2	0.2661	-2.665	0.574955125	13.01	27.79	2.444	1.67
SPAC1071.04C	spc2	0.4042	-2.653	0.393403691	5.207	13.93	3.792	2.041

SPCC1259.10	pgp1	0.3457	-2.652	0.46130062	14.86	31.04	0.9356	2.356
SPCC736.02	SPCC736.02	0.2653	-2.64	0.57626275	12.61	27.02	1.737	1.837
SPAC3C7.14C	obr1	0.3746	-2.629	0.426432227	15.2	31.59	1.781	2.433
SPBC18H10.06C	swd2	0.4487	-2.623	0.34804393	12.53	26.85	4.738	2.033
SPAC11D3.14C	SPAC11D3.14c	0.001661	-2.622	2.779630368	12.89	27.48	0.5722	0.3581
SPBC16A3.12C	SPBC16A3.12c	0.2753	-2.62	0.560193789	13.46	28.5	1.059	1.946
SPBC1815.01	eno101	0.001674	-2.618	2.776244546	12.71	27.17	0.2914	0.3394
SPAC1250.03	ubc14	0.01669	-2.611	1.777543663	11.05	24.2	0.8812	0.6058
SPBC16H5.11C	skb1	0.3645	-2.609	0.438302467	14.56	30.43	1.78	2.351
SPBC30D10.10C	tor1	0.0005172	-2.606	3.286341484	12.09	26.05	0.515	0.2187
SPAC22H12.03	SPAC22H12.03	0.1939	-2.602	0.712422191	11.93	25.75	0.9595	1.552
SPAC17A5.05C	SPAC17A5.05c	0.05055	-2.597	1.29627884	14.84	30.89	1.541	0.3852
SPAC140.03	arb1	0.4653	-2.597	0.332266947	13.49	28.51	3.28	2.733
SPAC105.01C	kha1	0.03241	-2.594	1.489320969	13.98	29.37	1.334	0.4468
SPAC26H5.07C	SPAC26H5.07c	0.563	-2.589	0.249491605	6.078	15.36	5.648	2.776
SPBC106.03	SPBC106.03	0.2659	-2.585	0.575281663	13.29	28.13	2.347	1.627
SPAC6B12.05C	ies2	0.1279	-2.581	0.893129456	10.74	23.61	1.647	1.119
SPAC5D6.04	SPAC5D6.04	0.1764	-2.579	0.753501419	11.98	25.8	1.378	1.42
SPBC29A3.07C	sab14	0.05023	-2.578	1.299036822	7.635	18.1	1.349	0.7303
SPBC1773.02C	SPBC1773.02c	0.4018	-2.571	0.395990068	16.35	33.53	1.427	2.584
SPBC359.05	abc3	0.2946	-2.567	0.530767257	12.38	26.48	2.539	1.708
SPAC29A4.17C	SPAC29A4.17c	0.5832	-2.558	0.234182485	10.23	22.66	2.139	4.067
SPAC6F6.01	cch1	0.1937	-2.557	0.712870379	11.41	24.75	1.286	1.497
SPAPB1E7.12	rps602	0.01993	-2.545	1.700492701	13.58	28.58	1.135	0.4237
SPAC20G8.07C	erg2	0.396	-2.543	0.402304814	15.77	32.45	1.859	2.468
SPCC1322.03	SPCC1322.03	0.4203	-2.543	0.37644061	9.508	21.35	4.746	0.7776
SPAC12B10.01C	SPAC12B10.01c	0.2148	-2.54	0.667965723	12.03	25.82	1.304	1.58
SPBC1861.06C	mug131	0.3375	-2.538	0.471726223	14.48	30.16	2.252	2.031
SPCC757.03C	SPCC757.03c	0.002871	-2.536	2.541966808	12.99	27.51	0.5306	0.401
SPAC683.02C	SPAC683.02c	0.1986	-2.526	0.702020756	8.579	19.68	2.082	1.292
SPBC215.13	SPBC215.13	0.328	-2.524	0.484126156	12.12	25.96	1.246	2.125
SPBC776.03	SPBC776.03	0.3589	-2.521	0.445026542	14.83	30.75	1.315	2.285
SPBC16H5.03C	fub2	0.05117	-2.513	1.290984583	14.81	30.71	1.468	0.2415
SPBC3H7.10	elp6	0.3411	-2.498	0.467118281	4.607	12.59	3.159	1.63
SPBC27B12.08	sip1	0.24	-2.489	0.619788758	9.095	20.53	2.973	0.6274
SPBC660.07	ntp1	0.5335	-2.488	0.272865576	9.09	20.52	2.185	3.421
SPBCPT2R1.02	SPBCPT2R1.02	0.3573	-2.479	0.446966984	13.88	28.99	1.49	2.219
SPAC25G10.02	cce1	0.4128	-2.476	0.384260311	12.9	27.25	2.032	2.475
SPBC19F5.01C	puc1	0.07267	-2.473	1.13864484	13.14	27.67	1.643	0.209
SPBC17G9.02C	SPBC17G9.02c	0.3129	-2.469	0.504594437	15.23	31.36	1.896	1.906
SPBC18H10.16	can1	0.03953	-2.465	1.403073186	11.44	24.63	1.319	0.242
SPBC215.03C	csn1	0.1974	-2.448	0.704652852	8.603	19.58	1.375	1.43
SPBC2A9.13	SPBC2A9.13	0.3537	-2.447	0.45136494	13.01	27.39	2.034	2.082
SPAPB17E12.13	rpl1802	0.4737	-2.44	0.324496615	10.86	23.56	4.224	2.121
SPCC1235.09	hif2	0.2915	-2.438	0.535361441	13.67	28.55	2.279	1.655
SPAC23C11.08	php3	0.2352	-2.419	0.628562683	13.08	27.46	0.8973	1.62
SPBC2D10.20	ubc1	0.5151	-2.417	0.28810845	13.29	27.84	2.04	3.168
SPBC18H10.18C	SPBC18H10.18c	0.2805	-2.406	0.552067134	12.8	26.94	1.636	1.737
SPCC1393.02C	spt2	0.02815	-2.395	1.550521601	10.22	22.36	0.8463	0.6535
SPBC30D10.05C	SPBC30D10.05c	0.3502	-2.392	0.455683858	12.59	26.56	2.056	2.001
SPBC530.01	gyp1	0.1433	-2.389	0.84375381	6.905	16.47	1.909	0.9153
SPBC13G1.03C	pex14	0.4545	-2.383	0.342466112	8.29	18.91	4.317	1.672
SPBC1E8.02	SPBC1E8.02	0.5448	-2.381	0.263762901	16.56	33.57	3.364	3.134
SPACUNK4.09	SPACUNK4.09	0.2805	-2.379	0.552067134	14.01	29.05	1.614	1.718
SPBC609.05	pob3	0.39	-2.373	0.408935393	6.563	15.84	2.07	2.21
SPAC227.18	lys3	0.000819	-2.373	3.086716098	14.43	29.78	0.5129	0.211
SPBC839.07	ibp1	0.005123	-2.372	2.290475644	11.75	25.02	0.4639	0.4249
SPBC14F5.07	doa10	0.4491	-2.364	0.347656945	12.61	26.54	2.084	2.579
SPCC663.13C	naa50	0.379	-2.354	0.42136079	16.6	33.59	1.109	2.252
SPBC651.03C	gyp10	0.6685	-2.353	0.174898588	13.22	27.6	7.575	2.956
SPCC737.07C	SPCC737.07c	0.07591	-2.351	1.119701009	16.01	32.55	1.602	0.5118
SPAC2F7.02C	SPAC2F7.02c	0.5552	-2.351	0.255550543	12.75	26.75	3.556	3.139
SPCC1827.07C	SPCC1827.07c	0.3857	-2.35	0.413750361	15.49	31.61	2.084	2.155
SPBC1706.03	fzo1	0.3533	-2.344	0.451856363	12.92	27.04	2.395	1.874
SPBC15C4.04C	SPBC15C4.04c	0.04462	-2.342	1.350470434	18.98	37.78	1.209	0.6219
SPAC4H3.03C	SPAC4H3.03c	0.2773	-2.341	0.55705013	12.35	26.04	1.619	1.67
SPBC1773.06C	SPBC1773.06c	0.2707	-2.34	0.567511744	11.97	25.36	1.623	1.637
SPBC1921.04C	SPBC1921.04c	0.4407	-2.338	0.355856949	13.43	27.95	2.818	2.314
SPBC2G5.03	ctu1	0.6104	-2.336	0.214385475	12.53	26.35	2.97	3.893

SPAC4F10.18	nup37	0.7229	-2.334	0.140921775	10.8	23.28	7.926	4.407
SPBC1271.03C	SPBC1271.03c	0.3748	-2.332	0.426200418	13.21	27.54	1.935	2.103
SPAC1805.08	dlc1	0.3858	-2.332	0.413637777	15.39	31.41	1.181	2.261
SPAC26F1.10C	pyp1	0.632	-2.329	0.199282922	15.88	32.26	2.479	4.241
SPAC17H9.06C	SPAC17H9.06c	0.3151	-2.326	0.501551597	12.94	27.05	1.58	1.844
SPBC1734.15	rsc4	0.7162	-2.325	0.144965683	13.62	28.25	3.747	5.551
SPBC1778.10C	ppk21	0.01187	-2.324	1.925549281	13.68	28.36	0.7708	0.4822
SPCC11E10.05C	ynd1	0.3097	-2.322	0.509058795	12.42	26.12	1.474	1.83
SPBC12D12.09	rev7	0.3135	-2.317	0.503762455	11.38	24.28	1.746	1.799
SPBC23E6.08	sat1	0.4254	-2.315	0.371202514	1.47	6.707	4.299	0.9423
SPBC19C2.09	sre1	0.5111	-2.31	0.291494119	8.752	19.6	4.419	2.167
SPCC965.10	SPCC965.10	0.4793	-2.309	0.319392571	15.61	31.76	1.289	2.818
SPBC1709.09	rrf1	0.5192	-2.308	0.284665316	6.929	16.37	4.208	2.393
SPAC12B10.16C	mug157	0.1801	-2.307	0.744486287	11.02	23.62	1.378	1.261
SPAPJ691.03	SPAPJ691.03	0.005137	-2.304	2.289290434	13.26	27.59	0.621	0.4006
SPCC1494.08C	SPCC1494.08c	0.1474	-2.299	0.831502516	9.727	21.31	2.062	0.6256
SPAC1805.15C	pub2	0.09226	-2.296	1.03498655	11.65	24.71	0.9747	0.9469
SPAC7D4.02C	sfp47	0.295	-2.293	0.530177984	12.22	25.72	0.8401	1.795
SPAC3C7.10	pex13	0.3228	-2.291	0.491066474	12.07	25.46	1.679	1.833
SPBC4F6.06	kin1	0.3663	-2.29	0.436163081	0.7409	5.372	3.727	0.7409
SPCC1442.14C	SPCC1442.14c	0.1396	-2.287	0.855114582	12.87	26.87	0.9787	1.139
SPCC1259.07	rxt3	0.3367	-2.286	0.472756884	2.311	8.146	3.038	1.345
SPAC17A5.01	pex6	0.002801	-2.279	2.552686891	9.481	20.84	0.5596	0.3433
SPCC16A11.03C	SPCC16A11.03c	0.002954	-2.276	2.529589509	15.69	31.83	0.416	0.361
SPAC1A6.08C	mug125	0.6131	-2.273	0.212468684	8.538	19.16	6.787	1.609
SPBC3B9.06C	atg3	0.1633	-2.272	0.787013815	11.98	25.26	2.197	0.2508
SPCC622.12C	gdh1	0.6802	-2.263	0.167363372	8.788	19.58	7.128	3.326
SPBC16A3.14	SPBC16A3.14	0.2439	-2.258	0.6127882	13.95	28.72	0.938	1.543
SPBC21B10.10	rps402	0.3982	-2.256	0.399898744	11.28	23.98	3.581	1.398
SPBC146.12	coq6	0.7419	-2.255	0.129654629	18.11	36.08	5.018	5.769
SPBC8D2.03C	hhf2	0.6135	-2.254	0.212185433	10.34	22.32	6.227	2.294
SPAC18G6.05C	SPAC18G6.05c	0.3418	-2.246	0.466227942	7.408	17.11	3.359	0.9142
SPBC18H10.20C	SPBC18H10.20c	0.3323	-2.246	0.478469659	13.62	28.12	1.304	1.892
SPBCPT2R1.08C	tlh2	0.6839	-2.242	0.165007396	6.55	15.58	6.434	3.783
SPBC106.08C	mug2	0.3296	-2.236	0.482012797	13.3	27.53	1.723	1.807
SPAC17H9.12C	SPAC17H9.12c	0.277	-2.235	0.557520231	10.03	21.73	1.654	1.571
SPBC21B10.09	SPBC21B10.09	0.7147	-2.233	0.145876218	10.79	23.08	7.835	3.783
SPAC23D3.09	arp42	0.6249	-2.232	0.204189475	8.214	18.51	5.384	3.09
SPBC17A3.05C	SPBC17A3.05c	0.5028	-2.228	0.298604731	7.515	17.27	1.402	2.877
SPCC1620.13	SPCC1620.13	0.7207	-2.216	0.142245478	10.83	23.11	7.887	3.885
SPAC22A12.14C	SPAC22A12.14c	0.3139	-2.215	0.503208684	12.65	26.34	1.331	1.775
SPCC895.05	for3	0.4733	-2.214	0.324863496	4.454	11.82	4.191	1.625
SPAC1834.07	klp3	0.4568	-2.21	0.340273905	14.15	28.99	2.688	2.295
SPBC216.04C	SPBC216.04c	0.1688	-2.21	0.772627558	12.69	26.41	0.5885	1.227
SPAC22F3.02	atf31	0.2476	-2.204	0.60624936	13.09	27.11	1.577	1.431
SPBC106.13	SPBC106.13	0.5287	-2.199	0.27679069	11.88	24.94	5.406	0.7113
SPAC29A4.19C	cta5	0.6771	-2.197	0.169347186	13.68	28.14	3.903	4.415
SPAC5D6.10C	mug116	0.1317	-2.187	0.880414225	14.79	30.09	1.9	0.3435
SPAC22A12.07C	ogm1	0.641	-2.186	0.19314197	13.31	27.46	5.38	3.258
SPCC18B5.01C	bfr1	0.5493	-2.182	0.260190401	12.12	25.35	4.637	2.226
SPAC1834.05	alg9	0.5261	-2.182	0.278931698	13.49	27.76	1.462	2.989
SPBC530.11C	SPBC530.11c	0.496	-2.18	0.304518324	13.42	27.64	2.373	2.622
SPBC19G7.04	SPBC19G7.04	0.5309	-2.178	0.274987275	18.38	36.43	1.899	3.159
SPBC29A3.14C	trt1	0.6183	-2.175	0.208800754	6.946	16.16	5.753	2.55
SPAC637.03	SPAC637.03	0.4434	-2.173	0.353204311	14.98	30.4	1.946	2.329
SPBC3F6.01C	SPBC3F6.01c	0.4364	-2.172	0.360115258	12.9	26.71	1.877	2.296
SPBC3H7.06C	pof9	0.2778	-2.169	0.556267759	12.12	25.32	1.771	1.487
SPBC11C11.08	srp1	0.4319	-2.168	0.364616796	12.76	26.46	2.428	2.141
SPBC3B8.08	SPBC3B8.08	0.007809	-2.166	2.107404577	13.69	28.1	0.6641	0.4043
SPBC1709.14	SPBC1709.14	0.5201	-2.16	0.283913146	13.22	27.26	2.18	2.817
SPBC2D10.04	SPBC2D10.04	0.6266	-2.159	0.203009609	17.24	34.37	1.952	3.903
SPBC3B8.06	SPBC3B8.06	0.2447	-2.158	0.611366031	12.48	25.94	2.362	0.9858
SPCC895.07	alp14	0.4444	-2.153	0.35222595	14.4	29.34	2.214	2.257
SPAC4H3.06	SPAC4H3.06	0.02097	-2.146	1.67840157	13.62	27.93	0.8069	0.5153
SPBC21D10.07	cmc1	0.417	-2.144	0.379863945	14.82	30.06	1.775	2.165
SPBC685.04C	aps2	0.4862	-2.143	0.313185045	13.22	27.22	1.432	2.641
SPCC1840.05C	SPCC1840.05c	0.1612	-2.133	0.792634963	13.96	28.51	1.44	1.045
SPAC806.07	ndk1	0.4222	-2.127	0.374481771	14.59	29.63	2.622	1.961
SPAC5H10.12C	SPAC5H10.12c	0.329	-2.125	0.482804102	11.77	24.62	1.353	1.76

SPCC188.09C	SPCC188.09c	0.4908	-2.121	0.309095446	12.03	25.08	1.61	2.625
SPCC285.04	SPCC285.04	0.4495	-2.115	0.347270304	13.37	27.45	2.782	2.074
SPBC947.09	SPBC947.09	0.6102	-2.109	0.214527797	15.05	30.4	1.665	3.646
SPBC21B10.07	SPBC21B10.07	0.3242	-2.102	0.489186989	9.219	20.06	3.044	0.7641
SPAC31G5.21	SPAC31G5.21	0.365	-2.096	0.437707136	11.09	23.36	2.698	1.503
SPAC17A2.12	SPAC17A2.12	0.547	-2.093	0.262012674	16.38	32.74	3.115	2.73
SPBC530.05	SPBC530.05	0.5487	-2.092	0.26066504	10.84	22.92	5.235	1.224
SPCC1620.03	mug163	0.0003396	-2.089	3.469032318	11.66	24.37	0.3714	0.1929
SPAC12G12.09	SPAC12G12.09	0.4306	-2.088	0.365925975	12.63	26.08	1.17	2.254
SPAC607.08C	SPAC607.08c	0.3595	-2.085	0.444301105	13.76	28.08	2.073	1.719
SPAC3A11.04	SPAC3A11.04	0.6929	-2.083	0.159329439	9.189	19.98	6.989	3.091
SPBC106.11C	plg7	0.2999	-2.082	0.523023534	13.35	27.35	1.724	1.52
SPAC3H1.10	SPAC3H1.10	0.5442	-2.082	0.264241463	15.47	31.1	1.906	2.939
SPBC21C3.14C	SPBC21C3.14c	0.006744	-2.081	2.171082438	13.83	28.2	0.6704	0.3452
SPAC1687.19C	SPAC1687.19c	0.3007	-2.075	0.521866572	14.11	28.68	1.464	1.574
SPBC4C3.12	1-Sep	0.7285	-2.058	0.137570444	6.635	15.41	7.333	3.856
SPBC36B7.04	SPBC36B7.04	0.2937	-2.05	0.532096053	13.14	26.92	1.491	1.517
SPBC1215.01	shy1	0.2403	-2.05	0.619246229	9.732	20.88	2.362	0.7418
SPBC17A3.02	SPBC17A3.02	0.3391	-2.042	0.46967221	14.98	30.17	1.848	1.632
SPAC1F8.06	fta5	0.2972	-2.039	0.526951195	15.45	30.99	0.3826	1.617
SPAC6G10.02C	tea3	0.4646	-2.037	0.332920795	9.634	20.68	1.14	2.395
SPBPJ4664.02	SPBPJ4664.02	0.3736	-2.036	0.427593132	13.66	27.82	0.7712	1.934
SPAC1039.08	SPAC1039.08	0.4977	-2.027	0.303032359	4.528	11.62	3.898	1.731
SPAC1002.14	itt1	0.491	-2.026	0.308918508	12.02	24.9	2.511	2.329
SPBP8B7.04	mug45	0.4051	-2.022	0.392437757	13.05	26.71	0.8811	2.068
SPCC417.07C	mto1	0.6753	-2.02	0.17050325	11.15	23.34	3.115	4.123
SPACUNK4.11C	SPACUNK4.11c	0.5107	-2.011	0.291834142	12.53	25.78	2.728	2.394
SPBP4H10.08	qcr10	0.3882	-2.009	0.410944469	13.52	27.52	1.194	1.945
SPACUNK4.08	SPACUNK4.08	0.2821	-2.003	0.549596914	14.7	29.6	1.841	1.328
SPAC1D4.03C	aut12	0.3191	-2.001	0.496073196	9.91	21.11	2.456	1.21
SPBC36B7.06C	mug20	0.7649	-1.997	0.116395339	11.89	24.61	8.295	4.333
SPAC1093.06C	dhc1	0.4903	-1.992	0.309538107	12.44	25.58	2.036	2.383
SPCC1442.07C	SPCC1442.07c	0.03467	-1.991	1.460046158	11.17	23.32	0.6042	0.5903
SPAC1296.01C	SPAC1296.01c	0.005085	-1.985	2.293709043	14.03	28.38	0.5763	0.3273
SPAC17A5.18C	rec25	0.3744	-1.979	0.42666416	11.61	24.08	1.385	1.824
SPAC17H9.10C	ddb1	0.4817	-1.976	0.317223354	7.832	17.38	3.535	1.715
SPAC12B10.09	SPAC12B10.09	0.5853	-1.974	0.232621476	10.59	22.26	2.999	2.924
SPCC550.11	SPCC550.11	0.1009	-1.973	0.996108834	0	3.497	1.492	0
SPCC576.13	swc5	0.7445	-1.966	0.128135298	10.68	22.42	7.648	3.806
SPCC74.02C	SPCC74.02c	0.7392	-1.958	0.131238042	9.499	20.3	6.833	4.079
SPCC18B5.05C	SPCC18B5.05c	0.4412	-1.955	0.355364496	11.1	23.13	2.031	2.026
SPBC4C3.09	SPBC4C3.09	0.3515	-1.953	0.454074671	13.87	28.04	1.098	1.731
SPBC1348.01	SPBC1348.01	0.5509	-1.947	0.258927228	14.11	28.45	2.955	2.558
SPBC725.07	pex5	0.2608	-1.946	0.583692413	9.663	20.57	2.277	0.8565
SPAC19B12.07C	SPAC19B12.07c	0.2618	-1.938	0.582030358	12.88	26.26	1.52	1.281
SPAC3G9.07C	hos2	0.3398	-1.935	0.468776625	13.24	26.9	0.673	1.695
SPBC337.15C	coq7	0.4539	-1.934	0.343039817	3.414	9.477	3.648	1.183
SPAC1952.15C	rec24	0.4762	-1.927	0.322210609	12.9	26.28	3.699	1.4
SPAC1002.03C	gls2	0.4071	-1.926	0.390298898	13.09	26.61	1.015	1.965
SPCC962.05	ast1	0.11	-1.919	0.958607315	13.6	27.5	1.34	0.6883
SPAC1039.03	SPAC1039.03	0.0165	-1.916	1.782516056	12.56	25.65	0.4284	0.4575
SPCC1620.12C	SPCC1620.12c	0.6191	-1.913	0.208239196	12.07	24.79	2.457	3.277
SPAC6F6.04C	SPAC6F6.04c	0.5538	-1.911	0.256647049	15.03	30.02	0.992	2.846
SPAC17G8.05	med20	0.7565	-1.91	0.121191068	10.94	22.78	8.002	3.757
SPAC16C9.04C	mot2	0.5859	-1.903	0.232176502	12.4	25.35	4.896	1.756
SPCC1739.06C	SPCC1739.06c	0.4493	-1.889	0.347463581	5.685	13.42	1.02	2.14
SPCC576.12C	mhf2	0.3464	-1.883	0.460422117	12.8	26.03	0.7702	1.671
SPBC839.17C	fkh1	0.3369	-1.881	0.472498989	12.33	25.18	0.9836	1.614
SPBC25B2.10	SPBC25B2.10	0.5964	-1.876	0.224462365	6.665	15.13	4.434	2.233
SPAC6G9.14	SPAC6G9.14	0.6961	-1.872	0.157328366	10.32	21.61	4.648	3.709
SPCC1183.06	ung1	0.4392	-1.871	0.357337669	12.74	25.9	2.332	1.817
SPBC337.13C	gtr1	0.3735	-1.867	0.427709394	9.271	19.74	1.423	1.701
SPBC215.07C	SPBC215.07c	0.4149	-1.866	0.382056565	12.52	25.49	2.966	1.305
SPAC56E4.06C	ggt2	0.4126	-1.866	0.384470776	9.923	20.89	3.263	0.9367
SPAC1002.05C	jmj2	0.5453	-1.864	0.263364502	16.22	32.06	2.793	2.412
SPAPB24D3.07C	SPAPB24D3.07c	0.7437	-1.856	0.128602219	9.782	20.62	7.232	3.56
SPBC336.10C	tif512	0.05881	-1.85	1.230548821	13.81	27.75	1.103	0.4726
SPAC19G12.08	scs7	0.422	-1.846	0.374687549	12.69	25.77	2.45	1.632
SPAC637.11	rpm2	0.2599	-1.843	0.58519372	10.11	21.18	1.963	0.9801

SPAC17A5.14	exo2	0.2947	-1.841	0.530619864	12.6	25.59	1.062	1.409
SPBC582.08	SPBC582.08	0.5474	-1.84	0.261695207	15.65	30.99	1.495	2.645
SPCC737.03C	ima1	0.2193	-1.84	0.658961368	14.44	28.85	1.018	1.15
SPCC1739.04C	SPCC1739.04c	0.7171	-1.837	0.144420277	11.44	23.53	4.637	4.021
SPAC20H4.05C	SPAC20H4.05c	0.4839	-1.836	0.315244378	15.91	31.45	1.489	2.218
SPBPB2B2.07C	SPBPB2B2.07c	0.615	-1.83	0.211124884	12.02	24.55	5.409	1.449
SPBC26H8.03	cho2	0.7799	-1.827	0.10796108	11.66	23.9	8.037	4.302
SPBC2G2.02	syj1	0.5469	-1.823	0.262092077	14.77	29.41	2.277	2.488
SPBC3B8.10C	nem1	0.7133	-1.821	0.146727776	15.01	29.83	2.526	4.356
SPAC212.02	SPAC212.02	0.5145	-1.821	0.288614621	14.23	28.45	1.947	2.318
SPAC12G12.03	cip2	0.4053	-1.816	0.392223396	13.63	27.38	1.246	1.816
SPAPB1E7.05	gde1	0.3922	-1.814	0.406492411	14.81	29.46	1.582	1.699
SPAPJ695.01C	SPAPJ695.01c	0.7274	-1.811	0.138226703	8.713	18.65	6.769	3.147
SPBC215.05	gpd1	0.2746	-1.811	0.561299467	13.62	27.34	1.07	1.311
SPBC1604.08C	imp1	0.3675	-1.808	0.434742657	10.69	22.14	0.4712	1.701
SPBC21B10.02	SPBC21B10.02	0.5153	-1.804	0.287939858	14.52	28.92	1.687	2.343
SPBC119.16C	SPBC119.16c	0.2343	-1.804	0.630227711	13.73	27.52	1.284	1.126
SPAC26A3.02	myh1	0.6078	-1.797	0.216239304	12.38	25.12	5.011	1.659
SPAC186.03	SPAC186.03	0.5783	-1.795	0.237846808	14.45	28.79	1.209	2.84
SPBP16F5.03C	tra1	0.004899	-1.794	2.309892561	10.97	22.62	0.5288	0.286
SPCC1393.03	rps1501	0.4774	-1.791	0.321117585	14.31	28.53	0.6657	2.196
SPBC1683.06C	SPBC1683.06c	0.563	-1.783	0.249491605	13.99	27.95	1.142	2.708
SPBC8D2.04	hht2	0.7717	-1.782	0.1125515	11.41	23.37	7.445	4.102
SPCC594.07C	bqt3	0.5989	-1.782	0.222645687	12.47	25.26	3.459	2.534
SPAC3H1.13	ppk13	0.197	-1.78	0.705533774	12.25	24.86	0.7156	1.068
SPAC1B3.02C	SPAC1B3.02c	0.5659	-1.779	0.247260306	15.41	30.47	1.038	2.731
SPAC1610.02C	SPAC1610.02c	0.5677	-1.775	0.245881106	7.635	16.68	1.991	2.628
SPAC1705.02	SPAC1705.02	0.4681	-1.774	0.329661359	10.86	22.39	1.503	2.047
SPBC8D2.17	SPBC8D2.17	0.7725	-1.771	0.112101512	10.99	22.61	7.741	3.893
SPBC1685.09	rps29	0.3932	-1.769	0.405386491	7.304	16.08	0.6969	1.761
SPAC328.04	SPAC328.04	0.7543	-1.765	0.122455892	15.31	30.26	3.382	4.896
SPAC23H3.06	apl6	0.3422	-1.763	0.465719995	10.97	22.57	2.761	0.2858
SPCC16C4.09	sts5	0.2968	-1.76	0.527536103	12.73	25.68	1.001	1.356
SPAC11E3.01C	swr1	0.1994	-1.759	0.700274846	15.57	30.71	1.162	1
SPAC22A12.06C	SPAC22A12.06c	0.6501	-1.758	0.187019834	9.011	19.08	4.239	2.795
SPBC27B12.05	SPBC27B12.05	0.4313	-1.751	0.365220542	15.84	31.18	2.651	1.439
SPAC11D3.17	SPAC11D3.17	0.511	-1.748	0.2915791	12.2	24.72	2.518	2.037
SPAP11E10.01	SPAP11E10.01	0.5752	-1.748	0.240181123	14.68	29.12	2.443	2.552
SPAC1952.08C	SPAC1952.08c	0.4923	-1.746	0.307770164	13.69	27.36	1.439	2.156
SPAC8C9.12C	SPAC8C9.12c	0.785	-1.735	0.105130343	12.76	25.7	7.391	4.426
SPAC1A6.03C	SPAC1A6.03c	0.005715	-1.732	2.242983765	14.39	28.56	0.5608	0.2464
SPAC14C4.04	B22918-2	0.5334	-1.728	0.272946989	12.51	25.23	1.645	2.357
SPAC13G6.13	SPAC13G6.13	0.4255	-1.726	0.371100436	13.77	27.47	1.176	1.818
SPBC1685.06	cid11	0.5132	-1.719	0.289713352	15.72	30.9	1.852	2.177
SPAC23A1.14C	SPAC23A1.14c	0.2036	-1.716	0.691222226	12.6	25.38	0.9977	1.016
SPAC1565.03	SPAC1565.03	0.2983	-1.714	0.525346747	13.6	27.14	0.4969	1.358
SPBC29A10.09C	SPBC29A10.09c	0.4472	-1.711	0.349498205	13.96	27.78	0.7751	1.939
SPAC12G12.16C	SPAC12G12.16c	0.5838	-1.709	0.233735909	14.92	29.47	2.787	2.464
SPAPB21F2.03	SPAPB21F2.03	0.7201	-1.703	0.142607189	7.941	17.09	6.106	2.94
SPBC31F10.03	SPBC31F10.03	0.2951	-1.702	0.530030791	14.19	28.17	1.086	1.29
SPBC27B12.11C	SPBC27B12.11c	0.1948	-1.698	0.710411047	7.011	15.43	1.784	0.4292
SPBC24C6.05	sec28	0.4376	-1.694	0.358922687	10.8	22.14	2.228	1.593
SPAC922.03	SPAC922.03	0.3635	-1.691	0.439495585	13.8	27.46	1.499	1.458
SPAC26H5.04	SPAC26H5.04	0.7564	-1.689	0.12124848	16.38	32.01	2.945	4.776
SPAC227.14	SPAC227.14	0.06457	-1.686	1.189969214	13.58	27.05	0.9723	0.5037
SPCP31B10.07	eft202	0.2864	-1.684	0.543026986	12.85	25.75	1.275	1.21
SPBC18H10.04C	sce3	0.6655	-1.679	0.17685194	13.44	26.8	4.198	2.829
SPCP25A2.02C	rhp26	0.374	-1.676	0.427128398	13.43	26.77	1.423	1.501
SPAP8A3.14C	SPAP8A3.14c	0.4567	-1.672	0.340368988	13.71	27.25	1.498	1.857
SPAC4C5.03	SPAC4C5.03	0.07487	-1.67	1.125692167	11.63	23.58	0.8677	0.5946
SPBC1711.09C	SPBC1711.09c	0.3969	-1.669	0.401318901	13.95	27.68	2.234	1.326
SPBC30B4.01C	wsc1	0.8135	-1.66	0.089642443	12.42	24.94	8.62	4.656
SPAC9G1.07	SPAC9G1.07	0.5403	-1.659	0.267365032	13.66	27.14	1.903	2.254
SPAC4H3.07C	SPAC4H3.07c	0.6328	-1.655	0.19873353	10.84	22.14	4.556	2.038
SPBPB10D8.07C	SPBPB10D8.07c	0.6906	-1.654	0.160773426	18.75	36.16	2.24	3.629
SPBC1718.02	hop1	0.5156	-1.649	0.287687091	14.82	29.19	0.8328	2.218
SPAC977.11	SPAC977.11	0.573	-1.649	0.241845378	13.51	26.86	1.98	2.458
SPAC11D3.05	mfs2	0.5828	-1.644	0.234480457	12.45	24.97	1.842	2.549
SPBC2D10.13	est1	0.7567	-1.643	0.121076266	10.94	22.3	5.692	3.908

SPAC1786.04	SPAC1786.04	0.5747	-1.64	0.240558803	15.92	31.12	2.132	2.427
SPBC23E6.09	ssn6	0.5266	-1.638	0.278519145	14.83	29.19	1.266	2.229
SPCC970.10C	brl2	0.6793	-1.637	0.167938385	6.523	14.46	5.074	2.447
SPCC70.09C	mug9	0.118	-1.636	0.928117993	14.27	28.19	0.5646	0.761
SPAC11E3.10	SPAC11E3.10	0.4209	-1.634	0.375821074	12.19	24.49	1.214	1.689
SPAC13G6.01C	rad8	0.5191	-1.631	0.284748971	12.84	25.64	2.157	2.011
SPAC10F6.07C	mug94	0.5329	-1.629	0.27335428	12.9	25.75	1.85	2.167
SPAC140.01	sdh2	0.7696	-1.629	0.113734941	7.501	16.18	7.379	3.279
SPAC664.03	SPAC664.03	0.4361	-1.628	0.360413913	5.273	12.23	0.9869	1.774
SPAC17A5.08	SPAC17A5.08	0.7488	-1.627	0.125634164	9.917	20.46	6.986	2.785
SPCC24B10.14C	xlf1	0.5806	-1.627	0.236122969	15.69	30.69	2.132	2.449
SPAC1420.03	rpn501	0.5675	-1.626	0.246034134	14.72	28.96	1.627	2.436
SPBC29A10.16C	SPBC29A10.16c	0.7835	-1.625	0.105960999	10.74	21.92	7.407	3.807
SPAC5D6.06C	alg14	0.6345	-1.625	0.197568374	10.37	21.25	3.998	2.466
SPAC186.08C	SPAC186.08c	0.2255	-1.625	0.646853454	13.75	27.25	1.312	0.9387
SPCC191.05C	SPCC191.05c	0.808	-1.622	0.092588639	13.07	26.03	6.642	5.141
SPBC1289.06C	ppr8	0.01783	-1.621	1.748848657	8.757	18.39	0.5306	0.3882
SPAC926.09C	fas1	0.5171	-1.618	0.286425462	14.05	27.77	0.5444	2.2
SPBC83.17	SPBC83.17	0.4006	-1.609	0.397289055	13.77	27.25	1.601	1.498
SPAC1639.01C	SPAC1639.01c	0.6714	-1.608	0.173018663	11.2	22.7	4.239	2.834
SPCC364.04C	SPCC364.04c	0.6145	-1.605	0.211478113	18.39	35.44	0.9084	2.839
SPBC32F12.12C	SPBC32F12.12c	0.6621	-1.603	0.179076412	17.35	33.59	1.928	3.201
SPBC3B8.05	SPBC3B8.05	0.5532	-1.602	0.257117829	15.15	29.69	0.7694	2.385
SPAC16C9.01C	SPAC16C9.01c	0.5472	-1.601	0.261853911	13.69	27.1	2.022	2.183
SPAC3H5.04	aar2	0.1551	-1.596	0.809388202	13.98	27.6	0.938	0.8012
SPAC23A1.04C	mnl1	0.3707	-1.586	0.430977414	14.48	28.47	2.179	1.081
SPCC285.05	SPCC285.05	0.5242	-1.583	0.280502983	12.72	25.34	0.8349	2.175
SPCC1795.03	gms1	0.3583	-1.583	0.445753192	13.37	26.49	1.337	1.36
SPBC29A3.05	vps71	0.5043	-1.583	0.297311032	13.61	26.92	1.816	1.93
SPCC613.12C	raf1	0.5759	-1.582	0.239652921	10.9	22.12	4.339	0.8149
SPAC167.04	pam17	0.6914	-1.581	0.160270625	14.64	28.74	4.515	2.808
SPAC823.15	ppa1	0.3696	-1.579	0.432268037	13.24	26.26	1.406	1.384
SPCC24B10.03	SPCC24B10.03	0.6196	-1.576	0.207888591	15.6	30.43	1.54	2.773
SPBC428.17C	wpl1	0.6437	-1.57	0.191316491	15.11	29.56	2.478	2.84
SPAC694.06C	mrc1	0.5631	-1.565	0.249414473	12.9	25.63	2.98	1.924
SPBC1709.13C	set10	0.7679	-1.558	0.114695332	11.09	22.41	7.495	2.669
SPAPB17E12.12C	SPAPB17E12.12c	0.5114	-1.554	0.291239276	14.1	27.75	1.379	2.007
SPAC343.04C	SPAC343.04c	0.3648	-1.55	0.43794517	13.74	27.1	1.282	1.361
SPAC19G12.15C	tpp1	0.59	-1.549	0.229147988	2.821	7.745	3.763	1.692
SPAC12G12.07C	SPAC12G12.07c	0.5848	-1.548	0.232992636	15.96	31.02	3.316	1.923
SPCC285.15C	rps2802	0.5983	-1.547	0.223080997	12.6	25.07	4.484	0.9288
SPBC23G7.04C	nif1	0.6059	-1.544	0.217599048	15.05	29.41	1.155	2.64
SPAC1D4.02C	SPAC1D4.02c	0.514	-1.54	0.289036881	14.38	28.2	2.949	1.467
SPAPB1E7.08C	SPAPB1E7.08c	0.5532	-1.538	0.257117829	12.72	25.26	1.83	2.16
SPBC29A3.13	pdp1	0.4734	-1.537	0.324771746	12.29	24.5	1.625	1.741
SPAC1687.14C	SPAC1687.14c	0.448	-1.537	0.348721986	14.32	28.09	1.715	1.595
SPAPB1A11.01	mfc1	0.4799	-1.537	0.31884925	11.41	22.95	1.864	1.717
SPAC824.09C	SPAC824.09c	0.7826	-1.533	0.106460156	10.15	20.71	6.298	3.944
SPCC16C4.04	SPCC16C4.04	0.277	-1.52	0.557520231	15.04	29.35	1.332	1.012
SPAC19B12.08	atg4	0.5024	-1.519	0.298950369	10.38	21.08	2.186	1.719
SPCC1322.09	SPCC1322.09	0.7729	-1.517	0.111876693	9.268	19.11	6.774	3.253
SPBC11B10.05C	rsp1	0.0175	-1.515	1.756961951	13.95	27.42	0.6019	0.3189
SPCP20C8.02C	SPCP20C8.02c	0.5237	-1.515	0.280917426	14.87	29.05	1.817	1.942
SPAC1002.01	SPAC1002.01	0.555	-1.515	0.255707017	14.98	29.23	0.7794	2.263
SPBC354.07C	SPBC354.07c	0.5799	-1.509	0.236646891	13.18	26.03	2.806	2.027
SPAC11E3.12	SPAC11E3.12	0.5682	-1.509	0.245498771	13.21	26.09	2.467	2.054
SPAC57A10.03	cyp1	0.5034	-1.509	0.298086789	14.68	28.69	2.016	1.762
SPBC3E7.10	fma1	0.7681	-1.508	0.114582235	10.81	21.84	6.565	3.181
SPBC1685.05	SPBC1685.05	0.02519	-1.507	1.598771833	13.75	27.03	0.5944	0.3792
SPAC18B11.08C	SPAC18B11.08c	0.5712	-1.499	0.243211801	14.92	29.1	1.749	2.231
SPBC20F10.06	mad2	0.5813	-1.496	0.235599677	15.91	30.85	0.6272	2.413
SPBC25H2.09	SPBC25H2.09	0.538	-1.495	0.269217724	14.98	29.2	2.225	1.891
SPAC23H3.15C	SPAC23H3.15c	0.7277	-1.493	0.138047625	11.15	22.41	6.475	1.645
SPCC1739.05	set5	0.6415	-1.482	0.192803339	5.195	11.83	4.069	1.962
SPBC30D10.14	SPBC30D10.14	0.5877	-1.481	0.230844309	13.5	26.55	1.403	2.368
SPBC215.06C	SPBC215.06c	0.3623	-1.48	0.440931666	13.17	25.96	1.285	1.277
SPCC364.02C	bis1	0.5099	-1.478	0.292514988	13.91	27.26	1.328	1.898
SPAC1002.18	urg3	0.4708	-1.477	0.327163546	13.75	26.99	2.174	1.471
SPAC3F10.15C	spo12	0.7503	-1.474	0.124765054	10.47	21.17	1.246	4.19

SPBC776.15C	kgd2	0.6718	-1.469	0.17276	7.644	16.15	2.833	2.838
SPBC1198.07C	SPBC1198.07c	0.7175	-1.469	0.144178095	14.68	28.61	3.605	3.255
SPCC162.06C	SPCC162.06c	0.7483	-1.469	0.125924255	15.04	29.26	3.365	3.854
SPAC5D6.12	SPAC5D6.12	0.5771	-1.468	0.238748926	15.78	30.56	1.718	2.225
SPAC11D3.13	SPAC11D3.13	0.6446	-1.459	0.190709699	9.845	20.03	4.636	1.379
SPBC1703.07	SPBC1703.07	0.5263	-1.456	0.27876663	14.44	28.17	2.111	1.785
SPAC2F3.11	SPAC2F3.11	0.5875	-1.45	0.230992129	15.51	30.06	1.708	2.268
SPAC1805.14	SPAC1805.14	0.6872	-1.449	0.162916849	15.31	29.7	2.519	3.043
SPBPB21E7.01C	eno102	0.5811	-1.449	0.235749125	15.74	30.46	2.278	2.094
SPAC14C4.08	mug5	0.5525	-1.447	0.257667718	13.46	26.41	1.929	1.983
SPAC22G7.04	ubp13	0.737	-1.443	0.132532512	17.36	33.33	3.252	3.596
SPAC22E12.11C	set3	0.2495	-1.443	0.60292945	9.889	20.08	1.163	0.9079
SPBC839.15C	ef1a-c	0.6996	-1.438	0.155150199	6.475	14.02	4.828	2.271
SPAC2G11.07C	ptc3	0.3065	-1.438	0.513569521	14.61	28.44	1.597	0.9186
SPAC4D7.07C	SPAC4D7.07c	0.6462	-1.431	0.189633046	14.09	27.51	2.783	2.481
SPAC6G9.10C	sen1	0.7967	-1.431	0.098705183	11.46	22.85	3.258	4.847
SPBC21C3.11	ubx4	0.6359	-1.428	0.196611175	14.31	27.89	2.125	2.537
SPAC6G10.06	SPAC6G10.06	0.7056	-1.426	0.151441428	15.29	29.63	2.839	3.156
SPAC821.13C	SPAC821.13c	0.8056	-1.426	0.093880542	10.55	21.23	7.399	3.644
SPCC16C4.14C	sfc4	0.3818	-1.425	0.418164076	12.69	25.02	1.343	1.276
SPAC57A7.04C	pabp	0.6029	-1.424	0.219754716	13.15	25.82	1.907	2.298
SPBC530.14C	dsk1	0.6202	-1.423	0.207468238	12.92	25.42	1.806	2.451
SPAC1952.12C	csn71	0.5445	-1.422	0.264002116	14.94	28.99	2.602	1.656
SPCC569.02C	SPCC569.02c	0.7765	-1.422	0.10985854	8.98	18.43	6.432	3.114
SPAC29E6.09	SPAC29E6.09	0.6293	-1.42	0.201142268	14.35	27.94	1.417	2.575
SPAC19D5.07	uga1	0.5658	-1.416	0.247337057	13.52	26.47	1.481	2.102
SPAC32A11.01	mug8	0.7945	-1.416	0.099906098	11.55	22.99	7.209	3.219
SPAC23H4.09	cdb4	0.6498	-1.416	0.187220293	15.6	30.16	1.608	2.721
SPBC26H8.01	thi2	0.5706	-1.413	0.243668233	11.96	23.71	1.881	2.053
SPAC13A11.06	SPAC13A11.06	0.545	-1.411	0.263603498	13.98	27.28	1.515	1.963
SPBC2G2.07C	mug178	0.6352	-1.409	0.197089511	6.754	14.47	0.7193	2.66
SPBC1289.15	SPBC1289.15	0.5535	-1.409	0.256882375	12.09	23.92	3.273	1.245
SPBC1198.06C	SPBC1198.06c	0.7368	-1.394	0.132650383	7.186	15.21	5.387	2.698
SPBC17A3.10	pas4	0.6963	-1.393	0.157203605	7.014	14.9	5.404	1.328
SPAC11D3.01C	SPAC11D3.01c	0.6612	-1.391	0.179667155	14.97	28.99	2.637	2.583
SPAC4G9.15	SPAC4G9.15	0.3746	-1.39	0.426432227	4.156	9.828	2.351	0.258
SPAC25H1.03	mug66	0.7275	-1.388	0.138167002	13.95	27.19	2.2	3.481
SPAC56F8.02	SPAC56F8.02	0.4571	-1.386	0.339988779	11.16	22.23	0.9965	1.576
SPAC4D7.06C	SPAC4D7.06c	0.1275	-1.383	0.894489815	11.36	22.58	0.8453	0.6109
SPBC146.11C	mug97	0.5792	-1.379	0.237171447	13.72	26.77	1.689	2.089
SPBC691.01	pfa5	0.658	-1.375	0.181774106	14.83	28.72	1.827	2.681
SPAC3A12.06C	SPAC3A12.06c	0.5943	-1.371	0.22599427	14.87	28.78	2.032	2.107
SPCC613.03	SPCC613.03	0.546	-1.369	0.262807357	13.1	25.64	3.082	1.221
SPAC29E6.05C	SPAC29E6.05c	0.6433	-1.368	0.191586449	16.62	31.89	2.15	2.472
SPAC1952.03	otu2	0.7486	-1.367	0.125750177	7.578	15.85	5.439	2.675
SPCC297.04C	set7	0.6153	-1.365	0.210913085	15.73	30.3	2.118	2.236
SPBC1773.03C	SPBC1773.03c	0.5745	-1.362	0.240709967	15.42	29.74	1.479	2.067
SPAC1071.09C	SPAC1071.09c	0.629	-1.359	0.201349355	14.37	27.88	0.8322	2.509
SPCC297.06C	SPCC297.06c	0.6666	-1.359	0.176134691	16.45	31.56	2.352	2.633
SPBC557.04	ppk29	0.8099	-1.355	0.091568601	15.5	29.86	3.922	4.808
SPAC26F1.07	SPAC26F1.07	0.7293	-1.349	0.137093786	8.506	17.46	4.228	2.848
SPAC19A8.01C	sec73	0.3946	-1.345	0.403842919	11.81	23.31	0.5334	1.343
SPBC32C12.03C	ppk25	0.4654	-1.341	0.332173621	11.71	23.12	2.172	1.207
SPAC19E9.01C	nup40	0.5886	-1.34	0.230179742	13.66	26.58	0.6723	2.201
SPAC1A6.05C	SPAC1A6.05c	0.6552	-1.34	0.183626111	15.26	29.42	1.943	2.56
SPAC589.02C	med13	0.8113	-1.338	0.090818524	10.59	21.14	7.108	3.557
SPAC3A11.14C	pkl1	0.5103	-1.338	0.292174432	15.58	29.99	1.302	1.705
SPBC2F12.04	rpl1701	0.6335	-1.334	0.198253381	15.8	30.36	2.638	2.18
SPBC25H2.10C	SPBC25H2.10c	0.5721	-1.334	0.242528052	12.22	24.02	2.414	1.759
SPAPYUG7.02C	sin1	0.4827	-1.331	0.316322701	4.138	9.693	2.511	1.18
SPCC24B10.12	cgi121	0.6061	-1.331	0.217455716	13.06	25.51	2.442	2.001
SPAC25A8.03C	SPAC25A8.03c	0.345	-1.331	0.462180905	13.71	26.65	1.375	1.03
SPCC1682.08C	SPCC1682.08c	0.7852	-1.33	0.105019709	8.666	17.71	6.451	2.899
SPAC926.07C	dlc2	0.63	-1.327	0.200659451	17.49	33.35	2.782	2.078
SPAC644.14C	rhp51	0.8004	-1.323	0.09669292	14.31	27.71	1.685	4.729
SPAC644.08	SPAC644.08	0.4662	-1.321	0.331427731	13.95	27.07	1.664	1.396
SPAC1A6.07	SPAC1A6.07	0.5521	-1.314	0.257982253	13.41	26.1	1.899	1.762
SPCC126.12	SPCC126.12	0.1621	-1.314	0.790216985	13.82	26.81	1.07	0.5606
SPCC1322.05C	SPCC1322.05c	0.4104	-1.312	0.386792648	13.47	26.2	1.194	1.282

SPCC364.06	nap1	0.2379	-1.312	0.623605558	12.68	24.79	0.9261	0.8295
SPAPJ760.02C	abp1	0.06784	-1.309	1.168514161	12.03	23.63	0.4484	0.4858
SPAC3H8.05C	mms1	0.7458	-1.309	0.127377621	7.287	15.23	5.429	2.317
SPCC16C4.17	mug123	0.7896	-1.3	0.10259286	8.478	17.33	6.55	2.812
SPAC3A11.10C	SPAC3A11.10c	0.6505	-1.291	0.186752699	16.66	31.81	1.385	2.498
SPAC212.01C	SPAC212.01c	0.5854	-1.29	0.232547282	14.06	27.21	1.348	2.031
SPCC188.12	spn6	0.6293	-1.29	0.201142268	15.97	30.6	2.414	2.114
SPAC3F10.09	SPAC3F10.09	0.6529	-1.283	0.185153331	4.66	10.53	3.521	1.844
SPAC1A6.06C	meu31	0.8156	-1.279	0.088522783	9.858	19.74	6.916	3.503
SPBC1685.11	rlp1	0.6776	-1.271	0.169026603	16.48	31.47	0.9218	2.739
SPAC1610.04	mug99	0.8549	-1.266	0.068084683	12.86	25.03	8.654	4.482
SPBC16E9.09C	SPBC16E9.09c	0.853	-1.266	0.069050969	12.37	24.17	8.102	4.68
SPAC29B12.10C	pgt1	0.8273	-1.262	0.082336976	10.62	21.05	7.144	3.799
SPAC23H3.13C	gpa2	0.6834	-1.262	0.165325026	5.101	11.28	4.078	1.827
SPAC1782.02C	SPAC1782.02c	0.6245	-1.255	0.204467557	13.35	25.88	3.634	1.263
SPAC25H1.04	mug105	0.7209	-1.252	0.142124974	12.98	25.23	5.104	1.613
SPBC691.03C	apl3	0.8267	-1.252	0.082652062	14.12	27.23	3.701	4.938
SPAC27D7.05C	apc14	0.6138	-1.25	0.211973116	16.86	32.1	1.091	2.176
SPAC6B12.03C	SPAC6B12.03c	0.5772	-1.247	0.238673678	10.57	20.94	3.444	0.608
SPCC191.10	SPCC191.10	0.6679	-1.245	0.175288557	16.07	30.68	1.592	2.525
SPBC651.11C	apm3	0.3075	-1.244	0.51215488	12.74	24.78	0.48	1.004
SPBC20F10.05	nrl1	0.6261	-1.243	0.203356296	15.02	28.83	2.157	2.06
SPAC3C7.02C	SPAC3C7.02c	0.6979	-1.237	0.156206802	16.39	31.24	2.553	2.624
SPCC1919.11	mug137	0.5519	-1.233	0.258139606	13.99	26.98	0.7907	1.815
SPBC577.12	mug71	0.722	-1.232	0.141462802	12.82	24.9	3.156	2.75
SPAC13D6.02C	byr3	0.4011	-1.231	0.396747338	9.694	19.36	1.187	1.157
SPBC13G1.13	tfb2	0.711	-1.23	0.148130399	14.43	27.75	2.9	2.673
SPAC1687.23C	SPAC1687.23c	0.6266	-1.218	0.203009609	15.48	29.59	1.343	2.173
SPAC16C9.07	pom2	0.7191	-1.217	0.143210711	14.37	27.63	3.827	2.395
SPAC8C9.06C	ppr4	0.5191	-1.215	0.284748971	14.85	28.47	1.633	1.491
SPBC25B2.11	pof2	0.596	-1.211	0.22475374	14.62	28.05	0.8351	2.016
SPBC1105.08	SPBC1105.08	0.526	-1.21	0.279014256	12.05	23.5	1.481	1.554
SPAC8E11.02C	rad24	0.756	-1.206	0.121478204	6.875	14.32	4.776	2.546
SPBC106.12C	SPBC106.12c	0.7386	-1.203	0.131590697	13.43	25.93	4.973	1.962
SPAC20G4.04C	hus1	0.5623	-1.2	0.250031916	11.66	22.8	2.05	1.567
SPBC543.10	get1	0.7234	-1.198	0.140621496	13.93	26.8	2.766	2.78
SPAC1002.17C	urg2	0.6714	-1.192	0.173018663	13.07	25.27	1.585	2.439
SPBC16G5.15C	fkh2	0.6393	-1.189	0.194295296	9.621	19.16	1.242	2.22
SPAC22F3.08C	rok1	0.7267	-1.188	0.13864484	9.219	18.44	4.407	2.075
SPBC6B1.09C	nbs1	0.6255	-1.188	0.203772686	15.8	30.1	0.9918	2.147
SPAC23H4.02	ppk9	0.4861	-1.187	0.313274379	13.09	25.31	1.389	1.364
SPCC4B3.12	set9	0.2026	-1.185	0.693360559	13.99	26.9	1.259	0.3462
SPBC1703.11	SPBC1703.11	0.6853	-1.185	0.164119268	14.47	27.74	2.752	2.288
SPAC2G11.10C	SPAC2G11.10c	0.6953	-1.182	0.157827771	12.97	25.09	2.621	2.432
SPAC24B11.08C	SPAC24B11.08c	0.6279	-1.182	0.202109517	15.29	29.2	1.686	2.056
SPBC11G11.01	fis1	0.7819	-1.182	0.106848787	9.664	19.22	5.517	2.633
SPCC285.10C	SPCC285.10c	0.4353	-1.179	0.361211333	12.43	24.12	2.197	0.5864
SPBC1778.05C	SPBC1778.05c	0.5318	-1.177	0.274251667	2.27	6.107	2.109	1.314
SPCC1235.02	bio2	0.5929	-1.176	0.22701855	14.06	27	2.185	1.668
SPBC30B4.04C	sol1	0.4937	-1.173	0.306536873	14.89	28.47	1.553	1.331
SPBC1289.13C	SPBC1289.13c	0.8036	-1.172	0.094960072	9.162	18.31	5.697	3.16
SPBC11C11.01	SPBC11C11.01	0.301	-1.169	0.521433504	13.63	26.22	1.114	0.8118
SPCC830.08C	yop1	0.676	-1.168	0.170053304	14	26.89	2.869	2.101
SPBC29A10.12	SPBC29A10.12	0.7084	-1.167	0.149721447	14.18	27.19	2.792	2.493
SPAC750.08C	SPAC750.08c	0.7231	-1.167	0.140801638	10.93	21.43	2.272	2.799
SPBC23E6.01C	SPBC23E6.01c	0.6781	-1.16	0.168706256	10.94	21.43	3.82	1.533
SPAC1F7.10	SPAC1F7.10	0.4857	-1.158	0.313631897	13.91	26.7	1.357	1.329
SPAC4H3.02C	swc3	0.8749	-1.156	0.058041583	13.43	25.86	8.65	5.069
SPBC4F6.12	pxl1	0.5724	-1.155	0.242300375	13.7	26.32	1.427	1.711
SPAC3A11.11C	SPAC3A11.11c	0.8053	-1.154	0.094042301	10.79	21.16	5.945	2.963
SPCC1840.11	csl4	0.7192	-1.147	0.143150321	9.348	18.6	4.551	1.578
SPAC13F5.03C	gld1	0.8442	-1.147	0.073554652	10.12	19.97	7.427	3.694
SPBP26C9.02C	car1	0.5853	-1.142	0.232621476	15.13	28.83	1.667	1.708
SPBC530.06C	SPBC530.06c	0.7009	-1.14	0.15434394	5.075	11.01	3.784	1.849
SPBC660.06	SPBC660.06	0.7316	-1.14	0.135726303	12.01	23.3	3.203	2.585
SPCC18B5.09C	SPCC18B5.09c	0.7325	-1.137	0.135192371	13.2	25.4	1.835	2.909
SPBC29B5.01	atf1	0.2576	-1.131	0.589054141	8.057	16.28	1.388	0.3683
SPBC1683.10C	pcl1	0.6912	-1.129	0.160396271	10.41	20.44	3.329	1.951
SPBC1A4.02C	leu1	0.6791	-1.127	0.16806627	16.27	30.82	2.148	2.25

SPAC15E1.06	vps29	0.5639	-1.123	0.248797905	13.69	26.24	1.963	1.459
SPBC3D6.04C	mad1	0.7369	-1.122	0.132591443	6.019	12.65	4.279	2.076
SPBC83.02C	rpl4302	0.8524	-1.121	0.069356559	8.587	17.21	8.399	3.235
SPBC428.15	SPBC428.15	0.6148	-1.121	0.211266141	14.13	27.03	1.455	1.89
SPBC16D10.07C	sir2	0.836	-1.121	0.077793723	19.49	36.52	3.16	4.943
SPBC25H2.08C	mrs2	0.1005	-1.118	0.997833938	9.71	19.19	0.4623	0.48
SPAC27E2.01	SPAC27E2.01	0.5446	-1.117	0.263922363	14.38	27.47	1.211	1.55
SPBC691.05C	SPBC691.05c	0.8109	-1.115	0.0910327	14.88	28.34	1.283	4.226
SPAC17G8.06C	SPAC17G8.06c	0.6937	-1.114	0.158828306	5.583	11.87	2.874	2.14
SPBC16A3.18	cip1	0.8303	-1.113	0.080764962	19.74	36.95	2.761	4.58
SPCC191.03C	SPCC191.03c	0.6818	-1.111	0.166343003	14.37	27.44	2.248	2.209
SPBC13G1.02	SPBC13G1.02	0.5891	-1.111	0.229810977	13.55	25.99	2.325	1.438
SPAPJ698.02C	rps002	0.391	-1.11	0.407823243	3.93E-18	1.967	1.967	3.93E-18
SPCC1494.07	SPCC1494.07	0.5993	-1.106	0.222355722	13.81	26.44	1.319	1.793
SPAC12G12.10	wdr21	0.7342	-1.105	0.13418562	13.24	25.43	4.409	1.841
SPAPYUG7.04C	rpb9	0.09331	-1.102	1.030071811	11.37	22.1	0.4152	0.4615
SPBC1348.14C	ght7	0.8146	-1.098	0.089055594	12.9	24.81	1.74	4.214
SPBC21B10.05C	pop3	0.76	-1.092	0.119186408	14.29	27.25	3.374	2.81
SPAC2C4.05	SPAC2C4.05	0.5531	-1.091	0.257196342	13.88	26.53	2.022	1.307
SPAC26A3.07C	rpl1101	0.7162	-1.088	0.144965683	11.08	21.56	1.327	2.653
SPBC21H7.06C	SPBC21H7.06c	0.699	-1.086	0.155522824	15.96	30.21	2.624	2.208
SPCC126.09	SPCC126.09	0.72	-1.084	0.142667504	15.05	28.59	1.767	2.628
SPCC338.11C	rrg1	0.6873	-1.083	0.162853656	12.59	24.23	1.771	2.297
SPAC1399.01C	SPAC1399.01c	0.6047	-1.081	0.218460031	15.13	28.73	1.265	1.788
SPBC13G1.04C	SPBC13G1.04c	0.655	-1.08	0.1837587	15.18	28.82	2.99	1.56
SPCC126.07C	SPCC126.07c	0.7746	-1.08	0.110922507	14.79	28.12	2.718	3.194
SPCC1020.09	gnr1	0.4992	-1.079	0.301725423	13.17	25.25	0.9456	1.349
SPAC343.16	lys2	0.6843	-1.079	0.16475346	5.263	11.24	3.217	1.756
SPAC5D6.08C	mes1	0.6828	-1.078	0.165706488	16.22	30.65	1.878	2.223
SPBC2D10.15C	pth1	0.7284	-1.076	0.137630063	17.48	32.89	1.54	2.73
SPAP8A3.07C	SPAP8A3.07c	0.8403	-1.073	0.075565636	10.06	19.72	6.957	3.244
SPCC1840.06	atp5	0.7446	-1.069	0.128076968	15.14	28.72	1.856	2.865
SPAC1F7.12	yak3	0.8477	-1.063	0.071757817	13.62	26.02	4.018	4.692
SPBC354.15	fap1	0.7401	-1.059	0.130709596	16.57	31.24	1.663	2.808
SPBC1773.08C	omh4	0.7303	-1.059	0.136498699	14.59	27.73	2.396	2.552
SPCC1259.14C	meu27	0.5634	-1.052	0.249183157	12.56	24.11	0.5779	1.605
SPAC57A7.09	SPAC57A7.09	0.6344	-1.051	0.197636826	15.25	28.9	1.73	1.821
SPBC11B10.02C	his3	0.7503	-1.048	0.124765054	5.923	12.35	3.751	2.326
SPAC688.03C	SPAC688.03c	0.6152	-1.042	0.210983673	12.61	24.2	0.6327	1.844
SPAC17A5.04C	mde10	0.5627	-1.042	0.249723085	9.487	18.66	2.147	1.194
SPBC3D6.02	but2	0.7117	-1.04	0.147703034	17.96	33.67	2.12	2.352
SPBC1734.06	rhp18	0.8816	-1.036	0.054728418	7.582	15.27	8.844	4.406
SPAC17A2.01	bsu1	0.6781	-1.035	0.168706256	14.04	26.72	1.295	2.182
SPBC211.06	gfh1	0.7573	-1.035	0.120732043	14.23	27.05	0.947	3.029
SPAC3H1.04C	mdm31	0.5519	-1.032	0.258139606	10.69	20.78	2.151	1.099
SPAC27D7.12C	but1	0.7051	-1.032	0.151749285	12.66	24.26	2.841	2.038
SPAC15A10.06	SPAC15A10.06	0.2731	-1.032	0.5636783	0	1.829	1.366	0
SPAC2E1P5.02C	mug109	0.6444	-1.027	0.190844468	14.7	27.86	0.9256	1.965
SPAC24H6.07	rps901	0.6735	-1.025	0.1716624	3.976	8.863	3.06	1.542
SPAC20G4.01	caf16	0.5622	-1.023	0.250109159	12.34	23.67	1.276	1.465
SPAC31G5.17C	rps1001	0.5933	-1.022	0.226725652	14.15	26.89	1.384	1.595
SPCC965.14C	SPCC965.14c	0.7491	-1.022	0.125460203	15.65	29.54	2.629	2.623
SPAC6G9.04	spo7	0.6184	-1.021	0.208730519	14.02	26.66	1.652	1.674
SPAC6B12.08	mug185	0.6883	-1.021	0.16222223	10.26	19.99	3.75	1.1
SPBC29A3.02C	his7	0.8501	-1.02	0.070529984	11.57	22.31	7.742	2.673
SPAC23H3.03C	npr2	0.6972	-1.02	0.156642622	8.975	17.71	1.757	2.234
SPBC83.19C	SPBC83.19c	0.7074	-1.014	0.150334945	13.16	25.12	2.199	2.217
SPBC32H8.11	mei4	0.7274	-1.008	0.138226703	16.44	30.92	1.678	2.514
SPBC365.10	arp5	0.3299	-1.008	0.481617684	0.5015	2.674	1.399	0.5015
SPAC227.11C	SPAC227.11c	0.6234	-1.002	0.205233202	12.94	24.71	1.201	1.756
SPCC16A11.08	atg20	0.1404	-0.9984	0.852632892	12.62	24.14	0.3531	0.5034
SPCC285.09C	cgs2	0.5491	-0.9974	0.260348556	11.45	22.05	2.125	1.012
SPAC9.10	thi9	0.7887	-0.9971	0.103088159	12.82	24.48	1.764	3.303
SPAC1786.02	SPAC1786.02	0.8595	-0.9969	0.065754119	10.33	20.08	6.832	3.773
SPAC20H4.07	rhp57	0.5014	-0.9953	0.29981567	9.578	18.74	1.183	1.194
SPCC61.03	SPCC61.03	0.6054	-0.9943	0.217957583	12.08	23.18	2.162	1.355
SPBC21C3.09C	SPBC21C3.09c	0.6849	-0.9934	0.164372834	13.87	26.35	0.4182	2.213
SPBC839.05C	rps1701	0.8527	-0.9922	0.069203737	8.518	16.85	7.064	3.192
SPAPB17E12.03	SPAPB17E12.03	0.6333	-0.9845	0.198390512	14.04	26.62	2.183	1.518

SPCC794.01C	SPCC794.01c	0.06754	-0.9841	1.170438944	14.28	27.04	0.3797	0.3615
SPAC139.06	hat1	0.7852	-0.9813	0.105019709	15.83	29.79	0.9215	3.266
SPBPB8B6.05C	SPBPB8B6.05c	0.7904	-0.9797	0.102153068	14.19	26.88	1.926	3.251
SPAC23A1.02C	SPAC23A1.02c	0.8456	-0.9778	0.072835026	10.17	19.75	6.876	2.807
SPAC22F8.12C	shf1	0.8513	-0.9776	0.069917367	10.65	20.6	2.889	4.589
SPBC1734.07C	SPBC1734.07c	0.7679	-0.9774	0.114695332	15.81	29.75	3.569	2.434
SPAC3G9.11C	SPAC3G9.11c	0.8603	-0.9758	0.065350077	10.39	20.14	7.117	3.468
SPBP19A11.02C	SPBP19A11.02c	0.5912	-0.9755	0.228265575	15.26	28.77	0.8015	1.59
SPBC18E5.08	SPBC18E5.08	0.63	-0.9717	0.200659451	14.38	27.2	1.666	1.639
SPAC1A6.04C	plb1	0.8317	-0.9712	0.080033299	6.674	13.55	7.007	1.649
SPAC1805.02C	SPAC1805.02c	0.7473	-0.964	0.126505018	17.24	32.26	1.764	2.601
SPBC1921.07C	sgf29	0.8224	-0.963	0.084916898	7.144	14.37	5.837	2.428
SPAC167.06C	mug143	0.5817	-0.9629	0.235300936	12.89	24.55	1.985	1.217
SPBC337.16	cho1	0.491	-0.9597	0.308918508	0.8058	3.129	1.817	0.8058
SPBC25B2.07C	mug164	0.6357	-0.9591	0.196747789	12.57	23.98	0.9584	1.775
SPAC1635.01	SPAC1635.01	0.6539	-0.958	0.184488663	12.33	23.54	2.214	1.595
SPBC1773.04	SPBC1773.04	0.7276	-0.9558	0.13810731	15.01	28.29	0.9925	2.458
SPAC22F3.11C	snu23	0.5314	-0.9514	0.27457845	14.87	28.03	1.849	0.9814
SPBC713.11C	pmp3	0.7495	-0.951	0.125228363	13.83	26.2	2.092	2.53
SPAC8C9.14	prr1	0.8726	-0.9476	0.059184791	10.48	20.25	6.874	4.126
SPAC17C9.05C	pmc3	0.8186	-0.9472	0.08692826	15.03	28.31	2.297	3.627
SPAC3G6.02	dss1	0.8762	-0.9469	0.057396751	11.24	21.6	7.564	3.961
SPCC550.09	SPCC550.09	0.6877	-0.9454	0.162600976	13.09	24.88	1.889	1.935
SPCC1739.14	npp106	0.8852	-0.9404	0.052958595	11.42	21.9	7.444	4.619
SPAC212.08C	SPAC212.08c	0.7609	-0.9384	0.118672416	13.52	25.62	2.859	2.441
SPAC24C9.14	otu1	0.7133	-0.9367	0.146727776	16	30.02	1.973	2.119
SPBC365.13C	hba1	0.7095	-0.9357	0.1490476	14.6	27.54	1.752	2.129
SPAC15F9.01C	SPAC15F9.01c	0.7043	-0.935	0.152242312	13.49	25.56	1.141	2.177
SPBC1683.03C	SPBC1683.03c	0.7508	-0.9323	0.124475736	11.89	22.73	0.9531	2.643
SPAC19G12.02C	pms1	0.6856	-0.9284	0.163929191	12.06	23.02	1.11	2.018
SPBC23E6.02	SPBC23E6.02	0.7531	-0.928	0.123147352	13.33	25.27	1.838	2.55
SPBC365.02C	cox10	0.7805	-0.9254	0.107627093	13.56	25.68	1.828	2.914
SPCC1753.03C	rec7	0.7664	-0.9246	0.115544504	14.79	27.84	2.111	2.661
SPAC16E8.05C	mde1	0.8467	-0.9243	0.07227044	8.47	16.65	6.683	2.547
SPAC4A8.03C	ptc4	0.6017	-0.923	0.220619989	1.343	4.016	2.544	0.8184
SPAC2F3.15	lsk1	0.7747	-0.9217	0.110866444	12.08	23.04	3.846	2.183
SPAC3C7.12	tip1	0.6204	-0.9161	0.207328211	10.54	20.31	2.932	0.2381
SPAC644.09	SPAC644.09	0.7827	-0.9127	0.106404666	14.43	27.19	1.882	2.895
SPCC24B10.18	SPCC24B10.18	0.7746	-0.9126	0.110922507	14.18	26.74	2.71	2.597
SPCC613.01	SPCC613.01	0.1934	-0.9119	0.71354353	11.12	21.32	0.751	0.4556
SPBC1289.08	uap1	0.7687	-0.9103	0.114243119	13.39	25.34	2.376	2.587
SPAP27G11.16	SPAP27G11.16	0.6285	-0.9082	0.201694718	14.29	26.93	1.156	1.607
SPAC637.07	moe1	0.7789	-0.9063	0.108518296	5.533	11.41	4.021	2.09
SPBC354.10	def1	0.8741	-0.9055	0.05843888	8.669	16.97	7.793	3.226
SPBC216.05	rad3	0.7351	-0.898	0.133653577	13.62	25.72	2.572	2.06
SPCC1259.04	iec3	0.7271	-0.896	0.138405855	15.04	28.23	1.86	2.166
SPBC19G7.10C	SPBC19G7.10c	0.6461	-0.8952	0.189700259	8.995	17.53	2.579	1.138
SPAC1782.12C	SPAC1782.12c	0.01454	-0.8948	1.837435593	7.378	14.66	0.2467	0.2072
SPBC1711.03	aim27	0.557	-0.8939	0.254144805	14.67	27.58	1.062	1.27
SPCC736.11	ago1	0.391	-0.8878	0.407823243	0	1.573	1.573	0
SPCC794.10	SPCC794.10	0.5807	-0.8846	0.236048174	15.51	29.06	1.14	1.336
SPBC119.06	sco1	0.6259	-0.8843	0.203495048	1.532	4.282	2.577	0.886
SPBC3B9.15C	scp1	0.7597	-0.8814	0.119357874	15.32	28.71	2.497	2.333
SPBC428.12C	SPBC428.12c	0.6949	-0.8792	0.158077688	15.5	29.03	1.586	1.894
SPCC24B10.16C	SPCC24B10.16c	0.8912	-0.8751	0.050024822	13.28	25.08	6.867	4.741
SPCC584.01C	SPCC584.01c	0.107	-0.8722	0.970616222	8.643	16.86	0.5675	0.3307
SPBC18E5.05C	iki1	0.4687	-0.8717	0.329105046	0.6832	2.756	1.883	0.4517
SPAC15A10.05C	mug182	0.8826	-0.8704	0.054236077	10.92	20.89	7.355	3.831
SPBC29A10.01	ccr1	0.7248	-0.8704	0.139781815	15.88	29.68	1.983	2.04
SPBP8B7.25	cyp4	0.6447	-0.8691	0.19064233	12.26	23.26	1.034	1.636
SPAC869.11	cat1	0.4703	-0.8675	0.327625021	8.93	17.36	1.065	0.9369
SPBC215.10	SPBC215.10	0.7723	-0.8654	0.112213965	17.02	31.69	2.01	2.562
SPCC663.08C	SPCC663.08c	0.1972	-0.8647	0.705093089	13.53	25.52	0.634	0.4704
SPAC343.12	rds1	0.7311	-0.8645	0.136023216	15.67	29.3	1.375	2.201
SPAC13C5.06C	mug121	0.8189	-0.864	0.086769129	13.87	26.12	4.812	2.378
SPAC31G5.10	eta2	0.7735	-0.8619	0.111539682	15.12	28.32	2.417	2.475
SPAC21E11.03C	pcr1	0.5168	-0.8589	0.286677495	14.58	27.36	0.6755	1.137
SPAPB24D3.08C	SPAPB24D3.08c	0.8009	-0.8578	0.096421706	17.72	32.93	2.175	2.938
SPAC16E8.18	SPAC16E8.18	0.7219	-0.8574	0.141522958	15.11	28.29	1.622	2.055

SPAC25G10.04C	rec10	0.727	-0.8548	0.138465589	16.95	31.56	1.082	2.174
SPBC1198.11C	reb1	0.6118	-0.8548	0.213390527	10.59	20.29	2.17	1.022
SPAC19E9.02	fin1	0.7435	-0.8533	0.128719027	12.55	23.75	2.976	1.837
SPBC1271.06C	mug96	0.7049	-0.853	0.151872489	14.5	27.21	2.733	1.494
SPAC1782.08C	rex3	0.7078	-0.8529	0.150089442	10.09	19.39	3.588	0.5256
SPBC15C4.05	SPBC15C4.05	0.7498	-0.8518	0.125054564	16.53	30.81	2.329	2.158
SPAC17H9.14C	SPAC17H9.14c	0.6532	-0.8517	0.184953824	13.3	25.08	1.572	1.542
SPAC16A10.05C	dad1	0.6382	-0.8497	0.1950432	3.031	6.877	2.221	1.246
SPAC589.03C	SPAC589.03c	0.6981	-0.8494	0.156082362	16.15	30.12	1.309	1.894
SPAC688.10	rev3	0.7343	-0.8459	0.134126472	14.14	26.55	1.644	2.135
SPAC4G8.13C	prz1	0.8959	-0.8423	0.047740463	11.95	22.67	7.63	4.419
SPBC1198.09	ubc16	0.7449	-0.838	0.127902026	13.14	24.76	1.466	2.248
SPBC887.15C	sur2	0.8687	-0.8376	0.061130178	8.84	17.15	5.986	3.485
SPBP8B7.02	SPBP8B7.02	0.9076	-0.834	0.042105513	18.7	34.61	4.618	6.227
SPBC3B9.04	SPBC3B9.04	0.7475	-0.8328	0.126388803	12.61	23.82	1.919	2.175
SPCC584.11C	SPCC584.11c	0.6487	-0.8304	0.187956102	7.257	14.33	1.518	1.481
SPBC17F3.01C	rga5	0.7885	-0.8298	0.103198302	15.19	28.38	3.083	2.377
SPAC23E2.01	fep1	0.8161	-0.8294	0.088256622	8.96	17.35	1.553	3.186
SPBP23A10.05	ssr4	0.7185	-0.8217	0.143573228	16.52	30.73	0.6322	2.055
SPAC2F7.17	SPAC2F7.17	0.7586	-0.8212	0.119987162	15.91	29.65	2.496	2.108
SPBC354.01	gtp1	0.7276	-0.8205	0.13810731	17.66	32.75	1.123	2.082
SPAC222.08C	SPAC222.08c	0.7189	-0.8204	0.143331516	11.31	21.51	1.684	1.914
SPBC31F10.08	mde2	0.6349	-0.8194	0.197294673	14.05	26.35	1.193	1.456
SPAC17H9.03C	rdl1	0.8711	-0.8194	0.059931986	9.511	18.31	6.4	3.215
SPBC1347.03	meu14	0.733	-0.8192	0.134896025	14.87	27.81	0.9698	2.142
SPBC11C11.06C	SPBC11C11.06c	0.7928	-0.8171	0.100836359	16.12	30.02	1.164	2.796
SPAC1F5.05C	SPAC1F5.05c	0.8863	-0.8138	0.052419251	16	29.8	3.054	5.03
SPAC1250.05	rpl3002	0.6903	-0.813	0.160962127	15.35	28.64	1.541	1.703
SPBC13A2.04C	SPBC13A2.04c	0.8008	-0.8094	0.096475936	10.8	20.58	5.097	0.7253
SPBC405.04C	ypt7	0.7941	-0.8087	0.100124804	15.92	29.64	2.593	2.54
SPBC2G2.09C	crs1	0.6855	-0.8085	0.163992541	14.45	27.04	1.841	1.576
SPAC10F6.15	SPAC10F6.15	0.7699	-0.8077	0.11356568	16.89	31.36	2.036	2.328
SPBC21B10.08C	SPBC21B10.08c	0.1831	-0.8058	0.737311656	16.06	29.89	0.4172	0.4552
SPAC3A11.05C	kms1	0.7884	-0.8052	0.103253384	16.04	29.85	1.764	2.616
SPAC694.02	SPAC694.02	0.7181	-0.8039	0.143815073	5.593	11.34	1.335	1.928
SPAC3F10.17	SPAC3F10.17	0.6559	-0.8029	0.183162369	15.11	28.2	1.438	1.481
SPAC17G6.06	rps2401	0.7876	-0.8012	0.103694293	14.22	26.61	4.47	1.2
SPBC887.11	pus2	0.8926	-0.8004	0.049343117	17.22	31.93	3.042	5.258
SPAC589.09	SPAC589.09	0.7259	-0.7941	0.139123204	15.6	29.05	1.057	2.005
SPAC2H10.01	SPAC2H10.01	0.784	-0.7937	0.105683937	14.83	27.7	1.421	2.564
SPBC16E9.12C	pab2	0.7804	-0.7918	0.107682739	14.94	27.88	1.464	2.505
SPAC31F12.01	zds1	0.8173	-0.7916	0.087618501	3.192	7.06	4.64	2.105
SPAC27F1.10	SPAC27F1.10	0.7759	-0.7915	0.110194248	14	26.22	1.486	2.445
SPCC16C4.20C	SPCC16C4.20c	0.6352	-0.7893	0.197089511	9.795	18.76	2.236	0.9404
SPCC338.02	mug112	0.71	-0.7874	0.148741651	14.01	26.22	1.558	1.778
SPCC61.02	spt3	0.7969	-0.7855	0.098596173	17.29	32.03	2.416	2.538
SPAC9E9.13	wos2	0.7153	-0.7851	0.145511775	13.9	26.03	0.69	1.933
SPAC1296.03C	sxa2	0.8062	-0.7848	0.093557206	15.45	28.77	2.445	2.682
SPBC8D2.10C	rmt3	0.733	-0.7799	0.134896025	13.13	24.64	2.822	1.492
SPBC557.02C	SPBC557.02c	0.8832	-0.7771	0.05394094	10.07	19.22	7.279	2.936
SPCC1840.09	SPCC1840.09	0.6321	-0.7756	0.19921421	9.466	18.15	2.219	0.8776
SPCC970.07C	raf2	0.8723	-0.7748	0.059334128	8.97	17.27	6.204	3.006
SPAC29A4.05	cam2	0.7446	-0.7742	0.128076968	14.17	26.48	1.286	2.083
SPAC1805.01C	ppk6	0.7972	-0.7711	0.09843271	12.82	24.09	0.8606	2.718
SPBP8B7.31	SPBP8B7.31	0.5538	-0.77	0.256647049	12.12	22.84	1.871	0.588
SPAC637.10C	rpn10	0.04974	-0.7698	1.303294219	7.29	14.28	0.3773	0.2303
SPCC1827.04	SPCC1827.04	0.792	-0.7698	0.101274818	15.76	29.29	2.99	2.213
SPBC2G5.04C	SPBC2G5.04c	0.7837	-0.7691	0.105850153	14.8	27.59	2.007	2.376
SPAC3C7.05C	mug191	0.766	-0.7636	0.11577123	13.63	25.52	2.635	1.94
SPBC660.12C	SPBC660.12c	0.7675	-0.7636	0.114921616	16.19	30.04	2.274	2.082
SPBC2A9.04C	SPBC2A9.04c	0.7822	-0.7601	0.106682188	11.73	22.13	3.105	1.957
SPCC622.16C	epe1	0.7987	-0.7585	0.097616316	16.77	31.07	1.39	2.644
SPAC1805.12C	uep1	0.7668	-0.7573	0.115317896	16.53	30.63	2.987	1.764
SPAC17A2.09C	csx1	0.8697	-0.7572	0.06063053	13.24	24.81	1.659	4.169
SPAC869.05C	SPAC869.05c	0.7014	-0.7565	0.154034238	12.61	23.69	1.987	1.501
SPBP35G2.12	SPBP35G2.12	0.8158	-0.7558	0.088416299	15.72	29.2	2.194	2.783
SPAC6G9.15C	SPAC6G9.15c	0.816	-0.7537	0.088309841	14.38	26.81	3.003	2.572
SPBC28F2.10C	ngg1	0.8267	-0.7489	0.082652062	7.65	14.88	2.719	2.845
SPBP35G2.13C	swc2	0.8644	-0.7467	0.063285242	17.25	31.9	0.2964	4.014

SPAC26A3.09C	rga2	0.9071	-0.7451	0.042344833	14.47	26.96	7.725	4.299
SPAC186.06	SPAC186.06	0.7993	-0.7441	0.097290187	15.48	28.75	2.023	2.499
SPCC584.03C	SPCC584.03c	0.7933	-0.7375	0.100562546	10.52	19.94	2.848	2.147
SPAC9.05	fml1	0.7202	-0.7373	0.142546883	12.99	24.33	1.992	1.597
SPBC17D11.03C	SPBC17D11.03c	0.7574	-0.7351	0.120674699	14.32	26.68	1.198	2.1
SPBC2D10.03C	SPBC2D10.03c	0.7922	-0.7349	0.101165162	15.59	28.93	1.36	2.472
SPBPB10D8.02C	SPBPB10D8.02c	0.8479	-0.7302	0.071655365	13.96	26.04	1.292	3.442
SPAC29A4.12C	mug108	0.7727	-0.7269	0.111989088	10.94	20.67	1.937	2.103
SPAC139.02C	oac1	0.8188	-0.7258	0.086822166	16.66	30.81	1.77	2.781
SPAC977.16C	dak2	0.7759	-0.7242	0.110194248	14.13	26.32	2.215	2.06
SPAC110.02	pds5	0.8089	-0.724	0.092105165	4.866	9.906	3.665	1.982
SPBC800.02	whi5	0.8669	-0.7208	0.062030997	16.05	29.73	2.387	3.786
SPAC13C5.03	tht1	0.8211	-0.7203	0.085603948	12.4	23.25	3.904	2.217
SPCC736.06	SPCC736.06	0.8447	-0.718	0.073297506	6.84	13.39	4.468	2.442
SPAC1071.03C	SPAC1071.03c	0.7507	-0.7167	0.124533584	15.55	28.82	1.809	1.865
SPCC1020.08	SPCC1020.08	0.7264	-0.7155	0.138824164	14.94	27.75	0.8309	1.823
SPAC8C9.16C	mug63	0.9111	-0.7133	0.040433953	11.95	22.44	7.492	4.433
SPAC4G9.02	rnh201	0.7922	-0.708	0.101165162	15.21	28.21	1.968	2.271
SPCC4B3.03C	SPCC4B3.03c	0.9165	-0.703	0.037867531	12.29	23.02	8.428	4.322
SPAC19G12.06C	hta2	0.822	-0.6992	0.085128182	15.12	28.03	1.714	2.732
SPBP22H7.06	SPBP22H7.06	0.3927	-0.6974	0.405939099	15.21	28.19	0.9619	0.5281
SPCC965.08C	alr1	0.6792	-0.6973	0.168002323	13.52	25.19	1.615	1.31
SPBC2F12.11C	rep2	0.7224	-0.6972	0.141222263	3.388	7.24	2.501	1.228
SPBC1539.10	nop16	0.9068	-0.6948	0.042488489	9.346	17.79	7.666	3.683
SPAC57A10.12C	ura3	0.8721	-0.6927	0.059433714	7.491	14.5	5.513	2.856
SPBC23G7.08C	rga7	0.4925	-0.6924	0.307593765	6.213	12.24	1.157	0.6864
SPAC57A10.09C	nhp6	0.6476	-0.6905	0.18869316	16.96	31.29	2.078	0.8127
SPAC17G8.08C	SPAC17G8.08c	0.8325	-0.6896	0.079615758	17	31.35	1.317	2.928
SPBC2D10.19C	SPBC2D10.19c	0.7654	-0.6855	0.116111542	12.96	24.18	3.226	1.198
SPCC417.05C	chr2	0.9022	-0.6849	0.044697177	9.564	18.16	7.544	3.204
SPAC15A10.16	bud6	0.8615	-0.6848	0.064744718	11.26	21.16	4.863	2.707
SPAC8C9.17C	spc34	0.8766	-0.6839	0.057198534	11.65	21.85	6.61	1.832
SPAC8C9.10C	SPAC8C9.10c	0.9132	-0.6837	0.039434097	11.61	21.79	7.418	4.317
SPCC1393.10	ctr4	0.8536	-0.6826	0.068745594	9.28	17.65	5.508	1.593
SPAC644.13C	SPAC644.13c	0.9002	-0.6767	0.045660991	16.35	30.17	2.286	4.843
SPAC2C4.14C	ppk11	0.684	-0.6763	0.164943898	14.38	26.68	0.9997	1.434
SPAC3A12.09C	SPAC3A12.09c	0.764	-0.676	0.116906641	17.66	32.49	2.066	1.789
SPCC4G3.08	psk1	0.7944	-0.6743	0.099960765	14.97	27.72	2.028	2.155
SPAC4D7.02C	SPAC4D7.02c	0.7753	-0.6731	0.110530216	14.19	26.34	1.043	2.1
SPAC688.11	end4	0.8328	-0.6716	0.079459283	6.484	12.68	4.47	1.672
SPCC970.02	SPCC970.02	0.7732	-0.6711	0.111708155	14.06	26.1	2.616	1.664
SPAC1D4.11C	lkh1	0.8741	-0.6702	0.05843888	16.38	30.21	2.676	3.67
SPBP4G3.02	pho1	0.843	-0.67	0.074172425	15.94	29.43	1.716	2.998
SPBC29B5.02C	isp4	0.861	-0.6658	0.064996849	15.05	27.85	1.005	3.462
SPAC26A3.14C	SPAC26A3.14c	0.8091	-0.6622	0.091997799	13.38	24.89	2.049	2.309
SPBC25B2.01	SPBC25B2.01	0.9185	-0.6619	0.036920839	14.83	27.45	3.409	5.728
SPCP1E11.03	mug170	0.7651	-0.6544	0.116281798	14.32	26.53	1.504	1.868
SPAC1687.22C	puf3	0.7942	-0.6517	0.100070117	12.67	23.61	1.729	2.132
SPAC4A8.02C	SPAC4A8.02c	0.8967	-0.6508	0.04735283	17.03	31.34	3.234	4.343
SPAC1F12.05	SPAC1F12.05	0.872	-0.6477	0.059483515	12.41	23.14	5.682	2.087
SPBC902.04	SPBC902.04	0.7495	-0.6475	0.125228363	13.01	24.21	0.7321	1.82
SPAC12B10.13	SPAC12B10.13	0.8259	-0.6471	0.083072534	13.35	24.81	1.139	2.645
SPAC22E12.14C	sck2	0.8733	-0.6461	0.05883654	14.49	26.82	2.178	3.577
SPCC4G3.17	SPCC4G3.17	0.8002	-0.6455	0.096801453	14.54	26.91	1.864	2.157
SPBC337.10C	SPBC337.10c	0.8215	-0.6407	0.085392432	13.92	25.8	1.7	2.475
SPBC14C8.03	fma2	0.7437	-0.6388	0.128602219	13.59	25.21	2.239	1.371
SPBC19G7.09	ulp1	0.7642	-0.6387	0.116792967	14.47	26.78	1.212	1.86
SPAC22H12.05C	SPAC22H12.05c	0.8667	-0.638	0.062131203	13.89	25.74	0.7235	3.475
SPBC365.04C	SPBC365.04c	0.9025	-0.6335	0.044552789	13.18	24.49	1.846	4.679
SPCC777.15	SPCC777.15	0.8484	-0.6296	0.07139934	14.76	27.27	1.304	2.962
SPAPB18E9.01	trm5	0.832	-0.6284	0.079876674	15.93	29.35	2.124	2.518
SPAC25B8.05	SPAC25B8.05	0.6207	-0.628	0.207118255	11.2	20.96	1.181	0.9921
SPAC12B10.10	SPAC12B10.10	0.8466	-0.6276	0.072321736	14.17	26.22	2.419	2.737
SPBC146.13C	myo1	0.8628	-0.6221	0.064089864	6.74	13.05	4.354	2.422
SPAC4G8.06C	trm12	0.8427	-0.6217	0.074327006	15.38	28.35	2.556	2.591
SPAC821.03C	SPAC821.03c	0.825	-0.6204	0.083546051	15.19	28.01	1.662	2.449
SPAC631.01C	acp2	0.7824	-0.6181	0.106571158	13.87	25.68	2.986	1.309
SPAC222.13C	SPAC222.13c	0.8652	-0.6157	0.062883489	15.39	28.36	1.364	3.271
SPAC8E11.06	SPAC8E11.06	0.7851	-0.6153	0.105075023	13.86	25.65	1.139	1.994

SPAC57A10.02	cdr2	0.8596	-0.6153	0.065703593	6.26	12.18	4.55	2.115
SPCC1494.09C	SPCC1494.09c	0.8166	-0.6151	0.087990624	13.94	25.8	3.178	1.796
SPCC584.16C	SPCC584.16c	0.9139	-0.6151	0.039101323	12.19	22.68	5.143	4.574
SPBC12C2.07C	SPBC12C2.07c	0.8197	-0.614	0.086345065	14.17	26.2	0.347	2.465
SPAC27D7.10C	EMPTY	0.8188	-0.6131	0.086822166	15.49	28.53	1.794	2.298
SPAC6G9.03C	mug183	0.8375	-0.6072	0.077015184	15.88	29.22	2.007	2.541
SPBC902.03	SPBC902.03	0.9236	-0.6034	0.034516076	15.13	27.89	3.957	5.473
SPBPB2B2.12C	gal10	0.8402	-0.5997	0.075617323	9.246	17.45	3.447	2.08
SPAC139.04C	fap2	0.6991	-0.5994	0.155460698	14.72	27.15	1.618	1.157
SPBC609.02	ptn1	0.8948	-0.5993	0.048274025	15.37	28.31	2.136	4.045
SPAC222.16C	csn3	0.8207	-0.5983	0.085815567	15.64	28.77	1.883	2.245
SPAC1F3.07C	rsc58	0.8636	-0.5982	0.063687366	9.557	18	4.301	2.409
SPCC970.05	rpl3601	0.708	-0.5969	0.149966742	13.85	25.59	2.068	0.9685
SPBC1773.17C	SPBC1773.17c	0.826	-0.5946	0.083019953	16.47	30.23	1.542	2.37
SPCP1E11.05C	are2	0.8555	-0.5894	0.067779986	16.6	30.46	1.556	2.88
SPAC607.10	spo3	0.84	-0.5891	0.075720714	15.69	28.85	1.694	2.555
SPAC21E11.04	ppr1	0.8171	-0.589	0.08772479	16.2	29.75	2.25	2.056
SPCC613.02	SPCC613.02	0.8427	-0.5885	0.074327006	15.94	29.29	1.607	2.613
SPAC1687.12C	coq4	0.8001	-0.5872	0.09685573	2.701	5.827	2.686	1.618
SPBC115.03	SPBC115.03	0.8862	-0.5857	0.052468254	12.23	22.71	5.759	2.156
SPCC777.06C	SPCC777.06c	0.8072	-0.5838	0.093018847	12.98	24.03	1.757	2.021
SPBC23E6.03C	nta1	0.8158	-0.5802	0.088416299	13.08	24.21	1.35	2.193
SPCC757.02C	SPCC757.02c	0.7601	-0.5796	0.119129267	13.25	24.52	1.76	1.502
SPAC25B8.04C	mss51	0.8067	-0.579	0.093287943	13.84	25.55	1.674	2.014
SPCC550.07	SPCC550.07	0.8452	-0.5787	0.073040512	8.523	16.13	3.381	2.102
SPAC3G6.03C	SPAC3G6.03c	0.8956	-0.578	0.047885915	15.91	29.21	1.961	3.946
SPAC13G6.06C	gcv2	0.9204	-0.5774	0.03602339	16.28	29.88	3.142	5.1
SPBP8B7.18C	SPBP8B7.18c	0.7972	-0.5772	0.09843271	10	18.74	2.767	1.475
SPBC649.02	rps1902	0.9208	-0.5709	0.035834689	11.16	20.8	6.984	3.845
SPCC126.10	iah1	0.8781	-0.5631	0.056456023	16.66	30.53	1.373	3.312
SPAC18G6.15	mal3	0.8343	-0.563	0.078677756	11.75	21.83	3.686	1.502
SPAC186.05C	SPAC186.05c	0.7757	-0.5627	0.110306209	14.09	25.96	1.142	1.723
SPCC1450.12	SPCC1450.12	0.9418	-0.5614	0.026041314	14.01	25.82	8.765	5.464
SPCC188.13C	dcr1	0.7797	-0.5588	0.108072466	6.722	12.9	1.21	1.735
SPBC2D10.14C	myo51	0.855	-0.5555	0.068033885	18.55	33.85	1.122	2.742
SPCC965.13	SPCC965.13	0.862	-0.5551	0.064492734	16.28	29.83	2.984	2.532
SPCC550.01C	SPCC550.01c	0.1606	-0.5549	0.794254459	10.3	19.24	0.447	0.2376
SPAC23C4.07	tht2	0.8222	-0.554	0.085022528	13.93	25.68	1.737	2.101
SPAC16E8.17C	SPAC16E8.17c	0.8244	-0.5535	0.083862017	11.84	21.96	3.806	0.9297
SPAC4H3.04C	SPAC4H3.04c	0.8327	-0.5528	0.079511435	11.84	21.96	3.232	1.717
SPAC823.03	ppk15	0.9015	-0.5479	0.045034269	15.48	28.41	2.95	3.815
SPBC16G5.09	SPBC16G5.09	0.782	-0.5479	0.106793247	13.97	25.73	1.595	1.638
SPAC17G6.15C	SPAC17G6.15c	0.8259	-0.5477	0.083072534	14.97	27.5	3.473	1.333
SPCC1620.04C	mug55	0.8145	-0.543	0.089108911	13.86	25.52	1.351	2.023
SPAC29B12.12	SPAC29B12.12	0.8237	-0.5392	0.084230934	15.81	28.97	1.357	2.124
SPAC3H1.14	SPAC3H1.14	0.8903	-0.537	0.050463627	15.43	28.29	2.382	3.394
SPBC1347.12	arp1	0.8072	-0.5351	0.093018847	14.49	26.63	1.117	1.939
SPAP27G11.06C	vas2	0.8848	-0.5333	0.053154886	6.611	12.66	4.423	2.494
SPAC56F8.16	esc1	0.8309	-0.5318	0.080451241	16.03	29.35	1.868	2.098
SPBC106.02C	srx1	0.857	-0.5294	0.067019178	12.8	23.63	1.215	2.637
SPCC1223.13	cbf12	0.5988	-0.5259	0.222718208	17.55	32.04	1.323	0.5777
SPAC3C7.13C	SPAC3C7.13c	0.8819	-0.5252	0.054580657	12.96	23.89	5.494	1.153
SPAC57A7.08	pzh1	0.4078	-0.5245	0.389552779	17.64	32.19	0.854	0.3294
SPCC1235.03	SPCC1235.03	0.8567	-0.5201	0.067171233	15.4	28.22	3.385	1.99
SPBC1A4.09	SPBC1A4.09	0.8956	-0.5198	0.047885915	4.067	8.129	5.012	2.672
SPBC2D10.05	exg3	0.8766	-0.5183	0.057198534	13.73	25.25	3.245	2.605
SPCC338.04	cid2	0.8519	-0.5178	0.069611382	16.32	29.84	1.949	2.368
SPBC106.16	pre6	0.8709	-0.5176	0.060031709	14.38	26.41	2.428	2.678
SPBC6B1.10	prp17	0.8694	-0.5152	0.060780364	13.11	24.15	3.33	2.335
SPCC1739.12	ppe1	0.858	-0.5118	0.066512712	15.62	28.58	2.117	2.419
SPAC18B11.03C	SPAC18B11.03c	0.9311	-0.5111	0.031003673	13.36	24.57	8.233	3.204
SPBC660.05	SPBC660.05	0.8926	-0.5094	0.049343117	14.32	26.29	4.091	2.776
SPAC9G1.05	SPAC9G1.05	0.8758	-0.5061	0.057595059	13.98	25.68	2.131	2.795
SPAC6G9.16C	SPAC6G9.16c	0.8778	-0.5061	0.056604423	13.27	24.42	2.366	2.799
SPAC17A2.13C	rad25	0.7596	-0.5058	0.119415044	11.99	22.15	1.327	1.366
SPAC227.06	SPAC227.06	0.8545	-0.5055	0.068287933	14.61	26.79	1.751	2.388
SPBC1306.02	SPBC1306.02	0.904	-0.504	0.04383157	7.933	14.95	5.088	2.797
SPBC1711.01C	mat1-m	0.8289	-0.5017	0.08149786	14.36	26.33	1.613	1.983
SPCC777.04	SPCC777.04	0.86	-0.4989	0.065501549	15.57	28.48	0.8466	2.568

SPAC27D7.11C	SPAC27D7.11c	0.9361	-0.4966	0.028677755	12.01	22.17	5.329	5.062
SPAC16C9.05	cph1	0.938	-0.4947	0.027797162	12.33	22.73	7.851	4.195
SPBC3H7.03C	SPBC3H7.03c	0.8768	-0.4932	0.057099459	3.415	6.927	4.095	1.985
SPAC23E2.03C	ste7	0.9081	-0.4928	0.041866324	12.96	23.85	4.574	3.298
SPBC29B5.03C	rpl26	0.8831	-0.4927	0.053990115	16.54	30.19	2.923	2.721
SPBC29A3.18	cyt1	0.7901	-0.4907	0.102317938	2.58	5.443	1.911	1.449
SPBC19F8.02	SPBC19F8.02	0.864	-0.4865	0.063486258	15.66	28.61	1.673	2.484
SPCC757.11C	SPCC757.11c	0.9233	-0.4833	0.034657164	15.74	28.74	0.6742	4.608
SPAC1556.03	azr1	0.8526	-0.482	0.069254672	16.28	29.71	1.62	2.252
SPCC417.12	SPCC417.12	0.8545	-0.4816	0.068287933	14.12	25.87	1.806	2.25
SPBC31F10.15C	atp15	0.8168	-0.4785	0.087884271	8.669	16.21	2.744	1.307
SPAC17G6.02C	tco1	0.8666	-0.4783	0.062181315	17	30.97	1.266	2.547
SPBC685.03	SPBC685.03	0.7576	-0.474	0.120560034	14.47	26.48	1.242	1.267
SPAC664.13	SPAC664.13	0.8002	-0.472	0.096801453	14.59	26.7	2.019	1.37
SPCC4G3.02	aph1	0.7976	-0.4716	0.098214855	7.252	13.69	0.8246	1.639
SPBC83.01	ucp8	0.6777	-0.4716	0.168962514	15.42	28.16	0.7543	0.9665
SPAC1F3.06C	spo15	0.6413	-0.4689	0.19293876	15.17	27.72	1.276	0.6278
SPAC630.10	SPAC630.10	0.6389	-0.468	0.194567112	5.785	11.08	0.4512	0.8769
SPAC323.04	SPAC323.04	0.8361	-0.465	0.077741777	3.106	6.328	2.671	1.611
SPAC1D4.13	byr1	0.8835	-0.463	0.053793446	13.02	23.89	4.077	2.08
SPBC2G5.01	SPBC2G5.01	0.8381	-0.4625	0.076704159	15.06	27.5	2.069	1.807
SPAC11D3.18C	SPAC11D3.18c	0.9021	-0.461	0.044745317	12.88	23.64	5	2.362
SPAC56F8.06C	alg10	0.9271	-0.4596	0.032873419	12	22.08	2.172	4.51
SPAC9E9.15	SPAC9E9.15	0.8136	-0.4595	0.08958906	16.32	29.73	0.9719	1.727
SPAC16A10.02	sub1	0.9035	-0.4592	0.044071843	16.02	29.21	1.26	3.434
SPBC32F12.05C	cwf12	0.8972	-0.4587	0.047110735	6.487	12.31	4.351	2.358
SPAC11D3.02C	SPAC11D3.02c	0.8469	-0.4578	0.072167867	14.73	26.92	1.364	2.077
SPAC22A12.10	SPAC22A12.10	0.8743	-0.456	0.058339522	13.67	25.04	2.772	2.258
SPCC417.02	dad5	0.9368	-0.4559	0.028353118	11.31	20.86	7.099	3.786
SPAC4G9.05	mpf1	0.8427	-0.4525	0.074327006	14.8	27.02	1.676	1.93
SPCC737.05	SPCC737.05	0.8253	-0.4524	0.083388155	14.41	26.35	0.7019	1.851
SPBC19F8.08	rps401	0.9351	-0.4521	0.029141943	10.3	19.05	6.86	3.661
SPCC569.03	SPCC569.03	0.9134	-0.4505	0.039338993	15.07	27.51	6.157	1.807
SPAC652.01	SPAC652.01	0.9027	-0.4493	0.044456558	14.12	25.83	4.628	2.493
SPAC9E9.03	leu2	0.8429	-0.4476	0.074223946	12.16	22.34	1.89	1.855
SPAP27G11.12	SPAP27G11.12	0.916	-0.4464	0.038104526	14.3	26.14	2.323	3.734
SPAC26H5.11	mug56	0.8395	-0.4463	0.0759793	15.25	27.81	1.866	1.804
SPAC1F7.08	fio1	0.9212	-0.4461	0.035646071	8.888	16.54	5.412	3.065
SPAC11H11.02C	mug162	0.8363	-0.4458	0.077637903	15.88	28.93	2.132	1.668
SPBC428.02C	eca39	0.8712	-0.4433	0.059882133	5.168	9.944	3.477	1.734
SPAC1805.09C	fmt1	0.7711	-0.4408	0.112889297	10.75	19.82	1.803	1.03
SPCC569.04	SPCC569.04	0.9013	-0.4406	0.045130629	14.8	27.01	1.26	3.215
SPAC823.02	SPAC823.02	0.8379	-0.44	0.07680781	13.41	24.54	1.673	1.798
SPAC23H3.08C	bub3	0.8499	-0.4397	0.070632171	14.41	26.31	1.935	1.911
SPAC17H9.13C	SPAC17H9.13c	0.8743	-0.4385	0.058339522	5.728	10.93	2.89	2.088
SPAC6G10.08	idp1	0.8944	-0.4377	0.048468209	17.76	32.26	4.927	1.402
SPAC6G9.13C	bqt1	0.8722	-0.4337	0.059383918	15.9	28.95	0.7142	2.455
SPAC11D3.07C	SPAC11D3.07c	0.9306	-0.4318	0.031236952	16.82	30.57	3.12	4.308
SPAC25H1.05	meu29	0.813	-0.4294	0.089909454	2.425	5.058	2.111	1.264
SPAC19G12.12	dlp1	0.8649	-0.4251	0.063034103	6.996	13.15	3.032	1.758
SPCC1442.04C	SPCC1442.04c	0.8996	-0.4243	0.045950553	8.041	15	5.405	0.6271
SPBP35G2.14	SPBP35G2.14	0.8865	-0.4205	0.05232126	15.58	28.35	0.9616	2.672
SPAC11E3.15	rpl22	0.9362	-0.4171	0.028631363	16.87	30.64	2.588	4.638
SPCC1020.13C	SPCC1020.13c	0.8908	-0.4151	0.050219792	12.44	22.78	1.619	2.671
SPCC132.02	hst2	0.8881	-0.4137	0.05153813	15.87	28.86	1.427	2.617
SPAC2G11.13	atg22	0.8514	-0.4134	0.069866354	14.84	27.03	1.642	1.863
SPAC4F8.01	did4	0.9073	-0.4077	0.042249089	17.08	31	1.514	3.141
SPCC330.01C	rhp16	0.9381	-0.4061	0.027750864	16.82	30.53	3.146	4.569
SPAC13G7.12C	SPAC13G7.12c	0.9065	-0.4	0.042632192	6.12	11.55	4.477	2.058
SPBC29A10.10C	SPBC29A10.10c	0.8684	-0.3997	0.061280185	18	32.6	1.191	2.144
SPBC1289.14	SPBC1289.14	0.9154	-0.3975	0.038389092	13.01	23.76	3.717	2.891
SPAC4G8.08	SPAC4G8.08	0.8907	-0.3944	0.050268548	15.59	28.33	2.089	2.436
SPBPJ4664.05	SPBPJ4664.05	0.8905	-0.3922	0.050366076	17.07	30.95	1.147	2.564
SPAC29B12.11C	SPAC29B12.11c	0.8867	-0.3916	0.052223292	15.14	27.53	2.277	2.267
SPBC1683.11C	SPBC1683.11c	0.8412	-0.3894	0.075100736	14.92	27.13	1.211	1.687
SPAC222.14C	SPAC222.14c	0.9093	-0.3889	0.041292809	14.59	26.54	1.577	3.053
SPAC17C9.16C	SPAC17C9.16c	0.9345	-0.3864	0.029420694	16.96	30.74	2.34	4.184
SPBC18H10.11C	ppr2	0.8229	-0.3831	0.084652938	6.157	11.59	2.273	1.013
SPCC1739.03	hrr1	0.895	-0.3824	0.048176965	13.55	24.69	1.936	2.496

SPCC1620.08	SPCC1620.08	0.844	-0.3805	0.073657553	15.46	28.06	1.444	1.632
SPAC20G8.09C	SPAC20G8.09c	0.8648	-0.38	0.063084319	15.09	27.42	1.286	1.956
SPAC16C9.02C	SPAC16C9.02c	0.8106	-0.3787	0.091193401	15.09	27.41	0.7761	1.402
SPBC1A4.04	SPBC1A4.04	0.9174	-0.3758	0.037441264	7.298	13.6	4.748	2.202
SPAC637.06	SPAC637.06	0.9464	-0.3734	0.023925268	9.841	18.1	7.045	3.55
SPAC13D6.04C	btb3	0.8614	-0.3728	0.064795133	14.11	25.66	1.91	1.719
SPAC343.19	lsb6	0.8436	-0.3728	0.073863429	14.63	26.58	0.8869	1.683
SPAPB1A10.03	nxt1	0.9174	-0.3691	0.037441264	15.54	28.19	2.013	3.133
SPBC6B1.02	ppk30	0.7447	-0.3651	0.128018646	18.06	32.65	1.791	0.1931
SPBC1711.13	his2	0.9011	-0.3636	0.04522701	6.731	12.57	3.737	1.849
SPBC1105.04C	cbp1	0.8715	-0.3636	0.059732609	16.3	29.53	1.023	2.008
SPCC417.09C	SPCC417.09c	0.9445	-0.3605	0.024798038	15.41	27.94	3.782	4.398
SPAC144.01	SPAC144.01	0.9057	-0.359	0.043015632	16.28	29.49	1.31	2.72
SPBC336.03	efc25	0.9394	-0.358	0.027149444	14.82	26.9	3.415	4.003
SPCC162.10	ppk33	0.9135	-0.3562	0.039291448	14.76	26.79	1.28	2.956
SPAC13C5.07	rad32	0.9324	-0.3529	0.030397735	7.891	14.61	5.156	2.725
SPAPB1E7.04C	SPAPB1E7.04c	0.9493	-0.3516	0.022596519	14.69	26.66	5.377	4.314
SPAC1782.11	met14	0.9326	-0.3487	0.030304589	12.34	22.48	5.069	2.868
SPAPYUG7.06	mug67	0.9579	-0.3473	0.018679827	12.6	22.94	8.209	4.273
SPAC17A5.11	rec12	0.9271	-0.3471	0.032873419	16.36	29.61	1.511	3.421
SPBC16A3.03C	ppr7	0.9344	-0.3467	0.02946717	15.51	28.1	3.638	3.439
SPBC1604.16C	SPBC1604.16c	0.8777	-0.3462	0.056653902	14.11	25.63	0.9458	2.02
SPBC13E7.04	atp16	0.9242	-0.3457	0.034234036	10.91	19.94	4.432	2.546
SPAC1805.07C	dad2	0.9159	-0.3424	0.038151941	8.425	15.54	5.198	0.6607
SPBP4H10.09	rsv1	0.9597	-0.3423	0.017864505	12.35	22.49	7.823	4.77
SPBC21H7.07C	his5	0.8866	-0.3409	0.052272273	4.81	9.127	2.825	1.646
SPAC19G12.16C	adg2	0.9452	-0.3409	0.024476287	9.951	18.24	6.774	2.794
SPAC10F6.16	mug134	0.8275	-0.3378	0.082231998	14.18	25.73	0.8074	1.37
SPAC24H6.13	SPAC24H6.13	0.9241	-0.336	0.03428103	14.46	26.23	1.082	3.208
SPCC306.05C	ins1	0.8661	-0.3307	0.062431961	15.36	27.8	1.482	1.651
SPAC4D7.03	pop2	0.6266	-0.3297	0.203009609	16.78	30.33	0.9092	0.3833
SPAC4C5.01	SPAC4C5.01	0.9204	-0.3287	0.03602339	13.5	24.51	2.585	2.751
SPBP4H10.19C	SPBP4H10.19c	0.9087	-0.3252	0.041579472	16.06	29.05	2.273	2.359
SPCC24B10.17	emp24	0.8654	-0.3251	0.062783109	13.41	24.35	1.568	1.586
SPBC2F12.03C	SPBC2F12.03c	0.9418	-0.3239	0.026041314	16.21	29.31	2.841	3.848
SPBC28E12.02	SPBC28E12.02	0.8638	-0.3226	0.0635868	13.15	23.87	1.031	1.656
SPAC8E11.03C	dmc1	0.7513	-0.3197	0.124186611	15.89	28.73	0.8899	0.8117
SPAC11E3.13C	gas5	0.9157	-0.3163	0.038246786	11.48	20.91	4.594	1.082
SPAC26H5.02C	SPAC26H5.02c	0.886	-0.3155	0.052566278	14.81	26.81	1.676	1.852
SPBC31F10.14C	hip3	0.837	-0.3128	0.077274542	9.409	17.23	2.346	0.5207
SPBC31F10.13C	hip1	0.9546	-0.3114	0.02017857	17.2	31.04	1.778	4.969
SPCC330.14C	rpl2402	0.9663	-0.3081	0.01488802	13.35	24.21	8.518	5.089
SPAC1071.12C	stp1	0.9603	-0.3071	0.017593071	15.55	28.09	2.697	5.541
SPCC794.15	SPCC794.15	0.6441	-0.3062	0.191046701	16.74	30.21	0.9022	0.365
SPCC757.09C	rnc1	0.8875	-0.3001	0.051831638	11.1	20.21	2.681	1.356
SPAC1687.16C	erg31	0.9526	-0.2968	0.021089423	16.65	30.03	2.602	4.428
SPAC22E12.18	SPAC22E12.18	0.8576	-0.2965	0.066715228	2.686	5.286	2.249	0.9364
SPBC409.17C	SPBC409.17c	0.8649	-0.2944	0.063034103	14.67	26.52	1.217	1.478
SPCC18.17C	SPCC18.17c	0.9134	-0.2943	0.039338993	16.4	29.58	1.236	2.419
SPBC2A9.05C	tvp23	0.9425	-0.2939	0.025718641	15.14	27.35	2.326	3.585
SPBC691.04	SPBC691.04	0.9113	-0.2928	0.04033863	15.38	27.78	1.555	2.302
SPBC32F12.06	pch1	0.9491	-0.2918	0.022688027	15.47	27.93	2.462	4.044
SPAC1039.02	SPAC1039.02	0.938	-0.2908	0.027797162	13.92	25.18	1.927	3.317
SPAC20G8.04C	SPAC20G8.04c	0.9356	-0.2846	0.028909787	16.54	29.82	1.418	3.172
SPAC15A10.10	mde6	0.9619	-0.2835	0.016870075	11	20	7.467	3.837
SPAC2E12.03C	SPAC2E12.03c	0.9267	-0.2823	0.033060837	16.68	30.06	1.728	2.702
SPAC30.02C	SPAC30.02c	0.9349	-0.277	0.02923484	13.02	23.56	5.077	1.442
SPAC4A8.05C	myp2	0.9198	-0.2745	0.036306595	15	27.06	3.3	1.836
SPAC1751.01C	gti1	0.9245	-0.2737	0.034093085	14.39	25.99	2.013	2.472
SPAC10F6.13C	SPAC10F6.13c	0.924	-0.271	0.034328029	5.456	10.15	3.487	1.882
SPAC24B11.07C	SPAC24B11.07c	0.8526	-0.2706	0.069254672	14.63	26.4	1.659	1.034
SPCC1322.15	rpl3402	0.9049	-0.2703	0.043399412	14.9	26.89	1.53	1.947
SPBP16F5.07	apm1	0.9499	-0.2699	0.022322112	12.15	22.02	4.408	3.273
SPBC354.08C	SPBC354.08c	0.9597	-0.2678	0.017864505	14.58	26.31	3.827	4.517
SPAC10F6.04	SPAC10F6.04	0.9015	-0.2631	0.045034269	13.73	24.79	2.063	1.662
SPCC1906.02C	SPCC1906.02c	0.9073	-0.2618	0.042249089	16.62	29.91	2.008	1.815
SPBC56F2.01	pof12	0.9752	-0.2603	0.010906307	10.31	18.73	9.509	5.969
SPAC664.01C	swi6	0.9254	-0.2595	0.033670505	13.83	24.96	2.815	2.118
SPBC31E1.02C	pmr1	0.934	-0.2593	0.029653124	18.75	33.69	1.631	2.779

SPBP35G2.07	ilv1	0.9281	-0.2572	0.032405227	4.266	8.015	3.165	2.069
SPAC11H11.05C	fta6	0.9547	-0.2564	0.020133077	15.39	27.73	2.099	4.038
SPBC609.03	iqw1	0.9695	-0.2558	0.013452187	16.43	29.57	3.172	5.972
SPAC29E6.01	pof11	0.8843	-0.2545	0.053400375	12.53	22.65	1.895	1.288
SPAC26H5.08C	bgl2	0.9647	-0.2522	0.015607721	10.97	19.89	6.873	3.857
SPCC23B6.03C	tel1	0.8641	-0.2516	0.063435995	14.35	25.87	0.6602	1.315
SPACUNK4.17	SPACUNK4.17	0.9572	-0.2486	0.01899731	16.36	29.44	1.744	4.181
SPAPB8E5.04C	SPAPB8E5.04c	0.9662	-0.2472	0.014932967	11	19.93	6.988	3.961
SPAC15F9.02	seh1	0.9625	-0.2472	0.016599262	17.77	31.93	3.858	4.467
SPBC11G11.03	mrt4	0.949	-0.2439	0.022733788	18.36	32.98	3.3	3.109
SPBC106.10	pka1	0.921	-0.2401	0.03574037	13.28	23.95	1.891	2.028
SPCC613.11C	meu23	0.9268	-0.2401	0.033013975	14.37	25.89	1.019	2.358
SPAC4G9.12	SPAC4G9.12	0.9427	-0.2396	0.025626493	16.94	30.45	1.946	2.924
SPBC16G5.07C	SPBC16G5.07c	0.9369	-0.2392	0.028306761	15.09	27.17	1.498	2.691
SPAC22A12.01C	pso2	0.9275	-0.2355	0.032686082	14.92	26.86	2.355	2.076
SPAC1565.04C	ste4	0.9496	-0.2317	0.022459294	12.7	22.92	4.663	2.455
SPAC31G5.15	psd3	0.9688	-0.2313	0.01376587	11.3	20.43	7.314	3.896
SPBC660.09	mug168	0.9584	-0.2309	0.018453195	14.74	26.53	2.414	3.907
SPAC20G4.02C	fus1	0.951	-0.2305	0.021819483	13.73	24.75	1.016	3.422
SPCC1450.08C	wtf16	0.9473	-0.2302	0.023512463	13.7	24.69	4.353	2.265
SPBC2A9.02	SPBC2A9.02	0.9291	-0.2301	0.03193754	13.8	24.86	1.994	2.173
SPAC27F1.06C	SPAC27F1.06c	0.9622	-0.2288	0.016734647	16.45	29.56	2.578	4.272
SPCC777.10C	ubc12	0.8983	-0.228	0.0465786	8.95	16.26	1.524	1.459
SPCC63.02C	aah3	0.391	-0.2278	0.407823243	0	0.4037	0.4037	0
SPAC27E2.02	SPAC27E2.02	0.4641	-0.2275	0.333388432	7.038	12.87	0.4511	0.1256
SPCC1919.09	tif6	0.9396	-0.2262	0.027056992	15.82	28.43	1.679	2.627
SPAC1687.07	SPAC1687.07	0.9541	-0.2175	0.020406104	14.78	26.58	2.573	3.248
SPBC29A10.07	pom152	0.9401	-0.2133	0.026825947	16.66	29.9	2.174	2.387
SPAC688.14	set13	0.9234	-0.2108	0.03461013	14.66	26.36	1.957	1.769
SPBC56F2.10C	alg5	0.9549	-0.2106	0.020042107	9.507	17.22	2.405	3.231
SPCC306.08C	SPCC306.08c	0.8853	-0.2091	0.052909536	16.14	28.97	1.648	1.032
SPAC2F3.16	SPAC2F3.16	0.9261	-0.2088	0.033342116	15.03	27.01	1.587	1.924
SPAC6G10.12C	ace2	0.951	-0.2084	0.021819483	6.542	11.96	4.24	2.205
SPAC23A1.06C	cmk2	0.9326	-0.2062	0.030304589	13.95	25.09	2.798	1.725
SPBC337.07C	SPBC337.07c	0.9125	-0.2055	0.039767127	13.96	25.11	1.441	1.571
SPCC306.02C	SPCC306.02c	0.8954	-0.2041	0.04798291	11.15	20.13	1.62	1.17
SPAC186.09	SPAC186.09	0.9281	-0.2039	0.032405227	13.94	25.07	1.279	1.99
SPAC23G3.07C	snf30	0.9577	-0.2036	0.018770513	16.03	28.76	2.134	3.39
SPAC4F10.11	spn1	0.8783	-0.2033	0.056357117	15.23	27.36	1.197	1.067
SPAC22A12.04C	rps2201	0.942	-0.2026	0.025949097	13.25	23.84	3.423	1.846
SPBC16A3.16	SPBC16A3.16	0.671	-0.2021	0.17327748	13.22	23.79	0.4792	0.361
SPAC7D4.03C	SPAC7D4.03c	0.9562	-0.2007	0.019451261	12.76	22.96	3.953	2.7
SPAC1805.05	cki3	0.9619	-0.1986	0.016870075	16.45	29.5	1.162	3.794
SPCC1235.08C	pdh1	0.9395	-0.1982	0.027103216	12.59	22.66	3.093	1.798
SPBC18E5.04	rpl1001	0.9642	-0.198	0.015832873	9.097	16.47	3.773	3.614
SPCC330.11	btb1	0.9737	-0.1977	0.01157483	13.3	23.93	8.475	3.137
SPAC25B8.17	SPAC25B8.17	0.9483	-0.1975	0.023054249	17.67	31.66	2.942	2.389
SPBC887.02	SPBC887.02	0.8887	-0.1952	0.05124482	15.6	27.98	0.57	1.254
SPBC1685.02C	rps1202	0.9706	-0.1942	0.012959713	9.82	17.75	5.932	3.772
SPAC1142.08	fhl1	0.9747	-0.1917	0.011129034	11.22	20.22	7.122	4.183
SPAC1565.07C	SPAC1565.07c	0.9564	-0.191	0.019360433	14.78	26.53	2.292	3.023
SPCC1884.02	nic1	0.9363	-0.1793	0.028584977	13.2	23.71	3	1.322
SPAC644.07	SPAC644.07	0.9329	-0.178	0.030164907	2.408	4.582	2.748	1.38
SPBP4H10.18C	SPBP4H10.18c	0.9552	-0.1767	0.019905686	17.79	31.84	1.593	2.796
SPBC16H5.13	SPBC16H5.13	0.9496	-0.1749	0.022459294	5.088	9.327	3.113	1.986
SPBC16A3.07C	nrm1	0.9651	-0.1719	0.015427684	6.257	11.39	3.838	3.058
SPBC6B1.03C	SPBC6B1.03c	0.9533	-0.171	0.020770407	14.84	26.6	2.371	2.423
SPAC1420.01C	SPAC1420.01c	0.9464	-0.1692	0.023925268	13.26	23.79	1.792	2.146
SPBC577.03C	SPBC577.03c	0.9457	-0.169	0.024246611	14.77	26.47	1.65	2.141
SPAC1F12.02C	SPAC1F12.02c	0.9557	-0.1675	0.019678414	13.7	24.57	2.828	2.387
SPAC13G7.09C	SPAC13G7.09c	0.9738	-0.1667	0.01153023	15.39	27.57	1.745	4.603
SPAC1F7.07C	fip1	0.9524	-0.1583	0.021180613	16.6	29.69	1.963	2.246
SPBC1347.09	SPBC1347.09	0.9527	-0.1572	0.021043835	14.2	25.45	1.367	2.352
SPAC23C11.15	pst2	0.9433	-0.1543	0.025350166	13.43	24.07	1.289	1.901
SPAC26F1.01	sec74	0.9613	-0.1512	0.017141058	16.1	28.79	2.119	2.679
SPAC4G8.11C	atp10	0.9631	-0.1511	0.016328617	5.929	10.77	3.947	2.209
SPBP4H10.10	SPBP4H10.10	0.9643	-0.15	0.015787833	16.66	29.78	1.978	2.937
SPAC3C7.09	set8	0.9523	-0.1497	0.021226216	15.02	26.88	2.076	2.066
SPBC16C6.06	vps10	0.9398	-0.1485	0.026964559	12.61	22.6	0.9317	1.757

SPBC530.04	mod5	0.9668	-0.1483	0.014663358	11.82	21.2	2.753	2.997
SPACUNK4.13C	SPACUNK4.13c	0.9757	-0.1474	0.010683695	18.03	32.22	2.841	4.253
SPAPB2B4.03	cig2	0.9466	-0.1466	0.0238335	15.25	27.29	1.1	1.948
SPAC2C4.06C	SPAC2C4.06c	0.9709	-0.1455	0.012825499	14.77	26.44	1.64	3.598
SPBC1289.11	spf38	0.9802	-0.1434	0.008685302	17.18	30.7	3.183	5.104
SPAC1639.02C	trk2	0.948	-0.1406	0.023191663	2.216	4.176	2.736	1.451
SPCC126.11C	SPCC126.11c	0.9341	-0.139	0.029606628	14.55	26.04	1.017	1.468
SPBC947.02	apl2	0.9805	-0.1384	0.008552402	17.6	31.44	2.964	5.016
SPBC21.03C	SPBC21.03c	0.9512	-0.1382	0.021728158	10.12	18.17	2.138	1.785
SPBC365.03C	rpl2101	0.9498	-0.1369	0.022367835	2.266	4.259	2.707	1.49
SPBC83.09C	SPBC83.09c	0.9585	-0.133	0.018407883	15.19	27.15	1.69	2.21
SPAC1002.06C	bqt2	0.9795	-0.133	0.00899556	18.59	33.17	2.533	4.616
SPAC2G11.03C	vps45	0.9223	-0.1327	0.035127791	9.767	17.54	0.7054	1.206
SPBC13E7.09	vrp1	0.9585	-0.1316	0.018407883	13.72	24.55	0.7987	2.298
SPAC8F11.09C	nnt1	0.9764	-0.1301	0.010372229	16.64	29.71	1.705	3.976
SPBC776.14	plh1	0.9735	-0.1299	0.011664044	15.29	27.32	1.666	3.513
SPBC83.16C	SPBC83.16c	0.9521	-0.1256	0.021317435	15.97	28.52	2.959	1.087
SPAPB24D3.04C	mag1	0.9419	-0.1252	0.025995203	14.7	26.28	1.612	1.364
SPAC14C4.01C	SPAC14C4.01c	0.9538	-0.1247	0.020542682	15	26.8	1.619	1.816
SPBC839.04	rpl803	0.956	-0.1175	0.019542108	14.06	25.13	1.959	1.7
SPAC25B8.15C	SPAC25B8.15c	0.9809	-0.1174	0.008375265	17.76	31.68	2.753	4.317
SPAC2F7.04	pmc2	0.8698	-0.1144	0.060580597	0.589	1.247	0.6097	0.589
SPAC13G7.02C	ssa1	0.9862	-0.1084	0.006035002	16.46	29.36	5.161	5.204
SPAC57A10.04	mug10	0.9692	-0.1074	0.013586595	16.33	29.14	2.158	2.33
SPBC3E7.09	SPBC3E7.09	0.9736	-0.1066	0.011619435	15.85	28.27	1.797	2.841
SPCC162.12	tco89	0.9772	-0.1065	0.010016542	7.81	14.03	5.371	1.824
SPBC27.04	uds1	0.9593	-0.1054	0.018045556	15.19	27.1	1.315	1.795
SPBC29A3.21	SPBC29A3.21	0.975	-0.105	0.010995384	15.7	28	4.703	1.769
SPAP27G11.02	SPAP27G11.02	0.9811	-0.1049	0.008286724	14.77	26.36	1.92	3.967
SPCC737.09C	hmt1	0.972	-0.1035	0.012333735	15.41	27.49	1.901	2.555
SPAC13G7.07	arb2	0.9825	-0.09825	0.007667441	15.14	27.01	2.335	3.963
SPAC12B10.07	acp1	0.9866	-0.09372	0.005858889	16.05	28.6	3.341	4.904
SPAC23D3.01	SPAC23D3.01	0.9842	-0.09314	0.006916639	16.92	30.14	1.727	4.242
SPAC1B2.04	cox6	0.8707	-0.09172	0.060131456	0.4381	0.939	0.5522	0.4381
SPBC1271.15C	SPBC1271.15c	0.9717	-0.09128	0.012467797	15.43	27.5	1.971	2.164
SPAC9E9.09C	SPAC9E9.09c	0.9768	-0.09053	0.010194349	5.972	10.74	3.933	2
SPBC1348.07	SPBC1348.07	0.9866	-0.08412	0.005858889	16.47	29.34	2.516	4.448
SPAC4H3.14C	SPAC4H3.14c	0.9888	-0.08406	0.004891542	14.76	26.31	7.975	3.588
SPBC2F12.12C	SPBC2F12.12c	0.9813	-0.08101	0.008198201	5.702	10.25	3.553	2.636
SPAC4G8.03C	SPAC4G8.03c	0.9757	-0.08025	0.010683695	18.21	32.42	1.216	2.354
SPACUNK12.02C	cmk1	0.979	-0.08024	0.009217308	17.65	31.42	1.528	2.709
SPAC15E1.05C	SPAC15E1.05c	0.9916	-0.07286	0.003663482	12.18	21.72	7.312	5.138
SPBC2G2.14	SPBC2G2.14	0.9877	-0.07248	0.005374946	14.95	26.62	2.494	4.156
SPBC365.14C	uge1	0.9879	-0.07051	0.005287015	8.71	15.56	5.254	3.308
SPAC823.13C	SPAC823.13c	0.9849	-0.07046	0.006607863	16.86	30.01	2.407	3.235
SPBC36B7.08C	SPBC36B7.08c	0.982	-0.06806	0.007888512	18.17	32.32	1.675	2.663
SPBC1348.02	SPBC1348.02	0.9815	-0.06436	0.008109696	10.98	19.57	3.624	1.692
SPAC11D3.06	SPAC11D3.06	0.9901	-0.06389	0.004320939	17	30.24	2.001	4.667
SPAC57A10.06	mug15	0.9245	-0.06357	0.034093085	15.91	28.31	0.5389	0.5577
SPBC119.14	rti1	0.9835	-0.06236	0.007225636	17.48	31.09	1.922	2.625
SPAC13F5.04C	SPAC13F5.04c	0.9848	-0.05935	0.00665196	16.49	29.33	2.238	2.656
SPCC1682.13	laf2	0.9796	-0.05876	0.008951224	14.76	26.26	1.273	2.032
SPAC1F3.03	SPAC1F3.03	0.9848	-0.05599	0.00665196	10.74	19.14	3.915	1.748
SPBC651.07	EMPTY	0.9925	-0.05532	0.003269485	16.16	28.73	2.529	5.316
SPBC1778.04	spo6	0.9842	-0.05464	0.006916639	16.81	29.88	1.815	2.391
SPAC521.03	SPAC521.03	0.965	-0.05004	0.015472687	16.12	28.65	0.6324	1.006
SPBP35G2.03C	sgo1	0.9868	-0.04974	0.005770859	15.89	28.25	2.085	2.579
SPCC736.08	cbf11	0.9829	-0.04667	0.007490665	14.69	26.12	1.238	1.912
SPBC21C3.06	SPBC21C3.06	0.9762	-0.04569	0.010461197	14.12	25.11	1.499	1.195
SPBC8E4.04	SPBC8E4.04	0.9893	-0.04563	0.004671991	14.94	26.56	1.603	3.037
SPCC5E4.05C	SPCC5E4.05c	0.9836	-0.0447	0.00718148	14.75	26.22	1.793	1.803
SPCC1223.03C	gut2	0.9899	-0.04452	0.004408676	15.79	28.06	1.138	3.211
SPBC24C6.06	gpa1	0.9921	-0.04436	0.00344455	15.66	27.82	5.664	3.048
SPAC1039.05C	klf1	0.9878	-0.04187	0.005330978	14.41	25.62	1.43	2.426
SPAC14C4.14	atp1	0.9899	-0.04184	0.004408676	6.346	11.32	3.332	2.528
SPBC25H2.11C	spt7	0.3506	-0.04142	0.455188088	7.93E-18	0.0734	0.0734	7.93E-18
SPBC887.06C	snx3	0.9854	-0.0402	0.006387442	16.82	29.89	1.169	1.951
SPAPB2B4.07	SPAPB2B4.07	0.9908	-0.0392	0.004014002	11.85	21.07	3.255	2.674
SPBC106.19	SPBC106.19	0.9926	-0.03497	0.003225729	14.13	25.11	3.158	3.112

SPACUNK4.14	mdb1	0.9937	-0.03356	0.00274471	16.94	30.09	2.317	3.727
SPCC1450.07C	SPCC1450.07c	0.9911	-0.03024	0.003882524	15.55	27.61	1.676	2.363
SPBC1709.10C	atx1	0.9915	-0.02979	0.003707281	16.4	29.11	2.874	2.137
SPBP23A10.14C	ell1	0.9954	-0.02922	0.002002364	14.88	26.41	2.936	4.474
SPCC663.12	cid12	0.9953	-0.02661	0.002045996	11	19.53	5.702	2.907
SPAPB8E5.05	mfm1	0.9942	-0.02656	0.002526241	16.72	29.68	2.583	3.129
SPCC2H8.05C	SPCC2H8.05c	0.9944	-0.02076	0.002438884	14.88	26.4	1.755	2.603
SPAC22H10.08	SPAC22H10.08	0.9952	-0.01871	0.002089633	14.5	25.73	1.276	2.831
SPAC4F10.07C	atg13	0.9962	-0.0178	0.001653463	11.98	21.26	2.477	3.215
SPBC713.05	SPBC713.05	0.9939	-0.01659	0.002657309	14.1	25.02	1.346	1.893
SPCC1795.01C	mad3	0.9949	-0.01309	0.002220569	13.22	23.46	1.954	1.609
SPAC22F8.04	SPAC22F8.04	0.9972	-0.01162	0.00121773	8.945	15.87	0.999	2.977
SPAC23H4.01C	SPAC23H4.01c	0.9985	-0.01037	0.000651931	15.27	27.08	2.648	4.882
SPAC8E11.05C	SPAC8E11.05c	0.9987	-0.008998	0.00056495	9.98	17.7	5.913	3.878
SPCC1281.04	SPCC1281.04	0.9981	-0.00766	0.000825944	12.82	22.74	1.941	2.828
SPAC25B8.10	SPAC25B8.10	0.9973	-0.006242	0.001174181	15.79	28	0.989	1.622
SPAC17G8.14C	pck1	0.9992	-0.00542	0.000347575	16.05	28.45	3.028	5.048
SPBP35G2.08C	air1	1	0	0	0	0	0	0
SPBC25D12.02C	dnt1	1	0	0	0	0	0	0
SPAC12G12.13C	cid14	1	0	0	0	0	0	0
SPBC21C3.16C	spt4	1	0	0	0	0	0	0
SPBC11C11.09C	rpl502	1	0	0	0	0	0	0
SPBC685.07C	rpl2701	1	0	0	0	0	0	0
SPBC1685.07C	avt5	1	0	0	0	0	0	0
SPBC21C3.08C	car2	1	0	0	0	0	0	0
SPAC27E2.11C	SPAC27E2.11c	1	0	0	0	0	0	0
SPBC25D12.06	SPBC25D12.06	0.3506	6.51E-18	0.455188088	6.51E-18	0	0	6.51E-18
SPBC800.11	SPBC800.11	0.9997	0.0008978	0.000130308	16.21	28.73	1.998	2.354
SPAC17G8.11C	imt3	0.9986	0.001417	0.000608438	16.95	30.04	1.216	0.2722
SPBC27B12.10C	tom7	0.9996	0.001789	0.000173753	7.119	12.61	4.517	2.476
SPAC23H3.11C	SPAC23H3.11c	0.9994	0.002277	0.000260655	15.49	27.45	1.698	2.69
SPAC23H3.09C	gly1	0.9979	0.008434	0.000912977	6.331	11.2	3.778	2.214
SPBC16A3.06	SPBC16A3.06	0.9971	0.01029	0.001261284	13.94	24.69	3.357	2.007
SPAC6C3.02C	SPAC6C3.02c	0.9971	0.01097	0.001261284	18.65	33.02	1.341	2.709
SPCC126.06	twf1	0.9967	0.01169	0.001435542	12.87	22.79	2.204	2.373
SPBC3D6.05	ptp4	0.9955	0.01235	0.001958736	10.65	18.86	3.487	0.5572
SPBC3H7.07C	ser2	0.9946	0.01592	0.002351545	15.5	27.44	1.625	2.039
SPAC16A10.07C	taz1	0.9971	0.01612	0.001261284	16.66	29.5	1.996	4.028
SPBC16G5.05C	SPBC16G5.05c	0.9952	0.01854	0.002089633	11.48	20.31	3.909	1.956
SPBC725.01	SPBC725.01	0.9913	0.019	0.003794894	4.012	7.076	1.72	1.36
SPAC23D3.10C	eng2	0.9953	0.01923	0.002045996	15.63	27.67	3.23	2.505
SPBC800.10C	SPBC800.10c	0.9922	0.01975	0.003400777	14.56	25.77	1.627	1.682
SPAC1556.05C	cgr1	0.9955	0.02233	0.001958736	13.77	24.36	4.098	2.975
SPAC24H6.03	cul3	0.9957	0.02439	0.001871493	15.24	26.97	2.301	4.033
SPAC29B12.08	SPAC29B12.08	0.9912	0.02708	0.003838707	10.21	18.04	3.597	1.164
SPAC4F10.17	SPAC4F10.17	0.9923	0.02803	0.003357009	16.05	28.39	1.977	2.501
SPBP4H10.04	ppb1	0.993	0.02972	0.003050752	16.43	29.07	1.276	3.072
SPCC1450.05C	rox3	0.995	0.03257	0.002176919	16.04	28.37	3.755	4.468
SPBP35G2.02	SPBP35G2.02	0.9949	0.03731	0.002220569	15.93	28.17	2.23	5.241
SPBC6B1.04	mde4	0.9895	0.03938	0.004584201	5.797	10.2	2.146	2.554
SPCC5E4.10C	SPCC5E4.10c	0.9917	0.04046	0.003619687	15.98	28.24	2.61	3.351
SPAC922.07C	SPAC922.07c	0.9904	0.04056	0.004189368	13.81	24.4	1.944	2.971
SPAC25G10.05C	his1	0.9805	0.0416	0.008552402	8.696	15.34	1.616	1.346
SPAC1834.10C	SPAC1834.10c	0.9834	0.04354	0.007269796	13	22.97	2.166	1.591
SPAC22F3.12C	rgs1	0.9943	0.04579	0.002482561	12.21	21.56	7.512	4.466
SPAC25G10.03	zip1	0.9873	0.04733	0.005550863	6.659	11.72	2.137	2.527
SPCC1795.06	map2	0.9895	0.04947	0.004584201	14.92	26.34	5.723	1.458
SPAC977.12	SPAC977.12	0.9785	0.04977	0.00943917	10.23	18.05	2.224	1.251
SPCC338.08	ctp1	0.9906	0.05078	0.004101676	5.011	8.79	5.172	2.908
SPAC1142.03C	swi2	0.9761	0.05204	0.010505687	14.86	26.24	1.739	1.335
SPAC637.09	SPAC637.09	0.9899	0.05538	0.004408676	16.34	28.86	1.774	3.925
SPCC11E10.08	rik1	0.9912	0.05705	0.003838707	9.591	16.9	6.127	3.563
SPCC1827.08C	pof7	0.9857	0.05957	0.006255243	16.54	29.2	2.239	2.863
SPBC29A10.06C	ely5	0.9733	0.06109	0.011753277	15.61	27.56	1.837	1.405
SPAC9E9.05	SPAC9E9.05	0.9829	0.06156	0.007490665	15.75	27.81	0.7632	2.615
SPBC119.12	SPBC119.12	0.9877	0.06541	0.005374946	13.47	23.76	2.435	3.729
SPAC9.11	SPAC9.11	0.9845	0.06581	0.00678428	12.36	21.79	0.8472	3.099
SPAC27D7.08C	SPAC27D7.08c	0.9709	0.06635	0.012825499	14.28	25.19	1.247	1.565
SPBC1105.10	rav1	0.391	0.06661	0.407823243	0.06661	0	0	0.06661

SPAC8E11.07C	alp31	0.9906	0.06671	0.004101676	10.68	18.81	6.38	4.063
SPAC10F6.17C	SPAC10F6.17c	0.977	0.06917	0.010105436	15.63	27.58	0.8921	2.171
SPBC3B8.04C	SPBC3B8.04c	0.9864	0.07183	0.005946936	18.17	32.08	2.527	3.693
SPAC56F8.09	rrp8	0.9805	0.07208	0.008552402	14.9	26.27	3.617	1.964
SPBC776.01	rpl29	0.9796	0.07219	0.008951224	14.13	24.91	3.922	1.54
SPBC27.02C	ask1	0.9826	0.07384	0.00762324	16.66	29.4	1.666	3.01
SPBC3D6.15	rps2501	0.9852	0.07558	0.006475597	12.07	21.26	4.879	2.915
SPAC3G9.01	nsk1	0.9793	0.07847	0.009084245	15.11	26.64	3.441	2.152
SPCC24B10.07	gad8	0.391	0.07889	0.407823243	0.07889	0	0	0.07889
SPCC1259.02C	SPCC1259.02c	0.9848	0.07972	0.00665196	16.98	29.95	2.017	3.737
SPAC31G5.09C	spk1	0.9693	0.08144	0.013541787	15.22	26.83	2.825	1.33
SPAC1783.07C	pap1	0.9273	0.08163	0.03277974	7.929	13.91	0.6186	0.7667
SPAC1782.06C	phb1	0.9537	0.08406	0.020588217	11.63	20.46	1.003	1.243
SPAC22E12.06C	gmh3	0.9744	0.08428	0.011262725	16.01	28.23	2.338	2.127
SPBC21D10.10	bdc1	0.9828	0.08711	0.007534852	12.59	22.16	1.995	3.595
SPCC622.17	apn1	0.9543	0.08957	0.020315076	15.71	27.67	1.286	1.293
SPBC1711.06	rpl401	0.975	0.08985	0.010995384	16.39	28.88	2.255	2.395
SPBPJ4664.03	mfm3	0.9694	0.09029	0.013496985	16.87	29.74	2.188	1.877
SPBC1198.14C	fbp1	0.9862	0.09073	0.006035002	16.06	28.3	3.031	4.627
SPBC19G7.16	iws1	0.9724	0.09126	0.01215505	6.584	11.51	2.95	1.981
SPAC1327.01C	SPAC1327.01c	0.9698	0.0914	0.01331782	12.73	22.41	1.709	2.062
SPAC25B8.06C	SPAC25B8.06c	0.9806	0.09543	0.008508111	4.202	7.278	4.768	2.756
SPAC1039.06	SPAC1039.06	0.9669	0.1005	0.01461844	14.33	25.23	2.509	1.835
SPCC594.04C	SPCC594.04c	0.9863	0.1031	0.005990967	11.9	20.91	7.348	4.004
SPAC16E8.14C	tae1	0.9735	0.1036	0.011664044	14.43	25.39	1.572	2.769
SPAC30C2.07	SPAC30C2.07	0.9842	0.1044	0.006916639	12.8	22.5	5.404	4.019
SPAC23H4.08	iwr1	0.9798	0.1047	0.008862565	11.36	19.94	3.252	3.462
SPBC1289.09	tim21	0.9504	0.1074	0.022093572	14.86	26.14	1.959	1.232
SPBC23G7.16	ctr6	0.9755	0.1096	0.010772726	10.1	17.71	1.408	3.217
SPBP16F5.08C	SPBP16F5.08c	0.9436	0.1097	0.025212068	15.13	26.62	0.6088	1.398
SPAC17G6.04C	cpp1	0.9637	0.1124	0.016058141	12.06	21.17	1.553	2.146
SPAC688.12C	SPAC688.12c	0.9699	0.113	0.013273041	16.58	29.17	2.814	2.375
SPCC338.16	pof3	0.9752	0.1139	0.010906307	6.989	12.18	4.451	2.459
SPBC106.20	exo70	0.9648	0.1167	0.015562705	16.26	28.61	1.715	2.286
SPAC31G5.03	rps1101	0.9274	0.118	0.032732908	0.9339	1.446	1.446	0.9339
SPCC1840.02C	bgs4	0.9741	0.1181	0.011396457	17.98	31.65	2.559	3.109
SPAC3A12.12	atp11	0.9434	0.1201	0.025304128	9.34	16.34	1.833	1.248
SPCC1393.05	ers1	0.9675	0.1231	0.014349026	14.42	25.34	2.172	2.573
SPAC30C2.04	SPAC30C2.04	0.9514	0.124	0.021636853	13.36	23.46	1.614	1.697
SPBC2D10.17	clr1	0.9767	0.1243	0.010238812	16.53	29.08	1.51	3.857
SPCP31B10.05	tdp1	0.9777	0.1248	0.009794385	11.58	20.31	6.639	1.933
SPBC1683.08	ght4	0.9804	0.1263	0.008596697	15.18	26.67	3.464	4.428
SPBC582.10C	SPBC582.10c	0.9613	0.1291	0.017141058	14.98	26.33	2.961	1.925
SPBC21C3.01C	vps13a	0.9727	0.131	0.012021084	13.81	24.24	3.931	2.914
SPAC12B10.05	icp55	0.9535	0.1329	0.020679303	16.57	29.13	1.043	2.04
SPCC16C4.12	naa20	0.9834	0.133	0.007269796	10.79	18.89	8.33	3.944
SPBC14F5.11C	mug186	0.9648	0.1379	0.015562705	17.43	30.64	0.9147	2.847
SPAC4G9.09C	arg11	0.9399	0.1397	0.02691835	7.122	12.37	0.8344	1.66
SPBC342.06C	rtt109	0.9582	0.1451	0.018543833	15.35	26.94	1.751	2.409
SPBC13G1.08C	ash2	0.93	0.1461	0.031517051	14.14	24.8	1.68	1.277
SPCC4F11.04C	imt2	0.9802	0.1465	0.008685302	8.105	14.1	8.143	3.242
SPAC13G6.14	aps1	0.9643	0.1502	0.015787833	6.334	10.96	3.712	2.436
SPAC2F3.08	sut1	0.966	0.1536	0.015022874	16.83	29.56	1.827	3.205
SPAC29B12.02C	set2	0.971	0.1562	0.01278077	18.02	31.67	1.351	3.904
SPAPB18E9.04C	SPAPB18E9.04c	0.9409	0.1569	0.026456531	16	28.07	2.027	1.664
SPBP8B7.13	vac7	0.9725	0.157	0.01211039	15.38	26.98	2.469	4.022
SPBC1734.11	mas5	0.9661	0.157	0.014977918	4.327	7.39	4.451	2.504
SPCC757.04	SPCC757.04	0.9691	0.1572	0.013631406	15.08	26.44	2.29	3.57
SPBC582.06C	mcp6	0.9826	0.1572	0.00762324	13.01	22.77	9.283	4.531
SPBC14C8.15	SPBC14C8.15	0.9712	0.1589	0.012691326	13.89	24.34	2.699	3.835
SPBC725.04	SPBC725.04	0.952	0.1602	0.021363052	14.43	25.28	2.027	2.244
SPCC594.02C	SPCC594.02c	0.9669	0.1607	0.01461844	7.429	12.88	4.493	2.713
SPBC1D7.04	mlo3	0.9643	0.1633	0.015787833	7.235	12.53	4.234	2.553
SPAC926.02	SPAC926.02	0.8805	0.1686	0.05527064	14.3	25.05	1.087	0.8767
SPCC338.05C	mms2	0.9813	0.1699	0.008198201	13.39	23.43	9.139	4.655
SPCC1259.08	SPCC1259.08	0.9585	0.1701	0.018407883	15.29	26.79	3.277	2.516
SPBC17D11.01	nep1	0.9806	0.1741	0.008508111	19.57	34.38	3.63	6.353
SPBC16H5.09C	omh2	0.8912	0.1748	0.050024822	13.15	22.99	1.329	0.9642
SPCC285.16C	msh6	0.954	0.1794	0.020451625	16.6	29.11	1.592	2.765

SPAC3F10.18C	rpl4102	0.9428	0.1794	0.025580426	12.92	22.58	3.683	1.142
SPAC3F10.16C	SPAC3F10.16c	0.7662	0.1817	0.115657852	0.5032	0.5698	0.5698	0.5032
SPBC1685.01	pmp1	0.9216	0.1834	0.035457534	14.46	25.29	1.545	1.539
SPAC2F3.07C	SPAC2F3.07c	0.9505	0.1839	0.022047879	15.95	27.93	1.961	2.561
SPBP8B7.24C	atg8	0.9567	0.1862	0.019224226	15.81	27.68	3.893	2.45
SPCC790.02	pep3	0.9812	0.1888	0.00824246	9.581	16.64	9.663	5.399
SPAC1527.03	SPAC1527.03	0.9379	0.1903	0.027843464	16.86	29.54	2.354	1.914
SPAPB24D3.03	SPAPB24D3.03	0.9769	0.1929	0.01014989	19.1	33.5	4.293	5.788
SPAC644.11C	pkp1	0.9126	0.1966	0.039719536	12.09	21.07	1.705	1.412
SPBC56F2.09C	arg5	0.9536	0.1997	0.020633758	4.112	6.933	4.025	2.382
SPBC19G7.03C	rps3002	0.9638	0.2018	0.016013078	19.24	33.75	2.624	3.899
SPAC227.03C	SPAC227.03c	0.9664	0.2059	0.014843079	18.41	32.25	2.222	4.383
SPBC21D10.08C	SPBC21D10.08c	0.9341	0.2072	0.029606628	14.42	25.18	2.108	2.06
SPAC15A10.03C	rhp54	0.9641	0.2082	0.015877917	8.307	14.35	5.051	3.391
SPCC1393.09C	SPCC1393.09c	0.93	0.2123	0.031517051	16.07	28.1	1.617	2.085
SPAPYUK71.03C	syn1	0.9464	0.213	0.023925268	13.84	24.15	4.676	1.434
SPAC959.08	rpl2102	0.9385	0.2169	0.027565723	14.05	24.51	4.154	1.269
SPAPJ696.01C	vps17	0.9625	0.2172	0.016599262	8.44	14.57	4.879	3.465
SPAC6F6.11C	SPAC6F6.11c	0.963	0.2174	0.016373713	16.69	29.2	2.038	4.204
SPAC9.02C	SPAC9.02c	0.9545	0.2208	0.020224067	9.649	16.71	1.753	3.464
SPAC15E1.02C	SPAC15E1.02c	0.9711	0.2251	0.012736046	12.16	21.15	7.592	4.15
SPAC56E4.03	SPAC56E4.03	0.913	0.2261	0.039529222	6.426	10.99	2.966	1.034
SPCC162.03	SPCC162.03	0.9343	0.23	0.029513651	15.94	27.83	1.9	2.399
SPAC3F10.11C	abc2	0.9292	0.2307	0.031890799	14.93	26.05	2.302	2.101
SPBC409.19C	SPBC409.19c	0.9426	0.2316	0.025672565	5.78	9.833	3.436	2.393
SPBC119.04	mei3	0.9151	0.2326	0.038531445	12.55	21.84	1.97	1.755
SPCC126.13C	SPCC126.13c	0.9204	0.2336	0.03602339	15.15	26.43	0.7865	2.119
SPBC24C6.08C	bhd1	0.9248	0.2344	0.033952179	16.33	28.52	1.768	2.12
SPBC660.11	tcg1	0.8209	0.2358	0.085709744	0.8098	1.017	1.017	0.8098
SPBC16A3.08C	SPBC16A3.08c	0.9481	0.2371	0.023145853	6.966	11.92	4.419	2.45
SPBC16C6.02C	vps1302	0.9269	0.2381	0.032967118	7.572	13	2.936	1.934
SPAC19G12.04	SPAC19G12.04	0.94	0.2424	0.026872146	13.6	23.67	2.181	2.773
SPCC584.02	cuf2	0.9471	0.252	0.023604163	14.31	24.92	0.9372	3.47
SPCC663.04	rpl39	0.9011	0.254	0.04522701	2.505	3.989	2.308	1.463
SPBC30B4.08	eri1	0.8847	0.2541	0.053203973	12.82	22.27	0.4576	1.597
SPAC19A8.03	SPAC19A8.03	0.8949	0.2607	0.048225492	11.5	19.92	1.114	1.735
SPAC1142.06	get3	0.9318	0.2637	0.030677294	17.15	29.92	2.584	2.534
SPAC6G10.11C	ubi3	0.9322	0.2666	0.030490901	17.21	30.03	2.21	2.677
SPBP4H10.17C	SPBP4H10.17c	0.92	0.2709	0.036212173	15.36	26.74	2.237	2.225
SPAC1952.16	rga9	0.9537	0.2713	0.020588217	17.31	30.2	3.649	3.914
SPAC17H9.09C	ras1	0.9364	0.2747	0.028538595	10.74	18.54	3.768	2.627
SPAC890.06	nup155	0.9049	0.2792	0.043399412	16.08	28	2.241	1.835
SPBC1198.01	SPBC1198.01	0.9169	0.2831	0.037678027	13.64	23.67	1.076	2.446
SPBC1709.16C	SPBC1709.16c	0.9319	0.2857	0.030630688	16.42	28.58	4.71	1.76
SPAC824.02	SPAC824.02	0.9495	0.2869	0.022505031	10.37	17.86	5.234	3.187
SPBC1861.03	mak10	0.9365	0.2912	0.028492218	14.02	24.32	0.6721	3.349
SPBC8E4.03	SPBC8E4.03	0.9188	0.2913	0.036779013	14.92	25.92	1.847	2.477
SPAC13G7.05	are1	0.9032	0.2918	0.044216071	13.94	24.19	2.945	1.591
SPAC6F12.09	rdp1	0.9298	0.2947	0.031610458	15.9	27.66	1.185	3.03
SPAC11D3.09	SPAC11D3.09	0.9733	0.2991	0.011753277	18.66	32.54	6.217	7.649
SPBC776.17	SPBC776.17	0.9416	0.3003	0.02613355	7.957	13.57	4.736	2.88
SPAC13G6.08	SPAC13G6.08	0.9329	0.3006	0.030164907	15.67	27.23	1.955	3.152
SPAC140.02	gar2	0.9261	0.3021	0.033342116	15.49	26.92	3.483	2.418
SPAC23D3.13C	SPAC23D3.13c	0.8601	0.3021	0.065451052	15.26	26.51	1.457	1.403
SPAC630.06C	SPAC630.06c	0.9115	0.3028	0.040243327	14.2	24.63	1.708	2.368
SPBC83.18C	fic1	0.9114	0.3041	0.040290976	15.23	26.46	2.829	2.069
SPBP8B7.27	mug30	0.9515	0.3052	0.021591207	16.66	28.98	2.624	4.446
SPBC4C3.08	mug136	0.8707	0.3063	0.060131456	16.26	28.27	1.479	1.572
SPAC7D4.05	SPAC7D4.05	0.9484	0.309	0.023008455	17.43	30.34	1.626	4.332
SPBC23G7.10C	SPBC23G7.10c	0.9027	0.3091	0.044456558	15.49	26.91	2.072	2.092
SPCP1E11.04C	pal1	0.9358	0.3126	0.028816959	9.706	16.65	4.421	2.758
SPBC25H2.14	mug16	0.9416	0.3128	0.02613355	12.18	21.03	6.211	2.041
SPBC32F12.01C	css1	0.9577	0.3143	0.018770513	11.92	20.57	7.105	4.03
SPBC839.03C	SPBC839.03c	0.9202	0.3175	0.036117771	16.24	28.21	1.69	2.806
SPAC22E12.19	snt1	0.8134	0.3199	0.089695832	14.84	25.73	0.9768	1.15
SPAC1556.04C	cdd1	0.959	0.3227	0.018181393	16.24	28.22	2.468	5.669
SPBC354.13	rga6	0.9499	0.326	0.022322112	17.32	30.12	4.638	4.19
SPBC1652.02	SPBC1652.02	0.8841	0.3283	0.053498609	13.29	22.98	1.265	1.981
SPAC1A6.09C	lag1	0.9461	0.3317	0.024062958	14.81	25.66	4.556	3.906

SPAC56F8.04C	ppt1	0.9258	0.3328	0.033482824	6.752	11.38	3.855	2.644
SPAC6F6.13C	SPAC6F6.13c	0.9017	0.3368	0.04493793	18.4	32.01	1.593	2.39
SPAC664.12C	SPAC664.12c	0.9398	0.3453	0.026964559	18.13	31.52	2.236	4.076
SPAC3A11.07	SPAC3A11.07	0.9447	0.3469	0.024706085	9.982	17.07	5.834	3.488
SPBC13G1.14C	SPBC13G1.14c	0.9615	0.3486	0.017050711	13.94	24.09	8.092	5.202
SPBC17D11.02C	hrd1	0.9037	0.3497	0.043975718	17.69	30.73	1.797	2.516
SPBC32H8.06	mug93	0.8942	0.3497	0.048565334	15.19	26.31	2.402	2.104
SPAC29B12.13	SPAC29B12.13	0.8901	0.3498	0.050561199	16.02	27.77	0.7897	2.299
SPBC15D4.15	pho2	0.8808	0.354	0.055122694	17.2	29.85	1.583	2.033
SPAC6B12.04C	SPAC6B12.04c	0.8773	0.355	0.056851871	15.41	26.68	2.046	1.855
SPAC26H5.03	pcf2	0.9432	0.3555	0.025396208	15.5	26.85	2.484	4.444
SPCC1753.02C	git3	0.8759	0.3558	0.057545474	14.73	25.47	1.665	1.933
SPAC1A6.10	SPAC1A6.10	0.9127	0.3607	0.039671949	13.64	23.53	2.962	2.647
SPBC1271.07C	SPBC1271.07c	0.8986	0.3625	0.046433586	17.58	30.5	1.904	2.452
SPAC30D11.10	rad22	0.7763	0.3627	0.109970414	1.049	1.215	1.056	1.049
SPBP4H10.14C	SPBP4H10.14c	0.8955	0.3636	0.04793441	15.27	26.42	1.464	2.45
SPBC530.09C	SPBC530.09c	0.9061	0.3639	0.04282387	15.02	25.98	1.745	2.712
SPBC25B2.06C	btb2	0.8852	0.3647	0.052958595	16	27.71	0.8301	2.289
SPCC31H12.02C	mug73	0.9417	0.3659	0.02608743	15.89	27.51	2.453	4.455
SPBC776.04	sec2302	0.8777	0.366	0.056653902	15.33	26.52	1.951	1.966
SPAC3H8.03	img2	0.8899	0.3706	0.050658793	13.06	22.48	1.68	2.327
SPBP4H10.11C	lcf2	0.9333	0.3708	0.029978734	16.01	27.72	2.922	3.828
SPAC227.01C	SPAC227.01c	0.8789	0.3716	0.056060536	15.67	27.1	1.463	2.129
SPBC1685.10	rps27	0.8505	0.373	0.070325682	17	29.46	1.805	1.582
SPBCPT2R1.01C	SPBCPT2R1.01c	0.9139	0.3749	0.039101323	13.03	22.42	2.457	2.961
SPAC6B12.16	meu26	0.848	0.3762	0.071604148	14.5	25.03	1.213	1.706
SPBC725.05C	SPBC725.05c	0.9444	0.3793	0.024844022	15.73	27.2	2.711	4.841
SPAC4D7.10C	spt20	0.2018	0.3814	0.695078838	0.3814	0	0	0.2707
SPBC18H10.15	ppk23	0.9407	0.3842	0.026548856	16.65	28.82	2.752	4.572
SPBC14C8.11C	SPBC14C8.11c	0.8844	0.3858	0.053351266	15.9	27.48	0.8176	2.411
SPAPB1A10.09	ase1	0.9296	0.3864	0.031703885	16.24	28.1	2.816	3.79
SPBC530.10C	anc1	0.8425	0.3868	0.07443009	13.37	23.01	1.013	1.722
SPBP8B7.11	nxt3	0.8049	0.3894	0.094258073	16.27	28.14	1.987	1.008
SPAC16E8.08	SPAC16E8.08	0.8857	0.3938	0.052713355	14.22	24.5	1.936	2.338
SPBC947.14C	cbp6	0.9309	0.3992	0.03109697	6.313	10.48	4.27	3.667
SPAC25B8.01	dap1	0.8493	0.3999	0.070938876	15.52	26.8	3.183	0.8354
SPAC222.12C	atp2	0.8629	0.4007	0.064039531	14.46	24.92	2.455	1.734
SPCC1919.05	SPCC1919.05	0.9373	0.4008	0.028121383	9.998	17.01	6.13	3.457
SPAC17C9.09C	tim13	0.8034	0.4021	0.095068173	14.44	24.87	1.621	1.238
SPCP31B10.06	mug190	0.9028	0.4022	0.04440845	18.5	32.07	1.43	2.952
SPCC965.05C	thp1	0.7938	0.4038	0.100288905	15.67	27.06	1.906	1.012
SPBC16C6.11	rpl3201	0.9495	0.4077	0.022505031	7.767	13.04	7.54	4.467
SPAC513.03	mfm2	0.9188	0.4079	0.036779013	10.23	17.41	2.856	3.413
SPCC830.06	SPCC830.06	0.9232	0.409	0.034704204	10.01	17.01	5.679	2.489
SPBC2A9.03	SPBC2A9.03	0.8858	0.4096	0.052664324	16.58	28.66	2.068	2.422
SPBC21B10.13C	yox1	0.7924	0.4096	0.101055533	8.761	14.8	2.199	0.8019
SPCC13B11.04C	SPCC13B11.04c	0.8868	0.4108	0.052174316	15.16	26.13	0.4267	2.646
SPBC19F8.01C	spn7	0.9294	0.4128	0.031797332	11.06	18.87	5.23	3.348
SPBC18H10.02	lcf1	0.8598	0.414	0.065602559	9.102	15.4	2.833	1.656
SPAC22H12.01C	mug35	0.8773	0.4146	0.056851871	14.18	24.39	0.9355	2.429
SPAC13A11.03	mcp7	0.8565	0.417	0.067272633	15.75	27.17	1.417	2.007
SPAC1782.04	cox24	0.9355	0.4175	0.028956208	9.329	15.79	5.312	3.924
SPBC28F2.02	mep33	0.9453	0.419	0.024430342	20.05	34.79	3.862	5.313
SPBC29A3.12	rps902	0.9463	0.4196	0.02397116	13.01	22.31	6.45	4.715
SPAPB8E5.02C	rpn502	0.8971	0.4217	0.047159143	14.48	24.91	2.176	2.809
SPAC15E1.04	SPAC15E1.04	0.9238	0.4233	0.034422042	8.512	14.33	5.4	2.951
SPAC1851.04C	ric1	0.8399	0.4279	0.075772419	5.035	8.165	2.452	1.551
SPBC887.08	SPBC887.08	0.8833	0.4286	0.05389177	13.09	22.44	2.338	2.43
SPBC4C3.04C	SPBC4C3.04c	0.8524	0.4297	0.069356559	16.22	27.99	0.6599	2.098
SPAC4G9.11C	cmb1	0.9094	0.4307	0.04124505	15.94	27.49	0.6599	3.47
SPAC139.05	SPAC139.05	0.8547	0.4314	0.068186296	15.98	27.55	1.439	2.052
SPBC1604.02C	ppr1	0.8482	0.4328	0.071501732	3.323	5.121	2.633	1.648
SPAC23C11.02C	rps23	0.9178	0.4357	0.037251947	11.31	19.28	5.566	2.542
SPCC364.07	SPCC364.07	0.882	0.4368	0.054531415	13.5	23.15	2.287	2.465
SPAC806.04C	SPAC806.04c	0.9479	0.4455	0.023237477	18.28	31.6	3.642	6.036
SPAC14C4.12C	laf1	0.8213	0.4456	0.085498177	11.64	19.83	0.4463	1.798
SPAC23C4.03	hrk1	0.9158	0.4478	0.038199361	11.15	18.97	6.392	1.714
SPAC227.15	SPAC227.15	0.9244	0.4487	0.034140063	18.35	31.72	4.056	3.862
SPAC19D5.06C	din1	0.8668	0.4491	0.062081097	15.11	25.99	3.028	1.913

SPAC1F7.13C	rpl801	0.7179	0.4496	0.143936047	2.406	3.467	1.394	0.9313
SPBC1711.15C	SPBC1711.15c	0.8242	0.4509	0.08396739	15.85	27.29	1.233	1.766
SPAC19A8.10	rfp1	0.8719	0.4557	0.059533322	16.3	28.08	2.159	2.376
SPAC10F6.05C	ubc6	0.9331	0.4568	0.030071811	17.1	29.49	2.634	4.852
SPBC713.09	SPBC713.09	0.8733	0.4568	0.05883654	16.96	29.24	1.876	2.474
SPAC27D7.09C	SPAC27D7.09c	0.9424	0.4578	0.025764723	19.21	33.23	2.871	5.67
SPAC12B10.04	SPAC12B10.04	0.9168	0.458	0.037725395	14.31	24.55	6.639	1.739
SPCC24B10.21	tpi1	0.7494	0.4598	0.125286311	16.45	28.34	0.6055	1.284
SPAC8C9.08	rps5	0.8516	0.4599	0.069764347	15.16	26.04	2.33	1.937
SPBC428.14	SPBC428.14	0.905	0.4606	0.043351421	18.15	31.35	2.916	3.252
SPAC1B3.17	clr2	0.9197	0.4642	0.036353814	18.08	31.22	1.717	4.158
SPAC806.08C	mod21	0.8362	0.4656	0.077689837	13.75	23.55	2.01	1.813
SPAC12G12.01C	SPAC12G12.01c	0.8905	0.4696	0.050366076	15.01	25.77	1.724	3.027
SPAC688.06C	slx4	0.6345	0.4725	0.197568374	17.52	30.21	1.567	0.1849
SPAC3G6.06C	rad2	0.8848	0.4738	0.053154886	14.72	25.25	2.584	2.73
SPCC1235.05C	fft2	0.932	0.4742	0.030584088	15.03	25.79	2.996	4.915
SPBPJ4664.06	gpt1	0.9298	0.475	0.031610458	18	31.06	3.94	4.58
SPBC1921.05	ape2	0.8532	0.4773	0.068949153	14.23	24.37	2.273	2.087
SPAC13G6.09	SPAC13G6.09	0.8649	0.4779	0.063034103	6.729	11.08	3.297	2.034
SPBC530.15C	SPBC530.15c	0.9003	0.4788	0.04561275	15.22	26.13	3.874	2.926
SPAC19A8.02	SPAC19A8.02	0.9316	0.4807	0.03077052	18.09	31.2	2.823	4.974
SPBC16G5.16	SPBC16G5.16	0.8282	0.4842	0.081864774	19.32	33.39	1.042	1.987
SPCC1672.06C	asp1	0.8762	0.4878	0.057396751	17.12	29.48	2.157	2.683
SPAC1610.01	SPAC1610.01	0.9191	0.4883	0.036637234	9.519	16	5.721	3.293
SPBC1D7.05	byr2	0.9049	0.4884	0.043399412	12.32	20.96	4.96	2.879
SPBC577.15C	sim3	0.8341	0.4899	0.078781879	2.889	4.251	2.509	1.73
SPAC6B12.14C	SPAC6B12.14c	0.8518	0.4928	0.069662364	17.25	29.69	0.6165	2.405
SPCC1919.10C	myo52	0.8466	0.4928	0.072321736	7.365	12.18	1.098	2.281
SPCC16C4.06C	SPCC16C4.06c	0.8322	0.4985	0.079772289	14.43	24.7	1.36	2.06
SPAC16.04	dus3	0.5875	0.4992	0.230992129	13.58	23.18	1.296	0.4561
SPBC24C6.09C	SPBC24C6.09c	0.8316	0.502	0.080085519	15.13	25.92	1.379	2.065
SPAC18B11.09C	SPAC18B11.09c	0.3801	0.5025	0.42010213	15.8	27.11	0.3071	0.4757
SPAC1565.01	SPAC1565.01	0.8881	0.5044	0.05153813	14.82	25.37	1.23	3.245
SPBC17D1.07C	SPBC17D1.07c	0.9226	0.507	0.03498655	15.83	27.15	3.418	4.515
SPBC418.01C	his4	0.928	0.5091	0.032452024	15.95	27.37	2.969	4.989
SPBPB2B2.01	SPBPB2B2.01	0.9178	0.5112	0.037251947	9.414	15.78	6.286	3.153
SPCC1682.01	qcr9	0.9051	0.5116	0.043303435	5.279	8.448	4.885	3.054
SPBC3E7.15C	lac1	0.783	0.512	0.106238238	1.477	1.711	1.711	1.477
SPACUNK4.10	SPACUNK4.10	0.7983	0.5122	0.097833871	16.11	27.64	1.528	1.681
SPCC1442.13C	SPCC1442.13c	0.8609	0.5125	0.065047292	7.067	11.62	3.984	1.656
SPAC24H6.08	SPAC24H6.08	0.8231	0.5162	0.084547398	15.5	26.56	3.188	1.266
SPCC23B6.01C	SPCC23B6.01c	0.8134	0.5174	0.089695832	8.771	14.63	0.8056	1.974
SPBC1711.04	SPBC1711.04	0.8526	0.5177	0.069254672	14.33	24.47	3.102	2.012
SPBC15D4.03	slm9	0.8482	0.5195	0.071501732	15.11	25.86	3.098	1.922
SPCC320.12	atp23	0.6686	0.5196	0.174833628	5.575	8.959	1.703	0.6238
SPCC330.07C	SPCC330.07c	0.9317	0.5216	0.030723905	17.69	30.42	2.89	5.429
SPBC32H8.09	SPBC32H8.09	0.8966	0.5226	0.047401265	7.93	13.13	4.454	2.916
SPBC1861.01C	cnp3	0.8389	0.5228	0.076289806	16.37	28.09	1.379	2.268
SPCC126.02C	pku70	0.8873	0.5235	0.051929518	14.76	25.22	2.622	3.15
SPBC1604.07	atp4	0.9293	0.5268	0.031844063	17.16	29.48	3.961	5.122
SPBC2G5.02C	SPBC2G5.02c	0.9115	0.5274	0.040243327	10.38	17.46	4.127	3.86
SPAPB1E7.11C	SPAPB1E7.11c	0.8914	0.5304	0.04992737	5.801	9.339	4.823	2.675
SPCC70.02C	SPCC70.02c	0.8691	0.5314	0.06093025	15.12	25.85	2.812	2.618
SPBC660.14	mik1	0.95	0.5335	0.022276395	14.48	24.72	7.126	7.002
SPBP8B7.23	SPBP8B7.23	0.3587	0.537	0.445268623	17.56	30.16	0.6157	0.4123
SPAC8E11.04C	SPAC8E11.04c	0.8806	0.5387	0.055221319	17.27	29.65	3.176	2.899
SPCC663.01C	ekc1	0.391	0.5428	0.407823243	0.5428	0	0	0.5428
SPAC1B2.03C	SPAC1B2.03c	0.8894	0.5447	0.050902875	12.26	20.76	2.078	3.465
SPAC17G6.03	SPAC17G6.03	0.8233	0.547	0.084441885	15.51	26.51	1.549	2.121
SPCC1020.12C	xap5	0.8438	0.552	0.073760479	14.87	25.38	2.633	2.214
SPBC4B4.07C	usp102	0.6804	0.5522	0.167235695	10.48	17.58	1.653	0.8708
SPBC30B4.06C	SPBC30B4.06c	0.8609	0.5554	0.065047292	14.72	25.1	2.073	2.737
SPBP4H10.03	oxa102	0.8899	0.5563	0.050658793	8.118	13.4	4.594	2.849
SPAC13C5.02	dre4	0.8489	0.5569	0.071143466	5.922	9.508	3.484	1.993
SPBC32F12.08C	duo1	0.9017	0.5621	0.04493793	9.083	15.1	5.095	3.276
SPAC977.14C	SPAC977.14c	0.8334	0.5631	0.079146504	12.83	21.73	1.322	2.375
SPAC29A4.14C	SPAC29A4.14c	0.7232	0.5652	0.140741583	14.84	25.29	0.8492	1.399
SPAC8F11.03	msh3	0.9256	0.5653	0.033576654	17.68	30.32	2.876	5.406
SPAC6B12.06C	rrg9	0.7603	0.5668	0.11901501	15.99	27.34	1.01	1.629

SPCC794.07	lat1	0.8411	0.5674	0.075152367	3.523	5.238	3.26	1.989
SPAC328.06	ubp2	0.9178	0.5726	0.037251947	18.2	31.25	3.756	4.774
SPAC19A8.14	SPAC19A8.14	0.8511	0.5748	0.070019409	16.84	28.83	2.021	2.639
SPAC30D11.09	cwf19	0.8623	0.5794	0.064341614	9.658	16.09	3.119	2.648
SPCC584.12	mug42	0.8521	0.58	0.069509435	18.29	31.39	2.445	2.596
SPBC660.10	SPBC660.10	0.8504	0.5806	0.070376748	10.3	17.23	2.232	2.613
SPCC11E10.04	ppr6	0.9241	0.5812	0.03428103	16.74	28.64	3.374	5.379
SPBC21.05C	ral2	0.857	0.5858	0.067019178	12.95	21.91	3.237	2.61
SPBPB10D8.01	SPBPB10D8.01	0.7855	0.587	0.104853811	14.11	23.97	2.011	1.705
SPCC1235.12C	mug146	0.8174	0.5874	0.087565367	17.16	29.37	0.4751	2.322
SPAC4G8.10	gos1	0.9051	0.5921	0.043303435	15.59	26.57	4.794	3.889
SPAC22G7.05	SPAC22G7.05	0.793	0.5926	0.100726813	6.983	11.32	2.558	1.679
SPAC890.05	SPAC890.05	0.7814	0.5936	0.107126593	7.995	13.12	2.986	1.136
SPAC1142.05	ctr5	0.771	0.5939	0.112945622	2.636	3.62	2.096	1.543
SPAC1610.03C	crp79	0.8127	0.5957	0.09006974	16.71	28.55	1.642	2.167
SPCC965.06	SPCC965.06	0.8355	0.596	0.078053546	15.6	26.58	1.846	2.481
SPCC1442.16C	zta1	0.6811	0.6008	0.16678912	15.61	26.59	1.957	0.8396
SPBC16E9.08	mcp4	0.7817	0.6011	0.106959888	14.2	24.11	2.054	1.705
SPAC139.03	SPAC139.03	0.8979	0.6013	0.046772028	17.35	29.68	2.613	4.124
SPCC18.10	SPCC18.10	0.7388	0.6028	0.131473113	15.69	26.73	1.414	1.502
SPCC24B10.10C	SPCC24B10.10c	0.8273	0.6029	0.082336976	15.94	27.19	2.426	2.236
SPCC1223.11	ptc2	0.8802	0.6046	0.055418636	14.4	24.45	3.764	3.177
SPBPB2B2.18	SPBPB2B2.18	0.9009	0.6057	0.045323413	17.96	30.76	1.912	4.384
SPAC22H10.02	SPAC22H10.02	0.7937	0.6059	0.10034362	14.13	23.97	0.1321	2.12
SPAC222.07C	hri2	0.8275	0.6073	0.082231998	14.48	24.58	1.627	2.436
SPAC22F3.09C	res2	0.8093	0.6073	0.09189046	7.906	12.93	2.957	1.819
SPAC22G7.06C	ura1	0.8204	0.6085	0.085974348	13.52	22.88	2.257	2.195
SPCC320.04C	SPCC320.04c	0.8289	0.6089	0.08149786	17.39	29.74	2.519	2.266
SPAC29A4.11	rga3	0.9234	0.6105	0.03461013	18.15	31.08	3.739	5.565
SPAC24C9.05C	mug70	0.914	0.612	0.039053804	10.67	17.82	5.981	4.244
SPAC8F11.10C	pvg1	0.8339	0.612	0.078886026	5.542	8.737	3.238	2.11
SPCC338.06C	SPCC338.06c	0.8146	0.6132	0.089055594	14.81	25.15	1.678	2.26
SPAC15A10.11	ubr11	0.8805	0.6161	0.05527064	6.237	9.961	4.147	3.14
SPBC2D10.18	abc1	0.3506	0.6166	0.455188088	0.6166	0	0	0.6166
SPBC1685.13	fhn1	0.5137	0.6166	0.289290434	11.52	19.32	0.9694	0.6965
SPAC11E3.09	pyp3	0.847	0.6169	0.07211659	15.91	27.1	2.858	2.573
SPBC1718.06	msp1	0.8697	0.617	0.06063053	12.63	21.28	4.296	2.664
SPCC553.04	cyp9	0.8684	0.6179	0.061280185	10.37	17.29	4.461	2.537
SPAC6C3.07	mug68	0.8721	0.6186	0.059433714	18.36	31.44	1.684	3.441
SPBC1198.12	mfr1	0.9166	0.6215	0.037820147	16.09	27.41	3.813	5.145
SPAC4G9.16C	rpl901	0.1716	0.6251	0.765482716	0.6251	0	0	0.4105
SPBC577.05C	rec27	0.9325	0.6251	0.03035116	8.966	14.78	8.538	5.187
SPAC664.10	klp2	0.7826	0.6277	0.106460156	13.54	22.89	2.917	1.419
SPAC24H6.11C	SPAC24H6.11c	0.8425	0.6279	0.07443009	11.27	18.85	4.582	1.512
SPBC146.10	mug57	0.7376	0.6281	0.132179092	16.55	28.21	1.855	1.441
SPCC285.13C	nup60	0.8209	0.6291	0.085709744	12.89	21.72	2.842	2.111
SPAC5D6.02C	mug165	0.7505	0.6312	0.124649303	13.31	22.47	1.517	1.659
SPBC21H7.04	dbp7	0.5706	0.6355	0.243668233	1.736	1.95	1.354	0.7833
SPCPJ732.03	meu15	0.7981	0.6375	0.097942689	5.382	8.408	3.365	1.428
SPAC824.05	vps16	0.8767	0.6381	0.057148993	12.71	21.39	4.767	3.011
SPAC17A2.14	SPAC17A2.14	0.8651	0.6429	0.062933688	16	27.21	2.191	3.316
SPBC14F5.10C	SPBC14F5.10c	0.838	0.6434	0.076755981	8.746	14.36	3.95	2.026
SPBC31A8.01C	rtn1	0.8145	0.6461	0.089108911	17.61	30.07	0.7208	2.503
SPAC5H10.09C	SPAC5H10.09c	0.8207	0.648	0.085815567	14.89	25.23	1.849	2.469
SPAC17H9.08	SPAC17H9.08	0.8356	0.6494	0.078001569	6.609	10.56	4.833	1.067
SPBC660.08	SPBC660.08	0.8938	0.6512	0.04875965	19.15	32.79	2.126	4.373
SPBC30D10.13C	pdb1	0.5647	0.6527	0.248182212	1.536	1.565	1.035	0.8868
SPBC725.10	SPBC725.10	0.5785	0.6529	0.237696637	10.34	17.16	0.639	1.013
SPAC1F5.03C	SPAC1F5.03c	0.8214	0.6549	0.085445301	18.06	30.84	0.6732	2.642
SPAC5H10.04	SPAC5H10.04	0.8533	0.6554	0.068898254	16.59	28.24	4.867	1.972
SPBC3H7.12	rav2	0.6206	0.6571	0.207188229	1.132	0.8415	0.8415	1.132
SPAC20H4.02	dsc3	0.8445	0.6599	0.073400346	4.467	6.746	3.576	2.504
SPBC428.05C	arg12	0.8563	0.6615	0.067374056	7.205	11.6	4.093	2.626
SPAC1952.10C	SPAC1952.10c	0.7237	0.6626	0.140441427	13.69	23.08	1.604	1.52
SPAC3F10.04	gsa1	0.8457	0.6657	0.072783669	7.08	11.37	3.873	2.438
SPCC4F11.03C	SPCC4F11.03c	0.7504	0.6658	0.124707175	16.33	27.76	0.7049	1.883
SPBC11B10.10C	pht1	0.7568	0.6705	0.121018877	9.61	15.84	2.877	1.273
SPCC188.07	ccq1	0.793	0.6709	0.100726813	8.297	13.51	2.839	1.845
SPCC1450.11C	cek1	0.7325	0.6736	0.135192371	14.87	25.16	1.696	1.596

SPCC1223.10C	eaf1	0.7991	0.6741	0.097398869	15.82	26.85	2.814	1.969
SPAC19G12.13C	poz1	0.8619	0.6744	0.064543119	16.27	27.64	2.558	3.342
SPAC1486.04C	alm1	0.7985	0.6759	0.09772508	13.2	22.2	3.209	1.77
SPCC61.05	SPCC61.05	0.8111	0.6766	0.090925599	10.02	16.56	2.028	2.403
SPBC3H7.14	mug176	0.8984	0.6798	0.046530257	15.83	26.85	1.201	4.865
SPBC36.10	SPBC36.10	0.7959	0.6803	0.099141495	12.21	20.43	4.026	0.9465
SPCC24B10.22	pog1	0.9064	0.6819	0.042680103	17.62	30.03	2.721	5.176
SPBC21.07C	ppk24	0.666	0.6831	0.176525771	15.54	26.33	1.704	1.156
SPBC354.05C	sre2	0.6918	0.6837	0.160019442	2.967	4.047	1.936	1.287
SPAC824.08	gda1	0.6549	0.6882	0.18382501	15.14	25.6	1.366	1.228
SPBC1706.01	tea4	0.8754	0.6899	0.057793458	9.155	15	5.082	3.092
SPBC146.06C	SPBC146.06c	0.8177	0.6904	0.087406002	15.63	26.47	1.853	2.6
SPBC405.02C	SPBC405.02c	0.8289	0.6926	0.08149786	17.41	29.63	1.522	2.852
SPBC1921.06C	pvg3	0.7041	0.6926	0.152365656	11.26	18.73	1.98	1.329
SPBP22H7.08	rps1002	0.8848	0.6937	0.053154886	19.44	33.21	2.479	4.241
SPCC297.03	ssp1	0.9132	0.6941	0.039434097	16.71	28.37	3.385	5.632
SPBC16E9.14C	zrg17	0.9086	0.698	0.041627268	20.12	34.42	4.601	5.125
SPBC146.04	SPBC146.04	0.8789	0.6999	0.056060536	19.06	32.54	2.61	4.037
SPCC1183.09C	pmp31	0.8983	0.7	0.0465786	16.84	28.6	3.104	4.817
SPBC25B2.08	SPBC25B2.08	0.8136	0.7004	0.08958906	16.71	28.37	1.585	2.619
SPBC902.02C	ctf18	0.7783	0.7005	0.10885297	14.99	25.31	1.746	2.117
SPAC17A5.09C	SPAC17A5.09c	0.8439	0.7017	0.073709013	15.65	26.48	2.256	3.089
SPCC1682.15	mug122	0.9173	0.7054	0.037488606	16.46	27.92	6.192	5.44
SPBC17G9.12C	SPBC17G9.12c	0.7903	0.7055	0.102208018	15.1	25.5	2.728	2.008
SPCC1442.01	ste6	0.9081	0.7072	0.041866324	11.8	19.65	6.585	4.538
SPBP8B7.10C	SPBP8B7.10c	0.9082	0.7076	0.041818502	18.41	31.38	3.22	5.432
SPAC1296.06	tah18	0.8681	0.7128	0.061430244	6.279	9.864	5.109	3.073
SPCC825.05C	SPCC825.05c	0.7927	0.715	0.100891142	7.886	12.71	3.254	1.843
SPBC947.15C	SPBC947.15c	0.7604	0.7184	0.118957892	12.32	20.55	2.56	1.722
SPAC683.03	SPAC683.03	0.863	0.7202	0.063989204	20.19	34.5	3.155	3.516
SPBC21B10.03C	SPBC21B10.03c	0.8029	0.7208	0.095338542	9.239	15.1	3.164	2.103
SPAC24B11.06C	sty1	0.254	0.7237	0.595166283	0.8795	0.276	0.1908	0.5758
SPBP22H7.04	SPBP22H7.04	0.8902	0.7247	0.05051241	18.38	31.29	2.861	4.633
SPAC6G10.03C	SPAC6G10.03c	0.6909	0.7259	0.160584807	16.12	27.28	1.423	1.513
SPAC1296.04	mug65	0.7304	0.7294	0.136439235	13.7	22.98	2.996	1.074
SPAC17C9.10	stm1	0.7486	0.7331	0.125750177	12.67	21.15	0.4525	2.077
SPBC19C7.08C	SPBC19C7.08c	0.879	0.7409	0.056011125	18.41	31.31	2.335	4.337
SPBC1773.14	arg7	0.5364	0.7423	0.270511231	14.28	23.99	0.5504	1.04
SPAC26F1.14C	aif1	0.8725	0.7424	0.059234564	17.42	29.55	2.029	4.143
SPBC4B4.08	ght2	0.8152	0.7444	0.088735829	16.95	28.72	3.205	2.439
SPBC800.04C	rpl4301	0.6312	0.7476	0.19983301	2.117	2.426	1.406	1.232
SPBC1734.09	SPBC1734.09	0.7198	0.7501	0.142788158	15.14	25.49	1.303	1.803
SPAC17D4.03C	cis4	0.7946	0.7503	0.099851439	17.57	29.8	1.457	2.549
SPBC428.03C	pho4	0.8835	0.7526	0.053793446	16.24	27.44	2.181	4.61
SPAC10F6.06	vip1	0.7255	0.7553	0.139362583	15.69	26.46	1.025	1.901
SPBC2A9.06C	SPBC2A9.06c	0.8653	0.7612	0.062833296	16.48	27.85	2.322	3.974
SPBC18E5.09C	EMPTY	0.8335	0.7622	0.079094396	16.52	27.93	1.778	3.22
SPAC20G8.08C	fft1	0.8626	0.7624	0.064190546	8.327	13.4	5.103	3.087
SPBC16E9.07	mug100	0.1356	0.7631	0.86774031	11.61	19.22	0.5477	0.3171
SPBC18A7.01	SPBC18A7.01	0.785	0.7671	0.105130343	16.36	27.63	1.597	2.46
SPAC589.06C	SPAC589.06c	0.7635	0.7674	0.117190959	13.17	21.98	1.217	2.26
SPCC663.14C	SPCC663.14c	0.7798	0.7708	0.108016769	14.38	24.12	1.195	2.46
SPCC330.06C	SPCC330.06c	0.4896	0.7711	0.310158591	0.9718	0.3555	0.3555	0.9718
SPCC1322.10	SPCC1322.10	0.8148	0.7717	0.08894898	6.4	9.975	3.342	2.513
SPAC12B10.11	exg2	0.8569	0.7721	0.067069857	16.42	27.72	2.373	3.769
SPAC31G5.04	SPAC31G5.04	0.8455	0.7734	0.072886388	7.957	12.73	4.768	2.685
SPAP27G11.14C	SPAP27G11.14c	0.7496	0.7734	0.125170422	5.992	9.249	1.824	2.032
SPAC1782.05	SPAC1782.05	0.6369	0.7754	0.195928751	15.21	25.59	1.413	1.321
SPAC29B12.14C	SPAC29B12.14c	0.7431	0.7769	0.128952739	12.4	20.6	3.591	0.8975
SPBC725.06C	ppk31	0.7918	0.777	0.101384503	15.97	26.92	3.346	2.087
SPBC776.09	ste13	0.836	0.7771	0.077793723	8.177	13.11	4.381	2.608
SPAC30D11.13	hus5	0.7852	0.7793	0.105019709	2.269	2.64	2.64	2.269
SPBC1E8.03C	SPBC1E8.03c	0.8053	0.7798	0.094042301	17.72	30.03	1.884	2.757
SPBC3E7.05C	SPBC3E7.05c	0.6982	0.7808	0.156020156	13.1	21.83	1.07	1.762
SPAC23A1.07	SPAC23A1.07	0.7497	0.7843	0.125112489	10.28	16.82	1.027	2.192
SPBPB2B2.08	SPBPB2B2.08	0.7203	0.7851	0.142486585	15.28	25.68	0.7243	1.968
SPBC21C3.20C	git1	0.475	0.7856	0.32330639	10.6	17.39	1.302	0.7234
SPAC1527.02	sft2	0.6451	0.7892	0.190372958	14.52	24.33	0.5995	1.524
SPBC18E5.10	SPBC18E5.10	0.7667	0.7901	0.115374537	15.39	25.87	2.109	2.21

SPAC1071.08	rpp203	0.8674	0.7904	0.061780582	14.71	24.67	7.452	1.324
SPCC1739.15	wtf21	0.7094	0.7926	0.149108816	2.818	3.59	2.073	1.644
SPBC1271.09	SPBC1271.09	0.8691	0.7929	0.06093025	16.7	28.19	3.411	4.102
SPCC1840.04	pca1	0.866	0.7932	0.062482108	17.89	30.3	2.018	4.215
SPAC1805.11C	rps2602	0.7871	0.7972	0.103970088	17.3	29.25	1.843	2.556
SPBC56F2.05C	SPBC56F2.05c	0.8793	0.7997	0.055862927	8.643	13.9	5.665	3.894
SPCPB16A4.04C	trm8	0.6392	0.8005	0.194363234	12.42	20.6	0.5705	1.52
SPBC4F6.10	vps901	0.8553	0.8027	0.067881528	8.835	14.23	4.785	3.23
SPBC36B7.02	SPBC36B7.02	0.7012	0.8054	0.154158093	14.25	23.83	2.184	1.569
SPAC31A2.15C	dcc1	0.7593	0.8068	0.1195866	14.29	23.9	1.861	2.235
SPAC926.06C	SPAC926.06c	0.8567	0.8073	0.067171233	17.35	29.32	3.91	3.626
SPBC17A3.08	SPBC17A3.08	0.7871	0.8094	0.103970088	17.4	29.41	3.436	2.109
SPAC823.09C	SPAC823.09c	0.7559	0.8094	0.121535655	17.1	28.87	3.241	1.685
SPAC30D11.02C	SPAC30D11.02c	0.8098	0.8099	0.091622228	17.09	28.85	3.94	2.33
SPAC8F11.08C	SPAC8F11.08c	0.7069	0.81	0.150642018	15.03	25.21	2.198	1.628
SPAC56F8.05C	mug64	0.6957	0.8109	0.157577997	14.94	25.03	0.7958	1.848
SPACUNK4.12C	mug138	0.8799	0.8119	0.055566682	22.18	37.86	3.415	4.86
SPAC4G8.04	SPAC4G8.04	0.8967	0.8123	0.04735283	12.47	20.66	7.035	4.488
SPAC1296.02	cox4	0.7227	0.8134	0.141041945	9.689	15.73	3.242	1.164
SPAC959.06C	SPAC959.06c	0.8393	0.8213	0.076082777	16.75	28.22	1.098	3.682
SPAC1006.06	rgf2	0.8666	0.8213	0.062181315	18.42	31.19	2.289	4.36
SPBC21B10.06C	inp2	0.6982	0.8227	0.156020156	8.834	14.2	2.389	1.506
SPBC4F6.05C	SPBC4F6.05c	0.898	0.8232	0.046723663	19.48	33.06	4.065	5.575
SPAC323.03C	SPAC323.03c	0.4796	0.8234	0.319120826	16.71	28.15	1.355	0.7805
SPAC823.05C	tlg2	0.833	0.8236	0.079354999	3.29	4.371	4.371	2.808
SPAP7G5.06	per1	0.8351	0.8243	0.078261516	16.12	27.11	3.003	3.33
SPAC607.06C	SPAC607.06c	0.6936	0.8244	0.158890916	17.11	28.86	1.618	1.737
SPCC645.02	gep4	0.8092	0.8253	0.091944126	19.29	32.72	2.302	2.931
SPBC19G7.02	SPBC19G7.02	0.6472	0.8269	0.188961491	14.01	23.35	2.35	1.086
SPBPB2B2.11	SPBPB2B2.11	0.8292	0.8282	0.081340707	14.95	25.02	2.143	3.373
SPCC162.04C	wtf13	0.8464	0.8282	0.072424345	16.37	27.54	2.069	3.801
SPAC9.08C	SPAC9.08c	0.781	0.8282	0.107348966	15.23	25.52	1.899	2.572
SPBC21C3.13	rps1901	0.8505	0.8291	0.070325682	8.819	14.16	5.331	2.951
SPBC1539.04	tts1	0.7575	0.83	0.120617363	16.04	26.95	1.752	2.31
SPCC162.05	coq3	0.8385	0.8346	0.076496933	5.194	7.726	4.462	2.999
SPAC1805.10	SPAC1805.10	0.6288	0.8367	0.201487467	15.8	26.52	0.9908	1.494
SPAC27D7.04	omt2	0.7344	0.8393	0.134067332	14.96	25.02	0.6717	2.234
SPBC646.06C	agn2	0.7818	0.8411	0.106904334	16.57	27.87	2.85	2.395
SPAC1786.01C	SPAC1786.01c	0.8594	0.8413	0.065804651	16.88	28.42	1.393	4.315
SPCC1223.01	SPCC1223.01	0.7784	0.8426	0.108797173	18.41	31.13	1.539	2.642
SPAC1006.01	psp3	0.8788	0.843	0.056109952	15.97	26.8	3.872	4.726
SPAC23C11.13C	hpt1	0.8133	0.8438	0.089749228	11.87	19.54	3.743	2.679
SPBC36.04	cys11	0.7584	0.8458	0.120101676	16.1	27.02	2.302	2.249
SPCC736.07C	SPCC736.07c	0.8598	0.8459	0.065602559	8.391	13.37	4.83	3.669
SPAC1F5.10	SPAC1F5.10	0.7182	0.8569	0.143754599	8.792	14.06	3.23	1.327
SPAC1952.07	rad1	0.6646	0.8585	0.177439663	15.91	26.67	1.565	1.632
SPCC1795.10C	SPCC1795.10c	0.7562	0.8599	0.121363327	14.97	25	1.975	2.347
SPAC23D3.11	ayr1	0.8473	0.8616	0.071962794	17.98	30.33	0.6157	4.1
SPCC16C4.11	pef1	0.6888	0.8632	0.161906862	12.92	21.36	0.5387	1.941
SPAC17A2.02C	SPAC17A2.02c	0.7171	0.8646	0.144420277	14.29	23.79	3.619	0.8873
SPAC6G9.05	pcd1	0.8483	0.8651	0.071450533	16.19	27.16	2.31	4.006
SPBC11C11.11C	SPBC11C11.11c	0.732	0.8662	0.135488919	14.21	23.65	2.607	1.905
SPCC63.06	SPCC63.06	0.8776	0.8724	0.056703385	10.84	17.67	6.985	3.734
SPAC6F6.17	rif1	0.8005	0.8747	0.096638664	16.67	27.99	3.162	2.761
SPBC8D2.12C	SPBC8D2.12c	0.874	0.8751	0.058488567	18.01	30.36	2.794	4.898
SPAC821.10C	sod1	0.8844	0.8757	0.053351266	18.23	30.75	3.311	5.308
SPCC1840.03	sal3	0.797	0.8763	0.098541679	8.107	12.81	4.475	2.05
SPBC32F12.07C	SPBC32F12.07c	0.8545	0.8862	0.068287933	17.34	29.16	2.341	4.298
SPBC800.05C	atb2	0.8294	0.8864	0.081235969	12.35	20.32	3.546	3.349
SPAC1B3.10C	SPAC1B3.10c	0.8705	0.8897	0.060231225	19.87	33.64	3.076	4.796
SPAC8C9.09C	mug129	0.8759	0.8937	0.057545474	8.551	13.57	6.816	3.907
SPAC869.10C	put4	0.8442	0.8947	0.073554652	16.87	28.31	2.121	4.059
SPCC1235.11	SPCC1235.11	0.8256	0.899	0.083230316	8.081	12.73	4.501	2.961
SPBC32F12.02	rec14	0.7789	0.8999	0.108518296	14.78	24.6	2.393	2.696
SPAC1486.01	SPAC1486.01	0.6506	0.9021	0.186685941	13.03	21.5	2.594	1.195
SPCC18.03	SPCC18.03	0.6114	0.9024	0.213674566	15.06	25.09	1.821	1.328
SPBC12D12.06	srb11	0.7973	0.9077	0.098378236	4.558	6.468	3.734	2.635
SPCC1281.03C	emc4	0.8736	0.9098	0.058687375	19.03	32.11	1.919	5.181
SPCC74.04	SPCC74.04	0.8646	0.9155	0.063184769	11.07	17.99	6.019	3.86

SPAP14E8.04	oma1	0.4471	0.9181	0.34959533	16.21	27.11	1.18	0.903
SPBC27.08C	sua1	0.6815	0.9188	0.16653414	4.417	6.199	3.58	0.2534
SPAC18B11.02C	SPAC18B11.02c	0.5353	0.9219	0.271402757	15.76	26.3	1.33	1.168
SPBP8B7.09C	los1	0.8361	0.9248	0.077741777	9.236	14.73	5.733	2.795
SPAC13G7.04C	mac1	0.7788	0.9255	0.108574057	16.76	28.07	3.176	2.57
SPBC17G9.09	tif213	0.6346	0.93	0.197499932	16.01	26.72	0.3956	1.756
SPCC737.04	SPCC737.04	0.6519	0.9301	0.185819019	13.8	22.81	2.842	1.104
SPAC144.11	rps1102	0.731	0.9318	0.136082623	13.57	22.39	3.978	1.21
SPBC8D2.18C	SPBC8D2.18c	0.6594	0.9321	0.180851057	15.44	25.71	1.626	1.753
SPBC2G2.03C	sbh1	0.9047	0.9347	0.04349541	9.596	15.35	8.862	5.564
SPAC16E8.06C	nop12	0.841	0.9361	0.075204004	18.62	31.34	0.1982	4.281
SPBC19C2.13C	ctu2	0.7818	0.9361	0.106904334	15	24.92	1.009	3.057
SPAC26F1.12C	SPAC26F1.12c	0.6325	0.9368	0.19893947	16.79	28.09	1.259	1.669
SPBC3B9.05	SPBC3B9.05	0.7886	0.9369	0.103143227	14.22	23.54	2.342	2.997
SPCC74.09	mug24	0.6372	0.9371	0.195724233	15.3	25.46	1.802	1.57
SPAC24H6.09	gef1	0.6165	0.9382	0.210066919	15.86	26.44	1.531	1.525
SPBC56F2.03	SPBC56F2.03	0.859	0.9389	0.066006836	17.98	30.2	2.494	4.712
SPAC22F3.04	mug62	0.6848	0.9395	0.164436248	15.65	26.07	2.836	1.516
SPCC777.17C	SPCC777.17c	0.7416	0.9427	0.129830279	16.61	27.77	2.332	2.352
SPBC83.03C	tas3	0.541	0.9432	0.266802735	11.41	18.55	2.032	0.8874
SPBC20F10.03	SPBC20F10.03	0.8273	0.9446	0.082336976	16.51	27.58	2.508	3.789
SPAC977.15	SPAC977.15	0.726	0.9452	0.139063379	15.51	25.81	2.568	2.108
SPCC1494.03	arz1	0.8601	0.9524	0.065451052	17.2	28.79	2.057	4.868
SPBC18H10.09	SPBC18H10.09	0.7883	0.9539	0.103308473	14.55	24.09	3.125	2.867
SPAC20H4.04	fml2	0.8528	0.9573	0.069152808	19.77	33.34	2.541	4.584
SPAC869.09	SPAC869.09	0.6549	0.9574	0.18382501	16.56	27.64	1.945	1.696
SPBC1734.04	anp1	0.5748	0.9645	0.240483241	9.518	15.16	1.968	1.259
SPCC895.09C	ucp12	0.8454	0.9683	0.072937757	14.82	24.54	5.746	3.479
SPCC162.11C	SPCC162.11c	0.855	0.9712	0.068033885	23.05	39.13	2.347	4.754
SPBC1921.03C	mex67	0.7715	0.974	0.11266407	9.666	15.4	5.009	1.397
SPBC776.06C	SPBC776.06c	0.8998	0.9742	0.045854011	14.32	23.66	9.297	5.243
SPAC3F10.05C	mug113	0.7074	0.9749	0.150334945	15.56	25.85	1.348	2.278
SPACUNK4.16C	SPACUNK4.16c	0.838	0.9764	0.076755981	18.06	30.27	2.517	4.219
SPAC607.07C	SPAC607.07c	0.6768	0.9771	0.16953965	17.35	29.02	1.464	2.014
SPAC630.11	vps55	0.7332	0.9822	0.134777544	18.07	30.29	1.273	2.56
SPAP14E8.02	SPAP14E8.02	0.7773	0.9864	0.109411332	17.78	29.76	2.195	3.014
SPBC21C3.19	SPBC21C3.19	0.7864	0.9884	0.104356495	14.44	23.84	3.984	2.661
SPAC6F6.02C	pof5	0.744	0.9916	0.128427064	16.17	26.9	1.291	2.705
SPCC1450.03	SPCC1450.03	0.9043	0.9932	0.043687469	12.81	20.95	10.07	5.512
SPAC13G7.06	met16	0.6184	0.9967	0.208730519	8.786	13.8	1.623	1.632
SPBC577.14C	spa1	0.5663	0.9983	0.246953438	15.11	25.01	1.888	1.251
SPBC359.04C	SPBC359.04c	0.6602	1.001	0.18032448	15.36	25.45	1.733	1.892
SPCC757.12	SPCC757.12	0.8765	1.002	0.05724808	17.84	29.84	3.16	5.737
SPAC823.10C	SPAC823.10c	0.5169	1.004	0.286593468	15.04	24.87	1.038	1.293
SPCC191.06	SPCC191.06	0.7631	1.004	0.117418546	17.95	30.03	1.646	2.945
SPCC794.12C	mae2	0.799	1.004	0.097453221	14.46	23.84	5.112	2.424
SPAPB1A10.14	SPAPB1A10.14	0.8147	1.006	0.089002284	8.934	14.05	6.358	1.874
SPCC1840.07C	SPCC1840.07c	0.846	1.013	0.072629637	17.2	28.68	1.685	4.724
SPCC1281.07C	SPCC1281.07c	0.7453	1.018	0.127668879	15.93	26.43	1.05	2.816
SPCC613.10	qcr2	0.8137	1.019	0.089535684	7.757	11.94	4.002	3.433
SPCC622.15C	SPCC622.15c	0.5694	1.02	0.244582537	16.97	28.26	2.289	1.094
SPAC23C4.05C	SPAC23C4.05c	0.7567	1.02	0.121076266	16.52	27.46	2.388	2.782
SPBPB2B2.19C	SPBPB2B2.19c	0.8778	1.02	0.056604423	8.269	12.85	7.416	4.785
SPAC1006.03C	red1	0.8105	1.021	0.091246981	8.964	14.08	4.903	2.992
SPCC736.14	dis1	0.877	1.022	0.057000407	13.19	21.57	7.825	4.527
SPAC1687.10	mcp1	0.607	1.023	0.216811309	17.04	28.39	1.631	1.615
SPAC3H5.10	rpl3202	0.7875	1.024	0.103749438	5.977	8.777	5.069	2.225
SPAC5D6.13	SPAC5D6.13	0.8448	1.024	0.073246095	15.88	26.32	1.384	4.759
SPCC1739.10	mug33	0.7462	1.03	0.127144755	12.66	20.61	4.564	1.556
SPAC1952.06C	SPAC1952.06c	0.7213	1.03	0.141884068	14.44	23.76	0.962	2.593
SPBC12D12.07C	trx2	0.6811	1.033	0.16678912	3.298	4.013	2.528	1.911
SPAPB2B4.04C	SPAPB2B4.04c	0.4272	1.037	0.369368756	18.29	30.58	1.875	0.5404
SPAC15A10.15	sgo2	0.8402	1.037	0.075617323	13.16	21.48	7.197	2.734
SPBC8D2.16C	SPBC8D2.16c	0.5826	1.038	0.23462952	15.69	25.97	1.614	1.514
SPAC2E1P3.02C	amt3	0.7517	1.04	0.12395545	18.11	30.24	2.885	2.65
SPAC4H3.05	srs2	0.8832	1.043	0.05394094	13.37	21.85	7.404	5.347
SPBC4F6.11C	SPBC4F6.11c	0.5963	1.048	0.224535191	16.66	27.66	1.606	1.609
SPAC4G8.05	ppk14	0.7879	1.049	0.1035289	12.46	20.23	2.427	3.377
SPAC26H5.05	SPAC26H5.05	0.7858	1.049	0.104687976	11.44	18.41	4.735	2.547

SPBC20F10.07	SPBC20F10.07	0.7398	1.05	0.130885673	14.13	23.19	3.386	2.329
SPBC115.02C	SPBC115.02c	0.6226	1.058	0.205790884	17.83	29.71	1.972	1.69
SPBC215.01	SPBC215.01	0.03524	1.062	1.4529641	9.308	14.61	0.396	0.3092
SPAC343.15	tit1	0.7376	1.062	0.132179092	8.235	12.71	3.349	2.355
SPCC63.03	SPCC63.03	0.7286	1.062	0.137510833	16.51	27.38	0.6323	2.773
SPBC8E4.05C	SPBC8E4.05c	0.6795	1.062	0.167810539	15.62	25.79	1.972	2.137
SPAC56F8.14C	mug115	0.6729	1.064	0.172049472	14.59	23.98	1.664	2.15
SPAC959.07	rps403	0.7566	1.065	0.121133663	11.85	19.11	4.798	1.814
SPAC513.02	SPAC513.02	0.7793	1.065	0.108295324	17.8	29.66	1.219	3.432
SPAC750.06C	SPAC750.06c	0.7964	1.069	0.098868749	15.11	24.88	3.488	3.391
SPAC17A2.07C	SPAC17A2.07c	0.7576	1.071	0.120560034	16.64	27.59	1.267	3.113
SPAC3H1.03	mug151	0.4373	1.073	0.359220523	16.07	26.58	1.768	0.8122
SPBC3D6.06C	prs5	0.6296	1.074	0.200935281	14.91	24.52	1.594	1.866
SPBC21C3.17C	SPBC21C3.17c	0.8367	1.078	0.077430231	18.63	31.11	2.823	4.609
SPAC3H1.07	aru1	0.7576	1.079	0.120560034	15.84	26.17	2.215	3.015
SPBC1683.12	SPBC1683.12	0.6107	1.08	0.21417208	12.98	21.09	2.506	1.431
SPBC12C2.04	SPBC12C2.04	0.8347	1.081	0.078469586	12.14	19.6	6.642	3.242
SPBC29A10.03C	pcf1	0.7936	1.083	0.100398341	17.47	29.04	2.007	3.671
SPAC17G8.13C	mst2	0.7775	1.086	0.109299602	20.39	34.21	2.108	3.529
SPBC2G2.17C	SPBC2G2.17c	0.7706	1.087	0.113170995	16.41	27.16	2.362	3.218
SPAC31G5.14	gcv1	0.7106	1.087	0.148374797	17.44	28.99	1.33	2.593
SPAC1071.07C	rps1502	0.7603	1.088	0.11901501	7.289	10.99	3.677	2.688
SPAC3F10.10C	map3	0.8552	1.088	0.067932308	17.85	29.7	3.192	5.261
SPAC144.14	klp8	0.6379	1.089	0.195247398	15.4	25.37	0.5783	2.072
SPCC11E10.07C	SPCC11E10.07c	0.2444	1.09	0.611898798	8.369	12.9	0.2705	0.7528
SPBC365.07C	SPBC365.07c	0.6665	1.093	0.176199846	16.5	27.29	1.591	2.176
SPAC977.17	SPAC977.17	0.8776	1.095	0.056703385	16.42	27.15	6.586	5.656
SPAC22F3.07C	atp20	0.7245	1.097	0.13996161	14.1	23.04	2.017	2.671
SPBC32F12.11	tdh1	0.2263	1.098	0.645315446	10.4	16.48	0.6907	0.6871
SPAC4G9.13C	vps26	0.6519	1.098	0.185819019	7.678	11.66	2.556	1.891
SPAC12B10.14C	ppk2	0.6742	1.104	0.171211252	16.91	28.01	1.34	2.3
SPCC1739.01	SPCC1739.01	0.57	1.105	0.244125144	3.999	5.127	2.216	1.349
SPAC3G9.03	rpl2301	0.801	1.107	0.096367484	5.657	8.063	4.655	3.271
SPAC4A8.14	SPAC4A8.14	0.7981	1.108	0.097942689	13.6	22.14	6.153	2.2
SPAP7G5.04C	lys1	0.7908	1.109	0.101933339	8.377	12.88	4.699	2.99
SPAC20G8.10C	atg6	0.8152	1.112	0.088735829	15.13	24.84	1.66	4.291
SPBC19G7.01C	msh2	0.548	1.113	0.261219442	10.32	16.31	1.36	1.531
SPCC1322.01	rpm1	0.334	1.117	0.476253533	9.508	14.87	0.2838	0.9687
SPAC11D3.04C	SPAC11D3.04c	0.7281	1.119	0.137808969	13.68	22.26	1.671	2.829
SPBC29A10.14	rec8	0.4699	1.121	0.327994555	17.14	28.38	2.136	0.7848
SPAC24C9.16C	cox8	0.671	1.122	0.17327748	15.43	25.35	3.421	1.603
SPAC25B8.07C	SPAC25B8.07c	0.7569	1.125	0.120961495	13.25	21.48	2.389	3.12
SPAC1093.03	SPAC1093.03	0.6912	1.13	0.160396271	13.63	22.16	2.695	2.222
SPAC15E1.07C	moa1	0.8305	1.131	0.080660363	11.29	18	7.324	2.873
SPAP7G5.03	prm1	0.6873	1.132	0.162853656	14.21	23.17	3.249	1.952
SPAC13G6.15C	SPAC13G6.15c	0.765	1.133	0.116338565	17.47	28.95	2.367	3.278
SPBC337.09	erg28	0.7793	1.133	0.108295324	8.155	12.44	4.205	3.039
SPAC1002.07C	ats1	0.7295	1.134	0.136974704	16.1	26.52	1.465	2.911
SPBC902.05C	idh2	0.7548	1.135	0.122168109	15.72	25.84	2.481	3.1
SPAC589.12	SPAC589.12	0.7916	1.136	0.101494214	7.874	11.94	5.015	3.128
SPBC1105.13C	SPBC1105.13c	0.6016	1.138	0.220692172	15.6	25.63	1.574	1.818
SPCC965.12	SPCC965.12	0.7093	1.139	0.14917004	16.33	26.92	1.509	2.69
SPAC821.07C	moc3	0.6874	1.142	0.162790472	16.61	27.41	3.623	1.762
SPBC16E9.02C	SPBC16E9.02c	0.6164	1.142	0.21013737	14.72	24.06	2.306	1.717
SPBC2D10.07C	SPBC2D10.07c	0.6357	1.143	0.196747789	16.08	26.46	2.167	1.913
SPBC106.07C	SPBC106.07c	0.7404	1.144	0.13053359	4.516	5.976	3.519	2.618
SPBC16E9.17C	rem1	0.6755	1.146	0.170374647	16.98	28.06	0.7711	2.457
SPAC11E3.08C	nse6	0.6225	1.149	0.205860644	13.97	22.73	1.899	1.902
SPAC630.14C	tup12	0.6711	1.149	0.173212761	8.991	13.9	3.356	1.745
SPAC25B8.03	psd2	0.739	1.154	0.131355562	15.56	25.53	1.857	3.038
SPBC11B10.07C	ivn1	0.5017	1.159	0.299555899	10.46	16.48	1.694	1.299
SPCC613.08	SPCC613.08	0.7514	1.159	0.124128809	14.07	22.89	3.759	2.764
SPAC17A5.02C	dbr1	0.7075	1.16	0.150273556	6.158	8.856	3.076	2.366
SPBC530.03C	bag102	0.8256	1.161	0.083230316	18.31	30.39	0.9617	4.813
SPAC144.05	SPAC144.05	0.5915	1.163	0.228045251	16.33	26.88	1.681	1.78
SPCC663.02	wtf14	0.6735	1.165	0.1716624	15.94	26.18	1.574	2.399
SPAC19B12.04	rps3001	0.514	1.167	0.289036881	17.92	29.69	0.8106	1.545
SPBC30B4.02C	SPBC30B4.02c	0.7474	1.174	0.126446906	19.06	31.7	2.07	3.181
SPAC631.02	SPAC631.02	0.8258	1.175	0.083125121	11.17	17.71	6.14	3.755

SPAC17A2.10C	SPAC17A2.10c	0.7692	1.176	0.113960724	17.27	28.53	2.437	3.475
SPAPB1A10.13	SPAPB1A10.13	0.4363	1.178	0.360214787	16.33	26.85	1.646	1.062
SPBC1347.13C	SPBC1347.13c	0.7937	1.179	0.10034362	12.79	20.58	7.024	1.37
SPBC36.11	SPBC36.11	0.6119	1.183	0.213319547	15.65	25.64	1.773	1.928
SPCC737.06C	SPCC737.06c	0.3672	1.197	0.435097327	13.68	22.12	1.355	0.9545
SPCC830.10	SPCC830.10	0.7287	1.197	0.13745123	17.64	29.15	1.533	3.063
SPAC22F8.11	plc1	0.5921	1.198	0.227604939	12.6	20.2	2.778	1.421
SPAC212.04C	SPAC212.04c	0.7359	1.199	0.133181197	17.64	29.14	2.393	3.036
SPBC16E9.18	psd1	0.867	1.2	0.061980903	15.9	26.05	8.75	4.768
SPBC725.11C	php2	0.07757	1.201	1.110306209	15.15	24.72	0.8123	0.296
SPBC336.06C	rnh1	0.7999	1.205	0.096964303	17.84	29.47	1.943	4.258
SPAC3G6.11	chl1	0.8106	1.206	0.091193401	14.27	23.16	7.789	1.661
SPBC12C2.01C	SPBC12C2.01c	0.5379	1.206	0.269298456	8.03	12.09	2.119	1.404
SPBC14C8.16C	bot1	0.7772	1.209	0.109467208	11.9	18.94	6.439	1.702
SPBC428.06C	rxt2	0.8464	1.21	0.072424345	11.61	18.43	7.894	3.989
SPBC649.03	rhp14	0.7607	1.21	0.118786584	5.608	7.794	3.186	3.288
SPBC16D10.03	pgp2	0.4074	1.215	0.389978975	17.66	29.14	1.397	1.101
SPCC320.08	SPCC320.08	0.6336	1.218	0.198184831	17	27.96	2.294	2.026
SPAC22G7.08	ppk8	0.6687	1.219	0.174768677	17.52	28.88	3.792	1.65
SPBC216.01C	SPBC216.01c	0.8585	1.219	0.066259701	18.41	30.47	4.193	5.952
SPAC20G4.03C	hri1	0.7131	1.22	0.146849564	18.89	31.31	2.446	2.786
SPBC1773.05C	tms1	0.7537	1.221	0.122801485	17.75	29.3	4.077	2.908
SPAC1687.05	pli1	0.656	1.222	0.183096161	16.46	27.01	2.161	2.262
SPAC1486.02C	dsc2	0.7179	1.223	0.143936047	9.755	15.12	4.626	1.873
SPBC685.06	rps001	0.7904	1.224	0.102153068	10.45	16.35	5.855	2.905
SPCC622.18	rpl6	0.5752	1.224	0.240181123	16.79	27.58	1.178	1.883
SPAC1687.21	SPAC1687.21	0.8281	1.225	0.081917215	16.88	27.74	3.284	4.933
SPBC8D2.11	SPBC8D2.11	0.8112	1.232	0.090872058	15.51	25.3	5.261	3.922
SPBC3B9.11C	ctf1	0.8067	1.233	0.093287943	17.27	28.41	7.568	2.051
SPAC9E9.08	rad26	0.5965	1.233	0.224389552	16.47	27	1.558	1.966
SPAPB1E7.02C	mcl1	0.7332	1.233	0.134777544	7.155	10.49	4.379	2.411
SPBP4H10.16C	SPBP4H10.16c	0.6651	1.234	0.177113052	17.05	28.02	2.625	2.245
SPAC29B12.04	snz1	0.615	1.234	0.211124884	8.903	13.59	0.9441	2.167
SPCC306.07C	SPCC306.07c	0.6732	1.234	0.171855893	16.86	27.68	2.412	2.388
SPACUNK4.19	mug153	0.7533	1.237	0.123032033	16.8	27.59	1.691	3.505
SPAPB1A10.15	arv1	0.6569	1.237	0.182500738	17.58	28.97	1.64	2.403
SPAC31A2.09C	apm4	0.5975	1.239	0.22366209	9.316	14.31	1.179	2.039
SPCC1322.07C	mug150	0.6158	1.24	0.210560315	16.09	26.32	1.551	2.109
SPAC1B9.02C	sck1	0.5155	1.241	0.28777133	15.96	26.08	2.788	0.7799
SPBC651.02	SPBC651.02	0.7127	1.241	0.147093241	15.95	26.06	1.625	2.973
SPBC1709.04C	cyp3	0.6807	1.242	0.167044249	14.95	24.3	1.564	2.641
SPCC417.11C	SPCC417.11c	0.7133	1.245	0.146727776	17.39	28.61	4.412	2.049
SPBC418.02	SPBC418.02	0.4746	1.25	0.323672266	16.62	27.24	0.8277	1.496
SPAC5D6.05	11-Sep	0.7837	1.252	0.105850153	14.46	23.41	5.282	3.336
SPBC6B1.08C	ofd1	0.4229	1.253	0.373762315	16.27	26.62	2.018	0.9004
SPAC57A7.12	SPAC57A7.12	0.1558	1.255	0.807432547	1.545	0.513	0.513	0.757
SPAC22H10.07	scd2	0.7331	1.255	0.13483678	6.932	10.06	3.385	2.918
SPAC17G8.10C	dma1	0.7964	1.256	0.098868749	19.41	32.18	1.027	4.43
SPCC663.06C	SPCC663.06c	0.6999	1.256	0.154964006	14.88	24.15	0.9974	2.925
SPCC777.03C	SPCC777.03c	0.751	1.258	0.124360063	18.66	30.84	2.003	3.497
SPBC336.01	fbh1	0.8058	1.26	0.093772737	10.76	16.84	5.632	3.726
SPAC1A6.01C	SPAC1A6.01c	0.7348	1.261	0.133830852	16.96	27.82	2.288	3.219
SPAPB17E12.05	rpl3703	0.8149	1.261	0.088895682	6.863	9.926	5.773	3.986
SPBC32H8.02C	nep2	0.6551	1.261	0.183692401	16.68	27.33	2.164	2.342
SPBC1685.15C	klp6	0.4867	1.264	0.312738654	15.89	25.92	1.136	1.523
SPAC6F6.09	eaf6	0.5632	1.265	0.249337354	13.49	21.66	1.207	1.88
SPAC1B3.03C	wis2	0.8124	1.269	0.090230085	18.69	30.86	2.326	4.779
SPAC3H8.10	spo20	0.5576	1.272	0.253677235	18.01	29.67	1.383	1.834
SPBC23G7.11	mag2	0.8376	1.273	0.076963331	16.44	26.88	3.713	5.416
SPAC19A8.11C	SPAC19A8.11c	0.6135	1.273	0.212185433	16.54	27.06	1.252	2.196
SPCC584.15C	SPCC584.15c	0.6858	1.275	0.163802519	14.84	24.05	3.438	2.284
SPAC1687.06C	rpl44	0.4853	1.276	0.313989709	15.64	25.45	1.72	1.399
SPBP4G3.03	SPBP4G3.03	0.841	1.276	0.075204004	20.08	33.32	3.009	5.662
SPBC428.07	meu6	0.7971	1.286	0.098487191	17.33	28.43	2.691	4.401
SPAC30C2.06C	dml1	0.5494	1.286	0.260111345	10.16	15.72	2.433	1.492
SPAC24H6.10C	SPAC24H6.10c	0.6998	1.292	0.155026062	16.94	27.74	2.122	2.878
SPBC24C6.11	cwf14	0.7593	1.293	0.1195866	8.539	12.84	4.338	3.187
SPBC36.06C	spo9	0.7513	1.293	0.124186611	17.17	28.14	1.912	3.617
SPBC1539.07C	SPBC1539.07c	0.7905	1.298	0.102098126	19.18	31.7	6.974	2.438

SPCC1682.12C	ubp16	0.6016	1.302	0.220692172	17.55	28.79	1.508	2.134
SPBC725.09C	hob3	0.8264	1.303	0.082809691	11.75	18.52	7.155	4.005
SPAP8A3.02C	ofd2	0.6725	1.304	0.172307711	15.49	25.14	4.292	1.623
SPCC132.01C	SPCC132.01c	0.6642	1.305	0.177701129	16.81	27.47	2.016	2.553
SPAC343.06C	SPAC343.06c	0.7981	1.305	0.097942689	17.39	28.51	2.86	4.47
SPCC24B10.15	SPCC24B10.15	0.8178	1.306	0.087352894	18.64	30.71	3.142	4.982
SPBC216.02	mcp5	0.5517	1.306	0.258297016	17.38	28.48	1.581	1.821
SPBC1683.09C	frp1	0.3253	1.307	0.487715937	9.645	14.78	1.046	1.036
SPAC29B12.03	spd1	0.3742	1.312	0.426896217	16.25	26.48	1.267	1.141
SPBC216.03	SPBC216.03	0.856	1.318	0.067526235	11.75	18.49	8.269	5.156
SPAC16A10.04	rho4	0.7973	1.319	0.098378236	19.67	32.52	2.926	4.495
SPAC9G1.03C	rpl3001	0.6695	1.319	0.174249419	7.581	11.1	2.795	2.454
SPAC167.01	ire1	0.6744	1.324	0.171082438	13.18	21.02	3.033	2.443
SPAC4G8.07C	SPAC4G8.07c	0.3569	1.325	0.447453452	15	24.23	1.528	1.007
SPBC2D10.12	rhp23	0.5354	1.327	0.271321633	11.43	17.91	0.9184	1.861
SPBC16D10.05	mok13	0.5831	1.328	0.234256959	16.97	27.73	2.092	1.931
SPBC17D11.08	SPBC17D11.08	0.3691	1.33	0.432855955	17.46	28.59	1.009	1.198
SPBC16C6.04	SPBC16C6.04	0.835	1.331	0.078313525	13.17	20.98	7.76	4.277
SPCC320.14	SPCC320.14	0.6657	1.331	0.176721443	18.57	30.55	3.392	2.217
SPAPB2C8.01	SPAPB2C8.01	0.238	1.332	0.623423043	16.38	26.67	0.3359	0.904
SPAPJ691.02	SPAPJ691.02	0.532	1.333	0.274088368	14.15	22.72	3.212	0.7081
SPBC646.09C	int6	0.7592	1.335	0.119643801	16.93	27.64	1.988	3.868
SPAC1952.11C	ure2	0.6706	1.336	0.173536451	17.11	27.96	1.616	2.748
SPAC6G9.09C	rpl2401	0.734	1.338	0.13430394	19.46	32.12	3.007	3.288
SPAC11D3.08C	SPAC11D3.08c	0.1465	1.341	0.834162375	12.68	20.1	1.148	0.4538
SPBP8B7.06	rpp201	0.649	1.345	0.187755303	16.88	27.53	1.936	2.517
SPBC887.18C	hfi1	0.1825	1.346	0.738737131	1.346	0	0	0.7789
SPAC5D6.07C	pxa1	0.6668	1.348	0.176004409	17.75	29.06	1.989	2.682
SPBC800.08	gcd10	0.7499	1.349	0.124996646	19.5	32.16	2.382	3.697
SPCC16C4.01	sif2	0.5527	1.352	0.257510535	16.52	26.88	3.211	1.111
SPBC365.11	SPBC365.11	0.7229	1.355	0.140921775	16.18	26.28	2.072	3.342
SPAC3G6.04	rnp24	0.7545	1.355	0.122340756	19.87	32.81	2.513	3.776
SPAC1002.02	pom34	0.5258	1.355	0.279179418	14.56	23.4	1.693	1.732
SPAC2G11.05C	SPAC2G11.05c	0.7508	1.356	0.124475736	18.82	30.94	2.82	3.662
SPBC651.10	nse5	0.668	1.36	0.175223538	7.72	11.27	3.801	2.122
SPAC23C11.07	SPAC23C11.07	0.6375	1.361	0.195519811	17.43	28.48	1.99	2.438
SPAC31A2.12	SPAC31A2.12	0.5928	1.367	0.227091805	12.94	20.51	3.74	1.096
SPCC569.05C	SPCC569.05c	0.736	1.369	0.133122186	17.41	28.42	1.646	3.623
SPBC947.10	dsc1	0.7136	1.372	0.146545159	8.019	11.78	4.17	2.673
SPCC5E4.07	rpl2802	0.5406	1.373	0.267123959	3.197	3.232	1.67	1.847
SPAC186.01	SPAC186.01	0.8193	1.383	0.086557045	18.25	29.9	3.064	5.357
SPAC19B12.12C	yip11	0.6746	1.384	0.170953663	17.05	27.76	1.636	2.893
SPAC513.05	ams1	0.4972	1.39	0.30346888	18.32	29.99	2.696	1.165
SPBC947.01	SPBC947.01	0.552	1.393	0.258060922	16.44	26.67	2.038	1.858
SPAC17A2.05	osm1	0.6261	1.394	0.203356296	14.61	23.42	2.273	2.348
SPBC18H10.05	SPBC18H10.05	0.8866	1.395	0.052272273	22.15	36.78	6.65	8.41
SPAC17H9.11	gmf1	0.6626	1.396	0.178748568	17.36	28.28	1.497	2.816
SPAC20G4.08	SPAC20G4.08	0.5758	1.4	0.239728339	15.74	25.42	0.892	2.204
SPCC548.07C	ght1	0.6812	1.401	0.166725361	13.87	22.1	3.113	2.699
SPBC1778.09	SPBC1778.09	0.6657	1.402	0.176721443	18.74	30.72	2.989	2.557
SPAC13C5.05C	SPAC13C5.05c	0.6516	1.404	0.186018924	16.52	26.79	1.849	2.68
SPBC1778.01C	zuo1	0.6028	1.405	0.219826756	8.675	12.88	2.822	2.098
SPBC32H8.13C	mok12	0.7218	1.405	0.141583122	15.23	24.5	5.626	1.953
SPAC22H10.04	SPAC22H10.04	0.782	1.408	0.106793247	18.9	31	3.284	4.385
SPBC2A9.07C	SPBC2A9.07c	0.477	1.412	0.321481621	16.77	27.22	1.803	1.537
SPAC8C9.03	cgs1	0.5685	1.415	0.245269531	8.173	11.98	2.658	1.898
SPCC1906.04	wtf20	0.7528	1.415	0.12332039	16.45	26.63	3.27	3.793
SPBP16F5.05C	SPBP16F5.05c	0.6744	1.421	0.171082438	11.85	18.48	3.743	2.426
SPAC1556.01C	rad50	0.6828	1.424	0.165706488	4.626	5.675	3.359	2.7
SPAC27D7.03C	mei2	0.6253	1.425	0.203911571	17.11	27.79	2.863	2.232
SPBC26H8.12	SPBC26H8.12	0.7749	1.429	0.110754339	4.07	4.68	4.262	4.07
SPAPB2B4.06	SPAPB2B4.06	0.6761	1.436	0.169989064	18.7	30.59	2.09	2.963
SPBC16A3.01	spn3	0.7991	1.439	0.097398869	18.57	30.36	3.638	4.88
SPAC1F5.07C	hem14	0.5283	1.44	0.277119389	6.795	9.491	2.358	1.682
SPAC1B3.07C	vps28	0.3642	1.44	0.438660059	8.475	12.47	0.9959	1.295
SPBC1105.14	rsv2	0.6955	1.441	0.157702866	18.48	30.2	1.639	3.258
SPAP7G5.05	rpl1002	0.5918	1.442	0.227825039	16.08	25.94	4.052	0.957
SPAC12B10.12C	rhp41	0.6327	1.444	0.198802166	15.65	25.18	3.007	2.296
SPAC2E1P3.05C	SPAC2E1P3.05c	0.6967	1.451	0.156954189	14.66	23.41	2.925	3.08

SPAC17H9.19C	cdt2	0.7291	1.452	0.137212902	8.859	13.13	4.707	2.987
SPCC1672.04C	SPCC1672.04c	0.4741	1.457	0.324130045	10.91	16.76	0.8464	1.753
SPBC428.11	SPBC428.11	0.811	1.461	0.090979146	11.9	18.5	6.428	4.569
SPAC25H1.07	emc1	0.7646	1.463	0.116565706	11.25	17.35	6.308	3.013
SPBC11C11.10	SPBC11C11.10	0.4301	1.465	0.366430557	10.52	16.05	1.74	1.409
SPBC1271.14	SPBC1271.14	0.6031	1.468	0.219610672	6.266	8.502	2.9	2.107
SPCC4G3.04C	coq5	0.6421	1.471	0.19239733	6.236	8.445	3.707	2.159
SPAC23C4.16C	atg15	0.6161	1.471	0.210348791	16.25	26.19	2.286	2.415
SPCC1827.02C	SPCC1827.02c	0.6618	1.472	0.179273237	7.31	10.35	3.488	2.512
SPBC2G2.13C	SPBC2G2.13c	0.7507	1.473	0.124533584	19.22	31.45	2.183	4.109
SPAC57A10.07	SPAC57A10.07	0.6205	1.474	0.207258214	17.54	28.47	1.015	2.641
SPBP4H10.13	rps2302	0.4788	1.474	0.319845858	14.32	22.76	2.988	0.9068
SPAC7D4.08	SPAC7D4.08	0.5494	1.48	0.260111345	14.73	23.48	2.097	1.976
SPAC1071.02	mms19	0.5334	1.482	0.272946989	10.02	15.13	3.134	1.365
SPBC19G7.06	mbx1	0.6229	1.482	0.205581669	14.64	23.32	3.243	2.192
SPCC1020.05	SPCC1020.05	0.7896	1.485	0.10259286	17.99	29.24	4.005	4.717
SPBC342.04	rpn1301	0.8356	1.488	0.078001569	18.7	30.5	3.879	6.317
SPBC17G9.07	rps2402	0.5928	1.494	0.227091805	17.18	27.8	1.825	2.365
SPBC776.05	SPBC776.05	0.837	1.495	0.077274542	15.04	24.01	8.079	5.252
SPBC16D10.01C	SPBC16D10.01c	0.5506	1.499	0.259163793	17.74	28.78	1.291	2.166
SPAC22G7.01C	SPAC22G7.01c	0.679	1.504	0.168130226	17.29	27.97	0.4857	3.289
SPAC29B12.05C	SPAC29B12.05c	0.3896	1.507	0.409381052	5.921	7.822	1.901	1.313
SPBC4F6.04	rpl2502	0.711	1.512	0.148130399	14.46	22.95	5.434	2.377
SPAC222.15	meu13	0.7182	1.513	0.143754599	17.42	28.18	2.895	3.561
SPCPB1C11.03	SPCPB1C11.03	0.007622	1.513	2.117931056	10.76	16.39	0.5299	0.1991
SPAC3H5.09C	SPAC3H5.09c	0.486	1.514	0.313363731	16.09	25.83	2.234	1.595
SPBC713.08	mim1	0.6697	1.516	0.174119701	7.647	10.87	3.874	2.575
SPBC428.04	apq12	0.7876	1.516	0.103694293	18.39	29.9	2.96	4.958
SPBC18H10.07	SPBC18H10.07	0.7471	1.517	0.126621264	10.18	15.36	5.162	3.412
SPAC3A11.06	mvp1	0.6965	1.517	0.157078879	16.83	27.13	0.7412	3.515
SPCC320.06	SPCC320.06	0.8072	1.518	0.093018847	16.85	27.17	3.976	5.373
SPAC513.04	SPAC513.04	0.8082	1.518	0.092481154	19.62	32.08	3.712	5.452
SPCC576.01C	SPCC576.01c	0.4962	1.522	0.30434324	14.95	23.8	1.695	1.823
SPBC29A3.10C	atp14	0.1865	1.523	0.729321164	1.523	2.62E-18	2.62E-18	0.8928
SPBC1709.11C	png2	0.5444	1.523	0.264081883	16.86	27.18	2.821	1.756
SPBC16H5.05C	cyp7	0.6456	1.527	0.190036478	14.93	23.75	2.053	2.848
SPAC664.04C	rps1602	0.647	1.528	0.189095719	17.89	28.99	1.796	2.901
SPAC21E11.05C	cyp8	0.7529	1.528	0.123262703	18.5	30.08	2.343	4.295
SPAC869.03C	SPAC869.03c	0.451	1.53	0.345823458	16.5	26.52	1.542	1.641
SPBC16C6.01C	SPBC16C6.01c	0.6173	1.532	0.209503723	9.616	14.33	3.3	2.344
SPBC4B4.12C	SPBC4B4.12c	0.5766	1.532	0.239125362	17.38	28.09	0.5663	2.442
SPBC1A4.03C	top2	0.6387	1.533	0.194703084	18.99	30.93	3.518	2.378
SPBC1347.02	fkbp39	0.4762	1.534	0.322210609	16.34	26.23	3.163	0.8175
SPBC2D10.16	mhf1	0.6204	1.535	0.207328211	17.45	28.2	0.9355	2.76
SPBC21.02	SPBC21.02	0.6775	1.537	0.1690907	8.066	11.57	3.999	2.692
SPCP20C8.01C	SPCP20C8.01c	0.6354	1.54	0.19695279	16.5	26.52	3.928	2.145
SPBC106.17C	cys2	0.1905	1.541	0.72010502	8.349	12.06	0.6887	0.9025
SPAC22A12.03C	csn4	0.597	1.543	0.224025669	18.27	29.64	1.831	2.484
SPAC23A1.16C	rtr1	0.7606	1.544	0.118843679	18.29	29.68	2.259	4.507
SPCC338.14	SPCC338.14	0.6135	1.545	0.212185433	17.61	28.46	2.37	2.52
SPAC22E12.03C	SPAC22E12.03c	0.5567	1.549	0.254378779	13.61	21.38	2.283	2.096
SPCC31H12.05C	sds21	0.6979	1.55	0.156206802	21.87	36.01	1.691	3.544
SPAC11E3.11C	SPAC11E3.11c	0.5763	1.552	0.23935138	10.56	15.97	2.807	2.083
SPBP8B7.21	ubp3	0.5075	1.56	0.294563953	16.81	27.02	1.636	1.951
SPAC13D6.01	pof14	0.3873	1.562	0.411952503	16.25	26.02	2.16	1.15
SPAC4G9.19	SPAC4G9.19	0.4613	1.563	0.336016545	14.97	23.77	1.611	1.719
SPBC18H10.08C	ubp4	0.6418	1.565	0.192600287	15.95	25.49	2.885	2.709
SPBC582.04C	SPBC582.04c	0.6722	1.569	0.172501492	16.3	26.11	2.021	3.228
SPAC31G5.07	dni1	0.7941	1.569	0.100124804	18.55	30.09	2.83	5.341
SPCC1795.12C	SPCC1795.12c	0.6516	1.573	0.186018924	18.64	30.24	1.374	3.087
SPBC1105.11C	hht3	0.5006	1.578	0.300509155	17.14	27.57	2.133	1.819
SPBC365.01	SPBC365.01	0.8211	1.58	0.085603948	18.87	30.65	4.26	6.076
SPCC550.03C	SPCC550.03c	0.6998	1.581	0.155026062	13.77	21.59	3.458	3.334
SPBC3E7.11C	SPBC3E7.11c	0.7404	1.582	0.13053359	18.09	29.25	3.627	3.994
SPBC776.11	rpl2801	0.73	1.583	0.13667714	12.25	18.91	4.135	3.661
SPAC18G6.02C	chp1	0.6739	1.584	0.171404544	11.83	18.17	2.657	3.176
SPBC725.14	arg6	0.4495	1.587	0.347270304	9.831	14.61	0.9779	1.787
SPCC11E10.09C	SPCC11E10.09c	0.749	1.595	0.125518182	16.76	26.87	3.144	4.299
SPCC1672.03C	SPCC1672.03c	0.3527	1.599	0.45259454	17.28	27.8	0.9183	1.419

SPAC1296.05C	SPAC1296.05c	0.3334	1.601	0.477034405	17.06	27.4	1.014	1.342
SPCC777.12C	SPCC777.12c	0.6667	1.601	0.176069545	16.42	26.27	0.8161	3.345
SPAC3A12.10	rpl2001	0.3725	1.601	0.428873723	9.195	13.46	2.323	1.007
SPAC14C4.03	mek1	0.5996	1.603	0.222138376	16.42	26.26	2.48	2.484
SPAC22A12.17C	SPAC22A12.17c	0.5907	1.604	0.228633029	10.38	15.55	4.443	1.151
SPBC1734.08	hse1	0.3617	1.605	0.441651491	15.06	23.84	1.634	1.321
SPCC1259.01C	rps1802	0.5897	1.608	0.229368872	5.51	6.916	3.134	2.191
SPCC330.02	rhp7	0.5277	1.613	0.277612906	16.22	25.89	0.819	2.239
SPAC17H9.01	cid16	0.6804	1.613	0.167235695	17.23	27.67	0.543	3.542
SPAC30.01C	sec72	0.7755	1.614	0.110418198	21.47	35.18	5.495	4.398
SPCC825.01	SPCC825.01	0.5106	1.617	0.29191919	16.45	26.29	2.939	1.612
SPCC16A11.15C	SPCC16A11.15c	0.4412	1.618	0.355364496	16	25.48	2.176	1.523
SPBC1718.03	ker1	0.5034	1.622	0.298086789	5.148	6.25	2.446	1.802
SPAC328.10C	rps502	0.4002	1.625	0.397722916	13.89	21.74	2.816	0.6922
SPBC1921.01C	rpl35b	0.6797	1.628	0.16768273	16.65	26.63	2.14	3.437
SPAC17A5.07C	ulp2	0.6355	1.628	0.196884445	17.77	28.61	3.072	2.727
SPCC1442.03	SPCC1442.03	0.5628	1.628	0.249645911	16.61	26.55	2.4	2.25
SPAC26A3.10	cnt6	0.468	1.631	0.329754147	15.44	24.47	2.481	1.57
SPAC19D5.03	cid1	0.6623	1.631	0.178945245	15.53	24.63	0.9066	3.354
SPAC630.07C	SPAC630.07c	0.5314	1.633	0.27457845	15.28	24.19	2.985	1.791
SPBC543.08	SPBC543.08	0.67	1.634	0.173925197	18.14	29.26	1.284	3.424
SPBC1604.03C	SPBC1604.03c	0.4787	1.634	0.319936573	17.32	27.8	2.111	1.782
SPAP27G11.08C	meu32	0.5569	1.635	0.254222782	16.56	26.44	1.184	2.429
SPBC14C8.17C	spt8	0.3688	1.636	0.433209088	13.08	20.28	2.334	1.037
SPAC694.04C	SPAC694.04c	0.5653	1.636	0.247721015	17.8	28.65	2.719	2.188
SPBC31F10.09C	nut2	0.6428	1.641	0.191924132	7.492	10.37	3.533	2.689
SPBC3E7.06C	fnx2	0.7709	1.641	0.113001954	18.04	29.06	3.568	4.866
SPAC1D4.05C	SPAC1D4.05c	0.666	1.643	0.176525771	18.14	29.24	3.246	3.077
SPBC12C2.08	dnm1	0.5365	1.644	0.270430274	15.44	24.45	2.657	1.996
SPCC306.09C	cap1	0.2275	1.648	0.643018599	11.32	17.14	1.807	0.6284
SPCC24B10.19C	SPCC24B10.19c	0.5997	1.648	0.222065951	18.44	29.76	1.937	2.679
SPBC1711.12	SPBC1711.12	0.3022	1.652	0.51970554	19.12	30.95	2.052	0.8806
SPAC4A8.07C	SPAC4A8.07c	0.6359	1.653	0.196611175	19.03	30.79	1.754	3.049
SPBC13E7.06	msd1	0.5519	1.656	0.258139606	17.33	27.78	0.8076	2.455
SPBC16A3.17C	SPBC16A3.17c	0.5306	1.658	0.275232754	15.31	24.2	2.616	1.991
SPBC342.03	gas4	0.3942	1.66	0.40428338	17.17	27.49	1.477	1.557
SPAPB8E5.10	SPAPB8E5.10	0.726	1.66	0.139063379	18.33	29.54	1.753	4.238
SPAC19D5.11C	ctf8	0.5105	1.66	0.292004254	15.8	25.06	2.106	2.015
SPAC22F8.09	rrp16	0.6435	1.661	0.191451449	16.18	25.73	1.584	3.16
SPBC1105.02C	lys4	0.2725	1.67	0.564633493	9.607	14.06	0.5169	1.239
SPAC9G1.06C	cyk3	0.1388	1.672	0.857610534	18.32	29.5	1.03	0.7744
SPBC530.07C	SPBC530.07c	0.572	1.674	0.242603971	6.748	8.992	3.22	2.127
SPAC11E3.14	SPAC11E3.14	0.3982	1.676	0.399898744	15.39	24.3	2.161	1.379
SPAC22F8.05	SPAC22F8.05	0.7096	1.677	0.148986393	14.13	22.07	4.292	3.515
SPAC19D5.01	pyp2	0.5303	1.682	0.275478373	15.35	24.22	1.757	2.249
SPBC4B4.06	vps25	0.5346	1.683	0.271971046	15.62	24.7	4.234	0.3867
SPAC977.05C	SPAC977.05c	0.503	1.684	0.298432015	14.52	22.74	2.568	1.857
SPCC338.18	SPCC338.18	0.5147	1.688	0.288445832	17.83	28.61	0.7373	2.272
SPBC1271.08C	SPBC1271.08c	0.6478	1.691	0.188559056	18.96	30.61	1.746	3.249
SPAC1805.06C	hem2	0.09782	1.691	1.009572342	15.79	24.99	1.088	0.6066
SPBC16G5.11C	bag101	0.61	1.697	0.214670165	17.14	27.38	1.442	2.922
SPAC3H1.06C	SPAC3H1.06c	0.5224	1.7	0.281996832	15.2	23.93	3.289	1.677
SPBC1709.01	chs2	0.5874	1.7	0.231066058	15.31	24.12	2.457	2.568
SPAC6C3.05	SPAC6C3.05	0.5743	1.703	0.240861184	16.68	26.54	1.91	2.572
SPBC800.12C	SPBC800.12c	0.7347	1.708	0.13388996	18.29	29.38	2.701	4.418
SPAC5H10.07	SPAC5H10.07	0.7755	1.71	0.110418198	19.86	32.15	2.58	5.35
SPBC19G7.17	SPBC19G7.17	0.5859	1.714	0.232176502	13.85	21.5	2.983	2.433
SPAC13A11.01C	rga8	0.7092	1.716	0.149231273	17.26	27.54	3.897	3.738
SPBC16D10.11C	rps1801	0.7406	1.719	0.130416292	11.4	17.15	5.773	3.731
SPAC26F1.08C	SPAC26F1.08c	0.6338	1.72	0.198047765	16.98	27.05	1.504	3.185
SPBC21C3.12C	SPBC21C3.12c	0.7206	1.721	0.142305742	12.5	19.1	5.697	3.45
SPBC887.17	SPBC887.17	0.7874	1.726	0.10380459	19.08	30.75	3.66	5.598
SPBC646.08C	SPBC646.08c	0.2278	1.726	0.64244628	14.89	23.34	0.6848	1.131
SPAC30C2.02	mmd1	0.5461	1.727	0.262727823	15.1	23.69	2.577	2.244
SPAC3C7.08C	elf1	0.7076	1.729	0.150212176	11.53	17.36	5.839	2.906
SPAC343.10	met11	0.3915	1.731	0.407268234	9.928	14.53	0.6825	1.716
SPBC359.01	SPBC359.01	0.5447	1.732	0.263842625	14.68	22.94	3.17	2.018
SPBC23G7.13C	SPBC23G7.13c	0.4975	1.733	0.303206915	15.43	24.28	1.855	2.099
SPAC328.05	SPAC328.05	0.7692	1.734	0.113960724	19.13	30.83	1.915	5.327

SPBP8B7.28C	stc1	0.6004	1.736	0.221559316	14.48	22.59	3.438	2.458
SPBC3H7.05C	SPBC3H7.05c	0.4086	1.738	0.388701638	15.07	23.62	1.75	1.652
SPAC521.02	SPAC521.02	0.3098	1.742	0.508918587	13.75	21.28	1.813	1.187
SPAC15A10.07	SPAC15A10.07	0.5531	1.747	0.257196342	14.17	22.01	3.429	1.999
SPAC521.04C	SPAC521.04c	0.7034	1.748	0.152797636	16.87	26.79	4.183	3.642
SPCC23B6.04C	SPCC23B6.04c	0.5476	1.748	0.261536561	17.47	27.86	1.392	2.517
SPAP27G11.15	slx1	0.7607	1.75	0.118786584	18.85	30.3	3.329	5.011
SPBC23G7.07C	SPBC23G7.07c	0.5661	1.753	0.247106845	14.8	23.11	1.931	2.588
SPAC13G6.12C	chs1	0.4444	1.754	0.35222595	16.27	25.72	1.507	1.896
SPAC29A4.02C	SPAC29A4.02c	0.526	1.755	0.279014256	17.43	27.77	2.898	2.022
SPCC11E10.01	SPCC11E10.01	0.6042	1.759	0.218819279	13.67	21.11	4.456	1.99
SPAC2C4.16C	rps801	0.685	1.762	0.164309429	8.971	12.78	4.366	3.303
SPBC28F2.05C	SPBC28F2.05c	0.6566	1.762	0.182699122	16.53	26.17	0.9283	3.56
SPCP31B10.02	SPCP31B10.02	0.7497	1.763	0.125112489	20.04	32.39	2.245	4.935
SPBC4F6.09	str1	0.6959	1.766	0.157453164	18.23	29.18	1.754	4.026
SPAC922.05C	SPAC922.05c	0.1703	1.771	0.768785352	14.31	22.22	1.538	0.727
SPCC70.10	SPCC70.10	0.7169	1.771	0.14454142	16.75	26.54	6.751	2.634
SPAC23C11.10	SPAC23C11.10	0.5597	1.773	0.252044693	16.74	26.52	2.201	2.522
SPCC18.09C	SPCC18.09c	0.7481	1.775	0.126040345	18.03	28.81	2.8	4.871
SPBC543.09	yta12	0.5019	1.775	0.299382804	16.89	26.78	1.192	2.28
SPBC409.20C	psh3	0.6681	1.777	0.175158528	8.668	12.21	4.246	3.115
SPBC4.01	dni2	0.5259	1.779	0.279096829	18.18	29.07	2.232	2.273
SPAC4F8.08	mug114	0.7595	1.78	0.119472222	20.22	32.68	1.905	5.237
SPAC2F7.07C	cph2	0.6019	1.782	0.220475657	5.929	7.348	4.299	2.142
SPBC557.05	SPBC557.05	0.7552	1.785	0.121938019	18.62	29.84	3.156	5.014
SPAC22A12.02C	mug103	0.7985	1.786	0.09772508	22.07	35.95	4.136	6.101
SPAC12G12.11C	SPAC12G12.11c	0.5266	1.787	0.278519145	15.27	23.89	2.121	2.315
SPAC22G7.02	kap111	0.4414	1.789	0.355167671	20.83	33.74	1.697	1.89
SPAC27E2.09	mak2	0.7034	1.794	0.152797636	17.75	28.27	2.212	4.161
SPBC354.09C	SPBC354.09c	0.5845	1.795	0.233215485	18.05	28.81	2.568	2.689
SPAC11D3.16C	SPAC11D3.16c	0.1941	1.798	0.711974465	16.82	26.63	1.497	0.8929
SPCC70.03C	SPCC70.03c	0.7383	1.801	0.131767132	17.49	27.8	3.172	4.685
SPBC23E6.10C	SPBC23E6.10c	0.05649	1.803	1.248028425	17.44	27.7	1.105	0.3675
SPAC3A12.17C	cys12	0.2161	1.806	0.665345233	16.94	26.83	0.4426	1.155
SPCC4B3.04C	nte1	0.7726	1.814	0.112045296	19.05	30.54	3.292	5.53
SPAC2F3.02	SPAC2F3.02	0.4681	1.815	0.329661359	15.78	24.75	2.32	1.919
SPBC4F6.16C	ero11	0.3183	1.816	0.497163361	15.15	23.64	1.922	1.264
SPBC651.12C	SPBC651.12c	0.7101	1.819	0.148680487	16.3	25.66	2.386	4.313
SPAC589.11	mug82	0.567	1.821	0.246416941	14.82	23.04	0.7479	2.822
SPAC1783.01	SPAC1783.01	0.5141	1.825	0.288952396	17.61	27.98	2.298	2.244
SPBC9B6.03	SPBC9B6.03	0.7232	1.825	0.140741583	16.15	25.38	3.384	4.412
SPAC17C9.14	SPAC17C9.14	0.4984	1.825	0.302421966	14.77	22.94	3.617	1.475
SPAC11E3.03	pcs1	0.5176	1.826	0.286005732	13.84	21.3	4.056	1.256
SPAC14C4.05C	man1	0.3427	1.836	0.465085896	16.18	25.42	1.864	1.423
SPAC4A8.10	SPAC4A8.10	0.2585	1.837	0.587539453	14.95	23.24	1.682	1.119
SPCC1450.06C	grx3	0.7806	1.841	0.107571453	18.04	28.7	4.074	5.732
SPCC16A11.07	coq10	0.3604	1.846	0.443215218	15.04	23.39	1.872	1.514
SPBP35G2.04C	SPBP35G2.04c	0.7861	1.851	0.104522204	15.1	23.48	8.039	4.688
SPBC24C6.04	SPBC24C6.04	0.5401	1.854	0.267525823	12.68	19.19	3.213	2.197
SPBC336.05C	SPBC336.05c	0.3854	1.857	0.41408829	15.47	24.13	1.251	1.768
SPAC3H5.12C	rpl501	0.6995	1.862	0.155212281	9.828	14.12	5.109	3.568
SPAC3A11.09	sod22	0.3073	1.863	0.51243744	17.44	27.6	2.207	1.11
SPAC4F10.19C	SPAC4F10.19c	0.6113	1.873	0.213745604	16.44	25.81	0.6143	3.301
SPAC1071.11	SPAC1071.11	0.4975	1.875	0.303206915	9.225	13.03	3.782	1.438
SPAC323.07C	SPAC323.07c	0.6368	1.875	0.195996945	12.94	19.61	4.485	2.944
SPAC23C11.01	SPAC23C11.01	0.3724	1.879	0.428990328	16.15	25.29	1.832	1.621
SPCC285.17	spp27	0.5623	1.881	0.250031916	15.64	24.38	1.95	2.766
SPCC1393.08	SPCC1393.08	0.5736	1.883	0.241390857	7.68	10.27	3.14	2.594
SPAC1556.06.1	meu1-1	0.2582	1.885	0.588043762	15.18	23.57	1.546	1.212
SPAC8C9.04	SPAC8C9.04	0.7339	1.887	0.134363112	11.63	17.26	6.272	3.941
SPAC824.04	SPAC824.04	0.6505	1.892	0.186752699	21.43	34.63	0.5352	3.765
SPAC1782.07	qcr8	0.5989	1.894	0.222645687	12.54	18.87	3.45	2.775
SPAC4F10.02	aap1	0.3384	1.894	0.470569646	16.95	26.67	1.5	1.56
SPAC27D7.14C	tpr1	0.4468	1.896	0.349886836	8.849	12.32	2.758	1.854
SPBC19C2.04C	ubp11	0.434	1.898	0.36251027	15.37	23.87	2.563	1.736
SPBC1D7.03	mug80	0.5324	1.9	0.273761953	12.05	17.99	3.206	2.218
SPBC1604.20C	tea2	0.5521	1.9	0.257982253	12.4	18.61	2.584	2.588
SPBC16E9.06C	uvi31	0.6292	1.901	0.201211286	17.03	26.81	1	3.52
SPAC3C7.03C	rhp55	0.5593	1.905	0.252355181	15.38	23.88	1	2.878

SPAC13D6.03C	trm9	0.513	1.905	0.289882635	17.95	28.44	1.605	2.482
SPAC1F8.05	isp3	0.539	1.906	0.268411235	16.96	26.67	3.789	1.997
SPAC26F1.02	pnn1	0.7811	1.907	0.107293362	20.79	33.46	3.49	6.061
SPAC22F3.06C	lon1	0.263	1.911	0.580044252	15.03	23.25	2.218	0.8772
SPBPB10D8.04C	SPBPB10D8.04c	0.524	1.912	0.280668713	18.39	29.2	1.138	2.617
SPAC1952.02	SPAC1952.02	0.7865	1.914	0.104301273	13.7	20.88	7.063	5.425
SPAC6B12.02C	mus7	0.6275	1.918	0.20238627	18.79	29.91	0.3252	3.557
SPAC328.03	tps1	0.4572	1.923	0.339893778	4.583	4.714	3.156	1.639
SPAC1952.09C	SPAC1952.09c	0.7602	1.929	0.119072135	21.33	34.37	3.302	5.561
SPBC27B12.04C	SPBC27B12.04c	0.4277	1.929	0.36886075	17.53	27.64	1.198	2.056
SPAC3F10.06C	SPAC3F10.06c	0.7388	1.932	0.131473113	17.19	27.03	3.657	4.997
SPAP32A8.03C	SPAP32A8.03c	0.5954	1.935	0.22519117	17.04	26.77	1.195	3.227
SPCC126.15C	sec65	0.7686	1.937	0.11429962	11.24	16.48	8.575	4.009
SPAC8F11.05C	mug130	0.6127	1.94	0.21275212	18.01	28.47	1.72	3.361
SPAC23C4.06C	SPAC23C4.06c	0.8054	1.944	0.093988375	24.01	39.1	4.348	6.931
SPAC23H4.17C	srb10	0.1882	1.946	0.725380381	3.144	2.123	1.275	1.068
SPAC1039.09	isp5	0.3583	1.948	0.445753192	13.41	20.32	2.484	1.367
SPCC285.14	trs130	0.6796	1.953	0.16774663	10.07	14.38	4.96	3.513
SPAC29E6.07	SPAC29E6.07	0.6899	1.958	0.161213855	18.87	29.96	1.008	4.431
SPAC4F8.03	sdo1	0.2773	1.96	0.55705013	2.452	0.8725	0.5797	1.642
SPAC1093.01	ppr5	0.7208	1.961	0.142185222	16.45	25.67	3.767	4.667
SPAC1F12.04C	SPAC1F12.04c	0.6612	1.961	0.179667155	18.11	28.63	1.356	4.003
SPCC736.04C	gma12	0.3397	1.962	0.468904453	17.2	27.01	2.144	1.447
SPAC22G7.07C	SPAC22G7.07c	0.613	1.963	0.212539525	11.3	16.55	5.553	1.841
SPAC222.04C	ies6	0.6331	1.963	0.198527686	8.898	12.29	4.136	3.112
SPCC1223.06	tea1	0.5141	1.965	0.288952396	13.27	20.04	2.909	2.289
SPAC6F6.03C	SPAC6F6.03c	0.5015	1.969	0.299729063	19.11	30.37	2.202	2.393
SPAC823.14	ptf1	0.7247	1.969	0.139841739	20.33	32.54	2.731	4.933
SPAC227.13C	isu1	0.7446	1.971	0.128076968	18.45	29.2	3.917	5.198
SPAC23H4.10C	thi4	0.5679	1.974	0.245728131	12.03	17.82	1.929	2.968
SPBC3H7.09	erf2	0.6814	1.978	0.166597871	12.46	18.57	5.526	3.363
SPBC1734.13	atp3	0.4297	1.982	0.366834646	16.81	26.28	2.998	1.618
SPCC4B3.15	mid1	0.6068	1.982	0.216954428	16.26	25.31	4.386	2.683
SPAC1834.03C	hhf1	0.2228	1.983	0.652084813	17.19	26.95	1.204	1.236
SPAC2G11.09	SPAC2G11.09	0.5809	1.983	0.235898624	16.29	25.35	2.339	3.039
SPAPB8E5.06C	rpl302	0.3841	1.985	0.415555693	15.85	24.57	2.348	1.638
SPBPB2B2.05	SPBPB2B2.05	0.5204	1.985	0.283662712	18.5	29.26	1.4	2.671
SPCC576.11	rpl15	0.6741	1.99	0.171275673	7.476	9.722	5.626	3.195
SPAC22H10.03C	kap114	0.7139	1.992	0.146362618	17.51	27.5	2.375	4.822
SPBC28F2.07	sfr1	0.6989	1.993	0.15558496	20.2	32.27	2.334	4.558
SPBC11C11.02	imp2	0.2839	1.997	0.546834607	17.47	27.42	2.248	1.123
SPAC30D11.14C	SPAC30D11.14c	0.6949	2	0.158077688	16.5	25.7	2.578	4.476
SPBC3E7.08C	rad13	0.1656	2	0.780939668	18.38	29.02	1.118	1.054
SPAC3G9.08	png1	0.4292	2.002	0.367340287	8.322	11.2	2.643	1.823
SPBC20F10.10	psl1	0.4812	2.002	0.317674381	15.73	24.33	1.766	2.382
SPBC13G1.12	did2	0.3532	2.002	0.451979305	15.61	24.12	1.862	1.655
SPBC3E7.12C	chr1	0.5332	2.004	0.273109859	17.91	28.19	2.211	2.681
SPCC777.02	SPCC777.02	0.7084	2.004	0.149721447	18.44	29.12	3.041	4.663
SPAC4A8.06C	SPAC4A8.06c	0.217	2.005	0.663540266	15.5	23.92	1.468	1.171
SPBC19C2.10	SPBC19C2.10	0.5642	2.011	0.248566918	17.81	27.99	2.02	2.983
SPBC18E5.07	SPBC18E5.07	0.4101	2.012	0.387110231	19.14	30.36	2.564	1.746
SPCC18.06C	caf1	0.4197	2.02	0.377061031	16.74	26.09	2.513	1.838
SPAC1805.16C	SPAC1805.16c	0.3846	2.023	0.41499072	16.11	24.97	2.024	1.795
SPBC19F8.04C	SPBC19F8.04c	0.1937	2.023	0.712870379	15.41	23.72	1.62	1.034
SPCC1919.01	ppk34	0.4992	2.023	0.301725423	16.61	25.85	3.098	2.193
SPAC32A11.02C	SPAC32A11.02c	0.5367	2.023	0.270268405	16.29	25.28	1.06	2.875
SPAC1093.02	SPAC1093.02	0.291	2.025	0.536107011	17.33	27.12	0.8171	1.565
SPAC14C4.16	dad3	0.0711	2.026	1.148130399	17.35	27.15	1.153	0.6578
SPCC550.12	arp6	0.4745	2.028	0.323763783	17.22	26.93	1.675	2.387
SPBC12D12.02C	cdm1	0.592	2.033	0.227678293	18.55	29.27	0.6992	3.387
SPAC24C9.02C	SPAC24C9.02c	0.6514	2.034	0.186152246	19.47	30.9	1.374	4.022
SPCC1672.09	SPCC1672.09	0.3973	2.043	0.400881435	16.05	24.83	2.95	1.498
SPAC25H1.09	mde5	0.5347	2.043	0.271889816	16.82	26.19	1.158	2.881
SPAC688.13	scn1	0.5621	2.045	0.250186415	17.02	26.54	2.781	2.878
SPAC1F5.09C	shk2	0.6514	2.049	0.186152246	19.53	30.98	1.898	4.008
SPAC20G8.02	SPAC20G8.02	0.5106	2.054	0.29191919	16.81	26.16	0.7239	2.743
SPBC3H7.11	SPBC3H7.11	0.5952	2.058	0.225337077	18.46	29.07	1.273	3.429
SPAC11D3.10	SPAC11D3.10	0.7175	2.058	0.144178095	19.07	30.14	3.479	4.914
SPAC5H10.10	SPAC5H10.10	0.6017	2.06	0.220619989	19.1	30.2	2.08	3.42

SPAC1687.15	gsk3	0.4863	2.062	0.31309573	16.12	24.91	1.852	2.483
SPAC27F1.05C	SPAC27F1.05c	0.01661	2.066	1.779630368	18.89	29.82	0.8744	0.3441
SPBP8B7.26	SPBP8B7.26	0.3263	2.066	0.486382926	16.52	25.62	1.076	1.727
SPBPB10D8.05C	SPBPB10D8.05c	0.7215	2.07	0.141763665	18.84	29.72	3.332	5.054
SPAC31A2.16	gef2	0.3606	2.075	0.442974278	15.5	23.79	1.99	1.736
SPBC3D6.10	apn2	0.5803	2.075	0.236347429	14.63	22.24	2.361	3.187
SPBC646.17C	dic1	0.5965	2.078	0.224389552	17.65	27.6	2.142	3.389
SPBC342.05	crb2	0.2449	2.078	0.611011215	18.11	28.41	2.015	1.144
SPAC6F12.06	SPAC6F12.06	0.2331	2.081	0.632457726	16.37	25.33	1.043	1.366
SPAC12B10.15C	SPAC12B10.15c	0.6851	2.084	0.164246032	18.85	29.7	3.217	4.417
SPBC83.11	SPBC83.11	0.6718	2.084	0.17276	19.98	31.71	3.81	4.076
SPBC4.05	mlo2	0.6572	2.085	0.182302445	18.07	28.33	0.9025	4.23
SPAC1F7.01C	spt6	0.3243	2.086	0.489053051	15.2	23.24	0.6692	1.762
SPBC1198.03C	SPBC1198.03c	0.6064	2.091	0.217240807	17.95	28.1	2.275	3.5
SPAC2C4.09	SPAC2C4.09	0.3124	2.104	0.505288975	17.7	27.64	2.363	1.364
SPAC1687.08	SPAC1687.08	0.2634	2.105	0.579384229	5.149	5.393	2.322	1.084
SPCC162.01C	SPCC162.01c	0.769	2.109	0.11407366	19.87	31.48	3.823	6.313
SPAC1142.02C	SPAC1142.02c	0.1625	2.124	0.789146635	17.23	26.78	1.514	1.018
SPBC3E7.07C	SPBC3E7.07c	0.699	2.127	0.155522824	17.99	28.11	2.459	4.868
SPBC336.13C	SPBC336.13c	0.5317	2.13	0.27433334	7.323	9.202	3.144	2.643
SPBC215.08C	arg4	0.2215	2.133	0.654626269	7.229	9.03	1.978	1.095
SPCC18B5.03	wee1	0.5085	2.133	0.293709043	12.68	18.68	1.724	2.756
SPBC1861.05	SPBC1861.05	0.5594	2.134	0.252277538	5.177	5.392	2.822	2.995
SPAC18G6.01C	SPAC18G6.01c	0.2223	2.135	0.653060537	15	22.79	2.25	0.882
SPAC29A4.18	prw1	0.6603	2.149	0.180258703	15.96	24.48	4.491	4.036
SPAC4G9.06C	chz1	0.2224	2.154	0.652865217	15.41	23.5	1.935	1.144
SPAC22A12.11	dak1	0.6503	2.157	0.186886246	18.02	28.12	1.91	4.214
SPAC343.11C	msc1	0.3463	2.157	0.460547508	18.1	28.25	1.224	1.886
SPBC32H8.05	SPBC32H8.05	0.4962	2.161	0.30434324	17.8	27.72	1.233	2.752
SPAC323.01C	pos5	0.2405	2.166	0.618884919	2.503	0.5976	0.3942	1.483
SPBC354.04	SPBC354.04	0.7216	2.167	0.141703475	19.38	30.51	3.68	5.264
SPAC23D3.12	SPAC23D3.12	0.08372	2.17	1.07717078	12.44	18.2	1.12	0.8193
SPAC19G12.03	cda1	0.6391	2.171	0.194431182	19.48	30.68	2.601	4.006
SPAC9E9.12C	ybt1	0.3715	2.173	0.430041182	15.89	24.31	1.559	1.983
SPAC6G9.01C	SPAC6G9.01c	0.5198	2.173	0.284163725	15.5	23.62	4.3	2.046
SPCC4G3.10C	rhp42	0.5646	2.174	0.248259126	18.25	28.5	2.682	3.143
SPAC19D5.02C	SPAC19D5.02c	0.3969	2.176	0.401318901	16.72	25.78	1.229	2.159
SPAC1783.08C	rpl1502	0.7786	2.187	0.108685601	10.03	13.9	8.054	5.859
SPCC790.03	SPCC790.03	0.6096	2.187	0.214955042	9.497	12.95	4.431	3.184
SPAC23G3.12C	SPAC23G3.12c	0.5913	2.199	0.228192121	18.61	29.08	2.353	3.518
SPAC25B8.18	SPAC25B8.18	0.791	2.204	0.101823517	23.96	38.56	5.059	7.224
SPAC23C4.02	crn1	0.6793	2.205	0.167938385	20.06	31.65	4.269	4.39
SPBC2G2.10C	mug110	0.5196	2.206	0.284330858	19.8	31.19	2.503	2.988
SPAC732.02C	SPAC732.02c	0.4061	2.21	0.391367011	20.63	32.65	1.746	2.181
SPAC27D7.06	SPAC27D7.06	0.1763	2.217	0.753747688	16.83	25.89	2.028	0.861
SPAC922.04	SPAC922.04	0.7469	2.222	0.126737541	17.61	27.27	5.949	5.576
SPAC3G6.13C	rpl4101	0.3476	2.224	0.458920232	18.32	28.53	2.3	1.737
SPBC409.16C	SPBC409.16c	0.4753	2.233	0.323032186	17.8	27.59	2.877	2.411
SPBP4H10.12	SPBP4H10.12	0.5269	2.235	0.278271801	19.04	29.78	0.3616	3.129
SPCC1322.02	SPCC1322.02	0.5482	2.237	0.261060969	17.68	27.37	1.96	3.206
SPBC21C3.18	spo4	0.3077	2.238	0.511872504	14.59	21.9	2.152	1.583
SPBC1105.09	ubc15	0.2247	2.238	0.648396928	14.83	22.31	0.8363	1.456
SPAPB21F2.02	SPAPB21F2.02	0.7255	2.238	0.139362583	21.54	34.2	3.294	5.602
SPAC31A2.06	atp25	0.6606	2.242	0.180061431	19.14	29.95	2.02	4.53
SPAC8C9.11	SPAC8C9.11	0.5231	2.243	0.28141528	18.61	29.01	2.529	2.902
SPBC409.10	ade7	0.6085	2.243	0.215739417	15.36	23.24	2.744	3.734
SPCC965.11C	SPCC965.11c	0.3352	2.248	0.47469599	14.76	22.18	3.335	0.851
SPBC725.02	mpr1	0.6382	2.25	0.1950432	10.19	14.06	5.092	3.512
SPBC4B4.03	rsc1	0.6214	2.253	0.206628751	19.14	29.92	2.443	3.957
SPBC1861.09	ppk22	0.5622	2.254	0.250109159	17.24	26.56	4.169	2.816
SPCC825.04C	naa40	0.6417	2.256	0.192667961	17.36	26.76	2.726	4.197
SPAC4F10.05C	SPAC4F10.05c	0.1121	2.262	0.950394387	4.692	4.306	1.549	0.8443
SPAC8E11.10	SPAC8E11.10	0.2184	2.263	0.660747366	14.67	21.99	1.827	1.273
SPBC1773.09C	mug184	0.3894	2.264	0.409604053	15.76	23.91	1.427	2.189
SPAC31A2.11C	cuf1	0.7589	2.268	0.119815447	21.4	33.9	3.562	6.54
SPBPB2B2.09C	SPBPB2B2.09c	0.5856	2.268	0.232398932	18.31	28.44	3.241	3.412
SPBC18E5.13	SPBC18E5.13	0.3	2.269	0.522878745	17.81	27.54	0.858	1.798
SPBC1604.12	SPBC1604.12	0.6311	2.275	0.19990182	18.84	29.36	1.851	4.19
SPCC1494.01	SPCC1494.01	0.6699	2.275	0.173990022	18.33	28.46	2.281	4.722

SPBC365.08C	SPBC365.08c	0.1666	2.276	0.778325003	16.47	25.15	1.484	1.156
SPAC14C4.09	agn1	0.5049	2.277	0.296794629	12.86	18.76	3.31	2.587
SPCC1183.04C	pet127	0.4899	2.279	0.309892561	14.9	22.37	2.063	2.769
SPCC18.02	SPCC18.02	0.08789	2.283	1.056060536	16.2	24.66	1.196	0.8777
SPCC965.09	SPCC965.09	0.3406	2.285	0.467755356	17.58	27.1	0.6545	2.012
SPBC23G7.15C	rpp202	0.611	2.29	0.21395879	17.93	27.71	4.251	3.497
SPAC18B11.07C	rhp6	0.6383	2.291	0.194975156	16.94	25.96	2.199	4.285
SPBC21C3.07C	SPBC21C3.07c	0.6444	2.292	0.190844468	16.8	25.72	1.331	4.445
SPBC3E7.02C	hsp16	0.6531	2.293	0.185020316	18.86	29.37	2.573	4.461
SPCC622.11	SPCC622.11	0.2951	2.293	0.530030791	17.41	26.78	2.06	1.604
SPBC3H7.13	SPBC3H7.13	0.1357	2.295	0.867420152	19.11	29.81	1.698	0.9252
SPBC14C8.09C	SPBC14C8.09c	0.6885	2.297	0.162096055	21.03	33.2	3.104	4.998
SPBP23A10.10	ppk32	0.4687	2.303	0.329105046	15.3	23.03	2.732	2.501
SPAC1F8.04C	SPAC1F8.04c	0.5192	2.304	0.284665316	15.49	23.37	2.53	2.961
SPAC5H10.05C	SPAC5H10.05c	0.5784	2.305	0.237771716	17.28	26.54	0.4858	3.704
SPBC215.11C	SPBC215.11c	0.4809	2.322	0.317945223	20.17	31.63	1.809	2.794
SPAC5H10.02C	SPAC5H10.02c	0.5891	2.323	0.229810977	18.19	28.12	1.952	3.758
SPBC13G1.10C	mug81	0.5184	2.325	0.285335007	15.74	23.77	1.828	3.092
SPBC1271.01C	pof13	0.7261	2.329	0.139003563	19.1	29.72	4.22	5.72
SPCC965.07C	gst2	0.156	2.329	0.806875402	9.625	12.93	0.9066	1.233
SPBC14F5.03C	kap123	0.3389	2.332	0.469928431	14.94	22.35	1.351	1.998
SPBC1778.02	rap1	0.608	2.334	0.216096421	19.22	29.93	1.706	4.021
SPAC1399.05C	SPAC1399.05c	0.3143	2.351	0.502655619	16.53	25.12	1.865	1.81
SPCC757.05C	SPCC757.05c	0.2298	2.355	0.638649976	17.34	26.55	1.799	1.415
SPAC25H1.06	pcf3	0.722	2.356	0.141462802	21.18	33.36	3.106	5.857
SPBC1105.05	exg1	0.6278	2.356	0.202178689	18.71	28.98	3.966	3.961
SPBC3B8.03	SPBC3B8.03	0.6119	2.357	0.213319547	18.37	28.38	1.669	4.111
SPBC30D10.04	swi3	0.4548	2.36	0.342179544	14.49	21.49	1.093	2.724
SPAPB17E12.04C	csn2	0.2941	2.361	0.531504975	14.95	22.3	2.077	1.66
SPAC23H3.14	SPAC23H3.14	0.3766	2.361	0.424119684	17.7	27.19	0.7441	2.266
SPAC7D4.13C	SPAC7D4.13c	0.5105	2.362	0.292004254	16.67	25.35	4.817	1.968
SPAC57A10.08C	SPAC57A10.08c	0.4959	2.366	0.304605892	18.79	29.11	1.911	2.954
SPCC4B3.07	nro1	0.3653	2.368	0.437350328	16.88	25.71	1.343	2.17
SPBC839.13C	rpl1601	0.3166	2.37	0.499489089	16.53	25.1	2.936	1.391
SPCC777.08C	bit61	0.04877	2.375	1.311847244	18.72	28.97	1.323	0.5883
SPAC24B11.13	hem3	0.5684	2.379	0.245345931	19.37	30.11	1.042	3.699
SPAC140.04	SPAC140.04	0.3511	2.381	0.454569171	15.93	24.02	1.906	2.026
SPCC1919.07	SPCC1919.07	0.3645	2.384	0.438302467	16.44	24.9	2.676	1.892
SPBC1773.01	SPBC1773.01	0.4017	2.39	0.396098168	18.42	28.41	2.585	2.176
SPAC3H1.08C	SPAC3H1.08c	0.4767	2.392	0.321754848	18.03	27.71	1.987	2.83
SPBC32H8.03	bem46	0.4505	2.397	0.346305205	17.13	26.11	3.78	2.067
SPAC22G7.03	SPAC22G7.03	0.5826	2.399	0.23462952	20.14	31.45	2.691	3.717
SPBC23E6.05	arx1	0.4179	2.401	0.378927629	16.79	25.5	1.58	2.487
SPAC6G9.08	ubp6	0.6352	2.404	0.197089511	19.38	30.08	2.11	4.474
SPCC1739.08C	SPCC1739.08c	0.4779	2.405	0.320662969	18.27	28.11	3.509	2.473
SPAC1687.13C	csn5	0.2155	2.412	0.666552726	19.75	30.72	2.325	1.14
SPBC29A10.11C	vps902	0.384	2.415	0.415668776	16.1	24.25	1.752	2.274
SPBC428.08C	clr4	0.5264	2.415	0.278684119	17.12	26.05	2.062	3.262
SPAC23G3.02C	sib1	0.4534	2.417	0.343518484	16.13	24.3	3.193	2.397
SPAC20H4.08	SPAC20H4.08	0.5399	2.423	0.267686673	20.88	32.7	2.596	3.321
SPBC18H10.10C	saf4	0.6791	2.425	0.16806627	18.63	28.72	3.231	5.102
SPAC29A4.16	hal4	0.3686	2.426	0.433444669	15.53	23.22	2.241	2.101
SPBC18A7.02C	SPBC18A7.02c	0.4146	2.43	0.382370702	13.84	20.22	2.33	2.376
SPBC4.06	SPBC4.06	0.7044	2.434	0.152180653	19.8	30.77	3.458	5.609
SPAC1250.04C	atl1	0.4682	2.436	0.329568591	14.3	21.03	1.018	2.914
SPAC13G6.03	gpi7	0.7169	2.442	0.14454142	21.87	34.42	3.87	5.857
SPAC4F10.16C	SPAC4F10.16c	0.4158	2.443	0.381115515	17.29	26.31	1.064	2.564
SPBC409.11	meu18	0.4708	2.446	0.327163546	14.97	22.19	2.885	2.679
SPCC4G3.11	mug154	0.1509	2.448	0.82131076	15.03	22.29	1.53	1.186
SPBC18H10.13	rps1402	0.5656	2.452	0.247490599	17.97	27.51	1.187	3.776
SPBC30D10.18C	rpl102	0.2387	2.457	0.622147581	19.57	30.33	2.606	1.149
SPBP8B7.22	erd2	0.4631	2.461	0.334325219	18.49	28.41	0.4645	2.928
SPAC1006.04C	mcp3	0.1879	2.463	0.72607322	17.44	26.54	1.372	1.398
SPCC1183.02	SPCC1183.02	0.2807	2.465	0.551757587	18.41	28.25	2.494	1.529
SPAC6F12.04	SPAC6F12.04	0.1073	2.466	0.969400278	16.73	25.27	0.7895	1.101
SPBC409.07C	wis1	0.4359	2.473	0.360613131	16.53	24.91	3.678	2.114
SPAC23C4.17	SPAC23C4.17	0.6295	2.474	0.201004266	20.27	31.54	3.121	4.398
SPAC6B12.12	tom70	0.4574	2.475	0.33970384	12.77	18.24	2.714	2.654
SPAC13G6.04	tim8	0.684	2.478	0.164943898	19.57	30.29	3.632	5.259

SPBC577.04	SPBC577.04	0.6528	2.482	0.185219854	18.78	28.89	4.888	4.403
SPCC584.13	SPCC584.13	0.227	2.486	0.643974143	16.68	25.15	2.018	1.44
SPAC3G9.05	SPAC3G9.05	0.2636	2.489	0.579054594	18.1	27.66	2.865	1.175
SPAPB8E5.08	SPAPB8E5.08	0.3247	2.49	0.488517711	19.3	29.79	1.625	2.035
SPBC405.06	SPBC405.06	0.4073	2.491	0.39008559	14.05	20.48	3.763	1.811
SPBC4B4.04	SPBC4B4.04	0.426	2.492	0.370590401	17.15	25.98	2.243	2.546
SPAC11H11.01	sst6	0.09807	2.492	1.008463825	18.49	28.35	1.592	0.9025
SPCC13B11.02C	SPCC13B11.02c	0.04655	2.499	1.332080315	17.79	27.1	1.418	0.5394
SPBC1709.12	rid1	0.7235	2.501	0.140561465	19.94	30.9	4.207	6.13
SPAC3A12.03C	meu34	0.4943	2.508	0.30600939	16.28	24.4	2.136	3.103
SPAC11H11.04	mam2	0.185	2.508	0.732828272	15.16	22.42	1.66	1.357
SPAC3G6.05	SPAC3G6.05	0.3412	2.512	0.466990978	16.12	24.12	3.443	1.429
SPBC1773.12	SPBC1773.12	0.1177	2.512	0.929223537	9.218	11.88	0.7798	1.169
SPCC132.03	SPCC132.03	0.4098	2.514	0.387428046	19	29.22	1.937	2.513
SPAC9E9.14	vps24	0.07406	2.514	1.130416292	15.49	23	1.615	0.6974
SPAC139.01C	SPAC139.01c	0.3483	2.514	0.458046526	13.6	19.64	1.005	2.242
SPAC589.05C	SPAC589.05c	0.5965	2.526	0.224389552	19.18	29.52	1.698	4.216
SPBC25H2.05	egd2	0.004534	2.528	2.343518484	16.89	25.46	0.5722	0.4419
SPAPB17E12.14C	SPAPB17E12.14c	0.04788	2.531	1.319845858	19.21	29.55	1.238	0.7435
SPBC651.04	SPBC651.04	0.6633	2.534	0.178290003	20.31	31.49	3.962	4.935
SPAC22H10.13	zym1	0.3177	2.534	0.497982785	16.83	25.33	2.967	1.614
SPAC3F10.12C	SPAC3F10.12c	0.647	2.537	0.189095719	18.25	27.84	2.666	4.857
SPCC1322.12C	bub1	0.4615	2.541	0.335828295	17.37	26.29	3.291	2.615
SPAC1805.04	nup132	0.3108	2.543	0.50751899	18.49	28.26	2.509	1.796
SPBC12C2.02C	ste20	0.2446	2.544	0.611543547	9.072	11.57	2.028	1.737
SPBC1711.02	matmc_1	0.4403	2.548	0.356251315	19.35	29.77	1.791	2.78
SPBP23A10.16	sdh4	0.007997	2.549	2.097072904	23.97	37.96	0.7948	0.4746
SPACUNK4.15	SPACUNK4.15	0.7271	2.557	0.138405855	22.23	34.87	4.148	6.389
SPBC3D6.13C	pdi2	0.5094	2.557	0.292941059	19.19	29.48	1.717	3.349
SPCC1235.04C	SPCC1235.04c	0.0005111	2.559	3.291494119	16.34	24.42	0.5049	0.2201
SPAC2G11.04	SPAC2G11.04	0.4773	2.565	0.321208566	17.28	26.08	2.361	3.004
SPBP8B7.08C	SPBP8B7.08c	0.4717	2.571	0.326334124	17.57	26.58	1.731	3.051
SPAC26A3.06	SPAC26A3.06	0.616	2.581	0.210419288	19.62	30.19	2.265	4.52
SPCC63.14	SPCC63.14	0.5821	2.587	0.235002401	18.67	28.5	2.076	4.109
SPBC28F2.11	SPBC28F2.11	0.6653	2.59	0.176982477	18.72	28.59	3.026	5.241
SPAC2C4.17C	SPAC2C4.17c	0.2333	2.591	0.632085261	17.53	26.47	1.823	1.617
SPBC4C3.06	syp1	0.5815	2.592	0.235450281	19.86	30.6	2.765	4.025
SPAC26A3.17C	SPAC26A3.17c	0.5687	2.611	0.245116772	20.09	30.97	1.988	4
SPAC11G7.03	idh1	0.1368	2.612	0.863913903	18.81	28.71	1.63	1.193
SPAC23C11.14	zhf1	0.03667	2.615	1.43568909	11.81	16.3	1.385	0.5134
SPAC2C4.15C	ubx2	0.6116	2.628	0.213532523	14.71	21.41	7.402	2.46
SPAC589.10C	SPAC589.10c	0.6492	2.633	0.187621489	14.9	21.74	6.766	3.962
SPBC800.07C	tsf1	0.4129	2.644	0.384155117	7.635	8.845	2.46	2.59
SPCC645.12C	SPCC645.12c	0.4528	2.652	0.344093582	18.48	28.05	1.812	2.994
SPAC23H4.16C	SPAC23H4.16c	0.4887	2.655	0.310957661	17.46	26.24	1.588	3.311
SPAC13F5.05	SPAC13F5.05	0.7299	2.658	0.136736636	20.54	31.7	4.297	6.722
SPAC2E1P3.01	SPAC2E1P3.01	0.417	2.661	0.379863945	15.52	22.78	2.06	2.709
SPBC1861.07	SPBC1861.07	0.4856	2.663	0.313721322	19.3	29.48	1.401	3.309
SPBC2F12.15C	pfa3	0.1764	2.665	0.753501419	13.43	19.07	1.67	1.421
SPAC22H10.09	SPAC22H10.09	0.2145	2.672	0.668572703	11.08	14.91	1.128	1.683
SPCC31H12.06	mug111	0.3589	2.672	0.445026542	16.97	25.33	0.9366	2.452
SPAC57A10.14	sgf11	0.4806	2.674	0.318216234	13.71	19.55	4.004	2.737
SPCC548.06C	ght8	0.1974	2.679	0.704652852	7.989	9.41	1.966	1.458
SPAC18G6.12C	SPAC18G6.12c	0.2776	2.68	0.556580538	16.3	24.13	2.353	1.788
SPBC26H8.13C	SPBC26H8.13c	0.2308	2.686	0.636764196	15.27	22.3	0.9816	1.779
SPAC1B3.15C	SPAC1B3.15c	0.4395	2.687	0.357041121	19.36	29.54	1.797	2.936
SPCPJ732.02C	SPCPJ732.02c	0.3712	2.693	0.430392032	17.64	26.49	2.039	2.44
SPAPB1A10.10C	ypt71	0.6512	2.694	0.186285608	20.25	31.11	3.148	5.19
SPAC1F7.11C	SPAC1F7.11c	0.6117	2.694	0.21346152	18.14	27.37	2.64	4.623
SPCC1223.15C	spc19	0.3641	2.695	0.438779321	14.73	21.33	1.291	2.483
SPAC3A11.03	SPAC3A11.03	0.3538	2.701	0.451242171	17.87	26.89	1.391	2.419
SPCC24B10.13	skb5	0.3593	2.708	0.444542783	16.98	25.3	3.11	2.082
SPBC17G9.10	rpl1102	0.3863	2.712	0.413075292	18.87	28.63	1.58	2.615
SPAC3H1.11	hsr1	0.042	2.714	1.37675071	18.24	27.52	0.8806	0.8535
SPAC1751.04	SPAC1751.04	0.4355	2.715	0.361011841	19.89	30.44	2.14	2.896
SPCC23B6.05C	ssb3	0.5406	2.717	0.267123959	17.06	25.42	1.675	3.883
SPBC577.13	syj2	0.2638	2.723	0.578725209	15.49	22.62	1.991	1.847
SPCP31B10.04	SPCP31B10.04	0.4324	2.731	0.364114315	20.59	31.65	2.888	2.746
SPCC550.08	SPCC550.08	0.335	2.732	0.474955193	16.7	24.75	2.302	2.199

SPBC12C2.03C	SPBC12C2.03c	0.0656	2.732	1.183096161	16.54	24.47	1.154	0.9792
SPAC23A1.11	rpl1602	0.02201	2.733	1.657379957	18.39	27.74	1.153	0.6072
SPAC23H3.12C	SPAC23H3.12c	0.6079	2.735	0.216167857	19.59	29.86	2.883	4.612
SPAC20H4.11C	rho5	0.7373	2.736	0.132355766	21.51	33.27	3.866	7.218
SPBC354.03	swd3	0.246	2.748	0.609064893	21.72	33.62	0.7137	1.906
SPAC17D4.04	SPAC17D4.04	0.3331	2.753	0.477425367	16.32	24.04	1.997	2.269
SPBPJ4664.01	dps1	0.53	2.754	0.27572413	9.385	11.75	5.551	2.687
SPAC1002.12C	SPAC1002.12c	0.04779	2.757	1.320662969	18.53	27.95	0.7006	0.9049
SPBC29A10.02	spo5	0.03491	2.766	1.457050151	18.39	27.68	1.325	0.6899
SPCC622.14	SPCC622.14	0.5124	2.766	0.290390879	20.76	31.89	2.827	3.524
SPCC4B3.02C	SPCC4B3.02c	0.4381	2.779	0.358426747	20.61	31.6	1.654	3.047
SPAC9E9.11	plr1	0.5163	2.782	0.287097875	20.29	31.03	1.047	3.768
SPBC6B1.06C	ubp14	0.2594	2.783	0.586030028	10.99	14.54	0.9356	1.993
SPCPB16A4.02C	SPCPB16A4.02c	0.2996	2.787	0.523458191	16.05	23.51	3.182	1.673
SPBC16C6.09	ogm4	0.2041	2.788	0.690156995	18.94	28.62	2.798	1.11
SPAC4A8.04	isp6	0.04903	2.799	1.309538107	15.94	23.28	1.54	0.7181
SPAPB1A11.03	SPAPB1A11.03	0.4853	2.801	0.313989709	19.73	30	2.115	3.418
SPAC15A10.09C	SPAC15A10.09c	0.45	2.804	0.346787486	16.84	24.87	4.437	2.402
SPAC30C2.08	SPAC30C2.08	0.4804	2.805	0.318397001	18.56	27.92	1.695	3.423
SPCC4B3.13	SPCC4B3.13	0.09652	2.813	1.015382687	19.15	28.95	2.05	0.7722
SPBC15D4.05	SPBC15D4.05	0.5835	2.816	0.23395914	19.26	29.14	2.543	4.461
SPAC23D3.03C	SPAC23D3.03c	0.631	2.819	0.199970641	19.11	28.87	3.462	5.052
SPBC19G7.18C	SPBC19G7.18c	0.2936	2.821	0.532243949	16.82	24.81	2.783	1.873
SPAC869.07C	mel1	0.326	2.824	0.4867824	18.13	27.12	1.719	2.332
SPAC323.05C	SPAC323.05c	0.1403	2.828	0.852942329	11.47	15.31	2.322	0.9989
SPAC1834.08	mak1	0.4393	2.833	0.357238797	15.22	21.94	1.769	3.108
SPBC1703.03C	SPBC1703.03c	0.3581	2.836	0.445995679	16.13	23.56	4.436	1.139
SPBC725.15	ura5	0.4684	2.837	0.329383114	19.44	29.42	2.303	3.289
SPAC1B3.04C	SPAC1B3.04c	0.2119	2.838	0.673869043	15.35	22.17	1.892	1.678
SPAC27E2.07	pvg2	0.5898	2.848	0.229295232	17.64	26.21	1.873	4.665
SPAC5D6.01	rps2202	0.4719	2.853	0.326150023	18.09	27	2.684	3.282
SPAC19G12.09	SPAC19G12.09	0.1759	2.853	0.754734161	16.08	23.43	2.159	1.399
SPBC1685.14C	SPBC1685.14c	0.2097	2.855	0.67840157	20.3	30.92	1.766	1.705
SPBC9B6.11C	SPBC9B6.11c	0.06516	2.859	1.186018924	18.79	28.23	1.857	0.4497
SPBC947.08C	hip4	0.5918	2.86	0.227825039	18.39	27.52	3.114	4.574
SPAC1F5.08C	yam8	0.1673	2.862	0.776504059	14.38	20.41	2.274	1.293
SPCC613.06	rpl902	0.6728	2.866	0.172114017	9.296	11.4	5.312	5.609
SPBC16E9.15	SPBC16E9.15	0.6623	2.868	0.178945245	20.23	30.77	1.76	5.889
SPBC14F5.09C	ade8	0.1185	2.869	0.92628165	17.71	26.3	1.804	1.194
SPAC4F8.10C	stg1	0.3866	2.873	0.41273815	15.7	22.73	4.371	1.805
SPCPB16A4.06C	SPCPB16A4.06c	0.3504	2.878	0.455435903	18.48	27.66	1.887	2.512
SPAC1782.09C	clp1	0.5941	2.878	0.226140448	20.7	31.57	3.073	4.645
SPBC3B8.07C	dsd1	0.6298	2.882	0.200797344	17.28	25.51	3.293	5.179
SPCC576.02	SPCC576.02	0.3657	2.882	0.43687504	19.16	28.85	1.912	2.613
SPBC354.12	gpd3	0.6792	2.882	0.168002323	15.1	21.64	7.482	5.102
SPAC6B12.15	cpc2	0.4121	2.89	0.384997385	9.166	11.12	4.043	2.355
SPBC17D1.06	dbp3	0.5529	2.892	0.25735341	18.12	26.99	3.102	4.118
SPBC21C3.02C	dep1	0.6078	2.897	0.216239304	18.26	27.23	3.488	4.821
SPBC15D4.10C	amo1	0.699	2.898	0.155522824	10.26	13.05	6.513	6.038
SPCC70.08C	SPCC70.08c	0.5621	2.903	0.250186415	18.14	27	2.314	4.354
SPAC1D4.09C	rtf2	0.1718	2.905	0.764976841	13.87	19.44	2.883	0.1807
SPCC11E10.03	mug1	0.2175	2.913	0.662540739	16.66	24.36	2.08	1.72
SPCC1322.06	kap113	0.1822	2.919	0.739451627	13.78	19.25	1.215	1.674
SPAC13F5.07C	SPAC13F5.07c	0.1973	2.922	0.704872915	17.3	25.48	1.556	1.719
SPAC2F3.05C	SPAC2F3.05c	0.6832	2.928	0.165452142	19.91	30.1	4.42	6.175
SPCC622.01C	SPCC622.01c	0.5632	2.931	0.249337354	19.52	29.4	2.072	4.436
SPAC23A1.17	SPAC23A1.17	0.132	2.931	0.879426069	18.55	27.67	1.285	1.418
SPCC645.08C	snd1	0.2952	2.934	0.529883647	20.96	31.94	1.797	2.235
SPBC216.06C	swi1	0.5855	2.935	0.232473101	19.42	29.21	2.606	4.682
SPAC4F8.11	SPAC4F8.11	0.4329	2.936	0.363612414	16.88	24.72	2.179	3.13
SPBC1539.08	arf6	0.3492	2.938	0.456925765	6.121	5.64	3.318	2.199
SPBC2D10.06	rep1	0.3384	2.94	0.470569646	17.55	25.89	2.002	2.478
SPBC28F2.03	ppi1	0.5746	2.956	0.240634378	18.58	27.68	2.343	4.598
SPAP11E10.02C	mam3	0.4368	2.959	0.35971737	17.12	25.1	3.268	2.979
SPAC20H4.06C	SPAC20H4.06c	0.2829	2.964	0.548367053	14.69	20.78	2.149	2.129
SPAC664.15	caf4	0.1043	2.966	0.981715692	18.12	26.85	1.411	1.27
SPAC1F8.03C	str3	0.4232	2.969	0.373454341	16.97	24.81	3.329	2.854
SPAC19G12.10C	cpy1	0.6057	2.972	0.217742426	20.42	30.92	2.447	5.059
SPAPB1E7.06C	eme1	0.4601	2.972	0.337147767	7.547	8.107	4.916	2.546

SPAC15E1.10	SPAC15E1.10	0.5457	2.982	0.263046046	15.37	21.96	7.39	1.759
SPAC8E11.01C	SPAC8E11.01c	0.5062	2.986	0.295677859	20.31	30.7	1.566	3.914
SPAC6B12.07C	SPAC6B12.07c	0.2671	2.989	0.573326112	15.97	23.01	1.857	2.107
SPBC16C6.08C	qcr6	0.4372	3.002	0.359319847	18.7	27.82	2.689	3.163
SPAC1039.04	SPAC1039.04	0.6213	3.004	0.206698646	20.31	30.67	4.347	5.093
SPAC15E1.09	grx2	0.26	3.005	0.585026652	16.74	24.33	1.785	2.089
SPBC17G9.08C	cnt5	0.003405	3.006	2.467882884	9.576	11.64	0.5651	0.4922
SPBC16D10.02	trm11	0.1581	3.012	0.80106813	18.46	27.38	0.09821	1.61
SPBC1198.08	SPBC1198.08	0.2344	3.013	0.630042393	19.53	29.27	3.063	1.476
SPBP35G2.06C	nup131	0.06667	3.016	1.176069545	17.5	25.67	1.086	1.109
SPBPB2B2.06C	SPBPB2B2.06c	0.3618	3.017	0.441531437	18.05	26.65	1.608	2.751
SPCC1223.02	nmt1	0.352	3.017	0.453457337	14.6	20.52	1.573	2.688
SPBC1289.10C	adn2	0.3839	3.038	0.415781888	16.35	23.59	4.746	1.739
SPAC16E8.13	SPAC16E8.13	0.3228	3.052	0.491066474	18.6	27.55	0.837	2.573
SPAC17G8.07	yaf9	0.1586	3.058	0.799696817	17	24.71	1.915	1.526
SPAC16E8.12C	SPAC16E8.12c	0.5832	3.061	0.234182485	20.72	31.3	2.946	4.822
SPAC6G9.12	cfr1	0.5792	3.062	0.237171447	20.09	30.17	3.853	4.621
SPBC32H8.01C	SPBC32H8.01c	0.6292	3.063	0.201211286	20.96	31.72	3.457	5.499
SPAC694.03	SPAC694.03	0.3302	3.075	0.481222931	20.18	30.32	0.8592	2.64
SPAC688.04C	gst3	0.5527	3.077	0.257510535	19.02	28.26	3.016	4.423
SPCC1442.17C	ist1	0.5958	3.09	0.224899501	20.27	30.45	2.595	5.1
SPAC26F1.09	gyp51	0.1433	3.093	0.84375381	16.87	24.41	0.5486	1.577
SPAC23H3.05C	swd1	0.3502	3.099	0.455683858	21.5	32.61	2.008	2.707
SPCC126.03	pus1	0.376	3.103	0.424812155	13.45	18.33	1.637	2.933
SPAC222.05C	mss1	0.4526	3.107	0.34428545	18.73	27.69	1.339	3.572
SPCP1E11.10	SPCP1E11.10	0.6519	3.112	0.185819019	9.038	10.5	6.198	5.478
SPBP8B7.30C	thi5	0.134	3.113	0.872895202	14.53	20.23	2.31	1.236
SPBC16H5.06	rip1	0.18	3.115	0.744727495	10.95	13.89	1.34	1.771
SPAC2C4.10C	SPAC2C4.10c	0.4035	3.119	0.394156461	17.15	24.86	3.776	2.721
SPAC1071.05	SPAC1071.05	0.3372	3.12	0.472112434	15.82	22.51	1.108	2.715
SPBC543.05C	SPBC543.05c	0.4249	3.129	0.371713269	18.93	27.99	0.9344	3.379
SPAC1F8.02C	SPAC1F8.02c	0.251	3.141	0.600326279	11.25	14.37	2.379	2.028
SPBC26H8.11C	SPBC26H8.11c	0.3693	3.145	0.432620692	17.87	26.09	1.108	2.961
SPCC191.11	inv1	0.1958	3.148	0.708187313	16.8	24.19	2.994	1.326
SPAC212.03	SPAC212.03	0.4778	3.152	0.320753855	16.51	23.67	3.76	3.515
SPAC1F3.10C	1-Oct	0.2306	3.155	0.637140697	16.87	24.3	1.407	2.073
SPBC2A9.11C	SPBC2A9.11c	0.1755	3.161	0.755722879	19.98	29.81	2.321	1.576
SPCC1840.12	SPCC1840.12	0.3401	3.161	0.468393368	17.51	25.42	1.463	2.75
SPAC4D7.01C	sec71	0.0108	3.162	1.966576245	21.3	32.14	0.2796	0.6033
SPAC1B3.05	not3	0.314	3.168	0.503070352	8.793	9.968	3.684	1.997
SPAC13G6.02C	rps101	0.3434	3.173	0.464199709	18.54	27.23	1.98	2.733
SPBC1539.03C	SPBC1539.03c	0.05335	3.193	1.272865576	10.32	12.63	0.7749	1.086
SPBC19C7.10	bqt4	0.3431	3.199	0.464579282	19.4	28.7	1.4	2.809
SPBC409.18	SPBC409.18	0.1867	3.212	0.728855682	18.92	27.84	1.318	1.868
SPAC10F6.14C	SPAC10F6.14c	0.1594	3.217	0.797511683	18.69	27.42	1.45	1.707
SPAPB1A10.08	SPAPB1A10.08	0.2627	3.247	0.580539927	19.29	28.44	2.527	2.157
SPAPB17E12.02	yip12	0.2649	3.261	0.576918042	19.77	29.26	0.8754	2.376
SPAC15A10.08	ain1	0.5776	3.27	0.238372815	20.18	29.96	0.9629	5.235
SPBC25B2.04C	mtg1	0.02722	3.27	1.565111879	11.34	14.31	0.6342	0.8861
SPCPB1C11.01	amt1	0.07285	3.274	1.137570444	17.54	25.27	1.215	1.243
SPCC338.07C	naa15	0.4833	3.28	0.315783205	17.92	25.95	2.539	3.974
SPAC630.15	mug177	0.02566	3.287	1.590743348	17.67	25.49	1.15	0.8716
SPBC8D2.19	mde3	0.2611	3.288	0.583193128	14.81	20.42	3.602	1.686
SPBC56F2.02	rpl1901	0.4113	3.298	0.38584129	6.504	5.68	2.985	3.233
SPAC1805.03C	trm13	0.4939	3.301	0.306360974	21.56	32.36	1.452	4.209
SPBC1271.05C	SPBC1271.05c	0.2227	3.316	0.652279783	15.82	22.15	3.706	1.046
SPBC359.03C	aat1	0.06726	3.317	1.172243137	19.1	27.97	2.167	0.6796
SPBC106.01	mph1	0.2906	3.324	0.53670439	13.53	18.08	4.145	1.597
SPCC364.01	cif1	0.1163	3.324	0.934420285	17.77	25.59	2.055	1.381
SPAC8C9.05	SPAC8C9.05	0.4694	3.329	0.328456915	22.12	33.3	2.24	3.927
SPCC645.11C	mug117	0.2912	3.342	0.535808629	18.21	26.34	0.783	2.608
SPAC8F11.02C	dph3	0.2384	3.353	0.622693749	15.99	22.4	2.638	2.051
SPBC36B7.05C	SPBC36B7.05c	0.3258	3.357	0.48704892	17.49	25.04	1.806	2.797
SPBC30D10.03C	SPBC30D10.03c	0.1609	3.38	0.793443956	17.28	24.62	1.37	1.815
SPCC550.14	vgl1	0.1645	3.38	0.783834098	13.86	18.57	2.017	1.75
SPCC4G3.09C	gyp3	0.1253	3.39	0.902048929	15.99	22.33	2.318	1.382
SPCC962.04	rps1201	0.5147	3.394	0.288445832	13.14	17.27	6.008	3.541
SPBC2F12.05C	SPBC2F12.05c	0.1914	3.396	0.718058067	16.89	23.92	1.87	1.953
SPCC16A11.16C	rpn1302	0.3294	3.401	0.482276405	19.16	27.93	3.477	2.513

SPBC31F10.05	mug37	0.1834	3.407	0.736600669	20.69	30.63	0.9999	1.978
SPCC74.03C	ssp2	0.4735	3.412	0.324680017	20.98	31.14	1.77	4.115
SPAC27F1.08	pdt1	0.704	3.413	0.152427341	19.71	28.88	6.293	7.606
SPAC22F3.13	tsc1	0.1753	3.43	0.756218084	14.67	19.91	1.753	1.893
SPAC3A12.13C	SPAC3A12.13c	0.2444	3.434	0.611898798	16.37	22.93	1.272	2.358
SPBC725.03	SPBC725.03	0.6723	3.454	0.172436889	17.31	24.56	8.522	6.08
SPCC63.13	SPCC63.13	0.497	3.466	0.303643611	20.1	29.48	2.844	4.334
SPBC16H5.08C	SPBC16H5.08c	0.2477	3.468	0.606073993	14.19	19	1.972	2.343
SPAP8A3.04C	hsp9	0.2445	3.471	0.611721137	13.85	18.4	1.716	2.354
SPCC330.03C	SPCC330.03c	0.34	3.473	0.468521083	19.06	27.62	2.566	2.908
SPBC83.04	apc15	0.5934	3.478	0.226652458	20.64	30.4	2.482	5.741
SPBC8D2.01	gsk31	0.437	3.482	0.359518563	18.55	26.7	1.754	3.835
SPBC3F6.05	rga1	0.5749	3.486	0.240407691	22.56	33.81	2.638	5.438
SPCC1281.08	wtf11	0.2801	3.488	0.552686891	18.69	26.94	4.342	1.515
SPCC74.05	rpl2702	0.5907	3.49	0.228633029	14.61	19.7	7.29	4.561
SPCC1494.05C	ubp12	0.1203	3.491	0.919734373	11.62	14.41	0.4926	1.63
SPAC2G11.12	rqh1	0.2989	3.507	0.524474085	14.12	18.81	2.499	2.642
SPBC887.04C	lub1	0.5754	3.51	0.240030142	22.42	33.52	3.468	5.391
SPCC16A11.01	SPCC16A11.01	0.1269	3.511	0.896538378	18.79	27.08	2.917	0.9622
SPAC6B12.09	trm10	0.6822	3.562	0.166088285	21.92	32.53	4.19	7.654
SPCC594.06C	SPCC594.06c	0.05489	3.565	1.260506769	18.06	25.68	1.887	1.055
SPAC1F8.08	SPAC1F8.08	0.4112	3.566	0.385946894	21.15	31.16	2.137	3.654
SPCC663.09C	SPCC663.09c	0.004192	3.567	2.377578726	18.78	26.97	1.087	0.4324
SPCC63.08C	atg1	0.3034	3.572	0.517984424	16.25	22.47	2.815	2.67
SPAC9G1.12	cpd1	0.1294	3.574	0.888065724	10.95	13.07	1.096	1.737
SPAC8C9.07	SPAC8C9.07	0.08695	3.583	1.060730414	12.35	15.54	1.535	1.439
SPAC1B1.04C	SPAC1B1.04c	0.0972	3.585	1.012333735	16.52	22.93	2.196	1.338
SPCC1682.14	rpl1902	0.2372	3.594	0.624885315	16.93	23.63	3.899	1.576
SPAC1486.08	SPAC1486.08	0.5405	3.595	0.267204302	19.26	27.77	2.108	5.145
SPAC1556.02C	sdh1	0.2755	3.606	0.559878397	19.46	28.09	2.016	2.632
SPAC25B8.09	SPAC25B8.09	0.3727	3.618	0.428640607	19.63	28.37	2.049	3.379
SPBC1652.01	SPBC1652.01	0.4849	3.619	0.314347816	18.88	27.04	1.841	4.494
SPAC29E6.10C	SPAC29E6.10c	0.4131	3.623	0.383944805	13.06	16.72	3.015	3.618
SPCC777.13	vps35	0.3429	3.636	0.464832515	8.843	9.227	3.091	2.988
SPAC227.07C	pab1	0.07766	3.645	1.109802614	9.324	10.06	1.878	1.569
SPAC513.06C	SPAC513.06c	0.4818	3.651	0.317133204	20.44	29.76	1.916	4.495
SPAC2F7.03C	pom1	0.2568	3.652	0.590404981	16.79	23.29	1.696	2.569
SPAC2E1P5.03	SPAC2E1P5.03	0.001883	3.653	2.72514968	11.63	14.13	0.9327	0.3529
SPBC839.14C	SPBC839.14c	0.06779	3.656	1.168834366	16.48	22.73	1.335	1.352
SPBC19F8.06C	meu22	0.5061	3.661	0.295763663	21.05	30.82	3.052	4.686
SPAC11E3.06	map1	0.5694	3.668	0.244582537	22.09	32.65	3.489	5.551
SPCC1183.11	SPCC1183.11	0.3448	3.675	0.462432743	16.42	22.59	3.89	2.811
SPCP1E11.11	puf6	0.1392	3.684	0.856360765	18.16	25.65	1.439	1.843
SPAC25G10.09C	pan1	0.127	3.707	0.896196279	17.75	24.88	0.7383	1.783
SPBC16C6.03C	SPBC16C6.03c	0.203	3.71	0.692503962	18.62	26.42	3.821	1.33
SPBC365.16	SPBC365.16	0.06445	3.719	1.190777078	11.06	13	2.201	1.066
SPAC1B1.02C	SPAC1B1.02c	0.2917	3.724	0.535063571	20.35	29.46	2.243	2.816
SPBC119.05C	SPBC119.05c	0.0132	3.725	1.879426069	18.86	26.82	0.8033	0.8381
SPCPB16A4.03C	ade10	0.03163	3.729	1.499900808	10.75	12.44	1.394	1.045
SPAC3F10.13	ucp6	0.3621	3.729	0.441171475	20.43	29.6	2.075	3.396
SPBC27.06C	mgr2	0.06666	3.734	1.176134691	7.835	7.268	1.339	1.373
SPAC14C4.07	SPAC14C4.07	0.04718	3.737	1.326242063	17.37	24.17	1.515	1.188
SPBC3B9.13C	rpp102	0.5667	3.741	0.246646787	14.32	18.75	6.673	4.863
SPAPB1A11.02	SPAPB1A11.02	0.11	3.745	0.958607315	18.01	25.28	2.874	1.077
SPAC1783.02C	vps66	0.009409	3.75	2.026456531	16.55	22.68	0.8414	0.7787
SPBC405.07	rpl3602	0.5765	3.75	0.239200688	14.31	18.71	6.921	4.984
SPCC13B11.03C	SPCC13B11.03c	0.08816	3.754	1.054728418	19.6	28.08	2.206	1.359
SPAC4G9.20C	SPAC4G9.20c	0.08542	3.761	1.068440433	18.05	25.32	2.119	1.374
SPBC9B6.07	nop52	0.5456	3.766	0.263125638	8.434	8.272	5.172	5.01
SPAC26H5.09C	SPAC26H5.09c	0.2068	3.771	0.684449466	19.1	27.16	2.071	2.274
SPAC959.05C	SPAC959.05c	0.2681	3.779	0.571703186	19.97	28.7	3.793	2.237
SPAC4D7.11	dsc4	0.3136	3.784	0.503623946	9.132	9.479	3.569	2.754
SPBC16G5.17	SPBC16G5.17	0.124	3.802	0.906578315	17.48	24.25	2.239	1.678
SPBC11B10.08	SPBC11B10.08	0.548	3.803	0.261219442	22.86	33.77	3.339	5.447
SPBC21B10.12	rec6	0.1411	3.819	0.850472986	23.11	34.19	2.452	1.756
SPAC30C2.05	erv14	0.2991	3.831	0.524183587	21.06	30.53	2.478	2.93
SPAC6C3.06C	SPAC6C3.06c	0.02862	3.843	1.543330371	19.08	27	1.128	1.076
SPAC869.08	pcm2	0.1618	3.845	0.791021483	21.4	31.11	1.802	2.05
SPCC553.08C	SPCC553.08c	0.4168	3.853	0.38007229	15.07	19.87	6.858	1.859

SPBPB2B2.10C	gal7	0.135	3.877	0.869666232	18.04	25.11	2.495	1.731
SPBC16E9.13	ksp1	0.03754	3.901	1.425505732	20.13	28.76	1.643	1.119
SPAC23G3.05C	SPAC23G3.05c	0.06432	3.904	1.191653964	18.38	25.66	2.014	1.29
SPAC14C4.15C	SPAC14C4.15c	0.0364	3.916	1.438898616	18	24.96	1.122	1.18
SPBC28E12.04	SPBC28E12.04	0.3251	3.919	0.487983031	16.55	22.39	2.148	3.255
SPCC1442.05C	SPCC1442.05c	0.1435	3.923	0.843148099	17.04	23.24	3.174	1.473
SPAC2F7.08C	snf5	0.1386	3.94	0.85823677	22.45	32.81	3.079	1.507
SPAC4A8.09C	cwf21	0.356	3.94	0.448550002	20.57	29.46	3.215	3.385
SPAPB1A10.07C	SPAPB1A10.07c	0.2474	3.94	0.606600305	18.83	26.38	2.228	2.661
SPAC637.13C	SPAC637.13c	0.4316	3.946	0.364918564	23.45	34.56	4.713	3.804
SPBC1709.05	sks2	0.04771	3.948	1.321390583	18.78	26.29	1.989	1.128
SPBP22H7.05C	abo2	0.006179	3.959	2.209081805	19.68	27.86	1.284	0.3737
SPBC8D2.02C	SPBC8D2.02c	0.3959	3.966	0.402414498	18.22	25.25	2.386	3.909
SPCC1020.11C	SPCC1020.11c	0.5458	3.968	0.262966469	15.62	20.65	4.686	5.453
SPBCPT2R1.03	SPBCPT2R1.03	0.109	3.972	0.962573502	18.56	25.86	2.395	1.608
SPBC12C2.09C	SPBC12C2.09c	0.3946	3.972	0.403842919	21.07	30.3	0.9891	3.992
SPAC3H5.07	rpl702	0.08898	3.975	1.050707599	16.07	21.44	2.127	1.524
SPAC24B11.05	SPAC24B11.05	0.144	3.986	0.841637508	17.45	23.87	2.755	1.784
SPAC17C9.11C	SPAC17C9.11c	0.4262	3.986	0.370386555	22.91	33.53	1.567	4.302
SPBC1718.07C	zfs1	0.6768	3.996	0.16953965	14.18	18.05	7.379	7.955
SPBC30D10.16	pha2	0.001396	3.997	2.855114582	6.604	4.621	0.9123	0.2798
SPAC521.05	rps802	0.5654	3.999	0.247644196	14.37	18.38	6.452	5.412
SPAC25B8.19C	SPAC25B8.19c	0.3685	4.009	0.433562508	6.067	3.647	3.292	3.56
SPAC869.04	SPAC869.04	0.599	4.021	0.222573178	20.81	29.75	4.74	6.521
SPBP18G5.03	toc1	0.02134	4.022	1.670805585	20.76	29.66	1.834	0.5752
SPBC1711.05	SPBC1711.05	0.2807	4.028	0.551757587	18.54	25.72	3.015	2.855
SPAC2F7.10	akr1	0.1655	4.051	0.781202002	18.14	24.96	1.619	2.209
SPBPB10D8.06C	SPBPB10D8.06c	0.5465	4.052	0.262409834	20.89	29.84	4.16	5.693
SPCPB16A4.05C	SPCPB16A4.05c	0.07493	4.071	1.125344267	17.29	23.43	2.116	1.45
SPBC646.02	cwf11	0.4385	4.111	0.358030402	18.8	26.03	2.473	4.51
SPAC23G3.10C	ssr3	0.4151	4.111	0.381847267	19.54	27.34	3.552	4.105
SPBC646.13	sds23	0.2293	4.132	0.639595945	15.02	19.3	2.327	2.653
SPBC16D10.08C	SPBC16D10.08c	0.2873	4.132	0.541664374	19.58	27.37	3.101	2.983
SPCC24B10.09	rps1702	0.1256	4.134	0.901010361	14.42	18.22	2.808	1.697
SPBC4B4.11	SPBC4B4.11	0.2418	4.15	0.616543703	19.9	27.91	1.81	2.813
SPCC794.11C	SPCC794.11c	0.1345	4.174	0.871277716	22.4	32.29	3.216	1.581
SPBC3D6.09	dpb4	0.576	4.174	0.239577517	21.06	29.92	1.742	6.633
SPBC56F2.14	mrpl44	0.0617	4.175	1.209714836	18.22	24.88	2.05	1.389
SPCC338.10C	cox5	0.2873	4.177	0.541664374	10.24	10.74	3.602	2.896
SPAC56E4.07	SPAC56E4.07	0.04646	4.184	1.332920795	18.89	26.06	2.245	1.076
SPAC3H8.09C	nab3	0.0188	4.186	1.725842151	12.72	15.12	1.583	0.9578
SPAC2F3.18C	SPAC2F3.18c	0.4054	4.195	0.392116256	21.3	30.32	1.559	4.307
SPCC622.19	jmj4	0.1528	4.201	0.815876646	17.62	23.78	2.695	2.031
SPBC1604.09C	SPBC1604.09c	0.205	4.206	0.688246139	18.99	26.2	2.319	2.521
SPBC29A3.08	pof4	0.06259	4.211	1.203495048	19.9	27.81	2.605	1.037
SPBC24C6.10C	dip1	0.3422	4.212	0.465719995	22.96	33.22	2.233	3.659
SPAC343.20	SPAC343.20	0.4805	4.236	0.318306608	22.64	32.61	2.6	5.167
SPBC3H7.08C	SPBC3H7.08c	0.4584	4.238	0.338755391	20.25	28.38	2.844	4.86
SPCC18B5.11C	cds1	0.02922	4.252	1.534319788	21.32	30.24	1.547	1.167
SPBC106.04	ada1	0.304	4.26	0.517126416	8.759	7.972	3.853	3.059
SPAC30D11.01C	SPAC30D11.01c	0.07965	4.261	1.09881422	20.33	28.48	2.563	1.413
SPAC644.06C	cdr1	0.311	4.263	0.507239611	9.684	9.608	3.271	3.275
SPCC126.01C	SPCC126.01c	0.2578	4.268	0.588717087	12.83	15.17	4.857	1.976
SPBC18E5.11C	edc3	0.1055	4.281	0.97674754	20	27.85	1.667	1.886
SPAC10F6.12C	mam4	0.4075	4.287	0.389872387	21.28	30.12	2.196	4.385
SPAC23H3.04	SPAC23H3.04	0.5023	4.292	0.299036822	21.75	30.94	3.58	5.437
SPCC31H12.04C	rpl1202	0.1152	4.318	0.938547521	16.31	21.25	3.507	0.9351
SPAPB24D3.01	SPAPB24D3.01	0.2327	4.319	0.633203617	19.24	26.44	1.229	2.888
SPAC1F3.05	SPAC1F3.05	0.2631	4.335	0.579879152	22.42	32.05	2.095	3.093
SPCC1742.01	SPCC1742.01	0.1505	4.349	0.8224635	20.25	28.18	3.296	1.873
SPAC16.05C	sfp1	0.1821	4.35	0.739690054	16.92	22.27	0.4583	2.516
SPBC1773.16C	SPBC1773.16c	0.04946	4.384	1.305745888	19.99	27.65	2.175	1.295
SPBP35G2.11C	SPBP35G2.11c	0.0518	4.39	1.28567024	15.33	19.39	1.893	1.423
SPBC1539.06	SPBC1539.06	0.3438	4.404	0.463694128	16.55	21.53	4.415	3.443
SPAC11H11.03C	SPAC11H11.03c	0.2613	4.431	0.58286059	15.8	20.14	4.629	2.436
SPCC14G10.04	SPCC14G10.04	0.1302	4.439	0.885389016	12.45	14.2	0.6166	2.154
SPCC16A11.04	snx12	0.1305	4.45	0.884389488	19.91	27.4	1.772	2.156
SPCC553.12C	SPCC553.12c	0.0452	4.453	1.344861565	19.25	26.23	2.55	0.7371
SPAC6F6.06C	rax2	0.1975	4.462	0.7044329	16.7	21.68	4.058	2.052

SPBC31F10.07	lsb5	0.152	4.479	0.818156412	16.76	21.76	3.871	1.571
SPAC1399.03	fur4	0.0669	4.496	1.174573882	15.23	19.02	2.083	1.588
SPCPJ732.01	vps5	0.427	4.503	0.369572125	9.451	8.768	4.452	4.531
SPAC18G6.10	lem2	0.3901	4.508	0.40882405	20.55	28.44	2.098	4.434
SPCC550.15C	SPCC550.15c	0.504	4.522	0.297569464	21.94	30.87	2.458	5.889
SPAC1071.06	arp9	0.02653	4.543	1.57626275	18.96	25.55	1.31	1.244
SPAC57A7.13	SPAC57A7.13	0.01013	4.548	1.994390555	20.43	28.14	0.4465	0.8635
SPBC15C4.06C	SPBC15C4.06c	0.2234	4.557	0.650916831	17.56	23.04	4.056	2.452
SPAC27E2.03C	SPAC27E2.03c	0.01043	4.561	1.981715692	20.74	28.67	1.63	0.3662
SPBC19C7.02	ubr1	0.2664	4.579	0.57446578	18.74	25.1	5.165	2.307
SPAC14C4.11	SPAC14C4.11	0.4818	4.593	0.317133204	23.44	33.4	3.147	5.589
SPCP1E11.07C	cwf18	0.09564	4.6	1.019360433	13.51	15.79	3.179	1.456
SPBC409.06	uch2	0.04001	4.651	1.397831449	19.16	25.72	1.582	1.435
SPAC3H1.12C	snt2	0.05065	4.652	1.29542055	17.6	22.95	1.677	1.547
SPBC530.08	SPBC530.08	0.3186	4.655	0.496754229	21.78	30.35	3.615	3.644
SPCC663.10	SPCC663.10	0.1081	4.674	0.966174306	18.24	24.05	2.757	1.906
SPAC144.03	ade2	0.0009605	4.68	3.017502631	19.74	26.68	0.777	0.5899
SPAC1399.04C	SPAC1399.04c	0.08536	4.698	1.068745594	19.77	26.71	3.188	1.349
SPAC926.03	rlc1	0.2987	4.699	0.524764777	18.85	25.08	2.13	3.688
SPAC17A2.11	SPAC17A2.11	0.01344	4.704	1.871600731	22.27	31.12	1.673	0.9768
SPBC16A3.10	SPBC16A3.10	0.006653	4.738	2.176982477	20.95	28.73	1.6	0.5486
SPAC1B3.01C	SPAC1B3.01c	3.64E-05	4.745	4.438540829	12.74	14.17	0.5716	0.2546
SPCC4B3.05C	hem12	0.1774	4.747	0.751046385	21.05	28.9	2.098	2.673
SPAC11G7.02	pub1	0.005403	4.766	2.267365032	16.95	21.6	1.389	0.2915
SPBC337.11	SPBC337.11	0.4404	4.772	0.35615269	25.14	36.1	5.334	4.838
SPBC2G2.01C	liz1	0.06351	4.794	1.197157887	12.67	13.97	2.028	1.696
SPBC1347.07	rex2	0.03174	4.797	1.498393078	18.13	23.63	2.171	1.21
SPBC215.04	git11	0.2395	4.798	0.620694482	17.09	21.78	2.522	3.193
SPBC3B8.02	php5	0.008275	4.834	2.082231998	20.66	28.05	0.8898	0.9574
SPAC26A3.11	SPAC26A3.11	0.02611	4.838	1.583193128	17.15	21.82	1.922	1.23
SPAC26F1.05	mug106	0.007855	4.861	2.104853811	21.86	30.12	1.694	0.5267
SPBP35G2.10	mit1	0.1128	4.872	0.9476909	20.5	27.69	1.923	2.208
SPBC13E7.03C	SPBC13E7.03c	0.03495	4.895	1.45655282	21.16	28.82	1.046	1.441
SPAC13F5.01C	msh1	0.06856	4.899	1.163929191	17.95	23.12	1.984	1.799
SPAC328.02	SPAC328.02	0.2349	4.904	0.629116983	23.76	33.42	2.902	3.179
SPBC8E4.01C	SPBC8E4.01c	0.007663	4.987	2.115601174	19.81	26.27	1.375	0.9684
SPBC56F2.08C	SPBC56F2.08c	0.0296	4.99	1.528708289	19.15	25.09	1.721	1.391
SPAC9G1.10C	SPAC9G1.10c	0.001604	5.003	2.794795636	18.58	24.07	1.212	0.6013
SPAC56F8.12	SPAC56F8.12	0.03967	5.016	1.4015378	18.11	23.21	1.623	1.549
SPAC11D3.03C	SPAC11D3.03c	0.05003	5.025	1.300769497	22.22	30.47	2.313	1.565
SPBC365.20C	pnc1	0.01452	5.033	1.838033384	17.68	22.42	1.539	1.149
SPBC947.03C	naa38	0.1778	5.04	0.750068243	22.84	31.54	1.153	2.88
SPCC18B5.06	SPCC18B5.06	0.04827	5.056	1.316322701	16.76	20.73	1.323	1.666
SPBC1709.18	tif452	0.2218	5.076	0.654038458	23.12	31.97	2.432	3.239
SPBC29A10.05	exo1	0.06674	5.087	1.175613798	22.2	30.33	2.108	1.842
SPBC17A3.09C	aim22	0.3007	5.111	0.521866572	16.18	19.61	3.507	3.894
SPAC1B3.16C	vht1	0.05115	5.19	1.291154362	22.09	29.94	0.4955	1.672
SPCC126.04C	sgf73	0.1984	5.208	0.702458332	21.8	29.39	2.476	3.108
SPAC19B12.10	sst2	0.2274	5.231	0.64320954	12.54	12.95	2.765	3.363
SPAC6C3.08	SPAC6C3.08	0.1022	5.247	0.990549104	23.52	32.38	2.406	2.24
SPAC167.07C	SPAC167.07c	0.1522	5.265	0.817585348	15.37	17.9	2.723	2.684
SPCC188.02	par1	0.3933	5.269	0.405276054	14.26	15.92	5.339	4.779
SPAC890.03	ppk16	0.02855	5.292	1.544393887	21.15	28.1	1.675	1.474
SPBC31F10.10C	SPBC31F10.10c	0.244	5.294	0.612610174	21.12	28.05	3.069	3.532
SPAC513.07	SPAC513.07	0.009067	5.31	2.042536384	21.54	28.76	1.863	0.9065
SPAC11G7.04	ubi1	0.03303	5.326	1.481091426	16.01	18.93	2.721	1.053
SPAC17C9.08	pnu1	0.02088	5.333	1.680269506	21.18	28.09	1.925	1.304
SPAC5D6.09C	mug86	0.04123	5.335	1.384786665	20.03	26.04	1.737	1.667
SPAC2F7.06C	pol4	0.07453	5.336	1.127668879	23.7	32.54	3.155	1.719
SPBC16H5.07C	ppa2	0.001559	5.375	2.807153885	19.53	25.09	1.303	0.4581
SPBC4.02C	SPBC4.02c	0.132	5.413	0.879426069	24.64	34.08	3.307	2.442
SPCC23B6.02C	SPCC23B6.02c	0.02344	5.446	1.630042393	23.21	31.48	1.341	1.437
SPCC306.11	SPCC306.11	0.1132	5.457	0.946153573	13.81	14.8	2.616	2.426
SPCC13B11.01	adh1	0.1165	5.468	0.933674075	18.7	23.44	3.175	2.342
SPAPB17E12.08	SPAPB17E12.08	0.04025	5.503	1.395234115	15.64	17.97	2.407	1.597
SPAC4F10.13C	mpd2	0.04335	5.503	1.363010898	12.11	11.7	2.287	1.681
SPAPB24D3.09C	pdr1	0.06158	5.537	1.210560315	20.97	27.36	3.451	1.275
SPAC29A4.09	SPAC29A4.09	0.1726	5.541	0.762959209	8.797	5.77	3.327	2.947
SPAC26A3.04	rpl2002	0.03357	5.548	1.474048659	21.18	27.71	1.951	1.607

SPCC1739.07	cti1	0.1397	5.562	0.854803594	13.69	14.41	3.712	2.49
SPAC16A10.01	SPAC16A10.01	0.002104	5.594	2.676954265	19.03	23.82	1.233	0.8125
SPAC17G8.09	shg1	0.06517	5.595	1.185952279	16.83	19.91	1.555	2.05
SPBC17A3.06	SPBC17A3.06	0.085	5.634	1.070581074	18.38	22.59	1.537	2.284
SPBC365.06	pmt3	0.08027	5.653	1.095446737	20.87	26.96	2.6	2.166
SPBC317.01	mbx2	0.007587	5.664	2.119929916	15.37	17.2	1.186	1.114
SPBP4H10.05C	spe2	0.3414	5.725	0.466736483	23.41	31.34	2.777	4.986
SPAC11E3.05	SPAC11E3.05	0.1605	5.736	0.794524963	14.92	16.27	2.391	3.072
SPBC11C11.07	rpl1801	0.1951	5.746	0.709742731	10.2	7.897	1.466	3.458
SPAC767.01C	vps1	0.005858	5.756	2.232250633	19.51	24.38	1.894	0.7051
SPBC15C4.01C	oca3	0.007931	5.759	2.10067205	22.45	29.58	1.299	1.148
SPAC821.05	SPAC821.05	0.1653	5.787	0.781727146	12.07	11.13	3.163	3.06
SPCC1322.14C	vtc4	0.002524	5.83	2.597910649	20.23	25.52	1.588	0.6775
SPBC577.02	rpl3801	0.3953	5.897	0.403073186	17.29	20.19	7.06	5.031
SPCC757.07C	ctt1	0.09393	5.905	1.027195678	19.04	23.28	3.768	2.073
SPAC4F10.14C	btf3	0.07084	5.924	1.149721447	17.9	21.22	1.605	2.238
SPAC694.05C	rps2502	0.3563	5.954	0.448184178	26.48	36.38	1.76	5.443
SPBC27B12.09C	SPBC27B12.09c	0.1199	6.005	0.921180817	24.09	32.05	1.783	2.819
SPBC16E9.11C	pub3	0.04231	6.023	1.373556975	21.6	27.6	3.36	1.132
SPAC8C9.19	SPAC8C9.19	0.01795	6.029	1.745935547	18.37	21.87	2.617	0.9714
SPCC663.11	saf1	0.03804	6.048	1.419759492	17.08	19.54	1.451	1.84
SPCC306.04C	set1	0.008319	6.068	2.079928876	23.6	31.07	0.9675	1.179
SPBC18E5.14C	SPBC18E5.14c	0.3572	6.105	0.44708855	14.88	15.56	6.868	4.715
SPAC3C7.04	SPAC3C7.04	0.206	6.116	0.68613278	17.67	20.47	2.314	3.775
SPCC1450.16C	SPCC1450.16c	0.02974	6.198	1.526659036	19.54	23.65	2.109	1.734
SPAC1556.08C	cbs2	0.1455	6.227	0.837137007	19.16	22.92	3.484	3.458
SPBC146.09C	lsd1	0.09901	6.235	1.004320939	17.7	20.33	3.196	2.56
SPBC1703.08C	SPBC1703.08c	0.04117	6.283	1.385419133	21.09	26.24	1.357	1.949
SPBC31F10.16	SPBC31F10.16	0.0441	6.294	1.355561411	21.44	26.84	1.796	2.017
SPAC16A10.03C	SPAC16A10.03c	0.3587	6.309	0.445268623	14.65	14.77	5.272	5.436
SPAC13G7.03	upf3	0.07445	6.353	1.128135298	22.48	28.59	1.934	2.447
SPAC23C11.04C	pnk1	0.02402	6.362	1.619426997	23.76	30.83	2.976	1.144
SPBC18H10.19	atg14	0.1482	6.364	0.829151796	16.41	17.81	2.028	3.302
SPBC1778.03C	SPBC1778.03c	0.02446	6.38	1.611543547	23.59	30.49	2.525	1.583
SPBC2D10.09	SPBC2D10.09	0.03031	6.434	1.518414064	23.32	29.92	2.066	1.824
SPCC16C4.13C	rpl1201	0.3302	6.477	0.481222931	15.25	15.55	5.337	5.171
SPAC664.14	amt2	0.106	6.52	0.974694135	20.69	25.1	1.642	2.896
SPBC776.02C	dis2	0.005573	6.55	2.253910957	19.86	23.59	1.89	1.126
SPCC18B5.10C	SPCC18B5.10c	0.1612	6.58	0.792634963	15.32	15.5	1.708	3.567
SPAC2C4.07C	SPAC2C4.07c	0.007766	6.581	2.109802614	23.62	30.2	2.191	1.123
SPAC22F8.07C	rtf1	0.04109	6.669	1.386263859	15.58	15.79	1.966	2.089
SPAC16E8.01	shd1	0.06735	6.679	1.1716624	18.94	21.73	3.954	1.987
SPAC824.07	SPAC824.07	0.01626	6.735	1.788879459	20.53	24.44	1.017	1.537
SPBC25D12.05	trm1	0.002253	6.863	2.647238808	20.84	24.77	1.389	1.03
SPAC869.06C	SPAC869.06c	0.1154	6.878	0.937794191	19.23	21.89	4.507	2.735
SPCC594.05C	spf1	0.06535	6.899	1.184754408	25.11	32.27	1.364	2.505
SPAP8A3.03	SPAP8A3.03	0.02525	6.92	1.597738618	19.96	23.11	1.981	1.868
SPBC713.07C	SPBC713.07c	0.06279	6.927	1.202109517	21.26	25.4	2.832	2.454
SPCC1223.05C	rpl3702	0.03703	6.985	1.431446288	19.76	22.64	3.096	1.937
SPCC4G3.19	alp16	0.04962	7.003	1.30434324	23.02	28.38	1.604	2.318
SPBC365.12C	ish1	0.027	7.233	1.568636236	21.81	25.84	3.495	1.343
SPAC11D3.15	SPAC11D3.15	0.2399	7.242	0.619969752	28.06	36.89	5.98	4.362
SPAC31A2.02	trm112	0.01333	7.254	1.875169851	16.86	17.02	1.778	1.646
SPCC1919.03C	amk2	0.1687	7.265	0.772884917	19.94	22.45	3.783	3.915
SPCC1672.12C	get4	0.254	7.342	0.595166283	14.46	12.62	7.474	4.008
SPCC1902.01	gaf1	0.00116	7.448	2.935542011	22.31	26.34	1.3	0.9753
SPBC15D4.06	naa30	0.001511	7.778	2.820735536	22.1	25.38	1.743	1.014
SPAC25A8.01C	fft3	0.0006453	7.812	3.190238335	19.91	21.44	1.145	0.9062
SPAC6F12.03C	fsv1	0.01377	7.976	1.86106606	20.09	21.47	3.228	1.226
SPCC364.03	rpl1702	0.007904	8.004	2.102153068	21	23.03	1.685	1.589
SPCC1827.03C	SPCC1827.03c	0.001191	8.169	2.924088239	22.55	25.48	1.195	1.058
SPAC22H10.11C	SPAC22H10.11c	0.02649	8.3	1.576918042	14.38	10.78	2.891	2.226
SPAC1006.09	win1	0.2731	8.312	0.5636783	26.75	32.68	6.165	5.773
SPAC20H4.10	ufd2	0.03254	8.448	1.487582451	27.9	34.47	1.606	2.412
SPAC1142.07C	vps32	0.135	8.842	0.869666232	12.77	6.968	3.734	4.338
SPAC18G6.13	SPAC18G6.13	0.0001956	8.976	3.70863115	22.07	23.2	1.276	0.8255
SPAC17G6.05C	SPAC17G6.05c	0.000964	8.998	3.015922966	23.85	26.32	1.306	1.121
SPAC19B12.11C	SPAC19B12.11c	0.003054	8.999	2.515130967	22.97	24.75	1.687	1.44
SPAC17C9.12	SPAC17C9.12	0.08048	9.01	1.094312032	17.51	15.07	6.169	2.259

SPBC25B2.03	SPBC25B2.03	0.01021	9.159	1.990974258	26.05	29.94	3.11	1.755
SPBC15D4.13C	SPBC15D4.13c	0.01898	9.368	1.721703792	26.15	29.75	2.83	2.319
SPAC9G1.02	wis4	0.0399	9.391	1.399027104	32.36	40.7	4.015	2.744
SPCC364.05	vps3	0.2558	9.738	0.59209946	19.15	16.68	5.857	6.683
SPAC1851.02	slc1	0.001589	10.17	2.798876103	24.43	25.27	0.682	1.147
SPBC28E12.06C	lvs1	0.004282	10.37	2.368353337	28.59	32.29	2.565	1.762
SPAC17G6.08	pep7	0.007674	10.55	2.114978205	28.47	31.77	2.403	2.087
SPBC887.10	mcs4	0.0378	11.01	1.4225082	31.25	35.86	2.699	3.345
SPBC215.14C	vps20	0.01796	11.1	1.745693668	27.6	29.24	2.853	2.727
SPBC3B9.09	vps36	0.03154	11.96	1.501138311	28.24	28.85	3.042	3.442
SPAC11E3.04C	ubc13	3.08E-05	12.07	4.511731385	32.38	36	1.252	0.7947
SPAC19A8.05C	sst4	0.02699	12.83	1.568797115	29.59	29.71	4.434	3.468
SPAC17A2.06C	vps8	0.04138	15.7	1.383209514	30.25	25.79	2.46	4.782

Supplementary Table 8: Altered fitness on Ammonia + Torin1 [5um]

R package version: 0.0-10
Summary type: mean
Test type: t-test
Control medium: DMSO_ammonia_AC
Control libraries: PDLV4_384
Query medium: 5uM_Torin1_ammonia_AC
Query libraries: PDLV4_384

Sensitive to Torin1
Relative resistance to Torin1

##

ORF	name	description	P	Q	EGI	Torin1 Fitness_Sum	DMSO Fitness_Sum	Torin1_SE	DMSO_SE
SPCC4E9.02	cig1	cyclin Cig1	0.001074	0.03851	-10.45	8.943	34.36	1.874	0.3581
SPBC21D10.11C	nfs1	mitochondrial cysteine desulfurase Nfs1 (predicted)	0.0002309	0.02247	-9.911	7.246	30.4	0.9279	0.09295
SPAC4F10.08	mug126	Schizosaccharomyces pombe specific protein	0.0007203	0.03504	-9.817	6.25	28.47	1.738	1.158
SPCC663.03	pmd1	leptomycin transmembrane transporter Pmd1	1.12E-06	0.001856	-9.405	7.49	29.94	0.5597	0.3487
SPBC15D4.09C	met3	cystathionine gamma-synthase Met3	0.0001159	0.02157	-9.363	0	16.59	0.6226	0
SPAC3H8.08C	SPAC3H8.08c	transcription factor (predicted)	0.009334	0.1314	-9.107	7.563	29.54	3.295	1.452
SPCC24B10.11C	tho7	THO complex subunit Tho7 (predicted)	0.01503	0.1823	-8.785	3.333	21.47	1.181	1.935
SPAC26F1.04C	etr1	enoyl-[acyl-carrier protein] reductase (predicted)	2.28E-07	0.0007528	-8.737	6.784	27.5	0.1737	0.1734
SPBC36.07	elp1	elongator subunit Elp1 (predicted)	0.01914	0.2146	-8.148	2.853	19.49	3.35	1.721
SPAC18G6.04C	shm2	serine hydroxymethyltransferase Shm2 (predicted)	0.04037	0.358	-8.247	6.836	26.73	2.96	2.536
SPAC1F7.09C	dal2	allantoicase Dal2	0.0006646	0.03489	-8.041	10.89	33.55	1.64	0.6077
SPCC1840.10	lsm8	U6 snRNP-associated protein Lsm8 (predicted)	0.001419	0.04307	-7.846	9.809	31.29	1.719	1.018
SPAC3H5.08C	SPAC3H5.08c	WD repeat protein, human WDR44 family	0.01662	0.195	-7.805	7.082	26.38	3.326	1.106
SPCC16C4.10	SPCC16C4.10	6-phosphogluconolactonase (predicted)	0.02212	0.24	-7.764	5.758	23.96	2.556	1.988
SPBC26H8.09C	snf59	SWI/SNF complex subunit Snf59	0.0002721	0.02389	-7.695	10.48	32.2	0.9931	0.2107
SPBC3E7.16C	leu3	2-isopropylmalate synthase Leu3	0.000192	0.02157	-7.65	11.15	33.32	0.784	0.126
SPAC328.01C	msn5	karyopherin (predicted)	0.03079	0.3023	-7.565	9.944	31.03	3.3	1.943
SPCC31H12.03C	SPCC31H12.03c	RNA binding protein (predicted)	0.02894	0.2898	-7.185	8.278	27.4	3.574	1.217
SPAC1834.04	hht1	histone H3 h3.1	0.0003582	0.02521	-7.077	8.526	27.65	1.299	0.5411
SPCC1442.11C	SPCC1442.11c	Schizosaccharomyces pombe specific protein	0.0004827	0.03002	-6.905	9.728	29.48	1.346	0.6082
SPAC1527.01	mok11	alpha-1,3-glucan synthase Mok11	0.0007203	0.03504	-6.876	10.14	30.15	1.39	0.7458
SPAC1F3.09	mug161	CwfJ family protein, splicing factor (predicted)	0.001063	0.03851	-6.83	8.541	27.24	1.182	0.205
SPCC1235.13	ght6	hexose transmembrane transporter Ght6	0.002416	0.05513	-6.767	13.57	36.04	1.542	0.3035
SPAC1783.06C	atg12	autophagy associated ubiquitin-like protein modifier Atg12	0.04475	0.3845	-6.757	10.68	30.9	1.634	2.166
SPAC30D11.06C	hfl1	Lazarus1 family transmembrane transporter	0.0001817	0.02157	-6.735	9.453	28.69	1.07	0.4184
SPBC337.04	ppk27	serine/threonine protein kinase Ppk27 (predicted)	0.03499	0.3279	-6.722	10.65	30.79	1.422	1.978
SPBC2G2.06C	apl1	AP-2 adaptor complex beta subunit Apl1 (predicted)	0.000892	0.03555	-6.641	11.29	31.77	1.307	0.3566
SPAC227.05	gim3	prefoldin subunit 4, Gim3 (predicted)	2.89E-05	0.01118	-6.546	11.08	31.23	0.5855	0.4254
SPBC19C7.12C	omh1	alpha-1,2-mannosyltransferase Omh1	1.75E-05	0.01118	-6.509	7.937	25.6	0.5773	0.3897
SPBC4B4.10C	atg5	autophagy associated protein Atg5	0.0006939	0.03504	-6.468	5.318	20.89	1.336	0.5026

SPBC2G2.05	rpl1603	60S ribosomal protein L13/L16 (predicted)	0.0008298	0.03555	-6.453	12.23	33.11	1.045	0.1842
SPAC31A2.14	bun107	WD repeat protein, human WDR48 family Bun107	0.03551	0.3299	-6.426	5.361	20.89	1.502	1.911
SPAC3H8.07C	pac10	prefoldin subunit 3 Pac10 (predicted)	0.01108	0.1458	-6.278	11.86	32.14	1.357	1.353
SPAC18B11.04	ncs1	neuronal calcium sensor related protein Ncs1	0.0006155	0.03338	-6.254	7.931	25.14	1.149	0.3292
SPAC23C4.12	hhp2	serine/threonine protein kinase Hhp2	0.0003854	0.02602	-6.236	6.914	23.3	1.165	0.5107
SPBC28F2.08C	hrd3	Hrd1 ubiquitin ligase complex subunit (predicted)	3.04E-05	0.01118	-6.161	7.504	24.22	0.6134	0.4068
SPBC1347.08C	SPBC1347.08c	ribonuclease H2 complex subunit (predicted)	0.0008063	0.03555	-6.137	9.174	27.13	0.9395	0.1429
SPBC19G7.07C	ppr3	mitochondrial PPR repeat protein Ppr3	0.002417	0.05513	-6.066	12.61	33.09	1.43	0.3152
SPBC405.05	atg16	autophagy associated protein Atg16	0.01039	0.1402	-6.061	6.857	22.89	2.029	1.186
SPCC191.09C	gst1	glutathione S-transferase Gst1	0.0008629	0.03555	-6.023	11.67	31.36	1.215	0.3609
SPAP8A3.13C	SPAP8A3.13c	Vid24 family protein (predicted)	0.0002738	0.02389	-6.021	13.97	35.42	1.053	0.4861
SPAC23G3.03	sib2	ornithine N5 monooxygenase (predicted)	0.0001365	0.02157	-5.994	9.429	27.33	0.921	0.4172
SPBC582.09	pex11	peroxisomal biogenesis factor 11 (predicted)	0.00204	0.05112	-5.984	11.83	31.57	1.38	0.3279
SPAC2H10.02C	nas2	26S proteasome regulatory particle assembly protein Nas2 (predicted)	0.001959	0.04985	-5.931	12.56	32.77	1.44	0.4137
SPAC1B3.06C	SPAC1B3.06c	UbiE family methyltransferase (predicted)	0.0004959	0.03002	-5.925	11.25	30.44	1.162	0.5167
SPCC1682.11C	ctl1	protein implicated in autophagy Ctl1	0.02834	0.2867	-5.916	6.591	22.16	1.658	1.653
SPAC343.18	rfp2	SUMO-targeted ubiquitin-protein ligase subunit Rfp2	0.001248	0.04128	-5.895	9.274	26.88	0.6902	0.734
SPAC3C7.01C	sac12	inositol polyphosphate phosphatase (predicted)	2.43E-05	0.01118	-5.865	8.7	25.81	0.628	0.2438
SPAC29A4.20	elp3	elongator complex subunit Elp3 (predicted)	0.04448	0.3841	-5.787	8.801	25.85	2.269	1.813
SPBC1289.16C	cao2	copper amine oxidase-like protein Cao2	0.01091	0.1449	-5.751	12.33	32.04	1.893	0.2125
SPCC1494.10	adn3	transcription factor Adn3	0.0001275	0.02157	-5.701	7.727	23.8	0.4263	0.4323
SPBC337.03	rhn1	RNA polymerase II transcription termination factor homolog	0.02176	0.2375	-5.686	12.56	32.34	2.536	1.109
SPBC21D10.09C	rkr1	RQC complex ubiquitin-protein ligase E3 Rkr1 (predicted)	2.42E-06	0.00267	-5.644	9.734	27.25	0.4189	0.2237
SPAC1002.19	urg1	GTP cyclohydrolase II Urg1 (predicted)	0.003061	0.06251	-5.632	11.61	30.55	1.588	0.5574
SPAC3G9.04	ssu72	phosphoric ester hydrolase Ssu72 (predicted)	0.03836	0.3476	-5.557	11.02	29.38	1.708	1.701
SPBC3B9.08C	mnh1	exon junction complex subunit, Mago-nashi homolog Mnh1	0.001146	0.03948	-5.544	11.44	30.1	1.145	0.3021
SPAC13G6.10C	asl1	cell wall protein Asl1, predicted O-glucosyl hydrolase	0.03742	0.3438	-5.534	0.7238	11.09	2.962	0.7238
SPCC74.06	mak3	histidine kinase Mak3	0.0004177	0.02763	-5.469	16.63	39.17	0.9998	0.354
SPAC6C3.03C	SPAC6C3.03c	Schizosaccharomyces pombe specific protein	0.001047	0.03851	-5.457	9.96	27.32	0.4418	0.5952
SPCC777.07	omh3	alpha-1,2-mannosyltransferase Omh3 (predicted)	0.004659	0.08469	-5.415	11.77	30.45	1.445	0.2481
SPBC20F10.02C	SPBC20F10.02c	DUF1741 family protein	0.0005643	0.03164	-5.388	11.37	29.7	0.9368	0.2486
SPCC16C4.07	scw1	RNA-binding protein Scw1	0.001089	0.03851	-5.355	10.03	27.26	1.077	0.2741
SPBC1105.01	SPBC1105.01	rRNA processing protein Rrp12-like (predicted)	0.0002309	0.02247	-5.353	11.57	30	0.9015	0.4376
SPAC890.07C	rmt1	type I protein arginine N-methyltransferase Rmt1	0.000192	0.02157	-5.349	11.89	30.54	0.8079	0.2679
SPCC1620.02	wtf23	wtf element Wtf23	0.0007189	0.03504	-5.342	10.67	28.38	0.9594	0.2394
SPAC31G5.18C	sde2	silencing defective protein Sde2	0.009465	0.1321	-5.297	4.575	17.49	1.852	0.9376
SPBPB2B2.02	mug180	esterase/lipase (predicted)	0.0008895	0.03555	-5.294	9.606	26.4	1.048	0.2905
SPBC16H5.04	snd301	SRP-independent ER targeting protein Snd3a (predicted)	0.002175	0.05292	-5.281	11.06	28.96	1.057	0.7866
SPBC1683.13C	cha4	transcription factor Cha4 (predicted)	0.0003482	0.02504	-5.273	13.01	32.4	0.9253	0.325
SPBC29A3.09C	gcn20	AAA family ATPase Gcn20 (predicted)	0.0002196	0.02247	-5.266	10.46	27.88	0.8841	0.4004
SPBC543.03C	pku80	Ku domain protein Pku80	0.002852	0.06049	-5.246	13.44	33.11	1.335	0.3274
SPAC6F12.12	par2	protein phosphatase PP2A regulatory subunit B-56 Par2	0.001857	0.04866	-5.225	9.951	26.89	1.237	0.3423
SPAC186.07C	SPAC186.07c	hydroxyacid dehydrogenase (predicted)	0.001646	0.04682	-5.216	11.85	30.24	1.294	0.4913
SPAC9E9.10C	cbh1	kinetochore protein, CENP-B homolog Cbh1	0.0009244	0.03607	-5.216	12.35	31.13	1.122	0.3867

SPAC458.06	atg1803	autophagy associated WD repeat protein Atg18c	0.03409	0.3241	-5.151	4.263	16.68	1.731	1.509
SPAC17C9.15C	SPAC17C9.15c	Schizosaccharomyces specific protein	0.04547	0.3876	-5.136	2.764	14	1.128	1.648
SPBC1734.05C	spf31	DNAJ protein Spf31 (predicted)	0.001828	0.04866	-5.134	10.84	28.32	1.302	0.4895
SPAC3C7.06C	pit1	serine/threonine protein kinase, meiotic Pit1	0.01902	0.214	-5.127	7.337	22.09	1.891	1.197
SPCC18B5.07C	nup61	nucleoporin Nup61	0.00711	0.112	-5.115	11.73	29.85	1.721	0.5017
SPBC17G9.05	rct1	RRM-containing cyclophilin regulating transcription Rct1	0.02402	0.2547	-5.108	11.68	29.75	2.342	0.5016
SPBC23G7.12C	rpt6	19S proteasome regulatory subunit Rpt6 (predicted)	0.004845	0.08716	-5.107	11.42	29.28	1.539	0.4246
SPAC23A1.15C	sec20	SNARE Sec20 (predicted)	0.0009268	0.03607	-5.103	12.29	30.83	1.124	0.4341
SPBC31E1.01C	atg2	autophagy associated protein Atg2	0.02113	0.233	-5.095	10.89	28.32	1.727	1.277
SPAC458.04C	dli1	meiotic dynein intermediate light chain Dli1/Dil1	0.001399	0.04307	-5.057	11.61	29.53	0.9152	0.1405
SPAC4F10.06	bud22	ribosome small subunit biogenesis protein, BUD22 family (predicted)	0.005676	0.09531	-5.043	11.44	29.2	1.441	0.259
SPCC1919.12C	erm2	metallopeptidase (predicted)	0.007915	0.1151	-5.004	11.59	29.4	1.745	0.5356
SPAC24B11.12C	SPAC24B11.12c	P-type ATPase (predicted)	0.000132	0.02157	-4.985	9.327	25.36	0.7238	0.4071
SPAC1399.02	SPAC1399.02	transmembrane transporter (predicted)	0.008778	0.1246	-4.981	11.94	30	1.723	0.425
SPAP32A8.02	SPAP32A8.02	xylose and arabinose reductase (predicted)	0.01708	0.1989	-4.937	13.83	33.26	1.055	1.185
SPBC8E4.02C	SPBC8E4.02c	Schizosaccharomyces pombe specific protein	0.004466	0.0822	-4.926	12.46	30.82	1.508	0.5055
SPAC17A5.16	ftp105	Ubp5 interacting protein Ftp105	0.03518	0.3283	-4.925	12.27	30.47	1.558	1.465
SPAC25A8.02	atg14	autophagy associated protein Atg14	0.0304	0.2993	-4.916	9.501	25.55	1.729	1.378
SPAC5H10.01	SPAC5H10.01	DUF1445 family mitochondrial protein (predicted)	3.56E-05	0.01118	-4.889	9.904	26.22	0.5456	0.198
SPBC13A2.02	nup82	nucleoporin Nup82	0.01111	0.1458	-4.883	11.06	28.25	1.574	0.116
SPBC1703.14C	top1	DNA topoisomerase I	0.001883	0.04866	-4.883	12.86	31.45	0.6137	0.6664
SPAC12G12.12	gms2	UDP-galactose transmembrane transporter Gms2 (predicted)	0.0005595	0.03164	-4.874	8.375	23.48	0.8003	0.1887
SPAPYUG7.03C	mid2	medial ring protein Mid2	0.0004777	0.03002	-4.801	10.93	27.88	0.9157	0.3412
SPCC1620.14C	snf22	ATP-dependent DNA helicase Snf22	0.001996	0.05041	-4.796	11.37	28.64	1.169	0.335
SPBC9B6.09C	mdl1	mitochondrial peptide-transporting ATPase	0.001684	0.04682	-4.795	10.81	27.65	1.079	0.2758
SPCC1620.07C	lnp1	lunapark Lnp1	0.001458	0.04374	-4.791	11.13	28.21	0.9949	0.2258
SPBC19C7.05	SPBC19C7.05	vesicle-mediated transport protein (predicted)	0.0002203	0.02247	-4.779	10.48	27.03	0.752	0.2559
SPBC17A3.03C	SPBC17A3.03c	phosphoprotein phosphatase (predicted)	0.01761	0.2029	-4.763	12.44	30.48	1.829	0.1752
SPBC11B10.06	sws1	SWIM domain containing-Srs2 interacting protein 1	0.0002858	0.02389	-4.761	11.67	29.12	0.836	0.3461
SPAC23C4.09C	SPAC23C4.09c	DNA-binding TFAR19-related protein (predicted)	0.009505	0.1321	-4.758	13.11	31.67	1.682	0.4133
SPBC1D7.01	gim6	prefoldin subunit 1 (predicted)	0.001783	0.04826	-4.691	13.42	32.1	1.158	0.3857
SPAC2F7.11	nrd1	RNA-binding protein Nrd1	0.002287	0.05414	-4.655	10.76	27.32	1.195	0.6163
SPAC13A11.05	ysp2	peptidase family M17 cytoplasmic leucyl aminopeptidase yspII (LAP yspI	0.002291	0.05414	-4.643	11.07	27.85	1.2	0.6079
SPAC3A12.08	SPAC3A12.08	acyl-coenzyme A thioesterase	0.002345	0.05424	-4.627	14.26	33.48	1.193	0.3648
SPAC27D7.02C	grp1	GRIP domain protein Grp1 (predicted)	0.01035	0.1402	-4.619	12.05	29.54	1.657	0.3842
SPAC664.07C	rad9	checkpoint clamp complex protein Rad9	0.0007428	0.03555	-4.606	10.87	27.42	0.9602	0.3531
SPBC32F12.03C	gpx1	glutathione peroxidase Gpx1	0.0008797	0.03555	-4.602	9.96	25.81	0.9014	0.2454
SPBC1604.11	atp17	F0-ATPase subunit F (predicted)	0.000127	0.02157	-4.591	9.392	24.78	0.5638	0.1526
SPBC1711.11	SPBC1711.11	autophagy associated protein (predicted)	0.01319	0.1679	-4.575	12.38	30.05	1.666	0.2635
SPCC4G3.05C	mus81	Holliday junction resolvase subunit Mus81	0.0001381	0.02157	-4.563	11.27	28.06	0.6867	0.2685
SPAC1F7.06	hsp3105	ThiJ domain protein	0.0001611	0.02157	-4.559	12.3	29.87	0.6574	0.3978
SPBC1271.10C	SPBC1271.10c	transmembrane transporter (predicted)	0.004949	0.08801	-4.55	9.992	25.77	0.4054	0.7067
SPBC15D4.12C	mug98	Schizosaccharomyces specific protein Mug98	0.001468	0.04374	-4.493	13.63	32.11	1.094	0.4472
SPAC1F8.01	ght3	hexose transmembrane transporter Ght3	0.03985	0.3567	-4.449	13.06	31.03	2.441	0.6509

SPBC1683.04	SPBC1683.04	glycosyl hydrolase family 3 (predicted)	0.0118	0.153	-4.446	10.47	26.43	1.47	0.1178
SPAC26A3.16	dph1	UBA domain protein Dph1	0.0003114	0.02389	-4.432	10.8	26.99	0.7821	0.3949
SPCC553.01C	dbl2	meiotic chromosome segregation protein Dbl2	0.01413	0.1769	-4.416	8.161	22.29	1.234	1.014
SPBC16A3.13	meu7	alpha-amylase homolog Aah4	0.005835	0.09641	-4.411	12.6	30.14	1.445	0.5993
SPAC25H1.02	jmj1	histone demethylase Jmj1 (predicted)	0.001335	0.04246	-4.403	10.77	26.89	1.05	0.4233
SPBC21B10.04C	nrf1	GTPase regulator Nrf1	0.009898	0.137	-4.403	12.07	29.2	1.489	0.2651
SPCC613.07	bcd1	snoRNA biogenesis protein Bcd1 (predicted)	0.003393	0.06705	-4.396	11.16	27.57	1.274	0.4643
SPCC794.03	SPCC794.03	amino acid permease (predicted)	0.000769	0.03555	-4.379	9.419	24.45	0.6885	0.5277
SPAC25G10.01	SPAC25G10.01	RNA-binding protein involved in histone acetylation	0.007361	0.1147	-4.367	10.49	26.33	1.198	0.04664
SPCC297.05	SPCC297.05	DENN domain Rab GDP-GTP exchange factor, unknown biological role (	0.02278	0.2462	-4.363	8.364	22.55	1.933	0.354
SPCC18.15	dph7	WD repeat protein, involved in diphthamide biosynthesis Dph7 (predictec	0.001139	0.03948	-4.341	11.11	27.37	0.2071	0.4196
SPAC9G1.04	oxa101	mitochondrial inner membrane translocase Oxa101	0.0007914	0.03555	-4.307	11.88	28.69	0.4859	0.4911
SPBC15D4.01C	klp9	kinesin-like protein Klp9	0.004473	0.0822	-4.285	12.21	29.23	1.085	0.1514
SPBC713.03	dld2	mitochondrial D-lactate dehydrogenase, cytochrome Dld2 (predicted)	0.007055	0.1117	-4.285	14.01	32.42	1.421	0.3846
SPBC16G5.02C	rbk1	ribokinase Rbk1 (predicted)	0.01063	0.1424	-4.28	12.43	29.61	1.624	0.5554
SPAC22E12.01	pet3	phosphoenolpyruvate transmembrane transporter Pet3	0.002456	0.05564	-4.268	12.45	29.63	1.153	0.4307
SPCC622.08C	hta1	histone H2A alpha	0.004994	0.08834	-4.267	11.93	28.7	1.29	0.3478
SPAC17D4.01	pex7	peroxin-7 (predicted)	0.00318	0.06414	-4.26	11.11	27.25	0.8772	0.03797
SPBC776.16	mis20	centromere protein Mis20/Eic2	0.003985	0.0762	-4.254	11.65	28.18	1.263	0.4128
SPBC16A3.02C	SPBC16A3.02c	mitochondrial conserved protein (predicted)	0.01505	0.1823	-4.24	12.77	30.15	1.679	0.3606
SPAC24C9.15C	spn5	meiotic septin Spn5	0.001732	0.04776	-4.232	10.88	26.77	0.9834	0.2709
SPBC14F5.13C	pho8	vacuolar membrane alkaline phosphatase (predicted)	0.0001633	0.02157	-4.225	8.558	22.65	0.5727	0.377
SPBC25B2.02C	mam1	M-factor transmembrane transporter Mam1	0.0008277	0.03555	-4.225	11.52	27.9	0.9067	0.4344
SPCC1223.09	uro1	uricase Uro1	5.58E-05	0.01317	-4.224	11.79	28.37	0.4662	0.3108
SPBC359.06	mug14	adducin	0.00224	0.05401	-4.214	9.049	23.5	1.068	0.319
SPCC1322.16	phb2	prohibitin Phb2 (predicted)	0.04256	0.3705	-4.193	4.744	15.84	1.715	1.278
SPAPB8E5.03	mae1	malic acid transport protein Mae1	0.00217	0.05292	-4.188	12.63	29.8	1.105	0.4696
SPBPB8B6.04C	grt1	transcription factor Grt1 (predicted)	0.0002866	0.02389	-4.182	11.83	28.38	0.6032	0.4161
SPAC25G10.06	rps2801	40S ribosomal protein S28 (predicted)	0.0003019	0.02389	-4.173	12.55	29.63	0.7356	0.2924
SPCC1223.12C	meu10	GPI anchored cell surface protein involved in ascospore wall assembly M	0.006082	0.09911	-4.172	11.38	27.56	1.184	0.1831
SPAC105.03C	SPAC105.03c	transcription factor (predicted)	0.02538	0.2657	-4.167	14.05	32.28	1.835	0.1966
SPCC645.07	rgf1	RhoGEF for Rho1, Rgf1	0.04504	0.386	-4.156	9.057	23.42	2.017	1.189
SPAC23C4.11	atp18	F0-ATPase subunit J (predicted)	0.006032	0.09879	-4.131	11.76	28.16	0.6357	0.7424
SPBC119.03	SPBC119.03	human COMT catechol O-methyltransferase homolog 1	0.0001689	0.02157	-4.125	12.54	29.53	0.6025	0.1973
SPBC2G2.15C	mrm2	mitochondrial 2' O-ribose methyltransferase Mrm2 (predicted)	0.0003106	0.02389	-4.124	11.82	28.25	0.7038	0.3897
SPAC24C9.07C	bgs2	spore wall 1,3-beta-glucan synthase catalytic subunit Bgs2	0.0001907	0.02157	-4.108	11.3	27.3	0.6296	0.3621
SPCC4B3.06C	SPCC4B3.06c	NADPH-dependent FMN reductase (predicted)	5.28E-05	0.01317	-4.098	11.65	27.91	0.487	0.2927
SPBC21D10.12	hob1	BAR adaptor protein Hob1	0.03112	0.3037	-4.095	11.54	27.7	1.536	1.14
SPCC63.04	mok14	alpha-1,4-glucan synthase Mok14	0.01426	0.177	-4.088	12.79	29.91	1.563	0.2937
SPAC2C4.08	SPAC2C4.08	conserved fungal protein	0.01458	0.1779	-4.086	12.87	30.04	1.683	0.5849
SPBC19C7.01	mni1	exon-exon junction complex disassembly factor Mni1 (predicted)	0.000826	0.03555	-4.082	8.683	22.62	0.7168	0.156
SPAC25B8.11	SPAC25B8.11	transcription factor (predicted)	0.01883	0.2133	-4.059	14.35	32.62	1.602	0.1645
SPAC22F8.03C	SPAC22F8.03c	Schizosaccharomyces pombe specific protein	3.97E-05	0.01118	-4.054	12.88	30.01	0.3977	0.2804
SPAC5H10.11	gmh1	alpha-1,2-galactosyltransferase Gmh1 (predicted)	0.0004709	0.03002	-4.05	11.79	28.07	0.7739	0.2944

SPAC1687.09	irs4	autophagy/CVT pathway ENTH/VHS domain protein Irs4 (predicted)	0.01717	0.1993	-4.025	12.55	29.37	1.611	0.2739
SPAC13G6.07C	rps601	40S ribosomal protein S6	0.001955	0.04985	-4.023	12.96	30.09	0.9554	0.2565
SPAP8A3.12C	tpp2	tripeptidyl-peptidase II Tpp2	0.001094	0.03851	-4.023	13.94	31.84	0.9199	0.4141
SPAC105.02C	SPAC105.02c	ankyrin repeat protein, human PPP1R16A ortholog	0.000585	0.03225	-4.008	12.24	28.79	0.709	0.4494
SPAC30D11.11	SPAC30D11.11	Haemolysin-III family protein (predicted)	0.004848	0.08716	-4.008	15.64	34.82	1.119	0.6703
SPBPB7E8.02	SPBPB7E8.02	PSP1 family protein	0.000772	0.03555	-4.006	9.815	24.49	0.5784	0.07452
SPCC548.05C	dbl5	ubiquitin-protein ligase E3 Dbl5	0.01551	0.1859	-3.993	13	30.12	1.642	0.437
SPBC56F2.04	utp20	U3 snoRNP protein Utp20 (predicted)	0.002769	0.06025	-3.958	11.94	28.17	1.082	0.3605
SPAC630.13C	tsc2	tuberin, GTPase activator Tsc2	0.001329	0.04246	-3.957	11.49	27.38	0.8111	0.1898
SPCC663.15C	SPCC663.15c	conserved fungal protein	0.001384	0.04307	-3.951	12.71	29.52	0.8732	0.5049
SPAC1002.20	SPAC1002.20	Schizosaccharomyces pombe specific protein	0.00803	0.1155	-3.921	12.67	29.39	1.368	0.6086
SPBC56F2.06	mug147	Schizosaccharomyces specific protein Mug147	0.002558	0.05679	-3.906	11.59	27.46	1.066	0.4047
SPBC23G7.14	SPBC23G7.14	Schizosaccharomyces specific protein	0.01446	0.1779	-3.889	13.48	30.78	1.59	0.49
SPAPB1A10.05	SPAPB1A10.05	Schizosaccharomyces specific protein	0.0003178	0.02389	-3.887	13.17	30.22	0.6945	0.3369
SPBC800.03	clr3	histone deacetylase (class II) Clr3	0.04932	0.4089	-3.879	7.835	20.76	0.96	1.285
SPCC188.08C	ubp5	ubiquitin C-terminal hydrolase Ubp5	0.004115	0.07796	-3.878	12.63	29.25	1.082	0.6088
SPBC16G5.03	SPBC16G5.03	ubiquitin-protein ligase E3 (predicted)	0.0001928	0.02157	-3.849	11.97	28.04	0.541	0.3537
SPBC13E7.07	SPBC13E7.07	Schizosaccharomyces specific protein	0.00282	0.06049	-3.827	11.16	26.56	1.057	0.3676
SPAC11G7.06C	mug132	S. pombe specific UPF0300 family protein 3	0.006589	0.1063	-3.815	11.9	27.86	0.4225	0.6603
SPBC1347.11	sro1	stress responsive orphan 1	0.002959	0.06119	-3.802	14.39	32.24	1.073	0.4356
SPBC947.06C	SPBC947.06c	spermidine family transmembrane transporter (predicted)	0.00232	0.05423	-3.79	11.83	27.69	0.7899	0.5707
SPCC18.01C	adg3	beta-glucosidase Adg3 (predicted)	0.007715	0.1151	-3.789	12.42	28.72	1.313	0.5753
SPAC23H4.12	alp13	MRG family Clr6 histone deacetylase complex subunit Alp13	0.002508	0.05642	-3.757	12.67	29.11	0.9359	0.239
SPAC24C9.08	cps1	vacuolar carboxypeptidase (predicted)	0.003126	0.06345	-3.755	11.98	27.88	0.7572	0.6056
SPBC14C8.04	ilv6	acetolactate synthase regulatory unit Ilv6 (predicted)	0.001781	0.04826	-3.74	13.43	30.42	0.8164	0.5211
SPAC110.01	ppk1	serine/threonine protein kinase Ppk1 (predicted)	0.0005005	0.03002	-3.732	11.94	27.78	0.5986	0.4126
SPCC1020.07	SPCC1020.07	pseudouridine-5'-phosphatase (predicted)	0.001302	0.04222	-3.724	9.527	23.48	0.853	0.4434
SPCC126.08C	SPCC126.08c	lectin family glycoprotein receptor (predicted)	0.0208	0.2317	-3.72	14.02	31.44	1.616	0.7505
SPBP23A10.02	pkr1	V-ATPase assembly factor Pkr1 (predicted)	0.005052	0.08843	-3.718	11.14	26.34	1.136	0.3169
SPCC4G3.15C	not2	CCR4-Not complex NOT box subunit Not2	0.03449	0.3269	-3.704	11.81	27.5	1.84	0.2304
SPAC6C3.04	cit1	citrate synthase Cit1	0.0386	0.3488	-3.697	11.47	26.88	1.541	1.077
SPBC577.11	SPBC577.11	DUF3074 family protein	0.002855	0.06049	-3.68	11.1	26.19	0.9542	0.2491
SPAC589.08C	dam1	DASH complex subunit Dam1	0.02172	0.2375	-3.65	13.26	29.97	1.672	0.5133
SPBC1539.02	SPBC1539.02	conserved eukaryotic nuclear protein implicated in meiotic chromosome s	0.004411	0.08198	-3.644	12.27	28.19	1.077	0.3038
SPBC19C2.02	pmt1	tRNA methyltransferase Pmt1	0.007384	0.1147	-3.641	9.952	24.09	1.244	0.5585
SPCC1223.04C	set11	ribosomal protein lysine methyltransferase Set11	0.003811	0.0733	-3.617	11.04	25.98	1.073	0.4682
SPAC26H5.10C	tif51	translation elongation factor eIF5A (predicted)	0.003726	0.07268	-3.61	13.41	30.16	1.067	0.4611
SPAC22E12.04	ccs1	superoxide dismutase copper chaperone Ccs1	0.01148	0.1496	-3.582	3.284	12.17	1.387	0.5072
SPCC24B10.20	SPCC24B10.20	short chain dehydrogenase (predicted)	4.06E-05	0.01118	-3.572	11.93	27.47	0.3724	0.2482
SPBC83.05	SPBC83.05	mitochondrial RNA-binding protein (predicted)	0.01531	0.1842	-3.549	12.52	28.48	1.456	0.3923
SPCC569.07	SPCC569.07	aromatic aminotransferase (predicted)	0.001063	0.03851	-3.537	11.88	27.33	0.5723	0.4541
SPCC736.13	SPCC736.13	short chain dehydrogenase (predicted)	0.02318	0.2498	-3.527	13.51	30.19	1.63	0.4252
SPBC2G2.08	ade9	formyltetrahydrofolatesynthetase Ade9	0.0008432	0.03555	-3.524	10.26	24.43	0.7519	0.2719
SPAP27G11.10C	nup184	nucleoporin Nup184	0.007875	0.1151	-3.521	11.96	27.43	1.219	0.3586

SPAC227.10	gim4	prefoldin subunit 2, Gim4(predicted)	0.003801	0.0733	-3.514	12.57	28.5	1.008	0.2933
SPAC14C4.13	rad17	RFC related checkpoint protein Rad17	0.002918	0.06109	-3.461	12.12	27.62	0.9649	0.3378
SPAC15A10.13	ppk3	protein kinase domain and HEAT repeat protein Ppk3	0.007018	0.1116	-3.46	12.43	28.15	0.9072	0.6629
SPCC1183.10	wtf10	wtf element Wtf10	0.00123	0.04109	-3.448	13.69	30.38	0.7256	0.4387
SPBC13E7.11	rbd1	mitochondrial rhomboid protease (predicted)	0.005413	0.09226	-3.445	10.8	25.25	0.5893	0.6148
SPAC630.05	gyp7	GTPase activating protein Gyp7 (predicted)	0.0013	0.04222	-3.411	13.91	30.7	0.7231	0.4396
SPAC32A11.03C	phx1	stationary phase-specific homeobox transcription factor Phx1	0.01016	0.1395	-3.392	11.27	25.98	1.207	0.2758
SPCC1919.13C	bmt5	ribosome biogenesis protein Bmt5 (predicted)	0.01036	0.1402	-3.389	14.54	31.77	1.279	0.4687
SPCC320.07C	mde7	RNA-binding protein Mde7	0.007204	0.1129	-3.382	13.58	30.06	1.166	0.4686
SPAC869.02C	yhb1	nitric oxide dioxygenase Yhb1	0.0002874	0.02389	-3.356	11.81	26.89	0.52	0.3298
SPCC4B3.11C	fra3	mitochondrial transcriptional repressor protein BolA domain (predicted)	0.0007004	0.03504	-3.331	12.56	28.16	0.6676	0.3613
SPCC24B10.06	SPCC24B10.06	Schizosaccharomyces specific protein, predicted GPI anchor	0.029	0.2898	-3.329	13.72	30.21	1.537	0.1624
SPCC553.03	pex1	AAA family ATPase Pex1 (predicted)	0.02391	0.2547	-3.321	12.2	27.5	1.508	0.6796
SPBC685.02	exo5	mitochondrial single stranded DNA specific 5'-3' exodeoxyribonuclease E	0.00275	0.06025	-3.317	11.48	26.22	0.8977	0.287
SPCC1235.15	dga1	diacylglycerol O-acyltransferase Dga1	0.001413	0.04307	-3.301	12.2	27.48	0.5921	0.4501
SPBC4F6.08C	mrpl39	mitochondrial ribosomal protein subunit L39 (predicted)	0.01514	0.1828	-3.281	11.06	25.42	1.364	0.4853
SPBC2G5.06C	hmt2	sulfide-quinone oxidoreductase	0.0003759	0.02591	-3.277	5.314	15.22	0.5794	0.3206
SPAC1834.09	mug51	variant protein kinase 19 family protein	0.02355	0.2521	-3.266	12.55	28.04	1.532	0.5166
SPBC1604.01	egt1	Ergothioneine biosynthesis protein Egt1	0.03143	0.3058	-3.241	13.9	30.38	1.644	0.5983
SPBC19C2.06C	mug124	Schizosaccharomyces pombe specific protein	0.001795	0.04826	-3.237	11.74	26.55	0.8123	0.2917
SPCC1450.09C	SPCC1450.09c	phospholipase (predicted)	0.0008753	0.03555	-3.225	14.72	31.79	0.5415	0.399
SPBC1683.02	SPBC1683.02	adenine deaminase (predicted)	0.004124	0.07796	-3.214	11.6	26.25	0.9732	0.4119
SPBC1703.13C	SPBC1703.13c	mitochondrial inorganic phosphate transmembrane transporter (predictec	0.01428	0.177	-3.208	11.85	26.68	1.29	0.3467
SPAC17A5.10	hua1	conserved fungal protein, possibly DNA J domain Hua1	0.007859	0.1151	-3.206	12.57	27.96	1.123	0.3634
SPAC29A4.13	ure6	urease accessory protein UreF	0.01652	0.195	-3.16	13.09	28.8	1.29	0.6079
SPBC21C3.03	SPBC21C3.03	ABC1 kinase family protein	0.003387	0.06705	-3.157	10.55	24.3	0.6719	0.5177
SPBC23G7.06C	SPBC23G7.06c	ERMES complex complex subunit Mmm1-related protein	0.001867	0.04866	-3.129	11.88	26.6	0.7947	0.3636
SPBC1703.06	pof10	F-box protein Pof10	0.01618	0.1933	-3.097	13.12	28.74	1.242	0.2481
SPAC23D3.04C	gpd2	glycerol-3-phosphate dehydrogenase Gpd2	0.0282	0.2862	-3.084	7.632	18.99	1.48	0.2972
SPCC70.06	sac32	nuclear export factor Sac32 (predicted)	0.01228	0.158	-3.069	10.52	24.09	1.15	0.2571
SPBC18E5.01	SPBC18E5.01	cycloisomerase 2 family	0.01417	0.1769	-3.06	12.86	28.22	1.251	0.4252
SPBC17D1.05	SPBC17D1.05	Schizosaccharomyces specific protein	0.04585	0.3899	-3.042	8.699	20.81	1.7	0.6879
SPCC320.03	SPCC320.03	transcription factor (predicted)	0.01149	0.1496	-3.034	15.13	32.18	0.3842	0.617
SPBC1703.09	SPBC1703.09	Schizosaccharomyces specific protein	0.007732	0.1151	-3.021	15.38	32.61	1.039	0.4758
SPAC144.17C	SPAC144.17c	6-phosphofructo-2-kinase (predicted)	0.003735	0.07268	-3.005	10.7	24.29	0.8547	0.2447
SPBC17G9.08C	cnt5	Centaurin Cnt5	0.003405	0.06705	3.006	9.576	11.64	0.5651	0.4922
SPAC4D7.01C	sec71	Sec7 domain protein, ARF GEF (predicted)	0.0108	0.1441	3.162	21.3	32.14	0.2796	0.6033
SPBC25B2.04C	mtg1	mitochondrial GTPase involved in translation Mtg1 (predicted)	0.02722	0.278	3.27	11.34	14.31	0.6342	0.8861
SPAC630.15	mug177	Schizosaccharomyces pombe specific protein	0.02566	0.2678	3.287	17.67	25.49	1.15	0.8716
SPCC663.09C	SPCC663.09c	short chain dehydrogenase (predicted)	0.004192	0.07879	3.567	18.78	26.97	1.087	0.4324
SPAC2E1P5.03	erj5	DNAJ domain protein Erj5 (predicted)	0.001883	0.04866	3.653	11.63	14.13	0.9327	0.3529
SPBC119.05C	lsb1	Wiskott-Aldrich syndrome homolog binding protein Lsb1 (predicted)	0.0132	0.1679	3.725	18.86	26.82	0.8033	0.8381
SPCPB16A4.03C	ade10	bifunctional IMP cyclohydrolase/phosphoribosylaminoimidazolecarboxan	0.03163	0.3059	3.729	10.75	12.44	1.394	1.045
SPAC14C4.07	SPAC14C4.07	transmembrane transporter (predicted)	0.04718	0.3981	3.737	17.37	24.17	1.515	1.188

SPAC1783.02C	vps66	1-acylglycerol-3-phosphate O-acyltransferase Vps66 (predicted)	0.009409	0.1319	3.75	16.55	22.68	0.8414	0.7787
SPAC6C3.06C	SPAC6C3.06c	P-type ATPase, calcium transporting (predicted)	0.02862	0.2878	3.843	19.08	27	1.128	1.076
SPBC16E9.13	ksp1	serine/threonine protein kinase Ksp1 (predicted)	0.03754	0.344	3.901	20.13	28.76	1.643	1.119
SPAC14C4.15C	dpp1	dipeptidyl peptidase (predicted)	0.0364	0.3373	3.916	18	24.96	1.122	1.18
SPBC1709.05	sks2	heat shock protein, ribosome associated molecular chaperone Sks2	0.04771	0.401	3.948	18.78	26.29	1.989	1.128
SPBP22H7.05C	abo2	ATPase with bromodomain protein (predicted)	0.006179	0.1002	3.959	19.68	27.86	1.284	0.3737
SPBC30D10.16	pha2	phrenate dehydratase	0.001396	0.04307	3.997	6.604	4.621	0.9123	0.2798
SPBP18G5.03	toc1	Tor complex Tor2 interacting protein 1	0.02134	0.2345	4.022	20.76	29.66	1.834	0.5752
SPAC56E4.07	SPAC56E4.07	N-acetyltransferase (predicted)	0.04646	0.3939	4.184	18.89	26.06	2.245	1.076
SPAC3H8.09C	nab3	Nrd1 complex poly(A) binding protein Nab3 (predicted)	0.0188	0.2133	4.186	12.72	15.12	1.583	0.9578
SPCC18B5.11C	cds1	replication checkpoint kinase Cds1	0.02922	0.2911	4.252	21.32	30.24	1.547	1.167
SPBC1773.16C	SPBC1773.16c	transcription factor, zf-fungal binuclear cluster type(predicted)	0.04946	0.409	4.384	19.99	27.65	2.175	1.295
SPCC553.12C	SPCC553.12c	transmembrane transporter (predicted)	0.0452	0.3864	4.453	19.25	26.23	2.55	0.7371
SPAC1071.06	arp9	SWI/SNF and RSC complex subunit Arp9	0.02653	0.2742	4.543	18.96	25.55	1.31	1.244
SPAC57A7.13	SPAC57A7.13	RNA-binding protein, involved in splicing (predicted)	0.01013	0.1395	4.548	20.43	28.14	0.4465	0.8635
SPAC27E2.03C	SPAC27E2.03c	Obg-Like ATPase (predicted)	0.01043	0.1402	4.561	20.74	28.67	1.63	0.3662
SPBC409.06	uch2	ubiquitin C-terminal hydrolase Uch2	0.04001	0.3567	4.651	19.16	25.72	1.582	1.435
SPAC144.03	ade2	adenylosuccinate synthetase Ade2	0.0009605	0.03665	4.68	19.74	26.68	0.777	0.5899
SPAC17A2.11	SPAC17A2.11	Schizosaccharomyces pombe specific protein	0.01344	0.1697	4.704	22.27	31.12	1.673	0.9768
SPBC16A3.10	ale1	membrane bound O-acyltransferase, MBOAT Ale1 (predicted)	0.006653	0.1068	4.738	20.95	28.73	1.6	0.5486
SPAC1B3.01C	SPAC1B3.01c	uracil phosphoribosyltransferase (predicted)	3.64E-05	0.01118	4.745	12.74	14.17	0.5716	0.2546
SPAC11G7.02	pub1	HECT-type ubiquitin-protein ligase E3 Pub1	0.005403	0.09226	4.766	16.95	21.6	1.389	0.2915
SPBC1347.07	rex2	RNA exonuclease (predicted)	0.03174	0.3061	4.797	18.13	23.63	2.171	1.21
SPBC3B8.02	php5	CCAAT-binding factor complex subunit Php5	0.008275	0.1185	4.834	20.66	28.05	0.8898	0.9574
SPAC26A3.11	SPAC26A3.11	omega-amidase (predicted)	0.02611	0.2716	4.838	17.15	21.82	1.922	1.23
SPAC26F1.05	mug106	Schizosaccharomyces pombe specific protein Mug106	0.007855	0.1151	4.861	21.86	30.12	1.694	0.5267
SPBC13E7.03C	SPBC13E7.03c	Smaug family RNA binding protein involved in CCR4-NOT dependent de	0.03495	0.3279	4.895	21.16	28.82	1.046	1.441
SPBC8E4.01C	pho84	inorganic phosphate transmembrane transporter (predicted)	0.007663	0.1151	4.987	19.81	26.27	1.375	0.9684
SPBC56F2.08C	puf1	pumilio family RNA-binding protein Puf1 (predicted)	0.0296	0.294	4.99	19.15	25.09	1.721	1.391
SPAC9G1.10C	inp53	inositol polyphosphate phosphatase Inp53 (predicted)	0.001604	0.04614	5.003	18.58	24.07	1.212	0.6013
SPAC56F8.12	SPAC56F8.12	conserved fungal protein	0.03967	0.3566	5.016	18.11	23.21	1.623	1.549
SPBC365.20C	pnc1	nicotinamidase Pnc1 (predicted)	0.01452	0.1779	5.033	17.68	22.42	1.539	1.149
SPCC18B5.06	dom34	Dom34-Hbs1 translation release factor complex subunit, peloto ortholog	0.04827	0.4032	5.056	16.76	20.73	1.323	1.666
SPAC890.03	ppk16	serine/threonine protein kinase Ppk16 (predicted)	0.02855	0.2878	5.292	21.15	28.1	1.675	1.474
SPAC513.07	SPAC513.07	flavonol reductase/cinnamoyl-CoA reductase family	0.009067	0.1282	5.31	21.54	28.76	1.863	0.9065
SPAC11G7.04	ubi1	ribosomal-ubiquitin fusion protein Ubi1 (predicted)	0.03303	0.3158	5.326	16.01	18.93	2.721	1.053
SPAC17C9.08	pnu1	mitochondrial endodeoxyribonuclease Pnu1	0.02088	0.2317	5.333	21.18	28.09	1.925	1.304
SPAC5D6.09C	mug86	acetate transmembrane transporter (predicted)	0.04123	0.3627	5.335	20.03	26.04	1.737	1.667
SPBC16H5.07C	ppa2	serine/threonine protein phosphatase Ppa2	0.001559	0.04563	5.375	19.53	25.09	1.303	0.4581
SPCC23B6.02C	SPCC23B6.02c	pre-ribosomal factor (predicted)	0.02344	0.2517	5.446	23.21	31.48	1.341	1.437
SPAC4F10.13C	mpd2	GYF domain protein	0.04335	0.3764	5.503	12.11	11.7	2.287	1.681
SPAPB17E12.08	eos1	N-glycosylation protein Eos1 (predicted)	0.04025	0.3579	5.503	15.64	17.97	2.407	1.597
SPAC26A3.04	rpl2002	60S ribosomal protein L20 (predicted)	0.03357	0.32	5.548	21.18	27.71	1.951	1.607
SPAC16A10.01	SPAC16A10.01	ThrE amino acid transmembrane transporter family protein	0.002104	0.05214	5.594	19.03	23.82	1.233	0.8125

SPBC317.01	mbx2	MADS-box transcription factor Pvg4	0.007587	0.1151	5.664	15.37	17.2	1.186	1.114
SPAC767.01C	vps1	dynamin family protein Vps1	0.005858	0.09641	5.756	19.51	24.38	1.894	0.7051
SPBC15C4.01C	oca3	TPR repeat protein Oca3/ ER membrane protein complex Ecm2 (predicte	0.007931	0.1151	5.759	22.45	29.58	1.299	1.148
SPCC1322.14C	vtc4	vacuolar transporter chaperone (VTC) complex subunit (predicted)	0.002524	0.05642	5.83	20.23	25.52	1.588	0.6775
SPBC16E9.11C	pub3	HECT-type ubiquitin-protein ligase E3 Pub3 (predicted)	0.04231	0.3693	6.023	21.6	27.6	3.36	1.132
SPAC8C9.19	SPAC8C9.19	conserved fungal protein	0.01795	0.2049	6.029	18.37	21.87	2.617	0.9714
SPCC663.11	saf1	splicing associated factor Saf1	0.03804	0.3467	6.048	17.08	19.54	1.451	1.84
SPCC306.04C	set1	histone lysine methyltransferase Set1	0.008319	0.1186	6.068	23.6	31.07	0.9675	1.179
SPCC1450.16C	ptl1	triacylglycerol lipase ptl1	0.02974	0.2946	6.198	19.54	23.65	2.109	1.734
SPBC1703.08C	SPBC1703.08c	5-formyltetrahydrofolate cyclo-ligase (predicted)	0.04117	0.3627	6.283	21.09	26.24	1.357	1.949
SPBC31F10.16	bch1	exocyst complex ChAPs family (Chs5p-Arf1p-binding) protein Bch1	0.0441	0.3819	6.294	21.44	26.84	1.796	2.017
SPAC23C11.04C	pnk1	DNA kinase/phosphatase Pnk1	0.02402	0.2547	6.362	23.76	30.83	2.976	1.144
SPBC1778.03C	SPBC1778.03c	NADH pyrophosphatase (predicted)	0.02446	0.2585	6.38	23.59	30.49	2.525	1.583
SPBC2D10.09	snr1	3-hydroxyisobutyryl-CoA hydrolase snr1	0.03031	0.2993	6.434	23.32	29.92	2.066	1.824
SPBC776.02C	dis2	serine/threonine protein phosphatase PP1, Dis2	0.005573	0.09405	6.55	19.86	23.59	1.89	1.126
SPAC2C4.07C	dis32	3'-5'-exoribonuclease activity Dis3L2	0.007766	0.1151	6.581	23.62	30.2	2.191	1.123
SPAC22F8.07C	rtf1	replication termination factor Rtf1	0.04109	0.3627	6.669	15.58	15.79	1.966	2.089
SPAC824.07	glo2	glyoxalase II	0.01626	0.1934	6.735	20.53	24.44	1.017	1.537
SPBC25D12.05	trm1	tRNA (guanine-N2-)-methyltransferase Trm1	0.002253	0.05401	6.863	20.84	24.77	1.389	1.03
SPAP8A3.03	zrt2	ZIP zinc transmembrane transporter Zrt2 (predicted)	0.02525	0.2652	6.92	19.96	23.11	1.981	1.868
SPCC1223.05C	rpl3702	60S ribosomal protein L37 (predicted)	0.03703	0.3412	6.985	19.76	22.64	3.096	1.937
SPCC4G3.19	alp16	gamma tubulin complex subunit Alp16	0.04962	0.4093	7.003	23.02	28.38	1.604	2.318
SPBC365.12C	ish1	LEA domain protein	0.027	0.2766	7.233	21.81	25.84	3.495	1.343
SPAC31A2.02	trm112	eRF1 methyltransferase complex and tRNA (m2G10) methyltransferase (	0.01333	0.169	7.254	16.86	17.02	1.778	1.646
SPCC1902.01	gaf1	transcription factor Gaf1	0.00116	0.03955	7.448	22.31	26.34	1.3	0.9753
SPBC15D4.06	naa30	NatC N-acetyltransferase complex catalytic subunit Naa30 (predicted)	0.001511	0.04463	7.778	22.1	25.38	1.743	1.014
SPAC25A8.01C	fft3	SMARCAD1 family ATP-dependent DNA helicase Fft3	0.0006453	0.03443	7.812	19.91	21.44	1.145	0.9062
SPAC6F12.03C	fsv1	SNARE Fsv1	0.01377	0.1732	7.976	20.09	21.47	3.228	1.226
SPCC364.03	rpl1702	60S ribosomal protein L17 (predicted)	0.007904	0.1151	8.004	21	23.03	1.685	1.589
SPCC1827.03C	SPCC1827.03c	acetyl-CoA ligase (predicted)	0.001191	0.04021	8.169	22.55	25.48	1.195	1.058
SPAC22H10.11C	SPAC22H10.11c	TOR signaling pathway transcriptional corepressor Crf1 (predicted)	0.02649	0.2742	8.3	14.38	10.78	2.891	2.226
SPAC20H4.10	ufd2	ubiquitin-protein ligase E4 Ufd2 (predicted)	0.03254	0.312	8.448	27.9	34.47	1.606	2.412
SPAC18G6.13	SPAC18G6.13	Schizosaccharomyces specific protein	0.0001956	0.02157	8.976	22.07	23.2	1.276	0.8255
SPAC17G6.05C	bro1	BRO1 domain protein Bro1 (predicted)	0.000964	0.03665	8.998	23.85	26.32	1.306	1.121
SPAC19B12.11C	SPAC19B12.11c	zinc finger protein, human ZNF593 ortholog	0.003054	0.06251	8.999	22.97	24.75	1.687	1.44
SPBC25B2.03	SPBC25B2.03	zf-C3HC4 type zinc finger	0.01021	0.1395	9.159	26.05	29.94	3.11	1.755
SPBC15D4.13C	SPBC15D4.13c	human ASCC1 ortholog	0.01898	0.214	9.368	26.15	29.75	2.83	2.319
SPAC9G1.02	wis4	MAP kinase kinase kinase Wis4	0.0399	0.3567	9.391	32.36	40.7	4.015	2.744
SPAC1851.02	slc1	1-acylglycerol-3-phosphate O-acyltransferase Slc1 (predicted)	0.001589	0.04612	10.17	24.43	25.27	0.682	1.147
SPBC28E12.06C	lvs1	beige protein homolog Lvs1 (predicted)	0.004282	0.08002	10.37	28.59	32.29	2.565	1.762
SPAC17G6.08	pep7	prevacuole/endosomal FYVE tethering component Pep7 (predicted)	0.007674	0.1151	10.55	28.47	31.77	2.403	2.087
SPBC887.10	mcs4	response regulator Mcs4	0.0378	0.3455	11.01	31.25	35.86	2.699	3.345
SPBC215.14C	vps20	ESCRT III complex subunit Vps20	0.01796	0.2049	11.1	27.6	29.24	2.853	2.727
SPBC3B9.09	vps36	ESCRT II complex subunit Vps36	0.03154	0.3059	11.96	28.24	28.85	3.042	3.442

SPAC11E3.04C	ubc13	ubiquitin conjugating enzyme E2 Ubc13	3.08E-05	0.01118	12.07	32.38	36	1.252	0.7947
SPAC19A8.05C	sst4	sorting receptor for ubiquitinated membrane proteins, ESCRT 0 complex	0.02699	0.2766	12.83	29.59	29.71	4.434	3.468
SPAC17A2.06C	vps8	CORVET complex WD repeat/ ubiquitin-protein ligase E3 subunit Vps8 (p	0.04138	0.3631	15.7	30.25	25.79	2.46	4.782

Supplementary Table 9: Top 150 Red strains on Glutamate + Phloxin B = Top 4.5%

R package version: 0.0-10
Summary type: mean
medium: Glutamate_Phloxin B
libraries: PDLV4_384
##

All strains:
Average redness = 792
Average Standard error = 49.85

fitness on Glutamate vs YES

Red = strains shown in Fig 5.

ORF	name	description	Glutamate_redness Mean (4 repeats)	Glutamate _redness Standard Error	P	EGI	Glutamate Fitness_Sum	YES Fitness_Sum	Glutamate_SE	YES_SE
SPCC18B5.05C	SPCC18B5.05c	phosphomethylpyrimidine kinase (predicted)	1974	22.4	0.214	-6.593	30.18	51.6	5.679	2.1
SPAC1805.16C	SPAC1805.16c	purine nucleoside phosphorylase (predicted)	1727	13.82	0.4439	3.827	34.71	43.33	2.708	3.897
SPBC21D10.08C	SPBC21D10.08c	conserved fungal protein	1622	55.86	0.5889	-3.361	32.63	50.5	7.347	2.376
SPCC4B3.15	mid1	medial ring protein Mid1	1621	189.3	0.626	1.07	30.06	40.67	1.233	1.842
SPAC29E6.01	pof11	F-box protein Pof11	1602	63.81	0.5937	2.631	30.32	38.86	4.984	3.025
SPAC1006.09	win1	MAP kinase kinase kinase Win1	1496	78.36	0.7982	-1.899	35.6	52.61	7.785	4.412
SPBC3D6.05	ptp4	phosphatidate cytidylyltransferase Ptp4 (predicted)	1478	128.2	0.9341	-0.6773	25.69	37	7.955	5.429
SPAC13G7.07	arb2	argonaute binding protein 2	1431	53.44	0.1485	5.679	33.91	39.62	2.508	2.817
SPBC31F10.10C	SPBC31F10.10c	zf-MYND type zinc finger protein	1414	50.39	0.5501	-1.69	32.68	48.23	3.05	1.518
SPBC1685.02C	rps1202	40S ribosomal protein S12 (predicted)	1406	152.2	0.8784	1.601	21.78	28.32	9.557	7.362
SPBC354.03	swd3	WD repeat protein Swd3	1400	12.92	0.6637	1.354	36.66	49.54	3.624	1.349
SPAC10F6.08C	nht1	Ino80 complex HMG box subunit Nht1	1398	59.26	0.6074	1.82	35.15	46.76	3.769	1.99
SPBC83.18C	fic1	C2 domain protein Fic1	1397	64.46	0.6446	2.761	33.16	42.66	6.497	3.246
SPBC19C7.02	ubr1	N-end-recognizing protein, UBR ubiquitin-protein ligase E3 Ubr1	1395	36.43	0.9717	-0.251	25.62	36.3	7.636	4.015
SPAC3G6.01	hrp3	ATP-dependent DNA helicase Hrp3	1385	66.89	0.5687	2.495	36.97	48.37	4.67	2.423
SPCC16C4.20C	hap2	HMG box protein (predicted)	1361	96.91	0.7048	1.398	34.8	46.87	3.668	2.351
SPAC1A6.09C	lag1	sphingosine N-acyltransferase Lag1	1359	77.04	0.8261	1.653	35.06	46.87	8.011	4.358
SPAC17H9.10C	ddb1	damaged DNA binding protein Ddb1	1355	81.78	0.5592	4.488	26.48	30.86	7.464	4.937
SPBC16C6.01C	SPBC16C6.01c	lysine methyltransferase (predicted)	1354	124	0.6024	2.758	17.02	20.02	4.633	3.979
SPAC23H3.05C	swd1	Set1C complex subunit Swd1	1347	111.2	0.4329	3.52	36.06	45.66	5.185	1.687
SPCC594.05C	spf1	Set1C PHD Finger protein Spf1	1346	23.6	0.4231	3.272	35.97	45.88	4.601	1.766
SPAC139.01C	ath2	Ath1 complex protein Ath2 nuclease, XP-G family (predicted)	1343	17.07	0.5925	-3.521	31.48	49.11	7.377	3.208
SPCC1620.14C	snf22	ATP-dependent DNA helicase Snf22	1340	41.21	0.2646	-10.17	33.2	60.86	10.42	0.9908
SPAC6F12.09	rdp1	RNA-directed RNA polymerase Rdp1	1340	34.44	0.8413	-0.6335	33.26	47.56	3.575	1.594
SPBC336.01	fbh1	DNA helicase I, ubiquitin ligase F-box adaptor Fbh1	1337	199.9	0.9091	1.092	19.32	25.57	9.157	6.445
SPBC29A10.16C	SPBC29A10.16c	cytochrome b5 (predicted)	1336	86.67	0.9148	-1.51	27	40	13.87	9.243
SPAC13A11.01C	rga8	RhoGAP, GTPase activating protein Rga8	1331	68.07	0.8762	1.116	36.82	50.1	7.501	4.286
SPAC144.02	iec1	Ino80 complex subunit Iec1	1314	52.78	0.4594	-7.918	36.08	61.74	13.02	1.676
SPBC1778.01C	zuo1	zuotin (predicted)	1306	112.6	0.4742	3.665	17.57	19.51	4.396	3.867
SPAC11E3.08C	nse6	Smc5-6 complex non-SMC subunit Nse6	1290	11.28	0.5896	2.563	26.81	34.03	5.273	2.39
SPBP35G2.02	txc1	26S proteasome co-factor Txc1	1283	247.9	0.5474	3.349	36.47	46.48	4.629	4.071
SPAC5D6.05	med18	mediator complex subunit Med18	1281	103	0.6519	3.539	25.94	31.43	7.13	5.752
SPCC306.04C	set1	histone lysine methyltransferase Set1	1270	30.59	0.7174	-1.668	33.55	49.42	5.835	0.6811
SPBC1604.08C	imp1	importin alpha	1266	32.96	0.05014	5.166	33.22	39.36	1.867	1.617

SPBPB10D8.07C	SPBPB10D8.07c	transmembrane transporter (predicted)	1265	203.6	0.264	-4.504	37.66	59.15	4.54	1.135
SPCC285.14	trs130	TRAPP complex subunit Trs130 (predicted)	1249	147.7	0.6193	4.084	18.29	19.93	6.71	6.129
SPBC215.03C	csn1	COP9/signalosome complex subunit Csn1	1247	35.22	0.2005	5.491	26.86	29.99	4.036	2.499
SPCC645.07	rgf1	RhoGEF for Rho1, Rgf1	1245	141.7	0.6735	2.319	28.29	36.45	6.134	2.802
SPBC725.03	SPBC725.03	pyridoxamine 5'-phosphate oxidase (predicted)	1242	132.9	0.7823	3.578	27.76	33.94	11.51	9.259
SPAC1556.08C	cbs2	AMP-activated protein kinase gamma subunit cbs2	1241	114.1	0.6425	2.692	27.41	34.69	5.502	4.1
SPCC1393.05	ers1	RNA-silencing factor Ers1	1237	72.95	0.6776	1.843	33.19	43.99	5.026	2.145
SPBC83.03C	tas3	RITS complex subunit 3	1235	53.03	0.9498	-0.3096	25.87	36.73	5.829	2.073
SPBC16E9.18	psd1	phosphatidylserine decarboxylase Psd1	1234	132.7	0.8721	2.109	29.72	38.74	12.52	8.829
SPBC428.08C	clr4	histone H3 lysine methyltransferase Clr4	1232	71.1	0.8922	-0.6827	28.94	41.56	5.803	2.39
SPAC22E12.04	ccs1	superoxide dismutase copper chaperone Ccs1	1218	42.64	0.2626	-2.702	19.18	30.71	2.466	1.259
SPBC1703.12	ubp9	ubiquitin C-terminal hydrolase Ubp9	1213	38.41	0.1579	7.554	37.67	42.26	2.906	3.962
SPAC824.05	vps16	HOPS/CORVET complex subunit Vps16 (predicted)	1213	92.68	0.5304	5.09	27.58	31.56	7.127	6.058
SPACUNK4.12C	iph1	insulinase pombe homologue 1	1212	158	0.2499	-5.111	39.45	62.52	4.15	3.063
SPAC25G10.03	zip1	transcription factor Zip1	1210	93.33	0.2777	-3.808	14.49	25.68	2.467	2.609
SPAC16C9.06C	upf1	ATP-dependent RNA helicase Upf1	1210	126.7	0.9397	-0.4706	32.14	45.76	4.369	5.032
SPBC609.02	ptn1	phosphatidylinositol-3,4,5-trisphosphate3-phosphatase Ptn1	1209	169.6	0.4607	-3.521	33.9	52.5	5.749	1.122
SPBC216.01C	psy2	protein phosphatase PP4 complex regulatory subunit 3 Psy2 (predicted)	1209	66.75	0.6769	2.335	39.06	51.53	6.065	3.076
SPAC30D11.05	aps3	AP-3 adaptor complex subunit Aps3 (predicted)	1207	55.14	0.6122	-2.518	34.58	52.05	5.467	2.579
SPAC1B3.01C	SPAC1B3.01c	uracil phosphoribosyltransferase (predicted)	1197	13.88	0.06825	5.556	35.56	42.1	2	1.782
SPAC22F3.11C	snu23	U4/U6 x U5 tri-snRNP complex subunit Snu23	1197	13.87	0.6401	-5.487	31.49	51.88	14.51	2.871
SPBC19G7.04	SPBC19G7.04	DNA-binding metalloprotease involved in DNA-protein crosslink removal (	1196	144.2	0.3867	-2.971	38	57.48	3.819	1.887
SPCC18B5.03	wee1	M phase inhibitor protein kinase Wee1	1191	89.15	0.5648	1.33	23.42	30.99	2.409	1.337
SPBC29A3.14C	trt1	telomerase reverse transcriptase 1 protein Trt1	1189	80.84	0.8951	1.217	21.64	28.65	8.396	6.521
SPBC16C6.04	dbl6	double strand break localizing protein Dbl6	1185	165	0.7264	4.594	28.23	33.16	11.19	9.631
SPBC21B10.05C	pop3	WD repeat protein Pop3	1183	111.6	0.4874	-3.089	33.39	51.18	5.109	1.847
SPBC776.05	SPBC776.05	conserved transmembrane protein (predicted)	1181	149.2	0.8082	3	26.24	32.6	11.14	8.752
SPBC1271.12	kes1	oxysterol binding protein (predicted)	1180	127.9	0.6043	3.005	35.8	46.01	5.064	4.138
SPCC613.12C	raf1	CLRC ubiquitin E3 ligase complex specificiy factor Raf1/Dos1	1175	27.08	0.9651	0.4236	29.28	40.49	10.27	5.673
SPAC631.01C	acp2	F-actin capping protein beta subunit Acp2	1174	20.86	0.3595	-4.738	32.87	52.76	5.395	2.766
SPBC2F12.03C	ebs1	EST1 family nonsense-mediated mRNA decay (NMD) pathway protein Et	1173	54.7	0.5547	1.834	35.46	47.19	3.285	1.741
SPBC146.02	SPBC146.02	Schizosaccharomyces specific protein	1167	29.85	0.353	-4.08	35.23	55.15	5.088	1.31
SPBC27B12.08	sip1	Pof6 interacting protein Sip1, predicted AP-1 accessory protein	1167	116.1	0.9258	0.7624	31.2	42.7	7.536	5.731
SPBC557.02C	SPBC557.02c	conserved fungal protein	1166	288.5	0.9146	1.188	23.26	30.97	10.65	7.435
SPAC9G1.07	SPAC9G1.07	Schizosaccharomyces specific protein	1164	28.94	0.6614	-2.724	36.29	54.74	7.213	2.74
SPCC1919.03C	amk2	AMP-activated protein kinase beta subunit Amk2	1163	150.6	0.9837	0.05998	24.04	33.64	1.629	2.506
SPAC2E1P3.05C	SPAC2E1P3.05c	fungal cellulose binding domain protein	1161	19.76	0.2366	-3.923	31.83	50.17	3.645	1.125
SPAC227.07C	pab1	protein phosphatase PP2A regulatory subunit B-55 Pab1	1159	115.8	0.5837	1.884	13.46	16.24	3.31	2.39
SPCC663.12	cid12	poly(A) polymerase Cid12	1156	47.46	0.4638	5.98	23.15	24.09	7.519	5.45
SPAC1805.04	nup132	nucleoporin Nup132	1153	13.38	0.7161	1.937	32.79	43.28	5.312	3.381
SPBC21.02	rtc5	TLDc domain protein 2	1151	126.7	0.7164	2.522	14.99	17.49	5.846	5.131
SPAC20G4.07C	erg4	C-24(28) sterol reductase Erg4	1149	166.9	0.1755	7.884	25.92	25.3	6.159	2.102
SPAC1851.03	ckb1	CK2 family regulatory subunit Ckb1	1149	148.6	0.4247	3.401	34.53	43.67	4.433	2.37
SPBC13G1.08C	ash2	Ash2-trithorax family protein	1145	21.71	0.5587	-4.689	33.29	53.29	9.806	1.985

SPCC736.08	cbf11	CBF1/Su(H)/LAG-1 family transcription factor Cbf11	1143	57.34	0.3098	2.23	31.31	40.8	1.771	1.554
SPAC328.03	tps1	alpha,alpha-trehalose-phosphate synthase [UDP-forming]	1141	98.09	0.5142	2.845	9.275	9.023	3.515	3.232
SPCC417.07C	mto1	MT organizer Mto1	1135	70.63	0.1065	9.712	38.21	39.99	3.793	4.17
SPAC1B2.03C	SPAC1B2.03c	GNS1/SUR4 family protein (predicted)	1135	104.2	0.3961	3.282	30.92	38.78	4.442	1.428
SPBC947.14C	cbp6	mitochondrial respiratory chain complex assembly protein Cbp6 (predicte	1132	177.1	0.7667	2.147	12.67	14.77	6.305	5.247
SPCC4F11.04C	imt2	mannosyltransferase Imt2	1128	220.8	0.8882	1.699	17.77	22.55	11.06	8.489
SPBC3B9.11C	ctf1	mRNA cleavage and polyadenylation specificity factor complex subunit Ct	1126	58.18	0.9151	-1.243	32.25	47	12.41	6.79
SPAC11E3.03	csm1	microtubule-site clamp monopolin complex subunit Csm1/Pcs1	1122	68.44	0.644	-2.705	20.95	33.19	6.328	3.205
SPAC683.03	SPAC683.03	Schizosaccharomyces pombe specific protein	1112	76.19	0.631	1.755	42.22	56.77	3.93	2.018
SPCC188.02	par1	protein phosphatase PP2A regulatory subunit B-56 Par1	1111	113.1	0.8079	2.422	21.23	26.39	8.922	7.092
SPCC126.15C	sec65	signal recognition particle subunit Sec65 (predicted)	1107	150.4	0.8887	1.788	18.99	24.14	11.59	9.043
SPCC576.12C	mhf2	kinetochore protein, CENP-X ortholog, FANCM-MHF complex subunit Mh	1105	134.4	0.3216	2.355	31.77	41.28	1.629	1.806
SPAC2C4.16C	rps801	40S ribosomal protein S8 (predicted)	1105	164.6	0.6486	3.62	17.42	19.37	6.623	5.871
SPAC1F7.09C	dal2	allantoicase Dal2	1105	30.32	0.895	-0.766	37.33	53.45	6.133	3.422
SPAC664.02C	arp8	Ino80 complex actin-like protein Arp8	1104	29.66	0.5739	-5.619	31.09	51.51	11.54	4.292
SPBC530.06C	clu1	clustered mitochondria (cluA/CLU1) homolog Clu1 (predicted)	1103	153.6	0.7729	-2.455	15.44	25.1	8.386	5.514
SPAC6B12.05C	ies2	Ino80 complex subunit Ies2	1100	2.592	0.3685	-5.052	35.02	56.23	6.3	2.33
SPAC821.07C	moc3	transcription factor Moc3	1100	67.42	0.6278	-6.264	31.79	53.39	15.8	3.687
SPBC582.06C	mcp6	horsetail movement protein Hrs1/Mcp6	1098	154.6	0.8279	3.146	27.83	34.63	13.41	10.02
SPAC31A2.14	bun107	WD repeat protein, human WDR48 family Bun107	1097	15.97	0.8595	1.205	32.35	43.71	7.343	3.852
SPAC23C11.14	zhf1	zinc ion transmembrane transporter Zhf1	1095	82.34	0.1761	5.646	24.9	27.01	4.079	2.2
SPCC11E10.08	rik1	silencing protein Rik1	1095	57.25	0.9034	1.367	23.32	30.8	10.35	7.888
SPAC2F7.11	nrd1	RNA-binding protein Nrd1	1091	33.59	0.1002	-7.686	37.8	63.82	4.639	1.714
SPBC1E8.03C	SPBC1E8.03c	conserved fungal protein	1091	34.32	0.9713	-0.1452	38.14	53.72	4.582	2.02
SPBC13G1.14C	rns1	exon junction complex subunit, RNA-binding protein Rns1	1089	112.2	0.7697	4.088	29.88	36.19	12.44	9.967
SPBC409.20C	psh3	ER chaperone SHR3 homologue Psh3	1089	122	0.8545	1.622	25.66	33.73	8.645	5.807
SPCC777.13	vps35	retromer complex subunit Vps35	1088	141.9	0.7176	2.291	13.75	16.08	5.375	4.659
SPBC16D10.07C	sir2	Sirtuin family histone deacetylase Sir2	1088	151.2	0.718	-1.601	37.91	55.44	4.527	2.907
SPCC14G10.03C	ump1	proteasome maturation factor Ump1 (predicted)	1088	57.91	0.8017	-2.609	17.92	28.81	10.62	6.42
SPAPB1E7.06C	eme1	Holliday junction resolvase subunit Eme1	1085	92.12	0.5421	4.172	14.67	14.73	5.185	5.241
SPBC18H10.02	lcf1	long-chain-fatty-acid-CoA ligase Lcf1	1085	113.8	0.7271	-1.756	18.18	27.97	5.535	2.945
SPCC1259.07	rxt3	transcriptional regulatory protein Rxt3	1083	79.62	0.9604	0.5926	21.45	29.27	11.64	7.885
SPAC664.03	paf1	RNA polymerase II associated Paf1 complex (predicted)	1082	36.55	0.6624	-1.35	25.14	37.17	2.269	2.431
SPBC530.08	SPBC530.08	membrane-tethered transcription factor (predicted)	1082	40.84	0.73	-3.328	37.66	57.51	11.91	2.747
SPCC31H12.08C	ccr4	CCR4-Not complex 3'-5'-exoribonuclease subunit Ccr4	1081	50.88	0.7578	3.434	31.72	39.69	11.02	7.171
SPAC15F9.02	seh1	SEA complex subunit Seh1	1077	80.98	0.9548	0.1496	36.2	50.58	2.976	1.347
SPCC31H12.05C	sds21	serine/threonine protein phosphatase PP1 subfamily, Sds21	1076	178.5	0.8087	0.6872	39.16	53.99	3.623	0.3758
SPAC3G9.03	rpl2301	60S ribosomal protein L23	1076	195.3	0.8292	1.489	11.43	13.94	5.448	5.314
SPCC1223.02	nmt1	4-amino-5-hydroxymethyl-2-methylpyrimidine phosphate synthase Nmt1	1075	50.8	0.6014	3.181	32.82	41.58	1.669	5.124
SPCC24B10.12	cgi121	EKC/KEOPS complex subunit Cgi121 (predicted)	1072	87.2	0.6636	-4.528	33.29	53.06	12.91	2.557
SPAC26F1.09	gyp51	GTPase activating protein Gyp51 (predicted)	1072	85.35	0.8428	-0.5307	32.77	46.73	3.07	1.283
SPAC18G6.05C	gcn1	translation elongation regulator Gcn1 (predicted)	1069	79.69	0.2785	-3.378	32.09	49.77	3.078	1.777
SPBC29A3.21	SPBC29A3.21	Schizosaccharomyces pombe specific protein	1069	114.1	0.8462	-2.008	33.83	50.29	11.69	5.235
SPCC31H12.03C	SPCC31H12.03c	RNA binding protein (predicted)	1068	90.14	0.8662	1.242	33.5	45.26	7.776	4.351

SPBC4F6.12	pxl1	paxillin-like protein Pxl1	1068	49.07	0.9398	0.3147	33.24	46.2	4.733	2.086
SPAC25B8.18	SPAC25B8.18	mitochondrial thioredoxin-related protein (predicted)	1066	230.1	0.5984	-3.107	39.97	60.44	5.572	3.934
SPCC576.11	rpl15	60S ribosomal protein L15 (predicted)	1064	175.7	0.8281	1.865	12.83	15.38	7.358	6.315
SPBC216.03	SPBC216.03	conserved fungal protein	1064	138.4	0.8881	1.851	19.25	24.41	11.92	9.313
SPBC947.04	pfl3	cell surface glycoprotein, flocculin Pfl3, DIPSY family	1060	54.92	0.5332	-2.499	35.71	53.62	4.313	2.156
SPAC6G9.10C	sen1	Nrd1 complex ATP-dependent 5' to 3' DNA/RNA helicase Sen1	1060	52.18	0.8323	-1.572	31.26	46.06	8.65	3.344
SPCC1682.01	qcr9	ubiquinol-cytochrome-c reductase complex subunit 9 (predicted)	1058	206.5	0.8931	1.163	10.68	13.35	7.708	6.21
SPBC15D4.06	naa30	NatC N-acetyltransferase complex catalytic subunit Naa30 (predicted)	1053	31.72	0.3162	4.336	36.58	45.25	3.823	2.755
SPBC1685.01	pmp1	dual-specificity MAP kinase phosphatase Pmp1	1053	4.619	0.8805	0.803	38.21	52.48	6.524	1.865
SPCC162.11C	urk1	uridine kinase/uracil phosphoribosyltransferase (predicted)	1052	242.9	0.5139	-2.217	40.54	59.99	4.167	0.6203
SPBC337.15C	coq7	ubiquinone biosynthesis protein Coq7	1051	107.8	0.6554	3.195	11.44	11.56	7.622	4.058
SPCC1450.03	utp502	ribonucleoprotein (RNP) complex Utp502 (predicted)	1050	129.8	0.8472	2.819	23.72	29.33	13.76	10
SPBC3B8.10C	nem1	Nem1-Spo7 phosphatase complex catalytic subunit Nem1 (predicted)	1047	78.62	0.4459	3.576	37.77	47.97	4.968	2.532
SPAC17G8.13C	mst2	histone acetyltransferase Mst2	1047	80.11	0.6187	-2.084	36.99	54.82	5.264	1.517
SPBC337.04	ppk27	serine/threonine protein kinase Ppk27 (predicted)	1047	16.28	0.7405	0.7593	34.31	47.07	1.906	1.665
SPAC222.14C	sey1	GTP binding protein Sey1 (predicted)	1045	42.23	0.2634	3.914	36.01	45.04	3.84	1.35
SPCC790.02	pep3	HOPS/CORVET complex subunit, ubiquitin-protein ligase E3 Pep3/Vps18	1045	188.9	0.99	0.1777	19.17	26.65	13.02	9.959
SPCC1919.10C	myo52	myosin type V	1042	73.39	0.1153	5.324	31.58	36.85	3.245	1.651
SPAC13G6.10C	asl1	cell wall protein Asl1, predicted O-glucosyl hydrolase	1042	44.22	0.7464	2.051	15.22	18.48	6.239	4.107
SPAC17C9.15C	SPAC17C9.15c	Schizosaccharomyces specific protein	1041	136.9	0.4448	3.143	32.56	41.28	4.756	1.558
SPBC36B7.08C	ccp1	CENP-A nucleosome disassembly protein Ccp1	1036	34.09	0.5493	-6.187	37.15	60.81	12.54	2.851
SPAC18G6.02C	chp1	chromodomain protein Chp1	1034	85.62	0.5878	1.394	23.6	31.15	1.518	2.13
SPBC27.02C	ask1	DASH complex subunit Ask1	1033	77.36	0.191	5.413	36.32	43.37	3.939	2.345
SPAC23D3.09	arp42	SWI/SNF and RSC complex subunit Arp42	1033	69.68	0.738	3.155	24.19	29.51	9.809	5.646
SPAC19G12.02C	pms1	MutL family mismatch-repair protein Pms1	1032	154.4	0.5268	1.569	32.35	43.19	2.245	1.701
SPBC8D2.03C	hhf2	histone H4 h4.2	1032	37.21	0.9877	0.183	26.96	37.57	12.3	7.288
SPAC11G7.02	pub1	HECT-type ubiquitin-protein ligase E3 Pub1	1030	17.63	0.2088	3.762	26.82	32.36	1.874	2.232
SPAC3H8.05C	mms1	E3 ubiquitin ligase complex subunit Mms1 (predicted)	1030	73.13	0.2578	-3.682	24.94	40.16	3.201	1.84
